# Automated genomic context analysis and experimental validation platform for discovery of prokaryote transcriptional regulator functions

**DOI:** 10.1186/1471-2164-15-1142

**Published:** 2014-12-18

**Authors:** Ricardo Martí-Arbona, Fangping Mu, Kristy L Nowak-Lovato, Melinda S Wren, Clifford J Unkefer, Pat J Unkefer

**Affiliations:** Bioscience Division, Los Alamos National Laboratory, PO Box 1663, Los Alamos, NM 87545 USA; Theoretical Division, Los Alamos National Laboratory, PO Box 1663, Los Alamos, NM 87545 USA; Los Alamos National Laboratory, P.O. Box 1663, MS E529 Los Alamos, New Mexico 87545 USA

**Keywords:** Comparative genomics, Bxe_B3018, methylglyoxal, Transcriptional regulator, Anisotropy, EMSA, FAC-MS, rtPCR, *Burkholderia xenovorans* LB400, Protein function discovery

## Abstract

**Background:**

The clustering of genes in a pathway and the co-location of functionally related genes is widely recognized in prokaryotes. We used these characteristics to predict the metabolic involvement for a Transcriptional Regulator (TR) of unknown function, identified and confirmed its biological activity.

**Results:**

A software tool that identifies the genes encoded within a defined genomic neighborhood for the subject TR and its homologs was developed. The output lists of genes in the genetic neighborhoods, their annotated functions, the reactants/products, and identifies the metabolic pathway in which the encoded-proteins function. When a set of TRs of known function was analyzed, we observed that their homologs frequently had conserved genomic neighborhoods that co-located the metabolically related genes regulated by the subject TR. We postulate that TR effectors are metabolites in the identified pathways; indeed the known effectors were present. We analyzed Bxe_B3018 from *Burkholderia xenovorans,* a TR of unknown function and predicted that this TR was related to the glycine, threonine and serine degradation. We tested the binding of metabolites in these pathways and for those that bound, their ability to modulate TR binding to its specific DNA operator sequence. Using rtPCR, we confirmed that methylglyoxal was an effector of Bxe_3018.

**Conclusion:**

These studies provide the proof of concept and validation of a systematic approach to the discovery of the biological activity for proteins of unknown function, in this case a TR. Bxe_B3018 is a methylglyoxal responsive TR that controls the expression of an operon composed of a putative efflux system.

**Electronic supplementary material:**

The online version of this article (doi:10.1186/1471-2164-15-1142) contains supplementary material, which is available to authorized users.

## Background

Early researchers established that functional context is often associated with genome organization when they observed that genes for complex traits are often clustered within a genome [1, 2]. By capitalizing upon genomic sequence information and clustering, numerous computational studies have expanded and provided detailed analyses of the association between genomic organization and function [3–9]. The types of genomic associations with functions include conservation of gene order, gene fusion, shared regulatory elements and co-occurrence of genes (phylogenetic profiles) [10]. Of these genomic associations, gene order has been described as the most powerful [10]. Indeed proximity-based computational methods have been used effectively to associate gene cluster profiles with functionality [11], especially when combined with co-occurrence from phylogenetic analysis [11]. Such methods are effective because functionally related genes tend to be physically clustered together on the genome and these arrangements tend to be conserved due to selection pressures [1, 12]. One of the more powerful characteristics of bacterial genomic organization is the frequent collocation of an operon with the gene for its transcriptional regulator (TR) [12–17].

We sought to capitalize upon the physical clustering of genes of related function and the frequent collocation of the TR with its operon to help guide our experimental investigation of TR function. TRs allow or repress transcription in response to the presence of an effector molecule that influences their association or dis-association with their target DNA operator sequence (DOS) [18]. Bacterial transcriptional regulators often respond to small metabolite effector molecules although the identification of effector metabolites to which a given transcriptional regulator responds has been experimentally challenging [16]. Efforts to identify these metabolite effectors have been most successful by testing metabolites generated by the actions of enzyme (s) within a specific operon or metabolic pathway [15–17, 19]. Such efforts have tended to ignore similar metabolites or metabolites in related areas of metabolism, however, because of a lack of a suitable method for screening metabolites that can bind transcriptional regulators. These studies have for the most part found only single metabolite effectors [19]. Homology-based examinations of TRs often identify the TR class to which it belongs, but they provide little or no information regarding their specific function. *In-vivo* experiments that provide candidate effector metabolites can suffer from limitations of solubility, uptake or efflux of the candidate effector metabolite. Mutations either knocking out or over expressing the TR can be lethal or generate a global response, making interpretation extremely complex. Additional approaches are needed to overcome these limitations and facilitate TR function discovery and characterization.

**Figure 1:**
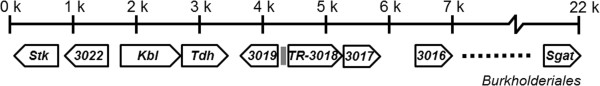
**Genomic neighborhood of** ***bxe_B3018.*** The annotations for these genes are as follow: *Stk* encodes for a non-specific serine/threonine protein kinase; *3022* (*bxe_B3022*) for a TR with unknown function; *Kbl* for glycine C-acetyltransferase; *Tdh* for L-threonine 3-dehydrogenase; *3019* (*bxe_B3019*) encodes for a putative transporter; *TR-3018* (*bxe_B3018*) for TR with unknown function; *3017* (*bxe_B3017*) for a putative transporter; *3016* (*bxe_B3016*) for a putative 50S ribosomal protein L21 family protein and *Sgat* (*bxe_B3001*) for serine-glyoxylate transaminase.

In our quest to discover the biological activity of TR with unknown function we stumbled onto the right question: How can we systematically predict the metabolic network associated with TRs? Then we developed a new method for finding and visualizing gene clusters that have the targeted TR as a member and co-occur in a significant number of organisms. In addition to describing the general applicability of our method here, we investigated a *Burkholderia xenovorans* TR, Bxe_B3018, of unknown function to test the method. Bxe_B3018 is widely conserved among the *Burkholderia* and *Pseudomonas* genus but absent in most other bacteria. Sequence homology analysis suggests that it is a member of the TetR-family of TRs. The DOS for Bxe_B3018 and its orthologs among the *Burkholderia* genus has the general sequence of C (T/G)AGAA (T/C)GATC (G/A)TTCT (C/A)C; this sequence is also present in its own promoter region and the promoter regions of an unknown function gene, *bxe_B3017* in *B. xenovorans*[20]. We identified the metabolite effector (s) of this TR and examined the influence of the effector (s) on the TR binding of its DOS. Based on gene neighborhoods, we used the functional annotation of the genes in clusters to identify the general metabolic network represented by these clusters to guide the construction of a metabolite library of potential effectors of this TR. *In-vitro* screening of this library found three metabolites that bound Bxe_B3018. We examined the interaction of Bxe_B3018 with its DOS and the influence of these TR-binding metabolites on the formation and/or collapse of the TR/DOS complex. Also we investigated the *in-vivo* effects of these metabolites on the expression of the genes within the operon (*bxe_B3016* [EMBL: ABE32981], *bxe_B3017* [EMBL: ABE32980], *bxe_B3018* [EMBL: ABE32979] and *bxe_B3019* [EMBL: ABE32978]) (Figure 1).

## Results

### Approach to the prediction of metabolic function associated with a TR

The goal of our prediction effort was not to find the specific operon or effector molecule associated with a given TR but rather to identify the metabolic area associated with the TR. This metabolic area could be comprised of one or more metabolic pathways. We used the information in the KEGG database [21] to analyze and compare gene clustering and genomic organization among a chosen number of organisms containing close homologs of our TR of interest and that were represented in the KEGG database to discover functional patterns conserved within the TR’s genomic neighborhood. Our experiments suggest that these neighborhoods are usually defined as 5 to 20 genes upstream and downstream of the TR. This method draws strength from examining all sequenced genomes containing homologs of the desired TR for the clustering of metabolically related genes and their conservation in a cluster (co-occurrence). It avoids the typical limitations of methods that require strict conservation of gene order or the definition of non-coding sequence lengths.

### Function Discovery V1.0, a gene neighborhood analysis tool

Function Discovery V1.0 is a Java™ based computational tool created to automate the approach to the prediction of metabolic function associated with a TR described above. The Additional file 1 contains all files required to run the Java™ application. Function Discovery V1.0 requires as input the three-letter KEGG Organism code followed by the locus tag of the desired protein, the number of organisms containing close homologs to the protein of interest (# of homologs), the number of genes upstream and downstream genes from the TR will define the size of the genomic neighborhood (# of genes) and the desired percent of amino acid identity cutoff (aa-IDc). Our experience has suggested that utilizing between 5 and 20 genes to define the neighborhood gives the best results. The aa-IDc will also limit the number of homologs, for example if 100 similar genes and an aa-IDc of 40% were selected, there will be cases where only a few genes will satisfy the aa-IDc of 40% and the list of homologs will be < 100. We have found that setting the aa-IDc < 40% can include TRs from the same protein family that are not functional homologs; while setting aa-IDc > 40% can filter functional homologs and limit the cross organism correlations. Because Function Discovery V1.0 performs a series of online database searches and re-arranges the information for easier analysis, the input of a large genomic neighborhood, a large number of homologs or a low aa-IDc, significantly lengthens the run time. The results are shown in a report that contains the gene information from the KEGG database and several tables summarizing the results of the search (see Additional files 2, 3, 4, 5, 6, 7, 8, 9, 10 and 11 for full examples). The first table, Homolog ID, shows the number of entries in the KEGG database that satisfy both the organisms containing close homologs to the query TR and aa-IDc. The second set of tables, called Neighborhood Representations, presents the genomic neighborhoods for each of the genes in the Homolog ID table. Next, the Over-represented Enzyme Summary table gathers information on all known function proteins in the genomic neighborhoods determined by the Neighborhood Representation tables and ranks them by the frequency of occurrence. This is followed by the Over-represented Metabolite Summary table, which recollects the metabolites identified as substrates or products of the proteins included in the Over-represented Enzyme Summary and ranks them by frequency of occurrence. Finally, the Over-represented Pathway Summary merges the information in the previous tables to identify KEGG metabolic pathways containing the proteins and metabolites and ranks them by the highest number of hits per pathway. The higher the ranking of a metabolic pathway in the Over-represented Pathway Summary, the more likely it is that the TR acts as a regulator for part or the entire metabolic pathway. The report file created by Function Discovery V1.0 is a HTLM file. All the tables have hyperlinks that allow the user to access the information in the KEGG database.

### Function Discovery V1.0 validation

**Figure 2:**
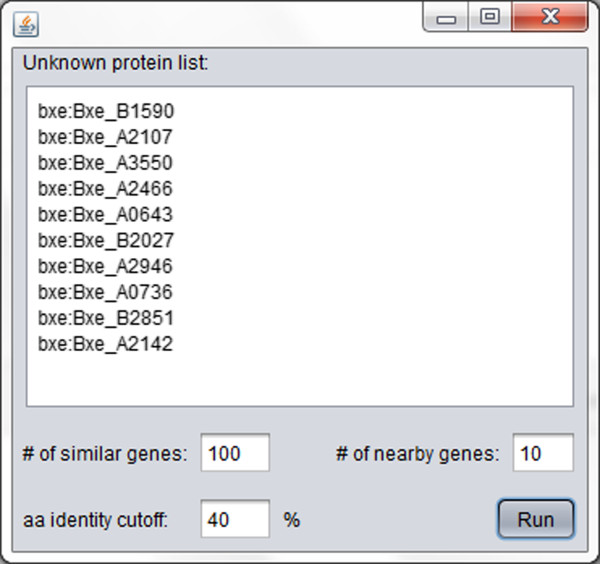
**Graphic user interface for Function Discovery V1.0.** The TR list represents the ten TRs used for validation of this approach: bxe:Bxe_B1590 (BetI), bxe:Bxe_A2107 (CatR), bxe:Bxe_A3550 (CymR), bxe:Bxe_A2466 (CysB), bxe:Bxe_A0643 (GlpR), bxe:Bxe_B2027 (HpaR), bxe:Bxe_A2946 (HutC), bxe:Bxe_A0736 (KynR), bxe:Bxe_B2851 (ModE) and bxe:Bxe_A2142 (RcoM). The parameters used were: “# of similar genes”, 100 (nearest homologs) “# of nearby genes”, 10 (meaning 10 genes upstream and 10 genes downstream); “aa identity cutoff”, 40%.

To validate the approach to the prediction of metabolic function associated with a TR, we analyzed ten transcriptional regulators with known functions and effectors utilizing our software named Function Discovery V1.0. In regard to these ten known TRs, our software was able to identify the metabolic pathway or genes regulated by each TR (Table 1, utilizing 10 genes up and down stream, a 100 homologs and a 40% aa-IDc). Figure 2 presents the graphic user interface (GUI) for Function Discovery V1.0 showing the parameters used for the validation test runs. For nine TRs, including BetI [EMBL: ABE34374] [22], CatR [EMBL: ABE30799] [23], CynR [EMBL: ABE29438] [24], CysB [EMBL: ABE30507] [25], GlpR [EMBL: ABE32291] [26], HpaR [EMBL: ABE33958] [27], KynR [EMBL: ABE32198] [28, 29], HutC [EMBL: ABE30031] [30, 31] and RcoM [EMBL: ABE30826] [32], the pathways known to be regulated by the TRs were identified in the first hit in the Over-represented Pathway Summary (Additional files 2, 3, 4, 5, 6, 7, 8, 9 and 10); in each case the TR effector molecules are metabolic intermediates in the pathway they regulate. The exception was ModE which required a closer analysis of the Function Discovery V1.0 report (Additional file 11). ModE [EMBL: ABE33144] [33] is involved in the regulation of the molybdate transport pump system which is encoded by the operon *modABCDE.* By observing the known genes and conservation patterns in the genomic neighborhoods (Neighborhood Representations table), we could easily identify most of the genes in the operon encoding for the molybdate transport system (Table 1). However, only one of the proteins involved in this system has been assigned an EC number, ModC (EC: 3.6.3.29), which was identified in the genome neighborhood with the highest frequency (see the Over-represented Enzyme Summary table), unfortunately none of the other components of the transport system have been assign EC numbers. In addition, ModC is not associated with any pathway in the KEGG database, which makes it impossible for the Function Discovery V1.0 software to make a pathway prediction in the Over-represented Pathway Summary. This problem is expected to be greater for operons encoding for efflux and transport systems because the genes associated with them rarely have assigned EC numbers and when they do, they may not be associated with any pathway in the KEGG database. In these cases a more detailed analysis of the Function Discovery V1.0 results file is necessary. Despite this limitation, the Function Discovery V1.0 software has shown a remarkable ability to gather the information necessary to associate TRs with their metabolic pathway.

**Table 1:** **Summary of results obtained by Function Discovery V1.0 when applied to ten known-function TRs**

TR ^***a***^	Pathway name	Known pathway genes	Genes clustered with the TR ^***b***^	TRE	Ref
*betI* (*B1590*)	glycine betaine synthesis	*betABTI*	*betABTI*	choline	[21]
*catR* (*A2107*)	benzoate degradation	*catABCR*	*catABCR*	cis, cis-muconate	[22]
*cymR* (*A3550*)	p-cumate catabolism	*cymAaAbAcAdBCDEFHG*	*cymAaAbAcAd─CDE─ ─ ─*	p-cumate	[23]
*cysB* (*A2466*)	sulfur metabolism	*cysABPTWA*	*cysABPTW*	N-acetyl-serine	[24]
*glpR* (*A0643*)	glycerol metabolism	*glpABCDKFR*	*glp─ ─ ─DKFR*	glycerol-3-phosphate	[25]
*hpaR* (*B2027*)	hydroxyphenylacetate catabolism	*hpaGEDFHIR*	*hpaGEDFHIR*	4-hydroxy-phenylacetate	[26]
*kynR* (*A0736*)	oxidative tryptophan degradation	*kynABU*	*kynABU*	L-kynurenine	[27]
*hutC* (*A2946*)	histidine degradation	*hutHUIFGC*	*hutHUIFGC*	urocanate	[28, 29]
*rcoM* (*A2142*)	carbon monoxide oxidation	*coxBCMSLDEFGHIK*	*cox ─ ─SMLDE ─G ─ ─ ─*	carbon monoxide	[30]
*modEB2851*)	molybdate transport	*modABCDE*	*modABC─E*	molybdenum	[31]

^*a*^Transcriptional regulators of interest, the locus tag of the gene is in parentheses and excludes the “*bxe*_” prefix.

^*b*^Genes missing in the cluster are identified by a dash (─). This table summarizes the analysis of 10 genes upstream and 10 genes downstream of the TR of interest.

### Prediction of metabolic function associated with TRs: the case of Bxe_B3018

The gene encoding for our target TR of unknown function, Bxe_B3018 is present in the genome of *B. xenovorans*. Amino acid sequence comparisons using a BLAST search of Bxe_B3018 with the entire set of completely and partially sequenced microbial genomes (utilizing a 40 aa-IDc) identified the existence of 81 homologs (Additional file 12). These homologs were found only in *Burkholderia* or *Pseudomonas* genomes.

**Figure 3:**
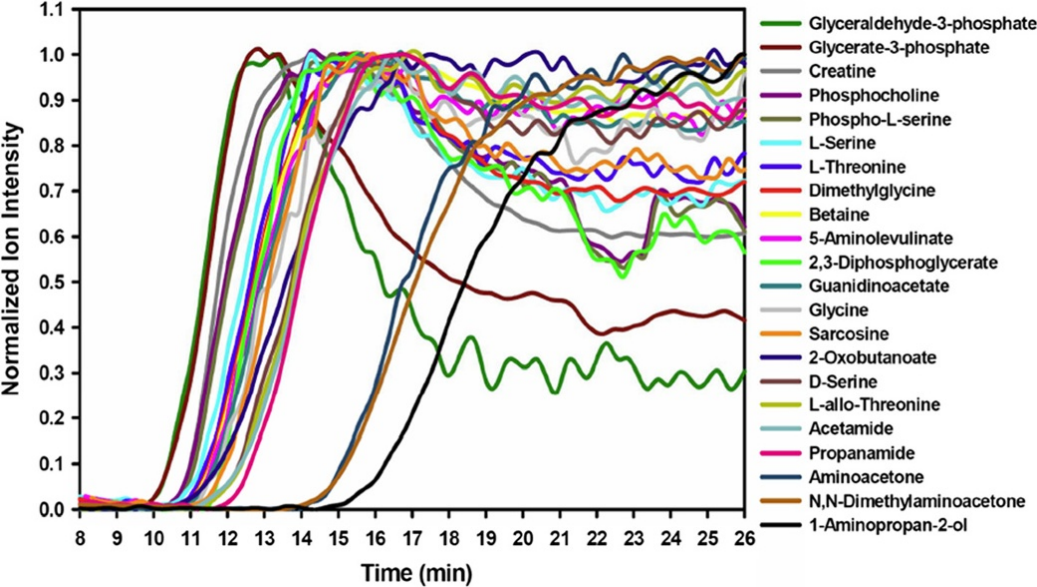
**Condense representation of the KEGG Mapper result.** This pathway representation was obtained from the first pathway hit in the Over-represented Pathway Summary of the Bxe_B3018 metabolic involvement analysis using the software Function Discovery V1.0. The results suggest the involvement of Bxe_B3018 in the glycine, threonine and serine. The yellow rectangles the identified genes from the Over-represented Enzyme Summary table and the red rectangles are the identified metabolites from the Over-represented Metabolite Summary.

Utilizing Function Discovery V1.0, we performed a careful examination of the genomic neighborhood of Bxe_B3018 (Neighborhood Representations) and its homologs (Additional file 13). The examination revealed that, in nearly every instance, these genes were found in close proximity to a cluster of genes involved in the degradation of glycine, threonine and serine; these degradation products provide substrates for the pyruvate metabolism pathway. Specifically, the genes for glycine C-acetyltransferase (EC: 2.3.1.29), L-threonine 3-dehydrogenase (EC: 1.1.1.103) and homoserine kinase (EC: 2.7.1.39) are in close proximity to either Bxe_B3018 or its homologs. The physical locations of other genes (such as serine-glyoxylate transaminase - EC: 2.6.1.45) involved in the degradation of glycine, threonine and serine metabolism were less well conserved but were present in relatively close proximity to Bxe_B3018 and its homologs. Our Function Discovery 1.0 analysis tool identified the glycine, threonine and serine degradation pathway as the top candidate (Over-represented Pathway Summary) and provided hyperlinks that allow the visualization of the identified proteins (yellow) and metabolites (red) in a pathway map (Figure 3).

### High-throughput screening of possible effectors of Bxe_B3018: FAC-MS experiments

**Figure 4:**
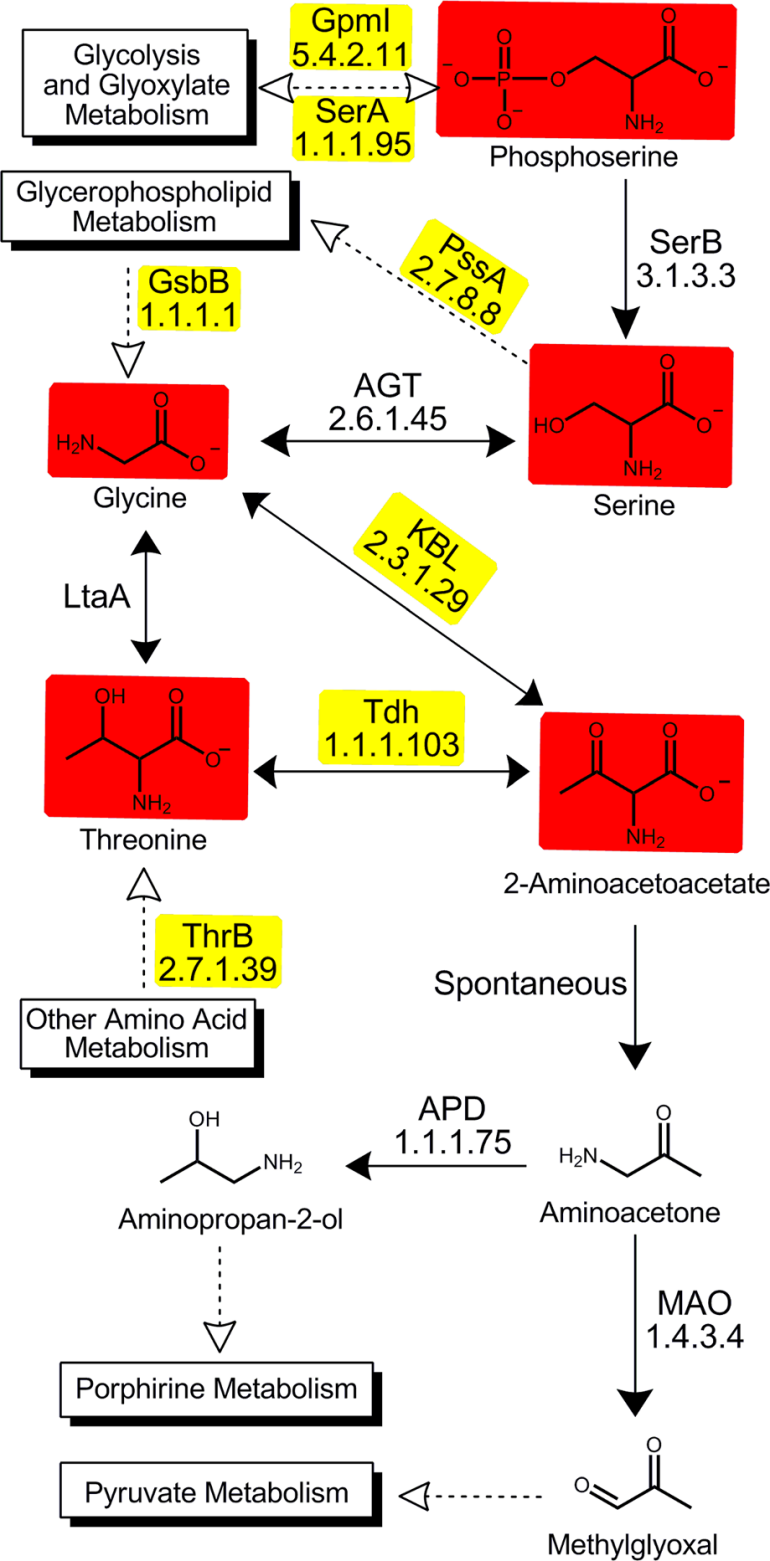
**FAC-MS elution profile for the candidate library.** FAC-MS elution profile of metabolites predicted to be effector molecules of Bxe_B3018. Chemical structures for each metabolite or metabolite analog are found in the Additional file 14.

The predicted association of the degradation of glycine, threonine and serine and subsequent pyruvate metabolism with Bxe_B3018 allowed us to assemble a library of candidate effector metabolites from these metabolic pathways (Additional file 14). These metabolites were screened for binding to immobilized Bxe_B3018 using frontal affinity chromatography coupled with mass spectrometry (FAC-MS) detection of metabolites eluting from the column (Figure 4). This method allowed for direct identification and ranking of metabolites based on their relative binding strengths because the order of elution of these compounds suggests their relative strengths of binding to the effector binding site on GST-Bxe_B3018. The ranking obtained from screening the library of compounds identified aminopropan-2-ol, N,N-dimethylaminoacetone and aminoacetone as potential effectors for Bxe_B3018. In addition to these compounds, the assembled library also contained methylglyoxal, a compound not readily detectable by our mass spectrometry method. For this reason, its potential interaction with Bxe_B3018 was examined using another method as described below.

### Impact of potential effector metabolites on the formation and/or collapse of the TR/DOS complex: Anisotropy experiments

GST-Bxe-B3018 binds its DOS to form a TR/DOS complex in the absence of an effector molecule with a *K*_d_ of 19.0 ± 0.9 nM as estimated by fluorescence polarization (anisotropy) experiments. We also used this method to examine the effects of the potential effector molecules (aminoacetone, N,N-dimethylaminoacetone, methylglyoxal and aminopropan-2-ol) on the affinity of GST-Bxe_B3018 to its DOS. We found that the presence of aminoacetone, N,N-dimethylaminoacetone and methylglyoxal caused the collapse of the TR/DOS complex. The *K*_d_ values for aminoacetone, N,N-dimethylaminoacetone and methylglyoxal were (2.1 ± 0.4) mM, (2.6 ± 0.2) mM and (2.1 ± 0.2) mM, respectively. The anisotropy experiments were not able to detect any significant disruption of the TR/DOS complex when aminopropan-2-ol was used as an effector molecule. Other structurally related molecules, such as acetamide, propionamide and the most weakly bound Glyceraldehyde 3-phosphate (from our FAC-MS experiments) did not affect the TR/DOS complex. We could therefore surmise that these compounds were not acting as effectors in these experiments. Dissociation constants in the presence of candidate effectors were calculated at a saturation concentration of GST-Bxe_B3018 (250 nM) where all DOS (1 nM) was bound. Because aminopropan-2-ol was among the most strongly bound compounds when assayed by FAC-MS, we wondered if it would impact the decrease in Bxe_B3018 binding to its DOS in the presence of aminoacetone, N,N-dimethylaminoacetone or methylglyoxal. Aminopropan-2-ol did not significantly affect the affinity of GST-Bxe_B3018 to its DOS when aminoacetone [(2.2 ± 0.2) mM], N,N-dimethylaminoacetone [(2.4 ± 0.2) mM] or methylglyoxal [(1.9 ± 0.5) mM] were added to the reaction mixture without previous pre-incubation; we therefore concluded that aminopropan-2-ol was not interfering with the binding of these other compounds in our assay.

### Impact of compounds bound by TR on its DOS association: EMSA Assays

**Figure 5:**
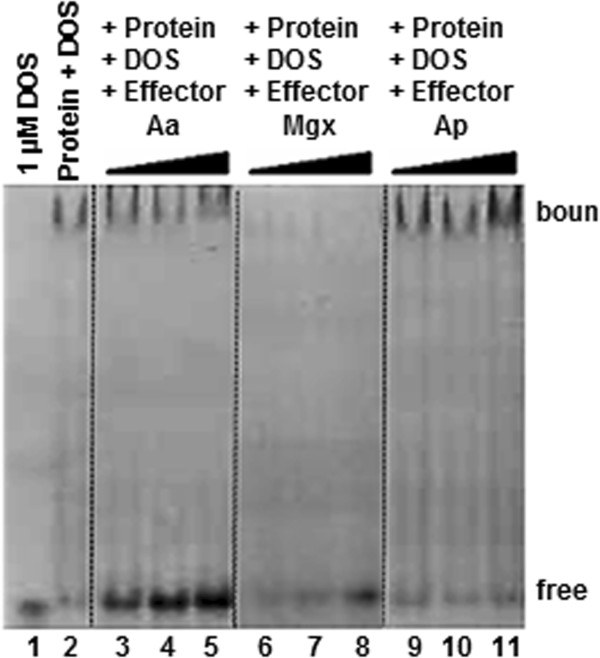
**EMSA for Bxe_B3018 and possible effectors.** EMSA examination of aminoacetone (Aa), methylglyoxal (Mgx) and aminopropan-2-ol (Ap) as potential disruptors of DOS/GST-Bxe_B3018 complex formation. Lane 1 contained unbound DOS (1 μM) as a control and lane 2 contained the DOS/TR complex control (10 μM TR and 1 μM DOS, the standard concentrations for each lane in this experiment). 10, 15 and 20 mM were the concentrations used for effectors. Lanes 3–5 show the simultaneous incubation of TR and its DOS in the presence of increasing concentrations of aminoacetone, lanes 6–8 with methylglyoxal and lanes 9–11 with aminopropan-2-ol. The image show here is representative of a single 20-lane gel, with empty lanes omitted at the dotted lines.

Results from electrophoresis mobility shift assays were consistent with the observations obtained from the anisotropy experiments for aminoacetone (Aa), methylglyoxal (Mgx) and aminopropna-2-ol (Ap) (Figure 5). The third compound that disrupted TR/DOS complex formation, dimethylaminoacetone, was not examined here because it is not a metabolite and therefore not a naturally occurring effector. As shown in Figure 5, lane 1 contained unbound DOS as a control and lane 2 contained the DOS/TR complex control. When the potential effector molecules ([Aa] or [Mgx]) were mixed with Bxe_B3018 and the DOS, the formation of the DOS/TR complex was disrupted (Lanes 3–5 for Aa and 6–8 for Mgx). As the concentration of Aa and Mgx effectors increased, there was an increase DOS/TR disruption, leading to an increase in unbound DOS. When aminopropan-2-ol was used as the effector, there was no disruption of the DOS/TR complex (Lanes 9–11).

### Effects of potential effectors on gene expression: real-time PCR

Real-time PCR experiments were utilized to determine whether the *in-vitro-*identified effector molecules (aminoacetone and methylglyoxal) as well as the non-effector (animopropan-2-ol) were capable of affecting the expression of the genes in the operon (*bxe_B3016, bxe_B3017, bxe_B3018* and *bxe_B3019*). Bxe_B3018 binds its DOS in the absence of an effector molecule; incubation of *B. xenovorans* with the effector molecules should cause Bxe_B3018 to release its DOS increasing the rate of translation of the genes encoding *bxe_B3016, bxe_B3017, bxe_B3018* and *bxe_B3019*. Aminoacetone and aminopropan-2-ol showed no effect on the expression of the regulated genes with concentrations up to 10 mM of each effector. Addition of 10 mM of methylglyoxal and incubation for an hour led to a 40-fold increase in the expression of the regulated genes (*bxe_B3016*, *bxe_B3017* and *bxe_B3019*) and a 100-fold increase in the expression of the TR (*bxe_B3018*), of as measured by real-time PCR (rtPCR).These *in-vivo* results suggest that methylglyoxal was the only effector molecule able to increase translation of the genes in the operon.

## Discussion

As early as the 1930’s, geneticists observed that genes encoding for proteins responsible for complex traits are often clustered within the genome [1, 2, 34, 35]. Computational approaches have estimated that an average of 58% of bacterial genes are found in operons [36]. Proximity-based computational methods have been used effectively to associate gene cluster profiles with functionality [11, 37], although this approach is confounded by insertions, deletions or changes in gene order [36]. To overcome these complications, we developed an automated gene/genomic region analysis tool that rapidly identifies, compares and presents conserved genomic neighborhoods encoding for metabolic pathways across many organisms. Our gene neighborhood examination tool, Function Discovery V1.0, allows us to find collocations in genomic neighborhoods across many organisms, associating metabolic pathways with the target TR. It does not restrict our investigation to a single organism in which gene clustering around the target TR might not have existed because of rearrangements, insertions or deletions of genetic material or other events. Recently, we reported the characterization of three TRs of unknown function in which we used a manual version of our approach to predict functionality and identify potential effector metabolites [29, 38, 39]. Our automated approach, Function Discovery V1.0 has also been effective in finding conserved genes clustered near our target genes and for ten randomly chosen TRs that have known effectors and functions (Table 1). Of course, the ability to gain functional insights using our method is limited by the extent to which the genes clustered near a target gene have been functionally annotated.

In this work, we investigated a *B. xenovorans* TR of unknown function, Bxe_B3018. It is widely conserved among the *Burkholderia* and *Pseudomonas* genus but absent in most other bacteria. There are more than 140 TRs common among the members of the *Burkholderia* genus that have not been fully functionally annotated. Characterization of these TRs is vital to the understanding of the metabolism of this genus, which is characterized by having members with excellent environmental adaptability and/or high pathogenicity. Function Discovery V1.0 analysis suggested that Bxe_B3018 may be involved in the glycine, serine and threonine degradation pathway. The operon regulated by Bxe_B3018 is found upstream from several genes involved in this metabolism. Our software analysis coupled with our *in-vitro* and *in-vivo* experiments suggests that it responds to a metabolite from the glycine, serine and threonine metabolism pathway, methylglyoxal. Fluorescence anisotropy experiments suggest that Bxe_B3018 binds methylglyoxal with a dissociation constant of ~2 mM. Millimolar concentrations of methylglyoxal in bacterial cells have been previously reported [40, 41]. The Bxe_B3018-regulated operon includes the genes *bxe_B3016*, *bxe_B3017*, *bxe_B3018* and *bxe_B3019*. Bxe*_*B3017 is a hypothetical protein that shows some sequence similarity to sn-glycerol-3-phosphate transporters (GlpT). GlpT is a transporter involved in antibiotic resistance in *Pseudomonas aeruginosa*[42]. *bxe_B3016* encodes a protein of unknown function belonging to the 50S ribosomal protein L21 family. 50S is the larger subunit of the 70S ribosome of prokaryotes and the site of inhibition of macrolides [43] and the pleuromutilins [44] antibiotics. *bxe_B3019* is a member of the YceI family of transport proteins, a widely conserved protein commonly involved in antimicrobial resistance mechanisms [45, 46].

## Conclusions

Based on its response to methylglyoxal, we propose the Bxe_B3018 be given functional name MgxR. The potential relationship between the functions encoded by the MgxR-regulated operon genes and serine, glycine and threonine degradation may be revealed by consideration of the effector methylglyoxal. It is a side product of both glycine degradation and glycolysis and is the major electrophile produced intracellularly by bacterial cells [41]. Methylglyoxal is a highly reactive 1,2-dicarbonylic compound that reacts readily with amino groups, causing structural changes in proteins and DNA and generating free radicals in the process [40, 47]. As is to be expected for such a deleterious compound, there are several paths known for detoxification of methylglyoxal. The sulphydryl group of glutathione can react spontaneously with methylglyoxal, followed by the actions of the glyoxalase I and glyoxalase II enzymes, thus producing D-lactate, which can be either excreted or recycled to pyruvate by lactate dehydrogenase [48, 49]. Alternatively, bacteria can utilize the KefB/KefC potassium pump system to acidify the cytoplasm and inactivate the methylglyoxal [41, 48, 49]. Our findings suggest that *B. xenovorans* may have another detoxification mechanism, a methylglyoxal efflux system encoded by the MgxR regulated operon. Further characterization of this putative efflux system encoded by the *bxe_B3016*, *bxe_B3017*, *bxe_B3018* and *bxe_B3019* operon is required.

## Methods

### Approach to the prediction of metabolic function associated with a TR

A flowchart diagram describing the steps used by the Function Discovery V1.0 software to predict the metabolic involvement of TR is shown in the Additional file 15. We used the KEGG Sequence Similarity DataBase (SSDB) [50] to find a set of genes similar to our target TR. The KEGG SSDB identified all possible pairwise genome comparisons and recorded the gene pairs with the Smith-Waterman similarity score of 100 or more. We used the information of bidirectional best hits (best-best hits) to select a set of similar genes in various species for our target TR with a cutoff of 40% amino acid identity. For each similar gene at a position in the chromosome, we found a set of nearby genes upstream and downstream from the position. From those nearby genes, we identified a subset of genes that encoded enzymes. We collected the metabolic reactions for those enzymes and recorded their metabolic pathways. For each metabolic reaction, we identified its substrates and products (metabolites involved). The metabolites, metabolic reactions and metabolic pathways identified from the whole set of similar genes were collected. We counted the over-representation of those metabolites, metabolic reactions and pathways. Over-represented metabolic reactions are more likely to be co-regulated and belong to the same KEGG metabolic pathway. The identified metabolites (and analogs) were good candidates for experimental validation of their activity as effector molecules of the queued TR. The software tools to implement the above procedure are written based on KEGG SOAP API , developed under Java™ 7.0 and tested under Windows, Mac OS and Linux operating systems. With the recent update of KEGG REST API (http://www.kegg.jp/kegg/rest/keggapi.html), we re-wrote the codes (Additional file 1). We used these tools to analyze the metabolic involvement of the 10 known-function TRs and the unknown-function TR Bxe_B3018. Then we manually checked the results for Bxe_B3018, determined the metabolic pathways represented in the results and chose a library of metabolites and analogs from this area of metabolism as candidate TR effectors for experimental testing and functional validation. A full description and step-by-step instructions on how to use the Function Discovery V1.0 software can be found in the supplemental materials.

### Preparation of Bxe_B3018 affinity column

The genomic DNA of *B. xenovorans* was isolated from the *B. xenovorans* LB400 cells by following the manufacturer’s specified protocol for the DNA Mini Kit. The gene encoding for Bxe_B3018 was cloned from the *Burkholderia xenovorans LB400* genomic DNA into the pGEX-KG expression vector. The Bxe_B3018 gene was amplified by conventional PCR methods using two primers: forward 5′-GATCCATGGATGAGAACAGCACACCGAACCT-3′ and reverse 5′-GATCGGATCCCTACGCCGCGTTCTCGT-3′, containing *Nco*I and *Bam*HI sites, respectively. The resulting DNA fragment was incorporated into the *Nco*I and *Bam*HI sites of the pGEX-T2 plasmid.

The pGEX-T2 plasmid containing the gene encoding for Bxe_B3018 was transformed into Arctic Express competent cells. A single colony was cultured overnight in 25 mL of LB media containing 100 mg/L ampicillin. Then, 4 mL were used to inoculate 6 L of the same media. Cell cultures were grown at 37°C with a rotary shaker until an Abs_600nm_ of ~ 0.6 OD was reached, after which the temperature was dropped to 16°C and protein expression was initiated by the addition of isopropyl-thiogalactoside to a final concentration of 1.0 mM. Then, the culture was incubated overnight at 16°C. After incubation, the bacterial cells were isolated by centrifugation at 7000 × *g* for 10 min at 4°C. The cell pellet was washed and re-suspended in 20 mM Na_2_PO_4_ buffer containing 100 mM NaCl at pH 7.2 (buffer A). Also, 5 μg/mL DNAse and 0.1 mg/mL of the protease inhibitor (PMSF) per gram of cells were added to buffer A. The resuspended cells were disrupted by sonication. The soluble protein was separated from the cell debris by centrifugation at 12,000 × *g* for 15 min at 4°C, then loaded onto a 5 mL GSTrap FF column equilibrated with buffer A and eluted with a lineal gradient of 50 mM Tris-Cl and 10 mM reduced glutathione at pH 8.1 (buffer B). Fractions containing Bxe_B3018 were pooled, concentrated and loaded onto a high load 26/60 Superdex 200 prep grade gel filtration column (GE Health Care) and eluted with 100 mM Na_2_PO_4_ buffer containing 150 mM NaCl at pH 7.2 (buffer C). The purity of the protein during the isolation procedure was monitored by SDS gel.

The GST-tagged Bxe_B3018 protein was diluted to a concentration of 1 mg/mL and loaded onto a 1 mL GSTrap FF affinity column. After loading the GST-tagged Bxe_B3018 protein, the FAC-MS columns were washed with 10 column volumes of buffer A. The preparation of the FAC-MS column was concluded by exchanging the binding buffer for a MS-friendly buffer such as 20 mM ammonium formate at pH 7.2 (buffer C).

### Screening the library of possible TR effectors

A library of metabolites and structurally related compounds was assembled as described in the “Approach to the prediction of metabolic function associated with a TR” section and used to investigate the effector binding specificity of GST-B3018. The compounds were acetamide, aminopropan-2-ol, aminoacetone, N,N-dimethylaminoacetone, propanamide, glycine, dimethylglycine, sarcosine, creatine, betaine, L-threonine, L-allo-threonine, L-serine, D-serine, phosphor-L-serine, 5-aminlevulinate, 2-oxobutanoate, guanidinoacetate, glycerate-3-phosphate, glyceraldehydes-3-phosphate, 2,3-diphosphoglycerate and phosphocholine (structures in Additional file 2). The compounds were each present in the mixture of potential effectors at 10 μM concentrations in buffer C and the final pH was adjusted to 7.4.

The affinity columns prepared with the tagged Bxe_B3018 protein were coupled to an Exactive Benchtop FT Mass Spectrometer (Thermo Fisher) and the mixture of potential effectors was continuously infused through the column. The effluent from the column was mixed with methanol containing 0.1% formic acid (makeup solution) immediately before infusion to the MS. The total flow rate used for the FAC-MS experiments was 170 μL/min into the mass spectrometer including 85 μL/min of the ligand solution and 85 μL/min of the makeup solution. The column was connected to the mass spectrometer and syringe pumps (OEM PSD/3 Syringe Pump, Hamilton Company) and was allowed to equilibrate with buffer C until the background level of the ion [M+H]^+^ signal was stable. Then, the system was switched to the analyte solution (mixture of potential effectors) and data were acquired continuously for 30 min. After each run, the column was regenerated with 10 column volumes of buffer C in preparation for the next run.

### Fluorescence anisotropy assays of Bxe_B3018 / DOS interaction

The Fluorescein-labeled DNA Operator Sequence (DOS) (5′-Fluo-CGAGGG*AGAA*TGATCG*TTCT*ACCCTT-3′) and its complement sequence (5′-AAGGGT*AGAA*CGATCA*TTCT*CCCTCG-3′) were dissolved in distilled water to a concentration of 100 μM. The annealing reaction was performed by incubating a 20 μM solution of the two oligonucleotides in 20 mM Tris-Cl, 100 mM NaCl and 1 mM MgCl_2_ at pH 7.5 at 95°C in a heat block for 5 min, then the block was removed from the heat source and allowed to reach room temperature. The progressive decline to room temperature allowed the annealing to occur. The fluorescence polarization experiments were performed in 20 mM Tris-Cl, 60 mM KCl, 40 mM NaCl, 0.5 mM EDTA, 1.0 mM DTT, 1.0 mM MgCl_2_ and 100 μM/mL BSA at pH 7.5 (buffer D). The 200 μL reaction mixture was prepared in the following order: first 10× Buffer D was added to the wells of a black 96-well; then 20× Fluorescein-labeled DOS was added to a final concentration of 1 nM; then distilled water was added to complete the 200 μL reaction volume minus the GST-Bxe_B3018 reaction volume. Finally, the reaction was initiated by the addition of the appropriate amount the GST-Bxe_B3018 (from 0 to 250 μM final concentration). The reactions were mixed by pipetting and incubated for one hour at 30°C in the dark. The Fluorescein-labeled DOS was excited with polarized light through an excitation filter of 485/20 nm and the emission was measured with an emission filter of 528/20 nm with a dichroic mirror of 510 nm. Fluorescence polarization was monitored with a BioTek - Synergy H4 hybrid Microplate Reader and anisotropy values calculated automatically with the Gen5 software using the standard G factor of 0.85.

The collected data were plotted using Sigma Plot 11.0. The dissociation constants of GST-Bxe_B3018 for its Fluorescein-labeled DOS in the presence of possible effectors were obtained from the fluorescence polarization experiments by fitting the change in anisotropy values vs. the GST-Bxe_B3018 protein concentration to a modified binding polynomial equation including the Hill coefficient [51]:
1

where *ΔA* is the change in fluorescence anisotropy, *ΔA*_*T*_ is the total change in anisotropy, *E* is the total MetJ concentration at each point in the titration, *K*_d_ is the dissociation constant, and *H* is the Hill coefficient.

### Electrophoresis mobility shift assay (EMSA) of Bxe_B3018/DOS interaction

The DOS utilized in these experiments was obtained from the upstream promoter region of Bxe_B3018. The double stranded DOS was obtained from the annealing of two synthetic oligonucleotides. The synthetic oligonucleotides used consist of a 5′-CGAGGGAGAATGATCGTTCTACCCTT-3′ sequence and its reverse complement of oligonucleotides (5′-AAGGGTAGAACGATCATTC-TCCCTCG-3′). The annealing reaction was performed as explained above. The DOS was further diluted to 2 μM. The GST-Bxe_B3018 protein was diluted to a stock solution of 17 μM in buffer B containing 25% glycerol. The reaction mixtures consisted of 1.0 μM of DOS and protein concentrations of 10 μM in 20 mM Tris-Cl, 500 μM EDTA, 5% glycerol, 200 μM DTT, 0.001% Triton X, 50 mM NaCl, 5 mM MgCl_2_, 2.5 mM CaCl_2_ at pH 8.0 (buffer E). The aminoacetone, aminopropan-2-ol and methylglyoxal concentrations ranged from 10 to 20 mM. The reaction mixtures were incubated at room temperature for 30 min. A 5% native polyacrylamide gel was pre-run for 20 min at 8 V/cm in TBE buffer composed of 44 mM Tris, 44 mM Borate, 1.0 mM EDTA at pH 8.3. Then, the samples were loaded and run at 8 V/cm until the bromophenol yellow (loading dye) migrated 1/3 down the gel. Gels were stained with ethidium bromide, imaged on a BIORAD ChemiDoc XRS imaging station.

### *In-vivo*validation of effectors by real-time PCR

*B. xenovorans* cells were grown in tripticase soy broth until they reached 0.6 OD_600nm_. One mL aliquots were treated with 1 mM aminoacetone, aminopropan-2-ol or methylglyoxal and incubated for 30 min under constant shaking. *B. xenovorans* cell were collected by centrifugation at 6,000 × *g* and RNA was isolated from the treated cells using the Qiagen RNeasy-mini-kit (Qiagen) followed by removal of DNA using a Turbo DNA-free kit (Life Technologies) according to manufacturer’s instructions. The RNA was then analyzed for purity and concentration on a Nano Drop 1000 spectrophotometer. 100 ng of RNA was converted to cDNA in a 20 μL reaction using High Capacity RNA-to-cDNA (Life Technologies) following the manufacturer’s protocol. Real-Time PCR assays were run in triplicate using 2 μL of a 1:10 dilution of cDNA per reaction. MGB TaqMan® assays for Bxe_B3016, Bxe_B3017, Bxe_B3018 and 16 s were designed and ordered from Life Technologies Custom TaqMan Gene Expression assay design tool (https://www.lifetechnologies.com/order/custom-genomic-products/tools/gene-expression/). Reactions were carried out using TaqMan® Fast Universal PCR Master Mix (2×), No AmpErase UNG (ABI #4352042) on ABI 7500 DX instruments run in fast mode with the following cycling parameters: 1 cycle 95°C for 20 s followed by 40 cycles 95°C for 3 s and 60°C for 30 s. Data were analyzed using the Data Assist v3.01 Real-Time PCR analysis software (Life Technologies). The relative gene expression values were determined using the 2-∆∆C_T_ method [52]. The fold change of the ΔΔC_T_ was compared across all data sets.

## Ethics

There were no vertebrate or invertebrate subjects used in this investigation.

## Electronic supplementary material

Additional file 1: **Code.** Zip folder containing all files required to run the Java™ application. The files need to be extracted to the same folder and then the FunctionDiscoveryV1.0.jar interface can be launched. For detailed instructions on how to use the interface please refer to the Function Discovery V1.0, a gene neighborhood analysis tool section in the Results part of the main text. (ZIP 415 KB)

Additional file 2: **Result BetI.** Function Discovery V1.0 output (.html format) for the glycine betaine biosynthesis regulator (BetI, Bxe_B1590). For detailed instructions on how to analyze the results please refer to the Function Discovery V1.0, a gene neighborhood analysis tool section in the Results part of the main text. (HTML 596 KB)

Additional file 3: **Result CatR.** Function Discovery V1.0 output (.html format) for the benzoate degradation regulator (CatR, Bxe_ A2107). For detailed instructions on how to analyze the results please refer to the Function Discovery V1.0, a gene neighborhood analysis tool section in the Results part of the main text. (HTML 258 KB)

Additional file 4: **Result CymR.** Function Discovery V1.0 output (.html format) for the p-cumate catabolism regulator (CymR, Bxe_ A3550). For detailed instructions on how to analyze the results please refer to the Function Discovery V1.0, a gene neighborhood analysis tool section in the Results part of the main text. (HTML 85 KB)

Additional file 5: **Result CysB.** Function Discovery V1.0 output (.html format) for the sulfur metabolism regulator (CysB, Bxe_ A2466). For detailed instructions on how to analyze the results please refer to the Function Discovery V1.0, a gene neighborhood analysis tool section in the Results part of the main text. (HTML 676 KB)

Additional file 6: **Result GlpR.** Function Discovery V1.0 output (.html format) for the glycerol metabolism regulator (GlpR, Bxe_ A0643). For detailed instructions on how to analyze the results please refer to the Function Discovery V1.0, a gene neighborhood analysis tool section in the Results part of the main text. (HTML 684 KB)

Additional file 7: **Result HpaR.** Function Discovery V1.0 output (.html format) for the hydroxyphenylacetate catabolism regulator (HpaR, Bxe_ B2027). For detailed instructions on how to analyze the results please refer to the Function Discovery V1.0, a gene neighborhood analysis tool section in the Results part of the main text. (HTML 380 KB)

Additional file 8: **Result KynR.** Function Discovery V1.0 output (.html format) for the oxidative tryptophan degradation regulator (KynR, Bxe_ A0736). For detailed instructions on how to analyze the results please refer to the Function Discovery V1.0, a gene neighborhood analysis tool section in the Results part of the main text. (HTML 476 KB)

Additional file 9: **Result HutC.** Function Discovery V1.0 output (.html format) for the histidine degradation regulator (HutC, Bxe_ A2946). For detailed instructions on how to analyze the results please refer to the Function Discovery V1.0, a gene neighborhood analysis tool section in the Results part of the main text. (HTML 726 KB)

Additional file 10: **Result RcoM.** Function Discovery V1.0 output (.html format) for the carbon monoxide oxidation regulator (RcoM, Bxe_ A2142). For detailed instructions on how to analyze the results please refer to the Function Discovery V1.0, a gene neighborhood analysis tool section in the Results part of the main text. (HTML 36 KB)

Additional file 11: **Result ModE.** Function Discovery V1.0 output (.html format) for the molybdate transport regulator (ModE, Bxe_ B2851). For detailed instructions on how to analyze the results please refer to the Function Discovery V1.0, a gene neighborhood analysis tool section in the Results part of the main text. (HTML 732 KB)

Additional file 12: Figure Af1: Dendrogram of microbial Bxe_B3018 Homologues. BLAST analysis (utilizing a 40 aa-IDc) identified the existence of 81 homologs. These homologs were found only in *Burkholderia* or *Pseudomonas* genomes. (PDF 15 KB)

Additional file 13: **Result Bxe_B3018.** Function Discovery V1.0 output (.html format) for the previously unknown function transcriptional regulator (Bxe_ B3018, MgxR). For detailed instructions on how to analyze the results please refer to the Function Discovery V1.0, a gene neighborhood analysis tool section in the Results part of the main text. (HTML 551 KB)

Additional file 14: Figure Af2: Structures for the library of candidate effector metabolites predicted from the analysis of the Function Discovery V1.0 output for Bxe_B3018. (PDF 16 KB)

Additional file 15: Figure Af3: Flowchart describing the steps used by the Function Discovery V1.0 software to predict the metabolic involvement of a given TR. (PDF 9 KB)

## Abbreviations

TR: Transcriptional regulator
rtPCR: Real-time polymerase chain reaction
DNA: Deoxyribonucleic acid
DOS: DNA operator sequence
KEGG: Kyoto encyclopedia of genes and genomes
aa-IDc: Amino acid identity cutoff
NCBI: National Center for Biotechnology Information
ID: Identity
EC: Enzyme commission
BLAST: Basic local alignment search tool
FAC-MS: Frontal affinity chromatography coupled to MS detection
G3P: Glyceraldehyde 3-phosphate
Aa: Aminoactone
Mgx: Methylglyoxal
Ap: Aminopropan-2-ol
SSDB: Sequence similarity data base
GST: Glutathione S-transferase
PMSF: Phenylmethylsulfonyl fluoride
DTT: Dithiothreitol
BSA: Bovine serum albumin
EMSA: Electrophoretic mobility shift assay
EDTA: Ethylenediaminetetraacetic acid.
