# Supplementary material for: Automated genomic context analysis and experimental validation platform for discovery of prokaryote transcriptional regulator functions
Source: BMC Genomics. 2014 Dec 18;15(1):1142. doi: 10.1186/1471-2164-15-1142 (PMC4349456; doi:10.1186/1471-2164-15-1142)
Supplement: Supplementary file 1 — Additional file 1: Code. Zip folder containing all files required to run the Java™ application. The files need to be extracted to the same folder and then the FunctionDiscoveryV1.0.jar interface can be launched. For detailed instructions on how to use the interface please refer to the Function Discovery V1.0, a gene neighborhood analysis tool section in the Results part of the main text. (ZIP 415 KB) [file 12864_2014_6995_MOESM1_ESM.zip › codeF/bxe_Bxe_B3018_s10_n10_i60.html]

```
ENTRY       Bxe_B3018         CDS       T00340
DEFINITION  TetR family transcriptional regulator
ORGANISM    bxe  Burkholderia xenovorans LB400
POSITION    2:12472..13137
MOTIF       Pfam: TetR_N
DBLINKS     NCBI-GI: 91777121
            NCBI-GeneID: 4006480
            JGI: BxeB3018
            UniProt: Q13SG0
AASEQ       221
            MATETHIESVPPGSARERLLDAAEALIYAGGIHATGVDAIVKQSGTARKSFYTHFESKDA
            LVAAALDRRDERWMNWFIAGTQRHGKTARKRLLGMFEVLREWFASKDFHGCAFLNASGEI
            ASADDPIRIVARKHKERLLAFVRTECDGLAAESGMDARRAARLSRQWLILLDGAIAVALV
            SGEPDAALDAQAAAQVLLDAECACERGTPPSKRPPSRRTAT
NTSEQ       666
            atggctaccgaaactcacattgaatcagtgccgccgggcagcgcccgcgagcggctgctg
            gatgcggcggaggcgttgatctacgccggcggcattcacgcaaccggcgtggacgcgatc
            gtcaagcagtccggcacggcgcgcaaaagcttctacacgcatttcgaatcgaaagacgca
            ctcgtggcggctgcgctcgaccggcgcgacgaacgctggatgaactggttcatcgcaggc
            acgcaacggcacggtaagacggcgcgcaaacgtctgctcggcatgttcgaggtgctgcgc
            gaatggtttgcgtcgaaagattttcatggttgcgcgtttctgaatgcgtccggtgagatc
            gcttccgcggacgatccgatcaggatcgtcgcgcgcaaacacaaggaacgcctgctggcg
            ttcgtgcggaccgaatgcgacgggttggctgcggagtcgggcatggatgcccgccgcgcc
            gcgcgtctgtcgcgtcaatggttgatcctgctcgacggcgcgattgccgtcgcgctggta
            agcggcgagcccgatgcggcgctcgacgcgcaagctgcagcccaagtcctgctcgacgcg
            gagtgtgcgtgcgagcgaggcacaccgccaagcaaacgaccgccatcccggcgaaccgca
            acctga
///
```

  
**Homolog ID**: Table of closest homologs  

```
                 Homologs                                       len   identity overlap
---------------------------------------------------------------------------------
bpy:Bphyt_7001 TetR family transcriptional regulator          221     0.932    221 
bge:BC1002_5113 TetR family transcriptional regulator         223     0.785    214 
bgf:BC1003_4755 regulatory protein TetR                       233     0.742    233 
bph:Bphy_4162 TetR family transcriptional regulator           210     0.797    197 
bte:BTH_II0010 TetR family transcriptional regulator          205     0.706    197 
bma:BMAA0008 TetR family transcriptional regulator            205     0.701    197 
bml:BMA10229_1436 TetR family transcriptional regulator       205     0.701    197 
bmn:BMA10247_A0011 TetR family transcriptional regulato       205     0.701    197 
bmv:BMASAVP1_1156 TetR family transcriptional regulator       205     0.701    197 
bpd:BURPS668_A0010 TetR family transcriptional regulato       205     0.701    197
```

**Neighborhood Representations**: Table of genes in the defined genetic neighborhoods of the entry protein and its closest homologs  
  
**Neighborhood Representations for "bxe:Bxe\_B3018"**  

| ID | Annotation | EC number |
| --- | --- | --- |
| bxe:Bxe\_B3028 | putative partition protein ParA; K03496 chromosome partitioning protein |  |
| bxe:Bxe\_B3027 | ParB family protein; K03497 chromosome partitioning protein, ParB family |  |
| bxe:Bxe\_B3026 | putative replication protein |  |
| bxe:Bxe\_B3025 | hypothetical protein |  |
| bxe:Bxe\_B3024 | putative phage integrase |  |
| bxe:Bxe\_B3023 | hypothetical protein |  |
| bxe:Bxe\_B3022 | XRE family transcriptional regulator |  |
| bxe:Bxe\_B3021 | 2-amino-3-ketobutyrate coenzyme A ligase (EC:2.3.1.29); K00639 glycine C-acetyltransferase [EC:2.3.1.29] | ec:2.3.1.29 |
| bxe:Bxe\_B3020 | tdh; L-threonine 3-dehydrogenase (EC:1.1.1.103); K00060 threonine 3-dehydrogenase [EC:1.1.1.103] | ec:1.1.1.103 |
| bxe:Bxe\_B3019 | putative YceI like family protein |  |
| bxe:Bxe\_B3018 | TetR family transcriptional regulator |  |
| bxe:Bxe\_B3017 | hypothetical protein; K09958 hypothetical protein |  |
| bxe:Bxe\_B3016 | hypothetical protein |  |
| bxe:Bxe\_B3015 | MarR family transcriptional regulator |  |
| bxe:Bxe\_B3014 | hypothetical protein |  |
| bxe:Bxe\_B3013 | DNA-7-methylguanine glycosylase (EC:3.2.2.-) |  |
| bxe:Bxe\_B3012 | hypothetical protein |  |
| bxe:Bxe\_B3011 | CheA signal transduction histidine kinases (STHK) (EC:2.7.3.-); K00936 [EC:2.7.3.-] |  |
| bxe:Bxe\_B3010 | methyl-accepting chemotaxis sensory transducer |  |
| bxe:Bxe\_B3009 | hypothetical protein |  |
| bxe:Bxe\_B3008 | hypothetical protein |  |

  
**Neighborhood Representations for "bpy:Bphyt\_7001"**  

| ID | Annotation | EC number |
| --- | --- | --- |
| bpy:Bphyt\_6991 | hydrophobe/amphiphile efflux-1 (HAE1) family transporter; K18138 multidrug efflux pump |  |
| bpy:Bphyt\_6992 | RND family efflux transporter MFP subunit; K03585 membrane fusion protein |  |
| bpy:Bphyt\_6993 | two component winged helix family transcriptional regulator |  |
| bpy:Bphyt\_6994 | integral membrane sensor signal transduction histidine kinase; K07638 two-component system, OmpR family, osmolarity sensor histidine kinase EnvZ [EC:2.7.13.3] | ec:2.7.13.3 |
| bpy:Bphyt\_6995 | hypothetical protein |  |
| bpy:Bphyt\_6996 | hypothetical protein |  |
| bpy:Bphyt\_6997 | hypothetical protein |  |
| bpy:Bphyt\_6998 | signal peptide transmembrane protein |  |
| bpy:Bphyt\_6999 | hypothetical protein |  |
| bpy:Bphyt\_7000 | hypothetical protein; K09958 hypothetical protein |  |
| bpy:Bphyt\_7001 | TetR family transcriptional regulator |  |
| bpy:Bphyt\_7002 | hypothetical protein |  |
| bpy:Bphyt\_7003 | tdh; L-threonine 3-dehydrogenase; K00060 threonine 3-dehydrogenase [EC:1.1.1.103] | ec:1.1.1.103 |
| bpy:Bphyt\_7004 | 2-amino-3-ketobutyrate coenzyme A ligase (EC:2.3.1.29); K00639 glycine C-acetyltransferase [EC:2.3.1.29] | ec:2.3.1.29 |
| bpy:Bphyt\_7005 | XRE family transcriptional regulator |  |
| bpy:Bphyt\_7006 | hypothetical protein |  |
| bpy:Bphyt\_7007 | integrase family protein |  |
| bpy:Bphyt\_7008 | hypothetical protein |  |
| bpy:Bphyt\_7009 | replication protein |  |
| bpy:Bphyt\_7010 | parB-like partition protein; K03497 chromosome partitioning protein, ParB family |  |
| bpy:Bphyt\_7011 | cobyrinic acid a,c-diamide synthase; K03496 chromosome partitioning protein |  |

  
**Neighborhood Representations for "bge:BC1002\_5113"**  

| ID | Annotation | EC number |
| --- | --- | --- |
| bge:BC1002\_5103 | cobyrinic acid ac-diamide synthase; K03496 chromosome partitioning protein |  |
| bge:BC1002\_5104 | parB-like partition protein; K03497 chromosome partitioning protein, ParB family |  |
| bge:BC1002\_5105 | initiator RepB protein |  |
| bge:BC1002\_5106 | hypothetical protein |  |
| bge:BC1002\_5107 | integrase family protein |  |
| bge:BC1002\_5108 | hypothetical protein |  |
| bge:BC1002\_5109 | XRE family transcriptional regulator |  |
| bge:BC1002\_5110 | 2-amino-3-ketobutyrate coenzyme A ligase (EC:2.3.1.29); K00639 glycine C-acetyltransferase [EC:2.3.1.29] | ec:2.3.1.29 |
| bge:BC1002\_5111 | L-threonine 3-dehydrogenase; K00060 threonine 3-dehydrogenase [EC:1.1.1.103] | ec:1.1.1.103 |
| bge:BC1002\_5112 | hypothetical protein |  |
| bge:BC1002\_5113 | TetR family transcriptional regulator |  |
| bge:BC1002\_5114 | hypothetical protein; K09958 hypothetical protein |  |
| bge:BC1002\_5115 | hypothetical protein |  |
| bge:BC1002\_5116 | general substrate transporter |  |
| bge:BC1002\_5117 | major facilitator superfamily protein |  |
| bge:BC1002\_5118 | ferredoxin; K03863 vanillate O-demethylase ferredoxin subunit [EC:1.14.13.82] | ec:1.14.13.82 |
| bge:BC1002\_5119 | LysR family transcriptional regulator |  |
| bge:BC1002\_5120 | Rieske (2Fe-2S) iron-sulfur domain-containing protein |  |
| bge:BC1002\_5121 | aromatic-ring-hydroxylating dioxygenase subunit beta |  |
| bge:BC1002\_5122 | 4-oxalocrotonate tautomerase; K01821 4-oxalocrotonate tautomerase [EC:5.3.2.6] | ec:5.3.2.6 |
| bge:BC1002\_5123 | PAS/PAC sensor signal transduction histidine kinase |  |

  
**Neighborhood Representations for "bgf:BC1003\_4755"**  

| ID | Annotation | EC number |
| --- | --- | --- |
| bgf:BC1003\_4745 | ribose-phosphate pyrophosphokinase (EC:2.7.6.1); K00948 ribose-phosphate pyrophosphokinase [EC:2.7.6.1] | ec:2.7.6.1 |
| bgf:BC1003\_4746 | hypothetical protein |  |
| bgf:BC1003\_4747 | nicotinate phosphoribosyltransferase; K00763 nicotinate phosphoribosyltransferase [EC:6.3.4.21] | ec:6.3.4.21 |
| bgf:BC1003\_4748 | PHP domain-containing protein; K02347 DNA polymerase (family X) |  |
| bgf:BC1003\_4749 | AMP-dependent synthetase and ligase |  |
| bgf:BC1003\_4750 | AMP-dependent synthetase and ligase |  |
| bgf:BC1003\_4751 | pseudogene |  |
| bgf:BC1003\_4752 | hypothetical protein |  |
| bgf:BC1003\_4753 | hypothetical protein |  |
| bgf:BC1003\_4754 | hypothetical protein; K09958 hypothetical protein |  |
| bgf:BC1003\_4755 | regulatory protein TetR |  |
| bgf:BC1003\_4756 | YceI family protein |  |
| bgf:BC1003\_4757 | L-threonine 3-dehydrogenase; K00060 threonine 3-dehydrogenase [EC:1.1.1.103] | ec:1.1.1.103 |
| bgf:BC1003\_4758 | 2-amino-3-ketobutyrate coenzyme A ligase (EC:2.3.1.29); K00639 glycine C-acetyltransferase [EC:2.3.1.29] | ec:2.3.1.29 |
| bgf:BC1003\_4759 | helix-turn-helix domain-containing protein |  |
| bgf:BC1003\_4760 | hypothetical protein |  |
| bgf:BC1003\_4761 | integrase family protein |  |
| bgf:BC1003\_4762 | hypothetical protein |  |
| bgf:BC1003\_4763 | hypothetical protein |  |
| bgf:BC1003\_4764 | initiator RepB protein |  |
| bgf:BC1003\_4765 | parB-like partition protein; K03497 chromosome partitioning protein, ParB family |  |

  
**Neighborhood Representations for "bph:Bphy\_4162"**  

| ID | Annotation | EC number |
| --- | --- | --- |
| bph:Bphy\_4152 | parB-like partition protein; K03497 chromosome partitioning protein, ParB family |  |
| bph:Bphy\_4153 | putative replication protein |  |
| bph:Bphy\_4154 | hypothetical protein |  |
| bph:Bphy\_4155 | hypothetical protein |  |
| bph:Bphy\_4156 | integrase family protein |  |
| bph:Bphy\_4157 | hypothetical protein |  |
| bph:Bphy\_4158 | XRE family transcriptional regulator |  |
| bph:Bphy\_4159 | 2-amino-3-ketobutyrate coenzyme A ligase (EC:2.3.1.29); K00639 glycine C-acetyltransferase [EC:2.3.1.29] | ec:2.3.1.29 |
| bph:Bphy\_4160 | tdh; L-threonine 3-dehydrogenase; K00060 threonine 3-dehydrogenase [EC:1.1.1.103] | ec:1.1.1.103 |
| bph:Bphy\_4161 | hypothetical protein |  |
| bph:Bphy\_4162 | TetR family transcriptional regulator |  |
| bph:Bphy\_4163 | hypothetical protein; K09958 hypothetical protein |  |
| bph:Bphy\_4164 | hypothetical protein |  |
| bph:Bphy\_4165 | LysR family transcriptional regulator |  |
| bph:Bphy\_4166 | alcohol dehydrogenase |  |
| bph:Bphy\_4167 | GAF sensor hybrid histidine kinase (EC:2.7.13.3) |  |
| bph:Bphy\_4168 | glutathione-dependent formaldehyde-activating GFA |  |
| bph:Bphy\_4169 | hypothetical protein |  |
| bph:Bphy\_4170 | hypothetical protein |  |
| bph:Bphy\_4171 | hypothetical protein |  |
| bph:Bphy\_4172 | short-chain dehydrogenase/reductase SDR |  |

  
**Neighborhood Representations for "bte:BTH\_II0010"**  

| ID | Annotation | EC number |
| --- | --- | --- |
| bte:BTH\_II2373 | plasmid replication protein |  |
| bte:BTH\_II0001 | hypothetical protein |  |
| bte:BTH\_II0002 | phage integrase |  |
| bte:BTH\_II0003 | hypothetical protein |  |
| bte:BTH\_II0004 | DNA-binding protein |  |
| bte:BTH\_II0005 | kbl; 2-amino-3-ketobutyrate CoA ligase (EC:2.3.1.29); K00639 glycine C-acetyltransferase [EC:2.3.1.29] | ec:2.3.1.29 |
| bte:BTH\_II0006 | tdh; L-threonine 3-dehydrogenase (EC:1.1.1.103); K00060 threonine 3-dehydrogenase [EC:1.1.1.103] | ec:1.1.1.103 |
| bte:BTH\_II0007 | K+-transporting ATPase subunit A |  |
| bte:BTH\_II0008 | acetyltransferase |  |
| bte:BTH\_II0009 | hypothetical protein |  |
| bte:BTH\_II0010 | TetR family transcriptional regulator |  |
| bte:BTH\_II0011 | hypothetical protein; K09958 hypothetical protein |  |
| bte:BTH\_II0012 | succinylglutamate desuccinylase / aspartoacylase |  |
| bte:BTH\_II0013 | tartrate dehydrogenase (EC:1.1.1.93); K07246 tartrate dehydrogenase/decarboxylase / D-malate dehydrogenase [EC:1.1.1.93 4.1.1.73 1.1.1.83] | ec:4.1.1.73 ec:1.1.1.83 ec:1.1.1.93 |
| bte:BTH\_II0014 | LysR family transcriptional regulator; K16135 LysR family transcriptional regulator, transcriptional activator for dmlA |  |
| bte:BTH\_II0015 | hypothetical protein |  |
| bte:BTH\_II0016 | glutathione S-transferase; K00799 glutathione S-transferase [EC:2.5.1.18] | ec:2.5.1.18 |
| bte:BTH\_II0017 | hypothetical protein |  |
| bte:BTH\_II0018 | patatin-like phospholipase; K07001 NTE family protein |  |
| bte:BTH\_II0019 | 3-hydroxybutyrate dehydrogenase (EC:1.1.1.30); K00019 3-hydroxybutyrate dehydrogenase [EC:1.1.1.30] | ec:1.1.1.30 |
| bte:BTH\_II0020 | acetoacetate decarboxylase (EC:4.1.1.4); K01574 acetoacetate decarboxylase [EC:4.1.1.4] | ec:4.1.1.4 |

  
**Neighborhood Representations for "bma:BMAA0008"**  

| ID | Annotation | EC number |
| --- | --- | --- |
| bma:BMAA2116 | ISBma2, transposase |  |
| bma:BMAA2117 | plasmid replication protein |  |
| bma:BMAA2118 | hypothetical protein |  |
| bma:BMAA0001 | hypothetical protein |  |
| bma:BMAA0002 | phage integrase family protein |  |
| bma:BMAA0003 | hypothetical protein |  |
| bma:BMAA0004 | DNA-binding protein |  |
| bma:BMAA0005 | kbl; 2-amino-3-ketobutyrate CoA ligase (EC:2.3.1.29); K00639 glycine C-acetyltransferase [EC:2.3.1.29] | ec:2.3.1.29 |
| bma:BMAA0006 | tdh; L-threonine 3-dehydrogenase (EC:1.1.1.103); K00060 threonine 3-dehydrogenase [EC:1.1.1.103] | ec:1.1.1.103 |
| bma:BMAA0007 | hypothetical protein |  |
| bma:BMAA0008 | TetR family transcriptional regulator |  |
| bma:BMAA0009 | hypothetical protein; K09958 hypothetical protein |  |
| bma:BMAA0010 | succinylglutamate desuccinylase |  |
| bma:BMAA0011 | ttuC; tartrate dehydrogenase (EC:1.1.1.93); K07246 tartrate dehydrogenase/decarboxylase / D-malate dehydrogenase [EC:1.1.1.93 4.1.1.73 1.1.1.83] | ec:4.1.1.73 ec:1.1.1.83 ec:1.1.1.93 |
| bma:BMAA0012 | LysR family transcriptional regulator; K16135 LysR family transcriptional regulator, transcriptional activator for dmlA |  |
| bma:BMAA0013 | hypothetical protein |  |
| bma:BMAA0014 | glutathione S-transferase; K00799 glutathione S-transferase [EC:2.5.1.18] | ec:2.5.1.18 |
| bma:BMAA0015 | hypothetical protein |  |
| bma:BMAA0016 | patatin-like phospholipase; K07001 NTE family protein |  |
| bma:BMAA0017 | bdhA-1; 3-hydroxybutyrate dehydrogenase (EC:1.1.1.30); K00019 3-hydroxybutyrate dehydrogenase [EC:1.1.1.30] | ec:1.1.1.30 |
| bma:BMAA0018 | adc; acetoacetate decarboxylase (EC:4.1.1.4); K01574 acetoacetate decarboxylase [EC:4.1.1.4] | ec:4.1.1.4 |

  
**Neighborhood Representations for "bml:BMA10229\_1436"**  

| ID | Annotation | EC number |
| --- | --- | --- |
| bml:BMA10229\_1426 | hypothetical protein |  |
| bml:BMA10229\_1427 | hypothetical protein |  |
| bml:BMA10229\_1428 | phage integrase family protein |  |
| bml:BMA10229\_1429 | hypothetical protein |  |
| bml:BMA10229\_1430 | DNA-binding protein |  |
| bml:BMA10229\_1431 | kbl; 2-amino-3-ketobutyrate CoA ligase; K00639 glycine C-acetyltransferase [EC:2.3.1.29] | ec:2.3.1.29 |
| bml:BMA10229\_1432 | tdh; L-threonine 3-dehydrogenase; K00060 threonine 3-dehydrogenase [EC:1.1.1.103] | ec:1.1.1.103 |
| bml:BMA10229\_1433 | hypothetical protein |  |
| bml:BMA10229\_1434 | hypothetical protein |  |
| bml:BMA10229\_1435 | hypothetical protein |  |
| bml:BMA10229\_1436 | TetR family transcriptional regulator |  |
| bml:BMA10229\_1437 | hypothetical protein; K09958 hypothetical protein |  |
| bml:BMA10229\_1438 | succinylglutamate desuccinylase |  |
| bml:BMA10229\_1439 | hypothetical protein |  |
| bml:BMA10229\_1440 | ttuC; tartrate dehydrogenase; K07246 tartrate dehydrogenase/decarboxylase / D-malate dehydrogenase [EC:1.1.1.93 4.1.1.73 1.1.1.83] | ec:4.1.1.73 ec:1.1.1.83 ec:1.1.1.93 |
| bml:BMA10229\_1441 | LysR family transcriptional regulator; K16135 LysR family transcriptional regulator, transcriptional activator for dmlA |  |
| bml:BMA10229\_1442 | hypothetical protein |  |
| bml:BMA10229\_1443 | glutathione S-transferase; K00799 glutathione S-transferase [EC:2.5.1.18] | ec:2.5.1.18 |
| bml:BMA10229\_1444 | hypothetical protein |  |
| bml:BMA10229\_1445 | hypothetical protein |  |
| bml:BMA10229\_1446 | patatin-like phospholipase; K07001 NTE family protein |  |

  
**Neighborhood Representations for "bmn:BMA10247\_A0011"**  

| ID | Annotation | EC number |
| --- | --- | --- |
| bmn:BMA10247\_A0001 | hypothetical protein |  |
| bmn:BMA10247\_A0002 | hypothetical protein |  |
| bmn:BMA10247\_A0003 | phage integrase family site specific recombinase |  |
| bmn:BMA10247\_A0004 | hypothetical protein |  |
| bmn:BMA10247\_A0005 | DNA-binding protein |  |
| bmn:BMA10247\_A0006 | kbl; 2-amino-3-ketobutyrate coenzyme A ligase (EC:2.3.1.29); K00639 glycine C-acetyltransferase [EC:2.3.1.29] | ec:2.3.1.29 |
| bmn:BMA10247\_A0007 | tdh; L-threonine 3-dehydrogenase (EC:1.1.1.103); K00060 threonine 3-dehydrogenase [EC:1.1.1.103] | ec:1.1.1.103 |
| bmn:BMA10247\_A0008 | hypothetical protein |  |
| bmn:BMA10247\_A0009 | putative lipoprotein |  |
| bmn:BMA10247\_A0010 | hypothetical protein |  |
| bmn:BMA10247\_A0011 | TetR family transcriptional regulator |  |
| bmn:BMA10247\_A0012 | hypothetical protein; K09958 hypothetical protein |  |
| bmn:BMA10247\_A0013 | succinylglutamate desuccinylase / aspartoacylase family protein |  |
| bmn:BMA10247\_A0014 | ttuC; tartrate dehydrogenase (EC:1.1.1.93); K07246 tartrate dehydrogenase/decarboxylase / D-malate dehydrogenase [EC:1.1.1.93 4.1.1.73 1.1.1.83] | ec:4.1.1.73 ec:1.1.1.83 ec:1.1.1.93 |
| bmn:BMA10247\_A0015 | LysR family transcriptional regulator; K16135 LysR family transcriptional regulator, transcriptional activator for dmlA |  |
| bmn:BMA10247\_A0016 | hypothetical protein |  |
| bmn:BMA10247\_A0017 | hypothetical protein |  |
| bmn:BMA10247\_A0018 | glutathione S-transferase; K00799 glutathione S-transferase [EC:2.5.1.18] | ec:2.5.1.18 |
| bmn:BMA10247\_A0019 | hypothetical protein |  |
| bmn:BMA10247\_A0020 | patatin family phospholipase; K07001 NTE family protein |  |
| bmn:BMA10247\_A0021 | bdhA-1; 3-hydroxybutyrate dehydrogenase (EC:1.1.1.30); K00019 3-hydroxybutyrate dehydrogenase [EC:1.1.1.30] | ec:1.1.1.30 |

  
**Neighborhood Representations for "bmv:BMASAVP1\_1156"**  

| ID | Annotation | EC number |
| --- | --- | --- |
| bmv:BMASAVP1\_1146 | hypothetical protein |  |
| bmv:BMASAVP1\_1147 | hypothetical protein |  |
| bmv:BMASAVP1\_1148 | phage integrase |  |
| bmv:BMASAVP1\_1149 | hypothetical protein |  |
| bmv:BMASAVP1\_1150 | DNA-binding protein |  |
| bmv:BMASAVP1\_1151 | kbl; 2-amino-3-ketobutyrate CoA ligase; K00639 glycine C-acetyltransferase [EC:2.3.1.29] | ec:2.3.1.29 |
| bmv:BMASAVP1\_1152 | tdh; L-threonine 3-dehydrogenase; K00060 threonine 3-dehydrogenase [EC:1.1.1.103] | ec:1.1.1.103 |
| bmv:BMASAVP1\_1153 | hypothetical protein |  |
| bmv:BMASAVP1\_1154 | lipoprotein |  |
| bmv:BMASAVP1\_1155 | hypothetical protein |  |
| bmv:BMASAVP1\_1156 | TetR family transcriptional regulator |  |
| bmv:BMASAVP1\_1157 | hypothetical protein; K09958 hypothetical protein |  |
| bmv:BMASAVP1\_1158 | succinylglutamate desuccinylase / aspartoacylase |  |
| bmv:BMASAVP1\_1159 | ttuC; tartrate dehydrogenase; K07246 tartrate dehydrogenase/decarboxylase / D-malate dehydrogenase [EC:1.1.1.93 4.1.1.73 1.1.1.83] | ec:4.1.1.73 ec:1.1.1.83 ec:1.1.1.93 |
| bmv:BMASAVP1\_1160 | LysR family transcriptional regulator; K16135 LysR family transcriptional regulator, transcriptional activator for dmlA |  |
| bmv:BMASAVP1\_1161 | hypothetical protein |  |
| bmv:BMASAVP1\_1162 | hypothetical protein |  |
| bmv:BMASAVP1\_1163 | hypothetical protein |  |
| bmv:BMASAVP1\_1164 | glutathione S-transferase; K00799 glutathione S-transferase [EC:2.5.1.18] | ec:2.5.1.18 |
| bmv:BMASAVP1\_1165 | hypothetical protein |  |
| bmv:BMASAVP1\_1166 | patatin family phospholipase; K07001 NTE family protein |  |

  
**Neighborhood Representations for "bpd:BURPS668\_A0010"**  

| ID | Annotation | EC number |
| --- | --- | --- |
| bpd:BURPS668\_A3293 | hypothetical protein |  |
| bpd:BURPS668\_A3294 | hypothetical protein |  |
| bpd:BURPS668\_A0001 | chromosome segregation ATPase |  |
| bpd:BURPS668\_A0002 | integrase |  |
| bpd:BURPS668\_A0003 | Serine/threonine protein kinase |  |
| bpd:BURPS668\_A0004 | DNA-binding protein |  |
| bpd:BURPS668\_A0005 | kbl; 2-amino-3-ketobutyrate coenzyme A ligase (EC:2.3.1.29); K00639 glycine C-acetyltransferase [EC:2.3.1.29] | ec:2.3.1.29 |
| bpd:BURPS668\_A0006 | tdh; L-threonine 3-dehydrogenase (EC:1.1.1.103); K00060 threonine 3-dehydrogenase [EC:1.1.1.103] | ec:1.1.1.103 |
| bpd:BURPS668\_A0007 | hypothetical protein |  |
| bpd:BURPS668\_A0008 | hypothetical protein |  |
| bpd:BURPS668\_A0010 | TetR family transcriptional regulator |  |
| bpd:BURPS668\_A0012 | hypothetical protein; K09958 hypothetical protein |  |
| bpd:BURPS668\_A0011 | ttbk1 protein |  |
| bpd:BURPS668\_A0013 | succinylglutamate desuccinylase |  |
| bpd:BURPS668\_A0014 | hypothetical protein |  |
| bpd:BURPS668\_A0015 | tartrate dehydrogenase (EC:1.1.1.93); K07246 tartrate dehydrogenase/decarboxylase / D-malate dehydrogenase [EC:1.1.1.93 4.1.1.73 1.1.1.83] | ec:4.1.1.73 ec:1.1.1.83 ec:1.1.1.93 |
| bpd:BURPS668\_A0017 | hypothetical protein |  |
| bpd:BURPS668\_A0018 | hypothetical protein |  |
| bpd:BURPS668\_A0019 | glutathione S-transferase family protein (EC:2.5.1.18); K00799 glutathione S-transferase [EC:2.5.1.18] | ec:2.5.1.18 |
| bpd:BURPS668\_A0020 | hypothetical protein |  |
| bpd:BURPS668\_A0021 | hypothetical protein |  |

  
**Over-represented Enzyme Summary**: Table of E.C. identified protein in the "Neighborhood Representation" ranked by frequency of occurrence  

| EC number | Frequency | Annotation | Reactions |
| --- | --- | --- | --- |
| ec:2.3.1.29 | 11 | glycine C-acetyltransferase; 2-amino-3-ketobutyrate CoA ligase; 2-amino-3-ketobutyrate coenzyme A ligase; 2-amino-3-ketobutyrate-CoA ligase; glycine acetyltransferase; aminoacetone synthase; aminoacetone synthetase; KBL; AKB ligase | acetyl-CoA + glycine = CoA + L-2-amino-3-oxobutanoate [RN:R00371] |
| ec:1.1.1.103 | 11 | L-threonine 3-dehydrogenase; L-threonine dehydrogenase; threonine 3-dehydrogenase; threonine dehydrogenase; TDH | L-threonine + NAD+ = L-2-amino-3-oxobutanoate + NADH + H+ [RN:R01465] |
| ec:1.1.1.83 | 6 | D-malate dehydrogenase (decarboxylating); D-malate dehydrogenase; D-malic enzyme; bifunctional L(+)-tartrate dehydrogenase-D(+)-malate (decarboxylating) | (R)-malate + NAD+ = pyruvate + CO2 + NADH [RN:R00215] |
| ec:2.5.1.18 | 6 | glutathione transferase; glutathione S-transferase; glutathione S-alkyltransferase; glutathione S-aryltransferase; S-(hydroxyalkyl)glutathione lyase; glutathione S-aralkyltransferase; glutathione S-alkyl transferase; GST | RX + glutathione = HX + R-S-glutathione [RN:R03522 R08511 R08512] |
| ec:1.1.1.93 | 6 | tartrate dehydrogenase; mesotartrate dehydrogenase | tartrate + NAD+ = oxaloglycolate + NADH + H+ [RN:R02545 R06180] |
| ec:4.1.1.73 | 6 | tartrate decarboxylase; (R,R)-tartrate carboxy-lyase | (R,R)-tartrate = D-glycerate + CO2 [RN:R01751] |
| ec:1.1.1.30 | 3 | 3-hydroxybutyrate dehydrogenase; NAD-beta-hydroxybutyrate dehydrogenase; hydroxybutyrate oxidoreductase; beta-hydroxybutyrate dehydrogenase; D-beta-hydroxybutyrate dehydrogenase; D-3-hydroxybutyrate dehydrogenase; D-(-)-3-hydroxybutyrate dehydrogenase; beta-hydroxybutyric acid dehydrogenase; 3-D-hydroxybutyrate dehydrogenase; beta-hydroxybutyric dehydrogenase | (R)-3-hydroxybutanoate + NAD+ = acetoacetate + NADH + H+ [RN:R01361] |
| ec:4.1.1.4 | 2 | acetoacetate decarboxylase; acetoacetic acid decarboxylase; acetoacetate carboxy-lyase | acetoacetate + H+ = acetone + CO2 [RN:R01366] |
| ec:2.7.13.3 | 1 | histidine kinase; EnvZ; histidine kinase (ambiguous); histidine protein kinase (ambiguous); protein histidine kinase (ambiguous); protein kinase (histidine) (ambiguous); HK1; HP165; Sln1p | ATP + protein L-histidine = ADP + protein N-phospho-L-histidine |
| ec:6.3.4.21 | 1 | nicotinate phosphoribosyltransferase; niacin ribonucleotidase; nicotinic acid mononucleotide glycohydrolase; nicotinic acid mononucleotide pyrophosphorylase; nicotinic acid phosphoribosyltransferase; nicotinate-nucleotide:diphosphate phospho-alpha-D-ribosyltransferase | nicotinate + 5-phospho-alpha-D-ribose 1-diphosphate + ATP + H2O = beta-nicotinate D-ribonucleotide + diphosphate + ADP + phosphate [RN:R01724] |
| ec:1.14.13.82 | 1 | vanillate monooxygenase; 4-hydroxy-3-methoxybenzoate demethylase; vanillate demethylase | vanillate + O2 + NADH + H+ = 3,4-dihydroxybenzoate + NAD+ + H2O + formaldehyde [RN:R05274] |
| ec:2.7.6.1 | 1 | ribose-phosphate diphosphokinase; ribose-phosphate pyrophosphokinase; PRPP synthetase; phosphoribosylpyrophosphate synthetase; PPRibP synthetase; PP-ribose P synthetase; 5-phosphoribosyl-1-pyrophosphate synthetase; 5-phosphoribose pyrophosphorylase; 5-phosphoribosyl-alpha-1-pyrophosphate synthetase; phosphoribosyl-diphosphate synthetase; phosphoribosylpyrophosphate synthase; pyrophosphoribosylphosphate synthetase; ribophosphate pyrophosphokinase; ribose-5-phosphate pyrophosphokinase | ATP + D-ribose 5-phosphate = AMP + 5-phospho-alpha-D-ribose 1-diphosphate [RN:R01049] |
| ec:5.3.2.6 | 1 | 2-hydroxymuconate tautomerase; 4-oxalocrotonate tautomerase (misleading); 4-oxalocrotonate isomerase (misleading); cnbG (gene name); praC (gene name); xylH (gene name) | (2Z,4E)-2-hydroxyhexa-2,4-dienedioate = (3E)-2-oxohex-3-enedioate |

  
**Over-represented Metabolite Summary**: Collection of the metabolites identified as substrates or products of the proteins representaed the "Over-represented Enzyme Summary" ranked by frequency of occurrence  

| ID | Structure | Name | Frequency | EC |
| --- | --- | --- | --- | --- |
| cpd:C00080 |  | H+; Hydron | 27 | ec:6.3.4.21 ec:1.1.1.103 ec:4.1.1.73 ec:1.1.1.30 ec:1.1.1.83 ec:1.1.1.93 ec:2.5.1.18 |
| cpd:C03508 |  | L-2-Amino-3-oxobutanoic acid; L-2-Amino-3-oxobutanoate; L-2-Amino-acetoacetate; (S)-2-Amino-3-oxobutanoic acid | 22 | ec:1.1.1.103 ec:2.3.1.29 |
| cpd:C00004 |  | NADH; DPNH; Reduced nicotinamide adenine dinucleotide | 20 | ec:1.1.1.103 ec:4.1.1.73 ec:1.1.1.30 ec:1.1.1.83 ec:1.1.1.93 |
| cpd:C00003 |  | NAD+; NAD; Nicotinamide adenine dinucleotide; DPN; Diphosphopyridine nucleotide; Nadide; beta-NAD+ | 20 | ec:1.1.1.103 ec:4.1.1.73 ec:1.1.1.30 ec:1.1.1.83 ec:1.1.1.93 |
| cpd:C00010 |  | CoA; Coenzyme A; CoA-SH | 11 | ec:2.3.1.29 |
| cpd:C00037 |  | Glycine; Aminoacetic acid; Gly | 11 | ec:2.3.1.29 |
| cpd:C00188 |  | L-Threonine; 2-Amino-3-hydroxybutyric acid | 11 | ec:1.1.1.103 |
| cpd:C00024 |  | Acetyl-CoA; Acetyl coenzyme A | 11 | ec:2.3.1.29 |
| cpd:C00011 |  | CO2; Carbon dioxide | 8 | ec:4.1.1.73 ec:1.1.1.83 ec:1.1.1.93 ec:4.1.1.4 |
| cpd:C00001 |  | H2O; Water | 7 | ec:6.3.4.21 ec:2.5.1.18 |
| cpd:C14863 |  | 2-(S-Glutathionyl)acetyl glutathione | 6 | ec:2.5.1.18 |
| cpd:C14861 |  | S-(2,2-Dichloro-1-hydroxy)ethyl glutathione | 6 | ec:2.5.1.18 |
| cpd:C14859 |  | Chloroacetyl chloride | 6 | ec:2.5.1.18 |
| cpd:C14858 |  | 2,2-Dichloroacetaldehyde | 6 | ec:2.5.1.18 |
| cpd:C14857 |  | 1,1-Dichloroethylene epoxide; 2,2-Dichlorooxirane | 6 | ec:2.5.1.18 |
| cpd:C14856 |  | 7,8-Dihydro-7-hydroxy-8-S-glutathionyl-benzo[a]pyrene | 6 | ec:2.5.1.18 |
| cpd:C14855 |  | 4,5-Dihydro-4-hydroxy-5-S-glutathionyl-benzo[a]pyrene | 6 | ec:2.5.1.18 |
| cpd:C14852 |  | Benzo[a]pyrene-7,8-diol; Benzo[a]pyrene-7,8-dihydrodiol | 6 | ec:2.5.1.18 |
| cpd:C14851 |  | Benzo[a]pyrene-4,5-oxide; Benzo[a]pyrene-4,5-epoxide | 6 | ec:2.5.1.18 |
| cpd:C00462 |  | Halide; Hydrogen halide; HX; Halo acid | 6 | ec:2.5.1.18 |
| cpd:C14848 |  | 2,3-Dihydro-2-S-glutathionyl-3-hydroxy bromobenzene | 6 | ec:2.5.1.18 |
| cpd:C14847 |  | 3,4-Dihydro-3-hydroxy-4-S-glutathionyl bromobenzene | 6 | ec:2.5.1.18 |
| cpd:C14840 |  | Bromobenzene-2,3-oxide; Bromobenzene-2,3-epoxide | 6 | ec:2.5.1.18 |
| cpd:C00552 |  | meso-Tartaric acid; meso-Tartrate | 6 | ec:4.1.1.73 ec:1.1.1.83 ec:1.1.1.93 |
| cpd:C13645 |  | Hydrobromic acid; HBr | 6 | ec:2.5.1.18 |
| cpd:C00258 |  | D-Glycerate; Glycerate; (R)-Glycerate; Glyceric acid | 6 | ec:4.1.1.73 ec:1.1.1.83 ec:1.1.1.93 |
| cpd:C03459 |  | 2-Hydroxy-3-oxosuccinate; Oxaloglycolate | 6 | ec:4.1.1.73 ec:1.1.1.83 ec:1.1.1.93 |
| cpd:C14839 |  | Bromobenzene-3,4-oxide; Bromobenzene-3,4-epoxide | 6 | ec:2.5.1.18 |
| cpd:C00051 |  | Glutathione; 5-L-Glutamyl-L-cysteinylglycine; N-(N-gamma-L-Glutamyl-L-cysteinyl)glycine; gamma-L-Glutamyl-L-cysteinyl-glycine; GSH; Reduced glutathione | 6 | ec:2.5.1.18 |
| cpd:C14793 |  | (1R)-Glutathionyl-(2R)-hydroxy-1,2-dihydronaphthalene | 6 | ec:2.5.1.18 |
| cpd:C14792 |  | (1S)-Hydroxy-(2S)-glutathionyl-1,2-dihydronaphthalene | 6 | ec:2.5.1.18 |
| cpd:C14791 |  | (1R)-Hydroxy-(2R)-glutathionyl-1,2-dihydronaphthalene | 6 | ec:2.5.1.18 |
| cpd:C00898 |  | (R,R)-Tartaric acid; (R,R)-Tartrate; L-Tartaric acid; Tartaric acid; Tartrate; 2,3-Dihydroxybutanedioic acid; (2R,3R)-Tartaric acid; (+)-Tartaric acid | 6 | ec:4.1.1.73 ec:1.1.1.83 ec:1.1.1.93 |
| cpd:C07645 |  | Aldophosphamide | 6 | ec:2.5.1.18 |
| cpd:C14787 |  | (1S,2R)-Naphthalene 1,2-oxide; (1S,2R)-Naphthalene epoxide | 6 | ec:2.5.1.18 |
| cpd:C11583 |  | 4-Glutathionyl cyclophosphamide | 6 | ec:2.5.1.18 |
| cpd:C14786 |  | (1R,2S)-Naphthalene 1,2-oxide; (1R,2S)-Naphthalene epoxide | 6 | ec:2.5.1.18 |
| cpd:C00497 |  | (R)-Malate; D-Malate; D-Malic acid | 6 | ec:4.1.1.73 ec:1.1.1.83 ec:1.1.1.93 |
| cpd:C06790 |  | Trichloroethene; Trichloroethylene; TCE | 6 | ec:2.5.1.18 |
| cpd:C11088 |  | 1,2-Dibromoethane; Ethylene dibromide | 6 | ec:2.5.1.18 |
| cpd:C14874 |  | Glutathione episulfonium ion | 6 | ec:2.5.1.18 |
| cpd:C14871 |  | S-(Formylmethyl)glutathione | 6 | ec:2.5.1.18 |
| cpd:C14870 |  | 2-Bromoacetaldehyde | 6 | ec:2.5.1.18 |
| cpd:C19586 |  | Aflatoxin B1-exo-8,9-epoxide; 2,3-Epoxyaflatoxin B1 | 6 | ec:2.5.1.18 |
| cpd:C01327 |  | Hydrochloric acid; HCl; Hydrogen chloride; Hydrochloride | 6 | ec:2.5.1.18 |
| cpd:C11278 |  | Aflatoxin B1exo-8,9-epoxide-GSH; 8,9-Dihydro-8-(S-glutathionyl)-9-hydroxyaflatoxin B1 | 6 | ec:2.5.1.18 |
| cpd:C01322 |  | RX; Organic halide | 6 | ec:2.5.1.18 |
| cpd:C02320 |  | R-S-Glutathione | 6 | ec:2.5.1.18 |
| cpd:C14806 |  | 1-Nitro-5-glutathionyl-6-hydroxy-5,6-dihydronaphthalene | 6 | ec:2.5.1.18 |
| cpd:C14805 |  | 1-Nitro-5-hydroxy-6-glutathionyl-5,6-dihydronaphthalene | 6 | ec:2.5.1.18 |
| cpd:C14804 |  | 1-Nitro-7-glutathionyl-8-hydroxy-7,8-dihydronaphthalene | 6 | ec:2.5.1.18 |
| cpd:C14803 |  | 1-Nitro-7-hydroxy-8-glutathionyl-7,8-dihydronaphthalene | 6 | ec:2.5.1.18 |
| cpd:C14868 |  | S-(1,2-Dichlorovinyl)glutathione; DCVG | 6 | ec:2.5.1.18 |
| cpd:C14802 |  | 1-Nitronaphthalene-7,8-oxide | 6 | ec:2.5.1.18 |
| cpd:C00022 |  | Pyruvate; Pyruvic acid; 2-Oxopropanoate; 2-Oxopropanoic acid; Pyroracemic acid | 6 | ec:4.1.1.73 ec:1.1.1.83 ec:1.1.1.93 |
| cpd:C14800 |  | 1-Nitronaphthalene-5,6-oxide | 6 | ec:2.5.1.18 |
| cpd:C14865 |  | 2-(S-Glutathionyl)acetyl chloride | 6 | ec:2.5.1.18 |
| cpd:C14864 |  | S-(2-Chloroacetyl)glutathione | 6 | ec:2.5.1.18 |
| cpd:C00164 |  | Acetoacetate; 3-Oxobutanoic acid; beta-Ketobutyric acid; Acetoacetic acid | 5 | ec:1.1.1.30 ec:4.1.1.4 |
| cpd:C01089 |  | (R)-3-Hydroxybutanoate; (R)-3-Hydroxybutanoic acid; (R)-3-Hydroxybutyric acid; D-beta-Hydroxybutyric acid | 3 | ec:1.1.1.30 |
| cpd:C00119 |  | 5-Phospho-alpha-D-ribose 1-diphosphate; 5-Phosphoribosyl diphosphate; 5-Phosphoribosyl 1-pyrophosphate; PRPP | 2 | ec:6.3.4.21 ec:2.7.6.1 |
| cpd:C00207 |  | Acetone; Dimethyl ketone; 2-Propanone | 2 | ec:4.1.1.4 |
| cpd:C00002 |  | ATP; Adenosine 5'-triphosphate | 2 | ec:6.3.4.21 ec:2.7.6.1 |
| cpd:C00020 |  | AMP; Adenosine 5'-monophosphate; Adenylic acid; Adenylate; 5'-AMP; 5'-Adenylic acid; 5'-Adenosine monophosphate; Adenosine 5'-phosphate | 1 | ec:2.7.6.1 |
| cpd:C00117 |  | D-Ribose 5-phosphate; Ribose 5-phosphate | 1 | ec:2.7.6.1 |
| cpd:C00013 |  | Diphosphate; Diphosphoric acid; Pyrophosphate; Pyrophosphoric acid; PPi | 1 | ec:6.3.4.21 |
| cpd:C07479 |  | 2-Oxo-5-methyl-cis-muconate | 1 | ec:5.3.2.6 |
| cpd:C07478 |  | 2-Hydroxy-5-methyl-cis,cis-muconate | 1 | ec:5.3.2.6 |
| cpd:C02501 |  | 2-Hydroxymuconate | 1 | ec:5.3.2.6 |
| cpd:C00009 |  | Orthophosphate; Phosphate; Phosphoric acid; Orthophosphoric acid | 1 | ec:6.3.4.21 |
| cpd:C00008 |  | ADP; Adenosine 5'-diphosphate | 1 | ec:6.3.4.21 |
| cpd:C00253 |  | Nicotinate; Nicotinic acid; Niacin; 3-Pyridinecarboxylic acid | 1 | ec:6.3.4.21 |
| cpd:C03453 |  | gamma-Oxalocrotonate; (Z)-5-Oxohex-2-enedioate; 4-Oxalocrotonate | 1 | ec:5.3.2.6 |
| cpd:C01185 |  | Nicotinate D-ribonucleotide; beta-Nicotinate D-ribonucleotide; Nicotinate ribonucleotide; Nicotinic acid ribonucleotide | 1 | ec:6.3.4.21 |

  
**Over-represented Pathway Summary**: Collection of the KEGG metabolic pathways containing the proteins identified in the "Over-represented Metabolite Summary" ranked by the highest number of hits per pathway  

| Pathway ID | EC | EC Frequency | Name |
| --- | --- | --- | --- |
| map00260 | ec:1.1.1.103 ec:2.3.1.29 | 22 | path:map00260 Glycine, serine and threonine metabolism |
| map00650 | ec:1.1.1.83 ec:1.1.1.30 | 9 | path:map00650 Butanoate metabolism |
| map00630 | ec:1.1.1.93 | 6 | path:map00630 Glyoxylate and dicarboxylate metabolism |
| map00982 | ec:2.5.1.18 | 6 | path:map00982 Drug metabolism - cytochrome P450 |
| map00980 | ec:2.5.1.18 | 6 | path:map00980 Metabolism of xenobiotics by cytochrome P450 |
| map00480 | ec:2.5.1.18 | 6 | path:map00480 Glutathione metabolism |
| map00072 | ec:4.1.1.4 ec:1.1.1.30 | 5 | path:map00072 Synthesis and degradation of ketone bodies |
| map00640 | ec:4.1.1.4 | 2 | path:map00640 Propanoate metabolism |
| map00362 | ec:5.3.2.6 | 1 | path:map00362 Benzoate degradation |
| map00627 | ec:1.14.13.82 | 1 | path:map00627 Aminobenzoate degradation |
| map00760 | ec:6.3.4.21 | 1 | path:map00760 Nicotinate and nicotinamide metabolism |
| map00622 | ec:5.3.2.6 | 1 | path:map00622 Xylene degradation |
| map00621 | ec:5.3.2.6 | 1 | path:map00621 Dioxin degradation |
| map00030 | ec:2.7.6.1 | 1 | path:map00030 Pentose phosphate pathway |
| map00230 | ec:2.7.6.1 | 1 | path:map00230 Purine metabolism |

  
Analysis performed on 2014/11/20 15:46:47
