# Supplementary material for: Automated genomic context analysis and experimental validation platform for discovery of prokaryote transcriptional regulator functions
Source: BMC Genomics. 2014 Dec 18;15(1):1142. doi: 10.1186/1471-2164-15-1142 (PMC4349456; doi:10.1186/1471-2164-15-1142)
Supplement: Supplementary file 1 — Additional file 1: Code. Zip folder containing all files required to run the Java™ application. The files need to be extracted to the same folder and then the FunctionDiscoveryV1.0.jar interface can be launched. For detailed instructions on how to use the interface please refer to the Function Discovery V1.0, a gene neighborhood analysis tool section in the Results part of the main text. (ZIP 415 KB) [file 12864_2014_6995_MOESM1_ESM.zip › codeF/readme 2.docx]

Function Discovery V1.0 takes as input a transcription regulator (TR) or any protein, number of similar genes, number of nearby genes (sg) and amino acid similarity cutoff (aa). The software can be run through GUI as shown in Figure 1.


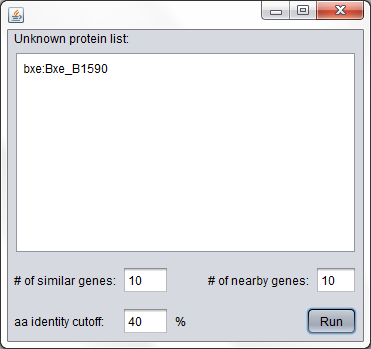


Figure 1. GUI of function discovery.

The software can also be run through command line. Command line template is as follows.

Java –jar FunctionDiscoveryV1.0.jar name n m p

where name is KEGG ID of transcription regulator, n is number of similar genes, m is number of nearby genes and p is amino acid identify cutoff.

The command line for figure 1 is as follows.

Java –jar FunctionDiscoveryV1.0.jar “bxe:Bxe_B1590” 10 10 40

The software uses the KEGG Sequence Similarity DataBase (SSDB) to find a set of genes similar to our target TR. For each similar gene at a position in the chromosome, the software found a set of nearby genes upstream and downstream from the position. From those nearby genes, the software identified a subset of genes that encoded enzymes. The metabolic reactions for those enzymes and recorded their metabolic pathways are collected. For each metabolic reaction, its substrates and products (metabolites involved) are identified. The metabolites, metabolic reactions and metabolic pathways identified from the whole set of similar genes were collected. The over-representation of those metabolites, metabolic reactions and pathways are counted. Over-represented metabolic reactions are more likely to be co-regulated and belong to the same metabolic pathway. Flow chart of the software is shown in Figure 2.

Figure 2. Flowchart of the steps taken by Function Discovery V1.0.
