# Supplementary material for: Automated genomic context analysis and experimental validation platform for discovery of prokaryote transcriptional regulator functions
Source: BMC Genomics. 2014 Dec 18;15(1):1142. doi: 10.1186/1471-2164-15-1142 (PMC4349456; doi:10.1186/1471-2164-15-1142)
Supplement: Supplementary file 2 — Additional file 2: Result BetI. Function Discovery V1.0 output (.html format) for the glycine betaine biosynthesis regulator (BetI, Bxe_B1590). For detailed instructions on how to analyze the results please refer to the Function Discovery V1.0, a gene neighborhood analysis tool section in the Results part of the main text. (HTML 596 KB) [file 12864_2014_6995_MOESM2_ESM.html]

```
ENTRY       Bxe_B1590         CDS       T00340
DEFINITION  transcriptional regulator BetI
ORTHOLOGY   K02167  TetR/AcrR family transcriptional regulator, transcriptional repressor of bet genes
ORGANISM    bxe  Burkholderia xenovorans
POSITION    2:complement(1614524..1615111)
MOTIF       Pfam: TetR_C_6 TetR_N
DBLINKS     NCBI-GI: 91778516
            NCBI-GeneID: 4007921
            JGI: BxeB1590
            UniProt: Q13NG5
AASEQ       195
            MPKLGMREIRRAQLIDATLLTIDQTGLAGTTLASVAQRASISTGIVSHYFGDKDGLLEAT
            MRHVLRDLWQATSRRRRAARADPRSKLRAVVAANFDAEQTSGPVMKTWLAFWSESMHKPQ
            LRRLQHVNTRRLYSNLCADFSKALPRAAARRAASGLAALIDGLWLRGALSGEPFDTKAAL
            RTANDYIDLVLASRE
NTSEQ       588
            atgcccaaactcggaatgcgcgaaatccgccgcgcgcaactgatcgacgccacgcttctc
            acgatcgaccagaccggtctcgcggggaccacgctggcttcggtcgcgcagcgcgcgagc
            atttccaccggcattgtcagccactatttcggcgacaaggacggtttgctcgaagccacc
            atgcgtcacgtgctgcgcgatctgtggcaggccacctcgcgccggcgccgggcggccagg
            gcggacccgcgttcgaaattgcgcgcggtggtcgccgcgaatttcgacgccgagcagacc
            agcggcccggtcatgaaaacctggctggcgttctggtccgagagcatgcacaagccgcaa
            ttgcggcggctgcaacacgtgaacacgcgccggctgtattcgaacctgtgcgcggatttt
            tccaaagccttgccgcgtgcggcggcgcgccgtgcggcaagcggccttgcggcgctgatc
            gacgggctgtggttgcgcggtgcgctgtcgggcgaaccgttcgacaccaaagcggcgctg
            cgcacggccaacgactatatcgacctggtgctcgcatcgcgcgagtaa
///
```

  
**Homolog ID**: Table of closest homologs  

```
                 Homologs                                       len   identity overlap
---------------------------------------------------------------------------------
bpy:Bphyt_5060 transcriptional regulator BetI           K     195     0.959    195 
bgf:BC1003_5873 transcriptional repressor BetI          K     195     0.949    195 
bph:Bphy_3308 transcriptional regulator BetI            K     196     0.859    192 
bmj:BMULJ_04979 transcriptional regulator BetI          K     194     0.819    193 
bmu:Bmul_3538 transcriptional regulator BetI            K     194     0.819    193 
bcj:BCAM2341 transcriptional regulator BetI             K     194     0.824    193 
bte:BTH_II1074 transcriptional regulator BetI           K     195     0.830    194 
bch:Bcen2424_5098 transcriptional regulator BetI        K     194     0.824    193 
bcm:Bcenmc03_5184 transcriptional regulator BetI        K     194     0.824    193 
bcn:Bcen_3270 transcriptional regulator BetI            K     194     0.824    193 
bvi:Bcep1808_3429 transcriptional regulator BetI        K     194     0.813    193 
bur:Bcep18194_B0555 transcriptional regulator BetI      K     194     0.813    193 
bac:BamMC406_5035 transcriptional regulator BetI        K     194     0.813    193 
bam:Bamb_4510 transcriptional regulator BetI            K     194     0.813    193 
bml:BMA10229_0181 transcriptional regulator BetI        K     195     0.820    194 
bmn:BMA10247_A1426 transcriptional regulator BetI       K     195     0.820    194 
bmv:BMASAVP1_0466 transcriptional regulator BetI        K     202     0.820    194 
bpd:BURPS668_A1923 transcriptional regulator BetI       K     195     0.820    194 
bpl:BURPS1106A_A1835 transcriptional regulator BetI     K     195     0.820    194 
bpm:BURPS1710b_A0375 transcriptional regulator BetI     K     202     0.820    194 
bps:BPSS1353 transcriptional regulator BetI             K     195     0.820    194 
bma:BMAA0916 transcriptional regulator BetI             K     202     0.814    194 
bgl:bglu_2g05640 transcriptional regulator BetI         K     201     0.802    192 
pmy:Pmen_0483 transcriptional regulator BetI            K     196     0.620    192 
pen:PSEEN0374 transcriptional regulator BetI            K     218     0.566    196 
pae:PA5374 BetI family transcriptional regulator        K     197     0.602    196 
pag:PLES_57691 transcriptional regulator BetI           K     197     0.602    196 
pap:PSPA7_6159 transcriptional regulator BetI           K     197     0.602    196 
pau:PA14_70970 transcriptional regulator BetI           K     197     0.602    196 
psb:Psyr_4734 transcriptional regulator BetI            K     197     0.575    193 
psp:PSPPH_4768 transcriptional regulator BetI           K     197     0.575    193 
pst:PSPTO_0440 regulatory protein BetI                  K     197     0.575    193 
pfl:PFL_5766 transcriptional regulator BetI             K     197     0.561    196 
ppf:Pput_4935 transcriptional regulator BetI            K     218     0.564    195 
ppw:PputW619_0403 transcriptional regulator BetI        K     218     0.556    196 
hel:HELO_1861 BetI family transcriptional regulator     K     210     0.561    196 
ppg:PputGB1_5113 transcriptional regulator BetI         K     218     0.559    195 
pfs:PFLU5684 transcriptional regulator BetI             K     197     0.559    195 
pfo:Pfl01_5242 transcriptional regulator BetI           K     197     0.549    195 
xbo:XBJ1_3306 TetR family transcriptional regulator     K     198     0.552    194 
ddd:Dda3937_03413 DNA-binding transcriptional regulator K     204     0.577    194 
xne:XNC1_1246 osmotic stress transcriptional repressor  K     198     0.541    194 
spe:Spro_1513 transcriptional regulator BetI            K     198     0.568    192 
smt:Smal_1834 TetR family transcriptional regulator     K     196     0.545    191 
sml:Smlt2239 TetR family regulatory protein             K     211     0.545    191 
ypa:YPA_1075 transcriptional regulator BetI             K     198     0.550    189 
ypb:YPTS_1277 transcriptional regulator BetI            K     198     0.550    189 
ype:YPO1167 transcriptional regulator BetI              K     198     0.550    189 
ypi:YpsIP31758_2829 BetI family transcriptional regulat K     198     0.550    189 
ypn:YPN_2834 transcriptional regulator BetI             K     198     0.550    189 
ypp:YPDSF_2530 transcriptional regulator BetI           K     198     0.550    189 
yps:YPTB1197 transcriptional regulator BetI             K     198     0.550    189 
ypz:YPZ3_1057 transcriptional regulator BetI            K     198     0.550    189 
ypm:YP_0992 transcriptional regulator BetI              K     206     0.545    189 
csa:Csal_1516 transcriptional regulator BetI            K     197     0.572    187 
pmr:PMI1461 TetR family transcriptional regulator       K     200     0.547    192 
ebi:EbC_19400 HTH-type transcriptional regulator        K     198     0.578    173 
pct:PC1_2556 TetR family transcriptional regulator      K     195     0.556    189 
pam:PANA_2166 BetI                                      K     200     0.554    193 
eca:ECA1744 transcriptional regulator BetI              K     195     0.550    189 
pva:Pvag_1614 HTH-type transcriptional regulator betI   K     197     0.560    193 
esa:ESA_02047 transcriptional regulator BetI            K     202     0.539    193 
eta:ETA_17830 HTH-type transcriptional regulator        K     199     0.554    175 
eoh:ECO103_0290 DNA-binding transcriptional repressor B K     195     0.543    188 
ctu:CTU_19220 transcriptional regulator BetI            K     198     0.534    193 
cko:CKO_02582 transcriptional regulator BetI            K     203     0.537    188 
eck:EC55989_0315 transcriptional regulator BetI         K     195     0.537    188 
ecm:EcSMS35_0344 transcriptional regulator BetI         K     195     0.537    188 
ecr:ECIAI1_0310 transcriptional regulator BetI          K     195     0.537    188 
ecw:EcE24377A_0328 transcriptional regulator BetI       K     195     0.537    188 
ecx:EcHS_A0372 transcriptional regulator BetI           K     195     0.537    188 
ecy:ECSE_0334 transcriptional regulator BetI            K     195     0.537    188 
eoi:ECO111_0347 DNA-binding transcriptional repressor B K     195     0.537    188 
eoj:ECO26_0347 transcriptional regulator BetI           K     195     0.537    188 
sfv:SFV_0324 transcriptional regulator BetI             K     195     0.537    188 
ecc:c0433 transcriptional regulator BetI                K     201     0.537    188 
ecd:ECDH10B_0300 transcriptional regulator BetI         K     195     0.537    188 
ece:Z0400 transcriptional regulator BetI                K     195     0.537    188 
ecf:ECH74115_0375 transcriptional regulator BetI        K     195     0.537    188 
ecg:E2348C_0275 transcriptional regulator BetI          K     195     0.537    188 
ecj:Y75_p0303 DNA-binding transcriptional repressor     K     195     0.537    188 
ecl:EcolC_3310 transcriptional regulator BetI           K     195     0.537    188 
eco:b0313 DNA-binding transcriptional repressor         K     195     0.537    188 
ecs:ECs0359 transcriptional regulator BetI              K     201     0.537    188 
eok:G2583_0417 transcriptional regulator                K     201     0.537    188 
etw:ECSP_0368 transcriptional regulator BetI            K     195     0.537    188 
ecp:ECP_0388 transcriptional regulator BetI             K     201     0.526    194 
eum:ECUMN_0351 transcriptional regulator BetI           K     195     0.537    188 
ect:ECIAI39_0373 transcriptional regulator BetI         K     195     0.537    188 
ebd:ECBD_3345 transcriptional regulator BetI            K     195     0.537    188 
ebr:ECB_00269 transcriptional regulator BetI            K     195     0.537    188 
kpe:KPK_3993 transcriptional regulator BetI             K     195     0.537    188 
kpn:KPN_00586 transcriptional regulator BetI            K     201     0.537    188 
kpu:KP1_1530 transcriptional regulator BetI             K     201     0.537    188 
kva:Kvar_3783 TetR family transcriptional regulator     K     195     0.537    188 
enc:ECL_03121 transcriptional regulator BetI            K     195     0.527    188 
eam:EAMY_1713 HTH-type transcriptional regulator BetI   K     199     0.543    175 
eay:EAM_1685 TetR family transcriptional regulator      K     199     0.543    175 
eci:UTI89_C0342 transcriptional regulator BetI          K     201     0.532    188 
ecv:APECO1_1677 transcriptional regulator BetI          K     198     0.532    188
```

**Neighborhood Representations**: Table of genes in the defined genetic neighborhoods of the entry protein and its closest homologs  
  
**Neighborhood Representations for "bxe:Bxe\_B1590"**  

| ID | Annotation | EC number |
| --- | --- | --- |
| bxe:Bxe\_B1600 | sarcosine oxidase subunit beta (EC:1.5.3.1); K00303 sarcosine oxidase, subunit beta [EC:1.5.3.1] | ec:1.5.3.1 |
| bxe:Bxe\_B1599 | SoxD (EC:1.5.3.1); K00304 sarcosine oxidase, subunit delta [EC:1.5.3.1] | ec:1.5.3.1 |
| bxe:Bxe\_B1598 | sarcosine oxidase subunit alpha (EC:1.5.3.1); K00302 sarcosine oxidase, subunit alpha [EC:1.5.3.1] | ec:1.5.3.1 |
| bxe:Bxe\_B1597 | sarcosine oxidase subunit gamma (EC:1.5.3.1); K00305 sarcosine oxidase, subunit gamma [EC:1.5.3.1] | ec:1.5.3.1 |
| bxe:Bxe\_B1596 | hypothetical protein |  |
| bxe:Bxe\_B1595 | major facilitator superfamily metabolite/H(+) symporter; K08369 MFS transporter, putative metabolite:H+ symporter |  |
| bxe:Bxe\_B1594 | hypothetical protein |  |
| bxe:Bxe\_B1593 | ampC; beta-lactamase (EC:3.5.2.6); K01467 beta-lactamase class C [EC:3.5.2.6] | ec:3.5.2.6 |
| bxe:Bxe\_B1592 | choline dehydrogenase (EC:1.1.99.1); K00108 choline dehydrogenase [EC:1.1.99.1] | ec:1.1.99.1 |
| bxe:Bxe\_B1591 | betaine aldehyde dehydrogenase (EC:1.2.99.3); K00130 betaine-aldehyde dehydrogenase [EC:1.2.1.8] | ec:1.2.1.8 |
| bxe:Bxe\_B1590 | transcriptional regulator BetI; K02167 TetR/AcrR family transcriptional regulator, transcriptional repressor of bet genes |  |
| bxe:Bxe\_B1589 | fdsA; glutathione-independent formaldehyde dehydrogenase (EC:1.2.1.46); K00148 glutathione-independent formaldehyde dehydrogenase [EC:1.2.1.46] | ec:1.2.1.46 |
| bxe:Bxe\_B1588 | hypothetical protein |  |
| bxe:Bxe\_B1587 | hypothetical protein |  |
| bxe:Bxe\_B1586 | transcriptional regulator |  |
| bxe:Bxe\_B1585 | serine hydroxymethyltransferase (EC:2.1.2.1); K00600 glycine hydroxymethyltransferase [EC:2.1.2.1] | ec:2.1.2.1 |
| bxe:Bxe\_B1584 | membrane dipeptidase (EC:3.4.13.19); K01273 membrane dipeptidase [EC:3.4.13.19] | ec:3.4.13.19 |
| bxe:Bxe\_B1583 | hypothetical protein |  |
| bxe:Bxe\_B1582 | NADH-flavin oxidoreductase/NADH oxidase family protein |  |
| bxe:Bxe\_B1581 | hypothetical protein |  |
| bxe:Bxe\_B1580 | putative electron transfer flavoprotein alpha-subunit; K03522 electron transfer flavoprotein alpha subunit |  |

  
**Neighborhood Representations for "bpy:Bphyt\_5060"**  

| ID | Annotation | EC number |
| --- | --- | --- |
| bpy:Bphyt\_5050 | sarcosine oxidase subunit alpha; K00302 sarcosine oxidase, subunit alpha [EC:1.5.3.1] | ec:1.5.3.1 |
| bpy:Bphyt\_5051 | sarcosine oxidase subunit gamma; K00305 sarcosine oxidase, subunit gamma [EC:1.5.3.1] | ec:1.5.3.1 |
| bpy:Bphyt\_5052 | hypothetical protein |  |
| bpy:Bphyt\_5053 | major facilitator superfamily protein; K08369 MFS transporter, putative metabolite:H+ symporter |  |
| bpy:Bphyt\_5054 | hypothetical protein |  |
| bpy:Bphyt\_5055 | hypothetical protein |  |
| bpy:Bphyt\_5056 | cupin |  |
| bpy:Bphyt\_5057 | ampC; beta-lactamase; K01467 beta-lactamase class C [EC:3.5.2.6] | ec:3.5.2.6 |
| bpy:Bphyt\_5058 | choline dehydrogenase; K00108 choline dehydrogenase [EC:1.1.99.1] | ec:1.1.99.1 |
| bpy:Bphyt\_5059 | betaine aldehyde dehydrogenase; K00130 betaine-aldehyde dehydrogenase [EC:1.2.1.8] | ec:1.2.1.8 |
| bpy:Bphyt\_5060 | transcriptional regulator BetI; K02167 TetR/AcrR family transcriptional regulator, transcriptional repressor of bet genes |  |
| bpy:Bphyt\_5061 | formaldehyde dehydrogenase; K00148 glutathione-independent formaldehyde dehydrogenase [EC:1.2.1.46] | ec:1.2.1.46 |
| bpy:Bphyt\_5062 | AraC family transcriptional regulator |  |
| bpy:Bphyt\_5063 | serine hydroxymethyltransferase (EC:2.1.2.1); K00600 glycine hydroxymethyltransferase [EC:2.1.2.1] | ec:2.1.2.1 |
| bpy:Bphyt\_5064 | membrane dipeptidase (EC:3.4.13.19); K01273 membrane dipeptidase [EC:3.4.13.19] | ec:3.4.13.19 |
| bpy:Bphyt\_5065 | 4-vinyl reductase 4VR |  |
| bpy:Bphyt\_5066 | NADH:flavin oxidoreductase |  |
| bpy:Bphyt\_5067 | hypothetical protein |  |
| bpy:Bphyt\_5068 | electron transfer flavoprotein subunit alpha; K03522 electron transfer flavoprotein alpha subunit |  |
| bpy:Bphyt\_5069 | electron transfer flavoprotein beta-subunit; K03521 electron transfer flavoprotein beta subunit |  |
| bpy:Bphyt\_5070 | Rieske (2Fe-2S) domain-containing protein; K00479 Rieske 2Fe-2S family protein |  |

  
**Neighborhood Representations for "bgf:BC1003\_5873"**  

| ID | Annotation | EC number |
| --- | --- | --- |
| bgf:BC1003\_5863 | L-serine dehydratase 1 (EC:4.3.1.17); K01752 L-serine dehydratase [EC:4.3.1.17] | ec:4.3.1.17 |
| bgf:BC1003\_5864 | sarcosine oxidase subunit beta family; K00303 sarcosine oxidase, subunit beta [EC:1.5.3.1] | ec:1.5.3.1 |
| bgf:BC1003\_5865 | Sarcosine oxidase subunit delta heterotetrameric; K00304 sarcosine oxidase, subunit delta [EC:1.5.3.1] | ec:1.5.3.1 |
| bgf:BC1003\_5866 | sarcosine oxidase subunit alpha family; K00302 sarcosine oxidase, subunit alpha [EC:1.5.3.1] | ec:1.5.3.1 |
| bgf:BC1003\_5867 | Sarcosine oxidase subunit gamma; K00305 sarcosine oxidase, subunit gamma [EC:1.5.3.1] | ec:1.5.3.1 |
| bgf:BC1003\_5868 | hypothetical protein |  |
| bgf:BC1003\_5869 | major facilitator superfamily protein; K08369 MFS transporter, putative metabolite:H+ symporter |  |
| bgf:BC1003\_5870 | Beta-lactamase (EC:3.5.2.6); K01467 beta-lactamase class C [EC:3.5.2.6] | ec:3.5.2.6 |
| bgf:BC1003\_5871 | choline dehydrogenase; K00108 choline dehydrogenase [EC:1.1.99.1] | ec:1.1.99.1 |
| bgf:BC1003\_5872 | betaine aldehyde dehydrogenase; K00130 betaine-aldehyde dehydrogenase [EC:1.2.1.8] | ec:1.2.1.8 |
| bgf:BC1003\_5873 | transcriptional repressor BetI; K02167 TetR/AcrR family transcriptional regulator, transcriptional repressor of bet genes |  |
| bgf:BC1003\_5874 | formaldehyde dehydrogenase, glutathione-independent; K00148 glutathione-independent formaldehyde dehydrogenase [EC:1.2.1.46] | ec:1.2.1.46 |
| bgf:BC1003\_5875 | AraC family transcriptional regulator |  |
| bgf:BC1003\_5876 | glycine hydroxymethyltransferase (EC:2.1.2.1); K00600 glycine hydroxymethyltransferase [EC:2.1.2.1] | ec:2.1.2.1 |
| bgf:BC1003\_5877 | Membrane dipeptidase (EC:3.4.13.19); K01273 membrane dipeptidase [EC:3.4.13.19] | ec:3.4.13.19 |
| bgf:BC1003\_5878 | 4-vinyl reductase 4VR |  |
| bgf:BC1003\_5879 | NADH:flavin oxidoreductase/NADH oxidase |  |
| bgf:BC1003\_5880 | hypothetical protein |  |
| bgf:BC1003\_5881 | Electron transfer flavoprotein subunit alpha; K03522 electron transfer flavoprotein alpha subunit |  |
| bgf:BC1003\_5882 | Electron transfer flavoprotein alpha/beta-subunit; K03521 electron transfer flavoprotein beta subunit |  |
| bgf:BC1003\_5883 | Rieske (2Fe-2S) iron-sulfur domain-containing protein; K00479 Rieske 2Fe-2S family protein |  |

  
**Neighborhood Representations for "bph:Bphy\_3308"**  

| ID | Annotation | EC number |
| --- | --- | --- |
| bph:Bphy\_3298 | electron transfer flavoprotein alpha subunit; K03522 electron transfer flavoprotein alpha subunit |  |
| bph:Bphy\_3299 | hypothetical protein |  |
| bph:Bphy\_3300 | NADH:flavin oxidoreductase |  |
| bph:Bphy\_3301 | 4-vinyl reductase 4VR |  |
| bph:Bphy\_3302 | membrane dipeptidase (EC:3.4.13.19); K01273 membrane dipeptidase [EC:3.4.13.19] | ec:3.4.13.19 |
| bph:Bphy\_3303 | serine hydroxymethyltransferase (EC:2.1.2.1); K00600 glycine hydroxymethyltransferase [EC:2.1.2.1] | ec:2.1.2.1 |
| bph:Bphy\_3304 | AraC family transcriptional regulator |  |
| bph:Bphy\_3305 | ribonucleoside-diphosphate reductase; K00525 ribonucleoside-diphosphate reductase alpha chain [EC:1.17.4.1] | ec:1.17.4.1 |
| bph:Bphy\_3306 | hypothetical protein |  |
| bph:Bphy\_3307 | formaldehyde dehydrogenase; K00148 glutathione-independent formaldehyde dehydrogenase [EC:1.2.1.46] | ec:1.2.1.46 |
| bph:Bphy\_3308 | transcriptional regulator BetI; K02167 TetR/AcrR family transcriptional regulator, transcriptional repressor of bet genes |  |
| bph:Bphy\_3309 | betaine aldehyde dehydrogenase; K00130 betaine-aldehyde dehydrogenase [EC:1.2.1.8] | ec:1.2.1.8 |
| bph:Bphy\_3310 | choline dehydrogenase; K00108 choline dehydrogenase [EC:1.1.99.1] | ec:1.1.99.1 |
| bph:Bphy\_3311 | major facilitator transporter; K08369 MFS transporter, putative metabolite:H+ symporter |  |
| bph:Bphy\_3312 | metallophosphoesterase |  |
| bph:Bphy\_3313 | response regulator receiver protein |  |
| bph:Bphy\_3314 | OsmC family protein |  |
| bph:Bphy\_3315 | D-isomer specific 2-hydroxyacid dehydrogenase NAD-binding subunit |  |
| bph:Bphy\_3316 | hypothetical protein |  |
| bph:Bphy\_3317 | aldo/keto reductase |  |
| bph:Bphy\_3318 | LysR family transcriptional regulator (EC:4.2.1.1) |  |

  
**Neighborhood Representations for "bmj:BMULJ\_04979"**  

| ID | Annotation | EC number |
| --- | --- | --- |
| bmj:BMULJ\_04969 | 4-vinyl reductase |  |
| bmj:BMULJ\_04970 | dpeP; membrane dipeptidase (EC:3.4.13.19); K01273 membrane dipeptidase [EC:3.4.13.19] | ec:3.4.13.19 |
| bmj:BMULJ\_04971 | glyA; serine hydroxymethyltransferase (EC:2.1.2.1); K00600 glycine hydroxymethyltransferase [EC:2.1.2.1] | ec:2.1.2.1 |
| bmj:BMULJ\_04972 | AraC family transcriptional regulator |  |
| bmj:BMULJ\_04973 | periplasmic component of the Tol biopolymer transporter |  |
| bmj:BMULJ\_04974 | aspB; aspartate aminotransferase (EC:2.6.1.1) |  |
| bmj:BMULJ\_04975 | ATP-dependent serine protease |  |
| bmj:BMULJ\_04976 | drug/metabolite transporter (DMT) superfamily permease |  |
| bmj:BMULJ\_04977 | LysR family transcriptional regulator; K03566 LysR family transcriptional regulator, glycine cleavage system transcriptional activator |  |
| bmj:BMULJ\_04978 | fdhA2; glutathione-independent formaldehyde dehydrogenase (EC:1.2.1.46); K00148 glutathione-independent formaldehyde dehydrogenase [EC:1.2.1.46] | ec:1.2.1.46 |
| bmj:BMULJ\_04979 | betI; transcriptional regulator BetI; K02167 TetR/AcrR family transcriptional regulator, transcriptional repressor of bet genes |  |
| bmj:BMULJ\_04980 | betB; betaine aldehyde dehydrogenase (EC:1.2.1.8); K00130 betaine-aldehyde dehydrogenase [EC:1.2.1.8] | ec:1.2.1.8 |
| bmj:BMULJ\_04981 | betA; choline dehydrogenase (EC:1.1.99.1); K00108 choline dehydrogenase [EC:1.1.99.1] | ec:1.1.99.1 |
| bmj:BMULJ\_04982 | ABC-2 type transporter ATP-binding protein; K01990 ABC-2 type transport system ATP-binding protein |  |
| bmj:BMULJ\_04983 | ABC-2 type transporter permease; K01992 ABC-2 type transport system permease protein |  |
| bmj:BMULJ\_04984 | ydjE; metabolite:H+ symporter; K08369 MFS transporter, putative metabolite:H+ symporter |  |
| bmj:BMULJ\_04985 | hypothetical protein |  |
| bmj:BMULJ\_04986 | hypothetical protein |  |
| bmj:BMULJ\_04987 | esterase |  |
| bmj:BMULJ\_04988 | short-chain alcohol dehydrogenase |  |
| bmj:BMULJ\_04989 | LysR family transcriptional regulator |  |

  
**Neighborhood Representations for "bmu:Bmul\_3538"**  

| ID | Annotation | EC number |
| --- | --- | --- |
| bmu:Bmul\_3528 | LysR family transcriptional regulator |  |
| bmu:Bmul\_3529 | short-chain dehydrogenase/reductase SDR |  |
| bmu:Bmul\_3530 | esterase |  |
| bmu:Bmul\_3531 | DGPFAETKE family protein |  |
| bmu:Bmul\_3532 | hypothetical protein |  |
| bmu:Bmul\_3533 | major facilitator transporter; K08369 MFS transporter, putative metabolite:H+ symporter |  |
| bmu:Bmul\_3534 | ABC-2 type transporter; K01992 ABC-2 type transport system permease protein |  |
| bmu:Bmul\_3535 | ABC transporter; K01990 ABC-2 type transport system ATP-binding protein |  |
| bmu:Bmul\_3536 | choline dehydrogenase; K00108 choline dehydrogenase [EC:1.1.99.1] | ec:1.1.99.1 |
| bmu:Bmul\_3537 | betaine aldehyde dehydrogenase; K00130 betaine-aldehyde dehydrogenase [EC:1.2.1.8] | ec:1.2.1.8 |
| bmu:Bmul\_3538 | transcriptional regulator BetI; K02167 TetR/AcrR family transcriptional regulator, transcriptional repressor of bet genes |  |
| bmu:Bmul\_3539 | formaldehyde dehydrogenase; K00148 glutathione-independent formaldehyde dehydrogenase [EC:1.2.1.46] | ec:1.2.1.46 |
| bmu:Bmul\_3540 | LysR family transcriptional regulator; K03566 LysR family transcriptional regulator, glycine cleavage system transcriptional activator |  |
| bmu:Bmul\_3541 | hypothetical protein |  |
| bmu:Bmul\_3542 | hypothetical protein |  |
| bmu:Bmul\_3543 | transcriptional regulator |  |
| bmu:Bmul\_3544 | coagulation factor 5/8 type domain-containing protein |  |
| bmu:Bmul\_3545 | AraC family transcriptional regulator |  |
| bmu:Bmul\_3546 | serine hydroxymethyltransferase (EC:2.1.2.1); K00600 glycine hydroxymethyltransferase [EC:2.1.2.1] | ec:2.1.2.1 |
| bmu:Bmul\_3547 | membrane dipeptidase (EC:3.4.13.19); K01273 membrane dipeptidase [EC:3.4.13.19] | ec:3.4.13.19 |
| bmu:Bmul\_3548 | 4-vinyl reductase 4VR |  |

  
**Neighborhood Representations for "bcj:BCAM2341"**  

| ID | Annotation | EC number |
| --- | --- | --- |
| bcj:BCAM2331 | hypothetical protein |  |
| bcj:BCAM2332 | LysR family regulatory protein; K03566 LysR family transcriptional regulator, glycine cleavage system transcriptional activator |  |
| bcj:BCAM2333 | fdhA; putative glutathione-independent formaldehyde dehydrogenase (EC:1.2.1.46); K00148 glutathione-independent formaldehyde dehydrogenase [EC:1.2.1.46] | ec:1.2.1.46 |
| bcj:BCAM2334 | efflux system transport protein; K03543 multidrug resistance protein A |  |
| bcj:BCAM2335 | outer membrane efflux protein |  |
| bcj:BCAM2336 | putative sugar transferase; K12990 rhamnosyltransferase [EC:2.4.1.-] |  |
| bcj:BCAM2337 | putative multidrug resistance transporter protein |  |
| bcj:BCAM2338 | putative glycosyltransferase |  |
| bcj:BCAM2339 | putative methyltransferase |  |
| bcj:BCAM2340 | phaG; putative (R)-3-hydroxydecanoyl-ACP:CoA transacylase (EC:2.4.1.-) |  |
| bcj:BCAM2341 | betI; transcriptional regulator BetI; K02167 TetR/AcrR family transcriptional regulator, transcriptional repressor of bet genes |  |
| bcj:BCAM2342 | betB; betaine aldehyde dehydrogenase (EC:1.2.1.8); K00130 betaine-aldehyde dehydrogenase [EC:1.2.1.8] | ec:1.2.1.8 |
| bcj:BCAM2343 | betA; choline dehydrogenase (EC:1.1.99.1); K00108 choline dehydrogenase [EC:1.1.99.1] | ec:1.1.99.1 |
| bcj:BCAM2344 | hypothetical protein |  |
| bcj:BCAM2345 | hypothetical protein |  |
| bcj:BCAM2346 | putative OmpW-family exported protein; K07275 outer membrane protein |  |
| bcj:BCAM2347 | putative lipoprotein |  |
| bcj:BCAM2348 | putative lipoprotein |  |
| bcj:BCAM2350 | ABC transporter ATP-binding protein; K01990 ABC-2 type transport system ATP-binding protein |  |
| bcj:BCAM2351 | putative ABC transporter transmembrane protein; K01992 ABC-2 type transport system permease protein |  |
| bcj:BCAM2352 | major facilitator superfamily protein; K08369 MFS transporter, putative metabolite:H+ symporter |  |

  
**Neighborhood Representations for "bte:BTH\_II1074"**  

| ID | Annotation | EC number |
| --- | --- | --- |
| bte:BTH\_II1064 | hypothetical protein |  |
| bte:BTH\_II1065 | holin |  |
| bte:BTH\_II1066 | hypothetical protein |  |
| bte:BTH\_II1067 | gp23 |  |
| bte:BTH\_II1068 | DNA adenine methylase; K06223 DNA adenine methylase [EC:2.1.1.72] | ec:2.1.1.72 |
| bte:BTH\_II1069 | hypothetical protein |  |
| bte:BTH\_II1070 | MFS transporter |  |
| bte:BTH\_II1071 | hypothetical protein |  |
| bte:BTH\_II1072 | betA; choline dehydrogenase (EC:1.1.99.1); K00108 choline dehydrogenase [EC:1.1.99.1] | ec:1.1.99.1 |
| bte:BTH\_II1073 | betB; betaine aldehyde dehydrogenase (EC:1.2.1.8); K00130 betaine-aldehyde dehydrogenase [EC:1.2.1.8] | ec:1.2.1.8 |
| bte:BTH\_II1074 | transcriptional regulator BetI; K02167 TetR/AcrR family transcriptional regulator, transcriptional repressor of bet genes |  |
| bte:BTH\_II1075 | rhamnosyltransferase 1 subunit A |  |
| bte:BTH\_II1076 | rhamnosyltransferase I subunit B |  |
| bte:BTH\_II1077 | EmrB/QacA family drug resistance transporter |  |
| bte:BTH\_II1078 | hypothetical protein |  |
| bte:BTH\_II1079 | rhamnosyltransferase; K12990 rhamnosyltransferase [EC:2.4.1.-] |  |
| bte:BTH\_II1080 | RND efflux system outer membrane lipoprotein |  |
| bte:BTH\_II1081 | multidrug resistance protein; K03543 multidrug resistance protein A |  |
| bte:BTH\_II1082 | transposase mutator family protein |  |
| bte:BTH\_II1083 | prolyl oligopeptidase; K01322 prolyl oligopeptidase [EC:3.4.21.26] | ec:3.4.21.26 |
| bte:BTH\_II1084 | cytochrome b561 family protein |  |

  
**Neighborhood Representations for "bch:Bcen2424\_5098"**  

| ID | Annotation | EC number |
| --- | --- | --- |
| bch:Bcen2424\_5088 | GntR family transcriptional regulator |  |
| bch:Bcen2424\_5089 | hypothetical protein |  |
| bch:Bcen2424\_5090 | alcohol dehydrogenase; K00148 glutathione-independent formaldehyde dehydrogenase [EC:1.2.1.46] | ec:1.2.1.46 |
| bch:Bcen2424\_5091 | secretion protein HlyD family protein; K03543 multidrug resistance protein A |  |
| bch:Bcen2424\_5092 | RND efflux system outer membrane lipoprotein |  |
| bch:Bcen2424\_5093 | rhamnosyltransferase; K12990 rhamnosyltransferase [EC:2.4.1.-] |  |
| bch:Bcen2424\_5094 | EmrB/QacA family drug resistance transporter |  |
| bch:Bcen2424\_5095 | glycosyl transferase family protein |  |
| bch:Bcen2424\_5096 | isoprenylcysteine carboxyl methyltransferase |  |
| bch:Bcen2424\_5097 | alpha/beta hydrolase |  |
| bch:Bcen2424\_5098 | transcriptional regulator BetI; K02167 TetR/AcrR family transcriptional regulator, transcriptional repressor of bet genes |  |
| bch:Bcen2424\_5099 | betaine aldehyde dehydrogenase (EC:1.2.1.8); K00130 betaine-aldehyde dehydrogenase [EC:1.2.1.8] | ec:1.2.1.8 |
| bch:Bcen2424\_5100 | choline dehydrogenase (EC:1.1.99.1); K00108 choline dehydrogenase [EC:1.1.99.1] | ec:1.1.99.1 |
| bch:Bcen2424\_5101 | hypothetical protein |  |
| bch:Bcen2424\_5102 | hypothetical protein |  |
| bch:Bcen2424\_5103 | OmpW family protein; K07275 outer membrane protein |  |
| bch:Bcen2424\_5104 | hypothetical protein |  |
| bch:Bcen2424\_5105 | hypothetical protein |  |
| bch:Bcen2424\_5106 | cupin |  |
| bch:Bcen2424\_5107 | ABC transporter; K01990 ABC-2 type transport system ATP-binding protein |  |
| bch:Bcen2424\_5108 | ABC-2 type transporter; K01992 ABC-2 type transport system permease protein |  |

  
**Neighborhood Representations for "bcm:Bcenmc03\_5184"**  

| ID | Annotation | EC number |
| --- | --- | --- |
| bcm:Bcenmc03\_5174 | ABC transporter-like protein; K01990 ABC-2 type transport system ATP-binding protein |  |
| bcm:Bcenmc03\_5175 | cupin 2 domain-containing protein |  |
| bcm:Bcenmc03\_5176 | hypothetical protein |  |
| bcm:Bcenmc03\_5177 | hypothetical protein |  |
| bcm:Bcenmc03\_5178 | OmpW family protein; K07275 outer membrane protein |  |
| bcm:Bcenmc03\_5179 | hypothetical protein |  |
| bcm:Bcenmc03\_5180 | hypothetical protein |  |
| bcm:Bcenmc03\_5181 | hypothetical protein |  |
| bcm:Bcenmc03\_5182 | choline dehydrogenase; K00108 choline dehydrogenase [EC:1.1.99.1] | ec:1.1.99.1 |
| bcm:Bcenmc03\_5183 | betaine aldehyde dehydrogenase; K00130 betaine-aldehyde dehydrogenase [EC:1.2.1.8] | ec:1.2.1.8 |
| bcm:Bcenmc03\_5184 | transcriptional regulator BetI; K02167 TetR/AcrR family transcriptional regulator, transcriptional repressor of bet genes |  |
| bcm:Bcenmc03\_5185 | alpha/beta hydrolase |  |
| bcm:Bcenmc03\_5186 | isoprenylcysteine carboxyl methyltransferase |  |
| bcm:Bcenmc03\_5187 | glycosyl transferase family protein |  |
| bcm:Bcenmc03\_5188 | EmrB/QacA family drug resistance transporter |  |
| bcm:Bcenmc03\_5189 | rhamnosyltransferase; K12990 rhamnosyltransferase [EC:2.4.1.-] |  |
| bcm:Bcenmc03\_5190 | RND efflux system outer membrane lipoprotein |  |
| bcm:Bcenmc03\_5191 | secretion protein HlyD family protein; K03543 multidrug resistance protein A |  |
| bcm:Bcenmc03\_5192 | formaldehyde dehydrogenase, glutathione-independent; K00148 glutathione-independent formaldehyde dehydrogenase [EC:1.2.1.46] | ec:1.2.1.46 |
| bcm:Bcenmc03\_5193 | hypothetical protein |  |
| bcm:Bcenmc03\_5194 | transcriptional regulator |  |

  
**Neighborhood Representations for "bcn:Bcen\_3270"**  

| ID | Annotation | EC number |
| --- | --- | --- |
| bcn:Bcen\_3260 | ABC-2 type transporter; K01992 ABC-2 type transport system permease protein |  |
| bcn:Bcen\_3261 | ABC transporter; K01990 ABC-2 type transport system ATP-binding protein |  |
| bcn:Bcen\_3262 | cupin |  |
| bcn:Bcen\_3263 | hypothetical protein |  |
| bcn:Bcen\_3264 | hypothetical protein |  |
| bcn:Bcen\_3265 | OmpW; K07275 outer membrane protein |  |
| bcn:Bcen\_3266 | hypothetical protein |  |
| bcn:Bcen\_3267 | hypothetical protein |  |
| bcn:Bcen\_3268 | choline dehydrogenase (EC:1.1.99.1); K00108 choline dehydrogenase [EC:1.1.99.1] | ec:1.1.99.1 |
| bcn:Bcen\_3269 | betaine aldehyde dehydrogenase (EC:1.2.1.8); K00130 betaine-aldehyde dehydrogenase [EC:1.2.1.8] | ec:1.2.1.8 |
| bcn:Bcen\_3270 | transcriptional regulator BetI; K02167 TetR/AcrR family transcriptional regulator, transcriptional repressor of bet genes |  |
| bcn:Bcen\_3271 | alpha/beta hydrolase |  |
| bcn:Bcen\_3272 | isoprenylcysteine carboxyl methyltransferase |  |
| bcn:Bcen\_3273 | glycosyl transferase family protein |  |
| bcn:Bcen\_3274 | EmrB/QacA family drug resistance transporter |  |
| bcn:Bcen\_3275 | rhamnosyltransferase; K12990 rhamnosyltransferase [EC:2.4.1.-] |  |
| bcn:Bcen\_3276 | RND efflux system, outer membrane lipoprotein, NodT |  |
| bcn:Bcen\_3277 | secretion protein HlyD; K03543 multidrug resistance protein A |  |
| bcn:Bcen\_3278 | alcohol dehydrogenase GroES-like protein; K00148 glutathione-independent formaldehyde dehydrogenase [EC:1.2.1.46] | ec:1.2.1.46 |
| bcn:Bcen\_3279 | hypothetical protein |  |
| bcn:Bcen\_3280 | GntR family transcriptional regulator |  |

  
**Neighborhood Representations for "bvi:Bcep1808\_3429"**  

| ID | Annotation | EC number |
| --- | --- | --- |
| bvi:Bcep1808\_3419 | hypothetical protein |  |
| bvi:Bcep1808\_3420 | NADH:flavin oxidoreductase |  |
| bvi:Bcep1808\_3421 | 4-vinyl reductase, 4VR |  |
| bvi:Bcep1808\_3422 | membrane dipeptidase (EC:3.4.13.19); K01273 membrane dipeptidase [EC:3.4.13.19] | ec:3.4.13.19 |
| bvi:Bcep1808\_3423 | serine hydroxymethyltransferase (EC:2.1.2.1); K00600 glycine hydroxymethyltransferase [EC:2.1.2.1] | ec:2.1.2.1 |
| bvi:Bcep1808\_3424 | transcriptional regulator |  |
| bvi:Bcep1808\_3425 | hypothetical protein |  |
| bvi:Bcep1808\_3426 | hypothetical protein |  |
| bvi:Bcep1808\_3427 | natural resistance-associated macrophage protein |  |
| bvi:Bcep1808\_3428 | alcohol dehydrogenase; K00148 glutathione-independent formaldehyde dehydrogenase [EC:1.2.1.46] | ec:1.2.1.46 |
| bvi:Bcep1808\_3429 | transcriptional regulator BetI; K02167 TetR/AcrR family transcriptional regulator, transcriptional repressor of bet genes |  |
| bvi:Bcep1808\_3430 | betaine aldehyde dehydrogenase (EC:1.2.99.3); K00130 betaine-aldehyde dehydrogenase [EC:1.2.1.8] | ec:1.2.1.8 |
| bvi:Bcep1808\_3431 | choline dehydrogenase (EC:1.1.99.1); K00108 choline dehydrogenase [EC:1.1.99.1] | ec:1.1.99.1 |
| bvi:Bcep1808\_3432 | hypothetical protein |  |
| bvi:Bcep1808\_3433 | hypothetical protein |  |
| bvi:Bcep1808\_3434 | OmpW family protein; K07275 outer membrane protein |  |
| bvi:Bcep1808\_3435 | hypothetical protein |  |
| bvi:Bcep1808\_3436 | hypothetical protein |  |
| bvi:Bcep1808\_3437 | ABC transporter; K01990 ABC-2 type transport system ATP-binding protein |  |
| bvi:Bcep1808\_3438 | ABC-2 type transporter; K01992 ABC-2 type transport system permease protein |  |
| bvi:Bcep1808\_3439 | beta-lactamase domain-containing protein |  |

  
**Neighborhood Representations for "bur:Bcep18194\_B0555"**  

| ID | Annotation | EC number |
| --- | --- | --- |
| bur:Bcep18194\_B0545 | hypothetical protein |  |
| bur:Bcep18194\_B0546 | OmpW family protein; K07275 outer membrane protein |  |
| bur:Bcep18194\_B0547 | hypothetical protein |  |
| bur:Bcep18194\_B0548 | hypothetical protein |  |
| bur:Bcep18194\_B0549 | hypothetical protein |  |
| bur:Bcep18194\_B0550 | monogalactosyldiacylglycerol synthase (EC:2.4.1.46) |  |
| bur:Bcep18194\_B0551 | hypothetical protein |  |
| bur:Bcep18194\_B0552 | protein tyrosine/serine phosphatase |  |
| bur:Bcep18194\_B0553 | choline dehydrogenase (EC:1.1.99.1); K00108 choline dehydrogenase [EC:1.1.99.1] | ec:1.1.99.1 |
| bur:Bcep18194\_B0554 | betaine aldehyde dehydrogenase (EC:1.2.99.3); K00130 betaine-aldehyde dehydrogenase [EC:1.2.1.8] | ec:1.2.1.8 |
| bur:Bcep18194\_B0555 | transcriptional regulator BetI; K02167 TetR/AcrR family transcriptional regulator, transcriptional repressor of bet genes |  |
| bur:Bcep18194\_B0556 | alpha/beta hydrolase |  |
| bur:Bcep18194\_B0557 | isoprenylcysteine carboxyl methyltransferase |  |
| bur:Bcep18194\_B0558 | glycosyl transferase |  |
| bur:Bcep18194\_B0559 | EmrB/QacA family drug resistance transporter |  |
| bur:Bcep18194\_B0560 | rhamnosyltransferase; K12990 rhamnosyltransferase [EC:2.4.1.-] |  |
| bur:Bcep18194\_B0561 | RND efflux system outer membrane lipoprotein |  |
| bur:Bcep18194\_B0562 | HlyD family secretion protein; K03543 multidrug resistance protein A |  |
| bur:Bcep18194\_B0563 | Short-chain dehydrogenase/reductase SDR (EC:1.1.1.100); K00065 2-deoxy-D-gluconate 3-dehydrogenase [EC:1.1.1.125] | ec:1.1.1.125 |
| bur:Bcep18194\_B0564 | zinc-containing alcohol dehydrogenase superfamily protein (EC:1.2.1.46); K00148 glutathione-independent formaldehyde dehydrogenase [EC:1.2.1.46] | ec:1.2.1.46 |
| bur:Bcep18194\_B0565 | fructose-bisphosphate aldolase (EC:4.1.2.13); K01623 fructose-bisphosphate aldolase, class I [EC:4.1.2.13] | ec:4.1.2.13 |

  
**Neighborhood Representations for "bac:BamMC406\_5035"**  

| ID | Annotation | EC number |
| --- | --- | --- |
| bac:BamMC406\_5025 | transcriptional regulator |  |
| bac:BamMC406\_5026 | hypothetical protein |  |
| bac:BamMC406\_5027 | formaldehyde dehydrogenase, glutathione-independent; K00148 glutathione-independent formaldehyde dehydrogenase [EC:1.2.1.46] | ec:1.2.1.46 |
| bac:BamMC406\_5028 | secretion protein HlyD family protein; K03543 multidrug resistance protein A |  |
| bac:BamMC406\_5029 | RND efflux system outer membrane lipoprotein |  |
| bac:BamMC406\_5030 | rhamnosyltransferase; K12990 rhamnosyltransferase [EC:2.4.1.-] |  |
| bac:BamMC406\_5031 | EmrB/QacA family drug resistance transporter |  |
| bac:BamMC406\_5032 | glycosyl transferase family protein |  |
| bac:BamMC406\_5033 | isoprenylcysteine carboxyl methyltransferase |  |
| bac:BamMC406\_5034 | alpha/beta hydrolase fold protein |  |
| bac:BamMC406\_5035 | transcriptional regulator BetI; K02167 TetR/AcrR family transcriptional regulator, transcriptional repressor of bet genes |  |
| bac:BamMC406\_5036 | betaine aldehyde dehydrogenase; K00130 betaine-aldehyde dehydrogenase [EC:1.2.1.8] | ec:1.2.1.8 |
| bac:BamMC406\_5037 | choline dehydrogenase; K00108 choline dehydrogenase [EC:1.1.99.1] | ec:1.1.99.1 |
| bac:BamMC406\_5038 | hypothetical protein |  |
| bac:BamMC406\_5039 | hypothetical protein |  |
| bac:BamMC406\_5040 | OmpW family protein; K07275 outer membrane protein |  |
| bac:BamMC406\_5041 | hypothetical protein |  |
| bac:BamMC406\_5042 | hypothetical protein |  |
| bac:BamMC406\_5043 | ABC transporter-like protein; K01990 ABC-2 type transport system ATP-binding protein |  |
| bac:BamMC406\_5044 | ABC-2 type transporter; K01992 ABC-2 type transport system permease protein |  |
| bac:BamMC406\_5045 | beta-lactamase domain-containing protein |  |

  
**Neighborhood Representations for "bam:Bamb\_4510"**  

| ID | Annotation | EC number |
| --- | --- | --- |
| bam:Bamb\_4500 | GntR family transcriptional regulator |  |
| bam:Bamb\_4501 | hypothetical protein |  |
| bam:Bamb\_4502 | alcohol dehydrogenase; K00148 glutathione-independent formaldehyde dehydrogenase [EC:1.2.1.46] | ec:1.2.1.46 |
| bam:Bamb\_4503 | secretion protein HlyD family protein; K03543 multidrug resistance protein A |  |
| bam:Bamb\_4504 | RND efflux system outer membrane lipoprotein |  |
| bam:Bamb\_4505 | rhamnosyltransferase; K12990 rhamnosyltransferase [EC:2.4.1.-] |  |
| bam:Bamb\_4506 | EmrB/QacA family drug resistance transporter |  |
| bam:Bamb\_4507 | glycosyl transferase family protein |  |
| bam:Bamb\_4508 | isoprenylcysteine carboxyl methyltransferase |  |
| bam:Bamb\_4509 | alpha/beta hydrolase |  |
| bam:Bamb\_4510 | transcriptional regulator BetI; K02167 TetR/AcrR family transcriptional regulator, transcriptional repressor of bet genes |  |
| bam:Bamb\_4511 | betaine aldehyde dehydrogenase (EC:1.2.1.8); K00130 betaine-aldehyde dehydrogenase [EC:1.2.1.8] | ec:1.2.1.8 |
| bam:Bamb\_4512 | choline dehydrogenase (EC:1.1.99.1); K00108 choline dehydrogenase [EC:1.1.99.1] | ec:1.1.99.1 |
| bam:Bamb\_4513 | hypothetical protein |  |
| bam:Bamb\_4514 | hypothetical protein |  |
| bam:Bamb\_4515 | OmpW family protein; K07275 outer membrane protein |  |
| bam:Bamb\_4516 | hypothetical protein |  |
| bam:Bamb\_4517 | hypothetical protein |  |
| bam:Bamb\_4518 | hypothetical protein |  |
| bam:Bamb\_4519 | hypothetical protein |  |
| bam:Bamb\_4520 | ABC transporter; K01990 ABC-2 type transport system ATP-binding protein |  |

  
**Neighborhood Representations for "bml:BMA10229\_0181"**  

| ID | Annotation | EC number |
| --- | --- | --- |
| bml:BMA10229\_0171 | hypothetical protein |  |
| bml:BMA10229\_0172 | glyoxylate reductase |  |
| bml:BMA10229\_0173 | hypothetical protein |  |
| bml:BMA10229\_0174 | sensory box histidine kinase; K00936 [EC:2.7.3.-] |  |
| bml:BMA10229\_0175 | response regulator |  |
| bml:BMA10229\_0176 | sensory box histidine kinase/response regulator; K07675 two-component system, NarL family, sensor histidine kinase UhpB [EC:2.7.13.3] | ec:2.7.13.3 |
| bml:BMA10229\_0177 | major facilitator family transporter; K08369 MFS transporter, putative metabolite:H+ symporter |  |
| bml:BMA10229\_0178 | hypothetical protein |  |
| bml:BMA10229\_0179 | betA; choline dehydrogenase; K00108 choline dehydrogenase [EC:1.1.99.1] | ec:1.1.99.1 |
| bml:BMA10229\_0180 | betB; betaine aldehyde dehydrogenase; K00130 betaine-aldehyde dehydrogenase [EC:1.2.1.8] | ec:1.2.1.8 |
| bml:BMA10229\_0181 | betI; transcriptional regulator BetI; K02167 TetR/AcrR family transcriptional regulator, transcriptional repressor of bet genes |  |
| bml:BMA10229\_0182 | IS407A, transposase OrfA; K07497 putative transposase |  |
| bml:BMA10229\_0183 | IS407A, transposase OrfB; K07497 putative transposase |  |
| bml:BMA10229\_0184 | rhlA-2; rhamnosyltransferase I, subunit A |  |
| bml:BMA10229\_0185 | rhlB-2; rhamnosyltransferase I, subunit B |  |
| bml:BMA10229\_0186 | EmrB/QacA family drug resistance transporter |  |
| bml:BMA10229\_0187 | hypothetical protein |  |
| bml:BMA10229\_0188 | hypothetical protein |  |
| bml:BMA10229\_0189 | dTDP-rhamnosyl transferase rfbf protein; K12990 rhamnosyltransferase [EC:2.4.1.-] |  |
| bml:BMA10229\_0190 | hypothetical protein |  |
| bml:BMA10229\_0191 | hypothetical protein |  |

  
**Neighborhood Representations for "bmn:BMA10247\_A1426"**  

| ID | Annotation | EC number |
| --- | --- | --- |
| bmn:BMA10247\_A1416 | multidrug resistance protein; K03543 multidrug resistance protein A |  |
| bmn:BMA10247\_A1417 | pseudogene |  |
| bmn:BMA10247\_A1418 | hypothetical protein |  |
| bmn:BMA10247\_A1419 | rhamnosyltransferase family protein; K12990 rhamnosyltransferase [EC:2.4.1.-] |  |
| bmn:BMA10247\_A1420 | EmrB/QacA family drug resistance transporter |  |
| bmn:BMA10247\_A1421 | rhlB-2; rhamnosyltransferase I subunit B (EC:2.4.1.-) |  |
| bmn:BMA10247\_A1422 | hypothetical protein |  |
| bmn:BMA10247\_A1423 | rhlA-2; rhamnosyltransferase I subunit A (EC:2.4.1.-) |  |
| bmn:BMA10247\_A1424 | IS407A, transposase OrfB; K07497 putative transposase |  |
| bmn:BMA10247\_A1425 | IS407A, transposase OrfA; K07497 putative transposase |  |
| bmn:BMA10247\_A1426 | betI; transcriptional regulator BetI; K02167 TetR/AcrR family transcriptional regulator, transcriptional repressor of bet genes |  |
| bmn:BMA10247\_A1427 | betB; betaine aldehyde dehydrogenase (EC:1.2.1.8); K00130 betaine-aldehyde dehydrogenase [EC:1.2.1.8] | ec:1.2.1.8 |
| bmn:BMA10247\_A1428 | betA; choline dehydrogenase (EC:1.1.99.1); K00108 choline dehydrogenase [EC:1.1.99.1] | ec:1.1.99.1 |
| bmn:BMA10247\_A1429 | DNA polymerase III subunits gamma and tau |  |
| bmn:BMA10247\_A1430 | hypothetical protein |  |
| bmn:BMA10247\_A1431 | major facilitator family transporter; K08369 MFS transporter, putative metabolite:H+ symporter |  |
| bmn:BMA10247\_A1432 | sensory box histidine kinase/response regulator; K07675 two-component system, NarL family, sensor histidine kinase UhpB [EC:2.7.13.3] | ec:2.7.13.3 |
| bmn:BMA10247\_A1433 | response regulator |  |
| bmn:BMA10247\_A1434 | sensory box histidine kinase (EC:2.7.3.-); K00936 [EC:2.7.3.-] |  |
| bmn:BMA10247\_A1435 | hypothetical protein |  |
| bmn:BMA10247\_A1436 | glyoxylate reductase |  |

  
**Neighborhood Representations for "bmv:BMASAVP1\_0466"**  

| ID | Annotation | EC number |
| --- | --- | --- |
| bmv:BMASAVP1\_0456 | multidrug resistance protein; K03543 multidrug resistance protein A |  |
| bmv:BMASAVP1\_0457 | NodT family efflux transporter outer membrane lipoprotein |  |
| bmv:BMASAVP1\_0458 | hypothetical protein |  |
| bmv:BMASAVP1\_0459 | rhamnosyltransferase; K12990 rhamnosyltransferase [EC:2.4.1.-] |  |
| bmv:BMASAVP1\_0460 | EmrB/QacA family drug resistance transporter |  |
| bmv:BMASAVP1\_0461 | rhlB-2; rhamnosyltransferase I, subunit B |  |
| bmv:BMASAVP1\_0462 | hypothetical protein |  |
| bmv:BMASAVP1\_0463 | rhlA-2; rhamnosyltransferase I, subunit A |  |
| bmv:BMASAVP1\_0464 | IS407A, transposase OrfB; K07497 putative transposase |  |
| bmv:BMASAVP1\_0465 | IS407A, transposase OrfA; K07497 putative transposase |  |
| bmv:BMASAVP1\_0466 | betI; transcriptional regulator BetI; K02167 TetR/AcrR family transcriptional regulator, transcriptional repressor of bet genes |  |
| bmv:BMASAVP1\_0467 | betB; betaine aldehyde dehydrogenase; K00130 betaine-aldehyde dehydrogenase [EC:1.2.1.8] | ec:1.2.1.8 |
| bmv:BMASAVP1\_0468 | betA; choline dehydrogenase; K00108 choline dehydrogenase [EC:1.1.99.1] | ec:1.1.99.1 |
| bmv:BMASAVP1\_0469 | hypothetical protein |  |
| bmv:BMASAVP1\_0470 | hypothetical protein |  |
| bmv:BMASAVP1\_0471 | major facilitator family transporter; K08369 MFS transporter, putative metabolite:H+ symporter |  |
| bmv:BMASAVP1\_0472 | sensory box histidine kinase/response regulator; K07675 two-component system, NarL family, sensor histidine kinase UhpB [EC:2.7.13.3] | ec:2.7.13.3 |
| bmv:BMASAVP1\_0473 | response regulator |  |
| bmv:BMASAVP1\_0474 | sensory box histidine kinase; K00936 [EC:2.7.3.-] |  |
| bmv:BMASAVP1\_0475 | hypothetical protein |  |
| bmv:BMASAVP1\_0476 | glyoxylate reductase |  |

  
**Neighborhood Representations for "bpd:BURPS668\_A1923"**  

| ID | Annotation | EC number |
| --- | --- | --- |
| bpd:BURPS668\_A1912 | hypothetical protein |  |
| bpd:BURPS668\_A1914 | multidrug resistance efflux pump |  |
| bpd:BURPS668\_A1915 | hypothetical protein |  |
| bpd:BURPS668\_A1916 | Outer membrane protein |  |
| bpd:BURPS668\_A1917 | rhamnosyltransferase II; K12990 rhamnosyltransferase [EC:2.4.1.-] |  |
| bpd:BURPS668\_A1918 | major facilitator superfamily permease |  |
| bpd:BURPS668\_A1919 | rhamnosyl transferase |  |
| bpd:BURPS668\_A1920 | hypothetical protein |  |
| bpd:BURPS668\_A1921 | rhamnosyltransferase 1 subunit A (EC:2.4.1.-) |  |
| bpd:BURPS668\_A1922 | hypothetical protein |  |
| bpd:BURPS668\_A1923 | transcriptional regulator BetI; K02167 TetR/AcrR family transcriptional regulator, transcriptional repressor of bet genes |  |
| bpd:BURPS668\_A1924 | betB; betaine aldehyde dehydrogenase (EC:1.2.1.8); K00130 betaine-aldehyde dehydrogenase [EC:1.2.1.8] | ec:1.2.1.8 |
| bpd:BURPS668\_A1925 | betA; choline dehydrogenase (EC:1.1.99.1); K00108 choline dehydrogenase [EC:1.1.99.1] | ec:1.1.99.1 |
| bpd:BURPS668\_A1926 | hypothetical protein |  |
| bpd:BURPS668\_A1927 | hypothetical protein |  |
| bpd:BURPS668\_A1929 | hypothetical protein |  |
| bpd:BURPS668\_A1930 | hypothetical protein |  |
| bpd:BURPS668\_A1931 | major facilitator superfamily permease; K08369 MFS transporter, putative metabolite:H+ symporter |  |
| bpd:BURPS668\_A1932 | Signal transduction histidine kinase; K07675 two-component system, NarL family, sensor histidine kinase UhpB [EC:2.7.13.3] | ec:2.7.13.3 |
| bpd:BURPS668\_A1933 | response regulator |  |
| bpd:BURPS668\_A1934 | two-component sensor kinase protein |  |

  
**Neighborhood Representations for "bpl:BURPS1106A\_A1835"**  

| ID | Annotation | EC number |
| --- | --- | --- |
| bpl:BURPS1106A\_A1825 | multidrug resistance protein; K03543 multidrug resistance protein A |  |
| bpl:BURPS1106A\_A1826 | hypothetical protein |  |
| bpl:BURPS1106A\_A1827 | NodT family efflux transporter outer membrane lipoprotein |  |
| bpl:BURPS1106A\_A1828 | hypothetical protein |  |
| bpl:BURPS1106A\_A1829 | rhamnosyltransferase (EC:2.4.1.-); K12990 rhamnosyltransferase [EC:2.4.1.-] |  |
| bpl:BURPS1106A\_A1830 | hypothetical protein |  |
| bpl:BURPS1106A\_A1831 | EmrB/QacA family drug resistance transporter |  |
| bpl:BURPS1106A\_A1832 | rhlB; rhamnosyltransferase I subunit B (EC:2.4.1.-) |  |
| bpl:BURPS1106A\_A1833 | hypothetical protein |  |
| bpl:BURPS1106A\_A1834 | rhlA; rhamnosyltransferase I subunit A (EC:2.4.1.-) |  |
| bpl:BURPS1106A\_A1835 | betI; transcriptional regulator BetI; K02167 TetR/AcrR family transcriptional regulator, transcriptional repressor of bet genes |  |
| bpl:BURPS1106A\_A1836 | betB; betaine aldehyde dehydrogenase (EC:1.2.1.8); K00130 betaine-aldehyde dehydrogenase [EC:1.2.1.8] | ec:1.2.1.8 |
| bpl:BURPS1106A\_A1837 | betA; choline dehydrogenase (EC:1.1.99.1); K00108 choline dehydrogenase [EC:1.1.99.1] | ec:1.1.99.1 |
| bpl:BURPS1106A\_A1838 | hypothetical protein |  |
| bpl:BURPS1106A\_A1839 | hypothetical protein |  |
| bpl:BURPS1106A\_A1840 | hypothetical protein |  |
| bpl:BURPS1106A\_A1841 | major facilitator family transporter; K08369 MFS transporter, putative metabolite:H+ symporter |  |
| bpl:BURPS1106A\_A1842 | response regulator/sensory box histidine kinase; K07675 two-component system, NarL family, sensor histidine kinase UhpB [EC:2.7.13.3] | ec:2.7.13.3 |
| bpl:BURPS1106A\_A1843 | response regulator |  |
| bpl:BURPS1106A\_A1844 | sensory box histidine kinase (EC:2.7.3.-); K00936 [EC:2.7.3.-] |  |
| bpl:BURPS1106A\_A1845 | hypothetical protein |  |

  
**Neighborhood Representations for "bpm:BURPS1710b\_A0375"**  

| ID | Annotation | EC number |
| --- | --- | --- |
| bpm:BURPS1710b\_A0365 | srpH; serine acetyltransferase; K00640 serine O-acetyltransferase [EC:2.3.1.30] | ec:2.3.1.30 |
| bpm:BURPS1710b\_A0366 | rhodanese-like domain-containing protein |  |
| bpm:BURPS1710b\_A0367 | ptrB; prolyl oligopeptidase; K01322 prolyl oligopeptidase [EC:3.4.21.26] | ec:3.4.21.26 |
| bpm:BURPS1710b\_A0369 | hypothetical protein |  |
| bpm:BURPS1710b\_A0368 | emrA; multidrug resistance protein; K03543 multidrug resistance protein A |  |
| bpm:BURPS1710b\_A0370 | RND efflux system outer membrane lipoprotein |  |
| bpm:BURPS1710b\_A0371 | rhlC; rhamnosyltransferase II; K12990 rhamnosyltransferase [EC:2.4.1.-] |  |
| bpm:BURPS1710b\_A0372 | bcrA; multidrug resistance protein |  |
| bpm:BURPS1710b\_A0373 | rhlB-1; rhamnosyltransferase I subunit B |  |
| bpm:BURPS1710b\_A0374 | rhlA-2; rhamnosyltransferase 1 subunit A |  |
| bpm:BURPS1710b\_A0375 | betI; transcriptional regulator BetI; K02167 TetR/AcrR family transcriptional regulator, transcriptional repressor of bet genes |  |
| bpm:BURPS1710b\_A0376 | betB; betaine aldehyde dehydrogenase; K00130 betaine-aldehyde dehydrogenase [EC:1.2.1.8] | ec:1.2.1.8 |
| bpm:BURPS1710b\_A0377 | betA; choline dehydrogenase; K00108 choline dehydrogenase [EC:1.1.99.1] | ec:1.1.99.1 |
| bpm:BURPS1710b\_A0378 | hypothetical protein |  |
| bpm:BURPS1710b\_A0379 | major facilitator family transporter; K08369 MFS transporter, putative metabolite:H+ symporter |  |
| bpm:BURPS1710b\_A0380 | sensory box histidine kinase/response regulator; K07675 two-component system, NarL family, sensor histidine kinase UhpB [EC:2.7.13.3] | ec:2.7.13.3 |
| bpm:BURPS1710b\_A0381 | rre-1; response regulator |  |
| bpm:BURPS1710b\_A0382 | sensory box histidine kinase |  |
| bpm:BURPS1710b\_A0383 | OsmC-like protein |  |
| bpm:BURPS1710b\_A0384 | 2-hydroxyacid dehydrogenase |  |
| bpm:BURPS1710b\_A0385 | putative lipoprotein |  |

  
**Neighborhood Representations for "bps:BPSS1353"**  

| ID | Annotation | EC number |
| --- | --- | --- |
| bps:BPSS1343 | DNA-binding protein |  |
| bps:BPSS1344 | serine acetyltransferase; K00640 serine O-acetyltransferase [EC:2.3.1.30] | ec:2.3.1.30 |
| bps:BPSS1345 | hypothetical protein |  |
| bps:BPSS1346 | peptidase; K01322 prolyl oligopeptidase [EC:3.4.21.26] | ec:3.4.21.26 |
| bps:BPSS1347 | HlyD family efflux pump protein; K03543 multidrug resistance protein A |  |
| bps:BPSS1348 | outer membrane efflux protein |  |
| bps:BPSS1349 | dTDP-rhamnosyl transferase |  |
| bps:BPSS1350 | efflux/sugar transport/multidrug resistance protein |  |
| bps:BPSS1351 | rhamnosyltransferase |  |
| bps:BPSS1352 | fatty acid biosynthetic protein |  |
| bps:BPSS1353 | betI; transcriptional regulator BetI; K02167 TetR/AcrR family transcriptional regulator, transcriptional repressor of bet genes |  |
| bps:BPSS1354 | betB; betaine aldehyde dehydrogenase (EC:1.2.1.8); K00130 betaine-aldehyde dehydrogenase [EC:1.2.1.8] | ec:1.2.1.8 |
| bps:BPSS1355 | betA; choline dehydrogenase (EC:1.1.99.1); K00108 choline dehydrogenase [EC:1.1.99.1] | ec:1.1.99.1 |
| bps:BPSS1356 | hypothetical protein |  |
| bps:BPSS1357 | MFS sugar transporter; K08369 MFS transporter, putative metabolite:H+ symporter |  |
| bps:BPSS1358 | sensor kinase; K07675 two-component system, NarL family, sensor histidine kinase UhpB [EC:2.7.13.3] | ec:2.7.13.3 |
| bps:BPSS1359 | response regulator protein |  |
| bps:BPSS1360 | two-component sensor kinase |  |
| bps:BPSS1361 | stress-induced protein |  |
| bps:BPSS1362 | 2-hydroxyacid dehydrogenase |  |
| bps:BPSS1363 | hypothetical protein |  |

  
**Neighborhood Representations for "bma:BMAA0916"**  

| ID | Annotation | EC number |
| --- | --- | --- |
| bma:BMAA0906 | 2-hydroxyacid dehydrogenase |  |
| bma:BMAA0907 | hypothetical protein |  |
| bma:BMAA0908 | sensory box histidine kinase |  |
| bma:BMAA0909 | response regulator |  |
| bma:BMAA0910 | sensory box histidine kinase/response regulator; K07675 two-component system, NarL family, sensor histidine kinase UhpB [EC:2.7.13.3] | ec:2.7.13.3 |
| bma:BMAA0911 | major facilitator family transporter; K08369 MFS transporter, putative metabolite:H+ symporter |  |
| bma:BMAA0912 | hypothetical protein |  |
| bma:BMAA0913 | hypothetical protein |  |
| bma:BMAA0914 | betA; choline dehydrogenase (EC:1.1.99.1); K00108 choline dehydrogenase [EC:1.1.99.1] | ec:1.1.99.1 |
| bma:BMAA0915 | betB; betaine aldehyde dehydrogenase (EC:1.2.1.8); K00130 betaine-aldehyde dehydrogenase [EC:1.2.1.8] | ec:1.2.1.8 |
| bma:BMAA0916 | betI; transcriptional regulator BetI; K02167 TetR/AcrR family transcriptional regulator, transcriptional repressor of bet genes |  |
| bma:BMAA0917 | IS407A, transposase OrfA; K07497 putative transposase |  |
| bma:BMAA0918 | IS407A, transposase OrfB; K07497 putative transposase |  |
| bma:BMAA0919 | rhlA-2; rhamnosyltransferase 1, subunit A (EC:2.4.1.-) |  |
| bma:BMAA0920 | hypothetical protein |  |
| bma:BMAA0921 | rhlB-2; rhamnosyltransferase I, subunit B (EC:2.4.1.-) |  |
| bma:BMAA0922 | EmrB/QacA family drug resistance transporter |  |
| bma:BMAA0923 | rhamnosyltransferase family protein; K12990 rhamnosyltransferase [EC:2.4.1.-] |  |
| bma:BMAA0924 | RND efflux system outer membrane lipoprotein |  |
| bma:BMAA0925 | multidrug resistance protein; K03543 multidrug resistance protein A |  |
| bma:BMAA0926 | hypothetical protein |  |

  
**Neighborhood Representations for "bgl:bglu\_2g05640"**  

| ID | Annotation | EC number |
| --- | --- | --- |
| bgl:bglu\_2g05540 | Ornithine cyclodeaminase/mu-crystallin; K01750 ornithine cyclodeaminase [EC:4.3.1.12] | ec:4.3.1.12 |
| bgl:bglu\_2g05550 | serine/threonine dehydratase; K01754 threonine dehydratase [EC:4.3.1.19] | ec:4.3.1.19 |
| bgl:bglu\_2g05560 | SMP-30/gluconolaconase/LRE-like region-containing protein |  |
| bgl:bglu\_2g05570 | 3-carboxymuconate cyclase-like protein; K07404 6-phosphogluconolactonase [EC:3.1.1.31] | ec:3.1.1.31 |
| bgl:bglu\_2g05580 | major facilitator family transporter; K08369 MFS transporter, putative metabolite:H+ symporter |  |
| bgl:bglu\_2g05590 | ABC transporter inner membrane subunit; K01992 ABC-2 type transport system permease protein |  |
| bgl:bglu\_2g05600 | ABC transporter ATP-binding protein; K01990 ABC-2 type transport system ATP-binding protein |  |
| bgl:bglu\_2g05610 | response regulator receiver protein |  |
| bgl:bglu\_2g05620 | choline dehydrogenase; K00108 choline dehydrogenase [EC:1.1.99.1] | ec:1.1.99.1 |
| bgl:bglu\_2g05630 | betaine aldehyde dehydrogenase; K00130 betaine-aldehyde dehydrogenase [EC:1.2.1.8] | ec:1.2.1.8 |
| bgl:bglu\_2g05640 | transcriptional regulator BetI; K02167 TetR/AcrR family transcriptional regulator, transcriptional repressor of bet genes |  |
| bgl:bglu\_2g05650 | Rhamnosyltransferase 1 subunit A |  |
| bgl:bglu\_2g05660 | glycosyl transferase family protein |  |
| bgl:bglu\_2g05670 | EmrB/QacA subfamily drug resistance transporter |  |
| bgl:bglu\_2g05680 | Rhamnosyltransferase; K12990 rhamnosyltransferase [EC:2.4.1.-] |  |
| bgl:bglu\_2g05690 | NodT family RND efflux system outer membrane lipoprotein |  |
| bgl:bglu\_2g05700 | Secretion protein HlyD; K03543 multidrug resistance protein A |  |
| bgl:bglu\_2g05710 | chitinase |  |
| bgl:bglu\_2g05720 | MhpE-like protein; K01666 4-hydroxy 2-oxovalerate aldolase [EC:4.1.3.39] | ec:4.1.3.39 |
| bgl:bglu\_2g05730 | putative coenzyme PQQ synthesis protein c; K06137 pyrroloquinoline-quinone synthase [EC:1.3.3.11] | ec:1.3.3.11 |
| bgl:bglu\_2g05740 | putative Branched-chain amino acid aminotransferase; K00826 branched-chain amino acid aminotransferase [EC:2.6.1.42] | ec:2.6.1.42 |

  
**Neighborhood Representations for "pmy:Pmen\_0483"**  

| ID | Annotation | EC number |
| --- | --- | --- |
| pmy:Pmen\_0473 | L-alanine dehydrogenase (EC:1.4.1.1); K00259 alanine dehydrogenase [EC:1.4.1.1] | ec:1.4.1.1 |
| pmy:Pmen\_0474 | intradiol ring-cleavage dioxygenase; K00449 protocatechuate 3,4-dioxygenase, beta subunit [EC:1.13.11.3] | ec:1.13.11.3 |
| pmy:Pmen\_0475 | citrate transporter |  |
| pmy:Pmen\_0476 | hypothetical protein; K07007 |  |
| pmy:Pmen\_0477 | extracellular solute-binding protein; K02030 polar amino acid transport system substrate-binding protein |  |
| pmy:Pmen\_0478 | response regulator receiver modulated diguanylate cyclase |  |
| pmy:Pmen\_0479 | ATP-dependent RNA helicase DbpA; K05591 ATP-independent RNA helicase DbpA [EC:3.6.4.13] | ec:3.6.4.13 |
| pmy:Pmen\_0480 | major facilitator superfamily transporter |  |
| pmy:Pmen\_0481 | choline dehydrogenase (EC:1.1.99.1); K00108 choline dehydrogenase [EC:1.1.99.1] | ec:1.1.99.1 |
| pmy:Pmen\_0482 | betaine aldehyde dehydrogenase (EC:1.2.1.8); K00130 betaine-aldehyde dehydrogenase [EC:1.2.1.8] | ec:1.2.1.8 |
| pmy:Pmen\_0483 | transcriptional regulator BetI; K02167 TetR/AcrR family transcriptional regulator, transcriptional repressor of bet genes |  |
| pmy:Pmen\_0484 | glycine betaine ABC transporter substrate-binding protein; K02002 glycine betaine/proline transport system substrate-binding protein |  |
| pmy:Pmen\_0485 | glycine betaine ABC transporter substrate-binding protein; K02002 glycine betaine/proline transport system substrate-binding protein |  |
| pmy:Pmen\_0486 | binding-protein-dependent transport system inner membrane protein; K02001 glycine betaine/proline transport system permease protein |  |
| pmy:Pmen\_0487 | glycine betaine/L-proline ABC transporter ATPase; K02000 glycine betaine/proline transport system ATP-binding protein [EC:3.6.3.32] | ec:3.6.3.32 |
| pmy:Pmen\_0488 | glycine betaine ABC transporter substrate-binding protein; K02002 glycine betaine/proline transport system substrate-binding protein |  |
| pmy:Pmen\_0489 | hypothetical protein |  |
| pmy:Pmen\_0490 | transcriptional regulator |  |
| pmy:Pmen\_0491 | hypothetical protein |  |
| pmy:Pmen\_0492 | electron transfer flavoprotein subunit beta; K03521 electron transfer flavoprotein beta subunit |  |
| pmy:Pmen\_0493 | electron transfer flavoprotein subunit alpha; K03522 electron transfer flavoprotein alpha subunit |  |

  
**Neighborhood Representations for "pen:PSEEN0374"**  

| ID | Annotation | EC number |
| --- | --- | --- |
| pen:PSEEN0364 | hypothetical protein |  |
| pen:PSEEN0365 | ParA family chromosome partitioning ATPase (EC:2.7.10.1); K03496 chromosome partitioning protein |  |
| pen:PSEEN0366 | hypothetical protein |  |
| pen:PSEEN0367 | hypothetical protein |  |
| pen:PSEEN0368 | thioredoxin; K03672 thioredoxin 2 [EC:1.8.1.8] | ec:1.8.1.8 |
| pen:PSEEN0369 | hypothetical protein; K08997 hypothetical protein |  |
| pen:PSEEN0370 | potassium efflux protein KefA; K05802 potassium efflux system protein |  |
| pen:PSEEN0371 | cvrA; potassium/proton antiporter; K11105 cell volume regulation protein A |  |
| pen:PSEEN0372 | betA; choline dehydrogenase (EC:1.1.99.1); K00108 choline dehydrogenase [EC:1.1.99.1] | ec:1.1.99.1 |
| pen:PSEEN0373 | betB; betaine aldehyde dehydrogenase (EC:1.2.1.8); K00130 betaine-aldehyde dehydrogenase [EC:1.2.1.8] | ec:1.2.1.8 |
| pen:PSEEN0374 | betI; transcriptional regulator BetI; K02167 TetR/AcrR family transcriptional regulator, transcriptional repressor of bet genes |  |
| pen:PSEEN0375 | betT; choline transporter BetT; K02168 choline/glycine/proline betaine transport protein |  |
| pen:PSEEN0376 | hypothetical protein |  |
| pen:PSEEN0377 | hypothetical protein; K09798 hypothetical protein |  |
| pen:PSEEN0378 | ctpA; carboxy-terminal processing protease (EC:3.4.21.102); K03797 carboxyl-terminal processing protease [EC:3.4.21.102] | ec:3.4.21.102 |
| pen:PSEEN0379 | M23/M37 family metalloendopeptidase |  |
| pen:PSEEN0380 | gpmA; phosphoglyceromutase (EC:5.4.2.1); K15633 2,3-bisphosphoglycerate-independent phosphoglycerate mutase [EC:5.4.2.12] | ec:5.4.2.12 |
| pen:PSEEN0381 | hypothetical protein |  |
| pen:PSEEN0382 | grxC; glutaredoxin; K03676 glutaredoxin 3 |  |
| pen:PSEEN0383 | secB; preprotein translocase subunit SecB; K03071 preprotein translocase subunit SecB |  |
| pen:PSEEN0384 | RNA methyltransferase (EC:2.1.1.-); K03216 tRNA (cytidine/uridine-2'-O-)-methyltransferase [EC:2.1.1.207] | ec:2.1.1.207 |

  
**Neighborhood Representations for "pae:PA5374"**  

| ID | Annotation | EC number |
| --- | --- | --- |
| pae:PA5364 | two-component response regulator |  |
| pae:PA5365 | phoU; phosphate uptake regulatory protein PhoU; K02039 phosphate transport system protein |  |
| pae:PA5366 | pstB; phosphate transporter ATP-binding protein; K02036 phosphate transport system ATP-binding protein [EC:3.6.3.27] | ec:3.6.3.27 |
| pae:PA5367 | pstA; phosphate ABC transporter membrane protein; K02038 phosphate transport system permease protein |  |
| pae:PA5368 | pstC; phosphate ABC transporter membrane protein; K02037 phosphate transport system permease protein |  |
| pae:PA5369 | pstS; phosphate ABC transporter substrate-binding protein; K02040 phosphate transport system substrate-binding protein |  |
| pae:PA5370 | major facilitator superfamily (MFS) transporter |  |
| pae:PA5371 | hypothetical protein; K10806 acyl-CoA thioesterase YciA [EC:3.1.2.-] |  |
| pae:PA5372 | betA; choline dehydrogenase (EC:1.1.99.1); K00108 choline dehydrogenase [EC:1.1.99.1] | ec:1.1.99.1 |
| pae:PA5373 | betB; betaine aldehyde dehydrogenase (EC:1.2.1.8); K00130 betaine-aldehyde dehydrogenase [EC:1.2.1.8] | ec:1.2.1.8 |
| pae:PA5374 | betI; BetI family transcriptional regulator; K02167 TetR/AcrR family transcriptional regulator, transcriptional repressor of bet genes |  |
| pae:PA5375 | betT1; choline transporter BetT; K02168 choline/glycine/proline betaine transport protein |  |
| pae:PA5376 | ABC transporter ATP-binding protein; K02000 glycine betaine/proline transport system ATP-binding protein [EC:3.6.3.32] | ec:3.6.3.32 |
| pae:PA5377 | ABC transporter permease; K02001 glycine betaine/proline transport system permease protein |  |
| pae:PA5378 | hypothetical protein; K02002 glycine betaine/proline transport system substrate-binding protein |  |
| pae:PA5379 | sdaB; L-serine dehydratase; K01752 L-serine dehydratase [EC:4.3.1.17] | ec:4.3.1.17 |
| pae:PA5380 | gbdR; protein GbdR |  |
| pae:PA5381 | hypothetical protein |  |
| pae:PA5382 | transcriptional regulator |  |
| pae:PA5383 | hypothetical protein |  |
| pae:PA5384 | lipolytic protein |  |

  
**Neighborhood Representations for "pag:PLES\_57691"**  

| ID | Annotation | EC number |
| --- | --- | --- |
| pag:PLES\_57641 | pstS; phosphate ABC transporter substrate-binding protein; K02040 phosphate transport system substrate-binding protein |  |
| pag:PLES\_57642 | 5S ribosomal RNA; K01985 5S ribosomal RNA |  |
| pag:PLES\_57643 | 23S ribosomal RNA; K01980 23S ribosomal RNA |  |
| pag:PLES\_57644 | tRNA-Ala; K14218 tRNA Ala |  |
| pag:PLES\_57645 | tRNA-Ile; K14227 tRNA Ile |  |
| pag:PLES\_57646 | 16S ribosomal RNA; K01977 16S ribosomal RNA |  |
| pag:PLES\_57651 | putative major facilitator superfamily transporter |  |
| pag:PLES\_57661 | putative long-chain acyl-CoA thioester hydrolase; K10806 acyl-CoA thioesterase YciA [EC:3.1.2.-] |  |
| pag:PLES\_57671 | betA; choline dehydrogenase; K00108 choline dehydrogenase [EC:1.1.99.1] | ec:1.1.99.1 |
| pag:PLES\_57681 | betB; betaine aldehyde dehydrogenase; K00130 betaine-aldehyde dehydrogenase [EC:1.2.1.8] | ec:1.2.1.8 |
| pag:PLES\_57691 | betI; transcriptional regulator BetI; K02167 TetR/AcrR family transcriptional regulator, transcriptional repressor of bet genes |  |
| pag:PLES\_57701 | betT1; choline transporter BetT; K02168 choline/glycine/proline betaine transport protein |  |
| pag:PLES\_57711 | putative ATP-binding component of ABC transporter; K02000 glycine betaine/proline transport system ATP-binding protein [EC:3.6.3.32] | ec:3.6.3.32 |
| pag:PLES\_57721 | ABC transporter permease; K02001 glycine betaine/proline transport system permease protein |  |
| pag:PLES\_57731 | putative glycine betaine/L-proline ABC transporter, periplasmic component; K02002 glycine betaine/proline transport system substrate-binding protein |  |
| pag:PLES\_57741 | hypothetical protein |  |
| pag:PLES\_57751 | sdaB; L-serine dehydratase; K01752 L-serine dehydratase [EC:4.3.1.17] | ec:4.3.1.17 |
| pag:PLES\_57761 | putative transcriptional regulator |  |
| pag:PLES\_57771 | hypothetical protein |  |
| pag:PLES\_57781 | putative transcriptional regulator |  |
| pag:PLES\_57791 | hypothetical protein |  |

  
**Neighborhood Representations for "pap:PSPA7\_6159"**  

| ID | Annotation | EC number |
| --- | --- | --- |
| pap:PSPA7\_6149 | rrfD; 5S ribosomal RNA; K01985 5S ribosomal RNA |  |
| pap:PSPA7\_6150 | rrlD; 23S ribosomal RNA; K01980 23S ribosomal RNA |  |
| pap:PSPA7\_6151 | tRNA-Ala; K14218 tRNA Ala |  |
| pap:PSPA7\_6152 | tRNA-Ile; K14227 tRNA Ile |  |
| pap:PSPA7\_6153 | rrsD; 16S ribosomal RNA; K01977 16S ribosomal RNA |  |
| pap:PSPA7\_6154 | hypothetical protein |  |
| pap:PSPA7\_6155 | MFS family transporter |  |
| pap:PSPA7\_6156 | hypothetical protein; K10806 acyl-CoA thioesterase YciA [EC:3.1.2.-] |  |
| pap:PSPA7\_6157 | betA; choline dehydrogenase (EC:1.1.99.1); K00108 choline dehydrogenase [EC:1.1.99.1] | ec:1.1.99.1 |
| pap:PSPA7\_6158 | betB; betaine aldehyde dehydrogenase (EC:1.2.1.8); K00130 betaine-aldehyde dehydrogenase [EC:1.2.1.8] | ec:1.2.1.8 |
| pap:PSPA7\_6159 | betI; transcriptional regulator BetI; K02167 TetR/AcrR family transcriptional regulator, transcriptional repressor of bet genes |  |
| pap:PSPA7\_6160 | betT1; choline transporter BetT; K02168 choline/glycine/proline betaine transport protein |  |
| pap:PSPA7\_6161 | choV; choline ABC transporter ATP-binding protein; K02000 glycine betaine/proline transport system ATP-binding protein [EC:3.6.3.32] | ec:3.6.3.32 |
| pap:PSPA7\_6162 | choW; choline ABC transporter permease; K02001 glycine betaine/proline transport system permease protein |  |
| pap:PSPA7\_6163 | hypothetical protein; K02002 glycine betaine/proline transport system substrate-binding protein |  |
| pap:PSPA7\_6164 | hypothetical protein |  |
| pap:PSPA7\_6165 | sdaA1; L-serine dehydratase (EC:4.3.1.17); K01752 L-serine dehydratase [EC:4.3.1.17] | ec:4.3.1.17 |
| pap:PSPA7\_6166 | putative transcriptional regulator |  |
| pap:PSPA7\_6167 | hypothetical protein |  |
| pap:PSPA7\_6168 | putative transcriptional regulator |  |
| pap:PSPA7\_6170 | hypothetical protein |  |

  
**Neighborhood Representations for "pau:PA14\_70970"**  

| ID | Annotation | EC number |
| --- | --- | --- |
| pau:PA14\_70860 | hypothetical protein; K02040 phosphate transport system substrate-binding protein |  |
| pau:PA14\_70870 | 5S ribosomal RNA; K01985 5S ribosomal RNA |  |
| pau:PA14\_70880 | 23S ribosomal RNA; K01980 23S ribosomal RNA |  |
| pau:PA14\_70890 | tRNA-Ala; K14218 tRNA Ala |  |
| pau:PA14\_70900 | tRNA-Ile; K14227 tRNA Ile |  |
| pau:PA14\_70910 | 16S ribosomal RNA; K01977 16S ribosomal RNA |  |
| pau:PA14\_70920 | major facilitator transporter |  |
| pau:PA14\_70930 | hypothetical protein; K10806 acyl-CoA thioesterase YciA [EC:3.1.2.-] |  |
| pau:PA14\_70940 | betA; choline dehydrogenase (EC:1.1.99.1); K00108 choline dehydrogenase [EC:1.1.99.1] | ec:1.1.99.1 |
| pau:PA14\_70950 | betB; betaine aldehyde dehydrogenase (EC:1.2.1.8); K00130 betaine-aldehyde dehydrogenase [EC:1.2.1.8] | ec:1.2.1.8 |
| pau:PA14\_70970 | betI; transcriptional regulator BetI; K02167 TetR/AcrR family transcriptional regulator, transcriptional repressor of bet genes |  |
| pau:PA14\_70980 | betT1; choline transporter BetT; K02168 choline/glycine/proline betaine transport protein |  |
| pau:PA14\_71000 | lycine betaine/L-proline ABC transporter, ATP-binding subunit; K02000 glycine betaine/proline transport system ATP-binding protein [EC:3.6.3.32] | ec:3.6.3.32 |
| pau:PA14\_71020 | BC-type proline/glycine betaine transport system, permease component; K02001 glycine betaine/proline transport system permease protein |  |
| pau:PA14\_71030 | hypothetical protein; K02002 glycine betaine/proline transport system substrate-binding protein |  |
| pau:PA14\_71060 | sdaB; L-serine dehydratase; K01752 L-serine dehydratase [EC:4.3.1.17] | ec:4.3.1.17 |
| pau:PA14\_71070 | AraC family transcriptional regulator |  |
| pau:PA14\_71080 | hypothetical protein |  |
| pau:PA14\_71090 | yeiE; LysR family transcriptional regulator |  |
| pau:PA14\_71100 | yeiH; hypothetical protein |  |
| pau:PA14\_71110 | lipolytic protein |  |

  
**Neighborhood Representations for "psb:Psyr\_4734"**  

| ID | Annotation | EC number |
| --- | --- | --- |
| psb:Psyr\_4724 | acyltransferase |  |
| psb:Psyr\_4725 | hypothetical protein |  |
| psb:Psyr\_4726 | hypothetical protein |  |
| psb:Psyr\_4727 | glutathione S-transferase |  |
| psb:Psyr\_4728 | hypothetical protein |  |
| psb:Psyr\_4729 | PepSY-associated TM helix |  |
| psb:Psyr\_4730 | FecR protein; K07165 transmembrane sensor |  |
| psb:Psyr\_4731 | RNA polymerase sigma factor; K03088 RNA polymerase sigma-70 factor, ECF subfamily |  |
| psb:Psyr\_4732 | choline dehydrogenase (EC:1.1.99.1); K00108 choline dehydrogenase [EC:1.1.99.1] | ec:1.1.99.1 |
| psb:Psyr\_4733 | betaine aldehyde dehydrogenase (EC:1.2.1.8); K00130 betaine-aldehyde dehydrogenase [EC:1.2.1.8] | ec:1.2.1.8 |
| psb:Psyr\_4734 | transcriptional regulator BetI; K02167 TetR/AcrR family transcriptional regulator, transcriptional repressor of bet genes |  |
| psb:Psyr\_4735 | HSR1-like GTP-binding protein |  |
| psb:Psyr\_4736 | hypothetical protein |  |
| psb:Psyr\_4737 | dihydrofolate reductase (EC:1.5.1.3); K00287 dihydrofolate reductase [EC:1.5.1.3] | ec:1.5.1.3 |
| psb:Psyr\_4738 | hypothetical protein |  |
| psb:Psyr\_4739 | trmB; tRNA (guanine-N(7)-)-methyltransferase (EC:2.1.1.33); K03439 tRNA (guanine-N7-)-methyltransferase [EC:2.1.1.33] | ec:2.1.1.33 |
| psb:Psyr\_4740 | thiG; thiazole synthase; K03149 thiamine biosynthesis ThiG |  |
| psb:Psyr\_4741 | sulfur carrier protein ThiS; K03154 sulfur carrier protein |  |
| psb:Psyr\_4742 | hypothetical protein |  |
| psb:Psyr\_4743 | mtgA; monofunctional biosynthetic peptidoglycan transglycosylase; K03814 monofunctional biosynthetic peptidoglycan transglycosylase [EC:2.4.1.-] |  |
| psb:Psyr\_4744 | hypothetical protein |  |

  
**Neighborhood Representations for "psp:PSPPH\_4768"**  

| ID | Annotation | EC number |
| --- | --- | --- |
| psp:PSPPH\_4758 | hypothetical protein |  |
| psp:PSPPH\_4759 | acyltransferase (EC:2.7.7.19) |  |
| psp:PSPPH\_4760 | hypothetical protein |  |
| psp:PSPPH\_4761 | glutathione S-transferase |  |
| psp:PSPPH\_4762 | hypothetical protein |  |
| psp:PSPPH\_4763 | hypothetical protein |  |
| psp:PSPPH\_4764 | transmembrane sensor |  |
| psp:PSPPH\_4765 | RNA polymerase sigma factor; K03088 RNA polymerase sigma-70 factor, ECF subfamily |  |
| psp:PSPPH\_4766 | betA; choline dehydrogenase (EC:1.1.99.1); K00108 choline dehydrogenase [EC:1.1.99.1] | ec:1.1.99.1 |
| psp:PSPPH\_4767 | betB; betaine aldehyde dehydrogenase (EC:1.2.1.8); K00130 betaine-aldehyde dehydrogenase [EC:1.2.1.8] | ec:1.2.1.8 |
| psp:PSPPH\_4768 | betI; transcriptional regulator BetI; K02167 TetR/AcrR family transcriptional regulator, transcriptional repressor of bet genes |  |
| psp:PSPPH\_4769 | hypothetical protein |  |
| psp:PSPPH\_4770 | hypothetical protein |  |
| psp:PSPPH\_4771 | folA; dihydrofolate reductase (EC:1.5.1.3); K00287 dihydrofolate reductase [EC:1.5.1.3] | ec:1.5.1.3 |
| psp:PSPPH\_4772 | hypothetical protein |  |
| psp:PSPPH\_4773 | trmB; tRNA (guanine-N(7)-)-methyltransferase (EC:2.1.1.33); K03439 tRNA (guanine-N7-)-methyltransferase [EC:2.1.1.33] | ec:2.1.1.33 |
| psp:PSPPH\_4774 | thiG; thiazole synthase; K03149 thiamine biosynthesis ThiG |  |
| psp:PSPPH\_4775 | thiS; sulfur carrier protein ThiS; K03154 sulfur carrier protein |  |
| psp:PSPPH\_4776 | hypothetical protein |  |
| psp:PSPPH\_4777 | mtgA; monofunctional biosynthetic peptidoglycan transglycosylase (EC:2.4.2.-); K03814 monofunctional biosynthetic peptidoglycan transglycosylase [EC:2.4.1.-] |  |
| psp:PSPPH\_4778 | rpoH; RNA polymerase factor sigma-32; K03089 RNA polymerase sigma-32 factor |  |

  
**Neighborhood Representations for "pst:PSPTO\_0440"**  

| ID | Annotation | EC number |
| --- | --- | --- |
| pst:PSPTO\_0430 | rpoH; RNA polymerase sigma-32 factor; K03089 RNA polymerase sigma-32 factor |  |
| pst:PSPTO\_0431 | mtgA; monofunctional biosynthetic peptidoglycan transglycosylase; K03814 monofunctional biosynthetic peptidoglycan transglycosylase [EC:2.4.1.-] |  |
| pst:PSPTO\_0432 | hypothetical protein |  |
| pst:PSPTO\_0433 | thiS; thiamine biosynthesis protein ThiS; K03154 sulfur carrier protein |  |
| pst:PSPTO\_0434 | thiG; thiazole biosynthesis protein ThiG; K03149 thiamine biosynthesis ThiG |  |
| pst:PSPTO\_0435 | tRNA (guanine-N(7)-)-methyltransferase; K03439 tRNA (guanine-N7-)-methyltransferase [EC:2.1.1.33] | ec:2.1.1.33 |
| pst:PSPTO\_0436 | hypothetical protein |  |
| pst:PSPTO\_0437 | folA; dihydrofolate reductase; K00287 dihydrofolate reductase [EC:1.5.1.3] | ec:1.5.1.3 |
| pst:PSPTO\_0438 | hypothetical protein |  |
| pst:PSPTO\_0439 | hypothetical protein |  |
| pst:PSPTO\_0440 | betI; regulatory protein BetI; K02167 TetR/AcrR family transcriptional regulator, transcriptional repressor of bet genes |  |
| pst:PSPTO\_0441 | betB; betaine aldehyde dehydrogenase BADH; K00130 betaine-aldehyde dehydrogenase [EC:1.2.1.8] | ec:1.2.1.8 |
| pst:PSPTO\_0442 | hypothetical protein |  |
| pst:PSPTO\_0443 | betA; choline dehydrogenase; K00108 choline dehydrogenase [EC:1.1.99.1] | ec:1.1.99.1 |
| pst:PSPTO\_0444 | RNA polymerase sigma-70 family protein; K03088 RNA polymerase sigma-70 factor, ECF subfamily |  |
| pst:PSPTO\_0445 | regulatory protein |  |
| pst:PSPTO\_0446 | membrane protein |  |
| pst:PSPTO\_0447 | hypothetical protein |  |
| pst:PSPTO\_0448 | glutathione S-transferase |  |
| pst:PSPTO\_0449 | ISPsy8, transposase OrfA; K07483 transposase |  |
| pst:PSPTO\_0450 | ISPsy8, transposase OrfB |  |

  
**Neighborhood Representations for "pfl:PFL\_5766"**  

| ID | Annotation | EC number |
| --- | --- | --- |
| pfl:PFL\_5756 | gamma-butyrobetaine dioxygenase (EC:1.14.11.1); K00471 gamma-butyrobetaine dioxygenase [EC:1.14.11.1] | ec:1.14.11.1 |
| pfl:PFL\_5757 | hypothetical protein |  |
| pfl:PFL\_5758 | hypothetical protein |  |
| pfl:PFL\_5759 | aacA7; aminoglycoside N(6')-acetyltransferase type 1 (EC:2.3.1.82) |  |
| pfl:PFL\_5760 | gbdR; transcriptional regulator GbdR |  |
| pfl:PFL\_5761 | sdaA; L-serine ammonia-lyase 1 (EC:4.3.1.17); K01752 L-serine dehydratase [EC:4.3.1.17] | ec:4.3.1.17 |
| pfl:PFL\_5762 | choX; choline ABC transporter periplasmic choline-binding protein; K02002 glycine betaine/proline transport system substrate-binding protein |  |
| pfl:PFL\_5763 | choW; choline ABC transporter permease; K02001 glycine betaine/proline transport system permease protein |  |
| pfl:PFL\_5764 | choV; choline ABC transporter ATP-binding protein; K02000 glycine betaine/proline transport system ATP-binding protein [EC:3.6.3.32] | ec:3.6.3.32 |
| pfl:PFL\_5765 | betaine/carnitine/choline family transporter; K02168 choline/glycine/proline betaine transport protein |  |
| pfl:PFL\_5766 | betI; transcriptional regulator BetI; K02167 TetR/AcrR family transcriptional regulator, transcriptional repressor of bet genes |  |
| pfl:PFL\_5767 | betB; betaine aldehyde dehydrogenase (EC:1.2.1.8); K00130 betaine-aldehyde dehydrogenase [EC:1.2.1.8] | ec:1.2.1.8 |
| pfl:PFL\_5768 | betA; choline dehydrogenase (EC:1.1.99.1); K00108 choline dehydrogenase [EC:1.1.99.1] | ec:1.1.99.1 |
| pfl:PFL\_5769 | TldD/PmbA family protein |  |
| pfl:PFL\_5770 | hypothetical protein |  |
| pfl:PFL\_5771 | EmrB/QacA family drug resistance transporter |  |
| pfl:PFL\_5772 | dbpA; ATP-dependent RNA helicase DbpA (EC:3.6.1.-); K05591 ATP-independent RNA helicase DbpA [EC:3.6.4.13] | ec:3.6.4.13 |
| pfl:PFL\_5773 | pyridine nucleotide-disulfide oxidoreductase; K07007 |  |
| pfl:PFL\_5774 | hypothetical protein |  |
| pfl:PFL\_5775 | hypothetical protein; K02030 polar amino acid transport system substrate-binding protein |  |
| pfl:PFL\_5776 | GNAT family acetyltransferase; K02348 ElaA protein |  |

  
**Neighborhood Representations for "ppf:Pput\_4935"**  

| ID | Annotation | EC number |
| --- | --- | --- |
| ppf:Pput\_4925 | RNA methyltransferase; K03216 tRNA (cytidine/uridine-2'-O-)-methyltransferase [EC:2.1.1.207] | ec:2.1.1.207 |
| ppf:Pput\_4926 | preprotein translocase subunit SecB; K03071 preprotein translocase subunit SecB |  |
| ppf:Pput\_4927 | glutaredoxin 3; K03676 glutaredoxin 3 |  |
| ppf:Pput\_4928 | rhodanese domain-containing protein |  |
| ppf:Pput\_4929 | phosphoglyceromutase; K15633 2,3-bisphosphoglycerate-independent phosphoglycerate mutase [EC:5.4.2.12] | ec:5.4.2.12 |
| ppf:Pput\_4930 | peptidase M23B |  |
| ppf:Pput\_4931 | carboxyl-terminal protease; K03797 carboxyl-terminal processing protease [EC:3.4.21.102] | ec:3.4.21.102 |
| ppf:Pput\_4932 | hypothetical protein; K09798 hypothetical protein |  |
| ppf:Pput\_4933 | hypothetical protein |  |
| ppf:Pput\_4934 | choline/carnitine/betaine transporter; K02168 choline/glycine/proline betaine transport protein |  |
| ppf:Pput\_4935 | transcriptional regulator BetI; K02167 TetR/AcrR family transcriptional regulator, transcriptional repressor of bet genes |  |
| ppf:Pput\_4936 | betaine aldehyde dehydrogenase; K00130 betaine-aldehyde dehydrogenase [EC:1.2.1.8] | ec:1.2.1.8 |
| ppf:Pput\_4937 | choline dehydrogenase; K00108 choline dehydrogenase [EC:1.1.99.1] | ec:1.1.99.1 |
| ppf:Pput\_4938 | hypothetical protein |  |
| ppf:Pput\_4939 | cvrA, nhaP2, ycgO; potassium/proton antiporter; K11105 cell volume regulation protein A |  |
| ppf:Pput\_4940 | potassium efflux protein KefA; K05802 potassium efflux system protein |  |
| ppf:Pput\_4941 | hypothetical protein |  |
| ppf:Pput\_4942 | thioredoxin; K03672 thioredoxin 2 [EC:1.8.1.8] | ec:1.8.1.8 |
| ppf:Pput\_4943 | cobyrinic acid a,c-diamide synthase; K03496 chromosome partitioning protein |  |
| ppf:Pput\_4944 | LysR family transcriptional regulator |  |
| ppf:Pput\_4945 | major facilitator superfamily transporter; K08224 MFS transporter, YNFM family, putative membrane transport protein |  |

  
**Neighborhood Representations for "ppw:PputW619\_0403"**  

| ID | Annotation | EC number |
| --- | --- | --- |
| ppw:PputW619\_0393 | hypothetical protein |  |
| ppw:PputW619\_0394 | major facilitator transporter; K08224 MFS transporter, YNFM family, putative membrane transport protein |  |
| ppw:PputW619\_0395 | LysR family transcriptional regulator |  |
| ppw:PputW619\_0396 | cobyrinic acid ac-diamide synthase; K03496 chromosome partitioning protein |  |
| ppw:PputW619\_0397 | thioredoxin; K03672 thioredoxin 2 [EC:1.8.1.8] | ec:1.8.1.8 |
| ppw:PputW619\_0398 | hypothetical protein |  |
| ppw:PputW619\_0399 | potassium efflux protein KefA; K05802 potassium efflux system protein |  |
| ppw:PputW619\_0400 | potassium/proton antiporter; K11105 cell volume regulation protein A |  |
| ppw:PputW619\_0401 | choline dehydrogenase (EC:1.1.99.1); K00108 choline dehydrogenase [EC:1.1.99.1] | ec:1.1.99.1 |
| ppw:PputW619\_0402 | betaine aldehyde dehydrogenase (EC:1.2.1.8); K00130 betaine-aldehyde dehydrogenase [EC:1.2.1.8] | ec:1.2.1.8 |
| ppw:PputW619\_0403 | transcriptional regulator BetI; K02167 TetR/AcrR family transcriptional regulator, transcriptional repressor of bet genes |  |
| ppw:PputW619\_0404 | choline/carnitine/betaine transporter; K02168 choline/glycine/proline betaine transport protein |  |
| ppw:PputW619\_0405 | hypothetical protein |  |
| ppw:PputW619\_0406 | hypothetical protein; K09798 hypothetical protein |  |
| ppw:PputW619\_0407 | carboxyl-terminal protease (EC:3.4.21.102); K03797 carboxyl-terminal processing protease [EC:3.4.21.102] | ec:3.4.21.102 |
| ppw:PputW619\_0408 | peptidase M23B |  |
| ppw:PputW619\_0409 | phosphoglyceromutase (EC:5.4.2.1); K15633 2,3-bisphosphoglycerate-independent phosphoglycerate mutase [EC:5.4.2.12] | ec:5.4.2.12 |
| ppw:PputW619\_0410 | rhodanese domain-containing protein |  |
| ppw:PputW619\_0411 | glutaredoxin 3; K03676 glutaredoxin 3 |  |
| ppw:PputW619\_0412 | preprotein translocase subunit SecB; K03071 preprotein translocase subunit SecB |  |
| ppw:PputW619\_0413 | RNA methyltransferase; K03216 tRNA (cytidine/uridine-2'-O-)-methyltransferase [EC:2.1.1.207] | ec:2.1.1.207 |

  
**Neighborhood Representations for "hel:HELO\_1861"**  

| ID | Annotation | EC number |
| --- | --- | --- |
| hel:HELO\_1851 | hypothetical protein |  |
| hel:HELO\_1852 | azu; azurin |  |
| hel:HELO\_1853 | qor; quinone oxidoreductase (EC:1.6.5.5); K00344 NADPH2:quinone reductase [EC:1.6.5.5] | ec:1.6.5.5 |
| hel:HELO\_1854 | RpiR family transcriptional regulator |  |
| hel:HELO\_1855 | hypothetical protein |  |
| hel:HELO\_1856 | pcaC; hypothetical protein; K01607 4-carboxymuconolactone decarboxylase [EC:4.1.1.44] | ec:4.1.1.44 |
| hel:HELO\_1857 | TrkA-C domain-containing protein |  |
| hel:HELO\_1858 | fadE; acyl-CoA dehydrogenase (EC:1.3.99.-); K06445 acyl-CoA dehydrogenase [EC:1.3.99.-] |  |
| hel:HELO\_1859 | betA; choline dehydrogenase BetA (EC:1.1.99.1); K00108 choline dehydrogenase [EC:1.1.99.1] | ec:1.1.99.1 |
| hel:HELO\_1860 | betB; betaine aldehyde dehydrogenase BetB (EC:1.2.1.8); K00130 betaine-aldehyde dehydrogenase [EC:1.2.1.8] | ec:1.2.1.8 |
| hel:HELO\_1861 | betI; BetI family transcriptional regulator; K02167 TetR/AcrR family transcriptional regulator, transcriptional repressor of bet genes |  |
| hel:HELO\_1862 | ABC transporter periplasmic protein; K02002 glycine betaine/proline transport system substrate-binding protein |  |
| hel:HELO\_1863 | copB; copper resistance B; K07233 copper resistance protein B |  |
| hel:HELO\_1864 | pcoA; CopA family copper-resistance protein |  |
| hel:HELO\_1865 | phoQ; integral membrane sensor signal transduction histidine kinase (EC:2.7.3.-) |  |
| hel:HELO\_1866 | phoP; transcriptional regulator |  |
| hel:HELO\_1867 | copC; copper-binding protein |  |
| hel:HELO\_1868 | major facilitator superfamily transporter |  |
| hel:HELO\_1869 | hypothetical protein |  |
| hel:HELO\_1870 | hypothetical protein |  |
| hel:HELO\_1871 | hypothetical protein |  |

  
**Neighborhood Representations for "ppg:PputGB1\_5113"**  

| ID | Annotation | EC number |
| --- | --- | --- |
| ppg:PputGB1\_5103 | RNA methyltransferase; K03216 tRNA (cytidine/uridine-2'-O-)-methyltransferase [EC:2.1.1.207] | ec:2.1.1.207 |
| ppg:PputGB1\_5104 | preprotein translocase subunit SecB; K03071 preprotein translocase subunit SecB |  |
| ppg:PputGB1\_5105 | glutaredoxin 3; K03676 glutaredoxin 3 |  |
| ppg:PputGB1\_5106 | rhodanese domain-containing protein |  |
| ppg:PputGB1\_5107 | phosphoglyceromutase (EC:5.4.2.1); K15633 2,3-bisphosphoglycerate-independent phosphoglycerate mutase [EC:5.4.2.12] | ec:5.4.2.12 |
| ppg:PputGB1\_5108 | peptidase M23B |  |
| ppg:PputGB1\_5109 | carboxyl-terminal protease (EC:3.4.21.102); K03797 carboxyl-terminal processing protease [EC:3.4.21.102] | ec:3.4.21.102 |
| ppg:PputGB1\_5110 | hypothetical protein; K09798 hypothetical protein |  |
| ppg:PputGB1\_5111 | hypothetical protein |  |
| ppg:PputGB1\_5112 | choline/carnitine/betaine transporter; K02168 choline/glycine/proline betaine transport protein |  |
| ppg:PputGB1\_5113 | transcriptional regulator BetI; K02167 TetR/AcrR family transcriptional regulator, transcriptional repressor of bet genes |  |
| ppg:PputGB1\_5114 | betaine aldehyde dehydrogenase (EC:1.2.1.8); K00130 betaine-aldehyde dehydrogenase [EC:1.2.1.8] | ec:1.2.1.8 |
| ppg:PputGB1\_5115 | choline dehydrogenase; K00108 choline dehydrogenase [EC:1.1.99.1] | ec:1.1.99.1 |
| ppg:PputGB1\_5116 | potassium/proton antiporter; K11105 cell volume regulation protein A |  |
| ppg:PputGB1\_5117 | potassium efflux protein KefA; K05802 potassium efflux system protein |  |
| ppg:PputGB1\_5118 | hypothetical protein |  |
| ppg:PputGB1\_5119 | thioredoxin; K03672 thioredoxin 2 [EC:1.8.1.8] | ec:1.8.1.8 |
| ppg:PputGB1\_5120 | cobyrinic acid ac-diamide synthase; K03496 chromosome partitioning protein |  |
| ppg:PputGB1\_5121 | LysR family transcriptional regulator |  |
| ppg:PputGB1\_5122 | major facilitator superfamily transporter; K08224 MFS transporter, YNFM family, putative membrane transport protein |  |
| ppg:PputGB1\_5123 | hypothetical protein |  |

  
**Neighborhood Representations for "pfs:PFLU5684"**  

| ID | Annotation | EC number |
| --- | --- | --- |
| pfs:PFLU5674 | hypothetical protein |  |
| pfs:PFLU5675 | 3-hydroxybutyryl-CoA dehydrogenase; K17735 carnitine 3-dehydrogenase [EC:1.1.1.108] | ec:1.1.1.108 |
| pfs:PFLU5676 | hypothetical protein; K07107 acyl-CoA thioester hydrolase [EC:3.1.2.-] |  |
| pfs:PFLU5677 | hypothetical protein |  |
| pfs:PFLU5678 | AraC family transcriptional regulator |  |
| pfs:PFLU5679 | sdaA; L-serine dehydratase 1 (EC:4.3.1.17); K01752 L-serine dehydratase [EC:4.3.1.17] | ec:4.3.1.17 |
| pfs:PFLU5680 | putative glycine betaine/L-proline ABC transporter substrate-binding protein; K02002 glycine betaine/proline transport system substrate-binding protein |  |
| pfs:PFLU5681 | putative glycine betaine/L-proline ABC transporter membrane protein; K02001 glycine betaine/proline transport system permease protein |  |
| pfs:PFLU5682 | putative glycine betaine/L-proline ABC transporter ATP-binding protein; K02000 glycine betaine/proline transport system ATP-binding protein [EC:3.6.3.32] | ec:3.6.3.32 |
| pfs:PFLU5683 | betT; high-affinity choline ABC transporter membrane protein; K02168 choline/glycine/proline betaine transport protein |  |
| pfs:PFLU5684 | betI; transcriptional regulator BetI; K02167 TetR/AcrR family transcriptional regulator, transcriptional repressor of bet genes |  |
| pfs:PFLU5685 | betB; betaine aldehyde dehydrogenase (EC:1.2.1.8); K00130 betaine-aldehyde dehydrogenase [EC:1.2.1.8] | ec:1.2.1.8 |
| pfs:PFLU5686 | betA; choline dehydrogenase (EC:1.1.99.1); K00108 choline dehydrogenase [EC:1.1.99.1] | ec:1.1.99.1 |
| pfs:PFLU5687 | hypothetical protein |  |
| pfs:PFLU5688 | hypothetical protein |  |
| pfs:PFLU5689 | rhlA; rhamnosyltransferase 1 subunit A |  |
| pfs:PFLU5690 | putative transporter-like membrane protein |  |
| pfs:PFLU5691 | dbpA; ATP-dependent RNA helicase DbpA; K05591 ATP-independent RNA helicase DbpA [EC:3.6.4.13] | ec:3.6.4.13 |
| pfs:PFLU5692 | hypothetical protein; K07007 |  |
| pfs:PFLU5693 | putative transporter-like membrane protein |  |
| pfs:PFLU5694 | hypothetical protein; K02030 polar amino acid transport system substrate-binding protein |  |

  
**Neighborhood Representations for "pfo:Pfl01\_5242"**  

| ID | Annotation | EC number |
| --- | --- | --- |
| pfo:Pfl01\_5232 | 3-hydroxybutyryl-CoA dehydrogenase (EC:1.1.1.157); K17735 carnitine 3-dehydrogenase [EC:1.1.1.108] | ec:1.1.1.108 |
| pfo:Pfl01\_5233 | hypothetical protein; K07107 acyl-CoA thioester hydrolase [EC:3.1.2.-] |  |
| pfo:Pfl01\_5234 | gamma-butyrobetaine hydroxylase; K00471 gamma-butyrobetaine dioxygenase [EC:1.14.11.1] | ec:1.14.11.1 |
| pfo:Pfl01\_5235 | hypothetical protein |  |
| pfo:Pfl01\_5236 | transcriptional regulator |  |
| pfo:Pfl01\_5237 | L-serine ammonia-lyase (EC:4.3.1.17); K01752 L-serine dehydratase [EC:4.3.1.17] | ec:4.3.1.17 |
| pfo:Pfl01\_5238 | glycine betaine ABC transporter substrate-binding protein; K02002 glycine betaine/proline transport system substrate-binding protein |  |
| pfo:Pfl01\_5239 | binding-protein dependent transport system inner membrane protein; K02001 glycine betaine/proline transport system permease protein |  |
| pfo:Pfl01\_5240 | glycine betaine/L-proline transport ATP-binding subunit; K02000 glycine betaine/proline transport system ATP-binding protein [EC:3.6.3.32] | ec:3.6.3.32 |
| pfo:Pfl01\_5241 | BCCT transporter; K02168 choline/glycine/proline betaine transport protein |  |
| pfo:Pfl01\_5242 | transcriptional regulator BetI; K02167 TetR/AcrR family transcriptional regulator, transcriptional repressor of bet genes |  |
| pfo:Pfl01\_5243 | betaine aldehyde dehydrogenase (EC:1.2.1.8); K00130 betaine-aldehyde dehydrogenase [EC:1.2.1.8] | ec:1.2.1.8 |
| pfo:Pfl01\_5244 | choline dehydrogenase (EC:1.1.99.1); K00108 choline dehydrogenase [EC:1.1.99.1] | ec:1.1.99.1 |
| pfo:Pfl01\_5245 | peptidase U62, modulator of DNA gyrase |  |
| pfo:Pfl01\_5246 | hypothetical protein |  |
| pfo:Pfl01\_5247 | EmrB/QacA family drug resistance transporter |  |
| pfo:Pfl01\_5248 | ATP-dependent RNA helicase DbpA; K05591 ATP-independent RNA helicase DbpA [EC:3.6.4.13] | ec:3.6.4.13 |
| pfo:Pfl01\_5249 | fumarate reductase/succinate dehydrogenase flavoprotein-like protein; K07007 |  |
| pfo:Pfl01\_5250 | intergral membrane protein |  |
| pfo:Pfl01\_5251 | extracellular solute-binding protein; K02030 polar amino acid transport system substrate-binding protein |  |
| pfo:Pfl01\_5252 | GCN5-like N-acetyltransferase; K02348 ElaA protein |  |

  
**Neighborhood Representations for "xbo:XBJ1\_3306"**  

| ID | Annotation | EC number |
| --- | --- | --- |
| xbo:XBJ1\_3296 | proB; gamma-glutamyl kinase (EC:2.7.2.11); K00931 glutamate 5-kinase [EC:2.7.2.11] | ec:2.7.2.11 |
| xbo:XBJ1\_3297 | crl; transcriptional regulator of cryptic genes for curli formation and fibronectin binding; K11926 sigma factor-binding protein Crl |  |
| xbo:XBJ1\_3298 | yafA; hydrolase with alpha/beta-hydrolase domain; K11750 esterase FrsA [EC:3.1.-.-] |  |
| xbo:XBJ1\_3299 | gpt; guanine-hypoxanthine phosphoribosyltransferase (EC:2.4.2.22); K00769 xanthine phosphoribosyltransferase [EC:2.4.2.22] | ec:2.4.2.22 |
| xbo:XBJ1\_3300 | hypothetical protein |  |
| xbo:XBJ1\_3301 | pepD; aminopeptidase D; K01270 dipeptidase D [EC:3.4.13.-] |  |
| xbo:XBJ1\_3302 | dinB; DNA polymerase IV (EC:2.7.7.7); K02346 DNA polymerase IV [EC:2.7.7.7] | ec:2.7.7.7 |
| xbo:XBJ1\_3303 | transporter |  |
| xbo:XBJ1\_3304 | hypothetical protein |  |
| xbo:XBJ1\_3305 | betT; high-affinity choline transporter (BCCT family); K02168 choline/glycine/proline betaine transport protein |  |
| xbo:XBJ1\_3306 | betI; TetR family transcriptional regulator; K02167 TetR/AcrR family transcriptional regulator, transcriptional repressor of bet genes |  |
| xbo:XBJ1\_3307 | betB; NAD+-dependent betaine aldehyde dehydrogenase (EC:1.2.1.8); K00130 betaine-aldehyde dehydrogenase [EC:1.2.1.8] | ec:1.2.1.8 |
| xbo:XBJ1\_3308 | betA; choline dehydrogenase (EC:1.1.99.1); K00108 choline dehydrogenase [EC:1.1.99.1] | ec:1.1.99.1 |
| xbo:XBJ1\_3309 | acetyl transferase (EC:2.3.1.-) |  |
| xbo:XBJ1\_3310 | hypothetical protein |  |
| xbo:XBJ1\_3311 | TonB-dependent heme receptor A; K16087 hemoglobin/transferrin/lactoferrin receptor protein |  |
| xbo:XBJ1\_3312 | KpLE2 phage-like element; sigma 19 factor of RNA polymerase (fragment); K03088 RNA polymerase sigma-70 factor, ECF subfamily |  |
| xbo:XBJ1\_3313 | insertion element iso-IS1n protein insB (fragment) |  |
| xbo:XBJ1\_3314 | insertion element iso-IS1N protein insA |  |
| xbo:XBJ1\_3315 | KpLE2 phage-like element; sigma 19 factor of RNA polymerase (fragment); K03088 RNA polymerase sigma-70 factor, ECF subfamily |  |
| xbo:XBJ1\_3316 | hypothetical protein |  |

  
**Neighborhood Representations for "ddd:Dda3937\_03413"**  

| ID | Annotation | EC number |
| --- | --- | --- |
| ddd:Dda3937\_00025 | hypothetical protein |  |
| ddd:Dda3937\_00024 | hypothetical protein |  |
| ddd:Dda3937\_04629 | hypothetical protein |  |
| ddd:Dda3937\_04630 | hypothetical protein |  |
| ddd:Dda3937\_00023 | ydcX; inner membrane protein |  |
| ddd:Dda3937\_00022 | ycbG; hypothetical protein; K09911 hypothetical protein |  |
| ddd:Dda3937\_03409 | ycbZ; peptidase; K04770 Lon-like ATP-dependent protease [EC:3.4.21.-] |  |
| ddd:Dda3937\_03410 | fabA; beta-hydroxydecanoyl thioester dehydrase; K01716 3-hydroxyacyl-[acyl-carrier-protein] dehydratase [EC:4.2.1.59] | ec:4.2.1.59 |
| ddd:Dda3937\_03411 | betA; choline dehydrogenase; K00108 choline dehydrogenase [EC:1.1.99.1] | ec:1.1.99.1 |
| ddd:Dda3937\_03412 | betB; betaine aldehyde dehydrogenase; K00130 betaine-aldehyde dehydrogenase [EC:1.2.1.8] | ec:1.2.1.8 |
| ddd:Dda3937\_03413 | betI; DNA-binding transcriptional regulator BetI; K02167 TetR/AcrR family transcriptional regulator, transcriptional repressor of bet genes |  |
| ddd:Dda3937\_03414 | spiX; galactose-6-phosphate isomerase subunit LacB |  |
| ddd:Dda3937\_04274 | tRNA-Ser; K14233 tRNA Ser |  |
| ddd:Dda3937\_03415 | methyl-accepting chemotaxis protein; K03406 methyl-accepting chemotaxis protein |  |
| ddd:Dda3937\_03416 | phoH; hypothetical protein; K06217 phosphate starvation-inducible protein PhoH and related proteins |  |
| ddd:Dda3937\_03417 | fliZ; regulator of FliA activity; K02425 FliZ protein |  |
| ddd:Dda3937\_03418 | fliA; RNA polymerase sigma-28 (sigma F) factor; K02405 RNA polymerase sigma factor for flagellar operon FliA |  |
| ddd:Dda3937\_03419 | hypothetical protein |  |
| ddd:Dda3937\_03420 | methyltransferase |  |
| ddd:Dda3937\_03421 | oxidoreductase |  |
| ddd:Dda3937\_03422 | carbamoyl-phosphate synthase small subunit |  |

  
**Neighborhood Representations for "xne:XNC1\_1246"**  

| ID | Annotation | EC number |
| --- | --- | --- |
| xne:XNC1\_1236 | hypothetical protein |  |
| xne:XNC1\_1237 | hypothetical protein |  |
| xne:XNC1\_1238 | hypothetical protein |  |
| xne:XNC1\_1239 | trasnposase |  |
| xne:XNC1\_1240 | hypothetical protein |  |
| xne:XNC1\_1241 | hypothetical protein |  |
| xne:XNC1\_1242 | hypothetical protein |  |
| xne:XNC1\_1243 | hypothetical protein |  |
| xne:XNC1\_1244 | betA; choline dehydrogenase (EC:1.1.99.1); K00108 choline dehydrogenase [EC:1.1.99.1] | ec:1.1.99.1 |
| xne:XNC1\_1245 | betB; NAD+-dependent betaine aldehyde dehydrogenase (EC:1.2.1.8); K00130 betaine-aldehyde dehydrogenase [EC:1.2.1.8] | ec:1.2.1.8 |
| xne:XNC1\_1246 | betI; osmotic stress transcriptional repressor; K02167 TetR/AcrR family transcriptional regulator, transcriptional repressor of bet genes |  |
| xne:XNC1\_1247 | betT; high-affinity choline transport protein; K02168 choline/glycine/proline betaine transport protein |  |
| xne:XNC1\_1248 | hypothetical protein |  |
| xne:XNC1\_1249 | hypothetical protein |  |
| xne:XNC1\_1250 | dinB; DNA polymerase IV (EC:2.7.7.7); K02346 DNA polymerase IV [EC:2.7.7.7] | ec:2.7.7.7 |
| xne:XNC1\_1251 | pepD; aminopeptidase; K01270 dipeptidase D [EC:3.4.13.-] |  |
| xne:XNC1\_1252 | gpt; guanine-hypoxanthine phosphoribosyltransferase (EC:2.4.2.22); K00769 xanthine phosphoribosyltransferase [EC:2.4.2.22] | ec:2.4.2.22 |
| xne:XNC1\_1253 | yafA; hydrolase; K11750 esterase FrsA [EC:3.1.-.-] |  |
| xne:XNC1\_1254 | crl; transcriptional regulator; K11926 sigma factor-binding protein Crl |  |
| xne:XNC1\_1255 | proB; gamma-glutamyl kinase (EC:2.7.2.11); K00931 glutamate 5-kinase [EC:2.7.2.11] | ec:2.7.2.11 |
| xne:XNC1\_1256 | proA; gamma-glutamylphosphate reductase (EC:1.2.1.41); K00147 glutamate-5-semialdehyde dehydrogenase [EC:1.2.1.41] | ec:1.2.1.41 |

  
**Neighborhood Representations for "spe:Spro\_1513"**  

| ID | Annotation | EC number |
| --- | --- | --- |
| spe:Spro\_1503 | hypothetical protein |  |
| spe:Spro\_1504 | hypothetical protein |  |
| spe:Spro\_1505 | putative receptor |  |
| spe:Spro\_1506 | hypothetical protein |  |
| spe:Spro\_1507 | integral membrane sensor signal transduction histidine kinase |  |
| spe:Spro\_1508 | two component transcriptional regulator |  |
| spe:Spro\_1509 | thiosulfate reductase cytochrome subunit B (membrane anchoring protein) |  |
| spe:Spro\_1510 | molybdopterin-binding oxidoreductase; K07147 sulfoxide reductase catalytic subunit YedY [EC:1.8.-.-] |  |
| spe:Spro\_1511 | pentapeptide MXKDX repeat-containing protein |  |
| spe:Spro\_1512 | choline transport protein BetT; K02168 choline/glycine/proline betaine transport protein |  |
| spe:Spro\_1513 | transcriptional regulator BetI; K02167 TetR/AcrR family transcriptional regulator, transcriptional repressor of bet genes |  |
| spe:Spro\_1514 | betaine aldehyde dehydrogenase; K00130 betaine-aldehyde dehydrogenase [EC:1.2.1.8] | ec:1.2.1.8 |
| spe:Spro\_1515 | choline dehydrogenase; K00108 choline dehydrogenase [EC:1.1.99.1] | ec:1.1.99.1 |
| spe:Spro\_1516 | ferredoxin-dependent glutamate synthase |  |
| spe:Spro\_1517 | LuxR family transcriptional regulator |  |
| spe:Spro\_1518 | diguanylate phosphodiesterase |  |
| spe:Spro\_1519 | fimbrial protein; K07345 major type 1 subunit fimbrin (pilin) |  |
| spe:Spro\_1520 | pili assembly chaperone |  |
| spe:Spro\_1521 | fimbrial biogenesis outer membrane usher protein; K07347 outer membrane usher protein |  |
| spe:Spro\_1522 | hypothetical protein |  |
| spe:Spro\_1523 | AraC family transcriptional regulator; K05804 right origin-binding protein |  |

  
**Neighborhood Representations for "smt:Smal\_1834"**  

| ID | Annotation | EC number |
| --- | --- | --- |
| smt:Smal\_1824 | XRE family transcriptional regulator |  |
| smt:Smal\_1825 | aldehyde dehydrogenase; K08324 succinate-semialdehyde dehydrogenase [EC:1.2.1.16 1.2.1.24] | ec:1.2.1.16 ec:1.2.1.24 |
| smt:Smal\_1826 | thiamine pyrophosphate domain-containing TPP-binding protein; K01652 acetolactate synthase I/II/III large subunit [EC:2.2.1.6] | ec:2.2.1.6 |
| smt:Smal\_1827 | lysS; lysyl-tRNA synthetase; K04567 lysyl-tRNA synthetase, class II [EC:6.1.1.6] | ec:6.1.1.6 |
| smt:Smal\_1828 | response regulator receiver modulated metal dependent phosphohydrolase; K13815 two-component system, response regulator RpfG |  |
| smt:Smal\_1829 | multi-sensor hybrid histidine kinase; K10715 two-component system, sensor histidine kinase RpfC [EC:2.7.13.3] | ec:2.7.13.3 |
| smt:Smal\_1830 | enoyl-CoA hydratase; K13816 DSF synthase |  |
| smt:Smal\_1831 | putative long-chain fatty acyl CoA ligase; K01897 long-chain acyl-CoA synthetase [EC:6.2.1.3] | ec:6.2.1.3 |
| smt:Smal\_1832 | choline dehydrogenase; K00108 choline dehydrogenase [EC:1.1.99.1] | ec:1.1.99.1 |
| smt:Smal\_1833 | betaine aldehyde dehydrogenase; K00130 betaine-aldehyde dehydrogenase [EC:1.2.1.8] | ec:1.2.1.8 |
| smt:Smal\_1834 | TetR family transcriptional regulator; K02167 TetR/AcrR family transcriptional regulator, transcriptional repressor of bet genes |  |
| smt:Smal\_1835 | choline/carnitine/betaine transporter; K02168 choline/glycine/proline betaine transport protein |  |
| smt:Smal\_1836 | aconitate hydratase; K01681 aconitate hydratase [EC:4.2.1.3] | ec:4.2.1.3 |
| smt:Smal\_1837 | AbrB family transcriptional regulator |  |
| smt:Smal\_1838 | hypothetical protein; K07062 |  |
| smt:Smal\_1839 | bifunctional aconitate hydratase 2/2-methylisocitrate dehydratase; K01682 aconitate hydratase 2 / 2-methylisocitrate dehydratase [EC:4.2.1.3 4.2.1.99] | ec:4.2.1.3 ec:4.2.1.99 |
| smt:Smal\_1840 | TonB family protein |  |
| smt:Smal\_1841 | TonB family protein |  |
| smt:Smal\_1842 | alpha/beta hydrolase fold domain-containing protein |  |
| smt:Smal\_1843 | chemotaxis-specific methylesterase (EC:3.1.1.61); K03412 two-component system, chemotaxis family, response regulator CheB [EC:3.1.1.61] | ec:3.1.1.61 |
| smt:Smal\_1844 | chemoreceptor glutamine deamidase CheD; K03411 chemotaxis protein CheD [EC:3.5.1.44] | ec:3.5.1.44 |

  
**Neighborhood Representations for "sml:Smlt2239"**  

| ID | Annotation | EC number |
| --- | --- | --- |
| sml:Smlt2227 | prfB; pseudogene |  |
| sml:Smlt2229 | hypothetical protein |  |
| sml:Smlt2230 | oxidoreductase/aldehyde-dehydrogenase; K08324 succinate-semialdehyde dehydrogenase [EC:1.2.1.16 1.2.1.24] | ec:1.2.1.16 ec:1.2.1.24 |
| sml:Smlt2231 | ilvK; acetolactate synthase (EC:2.2.1.6); K01652 acetolactate synthase I/II/III large subunit [EC:2.2.1.6] | ec:2.2.1.6 |
| sml:Smlt2232 | lysS; lysyl-tRNA synthetase (EC:6.1.1.6); K04567 lysyl-tRNA synthetase, class II [EC:6.1.1.6] | ec:6.1.1.6 |
| sml:Smlt2233 | rpfG; two-component response regulator transcriptional regulator, regulator of pathogenicity factors; K13815 two-component system, response regulator RpfG |  |
| sml:Smlt2234 | rpfC; histidine kinase/response regulator fusion protein, regulation of pathogenicity factors; K10715 two-component system, sensor histidine kinase RpfC [EC:2.7.13.3] | ec:2.7.13.3 |
| sml:Smlt2235 | rpfF; enoyl-CoA hydratase; K13816 DSF synthase |  |
| sml:Smlt2237 | betA; choline dehydrogenase (EC:1.1.99.1); K00108 choline dehydrogenase [EC:1.1.99.1] | ec:1.1.99.1 |
| sml:Smlt2238 | betB; betaine aldehyde dehydrogenase (EC:1.2.1.8); K00130 betaine-aldehyde dehydrogenase [EC:1.2.1.8] | ec:1.2.1.8 |
| sml:Smlt2239 | betI; TetR family regulatory protein; K02167 TetR/AcrR family transcriptional regulator, transcriptional repressor of bet genes |  |
| sml:Smlt2240 | high-affinity choline transport protein; K02168 choline/glycine/proline betaine transport protein |  |
| sml:Smlt2241 | acn; aconitate hydratase (EC:4.2.1.3); K01681 aconitate hydratase [EC:4.2.1.3] | ec:4.2.1.3 |
| sml:Smlt2242 | smpA; small protein A-like protein |  |
| sml:Smlt2243 | SpoVT/AbrB domain transcriptional regulator |  |
| sml:Smlt2244 | hypothetical protein; K07062 |  |
| sml:Smlt2245 | acnB; bifunctional aconitate hydratase 2/2-methylisocitrate dehydratase (EC:4.2.1.3); K01682 aconitate hydratase 2 / 2-methylisocitrate dehydratase [EC:4.2.1.3 4.2.1.99] | ec:4.2.1.3 ec:4.2.1.99 |
| sml:Smlt2246 | hypothetical protein |  |
| sml:Smlt2247 | hypothetical protein |  |
| sml:Smlt2248 | cheB; chemotaxis-specific methylesterase (EC:3.1.1.61); K03412 two-component system, chemotaxis family, response regulator CheB [EC:3.1.1.61] | ec:3.1.1.61 |
| sml:Smlt2249 | cheD; chemoreceptor glutamine deamidase CheD; K03411 chemotaxis protein CheD [EC:3.5.1.44] | ec:3.5.1.44 |

  
**Neighborhood Representations for "ypa:YPA\_1075"**  

| ID | Annotation | EC number |
| --- | --- | --- |
| ypa:YPA\_1065 | hypothetical protein |  |
| ypa:YPA\_1066 | moaA; molybdenum cofactor biosynthesis protein A; K03639 molybdenum cofactor biosynthesis protein |  |
| ypa:YPA\_1067 | moaC; molybdenum cofactor biosynthesis protein MoaC; K03637 molybdenum cofactor biosynthesis protein C |  |
| ypa:YPA\_1068 | moaD; molybdopterin synthase small subunit; K03636 molybdopterin synthase sulfur carrier subunit |  |
| ypa:YPA\_1069 | moaE; molybdopterin guanine dinucleotide biosynthesis protein MoaE; K03635 molybdopterin synthase catalytic subunit [EC:2.-.-.-] |  |
| ypa:YPA\_1070 | hypothetical protein; K06890 |  |
| ypa:YPA\_1071 | hypothetical protein; K06890 |  |
| ypa:YPA\_1072 | transposase for the IS1541 insertion element; K07491 putative transposase |  |
| ypa:YPA\_1073 | choline dehydrogenase (EC:1.1.99.1); K00108 choline dehydrogenase [EC:1.1.99.1] | ec:1.1.99.1 |
| ypa:YPA\_1074 | betaine aldehyde dehydrogenase (EC:1.2.1.8); K00130 betaine-aldehyde dehydrogenase [EC:1.2.1.8] | ec:1.2.1.8 |
| ypa:YPA\_1075 | transcriptional regulator BetI; K02167 TetR/AcrR family transcriptional regulator, transcriptional repressor of bet genes |  |
| ypa:YPA\_1076 | choline transport protein BetT; K02168 choline/glycine/proline betaine transport protein |  |
| ypa:YPA\_1077 | DNA-binding transcriptional activator XapR |  |
| ypa:YPA\_1078 | hypothetical protein |  |
| ypa:YPA\_1079 | purine nucleoside phosphorylase (EC:2.4.2.1); K03815 xanthosine phosphorylase [EC:2.4.2.-] |  |
| ypa:YPA\_1080 | hypothetical protein |  |
| ypa:YPA\_1081 | transposase for the IS1541 insertion element |  |
| ypa:YPA\_1082 | major facilitator superfamily xanthosine permease; K11537 MFS transporter, NHS family, xanthosine permease |  |
| ypa:YPA\_1083 | zraP; zinc resistance protein; K07803 zinc resistance-associated protein |  |
| ypa:YPA\_1084 | hypothetical protein; K07709 two-component system, NtrC family, sensor histidine kinase HydH [EC:2.7.13.3] | ec:2.7.13.3 |
| ypa:YPA\_1085 | helix-turn-helix, Fis-type; K07713 two-component system, NtrC family, response regulator HydG |  |

  
**Neighborhood Representations for "ypb:YPTS\_1277"**  

| ID | Annotation | EC number |
| --- | --- | --- |
| ypb:YPTS\_1267 | hypothetical protein |  |
| ypb:YPTS\_1268 | hypothetical protein |  |
| ypb:YPTS\_1269 | hypothetical protein |  |
| ypb:YPTS\_1270 | moaA; molybdenum cofactor biosynthesis protein A; K03639 molybdenum cofactor biosynthesis protein |  |
| ypb:YPTS\_1271 | moaC; molybdenum cofactor biosynthesis protein MoaC; K03637 molybdenum cofactor biosynthesis protein C |  |
| ypb:YPTS\_1272 | moaD; molybdopterin synthase small subunit; K03636 molybdopterin synthase sulfur carrier subunit |  |
| ypb:YPTS\_1273 | moaE; molybdopterin guanine dinucleotide biosynthesis protein MoaE; K03635 molybdopterin synthase catalytic subunit [EC:2.-.-.-] |  |
| ypb:YPTS\_1274 | hypothetical protein; K06890 |  |
| ypb:YPTS\_1275 | choline dehydrogenase; K00108 choline dehydrogenase [EC:1.1.99.1] | ec:1.1.99.1 |
| ypb:YPTS\_1276 | betaine aldehyde dehydrogenase; K00130 betaine-aldehyde dehydrogenase [EC:1.2.1.8] | ec:1.2.1.8 |
| ypb:YPTS\_1277 | transcriptional regulator BetI; K02167 TetR/AcrR family transcriptional regulator, transcriptional repressor of bet genes |  |
| ypb:YPTS\_1278 | choline transport protein BetT; K02168 choline/glycine/proline betaine transport protein |  |
| ypb:YPTS\_1279 | DNA-binding transcriptional activator XapR |  |
| ypb:YPTS\_1280 | hypothetical protein |  |
| ypb:YPTS\_1281 | purine nucleoside phosphorylase; K03815 xanthosine phosphorylase [EC:2.4.2.-] |  |
| ypb:YPTS\_1282 | nucleoside transporter; K11537 MFS transporter, NHS family, xanthosine permease |  |
| ypb:YPTS\_1283 | zraP; zinc resistance protein; K07803 zinc resistance-associated protein |  |
| ypb:YPTS\_1284 | signal transduction histidine kinase, nitrogen specific, NtrB; K07709 two-component system, NtrC family, sensor histidine kinase HydH [EC:2.7.13.3] | ec:2.7.13.3 |
| ypb:YPTS\_1285 | two component, sigma54 specific, Fis family transcriptional regulator; K07713 two-component system, NtrC family, response regulator HydG |  |
| ypb:YPTS\_1286 | NADH:flavin oxidoreductase |  |
| ypb:YPTS\_1287 | LysR family transcriptional regulator |  |

  
**Neighborhood Representations for "ype:YPO1167"**  

| ID | Annotation | EC number |
| --- | --- | --- |
| ype:YPO1157 | hypothetical protein |  |
| ype:YPO1158 | hypothetical protein |  |
| ype:YPO1159 | moaA; molybdenum cofactor biosynthesis protein A; K03639 molybdenum cofactor biosynthesis protein |  |
| ype:YPO1160 | moaC; molybdenum cofactor biosynthesis protein MoaC; K03637 molybdenum cofactor biosynthesis protein C |  |
| ype:YPO1161 | moaD; molybdopterin synthase small subunit; K03636 molybdopterin synthase sulfur carrier subunit |  |
| ype:YPO1162 | moaE; molybdopterin guanine dinucleotide biosynthesis protein MoaE; K03635 molybdopterin synthase catalytic subunit [EC:2.-.-.-] |  |
| ype:YPO1163 | hypothetical protein; K06890 |  |
| ype:YPO1164 | tnp; transposase for the IS1541 insertion element; K07491 putative transposase |  |
| ype:YPO1165 | betA; choline dehydrogenase (EC:1.1.99.1); K00108 choline dehydrogenase [EC:1.1.99.1] | ec:1.1.99.1 |
| ype:YPO1166 | betB; betaine aldehyde dehydrogenase (EC:1.2.1.8); K00130 betaine-aldehyde dehydrogenase [EC:1.2.1.8] | ec:1.2.1.8 |
| ype:YPO1167 | betI; transcriptional regulator BetI; K02167 TetR/AcrR family transcriptional regulator, transcriptional repressor of bet genes |  |
| ype:YPO1168 | betT; choline transport protein BetT; K02168 choline/glycine/proline betaine transport protein |  |
| ype:YPO1169 | DNA-binding transcriptional activator XapR |  |
| ype:YPO1170 | hypothetical protein |  |
| ype:YPO1171 | xapA; purine nucleoside phosphorylase (EC:2.4.2.1); K03815 xanthosine phosphorylase [EC:2.4.2.-] |  |
| ype:YPO1172 | hypothetical protein |  |
| ype:YPO1173 | tnp; transposase for the IS1541 insertion element; K07491 putative transposase |  |
| ype:YPO1174 | hypothetical protein |  |
| ype:YPO1175 | tRNA-dihydrouridine synthase C; K05541 tRNA-dihydrouridine synthase C [EC:1.-.-.-] |  |
| ype:YPO1176 | pbpG; D-alanyl-D-alanine endopeptidase (EC:3.4.-.-); K07262 D-alanyl-D-alanine endopeptidase (penicillin-binding protein 7) [EC:3.4.21.-] |  |
| ype:YPO1177 | dld; D-lactate dehydrogenase (EC:1.1.1.28); K03777 D-lactate dehydrogenase [EC:1.1.1.28] | ec:1.1.1.28 |

  
**Neighborhood Representations for "ypi:YpsIP31758\_2829"**  

| ID | Annotation | EC number |
| --- | --- | --- |
| ypi:YpsIP31758\_2819 | FAD/FMN-binding oxidoreductase |  |
| ypi:YpsIP31758\_2820 | zraR; ZraR family transcriptional regulator; K07713 two-component system, NtrC family, response regulator HydG |  |
| ypi:YpsIP31758\_2821 | sensory box histidine kinase; K07709 two-component system, NtrC family, sensor histidine kinase HydH [EC:2.7.13.3] | ec:2.7.13.3 |
| ypi:YpsIP31758\_2822 | zraP; zinc resistance protein; K07803 zinc resistance-associated protein |  |
| ypi:YpsIP31758\_2823 | xapB; xanthosine transporter XapB; K11537 MFS transporter, NHS family, xanthosine permease |  |
| ypi:YpsIP31758\_2824 | xapA; purine nucleoside phosphorylase (EC:2.4.2.1); K03815 xanthosine phosphorylase [EC:2.4.2.-] |  |
| ypi:YpsIP31758\_2825 | integral membrane protein |  |
| ypi:YpsIP31758\_2826 | xapR; DNA-binding transcriptional activator XapR |  |
| ypi:YpsIP31758\_2827 | hypothetical protein |  |
| ypi:YpsIP31758\_2828 | betT; choline transport protein BetT; K02168 choline/glycine/proline betaine transport protein |  |
| ypi:YpsIP31758\_2829 | betI; BetI family transcriptional regulator; K02167 TetR/AcrR family transcriptional regulator, transcriptional repressor of bet genes |  |
| ypi:YpsIP31758\_2830 | betB; betaine aldehyde dehydrogenase (EC:1.2.1.8); K00130 betaine-aldehyde dehydrogenase [EC:1.2.1.8] | ec:1.2.1.8 |
| ypi:YpsIP31758\_2831 | betA; choline dehydrogenase (EC:1.1.99.1); K00108 choline dehydrogenase [EC:1.1.99.1] | ec:1.1.99.1 |
| ypi:YpsIP31758\_2832 | hypothetical protein; K06890 |  |
| ypi:YpsIP31758\_2833 | moaE; molybdopterin guanine dinucleotide biosynthesis protein MoaE; K03635 molybdopterin synthase catalytic subunit [EC:2.-.-.-] |  |
| ypi:YpsIP31758\_2834 | moaD; molybdopterin synthase small subunit; K03636 molybdopterin synthase sulfur carrier subunit |  |
| ypi:YpsIP31758\_2835 | moaC; molybdenum cofactor biosynthesis protein MoaC; K03637 molybdenum cofactor biosynthesis protein C |  |
| ypi:YpsIP31758\_2836 | moaA; molybdenum cofactor biosynthesis protein A; K03639 molybdenum cofactor biosynthesis protein |  |
| ypi:YpsIP31758\_2837 | hypothetical protein |  |
| ypi:YpsIP31758\_2838 | hypothetical protein |  |
| ypi:YpsIP31758\_2839 | uvrB; excinuclease ABC subunit B; K03702 excinuclease ABC subunit B |  |

  
**Neighborhood Representations for "ypn:YPN\_2834"**  

| ID | Annotation | EC number |
| --- | --- | --- |
| ypn:YPN\_2824 | helix-turn-helix, Fis-type; K07713 two-component system, NtrC family, response regulator HydG |  |
| ypn:YPN\_2825 | hypothetical protein; K07709 two-component system, NtrC family, sensor histidine kinase HydH [EC:2.7.13.3] | ec:2.7.13.3 |
| ypn:YPN\_2826 | zraP; zinc resistance protein; K07803 zinc resistance-associated protein |  |
| ypn:YPN\_2827 | major facilitator superfamily xanthosine permease; K11537 MFS transporter, NHS family, xanthosine permease |  |
| ypn:YPN\_2828 | transposase for the IS1541 insertion element |  |
| ypn:YPN\_2829 | hypothetical protein |  |
| ypn:YPN\_2830 | purine nucleoside phosphorylase (EC:2.4.2.1); K03815 xanthosine phosphorylase [EC:2.4.2.-] |  |
| ypn:YPN\_2831 | membrane protein |  |
| ypn:YPN\_2832 | DNA-binding transcriptional activator XapR |  |
| ypn:YPN\_2833 | choline transport protein BetT; K02168 choline/glycine/proline betaine transport protein |  |
| ypn:YPN\_2834 | transcriptional regulator BetI; K02167 TetR/AcrR family transcriptional regulator, transcriptional repressor of bet genes |  |
| ypn:YPN\_2835 | betaine aldehyde dehydrogenase (EC:1.2.1.8); K00130 betaine-aldehyde dehydrogenase [EC:1.2.1.8] | ec:1.2.1.8 |
| ypn:YPN\_2836 | choline dehydrogenase (EC:1.1.99.1); K00108 choline dehydrogenase [EC:1.1.99.1] | ec:1.1.99.1 |
| ypn:YPN\_2837 | transposase for the IS1541 insertion element; K07491 putative transposase |  |
| ypn:YPN\_2838 | membrane protein; K06890 |  |
| ypn:YPN\_2839 | moaE; molybdopterin guanine dinucleotide biosynthesis protein MoaE; K03635 molybdopterin synthase catalytic subunit [EC:2.-.-.-] |  |
| ypn:YPN\_2840 | moaD; molybdopterin synthase small subunit; K03636 molybdopterin synthase sulfur carrier subunit |  |
| ypn:YPN\_2841 | moaC; molybdenum cofactor biosynthesis protein MoaC; K03637 molybdenum cofactor biosynthesis protein C |  |
| ypn:YPN\_2842 | moaA; molybdenum cofactor biosynthesis protein A; K03639 molybdenum cofactor biosynthesis protein |  |
| ypn:YPN\_2843 | hypothetical protein |  |
| ypn:YPN\_2844 | hypothetical protein |  |

  
**Neighborhood Representations for "ypp:YPDSF\_2530"**  

| ID | Annotation | EC number |
| --- | --- | --- |
| ypp:YPDSF\_2520 | pbpG; D-alanyl-D-alanine endopeptidase; K07262 D-alanyl-D-alanine endopeptidase (penicillin-binding protein 7) [EC:3.4.21.-] |  |
| ypp:YPDSF\_2521 | hypothetical protein |  |
| ypp:YPDSF\_2522 | tRNA-dihydrouridine synthase C; K05541 tRNA-dihydrouridine synthase C [EC:1.-.-.-] |  |
| ypp:YPDSF\_2523 | hypothetical protein |  |
| ypp:YPDSF\_2524 | transposase for the IS1541 insertion element; K07491 putative transposase |  |
| ypp:YPDSF\_2525 | hypothetical protein |  |
| ypp:YPDSF\_2526 | purine nucleoside phosphorylase (EC:2.4.2.1); K03815 xanthosine phosphorylase [EC:2.4.2.-] |  |
| ypp:YPDSF\_2527 | membrane protein |  |
| ypp:YPDSF\_2528 | DNA-binding transcriptional activator XapR |  |
| ypp:YPDSF\_2529 | choline transport protein BetT; K02168 choline/glycine/proline betaine transport protein |  |
| ypp:YPDSF\_2530 | transcriptional regulator BetI; K02167 TetR/AcrR family transcriptional regulator, transcriptional repressor of bet genes |  |
| ypp:YPDSF\_2531 | betaine aldehyde dehydrogenase (EC:1.2.1.8); K00130 betaine-aldehyde dehydrogenase [EC:1.2.1.8] | ec:1.2.1.8 |
| ypp:YPDSF\_2532 | choline dehydrogenase (EC:1.1.99.1); K00108 choline dehydrogenase [EC:1.1.99.1] | ec:1.1.99.1 |
| ypp:YPDSF\_2533 | transposase for the IS1541 insertion element |  |
| ypp:YPDSF\_2534 | membrane protein; K06890 |  |
| ypp:YPDSF\_2535 | moaE; molybdopterin guanine dinucleotide biosynthesis protein MoaE; K03635 molybdopterin synthase catalytic subunit [EC:2.-.-.-] |  |
| ypp:YPDSF\_2536 | moaD; molybdopterin synthase small subunit; K03636 molybdopterin synthase sulfur carrier subunit |  |
| ypp:YPDSF\_2537 | moaC; molybdenum cofactor biosynthesis protein MoaC; K03637 molybdenum cofactor biosynthesis protein C |  |
| ypp:YPDSF\_2538 | moaA; molybdenum cofactor biosynthesis protein A; K03639 molybdenum cofactor biosynthesis protein |  |
| ypp:YPDSF\_2539 | hypothetical protein |  |
| ypp:YPDSF\_2540 | hypothetical protein |  |

  
**Neighborhood Representations for "yps:YPTB1197"**  

| ID | Annotation | EC number |
| --- | --- | --- |
| yps:YPTB1187 | uvrB; excinuclease ABC subunit B; K03702 excinuclease ABC subunit B |  |
| yps:YPTB1188 | hypothetical protein |  |
| yps:YPTB1189 | hypothetical protein |  |
| yps:YPTB1190 | moaA; molybdenum cofactor biosynthesis protein A; K03639 molybdenum cofactor biosynthesis protein |  |
| yps:YPTB1191 | moaC; molybdenum cofactor biosynthesis protein MoaC; K03637 molybdenum cofactor biosynthesis protein C |  |
| yps:YPTB1192 | moaD; molybdopterin synthase small subunit; K03636 molybdopterin synthase sulfur carrier subunit |  |
| yps:YPTB1193 | moaE; molybdopterin guanine dinucleotide biosynthesis protein MoaE; K03635 molybdopterin synthase catalytic subunit [EC:2.-.-.-] |  |
| yps:YPTB1194 | stationary phase anti-death family (SAD), acetate uptake; K06890 |  |
| yps:YPTB1195 | betA; choline dehydrogenase (EC:1.1.99.1); K00108 choline dehydrogenase [EC:1.1.99.1] | ec:1.1.99.1 |
| yps:YPTB1196 | betB; betaine aldehyde dehydrogenase (EC:1.2.1.8); K00130 betaine-aldehyde dehydrogenase [EC:1.2.1.8] | ec:1.2.1.8 |
| yps:YPTB1197 | betI; transcriptional regulator BetI; K02167 TetR/AcrR family transcriptional regulator, transcriptional repressor of bet genes |  |
| yps:YPTB1198 | betT; choline transport protein BetT; K02168 choline/glycine/proline betaine transport protein |  |
| yps:YPTB1199 | DNA-binding transcriptional activator XapR |  |
| yps:YPTB1200 | DMT superfamily drug/metabolite efflux pump |  |
| yps:YPTB1201 | xapA; purine nucleoside phosphorylase (EC:2.4.2.1); K03815 xanthosine phosphorylase [EC:2.4.2.-] |  |
| yps:YPTB1202 | xapB; major facilitator superfamily xanthosine permease; K11537 MFS transporter, NHS family, xanthosine permease |  |
| yps:YPTB1203 | zraP; zinc resistance protein; K07803 zinc resistance-associated protein |  |
| yps:YPTB1204 | two component histidine kinase-sensor (EC:2.7.3.-); K07709 two-component system, NtrC family, sensor histidine kinase HydH [EC:2.7.13.3] | ec:2.7.13.3 |
| yps:YPTB1205 | hydG; transcriptional regulator hydG; K07713 two-component system, NtrC family, response regulator HydG |  |
| yps:YPTB1206 | morB; morphinone reductase |  |
| yps:YPTB1207 | LysR family transcriptional regulator |  |

  
**Neighborhood Representations for "ypz:YPZ3\_1057"**  

| ID | Annotation | EC number |
| --- | --- | --- |
| ypz:YPZ3\_1047 | putative amino acid transporter |  |
| ypz:YPZ3\_1048 | uvrB; excinuclease ABC subunit B; K03702 excinuclease ABC subunit B |  |
| ypz:YPZ3\_1049 | hypothetical protein |  |
| ypz:YPZ3\_1050 | moaA; molybdenum cofactor biosynthesis protein A; K03639 molybdenum cofactor biosynthesis protein |  |
| ypz:YPZ3\_1051 | moaC; molybdenum cofactor biosynthesis protein C; K03637 molybdenum cofactor biosynthesis protein C |  |
| ypz:YPZ3\_1052 | molybdopterin (mpt) converting factor subunit 2; K03635 molybdopterin synthase catalytic subunit [EC:2.-.-.-] |  |
| ypz:YPZ3\_1053 | hypothetical protein; K06890 |  |
| ypz:YPZ3\_1054 | transposase; K07491 putative transposase |  |
| ypz:YPZ3\_1055 | betA; choline dehydrogenase; K00108 choline dehydrogenase [EC:1.1.99.1] | ec:1.1.99.1 |
| ypz:YPZ3\_1056 | betB; betaine aldehyde dehydrogenase; K00130 betaine-aldehyde dehydrogenase [EC:1.2.1.8] | ec:1.2.1.8 |
| ypz:YPZ3\_1057 | betI; transcriptional regulator BetI; K02167 TetR/AcrR family transcriptional regulator, transcriptional repressor of bet genes |  |
| ypz:YPZ3\_1058 | high-affinity choline transport protein; K02168 choline/glycine/proline betaine transport protein |  |
| ypz:YPZ3\_1059 | LysR-family regulatory protein |  |
| ypz:YPZ3\_1060 | hypothetical protein |  |
| ypz:YPZ3\_1061 | xapA; purine nucleoside phosphorylase; K03815 xanthosine phosphorylase [EC:2.4.2.-] |  |
| ypz:YPZ3\_1062 | hypothetical protein |  |
| ypz:YPZ3\_1063 | tnp; transposase for the IS1541 insertion element |  |
| ypz:YPZ3\_1064 | MFS family xanthosine permease; K11537 MFS transporter, NHS family, xanthosine permease |  |
| ypz:YPZ3\_1065 | hypothetical protein; K07803 zinc resistance-associated protein |  |
| ypz:YPZ3\_1066 | Pas; K07709 two-component system, NtrC family, sensor histidine kinase HydH [EC:2.7.13.3] | ec:2.7.13.3 |
| ypz:YPZ3\_1067 | hydG; transcriptional regulator hydG; K07713 two-component system, NtrC family, response regulator HydG |  |

  
**Neighborhood Representations for "ypm:YP\_0992"**  

| ID | Annotation | EC number |
| --- | --- | --- |
| ypm:YP\_0982 | nemA1; NADH:flavin oxidoreductase |  |
| ypm:YP\_0983 | atoC1; response regulator; K07713 two-component system, NtrC family, response regulator HydG |  |
| ypm:YP\_0984 | baeS2; Signal transduction histidine kinase; K07709 two-component system, NtrC family, sensor histidine kinase HydH [EC:2.7.13.3] | ec:2.7.13.3 |
| ypm:YP\_0985 | zraP; zinc resistance protein; K07803 zinc resistance-associated protein |  |
| ypm:YP\_0986 | xapB; pseudogene |  |
| ypm:YP\_0987 | tnp\_11; transposase for the IS1541 insertion element |  |
| ypm:YP\_0988 | xapA; purine nucleoside phosphorylase (EC:2.4.2.1); K03815 xanthosine phosphorylase [EC:2.4.2.-] |  |
| ypm:YP\_0989 | rhaT5; hypothetical protein |  |
| ypm:YP\_0990 | lysR5; DNA-binding transcriptional activator XapR |  |
| ypm:YP\_0991 | betT1; choline transport protein BetT; K02168 choline/glycine/proline betaine transport protein |  |
| ypm:YP\_0992 | betI; transcriptional regulator BetI; K02167 TetR/AcrR family transcriptional regulator, transcriptional repressor of bet genes |  |
| ypm:YP\_0993 | betB; betaine aldehyde dehydrogenase (EC:1.2.1.8); K00130 betaine-aldehyde dehydrogenase [EC:1.2.1.8] | ec:1.2.1.8 |
| ypm:YP\_0994 | betA; choline dehydrogenase (EC:1.1.99.1); K00108 choline dehydrogenase [EC:1.1.99.1] | ec:1.1.99.1 |
| ypm:YP\_0995 | tnp\_12; transposase for the IS1541 insertion element |  |
| ypm:YP\_0996 | hypothetical protein; K06890 |  |
| ypm:YP\_0997 | moaE; molybdopterin guanine dinucleotide biosynthesis protein MoaE; K03635 molybdopterin synthase catalytic subunit [EC:2.-.-.-] |  |
| ypm:YP\_0998 | moaD; molybdopterin synthase small subunit; K03636 molybdopterin synthase sulfur carrier subunit |  |
| ypm:YP\_0999 | moaC; molybdenum cofactor biosynthesis protein MoaC; K03637 molybdenum cofactor biosynthesis protein C |  |
| ypm:YP\_1000 | moaA; molybdenum cofactor biosynthesis protein A; K03639 molybdenum cofactor biosynthesis protein |  |
| ypm:YP\_1001 | hypothetical protein |  |
| ypm:YP\_1002 | hypothetical protein |  |

  
**Neighborhood Representations for "csa:Csal\_1516"**  

| ID | Annotation | EC number |
| --- | --- | --- |
| csa:Csal\_1506 | glycerophosphoryl diester phosphodiesterase; K01126 glycerophosphoryl diester phosphodiesterase [EC:3.1.4.46] | ec:3.1.4.46 |
| csa:Csal\_1507 | hypothetical protein |  |
| csa:Csal\_1508 | zinc-binding alcohol dehydrogenase; K00344 NADPH2:quinone reductase [EC:1.6.5.5] | ec:1.6.5.5 |
| csa:Csal\_1509 | rare lipoprotein A; K03642 rare lipoprotein A |  |
| csa:Csal\_1510 | fadE; acyl-CoA dehydrogenase; K06445 acyl-CoA dehydrogenase [EC:1.3.99.-] |  |
| csa:Csal\_1511 | acriflavin resistance protein; K07789 RND superfamily, multidrug transport protein MdtC |  |
| csa:Csal\_1512 | acriflavin resistance protein |  |
| csa:Csal\_1513 | secretion protein HlyD |  |
| csa:Csal\_1514 | choline dehydrogenase; K00108 choline dehydrogenase [EC:1.1.99.1] | ec:1.1.99.1 |
| csa:Csal\_1515 | betaine aldehyde dehydrogenase; K00130 betaine-aldehyde dehydrogenase [EC:1.2.1.8] | ec:1.2.1.8 |
| csa:Csal\_1516 | transcriptional regulator BetI; K02167 TetR/AcrR family transcriptional regulator, transcriptional repressor of bet genes |  |
| csa:Csal\_1517 | glycine betaine ABC transporter substrate-binding protein; K02002 glycine betaine/proline transport system substrate-binding protein |  |
| csa:Csal\_1518 | ligB; NAD-dependent DNA ligase LigB; K01972 DNA ligase (NAD+) [EC:6.5.1.2] | ec:6.5.1.2 |
| csa:Csal\_1519 | C-terminal processing peptidase-1; K03797 carboxyl-terminal processing protease [EC:3.4.21.102] | ec:3.4.21.102 |
| csa:Csal\_1520 | putative aminopeptidase 2; K01267 aspartyl aminopeptidase [EC:3.4.11.21] | ec:3.4.11.21 |
| csa:Csal\_1521 | ychF; GTP-dependent nucleic acid-binding protein EngD; K06942 |  |
| csa:Csal\_1522 | peptidyl-tRNA hydrolase; K01056 peptidyl-tRNA hydrolase, PTH1 family [EC:3.1.1.29] | ec:3.1.1.29 |
| csa:Csal\_1523 | 50S ribosomal protein L25; K02897 large subunit ribosomal protein L25 |  |
| csa:Csal\_1524 | ribose-phosphate pyrophosphokinase; K00948 ribose-phosphate pyrophosphokinase [EC:2.7.6.1] | ec:2.7.6.1 |
| csa:Csal\_1525 | 4-diphosphocytidyl-2-C-methyl-D-erythritol kinase; K00919 4-diphosphocytidyl-2-C-methyl-D-erythritol kinase [EC:2.7.1.148] | ec:2.7.1.148 |
| csa:Csal\_1526 | lolB; outer membrane lipoprotein LolB; K02494 outer membrane lipoprotein LolB |  |

  
**Neighborhood Representations for "pmr:PMI1461"**  

| ID | Annotation | EC number |
| --- | --- | --- |
| pmr:PMI1451 | uspG2; universal stress protein G; K11932 universal stress protein G |  |
| pmr:PMI1452 | hypothetical protein |  |
| pmr:PMI1453 | hypothetical protein |  |
| pmr:PMI1454 | hypothetical protein |  |
| pmr:PMI1455 | hemB; delta-aminolevulinic acid dehydratase (EC:4.2.1.24); K01698 porphobilinogen synthase [EC:4.2.1.24] | ec:4.2.1.24 |
| pmr:PMI1456 | hypothetical protein |  |
| pmr:PMI1457 | codA; cytosine deaminase (EC:3.5.4.1); K01485 cytosine deaminase [EC:3.5.4.1] | ec:3.5.4.1 |
| pmr:PMI1458 | codB; cytosine permease; K10974 cytosine permease |  |
| pmr:PMI1459 | betA; choline dehydrogenase (EC:1.1.99.1); K00108 choline dehydrogenase [EC:1.1.99.1] | ec:1.1.99.1 |
| pmr:PMI1460 | betB; betaine aldehyde dehydrogenase (EC:1.2.1.8); K00130 betaine-aldehyde dehydrogenase [EC:1.2.1.8] | ec:1.2.1.8 |
| pmr:PMI1461 | betI; TetR family transcriptional regulator; K02167 TetR/AcrR family transcriptional regulator, transcriptional repressor of bet genes |  |
| pmr:PMI1462 | betT; choline transport protein BetT; K02168 choline/glycine/proline betaine transport protein |  |
| pmr:PMI1463 | aminotransferase; K00817 histidinol-phosphate aminotransferase [EC:2.6.1.9] | ec:2.6.1.9 |
| pmr:PMI1464 | fimbrial subunit |  |
| pmr:PMI1465 | fimbrial adhesin; K07350 minor fimbrial subunit |  |
| pmr:PMI1466 | fimbrial outer membrane usher protein; K07347 outer membrane usher protein |  |
| pmr:PMI1467 | fimbrial chaperone protein; K07346 fimbrial chaperone protein |  |
| pmr:PMI1468 | fimbrial subunit; K07351 fimbrial protein |  |
| pmr:PMI1469 | fimbrial subunit; K07345 major type 1 subunit fimbrin (pilin) |  |
| pmr:PMI1470 | fimbrial operon regulator |  |
| pmr:PMI1471 | oppF; oligopeptide ABC transporter ATP-binding protein; K10823 oligopeptide transport system ATP-binding protein |  |

  
**Neighborhood Representations for "ebi:EbC\_19400"**  

| ID | Annotation | EC number |
| --- | --- | --- |
| ebi:EbC\_19300 | anmK; anhydro-N-acetylmuramic acid kinase; K09001 anhydro-N-acetylmuramic acid kinase [EC:2.7.1.170] | ec:2.7.1.170 |
| ebi:EbC\_19310 | lipoprotein |  |
| ebi:EbC\_19320 | pdxH; Pyridoxamine 5'-phosphate oxidase; K00275 pyridoxamine 5'-phosphate oxidase [EC:1.4.3.5] | ec:1.4.3.5 |
| ebi:EbC\_19330 | tyrS; tyrosyl-tRNA synthetase; K01866 tyrosyl-tRNA synthetase [EC:6.1.1.1] | ec:6.1.1.1 |
| ebi:EbC\_19340 | pdxY; Pyridoxal kinase; K00868 pyridoxine kinase [EC:2.7.1.35] | ec:2.7.1.35 |
| ebi:EbC\_19350 | gst; glutathione S-transferase; K00799 glutathione S-transferase [EC:2.5.1.18] | ec:2.5.1.18 |
| ebi:EbC\_19360 | LysR family transcripitonal regulator |  |
| ebi:EbC\_19370 | glutathione S-transferase; K00799 glutathione S-transferase [EC:2.5.1.18] | ec:2.5.1.18 |
| ebi:EbC\_19380 | Pyridoxamine 5'-phosphate oxidase-related FMN-binding protein; K07006 |  |
| ebi:EbC\_19390 | tppB; Tripeptide permease; K03305 proton-dependent oligopeptide transporter, POT family |  |
| ebi:EbC\_19400 | betI; HTH-type transcriptional regulator; K02167 TetR/AcrR family transcriptional regulator, transcriptional repressor of bet genes |  |
| ebi:EbC\_19410 | betB; betaine aldehyde dehydrogenase; K00130 betaine-aldehyde dehydrogenase [EC:1.2.1.8] | ec:1.2.1.8 |
| ebi:EbC\_19420 | betA; choline dehydrogenase; K00108 choline dehydrogenase [EC:1.1.99.1] | ec:1.1.99.1 |
| ebi:EbC\_19430 | major facilitator superfamily transporter |  |
| ebi:EbC\_19440 | endonuclease III; K10773 endonuclease III [EC:4.2.99.18] | ec:4.2.99.18 |
| ebi:EbC\_19450 | rnfE; Electron transport complex protein; K03613 electron transport complex protein RnfE |  |
| ebi:EbC\_19460 | rnfG; Electron transport complex protein; K03612 electron transport complex protein RnfG |  |
| ebi:EbC\_19470 | rnfD; Electron transport complex protein; K03614 electron transport complex protein RnfD |  |
| ebi:EbC\_19480 | rnfC; Electron transport complex protein; K03615 electron transport complex protein RnfC |  |
| ebi:EbC\_19490 | rnfB; Electron transport complex protein; K03616 electron transport complex protein RnfB |  |
| ebi:EbC\_19500 | rnfA; Electron transport complex protein; K03617 electron transport complex protein RnfA |  |

  
**Neighborhood Representations for "pct:PC1\_2556"**  

| ID | Annotation | EC number |
| --- | --- | --- |
| pct:PC1\_2546 | hypothetical protein |  |
| pct:PC1\_2547 | regulator of competence-specific genes; K07343 DNA transformation protein and related proteins |  |
| pct:PC1\_2548 | cell division inhibitor SulA; K13053 cell division inhibitor SulA |  |
| pct:PC1\_2549 | OmpA domain-containing protein transmembrane region-containing protein; K03286 OmpA-OmpF porin, OOP family |  |
| pct:PC1\_2550 | hypothetical protein; K09911 hypothetical protein |  |
| pct:PC1\_2551 | hypothetical protein; K04770 Lon-like ATP-dependent protease [EC:3.4.21.-] |  |
| pct:PC1\_2552 | beta-hydroxyacyl-(acyl-carrier-protein) dehydratase FabA (EC:4.2.1.60); K01716 3-hydroxyacyl-[acyl-carrier-protein] dehydratase [EC:4.2.1.59] | ec:4.2.1.59 |
| pct:PC1\_2553 | hypothetical protein |  |
| pct:PC1\_2554 | choline dehydrogenase; K00108 choline dehydrogenase [EC:1.1.99.1] | ec:1.1.99.1 |
| pct:PC1\_2555 | betaine aldehyde dehydrogenase; K00130 betaine-aldehyde dehydrogenase [EC:1.2.1.8] | ec:1.2.1.8 |
| pct:PC1\_2556 | TetR family transcriptional regulator; K02167 TetR/AcrR family transcriptional regulator, transcriptional repressor of bet genes |  |
| pct:PC1\_2557 | choline/carnitine/betaine transporter; K02168 choline/glycine/proline betaine transport protein |  |
| pct:PC1\_2558 | Ribose/galactose isomerase |  |
| pct:PC1\_2559 | PhoH family protein; K06217 phosphate starvation-inducible protein PhoH and related proteins |  |
| pct:PC1\_2560 | protein FliZ; K02425 FliZ protein |  |
| pct:PC1\_2561 | RNA polymerase sigma-28 subunit FliA/WhiG; K02405 RNA polymerase sigma factor for flagellar operon FliA |  |
| pct:PC1\_2562 | hypothetical protein |  |
| pct:PC1\_2563 | type 12 methyltransferase |  |
| pct:PC1\_2564 | GCN5-like N-acetyltransferase |  |
| pct:PC1\_2565 | hypothetical protein |  |
| pct:PC1\_2566 | hypothetical protein |  |

  
**Neighborhood Representations for "pam:PANA\_2166"**  

| ID | Annotation | EC number |
| --- | --- | --- |
| pam:PANA\_2156 | htpX; HtpX; K03799 heat shock protein HtpX [EC:3.4.24.-] |  |
| pam:PANA\_2157 | prc; Prc; K03797 carboxyl-terminal processing protease [EC:3.4.21.102] | ec:3.4.21.102 |
| pam:PANA\_2158 | proQ; ProQ; K03607 ProP effector |  |
| pam:PANA\_2159 | yebR; hypothetical protein; K07170 GAF domain-containing protein |  |
| pam:PANA\_2160 | yebS; hypothetical protein; K03808 paraquat-inducible protein A |  |
| pam:PANA\_2161 | yebT; hypothetical protein |  |
| pam:PANA\_2162 | yebU; hypothetical protein; K11392 16S rRNA (cytosine1407-C5)-methyltransferase [EC:2.1.1.178] | ec:2.1.1.178 |
| pam:PANA\_2163 | hypothetical Protein; K09984 hypothetical protein |  |
| pam:PANA\_2164 | ycaD; hypothetical protein |  |
| pam:PANA\_2165 | betT; BetT; K02168 choline/glycine/proline betaine transport protein |  |
| pam:PANA\_2166 | betI; BetI; K02167 TetR/AcrR family transcriptional regulator, transcriptional repressor of bet genes |  |
| pam:PANA\_2167 | betB; BetB; K00130 betaine-aldehyde dehydrogenase [EC:1.2.1.8] | ec:1.2.1.8 |
| pam:PANA\_2168 | betA; BetA; K00108 choline dehydrogenase [EC:1.1.99.1] | ec:1.1.99.1 |
| pam:PANA\_2169 | mdtJ; MdtJ; K11743 spermidine export protein MdtJ |  |
| pam:PANA\_2170 | mdtI; MdtI; K11742 spermidine export protein MdtI |  |
| pam:PANA\_2171 | hypothetical Protein |  |
| pam:PANA\_2172 | trpI; TrpI |  |
| pam:PANA\_2173 | dltE; DltE; K14189 uncharacterized oxidoreductase [EC:1.-.-.-] |  |
| pam:PANA\_2174 | bcsY; BcsY |  |
| pam:PANA\_2175 | hypothetical Protein |  |
| pam:PANA\_2176 | tar; Tar; K03406 methyl-accepting chemotaxis protein |  |

  
**Neighborhood Representations for "eca:ECA1744"**  

| ID | Annotation | EC number |
| --- | --- | --- |
| eca:ECA1735 | hypothetical protein |  |
| eca:ECA1736 | hypothetical protein |  |
| eca:ECA1737 | acetyltransferase |  |
| eca:ECA1738 | hypothetical protein |  |
| eca:ECA1739 | fliA; flagellar biosynthesis sigma factor; K02405 RNA polymerase sigma factor for flagellar operon FliA |  |
| eca:ECA1740 | fliZ; flagella biosynthesis protein FliZ; K02425 FliZ protein |  |
| eca:ECA1741 | phoH; hypothetical protein; K06217 phosphate starvation-inducible protein PhoH and related proteins |  |
| eca:ECAt035 | tRNA-Ser; K14233 tRNA Ser |  |
| eca:ECA1742 | hypothetical protein |  |
| eca:ECA1743 | betT; choline transport protein BetT; K02168 choline/glycine/proline betaine transport protein |  |
| eca:ECA1744 | betI; transcriptional regulator BetI; K02167 TetR/AcrR family transcriptional regulator, transcriptional repressor of bet genes |  |
| eca:ECA1745 | betB; betaine aldehyde dehydrogenase (EC:1.2.1.8); K00130 betaine-aldehyde dehydrogenase [EC:1.2.1.8] | ec:1.2.1.8 |
| eca:ECA1746 | betA; choline dehydrogenase (EC:1.1.99.1); K00108 choline dehydrogenase [EC:1.1.99.1] | ec:1.1.99.1 |
| eca:ECA1747 | hypothetical protein |  |
| eca:ECA1748 | fabA; 3-hydroxydecanoyl-ACP dehydratase (EC:4.2.1.60); K01716 3-hydroxyacyl-[acyl-carrier-protein] dehydratase [EC:4.2.1.59] | ec:4.2.1.59 |
| eca:ECA1749 | hypothetical protein; K04770 Lon-like ATP-dependent protease [EC:3.4.21.-] |  |
| eca:ECA1750 | hypothetical protein; K09911 hypothetical protein |  |
| eca:ECA1751 | ompA; outer membrane protein A; K03286 OmpA-OmpF porin, OOP family |  |
| eca:ECA1752 | sulA; SOS cell division inhibitor; K13053 cell division inhibitor SulA |  |
| eca:ECA1753 | hypothetical protein; K07343 DNA transformation protein and related proteins |  |
| eca:ECA1754 | hypothetical protein |  |

  
**Neighborhood Representations for "pva:Pvag\_1614"**  

| ID | Annotation | EC number |
| --- | --- | --- |
| pva:Pvag\_1604 | prc; tail-specific protease (EC:3.4.21.102); K03797 carboxyl-terminal processing protease [EC:3.4.21.102] | ec:3.4.21.102 |
| pva:Pvag\_1605 | proQ; ProP effector protein; K03607 ProP effector |  |
| pva:Pvag\_1606 | hypothetical protein; K07170 GAF domain-containing protein |  |
| pva:Pvag\_1607 | yebS; hypothetical protein; K03808 paraquat-inducible protein A |  |
| pva:Pvag\_1608 | pqib3; hypothetical protein |  |
| pva:Pvag\_1609 | yebU; rRNA methyltransferase F (EC:2.1.1.-); K11392 16S rRNA (cytosine1407-C5)-methyltransferase [EC:2.1.1.178] | ec:2.1.1.178 |
| pva:Pvag\_1610 | pphA; serine/threonine protein phosphatase 1 (EC:3.1.3.16); K07313 serine/threonine protein phosphatase 1 [EC:3.1.3.16] | ec:3.1.3.16 |
| pva:Pvag\_1611 | hypothetical protein |  |
| pva:Pvag\_1612 | MFS family transporter |  |
| pva:Pvag\_1613 | betT; High-affinity choline transport protein; K02168 choline/glycine/proline betaine transport protein |  |
| pva:Pvag\_1614 | betI; HTH-type transcriptional regulator betI; K02167 TetR/AcrR family transcriptional regulator, transcriptional repressor of bet genes |  |
| pva:Pvag\_1615 | betB; betaine aldehyde dehydrogenase (EC:1.2.1.8); K00130 betaine-aldehyde dehydrogenase [EC:1.2.1.8] | ec:1.2.1.8 |
| pva:Pvag\_1616 | betA; choline dehydrogenase (EC:1.1.99.1); K00108 choline dehydrogenase [EC:1.1.99.1] | ec:1.1.99.1 |
| pva:Pvag\_1617 | ydgF; SMR family transporter ydgF; K11743 spermidine export protein MdtJ |  |
| pva:Pvag\_1618 | ydgE; SMR family transporter ydgE; K11742 spermidine export protein MdtI |  |
| pva:Pvag\_1619 | oafA; O-acetyltransferase (EC:2.3.1.-) |  |
| pva:Pvag\_1620 | hypothetical protein |  |
| pva:Pvag\_1621 | hypothetical protein |  |
| pva:Pvag\_1622 | hypothetical protein |  |
| pva:Pvag\_1623 | adhP; alcohol dehydrogenase (EC:1.1.1.1); K13953 alcohol dehydrogenase, propanol-preferring [EC:1.1.1.1] | ec:1.1.1.1 |
| pva:Pvag\_1624 | hypothetical protein |  |

  
**Neighborhood Representations for "esa:ESA\_02047"**  

| ID | Annotation | EC number |
| --- | --- | --- |
| esa:ESA\_02037 | hypothetical protein |  |
| esa:ESA\_02038 | hypothetical protein; K11906 type VI secretion system protein VasD |  |
| esa:ESA\_02039 | hypothetical protein; K11895 type VI secretion system protein ImpH |  |
| esa:ESA\_02040 | hypothetical protein; K11896 type VI secretion system protein ImpG |  |
| esa:ESA\_02041 | hypothetical protein; K11891 type VI secretion system protein ImpL |  |
| esa:ESA\_02042 | hypothetical protein; K11900 type VI secretion system protein ImpC |  |
| esa:ESA\_02043 | hypothetical protein; K11901 type VI secretion system protein ImpB |  |
| esa:ESA\_02044 | hypothetical protein |  |
| esa:ESA\_02045 | hypothetical protein |  |
| esa:ESA\_02046 | choline transport protein BetT; K02168 choline/glycine/proline betaine transport protein |  |
| esa:ESA\_02047 | transcriptional regulator BetI; K02167 TetR/AcrR family transcriptional regulator, transcriptional repressor of bet genes |  |
| esa:ESA\_02048 | betaine aldehyde dehydrogenase; K00130 betaine-aldehyde dehydrogenase [EC:1.2.1.8] | ec:1.2.1.8 |
| esa:ESA\_02049 | choline dehydrogenase; K00108 choline dehydrogenase [EC:1.1.99.1] | ec:1.1.99.1 |
| esa:ESA\_02050 | hypothetical protein |  |
| esa:ESA\_02051 | hypothetical protein |  |
| esa:ESA\_02052 | hypothetical protein |  |
| esa:ESA\_02053 | hypothetical protein; K08169 MFS transporter, DHA2 family, multidrug resistance protein |  |
| esa:ESA\_02054 | hypothetical protein |  |
| esa:ESA\_02055 | pseudogene |  |
| esa:ESA\_02056 | hypothetical protein; K07241 high-affinity nickel-transport protein |  |
| esa:ESA\_02057 | hypothetical protein; K04656 hydrogenase maturation protein HypF |  |

  
**Neighborhood Representations for "eta:ETA\_17830"**  

| ID | Annotation | EC number |
| --- | --- | --- |
| eta:ETA\_17730 | ydgK; hypothetical protein |  |
| eta:ETA\_17740 | rnfA; electron transport complex protein; K03617 electron transport complex protein RnfA |  |
| eta:ETA\_17750 | rnfB; electron transport complex protein RnfB; K03616 electron transport complex protein RnfB |  |
| eta:ETA\_17760 | rnfC; Electron transport complex protein; K03615 electron transport complex protein RnfC |  |
| eta:ETA\_17770 | rnfD; electron transport complex protein RnfD; K03614 electron transport complex protein RnfD |  |
| eta:ETA\_17780 | rnfG; electron transport complex protein RnfG; K03612 electron transport complex protein RnfG |  |
| eta:ETA\_17790 | rnfE; electron transport complex protein RsxE; K03613 electron transport complex protein RnfE |  |
| eta:ETA\_17800 | nth; Endonuclease III (EC:4.2.99.18); K10773 endonuclease III [EC:4.2.99.18] | ec:4.2.99.18 |
| eta:ETA\_17810 | betA; choline dehydrogenase (EC:1.1.99.1); K00108 choline dehydrogenase [EC:1.1.99.1] | ec:1.1.99.1 |
| eta:ETA\_17820 | betB; betaine aldehyde dehydrogenase (EC:1.2.1.8); K00130 betaine-aldehyde dehydrogenase [EC:1.2.1.8] | ec:1.2.1.8 |
| eta:ETA\_17830 | betI; HTH-type transcriptional regulator; K02167 TetR/AcrR family transcriptional regulator, transcriptional repressor of bet genes |  |
| eta:ETA\_17840 | tppB; tripeptide transporter permease; K03305 proton-dependent oligopeptide transporter, POT family |  |
| eta:ETA\_17850 | gst; glutathionine S-transferase (EC:2.5.1.18); K00799 glutathione S-transferase [EC:2.5.1.18] | ec:2.5.1.18 |
| eta:ETA\_17860 | pdxY; pyridoxamine kinase (EC:2.7.1.35); K00868 pyridoxine kinase [EC:2.7.1.35] | ec:2.7.1.35 |
| eta:ETA\_17870 | tyrS; tyrosyl-tRNA synthetase (EC:6.1.1.1); K01866 tyrosyl-tRNA synthetase [EC:6.1.1.1] | ec:6.1.1.1 |
| eta:ETA\_17880 | pdxH; pyridoxamine 5'-phosphate oxidase (EC:1.4.3.5); K00275 pyridoxamine 5'-phosphate oxidase [EC:1.4.3.5] | ec:1.4.3.5 |
| eta:ETA\_17890 | anmK; anhydro-N-acetylmuramic acid kinase; K09001 anhydro-N-acetylmuramic acid kinase [EC:2.7.1.170] | ec:2.7.1.170 |
| eta:ETA\_17900 | slyB; outer membrane lipoprotein; K06077 outer membrane lipoprotein SlyB |  |
| eta:ETA\_17910 | slyA; transcriptional regulator SlyA; K06075 MarR family transcriptional regulator, transcriptional regulator for hemolysin |  |
| eta:ETA\_17920 | ydhI; hypothetical protein |  |
| eta:ETA\_17930 | ydhJ; hypothetical protein |  |

  
**Neighborhood Representations for "eoh:ECO103\_0290"**  

| ID | Annotation | EC number |
| --- | --- | --- |
| eoh:ECO103\_0280 | ykgC; oxidoreductase |  |
| eoh:ECO103\_0281 | ykgD; DNA-binding transcriptional regulator, ARAC-type |  |
| eoh:ECO103\_0282 | ykgE; oxidoreductase |  |
| eoh:ECO103\_0283 | ykgF; amino acid dehydrogenase |  |
| eoh:ECO103\_0284 | ykgG; transporter; K00782 hypothetical protein |  |
| eoh:ECO103\_0285 | hypothetical protein |  |
| eoh:ECO103\_0286 | ykgH; pseudogene |  |
| eoh:ECO103\_0287 | ykgH; pseudogene |  |
| eoh:ECO103\_0288 | betA; choline dehydrogenase; K00108 choline dehydrogenase [EC:1.1.99.1] | ec:1.1.99.1 |
| eoh:ECO103\_0289 | betB; betaine aldehyde dehydrogenase, NAD-dependent; K00130 betaine-aldehyde dehydrogenase [EC:1.2.1.8] | ec:1.2.1.8 |
| eoh:ECO103\_0290 | betI; DNA-binding transcriptional repressor BetI; K02167 TetR/AcrR family transcriptional regulator, transcriptional repressor of bet genes |  |
| eoh:ECO103\_0291 | betT; high affinity choline transporter BetT; K02168 choline/glycine/proline betaine transport protein |  |
| eoh:ECO103\_0292 | AidA-I adhesin-like protein; K12678 autotransporter family porin |  |
| eoh:ECO103\_0293 | yahA; DNA-binding transcriptional regulator; K13244 c-di-GMP-specific phosphodiesterase [EC:3.1.4.52] | ec:3.1.4.52 |
| eoh:ECO103\_0294 | yahB; pseudogene |  |
| eoh:ECO103\_0295 | yahC; inner membrane protein |  |
| eoh:ECO103\_0296 | yahD; transcriptional regulator; K06867 |  |
| eoh:ECO103\_0297 | yahE; hypothetical protein |  |
| eoh:ECO103\_0298 | yahF; acyl-CoA synthetase |  |
| eoh:ECO103\_0299 | yahG; hypothetical protein |  |
| eoh:ECO103\_0300 | yahI; carbamate kinase; K00926 carbamate kinase [EC:2.7.2.2] | ec:2.7.2.2 |

  
**Neighborhood Representations for "ctu:CTU\_19220"**  

| ID | Annotation | EC number |
| --- | --- | --- |
| ctu:CTU\_19120 | hypothetical protein; K08169 MFS transporter, DHA2 family, multidrug resistance protein |  |
| ctu:CTU\_19130 | hypothetical protein |  |
| ctu:CTU\_19140 | hypothetical protein |  |
| ctu:CTU\_19150 | hypothetical protein |  |
| ctu:CTU\_19160 | anti-RssB factor |  |
| ctu:CTU\_19170 | hypothetical protein |  |
| ctu:CTU\_19180 | hypothetical protein |  |
| ctu:CTU\_19190 | hypothetical protein |  |
| ctu:CTU\_19200 | betA; choline dehydrogenase (EC:1.1.99.1); K00108 choline dehydrogenase [EC:1.1.99.1] | ec:1.1.99.1 |
| ctu:CTU\_19210 | betB; betaine aldehyde dehydrogenase (EC:1.2.1.19 1.2.1.8); K00130 betaine-aldehyde dehydrogenase [EC:1.2.1.8] | ec:1.2.1.8 |
| ctu:CTU\_19220 | betI; transcriptional regulator BetI; K02167 TetR/AcrR family transcriptional regulator, transcriptional repressor of bet genes |  |
| ctu:CTU\_19230 | betT; choline transport protein BetT; K02168 choline/glycine/proline betaine transport protein |  |
| ctu:CTU\_19240 | hypothetical protein |  |
| ctu:CTU\_19250 | hypothetical protein |  |
| ctu:CTU\_19260 | hypothetical protein |  |
| ctu:CTU\_19270 | hypothetical protein |  |
| ctu:CTU\_19280 | hypothetical protein; K11901 type VI secretion system protein ImpB |  |
| ctu:CTU\_19290 | hypothetical protein; K11900 type VI secretion system protein ImpC |  |
| ctu:CTU\_19300 | hypothetical protein; K11893 type VI secretion system protein ImpJ |  |
| ctu:CTU\_19310 | hypothetical protein; K11892 type VI secretion system protein ImpK |  |
| ctu:CTU\_19320 | hypothetical protein |  |

  
**Neighborhood Representations for "cko:CKO\_02582"**  

| ID | Annotation | EC number |
| --- | --- | --- |
| cko:CKO\_02572 | iron-enterobactin transporter permease; K02015 iron complex transport system permease protein |  |
| cko:CKO\_02573 | iron-enterobactin transporter ATP-binding protein; K02013 iron complex transport system ATP-binding protein [EC:3.6.3.34] | ec:3.6.3.34 |
| cko:CKO\_02574 | ferric enterobactin transport protein FepE |  |
| cko:CKO\_02575 | entF; enterobactin synthase subunit F; K02364 enterobactin synthetase component F [EC:2.7.7.-] |  |
| cko:CKO\_02576 | hypothetical protein |  |
| cko:CKO\_02577 | enterobactin/ferric enterobactin esterase; K07214 enterochelin esterase and related enzymes |  |
| cko:CKO\_02578 | hypothetical protein |  |
| cko:CKO\_02579 | outer membrane receptor FepA; K16089 outer membrane receptor for ferrienterochelin and colicins |  |
| cko:CKO\_02580 | phosphopantetheinyltransferase component of enterobactin synthase multienzyme complex; K02362 enterobactin synthetase component D [EC:2.7.8.-] |  |
| cko:CKO\_02581 | choline transport protein BetT; K02168 choline/glycine/proline betaine transport protein |  |
| cko:CKO\_02582 | transcriptional regulator BetI; K02167 TetR/AcrR family transcriptional regulator, transcriptional repressor of bet genes |  |
| cko:CKO\_02583 | betaine aldehyde dehydrogenase; K00130 betaine-aldehyde dehydrogenase [EC:1.2.1.8] | ec:1.2.1.8 |
| cko:CKO\_02584 | choline dehydrogenase; K00108 choline dehydrogenase [EC:1.1.99.1] | ec:1.1.99.1 |
| cko:CKO\_02585 | carboxylate-amine ligase; K06048 carboxylate-amine ligase [EC:6.3.-.-] |  |
| cko:CKO\_02587 | hypothetical protein |  |
| cko:CKO\_02586 | hypothetical protein |  |
| cko:CKO\_02588 | hypothetical protein; K07214 enterochelin esterase and related enzymes |  |
| cko:CKO\_02589 | hypothetical protein; K02014 iron complex outermembrane recepter protein |  |
| cko:CKO\_02590 | hypothetical protein |  |
| cko:CKO\_02591 | hypothetical protein |  |
| cko:CKO\_02592 | hypothetical protein |  |

  
**Neighborhood Representations for "eck:EC55989\_0315"**  

| ID | Annotation | EC number |
| --- | --- | --- |
| eck:EC55989\_0305 | ykgI; hypothetical protein |  |
| eck:EC55989\_0306 | ykgC; pyridine nucleotide-disulfide oxidoreductase |  |
| eck:EC55989\_0307 | ykgD; AraC-type DNA-binding transcriptional regulator |  |
| eck:EC55989\_0308 | ykgE; hydroxyacid oxidoreductase (Fe-S centre) |  |
| eck:EC55989\_0309 | ykgF; oxidoreductase subunit with NAD(P)-binding domain and ferridoxin-like domain |  |
| eck:EC55989\_0310 | ykgG; hypothetical protein; K00782 hypothetical protein |  |
| eck:EC55989\_0311 | hypothetical protein |  |
| eck:EC55989\_0312 | ykgH; inner membrane protein |  |
| eck:EC55989\_0313 | betA; choline dehydrogenase (EC:1.1.99.1); K00108 choline dehydrogenase [EC:1.1.99.1] | ec:1.1.99.1 |
| eck:EC55989\_0314 | betB; betaine aldehyde dehydrogenase (EC:1.2.1.8); K00130 betaine-aldehyde dehydrogenase [EC:1.2.1.8] | ec:1.2.1.8 |
| eck:EC55989\_0315 | betI; transcriptional regulator BetI; K02167 TetR/AcrR family transcriptional regulator, transcriptional repressor of bet genes |  |
| eck:EC55989\_0316 | betT; choline transport protein BetT; K02168 choline/glycine/proline betaine transport protein |  |
| eck:EC55989\_0317 | AidA-I adhesin-like protein; K12678 autotransporter family porin |  |
| eck:EC55989\_0318 | yahA; cyclic di-GMP phosphodiesterase; K13244 c-di-GMP-specific phosphodiesterase [EC:3.1.4.52] | ec:3.1.4.52 |
| eck:EC55989\_0319 | yahB; DNA-binding transcriptional regulator |  |
| eck:EC55989\_0320 | yahC; inner membrane protein |  |
| eck:EC55989\_0321 | yahD; protein with ankyrin domain; K06867 |  |
| eck:EC55989\_0322 | yahE; hypothetical protein |  |
| eck:EC55989\_0323 | yahF; enzyme with acyl-CoA domain |  |
| eck:EC55989\_0324 | yahG; hypothetical protein |  |
| eck:EC55989\_0325 | yahI; carbamate kinase; K00926 carbamate kinase [EC:2.7.2.2] | ec:2.7.2.2 |

  
**Neighborhood Representations for "ecm:EcSMS35\_0344"**  

| ID | Annotation | EC number |
| --- | --- | --- |
| ecm:EcSMS35\_0334 | hypothetical protein |  |
| ecm:EcSMS35\_0335 | hypothetical protein |  |
| ecm:EcSMS35\_0336 | pyridine nucleotide-disulfide oxidoreductase |  |
| ecm:EcSMS35\_0337 | AraC family transcriptional regulator |  |
| ecm:EcSMS35\_0338 | hypothetical protein |  |
| ecm:EcSMS35\_0339 | iron-sulfur cluster binding protein |  |
| ecm:EcSMS35\_0340 | hypothetical protein; K00782 hypothetical protein |  |
| ecm:EcSMS35\_0341 | hypothetical protein |  |
| ecm:EcSMS35\_0342 | betA; choline dehydrogenase (EC:1.1.99.1); K00108 choline dehydrogenase [EC:1.1.99.1] | ec:1.1.99.1 |
| ecm:EcSMS35\_0343 | betB; betaine aldehyde dehydrogenase (EC:1.2.1.8); K00130 betaine-aldehyde dehydrogenase [EC:1.2.1.8] | ec:1.2.1.8 |
| ecm:EcSMS35\_0344 | betI; transcriptional regulator BetI; K02167 TetR/AcrR family transcriptional regulator, transcriptional repressor of bet genes |  |
| ecm:EcSMS35\_0345 | betT; choline transport protein BetT; K02168 choline/glycine/proline betaine transport protein |  |
| ecm:EcSMS35\_0346 | LuxR-family transcriptional regulator; K13244 c-di-GMP-specific phosphodiesterase [EC:3.1.4.52] | ec:3.1.4.52 |
| ecm:EcSMS35\_0347 | LysR family transcriptional regulator |  |
| ecm:EcSMS35\_0348 | hypothetical protein |  |
| ecm:EcSMS35\_0349 | ankyrin repeat-containing protein; K06867 |  |
| ecm:EcSMS35\_0350 | hypothetical protein |  |
| ecm:EcSMS35\_0351 | acyl-CoA synthetase |  |
| ecm:EcSMS35\_0352 | hypothetical protein |  |
| ecm:EcSMS35\_0353 | putative carbamate kinase; K00926 carbamate kinase [EC:2.7.2.2] | ec:2.7.2.2 |
| ecm:EcSMS35\_0354 | putative deaminase |  |

  
**Neighborhood Representations for "ecr:ECIAI1\_0310"**  

| ID | Annotation | EC number |
| --- | --- | --- |
| ecr:ECIAI1\_0300 | ykgI; hypothetical protein |  |
| ecr:ECIAI1\_0301 | ykgC; pyridine nucleotide-disulfide oxidoreductase |  |
| ecr:ECIAI1\_0302 | ykgD; putative AraC-type DNA-binding transcriptional regulator |  |
| ecr:ECIAI1\_0303 | ykgE; putative hydroxyacid oxidoreductase |  |
| ecr:ECIAI1\_0304 | ykgF; putative oxidoreductase subunit |  |
| ecr:ECIAI1\_0305 | ykgG; hypothetical protein; K00782 hypothetical protein |  |
| ecr:ECIAI1\_0306 | hypothetical protein |  |
| ecr:ECIAI1\_0307 | ykgH; hypothetical protein |  |
| ecr:ECIAI1\_0308 | betA; choline dehydrogenase (EC:1.1.99.1); K00108 choline dehydrogenase [EC:1.1.99.1] | ec:1.1.99.1 |
| ecr:ECIAI1\_0309 | betB; betaine aldehyde dehydrogenase (EC:1.2.1.8); K00130 betaine-aldehyde dehydrogenase [EC:1.2.1.8] | ec:1.2.1.8 |
| ecr:ECIAI1\_0310 | betI; transcriptional regulator BetI; K02167 TetR/AcrR family transcriptional regulator, transcriptional repressor of bet genes |  |
| ecr:ECIAI1\_0311 | betT; choline transport protein BetT; K02168 choline/glycine/proline betaine transport protein |  |
| ecr:ECIAI1\_0312 | AidA-I adhesin-like protein; K12678 autotransporter family porin |  |
| ecr:ECIAI1\_0313 | yahA; cyclic di-GMP phosphodiesterase; K13244 c-di-GMP-specific phosphodiesterase [EC:3.1.4.52] | ec:3.1.4.52 |
| ecr:ECIAI1\_0314 | yahB; putative DNA-binding transcriptional regulator |  |
| ecr:ECIAI1\_0315 | yahC; hypothetical protein |  |
| ecr:ECIAI1\_0316 | yahD; hypothetical protein; K06867 |  |
| ecr:ECIAI1\_0317 | yahE; hypothetical protein |  |
| ecr:ECIAI1\_0318 | yahF; hypothetical protein |  |
| ecr:ECIAI1\_0319 | yahG; hypothetical protein |  |
| ecr:ECIAI1\_0320 | yahI; putative carbamate kinase; K00926 carbamate kinase [EC:2.7.2.2] | ec:2.7.2.2 |

  
**Neighborhood Representations for "ecw:EcE24377A\_0328"**  

| ID | Annotation | EC number |
| --- | --- | --- |
| ecw:EcE24377A\_0318 | pyridine nucleotide-disulfide oxidoreductase |  |
| ecw:EcE24377A\_0319 | hypothetical protein |  |
| ecw:EcE24377A\_0320 | AraC family transcriptional regulator |  |
| ecw:EcE24377A\_0321 | hypothetical protein |  |
| ecw:EcE24377A\_0322 | iron-sulfur cluster binding protein |  |
| ecw:EcE24377A\_0323 | hypothetical protein; K00782 hypothetical protein |  |
| ecw:EcE24377A\_0324 | hypothetical protein |  |
| ecw:EcE24377A\_0325 | hypothetical protein |  |
| ecw:EcE24377A\_0326 | betA; choline dehydrogenase (EC:1.1.99.1); K00108 choline dehydrogenase [EC:1.1.99.1] | ec:1.1.99.1 |
| ecw:EcE24377A\_0327 | betB; betaine aldehyde dehydrogenase (EC:1.2.1.8); K00130 betaine-aldehyde dehydrogenase [EC:1.2.1.8] | ec:1.2.1.8 |
| ecw:EcE24377A\_0328 | betI; transcriptional regulator BetI; K02167 TetR/AcrR family transcriptional regulator, transcriptional repressor of bet genes |  |
| ecw:EcE24377A\_0329 | IS1, transposase orfA |  |
| ecw:EcE24377A\_0330 | IS1, transposase orfB; K07480 insertion element IS1 protein InsB |  |
| ecw:EcE24377A\_0331 | betT; choline transport protein BetT; K02168 choline/glycine/proline betaine transport protein |  |
| ecw:EcE24377A\_0332 | outer membrane autotransporter; K12678 autotransporter family porin |  |
| ecw:EcE24377A\_0333 | LuxR family transcriptional regulator; K13244 c-di-GMP-specific phosphodiesterase [EC:3.1.4.52] | ec:3.1.4.52 |
| ecw:EcE24377A\_0334 | LysR family transcriptional regulator |  |
| ecw:EcE24377A\_0335 | hypothetical protein |  |
| ecw:EcE24377A\_0336 | hypothetical protein |  |
| ecw:EcE24377A\_0337 | ankyrin repeat-containing protein; K06867 |  |
| ecw:EcE24377A\_0338 | hypothetical protein |  |

  
**Neighborhood Representations for "ecx:EcHS\_A0372"**  

| ID | Annotation | EC number |
| --- | --- | --- |
| ecx:EcHS\_A0362 | hypothetical protein |  |
| ecx:EcHS\_A0363 | outer membrane autotransporter |  |
| ecx:EcHS\_A0364 | hypothetical protein |  |
| ecx:EcHS\_A0365 | HTH DNA-binding domain-containing protein |  |
| ecx:EcHS\_A0366 | phage integrase site specific recombinase; K07357 type 1 fimbriae regulatory protein FimB |  |
| ecx:EcHS\_A0367 | IS1, transposase orfA |  |
| ecx:EcHS\_A0368 | hypothetical protein |  |
| ecx:EcHS\_A0369 | hypothetical protein |  |
| ecx:EcHS\_A0370 | betA; choline dehydrogenase (EC:1.1.99.1); K00108 choline dehydrogenase [EC:1.1.99.1] | ec:1.1.99.1 |
| ecx:EcHS\_A0371 | betB; betaine aldehyde dehydrogenase (EC:1.2.1.8); K00130 betaine-aldehyde dehydrogenase [EC:1.2.1.8] | ec:1.2.1.8 |
| ecx:EcHS\_A0372 | betI; transcriptional regulator BetI; K02167 TetR/AcrR family transcriptional regulator, transcriptional repressor of bet genes |  |
| ecx:EcHS\_A0373 | betT; choline transport protein BetT; K02168 choline/glycine/proline betaine transport protein |  |
| ecx:EcHS\_A0374 | outer membrane autotransporter; K12678 autotransporter family porin |  |
| ecx:EcHS\_A0375 | LuxR family transcriptional regulator; K13244 c-di-GMP-specific phosphodiesterase [EC:3.1.4.52] | ec:3.1.4.52 |
| ecx:EcHS\_A0376 | LysR family transcriptional regulator |  |
| ecx:EcHS\_A0377 | hypothetical protein |  |
| ecx:EcHS\_A0378 | hypothetical protein |  |
| ecx:EcHS\_A0379 | ankyrin repeat-containing protein; K06867 |  |
| ecx:EcHS\_A0380 | hypothetical protein |  |
| ecx:EcHS\_A0381 | acyl-CoA synthetase |  |
| ecx:EcHS\_A0382 | hypothetical protein |  |

  
**Neighborhood Representations for "ecy:ECSE\_0334"**  

| ID | Annotation | EC number |
| --- | --- | --- |
| ecy:ECSE\_0324 | hypothetical protein |  |
| ecy:ECSE\_0325 | putative transport protein; K00782 hypothetical protein |  |
| ecy:ECSE\_0326 | hypothetical protein |  |
| ecy:ECSE\_0327 | putative autotransporter |  |
| ecy:ECSE\_0328 | hypothetical protein |  |
| ecy:ECSE\_0329 | hypothetical protein |  |
| ecy:ECSE\_0330 | hypothetical protein |  |
| ecy:ECSE\_0331 | putative phage integrase; K07357 type 1 fimbriae regulatory protein FimB |  |
| ecy:ECSE\_0332 | choline dehydrogenase; K00108 choline dehydrogenase [EC:1.1.99.1] | ec:1.1.99.1 |
| ecy:ECSE\_0333 | betaine aldehyde dehydrogenase; K00130 betaine-aldehyde dehydrogenase [EC:1.2.1.8] | ec:1.2.1.8 |
| ecy:ECSE\_0334 | transcriptional regulator BetI; K02167 TetR/AcrR family transcriptional regulator, transcriptional repressor of bet genes |  |
| ecy:ECSE\_0335 | choline transport protein BetT; K02168 choline/glycine/proline betaine transport protein |  |
| ecy:ECSE\_0336 | hypothetical protein; K13244 c-di-GMP-specific phosphodiesterase [EC:3.1.4.52] | ec:3.1.4.52 |
| ecy:ECSE\_0337 | putative transcriptional regulator |  |
| ecy:ECSE\_0338 | hypothetical protein |  |
| ecy:ECSE\_0339 | hypothetical protein; K06867 |  |
| ecy:ECSE\_0340 | hypothetical protein |  |
| ecy:ECSE\_0341 | hypothetical protein |  |
| ecy:ECSE\_0342 | hypothetical protein |  |
| ecy:ECSE\_0343 | putative carbamate kinase; K00926 carbamate kinase [EC:2.7.2.2] | ec:2.7.2.2 |
| ecy:ECSE\_0344 | putative deaminase |  |

  
**Neighborhood Representations for "eoi:ECO111\_0347"**  

| ID | Annotation | EC number |
| --- | --- | --- |
| eoi:ECO111\_0337 | ykgI; hypothetical protein |  |
| eoi:ECO111\_0338 | ykgC; putative oxidoreductase |  |
| eoi:ECO111\_0339 | ykgD; putative DNA-binding transcriptional regulator, ARAC-type |  |
| eoi:ECO111\_0340 | ykgE; putative oxidoreductase |  |
| eoi:ECO111\_0341 | ykgF; putative amino acid dehydrogenase |  |
| eoi:ECO111\_0342 | ykgG; putative transporter; K00782 hypothetical protein |  |
| eoi:ECO111\_0343 | hypothetical protein |  |
| eoi:ECO111\_0344 | ykgH; putative inner membrane protein |  |
| eoi:ECO111\_0345 | betA; choline dehydrogenase; K00108 choline dehydrogenase [EC:1.1.99.1] | ec:1.1.99.1 |
| eoi:ECO111\_0346 | betB; betaine aldehyde dehydrogenase, NAD-dependent; K00130 betaine-aldehyde dehydrogenase [EC:1.2.1.8] | ec:1.2.1.8 |
| eoi:ECO111\_0347 | betI; DNA-binding transcriptional repressor BetI; K02167 TetR/AcrR family transcriptional regulator, transcriptional repressor of bet genes |  |
| eoi:ECO111\_0348 | betT; high affinity choline transporter BetT; K02168 choline/glycine/proline betaine transport protein |  |
| eoi:ECO111\_0349 | AidA-I adhesin-like protein; K12678 autotransporter family porin |  |
| eoi:ECO111\_0350 | yahA; putative DNA-binding transcriptional regulator; K13244 c-di-GMP-specific phosphodiesterase [EC:3.1.4.52] | ec:3.1.4.52 |
| eoi:ECO111\_0351 | yahB; putative DNA-binding transcriptional regulator, LYSR-type |  |
| eoi:ECO111\_0352 | yahC; putative inner membrane protein |  |
| eoi:ECO111\_0353 | yahD; putative transcriptional regulator; K06867 |  |
| eoi:ECO111\_0354 | yahE; hypothetical protein |  |
| eoi:ECO111\_0355 | yahF; putative acyl-CoA synthetase |  |
| eoi:ECO111\_0356 | yahG; hypothetical protein |  |
| eoi:ECO111\_0357 | yahI; putative carbamate kinase-like protein; K00926 carbamate kinase [EC:2.7.2.2] | ec:2.7.2.2 |

  
**Neighborhood Representations for "eoj:ECO26\_0347"**  

| ID | Annotation | EC number |
| --- | --- | --- |
| eoj:ECO26\_0337 | ykgI; hypothetical protein |  |
| eoj:ECO26\_0338 | ykgC; pyridine nucleotide-disulfide oxidoreductase |  |
| eoj:ECO26\_0339 | ykgD; DNA-binding transcriptional regulator, ARAC-type |  |
| eoj:ECO26\_0340 | ykgE; oxidoreductase |  |
| eoj:ECO26\_0341 | ykgF; amino acid dehydrogenase |  |
| eoj:ECO26\_0342 | ykgG; transporter; K00782 hypothetical protein |  |
| eoj:ECO26\_0343 | hypothetical protein |  |
| eoj:ECO26\_0344 | ykgH; hypothetical protein |  |
| eoj:ECO26\_0345 | betA; choline dehydrogenase; K00108 choline dehydrogenase [EC:1.1.99.1] | ec:1.1.99.1 |
| eoj:ECO26\_0346 | betB; betaine aldehyde dehydrogenase; K00130 betaine-aldehyde dehydrogenase [EC:1.2.1.8] | ec:1.2.1.8 |
| eoj:ECO26\_0347 | betI; transcriptional regulator BetI; K02167 TetR/AcrR family transcriptional regulator, transcriptional repressor of bet genes |  |
| eoj:ECO26\_0348 | betT; choline transport protein BetT; K02168 choline/glycine/proline betaine transport protein |  |
| eoj:ECO26\_0349 | AidA-I adhesin; K12678 autotransporter family porin |  |
| eoj:ECO26\_0350 | yahA; DNA-binding transcriptional regulator; K13244 c-di-GMP-specific phosphodiesterase [EC:3.1.4.52] | ec:3.1.4.52 |
| eoj:ECO26\_0351 | yahB; pseudogene |  |
| eoj:ECO26\_0352 | yahC; pseudogene |  |
| eoj:ECO26\_0353 | yahD; transcriptional regulator; K06867 |  |
| eoj:ECO26\_0354 | yahE; hypothetical protein |  |
| eoj:ECO26\_0355 | yahF; acyl-CoA synthetase |  |
| eoj:ECO26\_0356 | yahG; hypothetical protein |  |
| eoj:ECO26\_0357 | yahI; carbamate kinase; K00926 carbamate kinase [EC:2.7.2.2] | ec:2.7.2.2 |

  
**Neighborhood Representations for "sfv:SFV\_0324"**  

| ID | Annotation | EC number |
| --- | --- | --- |
| sfv:SFV\_0314 | Rhs family protein |  |
| sfv:SFV\_0315 | insB; IS1 ORF2; K07480 insertion element IS1 protein InsB |  |
| sfv:SFV\_0316 | IS1 encoded protein |  |
| sfv:SFV\_0317 | ykgE; dehydrogenase subunit |  |
| sfv:SFV\_0318 | ykgF; hypothetical protein |  |
| sfv:SFV\_0319 | ykgG; transporter; K00782 hypothetical protein |  |
| sfv:SFV\_0320 | hypothetical protein |  |
| sfv:SFV\_0321 | ykgH; hypothetical protein |  |
| sfv:SFV\_0322 | betA; choline dehydrogenase (EC:1.1.99.1); K00108 choline dehydrogenase [EC:1.1.99.1] | ec:1.1.99.1 |
| sfv:SFV\_0323 | betB; betaine aldehyde dehydrogenase (EC:1.2.1.8); K00130 betaine-aldehyde dehydrogenase [EC:1.2.1.8] | ec:1.2.1.8 |
| sfv:SFV\_0324 | betI; transcriptional regulator BetI; K02167 TetR/AcrR family transcriptional regulator, transcriptional repressor of bet genes |  |
| sfv:SFV\_0325 | betT; choline transport protein BetT; K02168 choline/glycine/proline betaine transport protein |  |
| sfv:SFV\_0326 | IS1 encoded protein |  |
| sfv:SFV\_0327 | insB; IS1 ORF2; K07480 insertion element IS1 protein InsB |  |
| sfv:SFV\_0328 | phage transposase; K07497 putative transposase |  |
| sfv:SFV\_0329 | tauA; taurine ABC transporter substrate-binding protein; K15551 taurine transport system substrate-binding protein |  |
| sfv:SFV\_0330 | tauB; taurine ABC transporter ATP-binding protein (EC:3.6.3.36); K10831 taurine transport system ATP-binding protein [EC:3.6.3.36] | ec:3.6.3.36 |
| sfv:SFV\_0331 | tauC; taurine transporter subunit; K15552 taurine transport system permease protein |  |
| sfv:SFV\_0332 | tauD; taurine dioxygenase (EC:1.14.11.17); K03119 taurine dioxygenase [EC:1.14.11.17] | ec:1.14.11.17 |
| sfv:SFV\_0333 | hemB; delta-aminolevulinic acid dehydratase (EC:4.2.1.24); K01698 porphobilinogen synthase [EC:4.2.1.24] | ec:4.2.1.24 |
| sfv:SFV\_0334 | yaiU; pseudogene |  |

  
**Neighborhood Representations for "ecc:c0433"**  

| ID | Annotation | EC number |
| --- | --- | --- |
| ecc:c0423 | ykgF; electron transport protein ykgF |  |
| ecc:c0424 | ykgG; hypothetical protein; K00782 hypothetical protein |  |
| ecc:c0425 | ykgH; hypothetical protein |  |
| ecc:c0426 | hypothetical protein |  |
| ecc:c0427 | hypothetical protein |  |
| ecc:c0428 | hypothetical protein |  |
| ecc:c0429 | hypothetical protein |  |
| ecc:c0430 | Type 1 fimbriae regulatory protein fimB; K07357 type 1 fimbriae regulatory protein FimB |  |
| ecc:c0431 | betA; choline dehydrogenase (EC:1.1.99.1); K00108 choline dehydrogenase [EC:1.1.99.1] | ec:1.1.99.1 |
| ecc:c0432 | betB; betaine aldehyde dehydrogenase (EC:1.2.1.8); K00130 betaine-aldehyde dehydrogenase [EC:1.2.1.8] | ec:1.2.1.8 |
| ecc:c0433 | betI; transcriptional regulator BetI; K02167 TetR/AcrR family transcriptional regulator, transcriptional repressor of bet genes |  |
| ecc:c0434 | betT; choline transport protein BetT; K02168 choline/glycine/proline betaine transport protein |  |
| ecc:c0435 | hypothetical protein; K13244 c-di-GMP-specific phosphodiesterase [EC:3.1.4.52] | ec:3.1.4.52 |
| ecc:c0436 | yahB; transcriptional regulator YahB |  |
| ecc:c0437 | yahC; hypothetical protein |  |
| ecc:c0438 | hypothetical protein |  |
| ecc:c0439 | yahD; ankyrin repeat-containing protein; K06867 |  |
| ecc:c0440 | yahE; hypothetical protein |  |
| ecc:c0441 | hypothetical protein |  |
| ecc:c0444 | carbamate kinase; K00926 carbamate kinase [EC:2.7.2.2] | ec:2.7.2.2 |
| ecc:c0445 | yahJ; deaminase |  |

  
**Neighborhood Representations for "ecd:ECDH10B\_0300"**  

| ID | Annotation | EC number |
| --- | --- | --- |
| ecd:ECDH10B\_0290 | ykgB; inner membrane protein |  |
| ecd:ECDH10B\_0291 | ykgI; hypothetical protein |  |
| ecd:ECDH10B\_0292 | ykgC; pyridine nucleotide-disulfide oxidoreductase |  |
| ecd:ECDH10B\_0293 | ykgD; DNA-binding transcriptional regulator |  |
| ecd:ECDH10B\_0294 | ykgE; oxidoreductase |  |
| ecd:ECDH10B\_0295 | ykgF; amino acid dehydrogenase |  |
| ecd:ECDH10B\_0296 | ykgG; transporter; K00782 hypothetical protein |  |
| ecd:ECDH10B\_0297 | ykgH; inner membrane protein |  |
| ecd:ECDH10B\_0298 | betA; choline dehydrogenase; K00108 choline dehydrogenase [EC:1.1.99.1] | ec:1.1.99.1 |
| ecd:ECDH10B\_0299 | betB; betaine aldehyde dehydrogenase; K00130 betaine-aldehyde dehydrogenase [EC:1.2.1.8] | ec:1.2.1.8 |
| ecd:ECDH10B\_0300 | betI; transcriptional regulator BetI; K02167 TetR/AcrR family transcriptional regulator, transcriptional repressor of bet genes |  |
| ecd:ECDH10B\_0301 | betT; choline transport protein BetT; K02168 choline/glycine/proline betaine transport protein |  |
| ecd:ECDH10B\_0302 | yahA; DNA-binding transcriptional regulator; K13244 c-di-GMP-specific phosphodiesterase [EC:3.1.4.52] | ec:3.1.4.52 |
| ecd:ECDH10B\_0303 | yahB; DNA-binding transcriptional regulator |  |
| ecd:ECDH10B\_0304 | yahC; inner membrane protein |  |
| ecd:ECDH10B\_0305 | yahD; transcriptional regulator; K06867 |  |
| ecd:ECDH10B\_0306 | yahE; hypothetical protein |  |
| ecd:ECDH10B\_0307 | yahF; acyl-CoA synthetase |  |
| ecd:ECDH10B\_0308 | mhpT; putative 3-hydroxyphenylpropionic transporter MhpT; K05819 MFS transporter, AAHS family, 3-hydroxyphenylpropionic acid transporter |  |
| ecd:ECDH10B\_0309 | yaiL; nucleoprotein/polynucleotide-associated enzyme; K09912 hypothetical protein |  |
| ecd:ECDH10B\_0310 | frmB; esterase; K01070 S-formylglutathione hydrolase [EC:3.1.2.12] | ec:3.1.2.12 |

  
**Neighborhood Representations for "ece:Z0400"**  

| ID | Annotation | EC number |
| --- | --- | --- |
| ece:Z0389 | hypothetical protein |  |
| ece:Z0390 | hypothetical protein |  |
| ece:Z0391 | hypothetical protein |  |
| ece:Z0392 | hypothetical protein |  |
| ece:Z0393 | hypothetical protein |  |
| ece:Z0394 | hypothetical protein |  |
| ece:Z0395 | hypothetical protein; K07357 type 1 fimbriae regulatory protein FimB |  |
| ece:Z0397 | hypothetical protein |  |
| ece:Z0398 | betA; choline dehydrogenase (EC:1.1.99.1); K00108 choline dehydrogenase [EC:1.1.99.1] | ec:1.1.99.1 |
| ece:Z0399 | betB; betaine aldehyde dehydrogenase (EC:1.2.1.8); K00130 betaine-aldehyde dehydrogenase [EC:1.2.1.8] | ec:1.2.1.8 |
| ece:Z0400 | betI; transcriptional regulator BetI; K02167 TetR/AcrR family transcriptional regulator, transcriptional repressor of bet genes |  |
| ece:Z0401 | betT; choline transport protein BetT; K02168 choline/glycine/proline betaine transport protein |  |
| ece:Z0402 | beta-barrel outer membrane protein; K12678 autotransporter family porin |  |
| ece:Z0403 | yahA; hypothetical protein; K13244 c-di-GMP-specific phosphodiesterase [EC:3.1.4.52] | ec:3.1.4.52 |
| ece:Z0404 | hypothetical protein |  |
| ece:Z0405 | hypothetical protein |  |
| ece:Z0406 | hypothetical protein |  |
| ece:Z0407 | yahD; transcription factor; K06867 |  |
| ece:Z0408 | yahE; hypothetical protein |  |
| ece:Z0409 | yahF; oxidoreductase subunit |  |
| ece:Z0410 | yahG; hypothetical protein |  |

  
**Neighborhood Representations for "ecf:ECH74115\_0375"**  

| ID | Annotation | EC number |
| --- | --- | --- |
| ecf:ECH74115\_0365 | pseudogene |  |
| ecf:ECH74115\_0366 | pseudogene |  |
| ecf:ECH74115\_0367 | hypothetical protein |  |
| ecf:ECH74115\_0368 | pseudogene |  |
| ecf:ECH74115\_0369 | hypothetical protein |  |
| ecf:ECH74115\_0370 | pseudogene |  |
| ecf:ECH74115\_0371 | pseudogene |  |
| ecf:ECH74115\_0372 | hypothetical protein |  |
| ecf:ECH74115\_0373 | betA; choline dehydrogenase (EC:1.1.99.1); K00108 choline dehydrogenase [EC:1.1.99.1] | ec:1.1.99.1 |
| ecf:ECH74115\_0374 | betB; betaine aldehyde dehydrogenase (EC:1.2.1.8); K00130 betaine-aldehyde dehydrogenase [EC:1.2.1.8] | ec:1.2.1.8 |
| ecf:ECH74115\_0375 | betI; transcriptional regulator BetI; K02167 TetR/AcrR family transcriptional regulator, transcriptional repressor of bet genes |  |
| ecf:ECH74115\_0376 | betT; choline transport protein BetT; K02168 choline/glycine/proline betaine transport protein |  |
| ecf:ECH74115\_0377 | outer membrane autotransporter; K12678 autotransporter family porin |  |
| ecf:ECH74115\_0378 | LuxR-family transcriptional regulator/cyclic diguanylate phosphodiesterase (EAL) domain-containing protein; K13244 c-di-GMP-specific phosphodiesterase [EC:3.1.4.52] | ec:3.1.4.52 |
| ecf:ECH74115\_0379 | pseudogene |  |
| ecf:ECH74115\_0380 | pseudogene |  |
| ecf:ECH74115\_0381 | pseudogene |  |
| ecf:ECH74115\_0382 | ankyrin; K06867 |  |
| ecf:ECH74115\_0383 | hypothetical protein |  |
| ecf:ECH74115\_0384 | FdrA protein |  |
| ecf:ECH74115\_0385 | hypothetical protein |  |

  
**Neighborhood Representations for "ecg:E2348C\_0275"**  

| ID | Annotation | EC number |
| --- | --- | --- |
| ecg:E2348C\_0265 | ykgI; hypothetical protein |  |
| ecg:E2348C\_0266 | ykgC; pyridine nucleotide-disulfide oxidoreductase |  |
| ecg:E2348C\_0267 | ykgD; DNA-binding transcriptional regulator |  |
| ecg:E2348C\_0268 | ykgE; oxidoreductase |  |
| ecg:E2348C\_0269 | ykgF; amino acid dehydrogenase with NAD(P)-binding domain and ferridoxin-like domain |  |
| ecg:E2348C\_0270 | ykgG; transporter; K00782 hypothetical protein |  |
| ecg:E2348C\_0271 | hypothetical protein |  |
| ecg:E2348C\_0272 | ykgH; pseudogene |  |
| ecg:E2348C\_0273 | betA; choline dehydrogenase; K00108 choline dehydrogenase [EC:1.1.99.1] | ec:1.1.99.1 |
| ecg:E2348C\_0274 | betB; betaine aldehyde dehydrogenase; K00130 betaine-aldehyde dehydrogenase [EC:1.2.1.8] | ec:1.2.1.8 |
| ecg:E2348C\_0275 | betI; transcriptional regulator BetI; K02167 TetR/AcrR family transcriptional regulator, transcriptional repressor of bet genes |  |
| ecg:E2348C\_0276 | betT; choline transport protein BetT; K02168 choline/glycine/proline betaine transport protein |  |
| ecg:E2348C\_0277 | yahA; DNA-binding transcriptional regulator; K13244 c-di-GMP-specific phosphodiesterase [EC:3.1.4.52] | ec:3.1.4.52 |
| ecg:E2348C\_0278 | yahB; DNA-binding transcriptional regulator |  |
| ecg:E2348C\_0279 | yahC; hypothetical protein |  |
| ecg:E2348C\_0280 | yahD; transcriptional regulator with ankyrin domain; K06867 |  |
| ecg:E2348C\_0281 | yahE; hypothetical protein |  |
| ecg:E2348C\_0282 | yahF; acyl-CoA synthetase with NAD(P)-binding domain and succinyl-CoA synthetase domain |  |
| ecg:E2348C\_0283 | yahG; hypothetical protein |  |
| ecg:E2348C\_0284 | yahH; hypothetical protein |  |
| ecg:E2348C\_0285 | yahI; carbamate kinase; K00926 carbamate kinase [EC:2.7.2.2] | ec:2.7.2.2 |

  
**Neighborhood Representations for "ecj:Y75\_p0303"**  

| ID | Annotation | EC number |
| --- | --- | --- |
| ecj:Y75\_p0293 | ykgB; inner membrane protein |  |
| ecj:Y75\_p0294 | ykgI; hypothetical protein |  |
| ecj:Y75\_p0295 | ykgC; oxidoreductase with FAD/NAD(P)-binding domain and dimerization domain |  |
| ecj:Y75\_p0296 | ykgD; DNA-binding transcriptional regulator |  |
| ecj:Y75\_p0297 | ykgE; oxidoreductase |  |
| ecj:Y75\_p0298 | ykgF; amino acid dehydrogenase |  |
| ecj:Y75\_p0299 | ykgG; transporter; K00782 hypothetical protein |  |
| ecj:Y75\_p0300 | ykgH; inner membrane protein |  |
| ecj:Y75\_p0301 | betA; choline dehydrogenase; K00108 choline dehydrogenase [EC:1.1.99.1] | ec:1.1.99.1 |
| ecj:Y75\_p0302 | betB; betaine aldehyde dehydrogenase; K00130 betaine-aldehyde dehydrogenase [EC:1.2.1.8] | ec:1.2.1.8 |
| ecj:Y75\_p0303 | betI; DNA-binding transcriptional repressor; K02167 TetR/AcrR family transcriptional regulator, transcriptional repressor of bet genes |  |
| ecj:Y75\_p0304 | betT; choline transporter of high affinity; K02168 choline/glycine/proline betaine transport protein |  |
| ecj:Y75\_p0305 | yahA; DNA-binding transcriptional regulator; K13244 c-di-GMP-specific phosphodiesterase [EC:3.1.4.52] | ec:3.1.4.52 |
| ecj:Y75\_p0306 | yahB; DNA-binding transcriptional regulator |  |
| ecj:Y75\_p0307 | yahC; inner membrane protein |  |
| ecj:Y75\_p0308 | yahD; transcriptional regulator with ankyrin domain; K06867 |  |
| ecj:Y75\_p0309 | yahE; hypothetical protein |  |
| ecj:Y75\_p0310 | yahF; acyl-CoA synthetase with NAD(P)-binding domain and succinyl-CoA synthetase domain |  |
| ecj:Y75\_p0311 | yahG; hypothetical protein |  |
| ecj:Y75\_p0312 | yahH; hypothetical protein |  |
| ecj:Y75\_p0313 | yahI; carbamate kinase-like protein; K00926 carbamate kinase [EC:2.7.2.2] | ec:2.7.2.2 |

  
**Neighborhood Representations for "ecl:EcolC\_3310"**  

| ID | Annotation | EC number |
| --- | --- | --- |
| ecl:EcolC\_3300 | putative carbamate kinase (EC:2.7.2.2); K00926 carbamate kinase [EC:2.7.2.2] | ec:2.7.2.2 |
| ecl:EcolC\_3301 | hypothetical protein |  |
| ecl:EcolC\_3302 | hypothetical protein |  |
| ecl:EcolC\_3303 | FdrA family protein |  |
| ecl:EcolC\_3304 | hypothetical protein |  |
| ecl:EcolC\_3305 | ankyrin; K06867 |  |
| ecl:EcolC\_3306 | hypothetical protein |  |
| ecl:EcolC\_3307 | LysR family transcriptional regulator |  |
| ecl:EcolC\_3308 | diguanylate phosphodiesterase; K13244 c-di-GMP-specific phosphodiesterase [EC:3.1.4.52] | ec:3.1.4.52 |
| ecl:EcolC\_3309 | choline transport protein BetT; K02168 choline/glycine/proline betaine transport protein |  |
| ecl:EcolC\_3310 | transcriptional regulator BetI; K02167 TetR/AcrR family transcriptional regulator, transcriptional repressor of bet genes |  |
| ecl:EcolC\_3311 | betaine aldehyde dehydrogenase; K00130 betaine-aldehyde dehydrogenase [EC:1.2.1.8] | ec:1.2.1.8 |
| ecl:EcolC\_3312 | choline dehydrogenase; K00108 choline dehydrogenase [EC:1.1.99.1] | ec:1.1.99.1 |
| ecl:EcolC\_3313 | hypothetical protein |  |
| ecl:EcolC\_3314 | hypothetical protein; K00782 hypothetical protein |  |
| ecl:EcolC\_3315 | iron-sulfur cluster binding protein |  |
| ecl:EcolC\_3316 | hypothetical protein |  |
| ecl:EcolC\_3317 | AraC family transcriptional regulator |  |
| ecl:EcolC\_3318 | pyridine nucleotide-disulfide oxidoreductase |  |
| ecl:EcolC\_3319 | hypothetical protein |  |
| ecl:EcolC\_3320 | pseudogene |  |

  
**Neighborhood Representations for "eco:b0313"**  

| ID | Annotation | EC number |
| --- | --- | --- |
| eco:b0301 | ykgB; inner membrane protein, DUF417 family |  |
| eco:b0303 | ykgI; predicted protein |  |
| eco:b0304 | ykgC; predicted pyridine nucleotide-disulfide oxidoreductase |  |
| eco:b0305 | ykgD; predicted DNA-binding transcriptional regulator |  |
| eco:b0306 | ykgE; predicted oxidoreductase |  |
| eco:b0307 | ykgF; predicted electron transport protein with ferridoxin-like domai |  |
| eco:b0308 | ykgG; predicted transporter; K00782 hypothetical protein |  |
| eco:b0310 | ykgH; predicted inner membrane protein |  |
| eco:b0311 | betA; choline dehydrogenase, a flavoprotein (EC:1.1.99.1); K00108 choline dehydrogenase [EC:1.1.99.1] | ec:1.1.99.1 |
| eco:b0312 | betB; betaine aldehyde dehydrogenase, NAD-dependent (EC:1.2.1.8); K00130 betaine-aldehyde dehydrogenase [EC:1.2.1.8] | ec:1.2.1.8 |
| eco:b0313 | betI; DNA-binding transcriptional repressor; K02167 TetR/AcrR family transcriptional regulator, transcriptional repressor of bet genes |  |
| eco:b0314 | betT; choline transporter of high affinity; K02168 choline/glycine/proline betaine transport protein |  |
| eco:b0315 | yahA; c-di-GMP-specific phosphodiesterase; K13244 c-di-GMP-specific phosphodiesterase [EC:3.1.4.52] | ec:3.1.4.52 |
| eco:b0316 | yahB; predicted DNA-bindng transcriptional regulator |  |
| eco:b0317 | yahC; predicted inner membrane protein |  |
| eco:b0318 | yahD; ankyrin repeat protein; K06867 |  |
| eco:b0319 | yahE; predicted protein |  |
| eco:b0320 | yahF; predicted acyl-CoA synthetase with NAD(P)-binding domain and succinyl-CoA synthetase domain |  |
| eco:b0321 | yahG; conserved protein |  |
| eco:b0323 | yahI; carbamate kinase-like protein; K00926 carbamate kinase [EC:2.7.2.2] | ec:2.7.2.2 |
| eco:b0324 | yahJ; predicted deaminase with metallo-dependent hydrolase domain |  |

  
**Neighborhood Representations for "ecs:ECs0359"**  

| ID | Annotation | EC number |
| --- | --- | --- |
| ecs:ECs0347 | hypothetical protein |  |
| ecs:ECs0348 | hypothetical protein |  |
| ecs:ECs0349 | hypothetical protein |  |
| ecs:ECs0350 | adhesin |  |
| ecs:ECs0351 | hypothetical protein |  |
| ecs:ECs0352 | hypothetical protein |  |
| ecs:ECs0353 | hypothetical protein |  |
| ecs:ECs5376 | hypothetical protein |  |
| ecs:ECs0357 | choline dehydrogenase (EC:1.1.99.1); K00108 choline dehydrogenase [EC:1.1.99.1] | ec:1.1.99.1 |
| ecs:ECs0358 | betaine aldehyde dehydrogenase (EC:1.2.1.8); K00130 betaine-aldehyde dehydrogenase [EC:1.2.1.8] | ec:1.2.1.8 |
| ecs:ECs0359 | transcriptional regulator BetI; K02167 TetR/AcrR family transcriptional regulator, transcriptional repressor of bet genes |  |
| ecs:ECs0360 | choline transport protein BetT; K02168 choline/glycine/proline betaine transport protein |  |
| ecs:ECs0362 | AidA-I adhesin-like protein; K12678 autotransporter family porin |  |
| ecs:ECs0363 | hypothetical protein; K13244 c-di-GMP-specific phosphodiesterase [EC:3.1.4.52] | ec:3.1.4.52 |
| ecs:ECs0366 | hypothetical protein |  |
| ecs:ECs0367 | transcription factor; K06867 |  |
| ecs:ECs0368 | hypothetical protein |  |
| ecs:ECs0369 | oxidoreductase subunit |  |
| ecs:ECs0370 | hypothetical protein |  |
| ecs:ECs0371 | hypothetical protein |  |
| ecs:ECs0372 | carbamate kinase; K00926 carbamate kinase [EC:2.7.2.2] | ec:2.7.2.2 |

  
**Neighborhood Representations for "eok:G2583\_0417"**  

| ID | Annotation | EC number |
| --- | --- | --- |
| eok:G2583\_0407 | ykgG; hypothetical protein; K00782 hypothetical protein |  |
| eok:G2583\_0408 | hypothetical protein |  |
| eok:G2583\_0409 | ykgH; pseudogene |  |
| eok:G2583\_0410 | autotransporter |  |
| eok:G2583\_0411 | hypothetical protein |  |
| eok:G2583\_0412 | hypothetical protein |  |
| eok:G2583\_0413 | fimX; pseudogene |  |
| eok:G2583\_0414 | hypothetical protein |  |
| eok:G2583\_0415 | betA; Choline dehydrogenase; K00108 choline dehydrogenase [EC:1.1.99.1] | ec:1.1.99.1 |
| eok:G2583\_0416 | betB; Betaine aldehyde dehydrogenase; K00130 betaine-aldehyde dehydrogenase [EC:1.2.1.8] | ec:1.2.1.8 |
| eok:G2583\_0417 | betI; transcriptional regulator; K02167 TetR/AcrR family transcriptional regulator, transcriptional repressor of bet genes |  |
| eok:G2583\_0418 | betT; High-affinity choline transport protein; K02168 choline/glycine/proline betaine transport protein |  |
| eok:G2583\_0419 | aidA; AidA-I adhesin-like protein; K12678 autotransporter family porin |  |
| eok:G2583\_0420 | yahA; LuxR family transcriptional regulator; K13244 c-di-GMP-specific phosphodiesterase [EC:3.1.4.52] | ec:3.1.4.52 |
| eok:G2583\_0421 | yahB; pseudogene |  |
| eok:G2583\_0422 | yahC; pseudogene |  |
| eok:G2583\_0423 | yahD; Ankyrin repeat protein; K06867 |  |
| eok:G2583\_0424 | yahE; hypothetical protein |  |
| eok:G2583\_0425 | yahF; bacterial FdrA protein |  |
| eok:G2583\_0426 | yahG; hypothetical protein |  |
| eok:G2583\_0427 | yahH; hypothetical protein |  |

  
**Neighborhood Representations for "etw:ECSP\_0368"**  

| ID | Annotation | EC number |
| --- | --- | --- |
| etw:ECSP\_0358 | pseudogene |  |
| etw:ECSP\_0359 | autotransporter |  |
| etw:ECSP\_0360 | hypothetical protein |  |
| etw:ECSP\_0361 | hypothetical protein |  |
| etw:ECSP\_0362 | hypothetical protein |  |
| etw:ECSP\_0363 | pseudogene |  |
| etw:ECSP\_0364 | hypothetical protein |  |
| etw:ECSP\_0365 | hypothetical protein |  |
| etw:ECSP\_0366 | betA; choline dehydrogenase; K00108 choline dehydrogenase [EC:1.1.99.1] | ec:1.1.99.1 |
| etw:ECSP\_0367 | betB; betaine aldehyde dehydrogenase; K00130 betaine-aldehyde dehydrogenase [EC:1.2.1.8] | ec:1.2.1.8 |
| etw:ECSP\_0368 | betI; transcriptional regulator BetI; K02167 TetR/AcrR family transcriptional regulator, transcriptional repressor of bet genes |  |
| etw:ECSP\_0369 | betT; choline transport protein BetT; K02168 choline/glycine/proline betaine transport protein |  |
| etw:ECSP\_0370 | beta-barrel outer membrane protein; K12678 autotransporter family porin |  |
| etw:ECSP\_0371 | yahA; DNA-binding transcriptional regulator; K13244 c-di-GMP-specific phosphodiesterase [EC:3.1.4.52] | ec:3.1.4.52 |
| etw:ECSP\_0372 | pseudogene |  |
| etw:ECSP\_0373 | hypothetical protein |  |
| etw:ECSP\_0374 | yahD; transcriptional regulator with ankyrin domain; K06867 |  |
| etw:ECSP\_0375 | yahE; hypothetical protein |  |
| etw:ECSP\_0376 | yahF; acyl-CoA synthetase with NAD(P)-binding domain and succinyl-CoA synthetase domain |  |
| etw:ECSP\_0377 | yahG; hypothetical protein |  |
| etw:ECSP\_0378 | hypothetical protein |  |

  
**Neighborhood Representations for "ecp:ECP\_0388"**  

| ID | Annotation | EC number |
| --- | --- | --- |
| ecp:ECP\_0378 | hypothetical protein |  |
| ecp:ECP\_0379 | autotransporter |  |
| ecp:ECP\_0380 | hypothetical protein |  |
| ecp:ECP\_0381 | hypothetical protein |  |
| ecp:ECP\_0382 | phage integrase; K07357 type 1 fimbriae regulatory protein FimB |  |
| ecp:ECP\_0383 | hypothetical protein |  |
| ecp:ECP\_0384 | hypothetical protein |  |
| ecp:ECP\_0385 | hypothetical protein |  |
| ecp:ECP\_0386 | choline dehydrogenase (EC:1.1.99.1); K00108 choline dehydrogenase [EC:1.1.99.1] | ec:1.1.99.1 |
| ecp:ECP\_0387 | betaine aldehyde dehydrogenase (EC:1.2.1.8); K00130 betaine-aldehyde dehydrogenase [EC:1.2.1.8] | ec:1.2.1.8 |
| ecp:ECP\_0388 | transcriptional regulator BetI; K02167 TetR/AcrR family transcriptional regulator, transcriptional repressor of bet genes |  |
| ecp:ECP\_0389 | hypothetical protein |  |
| ecp:ECP\_0390 | choline transport protein BetT; K02168 choline/glycine/proline betaine transport protein |  |
| ecp:ECP\_0391 | hypothetical protein; K13244 c-di-GMP-specific phosphodiesterase [EC:3.1.4.52] | ec:3.1.4.52 |
| ecp:ECP\_0392 | transcriptional regulator YahB |  |
| ecp:ECP\_0393 | hypothetical protein |  |
| ecp:ECP\_0394 | hypothetical protein |  |
| ecp:ECP\_0395 | ankyrin repeat-containing protein; K06867 |  |
| ecp:ECP\_0396 | hypothetical protein |  |
| ecp:ECP\_0397 | YahF/FdrA-like protein |  |
| ecp:ECP\_0398 | hypothetical protein |  |

  
**Neighborhood Representations for "eum:ECUMN\_0351"**  

| ID | Annotation | EC number |
| --- | --- | --- |
| eum:ECUMN\_0341 | ykgI; hypothetical protein |  |
| eum:ECUMN\_0342 | ykgC; pyridine nucleotide-disulfide oxidoreductase |  |
| eum:ECUMN\_0343 | ykgD; putative AraC-type DNA-binding transcriptional regulator |  |
| eum:ECUMN\_0344 | ykgE; putative hydroxyacid oxidoreductase (Fe-S centre) |  |
| eum:ECUMN\_0345 | ykgF; putative oxidoreductase |  |
| eum:ECUMN\_0346 | ykgG; hypothetical protein; K00782 hypothetical protein |  |
| eum:ECUMN\_0347 | hypothetical protein |  |
| eum:ECUMN\_0348 | ykgH; hypothetical protein |  |
| eum:ECUMN\_0349 | betA; choline dehydrogenase (EC:1.1.99.1); K00108 choline dehydrogenase [EC:1.1.99.1] | ec:1.1.99.1 |
| eum:ECUMN\_0350 | betB; betaine aldehyde dehydrogenase (EC:1.2.1.8); K00130 betaine-aldehyde dehydrogenase [EC:1.2.1.8] | ec:1.2.1.8 |
| eum:ECUMN\_0351 | betI; transcriptional regulator BetI; K02167 TetR/AcrR family transcriptional regulator, transcriptional repressor of bet genes |  |
| eum:ECUMN\_0352 | betT; choline transport protein BetT; K02168 choline/glycine/proline betaine transport protein |  |
| eum:ECUMN\_0353 | hypothetical protein |  |
| eum:ECUMN\_0354 | yahA; cyclic di-GMP phosphodiesterase; K13244 c-di-GMP-specific phosphodiesterase [EC:3.1.4.52] | ec:3.1.4.52 |
| eum:ECUMN\_0355 | yahB; putative DNA-binding transcriptional regulator |  |
| eum:ECUMN\_0356 | yahC; hypothetical protein |  |
| eum:ECUMN\_0357 | yahD; hypothetical protein; K06867 |  |
| eum:ECUMN\_0358 | yahE; hypothetical protein |  |
| eum:ECUMN\_0359 | yahF; hypothetical protein |  |
| eum:ECUMN\_0360 | yahG; hypothetical protein |  |
| eum:ECUMN\_0361 | yahI; putative carbamate kinase; K00926 carbamate kinase [EC:2.7.2.2] | ec:2.7.2.2 |

  
**Neighborhood Representations for "ect:ECIAI39\_0373"**  

| ID | Annotation | EC number |
| --- | --- | --- |
| ect:ECIAI39\_0363 | yahJ; putative deaminase |  |
| ect:ECIAI39\_0364 | yahI; putative carbamate kinase; K00926 carbamate kinase [EC:2.7.2.2] | ec:2.7.2.2 |
| ect:ECIAI39\_0365 | yahG; hypothetical protein |  |
| ect:ECIAI39\_0366 | yahF; putative enzyme with acyl-CoA domain |  |
| ect:ECIAI39\_0367 | yahE; hypothetical protein |  |
| ect:ECIAI39\_0368 | yahD; hypothetical protein; K06867 |  |
| ect:ECIAI39\_0369 | yahC; hypothetical protein |  |
| ect:ECIAI39\_0370 | yahB; putative DNA-binding transcriptional regulator |  |
| ect:ECIAI39\_0371 | yahA; cyclic di-GMP phosphodiesterase; K13244 c-di-GMP-specific phosphodiesterase [EC:3.1.4.52] | ec:3.1.4.52 |
| ect:ECIAI39\_0372 | betT; choline transport protein BetT; K02168 choline/glycine/proline betaine transport protein |  |
| ect:ECIAI39\_0373 | betI; transcriptional regulator BetI; K02167 TetR/AcrR family transcriptional regulator, transcriptional repressor of bet genes |  |
| ect:ECIAI39\_0374 | betB; betaine aldehyde dehydrogenase (EC:1.2.1.8); K00130 betaine-aldehyde dehydrogenase [EC:1.2.1.8] | ec:1.2.1.8 |
| ect:ECIAI39\_0375 | betA; choline dehydrogenase (EC:1.1.99.1); K00108 choline dehydrogenase [EC:1.1.99.1] | ec:1.1.99.1 |
| ect:ECIAI39\_0376 | hypothetical protein |  |
| ect:ECIAI39\_0377 | ykgG; hypothetical protein; K00782 hypothetical protein |  |
| ect:ECIAI39\_0378 | ykgF; putative oxidoreductase |  |
| ect:ECIAI39\_0379 | ykgE; putative hydroxyacid oxidoreductase (Fe-S centre) |  |
| ect:ECIAI39\_0380 | ykgD; putative AraC-type DNA-binding transcriptional regulator |  |
| ect:ECIAI39\_0381 | ykgC; pyridine nucleotide-disulfide oxidoreductase |  |
| ect:ECIAI39\_0382 | ykgI; hypothetical protein |  |
| ect:ECIAI39\_0383 | ykgB; hypothetical protein |  |

  
**Neighborhood Representations for "ebd:ECBD\_3345"**  

| ID | Annotation | EC number |
| --- | --- | --- |
| ebd:ECBD\_3335 | hypothetical protein |  |
| ebd:ECBD\_3336 | hypothetical protein |  |
| ebd:ECBD\_3337 | FdrA family protein |  |
| ebd:ECBD\_3338 | hypothetical protein |  |
| ebd:ECBD\_3339 | ankyrin; K06867 |  |
| ebd:ECBD\_3340 | hypothetical protein |  |
| ebd:ECBD\_3341 | LysR family transcriptional regulator |  |
| ebd:ECBD\_3342 | diguanylate phosphodiesterase; K13244 c-di-GMP-specific phosphodiesterase [EC:3.1.4.52] | ec:3.1.4.52 |
| ebd:ECBD\_3343 | hypothetical protein |  |
| ebd:ECBD\_3344 | choline transporter BetT; K02168 choline/glycine/proline betaine transport protein |  |
| ebd:ECBD\_3345 | transcriptional regulator BetI; K02167 TetR/AcrR family transcriptional regulator, transcriptional repressor of bet genes |  |
| ebd:ECBD\_3346 | betaine aldehyde dehydrogenase; K00130 betaine-aldehyde dehydrogenase [EC:1.2.1.8] | ec:1.2.1.8 |
| ebd:ECBD\_3347 | choline dehydrogenase; K00108 choline dehydrogenase [EC:1.1.99.1] | ec:1.1.99.1 |
| ebd:ECBD\_3348 | hypothetical protein |  |
| ebd:ECBD\_3349 | hypothetical protein; K00782 hypothetical protein |  |
| ebd:ECBD\_3350 | iron-sulfur cluster binding protein |  |
| ebd:ECBD\_3351 | hypothetical protein |  |
| ebd:ECBD\_3352 | hypothetical protein |  |
| ebd:ECBD\_3353 | AraC family transcriptional regulator |  |
| ebd:ECBD\_3354 | pyridine nucleotide-disulfide oxidoreductase |  |
| ebd:ECBD\_3355 | hypothetical protein |  |

  
**Neighborhood Representations for "ebr:ECB\_00269"**  

| ID | Annotation | EC number |
| --- | --- | --- |
| ebr:ECB\_00259 | ykgI; hypothetical protein |  |
| ebr:ECB\_00260 | ykgC; pyridine nucleotide-disulfide oxidoreductase |  |
| ebr:ECB\_00261 | ykgD; putative DNA-binding transcriptional regulator |  |
| ebr:ECB\_00262 | ykgE; putative oxidoreductase |  |
| ebr:ECB\_00263 | ykgF; putative amino acid dehydrogenase with NAD(P)-binding domain and ferridoxin-like domain |  |
| ebr:ECB\_00264 | ykgG; putative transporter; K00782 hypothetical protein |  |
| ebr:ECB\_00265 | hypothetical protein |  |
| ebr:ECB\_00266 | ykgH; putative inner membrane protein |  |
| ebr:ECB\_00267 | betA; choline dehydrogenase (EC:1.1.99.1); K00108 choline dehydrogenase [EC:1.1.99.1] | ec:1.1.99.1 |
| ebr:ECB\_00268 | betB; betaine aldehyde dehydrogenase (EC:1.2.1.8); K00130 betaine-aldehyde dehydrogenase [EC:1.2.1.8] | ec:1.2.1.8 |
| ebr:ECB\_00269 | betI; transcriptional regulator BetI; K02167 TetR/AcrR family transcriptional regulator, transcriptional repressor of bet genes |  |
| ebr:ECB\_00270 | betT; choline transport protein BetT; K02168 choline/glycine/proline betaine transport protein |  |
| ebr:ECB\_00271 | yahA; putative DNA-binding transcriptional regulator; K13244 c-di-GMP-specific phosphodiesterase [EC:3.1.4.52] | ec:3.1.4.52 |
| ebr:ECB\_00272 | yahB; putative DNA-binding transcriptional regulator |  |
| ebr:ECB\_00273 | yahC; putative inner membrane protein |  |
| ebr:ECB\_00274 | yahD; putative transcriptional regulator with ankyrin domain; K06867 |  |
| ebr:ECB\_00275 | yahE; hypothetical protein |  |
| ebr:ECB\_00276 | yahF; putative acyl-CoA synthetase with NAD(P)-binding domain and succinyl-CoA synthetase domain |  |
| ebr:ECB\_00277 | yahG; hypothetical protein |  |
| ebr:ECB\_00278 | yahI; putative carbamate kinase; K00926 carbamate kinase [EC:2.7.2.2] | ec:2.7.2.2 |
| ebr:ECB\_00279 | yahJ; putative deaminase |  |

  
**Neighborhood Representations for "kpe:KPK\_3993"**  

| ID | Annotation | EC number |
| --- | --- | --- |
| kpe:KPK\_3983 | lipoprotein |  |
| kpe:KPK\_3984 | sugar ABC transporter ATP-binding protein; K17204 erythritol transport system ATP-binding protein |  |
| kpe:KPK\_3985 | sugar ABC transporter permease; K17203 erythritol transport system permease protein |  |
| kpe:KPK\_3986 | short chain dehydrogenase/reductase family oxidoreductase |  |
| kpe:KPK\_3987 | short chain dehydrogenase |  |
| kpe:KPK\_3988 | glutathione S-transferase |  |
| kpe:KPK\_3990 | LysR family transcriptional regulator; K03566 LysR family transcriptional regulator, glycine cleavage system transcriptional activator |  |
| kpe:KPK\_3989 | transporter small conductance mechanosensitive ion channel (MscS) family; K16053 miniconductance mechanosensitive channel |  |
| kpe:KPK\_3991 | C-lysozyme inhibitor |  |
| kpe:KPK\_3992 | betT; choline transport protein BetT; K02168 choline/glycine/proline betaine transport protein |  |
| kpe:KPK\_3993 | betI; transcriptional regulator BetI; K02167 TetR/AcrR family transcriptional regulator, transcriptional repressor of bet genes |  |
| kpe:KPK\_3994 | betB; betaine aldehyde dehydrogenase; K00130 betaine-aldehyde dehydrogenase [EC:1.2.1.8] | ec:1.2.1.8 |
| kpe:KPK\_3995 | betA; choline dehydrogenase; K00108 choline dehydrogenase [EC:1.1.99.1] | ec:1.1.99.1 |
| kpe:KPK\_3996 | lipoprotein |  |
| kpe:KPK\_3997 | hypothetical protein |  |
| kpe:KPK\_3998 | phenazine biosynthesis protein PhzF family protein |  |
| kpe:KPK\_3999 | hypothetical protein |  |
| kpe:KPK\_4000 | lipoprotein |  |
| kpe:KPK\_4001 | GNAT family acetyltransferase |  |
| kpe:KPK\_4002 | ABC transporter substrate-binding protein; K02035 peptide/nickel transport system substrate-binding protein |  |
| kpe:KPK\_4003 | ABC transporter permease; K02033 peptide/nickel transport system permease protein |  |

  
**Neighborhood Representations for "kpn:KPN\_00586"**  

| ID | Annotation | EC number |
| --- | --- | --- |
| kpn:KPN\_00576 | putative oxidoreductase; K15373 sulfoacetaldehyde reductase [EC:1.1.1.313] | ec:1.1.1.313 |
| kpn:KPN\_00577 | hypothetical protein; K15372 taurine---2-oxoglutarate transaminase [EC:2.6.1.55] | ec:2.6.1.55 |
| kpn:KPN\_00578 | ATPase; K02032 peptide/nickel transport system ATP-binding protein |  |
| kpn:KPN\_00579 | putative ABC transporter; K02031 peptide/nickel transport system ATP-binding protein |  |
| kpn:KPN\_00580 | putative ABC transport system permease; K02034 peptide/nickel transport system permease protein |  |
| kpn:KPN\_00581 | putative ABC transport system permease; K02033 peptide/nickel transport system permease protein |  |
| kpn:KPN\_00582 | putative ABC transport system periplasmic binding protein; K02035 peptide/nickel transport system substrate-binding protein |  |
| kpn:KPN\_00583 | putative GCN5-related N-acetyltransferase |  |
| kpn:KPN\_00584 | choline dehydrogenase; K00108 choline dehydrogenase [EC:1.1.99.1] | ec:1.1.99.1 |
| kpn:KPN\_00585 | betaine aldehyde dehydrogenase; K00130 betaine-aldehyde dehydrogenase [EC:1.2.1.8] | ec:1.2.1.8 |
| kpn:KPN\_00586 | transcriptional regulator BetI; K02167 TetR/AcrR family transcriptional regulator, transcriptional repressor of bet genes |  |
| kpn:KPN\_00587 | choline transport protein BetT; K02168 choline/glycine/proline betaine transport protein |  |
| kpn:KPN\_00588 | putative transport |  |
| kpn:KPN\_00589 | transcriptional regulator LysR; K03566 LysR family transcriptional regulator, glycine cleavage system transcriptional activator |  |
| kpn:KPN\_00590 | putative S-transferase |  |
| kpn:KPN\_00591 | putative carbohydrate kinase; K00864 glycerol kinase [EC:2.7.1.30] | ec:2.7.1.30 |
| kpn:KPN\_00592 | putative L-fucose isomerase, C-terminal |  |
| kpn:KPN\_00593 | putative transketolase C-terminal section; K00615 transketolase [EC:2.2.1.1] | ec:2.2.1.1 |
| kpn:KPN\_00594 | putative transketolase; K00615 transketolase [EC:2.2.1.1] | ec:2.2.1.1 |
| kpn:KPN\_00595 | putative sugar-binding region |  |
| kpn:KPN\_00596 | putative transposase, YhgA-like protein |  |

  
**Neighborhood Representations for "kpu:KP1\_1530"**  

| ID | Annotation | EC number |
| --- | --- | --- |
| kpu:KP1\_1520 | IS150 putative transposase |  |
| kpu:KP1\_1521 | hypothetical protein; K07483 transposase |  |
| kpu:KP1\_1522 | yddO; putative ABC transport system ATP-binding component; K02032 peptide/nickel transport system ATP-binding protein |  |
| kpu:KP1\_1523 | yddP; putative ABC transport system oligopeptide translocator; K02031 peptide/nickel transport system ATP-binding protein |  |
| kpu:KP1\_1524 | yddQ; putative ABC transport system permease component; K02034 peptide/nickel transport system permease protein |  |
| kpu:KP1\_1525 | yddR; putative ABC transport system permease component; K02033 peptide/nickel transport system permease protein |  |
| kpu:KP1\_1526 | putative ABC transport system periplasmic dipeptide binding component; K02035 peptide/nickel transport system substrate-binding protein |  |
| kpu:KP1\_1527 | putative GCN5-related N-acetyltransferase |  |
| kpu:KP1\_1528 | betA; choline dehydrogenase; K00108 choline dehydrogenase [EC:1.1.99.1] | ec:1.1.99.1 |
| kpu:KP1\_1529 | betB; betaine aldehyde dehydrogenase; K00130 betaine-aldehyde dehydrogenase [EC:1.2.1.8] | ec:1.2.1.8 |
| kpu:KP1\_1530 | betI; transcriptional regulator BetI; K02167 TetR/AcrR family transcriptional regulator, transcriptional repressor of bet genes |  |
| kpu:KP1\_1531 | betT; choline transport protein BetT; K02168 choline/glycine/proline betaine transport protein |  |
| kpu:KP1\_1532 | ykfE; C-lysozyme inhibitor |  |
| kpu:KP1\_1533 | putative MscS mechanosensitive ion channel |  |
| kpu:KP1\_1534 | LysR family transcriptional regulator; K03566 LysR family transcriptional regulator, glycine cleavage system transcriptional activator |  |
| kpu:KP1\_1535 | putative S-transferase |  |
| kpu:KP1\_1536 | short chain dehydrogenase |  |
| kpu:KP1\_1537 | short chain dehydrogenase/reductase family oxidoreductase |  |
| kpu:KP1\_1538 | putative monosaccharide-transporting ATPase; K17203 erythritol transport system permease protein |  |
| kpu:KP1\_1539 | putative ribose ABC transport system ATP-binding component; K17204 erythritol transport system ATP-binding protein |  |
| kpu:KP1\_1540 | hypothetical protein |  |

  
**Neighborhood Representations for "kva:Kvar\_3783"**  

| ID | Annotation | EC number |
| --- | --- | --- |
| kva:Kvar\_3773 | periplasmic lipoprotein |  |
| kva:Kvar\_3774 | ABC transporter; K17204 erythritol transport system ATP-binding protein |  |
| kva:Kvar\_3775 | inner-membrane translocator; K17203 erythritol transport system permease protein |  |
| kva:Kvar\_3776 | short-chain dehydrogenase/reductase SDR |  |
| kva:Kvar\_3777 | short-chain dehydrogenase/reductase SDR |  |
| kva:Kvar\_3778 | glutathione S-transferase domain-containing protein |  |
| kva:Kvar\_3779 | LysR family transcriptional regulator; K03566 LysR family transcriptional regulator, glycine cleavage system transcriptional activator |  |
| kva:Kvar\_3780 | mechanosensitive ion channel MscS; K16053 miniconductance mechanosensitive channel |  |
| kva:Kvar\_3781 | lysozyme inhibitor |  |
| kva:Kvar\_3782 | choline/carnitine/betaine transporter; K02168 choline/glycine/proline betaine transport protein |  |
| kva:Kvar\_3783 | TetR family transcriptional regulator; K02167 TetR/AcrR family transcriptional regulator, transcriptional repressor of bet genes |  |
| kva:Kvar\_3784 | betaine aldehyde dehydrogenase; K00130 betaine-aldehyde dehydrogenase [EC:1.2.1.8] | ec:1.2.1.8 |
| kva:Kvar\_3785 | choline dehydrogenase; K00108 choline dehydrogenase [EC:1.1.99.1] | ec:1.1.99.1 |
| kva:Kvar\_3786 | lipoprotein |  |
| kva:Kvar\_3787 | YheO domain-containing protein |  |
| kva:Kvar\_3788 | phenazine biosynthesis protein PhzF family |  |
| kva:Kvar\_3789 | hypothetical protein |  |
| kva:Kvar\_3790 | GCN5-like N-acetyltransferase |  |
| kva:Kvar\_3791 | family 5 extracellular solute-binding protein; K02035 peptide/nickel transport system substrate-binding protein |  |
| kva:Kvar\_3792 | binding-protein-dependent transporters inner membrane component; K02033 peptide/nickel transport system permease protein |  |
| kva:Kvar\_3793 | binding-protein-dependent transporters inner membrane component; K02034 peptide/nickel transport system permease protein |  |

  
**Neighborhood Representations for "enc:ECL\_03121"**  

| ID | Annotation | EC number |
| --- | --- | --- |
| enc:ECL\_03111 | iron-enterobactin transporter membrane protein; K02015 iron complex transport system permease protein |  |
| enc:ECL\_03112 | iron-enterobactin transporter permease; K02015 iron complex transport system permease protein |  |
| enc:ECL\_03113 | iron-enterobactin transporter ATP-binding protein; K02013 iron complex transport system ATP-binding protein [EC:3.6.3.34] | ec:3.6.3.34 |
| enc:ECL\_03114 | entF; enterobactin synthase subunit F; K02364 enterobactin synthetase component F [EC:2.7.7.-] |  |
| enc:ECL\_03115 | MbtH domain-containing protein |  |
| enc:ECL\_03116 | enterobactin/ferric enterobactin esterase; K07214 enterochelin esterase and related enzymes |  |
| enc:ECL\_03117 | iron complex outermembrane recepter protein; K16089 outer membrane receptor for ferrienterochelin and colicins |  |
| enc:ECL\_03118 | entD; enterobactin synthetase component D; K02362 enterobactin synthetase component D [EC:2.7.8.-] |  |
| enc:ECL\_03119 | hypothetical protein |  |
| enc:ECL\_03120 | high-affinity choline transport protein; K02168 choline/glycine/proline betaine transport protein |  |
| enc:ECL\_03121 | transcriptional regulator BetI; K02167 TetR/AcrR family transcriptional regulator, transcriptional repressor of bet genes |  |
| enc:ECL\_03122 | betaine aldehyde dehydrogenase; K00130 betaine-aldehyde dehydrogenase [EC:1.2.1.8] | ec:1.2.1.8 |
| enc:ECL\_03123 | choline dehydrogenase; K00108 choline dehydrogenase [EC:1.1.99.1] | ec:1.1.99.1 |
| enc:ECL\_03124 | cold-shock protein |  |
| enc:ECL\_03125 | acetoin reductase; K03366 (R,R)-butanediol dehydrogenase / diacetyl reductase [EC:1.1.1.4 1.1.1.303] | ec:1.1.1.76 ec:1.1.1.304 |
| enc:ECL\_03126 | acetolactate synthase; K01652 acetolactate synthase I/II/III large subunit [EC:2.2.1.6] | ec:2.2.1.6 |
| enc:ECL\_03127 | alpha-acetolactate decarboxylase; K01575 acetolactate decarboxylase [EC:4.1.1.5] | ec:4.1.1.5 |
| enc:ECL\_03128 | LysR family transcriptional regulator |  |
| enc:ECL\_03129 | hypothetical protein |  |
| enc:ECL\_03130 | hypothetical protein |  |
| enc:ECL\_03131 | putative transposase |  |

  
**Neighborhood Representations for "eam:EAMY\_1713"**  

| ID | Annotation | EC number |
| --- | --- | --- |
| eam:EAMY\_1703 | hypothetical protein |  |
| eam:EAMY\_1704 | ydhH; anhydro-N-acetylmuramic acid kinase; K09001 anhydro-N-acetylmuramic acid kinase [EC:2.7.1.170] | ec:2.7.1.170 |
| eam:EAMY\_1705 | hypothetical protein |  |
| eam:EAMY\_1706 | pdxH; pyridoxinephosphate oxidase; K00275 pyridoxamine 5'-phosphate oxidase [EC:1.4.3.5] | ec:1.4.3.5 |
| eam:EAMY\_1707 | tyrS; tyrosyl-tRNA synthetase; K01866 tyrosyl-tRNA synthetase [EC:6.1.1.1] | ec:6.1.1.1 |
| eam:EAMY\_1708 | pdxY1; pyridoxal kinase 2/pyridoxine kinase; K00868 pyridoxine kinase [EC:2.7.1.35] | ec:2.7.1.35 |
| eam:EAMY\_1709 | pdxY3; pyridoxal kinase 2/pyridoxine kinase |  |
| eam:EAMY\_1710 | gst; glutathione S-transferase; K00799 glutathione S-transferase [EC:2.5.1.18] | ec:2.5.1.18 |
| eam:EAMY\_1711 | hypothetical protein |  |
| eam:EAMY\_1712 | ydgR; tripeptide transporter permease; K03305 proton-dependent oligopeptide transporter, POT family |  |
| eam:EAMY\_1713 | betI; HTH-type transcriptional regulator BetI; K02167 TetR/AcrR family transcriptional regulator, transcriptional repressor of bet genes |  |
| eam:EAMY\_1714 | betB; betaine aldehyde dehydrogenase; K00130 betaine-aldehyde dehydrogenase [EC:1.2.1.8] | ec:1.2.1.8 |
| eam:EAMY\_1715 | betA; choline dehydrogenase; K00108 choline dehydrogenase [EC:1.1.99.1] | ec:1.1.99.1 |
| eam:EAMY\_1716 | nth; endonuclease III; K10773 endonuclease III [EC:4.2.99.18] | ec:4.2.99.18 |
| eam:EAMY\_1717 | rnfE; NADH:ubiquinone oxidoreductase subunit RnfE; K03613 electron transport complex protein RnfE |  |
| eam:EAMY\_1718 | rfnG; electron transport complex protein RnfG; K03612 electron transport complex protein RnfG |  |
| eam:EAMY\_1719 | rnfD; electron transport complex protein RnfD; K03614 electron transport complex protein RnfD |  |
| eam:EAMY\_1720 | rnfC; electron transport complex protein RnfC; K03615 electron transport complex protein RnfC |  |
| eam:EAMY\_1721 | rnfB; electron transport complex protein RnfB; K03616 electron transport complex protein RnfB |  |
| eam:EAMY\_1722 | rnfA; electron transport complex protein RnfA; K03617 electron transport complex protein RnfA |  |
| eam:EAMY\_1723 | ydgK; hypothetical protein |  |

  
**Neighborhood Representations for "eay:EAM\_1685"**  

| ID | Annotation | EC number |
| --- | --- | --- |
| eay:EAM\_1675 | transposase (partial) |  |
| eay:EAM\_1676 | hypothetical protein |  |
| eay:EAM\_1677 | slyA; MarR family transcriptional regulator; K06075 MarR family transcriptional regulator, transcriptional regulator for hemolysin |  |
| eay:EAM\_1678 | slyB; outer membrane lipoprotein; K06077 outer membrane lipoprotein SlyB |  |
| eay:EAM\_1679 | hypothetical protein; K09001 anhydro-N-acetylmuramic acid kinase [EC:2.7.1.170] | ec:2.7.1.170 |
| eay:EAM\_1680 | pdxH; pyridoxamine 5'-phosphate oxidase; K00275 pyridoxamine 5'-phosphate oxidase [EC:1.4.3.5] | ec:1.4.3.5 |
| eay:EAM\_1681 | tyrS; tyrosyl-tRNA synthetase; K01866 tyrosyl-tRNA synthetase [EC:6.1.1.1] | ec:6.1.1.1 |
| eay:EAM\_1682 | pdxY; pseudogene |  |
| eay:EAM\_1683 | gst; glutathione S-transferase; K00799 glutathione S-transferase [EC:2.5.1.18] | ec:2.5.1.18 |
| eay:EAM\_1684 | tppB; tripeptide permease; K03305 proton-dependent oligopeptide transporter, POT family |  |
| eay:EAM\_1685 | betI; TetR family transcriptional regulator; K02167 TetR/AcrR family transcriptional regulator, transcriptional repressor of bet genes |  |
| eay:EAM\_1686 | betB; betaine aldehyde dehydrogenase; K00130 betaine-aldehyde dehydrogenase [EC:1.2.1.8] | ec:1.2.1.8 |
| eay:EAM\_1687 | betA; choline dehydrogenase; K00108 choline dehydrogenase [EC:1.1.99.1] | ec:1.1.99.1 |
| eay:EAM\_1688 | nth; endonuclease III; K10773 endonuclease III [EC:4.2.99.18] | ec:4.2.99.18 |
| eay:EAM\_1689 | rnfE; electron transport complex protein; K03613 electron transport complex protein RnfE |  |
| eay:EAM\_1690 | rnfG; electron transport complex protein; K03612 electron transport complex protein RnfG |  |
| eay:EAM\_1691 | rnfD; electron transport complex protein; K03614 electron transport complex protein RnfD |  |
| eay:EAM\_1692 | rnfC; electron transport complex protein; K03615 electron transport complex protein RnfC |  |
| eay:EAM\_1693 | rnfB; electron transport complex protein; K03616 electron transport complex protein RnfB |  |
| eay:EAM\_1694 | rnfA; electron transport complex protein; K03617 electron transport complex protein RnfA |  |
| eay:EAM\_1695 | membrane protein |  |

  
**Neighborhood Representations for "eci:UTI89\_C0342"**  

| ID | Annotation | EC number |
| --- | --- | --- |
| eci:UTI89\_C0332 | hypothetical protein |  |
| eci:UTI89\_C0333 | ykgH; hypothetical protein |  |
| eci:UTI89\_C0334 | hypothetical protein |  |
| eci:UTI89\_C0335 | hypothetical protein |  |
| eci:UTI89\_C0336 | hypothetical protein |  |
| eci:UTI89\_C0337 | hypothetical protein |  |
| eci:UTI89\_C0338 | fimX; type 1 fimbriae regulatory protein FimX; K07357 type 1 fimbriae regulatory protein FimB |  |
| eci:UTI89\_C0339 | hypothetical protein |  |
| eci:UTI89\_C0340 | betA; choline dehydrogenase (EC:1.1.99.1); K00108 choline dehydrogenase [EC:1.1.99.1] | ec:1.1.99.1 |
| eci:UTI89\_C0341 | betB; betaine aldehyde dehydrogenase (EC:1.2.1.8); K00130 betaine-aldehyde dehydrogenase [EC:1.2.1.8] | ec:1.2.1.8 |
| eci:UTI89\_C0342 | betI; transcriptional regulator BetI; K02167 TetR/AcrR family transcriptional regulator, transcriptional repressor of bet genes |  |
| eci:UTI89\_C0343 | betT; choline transport protein BetT; K02168 choline/glycine/proline betaine transport protein |  |
| eci:UTI89\_C0344 | yahA; hypothetical protein; K13244 c-di-GMP-specific phosphodiesterase [EC:3.1.4.52] | ec:3.1.4.52 |
| eci:UTI89\_C0345 | yahB; transcriptional regulator YahB |  |
| eci:UTI89\_C0346 | yahC; hypothetical protein |  |
| eci:UTI89\_C0347 | hypothetical protein |  |
| eci:UTI89\_C0348 | yahD; transcription factor (EC:3.1.26.-); K06867 |  |
| eci:UTI89\_C0349 | yahE; hypothetical protein |  |
| eci:UTI89\_C0350 | yahF; hypothetical protein |  |
| eci:UTI89\_C0351 | yahG; hypothetical protein |  |
| eci:UTI89\_C0352 | yahI; carbamate kinase (EC:2.7.2.2); K00926 carbamate kinase [EC:2.7.2.2] | ec:2.7.2.2 |

  
**Neighborhood Representations for "ecv:APECO1\_1677"**  

| ID | Annotation | EC number |
| --- | --- | --- |
| ecv:APECO1\_1687 | ykgG; hypothetical protein |  |
| ecv:APECO1\_1686 | ykgG; hypothetical protein; K00782 hypothetical protein |  |
| ecv:APECO1\_1685 | ykgH; hypothetical protein |  |
| ecv:APECO1\_1684 | hypothetical protein |  |
| ecv:APECO1\_1683 | hypothetical protein |  |
| ecv:APECO1\_1682 | hypothetical protein |  |
| ecv:APECO1\_1681 | hypothetical protein; K07357 type 1 fimbriae regulatory protein FimB |  |
| ecv:APECO1\_1680 | hypothetical protein |  |
| ecv:APECO1\_1679 | betA; choline dehydrogenase (EC:1.1.99.1); K00108 choline dehydrogenase [EC:1.1.99.1] | ec:1.1.99.1 |
| ecv:APECO1\_1678 | betB; betaine aldehyde dehydrogenase (EC:1.2.1.8); K00130 betaine-aldehyde dehydrogenase [EC:1.2.1.8] | ec:1.2.1.8 |
| ecv:APECO1\_1677 | betI; transcriptional regulator BetI; K02167 TetR/AcrR family transcriptional regulator, transcriptional repressor of bet genes |  |
| ecv:APECO1\_1676 | betT; choline transport protein BetT; K02168 choline/glycine/proline betaine transport protein |  |
| ecv:APECO1\_1675 | yahA; DNA-binding transcriptional regulator; K13244 c-di-GMP-specific phosphodiesterase [EC:3.1.4.52] | ec:3.1.4.52 |
| ecv:APECO1\_1674 | yahB; DNA-binding transcriptional regulator |  |
| ecv:APECO1\_1673 | yahC; hypothetical protein |  |
| ecv:APECO1\_1672 | yahD; transcription factor; K06867 |  |
| ecv:APECO1\_1671 | yahE; hypothetical protein |  |
| ecv:APECO1\_1670 | yahF; acyl-CoA synthetase |  |
| ecv:APECO1\_1669 | hypothetical protein |  |
| ecv:APECO1\_1668 | yahI; carbamate kinase; K00926 carbamate kinase [EC:2.7.2.2] | ec:2.7.2.2 |
| ecv:APECO1\_1667 | yahJ; deaminase |  |

  
**Over-represented Enzyme Summary**: Table of E.C. identified protein in the "Neighborhood Representation" ranked by frequency of occurrence  

| EC number | Frequency | Annotation | Reactions |
| --- | --- | --- | --- |
| ec:1.1.99.1 | 101 | choline dehydrogenase; choline oxidase; choline-cytochrome c reductase; choline:(acceptor) oxidoreductase; choline:(acceptor) 1-oxidoreductase | choline + acceptor = betaine aldehyde + reduced acceptor [RN:R01025] |
| ec:1.2.1.8 | 101 | betaine-aldehyde dehydrogenase; betaine aldehyde oxidase; BADH; betaine aldehyde dehydrogenase; BetB | betaine aldehyde + NAD+ + H2O = betaine + NADH + 2 H+ [RN:R02565] |
| ec:3.1.4.52 | 27 | cyclic-guanylate-specific phosphodiesterase; cyclic bis(3->5')diguanylate phosphodiesterase; c-di-GMP-specific phosphodiesterase; c-di-GMP phosphodiesterase; phosphodiesterase (misleading); phosphodiesterase A1; PDEA1; VieA | cyclic di-3',5'-guanylate + H2O = 5'-phosphoguanylyl(3'->5')guanosine [RN:R08991] |
| ec:2.7.2.2 | 18 | carbamate kinase; CKase ; carbamoyl phosphokinase; carbamyl phosphokinase | ATP + NH3 + CO2 = ADP + carbamoyl phosphate [RN:R00150] |
| ec:2.7.13.3 | 17 | histidine kinase; EnvZ; histidine kinase (ambiguous); histidine protein kinase (ambiguous); protein histidine kinase (ambiguous); protein kinase (histidine) (ambiguous); HK1; HP165; Sln1p | ATP + protein L-histidine = ADP + protein N-phospho-L-histidine |
| ec:1.2.1.46 | 14 | formaldehyde dehydrogenase; NAD+-linked formaldehyde dehydrogenase; NAD+-dependent formaldehyde dehydrogenase | formaldehyde + NAD+ + H2O = formate + NADH + 2 H+ [RN:R00604] |
| ec:1.5.3.1 | 10 | sarcosine oxidase | sarcosine + H2O + O2 = glycine + formaldehyde + H2O2 [RN:R00610] |
| ec:3.6.3.32 | 8 | quaternary-amine-transporting ATPase | ATP + H2O + quaternary amineout = ADP + phosphate + quaternary aminein [RN:R00086] |
| ec:4.3.1.17 | 8 | L-serine ammonia-lyase; serine deaminase; L-hydroxyaminoacid dehydratase; L-serine deaminase; L-serine dehydratase; L-serine hydro-lyase (deaminating) | L-serine = pyruvate + NH3 [RN:R00220] |
| ec:3.4.21.102 | 7 | C-terminal processing peptidase; CtpA gene product (Synechocystis sp.); photosystem II D1 protein processing peptidase; protease Re; tail-specific protease; Tsp protease | The enzyme shows specific recognition of a C-terminal tripeptide, Xaa-Yaa-Zaa, in which Xaa is preferably Ala or Leu, Yaa is preferably Ala or Tyr, and Zaa is preferably Ala, but then cleaves at a variable distance from the C-terminus. A typical cleavage is -Ala-Ala!Arg-Ala-Ala-Lys-Glu-Asn-Tyr-Ala-Leu-Ala-Ala. In the plant chloroplast, the enzyme removes the C-terminal extension of the D1 polypeptide of photosystem II |
| ec:3.4.13.19 | 7 | membrane dipeptidase; renal dipeptidase; dehydropeptidase I (DPH I); dipeptidase (ambiguous); aminodipeptidase; dipeptide hydrolase (ambiguous); dipeptidyl hydrolase (ambiguous); nonspecific dipeptidase; glycosyl-phosphatidylinositol-anchored renal dipeptidase; MDP | Hydrolysis of dipeptides |
| ec:2.1.2.1 | 7 | glycine hydroxymethyltransferase; serine aldolase; threonine aldolase; serine hydroxymethylase; serine hydroxymethyltransferase; allothreonine aldolase; L-serine hydroxymethyltransferase; L-threonine aldolase; serine hydroxymethyltransferase; serine transhydroxymethylase | 5,10-methylenetetrahydrofolate + glycine + H2O = tetrahydrofolate + L-serine [RN:R00945] |
| ec:2.5.1.18 | 5 | glutathione transferase; glutathione S-transferase; glutathione S-alkyltransferase; glutathione S-aryltransferase; S-(hydroxyalkyl)glutathione lyase; glutathione S-aralkyltransferase; glutathione S-alkyl transferase; GST | RX + glutathione = HX + R-S-glutathione [RN:R03522 R08511 R08512] |
| ec:5.4.2.12 | 4 | phosphoglycerate mutase (2,3-diphosphoglycerate-independent); cofactor independent phosphoglycerate mutase; 2,3-diphosphoglycerate-independent phosphoglycerate mutase; phosphoglycerate phosphomutase (ambiguous); phosphoglyceromutase (ambiguous); monophosphoglycerate mutase (ambiguous); monophosphoglyceromutase (ambiguous); GriP mutase (ambiguous); PGA mutase (ambiguous); iPGM; iPGAM; PGAM-i | 2-phospho-D-glycerate = 3-phospho-D-glycerate [RN:R01518] |
| ec:4.2.99.18 | 4 | DNA-(apurinic or apyrimidinic site) lyase; AP lyase; AP endonuclease class I; endodeoxyribonuclease (apurinic or apyrimidinic); deoxyribonuclease (apurinic or apyrimidinic); E. coli endonuclease III; phage-T4 UV endonuclease; Micrococcus luteus UV endonuclease; AP site-DNA 5'-phosphomonoester-lyase; X-ray endonuclease III | The C-O-P bond 3' to the apurinic or apyrimidinic site in DNA is broken by a beta-elimination reaction, leaving a 3'-terminal unsaturated sugar and a product with a terminal 5'-phosphate |
| ec:6.1.1.1 | 4 | tyrosine---tRNA ligase | ATP + L-tyrosine + tRNATyr = AMP + diphosphate + L-tyrosyl-tRNATyr [RN:R02918] |
| ec:4.2.1.3 | 4 | aconitate hydratase; cis-aconitase; aconitase; AcnB; 2-methylaconitate hydratase; citrate(isocitrate) hydro-lyase | citrate = isocitrate (overall reaction) [RN:R01324]; (1a) citrate = cis-aconitate + H2O [RN:R01325]; (1b) cis-aconitate + H2O = isocitrate [RN:R01900] |
| ec:1.4.3.5 | 4 | pyridoxal 5'-phosphate synthase; pyridoxamine 5'-phosphate oxidase; pyridoxamine phosphate oxidase; pyridoxine (pyridoxamine)phosphate oxidase; pyridoxine (pyridoxamine) 5'-phosphate oxidase; pyridoxaminephosphate oxidase (EC 1.4.3.5: deaminating); PMP oxidase; pyridoxol-5'-phosphate:oxygen oxidoreductase (deaminating) (incorrect); pyridoxamine-phosphate oxidase; PdxH | (1) pyridoxamine 5'-phosphate + H2O + O2 = pyridoxal 5'-phosphate + NH3 + H2O2 [RN:R00277]; (2) pyridoxine 5'-phosphate + O2 = pyridoxal 5'-phosphate + H2O2 [RN:R00278] |
| ec:2.7.1.170 | 4 | anhydro-N-acetylmuramic acid kinase; anhMurNAc kinase; AnmK | ATP + 1,6-anhydro-N-acetyl-beta-muramate + H2O = ADP + N-acetylmuramate 6-phosphate [RN:R09640] |
| ec:3.6.4.13 | 4 | RNA helicase; CSFV NS3 helicase; DBP2; DbpA; DDX17; DDX25; DDX3; DDX3X; DDX3Y; DDX4; DDX5; DEAD-box protein DED1; DEAD-box RNA helicase; DEAH-box protein 2; DEAH-box RNA helicase; DED1; Dex(H/D) RNA helicase; EhDEAD1; EhDEAD1 RNA helicase; eIF4A helicase; KOKV helicase; Mtr4p; nonstructural protein 3 helicase; NPH-II; RHA; RNA helicase A; RNA helicase DDX3; RNA helicase Hera; RNA-dependent ATPase; TGBp1 NTPase/helicase domain; VRH1; GRTH/DDX25 | ATP + H2O = ADP + phosphate [RN:R00086] |
| ec:2.1.1.207 | 4 | tRNA (cytidine34-2'-O)-methyltransferase; yibK (gene name); methyltransferase yibK; TrmL; tRNA methyltransferase L; tRNA (cytidine34/5-carboxymethylaminomethyluridine34-2'-O)-methyltransferase | (1) S-adenosyl-L-methionine + cytidine34 in tRNA = S-adenosyl-L-homocysteine + 2'-O-methylcytidine34 in tRNA; (2) S-adenosyl-L-methionine + 5-carboxymethylaminomethyluridine34 in tRNALeu = S-adenosyl-L-homocysteine + 5-carboxymethylaminomethyl-2'-O-methyluridine34 in tRNALeu |
| ec:1.8.1.8 | 4 | protein-disulfide reductase; protein disulphide reductase; insulin-glutathione transhydrogenase; disulfide reductase; NAD(P)H2:protein-disulfide oxidoreductase | protein-dithiol + NAD(P)+ = protein-disulfide + NAD(P)H + H+ [RN:R03913 R03914] |
| ec:3.4.21.26 | 3 | prolyl oligopeptidase; post-proline cleaving enzyme; proline-specific endopeptidase; post-proline endopeptidase; proline endopeptidase; endoprolylpeptidase; prolyl endopeptidase | Hydrolysis of ---Pro! and to a lesser extent ---Ala! in oligopeptides |
| ec:1.5.1.3 | 3 | dihydrofolate reductase; tetrahydrofolate dehydrogenase; DHFR; pteridine reductase:dihydrofolate reductase; dihydrofolate reductase:thymidylate synthase; thymidylate synthetase-dihydrofolate reductase; folic acid reductase; folic reductase; dihydrofolic acid reductase; dihydrofolic reductase; 7,8-dihydrofolate reductase; NADPH-dihydrofolate reductase | 5,6,7,8-tetrahydrofolate + NADP+ = 7,8-dihydrofolate + NADPH + H+ [RN:R00939] |
| ec:4.2.1.59 | 3 | 3-hydroxyacyl-[acyl-carrier-protein] dehydratase; fabZ (gene name); fabA (gene name); D-3-hydroxyoctanoyl-[acyl carrier protein] dehydratase; D-3-hydroxyoctanoyl-acyl carrier protein dehydratase; beta-hydroxyoctanoyl-acyl carrier protein dehydrase; beta-hydroxyoctanoyl thioester dehydratase; beta-hydroxyoctanoyl-ACP-dehydrase; (3R)-3-hydroxyoctanoyl-[acyl-carrier-protein] hydro-lyase; (3R)-3-hydroxyoctanoyl-[acyl-carrier-protein] hydro-lyase (oct-2-enoyl-[acyl-carrier protein]-forming); 3-hydroxyoctanoyl-[acyl-carrier-protein] dehydratase | a (3R)-3-hydroxyacyl-[acyl-carrier protein] = a trans-2-enoyl-[acyl-carrier protein] + H2O [RN:R10208] |
| ec:2.2.1.6 | 3 | acetolactate synthase; alpha-acetohydroxy acid synthetase; alpha-acetohydroxyacid synthase; alpha-acetolactate synthase; alpha-acetolactate synthetase; acetohydroxy acid synthetase; acetohydroxyacid synthase; acetolactate pyruvate-lyase (carboxylating); acetolactic synthetase | 2 pyruvate = 2-acetolactate + CO2 [RN:R00006] |
| ec:2.7.1.35 | 3 | pyridoxal kinase; pyridoxal kinase (phosphorylating); pyridoxal 5-phosphate-kinase; pyridoxal phosphokinase; pyridoxine kinase | ATP + pyridoxal = ADP + pyridoxal 5'-phosphate [RN:R00174] |
| ec:3.5.2.6 | 3 | beta-lactamase; penicillinase; cephalosporinase; neutrapen; penicillin beta-lactamase; exopenicillinase; ampicillinase; penicillin amido-beta-lactamhydrolase; penicillinase I, II; beta-lactamase I-III; beta-lactamase A, B, C; beta-lactamase AME I; cephalosporin-beta-lactamase | a beta-lactam + H2O = a substituted beta-amino acid [RN:R03743] |
| ec:2.1.1.33 | 3 | tRNA (guanine46-N7)-methyltransferase; Trm8/Trm82; TrmB; tRNA (m7G46) methyltransferase; transfer ribonucleate guanine 7-methyltransferase; 7-methylguanine transfer ribonucleate methylase; tRNA guanine 7-methyltransferase; N7-methylguanine methylase; S-adenosyl-L-methionine:tRNA (guanine-7-N-)-methyltransferase | S-adenosyl-L-methionine + guanine46 in tRNA = S-adenosyl-L-homocysteine + N7-methylguanine46 in tRNA [RN:R00600] |
| ec:4.2.1.24 | 2 | porphobilinogen synthase; aminolevulinate dehydratase; delta-aminolevulinate dehydratase; delta-aminolevulinic acid dehydrase; delta-aminolevulinic acid dehydratase; aminolevulinic dehydratase; delta-aminolevulinic dehydratase; 5-levulinic acid dehydratase; 5-aminolevulinate hydro-lyase (adding 5-aminolevulinate and cyclizing); hemB (gene name) | 2 5-aminolevulinate = porphobilinogen + 2 H2O [RN:R00036] |
| ec:6.1.1.6 | 2 | lysine---tRNA ligase; lysyl-tRNA synthetase; lysyl-transfer ribonucleate synthetase; lysyl-transfer RNA synthetase; L-lysine-transfer RNA ligase; lysine-tRNA synthetase; lysine translase | ATP + L-lysine + tRNALys = AMP + diphosphate + L-lysyl-tRNALys [RN:R03658] |
| ec:1.14.11.1 | 2 | gamma-butyrobetaine dioxygenase; alpha-butyrobetaine hydroxylase; gamma-butyrobetaine hydroxylase; butyrobetaine hydroxylase | 4-trimethylammoniobutanoate + 2-oxoglutarate + O2 = 3-hydroxy-4-trimethylammoniobutanoate + succinate + CO2 [RN:R02397] |
| ec:3.6.3.34 | 2 | iron-chelate-transporting ATPase | ATP + H2O + iron chelateout = ADP + phosphate + iron chelatein [RN:R00086] |
| ec:2.7.2.11 | 2 | glutamate 5-kinase; ATP-L-glutamate 5-phosphotransferase; ATP:gamma-L-glutamate phosphotransferase; gamma-glutamate kinase; gamma-glutamyl kinase; glutamate kinase | ATP + L-glutamate = ADP + L-glutamate 5-phosphate [RN:R00239] |
| ec:1.1.1.108 | 2 | carnitine 3-dehydrogenase | carnitine + NAD+ = 3-dehydrocarnitine + NADH + H+ [RN:R02395] |
| ec:2.3.1.30 | 2 | serine O-acetyltransferase; SATase; L-serine acetyltransferase; serine acetyltransferase; serine transacetylase | acetyl-CoA + L-serine = CoA + O-acetyl-L-serine [RN:R00586] |
| ec:2.1.1.178 | 2 | 16S rRNA (cytosine1407-C5)-methyltransferase; RNA m5C methyltransferase YebU; RsmF; YebU | S-adenosyl-L-methionine + cytosine1407 in 16S rRNA = S-adenosyl-L-homocysteine + 5-methylcytosine1407 in 16S rRNA |
| ec:2.4.2.22 | 2 | xanthine phosphoribosyltransferase; Xan phosphoribosyltransferase; xanthosine 5'-phosphate pyrophosphorylase; xanthylate pyrophosphorylase; xanthylic pyrophosphorylase; XMP pyrophosphorylase; 5-phospho-alpha-D-ribose-1-diphosphate:xanthine phospho-D-ribosyltransferase; 9-(5-phospho-beta-D-ribosyl)xanthine:diphosphate 5-phospho-alpha-D-ribosyltransferase | XMP + diphosphate = 5-phospho-alpha-D-ribose 1-diphosphate + xanthine [RN:R02142] |
| ec:2.7.7.7 | 2 | DNA-directed DNA polymerase; DNA polymerase I; DNA polymerase II; DNA polymerase III; DNA polymerase alpha; DNA polymerase beta; DNA polymerase gamma; DNA nucleotidyltransferase (DNA-directed); DNA nucleotidyltransferase (DNA-directed); deoxyribonucleate nucleotidyltransferase; deoxynucleate polymerase; deoxyribonucleic acid duplicase; deoxyribonucleic acid polymerase; deoxyribonucleic duplicase; deoxyribonucleic polymerase; deoxyribonucleic polymerase I; DNA duplicase; DNA nucleotidyltransferase; DNA polymerase; DNA replicase; DNA-dependent DNA polymerase; duplicase; Klenow fragment; sequenase; Taq DNA polymerase; Taq Pol I; Tca DNA polymerase | deoxynucleoside triphosphate + DNAn = diphosphate + DNAn+1 [RN:R00379] |
| ec:1.2.1.24 | 2 | succinate-semialdehyde dehydrogenase (NAD+); succinate semialdehyde dehydrogenase (NAD+); succinic semialdehyde dehydrogenase (NAD+); succinyl semialdehyde dehydrogenase (NAD+); succinate semialdehyde:NAD+ oxidoreductase | succinate semialdehyde + NAD+ + H2O = succinate + NADH + 2 H+ [RN:R00713] |
| ec:2.2.1.1 | 2 | transketolase; glycolaldehydetransferase | sedoheptulose 7-phosphate + D-glyceraldehyde 3-phosphate = D-ribose 5-phosphate + D-xylulose 5-phosphate [RN:R01641] |
| ec:1.2.1.16 | 2 | succinate-semialdehyde dehydrogenase [NAD(P)+]; succinate semialdehyde dehydrogenase (nicotinamide adenine dinucleotide (phosphate)); succinate-semialdehyde dehydrogenase [NAD(P)] | succinate semialdehyde + NAD(P)+ + H2O = succinate + NAD(P)H + 2 H+ [RN:R00713 R00714] |
| ec:3.5.1.44 | 2 | protein-glutamine glutaminase; peptidoglutaminase II; glutaminyl-peptide glutaminase; destabilase; peptidylglutaminase II | protein L-glutamine + H2O = protein L-glutamate + NH3 [RN:R02622] |
| ec:3.1.1.61 | 2 | protein-glutamate methylesterase; chemotaxis-specific methylesterase; methyl-accepting chemotaxis protein methyl-esterase; CheB methylesterase; methylesterase CheB; protein methyl-esterase; protein carboxyl methylesterase; PME; protein methylesterase; protein-L-glutamate-5-O-methyl-ester acylhydrolase | protein L-glutamate O5-methyl ester + H2O = protein L-glutamate + methanol [RN:R02624] |
| ec:4.2.1.99 | 2 | 2-methylisocitrate dehydratase; (2S,3R)-3-hydroxybutane-1,2,3-tricarboxylate hydro-lyase | (2S,3R)-3-hydroxybutane-1,2,3-tricarboxylate = (Z)-but-2-ene-1,2,3-tricarboxylate + H2O [RN:R04425] |
| ec:1.6.5.5 | 2 | NADPH:quinone reductase; NADPH2:quinone reductase | NADPH + H+ + 2 quinone = NADP+ + 2 semiquinone [RN:R02364] |
| ec:1.1.1.313 | 1 | sulfoacetaldehyde reductase; isfD (gene name) | isethionate + NADP+ = 2-sulfoacetaldehyde + NADPH + H+ [RN:R02600] |
| ec:1.3.3.11 | 1 | pyrroloquinoline-quinone synthase; PqqC; 6-(2-amino-2-carboxyethyl)-7,8-dioxo-1,2,3,4,5,6,7,8-octahydroquinoline-2,4-dicarboxylate:oxygen oxidoreductase (cyclizing) [incorrect] | 6-(2-amino-2-carboxyethyl)-7,8-dioxo-1,2,3,4,7,8-hexahydroquinoline-2,4-dicarboxylate + 3 O2 = 4,5-dioxo-4,5-dihydro-1H-pyrrolo[2,3-f]quinoline-2,7,9-tricarboxylate + 2 H2O2 + 2 H2O [RN:R07353] |
| ec:1.1.1.1 | 1 | alcohol dehydrogenase; aldehyde reductase; ADH; alcohol dehydrogenase (NAD); aliphatic alcohol dehydrogenase; ethanol dehydrogenase; NAD-dependent alcohol dehydrogenase; NAD-specific aromatic alcohol dehydrogenase; NADH-alcohol dehydrogenase; NADH-aldehyde dehydrogenase; primary alcohol dehydrogenase; yeast alcohol dehydrogenase | (1) a primary alcohol + NAD+ = an aldehyde + NADH + H+ [RN:R07326]; (2) a secondary alcohol + NAD+ = a ketone + NADH + H+ [RN:R07327] |
| ec:4.1.2.13 | 1 | fructose-bisphosphate aldolase; aldolase; fructose-1,6-bisphosphate triosephosphate-lyase; fructose diphosphate aldolase; diphosphofructose aldolase; fructose 1,6-diphosphate aldolase; ketose 1-phosphate aldolase; phosphofructoaldolase; zymohexase; fructoaldolase; fructose 1-phosphate aldolase; fructose 1-monophosphate aldolase; 1,6-Diphosphofructose aldolase; SMALDO; D-fructose-1,6-bisphosphate D-glyceraldehyde-3-phosphate-lyase | D-fructose 1,6-bisphosphate = glycerone phosphate + D-glyceraldehyde 3-phosphate [RN:R01068] |
| ec:3.6.3.36 | 1 | taurine-transporting ATPase | ATP + H2O + taurineout = ADP + phosphate + taurinein [RN:R00086] |
| ec:1.1.1.304 | 1 | diacetyl reductase [(S)-acetoin forming]; (S)-acetoin dehydrogenase | (S)-acetoin + NAD+ = diacetyl + NADH + H+ [RN:R09078] |
| ec:1.17.4.1 | 1 | ribonucleoside-diphosphate reductase; ribonucleotide reductase; CDP reductase; ribonucleoside diphosphate reductase; UDP reductase; ADP reductase; nucleoside diphosphate reductase; ribonucleoside 5'-diphosphate reductase; ribonucleotide diphosphate reductase; 2'-deoxyribonucleoside-diphosphate:oxidized-thioredoxin 2'-oxidoreductase; RR | 2'-deoxyribonucleoside diphosphate + thioredoxin disulfide + H2O = ribonucleoside diphosphate + thioredoxin [RN:R04294] |
| ec:3.1.4.46 | 1 | glycerophosphodiester phosphodiesterase; gene hpd protein; glycerophosphoryl diester phosphodiesterase; IgD-binding protein D | a glycerophosphodiester + H2O = an alcohol + sn-glycerol 3-phosphate [RN:R00857] |
| ec:2.1.1.72 | 1 | site-specific DNA-methyltransferase (adenine-specific); modification methylase; restriction-modification system | S-adenosyl-L-methionine + DNA adenine = S-adenosyl-L-homocysteine + DNA 6-methylaminopurine [RN:R02961] |
| ec:3.6.3.27 | 1 | phosphate-transporting ATPase; ABC phosphate transporter | ATP + H2O + phosphateout = ADP + phosphate + phosphatein [RN:R00086] |
| ec:1.2.1.41 | 1 | glutamate-5-semialdehyde dehydrogenase; beta-glutamylphosphate reductase; gamma-glutamyl phosphate reductase; beta-glutamylphosphate reductase; glutamate semialdehyde dehydrogenase; glutamate-gamma-semialdehyde dehydrogenase | L-glutamate 5-semialdehyde + phosphate + NADP+ = L-glutamyl 5-phosphate + NADPH + H+ [RN:R03313] |
| ec:2.6.1.55 | 1 | taurine---2-oxoglutarate transaminase; taurine aminotransferase; taurine transaminase; taurine---alpha-ketoglutarate aminotransferase; taurine---glutamate transaminase | taurine + 2-oxoglutarate = 2-sulfoacetaldehyde + L-glutamate [RN:R01684] |
| ec:3.1.1.31 | 1 | 6-phosphogluconolactonase; phosphogluconolactonase; 6-PGL | 6-phospho-D-glucono-1,5-lactone + H2O = 6-phospho-D-gluconate [RN:R02035] |
| ec:4.3.1.19 | 1 | threonine ammonia-lyase; threonine deaminase; L-serine dehydratase; serine deaminase; L-threonine dehydratase; threonine dehydrase; L-threonine deaminase; threonine dehydratase; L-threonine hydro-lyase (deaminating); L-threonine ammonia-lyase | L-threonine = 2-oxobutanoate + NH3 [RN:R00996] |
| ec:6.5.1.2 | 1 | DNA ligase (NAD+); polydeoxyribonucleotide synthase (NAD+); polynucleotide ligase (NAD+); DNA repair enzyme; DNA joinase; polynucleotide synthetase (nicotinamide adenine dinucleotide); deoxyribonucleic-joining enzyme; deoxyribonucleic ligase; deoxyribonucleic repair enzyme; deoxyribonucleic joinase; DNA ligase; deoxyribonucleate ligase; polynucleotide ligase; deoxyribonucleic acid ligase; polynucleotide synthetase; deoxyribonucleic acid joinase; DNA-joining enzyme; polynucleotide ligase (nicotinamide adenine dinucleotide) | NAD+ + (deoxyribonucleotide)n + (deoxyribonucleotide)m = AMP + beta-nicotinamide D-ribonucleotide + (deoxyribonucleotide)n+m [RN:R00382] |
| ec:4.3.1.12 | 1 | ornithine cyclodeaminase; ornithine cyclase; ornithine cyclase (deaminating); L-ornithine ammonia-lyase (cyclizing) | L-ornithine = L-proline + NH3 [RN:R00671] |
| ec:3.4.11.21 | 1 | aspartyl aminopeptidase | Release of an N-terminal aspartate or glutamate from a peptide, with a preference for aspartate |
| ec:1.1.1.28 | 1 | D-lactate dehydrogenase; lactic acid dehydrogenase; lactic acid dehydrogenase; D-specific lactic dehydrogenase; D-(-)-lactate dehydrogenase (NAD+); D-lactic acid dehydrogenase; D-lactic dehydrogenase | (R)-lactate + NAD+ = pyruvate + NADH + H+ [RN:R00704] |
| ec:3.1.1.29 | 1 | aminoacyl-tRNA hydrolase; aminoacyl-transfer ribonucleate hydrolase; N-substituted aminoacyl transfer RNA hydrolase; peptidyl-tRNA hydrolase | N-substituted aminoacyl-tRNA + H2O = N-substituted amino acid + tRNA [RN:R04238] |
| ec:1.4.1.1 | 1 | alanine dehydrogenase; AlaDH; L-alanine dehydrogenase; NAD+-linked alanine dehydrogenase; alpha-alanine dehydrogenase; NAD+-dependent alanine dehydrogenase; alanine oxidoreductase; NADH-dependent alanine dehydrogenase | L-alanine + H2O + NAD+ = pyruvate + NH3 + NADH + H+ [RN:R00396] |
| ec:4.1.1.5 | 1 | acetolactate decarboxylase; alpha-acetolactate decarboxylase; (S)-2-hydroxy-2-methyl-3-oxobutanoate carboxy-lyase; (S)-2-hydroxy-2-methyl-3-oxobutanoate carboxy-lyase [(R)-2-acetoin-forming]; (S)-2-hydroxy-2-methyl-3-oxobutanoate carboxy-lyase [(3R)-3-hydroxybutan-2-one-forming] | (2S)-2-hydroxy-2-methyl-3-oxobutanoate = (3R)-3-hydroxybutan-2-one + CO2 [RN:R02948] |
| ec:2.6.1.42 | 1 | branched-chain-amino-acid transaminase; transaminase B; branched-chain amino acid aminotransferase; branched-chain amino acid-glutamate transaminase; branched-chain aminotransferase; L-branched chain amino acid aminotransferase; glutamate-branched-chain amino acid transaminase | L-leucine + 2-oxoglutarate = 4-methyl-2-oxopentanoate + L-glutamate [RN:R01090] |
| ec:4.1.1.44 | 1 | 4-carboxymuconolactone decarboxylase; gamma-4-carboxymuconolactone decarboxylase; 4-carboxymuconolactone carboxy-lyase; 2-carboxy-2,5-dihydro-5-oxofuran-2-acetate carboxy-lyase (4,5-dihydro-5-oxofuran-2-acetate-forming) | (R)-2-carboxy-2,5-dihydro-5-oxofuran-2-acetate = 4,5-dihydro-5-oxofuran-2-acetate + CO2 [RN:R03470] |
| ec:2.7.6.1 | 1 | ribose-phosphate diphosphokinase; ribose-phosphate pyrophosphokinase; PRPP synthetase; phosphoribosylpyrophosphate synthetase; PPRibP synthetase; PP-ribose P synthetase; 5-phosphoribosyl-1-pyrophosphate synthetase; 5-phosphoribose pyrophosphorylase; 5-phosphoribosyl-alpha-1-pyrophosphate synthetase; phosphoribosyl-diphosphate synthetase; phosphoribosylpyrophosphate synthase; pyrophosphoribosylphosphate synthetase; ribophosphate pyrophosphokinase; ribose-5-phosphate pyrophosphokinase | ATP + D-ribose 5-phosphate = AMP + 5-phospho-alpha-D-ribose 1-diphosphate [RN:R01049] |
| ec:1.1.1.76 | 1 | (S,S)-butanediol dehydrogenase; L-butanediol dehydrogenase; L-BDH; L(+)-2,3-butanediol dehydrogenase (L-acetoin forming); (S)-acetoin reductase [(S,S)-butane-2,3-diol forming] | (2S,3S)-butane-2,3-diol + NAD+ = (S)-acetoin + NADH + H+ [RN:R03707] |
| ec:3.1.2.12 | 1 | S-formylglutathione hydrolase | S-formylglutathione + H2O = glutathione + formate [RN:R00527] |
| ec:3.1.3.16 | 1 | protein-serine/threonine phosphatase; phosphoprotein phosphatase (ambiguous); protein phosphatase-1; protein phosphatase-2A; protein phosphatase-2B; protein phosphatase-2C; protein D phosphatase; phosphospectrin phosphatase; casein phosphatase; Aspergillus awamori acid protein phosphatase; calcineurin; phosphatase 2A; phosphatase 2B; phosphatase II; phosphatase IB; phosphatase C-II; polycation modulated (PCM-) phosphatase; phosphopyruvate dehydrogenase phosphatase; phosphatase SP; branched-chain alpha-keto acid dehydrogenase phosphatase; BCKDH phosphatase; 3-hydroxy 3-methylglutaryl coenzymeA reductase phosphatase; HMG-CoA reductase phosphatase; phosphatase H-II; phosphatase III; phosphatase I; protein phosphatase; phosphatase IV; phosphoprotein phosphohydrolase | [a protein]-serine/threonine phosphate + H2O = [a protein]-serine/threonine + phosphate [RN:R00164] |
| ec:4.1.3.39 | 1 | 4-hydroxy-2-oxovalerate aldolase; 4-hydroxy-2-ketovalerate aldolase; HOA; DmpG; 4-hydroxy-2-oxovalerate pyruvate-lyase; 4-hydroxy-2-oxopentanoate pyruvate-lyase; BphI; 4-hydroxy-2-oxopentanoate pyruvate-lyase (acetaldehyde-forming) | (S)-4-hydroxy-2-oxopentanoate = acetaldehyde + pyruvate [RN:R00750] |
| ec:3.5.4.1 | 1 | cytosine deaminase; isocytosine deaminase | cytosine + H2O = uracil + NH3 [RN:R00974] |
| ec:2.7.1.30 | 1 | glycerol kinase; glycerokinase; GK; ATP:glycerol-3-phosphotransferase; glycerol kinase (phosphorylating); glyceric kinase | ATP + glycerol = ADP + sn-glycerol 3-phosphate [RN:R00847] |
| ec:1.1.1.125 | 1 | 2-deoxy-D-gluconate 3-dehydrogenase; 2-deoxygluconate dehydrogenase | 2-deoxy-D-gluconate + NAD+ = 3-dehydro-2-deoxy-D-gluconate + NADH + H+ [RN:R04049] |
| ec:2.7.1.148 | 1 | 4-(cytidine 5'-diphospho)-2-C-methyl-D-erythritol kinase; CDP-ME kinase | ATP + 4-(cytidine 5'-diphospho)-2-C-methyl-D-erythritol = ADP + 2-phospho-4-(cytidine 5'-diphospho)-2-C-methyl-D-erythritol [RN:R05634] |
| ec:2.6.1.9 | 1 | histidinol-phosphate transaminase; imidazolylacetolphosphate transaminase; glutamic-imidazoleacetol phosphate transaminase; histidinol phosphate aminotransferase; imidazoleacetol phosphate transaminase; L-histidinol phosphate aminotransferase; histidine:imidazoleacetol phosphate transaminase; IAP transaminase; imidazolylacetolphosphate aminotransferase | L-histidinol phosphate + 2-oxoglutarate = 3-(imidazol-4-yl)-2-oxopropyl phosphate + L-glutamate [RN:R03243] |
| ec:6.2.1.3 | 1 | long-chain-fatty-acid---CoA ligase; acyl-CoA synthetase; fatty acid thiokinase (long chain); acyl-activating enzyme; palmitoyl-CoA synthase; lignoceroyl-CoA synthase; arachidonyl-CoA synthetase; acyl coenzyme A synthetase; acyl-CoA ligase; palmitoyl coenzyme A synthetase; thiokinase; palmitoyl-CoA ligase; acyl-coenzyme A ligase; fatty acid CoA ligase; long-chain fatty acyl coenzyme A synthetase; oleoyl-CoA synthetase; stearoyl-CoA synthetase; long chain fatty acyl-CoA synthetase; long-chain acyl CoA synthetase; fatty acid elongase; LCFA synthetase; pristanoyl-CoA synthetase; ACS3; long-chain acyl-CoA synthetase I; long-chain acyl-CoA synthetase II; fatty acyl-coenzyme A synthetase; long-chain acyl-coenzyme A synthetase; FAA1 | ATP + a long-chain fatty acid + CoA = AMP + diphosphate + an acyl-CoA [RN:R00390] |
| ec:1.14.11.17 | 1 | taurine dioxygenase; 2-aminoethanesulfonate dioxygenase; alpha-ketoglutarate-dependent taurine dioxygenase | taurine + 2-oxoglutarate + O2 = sulfite + aminoacetaldehyde + succinate + CO2 [RN:R05320] |
| ec:1.13.11.3 | 1 | protocatechuate 3,4-dioxygenase; protocatechuate oxygenase; protocatechuic acid oxidase; protocatechuic 3,4-dioxygenase; protocatechuic 3,4-oxygenase | 3,4-dihydroxybenzoate + O2 = 3-carboxy-cis,cis-muconate [RN:R01631] |

  
**Over-represented Metabolite Summary**: Collection of the metabolites identified as substrates or products of the proteins representaed the "Over-represented Enzyme Summary" ranked by frequency of occurrence  

| ID | Structure | Name | Frequency | EC |
| --- | --- | --- | --- | --- |
| cpd:C00001 |  | H2O; Water | 218 | ec:3.1.4.52 ec:4.2.1.3 ec:4.3.1.17 ec:3.1.2.12 ec:1.2.1.24 ec:4.2.1.59 ec:1.13.11.3 ec:1.2.1.8 ec:3.1.1.31 ec:1.2.1.46 ec:2.1.2.1 ec:4.2.1.24 ec:1.17.4.1 ec:1.4.1.1 ec:3.5.2.6 ec:2.5.1.18 ec:1.2.1.16 ec:1.4.3.5 ec:3.5.4.1 ec:3.1.4.46 ec:4.2.1.99 ec:1.1.1.1 ec:1.5.3.1 |
| cpd:C00576 |  | Betaine aldehyde | 202 | ec:1.1.99.1 ec:1.2.1.8 |
| cpd:C00080 |  | H+; Hydron | 131 | ec:1.4.1.1 ec:1.5.1.3 ec:1.1.1.125 ec:2.5.1.18 ec:1.2.1.16 ec:1.2.1.41 ec:1.2.1.24 ec:1.2.1.8 ec:1.1.1.28 ec:1.2.1.46 ec:1.1.1.313 ec:1.1.1.1 |
| cpd:C00004 |  | NADH; DPNH; Reduced nicotinamide adenine dinucleotide | 124 | ec:1.4.1.1 ec:1.5.1.3 ec:1.1.1.125 ec:1.2.1.16 ec:1.2.1.24 ec:1.2.1.8 ec:1.1.1.28 ec:1.2.1.46 ec:1.1.1.1 |
| cpd:C00003 |  | NAD+; NAD; Nicotinamide adenine dinucleotide; DPN; Diphosphopyridine nucleotide; Nadide | 124 | ec:1.4.1.1 ec:1.5.1.3 ec:1.1.1.125 ec:1.2.1.16 ec:1.2.1.24 ec:1.2.1.8 ec:1.1.1.28 ec:1.2.1.46 ec:1.1.1.1 |
| cpd:C00006 |  | NADP+; NADP; Nicotinamide adenine dinucleotide phosphate; beta-Nicotinamide adenine dinucleotide phosphate; TPN; Triphosphopyridine nucleotide | 108 | ec:1.5.1.3 ec:1.2.1.16 ec:1.2.1.41 ec:1.2.1.24 ec:1.2.1.8 ec:1.1.1.313 |
| cpd:C00005 |  | NADPH; TPNH; Reduced nicotinamide adenine dinucleotide phosphate | 108 | ec:1.5.1.3 ec:1.2.1.16 ec:1.2.1.41 ec:1.2.1.24 ec:1.2.1.8 ec:1.1.1.313 |
| cpd:C00114 |  | Choline; Bilineurine | 102 | ec:1.1.99.1 ec:3.1.4.46 |
| cpd:C00719 |  | Betaine; Trimethylaminoacetate; Glycine betaine; N,N,N-Trimethylglycine; Trimethylammonioacetate | 101 | ec:1.2.1.8 |
| cpd:C00030 |  | Reduced acceptor; AH2; Hydrogen-donor; Donor | 101 | ec:1.1.99.1 |
| cpd:C00028 |  | Acceptor; Hydrogen-acceptor; A; Oxidized donor | 101 | ec:1.1.99.1 |
| cpd:C18239 |  | Precursor Z; Cyclic pyranopterin monophosphate; Cyclic pyranopterin phosphate; cPMP | 35 |  |
| cpd:C00014 |  | Ammonia; NH3 | 34 | ec:2.7.2.2 ec:1.4.1.1 ec:4.3.1.19 ec:4.3.1.17 ec:1.4.3.5 ec:4.3.1.12 ec:3.5.4.1 |
| cpd:C00002 |  | ATP; Adenosine 5'-triphosphate | 33 | ec:2.7.2.2 ec:2.7.1.30 ec:6.1.1.1 ec:2.7.2.11 ec:2.7.1.35 ec:2.7.1.148 ec:6.2.1.3 ec:6.1.1.6 ec:2.7.6.1 |
| cpd:C16463 |  | 3',5'-Cyclic diGMP; cdiGMP; 3',5'-Cyclic diguanylic acid; Bis-(3',5')-cyclic diGMP; Cyclic di-3',5'-guanylate | 27 | ec:3.1.4.52 |
| cpd:C18076 |  | 5'-Phosphoguanylyl(3'->5')guanosine | 27 | ec:3.1.4.52 |
| cpd:C00013 |  | Diphosphate; Diphosphoric acid; Pyrophosphate; Pyrophosphoric acid; PPi | 27 | ec:6.1.1.1 ec:6.2.1.3 ec:6.1.1.6 ec:2.4.2.22 |
| cpd:C00011 |  | CO2; Carbon dioxide | 26 | ec:2.7.2.2 ec:1.14.11.1 ec:2.2.1.6 ec:4.1.1.5 ec:1.14.11.17 ec:4.1.1.44 |
| cpd:C00008 |  | ADP; Adenosine 5'-diphosphate | 26 | ec:2.7.2.2 ec:1.17.4.1 ec:2.7.1.30 ec:2.7.2.11 ec:2.7.1.35 ec:2.7.1.148 |
| cpd:C00067 |  | Formaldehyde; Methanal; Oxomethane; Oxomethylene; Methylene oxide; Formalin | 24 | ec:1.2.1.46 ec:1.5.3.1 |
| cpd:C00037 |  | Glycine; Aminoacetic acid; Gly | 19 | ec:2.1.2.1 ec:1.5.3.1 |
| cpd:C00065 |  | L-Serine; L-2-Amino-3-hydroxypropionic acid; L-3-Hydroxy-alanine; Serine | 18 | ec:4.3.1.19 ec:2.3.1.30 ec:4.3.1.17 ec:2.1.2.1 |
| cpd:C00044 |  | GTP; Guanosine 5'-triphosphate | 18 |  |
| cpd:C00169 |  | Carbamoyl phosphate | 18 | ec:2.7.2.2 |
| cpd:C00007 |  | Oxygen; O2 | 18 | ec:1.14.11.1 ec:1.4.3.5 ec:1.13.11.3 ec:1.14.11.17 ec:1.5.3.1 |
| cpd:C01563 |  | Carbamate; Carbamic acid; Aminoformic acid | 18 | ec:2.7.2.2 |
| cpd:C05924 |  | Molybdopterin; Pyranopterin; H2Dtpp-mP | 17 |  |
| cpd:C17023 |  | Sulfur donor; S-donor | 17 |  |
| cpd:C00058 |  | Formate; Methanoic acid; Formic acid | 15 | ec:3.1.2.12 ec:1.2.1.46 |
| cpd:C00022 |  | Pyruvate; Pyruvic acid; 2-Oxopropanoate; 2-Oxopropanoic acid; Pyroracemic acid | 15 | ec:1.4.1.1 ec:4.3.1.19 ec:4.3.1.17 ec:4.1.3.39 ec:2.2.1.6 ec:1.1.1.28 |
| cpd:C00027 |  | Hydrogen peroxide; H2O2; Oxydol | 14 | ec:1.4.3.5 ec:1.5.3.1 |
| cpd:C00385 |  | Xanthine | 11 | ec:2.4.2.22 |
| cpd:C00101 |  | Tetrahydrofolate; 5,6,7,8-Tetrahydrofolate; Tetrahydrofolic acid; THF; (6S)-Tetrahydrofolate; (6S)-Tetrahydrofolic acid; (6S)-THFA | 10 | ec:1.5.1.3 ec:2.1.2.1 |
| cpd:C00213 |  | Sarcosine; N-Methylglycine | 10 | ec:1.5.3.1 |
| cpd:C00009 |  | Orthophosphate; Phosphate; Phosphoric acid; Orthophosphoric acid | 10 | ec:1.2.1.41 |
| cpd:C00620 |  | alpha-D-Ribose 1-phosphate; Ribose 1-phosphate; D-Ribose 1-phosphate | 9 |  |
| cpd:C01762 |  | Xanthosine | 9 |  |
| cpd:C02218 |  | Dehydroalanine; 2-Aminoacrylate | 8 | ec:4.3.1.17 |
| cpd:C00082 |  | L-Tyrosine; (S)-3-(p-Hydroxyphenyl)alanine; (S)-2-Amino-3-(p-hydroxyphenyl)propionic acid; Tyrosine | 8 | ec:6.1.1.1 ec:2.6.1.9 |
| cpd:C00020 |  | AMP; Adenosine 5'-monophosphate; Adenylic acid; Adenylate; 5'-AMP; 5'-Adenylic acid; 5'-Adenosine monophosphate; Adenosine 5'-phosphate | 8 | ec:6.1.1.1 ec:6.2.1.3 ec:6.1.1.6 ec:2.7.6.1 |
| cpd:C00534 |  | Pyridoxamine; PM | 7 | ec:2.7.1.35 ec:1.4.3.5 |
| cpd:C00314 |  | Pyridoxine; Pyridoxol | 7 | ec:2.7.1.35 ec:1.4.3.5 |
| cpd:C00250 |  | Pyridoxal | 7 | ec:2.7.1.35 ec:1.4.3.5 |
| cpd:C00647 |  | Pyridoxamine phosphate; Pyridoxamine 5-phosphate; Pyridoxamine 5'-phosphate | 7 | ec:2.7.1.35 ec:1.4.3.5 |
| cpd:C04377 |  | 5,10-Methylenetetrahydromethanopterin; N5,N10-Methylenetetrahydromethanopterin | 7 | ec:2.1.2.1 |
| cpd:C00627 |  | Pyridoxine phosphate; Pyridoxine 5-phosphate; Pyridoxine 5'-phosphate; Pyridoxol 5'-phosphate | 7 | ec:2.7.1.35 ec:1.4.3.5 |
| cpd:C00018 |  | Pyridoxal phosphate; Pyridoxal 5-phosphate; Pyridoxal 5'-phosphate; PLP | 7 | ec:2.7.1.35 ec:1.4.3.5 |
| cpd:C01217 |  | 5,6,7,8-Tetrahydromethanopterin; H4MPT; THMPT; Tetrahydromethanopterin | 7 | ec:2.1.2.1 |
| cpd:C00143 |  | 5,10-Methylenetetrahydrofolate; (6R)-5,10-Methylenetetrahydrofolate; 5,10-Methylene-THF | 7 | ec:2.1.2.1 |
| cpd:C00051 |  | Glutathione; 5-L-Glutamyl-L-cysteinylglycine; N-(N-gamma-L-Glutamyl-L-cysteinyl)glycine; gamma-L-Glutamyl-L-cysteinyl-glycine; GSH; Reduced glutathione | 6 | ec:3.1.2.12 ec:2.5.1.18 |
| cpd:C00026 |  | 2-Oxoglutarate; Oxoglutaric acid; 2-Ketoglutaric acid; alpha-Ketoglutaric acid | 6 | ec:2.6.1.55 ec:2.6.1.42 ec:1.14.11.1 ec:1.14.11.17 ec:2.6.1.9 |
| cpd:C07645 |  | Aldophosphamide | 6 | ec:2.5.1.18 ec:1.1.1.1 |
| cpd:C14839 |  | Bromobenzene-3,4-oxide; Bromobenzene-3,4-epoxide | 5 | ec:2.5.1.18 |
| cpd:C11088 |  | 1,2-Dibromoethane; Ethylene dibromide | 5 | ec:2.5.1.18 |
| cpd:C14793 |  | (1R)-Glutathionyl-(2R)-hydroxy-1,2-dihydronaphthalene | 5 | ec:2.5.1.18 |
| cpd:C14792 |  | (1S)-Hydroxy-(2S)-glutathionyl-1,2-dihydronaphthalene | 5 | ec:2.5.1.18 |
| cpd:C14791 |  | (1R)-Hydroxy-(2R)-glutathionyl-1,2-dihydronaphthalene | 5 | ec:2.5.1.18 |
| cpd:C11278 |  | Aflatoxin B1exo-8,9-epoxide-GSH; 8,9-Dihydro-8-(S-glutathionyl)-9-hydroxyaflatoxin B1 | 5 | ec:2.5.1.18 |
| cpd:C14787 |  | (1S,2R)-Naphthalene 1,2-oxide; (1S,2R)-Naphthalene epoxide | 5 | ec:2.5.1.18 |
| cpd:C14786 |  | (1R,2S)-Naphthalene 1,2-oxide; (1R,2S)-Naphthalene epoxide | 5 | ec:2.5.1.18 |
| cpd:C19586 |  | Aflatoxin B1-exo-8,9-epoxide; 2,3-Epoxyaflatoxin B1 | 5 | ec:2.5.1.18 |
| cpd:C00462 |  | Halide; Hydrogen halide; HX; Halo acid | 5 | ec:2.5.1.18 |
| cpd:C14806 |  | 1-Nitro-5-glutathionyl-6-hydroxy-5,6-dihydronaphthalene | 5 | ec:2.5.1.18 |
| cpd:C14805 |  | 1-Nitro-5-hydroxy-6-glutathionyl-5,6-dihydronaphthalene | 5 | ec:2.5.1.18 |
| cpd:C14804 |  | 1-Nitro-7-glutathionyl-8-hydroxy-7,8-dihydronaphthalene | 5 | ec:2.5.1.18 |
| cpd:C14803 |  | 1-Nitro-7-hydroxy-8-glutathionyl-7,8-dihydronaphthalene | 5 | ec:2.5.1.18 |
| cpd:C14802 |  | 1-Nitronaphthalene-7,8-oxide | 5 | ec:2.5.1.18 |
| cpd:C01327 |  | Hydrochloric acid; HCl; Hydrogen chloride; Hydrochloride | 5 | ec:2.5.1.18 |
| cpd:C14800 |  | 1-Nitronaphthalene-5,6-oxide | 5 | ec:2.5.1.18 |
| cpd:C01322 |  | RX; Organic halide | 5 | ec:2.5.1.18 |
| cpd:C00042 |  | Succinate; Succinic acid; Butanedionic acid; Ethylenesuccinic acid | 5 | ec:1.14.11.1 ec:1.2.1.16 ec:1.2.1.24 ec:1.14.11.17 |
| cpd:C11583 |  | 4-Glutathionyl cyclophosphamide | 5 | ec:2.5.1.18 |
| cpd:C02320 |  | R-S-Glutathione | 5 | ec:2.5.1.18 |
| cpd:C00025 |  | L-Glutamate; L-Glutamic acid; L-Glutaminic acid; Glutamate | 5 | ec:2.6.1.55 ec:2.6.1.42 ec:2.7.2.11 ec:2.6.1.9 |
| cpd:C14874 |  | Glutathione episulfonium ion | 5 | ec:2.5.1.18 |
| cpd:C14871 |  | S-(Formylmethyl)glutathione | 5 | ec:2.5.1.18 |
| cpd:C14870 |  | 2-Bromoacetaldehyde | 5 | ec:2.5.1.18 |
| cpd:C13645 |  | Hydrobromic acid; HBr | 5 | ec:2.5.1.18 |
| cpd:C14868 |  | S-(1,2-Dichlorovinyl)glutathione; DCVG | 5 | ec:2.5.1.18 |
| cpd:C06790 |  | Trichloroethene; Trichloroethylene; TCE | 5 | ec:2.5.1.18 |
| cpd:C14865 |  | 2-(S-Glutathionyl)acetyl chloride | 5 | ec:2.5.1.18 |
| cpd:C14864 |  | S-(2-Chloroacetyl)glutathione | 5 | ec:2.5.1.18 |
| cpd:C14863 |  | 2-(S-Glutathionyl)acetyl glutathione | 5 | ec:2.5.1.18 |
| cpd:C14861 |  | S-(2,2-Dichloro-1-hydroxy)ethyl glutathione | 5 | ec:2.5.1.18 |
| cpd:C14859 |  | Chloroacetyl chloride | 5 | ec:2.5.1.18 |
| cpd:C14858 |  | 2,2-Dichloroacetaldehyde | 5 | ec:2.5.1.18 |
| cpd:C14857 |  | 1,1-Dichloroethylene epoxide; 2,2-Dichlorooxirane | 5 | ec:2.5.1.18 |
| cpd:C14856 |  | 7,8-Dihydro-7-hydroxy-8-S-glutathionyl-benzo[a]pyrene | 5 | ec:2.5.1.18 |
| cpd:C14855 |  | 4,5-Dihydro-4-hydroxy-5-S-glutathionyl-benzo[a]pyrene | 5 | ec:2.5.1.18 |
| cpd:C14852 |  | Benzo[a]pyrene-7,8-diol; Benzo[a]pyrene-7,8-dihydrodiol | 5 | ec:2.5.1.18 |
| cpd:C14851 |  | Benzo[a]pyrene-4,5-oxide; Benzo[a]pyrene-4,5-epoxide | 5 | ec:2.5.1.18 |
| cpd:C14848 |  | 2,3-Dihydro-2-S-glutathionyl-3-hydroxy bromobenzene | 5 | ec:2.5.1.18 |
| cpd:C14847 |  | 3,4-Dihydro-3-hydroxy-4-S-glutathionyl bromobenzene | 5 | ec:2.5.1.18 |
| cpd:C14840 |  | Bromobenzene-2,3-oxide; Bromobenzene-2,3-epoxide | 5 | ec:2.5.1.18 |
| cpd:C06010 |  | (S)-2-Acetolactate; (S)-2-Hydroxy-2-methyl-3-oxobutanoate | 4 | ec:2.2.1.6 ec:4.1.1.5 |
| cpd:C00311 |  | Isocitrate; Isocitric acid; 1-Hydroxytricarballylic acid; 1-Hydroxypropane-1,2,3-tricarboxylic acid | 4 | ec:4.2.1.3 ec:4.2.1.99 |
| cpd:C00109 |  | 2-Oxobutanoate; 2-Ketobutyric acid; 2-Oxobutyric acid; 2-Oxobutyrate; 2-Oxobutanoic acid; alpha-Ketobutyric acid; alpha-Ketobutyrate | 4 | ec:4.3.1.19 ec:2.2.1.6 |
| cpd:C00631 |  | 2-Phospho-D-glycerate; D-Glycerate 2-phosphate; 2-Phospho-(R)-glycerate | 4 | ec:5.4.2.12 |
| cpd:C00197 |  | 3-Phospho-D-glycerate; D-Glycerate 3-phosphate; 3-Phospho-(R)-glycerate; 3-Phosphoglycerate | 4 | ec:5.4.2.12 |
| cpd:C00787 |  | tRNA(Tyr) | 4 | ec:6.1.1.1 |
| cpd:C00417 |  | cis-Aconitate; cis-Aconitic acid | 4 | ec:4.2.1.3 ec:4.2.1.99 |
| cpd:C00158 |  | Citrate; Citric acid; 2-Hydroxy-1,2,3-propanetricarboxylic acid; 2-Hydroxytricarballylic acid | 4 | ec:4.2.1.3 ec:4.2.1.99 |
| cpd:C02839 |  | L-Tyrosyl-tRNA(Tyr) | 4 | ec:6.1.1.1 |
| cpd:C06567 |  | Penicilloic acid | 3 | ec:3.5.2.6 |
| cpd:C06006 |  | (S)-2-Aceto-2-hydroxybutanoate; (S)-2-Hydroxy-2-ethyl-3-oxobutanoate | 3 | ec:2.2.1.6 |
| cpd:C00119 |  | 5-Phospho-alpha-D-ribose 1-diphosphate; 5-Phosphoribosyl diphosphate; 5-Phosphoribosyl 1-pyrophosphate; PRPP | 3 | ec:2.4.2.22 ec:2.7.6.1 |
| cpd:C00118 |  | D-Glyceraldehyde 3-phosphate; (2R)-2-Hydroxy-3-(phosphonooxy)-propanal; Glyceraldehyde 3-phosphate | 3 | ec:2.2.1.1 ec:4.1.2.13 |
| cpd:C00117 |  | D-Ribose 5-phosphate; Ribose 5-phosphate | 3 | ec:2.2.1.1 ec:2.7.6.1 |
| cpd:C00279 |  | D-Erythrose 4-phosphate | 3 | ec:2.2.1.1 ec:4.1.2.13 |
| cpd:C05125 |  | 2-(alpha-Hydroxyethyl)thiamine diphosphate; 2-Hydroxyethyl-ThPP | 3 | ec:2.2.1.6 |
| cpd:C00900 |  | 2-Acetolactate | 3 | ec:2.2.1.6 |
| cpd:C00504 |  | Folate; Pteroylglutamic acid; Folic acid | 3 | ec:1.5.1.3 |
| cpd:C00068 |  | Thiamin diphosphate; Thiamine diphosphate; Thiamin pyrophosphate; TPP; ThPP | 3 | ec:2.2.1.6 |
| cpd:C04246 |  | But-2-enoyl-[acyl-carrier protein] | 3 | ec:4.2.1.59 |
| cpd:C04633 |  | (3R)-3-Hydroxypalmitoyl-[acyl-carrier protein]; (R)-3-Hydroxypalmitoyl-[acyl-carrier protein]; (3R)-3-Hydroxyhexadecanoyl-[acyl-carrier protein]; (R)-3-Hydroxyhexadecanoyl-[acyl-carrier protein] | 3 | ec:4.2.1.59 |
| cpd:C15815 |  | C15815; Thiamine biosynthesis intermediate 6 | 3 |  |
| cpd:C04620 |  | (3R)-3-Hydroxyoctanoyl-[acyl-carrier protein]; (R)-3-Hydroxyoctanoyl-[acyl-carrier protein] | 3 | ec:4.2.1.59 |
| cpd:C15809 |  | Iminoglycine; Iminoacetic acid; 2-Iminoacetate | 3 |  |
| cpd:C04619 |  | (3R)-3-Hydroxydecanoyl-[acyl-carrier protein]; (R)-3-Hydroxydecanoyl-[acyl-carrier protein] | 3 | ec:4.2.1.59 |
| cpd:C04618 |  | (3R)-3-Hydroxybutanoyl-[acyl-carrier protein]; (R)-3-Hydroxybutanoyl-[acyl-carrier protein] | 3 | ec:4.2.1.59 |
| cpd:C00395 |  | Penicillin; Penam | 3 | ec:3.5.2.6 |
| cpd:C00415 |  | Dihydrofolate; Dihydrofolic acid; 7,8-Dihydrofolate; 7,8-Dihydrofolic acid; 7,8-Dihydropteroylglutamate | 3 | ec:1.5.1.3 |
| cpd:C00010 |  | CoA; Coenzyme A; CoA-SH | 3 | ec:2.3.1.30 ec:6.2.1.3 |
| cpd:C03287 |  | L-Glutamyl 5-phosphate; L-Glutamate 5-phosphate | 3 | ec:2.7.2.11 ec:1.2.1.41 |
| cpd:C05763 |  | trans-Hexadec-2-enoyl-[acp]; trans-Hexadec-2-enoyl-[acyl-carrier protein]; (2E)-Hexadecenoyl-[acp] | 3 | ec:4.2.1.59 |
| cpd:C05760 |  | trans-Tetradec-2-enoyl-[acp]; trans-Tetradec-2-enoyl-[acyl-carrier protein]; (2E)-Tetradecenoyl-[acp] | 3 | ec:4.2.1.59 |
| cpd:C00154 |  | Palmitoyl-CoA; Hexadecanoyl-CoA | 3 | ec:6.2.1.3 |
| cpd:C05758 |  | trans-Dodec-2-enoyl-[acp]; trans-Dodec-2-enoyl-[acyl-carrier protein]; (2E)-Dodecenoyl-[acp] | 3 | ec:4.2.1.59 |
| cpd:C05757 |  | (R)-3-Hydroxydodecanoyl-[acp]; (R)-3-Hydroxydodecanoyl-[acyl-carrier protein]; D-3-Hydroxydodecanoyl-[acp]; D-3-Hydroxydodecanoyl-[acyl-carrier protein] | 3 | ec:4.2.1.59 |
| cpd:C05754 |  | trans-Dec-2-enoyl-[acp]; trans-Dec-2-enoyl-[acyl-carrier protein]; trans-2-Decenoyl-[acyl-carrier protein]; (2E)-Decenoyl-[acp] | 3 | ec:4.2.1.59 |
| cpd:C05751 |  | trans-Oct-2-enoyl-[acp]; trans-Oct-2-enoyl-[acyl-carrier protein]; Oct-2-enoyl-[acyl-carrier protein]; 2-Octenoyl-[acyl-carrier protein]; (2E)-Octenoyl-[acp] | 3 | ec:4.2.1.59 |
| cpd:C04688 |  | (3R)-3-Hydroxytetradecanoyl-[acyl-carrier protein]; (R)-3-Hydroxytetradecanoyl-[acyl-carrier protein]; beta-Hydroxymyristyl-[acyl-carrier protein]; HMA | 3 | ec:4.2.1.59 |
| cpd:C04327 |  | 4-Methyl-5-(2-phosphoethyl)-thiazole; 4-Methyl-5-(2-phosphono-oxyethyl)-thiazole | 3 |  |
| cpd:C05748 |  | trans-Hex-2-enoyl-[acp]; trans-Hex-2-enoyl-[acyl-carrier protein]; (2E)-Hexenoyl-[acp] | 3 | ec:4.2.1.59 |
| cpd:C05747 |  | (R)-3-Hydroxyhexanoyl-[acp]; (R)-3-Hydroxyhexanoyl-[acyl-carrier protein]; D-3-Hydroxyhexanoyl-[acp]; D-3-Hydroxyhexanoyl-[acyl-carrier protein] | 3 | ec:4.2.1.59 |
| cpd:C05345 |  | beta-D-Fructose 6-phosphate | 2 | ec:2.2.1.1 |
| cpd:C00136 |  | Butanoyl-CoA; Butyryl-CoA | 2 |  |
| cpd:C00097 |  | L-Cysteine; L-2-Amino-3-mercaptopropionic acid | 2 |  |
| cpd:C00094 |  | Sulfite; Sulfurous acid | 2 | ec:1.14.11.17 |
| cpd:C00093 |  | sn-Glycerol 3-phosphate; Glycerophosphoric acid; D-Glycerol 1-phosphate | 2 | ec:2.7.1.30 ec:3.1.4.46 |
| cpd:C01352 |  | FADH2 | 2 |  |
| cpd:C00487 |  | Carnitine; gamma-Trimethyl-hydroxybutyrobetaine; 3-Hydroxy-4-trimethylammoniobutanoate | 2 | ec:1.14.11.1 |
| cpd:C01944 |  | Octanoyl-CoA | 2 |  |
| cpd:C05729 |  | R-S-Cysteinylglycine | 2 |  |
| cpd:C00085 |  | D-Fructose 6-phosphate; D-Fructose 6-phosphoric acid; Neuberg ester | 2 | ec:2.2.1.1 |
| cpd:C00877 |  | Crotonoyl-CoA; Crotonyl-CoA; 2-Butenoyl-CoA; trans-But-2-enoyl-CoA; But-2-enoyl-CoA; (E)-But-2-enoyl-CoA | 2 |  |
| cpd:C05726 |  | S-Substituted L-cysteine; R-S-Cysteine | 2 |  |
| cpd:C00084 |  | Acetaldehyde; Ethanal | 2 | ec:4.1.3.39 ec:1.1.1.1 |
| cpd:C01931 |  | L-Lysyl-tRNA; L-Lysyl-tRNA(Lys) | 2 | ec:6.1.1.6 |
| cpd:C03221 |  | 2-trans-Dodecenoyl-CoA; (2E)-Dodec-2-enoyl-CoA; (2E)-Dodecenoyl-CoA | 2 |  |
| cpd:C05276 |  | trans-Oct-2-enoyl-CoA; (2E)-Octenoyl-CoA | 2 |  |
| cpd:C05275 |  | trans-Dec-2-enoyl-CoA; (2E)-Decenoyl-CoA | 2 |  |
| cpd:C05274 |  | Decanoyl-CoA | 2 |  |
| cpd:C05273 |  | trans-Tetradec-2-enoyl-CoA; (2E)-Tetradecenoyl-CoA | 2 |  |
| cpd:C05272 |  | trans-Hexadec-2-enoyl-CoA; trans-2-Hexadecenoyl-CoA; (2E)-Hexadecenoyl-CoA | 2 |  |
| cpd:C05271 |  | trans-Hex-2-enoyl-CoA; (2E)-Hexenoyl-CoA | 2 |  |
| cpd:C05270 |  | Hexanoyl-CoA | 2 |  |
| cpd:C00655 |  | Xanthosine 5'-phosphate; Xanthylic acid; XMP; (9-D-Ribosylxanthine)-5'-phosphate | 2 | ec:2.4.2.22 |
| cpd:C04593 |  | (2S,3R)-3-Hydroxybutane-1,2,3-tricarboxylate; Methylisocitrate; Methylisocitric acid | 2 | ec:4.2.1.3 ec:4.2.1.99 |
| cpd:C00245 |  | Taurine; 2-Aminoethanesulfonic acid; Aminoethylsulfonic acid | 2 | ec:2.6.1.55 ec:1.14.11.17 |
| cpd:C00242 |  | Guanine; 2-Amino-6-hydroxypurine | 2 | ec:2.4.2.22 |
| cpd:C00047 |  | L-Lysine; Lysine acid; 2,6-Diaminohexanoic acid | 2 | ec:6.1.1.6 |
| cpd:C04225 |  | (Z)-But-2-ene-1,2,3-tricarboxylate; cis-2-Methylaconitate | 2 | ec:4.2.1.3 ec:4.2.1.99 |
| cpd:C00593 |  | Sulfoacetaldehyde; 2-Sulfoacetaldehyde | 2 | ec:2.6.1.55 ec:1.1.1.313 |
| cpd:C00430 |  | 5-Aminolevulinate; 5-Amino-4-oxopentanoate; 5-Amino-4-oxovaleric acid | 2 | ec:4.2.1.24 |
| cpd:C00232 |  | Succinate semialdehyde; Succinic semialdehyde; 4-Oxobutanoate | 2 | ec:1.2.1.16 ec:1.2.1.24 |
| cpd:C00231 |  | D-Xylulose 5-phosphate | 2 | ec:2.2.1.1 |
| cpd:C01646 |  | tRNA(Lys) | 2 | ec:6.1.1.6 |
| cpd:C00024 |  | Acetyl-CoA; Acetyl coenzyme A | 2 | ec:2.3.1.30 |
| cpd:C00979 |  | O-Acetyl-L-serine; O3-Acetyl-L-serine | 2 | ec:2.3.1.30 |
| cpd:C01832 |  | Lauroyl-CoA; Lauroyl coenzyme A; Dodecanoyl-CoA | 2 |  |
| cpd:C05382 |  | Sedoheptulose 7-phosphate; D-Sedoheptulose 7-phosphate; D-altro-Heptulose 7-phosphate; altro-Heptulose 7-phosphate | 2 | ec:2.2.1.1 |
| cpd:C00016 |  | FAD; Flavin adenine dinucleotide | 2 |  |
| cpd:C12215 |  | Iminoerythrose 4-phosphate; Imino-D-erythrose 4-phosphate | 2 | ec:2.2.1.1 |
| cpd:C12214 |  | Aminofructose 6-phosphate; Amino-D-fructose 6-phosphate | 2 | ec:2.2.1.1 |
| cpd:C01181 |  | 4-Trimethylammoniobutanoate; Butyro-betaine; gamma-Butyrobetaine | 2 | ec:1.14.11.1 |
| cpd:C01419 |  | Cys-Gly; L-Cysteinylglycine | 2 |  |
| cpd:C02593 |  | Tetradecanoyl-CoA; Myristoyl-CoA | 2 |  |
| cpd:C00144 |  | GMP; Guanosine 5'-phosphate; Guanosine monophosphate; Guanosine 5'-monophosphate; Guanylic acid | 2 | ec:2.4.2.22 |
| cpd:C00931 |  | Porphobilinogen | 2 | ec:4.2.1.24 |
| cpd:C01165 |  | L-Glutamate 5-semialdehyde; L-Glutamate gamma-semialdehyde | 1 | ec:1.2.1.41 |
| cpd:C01163 |  | 3-Carboxy-cis,cis-muconate; beta-Carboxy-cis,cis-muconate; cis,cis-Butadiene-1,2,4-tricarboxylate | 1 | ec:1.13.11.3 |
| cpd:C06762 |  | 4-Hydroxy-2-oxohexanoic acid; 4-Hydroxy-2-oxohexanoate | 1 | ec:4.1.3.39 |
| cpd:C00099 |  | beta-Alanine; 3-Aminopropionic acid; 3-Aminopropanoate | 1 | ec:2.6.1.55 |
| cpd:C00123 |  | L-Leucine; 2-Amino-4-methylvaleric acid; (2S)-alpha-2-Amino-4-methylvaleric acid; (2S)-alpha-Leucine | 1 | ec:2.6.1.42 |
| cpd:C02376 |  | 5-Methylcytosine | 1 | ec:3.5.4.1 |
| cpd:C01346 |  | dUDP; 2'-Deoxyuridine 5'-diphosphate | 1 | ec:1.17.4.1 |
| cpd:C00479 |  | Propanal; Propionaldehyde | 1 | ec:4.1.3.39 |
| cpd:C00671 |  | (S)-3-Methyl-2-oxopentanoic acid; (S)-3-Methyl-2-oxopentanoate; (3S)-3-Methyl-2-oxopentanoic acid; (3S)-3-Methyl-2-oxopentanoate | 1 | ec:2.6.1.42 |
| cpd:C00670 |  | sn-Glycero-3-phosphocholine; Glycerophosphocholine | 1 | ec:3.1.4.46 |
| cpd:C00473 |  | Retinol; all-trans-Retinol; Vitamin A; Vitamin A1 | 1 | ec:1.1.1.1 |
| cpd:C02565 |  | N-Methylhydantoin; N-Methylimidazolidine-2,4-dione | 1 | ec:3.5.4.1 |
| cpd:C00116 |  | Glycerol; Glycerin; 1,2,3-Trihydroxypropane; 1,2,3-Propanetriol | 1 | ec:2.7.1.30 |
| cpd:C05123 |  | 2-Hydroxyethanesulfonate; 2-Hydroxyethanesulfonic acid; 2-Hydroxyethane-1-sulfonic acid; Isethionic acid; Isethionate | 1 | ec:1.1.1.313 |
| cpd:C00112 |  | CDP; Cytidine 5'-diphosphate; Cytidine diphosphate | 1 | ec:1.17.4.1 |
| cpd:C00111 |  | Glycerone phosphate; Dihydroxyacetone phosphate | 1 | ec:4.1.2.13 |
| cpd:C00079 |  | L-Phenylalanine; (S)-alpha-Amino-beta-phenylpropionic acid | 1 | ec:2.6.1.9 |
| cpd:C00077 |  | L-Ornithine; (S)-2,5-Diaminovaleric acid; (S)-2,5-Diaminopentanoic acid; (S)-2,5-Diaminopentanoate | 1 | ec:4.3.1.12 |
| cpd:C00705 |  | dCDP; 2'-Deoxycytidine diphosphate; 2'-Deoxycytidine 5'-diphosphate | 1 | ec:1.17.4.1 |
| cpd:C00071 |  | Aldehyde; RCHO | 1 | ec:1.1.1.1 |
| cpd:C03589 |  | 4-Hydroxy-2-oxopentanoate; 4-Hydroxy-2-oxovalerate | 1 | ec:4.1.3.39 |
| cpd:C03586 |  | 2-Oxo-2,3-dihydrofuran-5-acetate; 3-Oxoadipate enol-lactone; 4,5-Dihydro-5-oxofuran-2-acetate; 5-Oxo-4,5-dihydrofuran-2-acetate | 1 | ec:4.1.1.44 |
| cpd:C00469 |  | Ethanol; Ethyl alcohol; Methylcarbinol | 1 | ec:1.1.1.1 |
| cpd:C06899 |  | Chloral hydrate | 1 | ec:1.1.1.1 |
| cpd:C00106 |  | Uracil | 1 | ec:3.5.4.1 |
| cpd:C01094 |  | D-Fructose 1-phosphate | 1 | ec:4.1.2.13 |
| cpd:C06735 |  | Aminoacetaldehyde; 2-Aminoacetaldehyde | 1 | ec:1.14.11.17 |
| cpd:C00256 |  | (R)-Lactate; D-Lactate; D-Lactic acid; D-2-Hydroxypropanoic acid; D-2-Hydroxypropionic acid | 1 | ec:1.1.1.28 |
| cpd:C00059 |  | Sulfate; Sulfuric acid | 1 |  |
| cpd:C01278 |  | 2-Carboxy-2,5-dihydro-5-oxofuran-2-acetate; 5-Carboxy-2,5-dihydro-2-oxofuran-5-acetate; 4-Carboxymuconolactone; gamma-Carboxymuconolactone | 1 | ec:4.1.1.44 |
| cpd:C00447 |  | Sedoheptulose 1,7-bisphosphate; D-Sedoheptulose 1,7-bisphosphate; D-altro-Heptulose 1,7-biphosphate | 1 | ec:4.1.2.13 |
| cpd:C00249 |  | Hexadecanoic acid; Hexadecanoate; Hexadecylic acid; Palmitic acid; Palmitate; Cetylic acid | 1 | ec:6.2.1.3 |
| cpd:C11436 |  | 2-Phospho-4-(cytidine 5'-diphospho)-2-C-methyl-D-erythritol | 1 | ec:2.7.1.148 |
| cpd:C11435 |  | 4-(Cytidine 5'-diphospho)-2-C-methyl-D-erythritol | 1 | ec:2.7.1.148 |
| cpd:C00041 |  | L-Alanine; L-2-Aminopropionic acid; L-alpha-Alanine | 1 | ec:1.4.1.1 |
| cpd:C01267 |  | 3-(Imidazol-4-yl)-2-oxopropyl phosphate; Imidazole-acetol phosphate | 1 | ec:2.6.1.9 |
| cpd:C00791 |  | Creatinine; 1-Methylglycocyamidine | 1 | ec:3.5.4.1 |
| cpd:C01100 |  | L-Histidinol phosphate | 1 | ec:2.6.1.9 |
| cpd:C16596 |  | 5-Phenyl-1,3-oxazinane-2,4-dione | 1 | ec:1.1.1.1 |
| cpd:C16595 |  | 4-Hydroxy-5-phenyltetrahydro-1,3-oxazin-2-one | 1 | ec:1.1.1.1 |
| cpd:C00233 |  | 4-Methyl-2-oxopentanoate; 2-Oxoisocaproate | 1 | ec:2.6.1.42 |
| cpd:C00230 |  | 3,4-Dihydroxybenzoate; 3,4-Dihydroxybenzoic acid; Protocatechuate; Protocatechuic acid | 1 | ec:1.13.11.3 |
| cpd:C00035 |  | GDP; Guanosine 5'-diphosphate; Guanosine diphosphate | 1 | ec:1.17.4.1 |
| cpd:C07490 |  | Trichloroethanol; 2,2,2-Trichloroethanol | 1 | ec:1.1.1.1 |
| cpd:C00226 |  | Primary alcohol; 1-Alcohol | 1 | ec:1.1.1.1 |
| cpd:C16587 |  | 3-Carbamoyl-2-phenylpropionaldehyde | 1 | ec:1.1.1.1 |
| cpd:C16586 |  | 2-Phenyl-1,3-propanediol monocarbamate | 1 | ec:1.1.1.1 |
| cpd:C00222 |  | 3-Oxopropanoate; Malonate semialdehyde | 1 | ec:2.6.1.55 |
| cpd:C00189 |  | Ethanolamine; Aminoethanol; 2-Hydroxyethylamine | 1 | ec:3.1.4.46 |
| cpd:C00188 |  | L-Threonine; 2-Amino-3-hydroxybutyric acid | 1 | ec:4.3.1.19 |
| cpd:C00380 |  | Cytosine | 1 | ec:3.5.4.1 |
| cpd:C02909 |  | (2-Naphthyl)methanol; 2-Naphthalenemethanol; 2-Hydroxymethylnaphthalene | 1 | ec:1.1.1.1 |
| cpd:C14099 |  | 2-Naphthaldehyde; 2-Naphthalenecarboxaldehyde | 1 | ec:1.1.1.1 |
| cpd:C00183 |  | L-Valine; 2-Amino-3-methylbutyric acid | 1 | ec:2.6.1.42 |
| cpd:C00810 |  | (R)-Acetoin; (R)-2-Acetoin; (R)-3-Hydroxy-2-butanone; (R)-Dimethylketol; (R)-3-Hydroxybutan-2-one | 1 | ec:4.1.1.5 |
| cpd:C14090 |  | 1-Naphthaldehyde; 1-Formylnaphthalene | 1 | ec:1.1.1.1 |
| cpd:C00577 |  | D-Glyceraldehyde | 1 | ec:4.1.2.13 |
| cpd:C00376 |  | Retinal; Vitamin A aldehyde; Retinene; all-trans-Retinal; all-trans-Vitamin A aldehyde; all-trans-Retinene | 1 | ec:1.1.1.1 |
| cpd:C00178 |  | Thymine; 5-Methyluracil | 1 | ec:3.5.4.1 |
| cpd:C00015 |  | UDP; Uridine 5'-diphosphate | 1 | ec:1.17.4.1 |
| cpd:C14089 |  | 1-Hydroxymethylnaphthalene; 1-Naphthalenemethanol | 1 | ec:1.1.1.1 |
| cpd:C01236 |  | D-Glucono-1,5-lactone 6-phosphate; 6-Phospho-D-glucono-1,5-lactone | 1 | ec:3.1.1.31 |
| cpd:C00407 |  | L-Isoleucine; 2-Amino-3-methylvaleric acid | 1 | ec:2.6.1.42 |
| cpd:C01233 |  | sn-Glycero-3-phosphoethanolamine; Glycerophosphoethanolamine | 1 | ec:3.1.4.46 |
| cpd:C05577 |  | 3,4-Dihydroxymandelaldehyde; 3,4-Dihydroxyphenylglycolaldehyde | 1 | ec:1.1.1.1 |
| cpd:C05576 |  | 3,4-Dihydroxyphenylethyleneglycol | 1 | ec:1.1.1.1 |
| cpd:C17234 |  | 2-Aminobut-2-enoate; (2Z)-2-Aminobut-2-enoic acid; 2-Ammoniobut-2-enoate | 1 | ec:4.3.1.19 |
| cpd:C00206 |  | dADP; 2'-Deoxyadenosine 5'-diphosphate | 1 | ec:1.17.4.1 |
| cpd:C05378 |  | beta-D-Fructose 1,6-bisphosphate | 1 | ec:4.1.2.13 |
| cpd:C01031 |  | S-Formylglutathione | 1 | ec:3.1.2.12 |
| cpd:C00204 |  | 2-Dehydro-3-deoxy-D-gluconate; 2-Keto-3-deoxy-D-gluconate | 1 | ec:1.1.1.125 |
| cpd:C00361 |  | dGDP; 2'-Deoxyguanosine 5'-diphosphate | 1 | ec:1.17.4.1 |
| cpd:C00166 |  | Phenylpyruvate; Phenylpyruvic acid; alpha-Ketohydrocinnamic acid; keto-Phenylpyruvate; 3-Phenyl-2-oxopropanoate; 2-Oxo-3-phenylpropanoate | 1 | ec:2.6.1.9 |
| cpd:C04349 |  | (4S)-4,6-Dihydroxy-2,5-dioxohexanoate; 3-Deoxy-D-glycero-2,5-hexodiulosonate; 2,5-Diketo-3-deoxy-D-gluconate | 1 | ec:1.1.1.125 |
| cpd:C01424 |  | Gallate; Gallic acid; 3,4,5-Trihydroxybenzoic acid; 3,4,5-Trihydroxybenzoate; Pyrogallol-5-carboxylic acid | 1 | ec:1.13.11.3 |
| cpd:C03671 |  | 2-Pyrone-4,6-dicarboxylate | 1 | ec:1.13.11.3 |
| cpd:C00354 |  | D-Fructose 1,6-bisphosphate | 1 | ec:4.1.2.13 |
| cpd:C16551 |  | Alcophosphamide | 1 | ec:1.1.1.1 |
| cpd:C01179 |  | 3-(4-Hydroxyphenyl)pyruvate; 4-Hydroxyphenylpyruvate; p-Hydroxyphenylpyruvic acid | 1 | ec:2.6.1.9 |
| cpd:C05359 |  | e-; Electron | 1 |  |
| cpd:C00345 |  | 6-Phospho-D-gluconate | 1 | ec:3.1.1.31 |
| cpd:C00343 |  | Thioredoxin disulfide; Oxidized thioredoxin; Thioredoxin sulfide | 1 | ec:1.17.4.1 |
| cpd:C00342 |  | Thioredoxin; Reduced thioredoxin | 1 | ec:1.17.4.1 |
| cpd:C16348 |  | cis-3-Chloroallyl aldehyde; cis-3-Chloro-2-propenal | 1 | ec:1.1.1.1 |
| cpd:C00148 |  | L-Proline; 2-Pyrrolidinecarboxylic acid | 1 | ec:4.3.1.12 |
| cpd:C06613 |  | trans-3-Chloroallyl aldehyde; trans-3-Chloro-2-propenal | 1 | ec:1.1.1.1 |
| cpd:C06612 |  | cis-3-Chloro-2-propene-1-ol; cis-3-Chloroallyl alcohol | 1 | ec:1.1.1.1 |
| cpd:C06611 |  | trans-3-Chloro-2-propene-1-ol; trans-3-Chloroallyl alcohol | 1 | ec:1.1.1.1 |
| cpd:C00141 |  | 3-Methyl-2-oxobutanoic acid; 3-Methyl-2-oxobutyric acid; 3-Methyl-2-oxobutanoate; 2-Oxo-3-methylbutanoate; 2-Oxoisovalerate; 2-Oxoisopentanoate; alpha-Ketovaline; 2-Ketovaline; 2-Keto-3-methylbutyric acid | 1 | ec:2.6.1.42 |

  
**Over-represented Pathway Summary**: Collection of the KEGG metabolic pathways containing the proteins identified in the "Over-represented Metabolite Summary" ranked by the highest number of hits per pathway  

| Pathway ID | EC | EC Frequency | Name |
| --- | --- | --- | --- |
| map00260 | ec:1.1.1.1 ec:4.3.1.19 ec:1.5.3.1 ec:4.3.1.17 ec:5.4.2.12 ec:1.1.99.1 ec:2.1.2.1 ec:1.2.1.8 | 233 | path:map00260 Glycine, serine and threonine metabolism |
| map00680 | ec:1.2.1.46 ec:5.4.2.12 ec:4.1.2.13 ec:2.1.2.1 ec:3.1.2.12 | 27 | path:map00680 Methane metabolism |
| map00230 | ec:2.7.2.2 ec:1.17.4.1 ec:2.7.6.1 ec:2.7.7.7 ec:2.4.2.22 | 24 | path:map00230 Purine metabolism |
| map00330 | ec:4.3.1.12 ec:2.7.2.2 ec:2.7.2.11 ec:3.5.4.1 ec:1.2.1.41 | 23 | path:map00330 Arginine and proline metabolism |
| map00910 | ec:2.7.2.2 | 18 | path:map00910 Nitrogen metabolism |
| map00625 | ec:1.1.1.1 ec:1.2.1.46 | 15 | path:map00625 Chloroalkane and chloroalkene degradation |
| map00630 | ec:4.2.1.3 ec:2.1.2.1 | 11 | path:map00630 Glyoxylate and dicarboxylate metabolism |
| map00670 | ec:1.5.1.3 ec:2.1.2.1 | 10 | path:map00670 One carbon pool by folate |
| map00270 | ec:2.3.1.30 ec:4.3.1.17 | 10 | path:map00270 Cysteine and methionine metabolism |
| map00650 | ec:1.1.1.76 ec:1.2.1.24 ec:2.2.1.6 ec:4.1.1.5 ec:1.1.1.304 ec:1.2.1.16 | 10 | path:map00650 Butanoate metabolism |
| map00750 | ec:1.4.3.5 ec:2.7.1.35 | 7 | path:map00750 Vitamin B6 metabolism |
| map00460 | ec:2.1.2.1 | 7 | path:map00460 Cyanoamino acid metabolism |
| map00480 | ec:2.5.1.18 ec:1.17.4.1 | 6 | path:map00480 Glutathione metabolism |
| map00982 | ec:1.1.1.1 ec:2.5.1.18 | 6 | path:map00982 Drug metabolism - cytochrome P450 |
| map00980 | ec:1.1.1.1 ec:2.5.1.18 | 6 | path:map00980 Metabolism of xenobiotics by cytochrome P450 |
| map00970 | ec:6.1.1.1 ec:6.1.1.6 | 6 | path:map00970 Aminoacyl-tRNA biosynthesis |
| map00010 | ec:1.1.1.1 ec:5.4.2.12 ec:4.1.2.13 | 6 | path:map00010 Glycolysis / Gluconeogenesis |
| map00030 | ec:2.7.6.1 ec:3.1.1.31 ec:2.2.1.1 ec:4.1.2.13 | 5 | path:map00030 Pentose phosphate pathway |
| map00250 | ec:1.2.1.24 ec:1.4.1.1 ec:1.2.1.16 | 5 | path:map00250 Alanine, aspartate and glutamate metabolism |
| map00290 | ec:2.6.1.42 ec:2.2.1.6 ec:4.3.1.19 | 5 | path:map00290 Valine, leucine and isoleucine biosynthesis |
| map00770 | ec:2.6.1.42 ec:2.2.1.6 | 4 | path:map00770 Pantothenate and CoA biosynthesis |
| map00350 | ec:1.1.1.1 ec:2.6.1.9 ec:1.2.1.16 | 4 | path:map00350 Tyrosine metabolism |
| map00920 | ec:1.14.11.17 ec:3.6.3.36 ec:2.3.1.30 | 4 | path:map00920 Sulfur metabolism |
| map00020 | ec:4.2.1.3 | 4 | path:map00020 Citrate cycle (TCA cycle) |
| map00660 | ec:2.2.1.6 ec:4.1.1.5 | 4 | path:map00660 C5-Branched dibasic acid metabolism |
| map00240 | ec:1.17.4.1 ec:3.5.4.1 ec:2.7.7.7 | 4 | path:map00240 Pyrimidine metabolism |
| map00720 | ec:4.2.1.3 | 4 | path:map00720 Carbon fixation pathways in prokaryotes |
| map00430 | ec:1.14.11.17 ec:2.6.1.55 ec:1.4.1.1 ec:1.1.1.313 | 4 | path:map00430 Taurine and hypotaurine metabolism |
| map00710 | ec:2.2.1.1 ec:4.1.2.13 | 3 | path:map00710 Carbon fixation in photosynthetic organisms |
| map00061 | ec:4.2.1.59 | 3 | path:map00061 Fatty acid biosynthesis |
| map00790 | ec:1.5.1.3 | 3 | path:map00790 Folate biosynthesis |
| map00312 | ec:3.5.2.6 | 3 | path:map00312 beta-Lactam resistance |
| map00311 | ec:3.5.2.6 | 3 | path:map00311 Penicillin and cephalosporin biosynthesis |
| map00780 | ec:4.2.1.59 | 3 | path:map00780 Biotin metabolism |
| map00362 | ec:4.1.3.39 ec:4.1.1.44 ec:1.13.11.3 | 3 | path:map00362 Benzoate degradation |
| map00640 | ec:4.2.1.99 | 2 | path:map00640 Propanoate metabolism |
| map00860 | ec:4.2.1.24 | 2 | path:map00860 Porphyrin and chlorophyll metabolism |
| map00310 | ec:1.14.11.1 | 2 | path:map00310 Lysine degradation |
| map00360 | ec:2.6.1.9 ec:4.1.3.39 | 2 | path:map00360 Phenylalanine metabolism |
| map00071 | ec:1.1.1.1 ec:6.2.1.3 | 2 | path:map00071 Fatty acid degradation |
| map00966 | ec:2.6.1.42 | 1 | path:map00966 Glucosinolate biosynthesis |
| map00900 | ec:2.7.1.148 | 1 | path:map00900 Terpenoid backbone biosynthesis |
| map04660 | ec:3.1.3.16 | 1 | path:map04660 T cell receptor signaling pathway |
| map00960 | ec:2.6.1.9 | 1 | path:map00960 Tropane, piperidine and pyridine alkaloid biosynthesis |
| map00830 | ec:1.1.1.1 | 1 | path:map00830 Retinol metabolism |
| map00280 | ec:2.6.1.42 | 1 | path:map00280 Valine, leucine and isoleucine degradation |
| map00410 | ec:2.6.1.55 | 1 | path:map00410 beta-Alanine metabolism |
| map00340 | ec:2.6.1.9 | 1 | path:map00340 Histidine metabolism |
| map00051 | ec:4.1.2.13 | 1 | path:map00051 Fructose and mannose metabolism |
| map00564 | ec:3.1.4.46 | 1 | path:map00564 Glycerophospholipid metabolism |
| map00561 | ec:2.7.1.30 | 1 | path:map00561 Glycerolipid metabolism |
| map00401 | ec:2.6.1.9 | 1 | path:map00401 Novobiocin biosynthesis |
| map00400 | ec:2.6.1.9 | 1 | path:map00400 Phenylalanine, tyrosine and tryptophan biosynthesis |
| map00626 | ec:1.1.1.1 | 1 | path:map00626 Naphthalene degradation |
| map00624 | ec:1.13.11.3 | 1 | path:map00624 Polycyclic aromatic hydrocarbon degradation |
| map00622 | ec:4.1.3.39 | 1 | path:map00622 Xylene degradation |
| map00621 | ec:4.1.3.39 | 1 | path:map00621 Dioxin degradation |
| map00620 | ec:1.1.1.28 | 1 | path:map00620 Pyruvate metabolism |
| map00040 | ec:1.1.1.125 | 1 | path:map00040 Pentose and glucuronate interconversions |

  
Analysis performed on 2014/02/14 20:32:02
