# Supplementary material for: Automated genomic context analysis and experimental validation platform for discovery of prokaryote transcriptional regulator functions
Source: BMC Genomics. 2014 Dec 18;15(1):1142. doi: 10.1186/1471-2164-15-1142 (PMC4349456; doi:10.1186/1471-2164-15-1142)
Supplement: Supplementary file 3 — Additional file 3: Result CatR. Function Discovery V1.0 output (.html format) for the benzoate degradation regulator (CatR, Bxe_ A2107). For detailed instructions on how to analyze the results please refer to the Function Discovery V1.0, a gene neighborhood analysis tool section in the Results part of the main text. (HTML 258 KB) [file 12864_2014_6995_MOESM3_ESM.html]

```
ENTRY       Bxe_A2107         CDS       T00340
NAME        catR
DEFINITION  LysR family transcriptional regulator
ORGANISM    bxe  Burkholderia xenovorans
POSITION    1:2601535..2602422
MOTIF       Pfam: LysR_substrate HTH_1 HTH_30
DBLINKS     NCBI-GI: 91783707
            NCBI-GeneID: 4005099
            JGI: BxeA2107
            UniProt: Q13YH8
AASEQ       295
            MELRQLRYFIAVAEEMNITRAANRLHMTQPPLSRQIQQIEENVGLPLFERGSRPLRLTEA
            GRIFYAQAKRLIDEADELAPLTRRLAQLAERVVIGFVPSTLYGALPAVIRAFREAAPHIE
            LSLIEMFTIEQLGALKGGRIDVGFGRLRFDDAQLAREVLVEERMIAALPQDHPLARQKKA
            LTLAALAQETLIVYPSTPRPSYADQQLSAMHDHALEPKAIHEVRELQTALGLVAAQVGVC
            LVPESVEGLRAHGVVYRPIPAANVASPIIMSRRLQDESPTTTLLCSLARELFKRV
NTSEQ       888
            atggaacttcgccaactccgctatttcatcgcggtcgcggaagaaatgaacatcacgcgg
            gccgccaatcgcctgcacatgacgcagccgccgctcagccggcagatccagcagatcgaa
            gaaaacgtcggcctgccgctgttcgaacgcggctcgcggccattgcggctgaccgaagcc
            ggccgcattttctatgcgcaggcaaagcgcctgatcgatgaagcggacgagctcgccccg
            ctcacgcggcgcctcgcgcaactggccgagcgcgtcgtgatcggcttcgtgccgtcgacg
            ctttatggcgcgctgcccgccgtgatccgtgcgtttcgtgaagccgcgccgcatatcgag
            ctctcgctgatcgaaatgttcacgatcgagcagctcggggcgctcaagggcggccgcatc
            gacgttggcttcgggcgcctgcgcttcgacgacgcgcaactcgcgcgcgaagtcctcgtc
            gaagaacggatgattgcggcgctgccgcaagaccatccgctggcgcgccagaagaaggcg
            ctgacattggcggcgctcgcgcaggaaaccctgatcgtctatccgagcacgccgcggccg
            agctatgccgaccagcagctatcggccatgcacgatcatgcattggagccgaaggcgatc
            catgaagtgcgggaattgcagaccgcgttgggtctggtcgcggcgcaggtgggcgtgtgc
            ctggtgccggagagcgtggagggtttgcgcgcgcacggcgtggtgtaccggccgattccg
            gcggccaacgttgcttcgccgatcatcatgagccgccgtttgcaggacgaatcgccgacc
            accacgttgctctgttcgctcgcacgtgagctgttcaaacgggtttga
///
```

  
**Homolog ID**: Table of closest homologs  

```
                 Homologs                                       len   identity overlap
---------------------------------------------------------------------------------
bpy:Bphyt_2154 LysR family transcriptional regulator          295     0.949    294 
bge:BC1002_1617 transcriptional regulator, LysR family        295     0.939    295 
bph:Bphy_4030 LysR family transcriptional regulator           296     0.810    294 
bvi:Bcep1808_4160 LysR family transcriptional regulator       296     0.774    292 
bur:Bcep18194_B2327 LysR family transcriptional regulat       296     0.784    291 
bte:BTH_II0486 transcriptional regulator CatR                 295     0.784    291 
bac:BamMC406_3663 LysR family transcriptional regulator       296     0.773    291 
bam:Bamb_5500 LysR family transcriptional regulator           296     0.770    291 
bma:BMAA0201 transcriptional regulator CatR                   295     0.777    291 
bmv:BMASAVP1_1376 cat operon transcriptional activator        295     0.777    291 
bch:Bcen2424_3771 LysR family transcriptional regulator       296     0.773    291 
bcm:Bcenmc03_3752 LysR family transcriptional regulator       296     0.773    291 
bcn:Bcen_4592 LysR family transcriptional regulator           296     0.773    291 
bpd:BURPS668_A2709 cat operon transcriptional activator       295     0.773    291 
bpl:BURPS1106A_A2565 cat operon transcriptional activat       295     0.773    291 
bpm:BURPS1710b_A0985 transcriptional regulator CatR           295     0.773    291 
bps:BPSS1890 LysR family transcriptional regulator            295     0.773    291 
bml:BMA10229_1573 cat operon transcriptional activator        295     0.773    291 
bmn:BMA10247_A0232 cat operon transcriptional activator       295     0.773    291 
bcj:BCAM0806 putative cat operon regulatory protein           296     0.773    291 
bmj:BMULJ_03555 LysR family transcriptional regulator         298     0.756    291 
bmu:Bmul_4959 LysR family transcriptional regulator           298     0.756    291 
pna:Pnap_2114 LysR family transcriptional regulator           313     0.553    293 
met:M446_1323 LysR family transcriptional regulator           302     0.517    296 
sjp:SJA_C1-25210 LysR family transcriptional regulator        303     0.517    290 
nar:Saro_0537 LysR family transcriptional regulator           308     0.502    285 
bgl:bglu_2g07130 LysR family transcriptional regulator        293     0.495    295 
spe:Spro_1405 LysR family transcriptional regulator           302     0.407    295 
apt:APA01_13830 LysR family transcriptional regulator         302     0.404    280 
dma:DMR_13820 LysR family transcriptional regulator           301     0.401    294
```

**Neighborhood Representations**: Table of genes in the defined genetic neighborhoods of the entry protein and its closest homologs  
  
**Neighborhood Representations for "bxe:Bxe\_A2107"**  

| ID | Annotation | EC number |
| --- | --- | --- |
| bxe:Bxe\_A2117 | phosphonoacetaldehyde hydrolase; K05306 phosphonoacetaldehyde hydrolase [EC:3.11.1.1] | ec:3.11.1.1 |
| bxe:Bxe\_A2116 | 2-aminoethylphosphonate--pyruvate transaminase (EC:2.6.1.-) |  |
| bxe:Bxe\_A2115 | LysR family transcriptional regulator |  |
| bxe:Bxe\_A2114 | short-chain dehydrogenase/reductase SDR (EC:1.1.1.100); K00059 3-oxoacyl-[acyl-carrier protein] reductase [EC:1.1.1.100] | ec:1.1.1.100 |
| bxe:Bxe\_A2113 | hypothetical protein; K07090 |  |
| bxe:Bxe\_A2112 | LysR family transcriptional regulator |  |
| bxe:Bxe\_A2111 | ferredoxin-oxidoreductase FAD/NAD(P)-binding, ring hydroxylating dioxygenase reductase subunit (EC:1.14.13.82); K03863 vanillate monooxygenase [EC:1.14.13.82] | ec:1.14.13.82 |
| bxe:Bxe\_A2110 | catC; muconolactone delta-isomerase (EC:5.3.3.4); K03464 muconolactone D-isomerase [EC:5.3.3.4] | ec:5.3.3.4 |
| bxe:Bxe\_A2109 | catA; catechol 1,2-dioxygenase (CatA) (EC:1.13.11.1); K03381 catechol 1,2-dioxygenase [EC:1.13.11.1] | ec:1.13.11.1 |
| bxe:Bxe\_A2108 | catB; muconate cycloisomerase (EC:5.5.1.1); K01856 muconate cycloisomerase [EC:5.5.1.1] | ec:5.5.1.1 |
| bxe:Bxe\_A2107 | catR; LysR family transcriptional regulator |  |
| bxe:Bxe\_A2106 | LysR family transcriptional regulator |  |
| bxe:Bxe\_A2105 | (poly)aromatic ring hydroxylating dioxygenase, alpha subunit (Rieske (2Fe-2S) region) |  |
| bxe:Bxe\_A2104 | aromatic-ring-hydroxylating dioxygenase, beta subunit |  |
| bxe:Bxe\_A2103 | major facilitator superfamily metabolite/H(+) symporter |  |
| bxe:Bxe\_A2102 | hypothetical protein |  |
| bxe:Bxe\_A2101 | hypothetical protein |  |
| bxe:Bxe\_A2100 | Asp-tRNA Asn/Glu-tRNAGln amidotransferase A subunit; K01426 amidase [EC:3.5.1.4] | ec:3.5.1.4 |
| bxe:Bxe\_A2099 | short-chain dehydrogenase/reductase, TsaC-like; K00059 3-oxoacyl-[acyl-carrier protein] reductase [EC:1.1.1.100] | ec:1.1.1.100 |
| bxe:Bxe\_A2098 | cytoplasmic protein; K11902 type VI secretion system protein ImpA |  |
| bxe:Bxe\_A2097 | hypothetical protein |  |

  
**Neighborhood Representations for "bpy:Bphyt\_2154"**  

| ID | Annotation | EC number |
| --- | --- | --- |
| bpy:Bphyt\_2144 | short-chain dehydrogenase/reductase SDR; K00059 3-oxoacyl-[acyl-carrier protein] reductase [EC:1.1.1.100] | ec:1.1.1.100 |
| bpy:Bphyt\_2145 | DNA-binding transcriptional regulator CynR; K11921 LysR family transcriptional regulator, cyn operon transcriptional activator |  |
| bpy:Bphyt\_2146 | carbonate dehydratase (EC:4.2.1.1); K01673 carbonic anhydrase [EC:4.2.1.1] | ec:4.2.1.1 |
| bpy:Bphyt\_2147 | cyanate hydratase (EC:4.2.1.104); K01725 cyanate lyase [EC:4.2.1.104] | ec:4.2.1.104 |
| bpy:Bphyt\_2148 | hypothetical protein; K07090 |  |
| bpy:Bphyt\_2149 | LysR family transcriptional regulator |  |
| bpy:Bphyt\_2150 | ferredoxin; K03863 vanillate monooxygenase [EC:1.14.13.82] | ec:1.14.13.82 |
| bpy:Bphyt\_2151 | muconolactone delta-isomerase (EC:5.3.3.4); K03464 muconolactone D-isomerase [EC:5.3.3.4] | ec:5.3.3.4 |
| bpy:Bphyt\_2152 | catechol 1,2-dioxygenase; K03381 catechol 1,2-dioxygenase [EC:1.13.11.1] | ec:1.13.11.1 |
| bpy:Bphyt\_2153 | muconate and chloromuconate cycloisomerase (EC:5.5.1.1); K01856 muconate cycloisomerase [EC:5.5.1.1] | ec:5.5.1.1 |
| bpy:Bphyt\_2154 | LysR family transcriptional regulator |  |
| bpy:Bphyt\_2155 | LysR family transcriptional regulator |  |
| bpy:Bphyt\_2156 | Rieske (2Fe-2S) domain-containing protein |  |
| bpy:Bphyt\_2157 | aromatic-ring-hydroxylating dioxygenase subunit beta |  |
| bpy:Bphyt\_2158 | 4-oxalocrotonate tautomerase |  |
| bpy:Bphyt\_2159 | major facilitator superfamily protein |  |
| bpy:Bphyt\_2160 | PAS/PAC sensor signal transduction histidine kinase |  |
| bpy:Bphyt\_2161 | acyl-CoA dehydrogenase type 2 domain |  |
| bpy:Bphyt\_2162 | hypothetical protein |  |
| bpy:Bphyt\_2163 | hypothetical protein |  |
| bpy:Bphyt\_2164 | amidase; K01426 amidase [EC:3.5.1.4] | ec:3.5.1.4 |

  
**Neighborhood Representations for "bge:BC1002\_1617"**  

| ID | Annotation | EC number |
| --- | --- | --- |
| bge:BC1002\_1607 | Lytic transglycosylase catalytic |  |
| bge:BC1002\_1608 | diguanylate cyclase |  |
| bge:BC1002\_1609 | argininosuccinate synthase (EC:6.3.4.5); K01940 argininosuccinate synthase [EC:6.3.4.5] | ec:6.3.4.5 |
| bge:BC1002\_1610 | amidohydrolase |  |
| bge:BC1002\_1611 | transcriptional regulator, LysR family |  |
| bge:BC1002\_1612 | phosphonoacetaldehyde hydrolase (EC:3.11.1.1); K05306 phosphonoacetaldehyde hydrolase [EC:3.11.1.1] | ec:3.11.1.1 |
| bge:BC1002\_1613 | 2-aminoethylphosphonate--pyruvate transaminase; K03430 2-aminoethylphosphonate-pyruvate transaminase [EC:2.6.1.37] | ec:2.6.1.37 |
| bge:BC1002\_1614 | transcriptional regulator, LysR family |  |
| bge:BC1002\_1615 | short-chain dehydrogenase/reductase SDR; K00059 3-oxoacyl-[acyl-carrier protein] reductase [EC:1.1.1.100] | ec:1.1.1.100 |
| bge:BC1002\_1616 | pseudogene |  |
| bge:BC1002\_1617 | transcriptional regulator, LysR family |  |
| bge:BC1002\_1618 | copper resistance protein CopC; K07156 |  |
| bge:BC1002\_1619 | hypothetical protein |  |
| bge:BC1002\_1620 | hypothetical protein |  |
| bge:BC1002\_1621 | TRAP-type transport system, periplasmic protein |  |
| bge:BC1002\_1622 | Fertility inhibition FinO-like protein; K03607 ProP effector |  |
| bge:BC1002\_1623 | integral membrane sensor signal transduction histidine kinase; K02484 two-component system, OmpR family, sensor kinase [EC:2.7.13.3] | ec:2.7.13.3 |
| bge:BC1002\_1624 | two component transcriptional regulator, winged helix family |  |
| bge:BC1002\_1625 | hopanoid biosynthesis associated protein HpnK |  |
| bge:BC1002\_1626 | hopanoid biosynthesis associated glycosyl transferase protein HpnI; K00720 ceramide glucosyltransferase [EC:2.4.1.80] | ec:2.4.1.80 |
| bge:BC1002\_1627 | hypothetical protein |  |

  
**Neighborhood Representations for "bph:Bphy\_4030"**  

| ID | Annotation | EC number |
| --- | --- | --- |
| bph:Bphy\_4020 | EmrB/QacA family drug resistance transporter; K03446 MFS transporter, DHA2 family, multidrug resistance protein B |  |
| bph:Bphy\_4021 | secretion protein HlyD family protein; K03543 multidrug resistance protein A |  |
| bph:Bphy\_4022 | RND efflux system outer membrane lipoprotein |  |
| bph:Bphy\_4023 | MarR family transcriptional regulator |  |
| bph:Bphy\_4024 | MarR family transcriptional regulator |  |
| bph:Bphy\_4025 | anaerobic nitric oxide reductase transcriptional regulator; K12266 anaerobic nitric oxide reductase transcription regulator |  |
| bph:Bphy\_4026 | nitric oxide dioxygenase; K05916 nitric oxide dioxygenase [EC:1.14.12.17] | ec:1.14.12.17 |
| bph:Bphy\_4027 | muconolactone delta-isomerase (EC:5.3.3.4); K03464 muconolactone D-isomerase [EC:5.3.3.4] | ec:5.3.3.4 |
| bph:Bphy\_4028 | catechol 1,2-dioxygenase; K03381 catechol 1,2-dioxygenase [EC:1.13.11.1] | ec:1.13.11.1 |
| bph:Bphy\_4029 | muconate and chloromuconate cycloisomerase; K01856 muconate cycloisomerase [EC:5.5.1.1] | ec:5.5.1.1 |
| bph:Bphy\_4030 | LysR family transcriptional regulator |  |
| bph:Bphy\_4031 | hypothetical protein |  |
| bph:Bphy\_4032 | LysR family transcriptional regulator |  |
| bph:Bphy\_4033 | cytosine/purines uracil thiamine allantoin permease |  |
| bph:Bphy\_4034 | hypothetical protein |  |
| bph:Bphy\_4035 | hypothetical protein |  |
| bph:Bphy\_4036 | lysine/ornithine N-monooxygenase; K10531 L-ornithine N5-oxygenase [EC:1.13.12.-] |  |
| bph:Bphy\_4037 | hypothetical protein |  |
| bph:Bphy\_4038 | amino acid adenylation domain-containing protein |  |
| bph:Bphy\_4039 | amino acid adenylation domain-containing protein |  |
| bph:Bphy\_4040 | cyclic peptide transporter; K06160 putative ATP-binding cassette transporter |  |

  
**Neighborhood Representations for "bvi:Bcep1808\_4160"**  

| ID | Annotation | EC number |
| --- | --- | --- |
| bvi:Bcep1808\_4150 | LysR family transcriptional regulator |  |
| bvi:Bcep1808\_4151 | MarR family transcriptional regulator |  |
| bvi:Bcep1808\_4152 | AraC family transcriptional regulator |  |
| bvi:Bcep1808\_4153 | 2,4-dienoyl-CoA reductase (EC:1.3.1.34); K00219 2,4-dienoyl-CoA reductase (NADPH2) [EC:1.3.1.34] | ec:1.3.1.34 |
| bvi:Bcep1808\_4154 | hypothetical protein |  |
| bvi:Bcep1808\_4155 | LysR family transcriptional regulator |  |
| bvi:Bcep1808\_4156 | NAD(P)H dehydrogenase (quinone) (EC:1.6.5.2); K00355 NAD(P)H dehydrogenase (quinone) [EC:1.6.5.2] | ec:1.6.5.2 |
| bvi:Bcep1808\_4157 | muconolactone Delta-isomerase (EC:5.3.3.4); K03464 muconolactone D-isomerase [EC:5.3.3.4] | ec:5.3.3.4 |
| bvi:Bcep1808\_4158 | catechol 1,2-dioxygenase; K03381 catechol 1,2-dioxygenase [EC:1.13.11.1] | ec:1.13.11.1 |
| bvi:Bcep1808\_4159 | muconate and chloromuconate cycloisomerase; K01856 muconate cycloisomerase [EC:5.5.1.1] | ec:5.5.1.1 |
| bvi:Bcep1808\_4160 | LysR family transcriptional regulator |  |
| bvi:Bcep1808\_4161 | AraC family transcriptional regulator |  |
| bvi:Bcep1808\_4162 | Rieske (2Fe-2S) domain-containing protein; K16319 anthranilate 1,2-dioxygenase large subunit [EC:1.14.12.1] | ec:1.14.12.1 |
| bvi:Bcep1808\_4163 | aromatic-ring-hydroxylating dioxygenase subunit beta; K16320 anthranilate 1,2-dioxygenase small subunit [EC:1.14.12.1] | ec:1.14.12.1 |
| bvi:Bcep1808\_4164 | Rieske (2Fe-2S) domain-containing protein; K05710 dioxygenase ferredoxin subunit |  |
| bvi:Bcep1808\_4165 | FAD-dependent pyridine nucleotide-disulfide oxidoreductase; K00529 ferredoxin--NAD+ reductase [EC:1.18.1.3] | ec:1.18.1.3 |
| bvi:Bcep1808\_4166 | hypothetical protein |  |
| bvi:Bcep1808\_4167 | putative transcriptional regulator |  |
| bvi:Bcep1808\_4168 | hypothetical protein |  |
| bvi:Bcep1808\_4169 | acetolactate synthase; K01652 acetolactate synthase I/II/III large subunit [EC:2.2.1.6] | ec:2.2.1.6 |
| bvi:Bcep1808\_4170 | aldehyde dehydrogenase |  |

  
**Neighborhood Representations for "bur:Bcep18194\_B2327"**  

| ID | Annotation | EC number |
| --- | --- | --- |
| bur:Bcep18194\_B2317 | hypothetical protein |  |
| bur:Bcep18194\_B2318 | aminotransferase class V (EC:2.8.1.7) |  |
| bur:Bcep18194\_B2319 | peptidase E (EC:3.4.13.21); K05995 dipeptidase E [EC:3.4.13.21] | ec:3.4.13.21 |
| bur:Bcep18194\_B2320 | porin |  |
| bur:Bcep18194\_B2321 | hypothetical protein |  |
| bur:Bcep18194\_B2322 | FAD-dependent pyridine nucleotide-disulfide oxidoreductase (EC:1.18.1.3); K00529 ferredoxin--NAD+ reductase [EC:1.18.1.3] | ec:1.18.1.3 |
| bur:Bcep18194\_B2323 | Rieske (2Fe-2S) protein; K05710 dioxygenase ferredoxin subunit |  |
| bur:Bcep18194\_B2324 | aromatic-ring-hydroxylating dioxygenase subunit beta; K16320 anthranilate 1,2-dioxygenase small subunit [EC:1.14.12.1] | ec:1.14.12.1 |
| bur:Bcep18194\_B2325 | ring hydroxylating dioxygenase, alpha subunit/Rieske (2Fe-2S) protein (EC:1.14.12.18); K16319 anthranilate 1,2-dioxygenase large subunit [EC:1.14.12.1] | ec:1.14.12.1 |
| bur:Bcep18194\_B2326 | AraC family transcriptional regulator |  |
| bur:Bcep18194\_B2327 | LysR family transcriptional regulator |  |
| bur:Bcep18194\_B2328 | muconate cycloisomerase (EC:5.5.1.1); K01856 muconate cycloisomerase [EC:5.5.1.1] | ec:5.5.1.1 |
| bur:Bcep18194\_B2329 | catechol dioxygenase (EC:1.13.11.1); K03381 catechol 1,2-dioxygenase [EC:1.13.11.1] | ec:1.13.11.1 |
| bur:Bcep18194\_B2330 | muconolactone delta-isomerase (EC:5.3.3.4); K03464 muconolactone D-isomerase [EC:5.3.3.4] | ec:5.3.3.4 |
| bur:Bcep18194\_B2331 | hypothetical protein |  |
| bur:Bcep18194\_B2332 | NAD(P)H dehydrogenase (quinone) (EC:1.6.5.2); K00355 NAD(P)H dehydrogenase (quinone) [EC:1.6.5.2] | ec:1.6.5.2 |
| bur:Bcep18194\_B2333 | LysR family transcriptional regulator |  |
| bur:Bcep18194\_B2334 | hypothetical protein |  |
| bur:Bcep18194\_B2335 | 2,4-dienoyl-CoA reductase (EC:1.3.1.34); K00219 2,4-dienoyl-CoA reductase (NADPH2) [EC:1.3.1.34] | ec:1.3.1.34 |
| bur:Bcep18194\_B2336 | AraC family transcriptional regulator |  |
| bur:Bcep18194\_B2337 | LysR family transcriptional regulator |  |

  
**Neighborhood Representations for "bte:BTH\_II0486"**  

| ID | Annotation | EC number |
| --- | --- | --- |
| bte:BTH\_II0475 | hypothetical protein |  |
| bte:BTH\_II0477 | AraC family transcriptional regulator |  |
| bte:BTH\_II0478 | 2,4-dienoyl-CoA reductase; K00219 2,4-dienoyl-CoA reductase (NADPH2) [EC:1.3.1.34] | ec:1.3.1.34 |
| bte:BTH\_II0479 | ubiquinol oxidase subunit II; K02297 cytochrome o ubiquinol oxidase subunit II [EC:1.10.3.-] |  |
| bte:BTH\_II0480 | ubiquinol oxidase subunit I; K02298 cytochrome o ubiquinol oxidase subunit I [EC:1.10.3.-] |  |
| bte:BTH\_II0481 | cytochrome c oxidase subunit III; K02299 cytochrome o ubiquinol oxidase subunit III [EC:1.10.3.-] |  |
| bte:BTH\_II0482 | ubiquinol oxidase subunit IV; K02300 cytochrome o ubiquinol oxidase operon protein cyoD |  |
| bte:BTH\_II0483 | muconolactone delta-isomerase; K03464 muconolactone D-isomerase [EC:5.3.3.4] | ec:5.3.3.4 |
| bte:BTH\_II0484 | catechol 1,2-dioxygenase; K03381 catechol 1,2-dioxygenase [EC:1.13.11.1] | ec:1.13.11.1 |
| bte:BTH\_II0485 | muconate cycloisomerase; K01856 muconate cycloisomerase [EC:5.5.1.1] | ec:5.5.1.1 |
| bte:BTH\_II0486 | transcriptional regulator CatR |  |
| bte:BTH\_II0487 | AraC family transcriptional regulator |  |
| bte:BTH\_II0488 | ortho-halobenzoate 1,2-dioxygenase alpha-ISP protein OhbB; K16319 anthranilate 1,2-dioxygenase large subunit [EC:1.14.12.1] | ec:1.14.12.1 |
| bte:BTH\_II0489 | ortho-halobenzoate 1,2-dioxygenase beta-ISP protein OhbA; K16320 anthranilate 1,2-dioxygenase small subunit [EC:1.14.12.1] | ec:1.14.12.1 |
| bte:BTH\_II0490 | Rieske family iron-sulfur cluster-binding protein; K05710 dioxygenase ferredoxin subunit |  |
| bte:BTH\_II0491 | ferredoxin reductase; K00529 ferredoxin--NAD+ reductase [EC:1.18.1.3] | ec:1.18.1.3 |
| bte:BTH\_II0492 | HIT family protein; K02503 histidine triad (HIT) family protein |  |
| bte:BTH\_II0493 | phospholipase D |  |
| bte:BTH\_II0494 | acetyltransferase |  |
| bte:BTH\_II0495 | hypothetical protein |  |
| bte:BTH\_II0496 | Na+/H+ antiporter; K03316 monovalent cation:H+ antiporter, CPA1 family |  |

  
**Neighborhood Representations for "bac:BamMC406\_3663"**  

| ID | Annotation | EC number |
| --- | --- | --- |
| bac:BamMC406\_3653 | hypothetical protein |  |
| bac:BamMC406\_3654 | hypothetical protein |  |
| bac:BamMC406\_3655 | hypothetical protein; K11891 type VI secretion system protein ImpL |  |
| bac:BamMC406\_3656 | type VI secretion-associated protein; K11910 type VI secretion system protein VasJ |  |
| bac:BamMC406\_3657 | type VI secretion protein; K11896 type VI secretion system protein ImpG |  |
| bac:BamMC406\_3658 | type VI secretion protein; K11895 type VI secretion system protein ImpH |  |
| bac:BamMC406\_3659 | hypothetical protein; K11906 type VI secretion system protein VasD |  |
| bac:BamMC406\_3660 | muconolactone delta-isomerase (EC:5.3.3.4); K03464 muconolactone D-isomerase [EC:5.3.3.4] | ec:5.3.3.4 |
| bac:BamMC406\_3661 | catechol 1,2-dioxygenase; K03381 catechol 1,2-dioxygenase [EC:1.13.11.1] | ec:1.13.11.1 |
| bac:BamMC406\_3662 | muconate and chloromuconate cycloisomerase; K01856 muconate cycloisomerase [EC:5.5.1.1] | ec:5.5.1.1 |
| bac:BamMC406\_3663 | LysR family transcriptional regulator |  |
| bac:BamMC406\_3664 | methyl-accepting chemotaxis sensory transducer; K03406 methyl-accepting chemotaxis protein |  |
| bac:BamMC406\_3665 | hypothetical protein |  |
| bac:BamMC406\_3666 | AraC family transcriptional regulator |  |
| bac:BamMC406\_3667 | Rieske (2Fe-2S) domain-containing protein; K16319 anthranilate 1,2-dioxygenase large subunit [EC:1.14.12.1] | ec:1.14.12.1 |
| bac:BamMC406\_3668 | aromatic-ring-hydroxylating dioxygenase subunit beta; K16320 anthranilate 1,2-dioxygenase small subunit [EC:1.14.12.1] | ec:1.14.12.1 |
| bac:BamMC406\_3669 | Rieske (2Fe-2S) domain-containing protein; K05710 dioxygenase ferredoxin subunit |  |
| bac:BamMC406\_3670 | FAD-dependent pyridine nucleotide-disulfide oxidoreductase; K00529 ferredoxin--NAD+ reductase [EC:1.18.1.3] | ec:1.18.1.3 |
| bac:BamMC406\_3671 | hypothetical protein |  |
| bac:BamMC406\_3672 | putative transcriptional regulator |  |
| bac:BamMC406\_3673 | addiction module killer protein |  |

  
**Neighborhood Representations for "bam:Bamb\_5500"**  

| ID | Annotation | EC number |
| --- | --- | --- |
| bam:Bamb\_5490 | hypothetical protein; K09123 hypothetical protein |  |
| bam:Bamb\_5491 | hypothetical protein; K09128 hypothetical protein |  |
| bam:Bamb\_5492 | AraC family transcriptional regulator |  |
| bam:Bamb\_5493 | NADH:flavin oxidoreductase; K00219 2,4-dienoyl-CoA reductase (NADPH2) [EC:1.3.1.34] | ec:1.3.1.34 |
| bam:Bamb\_5494 | hypothetical protein |  |
| bam:Bamb\_5495 | LysR family transcriptional regulator |  |
| bam:Bamb\_5496 | NAD(P)H dehydrogenase (quinone) (EC:1.6.5.2); K00355 NAD(P)H dehydrogenase (quinone) [EC:1.6.5.2] | ec:1.6.5.2 |
| bam:Bamb\_5497 | muconolactone delta-isomerase (EC:5.3.3.4); K03464 muconolactone D-isomerase [EC:5.3.3.4] | ec:5.3.3.4 |
| bam:Bamb\_5498 | catechol 1,2-dioxygenase; K03381 catechol 1,2-dioxygenase [EC:1.13.11.1] | ec:1.13.11.1 |
| bam:Bamb\_5499 | muconate and chloromuconate cycloisomerase; K01856 muconate cycloisomerase [EC:5.5.1.1] | ec:5.5.1.1 |
| bam:Bamb\_5500 | LysR family transcriptional regulator |  |
| bam:Bamb\_5501 | AraC family transcriptional regulator |  |
| bam:Bamb\_5502 | Rieske (2Fe-2S) domain-containing protein; K16319 anthranilate 1,2-dioxygenase large subunit [EC:1.14.12.1] | ec:1.14.12.1 |
| bam:Bamb\_5503 | aromatic-ring-hydroxylating dioxygenase subunit beta; K16320 anthranilate 1,2-dioxygenase small subunit [EC:1.14.12.1] | ec:1.14.12.1 |
| bam:Bamb\_5504 | Rieske (2Fe-2S) domain-containing protein; K05710 dioxygenase ferredoxin subunit |  |
| bam:Bamb\_5505 | FAD-dependent pyridine nucleotide-disulfide oxidoreductase; K00529 ferredoxin--NAD+ reductase [EC:1.18.1.3] | ec:1.18.1.3 |
| bam:Bamb\_5506 | hypothetical protein |  |
| bam:Bamb\_5507 | transcriptional regulator |  |
| bam:Bamb\_5508 | hypothetical protein |  |
| bam:Bamb\_5509 | acetolactate synthase; K01652 acetolactate synthase I/II/III large subunit [EC:2.2.1.6] | ec:2.2.1.6 |
| bam:Bamb\_5510 | aldehyde dehydrogenase |  |

  
**Neighborhood Representations for "bma:BMAA0201"**  

| ID | Annotation | EC number |
| --- | --- | --- |
| bma:BMAA0191 | AraC family transcriptional regulator |  |
| bma:BMAA0192 | fadH; 2,4-dienoyl-CoA reductase (EC:1.3.1.34); K00219 2,4-dienoyl-CoA reductase (NADPH2) [EC:1.3.1.34] | ec:1.3.1.34 |
| bma:BMAA0193 | hypothetical protein |  |
| bma:BMAA0194 | cyoA-2; ubiquinol oxidase subunit II (EC:1.10.3.-); K02297 cytochrome o ubiquinol oxidase subunit II [EC:1.10.3.-] |  |
| bma:BMAA0195 | cyoB; ubiquinol oxidase, subunit I (EC:1.10.3.-); K02298 cytochrome o ubiquinol oxidase subunit I [EC:1.10.3.-] |  |
| bma:BMAA0196 | cyoC; ubiquinol oxidase subunit III (EC:1.10.3.-); K02299 cytochrome o ubiquinol oxidase subunit III [EC:1.10.3.-] |  |
| bma:BMAA0197 | cyoD-2; ubiquinol oxidase subunit IV (EC:1.10.3.-); K02300 cytochrome o ubiquinol oxidase operon protein cyoD |  |
| bma:BMAA0198 | catC; muconolactone delta-isomerase; K03464 muconolactone D-isomerase [EC:5.3.3.4] | ec:5.3.3.4 |
| bma:BMAA0199 | catA; catechol 1,2-dioxygenase (EC:1.13.11.1); K03381 catechol 1,2-dioxygenase [EC:1.13.11.1] | ec:1.13.11.1 |
| bma:BMAA0200 | catB; muconate cycloisomerase (EC:5.5.1.1); K01856 muconate cycloisomerase [EC:5.5.1.1] | ec:5.5.1.1 |
| bma:BMAA0201 | transcriptional regulator CatR |  |
| bma:BMAA0202 | AraC family transcriptional regulator |  |
| bma:BMAA0203 | ortho-halobenzoate 1,2-dioxygenase alpha-ISP protein OhbB; K16319 anthranilate 1,2-dioxygenase large subunit [EC:1.14.12.1] | ec:1.14.12.1 |
| bma:BMAA0204 | ortho-halobenzoate 1,2-dioxygenase beta-ISP protein OhbA; K16320 anthranilate 1,2-dioxygenase small subunit [EC:1.14.12.1] | ec:1.14.12.1 |
| bma:BMAA0205 | Rieske family iron-sulfur cluster-binding protein; K05710 dioxygenase ferredoxin subunit |  |
| bma:BMAA0206 | ferredoxin reductase; K00529 ferredoxin--NAD+ reductase [EC:1.18.1.3] | ec:1.18.1.3 |
| bma:BMAA0207 | HIT family protein; K02503 histidine triad (HIT) family protein |  |
| bma:BMAA0208 | phospholipase D |  |
| bma:BMAA0209 | hypothetical protein |  |
| bma:BMAA0210 | pseudogene |  |
| bma:BMAA0211 | Na+/H+ antiporter; K03316 monovalent cation:H+ antiporter, CPA1 family |  |

  
**Neighborhood Representations for "bmv:BMASAVP1\_1376"**  

| ID | Annotation | EC number |
| --- | --- | --- |
| bmv:BMASAVP1\_1365 | AraC family transcriptional regulator |  |
| bmv:BMASAVP1\_1367 | FAD/FMN-binding/pyridine nucleotide-disulfide domain-containing oxidoreductase; K00219 2,4-dienoyl-CoA reductase (NADPH2) [EC:1.3.1.34] | ec:1.3.1.34 |
| bmv:BMASAVP1\_1368 | hypothetical protein |  |
| bmv:BMASAVP1\_1369 | cyoA-2; ubiquinol oxidase subunit II; K02297 cytochrome o ubiquinol oxidase subunit II [EC:1.10.3.-] |  |
| bmv:BMASAVP1\_1370 | cyoB; ubiquinol oxidase, subunit I; K02298 cytochrome o ubiquinol oxidase subunit I [EC:1.10.3.-] |  |
| bmv:BMASAVP1\_1371 | cyoC; ubiquinol oxidase subunit III; K02299 cytochrome o ubiquinol oxidase subunit III [EC:1.10.3.-] |  |
| bmv:BMASAVP1\_1372 | cyoD-2; ubiquinol oxidase subunit IV; K02300 cytochrome o ubiquinol oxidase operon protein cyoD |  |
| bmv:BMASAVP1\_1373 | catC; muconolactone delta-isomerase; K03464 muconolactone D-isomerase [EC:5.3.3.4] | ec:5.3.3.4 |
| bmv:BMASAVP1\_1374 | catA; catechol 1,2-dioxygenase; K03381 catechol 1,2-dioxygenase [EC:1.13.11.1] | ec:1.13.11.1 |
| bmv:BMASAVP1\_1375 | catB; muconate cycloisomerase; K01856 muconate cycloisomerase [EC:5.5.1.1] | ec:5.5.1.1 |
| bmv:BMASAVP1\_1376 | catR; cat operon transcriptional activator CatR |  |
| bmv:BMASAVP1\_1377 | AraC family transcriptional regulator |  |
| bmv:BMASAVP1\_1378 | aromatic-ring-hydroxylating dioxygenase, alpha subunit; K16319 anthranilate 1,2-dioxygenase large subunit [EC:1.14.12.1] | ec:1.14.12.1 |
| bmv:BMASAVP1\_1379 | aromatic-ring-hydroxylating dioxygenase subunit beta; K16320 anthranilate 1,2-dioxygenase small subunit [EC:1.14.12.1] | ec:1.14.12.1 |
| bmv:BMASAVP1\_1380 | aromatic-ring-hydroxylating dioxygenase, ferredoxin subunit; K05710 dioxygenase ferredoxin subunit |  |
| bmv:BMASAVP1\_1381 | aromatic-ring-hydroxylating dioxygenase, ferredoxin reductase subunit; K00529 ferredoxin--NAD+ reductase [EC:1.18.1.3] | ec:1.18.1.3 |
| bmv:BMASAVP1\_1382 | HIT family protein; K02503 histidine triad (HIT) family protein |  |
| bmv:BMASAVP1\_1383 | phospholipase D |  |
| bmv:BMASAVP1\_1384 | hypothetical protein |  |
| bmv:BMASAVP1\_1385 | Rex protein |  |
| bmv:BMASAVP1\_1386 | hypothetical protein |  |

  
**Neighborhood Representations for "bch:Bcen2424\_3771"**  

| ID | Annotation | EC number |
| --- | --- | --- |
| bch:Bcen2424\_3761 | hypothetical protein |  |
| bch:Bcen2424\_3762 | hypothetical protein |  |
| bch:Bcen2424\_3763 | AraC family transcriptional regulator |  |
| bch:Bcen2424\_3764 | NADH:flavin oxidoreductase; K00219 2,4-dienoyl-CoA reductase (NADPH2) [EC:1.3.1.34] | ec:1.3.1.34 |
| bch:Bcen2424\_3765 | hypothetical protein |  |
| bch:Bcen2424\_3766 | LysR family transcriptional regulator |  |
| bch:Bcen2424\_3767 | hypothetical protein; K00355 NAD(P)H dehydrogenase (quinone) [EC:1.6.5.2] | ec:1.6.5.2 |
| bch:Bcen2424\_3768 | muconolactone delta-isomerase (EC:5.3.3.4); K03464 muconolactone D-isomerase [EC:5.3.3.4] | ec:5.3.3.4 |
| bch:Bcen2424\_3769 | catechol 1,2-dioxygenase; K03381 catechol 1,2-dioxygenase [EC:1.13.11.1] | ec:1.13.11.1 |
| bch:Bcen2424\_3770 | muconate and chloromuconate cycloisomerase (EC:5.5.1.1); K01856 muconate cycloisomerase [EC:5.5.1.1] | ec:5.5.1.1 |
| bch:Bcen2424\_3771 | LysR family transcriptional regulator |  |
| bch:Bcen2424\_3772 | methyl-accepting chemotaxis sensory transducer; K03406 methyl-accepting chemotaxis protein |  |
| bch:Bcen2424\_3773 | hypothetical protein |  |
| bch:Bcen2424\_3774 | AraC family transcriptional regulator |  |
| bch:Bcen2424\_3775 | Rieske (2Fe-2S) domain-containing protein; K16319 anthranilate 1,2-dioxygenase large subunit [EC:1.14.12.1] | ec:1.14.12.1 |
| bch:Bcen2424\_3776 | aromatic-ring-hydroxylating dioxygenase subunit beta; K16320 anthranilate 1,2-dioxygenase small subunit [EC:1.14.12.1] | ec:1.14.12.1 |
| bch:Bcen2424\_3777 | Rieske (2Fe-2S) domain-containing protein; K05710 dioxygenase ferredoxin subunit |  |
| bch:Bcen2424\_3778 | FAD-dependent pyridine nucleotide-disulfide oxidoreductase; K00529 ferredoxin--NAD+ reductase [EC:1.18.1.3] | ec:1.18.1.3 |
| bch:Bcen2424\_3779 | hypothetical protein |  |
| bch:Bcen2424\_3780 | transcriptional regulator |  |
| bch:Bcen2424\_3781 | hypothetical protein |  |

  
**Neighborhood Representations for "bcm:Bcenmc03\_3752"**  

| ID | Annotation | EC number |
| --- | --- | --- |
| bcm:Bcenmc03\_3742 | addiction module killer protein |  |
| bcm:Bcenmc03\_3743 | putative transcriptional regulator |  |
| bcm:Bcenmc03\_3744 | hypothetical protein |  |
| bcm:Bcenmc03\_3745 | FAD-dependent pyridine nucleotide-disulfide oxidoreductase; K00529 ferredoxin--NAD+ reductase [EC:1.18.1.3] | ec:1.18.1.3 |
| bcm:Bcenmc03\_3746 | Rieske (2Fe-2S) domain-containing protein; K05710 dioxygenase ferredoxin subunit |  |
| bcm:Bcenmc03\_3747 | aromatic-ring-hydroxylating dioxygenase subunit beta; K16320 anthranilate 1,2-dioxygenase small subunit [EC:1.14.12.1] | ec:1.14.12.1 |
| bcm:Bcenmc03\_3748 | Rieske (2Fe-2S) domain-containing protein; K16319 anthranilate 1,2-dioxygenase large subunit [EC:1.14.12.1] | ec:1.14.12.1 |
| bcm:Bcenmc03\_3749 | AraC family transcriptional regulator |  |
| bcm:Bcenmc03\_3750 | hypothetical protein |  |
| bcm:Bcenmc03\_3751 | methyl-accepting chemotaxis sensory transducer; K03406 methyl-accepting chemotaxis protein |  |
| bcm:Bcenmc03\_3752 | LysR family transcriptional regulator |  |
| bcm:Bcenmc03\_3753 | muconate and chloromuconate cycloisomerase (EC:5.5.1.1); K01856 muconate cycloisomerase [EC:5.5.1.1] | ec:5.5.1.1 |
| bcm:Bcenmc03\_3754 | catechol 1,2-dioxygenase; K03381 catechol 1,2-dioxygenase [EC:1.13.11.1] | ec:1.13.11.1 |
| bcm:Bcenmc03\_3755 | muconolactone delta-isomerase (EC:5.3.3.4); K03464 muconolactone D-isomerase [EC:5.3.3.4] | ec:5.3.3.4 |
| bcm:Bcenmc03\_3756 | NAD(P)H dehydrogenase (quinone) (EC:1.6.5.2); K00355 NAD(P)H dehydrogenase (quinone) [EC:1.6.5.2] | ec:1.6.5.2 |
| bcm:Bcenmc03\_3757 | LysR family transcriptional regulator |  |
| bcm:Bcenmc03\_3758 | hypothetical protein |  |
| bcm:Bcenmc03\_3759 | NADH:flavin oxidoreductase/NADH oxidase; K00219 2,4-dienoyl-CoA reductase (NADPH2) [EC:1.3.1.34] | ec:1.3.1.34 |
| bcm:Bcenmc03\_3760 | AraC family transcriptional regulator |  |
| bcm:Bcenmc03\_3761 | hypothetical protein |  |
| bcm:Bcenmc03\_3762 | hypothetical protein |  |

  
**Neighborhood Representations for "bcn:Bcen\_4592"**  

| ID | Annotation | EC number |
| --- | --- | --- |
| bcn:Bcen\_4582 | hypothetical protein |  |
| bcn:Bcen\_4583 | transcriptional regulator |  |
| bcn:Bcen\_4584 | hypothetical protein |  |
| bcn:Bcen\_4585 | FAD-dependent pyridine nucleotide-disulfide oxidoreductase; K00529 ferredoxin--NAD+ reductase [EC:1.18.1.3] | ec:1.18.1.3 |
| bcn:Bcen\_4586 | Rieske (2Fe-2S) protein; K05710 dioxygenase ferredoxin subunit |  |
| bcn:Bcen\_4587 | aromatic-ring-hydroxylating dioxygenase subunit beta; K16320 anthranilate 1,2-dioxygenase small subunit [EC:1.14.12.1] | ec:1.14.12.1 |
| bcn:Bcen\_4588 | Rieske (2Fe-2S) protein; K16319 anthranilate 1,2-dioxygenase large subunit [EC:1.14.12.1] | ec:1.14.12.1 |
| bcn:Bcen\_4589 | AraC family transcriptional regulator |  |
| bcn:Bcen\_4590 | hypothetical protein |  |
| bcn:Bcen\_4591 | methyl-accepting chemotaxis sensory transducer; K03406 methyl-accepting chemotaxis protein |  |
| bcn:Bcen\_4592 | LysR family transcriptional regulator |  |
| bcn:Bcen\_4593 | muconate and chloromuconate cycloisomerase (EC:5.5.1.1); K01856 muconate cycloisomerase [EC:5.5.1.1] | ec:5.5.1.1 |
| bcn:Bcen\_4594 | catechol 1,2-dioxygenase; K03381 catechol 1,2-dioxygenase [EC:1.13.11.1] | ec:1.13.11.1 |
| bcn:Bcen\_4595 | muconolactone delta-isomerase (EC:5.3.3.4); K03464 muconolactone D-isomerase [EC:5.3.3.4] | ec:5.3.3.4 |
| bcn:Bcen\_4596 | NAD(P)H dehydrogenase (quinone) (EC:1.6.5.2); K00355 NAD(P)H dehydrogenase (quinone) [EC:1.6.5.2] | ec:1.6.5.2 |
| bcn:Bcen\_4597 | LysR family transcriptional regulator |  |
| bcn:Bcen\_4598 | hypothetical protein |  |
| bcn:Bcen\_4599 | NADH:flavin oxidoreductase; K00219 2,4-dienoyl-CoA reductase (NADPH2) [EC:1.3.1.34] | ec:1.3.1.34 |
| bcn:Bcen\_4600 | AraC family transcriptional regulator |  |
| bcn:Bcen\_4601 | hypothetical protein |  |
| bcn:Bcen\_4602 | hypothetical protein |  |

  
**Neighborhood Representations for "bpd:BURPS668\_A2709"**  

| ID | Annotation | EC number |
| --- | --- | --- |
| bpd:BURPS668\_A2699 | hypothetical protein |  |
| bpd:BURPS668\_A2700 | acetyltransferase |  |
| bpd:BURPS668\_A2701 | hypothetical protein |  |
| bpd:BURPS668\_A2702 | phospholipase D family protein |  |
| bpd:BURPS668\_A2703 | HIT family protein; K02503 histidine triad (HIT) family protein |  |
| bpd:BURPS668\_A2704 | aromatic-ring-hydroxylating dioxygenase, ferredoxin reductase subunit; K00529 ferredoxin--NAD+ reductase [EC:1.18.1.3] | ec:1.18.1.3 |
| bpd:BURPS668\_A2705 | aromatic-ring-hydroxylating dioxygenase, ferredoxin subunit; K05710 dioxygenase ferredoxin subunit |  |
| bpd:BURPS668\_A2706 | aromatic-ring-hydroxylating dioxygenase subunit beta; K16320 anthranilate 1,2-dioxygenase small subunit [EC:1.14.12.1] | ec:1.14.12.1 |
| bpd:BURPS668\_A2707 | aromatic-ring-hydroxylating dioxygenase subunit alpha; K16319 anthranilate 1,2-dioxygenase large subunit [EC:1.14.12.1] | ec:1.14.12.1 |
| bpd:BURPS668\_A2708 | AraC family transcriptional regulator |  |
| bpd:BURPS668\_A2709 | catR; cat operon transcriptional activator CatR |  |
| bpd:BURPS668\_A2710 | catB; muconate cycloisomerase (EC:5.5.1.1); K01856 muconate cycloisomerase [EC:5.5.1.1] | ec:5.5.1.1 |
| bpd:BURPS668\_A2711 | catA; catechol 1,2-dioxygenase (EC:1.13.11.1); K03381 catechol 1,2-dioxygenase [EC:1.13.11.1] | ec:1.13.11.1 |
| bpd:BURPS668\_A2712 | catC; muconolactone delta-isomerase 2 (EC:5.3.3.4); K03464 muconolactone D-isomerase [EC:5.3.3.4] | ec:5.3.3.4 |
| bpd:BURPS668\_A2713 | cyoD; ubiquinol oxidase subunit IV (EC:1.10.3.-); K02300 cytochrome o ubiquinol oxidase operon protein cyoD |  |
| bpd:BURPS668\_A2714 | cyoC; ubiquinol oxidase subunit III (EC:1.10.3.-); K02299 cytochrome o ubiquinol oxidase subunit III [EC:1.10.3.-] |  |
| bpd:BURPS668\_A2715 | cyoB; ubiquinol oxidase subunit I (EC:1.10.3.-); K02298 cytochrome o ubiquinol oxidase subunit I [EC:1.10.3.-] |  |
| bpd:BURPS668\_A2716 | cyoA; ubiquinol oxidase subunit II (EC:1.10.3.-); K02297 cytochrome o ubiquinol oxidase subunit II [EC:1.10.3.-] |  |
| bpd:BURPS668\_A2717 | hypothetical protein |  |
| bpd:BURPS668\_A2718 | hypothetical protein |  |
| bpd:BURPS668\_A2719 | FAD/FMN-binding/pyridine nucleotide-disulfide domain-containing oxidoreductase; K00219 2,4-dienoyl-CoA reductase (NADPH2) [EC:1.3.1.34] | ec:1.3.1.34 |

  
**Neighborhood Representations for "bpl:BURPS1106A\_A2565"**  

| ID | Annotation | EC number |
| --- | --- | --- |
| bpl:BURPS1106A\_A2555 | acetyltransferase |  |
| bpl:BURPS1106A\_A2556 | hypothetical protein |  |
| bpl:BURPS1106A\_A2557 | hypothetical protein |  |
| bpl:BURPS1106A\_A2558 | phospholipase D family protein |  |
| bpl:BURPS1106A\_A2559 | HIT family protein; K02503 histidine triad (HIT) family protein |  |
| bpl:BURPS1106A\_A2560 | aromatic-ring-hydroxylating dioxygenase, ferredoxin reductase subunit; K00529 ferredoxin--NAD+ reductase [EC:1.18.1.3] | ec:1.18.1.3 |
| bpl:BURPS1106A\_A2561 | aromatic-ring-hydroxylating dioxygenase, ferredoxin subunit; K05710 dioxygenase ferredoxin subunit |  |
| bpl:BURPS1106A\_A2562 | aromatic-ring-hydroxylating dioxygenase subunit beta; K16320 anthranilate 1,2-dioxygenase small subunit [EC:1.14.12.1] | ec:1.14.12.1 |
| bpl:BURPS1106A\_A2563 | aromatic-ring-hydroxylating dioxygenase subunit alpha; K16319 anthranilate 1,2-dioxygenase large subunit [EC:1.14.12.1] | ec:1.14.12.1 |
| bpl:BURPS1106A\_A2564 | AraC family transcriptional regulator |  |
| bpl:BURPS1106A\_A2565 | catR; cat operon transcriptional activator CatR |  |
| bpl:BURPS1106A\_A2566 | catB; muconate cycloisomerase (EC:5.5.1.1); K01856 muconate cycloisomerase [EC:5.5.1.1] | ec:5.5.1.1 |
| bpl:BURPS1106A\_A2567 | catA; catechol 1,2-dioxygenase (EC:1.13.11.1); K03381 catechol 1,2-dioxygenase [EC:1.13.11.1] | ec:1.13.11.1 |
| bpl:BURPS1106A\_A2568 | catC; muconolactone delta-isomerase 2 (EC:5.3.3.4); K03464 muconolactone D-isomerase [EC:5.3.3.4] | ec:5.3.3.4 |
| bpl:BURPS1106A\_A2569 | cyoD; ubiquinol oxidase subunit IV (EC:1.10.3.-); K02300 cytochrome o ubiquinol oxidase operon protein cyoD |  |
| bpl:BURPS1106A\_A2570 | cyoC; ubiquinol oxidase subunit III (EC:1.10.3.-); K02299 cytochrome o ubiquinol oxidase subunit III [EC:1.10.3.-] |  |
| bpl:BURPS1106A\_A2571 | cyoB; ubiquinol oxidase subunit I (EC:1.10.3.-); K02298 cytochrome o ubiquinol oxidase subunit I [EC:1.10.3.-] |  |
| bpl:BURPS1106A\_A2572 | cyoA; ubiquinol oxidase subunit II (EC:1.10.3.-); K02297 cytochrome o ubiquinol oxidase subunit II [EC:1.10.3.-] |  |
| bpl:BURPS1106A\_A2573 | hypothetical protein |  |
| bpl:BURPS1106A\_A2574 | hypothetical protein |  |
| bpl:BURPS1106A\_A2575 | FAD/FMN-binding/pyridine nucleotide-disulphide domain-containing oxidoreductase; K00219 2,4-dienoyl-CoA reductase (NADPH2) [EC:1.3.1.34] | ec:1.3.1.34 |

  
**Neighborhood Representations for "bpm:BURPS1710b\_A0985"**  

| ID | Annotation | EC number |
| --- | --- | --- |
| bpm:BURPS1710b\_A0975 | Na+/H+ antiporter; K03316 monovalent cation:H+ antiporter, CPA1 family |  |
| bpm:BURPS1710b\_A0976 | hypothetical protein |  |
| bpm:BURPS1710b\_A0977 | putative acetyltransferase |  |
| bpm:BURPS1710b\_A0978 | phospholipase D |  |
| bpm:BURPS1710b\_A0979 | hit; HIT family protein; K02503 histidine triad (HIT) family protein |  |
| bpm:BURPS1710b\_A0980 | ferredoxin reductase; K00529 ferredoxin--NAD+ reductase [EC:1.18.1.3] | ec:1.18.1.3 |
| bpm:BURPS1710b\_A0981 | Rieske family iron-sulfur cluster-binding protein; K05710 dioxygenase ferredoxin subunit |  |
| bpm:BURPS1710b\_A0982 | ortho-halobenzoate 1,2-dioxygenase beta-ISP protein OhbA; K16320 anthranilate 1,2-dioxygenase small subunit [EC:1.14.12.1] | ec:1.14.12.1 |
| bpm:BURPS1710b\_A0983 | ortho-halobenzoate 1,2-dioxygenase alpha-ISP protein OhbB; K16319 anthranilate 1,2-dioxygenase large subunit [EC:1.14.12.1] | ec:1.14.12.1 |
| bpm:BURPS1710b\_A0984 | andR; protein AndR |  |
| bpm:BURPS1710b\_A0985 | catR; transcriptional regulator CatR |  |
| bpm:BURPS1710b\_A0986 | catB; muconate cycloisomerase; K01856 muconate cycloisomerase [EC:5.5.1.1] | ec:5.5.1.1 |
| bpm:BURPS1710b\_A0987 | catA; catechol 1,2-dioxygenase; K03381 catechol 1,2-dioxygenase [EC:1.13.11.1] | ec:1.13.11.1 |
| bpm:BURPS1710b\_A0989 | hypothetical protein |  |
| bpm:BURPS1710b\_A0988 | catC; muconolactone delta-isomerase; K03464 muconolactone D-isomerase [EC:5.3.3.4] | ec:5.3.3.4 |
| bpm:BURPS1710b\_A0990 | cyoD-2; ubiquinol oxidase subunit IV; K02300 cytochrome o ubiquinol oxidase operon protein cyoD |  |
| bpm:BURPS1710b\_A0991 | cyoC; ubiquinol oxidase subunit III; K02299 cytochrome o ubiquinol oxidase subunit III [EC:1.10.3.-] |  |
| bpm:BURPS1710b\_A0992 | cyoB; ubiquinol oxidase subunit I; K02298 cytochrome o ubiquinol oxidase subunit I [EC:1.10.3.-] |  |
| bpm:BURPS1710b\_A0993 | cyoA-2; ubiquinol oxidase subunit II; K02297 cytochrome o ubiquinol oxidase subunit II [EC:1.10.3.-] |  |
| bpm:BURPS1710b\_A0994 | hypothetical protein |  |
| bpm:BURPS1710b\_A0995 | fadH; 2,4-dienoyl-CoA reductase; K00219 2,4-dienoyl-CoA reductase (NADPH2) [EC:1.3.1.34] | ec:1.3.1.34 |

  
**Neighborhood Representations for "bps:BPSS1890"**  

| ID | Annotation | EC number |
| --- | --- | --- |
| bps:BPSS1880 | Na+/H+ antiporter membrane protein; K03316 monovalent cation:H+ antiporter, CPA1 family |  |
| bps:BPSS1881 | hypothetical protein |  |
| bps:BPSS1882 | acetyltransferase |  |
| bps:BPSS1883 | hypothetical protein |  |
| bps:BPSS1884 | hypothetical protein; K02503 histidine triad (HIT) family protein |  |
| bps:BPSS1885 | aromatic hydrocarbons catabolism-related reductase; K00529 ferredoxin--NAD+ reductase [EC:1.18.1.3] | ec:1.18.1.3 |
| bps:BPSS1886 | aromatic hydrocarbons catabolism-related dioxygenase; K05710 dioxygenase ferredoxin subunit |  |
| bps:BPSS1887 | aromatic oxygenase; K16320 anthranilate 1,2-dioxygenase small subunit [EC:1.14.12.1] | ec:1.14.12.1 |
| bps:BPSS1888 | aromatic oxygenase; K16319 anthranilate 1,2-dioxygenase large subunit [EC:1.14.12.1] | ec:1.14.12.1 |
| bps:BPSS1889 | AraC family transcriptional regulator |  |
| bps:BPSS1890 | LysR family transcriptional regulator |  |
| bps:BPSS1891 | catB; muconate cycloisomerase I (EC:5.5.1.1); K01856 muconate cycloisomerase [EC:5.5.1.1] | ec:5.5.1.1 |
| bps:BPSS1892 | catA; catechol 1,2-dioxygenase (EC:1.13.11.1); K03381 catechol 1,2-dioxygenase [EC:1.13.11.1] | ec:1.13.11.1 |
| bps:BPSS1893 | catC; muconolactone delta-isomerase (EC:5.3.3.4); K03464 muconolactone D-isomerase [EC:5.3.3.4] | ec:5.3.3.4 |
| bps:BPSS1894 | cyoD; cytochrome O ubiquinol oxidase; K02300 cytochrome o ubiquinol oxidase operon protein cyoD |  |
| bps:BPSS1895 | cyoC; cytochrome O ubiquinol oxidase subunit III (EC:1.10.3.-); K02299 cytochrome o ubiquinol oxidase subunit III [EC:1.10.3.-] |  |
| bps:BPSS1896 | cyoB; ubiquinol oxidase polypeptide I (EC:1.10.3.-); K02298 cytochrome o ubiquinol oxidase subunit I [EC:1.10.3.-] |  |
| bps:BPSS1897 | cyoA; ubiquinol oxidase polypeptide II precursor (EC:1.10.3.-); K02297 cytochrome o ubiquinol oxidase subunit II [EC:1.10.3.-] |  |
| bps:BPSS1898 | fadH; 2,4-dienoyl-CoA reductase (EC:1.3.1.34); K00219 2,4-dienoyl-CoA reductase (NADPH2) [EC:1.3.1.34] | ec:1.3.1.34 |
| bps:BPSS1899 | AraC family transcriptional regulator |  |
| bps:BPSS1900 | LysR family transcriptional regulator |  |

  
**Neighborhood Representations for "bml:BMA10229\_1573"**  

| ID | Annotation | EC number |
| --- | --- | --- |
| bml:BMA10229\_1563 | hypothetical protein |  |
| bml:BMA10229\_1564 | hypothetical protein |  |
| bml:BMA10229\_1565 | hypothetical protein |  |
| bml:BMA10229\_1566 | cyoA-2; ubiquinol oxidase subunit II; K02297 cytochrome o ubiquinol oxidase subunit II [EC:1.10.3.-] |  |
| bml:BMA10229\_1567 | cyoB; ubiquinol oxidase, subunit I; K02298 cytochrome o ubiquinol oxidase subunit I [EC:1.10.3.-] |  |
| bml:BMA10229\_1568 | cyoC; ubiquinol oxidase subunit III; K02299 cytochrome o ubiquinol oxidase subunit III [EC:1.10.3.-] |  |
| bml:BMA10229\_1569 | cyoD-2; ubiquinol oxidase subunit IV; K02300 cytochrome o ubiquinol oxidase operon protein cyoD |  |
| bml:BMA10229\_1570 | catC; muconolactone delta-isomerase; K03464 muconolactone D-isomerase [EC:5.3.3.4] | ec:5.3.3.4 |
| bml:BMA10229\_1571 | catA; catechol 1,2-dioxygenase; K03381 catechol 1,2-dioxygenase [EC:1.13.11.1] | ec:1.13.11.1 |
| bml:BMA10229\_1572 | catB; muconate cycloisomerase; K01856 muconate cycloisomerase [EC:5.5.1.1] | ec:5.5.1.1 |
| bml:BMA10229\_1573 | catR; cat operon transcriptional activator CatR |  |
| bml:BMA10229\_1574 | AraC family transcriptional regulator |  |
| bml:BMA10229\_1575 | aromatic-ring-hydroxylating dioxygenase, alpha subunit; K16319 anthranilate 1,2-dioxygenase large subunit [EC:1.14.12.1] | ec:1.14.12.1 |
| bml:BMA10229\_1576 | aromatic-ring-hydroxylating dioxygenase subunit beta; K16320 anthranilate 1,2-dioxygenase small subunit [EC:1.14.12.1] | ec:1.14.12.1 |
| bml:BMA10229\_1577 | aromatic-ring-hydroxylating dioxygenase, ferredoxin subunit; K05710 dioxygenase ferredoxin subunit |  |
| bml:BMA10229\_1578 | aromatic-ring-hydroxylating dioxygenase, ferredoxin reductase subunit; K00529 ferredoxin--NAD+ reductase [EC:1.18.1.3] | ec:1.18.1.3 |
| bml:BMA10229\_1579 | HIT family protein; K02503 histidine triad (HIT) family protein |  |
| bml:BMA10229\_1580 | phospholipase D |  |
| bml:BMA10229\_1581 | hypothetical protein |  |
| bml:BMA10229\_1582 | hypothetical protein |  |
| bml:BMA10229\_1583 | hypothetical protein |  |

  
**Neighborhood Representations for "bmn:BMA10247\_A0232"**  

| ID | Annotation | EC number |
| --- | --- | --- |
| bmn:BMA10247\_A0222 | FAD/FMN-binding/pyridine nucleotide-disulfide domain-containing oxidoreductase; K00219 2,4-dienoyl-CoA reductase (NADPH2) [EC:1.3.1.34] | ec:1.3.1.34 |
| bmn:BMA10247\_A0223 | hypothetical protein |  |
| bmn:BMA10247\_A0224 | hypothetical protein |  |
| bmn:BMA10247\_A0225 | cyoA-2; ubiquinol oxidase subunit II (EC:1.10.3.-); K02297 cytochrome o ubiquinol oxidase subunit II [EC:1.10.3.-] |  |
| bmn:BMA10247\_A0226 | cyoB; ubiquinol oxidase subunit I (EC:1.10.3.-); K02298 cytochrome o ubiquinol oxidase subunit I [EC:1.10.3.-] |  |
| bmn:BMA10247\_A0227 | cyoC; ubiquinol oxidase subunit III (EC:1.10.3.-); K02299 cytochrome o ubiquinol oxidase subunit III [EC:1.10.3.-] |  |
| bmn:BMA10247\_A0228 | cyoD-2; ubiquinol oxidase subunit IV (EC:1.10.3.-); K02300 cytochrome o ubiquinol oxidase operon protein cyoD |  |
| bmn:BMA10247\_A0229 | catC; muconolactone delta-isomerase; K03464 muconolactone D-isomerase [EC:5.3.3.4] | ec:5.3.3.4 |
| bmn:BMA10247\_A0230 | catA; catechol 1,2-dioxygenase (EC:1.13.11.1); K03381 catechol 1,2-dioxygenase [EC:1.13.11.1] | ec:1.13.11.1 |
| bmn:BMA10247\_A0231 | catB; muconate cycloisomerase (EC:5.5.1.1); K01856 muconate cycloisomerase [EC:5.5.1.1] | ec:5.5.1.1 |
| bmn:BMA10247\_A0232 | catR; cat operon transcriptional activator CatR |  |
| bmn:BMA10247\_A0233 | AraC family transcriptional regulator |  |
| bmn:BMA10247\_A0234 | aromatic-ring-hydroxylating dioxygenase subunit alpha; K16319 anthranilate 1,2-dioxygenase large subunit [EC:1.14.12.1] | ec:1.14.12.1 |
| bmn:BMA10247\_A0235 | aromatic-ring-hydroxylating dioxygenase subunit beta; K16320 anthranilate 1,2-dioxygenase small subunit [EC:1.14.12.1] | ec:1.14.12.1 |
| bmn:BMA10247\_A0236 | aromatic-ring-hydroxylating dioxygenase, ferredoxin subunit; K05710 dioxygenase ferredoxin subunit |  |
| bmn:BMA10247\_A0237 | aromatic-ring-hydroxylating dioxygenase, ferredoxin reductase subunit; K00529 ferredoxin--NAD+ reductase [EC:1.18.1.3] | ec:1.18.1.3 |
| bmn:BMA10247\_A0238 | HIT family protein; K02503 histidine triad (HIT) family protein |  |
| bmn:BMA10247\_A0239 | putative phospholipase D |  |
| bmn:BMA10247\_A0240 | hypothetical protein |  |
| bmn:BMA10247\_A0241 | pseudogene |  |
| bmn:BMA10247\_A0242 | pseudogene |  |

  
**Neighborhood Representations for "bcj:BCAM0806"**  

| ID | Annotation | EC number |
| --- | --- | --- |
| bcj:BCAM0796 | hypothetical protein; K09123 hypothetical protein |  |
| bcj:BCAM0797 | hypothetical protein; K09128 hypothetical protein |  |
| bcj:BCAM0798 | AraC family regulatory protein |  |
| bcj:BCAM0799 | fadH; 2,4-dienoyl-CoA reductase (EC:1.3.1.34); K00219 2,4-dienoyl-CoA reductase (NADPH2) [EC:1.3.1.34] | ec:1.3.1.34 |
| bcj:BCAM0800 | hypothetical protein |  |
| bcj:BCAM0801 | LysR family regulatory protein |  |
| bcj:BCAM0802 | putative NAD(P)H dehydrogenase; K00355 NAD(P)H dehydrogenase (quinone) [EC:1.6.5.2] | ec:1.6.5.2 |
| bcj:BCAM0803 | catC; muconolactone delta-isomerase (EC:5.3.3.4); K03464 muconolactone D-isomerase [EC:5.3.3.4] | ec:5.3.3.4 |
| bcj:BCAM0804 | catA1; catechol 1,2-dioxygenase 1 (EC:1.13.11.1); K03381 catechol 1,2-dioxygenase [EC:1.13.11.1] | ec:1.13.11.1 |
| bcj:BCAM0805 | catB1; muconate cycloisomerase I 1 (EC:5.5.1.1); K01856 muconate cycloisomerase [EC:5.5.1.1] | ec:5.5.1.1 |
| bcj:BCAM0806 | putative cat operon regulatory protein |  |
| bcj:BCAM0807 | putative methyl-accepting chemotaxis protein; K03406 methyl-accepting chemotaxis protein |  |
| bcj:BCAM0808 | hypothetical protein |  |
| bcj:BCAM0809 | AraC family regulatory protein |  |
| bcj:BCAM0810 | putative aromatic oxygenase; K16319 anthranilate 1,2-dioxygenase large subunit [EC:1.14.12.1] | ec:1.14.12.1 |
| bcj:BCAM0811 | putative aromatic oxygenase; K16320 anthranilate 1,2-dioxygenase small subunit [EC:1.14.12.1] | ec:1.14.12.1 |
| bcj:BCAM0812 | putative aromatic hydrocarbons catabolism dioxygenase; K05710 dioxygenase ferredoxin subunit |  |
| bcj:BCAM0813 | putative aromatic hydrocarbons catabolism reductase; K00529 ferredoxin--NAD+ reductase [EC:1.18.1.3] | ec:1.18.1.3 |
| bcj:BCAM0814 | hypothetical protein |  |
| bcj:BCAM0815 | putative DNA binding protein |  |
| bcj:BCAM0816 | hypothetical protein |  |

  
**Neighborhood Representations for "bmj:BMULJ\_03555"**  

| ID | Annotation | EC number |
| --- | --- | --- |
| bmj:BMULJ\_03545 | ABC-type branched-chain amino acid transporter periplasmic component |  |
| bmj:BMULJ\_03546 | hypothetical protein |  |
| bmj:BMULJ\_03547 | AraC family transcriptional regulator |  |
| bmj:BMULJ\_03548 | fadH; NADPH2-dependent 2,4-dienoyl-CoA reductase (EC:1.3.1.34); K00219 2,4-dienoyl-CoA reductase (NADPH2) [EC:1.3.1.34] | ec:1.3.1.34 |
| bmj:BMULJ\_03549 | hypothetical protein |  |
| bmj:BMULJ\_03550 | LysR family transcriptional regulator |  |
| bmj:BMULJ\_03551 | nqo1; NAD(P)H dehydrogenase (EC:1.6.5.2); K00355 NAD(P)H dehydrogenase (quinone) [EC:1.6.5.2] | ec:1.6.5.2 |
| bmj:BMULJ\_03552 | catC; muconolactone D-isomerase (EC:5.3.3.4); K03464 muconolactone D-isomerase [EC:5.3.3.4] | ec:5.3.3.4 |
| bmj:BMULJ\_03553 | catA; catechol 1,2-dioxygenase (EC:1.13.11.1); K03381 catechol 1,2-dioxygenase [EC:1.13.11.1] | ec:1.13.11.1 |
| bmj:BMULJ\_03554 | catB; muconate cycloisomerase (EC:5.5.1.1); K01856 muconate cycloisomerase [EC:5.5.1.1] | ec:5.5.1.1 |
| bmj:BMULJ\_03555 | catR; LysR family transcriptional regulator |  |
| bmj:BMULJ\_03556 | andR; AraC family transcriptional regulator |  |
| bmj:BMULJ\_03557 | andAc; large terminal subunit of anthranilate dioxygenase; K16319 anthranilate 1,2-dioxygenase large subunit [EC:1.14.12.1] | ec:1.14.12.1 |
| bmj:BMULJ\_03558 | andAd; small terminal subunit of anthranilate dioxygenase; K16320 anthranilate 1,2-dioxygenase small subunit [EC:1.14.12.1] | ec:1.14.12.1 |
| bmj:BMULJ\_03559 | andAb; ferredoxin subunit of anthranilate dioxygenase; K05710 dioxygenase ferredoxin subunit |  |
| bmj:BMULJ\_03560 | andAa; ferredoxin-NAD+ reductase (EC:1.18.1.3); K00529 ferredoxin--NAD+ reductase [EC:1.18.1.3] | ec:1.18.1.3 |
| bmj:BMULJ\_03561 | hypothetical protein |  |
| bmj:BMULJ\_03562 | transcriptional regulator |  |
| bmj:BMULJ\_03563 | hypothetical protein |  |
| bmj:BMULJ\_03564 | ilvB; acetolactate synthase (EC:2.2.1.6); K01652 acetolactate synthase I/II/III large subunit [EC:2.2.1.6] | ec:2.2.1.6 |
| bmj:BMULJ\_03565 | NAD-dependent aldehyde dehydrogenase (EC:1.2.1.-) |  |

  
**Neighborhood Representations for "bmu:Bmul\_4959"**  

| ID | Annotation | EC number |
| --- | --- | --- |
| bmu:Bmul\_4949 | aldehyde dehydrogenase |  |
| bmu:Bmul\_4950 | acetolactate synthase; K01652 acetolactate synthase I/II/III large subunit [EC:2.2.1.6] | ec:2.2.1.6 |
| bmu:Bmul\_4951 | addiction module killer protein |  |
| bmu:Bmul\_4952 | transcriptional regulator |  |
| bmu:Bmul\_4953 | hypothetical protein |  |
| bmu:Bmul\_4954 | FAD-dependent pyridine nucleotide-disulfide oxidoreductase; K00529 ferredoxin--NAD+ reductase [EC:1.18.1.3] | ec:1.18.1.3 |
| bmu:Bmul\_4955 | Rieske (2Fe-2S) domain-containing protein; K05710 dioxygenase ferredoxin subunit |  |
| bmu:Bmul\_4956 | aromatic-ring-hydroxylating dioxygenase subunit beta; K16320 anthranilate 1,2-dioxygenase small subunit [EC:1.14.12.1] | ec:1.14.12.1 |
| bmu:Bmul\_4957 | Rieske (2Fe-2S) domain-containing protein; K16319 anthranilate 1,2-dioxygenase large subunit [EC:1.14.12.1] | ec:1.14.12.1 |
| bmu:Bmul\_4958 | AraC family transcriptional regulator |  |
| bmu:Bmul\_4959 | LysR family transcriptional regulator |  |
| bmu:Bmul\_4960 | muconate and chloromuconate cycloisomerase; K01856 muconate cycloisomerase [EC:5.5.1.1] | ec:5.5.1.1 |
| bmu:Bmul\_4961 | catechol 1,2-dioxygenase; K03381 catechol 1,2-dioxygenase [EC:1.13.11.1] | ec:1.13.11.1 |
| bmu:Bmul\_4962 | muconolactone delta-isomerase (EC:5.3.3.4); K03464 muconolactone D-isomerase [EC:5.3.3.4] | ec:5.3.3.4 |
| bmu:Bmul\_4963 | ribosyldihydronicotinamide dehydrogenase (EC:1.10.99.2); K00355 NAD(P)H dehydrogenase (quinone) [EC:1.6.5.2] | ec:1.6.5.2 |
| bmu:Bmul\_4964 | LysR family transcriptional regulator |  |
| bmu:Bmul\_4965 | hypothetical protein |  |
| bmu:Bmul\_4966 | NADH:flavin oxidoreductase; K00219 2,4-dienoyl-CoA reductase (NADPH2) [EC:1.3.1.34] | ec:1.3.1.34 |
| bmu:Bmul\_4967 | AraC family transcriptional regulator |  |
| bmu:Bmul\_4968 | hypothetical protein |  |
| bmu:Bmul\_4969 | extracellular ligand-binding receptor |  |

  
**Neighborhood Representations for "pna:Pnap\_2114"**  

| ID | Annotation | EC number |
| --- | --- | --- |
| pna:Pnap\_2104 | nitrilase/cyanide hydratase and apolipoprotein N-acyltransferase; K01502 aliphatic nitrilase [EC:3.5.5.7] | ec:3.5.5.7 |
| pna:Pnap\_2105 | hypothetical protein |  |
| pna:Pnap\_2106 | GntR family transcriptional regulator |  |
| pna:Pnap\_2107 | benzoate transporter; K05782 benzoate membrane transport protein |  |
| pna:Pnap\_2108 | major facilitator superfamily transporter; K05548 MFS transporter, AAHS family, benzoate transport protein |  |
| pna:Pnap\_2109 | benD; 1,6-dihydroxycyclohexa-2,4-diene-1-carboxylate dehydrogenase (EC:1.3.1.25); K05783 dihydroxycyclohexadiene carboxylate dehydrogenase [EC:1.3.1.25 1.3.1.-] | ec:1.3.1.25 |
| pna:Pnap\_2110 | FadD27 protein; K05784 benzoate/toluate 1,2-dioxygenase electron transfer component |  |
| pna:Pnap\_2111 | 2-chlorobenzoate 1,2-dioxygenase (EC:1.14.12.13); K05550 benzoate/toluate 1,2-dioxygenase subunit beta [EC:1.14.12.10 1.14.12.-] | ec:1.14.12.10 |
| pna:Pnap\_2112 | Rieske (2Fe-2S) domain-containing protein; K05549 benzoate/toluate 1,2-dioxygenase subunit alpha [EC:1.14.12.10 1.14.12.-] | ec:1.14.12.10 |
| pna:Pnap\_2113 | hypothetical protein |  |
| pna:Pnap\_2114 | LysR family transcriptional regulator |  |
| pna:Pnap\_2115 | hemerythrin-like metal-binding protein |  |
| pna:Pnap\_2116 | intradiol ring-cleavage dioxygenase; K03381 catechol 1,2-dioxygenase [EC:1.13.11.1] | ec:1.13.11.1 |
| pna:Pnap\_2117 | hypothetical protein |  |
| pna:Pnap\_2118 | muconolactone delta-isomerase (EC:5.3.3.4); K03464 muconolactone D-isomerase [EC:5.3.3.4] | ec:5.3.3.4 |
| pna:Pnap\_2119 | muconate and chloromuconate cycloisomerase (EC:5.5.1.1); K01856 muconate cycloisomerase [EC:5.5.1.1] | ec:5.5.1.1 |
| pna:Pnap\_2120 | LysR family transcriptional regulator |  |
| pna:Pnap\_2121 | alpha/beta hydrolase fold protein; K01055 3-oxoadipate enol-lactonase [EC:3.1.1.24] | ec:3.1.1.24 |
| pna:Pnap\_2122 | MarR family transcriptional regulator |  |
| pna:Pnap\_2123 | short-chain dehydrogenase/reductase SDR; K07535 2-hydroxycyclohexanecarboxyl-CoA dehydrogenase [EC:1.1.1.-] |  |
| pna:Pnap\_2124 | naphthoate synthase; K07536 2-ketocyclohexanecarboxyl-CoA hydrolase [EC:3.1.2.-] |  |

  
**Neighborhood Representations for "met:M446\_1323"**  

| ID | Annotation | EC number |
| --- | --- | --- |
| met:M446\_1313 | oxidoreductase domain-containing protein |  |
| met:M446\_1314 | ABC transporter-like protein; K10112 multiple sugar transport system ATP-binding protein |  |
| met:M446\_1315 | binding-protein-dependent transport system inner membrane protein; K02026 multiple sugar transport system permease protein |  |
| met:M446\_1316 | binding-protein-dependent transport system inner membrane protein; K02025 multiple sugar transport system permease protein |  |
| met:M446\_1317 | extracellular solute-binding protein; K02027 multiple sugar transport system substrate-binding protein |  |
| met:M446\_1318 | dihydroxy-acid dehydratase; K01687 dihydroxy-acid dehydratase [EC:4.2.1.9] | ec:4.2.1.9 |
| met:M446\_1319 | pseudogene |  |
| met:M446\_1320 | hypothetical protein |  |
| met:M446\_1321 | TRAP-type transport system periplasmic component-like protein |  |
| met:M446\_1322 | pseudogene |  |
| met:M446\_1323 | LysR family transcriptional regulator |  |
| met:M446\_1324 | benzoate 1,2-dioxygenase, large subunit; K05549 benzoate/toluate 1,2-dioxygenase subunit alpha [EC:1.14.12.10 1.14.12.-] | ec:1.14.12.10 |
| met:M446\_1325 | benzoate 1,2-dioxygenase small subunit (EC:1.14.12.10); K05550 benzoate/toluate 1,2-dioxygenase subunit beta [EC:1.14.12.10 1.14.12.-] | ec:1.14.12.10 |
| met:M446\_1326 | oxidoreductase FAD/NAD(P)-binding subunit; K05784 benzoate/toluate 1,2-dioxygenase electron transfer component |  |
| met:M446\_1327 | benD; 1,6-dihydroxycyclohexa-2,4-diene-1-carboxylate dehydrogenase; K05783 dihydroxycyclohexadiene carboxylate dehydrogenase [EC:1.3.1.25 1.3.1.-] | ec:1.3.1.25 |
| met:M446\_1328 | catechol 1,2-dioxygenase; K03381 catechol 1,2-dioxygenase [EC:1.13.11.1] | ec:1.13.11.1 |
| met:M446\_1329 | muconate and chloromuconate cycloisomerase; K01856 muconate cycloisomerase [EC:5.5.1.1] | ec:5.5.1.1 |
| met:M446\_1330 | muconolactone delta-isomerase; K03464 muconolactone D-isomerase [EC:5.3.3.4] | ec:5.3.3.4 |
| met:M446\_1331 | major facilitator superfamily transporter; K08195 MFS transporter, AAHS family, 4-hydroxybenzoate transporter |  |
| met:M446\_1332 | hypothetical protein |  |
| met:M446\_1333 | hypothetical protein |  |

  
**Neighborhood Representations for "sjp:SJA\_C1-25210"**  

| ID | Annotation | EC number |
| --- | --- | --- |
| sjp:SJA\_C1-25110 | putative transposase |  |
| sjp:SJA\_C1-25120 | putative hydrolase; K07047 |  |
| sjp:SJA\_C1-25130 | hypothetical protein |  |
| sjp:SJA\_C1-25140 | TonB-dependent receptor-like protein |  |
| sjp:SJA\_C1-25150 | hypothetical protein |  |
| sjp:SJA\_C1-25160 | catD; beta-ketoadipate enol-lactone hydrolase (EC:3.1.1.24); K01055 3-oxoadipate enol-lactonase [EC:3.1.1.24] | ec:3.1.1.24 |
| sjp:SJA\_C1-25170 | pcaI\_pcaF\_catF; PcaI/PcaF/CatF (EC:2.3.1.- 2.8.3.5) |  |
| sjp:SJA\_C1-25180 | catA\_pcaH; catechol 1,2-dioxygenase (EC:1.13.11.1 1.13.11.3); K03381 catechol 1,2-dioxygenase [EC:1.13.11.1] | ec:1.13.11.1 |
| sjp:SJA\_C1-25190 | catC; muconolactone delta-isomerase (EC:5.3.3.4); K03464 muconolactone D-isomerase [EC:5.3.3.4] | ec:5.3.3.4 |
| sjp:SJA\_C1-25200 | catB; muconate cycloisomerase I (EC:5.5.1.1); K01856 muconate cycloisomerase [EC:5.5.1.1] | ec:5.5.1.1 |
| sjp:SJA\_C1-25210 | catR; LysR family transcriptional regulator |  |
| sjp:SJA\_C1-25220 | andAc; large subunit of anthranilate dioxygenase (EC:1.14.12.-); K16319 anthranilate 1,2-dioxygenase large subunit [EC:1.14.12.1] | ec:1.14.12.1 |
| sjp:SJA\_C1-25230 | andAd; small subunit of anthranilate dioxygenase (EC:1.14.12.-); K16320 anthranilate 1,2-dioxygenase small subunit [EC:1.14.12.1] | ec:1.14.12.1 |
| sjp:SJA\_C1-25240 | andAb; anthranilate dioxygenase ferredoxin; K05710 dioxygenase ferredoxin subunit |  |
| sjp:SJA\_C1-25250 | andAa; anthranilate dioxygenase ferredoxin reductase (EC:1.18.1.3); K00529 ferredoxin--NAD+ reductase [EC:1.18.1.3] | ec:1.18.1.3 |
| sjp:SJA\_C1-25260 | andR; AraC family transcriptional regulator |  |
| sjp:SJA\_C1-25270 | putative transposase |  |
| sjp:SJA\_C1-25280 | putative alpha/beta hydrolase |  |
| sjp:SJA\_C1-25290 | cpo; chloride peroxidase (EC:1.11.1.10); K00433 Non-heme chloroperoxidase [EC:1.11.1.10] | ec:1.11.1.10 |
| sjp:SJA\_C1-25300 | hypothetical protein |  |
| sjp:SJA\_C1-25310 | putative hydrolase; K07047 |  |

  
**Neighborhood Representations for "nar:Saro\_0537"**  

| ID | Annotation | EC number |
| --- | --- | --- |
| nar:Saro\_0527 | ParB family protein; K03497 chromosome partitioning protein, ParB family |  |
| nar:Saro\_0528 | RepB plasmid partition |  |
| nar:Saro\_0529 | hypothetical protein |  |
| nar:Saro\_0530 | hypothetical protein; K01884 cysteinyl-tRNA synthetase, unknown class [EC:6.1.1.16] | ec:6.1.1.16 |
| nar:Saro\_0531 | acyltransferase |  |
| nar:Saro\_0532 | TetR family transcriptional regulator |  |
| nar:Saro\_0533 | sulfatase; K01130 arylsulfatase [EC:3.1.6.1] | ec:3.1.6.1 |
| nar:Saro\_0534 | sulfatase; K01130 arylsulfatase [EC:3.1.6.1] | ec:3.1.6.1 |
| nar:Saro\_0535 | TonB-dependent receptor |  |
| nar:Saro\_0536 | hypothetical protein |  |
| nar:Saro\_0537 | LysR family transcriptional regulator |  |
| nar:Saro\_0538 | Rieske (2Fe-2S) protein; K05549 benzoate/toluate 1,2-dioxygenase subunit alpha [EC:1.14.12.10 1.14.12.-] | ec:1.14.12.10 |
| nar:Saro\_0539 | 2-chlorobenzoate 1,2-dioxygenase (EC:1.14.12.13); K05550 benzoate/toluate 1,2-dioxygenase subunit beta [EC:1.14.12.10 1.14.12.-] | ec:1.14.12.10 |
| nar:Saro\_0540 | benD; 1,6-dihydroxycyclohexa-2,4-diene-1-carboxylate dehydrogenase (EC:1.3.1.25); K05783 dihydroxycyclohexadiene carboxylate dehydrogenase [EC:1.3.1.25 1.3.1.-] | ec:1.3.1.25 |
| nar:Saro\_0541 | LysR family transcriptional regulator |  |
| nar:Saro\_0542 | glycoside hydrolase |  |
| nar:Saro\_0543 | galactose-1-phosphate uridylyltransferase (EC:2.7.7.10 2.7.7.12); K00965 UDPglucose--hexose-1-phosphate uridylyltransferase [EC:2.7.7.12] | ec:2.7.7.12 |
| nar:Saro\_0544 | galactokinase (EC:2.7.1.6); K00849 galactokinase [EC:2.7.1.6] | ec:2.7.1.6 |
| nar:Saro\_0545 | Outer membrane autotransporter barrel protein |  |
| nar:Saro\_0546 | RNA polymerase sigma factor; K03088 RNA polymerase sigma-70 factor, ECF subfamily |  |
| nar:Saro\_0547 | hypothetical protein |  |

  
**Neighborhood Representations for "bgl:bglu\_2g07130"**  

| ID | Annotation | EC number |
| --- | --- | --- |
| bgl:bglu\_2g07030 | ornithine cyclodeaminase; K01750 ornithine cyclodeaminase [EC:4.3.1.12] | ec:4.3.1.12 |
| bgl:bglu\_2g07040 | isoprenylcysteine carboxyl methyltransferase |  |
| bgl:bglu\_2g07050 | iron-containing alcohol dehydrogenase; K13954 alcohol dehydrogenase [EC:1.1.1.1] | ec:1.1.1.1 |
| bgl:bglu\_2g07060 | polygalacturonase |  |
| bgl:bglu\_2g07070 | lytic transglycosylase catalytic subunit |  |
| bgl:bglu\_2g07080 | pseudogene |  |
| bgl:bglu\_2g07090 | yciF protein |  |
| bgl:bglu\_2g07100 | hypothetical protein |  |
| bgl:bglu\_2g07110 | succinate dehydrogenase iron-sulfur subunit; K00240 succinate dehydrogenase iron-sulfur subunit [EC:1.3.99.1] | ec:1.3.99.1 |
| bgl:bglu\_2g07120 | hypothetical protein |  |
| bgl:bglu\_2g07130 | LysR family transcriptional regulator |  |
| bgl:bglu\_2g07140 | Muconate and chloromuconate cycloisomerase; K01856 muconate cycloisomerase [EC:5.5.1.1] | ec:5.5.1.1 |
| bgl:bglu\_2g07150 | Muconolactone Delta-isomerase; K03464 muconolactone D-isomerase [EC:5.3.3.4] | ec:5.3.3.4 |
| bgl:bglu\_2g07160 | Catechol 1,2-dioxygenase; K03381 catechol 1,2-dioxygenase [EC:1.13.11.1] | ec:1.13.11.1 |
| bgl:bglu\_2g07170 | hypothetical protein |  |
| bgl:bglu\_2g07180 | small GTP-binding protein |  |
| bgl:bglu\_2g07190 | pseudogene |  |
| bgl:bglu\_2g07200 | glycogen debranching protein GlgX; K02438 glycogen operon protein [EC:3.2.1.-] |  |
| bgl:bglu\_2g07210 | glycoside hydrolase |  |
| bgl:bglu\_2g07220 | PAS/PAC sensor-containing diguanylate cyclase |  |
| bgl:bglu\_2g07230 | FKBP-type peptidylprolyl isomerase; K03772 FKBP-type peptidyl-prolyl cis-trans isomerase FkpA [EC:5.2.1.8] | ec:5.2.1.8 |

  
**Neighborhood Representations for "spe:Spro\_1405"**  

| ID | Annotation | EC number |
| --- | --- | --- |
| spe:Spro\_1395 | NLPA lipoprotein; K02073 D-methionine transport system substrate-binding protein |  |
| spe:Spro\_1396 | binding-protein-dependent transport systems inner membrane component; K02072 D-methionine transport system permease protein |  |
| spe:Spro\_1397 | ABC transporter-like protein; K02071 D-methionine transport system ATP-binding protein |  |
| spe:Spro\_1398 | GCN5-like N-acetyltransferase; K03827 putative acetyltransferase [EC:2.3.1.-] |  |
| spe:Spro\_1399 | LysR family transcriptional regulator (EC:4.2.1.1) |  |
| spe:Spro\_1400 | alcohol dehydrogenase; K00344 NADPH2:quinone reductase [EC:1.6.5.5] | ec:1.6.5.5 |
| spe:Spro\_1401 | hypothetical protein |  |
| spe:Spro\_1402 | hypothetical protein |  |
| spe:Spro\_1403 | isochorismatase hydrolase |  |
| spe:Spro\_1404 | short-chain dehydrogenase/reductase SDR; K00059 3-oxoacyl-[acyl-carrier protein] reductase [EC:1.1.1.100] | ec:1.1.1.100 |
| spe:Spro\_1405 | LysR family transcriptional regulator |  |
| spe:Spro\_1406 | tryptophanyl-tRNA synthetase II (EC:6.1.1.2); K01867 tryptophanyl-tRNA synthetase [EC:6.1.1.2] | ec:6.1.1.2 |
| spe:Spro\_1407 | major facilitator transporter |  |
| spe:Spro\_1408 | GntR family transcriptional regulator |  |
| spe:Spro\_1409 | alcohol dehydrogenase |  |
| spe:Spro\_1410 | phosphonate ABC transporter ATPase; K02041 phosphonate transport system ATP-binding protein [EC:3.6.3.28] | ec:3.6.3.28 |
| spe:Spro\_1411 | phosphonate ABC transporter periplasmic phosphonate-binding protein; K02044 phosphonate transport system substrate-binding protein |  |
| spe:Spro\_1412 | phosphonate ABC transporter inner membrane subunit; K02042 phosphonate transport system permease protein |  |
| spe:Spro\_1413 | phosphonate ABC transporter inner membrane subunit; K02042 phosphonate transport system permease protein |  |
| spe:Spro\_1414 | hypothetical protein |  |
| spe:Spro\_1415 | methyl-accepting chemotaxis sensory transducer |  |

  
**Neighborhood Representations for "apt:APA01\_13830"**  

| ID | Annotation | EC number |
| --- | --- | --- |
| apt:APA01\_13730 | transposase |  |
| apt:APA01\_13740 | transposase |  |
| apt:APA01\_13750 | transposase |  |
| apt:APA01\_13760 | TonB-dependent receptor |  |
| apt:APA01\_13770 | transporter |  |
| apt:APA01\_13780 | hypothetical protein |  |
| apt:APA01\_13790 | transposase |  |
| apt:APA01\_13800 | D-galactonate transporter; K03535 MFS transporter, ACS family, glucarate transporter |  |
| apt:APA01\_13810 | transposase |  |
| apt:APA01\_13820 | D-galactonate transporter |  |
| apt:APA01\_13830 | lysR; LysR family transcriptional regulator |  |
| apt:APA01\_13840 | dihydroxy-acid dehydratase; K01687 dihydroxy-acid dehydratase [EC:4.2.1.9] | ec:4.2.1.9 |
| apt:APA01\_13850 | 2,4-dihydroxyhept-2-ene-1,7-dioic acid aldolase; K02510 4-hydroxy-2-oxoheptanedioate aldolase [EC:4.1.2.52] | ec:4.1.2.52 |
| apt:APA01\_13860 | N-acyl-D-glutamate deacylase |  |
| apt:APA01\_13870 | transposase |  |
| apt:APA01\_13880 | N-acyl-D-glutamate deacylase |  |
| apt:APA01\_13890 | lysR; LysR family transcriptional regulator |  |
| apt:APA01\_13900 | alcohol dehydrogenase |  |
| apt:APA01\_13910 | sugar transporter |  |
| apt:APA01\_13920 | aldehyde/methylmalonate-semialdehyde dehydrogenase; K00140 malonate-semialdehyde dehydrogenase (acetylating) / methylmalonate-semialdehyde dehydrogenase [EC:1.2.1.18 1.2.1.27] | ec:1.2.1.18 ec:1.2.1.27 |
| apt:APA01\_13930 | nitrate/sulfonate/bicarbonate ABC transporter periplasmic protein; K02051 NitT/TauT family transport system substrate-binding protein |  |

  
**Neighborhood Representations for "dma:DMR\_13820"**  

| ID | Annotation | EC number |
| --- | --- | --- |
| dma:DMR\_13720 | glycosyltransferase |  |
| dma:DMR\_13730 | flagellar hook-length control protein; K02414 flagellar hook-length control protein FliK |  |
| dma:DMR\_13740 | flgD; flagellar basal-body rod modification protein FlgD; K02389 flagellar basal-body rod modification protein FlgD |  |
| dma:DMR\_13750 | flgE; flagellar hook protein FlgE; K02390 flagellar hook protein FlgE |  |
| dma:DMR\_13770 | hypothetical protein |  |
| dma:DMR\_13760 | fliC; flagellin; K02406 flagellin |  |
| dma:DMR\_13780 | rnc; ribonuclease III; K03685 ribonuclease III [EC:3.1.26.3] | ec:3.1.26.3 |
| dma:DMR\_13790 | hypothetical protein; K06873 |  |
| dma:DMR\_13800 | two-component hybrid sensor and regulator |  |
| dma:DMR\_13810 | hypothetical protein |  |
| dma:DMR\_13820 | LysR family transcriptional regulator |  |
| dma:DMR\_13830 | carboxymuconolactone decarboxylase family protein; K01607 4-carboxymuconolactone decarboxylase [EC:4.1.1.44] | ec:4.1.1.44 |
| dma:DMR\_13840 | hypothetical protein |  |
| dma:DMR\_13850 | hypothetical protein |  |
| dma:DMR\_13860 | hypothetical protein |  |
| dma:DMR\_13870 | hypothetical protein |  |
| dma:DMR\_13880 | npdA; NAD-dependent deacetylase; K12410 NAD-dependent deacetylase [EC:3.5.1.-] |  |
| dma:DMR\_13890 | protein TolQ; K03562 biopolymer transport protein TolQ |  |
| dma:DMR\_13900 | biopolymer transport protein; K03560 biopolymer transport protein TolR |  |
| dma:DMR\_13910 | hypothetical protein; K03646 colicin import membrane protein |  |
| dma:DMR\_13920 | TolB protein precursor; K03641 TolB protein |  |

  
**Over-represented Enzyme Summary**: Table of E.C. identified protein in the "Neighborhood Representation" ranked by frequency of occurrence  

| EC number | Frequency | Annotation | Reactions |
| --- | --- | --- | --- |
| ec:1.14.12.1 | 40 | anthranilate 1,2-dioxygenase (deaminating, decarboxylating); anthranilate hydroxylase; anthranilic hydroxylase; anthranilic acid hydroxylase | anthranilate + NAD(P)H + 2 H+ + O2 = catechol + CO2 + NAD(P)+ + NH3 [RN:R00823 R00825] |
| ec:5.3.3.4 | 26 | muconolactone Delta-isomerase; muconolactone isomerase | (S)-5-oxo-2,5-dihydrofuran-2-acetate = 5-oxo-4,5-dihydrofuran-2-acetate [RN:R06990] |
| ec:5.5.1.1 | 26 | muconate cycloisomerase; muconate cycloisomerase I; cis,cis-muconate-lactonizing enzyme; cis,cis-muconate cycloisomerase; muconate lactonizing enzyme; 4-carboxymethyl-4-hydroxyisocrotonolactone lyase (decyclizing); CatB; MCI | 2,5-dihydro-5-oxofuran-2-acetate = cis,cis-hexadienedioate [RN:R03959] |
| ec:1.13.11.1 | 26 | catechol 1,2-dioxygenase; catechol-oxygen 1,2-oxidoreductase; 1,2-pyrocatechase; catechase; catechol 1,2-oxygenase; catechol dioxygenase; pyrocatechase; pyrocatechol 1,2-dioxygenase; CD I; CD II | catechol + O2 = cis,cis-muconate [RN:R00817] |
| ec:1.18.1.3 | 20 | ferredoxin---NAD+ reductase; ferredoxin-nicotinamide adenine dinucleotide reductase; ferredoxin reductase (ambiguous); NAD+-ferredoxin reductase; NADH-ferredoxin oxidoreductase; reductase, reduced nicotinamide adenine dinucleotide-ferredoxin; ferredoxin-NAD+ reductase; NADH-ferredoxin reductase; NADH2-ferredoxin oxidoreductase; NADH flavodoxin oxidoreductase; NADH-ferredoxin NAP reductase (component of naphthalene dioxygenase multicomponent enzyme system); ferredoxin-linked NAD+ reductase; NADH-ferredoxin TOL reductase (component of toluene dioxygenase); ferredoxin---NAD reductase | (1) 2 reduced [2Fe-2S] ferredoxin + NAD+ + H+ = 2 oxidized [2Fe-2S] ferredoxin + NADH [RN:R05875]; (2) reduced 2[4Fe-4S] ferredoxin + NAD+ + H+ = oxidized 2[4Fe-4S] ferredoxin + NADH |
| ec:1.3.1.34 | 17 | 2,4-dienoyl-CoA reductase (NADPH); 4-enoyl-CoA reductase (NADPH); 4-enoyl coenzyme A (reduced nicotinamide adenine dinucleotide phosphate) reductase; 4-enoyl-CoA reductase; 2,4-dienoyl-CoA reductase (NADPH) | trans-2,3-didehydroacyl-CoA + NADP+ = trans,trans-2,3,4,5-tetradehydroacyl-CoA + NADPH + H+ [RN:R04319] |
| ec:1.6.5.2 | 9 | NAD(P)H dehydrogenase (quinone); menadione reductase; phylloquinone reductase; quinone reductase; dehydrogenase, reduced nicotinamide adenine dinucleotide (phosphate, quinone); DT-diaphorase; flavoprotein NAD(P)H-quinone reductase; menadione oxidoreductase; NAD(P)H dehydrogenase; NAD(P)H menadione reductase; NAD(P)H-quinone dehydrogenase; NAD(P)H-quinone oxidoreductase; NAD(P)H: (quinone-acceptor)oxidoreductase; NAD(P)H: menadione oxidoreductase; NADH-menadione reductase; naphthoquinone reductase; p-benzoquinone reductase; reduced NAD(P)H dehydrogenase; viologen accepting pyridine nucleotide oxidoreductase; vitamin K reductase; diaphorase; reduced nicotinamide-adenine dinucleotide (phosphate) dehydrogenase; vitamin-K reductase; NAD(P)H2 dehydrogenase (quinone); NQO1; QR1; NAD(P)H:(quinone-acceptor) oxidoreductase | NAD(P)H + H+ + a quinone = NAD(P)+ + a hydroquinone [RN:R07358 R07359] |
| ec:1.14.12.10 | 6 | benzoate 1,2-dioxygenase; benzoate hydroxylase; benzoate hydroxylase; benzoic hydroxylase; benzoate dioxygenase; benzoate,NADH:oxygen oxidoreductase (1,2-hydroxylating, decarboxylating) [incorrect] | benzoate + NADH + H+ + O2 = (1R,6S)-1,6-dihydroxycyclohexa-2,4-diene-1-carboxylate + NAD+ [RN:R07188] |
| ec:1.1.1.100 | 5 | 3-oxoacyl-[acyl-carrier-protein] reductase; beta-ketoacyl-[acyl-carrier protein](ACP) reductase; beta-ketoacyl acyl carrier protein (ACP) reductase; beta-ketoacyl reductase; beta-ketoacyl thioester reductase; beta-ketoacyl-ACP reductase; beta-ketoacyl-acyl carrier protein reductase; 3-ketoacyl acyl carrier protein reductase; NADPH-specific 3-oxoacyl-[acylcarrier protein]reductase; 3-oxoacyl-[ACP]reductase; (3R)-3-hydroxyacyl-[acyl-carrier-protein]:NADP+ oxidoreductase | a (3R)-3-hydroxyacyl-[acyl-carrier protein] + NADP+ = a 3-oxoacyl-[acyl-carrier protein] + NADPH + H+ [RN:R02767] |
| ec:2.2.1.6 | 4 | acetolactate synthase; alpha-acetohydroxy acid synthetase; alpha-acetohydroxyacid synthase; alpha-acetolactate synthase; alpha-acetolactate synthetase; acetohydroxy acid synthetase; acetohydroxyacid synthase; acetolactate pyruvate-lyase (carboxylating); acetolactic synthetase | 2 pyruvate = 2-acetolactate + CO2 [RN:R00006] |
| ec:1.3.1.25 | 3 | 1,6-dihydroxycyclohexa-2,4-diene-1-carboxylate dehydrogenase; 3,5-cyclohexadiene-1,2-diol-1-carboxylate dehydrogenase; 3,5-cyclohexadiene-1,2-diol-1-carboxylic acid dehydrogenase; dihydrodihydroxybenzoate dehydrogenase; DHBDH; cis-1,2-dihydroxycyclohexa-3,5-diene-1-carboxylate dehydrogenase; 2-hydro-1,2-dihydroxybenzoate dehydrogenase; cis-1,2-dihydroxycyclohexa-3,5-diene-1-carboxylate:NAD+ oxidoreductase; dihydrodihydroxybenzoate dehydrogenase; (1R,6R)-1,6-dihydroxycyclohexa-2,4-diene-1-carboxylate:NAD+ oxidoreductase (decarboxylating) | (1R,6S)-1,6-dihydroxycyclohexa-2,4-diene-1-carboxylate + NAD+ = catechol + CO2 + NADH + H+ [RN:R00813] |
| ec:3.11.1.1 | 2 | phosphonoacetaldehyde hydrolase; phosphonatase; 2-phosphonoacetylaldehyde phosphonohydrolase | phosphonoacetaldehyde + H2O = acetaldehyde + phosphate [RN:R00747] |
| ec:3.1.1.24 | 2 | 3-oxoadipate enol-lactonase; carboxymethylbutenolide lactonase; beta-ketoadipic enol-lactone hydrolase; 3-ketoadipate enol-lactonase; 3-oxoadipic enol-lactone hydrolase; beta-ketoadipate enol-lactone hydrolase | 3-oxoadipate enol-lactone + H2O = 3-oxoadipate [RN:R02991] |
| ec:3.1.6.1 | 2 | arylsulfatase; sulfatase; nitrocatechol sulfatase; phenolsulfatase; phenylsulfatase; p-nitrophenyl sulfatase; arylsulfohydrolase; 4-methylumbelliferyl sulfatase; estrogen sulfatase | a phenol sulfate + H2O = a phenol + sulfate [RN:R01243] |
| ec:1.14.13.82 | 2 | vanillate monooxygenase; 4-hydroxy-3-methoxybenzoate demethylase; vanillate demethylase | vanillate + O2 + NADH + H+ = 3,4-dihydroxybenzoate + NAD+ + H2O + formaldehyde [RN:R05274] |
| ec:4.2.1.9 | 2 | dihydroxy-acid dehydratase; acetohydroxyacid dehydratase; alpha,beta-dihydroxyacid dehydratase; 2,3-dihydroxyisovalerate dehydratase; alpha,beta-dihydroxyisovalerate dehydratase; dihydroxy acid dehydrase; DHAD; 2,3-dihydroxy-acid hydro-lyase | 2,3-dihydroxy-3-methylbutanoate = 3-methyl-2-oxobutanoate + H2O [RN:R01209] |
| ec:3.5.1.4 | 2 | amidase; acylamidase; acylase (misleading); amidohydrolase (ambiguous); deaminase (ambiguous); fatty acylamidase; N-acetylaminohydrolase (ambiguous) | a monocarboxylic acid amide + H2O = a monocarboxylate + NH3 [RN:R03909] |
| ec:2.7.1.6 | 1 | galactokinase; galactokinase (phosphorylating); ATP:D-galactose-1-phosphotransferase | ATP + alpha-D-galactose = ADP + alpha-D-galactose 1-phosphate [RN:R01092] |
| ec:1.14.12.17 | 1 | nitric oxide dioxygenase | 2 nitric oxide + 2 O2 + NAD(P)H = 2 nitrate + NAD(P)+ + H+ [RN:R05724 R05725] |
| ec:3.5.5.7 | 1 | aliphatic nitrilase | R-CN + 2 H2O = R-COOH + NH3 [RN:R00540] |
| ec:2.6.1.37 | 1 | 2-aminoethylphosphonate---pyruvate transaminase; (2-aminoethyl)phosphonate transaminase; (2-aminoethyl)phosphonate aminotransferase; (2-aminoethyl)phosphonic acid aminotransferase; 2-aminoethylphosphonate-pyruvate aminotransferase; 2-aminoethylphosphonate aminotransferase; 2-aminoethylphosphonate transaminase; AEP transaminase; AEPT | (2-aminoethyl)phosphonate + pyruvate = 2-phosphonoacetaldehyde + L-alanine [RN:R04152] |
| ec:3.4.13.21 | 1 | dipeptidase E; aspartyl dipeptidase; peptidase E; PepE gene product (Salmonella typhimurium) | Dipeptidase E catalyses the hydrolysis of dipeptides Asp!Xaa. It does not act on peptides with N-terminal Glu, Asn or Gln, nor does it cleave isoaspartyl peptides |
| ec:1.11.1.10 | 1 | chloride peroxidase; chloroperoxidase; CPO; vanadium haloperoxidase | RH + chloride + H2O2 = RCl + 2 H2O [RN:R00052] |
| ec:1.3.99.1 | 1 | succinate dehydrogenase; succinic dehydrogenase; fumarate reductase; fumaric hydrogenase; succinodehydrogenase; succinic acid dehydrogenase; succinate oxidoreductase; succinyl dehydrogenase; succinate:(acceptor) oxidoreductase | succinate + acceptor = fumarate + reduced acceptor [RN:R00412] |
| ec:1.2.1.27 | 1 | methylmalonate-semialdehyde dehydrogenase (acylating); MSDH; MMSA dehydrogenase | 2-methyl-3-oxopropanoate + CoA + H2O + NAD+ = propanoyl-CoA + HCO3- + NADH [RN:R00922] |
| ec:3.6.3.28 | 1 | phosphonate-transporting ATPase | ATP + H2O + phosphonateout = ADP + phosphate + phosphonatein [RN:R00086] |
| ec:5.2.1.8 | 1 | peptidylprolyl isomerase; PPIase; cyclophilin [misleading, see comments]; peptide bond isomerase; peptidyl-prolyl cis-trans isomerase | peptidylproline (omega=180) = peptidylproline (omega=0) [RN:R04273] |
| ec:4.1.1.44 | 1 | 4-carboxymuconolactone decarboxylase; gamma-4-carboxymuconolactone decarboxylase; 4-carboxymuconolactone carboxy-lyase; 2-carboxy-2,5-dihydro-5-oxofuran-2-acetate carboxy-lyase (4,5-dihydro-5-oxofuran-2-acetate-forming) | (R)-2-carboxy-2,5-dihydro-5-oxofuran-2-acetate = 4,5-dihydro-5-oxofuran-2-acetate + CO2 [RN:R03470] |
| ec:4.2.1.1 | 1 | carbonate dehydratase; carbonic anhydrase; anhydrase; carbonate anhydrase; carbonic acid anhydrase; carboxyanhydrase; carbonic anhydrase A; carbonate hydro-lyase | H2CO3 = CO2 + H2O [RN:R00132] |
| ec:4.3.1.12 | 1 | ornithine cyclodeaminase; ornithine cyclase; ornithine cyclase (deaminating); L-ornithine ammonia-lyase (cyclizing) | L-ornithine = L-proline + NH3 [RN:R00671] |
| ec:4.1.2.52 | 1 | 4-hydroxy-2-oxoheptanedioate aldolase; 2,4-dihydroxyhept-2-enedioate aldolase; HHED aldolase; 4-hydroxy-2-ketoheptanedioate aldolase; HKHD aldolase; HpcH; HpaI | 4-hydroxy-2-oxoheptanedioate = pyruvate + succinate semialdehyde [RN:R01645] |
| ec:2.4.1.80 | 1 | ceramide glucosyltransferase; UDP-glucose:ceramide glucosyltransferase; ceramide:UDP-Glc glucosyltransferase; uridine diphosphoglucose-ceramide glucosyltransferase; ceramide:UDP-glucose glucosyltransferase; glucosylceramide synthase | UDP-glucose + an N-acylsphingosine = UDP + a D-glucosyl-N-acylsphingosine [RN:R01497 R06275] |
| ec:6.1.1.16 | 1 | cysteine---tRNA ligase; cysteinyl-tRNA synthetase; cysteinyl-transferRNA synthetase; cysteinyl-transfer ribonucleate synthetase; cysteine translase | ATP + L-cysteine + tRNACys = AMP + diphosphate + L-cysteinyl-tRNACys [RN:R03650] |
| ec:1.2.1.18 | 1 | malonate-semialdehyde dehydrogenase (acetylating); malonic semialdehyde oxidative decarboxylase | 3-oxopropanoate + CoA + NAD(P)+ = acetyl-CoA + CO2 + NAD(P)H [RN:R00705 R00706] |
| ec:6.1.1.2 | 1 | tryptophan---tRNA ligase; tryptophanyl-tRNA synthetase; L-tryptophan-tRNATrp ligase (AMP-forming); tryptophanyl-transfer ribonucleate synthetase; tryptophanyl-transfer ribonucleic acid synthetase; tryptophanyl-transfer RNA synthetase; tryptophanyl ribonucleic synthetase; tryptophanyl-transfer ribonucleic synthetase; tryptophanyl-tRNA synthase; tryptophan translase; TrpRS | ATP + L-tryptophan + tRNATrp = AMP + diphosphate + L-tryptophyl-tRNATrp [RN:R03664] |
| ec:1.1.1.1 | 1 | alcohol dehydrogenase; aldehyde reductase; ADH; alcohol dehydrogenase (NAD); aliphatic alcohol dehydrogenase; ethanol dehydrogenase; NAD-dependent alcohol dehydrogenase; NAD-specific aromatic alcohol dehydrogenase; NADH-alcohol dehydrogenase; NADH-aldehyde dehydrogenase; primary alcohol dehydrogenase; yeast alcohol dehydrogenase | (1) a primary alcohol + NAD+ = an aldehyde + NADH + H+ [RN:R07326]; (2) a secondary alcohol + NAD+ = a ketone + NADH + H+ [RN:R07327] |
| ec:2.7.13.3 | 1 | histidine kinase; EnvZ; histidine kinase (ambiguous); histidine protein kinase (ambiguous); protein histidine kinase (ambiguous); protein kinase (histidine) (ambiguous); HK1; HP165; Sln1p | ATP + protein L-histidine = ADP + protein N-phospho-L-histidine |
| ec:4.2.1.104 | 1 | cyanase; cyanate lyase; cyanate hydrolase; cyanate aminohydrolase; cyanate C-N-lyase; cyanate hydratase | cyanate + HCO3- + 2 H+ = NH3 + 2 CO2 (overall reaction) [RN:R10079]; (1a) cyanate + HCO3- + H+ = carbamate + CO2 [RN:R03546]; (1b) carbamate + H+ = NH3 + CO2 (spontaneous) [RN:R07316] |
| ec:3.1.26.3 | 1 | ribonuclease III; RNase III; ribonuclease 3 | Endonucleolytic cleavage to a 5'-phosphomonoester |
| ec:6.3.4.5 | 1 | argininosuccinate synthase; citrulline---aspartate ligase; argininosuccinate synthetase; arginine succinate synthetase; argininosuccinic acid synthetase; arginosuccinate synthetase | ATP + L-citrulline + L-aspartate = AMP + diphosphate + 2-(Nomega-L-arginino)succinate [RN:R01954] |
| ec:2.7.7.12 | 1 | UDP-glucose---hexose-1-phosphate uridylyltransferase; uridyl transferase; hexose-1-phosphate uridylyltransferase; uridyltransferase; hexose 1-phosphate uridyltransferase; UDP-glucose:alpha-D-galactose-1-phosphate uridylyltransferase | UDP-alpha-D-glucose + alpha-D-galactose 1-phosphate = alpha-D-glucose 1-phosphate + UDP-alpha-D-galactose [RN:R00955] |
| ec:1.6.5.5 | 1 | NADPH:quinone reductase; NADPH2:quinone reductase | NADPH + H+ + 2 quinone = NADP+ + 2 semiquinone [RN:R02364] |

  
**Over-represented Metabolite Summary**: Collection of the metabolites identified as substrates or products of the proteins representaed the "Over-represented Enzyme Summary" ranked by frequency of occurrence  

| ID | Structure | Name | Frequency | EC |
| --- | --- | --- | --- | --- |
| cpd:C00011 |  | CO2; Carbon dioxide | 117 | ec:4.2.1.1 ec:5.5.1.1 ec:2.2.1.6 ec:1.18.1.3 ec:1.2.1.18 ec:1.14.12.1 ec:4.2.1.104 ec:1.2.1.27 ec:1.3.1.25 ec:4.1.1.44 |
| cpd:C00007 |  | Oxygen; O2 | 114 | ec:1.14.12.10 ec:1.18.1.3 ec:1.13.11.1 ec:1.14.12.1 |
| cpd:C00080 |  | H+; Hydron | 110 | ec:1.14.12.10 ec:1.6.5.2 ec:4.2.1.1 ec:1.18.1.3 ec:1.2.1.18 ec:1.14.12.1 ec:4.2.1.104 ec:1.2.1.27 ec:1.3.1.25 ec:1.1.1.100 ec:1.1.1.1 |
| cpd:C00090 |  | Catechol; 1,2-Benzenediol; o-Benzenediol; 1,2-Dihydroxybenzene; Brenzcatechin; Pyrocatechol | 109 | ec:1.18.1.3 ec:1.13.11.1 ec:1.14.12.1 ec:1.3.1.25 |
| cpd:C00004 |  | NADH; DPNH; Reduced nicotinamide adenine dinucleotide | 103 | ec:1.14.12.10 ec:1.6.5.2 ec:1.18.1.3 ec:1.2.1.18 ec:1.14.12.1 ec:1.2.1.27 ec:1.3.1.25 ec:1.1.1.1 |
| cpd:C00003 |  | NAD+; NAD; Nicotinamide adenine dinucleotide; DPN; Diphosphopyridine nucleotide; Nadide | 103 | ec:1.14.12.10 ec:1.6.5.2 ec:1.18.1.3 ec:1.2.1.18 ec:1.14.12.1 ec:1.2.1.27 ec:1.3.1.25 ec:1.1.1.1 |
| cpd:C00006 |  | NADP+; NADP; Nicotinamide adenine dinucleotide phosphate; beta-Nicotinamide adenine dinucleotide phosphate; TPN; Triphosphopyridine nucleotide | 94 | ec:1.14.12.10 ec:1.18.1.3 ec:1.2.1.18 ec:1.14.12.1 ec:1.2.1.27 ec:1.1.1.100 |
| cpd:C00005 |  | NADPH; TPNH; Reduced nicotinamide adenine dinucleotide phosphate | 94 | ec:1.14.12.10 ec:1.18.1.3 ec:1.2.1.18 ec:1.14.12.1 ec:1.2.1.27 ec:1.1.1.100 |
| cpd:C00014 |  | Ammonia; NH3 | 85 | ec:3.5.1.4 ec:1.18.1.3 ec:4.3.1.12 ec:1.14.12.1 ec:4.2.1.104 ec:3.5.5.7 |
| cpd:C00108 |  | Anthranilate; Anthranilic acid; o-Aminobenzoic acid; Vitamin L1; 2-Aminobenzoate | 80 | ec:1.18.1.3 ec:1.14.12.1 |
| cpd:C01327 |  | Hydrochloric acid; HCl; Hydrogen chloride; Hydrochloride | 66 | ec:5.5.1.1 ec:1.18.1.3 |
| cpd:C00001 |  | H2O; Water | 53 | ec:3.1.1.24  ec:3.1.6.1 ec:3.5.1.4 ec:4.2.1.1 ec:3.11.1.1 ec:1.18.1.3 ec:4.2.1.9 ec:3.5.5.7 |
| cpd:C04112 |  | 3-Methyl-cis,cis-hexadienedioate; 3-Methyl-cis,cis-muconate | 52 | ec:5.5.1.1 ec:1.13.11.1 |
| cpd:C16474 |  | 3-Fluoro-cis,cis-muconate | 52 | ec:5.5.1.1 ec:1.13.11.1 |
| cpd:C03585 |  | 3-Chloro-cis,cis-muconate | 52 | ec:5.5.1.1 ec:1.13.11.1 |
| cpd:C14610 |  | (S)-5-Oxo-2,5-dihydrofuran-2-acetate; (+)-Muconolactone | 52 | ec:5.5.1.1 ec:5.3.3.4 |
| cpd:C02480 |  | cis,cis-Muconate; cis,cis-Hexadienedioate; cis,cis-2,4-Hexadienedioic acid | 52 | ec:5.5.1.1 ec:1.13.11.1 |
| cpd:C06727 |  | cis-1,2-Dihydro-3-ethylcatechol; cis-2,3-Dihydroxy-2,3-dihydroethylbenzene; cis-3-Ethyl-cyclohexa-3,5-diene-1,2-diol | 40 | ec:1.18.1.3 |
| cpd:C00058 |  | Formate; Methanoic acid; Formic acid | 40 | ec:1.18.1.3 |
| cpd:C04592 |  | Toluene-cis-dihydrodiol; (1S,2R)-3-Methylcyclohexa-3,5-diene-1,2-diol | 40 | ec:1.18.1.3 |
| cpd:C00048 |  | Glyoxylate; Glyoxalate; Glyoxylic acid | 40 | ec:1.18.1.3 |
| cpd:C11588 |  | cis-3-(Carboxy-ethyl)-3,5-cyclo-hexadiene-1,2-diol; cis-3-(2-Carboxy-ethyl)-3,5-cyclo-hexadiene-1,2-diol; 3-(cis-5,6-Dihydroxycyclohexa-1,3-dien-1-yl)propanoate | 40 | ec:1.18.1.3 |
| cpd:C01455 |  | Toluene; Methylbenzene; Toluol | 40 | ec:1.18.1.3 |
| cpd:C00423 |  | trans-Cinnamate; trans-Cinnamic acid; (E)-Cinnamate | 40 | ec:1.18.1.3 |
| cpd:C05629 |  | Phenylpropanoate; 3-Phenyl-propionic acid; 3-Phenylpropanoic acid; 3-Phenylpropionic acid | 40 | ec:1.18.1.3 |
| cpd:C07111 |  | Ethylbenzene; Phenylethane; Ethylbenzol; Ethylenzene | 40 | ec:1.18.1.3 |
| cpd:C12622 |  | cis-3-(3-Carboxyethenyl)-3,5-cyclohexadiene-1,2-diol; (2E)-3-(cis-5,6-Dihydroxycyclohexa-1,3-dien-1-yl)prop-2-enoate | 40 | ec:1.18.1.3 |
| cpd:C06790 |  | Trichloroethene; Trichloroethylene; TCE | 40 | ec:1.18.1.3 |
| cpd:C06589 |  | cis-2,3-Dihydro-2,3-dihydroxybiphenyl; cis-3-Phenylcyclohexa-3,5-diene-1,2-diol; (1S,2R)-3-Phenylcyclohexa-3,5-diene-1,2-diol | 40 | ec:1.18.1.3 |
| cpd:C06588 |  | Biphenyl; Phenylbenzene; 1,1'-Biphenyl; 1,1'-Diphenyl | 40 | ec:1.18.1.3 |
| cpd:C06585 |  | cis-2,3-Dihydro-2,3-dihydroxy-4'-chlorobiphenyl | 40 | ec:1.18.1.3 |
| cpd:C06584 |  | 4-Chlorobiphenyl; 1-Chloro-4-phenyl benzene; 4-Monochloro-biphenyl | 40 | ec:1.18.1.3 |
| cpd:C04091 |  | cis-1,2-Dihydrobenzene-1,2-diol; cis-Benzeneglycol; cis-Cyclohexa-3,5-diene-1,2-diol | 40 | ec:1.18.1.3 |
| cpd:C01407 |  | Benzene | 40 | ec:1.18.1.3 |
| cpd:C06579 |  | cis-2,3-Dihydroxy-2,3-dihydro-p-cumate; cis-5,6-Dihydroxy-4-isopropylcyclohexa-1,3-dienecarboxylate | 40 | ec:1.18.1.3 |
| cpd:C06578 |  | p-Cumate | 40 | ec:1.18.1.3 |
| cpd:C16473 |  | 4-Fluorocatechol | 29 | ec:1.13.11.1 ec:1.3.1.25 |
| cpd:C16472 |  | 3-Fluorocatechol | 29 | ec:1.13.11.1 ec:1.3.1.25 |
| cpd:C03586 |  | 2-Oxo-2,3-dihydrofuran-5-acetate; 3-Oxoadipate enol-lactone; 4,5-Dihydro-5-oxofuran-2-acetate; 5-Oxo-4,5-dihydrofuran-2-acetate | 29 | ec:3.1.1.24 ec:4.1.1.44 ec:5.3.3.4 |
| cpd:C06730 |  | 4-Methylcatechol; 3,4-Dihydroxytoluene; 1,2-Dihydroxy-4-methylbenzene; 4-Methyl-1,2-benzenediol | 29 | ec:1.13.11.1 ec:1.3.1.25 |
| cpd:C02375 |  | 4-Chlorocatechol | 26 | ec:1.13.11.1 |
| cpd:C16476 |  | 4-Fluoromuconolactone | 26 | ec:5.5.1.1 |
| cpd:C16475 |  | 2-Fluoro-cis,cis-muconate | 26 | ec:1.13.11.1 |
| cpd:C03572 |  | 2-Chloro-cis,cis-muconate | 26 | ec:1.13.11.1 |
| cpd:C07090 |  | Protoanemonin; 4-Methylenebut-2-en-4-olide; cis-4-Methylenebut-2-en-4-olide | 26 | ec:5.5.1.1 |
| cpd:C12833 |  | 2,3,5-Trichloro-cis,cis-muconate | 26 | ec:1.13.11.1 |
| cpd:C12831 |  | 3,4,6-Trichlorocatechol | 26 | ec:1.13.11.1 |
| cpd:C04559 |  | 4-Methylmuconolactone; 4-Carboxymethyl-4-methylbut-2-en-1,4-olide | 26 | ec:5.5.1.1 |
| cpd:C04558 |  | 3-Methylmuconolactone; 4-Carboxymethyl-3-methylbut-2-en-1,4-olide | 26 | ec:5.5.1.1 |
| cpd:C05618 |  | 3-Chlorocatechol | 26 | ec:1.13.11.1 |
| cpd:C18241 |  | Tetrachloro-cis,cis-muconate | 26 | ec:1.13.11.1 |
| cpd:C18240 |  | Tetrachlorocatechol; Tetrachloro-1,2-benzenediol | 26 | ec:1.13.11.1 |
| cpd:C00435 |  | Oxidized rubredoxin | 20 | ec:1.18.1.3 |
| cpd:C00340 |  | Reduced rubredoxin | 20 | ec:1.18.1.3 |
| cpd:C16480 |  | 4-Fluorocyclohexadiene-cis,cis-1,2-diol-1-carboxylate | 11 | ec:1.14.12.10 ec:1.3.1.25 |
| cpd:C16479 |  | 5-Fluorocyclohexadiene-cis,cis-1,2-diol-1-carboxylate | 11 | ec:1.14.12.10 ec:1.3.1.25 |
| cpd:C16478 |  | 3-Fluorocyclohexadiene-cis,cis-1,2-diol-1-carboxylate | 11 | ec:1.14.12.10 ec:1.3.1.25 |
| cpd:C06731 |  | 1,2-Dihydroxy-6-methylcyclohexa-3,5-dienecarboxylate | 11 | ec:1.14.12.10 ec:1.3.1.25 |
| cpd:C06729 |  | cis-1,2-Dihydroxy-4-methylcyclohexa-3,5-diene-1-carboxylate; 4-Methylcyclohexa-3,5-diene-1,2-cis-diol-1-carboxylic acid | 11 | ec:1.14.12.10 ec:1.3.1.25 |
| cpd:C06720 |  | 1,6-Dihydroxy-5-methylcyclohexa-2,4-dienecarboxylate; 1,2-Dihydroxy-3-methylcyclohexa-3,5-dienecarboxylate | 11 | ec:1.14.12.10 ec:1.3.1.25 |
| cpd:C06321 |  | (1R,6S)-1,6-Dihydroxycyclohexa-2,4-diene-1-carboxylate; cis-1,2-Dihydroxycyclohexa-3,5-diene-1-carboxylate; cis-1,6-Dihydroxy-2,4-cyclohexadiene-1-carboxylic acid | 11 | ec:1.14.12.10 ec:1.3.1.25 |
| cpd:C00180 |  | Benzoate; Benzoic acid; Benzenecarboxylic acid; Phenylformic acid; Dracylic acid | 10 | ec:1.14.12.10 ec:3.5.1.4 |
| cpd:C05850 |  | Reduced Vitamin K; Vitamin K hydroquinone | 9 | ec:1.6.5.2 |
| cpd:C00828 |  | Menaquinone; Vitamin K2 | 9 | ec:1.6.5.2 |
| cpd:C05819 |  | Menaquinol; Reduced menaquinone; Vitamin K2 hydroquinone; Reduced vitamin K2 | 9 | ec:1.6.5.2 |
| cpd:C01628 |  | Vitamin K | 9 | ec:1.6.5.2 |
| cpd:C02059 |  | Phylloquinone; Vitamin K1; Phytonadione; 2-Methyl-3-phytyl-1,4-naphthoquinone | 9 | ec:1.6.5.2 |
| cpd:C03313 |  | Phylloquinol; Vitamin K1 hydroquinone; Phytonadiol | 9 | ec:1.6.5.2 |
| cpd:C16482 |  | 2-Fluorocyclohexadiene-cis,cis-1,2-diol-1-carboxylate | 8 | ec:1.14.12.10 |
| cpd:C16481 |  | 6-Fluorocyclohexadiene-cis,cis-1,2-diol-1-carboxylate | 8 | ec:1.14.12.10 |
| cpd:C02371 |  | 4-Fluorobenzoate; 4-Fluorobenzoic acid | 8 | ec:1.14.12.10 |
| cpd:C07215 |  | o-Toluate; o-Methylbenzoate; o-Toluic Acid; 2-Methylbenzoic acid | 8 | ec:1.14.12.10 |
| cpd:C02364 |  | 3-Fluorobenzoate; 3-Fluorobenzoic acid | 8 | ec:1.14.12.10 |
| cpd:C07211 |  | m-Methylbenzoate; m-Toluic Acid; beta-Bethylbenzoic acid; m-Toluylic acid | 8 | ec:1.14.12.10 |
| cpd:C02359 |  | 2-Fluorobenzoate; 2-Fluorobenzoic acid | 8 | ec:1.14.12.10 |
| cpd:C01454 |  | Toluate; p-Toluate; p-Toluic acid; 4-Methylbenzoic acid; Toluenecarboxylic acid; Crithminic acid | 8 | ec:1.14.12.10 |
| cpd:C00022 |  | Pyruvate; Pyruvic acid; 2-Oxopropanoate; 2-Oxopropanoic acid; Pyroracemic acid | 6 | ec:2.2.1.6 ec:4.1.2.52 ec:2.6.1.37 |
| cpd:C20377 |  | 3-Hydroxypimeloyl-[acp] methyl ester; 3-Hydroxypimeloyl-[acyl-carrier protein] methyl ester | 5 | ec:1.1.1.100 |
| cpd:C20376 |  | 3-Ketopimeloyl-[acp] methyl ester; 3-Ketopimeloyl-[acyl-carrier protein] methyl ester | 5 | ec:1.1.1.100 |
| cpd:C20373 |  | 3-Hydroxyglutaryl-[acp] methyl ester; 3-Hydroxyglutaryl-[acyl-carrier protein] methyl ester | 5 | ec:1.1.1.100 |
| cpd:C20372 |  | 3-Ketoglutaryl-[acp] methyl ester; 3-Ketoglutaryl-[acyl-carrier protein] methyl ester | 5 | ec:1.1.1.100 |
| cpd:C04633 |  | (3R)-3-Hydroxypalmitoyl-[acyl-carrier protein]; (R)-3-Hydroxypalmitoyl-[acyl-carrier protein]; (3R)-3-Hydroxyhexadecanoyl-[acyl-carrier protein]; (R)-3-Hydroxyhexadecanoyl-[acyl-carrier protein] | 5 | ec:1.1.1.100 |
| cpd:C04620 |  | (3R)-3-Hydroxyoctanoyl-[acyl-carrier protein]; (R)-3-Hydroxyoctanoyl-[acyl-carrier protein] | 5 | ec:1.1.1.100 |
| cpd:C04619 |  | (3R)-3-Hydroxydecanoyl-[acyl-carrier protein]; (R)-3-Hydroxydecanoyl-[acyl-carrier protein] | 5 | ec:1.1.1.100 |
| cpd:C04618 |  | (3R)-3-Hydroxybutanoyl-[acyl-carrier protein]; (R)-3-Hydroxybutanoyl-[acyl-carrier protein] | 5 | ec:1.1.1.100 |
| cpd:C16220 |  | 3-Hydroxyoctadecanoyl-[acp]; 3-Hydroxystearoyl-[acp] | 5 | ec:1.1.1.100 |
| cpd:C16219 |  | 3-Oxostearoyl-[acp]; 3-Oxooctadecanoyl-[acp]; beta-Ketostearoyl-[acp]; 3-Ketostearoyl-[acp] | 5 | ec:1.1.1.100 |
| cpd:C16217 |  | 3-Hydroxyoctadecanoyl-CoA; 3-Hydroxystearoyl-CoA; beta-Hydroxystearoyl-CoA | 5 | ec:1.1.1.100 |
| cpd:C16216 |  | 3-Oxostearoyl-CoA; 3-Oxooctadecanoyl-CoA; beta-Ketostearoyl-CoA; 3-Ketostearoyl-CoA | 5 | ec:1.1.1.100 |
| cpd:C05762 |  | 3-Oxohexadecanoyl-[acp]; 3-Oxohexadecanoyl-[acyl-carrier protein] | 5 | ec:1.1.1.100 |
| cpd:C05759 |  | 3-Oxotetradecanoyl-[acp]; 3-Oxotetradecanoyl-[acyl-carrier protein] | 5 | ec:1.1.1.100 |
| cpd:C05757 |  | (R)-3-Hydroxydodecanoyl-[acp]; (R)-3-Hydroxydodecanoyl-[acyl-carrier protein]; D-3-Hydroxydodecanoyl-[acp]; D-3-Hydroxydodecanoyl-[acyl-carrier protein] | 5 | ec:1.1.1.100 |
| cpd:C05756 |  | 3-Oxododecanoyl-[acp]; 3-Oxododecanoyl-[acyl-carrier protein] | 5 | ec:1.1.1.100 |
| cpd:C05753 |  | 3-Oxodecanoyl-[acp]; 3-Oxodecanoyl-[acyl-carrier protein] | 5 | ec:1.1.1.100 |
| cpd:C05750 |  | 3-Oxooctanoyl-[acp]; 3-Oxooctanoyl-[acyl-carrier protein] | 5 | ec:1.1.1.100 |
| cpd:C04688 |  | (3R)-3-Hydroxytetradecanoyl-[acyl-carrier protein]; (R)-3-Hydroxytetradecanoyl-[acyl-carrier protein]; beta-Hydroxymyristyl-[acyl-carrier protein]; HMA | 5 | ec:1.1.1.100 |
| cpd:C05747 |  | (R)-3-Hydroxyhexanoyl-[acp]; (R)-3-Hydroxyhexanoyl-[acyl-carrier protein]; D-3-Hydroxyhexanoyl-[acp]; D-3-Hydroxyhexanoyl-[acyl-carrier protein] | 5 | ec:1.1.1.100 |
| cpd:C05746 |  | 3-Oxohexanoyl-[acp]; 3-Oxohexanoyl-[acyl-carrier protein] | 5 | ec:1.1.1.100 |
| cpd:C05744 |  | Acetoacetyl-[acp]; Acetoacetyl-[acyl-carrier protein] | 5 | ec:1.1.1.100 |
| cpd:C06010 |  | (S)-2-Acetolactate; (S)-2-Hydroxy-2-methyl-3-oxobutanoate | 4 | ec:2.2.1.6 |
| cpd:C06006 |  | (S)-2-Aceto-2-hydroxybutanoate; (S)-2-Hydroxy-2-ethyl-3-oxobutanoate | 4 | ec:2.2.1.6 |
| cpd:C05125 |  | 2-(alpha-Hydroxyethyl)thiamine diphosphate; 2-Hydroxyethyl-ThPP | 4 | ec:2.2.1.6 |
| cpd:C00900 |  | 2-Acetolactate | 4 | ec:2.2.1.6 |
| cpd:C00109 |  | 2-Oxobutanoate; 2-Ketobutyric acid; 2-Oxobutyric acid; 2-Oxobutyrate; 2-Oxobutanoic acid; alpha-Ketobutyric acid; alpha-Ketobutyrate | 4 | ec:2.2.1.6 |
| cpd:C00068 |  | Thiamin diphosphate; Thiamine diphosphate; Thiamin pyrophosphate; TPP; ThPP | 4 | ec:2.2.1.6 |
| cpd:C00002 |  | ATP; Adenosine 5'-triphosphate | 4 | ec:2.7.1.6 ec:6.3.4.5 ec:6.1.1.2 ec:6.1.1.16 |
| cpd:C00084 |  | Acetaldehyde; Ethanal | 3 | ec:3.11.1.1 ec:1.1.1.1 |
| cpd:C00511 |  | Acrylic acid; Propenoate; Acrylate; 2-Propenoic acid; Vinylformic acid | 3 | ec:3.5.1.4 ec:3.5.5.7 |
| cpd:C03167 |  | Phosphonoacetaldehyde; 2-Phosphonoacetaldehyde; 2-Oxoethylphosphonate | 3 | ec:3.11.1.1 ec:2.6.1.37 |
| cpd:C02923 |  | 2,3-Dihydroxytoluene; 3-Methylcatechol | 3 | ec:1.3.1.25 |
| cpd:C00020 |  | AMP; Adenosine 5'-monophosphate; Adenylic acid; Adenylate; 5'-AMP; 5'-Adenylic acid; 5'-Adenosine monophosphate; Adenosine 5'-phosphate | 3 | ec:6.3.4.5 ec:6.1.1.2 ec:6.1.1.16 |
| cpd:C00013 |  | Diphosphate; Diphosphoric acid; Pyrophosphate; Pyrophosphoric acid; PPi | 3 | ec:6.3.4.5 ec:6.1.1.2 ec:6.1.1.16 |
| cpd:C04272 |  | (R)-2,3-Dihydroxy-3-methylbutanoate; (R)-2,3-Dihydroxy-isovalerate; (R)-2,3-Dihydroxy-isovaleric acid; (2R)-2,3-Dihydroxy-3-methylbutanoate | 2 | ec:4.2.1.9 |
| cpd:C06007 |  | (R)-2,3-Dihydroxy-3-methylpentanoate; (R)-2,3-Dihydroxy-3-methylvalerate; (2R,3R)-2,3-Dihydroxy-3-methylpentanoate | 2 | ec:4.2.1.9 |
| cpd:C00288 |  | HCO3-; Bicarbonate; Hydrogencarbonate; Acid carbonate | 2 | ec:4.2.1.1 ec:4.2.1.104 |
| cpd:C00671 |  | (S)-3-Methyl-2-oxopentanoic acid; (S)-3-Methyl-2-oxopentanoate; (3S)-3-Methyl-2-oxopentanoic acid; (3S)-3-Methyl-2-oxopentanoate | 2 | ec:4.2.1.9 |
| cpd:C03620 |  | Monocarboxylic acid amide | 2 | ec:3.5.1.4 |
| cpd:C00468 |  | Estrone; 3-Hydroxy-1,3,5(10)-estratrien-17-one | 2 | ec:3.1.6.1 |
| cpd:C00060 |  | Carboxylate; R-COOH; Monocarboxylate; Carboxylic acid | 2 | ec:3.5.1.4 |
| cpd:C00059 |  | Sulfate; Sulfuric acid | 2 | ec:3.1.6.1 |
| cpd:C00846 |  | 3-Oxoadipate; 3-Oxoadipic acid; 3-Keto-adipate | 2 | ec:3.1.1.24 |
| cpd:C04039 |  | 2,3-Dihydroxy-3-methylbutanoate; 2,3-Dihydroxy-isovalerate; 2,3-Dihydroxy-isovaleric acid | 2 | ec:4.2.1.9 |
| cpd:C06125 |  | Sulfatide; Galactosylceramidesulfate; Cerebroside 3-sulfate | 2 | ec:3.1.6.1 |
| cpd:C00446 |  | alpha-D-Galactose 1-phosphate; alpha-D-Galactopyranose 1-phosphate | 2 | ec:2.7.1.6 ec:2.7.7.12 |
| cpd:C02538 |  | Estrone 3-sulfate | 2 | ec:3.1.6.1 |
| cpd:C02693 |  | (Indol-3-yl)acetamide; Indole-3-acetamide | 2 | ec:3.5.1.4 |
| cpd:C02686 |  | Galactosylceramide; Galactocerebroside; D-Galactosyl-N-acylsphingosine; Cerebroside; D-Galactosylceramide | 2 | ec:3.1.6.1 |
| cpd:C01659 |  | Acrylamide; 2-Propenamide | 2 | ec:3.5.1.4 |
| cpd:C09815 |  | Benzamide | 2 | ec:3.5.1.4 |
| cpd:C09813 |  | 2-Ketocyclohexane-1-carboxyl-CoA; 2-Oxocyclohexane-1-carbonyl-CoA | 2 |  |
| cpd:C00029 |  | UDP-glucose; UDPglucose; UDP-D-glucose; Uridine diphosphate glucose; UDP-alpha-D-glucose | 2 | ec:2.7.7.12 ec:2.4.1.80 |
| cpd:C07086 |  | Phenylacetic acid; Benzylformic acid; Phenylacetate; Benzeneacetic acid | 2 | ec:3.5.1.4 |
| cpd:C02505 |  | 2-Phenylacetamide; alpha-Phenylacetamide | 2 | ec:3.5.1.4 |
| cpd:C01035 |  | 4-Guanidinobutanoate; 4-Guanidinobutyric acid | 2 | ec:3.5.1.4 |
| cpd:C00009 |  | Orthophosphate; Phosphate; Phosphoric acid; Orthophosphoric acid | 2 | ec:3.11.1.1 |
| cpd:C00954 |  | Indole-3-acetate; Indole-3-acetic acid; (Indol-3-yl)acetate; Indoleacetate; Indoleacetic acid; IAA | 2 | ec:3.5.1.4 |
| cpd:C03078 |  | 4-Guanidinobutanamide | 2 | ec:3.5.1.4 |
| cpd:C00141 |  | 3-Methyl-2-oxobutanoic acid; 3-Methyl-2-oxobutyric acid; 3-Methyl-2-oxobutanoate; 2-Oxo-3-methylbutanoate; 2-Oxoisovalerate; 2-Oxoisopentanoate; alpha-Ketovaline; 2-Ketovaline; 2-Keto-3-methylbutyric acid | 2 | ec:4.2.1.9 |
| cpd:C00097 |  | L-Cysteine; L-2-Amino-3-mercaptopropionic acid | 1 | ec:6.1.1.16 |
| cpd:C01353 |  | Carbonic acid; Dihydrogen carbonate; H2CO3 | 1 | ec:4.2.1.1 |
| cpd:C06201 |  | 2,4-Dihydroxyhept-2-enedioate; 2,4-Dihydroxyhept-2-1,7-dioate; 2,4-Dihydroxyhept-2-enedioic acid | 1 | ec:4.1.2.52 |
| cpd:C01352 |  | FADH2 | 1 | ec:1.3.99.1 |
| cpd:C00327 |  | L-Citrulline; 2-Amino-5-ureidovaleric acid; Citrulline | 1 | ec:6.3.4.5 |
| cpd:C06002 |  | (S)-Methylmalonate semialdehyde | 1 | ec:1.2.1.18 ec:1.2.1.27 |
| cpd:C00124 |  | D-Galactose; D-Galactopyranose | 1 | ec:2.7.1.6 |
| cpd:C00122 |  | Fumarate; Fumaric acid; trans-Butenedioic acid | 1 | ec:1.3.99.1 |
| cpd:C00078 |  | L-Tryptophan; Tryptophan; (S)-alpha-Amino-beta-(3-indolyl)-propionic acid | 1 | ec:6.1.1.2 |
| cpd:C00077 |  | L-Ornithine; (S)-2,5-Diaminovaleric acid; (S)-2,5-Diaminopentanoic acid; (S)-2,5-Diaminopentanoate | 1 | ec:4.3.1.12 |
| cpd:C00071 |  | Aldehyde; RCHO | 1 | ec:1.1.1.1 |
| cpd:C00469 |  | Ethanol; Ethyl alcohol; Methylcarbinol | 1 | ec:1.1.1.1 |
| cpd:C00103 |  | D-Glucose 1-phosphate; alpha-D-Glucose 1-phosphate; Cori ester; D-Glucose alpha-1-phosphate | 1 | ec:2.7.7.12 |
| cpd:C00100 |  | Propanoyl-CoA; Propionyl-CoA; Propionyl coenzyme A | 1 | ec:1.2.1.18 ec:1.2.1.27 |
| cpd:C01083 |  | alpha,alpha-Trehalose; alpha,alpha'-Trehalose; Trehalose | 1 |  |
| cpd:C00052 |  | UDP-alpha-D-galactose; UDP-D-galactose; UDP-galactose; UDP-D-galactopyranose; UDP-alpha-D-galactopyranose | 1 | ec:2.7.7.12 |
| cpd:C03406 |  | N-(L-Arginino)succinate; 2-(Nomega-L-Arginino)succinate; L-Argininosuccinate; L-Argininosuccinic acid; L-Arginosuccinic acid | 1 | ec:6.3.4.5 |
| cpd:C01278 |  | 2-Carboxy-2,5-dihydro-5-oxofuran-2-acetate; 5-Carboxy-2,5-dihydro-2-oxofuran-5-acetate; 4-Carboxymuconolactone; gamma-Carboxymuconolactone | 1 | ec:4.1.1.44 |
| cpd:C00049 |  | L-Aspartate; L-Aspartic acid; 2-Aminosuccinic acid; L-Asp | 1 | ec:6.3.4.5 |
| cpd:C00042 |  | Succinate; Succinic acid; Butanedionic acid; Ethylenesuccinic acid | 1 | ec:1.3.99.1 |
| cpd:C00041 |  | L-Alanine; L-2-Aminopropionic acid; L-alpha-Alanine | 1 | ec:2.6.1.37 |
| cpd:C03557 |  | 2-Aminoethylphosphonate; (2-Aminoethyl)phosphonate; Ciliatine | 1 | ec:2.6.1.37 |
| cpd:C01063 |  | 6-Carboxyhexanoyl-CoA; Pimeloyl-CoA | 1 |  |
| cpd:C00232 |  | Succinate semialdehyde; Succinic semialdehyde; 4-Oxobutanoate | 1 | ec:4.1.2.52 |
| cpd:C00195 |  | N-Acylsphingosine; Ceramide | 1 | ec:2.4.1.80 |
| cpd:C01652 |  | tRNA(Trp) | 1 | ec:6.1.1.2 |
| cpd:C00030 |  | Reduced acceptor; AH2; Hydrogen-donor; Donor | 1 | ec:1.3.99.1 |
| cpd:C09812 |  | 2-Hydroxycyclohexane-1-carboxyl-CoA | 1 |  |
| cpd:C00226 |  | Primary alcohol; 1-Alcohol | 1 | ec:1.1.1.1 |
| cpd:C00222 |  | 3-Oxopropanoate; Malonate semialdehyde | 1 | ec:1.2.1.18 ec:1.2.1.27 |
| cpd:C00028 |  | Acceptor; Hydrogen-acceptor; A; Oxidized donor | 1 | ec:1.3.99.1 |
| cpd:C00024 |  | Acetyl-CoA; Acetyl coenzyme A | 1 | ec:1.2.1.18 ec:1.2.1.27 |
| cpd:C02909 |  | (2-Naphthyl)methanol; 2-Naphthalenemethanol; 2-Hydroxymethylnaphthalene | 1 | ec:1.1.1.1 |
| cpd:C14099 |  | 2-Naphthaldehyde; 2-Naphthalenecarboxaldehyde | 1 | ec:1.1.1.1 |
| cpd:C14090 |  | 1-Naphthaldehyde; 1-Formylnaphthalene | 1 | ec:1.1.1.1 |
| cpd:C01998 |  | Acrylonitrile; Propenenitrile; Vinyl cyanide | 1 | ec:3.5.5.7 |
| cpd:C01639 |  | tRNA(Cys) | 1 | ec:6.1.1.16 |
| cpd:C00016 |  | FAD; Flavin adenine dinucleotide | 1 | ec:1.3.99.1 |
| cpd:C00015 |  | UDP; Uridine 5'-diphosphate | 1 | ec:2.4.1.80 |
| cpd:C14089 |  | 1-Hydroxymethylnaphthalene; 1-Naphthalenemethanol | 1 | ec:1.1.1.1 |
| cpd:C00010 |  | CoA; Coenzyme A; CoA-SH | 1 | ec:1.2.1.18 ec:1.2.1.27 |
| cpd:C05577 |  | 3,4-Dihydroxymandelaldehyde; 3,4-Dihydroxyphenylglycolaldehyde | 1 | ec:1.1.1.1 |
| cpd:C05576 |  | 3,4-Dihydroxyphenylethyleneglycol | 1 | ec:1.1.1.1 |
| cpd:C00369 |  | Starch | 1 |  |
| cpd:C03125 |  | L-Cysteinyl-tRNA(Cys) | 1 | ec:6.1.1.16 |
| cpd:C00008 |  | ADP; Adenosine 5'-diphosphate | 1 | ec:2.7.1.6 |
| cpd:C01190 |  | beta-D-Glucosyl-(1<->1)-ceramide; Glucosylceramide; Glucocerebroside; D-Glucosyl-N-acylsphingosine | 1 | ec:2.4.1.80 |
| cpd:C03512 |  | L-Tryptophanyl-tRNA(Trp) | 1 | ec:6.1.1.2 |
| cpd:C05601 |  | 4-Hydroxy-2-oxo-heptanedioate; 4-Hydroxy-2-ketopimelate; 4-Hydroxy-2-oxoheptanedioic acid; 4-Hydroxy-2-oxoheptanedioate | 1 | ec:4.1.2.52 |
| cpd:C01417 |  | Cyanate; Cyanic acid | 1 | ec:4.2.1.104 |
| cpd:C01213 |  | (R)-Methylmalonyl-CoA; L-Methylmalonyl-CoA | 1 | ec:1.2.1.18 ec:1.2.1.27 |
| cpd:C16348 |  | cis-3-Chloroallyl aldehyde; cis-3-Chloro-2-propenal | 1 | ec:1.1.1.1 |
| cpd:C00148 |  | L-Proline; 2-Pyrrolidinecarboxylic acid | 1 | ec:4.3.1.12 |
| cpd:C06613 |  | trans-3-Chloroallyl aldehyde; trans-3-Chloro-2-propenal | 1 | ec:1.1.1.1 |
| cpd:C06612 |  | cis-3-Chloro-2-propene-1-ol; cis-3-Chloroallyl alcohol | 1 | ec:1.1.1.1 |
| cpd:C06611 |  | trans-3-Chloro-2-propene-1-ol; trans-3-Chloroallyl alcohol | 1 | ec:1.1.1.1 |
| cpd:C01563 |  | Carbamate; Carbamic acid; Aminoformic acid | 1 | ec:4.2.1.104 |

  
**Over-represented Pathway Summary**: Collection of the KEGG metabolic pathways containing the proteins identified in the "Over-represented Metabolite Summary" ranked by the highest number of hits per pathway  

| Pathway ID | EC | EC Frequency | Name |
| --- | --- | --- | --- |
| map00362 | ec:1.3.1.25 ec:1.13.11.1 ec:3.1.1.24 ec:5.3.3.4 ec:5.5.1.1 ec:4.1.1.44 ec:1.14.12.10 | 90 | path:map00362 Benzoate degradation |
| map00364 | ec:1.3.1.25 ec:1.13.11.1 ec:5.5.1.1 ec:1.14.12.10 | 61 | path:map00364 Fluorobenzoate degradation |
| map00623 | ec:1.13.11.1 ec:5.5.1.1 ec:1.3.99.1 | 53 | path:map00623 Toluene degradation |
| map00361 | ec:1.13.11.1 ec:5.5.1.1 | 52 | path:map00361 Chlorocyclohexane and chlorobenzene degradation |
| map00627 | ec:1.14.13.82 ec:3.5.1.4 ec:1.14.12.1 | 44 | path:map00627 Aminobenzoate degradation |
| map00071 | ec:1.1.1.1 ec:1.18.1.3 | 21 | path:map00071 Fatty acid degradation |
| map00622 | ec:1.18.1.3 | 20 | path:map00622 Xylene degradation |
| map00130 | ec:1.6.5.2 | 9 | path:map00130 Ubiquinone and other terpenoid-quinone biosynthesis |
| map00770 | ec:2.2.1.6 ec:4.2.1.9 | 6 | path:map00770 Pantothenate and CoA biosynthesis |
| map00290 | ec:2.2.1.6 ec:4.2.1.9 | 6 | path:map00290 Valine, leucine and isoleucine biosynthesis |
| map00061 | ec:1.1.1.100 | 5 | path:map00061 Fatty acid biosynthesis |
| map00780 | ec:1.1.1.100 | 5 | path:map00780 Biotin metabolism |
| map00650 | ec:2.2.1.6 ec:1.3.99.1 | 5 | path:map00650 Butanoate metabolism |
| map00660 | ec:2.2.1.6 | 4 | path:map00660 C5-Branched dibasic acid metabolism |
| map00330 | ec:4.3.1.12 ec:3.5.1.4 ec:6.3.4.5 | 4 | path:map00330 Arginine and proline metabolism |
| map00643 | ec:3.5.1.4 ec:3.5.5.7 | 3 | path:map00643 Styrene degradation |
| map00440 | ec:3.11.1.1 ec:2.6.1.37 | 3 | path:map00440 Phosphonate and phosphinate metabolism |
| map00600 | ec:2.4.1.80 ec:3.1.6.1 | 3 | path:map00600 Sphingolipid metabolism |
| map00640 | ec:1.2.1.27 ec:1.2.1.18 | 2 | path:map00640 Propanoate metabolism |
| map00350 | ec:1.1.1.1 ec:4.1.2.52 | 2 | path:map00350 Tyrosine metabolism |
| map00380 | ec:3.5.1.4 | 2 | path:map00380 Tryptophan metabolism |
| map00052 | ec:2.7.7.12 ec:2.7.1.6 | 2 | path:map00052 Galactose metabolism |
| map00140 | ec:3.1.6.1 | 2 | path:map00140 Steroid hormone biosynthesis |
| map00910 | ec:4.2.1.104 ec:4.2.1.1 | 2 | path:map00910 Nitrogen metabolism |
| map00970 | ec:6.1.1.2 ec:6.1.1.16 | 2 | path:map00970 Aminoacyl-tRNA biosynthesis |
| map00360 | ec:3.5.1.4 | 2 | path:map00360 Phenylalanine metabolism |
| map00520 | ec:2.7.7.12 ec:2.7.1.6 | 2 | path:map00520 Amino sugar and nucleotide sugar metabolism |
| map00190 | ec:1.3.99.1 | 1 | path:map00190 Oxidative phosphorylation |
| map00830 | ec:1.1.1.1 | 1 | path:map00830 Retinol metabolism |
| map00250 | ec:6.3.4.5 | 1 | path:map00250 Alanine, aspartate and glutamate metabolism |
| map00280 | ec:1.2.1.27 | 1 | path:map00280 Valine, leucine and isoleucine degradation |
| map00410 | ec:1.2.1.18 | 1 | path:map00410 beta-Alanine metabolism |
| map00982 | ec:1.1.1.1 | 1 | path:map00982 Drug metabolism - cytochrome P450 |
| map00980 | ec:1.1.1.1 | 1 | path:map00980 Metabolism of xenobiotics by cytochrome P450 |
| map00020 | ec:1.3.99.1 | 1 | path:map00020 Citrate cycle (TCA cycle) |
| map00562 | ec:1.2.1.18 | 1 | path:map00562 Inositol phosphate metabolism |
| map00720 | ec:1.3.99.1 | 1 | path:map00720 Carbon fixation pathways in prokaryotes |
| map00626 | ec:1.1.1.1 | 1 | path:map00626 Naphthalene degradation |
| map00625 | ec:1.1.1.1 | 1 | path:map00625 Chloroalkane and chloroalkene degradation |
| map00010 | ec:1.1.1.1 | 1 | path:map00010 Glycolysis / Gluconeogenesis |
| map00260 | ec:1.1.1.1 | 1 | path:map00260 Glycine, serine and threonine metabolism |

  
Analysis performed on 2014/02/14 20:45:51
