# Supplementary material for: Automated genomic context analysis and experimental validation platform for discovery of prokaryote transcriptional regulator functions
Source: BMC Genomics. 2014 Dec 18;15(1):1142. doi: 10.1186/1471-2164-15-1142 (PMC4349456; doi:10.1186/1471-2164-15-1142)
Supplement: Supplementary file 4 — Additional file 4: Result CymR. Function Discovery V1.0 output (.html format) for the p-cumate catabolism regulator (CymR, Bxe_ A3550). For detailed instructions on how to analyze the results please refer to the Function Discovery V1.0, a gene neighborhood analysis tool section in the Results part of the main text. (HTML 85 KB) [file 12864_2014_6995_MOESM4_ESM.html]

```
ENTRY       Bxe_A3550         CDS       T00340
DEFINITION  TetR family transcriptional regulator
ORGANISM    bxe  Burkholderia xenovorans
POSITION    1:1011238..1011867
MOTIF       Pfam: TetR_N Plectin
DBLINKS     NCBI-GI: 91782284
            NCBI-GeneID: 4004907
            JGI: BxeA3550
            UniProt: Q143Q1
AASEQ       209
            MIKKVPERRRTQAERREETRSRILDAAVSELMNKGYAGFRVDQVAATARVSRGAQTHHFP
            TKESLVLAALQRLYQASTEASMTLIEGLESGDDVLDALMRDSASFYLGPNFIIAVSMLNL
            GDHEPGLRQKVRAISRKHRLPVEKAWLQALLDSGLAEEPAKTVLNITQSIYRGMVMRKFL
            RNDPEYTRFTTEQWSKIARAYMNLNAPSR
NTSEQ       630
            atgataaaaaaagtgccagaacgacgtcgcacccaggcggaaagacgcgaggaaacgcgt
            tcgcggatattggacgcagccgtcagcgagttgatgaacaagggctacgccgggttccgg
            gtcgaccaggttgcagccactgccagggtatcgcgcggcgcccagacccatcatttcccg
            accaaggaatcgctcgttctggcggcgcttcagcggctttaccaggcgtcgaccgaggcc
            agcatgacgctcatcgagggcctggagtcgggcgacgatgtgctggatgcgctgatgcgg
            gactcggccagtttctacctgggaccgaatttcatcattgcggtgtcgatgctcaatctt
            ggcgatcacgaacccgggctgaggcagaaggttcgggcgatctcgcgcaagcaccggttg
            ccggtcgagaaggcctggctgcaggcgctgttggactctggcctggccgaagagccggca
            aaaacggtgctgaacatcacccagagcatctaccgcgggatggtgatgcgcaagttcttg
            cgcaacgatcccgagtacacacgctttaccacggagcagtggtcgaagattgcccgtgca
            tacatgaacctgaacgctccgtcacggtag
///
```

  
**Homolog ID**: Table of closest homologs  

```
                 Homologs                                       len   identity overlap
---------------------------------------------------------------------------------
ppf:Pput_2892 TetR family transcriptional regulator           207     0.707    208 
rsp:RSP_2801 transcriptional regulator, TetR family           200     0.438    73  
rsh:Rsph17029_1498 TetR family transcriptional regulato       189     0.518    56  
rsk:RSKD131_1166 TetR family transcriptional regulator        189     0.518    56
```

**Neighborhood Representations**: Table of genes in the defined genetic neighborhoods of the entry protein and its closest homologs  
  
**Neighborhood Representations for "bxe:Bxe\_A3550"**  

| ID | Annotation | EC number |
| --- | --- | --- |
| bxe:Bxe\_A3560 | hypothetical protein |  |
| bxe:Bxe\_A3559 | p-cymene monooxygenase, reductase subunit(CymAb) (EC:1.17.1.-); K15758 xylene monooxygenase electron transfer component [EC:1.18.1.3] | ec:1.18.1.3 |
| bxe:Bxe\_A3558 | Outer membrane protein (CymD); K06076 long-chain fatty acid transport protein |  |
| bxe:Bxe\_A3557 | p-cumate dioxygenase ferredoxin reductase subunit (CmtAa); K00529 ferredoxin--NAD+ reductase [EC:1.18.1.3] | ec:1.18.1.3 |
| bxe:Bxe\_A3556 | p-cumate dioxygenase large subunit (CmtAb); K10619 p-cumate 2,3-dioxygenase subunit alpha [EC:1.14.12.-] |  |
| bxe:Bxe\_A3555 | p-cumate dioxygenase small subunit (CmtAc); K16303 p-cumate 2,3-dioxygenase subunit beta [EC:1.14.12.-] |  |
| bxe:Bxe\_A3554 | 2,3-dihydroxy-p-cumate-3,4-dioxygenase (CmtC); K10621 2,3-dihydroxy-p-cumate/2,3-dihydroxybenzoate 3,4-dioxygenase [EC:1.13.11.- 1.13.11.14] | ec:1.13.11.14 |
| bxe:Bxe\_A3553 | 2,3-dihydroxy-2,3-dihydrophenylpropionate dehydrogenase (EC:1.1.1.100); K10620 2,3-dihydroxy-2,3-dihydro-p-cumate dehydrogenase [EC:1.3.1.58] | ec:1.3.1.58 |
| bxe:Bxe\_A3552 | p-cumate dioxygenase ferredoxin subunit (CmtAd) (EC:1.-.-.-); K05710 dioxygenase ferredoxin subunit |  |
| bxe:Bxe\_A3551 | HCOMODA decarboxylase (CmtD); K10622 HCOMODA/2-hydroxy-3-carboxy-muconic semialdehyde decarboxylase [EC:4.1.1.-] |  |
| bxe:Bxe\_A3550 | TetR family transcriptional regulator |  |
| bxe:Bxe\_A3549 | HOMODA hydrolase (CmtE) (EC:3.7.1.-); K10623 HOMODA hydrolase [EC:3.7.1.-] |  |
| bxe:Bxe\_A3548 | 2-hydroxypenta-2,4-dienoate hydratase (EC:4.2.1.-); K02554 2-keto-4-pentenoate hydratase [EC:4.2.1.80] | ec:4.2.1.80 |
| bxe:Bxe\_A3547 | acetaldehyde dehydrogenase (EC:1.2.1.10); K04073 acetaldehyde dehydrogenase [EC:1.2.1.10] | ec:1.2.1.10 |
| bxe:Bxe\_A3546 | 4-hydroxy-2-ketovalerate aldolase (EC:4.1.3.-); K01666 4-hydroxy 2-oxovalerate aldolase [EC:4.1.3.39] | ec:4.1.3.39 |
| bxe:Bxe\_A3545 | hypothetical protein |  |
| bxe:Bxe\_A3544 | pseudogene |  |
| bxe:Bxe\_A3543 | LysR family transcriptional regulator |  |
| bxe:Bxe\_A3542 | vanillate O-demethylase oxygenase (EC:1.14.13.82); K03862 vanillate monooxygenase [EC:1.14.13.82] | ec:1.14.13.82 |
| bxe:Bxe\_A3541 | hypothetical protein |  |
| bxe:Bxe\_A3540 | hypothetical protein |  |

  
**Neighborhood Representations for "ppf:Pput\_2892"**  

| ID | Annotation | EC number |
| --- | --- | --- |
| ppf:Pput\_2882 | alpha/beta hydrolase fold family protein; K10702 2-hydroxy-6-oxohepta-2,4-dienoate hydroxylase [EC:3.7.1.-] |  |
| ppf:Pput\_2883 | aromatic hydrocarbon degradation membrane protein |  |
| ppf:Pput\_2884 | LysR family transcriptional regulator |  |
| ppf:Pput\_2885 | hypothetical protein |  |
| ppf:Pput\_2886 | pseudogene |  |
| ppf:Pput\_2887 | enoyl-CoA hydratase/isomerase |  |
| ppf:Pput\_2888 | 4-hydroxy-2-ketovalerate aldolase; K01666 4-hydroxy 2-oxovalerate aldolase [EC:4.1.3.39] | ec:4.1.3.39 |
| ppf:Pput\_2889 | acetaldehyde dehydrogenase; K04073 acetaldehyde dehydrogenase [EC:1.2.1.10] | ec:1.2.1.10 |
| ppf:Pput\_2890 | 4-oxalocrotonate decarboxylase; K02554 2-keto-4-pentenoate hydratase [EC:4.2.1.80] | ec:4.2.1.80 |
| ppf:Pput\_2891 | alpha/beta hydrolase fold family protein; K10623 HOMODA hydrolase [EC:3.7.1.-] |  |
| ppf:Pput\_2892 | TetR family transcriptional regulator |  |
| ppf:Pput\_2893 | class II aldolase/adducin family protein; K10622 HCOMODA/2-hydroxy-3-carboxy-muconic semialdehyde decarboxylase [EC:4.1.1.-] |  |
| ppf:Pput\_2894 | Rieske (2Fe-2S) domain-containing protein; K05710 dioxygenase ferredoxin subunit |  |
| ppf:Pput\_2895 | short-chain dehydrogenase/reductase SDR; K10620 2,3-dihydroxy-2,3-dihydro-p-cumate dehydrogenase [EC:1.3.1.58] | ec:1.3.1.58 |
| ppf:Pput\_2896 | glyoxalase/bleomycin resistance protein/dioxygenase; K10621 2,3-dihydroxy-p-cumate/2,3-dihydroxybenzoate 3,4-dioxygenase [EC:1.13.11.- 1.13.11.14] | ec:1.13.11.14 |
| ppf:Pput\_2897 | aromatic-ring-hydroxylating dioxygenase subunit beta; K16303 p-cumate 2,3-dioxygenase subunit beta [EC:1.14.12.-] |  |
| ppf:Pput\_2898 | ring hydroxylating dioxygenase subunit alpha; K10619 p-cumate 2,3-dioxygenase subunit alpha [EC:1.14.12.-] |  |
| ppf:Pput\_2899 | FAD-dependent pyridine nucleotide-disulfide oxidoreductase; K00529 ferredoxin--NAD+ reductase [EC:1.18.1.3] | ec:1.18.1.3 |
| ppf:Pput\_2900 | prpE; propionyl-CoA synthetase; K01908 propionyl-CoA synthetase [EC:6.2.1.17] | ec:6.2.1.17 |
| ppf:Pput\_2901 | aromatic hydrocarbon degradation membrane protein; K06076 long-chain fatty acid transport protein |  |
| ppf:Pput\_2902 | oxidoreductase FAD-binding subunit; K15758 xylene monooxygenase electron transfer component [EC:1.18.1.3] | ec:1.18.1.3 |

  
**Neighborhood Representations for "rsp:RSP\_2801"**  

| ID | Annotation | EC number |
| --- | --- | --- |
| rsp:RSP\_2810 | ABC transporter, ATPase subunit; K02065 putative ABC transport system ATP-binding protein |  |
| rsp:RSP\_2809 | ABC transporter, inner membrane subunit; K02066 putative ABC transport system permease protein |  |
| rsp:RSP\_2808 | hypothetical protein |  |
| rsp:RSP\_2807 | putative cytochrome b |  |
| rsp:RSP\_4328 | tRNA-Val; K14237 tRNA Val |  |
| rsp:RSP\_2806 | lon; ATP-dependent proteinase (EC:3.4.21.53); K01338 ATP-dependent Lon protease [EC:3.4.21.53] | ec:3.4.21.53 |
| rsp:RSP\_2805 | nemA; Putative NADH-flavin oxidoreductase (EC:1.-.-.-); K10680 N-ethylmaleimide reductase [EC:1.-.-.-] |  |
| rsp:RSP\_2804 | tgt; tRNA-guanine transglycosylase (EC:2.4.2.29); K00773 queuine tRNA-ribosyltransferase [EC:2.4.2.29] | ec:2.4.2.29 |
| rsp:RSP\_2803 | multidrug/cation efflux pump, RND superfamily |  |
| rsp:RSP\_2802 | multidrug/cation efflux pump, membrane fusion protein subunit |  |
| rsp:RSP\_2801 | tetR; transcriptional regulator, TetR family |  |
| rsp:RSP\_6075 | hypothetical protein |  |
| rsp:RSP\_2800 | acoR; Putative AcoR, Transcriptional activator of acetoin/glycerol metabolism |  |
| rsp:RSP\_2799 | Putative Zinc-containing alcohol dehydrogenase (EC:1.1.1.1); K13953 alcohol dehydrogenase, propanol-preferring [EC:1.1.1.1] | ec:1.1.1.1 |
| rsp:RSP\_2798 | groEL3; Putative chaperonin groEL; K04077 chaperonin GroEL |  |
| rsp:RSP\_2797 | putative metal-sulfur cluster biosynthetic enzyme |  |
| rsp:RSP\_2796 | putative TIM-barrel fold metal-dependent hydrolase; K07045 |  |
| rsp:RSP\_2795 | Putative regulatory protein of multicomponent monooxygenase |  |
| rsp:RSP\_2794 | Putative monooxygenase beta subunit; K16158 methane monooxygenase component A beta chain [EC:1.14.13.25] | ec:1.14.13.25 |
| rsp:RSP\_2793 | Putative reductase component of monooxygenase (EC:1.17.1.1) |  |
| rsp:RSP\_2792 | putative monooxygenase alpha subunit (EC:1.14.13.25); K16157 methane monooxygenase component A alpha chain [EC:1.14.13.25] | ec:1.14.13.25 |

  
**Neighborhood Representations for "rsh:Rsph17029\_1498"**  

| ID | Annotation | EC number |
| --- | --- | --- |
| rsh:Rsph17029\_1488 | ABC transporter; K02065 putative ABC transport system ATP-binding protein |  |
| rsh:Rsph17029\_1489 | hypothetical protein; K02066 putative ABC transport system permease protein |  |
| rsh:Rsph17029\_1490 | hypothetical protein |  |
| rsh:Rsph17029\_1491 | cytochrome B561 |  |
| rsh:Rsph17029\_1492 | phage integrase family protein |  |
| rsh:Rsph17029\_1493 | ATP-dependent protease La (EC:3.4.21.53); K01338 ATP-dependent Lon protease [EC:3.4.21.53] | ec:3.4.21.53 |
| rsh:Rsph17029\_1494 | NADH:flavin oxidoreductase; K10680 N-ethylmaleimide reductase [EC:1.-.-.-] |  |
| rsh:Rsph17029\_1495 | tgt; queuine tRNA-ribosyltransferase (EC:2.4.2.29); K00773 queuine tRNA-ribosyltransferase [EC:2.4.2.29] | ec:2.4.2.29 |
| rsh:Rsph17029\_1496 | acriflavin resistance protein |  |
| rsh:Rsph17029\_1497 | RND family efflux transporter MFP subunit |  |
| rsh:Rsph17029\_1498 | TetR family transcriptional regulator |  |
| rsh:Rsph17029\_1499 | hypothetical protein |  |
| rsh:Rsph17029\_1500 | Fis family GAF modulated sigma54 specific transcriptional regulator |  |
| rsh:Rsph17029\_1501 | alcohol dehydrogenase; K13953 alcohol dehydrogenase, propanol-preferring [EC:1.1.1.1] | ec:1.1.1.1 |
| rsh:Rsph17029\_1502 | groEL; chaperonin GroEL; K04077 chaperonin GroEL |  |
| rsh:Rsph17029\_1503 | hypothetical protein |  |
| rsh:Rsph17029\_1504 | amidohydrolase 2; K07045 |  |
| rsh:Rsph17029\_1505 | monooxygenase component MmoB/DmpM |  |
| rsh:Rsph17029\_1506 | methane/phenol/toluene hydroxylase; K16158 methane monooxygenase component A beta chain [EC:1.14.13.25] | ec:1.14.13.25 |
| rsh:Rsph17029\_1507 | oxidoreductase FAD/NAD(P)-binding subunit |  |
| rsh:Rsph17029\_1508 | methane monooxygenase (EC:1.14.13.25); K16157 methane monooxygenase component A alpha chain [EC:1.14.13.25] | ec:1.14.13.25 |

  
**Neighborhood Representations for "rsk:RSKD131\_1166"**  

| ID | Annotation | EC number |
| --- | --- | --- |
| rsk:RSKD131\_1156 | mammalian cell entry domain-containing protein; K02067 putative ABC transport system substrate-binding protein |  |
| rsk:RSKD131\_1157 | ABC transporter; K02065 putative ABC transport system ATP-binding protein |  |
| rsk:RSKD131\_1158 | ABC transporter permease; K02066 putative ABC transport system permease protein |  |
| rsk:RSKD131\_1159 | hypothetical protein |  |
| rsk:RSKD131\_1160 | cytochrome B561 |  |
| rsk:RSKD131\_1161 | ATP-dependent protease La; K01338 ATP-dependent Lon protease [EC:3.4.21.53] | ec:3.4.21.53 |
| rsk:RSKD131\_1162 | NADH:flavin oxidoreductase; K10680 N-ethylmaleimide reductase [EC:1.-.-.-] |  |
| rsk:RSKD131\_1163 | tgt; queuine tRNA-ribosyltransferase; K00773 queuine tRNA-ribosyltransferase [EC:2.4.2.29] | ec:2.4.2.29 |
| rsk:RSKD131\_1164 | Acriflavin resistance protein |  |
| rsk:RSKD131\_1165 | RND family efflux transporter MFP subunit |  |
| rsk:RSKD131\_1166 | TetR family transcriptional regulator |  |
| rsk:RSKD131\_1167 | hypothetical protein |  |
| rsk:RSKD131\_1168 | GAF modulated Fis family sigma-54 specific transcriptional regulator |  |
| rsk:RSKD131\_1169 | alcohol dehydrogenase GroES domain-containing protein; K13953 alcohol dehydrogenase, propanol-preferring [EC:1.1.1.1] | ec:1.1.1.1 |
| rsk:RSKD131\_1170 | groEL; chaperonin GroEL; K04077 chaperonin GroEL |  |
| rsk:RSKD131\_1171 | hypothetical protein |  |
| rsk:RSKD131\_1172 | amidohydrolase; K07045 |  |
| rsk:RSKD131\_1173 | Monooxygenase component MmoB/DmpM |  |
| rsk:RSKD131\_1174 | methane/phenol/toluene hydroxylase; K16158 methane monooxygenase component A beta chain [EC:1.14.13.25] | ec:1.14.13.25 |
| rsk:RSKD131\_1175 | Oxidoreductase FAD/NAD(P)-binding domain-containing protein; K00523 CDP-4-dehydro-6-deoxyglucose reductase [EC:1.17.1.1] | ec:1.17.1.1 |
| rsk:RSKD131\_1176 | methane monooxygenase; K16157 methane monooxygenase component A alpha chain [EC:1.14.13.25] | ec:1.14.13.25 |

  
**Over-represented Enzyme Summary**: Table of E.C. identified protein in the "Neighborhood Representation" ranked by frequency of occurrence  

| EC number | Frequency | Annotation | Reactions |
| --- | --- | --- | --- |
| ec:1.14.13.25 | 6 | methane monooxygenase (soluble); methane hydroxylase | methane + NAD(P)H + H+ + O2 = methanol + NAD(P)+ + H2O [RN:R01142 R01143] |
| ec:1.18.1.3 | 4 | ferredoxin---NAD+ reductase; ferredoxin-nicotinamide adenine dinucleotide reductase; ferredoxin reductase (ambiguous); NAD+-ferredoxin reductase; NADH-ferredoxin oxidoreductase; reductase, reduced nicotinamide adenine dinucleotide-ferredoxin; ferredoxin-NAD+ reductase; NADH-ferredoxin reductase; NADH2-ferredoxin oxidoreductase; NADH flavodoxin oxidoreductase; NADH-ferredoxin NAP reductase (component of naphthalene dioxygenase multicomponent enzyme system); ferredoxin-linked NAD+ reductase; NADH-ferredoxin TOL reductase (component of toluene dioxygenase); ferredoxin---NAD reductase | (1) 2 reduced [2Fe-2S] ferredoxin + NAD+ + H+ = 2 oxidized [2Fe-2S] ferredoxin + NADH [RN:R05875]; (2) reduced 2[4Fe-4S] ferredoxin + NAD+ + H+ = oxidized 2[4Fe-4S] ferredoxin + NADH |
| ec:2.4.2.29 | 3 | tRNA-guanosine34 transglycosylase; guanine insertion enzyme (ambiguous); tRNA transglycosylase (ambiguous); Q-insertase (ambiguous); queuine34 transfer ribonucleate ribosyltransferase; transfer ribonucleate glycosyltransferase (ambiguous); tRNA guanine34 transglycosidase; queuine tRNA-ribosyltransferase (ambiguous); TGT; [tRNA]-guanine34:queuine tRNA-D-ribosyltransferase; transfer ribonucleic acid guanine34 transglycosylase | (1) guanine34 in tRNA + queuine = queuine34 in tRNA + guanine [RN:R03789]; (2) guanine34 in tRNA + 7-aminomethyl-7-carbaguanine = 7-aminomethyl-7-carbaguanine34 in tRNA + guanine [RN:R10209] |
| ec:3.4.21.53 | 3 | endopeptidase La; ATP-dependent serine proteinase; lon proteinase; protease La; proteinase La; ATP-dependent lon proteinase; ATP-dependent protease La; Escherichia coli proteinase La; Escherichia coli serine proteinase La; gene lon protease; gene lon proteins; PIM1 protease; PIM1 proteinase; serine protease La | Hydrolysis of proteins in presence of ATP |
| ec:1.1.1.1 | 3 | alcohol dehydrogenase; aldehyde reductase; ADH; alcohol dehydrogenase (NAD); aliphatic alcohol dehydrogenase; ethanol dehydrogenase; NAD-dependent alcohol dehydrogenase; NAD-specific aromatic alcohol dehydrogenase; NADH-alcohol dehydrogenase; NADH-aldehyde dehydrogenase; primary alcohol dehydrogenase; yeast alcohol dehydrogenase | (1) a primary alcohol + NAD+ = an aldehyde + NADH + H+ [RN:R07326]; (2) a secondary alcohol + NAD+ = a ketone + NADH + H+ [RN:R07327] |
| ec:4.1.3.39 | 2 | 4-hydroxy-2-oxovalerate aldolase; 4-hydroxy-2-ketovalerate aldolase; HOA; DmpG; 4-hydroxy-2-oxovalerate pyruvate-lyase; 4-hydroxy-2-oxopentanoate pyruvate-lyase; BphI; 4-hydroxy-2-oxopentanoate pyruvate-lyase (acetaldehyde-forming) | (S)-4-hydroxy-2-oxopentanoate = acetaldehyde + pyruvate [RN:R00750] |
| ec:1.3.1.58 | 2 | 2,3-dihydroxy-2,3-dihydro-p-cumate dehydrogenase | cis-5,6-dihydroxy-4-isopropylcyclohexa-1,3-dienecarboxylate + NAD+ = 2,3-dihydroxy-p-cumate + NADH + H+ [RN:R05240] |
| ec:1.13.11.14 | 2 | 2,3-dihydroxybenzoate 3,4-dioxygenase; o-pyrocatechuate oxygenase; 2,3-dihydroxybenzoate 1,2-dioxygenase; 2,3-dihydroxybenzoic oxygenase; 2,3-dihydroxybenzoate oxygenase | 2,3-dihydroxybenzoate + O2 = 3-carboxy-2-hydroxymuconate semialdehyde [RN:R01507] |
| ec:4.2.1.80 | 2 | 2-oxopent-4-enoate hydratase; 2-keto-4-pentenoate hydratase; OEH; 2-keto-4-pentenoate (vinylpyruvate)hydratase; 4-hydroxy-2-oxopentanoate hydro-lyase | 4-hydroxy-2-oxopentanoate = 2-oxopent-4-enoate + H2O [RN:R02601] |
| ec:1.2.1.10 | 2 | acetaldehyde dehydrogenase (acetylating); aldehyde dehydrogenase (acylating); ADA; acylating acetaldehyde dehyrogenase; DmpF; BphJ | acetaldehyde + CoA + NAD+ = acetyl-CoA + NADH + H+ [RN:R00228] |
| ec:1.17.1.1 | 1 | CDP-4-dehydro-6-deoxyglucose reductase; CDP-4-keto-6-deoxyglucose reductase; cytidine diphospho-4-keto-6-deoxy-D-glucose reductase; cytidine diphosphate 4-keto-6-deoxy-D-glucose-3-dehydrogenase; CDP-4-keto-deoxy-glucose reductase; CDP-4-keto-6-deoxy-D-glucose-3-dehydrogenase system; NAD(P)H:CDP-4-keto-6-deoxy-D-glucose oxidoreductase | CDP-4-dehydro-3,6-dideoxy-D-glucose + NAD(P)+ + H2O = CDP-4-dehydro-6-deoxy-D-glucose + NAD(P)H + H+ [RN:R03391 R03392] |
| ec:1.14.13.82 | 1 | vanillate monooxygenase; 4-hydroxy-3-methoxybenzoate demethylase; vanillate demethylase | vanillate + O2 + NADH + H+ = 3,4-dihydroxybenzoate + NAD+ + H2O + formaldehyde [RN:R05274] |
| ec:6.2.1.17 | 1 | propionate---CoA ligase; propionyl-CoA synthetase | ATP + propanoate + CoA = AMP + diphosphate + propanoyl-CoA [RN:R00925] |

  
**Over-represented Metabolite Summary**: Collection of the metabolites identified as substrates or products of the proteins representaed the "Over-represented Enzyme Summary" ranked by frequency of occurrence  

| ID | Structure | Name | Frequency | EC |
| --- | --- | --- | --- | --- |
| cpd:C00080 |  | H+; Hydron | 27 | ec:1.2.1.10  ec:1.17.1.1 ec:1.3.1.58 ec:1.18.1.3 ec:1.14.13.25 ec:1.1.1.1 |
| cpd:C00004 |  | NADH; DPNH; Reduced nicotinamide adenine dinucleotide | 27 | ec:1.2.1.10  ec:1.17.1.1 ec:1.3.1.58 ec:1.18.1.3 ec:1.14.13.25 ec:1.1.1.1 |
| cpd:C00003 |  | NAD+; NAD; Nicotinamide adenine dinucleotide; DPN; Diphosphopyridine nucleotide; Nadide | 27 | ec:1.2.1.10  ec:1.17.1.1 ec:1.3.1.58 ec:1.18.1.3 ec:1.14.13.25 ec:1.1.1.1 |
| cpd:C00001 |  | H2O; Water | 24 | ec:1.17.1.1 ec:1.18.1.3 ec:4.2.1.80 ec:1.14.13.25 ec:1.1.1.1 |
| cpd:C00007 |  | Oxygen; O2 | 18 | ec:1.13.11.14 ec:1.18.1.3 ec:1.14.13.25 |
| cpd:C06579 |  | cis-2,3-Dihydroxy-2,3-dihydro-p-cumate; cis-5,6-Dihydroxy-4-isopropylcyclohexa-1,3-dienecarboxylate | 10 | ec:1.3.1.58 ec:1.18.1.3 |
| cpd:C06578 |  | p-Cumate | 8 | ec:1.18.1.3 |
| cpd:C00006 |  | NADP+; NADP; Nicotinamide adenine dinucleotide phosphate; beta-Nicotinamide adenine dinucleotide phosphate; TPN; Triphosphopyridine nucleotide | 8 | ec:1.17.1.1 ec:1.18.1.3 |
| cpd:C00005 |  | NADPH; TPNH; Reduced nicotinamide adenine dinucleotide phosphate | 8 | ec:1.17.1.1 ec:1.18.1.3 |
| cpd:C00084 |  | Acetaldehyde; Ethanal | 7 | ec:1.2.1.10 ec:4.1.3.39 ec:1.1.1.1 |
| cpd:C00011 |  | CO2; Carbon dioxide | 6 | ec:1.18.1.3 |
| cpd:C01455 |  | Toluene; Methylbenzene; Toluol | 6 | ec:1.18.1.3 |
| cpd:C01438 |  | Methane; CH4 | 6 | ec:1.14.13.25 |
| cpd:C00132 |  | Methanol; Methyl alcohol; CH3OH | 6 | ec:1.14.13.25 |
| cpd:C00596 |  | 2-Hydroxy-2,4-pentadienoate; cis-2-Hydroxypenta-2,4-dienoate; Oxopent-4-enoate; 2-Oxopent-4-enoate; 2-Hydroxypenta-2,4-dienoate | 5 | ec:4.2.1.80 |
| cpd:C06585 |  | cis-2,3-Dihydro-2,3-dihydroxy-4'-chlorobiphenyl | 4 | ec:1.18.1.3 |
| cpd:C06584 |  | 4-Chlorobiphenyl; 1-Chloro-4-phenyl benzene; 4-Monochloro-biphenyl | 4 | ec:1.18.1.3 |
| cpd:C04480 |  | 3-Carboxy-2-hydroxymuconate semialdehyde | 4 | ec:1.13.11.14 |
| cpd:C06582 |  | 2-Hydroxy-6-oxo-7-methylocta-2,4-dienoate | 4 |  |
| cpd:C06581 |  | 2-Hydroxy-3-carboxy-6-oxo-7-methylocta-2,4-dienoate | 4 | ec:1.13.11.14 |
| cpd:C06580 |  | 2,3-Dihydroxy-p-cumate | 4 | ec:1.13.11.14 ec:1.3.1.58 |
| cpd:C00014 |  | Ammonia; NH3 | 4 | ec:1.18.1.3 |
| cpd:C01407 |  | Benzene | 4 | ec:1.18.1.3 |
| cpd:C00108 |  | Anthranilate; Anthranilic acid; o-Aminobenzoic acid; Vitamin L1; 2-Aminobenzoate | 4 | ec:1.18.1.3 |
| cpd:C06762 |  | 4-Hydroxy-2-oxohexanoic acid; 4-Hydroxy-2-oxohexanoate | 4 | ec:4.1.3.39 ec:4.2.1.80 |
| cpd:C07111 |  | Ethylbenzene; Phenylethane; Ethylbenzol; Ethylenzene | 4 | ec:1.18.1.3 |
| cpd:C00058 |  | Formate; Methanoic acid; Formic acid | 4 | ec:1.18.1.3 |
| cpd:C00048 |  | Glyoxylate; Glyoxalate; Glyoxylic acid | 4 | ec:1.18.1.3 |
| cpd:C12622 |  | cis-3-(3-Carboxyethenyl)-3,5-cyclohexadiene-1,2-diol; (2E)-3-(cis-5,6-Dihydroxycyclohexa-1,3-dien-1-yl)prop-2-enoate | 4 | ec:1.18.1.3 |
| cpd:C11588 |  | cis-3-(Carboxy-ethyl)-3,5-cyclo-hexadiene-1,2-diol; cis-3-(2-Carboxy-ethyl)-3,5-cyclo-hexadiene-1,2-diol; 3-(cis-5,6-Dihydroxycyclohexa-1,3-dien-1-yl)propanoate | 4 | ec:1.18.1.3 |
| cpd:C04592 |  | Toluene-cis-dihydrodiol; (1S,2R)-3-Methylcyclohexa-3,5-diene-1,2-diol | 4 | ec:1.18.1.3 |
| cpd:C06790 |  | Trichloroethene; Trichloroethylene; TCE | 4 | ec:1.18.1.3 |
| cpd:C00423 |  | trans-Cinnamate; trans-Cinnamic acid; (E)-Cinnamate | 4 | ec:1.18.1.3 |
| cpd:C00090 |  | Catechol; 1,2-Benzenediol; o-Benzenediol; 1,2-Dihydroxybenzene; Brenzcatechin; Pyrocatechol | 4 | ec:1.18.1.3 |
| cpd:C01327 |  | Hydrochloric acid; HCl; Hydrogen chloride; Hydrochloride | 4 | ec:1.18.1.3 |
| cpd:C05629 |  | Phenylpropanoate; 3-Phenyl-propionic acid; 3-Phenylpropanoic acid; 3-Phenylpropionic acid | 4 | ec:1.18.1.3 |
| cpd:C03589 |  | 4-Hydroxy-2-oxopentanoate; 4-Hydroxy-2-oxovalerate | 4 | ec:4.1.3.39 ec:4.2.1.80 |
| cpd:C06727 |  | cis-1,2-Dihydro-3-ethylcatechol; cis-2,3-Dihydroxy-2,3-dihydroethylbenzene; cis-3-Ethyl-cyclohexa-3,5-diene-1,2-diol | 4 | ec:1.18.1.3 |
| cpd:C04091 |  | cis-1,2-Dihydrobenzene-1,2-diol; cis-Benzeneglycol; cis-Cyclohexa-3,5-diene-1,2-diol | 4 | ec:1.18.1.3 |
| cpd:C06589 |  | cis-2,3-Dihydro-2,3-dihydroxybiphenyl; cis-3-Phenylcyclohexa-3,5-diene-1,2-diol; (1S,2R)-3-Phenylcyclohexa-3,5-diene-1,2-diol | 4 | ec:1.18.1.3 |
| cpd:C06588 |  | Biphenyl; Phenylbenzene; 1,1'-Biphenyl; 1,1'-Diphenyl | 4 | ec:1.18.1.3 |
| cpd:C00473 |  | Retinol; all-trans-Retinol; Vitamin A; Vitamin A1 | 3 | ec:1.1.1.1 |
| cpd:C02909 |  | (2-Naphthyl)methanol; 2-Naphthalenemethanol; 2-Hydroxymethylnaphthalene | 3 | ec:1.1.1.1 |
| cpd:C00376 |  | Retinal; Vitamin A aldehyde; Retinene; all-trans-Retinal; all-trans-Vitamin A aldehyde; all-trans-Retinene | 3 | ec:1.1.1.1 |
| cpd:C06613 |  | trans-3-Chloroallyl aldehyde; trans-3-Chloro-2-propenal | 3 | ec:1.1.1.1 |
| cpd:C06612 |  | cis-3-Chloro-2-propene-1-ol; cis-3-Chloroallyl alcohol | 3 | ec:1.1.1.1 |
| cpd:C05577 |  | 3,4-Dihydroxymandelaldehyde; 3,4-Dihydroxyphenylglycolaldehyde | 3 | ec:1.1.1.1 |
| cpd:C06611 |  | trans-3-Chloro-2-propene-1-ol; trans-3-Chloroallyl alcohol | 3 | ec:1.1.1.1 |
| cpd:C05576 |  | 3,4-Dihydroxyphenylethyleneglycol | 3 | ec:1.1.1.1 |
| cpd:C20446 |  | tRNA 7-aminomethyl-7-carbaguanine; tRNA preQ1 | 3 | ec:2.4.2.29 |
| cpd:C00010 |  | CoA; Coenzyme A; CoA-SH | 3 | ec:1.2.1.10 ec:6.2.1.17 |
| cpd:C00469 |  | Ethanol; Ethyl alcohol; Methylcarbinol | 3 | ec:1.1.1.1 |
| cpd:C00071 |  | Aldehyde; RCHO | 3 | ec:1.1.1.1 |
| cpd:C16551 |  | Alcophosphamide | 3 | ec:1.1.1.1 |
| cpd:C16348 |  | cis-3-Chloroallyl aldehyde; cis-3-Chloro-2-propenal | 3 | ec:1.1.1.1 |
| cpd:C01449 |  | Queuine; Base Q | 3 | ec:2.4.2.29 |
| cpd:C00242 |  | Guanine; 2-Amino-6-hydroxypurine | 3 | ec:2.4.2.29 |
| cpd:C16596 |  | 5-Phenyl-1,3-oxazinane-2,4-dione | 3 | ec:1.1.1.1 |
| cpd:C07645 |  | Aldophosphamide | 3 | ec:1.1.1.1 |
| cpd:C16595 |  | 4-Hydroxy-5-phenyltetrahydro-1,3-oxazin-2-one | 3 | ec:1.1.1.1 |
| cpd:C06899 |  | Chloral hydrate | 3 | ec:1.1.1.1 |
| cpd:C16393 |  | 2-Hydroxylamino-4,6-dinitrotoluene | 3 |  |
| cpd:C16392 |  | 4-Hydroxylamino-2,6-dinitrotoluene | 3 |  |
| cpd:C14099 |  | 2-Naphthaldehyde; 2-Naphthalenecarboxaldehyde | 3 | ec:1.1.1.1 |
| cpd:C16391 |  | Trinitrotoluene; 2,4,6-Trinitrotoluene | 3 |  |
| cpd:C14090 |  | 1-Naphthaldehyde; 1-Formylnaphthalene | 3 | ec:1.1.1.1 |
| cpd:C16587 |  | 3-Carbamoyl-2-phenylpropionaldehyde | 3 | ec:1.1.1.1 |
| cpd:C16586 |  | 2-Phenyl-1,3-propanediol monocarbamate | 3 | ec:1.1.1.1 |
| cpd:C01978 |  | tRNA queuine | 3 | ec:2.4.2.29 |
| cpd:C14089 |  | 1-Hydroxymethylnaphthalene; 1-Naphthalenemethanol | 3 | ec:1.1.1.1 |
| cpd:C01977 |  | tRNA guanine | 3 | ec:2.4.2.29 |
| cpd:C00226 |  | Primary alcohol; 1-Alcohol | 3 | ec:1.1.1.1 |
| cpd:C07490 |  | Trichloroethanol; 2,2,2-Trichloroethanol | 3 | ec:1.1.1.1 |
| cpd:C16675 |  | 7-Aminomethyl-7-carbaguanine; 7-Aminomethyl-7-deazaguanine | 3 | ec:2.4.2.29 |
| cpd:C00479 |  | Propanal; Propionaldehyde | 2 | ec:4.1.3.39 |
| cpd:C01412 |  | Butanal; Butyraldehyde | 2 | ec:1.2.1.10 |
| cpd:C06576 |  | p-Cumic alcohol | 2 | ec:1.18.1.3 |
| cpd:C06575 |  | p-Cymene | 2 | ec:1.18.1.3 |
| cpd:C04115 |  | 4-Carboxy-4-hydroxy-2-oxoadipate; 4-Hydroxy-4-carboxymethyl-2-oxoglutarate; 2-Hydroxy-4-oxobutane-1,2,4-tricarboxylate | 2 | ec:4.2.1.80 |
| cpd:C11354 |  | 2-Hydroxy-cis-hex-2,4-dienoate | 2 | ec:4.2.1.80 |
| cpd:C07216 |  | 3-Methylbenzyl alcohol | 2 | ec:1.18.1.3 |
| cpd:C07213 |  | 2-Methylbenzyl alcohol | 2 | ec:1.18.1.3 |
| cpd:C07212 |  | o-Xylene; o-Dimethylbenzene; o-Methyltoluene | 2 | ec:1.18.1.3 |
| cpd:C00163 |  | Propanoate; Propionate; Propanoic acid; Propionic acid | 2 | ec:6.2.1.17 |
| cpd:C00556 |  | Benzyl alcohol; alpha-Hydroxytoluene; Benzenemethanol; Phenylmethanol; Phenylcarbinol; Hydroxymethylbenzene | 2 | ec:1.18.1.3 |
| cpd:C05364 |  | 4-Carboxy-2-oxo-3-hexenedioate | 2 | ec:4.2.1.80 |
| cpd:C07208 |  | m-Xylene; 1,3-Dimethylbenzene; 1,3-Xylene | 2 | ec:1.18.1.3 |
| cpd:C06757 |  | 4-Methylbenzyl alcohol; alpha-Hydroxy-p-xylene; 4-Tolylcarbinol | 2 | ec:1.18.1.3 |
| cpd:C06756 |  | p-Xylene; 1,4-Dimethylbenzene; p-Methyltoluene | 2 | ec:1.18.1.3 |
| cpd:C00340 |  | Reduced rubredoxin | 2 | ec:1.18.1.3 |
| cpd:C00435 |  | Oxidized rubredoxin | 2 | ec:1.18.1.3 |
| cpd:C02632 |  | 2-Methylpropanoate; 2-Methylpropanoic acid; Isobutyric acid; Isobutanoate; Dimethylacetic acid; Isobutyrate | 2 |  |
| cpd:C00139 |  | Oxidized ferredoxin | 2 | ec:1.18.1.3 |
| cpd:C00138 |  | Reduced ferredoxin | 2 | ec:1.18.1.3 |
| cpd:C00136 |  | Butanoyl-CoA; Butyryl-CoA | 2 | ec:1.2.1.10 |
| cpd:C00196 |  | 2,3-Dihydroxybenzoate; 2,3-Dihydroxybenzoic acid | 2 | ec:1.13.11.14 |
| cpd:C00682 |  | 2-Hydroxymuconate semialdehyde; 2-Hydroxymuconic semialdehyde; 2-Hydroxymuconate-6-semialdehyde | 2 |  |
| cpd:C00024 |  | Acetyl-CoA; Acetyl coenzyme A | 2 | ec:1.2.1.10 |
| cpd:C00022 |  | Pyruvate; Pyruvic acid; 2-Oxopropanoate; 2-Oxopropanoic acid; Pyroracemic acid | 2 | ec:4.1.3.39 |
| cpd:C00020 |  | AMP; Adenosine 5'-monophosphate; Adenylic acid; Adenylate; 5'-AMP; 5'-Adenylic acid; 5'-Adenosine monophosphate; Adenosine 5'-phosphate | 1 | ec:6.2.1.17 |
| cpd:C01219 |  | CDP-4-dehydro-6-deoxy-D-glucose | 1 | ec:1.17.1.1 |
| cpd:C07123 |  | 2-Hydroxy-6-oxo-octa-2,4-dienoate | 1 |  |
| cpd:C00013 |  | Diphosphate; Diphosphoric acid; Pyrophosphate; Pyrophosphoric acid; PPi | 1 | ec:6.2.1.17 |
| cpd:C06210 |  | 2-Hydroxy-6-keto-2,4-heptadienoate; 2-Hydroxy-6-oxo-hept-2,4-dienoate | 1 |  |
| cpd:C00100 |  | Propanoyl-CoA; Propionyl-CoA; Propionyl coenzyme A | 1 | ec:6.2.1.17 |
| cpd:C00002 |  | ATP; Adenosine 5'-triphosphate | 1 | ec:6.2.1.17 |
| cpd:C00033 |  | Acetate; Acetic acid; Ethanoic acid | 1 |  |
| cpd:C05983 |  | Propionyladenylate; Propionyl-adenosine monophosphate | 1 | ec:6.2.1.17 |
| cpd:C04297 |  | CDP-4-dehydro-3,6-dideoxy-D-glucose; CDP-3,6-dideoxy-D-erythro-hexos-4-ulose; CDP-4-dehydro-3,6-dideoxy-alpha-D-glucose | 1 | ec:1.17.1.1 |

  
**Over-represented Pathway Summary**: Collection of the KEGG metabolic pathways containing the proteins identified in the "Over-represented Metabolite Summary" ranked by the highest number of hits per pathway  

| Pathway ID | EC | EC Frequency | Name |
| --- | --- | --- | --- |
| map00622 | ec:1.3.1.58 ec:4.1.3.39 ec:1.2.1.10 ec:4.2.1.80 ec:1.18.1.3 | 12 | path:map00622 Xylene degradation |
| map00362 | ec:1.13.11.14 ec:4.1.3.39 ec:1.2.1.10 ec:4.2.1.80 | 8 | path:map00362 Benzoate degradation |
| map00071 | ec:1.1.1.1 ec:1.18.1.3 | 7 | path:map00071 Fatty acid degradation |
| map00621 | ec:4.1.3.39 ec:1.2.1.10 ec:4.2.1.80 | 6 | path:map00621 Dioxin degradation |
| map00680 | ec:1.14.13.25 | 6 | path:map00680 Methane metabolism |
| map00360 | ec:4.1.3.39 ec:4.2.1.80 | 4 | path:map00360 Phenylalanine metabolism |
| map00010 | ec:1.1.1.1 | 3 | path:map00010 Glycolysis / Gluconeogenesis |
| map00350 | ec:1.1.1.1 | 3 | path:map00350 Tyrosine metabolism |
| map00830 | ec:1.1.1.1 | 3 | path:map00830 Retinol metabolism |
| map00982 | ec:1.1.1.1 | 3 | path:map00982 Drug metabolism - cytochrome P450 |
| map00980 | ec:1.1.1.1 | 3 | path:map00980 Metabolism of xenobiotics by cytochrome P450 |
| map00626 | ec:1.1.1.1 | 3 | path:map00626 Naphthalene degradation |
| map00625 | ec:1.1.1.1 | 3 | path:map00625 Chloroalkane and chloroalkene degradation |
| map00260 | ec:1.1.1.1 | 3 | path:map00260 Glycine, serine and threonine metabolism |
| map00620 | ec:1.2.1.10 | 2 | path:map00620 Pyruvate metabolism |
| map00650 | ec:1.2.1.10 | 2 | path:map00650 Butanoate metabolism |
| map00520 | ec:1.17.1.1 | 1 | path:map00520 Amino sugar and nucleotide sugar metabolism |
| map00640 | ec:6.2.1.17 | 1 | path:map00640 Propanoate metabolism |
| map00627 | ec:1.14.13.82 | 1 | path:map00627 Aminobenzoate degradation |

  
Analysis performed on 2014/02/19 16:45:27
