# Supplementary material for: Automated genomic context analysis and experimental validation platform for discovery of prokaryote transcriptional regulator functions
Source: BMC Genomics. 2014 Dec 18;15(1):1142. doi: 10.1186/1471-2164-15-1142 (PMC4349456; doi:10.1186/1471-2164-15-1142)
Supplement: Supplementary file 5 — Additional file 5: Result CysB. Function Discovery V1.0 output (.html format) for the sulfur metabolism regulator (CysB, Bxe_ A2466). For detailed instructions on how to analyze the results please refer to the Function Discovery V1.0, a gene neighborhood analysis tool section in the Results part of the main text. (HTML 676 KB) [file 12864_2014_6995_MOESM5_ESM.html]

```
ENTRY       Bxe_A2466         CDS       T00340
DEFINITION  transcriptional regulator CysB-like protein
ORTHOLOGY   K13635  LysR family transcriptional regulator, cys regulon transcriptional activator
ORGANISM    bxe  Burkholderia xenovorans
POSITION    1:2197290..2198216
MOTIF       Pfam: LysR_substrate HTH_1 HTH_30 MTS MarR PBP_like SBP_bac_11
DBLINKS     NCBI-GI: 91783353
            NCBI-GeneID: 4004043
            JGI: BxeA2466
            UniProt: Q13ZI2
AASEQ       308
            MNFQQLRFVREAVRQNMNLTEVANVLYTSQSGVSKQIKDLEDELGVDIFIRRGKRLTGLT
            EPGKAVHQLIERMLLDAENLRRVARQYADQDSGHLVVATTHTQARYALPKVIRQFTEVFP
            KVHLALRQGSPQQIAQMIINGEADIGISTEALDRFPDIVTFPCYSWHHVVVVPKGHPLVG
            RPNLTLDEIAEFPIVTYDQDFTGRSHIDQAFAKAGALPDVVLTAIDADVIKTYVELGMGI
            GVVAAMAYDPKRDTELVALDTQHLFEASTTRVGLRKGAFLRAYAYRLIEMFAPQLNEADI
            AAQLREAV
NTSEQ       927
            atgaatttccagcaattgcgcttcgtgcgcgaagccgtacgtcagaacatgaatctgacc
            gaggtggcgaacgtgctgtacacgtcgcaatcaggcgtgtccaaacagatcaaggatctg
            gaggacgaactcggcgtcgatattttcatccggcgcggcaagcgtctgacgggcctcacc
            gagccgggcaaggcggtgcatcagttgatcgagcggatgctgctcgacgccgagaatcta
            cgccgcgtcgcgcgccagtacgccgatcaggatagcgggcacctcgtcgtggccaccacg
            cacacgcaggcgcgctacgcgctgccaaaggtgatccgccagttcaccgaggtgttcccc
            aaggtgcatctggcgctgcgccagggaagcccgcaacagatcgcgcagatgatcatcaac
            ggcgaagcggacatcggcatctccaccgaagcgctcgaccgcttcccggatatcgtcacg
            ttcccgtgctattcgtggcatcacgtggtggtcgtgccgaagggtcatccgctggtgggc
            cggccgaatctgacgctcgacgagatcgccgaattcccgatcgtcacgtacgaccaggat
            ttcacgggccgctcgcatatcgaccaggcgttcgcgaaagcgggcgcgttgcccgacgtc
            gtgctgaccgcgatcgacgccgacgtgatcaagacgtatgtcgaactgggcatgggtatc
            ggcgtggttgcggcgatggcctacgaccccaagcgcgacacggaactggtcgcgctcgac
            acgcagcatctgttcgaagcgagcacgacgcgggtcggtttgcgcaagggcgcgttcctg
            cgtgcttacgcgtaccggctgatcgagatgttcgcgccgcagttgaacgaagcggatatc
            gccgcgcagttgcgcgaagcagtctga
///
```

  
**Homolog ID**: Table of closest homologs  

```
                 Homologs                                       len   identity overlap
---------------------------------------------------------------------------------
bpy:Bphyt_1777 transcriptional regulator CysB-like prot K     308     1.000    308 
bgf:BC1003_1492 LysR family transcriptional regulator   K     308     0.984    308 
bge:BC1002_1348 transcriptional regulator, LysR family  K     308     0.980    307 
bph:Bphy_1625 transcriptional regulator CysB-like prote K     308     0.954    307 
bvi:Bcep1808_1557 transcriptional regulator CysB-like p K     308     0.951    308 
bmj:BMULJ_01610 transcriptional regulator CysB-like pro K     308     0.948    308 
bmu:Bmul_1632 transcriptional regulator CysB-like prote K     308     0.948    308 
bac:BamMC406_1521 transcriptional regulator CysB-like p K     308     0.945    308 
bam:Bamb_1501 transcriptional regulator CysB-like prote K     308     0.945    308 
bur:Bcep18194_A4743 transcriptional regulator CysB-like K     308     0.938    308 
bch:Bcen2424_1605 transcriptional regulator CysB-like p K     308     0.938    308 
bcj:BCAL1656 transcriptional regulator CysB-like protei K     308     0.938    308 
bcm:Bcenmc03_1582 transcriptional regulator CysB-like p K     308     0.938    308 
bcn:Bcen_1125 transcriptional regulator CysB-like prote K     308     0.935    308 
bma:BMA1205 transcriptional regulator CysB-like protein K     308     0.935    307 
bml:BMA10229_A0350 transcriptional regulator CysB-like  K     308     0.935    307 
bmn:BMA10247_0810 transcriptional regulator CysB-like p K     308     0.935    307 
bmv:BMASAVP1_A1694 transcriptional regulator CysB-like  K     308     0.935    307 
bpd:BURPS668_1851 transcriptional regulator CysB-like p K     308     0.935    307 
bpl:BURPS1106A_1865 transcriptional regulator CysB-like K     308     0.935    307 
bpm:BURPS1710b_2018 transcriptional regulator CysB-like K     308     0.935    307 
bpr:GBP346_A1888 transcriptional regulator CysB-like pr K     308     0.935    307 
bps:BPSL1835 transcriptional regulator CysB-like protei K     308     0.935    307 
bte:BTH_I2476 transcriptional regulator CysB-like prote K     308     0.928    307 
bgl:bglu_1g18690 transcriptional regulator CysB-like pr K     309     0.925    308 
brh:RBRH_00145 transcriptional regulator cbl            K     308     0.840    307 
hdn:Hden_2672 LysR family transcriptional regulator     K     310     0.626    302 
dar:Daro_2129 transcriptional regulator CysB-like prote K     311     0.593    305 
app:CAP2UW1_0224 transcriptional regulator CysB-like pr K     311     0.585    306 
hse:Hsero_1658 cys regulon transcription regulator prot K     311     0.580    307 
neu:NE0572 transcriptional regulator CysB-like protein  K     305     0.580    305 
nmu:Nmul_A1739 transcriptional regulator CysB-like prot K     314     0.593    305 
azo:azo1332 transcriptional regulator CysB-like protein K     311     0.584    308 
mpt:Mpe_A0127 LysR family transcriptional regulator     K     311     0.607    295 
tmz:Tmz1t_0621 transcriptional regulator CysB-like prot K     309     0.585    301 
aav:Aave_3039 LysR family transcriptional regulator     K     309     0.578    308 
dac:Daci_5610 LysR family transcriptional regulator     K     354     0.586    309 
cvi:CV_1827 transcriptional regulator CysB-like protein K     309     0.575    308 
lch:Lcho_3810 LysR family transcriptional regulator     K     312     0.592    306 
gca:Galf_1924 transcriptional regulator, LysR family    K     310     0.576    302 
mms:mma_0257 cys regulon transcriptional activator      K     304     0.588    301 
ctt:CtCNB1_3069 LysR family transcriptional regulator   K     310     0.571    308 
mmw:Mmwyl1_0963 LysR family transcriptional regulator   K     308     0.585    306 
slt:Slit_1030 LysR family transcriptional regulator     K     308     0.571    303 
har:HEAR0839 transcriptional regulator Cbl              K     308     0.560    307 
axy:AXYL_03028 transcriptional regulator                K     309     0.542    306 
rpf:Rpic12D_1287 CysB family transcriptional regulator  K     319     0.562    308 
alv:Alvin_2440 LysR family transcriptional regulator    K     310     0.568    308 
rsc:RCFBP_20081 LysR family transcriptional regulator   K     327     0.558    308 
rpi:Rpic_1224 transcriptional regulator CysB-like prote K     319     0.558    308 
rso:RSc1348 CysB family transcriptional regulator       K     327     0.555    308 
rsl:RPSI07_2019 cysteine biosynthesis and aliphatic sul K     319     0.555    308 
pna:Pnap_0071 LysR family transcriptional regulator     K     314     0.550    307 
vap:Vapar_0061 LysR family transcriptional regulator    K     314     0.572    304 
reh:H16_A2236 transcriptional regulator CysB-like prote K     314     0.562    306 
rme:Rmet_1379 transcriptional regulator CysB-like prote K     316     0.560    307 
reu:Reut_A1967 transcriptional regulator CysB-like prot K     315     0.560    307 
tau:Tola_0017 transcriptional regulator Cbl             K     318     0.546    304 
cti:RALTA_A1775 CysB family transcriptional regulator   K     314     0.559    306 
ddd:Dda3937_04117 LysR family transcriptional regulator K     317     0.543    304 
asa:ASA_0598 transcriptional regulator CysB-like protei K     315     0.545    303 
aha:AHA_0609 transcriptional regulator CysB-like protei K     315     0.548    303 
yen:YE2474 transcriptional regulator CysB-like protein  K     318     0.532    308 
net:Neut_1186 transcriptional regulator CysB-like prote K     309     0.541    305 
afe:Lferr_0847 LysR family transcriptional regulator    K     320     0.523    308 
afr:AFE_0699 sulfur assimilation LysR family transcript K     320     0.523    308 
ebd:ECBD_1661 transcriptional regulator Cbl             K     316     0.544    307 
ebr:ECB_01897 transcriptional regulator Cbl             K     316     0.544    307 
ebw:BWG_1782 transcriptional regulator Cbl              K     316     0.544    307 
ecd:ECDH10B_2131 transcriptional regulator Cbl          K     316     0.544    307 
ecj:Y75_p1949 DNA-binding transcriptional activator     K     316     0.544    307 
ecm:EcSMS35_1137 transcriptional regulator Cbl          K     316     0.546    306 
eco:b1987 DNA-binding transcriptional activator for the K     316     0.544    307 
ecw:EcE24377A_2269 transcriptional regulator Cbl        K     316     0.546    306 
ecy:ECSE_2272 transcriptional regulator Cbl             K     316     0.546    306 
eoh:ECO103_2449 DNA-binding transcriptional activator C K     316     0.546    306 
eoi:ECO111_2634 DNA-binding transcriptional activator   K     316     0.546    306 
cko:CKO_00956 transcriptional regulator Cbl             K     342     0.547    307 
eca:ECA1519 transcriptional regulator CysB-like protein K     317     0.543    300 
ece:Z3146 transcriptional regulator Cbl                 K     316     0.542    306 
ecf:ECH74115_2831 transcriptional regulator Cbl         K     316     0.542    306 
ecs:ECs2783 transcriptional regulator Cbl               K     316     0.542    306 
ecz:ECS88_2053 transcriptional regulator Cbl            K     316     0.542    306 
eok:G2583_2497 transcriptional regulator Cbl            K     316     0.542    306 
etw:ECSP_2651 transcriptional regulator Cbl             K     316     0.542    306 
dda:Dd703_1289 transcriptional regulator CysB-like prot K     317     0.530    304 
ddc:Dd586_2611 LysR family transcriptional regulator    K     317     0.526    304 
eck:EC55989_2222 transcriptional regulator Cbl          K     316     0.542    306 
mfa:Mfla_1669 transcriptional regulator CysB-like prote K     312     0.511    305 
ssn:SSON_2042 transcriptional regulator Cbl             K     316     0.546    306 
ecq:ECED1_2324 transcriptional regulator Cbl            K     316     0.544    307 
aci:ACIAD2597 CysB family transcriptional regulator     K     307     0.551    296 
ecl:EcolC_1658 transcriptional regulator Cbl            K     316     0.546    306 
sdy:SDY_2246 transcriptional regulator Cbl              K     316     0.546    306 
abb:ABBFA_000909 CysB family transcriptional regulator  K     307     0.543    302 
abc:ACICU_02812 transcriptional regulator CysB-like pro K     307     0.543    302 
abm:ABSDF0935 CysB family transcriptional regulator     K     307     0.543    302 
abn:AB57_2978 transcriptional regulator CysB-like prote K     307     0.543    302 
aby:ABAYE0925 transcriptional regulator CysB-like prote K     307     0.543    302 
acd:AOLE_04485 transcriptional regulator CysB-like prot K     307     0.546    302
```

**Neighborhood Representations**: Table of genes in the defined genetic neighborhoods of the entry protein and its closest homologs  
  
**Neighborhood Representations for "bxe:Bxe\_A2466"**  

| ID | Annotation | EC number |
| --- | --- | --- |
| bxe:Bxe\_A2476 | ligolipopolysacharide ABC exporter inner membrane subunit NodJ family protein; K09694 lipooligosaccharide transport system permease protein |  |
| bxe:Bxe\_A2475 | nodulation ABC transporter NodI; K09695 lipooligosaccharide transport system ATP-binding protein |  |
| bxe:Bxe\_A2474 | universal stress protein, UspA |  |
| bxe:Bxe\_A2473 | transmembrane protein |  |
| bxe:Bxe\_A2472 | hypothetical protein |  |
| bxe:Bxe\_A2471 | LexA repressor (EC:3.4.21.88); K01356 repressor LexA [EC:3.4.21.88] | ec:3.4.21.88 |
| bxe:Bxe\_A2470 | ABC sulfate/thiosulfate transporter, periplasmic ligand binding protein, CysP; K02048 sulfate transport system substrate-binding protein |  |
| bxe:Bxe\_A2469 | ABC sulfate/thiosulfate transporter, innermembrane subunit, CysT; K02046 sulfate transport system permease protein |  |
| bxe:Bxe\_A2468 | ABC sulfate/thiosulfate transporter, innermembrane subunit, CysW; K02047 sulfate transport system permease protein |  |
| bxe:Bxe\_A2467 | ABC sulfate/thiosulfate transporter, ATPase subunit, CysA; K02045 sulfate transport system ATP-binding protein [EC:3.6.3.25] | ec:3.6.3.25 |
| bxe:Bxe\_A2466 | transcriptional regulator CysB-like protein; K13635 LysR family transcriptional regulator, cys regulon transcriptional activator |  |
| bxe:Bxe\_A2465 | hypothetical protein |  |
| bxe:Bxe\_A2464 | L-asparaginase, type II (EC:3.5.1.1); K01424 L-asparaginase [EC:3.5.1.1] | ec:3.5.1.1 |
| bxe:Bxe\_A2463 | hypothetical protein |  |
| bxe:Bxe\_A2462 | 2-hydroxy-3-oxopropionate reductase (EC:1.1.1.60); K00042 2-hydroxy-3-oxopropionate reductase [EC:1.1.1.60] | ec:1.1.1.60 |
| bxe:Bxe\_A2461 | hydroxypyruvate isomerase (EC:5.3.1.22); K01816 hydroxypyruvate isomerase [EC:5.3.1.22] | ec:5.3.1.22 |
| bxe:Bxe\_A2460 | glyoxylate carboligase (EC:4.1.1.47); K01608 tartronate-semialdehyde synthase [EC:4.1.1.47] | ec:4.1.1.47 |
| bxe:Bxe\_A2459 | LysR family transcriptional regulator |  |
| bxe:Bxe\_A2458 | RNA polymerase sigma factor; K03088 RNA polymerase sigma-70 factor, ECF subfamily |  |
| bxe:Bxe\_A2457 | transmembrane transcriptional regulator (anti-sigma factor) |  |
| bxe:Bxe\_A2456 | rpsF; 30S ribosomal protein S6; K02990 small subunit ribosomal protein S6 |  |

  
**Neighborhood Representations for "bpy:Bphyt\_1777"**  

| ID | Annotation | EC number |
| --- | --- | --- |
| bpy:Bphyt\_1767 | sugar ABC transporter permease; K09694 lipooligosaccharide transport system permease protein |  |
| bpy:Bphyt\_1768 | nodulation ABC transporter NodI; K09695 lipooligosaccharide transport system ATP-binding protein |  |
| bpy:Bphyt\_1769 | UspA domain-containing protein |  |
| bpy:Bphyt\_1770 | transmembrane protein |  |
| bpy:Bphyt\_1771 | hypothetical protein |  |
| bpy:Bphyt\_1772 | LexA repressor (EC:3.4.21.88); K01356 repressor LexA [EC:3.4.21.88] | ec:3.4.21.88 |
| bpy:Bphyt\_1773 | sulfate ABC transporter substrate-binding protein; K02048 sulfate transport system substrate-binding protein |  |
| bpy:Bphyt\_1774 | sulfate ABC transporter permease; K02046 sulfate transport system permease protein |  |
| bpy:Bphyt\_1775 | sulfate ABC transporter permease; K02047 sulfate transport system permease protein |  |
| bpy:Bphyt\_1776 | sulfate ABC transporter ATPase; K02045 sulfate transport system ATP-binding protein [EC:3.6.3.25] | ec:3.6.3.25 |
| bpy:Bphyt\_1777 | transcriptional regulator CysB-like protein; K13635 LysR family transcriptional regulator, cys regulon transcriptional activator |  |
| bpy:Bphyt\_1778 | type II L-asparaginase; K01424 L-asparaginase [EC:3.5.1.1] | ec:3.5.1.1 |
| bpy:Bphyt\_1779 | hypothetical protein |  |
| bpy:Bphyt\_1780 | 2-hydroxy-3-oxopropionate reductase (EC:1.1.1.60); K00042 2-hydroxy-3-oxopropionate reductase [EC:1.1.1.60] | ec:1.1.1.60 |
| bpy:Bphyt\_1781 | hydroxypyruvate isomerase (EC:5.3.1.22); K01816 hydroxypyruvate isomerase [EC:5.3.1.22] | ec:5.3.1.22 |
| bpy:Bphyt\_1782 | glyoxylate carboligase; K01608 tartronate-semialdehyde synthase [EC:4.1.1.47] | ec:4.1.1.47 |
| bpy:Bphyt\_1783 | LysR family transcriptional regulator (EC:4.2.1.1) |  |
| bpy:Bphyt\_1784 | RNA polymerase sigma factor; K03088 RNA polymerase sigma-70 factor, ECF subfamily |  |
| bpy:Bphyt\_1785 | transmembrane anti-sigma factor |  |
| bpy:Bphyt\_1786 | rpsF; 30S ribosomal protein S6; K02990 small subunit ribosomal protein S6 |  |
| bpy:Bphyt\_1787 | primosomal replication protein N, PriB; K02686 primosomal replication protein N |  |

  
**Neighborhood Representations for "bgf:BC1003\_1492"**  

| ID | Annotation | EC number |
| --- | --- | --- |
| bgf:BC1003\_1482 | ABC-2 type transporter NodJ family; K09694 lipooligosaccharide transport system permease protein |  |
| bgf:BC1003\_1483 | nodulation ABC transporter NodI; K09695 lipooligosaccharide transport system ATP-binding protein |  |
| bgf:BC1003\_1484 | UspA domain-containing protein |  |
| bgf:BC1003\_1485 | putative transmembrane protein |  |
| bgf:BC1003\_1486 | hypothetical protein |  |
| bgf:BC1003\_1487 | LexA family transcriptional repressor; K01356 repressor LexA [EC:3.4.21.88] | ec:3.4.21.88 |
| bgf:BC1003\_1488 | sulfate ABC transporter substrate-binding protein; K02048 sulfate transport system substrate-binding protein |  |
| bgf:BC1003\_1489 | sulfate ABC transporter permease; K02046 sulfate transport system permease protein |  |
| bgf:BC1003\_1490 | sulfate ABC transporter permease; K02047 sulfate transport system permease protein |  |
| bgf:BC1003\_1491 | sulfate ABC transporter ATPase; K02045 sulfate transport system ATP-binding protein [EC:3.6.3.25] | ec:3.6.3.25 |
| bgf:BC1003\_1492 | LysR family transcriptional regulator; K13635 LysR family transcriptional regulator, cys regulon transcriptional activator |  |
| bgf:BC1003\_1493 | type II L-asparaginase; K01424 L-asparaginase [EC:3.5.1.1] | ec:3.5.1.1 |
| bgf:BC1003\_1494 | Peptidoglycan-binding lysin domain-containing protein |  |
| bgf:BC1003\_1495 | 2-hydroxy-3-oxopropionate reductase (EC:1.1.1.60); K00042 2-hydroxy-3-oxopropionate reductase [EC:1.1.1.60] | ec:1.1.1.60 |
| bgf:BC1003\_1496 | hydroxypyruvate isomerase (EC:5.3.1.22); K01816 hydroxypyruvate isomerase [EC:5.3.1.22] | ec:5.3.1.22 |
| bgf:BC1003\_1497 | glyoxylate carboligase; K01608 tartronate-semialdehyde synthase [EC:4.1.1.47] | ec:4.1.1.47 |
| bgf:BC1003\_1498 | LysR family transcriptional regulator |  |
| bgf:BC1003\_1499 | 30S ribosomal protein S6; K02990 small subunit ribosomal protein S6 |  |
| bgf:BC1003\_1500 | putative primosomal replication protein N, PriB; K02686 primosomal replication protein N |  |
| bgf:BC1003\_1501 | 30S ribosomal protein S18; K02963 small subunit ribosomal protein S18 |  |
| bgf:BC1003\_1502 | 50S ribosomal protein L9; K02939 large subunit ribosomal protein L9 |  |

  
**Neighborhood Representations for "bge:BC1002\_1348"**  

| ID | Annotation | EC number |
| --- | --- | --- |
| bge:BC1002\_1338 | NodJ family ABC transporter; K09694 lipooligosaccharide transport system permease protein |  |
| bge:BC1002\_1339 | nodulation ABC transporter NodI; K09695 lipooligosaccharide transport system ATP-binding protein |  |
| bge:BC1002\_1340 | UspA domain-containing protein |  |
| bge:BC1002\_1341 | transmembrane protein |  |
| bge:BC1002\_1342 | hypothetical protein |  |
| bge:BC1002\_1343 | LexA family transcriptional repressor (EC:3.4.21.88); K01356 repressor LexA [EC:3.4.21.88] | ec:3.4.21.88 |
| bge:BC1002\_1344 | sulfate ABC transporter, periplasmic sulfate-binding protein; K02048 sulfate transport system substrate-binding protein |  |
| bge:BC1002\_1345 | sulfate ABC transporter, inner membrane subunit CysT; K02046 sulfate transport system permease protein |  |
| bge:BC1002\_1346 | sulfate ABC transporter, inner membrane subunit CysW; K02047 sulfate transport system permease protein |  |
| bge:BC1002\_1347 | sulfate ABC transporter ATPase; K02045 sulfate transport system ATP-binding protein [EC:3.6.3.25] | ec:3.6.3.25 |
| bge:BC1002\_1348 | transcriptional regulator, LysR family; K13635 LysR family transcriptional regulator, cys regulon transcriptional activator |  |
| bge:BC1002\_1349 | YadA domain-containing protein |  |
| bge:BC1002\_1350 | hemagluttinin domain protein |  |
| bge:BC1002\_1351 | type II L-asparaginase; K01424 L-asparaginase [EC:3.5.1.1] | ec:3.5.1.1 |
| bge:BC1002\_1352 | 2-hydroxy-3-oxopropionate reductase; K00042 2-hydroxy-3-oxopropionate reductase [EC:1.1.1.60] | ec:1.1.1.60 |
| bge:BC1002\_1353 | hydroxypyruvate isomerase (EC:5.3.1.22); K01816 hydroxypyruvate isomerase [EC:5.3.1.22] | ec:5.3.1.22 |
| bge:BC1002\_1354 | glyoxylate carboligase; K01608 tartronate-semialdehyde synthase [EC:4.1.1.47] | ec:4.1.1.47 |
| bge:BC1002\_1355 | transcriptional regulator, LysR family |  |
| bge:BC1002\_1356 | ECF subfamily RNA polymerase sigma-24 subunit; K03088 RNA polymerase sigma-70 factor, ECF subfamily |  |
| bge:BC1002\_1357 | transmembrane anti-sigma factor |  |
| bge:BC1002\_1358 | 30S ribosomal protein S6; K02990 small subunit ribosomal protein S6 |  |

  
**Neighborhood Representations for "bph:Bphy\_1625"**  

| ID | Annotation | EC number |
| --- | --- | --- |
| bph:Bphy\_1615 | hypothetical protein; K07002 |  |
| bph:Bphy\_1616 | gamma-glutamyltransferase (EC:2.3.2.2); K00681 gamma-glutamyltranspeptidase [EC:2.3.2.2] | ec:2.3.2.2 |
| bph:Bphy\_1617 | Ser/Thr protein phosphatase family protein |  |
| bph:Bphy\_1618 | cytochrome c oxidase subunit II; K02275 cytochrome c oxidase subunit II [EC:1.9.3.1] | ec:1.9.3.1 |
| bph:Bphy\_1619 | LysR family transcriptional regulator (EC:4.2.1.1) |  |
| bph:Bphy\_1620 | glyoxylate carboligase; K01608 tartronate-semialdehyde synthase [EC:4.1.1.47] | ec:4.1.1.47 |
| bph:Bphy\_1621 | hydroxypyruvate isomerase (EC:5.3.1.22); K01816 hydroxypyruvate isomerase [EC:5.3.1.22] | ec:5.3.1.22 |
| bph:Bphy\_1622 | 2-hydroxy-3-oxopropionate reductase (EC:1.1.1.60); K00042 2-hydroxy-3-oxopropionate reductase [EC:1.1.1.60] | ec:1.1.1.60 |
| bph:Bphy\_1623 | L-sorbosone dehydrogenase |  |
| bph:Bphy\_1624 | type II L-asparaginase; K01424 L-asparaginase [EC:3.5.1.1] | ec:3.5.1.1 |
| bph:Bphy\_1625 | transcriptional regulator CysB-like protein; K13635 LysR family transcriptional regulator, cys regulon transcriptional activator |  |
| bph:Bphy\_1626 | sulfate ABC transporter ATPase; K02045 sulfate transport system ATP-binding protein [EC:3.6.3.25] | ec:3.6.3.25 |
| bph:Bphy\_1627 | sulfate ABC transporter inner membrane subunit CysW; K02047 sulfate transport system permease protein |  |
| bph:Bphy\_1628 | sulfate ABC transporter inner membrane subunit CysT; K02046 sulfate transport system permease protein |  |
| bph:Bphy\_1629 | sulfate ABC transporter periplasmic sulfate-binding protein; K02048 sulfate transport system substrate-binding protein |  |
| bph:Bphy\_1630 | LexA repressor (EC:3.4.21.88); K01356 repressor LexA [EC:3.4.21.88] | ec:3.4.21.88 |
| bph:Bphy\_1631 | hypothetical protein |  |
| bph:Bphy\_1632 | putative transmembrane protein |  |
| bph:Bphy\_1633 | UspA domain-containing protein |  |
| bph:Bphy\_1634 | nodulation ABC transporter NodI; K09695 lipooligosaccharide transport system ATP-binding protein |  |
| bph:Bphy\_1635 | ABC transporter; K09694 lipooligosaccharide transport system permease protein |  |

  
**Neighborhood Representations for "bvi:Bcep1808\_1557"**  

| ID | Annotation | EC number |
| --- | --- | --- |
| bvi:Bcep1808\_1547 | ABC transporter; K09694 lipooligosaccharide transport system permease protein |  |
| bvi:Bcep1808\_1548 | nodulation ABC transporter NodI; K09695 lipooligosaccharide transport system ATP-binding protein |  |
| bvi:Bcep1808\_1549 | UspA domain-containing protein |  |
| bvi:Bcep1808\_1550 | hypothetical protein |  |
| bvi:Bcep1808\_1551 | hypothetical protein |  |
| bvi:Bcep1808\_1552 | LexA repressor (EC:3.4.21.88); K01356 repressor LexA [EC:3.4.21.88] | ec:3.4.21.88 |
| bvi:Bcep1808\_1553 | sulfate ABC transporter periplasmic sulfate-binding protein; K02048 sulfate transport system substrate-binding protein |  |
| bvi:Bcep1808\_1554 | sulfate ABC transporter inner membrane subunit CysT; K02046 sulfate transport system permease protein |  |
| bvi:Bcep1808\_1555 | Fis family transcriptional regulator; K02047 sulfate transport system permease protein |  |
| bvi:Bcep1808\_1556 | sulfate ABC transporter ATPase subunit; K02045 sulfate transport system ATP-binding protein [EC:3.6.3.25] | ec:3.6.3.25 |
| bvi:Bcep1808\_1557 | transcriptional regulator CysB-like protein; K13635 LysR family transcriptional regulator, cys regulon transcriptional activator |  |
| bvi:Bcep1808\_1558 | periplasmic binding protein/LacI transcriptional regulator; K10439 ribose transport system substrate-binding protein |  |
| bvi:Bcep1808\_1559 | ABC transporter-like protein; K10441 ribose transport system ATP-binding protein [EC:3.6.3.17] | ec:3.6.3.17 |
| bvi:Bcep1808\_1560 | inner-membrane translocator; K10440 ribose transport system permease protein |  |
| bvi:Bcep1808\_1561 | LacI family transcription regulator; K02529 LacI family transcriptional regulator |  |
| bvi:Bcep1808\_1562 | ribokinase; K00852 ribokinase [EC:2.7.1.15] | ec:2.7.1.15 |
| bvi:Bcep1808\_1563 | methyl-accepting chemotaxis sensory transducer; K05874 methyl-accepting chemotaxis protein I, serine sensor receptor |  |
| bvi:Bcep1808\_1564 | putative serine protein kinase PrkA; K07180 serine protein kinase |  |
| bvi:Bcep1808\_1565 | hypothetical protein; K09786 hypothetical protein |  |
| bvi:Bcep1808\_1566 | SpoVR family protein |  |
| bvi:Bcep1808\_1567 | major facilitator superfamily metabolite/H(+) symporter; K03761 MFS transporter, MHS family, alpha-ketoglutarate permease |  |

  
**Neighborhood Representations for "bmj:BMULJ\_01610"**  

| ID | Annotation | EC number |
| --- | --- | --- |
| bmj:BMULJ\_01600 | ABC-2 type transporter permease; K09694 lipooligosaccharide transport system permease protein |  |
| bmj:BMULJ\_01601 | nodulation ABC transporter NodI; K09695 lipooligosaccharide transport system ATP-binding protein |  |
| bmj:BMULJ\_01602 | UspA family putative universal stress protein |  |
| bmj:BMULJ\_01603 | hypothetical protein |  |
| bmj:BMULJ\_01604 | hypothetical protein |  |
| bmj:BMULJ\_01605 | lexA; LexA repressor (EC:3.4.21.88); K01356 repressor LexA [EC:3.4.21.88] | ec:3.4.21.88 |
| bmj:BMULJ\_01606 | cysP; sulfate transporter substrate-binding protein; K02048 sulfate transport system substrate-binding protein |  |
| bmj:BMULJ\_01607 | cysU; sulfate transporter permease; K02046 sulfate transport system permease protein |  |
| bmj:BMULJ\_01608 | cysW; sulfate transporter permease; K02047 sulfate transport system permease protein |  |
| bmj:BMULJ\_01609 | cysA; sulfate transporter ATP-binding protein; K02045 sulfate transport system ATP-binding protein [EC:3.6.3.25] | ec:3.6.3.25 |
| bmj:BMULJ\_01610 | cbl; transcriptional regulator CysB-like protein; K13635 LysR family transcriptional regulator, cys regulon transcriptional activator |  |
| bmj:BMULJ\_01611 | simple sugar transporter substrate-binding protein; K10439 ribose transport system substrate-binding protein |  |
| bmj:BMULJ\_01612 | ABC-type sugar transporter ATPase; K10441 ribose transport system ATP-binding protein [EC:3.6.3.17] | ec:3.6.3.17 |
| bmj:BMULJ\_01613 | simple sugar transporter permease; K10440 ribose transport system permease protein |  |
| bmj:BMULJ\_01614 | LacI family transcriptional regulator; K02529 LacI family transcriptional regulator |  |
| bmj:BMULJ\_01615 | rbsK; ribokinase (EC:2.7.1.15); K00852 ribokinase [EC:2.7.1.15] | ec:2.7.1.15 |
| bmj:BMULJ\_01616 | pseudogene |  |
| bmj:BMULJ\_01617 | pseudogene |  |
| bmj:BMULJ\_01618 | hypothetical protein |  |
| bmj:BMULJ\_01619 | prkA; serine protein kinase; K07180 serine protein kinase |  |
| bmj:BMULJ\_01620 | hypothetical protein; K09786 hypothetical protein |  |

  
**Neighborhood Representations for "bmu:Bmul\_1632"**  

| ID | Annotation | EC number |
| --- | --- | --- |
| bmu:Bmul\_1622 | major facilitator superfamily metabolite/H(+) symporter; K03761 MFS transporter, MHS family, alpha-ketoglutarate permease |  |
| bmu:Bmul\_1623 | SpoVR family protein |  |
| bmu:Bmul\_1624 | hypothetical protein; K09786 hypothetical protein |  |
| bmu:Bmul\_1625 | putative serine protein kinase PrkA; K07180 serine protein kinase |  |
| bmu:Bmul\_1626 | methyl-accepting chemotaxis sensory transducer |  |
| bmu:Bmul\_1627 | ribokinase; K00852 ribokinase [EC:2.7.1.15] | ec:2.7.1.15 |
| bmu:Bmul\_1628 | LacI family transcriptional regulator; K02529 LacI family transcriptional regulator |  |
| bmu:Bmul\_1629 | monosaccharide-transporting ATPase (EC:3.6.3.17); K10440 ribose transport system permease protein |  |
| bmu:Bmul\_1630 | ABC transporter-like protein; K10441 ribose transport system ATP-binding protein [EC:3.6.3.17] | ec:3.6.3.17 |
| bmu:Bmul\_1631 | periplasmic binding protein/LacI transcriptional regulator; K10439 ribose transport system substrate-binding protein |  |
| bmu:Bmul\_1632 | transcriptional regulator CysB-like protein; K13635 LysR family transcriptional regulator, cys regulon transcriptional activator |  |
| bmu:Bmul\_1633 | sulfate ABC transporter ATPase; K02045 sulfate transport system ATP-binding protein [EC:3.6.3.25] | ec:3.6.3.25 |
| bmu:Bmul\_1634 | sulfate ABC transporter permease; K02047 sulfate transport system permease protein |  |
| bmu:Bmul\_1635 | sulfate ABC transporter inner membrane subunit CysT; K02046 sulfate transport system permease protein |  |
| bmu:Bmul\_1636 | sulfate ABC transporter periplasmic sulfate-binding protein; K02048 sulfate transport system substrate-binding protein |  |
| bmu:Bmul\_1637 | LexA repressor (EC:3.4.21.88); K01356 repressor LexA [EC:3.4.21.88] | ec:3.4.21.88 |
| bmu:Bmul\_1638 | hypothetical protein |  |
| bmu:Bmul\_1639 | hypothetical protein |  |
| bmu:Bmul\_1640 | UspA domain-containing protein |  |
| bmu:Bmul\_1641 | nodulation ABC transporter NodI; K09695 lipooligosaccharide transport system ATP-binding protein |  |
| bmu:Bmul\_1642 | ABC transporter; K09694 lipooligosaccharide transport system permease protein |  |

  
**Neighborhood Representations for "bac:BamMC406\_1521"**  

| ID | Annotation | EC number |
| --- | --- | --- |
| bac:BamMC406\_1511 | ABC transporter; K09694 lipooligosaccharide transport system permease protein |  |
| bac:BamMC406\_1512 | nodulation ABC transporter NodI; K09695 lipooligosaccharide transport system ATP-binding protein |  |
| bac:BamMC406\_1513 | UspA domain-containing protein |  |
| bac:BamMC406\_1514 | hypothetical protein |  |
| bac:BamMC406\_1515 | hypothetical protein |  |
| bac:BamMC406\_1516 | LexA repressor (EC:3.4.21.88); K01356 repressor LexA [EC:3.4.21.88] | ec:3.4.21.88 |
| bac:BamMC406\_1517 | sulfate ABC transporter substrate-binding protein; K02048 sulfate transport system substrate-binding protein |  |
| bac:BamMC406\_1518 | sulfate ABC transporter permease; K02046 sulfate transport system permease protein |  |
| bac:BamMC406\_1519 | sulfate ABC transporter permease; K02047 sulfate transport system permease protein |  |
| bac:BamMC406\_1520 | sulfate ABC transporter ATPase; K02045 sulfate transport system ATP-binding protein [EC:3.6.3.25] | ec:3.6.3.25 |
| bac:BamMC406\_1521 | transcriptional regulator CysB-like protein; K13635 LysR family transcriptional regulator, cys regulon transcriptional activator |  |
| bac:BamMC406\_1522 | periplasmic binding protein/LacI transcriptional regulator; K10439 ribose transport system substrate-binding protein |  |
| bac:BamMC406\_1523 | ABC transporter-like protein; K10441 ribose transport system ATP-binding protein [EC:3.6.3.17] | ec:3.6.3.17 |
| bac:BamMC406\_1524 | monosaccharide-transporting ATPase (EC:3.6.3.17); K10440 ribose transport system permease protein |  |
| bac:BamMC406\_1525 | LacI family transcriptional regulator; K02529 LacI family transcriptional regulator |  |
| bac:BamMC406\_1526 | ribokinase; K00852 ribokinase [EC:2.7.1.15] | ec:2.7.1.15 |
| bac:BamMC406\_1527 | methyl-accepting chemotaxis sensory transducer; K05874 methyl-accepting chemotaxis protein I, serine sensor receptor |  |
| bac:BamMC406\_1528 | putative serine protein kinase PrkA; K07180 serine protein kinase |  |
| bac:BamMC406\_1529 | hypothetical protein; K09786 hypothetical protein |  |
| bac:BamMC406\_1530 | SpoVR family protein |  |
| bac:BamMC406\_1531 | major facilitator superfamily metabolite/H(+) symporter; K03761 MFS transporter, MHS family, alpha-ketoglutarate permease |  |

  
**Neighborhood Representations for "bam:Bamb\_1501"**  

| ID | Annotation | EC number |
| --- | --- | --- |
| bam:Bamb\_1491 | ABC transporter; K09694 lipooligosaccharide transport system permease protein |  |
| bam:Bamb\_1492 | nodulation ABC transporter NodI; K09695 lipooligosaccharide transport system ATP-binding protein |  |
| bam:Bamb\_1493 | UspA domain-containing protein |  |
| bam:Bamb\_1494 | hypothetical protein |  |
| bam:Bamb\_1495 | hypothetical protein |  |
| bam:Bamb\_1496 | LexA repressor (EC:3.4.21.88); K01356 repressor LexA [EC:3.4.21.88] | ec:3.4.21.88 |
| bam:Bamb\_1497 | sulfate ABC transporter substrate-binding protein; K02048 sulfate transport system substrate-binding protein |  |
| bam:Bamb\_1498 | sulfate ABC transporter permease; K02046 sulfate transport system permease protein |  |
| bam:Bamb\_1499 | sulfate ABC transporter permease; K02047 sulfate transport system permease protein |  |
| bam:Bamb\_1500 | sulfate ABC transporter ATPase; K02045 sulfate transport system ATP-binding protein [EC:3.6.3.25] | ec:3.6.3.25 |
| bam:Bamb\_1501 | transcriptional regulator CysB-like protein; K13635 LysR family transcriptional regulator, cys regulon transcriptional activator |  |
| bam:Bamb\_1502 | hypothetical protein |  |
| bam:Bamb\_1503 | periplasmic binding protein/LacI transcriptional regulator; K10439 ribose transport system substrate-binding protein |  |
| bam:Bamb\_1504 | ABC transporter-like protein; K10441 ribose transport system ATP-binding protein [EC:3.6.3.17] | ec:3.6.3.17 |
| bam:Bamb\_1505 | inner-membrane translocator; K10440 ribose transport system permease protein |  |
| bam:Bamb\_1506 | LacI family transcriptional regulator; K02529 LacI family transcriptional regulator |  |
| bam:Bamb\_1507 | ribokinase; K00852 ribokinase [EC:2.7.1.15] | ec:2.7.1.15 |
| bam:Bamb\_1508 | methyl-accepting chemotaxis sensory transducer; K05874 methyl-accepting chemotaxis protein I, serine sensor receptor |  |
| bam:Bamb\_1509 | hypothetical protein |  |
| bam:Bamb\_1510 | serine protein kinase PrkA; K07180 serine protein kinase |  |
| bam:Bamb\_1511 | hypothetical protein; K09786 hypothetical protein |  |

  
**Neighborhood Representations for "bur:Bcep18194\_A4743"**  

| ID | Annotation | EC number |
| --- | --- | --- |
| bur:Bcep18194\_A4733 | ABC transporter inner membrane protein NodJ; K09694 lipooligosaccharide transport system permease protein |  |
| bur:Bcep18194\_A4734 | nodulation ABC transporter NodI (EC:3.6.3.25); K09695 lipooligosaccharide transport system ATP-binding protein |  |
| bur:Bcep18194\_A4735 | universal stress protein |  |
| bur:Bcep18194\_A4736 | hypothetical protein |  |
| bur:Bcep18194\_A4737 | hypothetical protein |  |
| bur:Bcep18194\_A4738 | LexA repressor (EC:3.4.21.88); K01356 repressor LexA [EC:3.4.21.88] | ec:3.4.21.88 |
| bur:Bcep18194\_A4739 | thiosulfate-binding protein; K02048 sulfate transport system substrate-binding protein |  |
| bur:Bcep18194\_A4740 | sulfate ABC transporter permease; K02046 sulfate transport system permease protein |  |
| bur:Bcep18194\_A4741 | sulfate ABC transporter permease; K02047 sulfate transport system permease protein |  |
| bur:Bcep18194\_A4742 | sulfate ABC transporter ATPase (EC:3.6.3.25); K02045 sulfate transport system ATP-binding protein [EC:3.6.3.25] | ec:3.6.3.25 |
| bur:Bcep18194\_A4743 | transcriptional regulator CysB-like protein; K13635 LysR family transcriptional regulator, cys regulon transcriptional activator |  |
| bur:Bcep18194\_A4744 | hypothetical protein |  |
| bur:Bcep18194\_A4745 | sugar ABC transporter periplasmic substrate-binding protein; K10439 ribose transport system substrate-binding protein |  |
| bur:Bcep18194\_A4746 | sugar ABC transporter ATPase (EC:3.6.3.25); K10441 ribose transport system ATP-binding protein [EC:3.6.3.17] | ec:3.6.3.17 |
| bur:Bcep18194\_A4747 | sugar ABC transporter inner membrane protein; K10440 ribose transport system permease protein |  |
| bur:Bcep18194\_A4748 | LacI family transcriptional regulator; K02529 LacI family transcriptional regulator |  |
| bur:Bcep18194\_A4749 | ribokinase (EC:2.7.1.15); K00852 ribokinase [EC:2.7.1.15] | ec:2.7.1.15 |
| bur:Bcep18194\_A4750 | methyl-accepting chemotaxis sensory transducer; K05874 methyl-accepting chemotaxis protein I, serine sensor receptor |  |
| bur:Bcep18194\_A4751 | serine protein kinase PrkA; K07180 serine protein kinase |  |
| bur:Bcep18194\_A4752 | hypothetical protein; K09786 hypothetical protein |  |
| bur:Bcep18194\_A4753 | SpoVR family protein |  |

  
**Neighborhood Representations for "bch:Bcen2424\_1605"**  

| ID | Annotation | EC number |
| --- | --- | --- |
| bch:Bcen2424\_1595 | ABC transporter; K09694 lipooligosaccharide transport system permease protein |  |
| bch:Bcen2424\_1596 | nodulation ABC transporter NodI; K09695 lipooligosaccharide transport system ATP-binding protein |  |
| bch:Bcen2424\_1597 | UspA domain-containing protein |  |
| bch:Bcen2424\_1598 | hypothetical protein |  |
| bch:Bcen2424\_1599 | hypothetical protein |  |
| bch:Bcen2424\_1600 | LexA repressor (EC:3.4.21.88); K01356 repressor LexA [EC:3.4.21.88] | ec:3.4.21.88 |
| bch:Bcen2424\_1601 | sulfate ABC transporter periplasmic sulfate-binding protein; K02048 sulfate transport system substrate-binding protein |  |
| bch:Bcen2424\_1602 | sulfate ABC transporter inner membrane subunit CysT; K02046 sulfate transport system permease protein |  |
| bch:Bcen2424\_1603 | sulfate ABC transporter inner membrane subunit CysW; K02047 sulfate transport system permease protein |  |
| bch:Bcen2424\_1604 | sulfate ABC transporter ATPase; K02045 sulfate transport system ATP-binding protein [EC:3.6.3.25] | ec:3.6.3.25 |
| bch:Bcen2424\_1605 | transcriptional regulator CysB-like protein; K13635 LysR family transcriptional regulator, cys regulon transcriptional activator |  |
| bch:Bcen2424\_1606 | periplasmic binding protein/LacI transcriptional regulator; K10439 ribose transport system substrate-binding protein |  |
| bch:Bcen2424\_1607 | ABC transporter-like protein; K10441 ribose transport system ATP-binding protein [EC:3.6.3.17] | ec:3.6.3.17 |
| bch:Bcen2424\_1608 | inner-membrane translocator; K10440 ribose transport system permease protein |  |
| bch:Bcen2424\_1609 | LacI family transcription regulator; K02529 LacI family transcriptional regulator |  |
| bch:Bcen2424\_1610 | ribokinase; K00852 ribokinase [EC:2.7.1.15] | ec:2.7.1.15 |
| bch:Bcen2424\_1611 | methyl-accepting chemotaxis sensory transducer; K05874 methyl-accepting chemotaxis protein I, serine sensor receptor |  |
| bch:Bcen2424\_1612 | hypothetical protein |  |
| bch:Bcen2424\_1613 | putative serine protein kinase PrkA; K07180 serine protein kinase |  |
| bch:Bcen2424\_1614 | hypothetical protein; K09786 hypothetical protein |  |
| bch:Bcen2424\_1615 | SpoVR family protein |  |

  
**Neighborhood Representations for "bcj:BCAL1656"**  

| ID | Annotation | EC number |
| --- | --- | --- |
| bcj:BCAL1646 | putative oligosaccharide ABC transporter protein; K09694 lipooligosaccharide transport system permease protein |  |
| bcj:BCAL1647 | nodulation ABC transporter NodI; K09695 lipooligosaccharide transport system ATP-binding protein |  |
| bcj:BCAL1648 | putative stress-related protein |  |
| bcj:BCAL1649 | hypothetical protein |  |
| bcj:BCAL1650 | hypothetical protein |  |
| bcj:BCAL1651 | lexA; LexA repressor (EC:3.4.21.88); K01356 repressor LexA [EC:3.4.21.88] | ec:3.4.21.88 |
| bcj:BCAL1652 | sbp; sulfate-binding protein; K02048 sulfate transport system substrate-binding protein |  |
| bcj:BCAL1653 | cysT; sulfate transport system permease; K02046 sulfate transport system permease protein |  |
| bcj:BCAL1654 | cysW; sulfate transport system permease; K02047 sulfate transport system permease protein |  |
| bcj:BCAL1655 | cysA; sulfate ABC transporter ATP-binding protein; K02045 sulfate transport system ATP-binding protein [EC:3.6.3.25] | ec:3.6.3.25 |
| bcj:BCAL1656 | ssuR; transcriptional regulator CysB-like protein; K13635 LysR family transcriptional regulator, cys regulon transcriptional activator |  |
| bcj:BCAL1657 | putative ribose transport system, substrate-binding protein; K10439 ribose transport system substrate-binding protein |  |
| bcj:BCAL1658 | putative ribose ABC transporter ATP-binding protein; K10441 ribose transport system ATP-binding protein [EC:3.6.3.17] | ec:3.6.3.17 |
| bcj:BCAL1659 | putative ribose transport system, permease; K10440 ribose transport system permease protein |  |
| bcj:BCAL1660 | putative ribose operon repressor; K02529 LacI family transcriptional regulator |  |
| bcj:BCAL1661 | putative ribokinase; K00852 ribokinase [EC:2.7.1.15] | ec:2.7.1.15 |
| bcj:BCAL1662 | putative methyl-accepting chemotaxis protein; K05874 methyl-accepting chemotaxis protein I, serine sensor receptor |  |
| bcj:BCAL1663 | PrkA family serine protein kinase; K07180 serine protein kinase |  |
| bcj:BCAL1664 | hypothetical protein; K09786 hypothetical protein |  |
| bcj:BCAL1665 | SpoVR family protein |  |
| bcj:BCAL1666 | kgtP; alpha-ketoglutarate permease; K03761 MFS transporter, MHS family, alpha-ketoglutarate permease |  |

  
**Neighborhood Representations for "bcm:Bcenmc03\_1582"**  

| ID | Annotation | EC number |
| --- | --- | --- |
| bcm:Bcenmc03\_1572 | ABC transporter; K09694 lipooligosaccharide transport system permease protein |  |
| bcm:Bcenmc03\_1573 | nodulation ABC transporter NodI; K09695 lipooligosaccharide transport system ATP-binding protein |  |
| bcm:Bcenmc03\_1574 | UspA domain-containing protein |  |
| bcm:Bcenmc03\_1575 | hypothetical protein |  |
| bcm:Bcenmc03\_1576 | hypothetical protein |  |
| bcm:Bcenmc03\_1577 | LexA repressor (EC:3.4.21.88); K01356 repressor LexA [EC:3.4.21.88] | ec:3.4.21.88 |
| bcm:Bcenmc03\_1578 | sulfate ABC transporter substrate-binding protein; K02048 sulfate transport system substrate-binding protein |  |
| bcm:Bcenmc03\_1579 | sulfate ABC transporter permease; K02046 sulfate transport system permease protein |  |
| bcm:Bcenmc03\_1580 | sulfate ABC transporter permease; K02047 sulfate transport system permease protein |  |
| bcm:Bcenmc03\_1581 | sulfate ABC transporter ATPase; K02045 sulfate transport system ATP-binding protein [EC:3.6.3.25] | ec:3.6.3.25 |
| bcm:Bcenmc03\_1582 | transcriptional regulator CysB-like protein; K13635 LysR family transcriptional regulator, cys regulon transcriptional activator |  |
| bcm:Bcenmc03\_1583 | periplasmic binding protein/LacI transcriptional regulator; K10439 ribose transport system substrate-binding protein |  |
| bcm:Bcenmc03\_1584 | ABC transporter-like protein; K10441 ribose transport system ATP-binding protein [EC:3.6.3.17] | ec:3.6.3.17 |
| bcm:Bcenmc03\_1585 | monosaccharide-transporting ATPase (EC:3.6.3.17); K10440 ribose transport system permease protein |  |
| bcm:Bcenmc03\_1586 | LacI family transcriptional regulator; K02529 LacI family transcriptional regulator |  |
| bcm:Bcenmc03\_1587 | ribokinase; K00852 ribokinase [EC:2.7.1.15] | ec:2.7.1.15 |
| bcm:Bcenmc03\_1588 | methyl-accepting chemotaxis sensory transducer; K05874 methyl-accepting chemotaxis protein I, serine sensor receptor |  |
| bcm:Bcenmc03\_1589 | putative serine protein kinase PrkA; K07180 serine protein kinase |  |
| bcm:Bcenmc03\_1590 | hypothetical protein; K09786 hypothetical protein |  |
| bcm:Bcenmc03\_1591 | SpoVR family protein |  |
| bcm:Bcenmc03\_1592 | major facilitator superfamily metabolite/H(+) symporter; K03761 MFS transporter, MHS family, alpha-ketoglutarate permease |  |

  
**Neighborhood Representations for "bcn:Bcen\_1125"**  

| ID | Annotation | EC number |
| --- | --- | --- |
| bcn:Bcen\_1115 | ABC transporter; K09694 lipooligosaccharide transport system permease protein |  |
| bcn:Bcen\_1116 | nodulation ABC transporter NodI; K09695 lipooligosaccharide transport system ATP-binding protein |  |
| bcn:Bcen\_1117 | hypothetical protein |  |
| bcn:Bcen\_1118 | hypothetical protein |  |
| bcn:Bcen\_1119 | hypothetical protein |  |
| bcn:Bcen\_1120 | LexA repressor (EC:3.4.21.88); K01356 repressor LexA [EC:3.4.21.88] | ec:3.4.21.88 |
| bcn:Bcen\_1121 | thiosulfate-binding protein; K02048 sulfate transport system substrate-binding protein |  |
| bcn:Bcen\_1122 | sulfate ABC transporter permease; K02046 sulfate transport system permease protein |  |
| bcn:Bcen\_1123 | sulfate ABC transporter permease; K02047 sulfate transport system permease protein |  |
| bcn:Bcen\_1124 | sulfate ABC transporter permease 1; K02045 sulfate transport system ATP-binding protein [EC:3.6.3.25] | ec:3.6.3.25 |
| bcn:Bcen\_1125 | transcriptional regulator CysB-like protein; K13635 LysR family transcriptional regulator, cys regulon transcriptional activator |  |
| bcn:Bcen\_1126 | periplasmic binding protein/LacI transcriptional regulator; K10439 ribose transport system substrate-binding protein |  |
| bcn:Bcen\_1127 | ABC transporter; K10441 ribose transport system ATP-binding protein [EC:3.6.3.17] | ec:3.6.3.17 |
| bcn:Bcen\_1128 | inner-membrane translocator; K10440 ribose transport system permease protein |  |
| bcn:Bcen\_1129 | LacI family transcriptional regulator; K02529 LacI family transcriptional regulator |  |
| bcn:Bcen\_1130 | ribokinase; K00852 ribokinase [EC:2.7.1.15] | ec:2.7.1.15 |
| bcn:Bcen\_1131 | methyl-accepting chemotaxis sensory transducer; K05874 methyl-accepting chemotaxis protein I, serine sensor receptor |  |
| bcn:Bcen\_1132 | hypothetical protein |  |
| bcn:Bcen\_1133 | serine protein kinase PrkA; K07180 serine protein kinase |  |
| bcn:Bcen\_1134 | hypothetical protein; K09786 hypothetical protein |  |
| bcn:Bcen\_1135 | SpoVR family protein |  |

  
**Neighborhood Representations for "bma:BMA1205"**  

| ID | Annotation | EC number |
| --- | --- | --- |
| bma:BMA1195 | zinc-binding dehydrogenase family oxidoreductase (EC:1.1.1.-); K00008 L-iditol 2-dehydrogenase [EC:1.1.1.14] | ec:1.1.1.14 |
| bma:BMA1196 | rbsC; ribose ABC transporter permease; K10440 ribose transport system permease protein |  |
| bma:BMA1197 | rbsA; ribose ABC transporter ATP-binding protein; K10441 ribose transport system ATP-binding protein [EC:3.6.3.17] | ec:3.6.3.17 |
| bma:BMA1198 | rbsB; ribose ABC transporter periplasmic ribose-binding protein; K10439 ribose transport system substrate-binding protein |  |
| bma:BMA1199 | AraC family transcriptional regulator |  |
| bma:BMA1200 | hypothetical protein |  |
| bma:BMA1201 | hypothetical protein |  |
| bma:BMA1202 | hypothetical protein |  |
| bma:BMA1203 | IS407A, transposase OrfB; K07497 putative transposase |  |
| bma:BMA1204 | IS407A, transposase OrfA; K07497 putative transposase |  |
| bma:BMA1205 | transcriptional regulator CysB-like protein; K13635 LysR family transcriptional regulator, cys regulon transcriptional activator |  |
| bma:BMA1206 | cysA; sulfate ABC transporter ATP-binding protein; K02045 sulfate transport system ATP-binding protein [EC:3.6.3.25] | ec:3.6.3.25 |
| bma:BMA1207 | cysW; sulfate ABC transporter permease; K02047 sulfate transport system permease protein |  |
| bma:BMA1208 | cysT; pseudogene |  |
| bma:BMA1209 | sbp; sulfate ABC transporter substrate-binding protein; K02048 sulfate transport system substrate-binding protein |  |
| bma:BMA1210 | pseudogene |  |
| bma:BMA1211 | lexA; LexA repressor (EC:3.4.21.88); K01356 repressor LexA [EC:3.4.21.88] | ec:3.4.21.88 |
| bma:BMA1212 | hypothetical protein |  |
| bma:BMA1213 | hypothetical protein |  |
| bma:BMA1214 | hypothetical protein |  |
| bma:BMA1215 | universal stress protein |  |

  
**Neighborhood Representations for "bml:BMA10229\_A0350"**  

| ID | Annotation | EC number |
| --- | --- | --- |
| bml:BMA10229\_A0340 | permease; K02050 NitT/TauT family transport system permease protein |  |
| bml:BMA10229\_A0341 | nitrate/sulfonate/bicarbonate ABC transporter ATPase; K02049 NitT/TauT family transport system ATP-binding protein |  |
| bml:BMA10229\_A0342 | hypothetical protein |  |
| bml:BMA10229\_A0343 | ABC transporter substrate-binding protein; K02051 NitT/TauT family transport system substrate-binding protein |  |
| bml:BMA10229\_A0344 | alpha-ketoglutarate permease; K03761 MFS transporter, MHS family, alpha-ketoglutarate permease |  |
| bml:BMA10229\_A0345 | SpoVR family protein |  |
| bml:BMA10229\_A0346 | hypothetical protein; K09786 hypothetical protein |  |
| bml:BMA10229\_A0347 | serine protein kinase; K07180 serine protein kinase |  |
| bml:BMA10229\_A0348 | IS407A, transposase OrfB; K07497 putative transposase |  |
| bml:BMA10229\_A0349 | IS407A, transposase OrfA |  |
| bml:BMA10229\_A0350 | transcriptional regulator CysB-like protein; K13635 LysR family transcriptional regulator, cys regulon transcriptional activator |  |
| bml:BMA10229\_A0351 | cysA; sulfate/thiosulfate ABC transporter ATP-binding protein (EC:3.6.3.25); K02045 sulfate transport system ATP-binding protein [EC:3.6.3.25] | ec:3.6.3.25 |
| bml:BMA10229\_A0352 | cysW; sulfate/thiosulfate ABC transporter permease CysW; K02047 sulfate transport system permease protein |  |
| bml:BMA10229\_A0353 | cysT; sulfate/thiosulfate ABC transporter permease CysT; K02046 sulfate transport system permease protein |  |
| bml:BMA10229\_A0354 | sbp; sulfate/thiosulfate ABC transporter sulfate-binding protein; K02048 sulfate transport system substrate-binding protein |  |
| bml:BMA10229\_A0355 | hypothetical protein |  |
| bml:BMA10229\_A0356 | lexA; LexA repressor (EC:3.4.21.88); K01356 repressor LexA [EC:3.4.21.88] | ec:3.4.21.88 |
| bml:BMA10229\_A0357 | hypothetical protein |  |
| bml:BMA10229\_A0358 | hypothetical protein |  |
| bml:BMA10229\_A0359 | universal stress protein |  |
| bml:BMA10229\_A0360 | nodI; nodulation ABC transporter NodI; K09695 lipooligosaccharide transport system ATP-binding protein |  |

  
**Neighborhood Representations for "bmn:BMA10247\_0810"**  

| ID | Annotation | EC number |
| --- | --- | --- |
| bmn:BMA10247\_0800 | nodJ; ABC transporter permease NodJ; K09694 lipooligosaccharide transport system permease protein |  |
| bmn:BMA10247\_0801 | nodI; nodulation ABC transporter NodI; K09695 lipooligosaccharide transport system ATP-binding protein |  |
| bmn:BMA10247\_0802 | universal stress family protein |  |
| bmn:BMA10247\_0803 | hypothetical protein |  |
| bmn:BMA10247\_0804 | hypothetical protein |  |
| bmn:BMA10247\_0805 | lexA; LexA repressor (EC:3.4.21.88); K01356 repressor LexA [EC:3.4.21.88] | ec:3.4.21.88 |
| bmn:BMA10247\_0806 | sbp; sulfate/thiosulfate ABC transporter sulfate-binding protein; K02048 sulfate transport system substrate-binding protein |  |
| bmn:BMA10247\_0807 | cysT; sulfate/thiosulfate ABC transporter permease CysT; K02046 sulfate transport system permease protein |  |
| bmn:BMA10247\_0808 | cysW; sulfate/thiosulfate ABC transporter permease CysW; K02047 sulfate transport system permease protein |  |
| bmn:BMA10247\_0809 | cysA; sulfate/thiosulfate ABC transporter ATP-binding protein (EC:3.6.3.25); K02045 sulfate transport system ATP-binding protein [EC:3.6.3.25] | ec:3.6.3.25 |
| bmn:BMA10247\_0810 | transcriptional regulator CysB-like protein; K13635 LysR family transcriptional regulator, cys regulon transcriptional activator |  |
| bmn:BMA10247\_0811 | hypothetical protein |  |
| bmn:BMA10247\_0812 | IS407A, transposase OrfA |  |
| bmn:BMA10247\_0813 | IS407A, transposase OrfB; K07497 putative transposase |  |
| bmn:BMA10247\_0814 | protein kinase; K07180 serine protein kinase |  |
| bmn:BMA10247\_0815 | hypothetical protein; K09786 hypothetical protein |  |
| bmn:BMA10247\_0816 | SpoVR family protein |  |
| bmn:BMA10247\_0817 | dicarboxylic acid transporter PcaT; K03761 MFS transporter, MHS family, alpha-ketoglutarate permease |  |
| bmn:BMA10247\_0818 | ABC transporter substrate binding protein; K02051 NitT/TauT family transport system substrate-binding protein |  |
| bmn:BMA10247\_0819 | hypothetical protein |  |
| bmn:BMA10247\_0820 | ABC transporter ATP-binding protein; K02049 NitT/TauT family transport system ATP-binding protein |  |

  
**Neighborhood Representations for "bmv:BMASAVP1\_A1694"**  

| ID | Annotation | EC number |
| --- | --- | --- |
| bmv:BMASAVP1\_A1684 | ABC nitrate/sulfonate/bicarbonate transporter, permease; K02050 NitT/TauT family transport system permease protein |  |
| bmv:BMASAVP1\_A1685 | ABC nitrate/sulfonate/bicarbonate transporter, ATPase subunit; K02049 NitT/TauT family transport system ATP-binding protein |  |
| bmv:BMASAVP1\_A1686 | hypothetical protein |  |
| bmv:BMASAVP1\_A1687 | ABC transporter substrate binding protein; K02051 NitT/TauT family transport system substrate-binding protein |  |
| bmv:BMASAVP1\_A1688 | alpha-ketoglutarate permease; K03761 MFS transporter, MHS family, alpha-ketoglutarate permease |  |
| bmv:BMASAVP1\_A1689 | SpoVR family protein |  |
| bmv:BMASAVP1\_A1690 | hypothetical protein; K09786 hypothetical protein |  |
| bmv:BMASAVP1\_A1691 | PrkA serine kinase; K07180 serine protein kinase |  |
| bmv:BMASAVP1\_A1692 | A, transposase OrfB; K07497 putative transposase |  |
| bmv:BMASAVP1\_A1693 | transposase subfamily protein; K07497 putative transposase |  |
| bmv:BMASAVP1\_A1694 | transcriptional regulator CysB-like protein; K13635 LysR family transcriptional regulator, cys regulon transcriptional activator |  |
| bmv:BMASAVP1\_A1695 | cysA; sulfate/thiosulfate ABC transporter ATP-binding protein (EC:3.6.3.25); K02045 sulfate transport system ATP-binding protein [EC:3.6.3.25] | ec:3.6.3.25 |
| bmv:BMASAVP1\_A1696 | cysW; sulfate/thiosulfate ABC transporter permease CysW; K02047 sulfate transport system permease protein |  |
| bmv:BMASAVP1\_A1697 | cysT; sulfate/thiosulfate ABC transporter permease CysT; K02046 sulfate transport system permease protein |  |
| bmv:BMASAVP1\_A1698 | sbp; sulfate/thiosulfate ABC transporter sulfate-binding protein; K02048 sulfate transport system substrate-binding protein |  |
| bmv:BMASAVP1\_A1699 | lexA; LexA repressor (EC:3.4.21.88); K01356 repressor LexA [EC:3.4.21.88] | ec:3.4.21.88 |
| bmv:BMASAVP1\_A1700 | hypothetical protein |  |
| bmv:BMASAVP1\_A1701 | hypothetical protein |  |
| bmv:BMASAVP1\_A1702 | universal stress protein |  |
| bmv:BMASAVP1\_A1703 | nodI; nodulation ABC transporter NodI; K09695 lipooligosaccharide transport system ATP-binding protein |  |
| bmv:BMASAVP1\_A1704 | nodJ; ABC transporter permease NodJ; K09694 lipooligosaccharide transport system permease protein |  |

  
**Neighborhood Representations for "bpd:BURPS668\_1851"**  

| ID | Annotation | EC number |
| --- | --- | --- |
| bpd:BURPS668\_1841 | universal stress family protein |  |
| bpd:BURPS668\_1842 | hypothetical protein |  |
| bpd:BURPS668\_1843 | hypothetical protein |  |
| bpd:BURPS668\_1844 | hypothetical protein |  |
| bpd:BURPS668\_1845 | lexA; LexA repressor (EC:3.4.21.88); K01356 repressor LexA [EC:3.4.21.88] | ec:3.4.21.88 |
| bpd:BURPS668\_1846 | hypothetical protein |  |
| bpd:BURPS668\_1847 | sulfate/thiosulfate ABC transporter periplasmic sulfate-binding protein; K02048 sulfate transport system substrate-binding protein |  |
| bpd:BURPS668\_1848 | cysT; sulfate/thiosulfate ABC transporter permease CysT; K02046 sulfate transport system permease protein |  |
| bpd:BURPS668\_1849 | cysW; sulfate/thiosulfate ABC transporter permease CysW; K02047 sulfate transport system permease protein |  |
| bpd:BURPS668\_1850 | cysA; sulfate/thiosulfate ABC transporter ATP-binding protein (EC:3.6.3.25); K02045 sulfate transport system ATP-binding protein [EC:3.6.3.25] | ec:3.6.3.25 |
| bpd:BURPS668\_1851 | transcriptional regulator CysB-like protein; K13635 LysR family transcriptional regulator, cys regulon transcriptional activator |  |
| bpd:BURPS668\_1852 | hypothetical protein |  |
| bpd:BURPS668\_1853 | ribose ABC transporter periplasmic ribose-binding protein; K10439 ribose transport system substrate-binding protein |  |
| bpd:BURPS668\_1854 | hypothetical protein |  |
| bpd:BURPS668\_1855 | ribose ABC transporter ATP-binding protein; K10441 ribose transport system ATP-binding protein [EC:3.6.3.17] | ec:3.6.3.17 |
| bpd:BURPS668\_1856 | ribose ABC transporter permease; K10440 ribose transport system permease protein |  |
| bpd:BURPS668\_1857 | rbsR; ribose operon repressor RbsR; K02529 LacI family transcriptional regulator |  |
| bpd:BURPS668\_1858 | rbsK; ribokinase (EC:2.7.1.15); K00852 ribokinase [EC:2.7.1.15] | ec:2.7.1.15 |
| bpd:BURPS668\_1859 | methyl-accepting chemotaxis protein; K05874 methyl-accepting chemotaxis protein I, serine sensor receptor |  |
| bpd:BURPS668\_1860 | hypothetical protein |  |
| bpd:BURPS668\_1861 | prkA; serine protein kinase (EC:2.7.11.1); K07180 serine protein kinase |  |

  
**Neighborhood Representations for "bpl:BURPS1106A\_1865"**  

| ID | Annotation | EC number |
| --- | --- | --- |
| bpl:BURPS1106A\_1855 | nodI; nodulation ABC transporter NodI; K09695 lipooligosaccharide transport system ATP-binding protein |  |
| bpl:BURPS1106A\_1856 | universal stress family protein |  |
| bpl:BURPS1106A\_1857 | hypothetical protein |  |
| bpl:BURPS1106A\_1858 | hypothetical protein |  |
| bpl:BURPS1106A\_1859 | lexA; LexA repressor (EC:3.4.21.88); K01356 repressor LexA [EC:3.4.21.88] | ec:3.4.21.88 |
| bpl:BURPS1106A\_1860 | hypothetical protein |  |
| bpl:BURPS1106A\_1861 | sulfate/thiosulfate ABC transporter periplasmic sulfate-binding protein; K02048 sulfate transport system substrate-binding protein |  |
| bpl:BURPS1106A\_1862 | cysT; sulfate/thiosulfate ABC transporter permease CysT; K02046 sulfate transport system permease protein |  |
| bpl:BURPS1106A\_1863 | cysW; sulfate/thiosulfate ABC transporter permease CysW; K02047 sulfate transport system permease protein |  |
| bpl:BURPS1106A\_1864 | cysA; sulfate/thiosulfate ABC transporter ATP-binding protein (EC:3.6.3.25); K02045 sulfate transport system ATP-binding protein [EC:3.6.3.25] | ec:3.6.3.25 |
| bpl:BURPS1106A\_1865 | transcriptional regulator CysB-like protein; K13635 LysR family transcriptional regulator, cys regulon transcriptional activator |  |
| bpl:BURPS1106A\_1866 | ribose ABC transporter periplasmic ribose-binding protein; K10439 ribose transport system substrate-binding protein |  |
| bpl:BURPS1106A\_1867 | hypothetical protein |  |
| bpl:BURPS1106A\_1868 | ribose ABC transporter ATP-binding protein; K10441 ribose transport system ATP-binding protein [EC:3.6.3.17] | ec:3.6.3.17 |
| bpl:BURPS1106A\_1869 | ribose ABC transporter permease; K10440 ribose transport system permease protein |  |
| bpl:BURPS1106A\_1870 | rbsR; ribose operon repressor RbsR; K02529 LacI family transcriptional regulator |  |
| bpl:BURPS1106A\_1871 | rbsK; ribokinase (EC:2.7.1.15); K00852 ribokinase [EC:2.7.1.15] | ec:2.7.1.15 |
| bpl:BURPS1106A\_1872 | methyl-accepting chemotaxis protein |  |
| bpl:BURPS1106A\_1873 | prkA; serine protein kinase (EC:2.7.11.1); K07180 serine protein kinase |  |
| bpl:BURPS1106A\_1874 | hypothetical protein; K09786 hypothetical protein |  |
| bpl:BURPS1106A\_1875 | SpoVR family protein |  |

  
**Neighborhood Representations for "bpm:BURPS1710b\_2018"**  

| ID | Annotation | EC number |
| --- | --- | --- |
| bpm:BURPS1710b\_2008 | nodI; nodulation ABC transporter NodI; K09695 lipooligosaccharide transport system ATP-binding protein |  |
| bpm:BURPS1710b\_2009 | phenol hydroxylase |  |
| bpm:BURPS1710b\_2010 | DNA binding protein |  |
| bpm:BURPS1710b\_2011 | hypothetical protein |  |
| bpm:BURPS1710b\_2012 | lexA; LexA repressor (EC:3.4.21.88); K01356 repressor LexA [EC:3.4.21.88] | ec:3.4.21.88 |
| bpm:BURPS1710b\_2013 | sulfate ABC transporter periplasmic sulfate-binding protein; K02048 sulfate transport system substrate-binding protein |  |
| bpm:BURPS1710b\_2014 | hypothetical protein |  |
| bpm:BURPS1710b\_2015 | cysT; sulfate ABC transporter permease CysT; K02046 sulfate transport system permease protein |  |
| bpm:BURPS1710b\_2016 | cysW; sulfate transport system permease; K02047 sulfate transport system permease protein |  |
| bpm:BURPS1710b\_2017 | cysA; sulfate ABC transporter ATP-binding protein (EC:3.6.3.25); K02045 sulfate transport system ATP-binding protein [EC:3.6.3.25] | ec:3.6.3.25 |
| bpm:BURPS1710b\_2018 | transcriptional regulator CysB-like protein; K13635 LysR family transcriptional regulator, cys regulon transcriptional activator |  |
| bpm:BURPS1710b\_2019 | rbsB-1; ribose ABC transporter periplasmic ribose-binding protein; K10439 ribose transport system substrate-binding protein |  |
| bpm:BURPS1710b\_2020 | ABC transporter ATP-binding protein; K10441 ribose transport system ATP-binding protein [EC:3.6.3.17] | ec:3.6.3.17 |
| bpm:BURPS1710b\_2021 | rbsC; ribose ABC transporter membrane protein; K10440 ribose transport system permease protein |  |
| bpm:BURPS1710b\_2022 | rbsR; ribose operon repressor RbsR; K02529 LacI family transcriptional regulator |  |
| bpm:BURPS1710b\_2023 | rbsK; ribokinase (EC:2.7.1.15); K00852 ribokinase [EC:2.7.1.15] | ec:2.7.1.15 |
| bpm:BURPS1710b\_2024 | cheD1; methyl-accepting chemotaxis protein I; K05874 methyl-accepting chemotaxis protein I, serine sensor receptor |  |
| bpm:BURPS1710b\_2025 | prkA; protein kinase (EC:2.7.11.1); K07180 serine protein kinase |  |
| bpm:BURPS1710b\_2026 | hypothetical protein; K09786 hypothetical protein |  |
| bpm:BURPS1710b\_2028 | hypothetical protein |  |
| bpm:BURPS1710b\_2029 | hypothetical protein |  |

  
**Neighborhood Representations for "bpr:GBP346\_A1888"**  

| ID | Annotation | EC number |
| --- | --- | --- |
| bpr:GBP346\_A1878 | universal stress family protein |  |
| bpr:GBP346\_A1879 | hypothetical protein |  |
| bpr:GBP346\_A1880 | hypothetical protein |  |
| bpr:GBP346\_A1881 | hypothetical protein |  |
| bpr:GBP346\_A1882 | lexA; LexA repressor (EC:3.4.21.88); K01356 repressor LexA [EC:3.4.21.88] | ec:3.4.21.88 |
| bpr:GBP346\_A1883 | hypothetical protein |  |
| bpr:GBP346\_A1884 | sulfate-binding protein (Sulfate starvation-induced protein2) (SSI2); K02048 sulfate transport system substrate-binding protein |  |
| bpr:GBP346\_A1885 | cysT; sulfate ABC transporter, permease protein CysT; K02046 sulfate transport system permease protein |  |
| bpr:GBP346\_A1886 | cysW; sulfate ABC transporter, permease protein CysW; K02047 sulfate transport system permease protein |  |
| bpr:GBP346\_A1887 | sulfate/thiosulfate import ATP-binding protein CysA (Sulfate-transporting ATPase) (EC:3.6.3.25); K02045 sulfate transport system ATP-binding protein [EC:3.6.3.25] | ec:3.6.3.25 |
| bpr:GBP346\_A1888 | transcriptional regulator CysB-like protein; K13635 LysR family transcriptional regulator, cys regulon transcriptional activator |  |
| bpr:GBP346\_A1889 | hypothetical protein |  |
| bpr:GBP346\_A1890 | binding protein component of ABC ribose transporter; K10439 ribose transport system substrate-binding protein |  |
| bpr:GBP346\_A1891 | hypothetical protein |  |
| bpr:GBP346\_A1892 | putative ribose ABC transporter, ATP-binding protein; K10441 ribose transport system ATP-binding protein [EC:3.6.3.17] | ec:3.6.3.17 |
| bpr:GBP346\_A1893 | membrane protein component of ABC ribose transporter; K10440 ribose transport system permease protein |  |
| bpr:GBP346\_A1894 | ribose operon repressor RbsR; K02529 LacI family transcriptional regulator |  |
| bpr:GBP346\_A1895 | rbsK\_1; ribokinase (EC:2.7.1.15); K00852 ribokinase [EC:2.7.1.15] | ec:2.7.1.15 |
| bpr:GBP346\_A1896 | methyl-accepting chemotaxis protein I; K05874 methyl-accepting chemotaxis protein I, serine sensor receptor |  |
| bpr:GBP346\_A1897 | hypothetical protein |  |
| bpr:GBP346\_A1898 | histidine kinase; K07180 serine protein kinase |  |

  
**Neighborhood Representations for "bps:BPSL1835"**  

| ID | Annotation | EC number |
| --- | --- | --- |
| bps:BPSL1825 | ABC transporter permease; K03761 MFS transporter, MHS family, alpha-ketoglutarate permease |  |
| bps:BPSL1826 | SpoVR family protein |  |
| bps:BPSL1827 | hypothetical protein; K09786 hypothetical protein |  |
| bps:BPSL1828 | hypothetical protein; K07180 serine protein kinase |  |
| bps:BPSL1829 | methyl-accepting chemotaxis protein; K05874 methyl-accepting chemotaxis protein I, serine sensor receptor |  |
| bps:BPSL1830 | ribokinase; K00852 ribokinase [EC:2.7.1.15] | ec:2.7.1.15 |
| bps:BPSL1831 | ribose operon repressor; K02529 LacI family transcriptional regulator |  |
| bps:BPSL1832 | ribose transport system, permease; K10440 ribose transport system permease protein |  |
| bps:BPSL1833 | ribose transport system, ATP-binding protein; K10441 ribose transport system ATP-binding protein [EC:3.6.3.17] | ec:3.6.3.17 |
| bps:BPSL1834 | ribose transport system, substrate-binding protein; K10439 ribose transport system substrate-binding protein |  |
| bps:BPSL1835 | cbl; transcriptional regulator CysB-like protein; K13635 LysR family transcriptional regulator, cys regulon transcriptional activator |  |
| bps:BPSL1836 | cysA; sulfate transport ATP-binding protein; K02045 sulfate transport system ATP-binding protein [EC:3.6.3.25] | ec:3.6.3.25 |
| bps:BPSL1837 | cysW; sulfate transport system permease; K02047 sulfate transport system permease protein |  |
| bps:BPSL1838 | cysT; sulfate transport system permease; K02046 sulfate transport system permease protein |  |
| bps:BPSL1839 | sbp; sulfate-binding protein; K02048 sulfate transport system substrate-binding protein |  |
| bps:BPSL1840 | lexA; LexA repressor (EC:3.4.21.88); K01356 repressor LexA [EC:3.4.21.88] | ec:3.4.21.88 |
| bps:BPSL1841 | hypothetical protein |  |
| bps:BPSL1842 | hypothetical protein |  |
| bps:BPSL1843 | stress-like protein |  |
| bps:BPSL1844 | nodulation ABC transporter NodI; K09695 lipooligosaccharide transport system ATP-binding protein |  |
| bps:BPSL1845 | ABC transporter membrane protein; K09694 lipooligosaccharide transport system permease protein |  |

  
**Neighborhood Representations for "bte:BTH\_I2476"**  

| ID | Annotation | EC number |
| --- | --- | --- |
| bte:BTH\_I2466 | alpha-ketoglutarate permease; K03761 MFS transporter, MHS family, alpha-ketoglutarate permease |  |
| bte:BTH\_I2467 | SpoVR family protein |  |
| bte:BTH\_I2468 | hypothetical protein; K09786 hypothetical protein |  |
| bte:BTH\_I2469 | protein kinase; K07180 serine protein kinase |  |
| bte:BTH\_I2470 | methyl-accepting chemotaxis protein; K05874 methyl-accepting chemotaxis protein I, serine sensor receptor |  |
| bte:BTH\_I2471 | rbsK; ribokinase (EC:2.7.1.15); K00852 ribokinase [EC:2.7.1.15] | ec:2.7.1.15 |
| bte:BTH\_I2472 | purR; ribose operon repressor RbsR; K02529 LacI family transcriptional regulator |  |
| bte:BTH\_I2473 | rbsC; ribose ABC transporter permease; K10440 ribose transport system permease protein |  |
| bte:BTH\_I2474 | D-xylose ABC transporter ATP-binding protein; K10441 ribose transport system ATP-binding protein [EC:3.6.3.17] | ec:3.6.3.17 |
| bte:BTH\_I2475 | binding protein component of ABC ribose transporter; K10439 ribose transport system substrate-binding protein |  |
| bte:BTH\_I2476 | transcriptional regulator CysB-like protein; K13635 LysR family transcriptional regulator, cys regulon transcriptional activator |  |
| bte:BTH\_I2477 | sulfate ABC transporter ATP-binding protein; K02045 sulfate transport system ATP-binding protein [EC:3.6.3.25] | ec:3.6.3.25 |
| bte:BTH\_I2478 | sulfate ABC transporter permease; K02047 sulfate transport system permease protein |  |
| bte:BTH\_I2479 | cysT; sulfate ABC transporter permease CysT; K02046 sulfate transport system permease protein |  |
| bte:BTH\_I2480 | sulfate ABC transporter periplasmic sulfate-binding protein; K02048 sulfate transport system substrate-binding protein |  |
| bte:BTH\_I2481 | lexA; LexA repressor (EC:3.4.21.88); K01356 repressor LexA [EC:3.4.21.88] | ec:3.4.21.88 |
| bte:BTH\_I2482 | hypothetical protein |  |
| bte:BTH\_I2483 | hypothetical protein |  |
| bte:BTH\_I2484 | universal stress protein |  |
| bte:BTH\_I2485 | nodI; nodulation ABC transporter NodI; K09695 lipooligosaccharide transport system ATP-binding protein |  |
| bte:BTH\_I2486 | ABC transporter permease NodJ; K09694 lipooligosaccharide transport system permease protein |  |

  
**Neighborhood Representations for "bgl:bglu\_1g18690"**  

| ID | Annotation | EC number |
| --- | --- | --- |
| bgl:bglu\_1g18590 | hypothetical protein |  |
| bgl:bglu\_1g18600 | major facilitator superfamily metabolite/H symporter; K03761 MFS transporter, MHS family, alpha-ketoglutarate permease |  |
| bgl:bglu\_1g18610 | SpoVR family protein |  |
| bgl:bglu\_1g18620 | hypothetical protein; K09786 hypothetical protein |  |
| bgl:bglu\_1g18630 | PrkA family serine protein kinase; K07180 serine protein kinase |  |
| bgl:bglu\_1g18640 | Ribokinase; K00852 ribokinase [EC:2.7.1.15] | ec:2.7.1.15 |
| bgl:bglu\_1g18650 | transcriptional regulators; K02529 LacI family transcriptional regulator |  |
| bgl:bglu\_1g18660 | Inner-membrane translocator; K10440 ribose transport system permease protein |  |
| bgl:bglu\_1g18670 | sugar ABC transporter ATPase; K10441 ribose transport system ATP-binding protein [EC:3.6.3.17] | ec:3.6.3.17 |
| bgl:bglu\_1g18680 | Periplasmic binding protein/LacI transcriptional regulator; K10439 ribose transport system substrate-binding protein |  |
| bgl:bglu\_1g18690 | transcriptional regulator CysB-like protein; K13635 LysR family transcriptional regulator, cys regulon transcriptional activator |  |
| bgl:bglu\_1g18700 | sulfate ABC transporter ATPase subunit; K02045 sulfate transport system ATP-binding protein [EC:3.6.3.25] | ec:3.6.3.25 |
| bgl:bglu\_1g18710 | sulfate ABC transporter inner membrane subunit CysW; K02047 sulfate transport system permease protein |  |
| bgl:bglu\_1g18720 | sulfate ABC transporter inner membrane subunit CysT; K02046 sulfate transport system permease protein |  |
| bgl:bglu\_1g18730 | Thiosulfate-binding protein; K02048 sulfate transport system substrate-binding protein |  |
| bgl:bglu\_1g18740 | LexA repressor; K01356 repressor LexA [EC:3.4.21.88] | ec:3.4.21.88 |
| bgl:bglu\_1g18750 | hypothetical protein |  |
| bgl:bglu\_1g18760 | hypothetical protein |  |
| bgl:bglu\_1g18770 | UspA domain-containing protein |  |
| bgl:bglu\_1g18780 | nodulation ABC transporter NodI; K09695 lipooligosaccharide transport system ATP-binding protein |  |
| bgl:bglu\_1g18790 | ABC-2 type transporter, NodJ family; K09694 lipooligosaccharide transport system permease protein |  |

  
**Neighborhood Representations for "brh:RBRH\_00145"**  

| ID | Annotation | EC number |
| --- | --- | --- |
| brh:RBRH\_00136 | hypothetical protein |  |
| brh:RBRH\_04151 | hypothetical protein |  |
| brh:RBRH\_00138 | GntR family transcriptional regulator (EC:2.6.1.-); K00375 GntR family transcriptional regulator / MocR family aminotransferase |  |
| brh:RBRH\_00139 | transposase |  |
| brh:RBRH\_00140 | sensory transduction protein kinase (EC:2.7.3.-) |  |
| brh:RBRH\_00141 | two-component response regulator |  |
| brh:RBRH\_00142 | sensory transduction protein kinase (EC:2.7.3.-) |  |
| brh:RBRH\_00143 | hypothetical protein |  |
| brh:RBRH\_04150 | hypothetical protein |  |
| brh:RBRH\_00144 | hypothetical protein |  |
| brh:RBRH\_00145 | transcriptional regulator cbl; K13635 LysR family transcriptional regulator, cys regulon transcriptional activator |  |
| brh:RBRH\_00146 | sulfate transport ATP-binding protein cysA; K02045 sulfate transport system ATP-binding protein [EC:3.6.3.25] | ec:3.6.3.25 |
| brh:RBRH\_00147 | sulfate transport system permease cysW; K02047 sulfate transport system permease protein |  |
| brh:RBRH\_04149 | sulfate transport system permease cysT; K02046 sulfate transport system permease protein |  |
| brh:RBRH\_00148 | sulfate-binding protein; K02048 sulfate transport system substrate-binding protein |  |
| brh:RBRH\_00149 | LexA repressor (EC:3.4.21.88); K01356 repressor LexA [EC:3.4.21.88] | ec:3.4.21.88 |
| brh:RBRH\_00150 | hypothetical protein |  |
| brh:RBRH\_00151 | hypothetical protein |  |
| brh:RBRH\_00153 | Universal stress protein family |  |
| brh:RBRH\_00154 | hypothetical protein |  |
| brh:RBRH\_00155 | hypothetical protein |  |

  
**Neighborhood Representations for "hdn:Hden\_2672"**  

| ID | Annotation | EC number |
| --- | --- | --- |
| hdn:Hden\_2662 | thiamine-phosphate pyrophosphorylase; K00788 thiamine-phosphate pyrophosphorylase [EC:2.5.1.3] | ec:2.5.1.3 |
| hdn:Hden\_2663 | thiazole biosynthesis protein; K03149 thiamine biosynthesis ThiG |  |
| hdn:Hden\_2664 | thiamine biosynthesis protein ThiS; K03154 sulfur carrier protein |  |
| hdn:Hden\_2665 | AMP-dependent synthetase and ligase; K05939 acyl-[acyl-carrier-protein]-phospholipid O-acyltransferase / long-chain-fatty-acid--[acyl-carrier-protein] ligase [EC:2.3.1.40 6.2.1.20] | ec:2.3.1.40 ec:6.2.1.20 |
| hdn:Hden\_2666 | FAD dependent oxidoreductase; K03153 glycine oxidase [EC:1.4.3.19] | ec:1.4.3.19 |
| hdn:Hden\_2667 | gamma-glutamyltransferase; K00681 gamma-glutamyltranspeptidase [EC:2.3.2.2] | ec:2.3.2.2 |
| hdn:Hden\_2668 | cupin |  |
| hdn:Hden\_2669 | ErfK/YbiS/YcfS/YnhG family protein |  |
| hdn:Hden\_2670 | lipoic acid synthetase (EC:2.8.1.8); K03644 lipoic acid synthetase [EC:2.8.1.8] | ec:2.8.1.8 |
| hdn:Hden\_2671 | hydroxypyruvate isomerase (EC:5.3.1.22); K01816 hydroxypyruvate isomerase [EC:5.3.1.22] | ec:5.3.1.22 |
| hdn:Hden\_2672 | LysR family transcriptional regulator; K13635 LysR family transcriptional regulator, cys regulon transcriptional activator |  |
| hdn:Hden\_2673 | sulfate ABC transporter ATPase; K02045 sulfate transport system ATP-binding protein [EC:3.6.3.25] | ec:3.6.3.25 |
| hdn:Hden\_2674 | sulfate ABC transporter inner membrane subunit CysW; K02047 sulfate transport system permease protein |  |
| hdn:Hden\_2675 | sulfate ABC transporter inner membrane subunit CysT; K02046 sulfate transport system permease protein |  |
| hdn:Hden\_2676 | XRE family transcriptional regulator |  |
| hdn:Hden\_2677 | hypothetical protein |  |
| hdn:Hden\_2678 | hypothetical protein; K07002 |  |
| hdn:Hden\_2679 | sulfate ABC transporter periplasmic sulfate-binding protein; K02048 sulfate transport system substrate-binding protein |  |
| hdn:Hden\_2680 | hypothetical protein; K09950 hypothetical protein |  |
| hdn:Hden\_2681 | hypothetical protein; K02004 putative ABC transport system permease protein |  |
| hdn:Hden\_2682 | ABC transporter; K02003 putative ABC transport system ATP-binding protein |  |

  
**Neighborhood Representations for "dar:Daro\_2129"**  

| ID | Annotation | EC number |
| --- | --- | --- |
| dar:Daro\_2119 | major facilitator transporter |  |
| dar:Daro\_2120 | pseudogene |  |
| dar:Daro\_2121 | hypothetical protein |  |
| dar:Daro\_2122 | hypothetical protein |  |
| dar:Daro\_2123 | UMUC-like DNA-repair protein; K02346 DNA polymerase IV [EC:2.7.7.7] | ec:2.7.7.7 |
| dar:Daro\_2124 | RNA polymerase sigma factor; K03088 RNA polymerase sigma-70 factor, ECF subfamily |  |
| dar:Daro\_2125 | hypothetical protein |  |
| dar:Daro\_2126 | cytochrome c, class I; K08738 cytochrome c |  |
| dar:Daro\_2127 | diguanylate cyclase/phosphodiesterase with PAS/PAC sensor(s) |  |
| dar:Daro\_2128 | phospholipase/carboxylesterase; K06999 phospholipase/carboxylesterase |  |
| dar:Daro\_2129 | transcriptional regulator CysB-like protein; K13635 LysR family transcriptional regulator, cys regulon transcriptional activator |  |
| dar:Daro\_2130 | pseudogene |  |
| dar:Daro\_2131 | hypothetical protein |  |
| dar:Daro\_2132 | formylmethionine deformylase; K01462 peptide deformylase [EC:3.5.1.88] | ec:3.5.1.88 |
| dar:Daro\_2133 | hypothetical protein; K07002 |  |
| dar:Daro\_2134 | thiosulphate-binding protein; K02048 sulfate transport system substrate-binding protein |  |
| dar:Daro\_2135 | ArsR family transcriptional regulator; K03892 ArsR family transcriptional regulator |  |
| dar:Daro\_2136 | hypothetical protein |  |
| dar:Daro\_2137 | cysteine desulfurase |  |
| dar:Daro\_2138 | cysteine synthase (EC:2.5.1.47); K01738 cysteine synthase A [EC:2.5.1.47] | ec:2.5.1.47 |
| dar:Daro\_2139 | rhodanese-like protein |  |

  
**Neighborhood Representations for "app:CAP2UW1\_0224"**  

| ID | Annotation | EC number |
| --- | --- | --- |
| app:CAP2UW1\_0214 | metH; B12-dependent methionine synthase; K00548 5-methyltetrahydrofolate--homocysteine methyltransferase [EC:2.1.1.13] | ec:2.1.1.13 |
| app:CAP2UW1\_0215 | diguanylate cyclase |  |
| app:CAP2UW1\_0216 | multiple antibiotic resistance (MarC)-like protein |  |
| app:CAP2UW1\_0217 | coenzyme A transferase; K01026 propionate CoA-transferase [EC:2.8.3.1] | ec:2.8.3.1 |
| app:CAP2UW1\_0218 | Sporulation domain-containing protein |  |
| app:CAP2UW1\_0219 | hypothetical protein |  |
| app:CAP2UW1\_0220 | sulfate ABC transporter periplasmic sulfate-binding protein; K02048 sulfate transport system substrate-binding protein |  |
| app:CAP2UW1\_0221 | sulfate ABC transporter inner membrane subunit CysT; K02046 sulfate transport system permease protein |  |
| app:CAP2UW1\_0222 | sulfate ABC transporter inner membrane subunit CysW; K02047 sulfate transport system permease protein |  |
| app:CAP2UW1\_0223 | sulfate ABC transporter ATPase subunit; K02045 sulfate transport system ATP-binding protein [EC:3.6.3.25] | ec:3.6.3.25 |
| app:CAP2UW1\_0224 | transcriptional regulator CysB-like protein; K13635 LysR family transcriptional regulator, cys regulon transcriptional activator |  |
| app:CAP2UW1\_0225 | hypothetical protein; K13651 motility quorum-sensing regulator / GCU-specific mRNA interferase toxin |  |
| app:CAP2UW1\_0226 | XRE family transcriptional regulator; K13655 HTH-type transcriptional regulator / antitoxin for MqsR toxin |  |
| app:CAP2UW1\_0227 | hypothetical protein |  |
| app:CAP2UW1\_0228 | acriflavin resistance protein |  |
| app:CAP2UW1\_0229 | RND family efflux transporter MFP subunit |  |
| app:CAP2UW1\_0230 | Gamma-glutamyltransferase (EC:2.3.2.2); K00681 gamma-glutamyltranspeptidase [EC:2.3.2.2] | ec:2.3.2.2 |
| app:CAP2UW1\_0231 | hypothetical protein |  |
| app:CAP2UW1\_0232 | pseudogene |  |
| app:CAP2UW1\_0233 | transposase IS4 family protein |  |
| app:CAP2UW1\_0234 | hypothetical protein |  |

  
**Neighborhood Representations for "hse:Hsero\_1658"**  

| ID | Annotation | EC number |
| --- | --- | --- |
| hse:Hsero\_1648 | ssuA; alkanesulfonates ABC transporter periplasmic protein; K15553 sulfonate transport system substrate-binding protein |  |
| hse:Hsero\_1649 | ssuD; alkanesulfonate monooxygenase (EC:1.14.14.5); K04091 alkanesulfonate monooxygenase [EC:1.14.14.5] | ec:1.14.14.5 |
| hse:Hsero\_1650 | ssuC; alkanesulfonates ABC transporter permease; K15554 sulfonate transport system permease protein |  |
| hse:Hsero\_1651 | ssuB; alkanesulfonates ABC transporter ATPase (EC:3.6.3.-); K15555 sulfonate transport system ATP-binding protein [EC:3.6.3.-] |  |
| hse:Hsero\_1652 | ssuF; molybdopterin-binding protein |  |
| hse:Hsero\_1653 | EAL domain-containing protein |  |
| hse:Hsero\_1654 | alpha/beta hydrolase fold esterase; K07002 |  |
| hse:Hsero\_1655 | cysU; sulfate ABC transporter permease; K02046 sulfate transport system permease protein |  |
| hse:Hsero\_1656 | cysW; sulfate ABC transporter permease; K02047 sulfate transport system permease protein |  |
| hse:Hsero\_1657 | cysA; sulfate/molybdate ABC transporter ATPase (EC:3.6.3.25); K02045 sulfate transport system ATP-binding protein [EC:3.6.3.25] | ec:3.6.3.25 |
| hse:Hsero\_1658 | cysB; cys regulon transcription regulator protein; K13635 LysR family transcriptional regulator, cys regulon transcriptional activator |  |
| hse:Hsero\_1659 | hypothetical protein |  |
| hse:Hsero\_1660 | hypothetical protein |  |
| hse:Hsero\_1661 | lipoprotein signal peptide protein |  |
| hse:Hsero\_1662 | livK; branched-chain amino acid ABC transporter periplasmic protein; K01999 branched-chain amino acid transport system substrate-binding protein |  |
| hse:Hsero\_1663 | livH; branched-chain amino acid ABC transporter permease; K01997 branched-chain amino acid transport system permease protein |  |
| hse:Hsero\_1664 | livM; branched-chain amino acid ABC transporter permease; K01998 branched-chain amino acid transport system permease protein |  |
| hse:Hsero\_1665 | livG; branched-chain amino acid ABC transporter ATPase; K01995 branched-chain amino acid transport system ATP-binding protein |  |
| hse:Hsero\_1666 | livF; branched-chain amino acid ABC transporter ATPase; K01996 branched-chain amino acid transport system ATP-binding protein |  |
| hse:Hsero\_1667 | hypothetical protein |  |
| hse:Hsero\_1668 | hypothetical protein |  |

  
**Neighborhood Representations for "neu:NE0572"**  

| ID | Annotation | EC number |
| --- | --- | --- |
| neu:NE0562 | integrase catalytic subunit |  |
| neu:NE0563 | pseudogene |  |
| neu:NE0564 | multicopper oxidase type 1 |  |
| neu:NE0565 | hypothetical protein |  |
| neu:NE0566 | hypothetical protein |  |
| neu:NE0567 | dfrA; dihydrofolate reductase (EC:1.5.1.3); K00287 dihydrofolate reductase [EC:1.5.1.3] | ec:1.5.1.3 |
| neu:NE0568 | thyA; thymidylate synthase (EC:2.1.1.45); K00560 thymidylate synthase [EC:2.1.1.45] | ec:2.1.1.45 |
| neu:NE0569 | cytosol aminopeptidase (EC:3.4.11.1); K01255 leucyl aminopeptidase [EC:3.4.11.1] | ec:3.4.11.1 |
| neu:NE0570 | lipase |  |
| neu:NE0571 | hypothetical protein |  |
| neu:NE0572 | cbl; transcriptional regulator CysB-like protein; K13635 LysR family transcriptional regulator, cys regulon transcriptional activator |  |
| neu:NE0573 | aminotransferase class-V; K11717 cysteine desulfurase / selenocysteine lyase [EC:2.8.1.7 4.4.1.16] | ec:2.8.1.7 ec:4.4.1.16 |
| neu:NE0574 | major membrane protein I |  |
| neu:NE0575 | hexapeptide repeat-containing transferase (EC:2.3.1.30); K00640 serine O-acetyltransferase [EC:2.3.1.30] | ec:2.3.1.30 |
| neu:NE0576 | cysA; sulfate transport ATP-binding ABC transporter protein CysA; K02045 sulfate transport system ATP-binding protein [EC:3.6.3.25] | ec:3.6.3.25 |
| neu:NE0577 | cysW; sulfate transport ABC transporter protein CysW; K02047 sulfate transport system permease protein |  |
| neu:NE0578 | cysU; sulfate transport ABC transporter protein CysU; K02046 sulfate transport system permease protein |  |
| neu:NE0579 | hypothetical protein |  |
| neu:NE0580 | hypothetical protein |  |
| neu:NE0581 | diverged CheY-domain-containing protein |  |
| neu:NE0582 | sbp1; sulfate-/thiosulfate-binding protein; K02048 sulfate transport system substrate-binding protein |  |

  
**Neighborhood Representations for "nmu:Nmul\_A1739"**  

| ID | Annotation | EC number |
| --- | --- | --- |
| nmu:Nmul\_A1729 | pseudogene |  |
| nmu:Nmul\_A1730 | hypothetical protein |  |
| nmu:Nmul\_A1731 | hypothetical protein |  |
| nmu:Nmul\_A1732 | group 1 glycosyl transferase |  |
| nmu:Nmul\_A1733 | hypothetical protein |  |
| nmu:Nmul\_A1734 | hypothetical protein |  |
| nmu:Nmul\_A1735 | hypothetical protein |  |
| nmu:Nmul\_A1736 | hypothetical protein |  |
| nmu:Nmul\_A1737 | glycine cleavage T protein (aminomethyl transferase); K06980 |  |
| nmu:Nmul\_A1738 | hypothetical protein |  |
| nmu:Nmul\_A1739 | transcriptional regulator CysB-like protein; K13635 LysR family transcriptional regulator, cys regulon transcriptional activator |  |
| nmu:Nmul\_A1740 | ABC transporter-like protein; K06147 ATP-binding cassette, subfamily B, bacterial |  |
| nmu:Nmul\_A1741 | hypothetical protein |  |
| nmu:Nmul\_A1742 | hypothetical protein |  |
| nmu:Nmul\_A1743 | L-aspartate oxidase (EC:1.4.3.16); K00278 L-aspartate oxidase [EC:1.4.3.16] | ec:1.4.3.16 |
| nmu:Nmul\_A1744 | cation diffusion facilitator family transporter; K16264 cobalt-zinc-cadmium efflux system protein |  |
| nmu:Nmul\_A1745 | hypothetical protein |  |
| nmu:Nmul\_A1746 | RNA polymerase sigma factor RpoE; K03088 RNA polymerase sigma-70 factor, ECF subfamily |  |
| nmu:Nmul\_A1747 | anti sigma-E protein RseA; K03597 sigma-E factor negative regulatory protein RseA |  |
| nmu:Nmul\_A1748 | sigma E regulatory protein, MucB/RseB; K03598 sigma-E factor negative regulatory protein RseB |  |
| nmu:Nmul\_A1749 | peptidase S1C, Do; K01362 [EC:3.4.21.-] |  |

  
**Neighborhood Representations for "azo:azo1332"**  

| ID | Annotation | EC number |
| --- | --- | --- |
| azo:azo1322 | hypothetical protein |  |
| azo:azo1323 | protein kinase |  |
| azo:azo1324 | GTP binding protein |  |
| azo:azo1325 | hypothetical protein |  |
| azo:azo1326 | hypothetical protein |  |
| azo:azo1327 | hflX2; GTP-binding protein HflX (EC:3.1.5.1); K03665 GTP-binding protein HflX |  |
| azo:azo1328 | hypothetical protein |  |
| azo:azo1329 | aapM; putative amino acid permease; K09971 general L-amino acid transport system permease protein |  |
| azo:azo1330 | aapQ; putative amino acid permease; K09970 general L-amino acid transport system permease protein |  |
| azo:azo1331 | aapJ; putative amino acid-binding protein; K09969 general L-amino acid transport system substrate-binding protein |  |
| azo:azo1332 | cbl; transcriptional regulator CysB-like protein; K13635 LysR family transcriptional regulator, cys regulon transcriptional activator |  |
| azo:azo1333 | cysA; putative sulfate transport ATP-binding protein (EC:3.6.3.25); K02045 sulfate transport system ATP-binding protein [EC:3.6.3.25] | ec:3.6.3.25 |
| azo:azo1334 | cysW; putative sulfate transport system permease; K02047 sulfate transport system permease protein |  |
| azo:azo1335 | cysT; sulfate transporter permease; K02046 sulfate transport system permease protein |  |
| azo:azo1336 | cysP; putative sulfate transport system substrate-binding protein; K02048 sulfate transport system substrate-binding protein |  |
| azo:azo1337 | peptidoglycan-binding protein |  |
| azo:azo1338 | short-chain dehydrogenase |  |
| azo:azo1339 | ccoI; putative cation-transporting ATPase (EC:3.6.3.-); K01533 Cu2+-exporting ATPase [EC:3.6.3.4] | ec:3.6.3.4 |
| azo:azo1340 | ccoS; cytochrome oxidase maturation protein |  |
| azo:azo1341 | ccoN; cbb3-type cytochrome c oxidase subunit I (EC:1.9.3.1); K00404 cytochrome c oxidase cbb3-type subunit I [EC:1.9.3.1] | ec:1.9.3.1 |
| azo:azo1342 | ccoO; cbb3-type cytochrome c oxidase subunit II; K00405 cytochrome c oxidase cbb3-type subunit II |  |

  
**Neighborhood Representations for "mpt:Mpe\_A0127"**  

| ID | Annotation | EC number |
| --- | --- | --- |
| mpt:Mpe\_A0117 | ssuB; ATPase; K15555 sulfonate transport system ATP-binding protein [EC:3.6.3.-] |  |
| mpt:Mpe\_A0118 | hypothetical protein |  |
| mpt:Mpe\_A0119 | molybdenum-pterin binding protein II |  |
| mpt:Mpe\_A0120 | alpha/beta hydrolase |  |
| mpt:Mpe\_A0121 | 1-Cys peroxiredoxin (EC:1.11.1.15) |  |
| mpt:Mpe\_A0122 | cysP; thiosulfate binding protein; K02048 sulfate transport system substrate-binding protein |  |
| mpt:Mpe\_A0123 | cysP; thiosulfate binding protein; K02048 sulfate transport system substrate-binding protein |  |
| mpt:Mpe\_A0124 | cysUT; ABC type transporter; K02046 sulfate transport system permease protein |  |
| mpt:Mpe\_A0125 | cysW; ABC type transporter; K02047 sulfate transport system permease protein |  |
| mpt:Mpe\_A0126 | cysA; ABC type ATPase (EC:3.6.3.25); K02045 sulfate transport system ATP-binding protein [EC:3.6.3.25] | ec:3.6.3.25 |
| mpt:Mpe\_A0127 | LysR family transcriptional regulator; K13635 LysR family transcriptional regulator, cys regulon transcriptional activator |  |
| mpt:Mpe\_A0128 | ATP-dependent DNA helicase rep protein (EC:3.6.1.-); K03656 ATP-dependent DNA helicase Rep [EC:3.6.4.12] | ec:3.6.4.12 |
| mpt:Mpe\_A0129 | malonate transporter; K07088 |  |
| mpt:Mpe\_A0130 | outer membrane porin protein |  |
| mpt:Mpe\_A0131 | glutamine transport system substrate-binding protein; K02030 polar amino acid transport system substrate-binding protein |  |
| mpt:Mpe\_A0132 | amino acid ABC transporter; K02029 polar amino acid transport system permease protein |  |
| mpt:Mpe\_A0133 | replication restart DNA helicase PriA; K04066 primosomal protein N' (replication factor Y) (superfamily II helicase) [EC:3.6.4.-] |  |
| mpt:Mpe\_A0134 | uroporphyrinogen decarboxylase (EC:4.1.1.37); K01599 uroporphyrinogen decarboxylase [EC:4.1.1.37] | ec:4.1.1.37 |
| mpt:Mpe\_A0135 | two component transcriptional regulator; K07663 two-component system, OmpR family, catabolic regulation response regulator CreB |  |
| mpt:Mpe\_A0136 | sensory histidine kinase CreC (EC:2.7.3.-); K07641 two-component system, OmpR family, sensor histidine kinase CreC [EC:2.7.13.3] | ec:2.7.13.3 |
| mpt:Mpe\_A0137 | hypothetical protein |  |

  
**Neighborhood Representations for "tmz:Tmz1t\_0621"**  

| ID | Annotation | EC number |
| --- | --- | --- |
| tmz:Tmz1t\_0611 | hypothetical protein |  |
| tmz:Tmz1t\_0612 | hypothetical protein |  |
| tmz:Tmz1t\_0613 | hypothetical protein; K08994 putative membrane protein |  |
| tmz:Tmz1t\_0614 | ribose-phosphate pyrophosphokinase (EC:2.7.6.1); K00948 ribose-phosphate pyrophosphokinase [EC:2.7.6.1] | ec:2.7.6.1 |
| tmz:Tmz1t\_0615 | sulfate transporter; K03321 sulfate permease, SulP family |  |
| tmz:Tmz1t\_0616 | hypothetical protein |  |
| tmz:Tmz1t\_0617 | hypothetical protein |  |
| tmz:Tmz1t\_0618 | major facilitator superfamily protein |  |
| tmz:Tmz1t\_0619 | hypothetical protein |  |
| tmz:Tmz1t\_0620 | tryptophanyl-tRNA synthetase (EC:6.1.1.2); K01867 tryptophanyl-tRNA synthetase [EC:6.1.1.2] | ec:6.1.1.2 |
| tmz:Tmz1t\_0621 | transcriptional regulator CysB-like protein; K13635 LysR family transcriptional regulator, cys regulon transcriptional activator |  |
| tmz:Tmz1t\_0622 | sulfate ABC transporter ATPase; K02045 sulfate transport system ATP-binding protein [EC:3.6.3.25] | ec:3.6.3.25 |
| tmz:Tmz1t\_0623 | sulfate ABC transporter inner membrane subunit CysW; K02047 sulfate transport system permease protein |  |
| tmz:Tmz1t\_0624 | sulfate ABC transporter inner membrane subunit CysT; K02046 sulfate transport system permease protein |  |
| tmz:Tmz1t\_0625 | hypothetical protein |  |
| tmz:Tmz1t\_0626 | cytochrome C class I |  |
| tmz:Tmz1t\_0627 | Flavocytochrome C sulfide dehydrogenase flavin-binding |  |
| tmz:Tmz1t\_0628 | sulfate ABC transporter periplasmic sulfate-binding protein; K02048 sulfate transport system substrate-binding protein |  |
| tmz:Tmz1t\_0629 | short-chain dehydrogenase/reductase SDR; K16066 malonic semialdehyde reductase [EC:1.1.1.-] |  |
| tmz:Tmz1t\_0630 | membrane carboxypeptidase |  |
| tmz:Tmz1t\_0631 | TrkA-C domain-containing protein |  |

  
**Neighborhood Representations for "aav:Aave\_3039"**  

| ID | Annotation | EC number |
| --- | --- | --- |
| aav:Aave\_3029 | pirin domain-containing protein |  |
| aav:Aave\_3030 | LysR family transcriptional regulator |  |
| aav:Aave\_3031 | FAD dependent oxidoreductase; K06955 |  |
| aav:Aave\_3032 | glutamate--tRNA ligase (EC:6.1.1.17); K01894 glutamyl-Q tRNA(Asp) synthetase [EC:6.1.1.-] |  |
| aav:Aave\_3033 | trmB; tRNA (guanine-N(7)-)-methyltransferase (EC:2.1.1.33); K03439 tRNA (guanine-N7-)-methyltransferase [EC:2.1.1.33] | ec:2.1.1.33 |
| aav:Aave\_3034 | diguanylate cyclase |  |
| aav:Aave\_3035 | alanyl-tRNA synthetase (EC:6.1.1.7); K01872 alanyl-tRNA synthetase [EC:6.1.1.7] | ec:6.1.1.7 |
| aav:Aave\_3036 | hypothetical protein |  |
| aav:Aave\_3037 | hypothetical protein |  |
| aav:Aave\_3038 | class I cytochrome c |  |
| aav:Aave\_3039 | LysR family transcriptional regulator; K13635 LysR family transcriptional regulator, cys regulon transcriptional activator |  |
| aav:Aave\_3040 | TOBE domain-containing protein |  |
| aav:Aave\_3041 | ABC transporter-like protein; K15555 sulfonate transport system ATP-binding protein [EC:3.6.3.-] |  |
| aav:Aave\_3042 | binding-protein-dependent transport system inner membrane protein; K15554 sulfonate transport system permease protein |  |
| aav:Aave\_3043 | glyoxalase/bleomycin resistance protein/dioxygenase |  |
| aav:Aave\_3044 | alkanesulfonate monooxygenase (EC:1.14.14.5); K04091 alkanesulfonate monooxygenase [EC:1.14.14.5] | ec:1.14.14.5 |
| aav:Aave\_3045 | aliphatic sulfonate ABC transporter periplasmic ligand-binding protein; K15553 sulfonate transport system substrate-binding protein |  |
| aav:Aave\_3046 | sulfate ABC transporter periplasmic sulfate-binding protein; K02048 sulfate transport system substrate-binding protein |  |
| aav:Aave\_3047 | hypothetical protein |  |
| aav:Aave\_3048 | hypothetical protein; K15461 tRNA 5-methylaminomethyl-2-thiouridine biosynthesis bifunctional protein [EC:2.1.1.61 1.5.-.-] | ec:2.1.1.61 |
| aav:Aave\_3049 | major facilitator superfamily transporter |  |

  
**Neighborhood Representations for "dac:Daci\_5610"**  

| ID | Annotation | EC number |
| --- | --- | --- |
| dac:Daci\_5600 | engB; ribosome biogenesis GTP-binding protein YsxC; K03978 GTP-binding protein |  |
| dac:Daci\_5601 | alpha/beta hydrolase fold protein |  |
| dac:Daci\_5602 | lipid A biosynthesis acyltransferase; K02517 lipid A biosynthesis lauroyl acyltransferase [EC:2.3.1.-] |  |
| dac:Daci\_5603 | lipid A biosynthesis acyltransferase; K02517 lipid A biosynthesis lauroyl acyltransferase [EC:2.3.1.-] |  |
| dac:Daci\_5604 | S-adenosylmethionine synthetase (EC:2.5.1.6); K00789 S-adenosylmethionine synthetase [EC:2.5.1.6] | ec:2.5.1.6 |
| dac:Daci\_5605 | hypothetical protein |  |
| dac:Daci\_5606 | nicotinate phosphoribosyltransferase (EC:2.4.2.11); K00763 nicotinate phosphoribosyltransferase [EC:6.3.4.21] | ec:6.3.4.21 |
| dac:Daci\_5607 | NAD+ synthetase (EC:6.3.5.1); K01950 NAD+ synthase (glutamine-hydrolysing) [EC:6.3.5.1] | ec:6.3.5.1 |
| dac:Daci\_5608 | cytidyltransferase-like protein; K13522 bifunctional NMN adenylyltransferase/nudix hydrolase [EC:2.7.7.1 3.6.1.-] | ec:2.7.7.1 |
| dac:Daci\_5609 | N-acetyltransferase GCN5 |  |
| dac:Daci\_5610 | LysR family transcriptional regulator; K13635 LysR family transcriptional regulator, cys regulon transcriptional activator |  |
| dac:Daci\_5611 | glyoxalase/bleomycin resistance protein/dioxygenase |  |
| dac:Daci\_5612 | binding-protein-dependent transport system inner membrane protein; K02072 D-methionine transport system permease protein |  |
| dac:Daci\_5613 | ABC transporter-like protein; K02071 D-methionine transport system ATP-binding protein |  |
| dac:Daci\_5614 | NLPA lipoprotein; K02073 D-methionine transport system substrate-binding protein |  |
| dac:Daci\_5615 | 2OG-Fe(II) oxygenase; K06892 |  |
| dac:Daci\_5616 | O-acetylhomoserine/O-acetylserine sulfhydrylase (EC:2.5.1.49) |  |
| dac:Daci\_5617 | nitrate/sulfonate/bicarbonate ABC transporter periplasmic protein; K02051 NitT/TauT family transport system substrate-binding protein |  |
| dac:Daci\_5618 | binding-protein-dependent transport system inner membrane protein; K02050 NitT/TauT family transport system permease protein |  |
| dac:Daci\_5619 | ABC transporter-like protein; K02049 NitT/TauT family transport system ATP-binding protein |  |
| dac:Daci\_5620 | cytochrome c, mono-and diheme variant-like protein |  |

  
**Neighborhood Representations for "cvi:CV\_1827"**  

| ID | Annotation | EC number |
| --- | --- | --- |
| cvi:CV\_1817 | phosphoglycolate phosphatase (EC:3.1.3.18); K01091 phosphoglycolate phosphatase [EC:3.1.3.18] | ec:3.1.3.18 |
| cvi:CV\_1818 | rluC; pseudouridine synthase (EC:4.2.1.70); K06179 23S rRNA pseudouridine955/2504/2580 synthase [EC:5.4.99.24] | ec:5.4.99.24 |
| cvi:CV\_1819 | hypothetical protein |  |
| cvi:CV\_1820 | ribonuclease E (EC:3.1.4.-); K08300 ribonuclease E [EC:3.1.26.12] | ec:3.1.26.12 |
| cvi:CV\_1821 | hypothetical protein |  |
| cvi:CV\_1822 | permease |  |
| cvi:CV\_1823 | transmembrane transporter protein |  |
| cvi:CV\_1824 | hypothetical protein |  |
| cvi:CV\_1825 | AraC family transcriptional regulator |  |
| cvi:CV\_1826 | FAD-dependent monooxygenase |  |
| cvi:CV\_1827 | cbl; transcriptional regulator CysB-like protein; K13635 LysR family transcriptional regulator, cys regulon transcriptional activator |  |
| cvi:CV\_1828 | cysA; sulfate transport ATP-binding ABC transporter protein; K02045 sulfate transport system ATP-binding protein [EC:3.6.3.25] | ec:3.6.3.25 |
| cvi:CV\_1829 | cysW; sulfate transport system permease CysW; K02047 sulfate transport system permease protein |  |
| cvi:CV\_1830 | cysU; sulfate transport system permease CysU; K02046 sulfate transport system permease protein |  |
| cvi:CV\_1831 | hypothetical protein |  |
| cvi:CV\_1832 | sbp; sulfate transport system sulfate-binding protein; K02048 sulfate transport system substrate-binding protein |  |
| cvi:CV\_1833 | hypothetical protein |  |
| cvi:CV\_1834 | hypothetical protein |  |
| cvi:CV\_1835 | hypothetical protein |  |
| cvi:CV\_1836 | hypothetical protein |  |
| cvi:CV\_1837 | TetR family transcriptional regulator |  |

  
**Neighborhood Representations for "lch:Lcho\_3810"**  

| ID | Annotation | EC number |
| --- | --- | --- |
| lch:Lcho\_3800 | CheW protein; K03408 purine-binding chemotaxis protein CheW |  |
| lch:Lcho\_3801 | chemotaxis-specific methylesterase (EC:3.1.1.61); K03412 two-component system, chemotaxis family, response regulator CheB [EC:3.1.1.61] | ec:3.1.1.61 |
| lch:Lcho\_3802 | hypothetical protein; K07112 |  |
| lch:Lcho\_3803 | hypothetical protein; K07112 |  |
| lch:Lcho\_3804 | sulfate ABC transporter substrate-binding protein; K02048 sulfate transport system substrate-binding protein |  |
| lch:Lcho\_3805 | sulfate ABC transporter permease; K02046 sulfate transport system permease protein |  |
| lch:Lcho\_3806 | sulfate ABC transporter permease; K02047 sulfate transport system permease protein |  |
| lch:Lcho\_3807 | sulfate ABC transporter ATPase; K02045 sulfate transport system ATP-binding protein [EC:3.6.3.25] | ec:3.6.3.25 |
| lch:Lcho\_3808 | protein PASTA domain-containing protein |  |
| lch:Lcho\_3809 | gamma-glutamyltransferase (EC:2.3.2.2); K00681 gamma-glutamyltranspeptidase [EC:2.3.2.2] | ec:2.3.2.2 |
| lch:Lcho\_3810 | LysR family transcriptional regulator; K13635 LysR family transcriptional regulator, cys regulon transcriptional activator |  |
| lch:Lcho\_3811 | hypothetical protein |  |
| lch:Lcho\_3812 | phosphoglycerate mutase; K15634 probable phosphoglycerate mutase [EC:5.4.2.12] | ec:5.4.2.12 |
| lch:Lcho\_3813 | preprotein translocase subunit SecB; K03071 preprotein translocase subunit SecB |  |
| lch:Lcho\_3814 | gpsA; NAD(P)H-dependent glycerol-3-phosphate dehydrogenase (EC:1.1.1.94); K00057 glycerol-3-phosphate dehydrogenase (NAD(P)+) [EC:1.1.1.94] | ec:1.1.1.94 |
| lch:Lcho\_3815 | RNA methyltransferase; K03216 tRNA (cytidine/uridine-2'-O-)-methyltransferase [EC:2.1.1.207] | ec:2.1.1.207 |
| lch:Lcho\_3816 | putative phosphoribosyl transferase |  |
| lch:Lcho\_3817 | biotin synthesis protein BioC; K02169 malonyl-CoA O-methyltransferase [EC:2.1.1.197] | ec:2.1.1.197 |
| lch:Lcho\_3818 | cytochrome c oxidase subunit II; K02275 cytochrome c oxidase subunit II [EC:1.9.3.1] | ec:1.9.3.1 |
| lch:Lcho\_3819 | cytochrome c oxidase subunit I (EC:1.9.3.1); K02274 cytochrome c oxidase subunit I [EC:1.9.3.1] | ec:1.9.3.1 |
| lch:Lcho\_3820 | hypothetical protein |  |

  
**Neighborhood Representations for "gca:Galf\_1924"**  

| ID | Annotation | EC number |
| --- | --- | --- |
| gca:Galf\_1914 | family 2 glycosyl transferase |  |
| gca:Galf\_1915 | FAD-dependent pyridine nucleotide-disulfide oxidoreductase; K00520 mercuric reductase [EC:1.16.1.1] | ec:1.16.1.1 |
| gca:Galf\_1916 | radical SAM domain-containing protein |  |
| gca:Galf\_1917 | pseudogene |  |
| gca:Galf\_1918 | pseudogene |  |
| gca:Galf\_1919 | sulfate ABC transporter, periplasmic sulfate-binding protein; K02048 sulfate transport system substrate-binding protein |  |
| gca:Galf\_1920 | diguanylate phosphodiesterase |  |
| gca:Galf\_1921 | sulfate ABC transporter, inner membrane subunit CysT; K02046 sulfate transport system permease protein |  |
| gca:Galf\_1922 | sulfate ABC transporter, inner membrane subunit CysW; K02047 sulfate transport system permease protein |  |
| gca:Galf\_1923 | sulfate ABC transporter ATPase; K02045 sulfate transport system ATP-binding protein [EC:3.6.3.25] | ec:3.6.3.25 |
| gca:Galf\_1924 | transcriptional regulator, LysR family; K13635 LysR family transcriptional regulator, cys regulon transcriptional activator |  |
| gca:Galf\_1925 | hypothetical protein |  |
| gca:Galf\_1926 | ECF subfamily RNA polymerase sigma-24 subunit; K03088 RNA polymerase sigma-70 factor, ECF subfamily |  |
| gca:Galf\_1927 | hypothetical protein |  |
| gca:Galf\_1928 | pyridine nucleotide-disulfide oxidoreductase family protein |  |
| gca:Galf\_1929 | methylthioadenosine phosphorylase; K00772 5'-methylthioadenosine phosphorylase [EC:2.4.2.28] | ec:2.4.2.28 |
| gca:Galf\_1930 | AraC family transcriptional regulator |  |
| gca:Galf\_1931 | Carboxymuconolactone decarboxylase |  |
| gca:Galf\_1932 | alkyl hydroperoxide reductase |  |
| gca:Galf\_1933 | hypothetical protein |  |
| gca:Galf\_1934 | hypothetical protein |  |

  
**Neighborhood Representations for "mms:mma\_0257"**  

| ID | Annotation | EC number |
| --- | --- | --- |
| mms:mma\_0247 | exbD2; biopolymer transport protein ExbD; K03559 biopolymer transport protein ExbD |  |
| mms:mma\_0248 | GAF domain-containing protein; K07170 GAF domain-containing protein |  |
| mms:mma\_0249 | hypothetical protein |  |
| mms:mma\_0250 | DszA family monooxygenase |  |
| mms:mma\_0251 | acyl-CoA dehydrogenase |  |
| mms:mma\_0252 | hypothetical protein |  |
| mms:mma\_0253 | multidrug ABC transporter ATPase and permease; K06147 ATP-binding cassette, subfamily B, bacterial |  |
| mms:mma\_0254 | multidrug ABC transporter ATPase and permease; K06147 ATP-binding cassette, subfamily B, bacterial |  |
| mms:mma\_0255 | hypothetical protein; K07112 |  |
| mms:mma\_0256 | sseA1; thiosulfate sulfurtransferase (EC:2.8.1.1); K01011 thiosulfate/3-mercaptopyruvate sulfurtransferase [EC:2.8.1.1 2.8.1.2] | ec:2.8.1.1 ec:2.8.1.2 |
| mms:mma\_0257 | cys regulon transcriptional activator; K13635 LysR family transcriptional regulator, cys regulon transcriptional activator |  |
| mms:mma\_0258 | nitrilotriacetate monooxygenase component A (EC:1.14.13.-) |  |
| mms:mma\_0259 | ABC transporter permease; K02034 peptide/nickel transport system permease protein |  |
| mms:mma\_0260 | ABC transporter ATP-binding protein; K02031 peptide/nickel transport system ATP-binding protein K02032 peptide/nickel transport system ATP-binding protein |  |
| mms:mma\_0261 | TonB-dependent receptor; K02014 iron complex outermembrane recepter protein |  |
| mms:mma\_0262 | TonB-dependent receptor; K02014 iron complex outermembrane recepter protein |  |
| mms:mma\_0263 | TonB-dependent receptor; K02014 iron complex outermembrane recepter protein |  |
| mms:mma\_0264 | ABC transporter periplasmic protein; K02035 peptide/nickel transport system substrate-binding protein |  |
| mms:mma\_0265 | ABC transporter permease; K02033 peptide/nickel transport system permease protein |  |
| mms:mma\_0266 | hypothetical protein |  |
| mms:mma\_0267 | hypothetical protein |  |

  
**Neighborhood Representations for "ctt:CtCNB1\_3069"**  

| ID | Annotation | EC number |
| --- | --- | --- |
| ctt:CtCNB1\_3059 | hypothetical protein |  |
| ctt:CtCNB1\_3060 | transcriptional regulator |  |
| ctt:CtCNB1\_3061 | NAD-dependent epimerase/dehydratase |  |
| ctt:CtCNB1\_3062 | hypothetical protein |  |
| ctt:CtCNB1\_3063 | hypothetical protein |  |
| ctt:CtCNB1\_3064 | methyl-accepting chemotaxis sensory transducer; K03776 aerotaxis receptor |  |
| ctt:CtCNB1\_3065 | AsnC family transcriptional regulator; K03719 Lrp/AsnC family transcriptional regulator, leucine-responsive regulatory protein |  |
| ctt:CtCNB1\_3066 | delta-1-pyrroline-5-carboxylate dehydrogenase; K00249 acyl-CoA dehydrogenase [EC:1.3.8.7] | ec:1.3.8.7 |
| ctt:CtCNB1\_3067 | delta-1-pyrroline-5-carboxylate dehydrogenase; K00249 acyl-CoA dehydrogenase [EC:1.3.8.7] | ec:1.3.8.7 |
| ctt:CtCNB1\_3068 | Molybdenum-pterin binding protein |  |
| ctt:CtCNB1\_3069 | LysR family transcriptional regulator; K13635 LysR family transcriptional regulator, cys regulon transcriptional activator |  |
| ctt:CtCNB1\_3070 | ABC transporter ATP-binding subunit; K15555 sulfonate transport system ATP-binding protein [EC:3.6.3.-] |  |
| ctt:CtCNB1\_3071 | ABC transporter; K15554 sulfonate transport system permease protein |  |
| ctt:CtCNB1\_3072 | luciferase-like protein |  |
| ctt:CtCNB1\_3073 | luciferase-like protein; K04091 alkanesulfonate monooxygenase [EC:1.14.14.5] | ec:1.14.14.5 |
| ctt:CtCNB1\_3074 | aliphatic sulfonates family ABC transporter; K15553 sulfonate transport system substrate-binding protein |  |
| ctt:CtCNB1\_3075 | aliphatic sulfonates family ABC transporter; K15553 sulfonate transport system substrate-binding protein |  |
| ctt:CtCNB1\_3076 | NADPH-dependent FMN reductase; K00299 FMN reductase [EC:1.5.1.38] | ec:1.5.1.38 |
| ctt:CtCNB1\_3077 | sulfate ABC transporter periplasmic; K02048 sulfate transport system substrate-binding protein |  |
| ctt:CtCNB1\_3078 | Fe(II) trafficking protein YggX |  |
| ctt:CtCNB1\_3079 | diguanylate cyclase |  |

  
**Neighborhood Representations for "mmw:Mmwyl1\_0963"**  

| ID | Annotation | EC number |
| --- | --- | --- |
| mmw:Mmwyl1\_0953 | urease accessory protein UreD; K03190 urease accessory protein |  |
| mmw:Mmwyl1\_0954 | ureA; urease subunit gamma (EC:3.5.1.5); K01430 urease subunit gamma [EC:3.5.1.5] | ec:3.5.1.5 |
| mmw:Mmwyl1\_0955 | urease subunit beta (EC:3.5.1.5); K01429 urease subunit beta [EC:3.5.1.5] | ec:3.5.1.5 |
| mmw:Mmwyl1\_0956 | ureC; urease subunit alpha (EC:3.5.1.5); K01428 urease subunit alpha [EC:3.5.1.5] | ec:3.5.1.5 |
| mmw:Mmwyl1\_0957 | urease accessory protein UreE; K03187 urease accessory protein |  |
| mmw:Mmwyl1\_0958 | urease accessory protein UreF; K03188 urease accessory protein |  |
| mmw:Mmwyl1\_0959 | urease accessory protein UreG; K03189 urease accessory protein |  |
| mmw:Mmwyl1\_0960 | HupE/UreJ protein; K03192 urease accessory protein |  |
| mmw:Mmwyl1\_0961 | integral membrane sensor hybrid histidine kinase |  |
| mmw:Mmwyl1\_0962 | response regulator receiver protein |  |
| mmw:Mmwyl1\_0963 | LysR family transcriptional regulator; K13635 LysR family transcriptional regulator, cys regulon transcriptional activator |  |
| mmw:Mmwyl1\_0964 | sulfate ABC transporter ATPase; K02045 sulfate transport system ATP-binding protein [EC:3.6.3.25] | ec:3.6.3.25 |
| mmw:Mmwyl1\_0965 | sulfate ABC transporter permease; K02047 sulfate transport system permease protein |  |
| mmw:Mmwyl1\_0966 | sulfate ABC transporter permease; K02046 sulfate transport system permease protein |  |
| mmw:Mmwyl1\_0967 | sulfate ABC transporter periplasmic sulfate-binding protein; K02048 sulfate transport system substrate-binding protein |  |
| mmw:Mmwyl1\_0968 | N-acetyltransferase GCN5 |  |
| mmw:Mmwyl1\_0969 | methyl-accepting chemotaxis sensory transducer; K03406 methyl-accepting chemotaxis protein |  |
| mmw:Mmwyl1\_0970 | dicarboxylate carrier MatC domain-containing protein |  |
| mmw:Mmwyl1\_0971 | amidohydrolase 2; K07046 |  |
| mmw:Mmwyl1\_0972 | GntR family transcriptional regulator; K03710 GntR family transcriptional regulator |  |
| mmw:Mmwyl1\_0973 | hypothetical protein |  |

  
**Neighborhood Representations for "slt:Slit\_1030"**  

| ID | Annotation | EC number |
| --- | --- | --- |
| slt:Slit\_1020 | hypothetical protein; K09913 hypothetical protein |  |
| slt:Slit\_1021 | hypothetical protein |  |
| slt:Slit\_1022 | argininosuccinate synthase (EC:6.3.4.5); K01940 argininosuccinate synthase [EC:6.3.4.5] | ec:6.3.4.5 |
| slt:Slit\_1023 | ornithine carbamoyltransferase (EC:2.1.3.3) |  |
| slt:Slit\_1024 | acetylornithine and succinylornithine aminotransferase; K00818 acetylornithine aminotransferase [EC:2.6.1.11] | ec:2.6.1.11 |
| slt:Slit\_1025 | ribosomal protein S20; K02968 small subunit ribosomal protein S20 |  |
| slt:Slit\_1026 | hypothetical protein |  |
| slt:Slit\_1027 | hypothetical protein |  |
| slt:Slit\_1028 | integral membrane protein MviN; K03980 virulence factor |  |
| slt:Slit\_1029 | diguanylate cyclase with PAS/PAC sensor |  |
| slt:Slit\_1030 | LysR family transcriptional regulator; K13634 LysR family transcriptional regulator, cys regulon transcriptional activator |  |
| slt:Slit\_1031 | hypothetical protein; K07090 |  |
| slt:Slit\_1032 | sulfite reductase (ferredoxin) (EC:1.8.7.1); K00381 sulfite reductase (NADPH) hemoprotein beta-component [EC:1.8.1.2] | ec:1.8.1.2 |
| slt:Slit\_1033 | adenylylsulfate reductase, thioredoxin dependent (EC:1.8.4.8); K00390 phosphoadenosine phosphosulfate reductase [EC:1.8.4.8] | ec:1.8.4.8 |
| slt:Slit\_1034 | sulfate adenylyltransferase, small subunit (EC:2.7.7.4); K00957 sulfate adenylyltransferase subunit 2 [EC:2.7.7.4] | ec:2.7.7.4 |
| slt:Slit\_1035 | sulfate adenylyltransferase, large subunit (EC:2.7.7.4); K00956 sulfate adenylyltransferase subunit 1 [EC:2.7.7.4] | ec:2.7.7.4 |
| slt:Slit\_1036 | cobalamin biosynthesis protein CbiB; K02227 adenosylcobinamide-phosphate synthase [EC:6.3.1.10] | ec:6.3.1.10 |
| slt:Slit\_1037 | 4-alpha-glucanotransferase (EC:2.4.1.25); K00705 4-alpha-glucanotransferase [EC:2.4.1.25] | ec:2.4.1.25 |
| slt:Slit\_1038 | hypothetical protein |  |
| slt:Slit\_1039 | ribosome small subunit-dependent GTPase A; K06949 ribosome biogenesis GTPase [EC:3.6.1.-] |  |
| slt:Slit\_1040 | transcriptional coactivator/pterin dehydratase; K01724 4a-hydroxytetrahydrobiopterin dehydratase [EC:4.2.1.96] | ec:4.2.1.96 |

  
**Neighborhood Representations for "har:HEAR0839"**  

| ID | Annotation | EC number |
| --- | --- | --- |
| har:HEAR0828 | selD; selenide, water dikinase (selenophosphate synthase) (EC:2.7.9.3); K01008 selenide, water dikinase [EC:2.7.9.3] | ec:2.7.9.3 |
| har:HEAR0829 | tRNA 2-selenouridine synthase; K06917 tRNA 2-selenouridine synthase [EC:2.9.1.-] |  |
| har:HEAR0830 | hypothetical protein |  |
| har:HEAR0831 | hypothetical protein |  |
| har:HEAR0832 | carboxynorspermide decarboxylase; K13747 carboxynorspermidine decarboxylase [EC:4.1.1.-] |  |
| har:HEAR0833 | carboxynorspermide hydratase |  |
| har:HEAR0834 | hypothetical protein |  |
| har:HEAR0836 | cysT; sulfate ABC transporter membrane protein; K02046 sulfate transport system permease protein |  |
| har:HEAR0837 | cysW; sulfate ABC transporter membrane protein; K02047 sulfate transport system permease protein |  |
| har:HEAR0838 | cysA; sulfate permease (EC:3.6.3.25); K02045 sulfate transport system ATP-binding protein [EC:3.6.3.25] | ec:3.6.3.25 |
| har:HEAR0839 | cbl; transcriptional regulator Cbl; K13635 LysR family transcriptional regulator, cys regulon transcriptional activator |  |
| har:HEAR0840 | sodC; superoxide dismutase (Cu-Zn) (EC:1.15.1.1); K04565 superoxide dismutase, Cu-Zn family [EC:1.15.1.1] | ec:1.15.1.1 |
| har:HEAR0841 | hypothetical protein |  |
| har:HEAR0842 | dut; deoxyuridine 5'-triphosphate nucleotidohydrolase (EC:3.6.1.23); K01520 dUTP pyrophosphatase [EC:3.6.1.23] | ec:3.6.1.23 |
| har:HEAR0843 | dfp; bifunctional 4'-phosphopantothenoylcysteine decarboxylase/phosphopantothenoylcysteine synthetase (EC:4.1.1.36 6.3.2.5); K13038 phosphopantothenoylcysteine decarboxylase / phosphopantothenate--cysteine ligase [EC:4.1.1.36 6.3.2.5] | ec:6.3.2.5 ec:4.1.1.36 |
| har:HEAR0844 | lspA; lipoprotein signal peptidase (EC:3.4.23.36); K03101 signal peptidase II [EC:3.4.23.36] | ec:3.4.23.36 |
| har:HEAR0845 | ileS; isoleucyl-tRNA synthetase (EC:6.1.1.5); K01870 isoleucyl-tRNA synthetase [EC:6.1.1.5] | ec:6.1.1.5 |
| har:HEAR0846 | ribF; bifunctional riboflavin kinase/FMN adenylyltransferase (EC:2.7.1.26 2.7.7.2); K11753 riboflavin kinase / FMN adenylyltransferase [EC:2.7.1.26 2.7.7.2] | ec:2.7.1.26 ec:2.7.7.2 |
| har:HEAR0847 | 16S ribosomal RNA methyltransferase RsmE; K09761 16S rRNA (uracil1498-N3)-methyltransferase [EC:2.1.1.193] | ec:2.1.1.193 |
| har:HEAR0848 | tkt; transketolase (EC:2.2.1.1); K00615 transketolase [EC:2.2.1.1] | ec:2.2.1.1 |
| har:HEAR0849 | acetyltransferase |  |

  
**Neighborhood Representations for "axy:AXYL\_03028"**  

| ID | Annotation | EC number |
| --- | --- | --- |
| axy:AXYL\_03018 | hypothetical protein |  |
| axy:AXYL\_03019 | hypothetical protein |  |
| axy:AXYL\_03020 | hypothetical protein |  |
| axy:AXYL\_03021 | hypothetical protein |  |
| axy:AXYL\_03022 | extracellular solute-binding protein family 3 protein 1; K02030 polar amino acid transport system substrate-binding protein |  |
| axy:AXYL\_03023 | amino ABC transporter permease, 3-TM region, His/Glu/Gln/Arg/opine family domain-containing protein 7; K02029 polar amino acid transport system permease protein |  |
| axy:AXYL\_03024 | amino ABC transporter permease, 3-TM region, His/Glu/Gln/Arg/opine family domain-containing protein 8; K02029 polar amino acid transport system permease protein |  |
| axy:AXYL\_03025 | ABC transporter (EC:3.6.3.-); K02028 polar amino acid transport system ATP-binding protein [EC:3.6.3.21] | ec:3.6.3.21 |
| axy:AXYL\_03026 | metC2; cystathionine beta-lyase (EC:4.4.1.8); K01760 cystathionine beta-lyase [EC:4.4.1.8] | ec:4.4.1.8 |
| axy:AXYL\_03027 | hypothetical protein |  |
| axy:AXYL\_03028 | cbl; transcriptional regulator; K13635 LysR family transcriptional regulator, cys regulon transcriptional activator |  |
| axy:AXYL\_03029 | rhodanese-like domain-containing protein 2 |  |
| axy:AXYL\_03030 | hypothetical protein |  |
| axy:AXYL\_03031 | hypothetical protein |  |
| axy:AXYL\_03032 | fixJ2; transcriptional regulator FixJ 2 |  |
| axy:AXYL\_03033 | His Kinase A phosphoacceptor domain-containing protein 7 (EC:2.7.13.3) |  |
| axy:AXYL\_03034 | hypothetical protein; K07168 CBS domain-containing membrane protein |  |
| axy:AXYL\_03035 | MarR family transcriptional regulator |  |
| axy:AXYL\_03036 | voltage gated chloride channel family protein 1 |  |
| axy:AXYL\_03037 | LysR family transcriptional regulator |  |
| axy:AXYL\_03038 | argC2; N-acetyl-gamma-glutamyl-phosphate reductase 2 (EC:1.2.1.38); K00145 N-acetyl-gamma-glutamyl-phosphate reductase [EC:1.2.1.38] | ec:1.2.1.38 |

  
**Neighborhood Representations for "rpf:Rpic12D\_1287"**  

| ID | Annotation | EC number |
| --- | --- | --- |
| rpf:Rpic12D\_1277 | aliphatic sulfonate ABC transporter substrate-binding protein; K15553 sulfonate transport system substrate-binding protein |  |
| rpf:Rpic12D\_1278 | alkanesulfonate monooxygenase (EC:1.14.14.5); K04091 alkanesulfonate monooxygenase [EC:1.14.14.5] | ec:1.14.14.5 |
| rpf:Rpic12D\_1279 | binding-protein-dependent transport system inner membrane protein; K15554 sulfonate transport system permease protein |  |
| rpf:Rpic12D\_1280 | ABC transporter; K15555 sulfonate transport system ATP-binding protein [EC:3.6.3.-] |  |
| rpf:Rpic12D\_1281 | TOBE domain-containing protein |  |
| rpf:Rpic12D\_1282 | diguanylate phosphodiesterase |  |
| rpf:Rpic12D\_1283 | hypothetical protein; K07002 |  |
| rpf:Rpic12D\_1284 | sulfate ABC transporter permease; K02046 sulfate transport system permease protein |  |
| rpf:Rpic12D\_1285 | sulfate ABC transporter permease; K02047 sulfate transport system permease protein |  |
| rpf:Rpic12D\_1286 | sulfate ABC transporter ATPase; K02045 sulfate transport system ATP-binding protein [EC:3.6.3.25] | ec:3.6.3.25 |
| rpf:Rpic12D\_1287 | CysB family transcriptional regulator; K13635 LysR family transcriptional regulator, cys regulon transcriptional activator |  |
| rpf:Rpic12D\_1288 | hypothetical protein |  |
| rpf:Rpic12D\_1289 | histidine kinase |  |
| rpf:Rpic12D\_1290 | LytTR family two component transcriptional regulator |  |
| rpf:Rpic12D\_1291 | thiamine pyrophosphate protein; K01652 acetolactate synthase I/II/III large subunit [EC:2.2.1.6] | ec:2.2.1.6 |
| rpf:Rpic12D\_1292 | ribonuclease activity regulator protein RraA; K02553 regulator of ribonuclease activity A |  |
| rpf:Rpic12D\_1293 | AIG2 family protein |  |
| rpf:Rpic12D\_1294 | hypothetical protein |  |
| rpf:Rpic12D\_1295 | isocitrate lyase; K01637 isocitrate lyase [EC:4.1.3.1] | ec:4.1.3.1 |
| rpf:Rpic12D\_1296 | GntR family transcriptional regulator |  |
| rpf:Rpic12D\_1297 | Asp/Glu/hydantoin racemase; K16841 allantoin racemase [EC:5.1.99.3] | ec:5.1.99.3 |

  
**Neighborhood Representations for "alv:Alvin\_2440"**  

| ID | Annotation | EC number |
| --- | --- | --- |
| alv:Alvin\_2430 | NADH-ubiquinone/plastoquinone oxidoreductase chain 3; K00330 NADH-quinone oxidoreductase subunit A [EC:1.6.5.3] | ec:1.6.5.3 |
| alv:Alvin\_2431 | preprotein translocase subunit SecG; K03075 preprotein translocase subunit SecG |  |
| alv:Alvin\_2432 | triosephosphate isomerase; K01803 triosephosphate isomerase (TIM) [EC:5.3.1.1] | ec:5.3.1.1 |
| alv:Alvin\_2433 | SsrA-binding protein; K03664 SsrA-binding protein |  |
| alv:Alvin\_2434 | hypothetical protein |  |
| alv:Alvin\_2435 | hypothetical protein |  |
| alv:Alvin\_2436 | hypothetical protein |  |
| alv:Alvin\_2437 | hypothetical protein |  |
| alv:Alvin\_2438 | HAD superfamily ATPase |  |
| alv:Alvin\_2439 | UBA/THIF-type NAD/FAD binding protein |  |
| alv:Alvin\_2440 | LysR family transcriptional regulator; K13635 LysR family transcriptional regulator, cys regulon transcriptional activator |  |
| alv:Alvin\_2441 | sulfate ABC transporter ATPase subunit; K02045 sulfate transport system ATP-binding protein [EC:3.6.3.25] | ec:3.6.3.25 |
| alv:Alvin\_2442 | sulfate ABC transporter inner membrane subunit CysW; K02047 sulfate transport system permease protein |  |
| alv:Alvin\_2443 | sulfate ABC transporter inner membrane subunit CysT; K02046 sulfate transport system permease protein |  |
| alv:Alvin\_2444 | sulfate ABC transporter periplasmic sulfate-binding protein; K02048 sulfate transport system substrate-binding protein |  |
| alv:Alvin\_2445 | LysR family transcriptional regulator; K13634 LysR family transcriptional regulator, cys regulon transcriptional activator |  |
| alv:Alvin\_2446 | nitrite and sulfite reductase 4Fe-4S region; K00381 sulfite reductase (NADPH) hemoprotein beta-component [EC:1.8.1.2] | ec:1.8.1.2 |
| alv:Alvin\_2447 | adenylylsulfate reductase, thioredoxin dependent (EC:1.8.4.8); K00390 phosphoadenosine phosphosulfate reductase [EC:1.8.4.8] | ec:1.8.4.8 |
| alv:Alvin\_2448 | sulfate adenylyltransferase small subunit (EC:2.7.7.4); K00957 sulfate adenylyltransferase subunit 2 [EC:2.7.7.4] | ec:2.7.7.4 |
| alv:Alvin\_2449 | sulfate adenylyltransferase large subunit (EC:2.7.7.4); K00956 sulfate adenylyltransferase subunit 1 [EC:2.7.7.4] | ec:2.7.7.4 |
| alv:Alvin\_2450 | selenide, water dikinase |  |

  
**Neighborhood Representations for "rsc:RCFBP\_20081"**  

| ID | Annotation | EC number |
| --- | --- | --- |
| rsc:RCFBP\_20069 | LysR family transcriptional regulator |  |
| rsc:RCFBP\_20070 | uspA; universal stress protein |  |
| rsc:RCFBP\_20071 | aceA; isocitrate lyase (EC:4.1.3.1); K01637 isocitrate lyase [EC:4.1.3.1] | ec:4.1.3.1 |
| rsc:RCFBP\_20072 | leucine-rich-repeat type III effector protein (gala7) |  |
| rsc:RCFBP\_20073 | leucine-rich-repeat type III effector protein (gala6) |  |
| rsc:RCFBP\_20075 | hypothetical protein |  |
| rsc:RCFBP\_20076 | rraA; ribonuclease E inhibitor; K02553 regulator of ribonuclease activity A |  |
| rsc:RCFBP\_20077 | ilvG; acetolactate synthase (EC:2.2.1.6); K01652 acetolactate synthase I/II/III large subunit [EC:2.2.1.6] | ec:2.2.1.6 |
| rsc:RCFBP\_20078 | hypothetical protein |  |
| rsc:RCFBP\_20079 | type III effector protein, ssph1 family |  |
| rsc:RCFBP\_20081 | cbl; LysR family transcriptional regulator; K13635 LysR family transcriptional regulator, cys regulon transcriptional activator |  |
| rsc:RCFBP\_20082 | cysA; thiosulfate ABC transporter ATP-binding protein (EC:3.6.3.25); K02045 sulfate transport system ATP-binding protein [EC:3.6.3.25] | ec:3.6.3.25 |
| rsc:RCFBP\_20083 | cysW; thiosulfate ABC transporter membrane component; K02047 sulfate transport system permease protein |  |
| rsc:RCFBP\_20084 | cysT; thiosulfate ABC transporter membrane component; K02046 sulfate transport system permease protein |  |
| rsc:RCFBP\_20085 | hypothetical protein; K07002 |  |
| rsc:RCFBP\_20086 | signalling protein eal (eal) |  |
| rsc:RCFBP\_20087 | ssuF; organosulfonate utilization protein ssuf; molybdopterin-binding protein |  |
| rsc:RCFBP\_20088 | ssuE; pseudogene |  |
| rsc:RCFBP\_20089 | cysP; thiosulfate ABC transporter substrate-binding protein; K02048 sulfate transport system substrate-binding protein |  |
| rsc:RCFBP\_20090 | hypothetical protein |  |
| rsc:RCFBP\_20091 | clpB; chaperone; K03695 ATP-dependent Clp protease ATP-binding subunit ClpB |  |

  
**Neighborhood Representations for "rpi:Rpic\_1224"**  

| ID | Annotation | EC number |
| --- | --- | --- |
| rpi:Rpic\_1214 | aliphatic sulfonates family ABC transporter periplsmic ligand-binding protein; K15553 sulfonate transport system substrate-binding protein |  |
| rpi:Rpic\_1215 | alkanesulfonate monooxygenase (EC:1.14.14.5); K04091 alkanesulfonate monooxygenase [EC:1.14.14.5] | ec:1.14.14.5 |
| rpi:Rpic\_1216 | binding-protein-dependent transport system inner membrane protein; K15554 sulfonate transport system permease protein |  |
| rpi:Rpic\_1217 | ABC transporter-like protein; K15555 sulfonate transport system ATP-binding protein [EC:3.6.3.-] |  |
| rpi:Rpic\_1218 | TOBE domain-containing protein |  |
| rpi:Rpic\_1219 | diguanylate phosphodiesterase |  |
| rpi:Rpic\_1220 | hypothetical protein; K07002 |  |
| rpi:Rpic\_1221 | sulfate ABC transporter permease; K02046 sulfate transport system permease protein |  |
| rpi:Rpic\_1222 | sulfate ABC transporter permease; K02047 sulfate transport system permease protein |  |
| rpi:Rpic\_1223 | sulfate ABC transporter ATPase; K02045 sulfate transport system ATP-binding protein [EC:3.6.3.25] | ec:3.6.3.25 |
| rpi:Rpic\_1224 | transcriptional regulator CysB-like protein; K13635 LysR family transcriptional regulator, cys regulon transcriptional activator |  |
| rpi:Rpic\_1225 | hypothetical protein |  |
| rpi:Rpic\_1226 | integral membrane sensor signal transduction histidine kinase |  |
| rpi:Rpic\_1227 | LytTR family two component transcriptional regulator |  |
| rpi:Rpic\_1228 | thiamine pyrophosphate protein; K01652 acetolactate synthase I/II/III large subunit [EC:2.2.1.6] | ec:2.2.1.6 |
| rpi:Rpic\_1229 | ribonuclease activity regulator protein RraA; K02553 regulator of ribonuclease activity A |  |
| rpi:Rpic\_1230 | AIG2 family protein |  |
| rpi:Rpic\_1231 | hypothetical protein |  |
| rpi:Rpic\_1232 | isocitrate lyase; K01637 isocitrate lyase [EC:4.1.3.1] | ec:4.1.3.1 |
| rpi:Rpic\_1233 | UspA domain-containing protein |  |
| rpi:Rpic\_1234 | LysR family transcriptional regulator |  |

  
**Neighborhood Representations for "rso:RSc1348"**  

| ID | Annotation | EC number |
| --- | --- | --- |
| rso:RSc1338 | ssuA1; alkanesulfonates binding signal peptide protein; K15553 sulfonate transport system substrate-binding protein |  |
| rso:RSc1339 | ssuD; alkanesulfonate monooxygenase (EC:1.14.14.5); K04091 alkanesulfonate monooxygenase [EC:1.14.14.5] | ec:1.14.14.5 |
| rso:RSc1340 | ssuC; aliphatic sulfonate ABC transporter transmembrane protein; K15554 sulfonate transport system permease protein |  |
| rso:RSc1341 | ssuB; aliphatic sulfonate ABC transporter ATP-binding protein; K15555 sulfonate transport system ATP-binding protein [EC:3.6.3.-] |  |
| rso:RSc1342 | ssuF; molybdopterin-binding protein |  |
| rso:RSc1343 | hypothetical protein |  |
| rso:RSc1344 | hypothetical protein; K07002 |  |
| rso:RSc1345 | cysU; sulfate transport ABC transporter protein; K02046 sulfate transport system permease protein |  |
| rso:RSc1346 | cysW; sulfate transport ABC transporter protein; K02047 sulfate transport system permease protein |  |
| rso:RSc1347 | cysA; sulfate transport ATP-binding ABC transporter protein; K02045 sulfate transport system ATP-binding protein [EC:3.6.3.25] | ec:3.6.3.25 |
| rso:RSc1348 | cysB1; CysB family transcriptional regulator; K13635 LysR family transcriptional regulator, cys regulon transcriptional activator |  |
| rso:RSc1349 | hypothetical protein |  |
| rso:RSc1350 | hypothetical protein |  |
| rso:RSc1351 | transmembrane sensor histidine kinase transcription regulator protein (EC:2.7.3.-) |  |
| rso:RSc1352 | two-component response regulator transcription regulator protein |  |
| rso:RSc1353 | ilvG; thiamine pyrophosphate protein (EC:2.2.1.6); K01652 acetolactate synthase I/II/III large subunit [EC:2.2.1.6] | ec:2.2.1.6 |
| rso:RSc1354 | menG; ribonuclease activity regulator protein RraA (EC:2.1.1.-); K02553 regulator of ribonuclease activity A |  |
| rso:RSc1355 | hypothetical protein |  |
| rso:RSc1356 | GALA protein 4 |  |
| rso:RSc1357 | GALA protein 5 |  |
| rso:RSc1358 | aceA; isocitrate lyase (EC:4.1.3.1); K01637 isocitrate lyase [EC:4.1.3.1] | ec:4.1.3.1 |

  
**Neighborhood Representations for "rsl:RPSI07\_2019"**  

| ID | Annotation | EC number |
| --- | --- | --- |
| rsl:RPSI07\_2009 | type III effector protein |  |
| rsl:RPSI07\_2010 | type III effector protein |  |
| rsl:RPSI07\_2011 | hypothetical protein |  |
| rsl:RPSI07\_2012 | rraA; ribonuclease E (RNase E) inhibitor protein; K02553 regulator of ribonuclease activity A |  |
| rsl:RPSI07\_2013 | ilvG; acetolactate synthase 2 catalytic subunit (EC:2.2.1.6); K01652 acetolactate synthase I/II/III large subunit [EC:2.2.1.6] | ec:2.2.1.6 |
| rsl:RPSI07\_2014 | response regulator, lytTr family |  |
| rsl:RPSI07\_2015 | sensor histidine kinase (EC:2.7.13.3) |  |
| rsl:RPSI07\_2016 | hypothetical protein |  |
| rsl:RPSI07\_2017 | type III effector protein, SspH1 family |  |
| rsl:RPSI07\_2018 | hypothetical protein |  |
| rsl:RPSI07\_2019 | cbl; cysteine biosynthesis and aliphatic sulfonates utilization LysR family transcriptional regulator; K13635 LysR family transcriptional regulator, cys regulon transcriptional activator |  |
| rsl:RPSI07\_2020 | cysA; thiosulfate transport protein; ABC transporter ATP-binding protein (EC:3.6.3.25); K02045 sulfate transport system ATP-binding protein [EC:3.6.3.25] | ec:3.6.3.25 |
| rsl:RPSI07\_2021 | cysW; thiosulfate ABC transporter permease; K02047 sulfate transport system permease protein |  |
| rsl:RPSI07\_2022 | cysT; thiosulfate ABC transporter permease; K02046 sulfate transport system permease protein |  |
| rsl:RPSI07\_2023 | hypothetical protein; K07002 |  |
| rsl:RPSI07\_2024 | signalling protein EAL (eal) |  |
| rsl:RPSI07\_2025 | ssuF; organosulfonate utilization protein SsuF; molybdopterin-binding protein |  |
| rsl:RPSI07\_2026 | ssuB; aliphatic sulfonate ABC transporter ATP-binding protein (EC:3.6.3.-); K15555 sulfonate transport system ATP-binding protein [EC:3.6.3.-] |  |
| rsl:RPSI07\_2027 | ssuC; alkanesulfonate ABC transporter; K15554 sulfonate transport system permease protein |  |
| rsl:RPSI07\_2028 | ssuD; alkanesulfonate monooxygenase (EC:1.14.14.5); K04091 alkanesulfonate monooxygenase [EC:1.14.14.5] | ec:1.14.14.5 |
| rsl:RPSI07\_2029 | ssuA; alkanesulfonates binding signal peptide protein; K15553 sulfonate transport system substrate-binding protein |  |

  
**Neighborhood Representations for "pna:Pnap\_0071"**  

| ID | Annotation | EC number |
| --- | --- | --- |
| pna:Pnap\_0061 | prevent-host-death family protein |  |
| pna:Pnap\_0062 | hypothetical protein |  |
| pna:Pnap\_0063 | gidA; tRNA uridine 5-carboxymethylaminomethyl modification protein GidA; K03495 tRNA uridine 5-carboxymethylaminomethyl modification enzyme |  |
| pna:Pnap\_0064 | gidB; 16S rRNA methyltransferase GidB; K03501 16S rRNA (guanine527-N7)-methyltransferase [EC:2.1.1.170] | ec:2.1.1.170 |
| pna:Pnap\_0065 | lysine exporter protein LysE/YggA |  |
| pna:Pnap\_0066 | cobyrinic acid a,c-diamide synthase; K03496 chromosome partitioning protein |  |
| pna:Pnap\_0067 | hypothetical protein; K07002 |  |
| pna:Pnap\_0068 | parB-like partition proteins; K03497 chromosome partitioning protein, ParB family |  |
| pna:Pnap\_0069 | FAD linked oxidase domain-containing protein; K00102 D-lactate dehydrogenase (cytochrome) [EC:1.1.2.4] | ec:1.1.2.4 |
| pna:Pnap\_0070 | ATP--cobalamin adenosyltransferase; K00798 cob(I)alamin adenosyltransferase [EC:2.5.1.17] | ec:2.5.1.17 |
| pna:Pnap\_0071 | LysR family transcriptional regulator; K13635 LysR family transcriptional regulator, cys regulon transcriptional activator |  |
| pna:Pnap\_0072 | hypothetical protein |  |
| pna:Pnap\_0073 | Acetyl-CoA hydrolase (EC:3.1.2.1); K01067 acetyl-CoA hydrolase [EC:3.1.2.1] | ec:3.1.2.1 |
| pna:Pnap\_0074 | hypothetical protein |  |
| pna:Pnap\_0075 | hypothetical protein |  |
| pna:Pnap\_0076 | uracil-xanthine permease |  |
| pna:Pnap\_0077 | twitching motility protein; K02670 twitching motility protein PilU |  |
| pna:Pnap\_0078 | putative transposase, IS891/IS1136/IS1341 |  |
| pna:Pnap\_0079 | IS605 family transposase OrfB |  |
| pna:Pnap\_0080 | RND family efflux transporter MFP subunit; K02005 HlyD family secretion protein |  |
| pna:Pnap\_0081 | hypothetical protein; K09808 lipoprotein-releasing system permease protein |  |

  
**Neighborhood Representations for "vap:Vapar\_0061"**  

| ID | Annotation | EC number |
| --- | --- | --- |
| vap:Vapar\_0051 | peptidoglycan-binding domain 1 protein |  |
| vap:Vapar\_0052 | FAD linked oxidase; K00102 D-lactate dehydrogenase (cytochrome) [EC:1.1.2.4] | ec:1.1.2.4 |
| vap:Vapar\_0053 | hypothetical protein |  |
| vap:Vapar\_0054 | ATP/cobalamin adenosyltransferase; K00798 cob(I)alamin adenosyltransferase [EC:2.5.1.17] | ec:2.5.1.17 |
| vap:Vapar\_0055 | hypothetical protein |  |
| vap:Vapar\_0056 | adenylate cyclase |  |
| vap:Vapar\_0057 | G-D-S-L family lipolytic protein |  |
| vap:Vapar\_0058 | hypothetical protein |  |
| vap:Vapar\_0059 | hypothetical protein |  |
| vap:Vapar\_0060 | hypothetical protein |  |
| vap:Vapar\_0061 | LysR family transcriptional regulator; K13635 LysR family transcriptional regulator, cys regulon transcriptional activator |  |
| vap:Vapar\_0062 | TetR family transcriptional regulator |  |
| vap:Vapar\_0063 | hypothetical protein |  |
| vap:Vapar\_0064 | hypothetical protein |  |
| vap:Vapar\_0065 | cell division topological specificity factor MinE; K03608 cell division topological specificity factor |  |
| vap:Vapar\_0066 | septum site-determining protein MinD; K03609 septum site-determining protein MinD |  |
| vap:Vapar\_0067 | septum site-determining protein MinC; K03610 septum site-determining protein MinC |  |
| vap:Vapar\_0068 | uracil-xanthine permease |  |
| vap:Vapar\_0069 | thioesterase superfamily protein |  |
| vap:Vapar\_0070 | hypothetical protein |  |
| vap:Vapar\_0071 | feruloyl-CoA synthase; K12508 feruloyl-CoA synthase [EC:6.2.1.34] | ec:6.2.1.34 |

  
**Neighborhood Representations for "reh:H16\_A2236"**  

| ID | Annotation | EC number |
| --- | --- | --- |
| reh:H16\_A2226 | vgrG; hypothetical protein |  |
| reh:H16\_A2227 | iclB; isocitrate lyase (EC:4.1.3.1); K01637 isocitrate lyase [EC:4.1.3.1] | ec:4.1.3.1 |
| reh:H16\_A2228 | h16\_A2228; hydrolase or acyltransferase (EC:3.-.-.-) |  |
| reh:H16\_A2229 | h16\_A2229; hypothetical protein |  |
| reh:H16\_A2230 | menG1; ribonuclease activity regulator protein RraA; K02553 regulator of ribonuclease activity A |  |
| reh:H16\_A2231 | h16\_A2231; thiamine pyrophosphate protein (EC:2.2.1.6); K01652 acetolactate synthase I/II/III large subunit [EC:2.2.1.6] | ec:2.2.1.6 |
| reh:H16\_A2232 | h16\_A2232; multi drug efflux transporter |  |
| reh:H16\_A2233 | lytT; response regulator |  |
| reh:H16\_A2234 | lytS; signal transduction histidine kinase (EC:2.7.3.-); K00936 [EC:2.7.3.-] |  |
| reh:H16\_A2235 | h16\_A2235; hypothetical protein |  |
| reh:H16\_A2236 | cysB; transcriptional regulator CysB-like protein; K13635 LysR family transcriptional regulator, cys regulon transcriptional activator |  |
| reh:H16\_A2237 | cysA; ABC transporter ATPase (EC:3.6.3.25); K02045 sulfate transport system ATP-binding protein [EC:3.6.3.25] | ec:3.6.3.25 |
| reh:H16\_A2238 | cysW; ABC transporter permease; K02047 sulfate transport system permease protein |  |
| reh:H16\_A2239 | cysT; ABC transporter permease; K02046 sulfate transport system permease protein |  |
| reh:H16\_A2240 | h16\_A2240; esterase; K07002 |  |
| reh:H16\_A2241 | eal; signalling protein EAL |  |
| reh:H16\_A2242 | ssuB1; ABC transporter ATPase (EC:3.6.3.-); K15555 sulfonate transport system ATP-binding protein [EC:3.6.3.-] |  |
| reh:H16\_A2243 | ssuC1; ABC transporter permease; K15554 sulfonate transport system permease protein |  |
| reh:H16\_A2244 | ssuD1; alkanesulfonate monooxygenase (EC:1.14.14.5); K04091 alkanesulfonate monooxygenase [EC:1.14.14.5] | ec:1.14.14.5 |
| reh:H16\_A2245 | ssuA1; ABC transporter periplasmic protein; K15553 sulfonate transport system substrate-binding protein |  |
| reh:H16\_A2246 | ssuE; flavoprotein; K00299 FMN reductase [EC:1.5.1.38] | ec:1.5.1.38 |

  
**Neighborhood Representations for "rme:Rmet\_1379"**  

| ID | Annotation | EC number |
| --- | --- | --- |
| rme:Rmet\_1369 | cysP; sulfate ABC transporter periplasmic-binding protein; K02048 sulfate transport system substrate-binding protein |  |
| rme:Rmet\_1370 | ssuA; alkanesulfonate ABC transporter periplasmic-binding protein; K15553 sulfonate transport system substrate-binding protein |  |
| rme:Rmet\_1371 | ssuD; alkanesulfonate monooxygenase (EC:1.14.14.5); K04091 alkanesulfonate monooxygenase [EC:1.14.14.5] | ec:1.14.14.5 |
| rme:Rmet\_1372 | ssuC; alkanesulfonate ABC transporter membrane protein; K15554 sulfonate transport system permease protein |  |
| rme:Rmet\_1373 | ssuB; alkanesulfonate ABC transporter ATP-binding protein; K15555 sulfonate transport system ATP-binding protein [EC:3.6.3.-] |  |
| rme:Rmet\_1374 | eal; putative signalling protein EAL |  |
| rme:Rmet\_1375 | putative alpha/beta hydrolase fold family esterase; K07002 |  |
| rme:Rmet\_1376 | cysU; sulfate/thiosulfate ABC transporter membrane protein; K02046 sulfate transport system permease protein |  |
| rme:Rmet\_1377 | cysW; sulfate/thiosulfate ABC transporter membrane protein; K02047 sulfate transport system permease protein |  |
| rme:Rmet\_1378 | cysA; sulfate/thiosulfate ABC transporter ATP-binding proteinsulfate/thiosulfate ABC transporter ATP-binding protein (EC:3.6.3.25); K02045 sulfate transport system ATP-binding protein [EC:3.6.3.25] | ec:3.6.3.25 |
| rme:Rmet\_1379 | cysB; transcriptional regulator CysB-like protein (EC:1.5.1.12); K13635 LysR family transcriptional regulator, cys regulon transcriptional activator |  |
| rme:Rmet\_1380 | signal transduction histidine kinase (EC:2.7.3.-) |  |
| rme:Rmet\_1381 | lytT; response regulator containing a LytTR DNA-binding domain |  |
| rme:Rmet\_1382 | rraA; ribonuclease activity regulator protein RraA; K02553 regulator of ribonuclease activity A |  |
| rme:Rmet\_1383 | ytfP; hypothetical protein |  |
| rme:Rmet\_1384 | putative hydrolase (EC:3.-.-.-) |  |
| rme:Rmet\_1385 | aceA; isocitrate lyase (EC:4.1.3.1); K01637 isocitrate lyase [EC:4.1.3.1] | ec:4.1.3.1 |
| rme:Rmet\_1386 | hypothetical protein |  |
| rme:Rmet\_1387 | uspA3; universal stress protein, UspA family |  |
| rme:Rmet\_1388 | lysR; transcriptional regulator, LysR-family |  |
| rme:Rmet\_1389 | dehII; putative hydrolase; K01560 2-haloacid dehalogenase [EC:3.8.1.2] | ec:3.8.1.2 |

  
**Neighborhood Representations for "reu:Reut\_A1967"**  

| ID | Annotation | EC number |
| --- | --- | --- |
| reu:Reut\_A1957 | methyl-accepting chemotaxis sensory transducer |  |
| reu:Reut\_A1958 | sensor histidine kinase |  |
| reu:Reut\_A1959 | hypothetical protein |  |
| reu:Reut\_A1960 | peptidase M48, Ste24p; K07387 putative metalloprotease [EC:3.4.24.-] |  |
| reu:Reut\_A1961 | isocitrate lyase (EC:4.1.3.1); K01637 isocitrate lyase [EC:4.1.3.1] | ec:4.1.3.1 |
| reu:Reut\_A1962 | hypothetical protein |  |
| reu:Reut\_A1963 | ribonuclease activity regulator protein RraA; K02553 regulator of ribonuclease activity A |  |
| reu:Reut\_A1964 | major facilitator transporter |  |
| reu:Reut\_A1965 | response regulator receiver:LytTr DNA-binding region |  |
| reu:Reut\_A1966 | sensor histidine kinase |  |
| reu:Reut\_A1967 | transcriptional regulator CysB-like protein; K13635 LysR family transcriptional regulator, cys regulon transcriptional activator |  |
| reu:Reut\_A1968 | sulfate transport system permease 1; K02045 sulfate transport system ATP-binding protein [EC:3.6.3.25] | ec:3.6.3.25 |
| reu:Reut\_A1969 | sulfate ABC transporter permease CysW; K02047 sulfate transport system permease protein |  |
| reu:Reut\_A1970 | sulfate ABC transporter permease CysT; K02046 sulfate transport system permease protein |  |
| reu:Reut\_A1971 | hypothetical protein; K07002 |  |
| reu:Reut\_A1972 | hypothetical protein |  |
| reu:Reut\_A1973 | molybdenum-pterin-binding protein |  |
| reu:Reut\_A1974 | ABC transporter; K15555 sulfonate transport system ATP-binding protein [EC:3.6.3.-] |  |
| reu:Reut\_A1975 | binding-protein dependent transport system inner membrane protein; K15554 sulfonate transport system permease protein |  |
| reu:Reut\_A1976 | alkanesulfonate monooxygenase (EC:1.14.14.5); K04091 alkanesulfonate monooxygenase [EC:1.14.14.5] | ec:1.14.14.5 |
| reu:Reut\_A1977 | aliphatic sulfonates ABC transporter substrate-binding protein; K15553 sulfonate transport system substrate-binding protein |  |

  
**Neighborhood Representations for "tau:Tola\_0017"**  

| ID | Annotation | EC number |
| --- | --- | --- |
| tau:Tola\_0007 | avtA; valine--pyruvate transaminase; K00835 valine--pyruvate aminotransferase [EC:2.6.1.66] | ec:2.6.1.66 |
| tau:Tola\_0008 | glycyl-tRNA synthetase subunit beta (EC:6.1.1.14); K01879 glycyl-tRNA synthetase beta chain [EC:6.1.1.14] | ec:6.1.1.14 |
| tau:Tola\_0009 | glyQ; glycyl-tRNA synthetase subunit alpha (EC:6.1.1.14); K01878 glycyl-tRNA synthetase alpha chain [EC:6.1.1.14] | ec:6.1.1.14 |
| tau:Tola\_0010 | DNA-3-methyladenine glycosylase I (EC:3.2.2.20); K01246 DNA-3-methyladenine glycosylase I [EC:3.2.2.20] | ec:3.2.2.20 |
| tau:Tola\_0011 | zntB; zinc transporter; K16074 zinc transporter |  |
| tau:Tola\_0012 | sulfate ABC transporter, periplasmic sulfate-binding protein; K02048 sulfate transport system substrate-binding protein |  |
| tau:Tola\_0013 | hypothetical protein; K07002 |  |
| tau:Tola\_0014 | sulfate ABC transporter, inner membrane subunit CysT; K02046 sulfate transport system permease protein |  |
| tau:Tola\_0015 | sulfate ABC transporter, inner membrane subunit CysW; K02047 sulfate transport system permease protein |  |
| tau:Tola\_0016 | sulfate ABC transporter ATPase; K02045 sulfate transport system ATP-binding protein [EC:3.6.3.25] | ec:3.6.3.25 |
| tau:Tola\_0017 | cbl; transcriptional regulator Cbl; K13635 LysR family transcriptional regulator, cys regulon transcriptional activator |  |
| tau:Tola\_0018 | SirA family protein; K04085 tRNA 2-thiouridine synthesizing protein A [EC:2.8.1.-] |  |
| tau:Tola\_0019 | extracellular solute-binding protein; K17315 glucose/mannose transport system substrate-binding protein |  |
| tau:Tola\_0020 | histidine kinase |  |
| tau:Tola\_0021 | two component transcriptional regulator, winged helix family |  |
| tau:Tola\_0022 | Protoporphyrinogen oxidase (EC:1.3.3.4); K00230 menaquinone-dependent protoporphyrinogen oxidase [EC:1.3.5.3] | ec:1.3.5.3 |
| tau:Tola\_0023 | hypothetical protein |  |
| tau:Tola\_0024 | integrase catalytic subunit |  |
| tau:Tola\_0025 | transposase IS3/IS911 family protein |  |
| tau:Tola\_0026 | group 1 glycosyl transferase |  |
| tau:Tola\_0027 | group 1 glycosyl transferase |  |

  
**Neighborhood Representations for "cti:RALTA\_A1775"**  

| ID | Annotation | EC number |
| --- | --- | --- |
| cti:RALTA\_A1765 | peptidase, lipoprotein; K07387 putative metalloprotease [EC:3.4.24.-] |  |
| cti:RALTA\_A1766 | aceA1; isocitrate lyase (EC:4.1.3.1); K01637 isocitrate lyase [EC:4.1.3.1] | ec:4.1.3.1 |
| cti:RALTA\_A1767 | oxidoreductase; alpha/beta hydrolase fold |  |
| cti:RALTA\_A1768 | hypothetical protein |  |
| cti:RALTA\_A1769 | rraA; ribonuclease activity regulator protein rraa; K02553 regulator of ribonuclease activity A |  |
| cti:RALTA\_A1770 | ilvG1; thiamine pyrophosphate protein (EC:2.2.1.6); K01652 acetolactate synthase I/II/III large subunit [EC:2.2.1.6] | ec:2.2.1.6 |
| cti:RALTA\_A1771 | transporter major facilitator superfamily mfs\_1 |  |
| cti:RALTA\_A1772 | response regulator; lyttr family |  |
| cti:RALTA\_A1773 | senson histidine kinase |  |
| cti:RALTA\_A1774 | hypothetical protein |  |
| cti:RALTA\_A1775 | cbl; CysB family transcriptional regulator; K13635 LysR family transcriptional regulator, cys regulon transcriptional activator |  |
| cti:RALTA\_A1776 | cysA; thiosulfate ABC transporter ATP-binding protein; K02045 sulfate transport system ATP-binding protein [EC:3.6.3.25] | ec:3.6.3.25 |
| cti:RALTA\_A1777 | cysW; thiosulfate transporter; ABC superfamily, membrane component; K02047 sulfate transport system permease protein |  |
| cti:RALTA\_A1778 | cysT; thiosulfate transporter; ABC superfamily, membrane component; K02046 sulfate transport system permease protein |  |
| cti:RALTA\_A1779 | hypothetical protein; K07002 |  |
| cti:RALTA\_A1780 | hypothetical protein |  |
| cti:RALTA\_A1781 | ssuB; aliphatic sulfonates transporter ATP-binding protein; K15555 sulfonate transport system ATP-binding protein [EC:3.6.3.-] |  |
| cti:RALTA\_A1782 | ssuC; alkanesulfonate transporter; ABC superfamily, membrane component; K15554 sulfonate transport system permease protein |  |
| cti:RALTA\_A1783 | ssuD; alkanesulfonate monooxygenase (EC:1.14.14.5); K04091 alkanesulfonate monooxygenase [EC:1.14.14.5] | ec:1.14.14.5 |
| cti:RALTA\_A1784 | ssuA1; alkanesulfonate transporter; ABC superfamily, substrate binding unit; K15553 sulfonate transport system substrate-binding protein |  |
| cti:RALTA\_A1785 | ssuE; NAD(P)h-dependent fmn reductase, sulfate starvation-induced protein (EC:1.5.1.29); K00299 FMN reductase [EC:1.5.1.38] | ec:1.5.1.38 |

  
**Neighborhood Representations for "ddd:Dda3937\_04117"**  

| ID | Annotation | EC number |
| --- | --- | --- |
| ddd:Dda3937\_00735 | hyfI; hydrogenase 4, Fe-S subunit; K12144 hydrogenase-4 component I [EC:1.-.-.-] |  |
| ddd:Dda3937\_00734 | hyfH; hydrogenase 4, Fe-S subunit; K12143 hydrogenase-4 component H |  |
| ddd:Dda3937\_00733 | hyfG; hydrogenase 4 subunit; K12142 hydrogenase-4 component G [EC:1.-.-.-] |  |
| ddd:Dda3937\_00732 | hyfF; hydrogenase 4, membrane subunit; K12141 hydrogenase-4 component F [EC:1.-.-.-] |  |
| ddd:Dda3937\_00731 | hyfE; hydrogenase 4, membrane subunit; K12140 hydrogenase-4 component E [EC:1.-.-.-] |  |
| ddd:Dda3937\_00730 | hyfD; hydrogenase 4, membrane subunit; K12139 hydrogenase-4 component D [EC:1.-.-.-] |  |
| ddd:Dda3937\_00729 | hyfC; hydrogenase 4, membrane subunit; K12138 hydrogenase-4 component C [EC:1.-.-.-] |  |
| ddd:Dda3937\_00728 | hyfB; hydrogenase 4, membrane subunit; K12137 hydrogenase-4 component B [EC:1.-.-.-] |  |
| ddd:Dda3937\_00727 | hyfA; hydrogenase 4, 4Fe-4S subunit; K12136 hydrogenase-4 component A [EC:1.-.-.-] |  |
| ddd:Dda3937\_04116 | [NiFe] hydrogenase metallocenter assembly protein HybG; K04653 hydrogenase expression/formation protein HypC |  |
| ddd:Dda3937\_04117 | cbl; LysR family transcriptional regulator; K13635 LysR family transcriptional regulator, cys regulon transcriptional activator |  |
| ddd:Dda3937\_04118 | gltP; proton glutamate symport protein; K11102 proton glutamate symport protein |  |
| ddd:Dda3937\_04119 | ydcF; protein ydcF |  |
| ddd:Dda3937\_04120 | ygjR; NAD-dependent oxidoreductase |  |
| ddd:Dda3937\_04121 | yghU; glutathionylspermidine-utilizing glutathione transferase; K11209 GST-like protein |  |
| ddd:Dda3937\_04122 | glycerol dehydrogenase |  |
| ddd:Dda3937\_04123 | Taurine transport system permease tauC; K02050 NitT/TauT family transport system permease protein |  |
| ddd:Dda3937\_04124 | Taurine transport ATP-binding protein tauB; K02049 NitT/TauT family transport system ATP-binding protein |  |
| ddd:Dda3937\_04315 | Taurine transporter substrate-binding protein; K02051 NitT/TauT family transport system substrate-binding protein |  |
| ddd:Dda3937\_02692 | malate/lactate dehydrogenase; K13574 uncharacterized oxidoreductase [EC:1.1.1.-] |  |
| ddd:Dda3937\_02691 | dcyD; D-cysteine desulfhydrase; K17950 L-cysteate sulfo-lyase [EC:4.4.1.25] | ec:4.4.1.25 |

  
**Neighborhood Representations for "asa:ASA\_0598"**  

| ID | Annotation | EC number |
| --- | --- | --- |
| asa:ASA\_0588 | hypothetical protein |  |
| asa:ASA\_0589 | hypothetical protein |  |
| asa:ASA\_0590 | hypothetical protein |  |
| asa:ASA\_0591 | maa; maltose O-acetyltransferase; K00661 maltose O-acetyltransferase [EC:2.3.1.79] | ec:2.3.1.79 |
| asa:ASA\_0592 | hypothetical protein |  |
| asa:ASA\_0593 | cysP; ABC-type sulfate transport system, periplasmic component; K02048 sulfate transport system substrate-binding protein |  |
| asa:ASA\_0594 | hypothetical protein; K07002 |  |
| asa:ASA\_0595 | cysU; ABC-type sulfate transporter permease component; K02046 sulfate transport system permease protein |  |
| asa:ASA\_0596 | cysW; ABC-type sulfate transporter permease component; K02047 sulfate transport system permease protein |  |
| asa:ASA\_0597 | cysA; ABC-type sulfate transporter ATPase component; K02045 sulfate transport system ATP-binding protein [EC:3.6.3.25] | ec:3.6.3.25 |
| asa:ASA\_0598 | transcriptional regulator CysB-like protein; K13635 LysR family transcriptional regulator, cys regulon transcriptional activator |  |
| asa:ASA\_0599 | thrC; threonine synthase (EC:4.2.3.1); K01733 threonine synthase [EC:4.2.3.1] | ec:4.2.3.1 |
| asa:ASA\_0600 | ABC-type glutamine/glutamate transporter permease protein; K02029 polar amino acid transport system permease protein |  |
| asa:ASA\_0601 | glnP; ABC-type glutamine/glutamate transporter permease protein; K02029 polar amino acid transport system permease protein |  |
| asa:ASA\_0602 | glnQ; ABC-type glutamine/glutamate transporter ATP-binding protein; K02028 polar amino acid transport system ATP-binding protein [EC:3.6.3.21] | ec:3.6.3.21 |
| asa:ASA\_0603 | glnH; ABC-type glutamine/glutamate transporter periplasmic binding protein; K02030 polar amino acid transport system substrate-binding protein |  |
| asa:ASA\_0604 | N-acetylglucosamine-binding protein A; K03933 chitin-binding protein |  |
| asa:ASA\_0605 | methyl-accepting chemotaxis protein; K03406 methyl-accepting chemotaxis protein |  |
| asa:ASA\_0606 | oligoendopeptidase F |  |
| asa:ASA\_0607 | hypothetical protein |  |
| asa:ASA\_0608 | hypothetical protein |  |

  
**Neighborhood Representations for "aha:AHA\_0609"**  

| ID | Annotation | EC number |
| --- | --- | --- |
| aha:AHA\_0599 | hypothetical protein |  |
| aha:AHA\_0600 | hypothetical protein |  |
| aha:AHA\_0601 | integral membrane protein |  |
| aha:AHA\_0602 | maltose O-acetyltransferase (EC:2.3.1.79); K00661 maltose O-acetyltransferase [EC:2.3.1.79] | ec:2.3.1.79 |
| aha:AHA\_0603 | MgtC/SapB transporter |  |
| aha:AHA\_0604 | sulfate-binding protein; K02048 sulfate transport system substrate-binding protein |  |
| aha:AHA\_0605 | hypothetical protein; K07002 |  |
| aha:AHA\_0606 | cysT; sulfate/thiosulfate ABC transporter permease CysT; K02046 sulfate transport system permease protein |  |
| aha:AHA\_0607 | cysW; sulfate/thiosulfate ABC transporter permease CysW; K02047 sulfate transport system permease protein |  |
| aha:AHA\_0608 | cysA; sulfate/thiosulfate ABC transporter ATP-binding protein (EC:3.6.3.25); K02045 sulfate transport system ATP-binding protein [EC:3.6.3.25] | ec:3.6.3.25 |
| aha:AHA\_0609 | transcriptional regulator CysB-like protein; K13635 LysR family transcriptional regulator, cys regulon transcriptional activator |  |
| aha:AHA\_0610 | N-acetylglucosamine-binding protein A; K03933 chitin-binding protein |  |
| aha:AHA\_0611 | methyl-accepting chemotaxis protein; K03406 methyl-accepting chemotaxis protein |  |
| aha:AHA\_0612 | oligoendopeptidase F |  |
| aha:AHA\_0613 | hypothetical protein; K02030 polar amino acid transport system substrate-binding protein |  |
| aha:AHA\_0614 | hypothetical protein |  |
| aha:AHA\_0615 | hypothetical protein |  |
| aha:AHA\_0616 | hypothetical protein |  |
| aha:AHA\_0617 | tricorn protease-like protein; K08676 tricorn protease [EC:3.4.21.-] |  |
| aha:AHA\_0618 | ErfK/YbiS/YcfS/YnhG family protein |  |
| aha:AHA\_0619 | AzlC family protein |  |

  
**Neighborhood Representations for "yen:YE2474"**  

| ID | Annotation | EC number |
| --- | --- | --- |
| yen:YE2463 | outer membrane porin protein |  |
| yen:YE2465 | astE; succinylglutamate desuccinylase; K05526 succinylglutamate desuccinylase [EC:3.5.1.96] | ec:3.5.1.96 |
| yen:YE2466 | astB; succinylarginine dihydrolase (EC:3.5.3.23); K01484 succinylarginine dihydrolase [EC:3.5.3.23] | ec:3.5.3.23 |
| yen:YE2467 | astD; succinylglutamic semialdehyde dehydrogenase; K06447 succinylglutamic semialdehyde dehydrogenase [EC:1.2.1.71] | ec:1.2.1.71 |
| yen:YE2468 | astA; arginine succinyltransferase; K00673 arginine N-succinyltransferase [EC:2.3.1.109] | ec:2.3.1.109 |
| yen:YE2469 | argM; bifunctional succinylornithine transaminase/acetylornithine transaminase; K00840 succinylornithine aminotransferase [EC:2.6.1.81] | ec:2.6.1.81 |
| yen:YE2470 | GntR family transcriptional regulator |  |
| yen:YE2471 | cytochrome oxidase subunit; K00425 cytochrome d ubiquinol oxidase subunit I [EC:1.10.3.-] |  |
| yen:YE2472 | cytochrome oxidase subunit; K00426 cytochrome d ubiquinol oxidase subunit II [EC:1.10.3.-] |  |
| yen:YE2473 | narR; nitrate/nitrite sensor protein NarX; K07673 two-component system, NarL family, nitrate/nitrite sensor histidine kinase NarX [EC:2.7.13.3] | ec:2.7.13.3 |
| yen:YE2474 | transcriptional regulator CysB-like protein; K13635 LysR family transcriptional regulator, cys regulon transcriptional activator |  |
| yen:YE2475 | membrane transport protein; K08156 MFS transporter, DHA1 family, arabinose polymer transporter |  |
| yen:YE2476 | hypothetical protein |  |
| yen:YE2477 | chaA; calcium/sodium:proton antiporter; K07300 Ca2+:H+ antiporter |  |
| yen:YE2478 | phoH; hypothetical protein; K06217 phosphate starvation-inducible protein PhoH and related proteins |  |
| yen:YE2480 | acetyltransferase |  |
| yen:YE2481 | hmsS; hemin storage system protein; K11937 biofilm PGA synthesis protein PgaD |  |
| yen:YE2482 | hmsR; N-glycosyltransferase; K11936 biofilm PGA synthesis N-glycosyltransferase PgaC [EC:2.4.-.-] |  |
| yen:YE2483 | hmsF; outer membrane N-deacetylase; K11931 biofilm PGA synthesis lipoprotein PgaB [EC:3.-.-.-] |  |
| yen:YE2484 | hmsH; outer membrane protein; K11935 biofilm PGA synthesis protein PgaA |  |
| yen:YE2485 | endopeptidase |  |

  
**Neighborhood Representations for "net:Neut\_1186"**  

| ID | Annotation | EC number |
| --- | --- | --- |
| net:Neut\_1176 | fructose-1,6-bisphosphatase (EC:3.1.3.11); K03841 fructose-1,6-bisphosphatase I [EC:3.1.3.11] | ec:3.1.3.11 |
| net:Neut\_1177 | glutathione S-transferase domain-containing protein; K07393 putative glutathione S-transferase |  |
| net:Neut\_1178 | DoxX family protein; K15977 putative oxidoreductase |  |
| net:Neut\_1179 | polysaccharide deacetylase |  |
| net:Neut\_1180 | phosphohistidine phosphatase, SixA; K08296 phosphohistidine phosphatase [EC:3.1.3.-] |  |
| net:Neut\_1181 | hypothetical protein |  |
| net:Neut\_1182 | carboxylesterase (EC:3.1.1.1); K06999 phospholipase/carboxylesterase |  |
| net:Neut\_1183 | twin-arginine translocation pathway signal |  |
| net:Neut\_1184 | sulfite reductase subunit beta; K00381 sulfite reductase (NADPH) hemoprotein beta-component [EC:1.8.1.2] | ec:1.8.1.2 |
| net:Neut\_1185 | sulfite reductase (NADPH) flavoprotein subunit alpha; K00380 sulfite reductase (NADPH) flavoprotein alpha-component [EC:1.8.1.2] | ec:1.8.1.2 |
| net:Neut\_1186 | transcriptional regulator CysB-like protein; K13634 LysR family transcriptional regulator, cys regulon transcriptional activator |  |
| net:Neut\_1187 | phosphoadenosine phosphosulfate reductase (EC:1.8.4.8); K00390 phosphoadenosine phosphosulfate reductase [EC:1.8.4.8] | ec:1.8.4.8 |
| net:Neut\_1188 | sulfate adenylyltransferase subunit 2 (EC:2.7.7.4); K00957 sulfate adenylyltransferase subunit 2 [EC:2.7.7.4] | ec:2.7.7.4 |
| net:Neut\_1189 | sulfate adenylyltransferase, large subunit (EC:2.7.7.4); K00956 sulfate adenylyltransferase subunit 1 [EC:2.7.7.4] | ec:2.7.7.4 |
| net:Neut\_1190 | FAD-dependent pyridine nucleotide-disulfide oxidoreductase |  |
| net:Neut\_1191 | NAD(P)(+) transhydrogenase (EC:1.6.1.2); K00324 NAD(P) transhydrogenase subunit alpha [EC:1.6.1.2] | ec:1.6.1.2 |
| net:Neut\_1192 | transmembrane NAD(P) transhydrogenase subunit alpha subunit part 2; K00324 NAD(P) transhydrogenase subunit alpha [EC:1.6.1.2] | ec:1.6.1.2 |
| net:Neut\_1193 | NAD(P) transhydrogenase subunit beta; K00325 NAD(P) transhydrogenase subunit beta [EC:1.6.1.2] | ec:1.6.1.2 |
| net:Neut\_1194 | Sel1 domain-containing protein; K07126 |  |
| net:Neut\_1195 | bacterioferritin; K03594 bacterioferritin |  |
| net:Neut\_1196 | BFD/(2Fe-2S)-binding domain-containing protein; K02192 bacterioferritin-associated ferredoxin |  |

  
**Neighborhood Representations for "afe:Lferr\_0847"**  

| ID | Annotation | EC number |
| --- | --- | --- |
| afe:Lferr\_0837 | hypothetical protein |  |
| afe:Lferr\_0838 | hypothetical protein |  |
| afe:Lferr\_0839 | formate dehydrogenase family accessory protein FdhD; K02379 FdhD protein |  |
| afe:Lferr\_0840 | hypothetical protein |  |
| afe:Lferr\_0841 | oxidoreductase molybdopterin subunit (EC:1.2.1.2); K00123 formate dehydrogenase major subunit [EC:1.2.1.2] | ec:1.2.1.2 |
| afe:Lferr\_0842 | Fis family sigma-54 specific transcriptional regulator |  |
| afe:Lferr\_0843 | hypothetical protein |  |
| afe:Lferr\_0844 | glutathione-dependent formaldehyde-activating protein (EC:4.4.1.22); K03396 S-(hydroxymethyl)glutathione synthase [EC:4.4.1.22] | ec:4.4.1.22 |
| afe:Lferr\_0845 | S-(hydroxymethyl)glutathione dehydrogenase/class III alcohol dehydrogenase; K00121 S-(hydroxymethyl)glutathione dehydrogenase / alcohol dehydrogenase [EC:1.1.1.284 1.1.1.1] | ec:1.1.1.284 ec:1.1.1.1 |
| afe:Lferr\_0846 | S-formylglutathione hydrolase (EC:3.1.2.12); K01070 S-formylglutathione hydrolase [EC:3.1.2.12] | ec:3.1.2.12 |
| afe:Lferr\_0847 | LysR family transcriptional regulator; K13634 LysR family transcriptional regulator, cys regulon transcriptional activator |  |
| afe:Lferr\_0848 | two component sigma-54 specific Fis family transcriptional regulator |  |
| afe:Lferr\_0849 | NADH ubiquinone oxidoreductase 20 kDa subunit; K06282 hydrogenase small subunit [EC:1.12.99.6] | ec:1.12.99.6 |
| afe:Lferr\_0850 | Hydrogen:quinone oxidoreductase (EC:1.12.5.1); K06281 hydrogenase large subunit [EC:1.12.99.6] | ec:1.12.99.6 |
| afe:Lferr\_0851 | hypothetical protein |  |
| afe:Lferr\_0852 | hypothetical protein |  |
| afe:Lferr\_0853 | hydrogenase maturation protease |  |
| afe:Lferr\_0854 | hypothetical protein |  |
| afe:Lferr\_0855 | hypothetical protein |  |
| afe:Lferr\_0856 | hypothetical protein |  |
| afe:Lferr\_0857 | hypothetical protein |  |

  
**Neighborhood Representations for "afr:AFE\_0699"**  

| ID | Annotation | EC number |
| --- | --- | --- |
| afr:AFE\_0689 | transposase, degenerate |  |
| afr:AFE\_0690 | fdhD-2; formate dehydrogenase family accessory protein FdhD; K02379 FdhD protein |  |
| afr:AFE\_0691 | hypothetical protein |  |
| afr:AFE\_0692 | oxidoreductase molybdopterin subunit; K00123 formate dehydrogenase major subunit [EC:1.2.1.2] | ec:1.2.1.2 |
| afr:AFE\_0693 | sigma-54 dependent transcriptional regulator |  |
| afr:AFE\_0695 | hypothetical protein |  |
| afr:AFE\_0694 | hypothetical protein |  |
| afr:AFE\_0696 | gfa; glutathione-dependent formaldehyde-activating protein (EC:4.4.1.22); K03396 S-(hydroxymethyl)glutathione synthase [EC:4.4.1.22] | ec:4.4.1.22 |
| afr:AFE\_0697 | S-(hydroxymethyl)glutathione dehydrogenase/class III alcohol dehydrogenase (EC:1.1.1.1 1.1.1.284); K00121 S-(hydroxymethyl)glutathione dehydrogenase / alcohol dehydrogenase [EC:1.1.1.284 1.1.1.1] | ec:1.1.1.284 ec:1.1.1.1 |
| afr:AFE\_0698 | fghA; S-formylglutathione hydrolase (EC:3.1.2.12); K01070 S-formylglutathione hydrolase [EC:3.1.2.12] | ec:3.1.2.12 |
| afr:AFE\_0699 | cysB; sulfur assimilation LysR family transcriptional regulator; K13634 LysR family transcriptional regulator, cys regulon transcriptional activator |  |
| afr:AFE\_0700 | hupR; hydrogenase sigma-54 dependent DNA-binding response regulator |  |
| afr:AFE\_0701 | hybA; hydrogenase-2, small subunit (EC:1.12.99.6); K06282 hydrogenase small subunit [EC:1.12.99.6] | ec:1.12.99.6 |
| afr:AFE\_0702 | hybC; hydrogenase-2, large subunit (EC:1.12.99.6); K06281 hydrogenase large subunit [EC:1.12.99.6] | ec:1.12.99.6 |
| afr:AFE\_0703 | hypothetical protein |  |
| afr:AFE\_0704 | hypothetical protein |  |
| afr:AFE\_0705 | hydrogenase maturation protease |  |
| afr:AFE\_0706 | hypothetical protein |  |
| afr:AFE\_0707 | hypothetical protein |  |
| afr:AFE\_0708 | hypothetical protein |  |
| afr:AFE\_0709 | hypothetical protein |  |

  
**Neighborhood Representations for "ebd:ECBD\_1661"**  

| ID | Annotation | EC number |
| --- | --- | --- |
| ebd:ECBD\_1651 | hypothetical protein |  |
| ebd:ECBD\_1652 | pseudogene |  |
| ebd:ECBD\_1653 | pseudogene |  |
| ebd:ECBD\_1654 | hypothetical protein |  |
| ebd:ECBD\_1655 | pseudogene |  |
| ebd:ECBD\_1656 | cobU; adenosylcobinamide kinase/adenosylcobinamide-phosphate guanylyltransferase (EC:2.7.7.62); K02231 adenosylcobinamide kinase / adenosylcobinamide-phosphate guanylyltransferase [EC:2.7.1.156 2.7.7.62] | ec:2.7.7.62 ec:2.7.1.156 |
| ebd:ECBD\_1657 | cobS; cobalamin synthase; K02233 adenosylcobinamide-GDP ribazoletransferase [EC:2.7.8.26] | ec:2.7.8.26 |
| ebd:ECBD\_1658 | cobT; nicotinate-nucleotide--dimethylbenzimidazole phosphoribosyltransferase (EC:2.4.2.21); K00768 nicotinate-nucleotide--dimethylbenzimidazole phosphoribosyltransferase [EC:2.4.2.21] | ec:2.4.2.21 |
| ebd:ECBD\_1659 | hypothetical protein; K16291 L,D-transpeptidase |  |
| ebd:ECBD\_1660 | nitrogen assimilation transcriptional regulator |  |
| ebd:ECBD\_1661 | cbl; transcriptional regulator Cbl; K13635 LysR family transcriptional regulator, cys regulon transcriptional activator |  |
| ebd:ECBD\_1662 | hypothetical protein |  |
| ebd:ECBD\_1663 | hypothetical protein |  |
| ebd:ECBD\_1664 | AMP nucleosidase (EC:3.2.2.4); K01241 AMP nucleosidase [EC:3.2.2.4] | ec:3.2.2.4 |
| ebd:ECBD\_1665 | shikimate transporter; K08172 MFS transporter, MHS family, shikimate and dehydroshikimate transport protein |  |
| ebd:ECBD\_1666 | pseudogene |  |
| ebd:ECBD\_1667 | Ig domain protein group 1 domain protein; K13735 adhesin/invasin |  |
| ebd:ECBD\_1668 | hypothetical protein |  |
| ebd:ECBD\_1669 | hypothetical protein; K09933 hypothetical protein |  |
| ebd:ECBD\_1670 | transposase IS4 family protein |  |
| ebd:ECBD\_1671 | pseudogene |  |

  
**Neighborhood Representations for "ebr:ECB\_01897"**  

| ID | Annotation | EC number |
| --- | --- | --- |
| ebr:ECB\_01887 | yedZ; putative sulfite oxidase subunit YedZ; K17247 sulfoxide reductase heme-binding subunit YedZ |  |
| ebr:ECB\_01888 | yodA; hypothetical protein |  |
| ebr:ECB\_01889 | yodB; putative cytochrome |  |
| ebr:ECB\_01890 | yeeI; hypothetical protein; K09933 hypothetical protein |  |
| ebr:ECB\_01891 | yeeJ; adhesin; K13735 adhesin/invasin |  |
| ebr:ECB\_01892 | yeeL; pseudogene |  |
| ebr:ECB\_01893 | shiA; shikimate transporter; K08172 MFS transporter, MHS family, shikimate and dehydroshikimate transport protein |  |
| ebr:ECB\_01894 | amn; AMP nucleosidase (EC:3.2.2.4); K01241 AMP nucleosidase [EC:3.2.2.4] | ec:3.2.2.4 |
| ebr:ECB\_01895 | yeeN; hypothetical protein |  |
| ebr:ECB\_01896 | yeeO; hypothetical protein |  |
| ebr:ECB\_01897 | cbl; transcriptional regulator Cbl; K13635 LysR family transcriptional regulator, cys regulon transcriptional activator |  |
| ebr:ECB\_01898 | nac; nitrogen assimilation transcriptional regulator |  |
| ebr:ECB\_01899 | erfK; hypothetical protein; K16291 L,D-transpeptidase |  |
| ebr:ECB\_01900 | cobT; nicotinate-nucleotide--dimethylbenzimidazole phosphoribosyltransferase (EC:2.4.2.21); K00768 nicotinate-nucleotide--dimethylbenzimidazole phosphoribosyltransferase [EC:2.4.2.21] | ec:2.4.2.21 |
| ebr:ECB\_01901 | cobS; cobalamin synthase; K02233 adenosylcobinamide-GDP ribazoletransferase [EC:2.7.8.26] | ec:2.7.8.26 |
| ebr:ECB\_01902 | cobU; adenosylcobinamide kinase/adenosylcobinamide-phosphate guanylyltransferase; K02231 adenosylcobinamide kinase / adenosylcobinamide-phosphate guanylyltransferase [EC:2.7.1.156 2.7.7.62] | ec:2.7.7.62 ec:2.7.1.156 |
| ebr:ECB\_01903 | yoeA; pseudogene |  |
| ebr:ECB\_01904 | insD; insertion element IS2 transposase InsD |  |
| ebr:ECB\_01905 | insC; pseudogene |  |
| ebr:ECB\_01906 | hypothetical protein |  |
| ebr:ECB\_01907 | hypothetical protein |  |

  
**Neighborhood Representations for "ebw:BWG\_1782"**  

| ID | Annotation | EC number |
| --- | --- | --- |
| ebw:BWG\_4150 | serU; tRNA-Ser; K14233 tRNA Ser |  |
| ebw:BWG\_1776 | mtfA; hypothetical protein; K09933 hypothetical protein |  |
| ebw:BWG\_4151 | asnT; tRNA-Asn; K14220 tRNA Asn |  |
| ebw:BWG\_1777 | yeeJ; adhesin; K13735 adhesin/invasin |  |
| ebw:BWG\_1778 | shiA; shikimate transporter; K08172 MFS transporter, MHS family, shikimate and dehydroshikimate transport protein |  |
| ebw:BWG\_1779 | amn; AMP nucleosidase; K01241 AMP nucleosidase [EC:3.2.2.4] | ec:3.2.2.4 |
| ebw:BWG\_1780 | yeeN; hypothetical protein |  |
| ebw:BWG\_4152 | asnW; tRNA-Asn; K14220 tRNA Asn |  |
| ebw:BWG\_1781 | yeeO; hypothetical protein |  |
| ebw:BWG\_4153 | asnU; tRNA-Asn; K14220 tRNA Asn |  |
| ebw:BWG\_1782 | cbl; transcriptional regulator Cbl; K13635 LysR family transcriptional regulator, cys regulon transcriptional activator |  |
| ebw:BWG\_1783 | nac; nitrogen assimilation transcriptional regulator |  |
| ebw:BWG\_4154 | asnV; tRNA-Asn; K14220 tRNA Asn |  |
| ebw:BWG\_1784 | erfK; hypothetical protein; K16291 L,D-transpeptidase |  |
| ebw:BWG\_1785 | cobT; nicotinate-nucleotide--dimethylbenzimidazole phosphoribosyltransferase; K00768 nicotinate-nucleotide--dimethylbenzimidazole phosphoribosyltransferase [EC:2.4.2.21] | ec:2.4.2.21 |
| ebw:BWG\_1786 | cobS; cobalamin synthase; K02233 adenosylcobinamide-GDP ribazoletransferase [EC:2.7.8.26] | ec:2.7.8.26 |
| ebw:BWG\_1787 | cobU; adenosylcobinamide kinase/adenosylcobinamide-phosphate guanylyltransferase; K02231 adenosylcobinamide kinase / adenosylcobinamide-phosphate guanylyltransferase [EC:2.7.1.156 2.7.7.62] | ec:2.7.7.62 ec:2.7.1.156 |
| ebw:BWG\_1788 | insH; IS5 transposase and trans-activator |  |
| ebw:BWG\_1789 | insD; insertion element IS2 transposase InsD |  |
| ebw:BWG\_1790 | insC; insertion sequence 2 OrfA protein |  |
| ebw:BWG\_4232 | isrC; Novel sRNA, function unknown, CP4-44; putative prophage remnant |  |

  
**Neighborhood Representations for "ecd:ECDH10B\_2131"**  

| ID | Annotation | EC number |
| --- | --- | --- |
| ecd:ECDH10B\_2118 | serU; tRNA-Ser; K14233 tRNA Ser |  |
| ecd:ECDH10B\_2119 | yeeI; hypothetical protein; K09933 hypothetical protein |  |
| ecd:ECDH10B\_2120 | asnT; tRNA-Asn; K14220 tRNA Asn |  |
| ecd:ECDH10B\_2121 | yeeJ; adhesin; K13735 adhesin/invasin |  |
| ecd:ECDH10B\_2125 | shiA; shikimate transporter; K08172 MFS transporter, MHS family, shikimate and dehydroshikimate transport protein |  |
| ecd:ECDH10B\_2126 | amn; AMP nucleosidase; K01241 AMP nucleosidase [EC:3.2.2.4] | ec:3.2.2.4 |
| ecd:ECDH10B\_2127 | yeeN; hypothetical protein |  |
| ecd:ECDH10B\_2128 | asnW; tRNA-Asn; K14220 tRNA Asn |  |
| ecd:ECDH10B\_2129 | yeeO; hypothetical protein |  |
| ecd:ECDH10B\_2130 | asnU; tRNA-Asn; K14220 tRNA Asn |  |
| ecd:ECDH10B\_2131 | cbl; transcriptional regulator Cbl; K13635 LysR family transcriptional regulator, cys regulon transcriptional activator |  |
| ecd:ECDH10B\_2132 | nac; nitrogen assimilation transcriptional regulator |  |
| ecd:ECDH10B\_2133 | asnV; tRNA-Asn; K14220 tRNA Asn |  |
| ecd:ECDH10B\_2134 | erfK; hypothetical protein; K16291 L,D-transpeptidase |  |
| ecd:ECDH10B\_2135 | cobT; nicotinate-nucleotide--dimethylbenzimidazole phosphoribosyltransferase; K00768 nicotinate-nucleotide--dimethylbenzimidazole phosphoribosyltransferase [EC:2.4.2.21] | ec:2.4.2.21 |
| ecd:ECDH10B\_2136 | cobS; cobalamin synthase; K02233 adenosylcobinamide-GDP ribazoletransferase [EC:2.7.8.26] | ec:2.7.8.26 |
| ecd:ECDH10B\_2137 | cobU; adenosylcobinamide kinase/adenosylcobinamide-phosphate guanylyltransferase; K02231 adenosylcobinamide kinase / adenosylcobinamide-phosphate guanylyltransferase [EC:2.7.1.156 2.7.7.62] | ec:2.7.7.62 ec:2.7.1.156 |
| ecd:ECDH10B\_2138 | insH-6; CP4-44 prophage; IS5 transposase and trans-activator; K07481 transposase, IS5 family |  |
| ecd:ECDH10B\_2141 | insD-3; insertion element IS2 transposase InsD |  |
| ecd:ECDH10B\_2142 | insC-3; insertion sequence 2 OrfA protein |  |
| ecd:ECDH10B\_2144 | yeeP; pseudogene |  |

  
**Neighborhood Representations for "ecj:Y75\_p1949"**  

| ID | Annotation | EC number |
| --- | --- | --- |
| ecj:Y75\_p1939 | yedZ; inner membrane protein; K17247 sulfoxide reductase heme-binding subunit YedZ |  |
| ecj:Y75\_p1940 | yodA; metal-binding protein |  |
| ecj:Y75\_p1941 | yodB; cytochrome |  |
| ecj:Y75\_p1942 | yeeI; hypothetical protein; K09933 hypothetical protein |  |
| ecj:Y75\_p1943 | yeeJ; adhesin; K13735 adhesin/invasin |  |
| ecj:Y75\_p1944 | yeeL; yeeL |  |
| ecj:Y75\_p1945 | shiA; shikimate transporter; K08172 MFS transporter, MHS family, shikimate and dehydroshikimate transport protein |  |
| ecj:Y75\_p1946 | amn; AMP nucleosidase; K01241 AMP nucleosidase [EC:3.2.2.4] | ec:3.2.2.4 |
| ecj:Y75\_p1947 | yeeN; hypothetical protein |  |
| ecj:Y75\_p1948 | yeeO; multidrug efflux system |  |
| ecj:Y75\_p1949 | cbl; DNA-binding transcriptional activator; K13635 LysR family transcriptional regulator, cys regulon transcriptional activator |  |
| ecj:Y75\_p1950 | nac; DNA-binding transcriptional dual regulator |  |
| ecj:Y75\_p1951 | erfK; hypothetical protein; K16291 L,D-transpeptidase |  |
| ecj:Y75\_p1952 | cobT; nicotinate-nucleotide dimethylbenzimidazole-P phophoribosyl transferase; K00768 nicotinate-nucleotide--dimethylbenzimidazole phosphoribosyltransferase [EC:2.4.2.21] | ec:2.4.2.21 |
| ecj:Y75\_p1953 | cobS; cobalamin 5'-phosphate synthase; K02233 adenosylcobinamide-GDP ribazoletransferase [EC:2.7.8.26] | ec:2.7.8.26 |
| ecj:Y75\_p1954 | cobU; bifunctional cobinamide kinase/cobinamide phosphate guanylyltransferase; K02231 adenosylcobinamide kinase / adenosylcobinamide-phosphate guanylyltransferase [EC:2.7.1.156 2.7.7.62] | ec:2.7.7.62 ec:2.7.1.156 |
| ecj:Y75\_p1955 | insH; IS5 transposase and trans-activator |  |
| ecj:Y75\_p1956 | yoeA; yoeA |  |
| ecj:Y75\_p1957 | insD; IS2 insertion element transposase InsAB' |  |
| ecj:Y75\_p1958 | insC; IS2 insertion element repressor InsA |  |
| ecj:Y75\_p1959 | yoeE; disrupted hemin or colicin receptor |  |

  
**Neighborhood Representations for "ecm:EcSMS35\_1137"**  

| ID | Annotation | EC number |
| --- | --- | --- |
| ecm:EcSMS35\_1127 | hypothetical protein |  |
| ecm:EcSMS35\_1128 | pseudogene |  |
| ecm:EcSMS35\_1129 | hypothetical protein |  |
| ecm:EcSMS35\_1130 | hypothetical protein |  |
| ecm:EcSMS35\_1131 | cobU; adenosylcobinamide kinase/adenosylcobinamide-phosphate guanylyltransferase (EC:2.7.1.156 2.7.7.62); K02231 adenosylcobinamide kinase / adenosylcobinamide-phosphate guanylyltransferase [EC:2.7.1.156 2.7.7.62] | ec:2.7.7.62 ec:2.7.1.156 |
| ecm:EcSMS35\_1132 | cobS; cobalamin synthase (EC:2.7.8.26); K02233 adenosylcobinamide-GDP ribazoletransferase [EC:2.7.8.26] | ec:2.7.8.26 |
| ecm:EcSMS35\_1133 | cobT; nicotinate-nucleotide--dimethylbenzimidazole phosphoribosyltransferase (EC:2.4.2.21); K00768 nicotinate-nucleotide--dimethylbenzimidazole phosphoribosyltransferase [EC:2.4.2.21] | ec:2.4.2.21 |
| ecm:EcSMS35\_1134 | hypothetical protein; K16291 L,D-transpeptidase |  |
| ecm:EcSMS35\_1135 | tRNA-Asn; K14220 tRNA Asn |  |
| ecm:EcSMS35\_1136 | nac; nitrogen assimilation transcriptional regulator |  |
| ecm:EcSMS35\_1137 | cbl; transcriptional regulator Cbl; K13635 LysR family transcriptional regulator, cys regulon transcriptional activator |  |
| ecm:EcSMS35\_1138 | tRNA-Asn; K14220 tRNA Asn |  |
| ecm:EcSMS35\_1139 | hypothetical protein |  |
| ecm:EcSMS35\_1140 | tRNA-Asn; K14220 tRNA Asn |  |
| ecm:EcSMS35\_1141 | hypothetical protein |  |
| ecm:EcSMS35\_1142 | hypothetical protein |  |
| ecm:EcSMS35\_1143 | amn; AMP nucleosidase (EC:3.2.2.4); K01241 AMP nucleosidase [EC:3.2.2.4] | ec:3.2.2.4 |
| ecm:EcSMS35\_1144 | shiA; shikimate transporter; K08172 MFS transporter, MHS family, shikimate and dehydroshikimate transport protein |  |
| ecm:EcSMS35\_1145 | hypothetical protein |  |
| ecm:EcSMS35\_1146 | putative invasin; K13735 adhesin/invasin |  |
| ecm:EcSMS35\_1147 | tRNA-Asn; K14220 tRNA Asn |  |

  
**Neighborhood Representations for "eco:b1987"**  

| ID | Annotation | EC number |
| --- | --- | --- |
| eco:b1976 | mtfA; anti-repressor for DgsA(Mlc); K09933 hypothetical protein |  |
| eco:b1977 | asnT; tRNA-Asn; K14220 tRNA Asn |  |
| eco:b1978 | yeeJ; putative adhesin; K13735 adhesin/invasin |  |
| eco:b4497 | yeeL; pseudogene |  |
| eco:b1981 | shiA; shikimate transporter; K08172 MFS transporter, MHS family, shikimate and dehydroshikimate transport protein |  |
| eco:b1982 | amn; AMP nucleosidase (EC:3.2.2.4); K01241 AMP nucleosidase [EC:3.2.2.4] | ec:3.2.2.4 |
| eco:b1983 | yeeN; conserved protein, UPF0082 family |  |
| eco:b1984 | asnW; tRNA-Asn; K14220 tRNA Asn |  |
| eco:b1985 | yeeO; predicted multdrug exporter, MATE family |  |
| eco:b1986 | asnU; tRNA-Asn; K14220 tRNA Asn |  |
| eco:b1987 | cbl; DNA-binding transcriptional activator for the ssuEADCB and tauABCD operons; K13635 LysR family transcriptional regulator, cys regulon transcriptional activator |  |
| eco:b1988 | nac; DNA-binding transcriptional dual regulator of nitrogen assimilation |  |
| eco:b1989 | asnV; tRNA-Asn; K14220 tRNA Asn |  |
| eco:b1990 | erfK; L,D-transpeptidase linking Lpp to murein; K16291 L,D-transpeptidase |  |
| eco:b1991 | cobT; nicotinate-nucleotide--dimethylbenzimidazole phosphoribosyltransferase (EC:2.4.2.21); K00768 nicotinate-nucleotide--dimethylbenzimidazole phosphoribosyltransferase [EC:2.4.2.21] | ec:2.4.2.21 |
| eco:b1992 | cobS; cobalamin synthase (EC:2.-.-.-); K02233 adenosylcobinamide-GDP ribazoletransferase [EC:2.7.8.26] | ec:2.7.8.26 |
| eco:b1993 | cobU; bifunctional cobinamide kinase/cobinamide phosphate guanylyltransferase; K02231 adenosylcobinamide kinase / adenosylcobinamide-phosphate guanylyltransferase [EC:2.7.1.156 2.7.7.62] | ec:2.7.7.62 ec:2.7.1.156 |
| eco:b4639 | yeeH; pseudogene |  |
| eco:b1994 | insH1; IS5 transposase and trans-activator |  |
| eco:b4640 | yoeG; pseudogene |  |
| eco:b4641 | yoeH; pseudogene |  |

  
**Neighborhood Representations for "ecw:EcE24377A\_2269"**  

| ID | Annotation | EC number |
| --- | --- | --- |
| ecw:EcE24377A\_2259 | hypothetical protein; K09933 hypothetical protein |  |
| ecw:EcE24377A\_5041 | tRNA-Asn; K14220 tRNA Asn |  |
| ecw:EcE24377A\_2261 | invasin; K13735 adhesin/invasin |  |
| ecw:EcE24377A\_2262 | hypothetical protein |  |
| ecw:EcE24377A\_2263 | shiA; shikimate transporter; K08172 MFS transporter, MHS family, shikimate and dehydroshikimate transport protein |  |
| ecw:EcE24377A\_2264 | amn; AMP nucleosidase (EC:3.2.2.4); K01241 AMP nucleosidase [EC:3.2.2.4] | ec:3.2.2.4 |
| ecw:EcE24377A\_2265 | hypothetical protein |  |
| ecw:EcE24377A\_5042 | tRNA-Asn; K14220 tRNA Asn |  |
| ecw:EcE24377A\_2267 | hypothetical protein |  |
| ecw:EcE24377A\_5043 | tRNA-Asn; K14220 tRNA Asn |  |
| ecw:EcE24377A\_2269 | cbl; transcriptional regulator Cbl; K13635 LysR family transcriptional regulator, cys regulon transcriptional activator |  |
| ecw:EcE24377A\_2270 | nac; nitrogen assimilation transcriptional regulator |  |
| ecw:EcE24377A\_5044 | tRNA-Asn; K14220 tRNA Asn |  |
| ecw:EcE24377A\_2272 | hypothetical protein; K16291 L,D-transpeptidase |  |
| ecw:EcE24377A\_2273 | cobT; nicotinate-nucleotide--dimethylbenzimidazole phosphoribosyltransferase (EC:2.4.2.21); K00768 nicotinate-nucleotide--dimethylbenzimidazole phosphoribosyltransferase [EC:2.4.2.21] | ec:2.4.2.21 |
| ecw:EcE24377A\_2274 | cobS; cobalamin synthase (EC:2.7.8.26); K02233 adenosylcobinamide-GDP ribazoletransferase [EC:2.7.8.26] | ec:2.7.8.26 |
| ecw:EcE24377A\_2275 | cobU; adenosylcobinamide kinase (EC:2.7.1.156 2.7.7.62); K02231 adenosylcobinamide kinase / adenosylcobinamide-phosphate guanylyltransferase [EC:2.7.1.156 2.7.7.62] | ec:2.7.7.62 ec:2.7.1.156 |
| ecw:EcE24377A\_2276 | cobalamin biosynthesis family protein |  |
| ecw:EcE24377A\_2277 | pocR; regulatory protein PocR |  |
| ecw:EcE24377A\_2278 | pduF; propanediol diffusion facilitator |  |
| ecw:EcE24377A\_2279 | pduA; propanediol utilization protein PduA |  |

  
**Neighborhood Representations for "ecy:ECSE\_2272"**  

| ID | Annotation | EC number |
| --- | --- | --- |
| ecy:ECSE\_2262 | hypothetical protein; K09933 hypothetical protein |  |
| ecy:ECSE\_2263 | hypothetical protein |  |
| ecy:ECSE\_2264 | hypothetical protein; K13735 adhesin/invasin |  |
| ecy:ECSE\_2265 | putative adhesin; K13735 adhesin/invasin |  |
| ecy:ECSE\_2266 | hypothetical protein |  |
| ecy:ECSE\_2267 | shikimate transporter; K08172 MFS transporter, MHS family, shikimate and dehydroshikimate transport protein |  |
| ecy:ECSE\_2268 | AMP nucleosidase; K01241 AMP nucleosidase [EC:3.2.2.4] | ec:3.2.2.4 |
| ecy:ECSE\_2269 | hypothetical protein |  |
| ecy:ECSE\_2270 | hypothetical protein |  |
| ecy:ECSE\_2271 | hypothetical protein |  |
| ecy:ECSE\_2272 | cbl; transcriptional regulator Cbl; K13635 LysR family transcriptional regulator, cys regulon transcriptional activator |  |
| ecy:ECSE\_2273 | nitrogen assimilation transcriptional regulator |  |
| ecy:ECSE\_2274 | hypothetical protein; K16291 L,D-transpeptidase |  |
| ecy:ECSE\_2275 | cobT; nicotinate-nucleotide--dimethylbenzimidazole phosphoribosyltransferase; K00768 nicotinate-nucleotide--dimethylbenzimidazole phosphoribosyltransferase [EC:2.4.2.21] | ec:2.4.2.21 |
| ecy:ECSE\_2276 | cobS; cobalamin synthase; K02233 adenosylcobinamide-GDP ribazoletransferase [EC:2.7.8.26] | ec:2.7.8.26 |
| ecy:ECSE\_2277 | cobU; adenosylcobinamide kinase/adenosylcobinamide-phosphate guanylyltransferase; K02231 adenosylcobinamide kinase / adenosylcobinamide-phosphate guanylyltransferase [EC:2.7.1.156 2.7.7.62] | ec:2.7.7.62 ec:2.7.1.156 |
| ecy:ECSE\_2278 | hypothetical protein |  |
| ecy:ECSE\_2279 | putative propanediol utilization protein; K04029 ethanolamine utilization protein EutP |  |
| ecy:ECSE\_2280 | hypothetical protein; K09802 hypothetical protein |  |
| ecy:ECSE\_2281 | hypothetical protein |  |
| ecy:ECSE\_2282 | DNA gyrase inhibitor; K07470 DNA gyrase inhibitor |  |

  
**Neighborhood Representations for "eoh:ECO103\_2449"**  

| ID | Annotation | EC number |
| --- | --- | --- |
| eoh:ECO103\_2439 | IS629 transposase OrfB |  |
| eoh:ECO103\_2440 | IS629 transposase OrfA; K07483 transposase |  |
| eoh:ECO103\_2441 | pseudogene |  |
| eoh:ECO103\_2442 | IS629 transposase OrfA; K07483 transposase |  |
| eoh:ECO103\_2443 | IS629 transposase OrfB |  |
| eoh:ECO103\_2444 | yeeL; ADP-heptose:LPS heptosyl transferase |  |
| eoh:ECO103\_2445 | shiA; shikimate transporter ShiA; K08172 MFS transporter, MHS family, shikimate and dehydroshikimate transport protein |  |
| eoh:ECO103\_2446 | amn; AMP nucleosidase; K01241 AMP nucleosidase [EC:3.2.2.4] | ec:3.2.2.4 |
| eoh:ECO103\_2447 | yeeN; hypothetical protein |  |
| eoh:ECO103\_2448 | yeeO; multidrug efflux system |  |
| eoh:ECO103\_2449 | cbl; DNA-binding transcriptional activator Cbl of cysteine biosynthesis; K13635 LysR family transcriptional regulator, cys regulon transcriptional activator |  |
| eoh:ECO103\_2450 | nac; DNA-binding transcriptional dual regulator Nac of nitrogen assimilation |  |
| eoh:ECO103\_2451 | erfK; hypothetical protein; K16291 L,D-transpeptidase |  |
| eoh:ECO103\_2452 | cobT; nicotinate-nucleotide dimethylbenzimidazole-P phophoribosyl transferase; K00768 nicotinate-nucleotide--dimethylbenzimidazole phosphoribosyltransferase [EC:2.4.2.21] | ec:2.4.2.21 |
| eoh:ECO103\_2453 | cobS; cobalamin 5'-phosphate synthase; K02233 adenosylcobinamide-GDP ribazoletransferase [EC:2.7.8.26] | ec:2.7.8.26 |
| eoh:ECO103\_2454 | cobU; bifunctional cobinamide kinase and cobinamide phosphate guanylyltransferase; K02231 adenosylcobinamide kinase / adenosylcobinamide-phosphate guanylyltransferase [EC:2.7.1.156 2.7.7.62] | ec:2.7.7.62 ec:2.7.1.156 |
| eoh:ECO103\_2455 | pseudogene |  |
| eoh:ECO103\_2456 | pseudogene |  |
| eoh:ECO103\_2457 | carbohydrate kinase |  |
| eoh:ECO103\_2458 | cytoplasmic protein |  |
| eoh:ECO103\_2459 | hypothetical protein; K09936 bacterial/archaeal transporter family-2 protein |  |

  
**Neighborhood Representations for "eoi:ECO111\_2634"**  

| ID | Annotation | EC number |
| --- | --- | --- |
| eoi:ECO111\_2624 | putative integrase |  |
| eoi:ECO111\_2625 | putative IS629 transposase OrfB |  |
| eoi:ECO111\_2626 | putative IS629 transposase OrfA; K07483 transposase |  |
| eoi:ECO111\_2627 | yeeL; putative ADP-heptose:LPS heptosyl transferase |  |
| eoi:ECO111\_2628 | pseudogene |  |
| eoi:ECO111\_2629 | pseudogene |  |
| eoi:ECO111\_2630 | amn; AMP nucleosidase; K01241 AMP nucleosidase [EC:3.2.2.4] | ec:3.2.2.4 |
| eoi:ECO111\_2631 | yeeN; hypothetical protein |  |
| eoi:ECO111\_2632 | hypothetical protein |  |
| eoi:ECO111\_2633 | hypothetical protein |  |
| eoi:ECO111\_2634 | cbl; DNA-binding transcriptional activator; K13635 LysR family transcriptional regulator, cys regulon transcriptional activator |  |
| eoi:ECO111\_2635 | nac; DNA-binding transcriptional dual regulator Nac |  |
| eoi:ECO111\_2636 | erfK; hypothetical protein; K16291 L,D-transpeptidase |  |
| eoi:ECO111\_2637 | cobT; nicotinate-nucleotide dimethylbenzimidazole-P phophoribosyl transferase; K00768 nicotinate-nucleotide--dimethylbenzimidazole phosphoribosyltransferase [EC:2.4.2.21] | ec:2.4.2.21 |
| eoi:ECO111\_2638 | cobS; cobalamin 5'-phosphate synthase; K02233 adenosylcobinamide-GDP ribazoletransferase [EC:2.7.8.26] | ec:2.7.8.26 |
| eoi:ECO111\_2639 | cobU; bifunctional cobinamide kinase and cobinamide phosphate guanylyltransferase; K02231 adenosylcobinamide kinase / adenosylcobinamide-phosphate guanylyltransferase [EC:2.7.1.156 2.7.7.62] | ec:2.7.7.62 ec:2.7.1.156 |
| eoi:ECO111\_2640 | putative IS629 transposase OrfA; K07483 transposase |  |
| eoi:ECO111\_2641 | putative IS629 transposase OrfB |  |
| eoi:ECO111\_2642 | pseudogene |  |
| eoi:ECO111\_2643 | hypothetical protein |  |
| eoi:ECO111\_2644 | hypothetical protein |  |

  
**Neighborhood Representations for "cko:CKO\_00956"**  

| ID | Annotation | EC number |
| --- | --- | --- |
| cko:CKO\_00946 | hypothetical protein |  |
| cko:CKO\_00947 | hypothetical protein |  |
| cko:CKO\_00948 | hypothetical protein |  |
| cko:CKO\_00949 | hypothetical protein; K02077 zinc/manganese transport system substrate-binding protein |  |
| cko:CKO\_00950 | hypothetical protein; K02075 zinc/manganese transport system permease protein |  |
| cko:CKO\_00951 | hypothetical protein; K02074 zinc/manganese transport system ATP-binding protein |  |
| cko:CKO\_00952 | hypothetical protein; K07093 |  |
| cko:CKO\_00953 | hypothetical protein |  |
| cko:CKO\_00954 | tRNA-Asn; K14220 tRNA Asn |  |
| cko:CKO\_00955 | nitrogen assimilation transcriptional regulator |  |
| cko:CKO\_00956 | cbl; transcriptional regulator Cbl; K13635 LysR family transcriptional regulator, cys regulon transcriptional activator |  |
| cko:CKO\_00957 | tRNA-Asn; K14220 tRNA Asn |  |
| cko:CKO\_00958 | hypothetical protein; K09933 hypothetical protein |  |
| cko:CKO\_00959 | tRNA-Ser; K14233 tRNA Ser |  |
| cko:CKO\_00960 | hypothetical protein |  |
| cko:CKO\_00961 | hypothetical protein; K07345 major type 1 subunit fimbrin (pilin) |  |
| cko:CKO\_00962 | hypothetical protein |  |
| cko:CKO\_00963 | hypothetical protein; K07347 outer membrane usher protein |  |
| cko:CKO\_00964 | hypothetical protein |  |
| cko:CKO\_00965 | hypothetical protein |  |
| cko:CKO\_00966 | hypothetical protein |  |

  
**Neighborhood Representations for "eca:ECA1519"**  

| ID | Annotation | EC number |
| --- | --- | --- |
| eca:ECA1509 | methyl-accepting chemotaxis protein; K03406 methyl-accepting chemotaxis protein |  |
| eca:ECA1510 | iron permease; K07243 high-affinity iron transporter |  |
| eca:ECA1511 | hypothetical protein; K07230 |  |
| eca:ECA1512 | hypothetical protein |  |
| eca:ECA1513 | permease; K02004 putative ABC transport system permease protein |  |
| eca:ECA1514 | permease; K02004 putative ABC transport system permease protein |  |
| eca:ECA1515 | ABC transporter ATP-binding protein; K02003 putative ABC transport system ATP-binding protein |  |
| eca:ECA1516 | lipoprotein |  |
| eca:ECA1517 | hypothetical protein |  |
| eca:ECA1518 | AraC family transcriptional regulator |  |
| eca:ECA1519 | cbl; transcriptional regulator CysB-like protein; K13635 LysR family transcriptional regulator, cys regulon transcriptional activator |  |
| eca:ECA1520 | gltP; glutamate/aspartate:proton symporter; K11102 proton glutamate symport protein |  |
| eca:ECA1521 | hypothetical protein |  |
| eca:ECA1522 | hypothetical protein |  |
| eca:ECA1523 | glycerol dehydrogenase (EC:1.1.1.6); K00005 glycerol dehydrogenase [EC:1.1.1.6] | ec:1.1.1.6 |
| eca:ECA1524 | ABC transporter permease; K02050 NitT/TauT family transport system permease protein |  |
| eca:ECA1525 | ABC transporter ATP-binding protein; K02049 NitT/TauT family transport system ATP-binding protein |  |
| eca:ECA1526 | ABC transporter periplasmic-binding protein; K02051 NitT/TauT family transport system substrate-binding protein |  |
| eca:ECA1527 | malate/lactate dehydrogenase; K13574 uncharacterized oxidoreductase [EC:1.1.1.-] |  |
| eca:ECA1528 | tauA; taurine-binding periplasmic protein; K15551 taurine transport system substrate-binding protein |  |
| eca:ECA1529 | tauC; taurine transport system permease; K15552 taurine transport system permease protein |  |

  
**Neighborhood Representations for "ece:Z3146"**  

| ID | Annotation | EC number |
| --- | --- | --- |
| ece:Z3135 | invasin; K13735 adhesin/invasin |  |
| ece:Z3136 | hypothetical protein |  |
| ece:Z3137 | hypothetical protein |  |
| ece:Z3138 | shiA; shikimate transporter; K08172 MFS transporter, MHS family, shikimate and dehydroshikimate transport protein |  |
| ece:Z3139 | amn; AMP nucleosidase (EC:3.2.2.4); K01241 AMP nucleosidase [EC:3.2.2.4] | ec:3.2.2.4 |
| ece:Z3140 | hypothetical protein |  |
| ece:Z3141 | asnW; tRNA-Asn; K14220 tRNA Asn |  |
| ece:Z3143 | hypothetical protein |  |
| ece:Z3144 | hypothetical protein |  |
| ece:Z3145 | asnU; tRNA-Asn; K14220 tRNA Asn |  |
| ece:Z3146 | cbl; transcriptional regulator Cbl; K13635 LysR family transcriptional regulator, cys regulon transcriptional activator |  |
| ece:Z3147 | nac; nitrogen assimilation transcriptional regulator |  |
| ece:Z3149 | asnV; tRNA-Asn; K14220 tRNA Asn |  |
| ece:Z3150 | erfK; hypothetical protein; K16291 L,D-transpeptidase |  |
| ece:Z3151 | cobT; nicotinate-nucleotide--dimethylbenzimidazole phosphoribosyltransferase (EC:2.4.2.21); K00768 nicotinate-nucleotide--dimethylbenzimidazole phosphoribosyltransferase [EC:2.4.2.21] | ec:2.4.2.21 |
| ece:Z3152 | cobS; cobalamin synthase; K02233 adenosylcobinamide-GDP ribazoletransferase [EC:2.7.8.26] | ec:2.7.8.26 |
| ece:Z3153 | cobU; adenosylcobinamide kinase (EC:2.7.1.156 2.7.7.62); K02231 adenosylcobinamide kinase / adenosylcobinamide-phosphate guanylyltransferase [EC:2.7.1.156 2.7.7.62] | ec:2.7.7.62 ec:2.7.1.156 |
| ece:Z3154 | hypothetical protein; K07483 transposase |  |
| ece:Z3155 | hypothetical protein; K07484 transposase |  |
| ece:Z3156 | hypothetical protein; K07484 transposase |  |
| ece:Z3159 | outer membrane receptor for iron compound or colicin; K16087 hemoglobin/transferrin/lactoferrin receptor protein |  |

  
**Neighborhood Representations for "ecf:ECH74115\_2831"**  

| ID | Annotation | EC number |
| --- | --- | --- |
| ecf:ECH74115\_2821 | hypothetical protein; K13735 adhesin/invasin |  |
| ecf:ECH74115\_2822 | hypothetical protein |  |
| ecf:ECH74115\_2823 | shiA; shikimate transporter; K08172 MFS transporter, MHS family, shikimate and dehydroshikimate transport protein |  |
| ecf:ECH74115\_2824 | amn; AMP nucleosidase (EC:3.2.2.4); K01241 AMP nucleosidase [EC:3.2.2.4] | ec:3.2.2.4 |
| ecf:ECH74115\_2825 | hypothetical protein |  |
| ecf:ECH74115\_2826 | hypothetical protein |  |
| ecf:ECH74115\_2827 | hypothetical protein |  |
| ecf:ECH74115\_2828 | tRNA-Asn; K14220 tRNA Asn |  |
| ecf:ECH74115\_2829 | hypothetical protein |  |
| ecf:ECH74115\_2830 | tRNA-Asn; K14220 tRNA Asn |  |
| ecf:ECH74115\_2831 | cbl; transcriptional regulator Cbl; K13635 LysR family transcriptional regulator, cys regulon transcriptional activator |  |
| ecf:ECH74115\_2832 | nac; nitrogen assimilation transcriptional regulator |  |
| ecf:ECH74115\_2833 | hypothetical protein |  |
| ecf:ECH74115\_2834 | tRNA-Asn; K14220 tRNA Asn |  |
| ecf:ECH74115\_2835 | hypothetical protein; K16291 L,D-transpeptidase |  |
| ecf:ECH74115\_2836 | cobT; nicotinate-nucleotide--dimethylbenzimidazole phosphoribosyltransferase (EC:2.4.2.21); K00768 nicotinate-nucleotide--dimethylbenzimidazole phosphoribosyltransferase [EC:2.4.2.21] | ec:2.4.2.21 |
| ecf:ECH74115\_2837 | cobS; cobalamin synthase (EC:2.7.8.26); K02233 adenosylcobinamide-GDP ribazoletransferase [EC:2.7.8.26] | ec:2.7.8.26 |
| ecf:ECH74115\_2838 | cobU; pseudogene |  |
| ecf:ECH74115\_2839 | pseudogene |  |
| ecf:ECH74115\_2840 | IS66 family element, orf2; K07484 transposase |  |
| ecf:ECH74115\_2841 | IS66 family element, transposase; K07484 transposase |  |

  
**Neighborhood Representations for "ecs:ECs2783"**  

| ID | Annotation | EC number |
| --- | --- | --- |
| ecs:ECs2774 | hypothetical protein; K09933 hypothetical protein |  |
| ecs:ECs5475 | asnT; tRNA-Asn; K14220 tRNA Asn |  |
| ecs:ECs2775 | hypothetical protein; K13735 adhesin/invasin |  |
| ecs:ECs2776 | hypothetical protein; K13735 adhesin/invasin |  |
| ecs:ECs2777 | hypothetical protein |  |
| ecs:ECs2778 | shikimate transporter; K08172 MFS transporter, MHS family, shikimate and dehydroshikimate transport protein |  |
| ecs:ECs2779 | AMP nucleosidase (EC:3.2.2.4); K01241 AMP nucleosidase [EC:3.2.2.4] | ec:3.2.2.4 |
| ecs:ECs2780 | hypothetical protein |  |
| ecs:ECs5476 | asnW; tRNA-Asn; K14220 tRNA Asn |  |
| ecs:ECs5477 | asnU; tRNA-Asn; K14220 tRNA Asn |  |
| ecs:ECs2783 | cbl; transcriptional regulator Cbl; K13635 LysR family transcriptional regulator, cys regulon transcriptional activator |  |
| ecs:ECs2784 | nitrogen assimilation transcriptional regulator |  |
| ecs:ECs5478 | asnV; tRNA-Asn; K14220 tRNA Asn |  |
| ecs:ECs2785 | hypothetical protein; K16291 L,D-transpeptidase |  |
| ecs:ECs2786 | cobT; nicotinate-nucleotide--dimethylbenzimidazole phosphoribosyltransferase (EC:2.4.2.21); K00768 nicotinate-nucleotide--dimethylbenzimidazole phosphoribosyltransferase [EC:2.4.2.21] | ec:2.4.2.21 |
| ecs:ECs2787 | cobS; cobalamin synthase; K02233 adenosylcobinamide-GDP ribazoletransferase [EC:2.7.8.26] | ec:2.7.8.26 |
| ecs:ECs2788 | cobU; adenosylcobinamide kinase (EC:2.7.1.156 2.7.7.62); K02231 adenosylcobinamide kinase / adenosylcobinamide-phosphate guanylyltransferase [EC:2.7.1.156 2.7.7.62] | ec:2.7.7.62 ec:2.7.1.156 |
| ecs:ECs2789 | hypothetical protein; K07483 transposase |  |
| ecs:ECs2790 | hypothetical protein; K07484 transposase |  |
| ecs:ECs2791 | hypothetical protein; K07484 transposase |  |
| ecs:ECs2792 | hypothetical protein; K16087 hemoglobin/transferrin/lactoferrin receptor protein |  |

  
**Neighborhood Representations for "ecz:ECS88\_2053"**  

| ID | Annotation | EC number |
| --- | --- | --- |
| ecz:ECS88\_2041 | irp; salicyl-AMP ligase YbtE; K04783 yersiniabactin salicyl-AMP ligase [EC:6.3.2.-] |  |
| ecz:ECS88\_2043 | fyuA; Yersiniabactin/pesticin outer membrane receptor (IRPC); K15721 pesticin/yersiniabactin receptor |  |
| ecz:ECS88\_2044 | yeeJ; pseudogene |  |
| ecz:ECS88\_2045 | yeeJ; pseudogene |  |
| ecz:ECS88\_2046 | yeeJ; pseudogene |  |
| ecz:ECS88\_2047 | hypothetical protein |  |
| ecz:ECS88\_2049 | shiA; shikimate transporter; K08172 MFS transporter, MHS family, shikimate and dehydroshikimate transport protein |  |
| ecz:ECS88\_2050 | amn; AMP nucleosidase (EC:3.2.2.4); K01241 AMP nucleosidase [EC:3.2.2.4] | ec:3.2.2.4 |
| ecz:ECS88\_2051 | yeeN; hypothetical protein |  |
| ecz:ECS88\_2052 | yeeO; hypothetical protein |  |
| ecz:ECS88\_2053 | cbl; transcriptional regulator Cbl; K13635 LysR family transcriptional regulator, cys regulon transcriptional activator |  |
| ecz:ECS88\_2054 | nac; nitrogen assimilation transcriptional regulator |  |
| ecz:ECS88\_2055 | erfK; hypothetical protein; K16291 L,D-transpeptidase |  |
| ecz:ECS88\_2056 | cobT; nicotinate-nucleotide--dimethylbenzimidazole phosphoribosyltransferase (EC:2.4.2.21); K00768 nicotinate-nucleotide--dimethylbenzimidazole phosphoribosyltransferase [EC:2.4.2.21] | ec:2.4.2.21 |
| ecz:ECS88\_2057 | cobS; cobalamin synthase; K02233 adenosylcobinamide-GDP ribazoletransferase [EC:2.7.8.26] | ec:2.7.8.26 |
| ecz:ECS88\_2058 | cobU; adenosylcobinamide kinase/adenosylcobinamide-phosphate guanylyltransferase; K02231 adenosylcobinamide kinase / adenosylcobinamide-phosphate guanylyltransferase [EC:2.7.1.156 2.7.7.62] | ec:2.7.7.62 ec:2.7.1.156 |
| ecz:ECS88\_2059 | pseudogene |  |
| ecz:ECS88\_2061 | hypothetical protein |  |
| ecz:ECS88\_2063 | transposase, IS3 family |  |
| ecz:ECS88\_2064 | hypothetical protein |  |
| ecz:ECS88\_2065 | hypothetical protein |  |

  
**Neighborhood Representations for "eok:G2583\_2497"**  

| ID | Annotation | EC number |
| --- | --- | --- |
| eok:G2583\_2487 | yeeJ; pseudogene |  |
| eok:G2583\_2488 | factor; K13735 adhesin/invasin |  |
| eok:G2583\_2489 | hypothetical protein |  |
| eok:G2583\_2490 | shiA; shikimate transporter; K08172 MFS transporter, MHS family, shikimate and dehydroshikimate transport protein |  |
| eok:G2583\_2491 | amn; AMP nucleosidase; K01241 AMP nucleosidase [EC:3.2.2.4] | ec:3.2.2.4 |
| eok:G2583\_2492 | yeeN; hypothetical protein |  |
| eok:G2583\_2493 | tRNA-Asn; K14220 tRNA Asn |  |
| eok:G2583\_2494 | yeeO; pseudogene |  |
| eok:G2583\_2495 | yeeO; MATE efflux family protein |  |
| eok:G2583\_2496 | tRNA-Asn; K14220 tRNA Asn |  |
| eok:G2583\_2497 | cbl; transcriptional regulator Cbl; K13635 LysR family transcriptional regulator, cys regulon transcriptional activator |  |
| eok:G2583\_2498 | nac; nitrogen assimilation regulatory protein Nac |  |
| eok:G2583\_2499 | tRNA-Asn; K14220 tRNA Asn |  |
| eok:G2583\_2500 | erfK; hypothetical protein; K16291 L,D-transpeptidase |  |
| eok:G2583\_2501 | cobT; nicotinate-nucleotide--dimethylbenzimidazole phosphoribosyltransferase (NN:DBI PRT) (N(1)-alpha-phosphoribosyltransferase); K00768 nicotinate-nucleotide--dimethylbenzimidazole phosphoribosyltransferase [EC:2.4.2.21] | ec:2.4.2.21 |
| eok:G2583\_2502 | cobS; cobalamin synthase; K02233 adenosylcobinamide-GDP ribazoletransferase [EC:2.7.8.26] | ec:2.7.8.26 |
| eok:G2583\_2503 | cobU; cobalamin synthase; K02231 adenosylcobinamide kinase / adenosylcobinamide-phosphate guanylyltransferase [EC:2.7.1.156 2.7.7.62] | ec:2.7.7.62 ec:2.7.1.156 |
| eok:G2583\_2504 | yoeE; outer membrane receptor for iron compound or colicin; K16087 hemoglobin/transferrin/lactoferrin receptor protein |  |
| eok:G2583\_2505 | ibrB; ParB-like nuclease |  |
| eok:G2583\_2506 | ibrA; Immunoglobulin-binding regulator A-like protein |  |
| eok:G2583\_2507 | insN; hypothetical protein; K07483 transposase |  |

  
**Neighborhood Representations for "etw:ECSP\_2651"**  

| ID | Annotation | EC number |
| --- | --- | --- |
| etw:ECSP\_2641 | yeeJ; adhesin; K13735 adhesin/invasin |  |
| etw:ECSP\_2642 | hypothetical protein |  |
| etw:ECSP\_2643 | shiA; shikimate transporter; K08172 MFS transporter, MHS family, shikimate and dehydroshikimate transport protein |  |
| etw:ECSP\_2644 | amn; AMP nucleosidase; K01241 AMP nucleosidase [EC:3.2.2.4] | ec:3.2.2.4 |
| etw:ECSP\_2645 | yeeN; hypothetical protein |  |
| etw:ECSP\_2646 | hypothetical protein |  |
| etw:ECSP\_2647 | hypothetical protein |  |
| etw:ECSP\_2648 | tRNA-Asn; K14220 tRNA Asn |  |
| etw:ECSP\_2649 | hypothetical protein |  |
| etw:ECSP\_2650 | tRNA-Asn; K14220 tRNA Asn |  |
| etw:ECSP\_2651 | cbl; transcriptional regulator Cbl; K13635 LysR family transcriptional regulator, cys regulon transcriptional activator |  |
| etw:ECSP\_2652 | nac; nitrogen assimilation transcriptional regulator |  |
| etw:ECSP\_2653 | hypothetical protein |  |
| etw:ECSP\_2654 | tRNA-Asn; K14220 tRNA Asn |  |
| etw:ECSP\_2655 | erfK; hypothetical protein; K16291 L,D-transpeptidase |  |
| etw:ECSP\_2656 | cobT; nicotinate-nucleotide--dimethylbenzimidazole phosphoribosyltransferase; K00768 nicotinate-nucleotide--dimethylbenzimidazole phosphoribosyltransferase [EC:2.4.2.21] | ec:2.4.2.21 |
| etw:ECSP\_2657 | cobS; cobalamin synthase; K02233 adenosylcobinamide-GDP ribazoletransferase [EC:2.7.8.26] | ec:2.7.8.26 |
| etw:ECSP\_2658 | cobU; adenosylcobinamide kinase; K02231 adenosylcobinamide kinase / adenosylcobinamide-phosphate guanylyltransferase [EC:2.7.1.156 2.7.7.62] | ec:2.7.7.62 ec:2.7.1.156 |
| etw:ECSP\_2659 | transposase, ISEc8; K07483 transposase |  |
| etw:ECSP\_2660 | transposase; K07484 transposase |  |
| etw:ECSP\_2661 | transposase, ISEc8; K07484 transposase |  |

  
**Neighborhood Representations for "dda:Dd703\_1289"**  

| ID | Annotation | EC number |
| --- | --- | --- |
| dda:Dd703\_1279 | hydrogenase 4 subunit H; K12143 hydrogenase-4 component H |  |
| dda:Dd703\_1280 | NADH dehydrogenase (ubiquinone) 30 kDa subunit; K12142 hydrogenase-4 component G [EC:1.-.-.-] |  |
| dda:Dd703\_1281 | hydrogenase 4 subunit F; K12141 hydrogenase-4 component F [EC:1.-.-.-] |  |
| dda:Dd703\_1282 | hyfE; hydrogenase 4 membrane subunit; K12140 hydrogenase-4 component E [EC:1.-.-.-] |  |
| dda:Dd703\_1283 | hydrogenase 4 subunit D; K12139 hydrogenase-4 component D [EC:1.-.-.-] |  |
| dda:Dd703\_1284 | respiratory-chain NADH dehydrogenase subunit 1; K12138 hydrogenase-4 component C [EC:1.-.-.-] |  |
| dda:Dd703\_1285 | hydrogenase 4 subunit B; K12137 hydrogenase-4 component B [EC:1.-.-.-] |  |
| dda:Dd703\_1286 | 4Fe-4S ferredoxin; K12136 hydrogenase-4 component A [EC:1.-.-.-] |  |
| dda:Dd703\_1287 | hydrogenase assembly chaperone HypC/HupF; K04653 hydrogenase expression/formation protein HypC |  |
| dda:Dd703\_1288 | hypothetical protein |  |
| dda:Dd703\_1289 | transcriptional regulator CysB-like protein; K13635 LysR family transcriptional regulator, cys regulon transcriptional activator |  |
| dda:Dd703\_1290 | sodium:dicarboxylate symporter; K11102 proton glutamate symport protein |  |
| dda:Dd703\_1291 | AraC family transcriptional regulator |  |
| dda:Dd703\_1292 | 2-isopropylmalate synthase; K01649 2-isopropylmalate synthase [EC:2.3.3.13] | ec:2.3.3.13 |
| dda:Dd703\_1293 | hypothetical protein |  |
| dda:Dd703\_1294 | hypothetical protein |  |
| dda:Dd703\_1295 | hypothetical protein |  |
| dda:Dd703\_1296 | iron-containing alcohol dehydrogenase; K00005 glycerol dehydrogenase [EC:1.1.1.6] | ec:1.1.1.6 |
| dda:Dd703\_1297 | binding-protein-dependent transporters inner membrane component; K02050 NitT/TauT family transport system permease protein |  |
| dda:Dd703\_1298 | ABC transporter; K02049 NitT/TauT family transport system ATP-binding protein |  |
| dda:Dd703\_1299 | ABC transporter substrate-binding protein; K02051 NitT/TauT family transport system substrate-binding protein |  |

  
**Neighborhood Representations for "ddc:Dd586\_2611"**  

| ID | Annotation | EC number |
| --- | --- | --- |
| ddc:Dd586\_2601 | LysR family transcriptional regulator |  |
| ddc:Dd586\_2602 | short-chain dehydrogenase/reductase SDR |  |
| ddc:Dd586\_2603 | hypothetical protein |  |
| ddc:Dd586\_2604 | hypothetical protein |  |
| ddc:Dd586\_2605 | putrescine aminotransferase (EC:2.6.1.82); K09251 putrescine aminotransferase [EC:2.6.1.82] | ec:2.6.1.82 |
| ddc:Dd586\_2606 | 1-pyrroline dehydrogenase (EC:1.2.1.19); K00137 aminobutyraldehyde dehydrogenase [EC:1.2.1.19] | ec:1.2.1.19 |
| ddc:Dd586\_2607 | pseudogene |  |
| ddc:Dd586\_2608 | Dcu family anaerobic c4-dicarboxylate antiporter; K07792 anaerobic C4-dicarboxylate transporter DcuB |  |
| ddc:Dd586\_2609 | oxidoreductase domain-containing protein |  |
| ddc:Dd586\_2610 | sodium:dicarboxylate symporter; K11102 proton glutamate symport protein |  |
| ddc:Dd586\_2611 | LysR family transcriptional regulator; K13635 LysR family transcriptional regulator, cys regulon transcriptional activator |  |
| ddc:Dd586\_2612 | methyltransferase FkbM family |  |
| ddc:Dd586\_2613 | hydrogenase assembly chaperone HypC/HupF; K04653 hydrogenase expression/formation protein HypC |  |
| ddc:Dd586\_2614 | 4Fe-4S ferredoxin iron-sulfur-binding domain-containing protein; K12136 hydrogenase-4 component A [EC:1.-.-.-] |  |
| ddc:Dd586\_2615 | NADH/Ubiquinone/plastoquinone (complex I); K12137 hydrogenase-4 component B [EC:1.-.-.-] |  |
| ddc:Dd586\_2616 | respiratory-chain NADH dehydrogenase subunit 1; K12138 hydrogenase-4 component C [EC:1.-.-.-] |  |
| ddc:Dd586\_2617 | NADH/Ubiquinone/plastoquinone (complex I); K12139 hydrogenase-4 component D [EC:1.-.-.-] |  |
| ddc:Dd586\_2618 | NADH-ubiquinone oxidoreductase chain 4L; K12140 hydrogenase-4 component E [EC:1.-.-.-] |  |
| ddc:Dd586\_2619 | NADH/Ubiquinone/plastoquinone (complex I); K12141 hydrogenase-4 component F [EC:1.-.-.-] |  |
| ddc:Dd586\_2620 | NADH dehydrogenase (ubiquinone) 30 kDa subunit; K12142 hydrogenase-4 component G [EC:1.-.-.-] |  |
| ddc:Dd586\_2621 | 4Fe-4S ferredoxin iron-sulfur-binding domain-containing protein; K00540 [EC:1.-.-.-] |  |

  
**Neighborhood Representations for "eck:EC55989\_2222"**  

| ID | Annotation | EC number |
| --- | --- | --- |
| eck:EC55989\_2212 | fyuA; Yersiniabactin/pesticin outer membrane receptor (IRPC); K15721 pesticin/yersiniabactin receptor |  |
| eck:EC55989\_2213 | yeeJ; pseudogene |  |
| eck:EC55989\_2214 | yeeJ; pseudogene |  |
| eck:EC55989\_2215 | yeeJ; pseudogene |  |
| eck:EC55989\_2216 | yeeJ; pseudogene |  |
| eck:EC55989\_2217 | hypothetical protein |  |
| eck:EC55989\_2218 | shiA; shikimate transporter; K08172 MFS transporter, MHS family, shikimate and dehydroshikimate transport protein |  |
| eck:EC55989\_2219 | amn; AMP nucleosidase (EC:3.2.2.4); K01241 AMP nucleosidase [EC:3.2.2.4] | ec:3.2.2.4 |
| eck:EC55989\_2220 | yeeN; hypothetical protein |  |
| eck:EC55989\_2221 | yeeO; hypothetical protein |  |
| eck:EC55989\_2222 | cbl; transcriptional regulator Cbl; K13635 LysR family transcriptional regulator, cys regulon transcriptional activator |  |
| eck:EC55989\_2223 | nac; nitrogen assimilation transcriptional regulator |  |
| eck:EC55989\_2224 | erfK; hypothetical protein; K16291 L,D-transpeptidase |  |
| eck:EC55989\_2225 | cobT; nicotinate-nucleotide--dimethylbenzimidazole phosphoribosyltransferase (EC:2.4.2.21); K00768 nicotinate-nucleotide--dimethylbenzimidazole phosphoribosyltransferase [EC:2.4.2.21] | ec:2.4.2.21 |
| eck:EC55989\_2226 | cobS; cobalamin synthase; K02233 adenosylcobinamide-GDP ribazoletransferase [EC:2.7.8.26] | ec:2.7.8.26 |
| eck:EC55989\_2227 | cobU; adenosylcobinamide kinase; K02231 adenosylcobinamide kinase / adenosylcobinamide-phosphate guanylyltransferase [EC:2.7.1.156 2.7.7.62] | ec:2.7.7.62 ec:2.7.1.156 |
| eck:EC55989\_2228 | pseudogene |  |
| eck:EC55989\_2229 | hypothetical protein |  |
| eck:EC55989\_2231 | hypothetical protein |  |
| eck:EC55989\_2232 | carbohydrate kinase (EC:2.7.1.15) |  |
| eck:EC55989\_2233 | hypothetical protein |  |

  
**Neighborhood Representations for "mfa:Mfla\_1669"**  

| ID | Annotation | EC number |
| --- | --- | --- |
| mfa:Mfla\_1659 | hypothetical protein |  |
| mfa:Mfla\_1660 | methylene tetrahydromethanopterin dehydrogenase/methylenetetrahydrofolate dehydrogenase; K10714 methylene-tetrahydromethanopterin dehydrogenase [EC:1.5.1.-] |  |
| mfa:Mfla\_1661 | beta-ribofuranosylaminobenzene 5'-phosphate synthase |  |
| mfa:Mfla\_1662 | formylmethanofuran dehydrogenase, subunit B (EC:1.2.99.5); K00201 formylmethanofuran dehydrogenase subunit B [EC:1.2.99.5] | ec:1.2.99.5 |
| mfa:Mfla\_1663 | formylmethanofuran dehydrogenase, subunit A (EC:1.2.99.5); K00200 formylmethanofuran dehydrogenase subunit A [EC:1.2.99.5] | ec:1.2.99.5 |
| mfa:Mfla\_1664 | formylmethanofuran--tetrahydromethanopterin formyltransferase (EC:2.3.1.101); K00672 formylmethanofuran--tetrahydromethanopterin N-formyltransferase [EC:2.3.1.101] | ec:2.3.1.101 |
| mfa:Mfla\_1665 | formylmethanofuran dehydrogenase, subunit C (EC:1.2.99.5); K00202 formylmethanofuran dehydrogenase subunit C [EC:1.2.99.5] | ec:1.2.99.5 |
| mfa:Mfla\_1666 | OmpA/MotB |  |
| mfa:Mfla\_1667 | hypothetical protein |  |
| mfa:Mfla\_1668 | hypothetical protein; K06915 |  |
| mfa:Mfla\_1669 | transcriptional regulator CysB-like protein; K13634 LysR family transcriptional regulator, cys regulon transcriptional activator |  |
| mfa:Mfla\_1670 | hypothetical protein; K07090 |  |
| mfa:Mfla\_1671 | nitrite and sulphite reductase 4Fe-4S region; K00381 sulfite reductase (NADPH) hemoprotein beta-component [EC:1.8.1.2] | ec:1.8.1.2 |
| mfa:Mfla\_1672 | hypothetical protein |  |
| mfa:Mfla\_1673 | sulfate adenylyltransferase subunit 2 (EC:2.7.7.4); K00957 sulfate adenylyltransferase subunit 2 [EC:2.7.7.4] | ec:2.7.7.4 |
| mfa:Mfla\_1674 | sulfate adenylyltransferase subunit 1 (EC:2.7.7.4); K00956 sulfate adenylyltransferase subunit 1 [EC:2.7.7.4] | ec:2.7.7.4 |
| mfa:Mfla\_1675 | 4Fe-4S ferredoxin, iron-sulfur binding; K05524 ferredoxin |  |
| mfa:Mfla\_1676 | trans-2-enoyl-CoA reductase; K00209 enoyl-[acyl-carrier protein] reductase / trans-2-enoyl-CoA reductase (NAD+) [EC:1.3.1.- 1.3.1.44] | ec:1.3.1.44 |
| mfa:Mfla\_1677 | Bcr/CflA subfamily drug resistance transporter; K07552 MFS transporter, DHA1 family, bicyclomycin/chloramphenicol resistance protein |  |
| mfa:Mfla\_1678 | cyclopropane-fatty-acyl-phospholipid synthase; K00574 cyclopropane-fatty-acyl-phospholipid synthase [EC:2.1.1.79] | ec:2.1.1.79 |
| mfa:Mfla\_1679 | phosphoadenylylsulfate reductase (thioredoxin) (EC:1.8.4.8); K00390 phosphoadenosine phosphosulfate reductase [EC:1.8.4.8] | ec:1.8.4.8 |

  
**Neighborhood Representations for "ssn:SSON\_2042"**  

| ID | Annotation | EC number |
| --- | --- | --- |
| ssn:SSON\_2032 | hypothetical protein |  |
| ssn:SSON\_2033 | yodB; pseudogene |  |
| ssn:SSON\_2034 | tRNA-Ser; K14233 tRNA Ser |  |
| ssn:SSON\_2035 | hypothetical protein; K09933 hypothetical protein |  |
| ssn:SSON\_2036 | tRNA-Asn; K14220 tRNA Asn |  |
| ssn:SSON\_2037 | pseudogene |  |
| ssn:SSON\_2038 | IS911 ORF2 |  |
| ssn:SSON\_2039 | IS630 orf |  |
| ssn:SSON\_2040 | hypothetical protein |  |
| ssn:SSON\_2041 | IS1 ORF; K07480 insertion element IS1 protein InsB |  |
| ssn:SSON\_2042 | cbl; transcriptional regulator Cbl; K13635 LysR family transcriptional regulator, cys regulon transcriptional activator |  |
| ssn:SSON\_2043 | tRNA-Asn; K14220 tRNA Asn |  |
| ssn:SSON\_2044 | yeeO; hypothetical protein |  |
| ssn:SSON\_2045 | tRNA-Asn; K14220 tRNA Asn |  |
| ssn:SSON\_2046 | hypothetical protein |  |
| ssn:SSON\_2047 | amn; AMP nucleosidase (EC:3.2.2.4); K01241 AMP nucleosidase [EC:3.2.2.4] | ec:3.2.2.4 |
| ssn:SSON\_2048 | IS1 ORF; K07480 insertion element IS1 protein InsB |  |
| ssn:SSON\_2049 | nac; nitrogen assimilation transcriptional regulator |  |
| ssn:SSON\_2050 | tRNA-Asn; K14220 tRNA Asn |  |
| ssn:SSON\_2051 | erfK; pseudogene |  |
| ssn:SSON\_2052 | cobT; nicotinate-nucleotide--dimethylbenzimidazole phosphoribosyltransferase (EC:2.4.2.21); K00768 nicotinate-nucleotide--dimethylbenzimidazole phosphoribosyltransferase [EC:2.4.2.21] | ec:2.4.2.21 |

  
**Neighborhood Representations for "ecq:ECED1\_2324"**  

| ID | Annotation | EC number |
| --- | --- | --- |
| ecq:ECED1\_2314 | hypothetical protein |  |
| ecq:ECED1\_2315 | hypothetical protein |  |
| ecq:ECED1\_2316 | hypothetical protein |  |
| ecq:ECED1\_2317 | hns; DNA-binding protein H-NS (Histone-like protein HLP-II); K03746 DNA-binding protein H-NS |  |
| ecq:ECED1\_2318 | hypothetical protein |  |
| ecq:ECED1\_2319 | hypothetical protein |  |
| ecq:ECED1\_2320 | hypothetical protein |  |
| ecq:ECED1\_2321 | hypothetical protein |  |
| ecq:ECED1\_2322 | putative DNA binding protein from phage origin; K07733 prophage regulatory protein |  |
| ecq:ECED1\_2323 | hypothetical protein |  |
| ecq:ECED1\_2324 | cbl; transcriptional regulator Cbl; K13635 LysR family transcriptional regulator, cys regulon transcriptional activator |  |
| ecq:ECED1\_2325 | nac; nitrogen assimilation transcriptional regulator |  |
| ecq:ECED1\_2326 | erfK; hypothetical protein; K16291 L,D-transpeptidase |  |
| ecq:ECED1\_2327 | cobT; nicotinate-nucleotide--dimethylbenzimidazole phosphoribosyltransferase (EC:2.4.2.21); K00768 nicotinate-nucleotide--dimethylbenzimidazole phosphoribosyltransferase [EC:2.4.2.21] | ec:2.4.2.21 |
| ecq:ECED1\_2328 | cobS; cobalamin synthase; K02233 adenosylcobinamide-GDP ribazoletransferase [EC:2.7.8.26] | ec:2.7.8.26 |
| ecq:ECED1\_2329 | cobU; adenosylcobinamide kinase/adenosylcobinamide-phosphate guanylyltransferase; K02231 adenosylcobinamide kinase / adenosylcobinamide-phosphate guanylyltransferase [EC:2.7.1.156 2.7.7.62] | ec:2.7.7.62 ec:2.7.1.156 |
| ecq:ECED1\_2330 | pseudogene |  |
| ecq:ECED1\_2332 | hypothetical protein |  |
| ecq:ECED1\_2333 | pseudogene |  |
| ecq:ECED1\_2334 | transposase ORF A, IS911 (fragment) |  |
| ecq:ECED1\_2335 | hypothetical protein |  |

  
**Neighborhood Representations for "aci:ACIAD2597"**  

| ID | Annotation | EC number |
| --- | --- | --- |
| aci:ACIAD2586 | serine protease; K01362 [EC:3.4.21.-] |  |
| aci:ACIAD2587 | nadB; L-aspartate oxidase (EC:1.4.3.16); K00278 L-aspartate oxidase [EC:1.4.3.16] | ec:1.4.3.16 |
| aci:ACIAD2588 | tmk; thymidylate kinase (EC:2.7.4.9); K00943 dTMP kinase [EC:2.7.4.9] | ec:2.7.4.9 |
| aci:ACIAD2589 | periplasmic solute-binding protein; K07082 UPF0755 protein |  |
| aci:ACIAD2590 | pabC; 4-amino-4-deoxychorismate lyase (EC:4.-.-.-); K02619 4-amino-4-deoxychorismate lyase [EC:4.1.3.38] | ec:4.1.3.38 |
| aci:ACIAD2591 | cysP; sulfate ABC transporter periplasmic substrate-binding protein; K02048 sulfate transport system substrate-binding protein |  |
| aci:ACIAD2592 | esterase; K07002 |  |
| aci:ACIAD2594 | cysT; sulfate ABC transporter; K02046 sulfate transport system permease protein |  |
| aci:ACIAD2595 | cysW; sulfate ABC transporter; K02047 sulfate transport system permease protein |  |
| aci:ACIAD2596 | cysA; sulfate permease A protein chromate resistance ABC transporter ATP-binding protein; K02045 sulfate transport system ATP-binding protein [EC:3.6.3.25] | ec:3.6.3.25 |
| aci:ACIAD2597 | cbl; CysB family transcriptional regulator; K13635 LysR family transcriptional regulator, cys regulon transcriptional activator |  |
| aci:ACIAD2598 | porin |  |
| aci:ACIAD2599 | dapD; 2,3,4,5-tetrahydropyridine-2,6-carboxylate N-succinyltransferase (EC:2.3.1.117); K00674 2,3,4,5-tetrahydropyridine-2-carboxylate N-succinyltransferase [EC:2.3.1.117] | ec:2.3.1.117 |
| aci:ACIAD2600 | radical activating enzyme; K10026 7-carboxy-7-deazaguanine synthase [EC:4.3.99.3] | ec:4.3.99.3 |
| aci:ACIAD2601 | hypothetical protein; K06920 7-cyano-7-deazaguanine synthase [EC:6.3.4.20] | ec:6.3.4.20 |
| aci:ACIAD2602 | hypothetical protein |  |
| aci:ACIAD2603 | bcp; bacterioferritin comigratory protein; K03564 peroxiredoxin Q/BCP [EC:1.11.1.15] | ec:1.11.1.15 |
| aci:ACIAD2604 | hypothetical protein |  |
| aci:ACIAD2606 | nicotinamide-nucleotide adenylyltransferase (EC:2.7.7.1) |  |
| aci:ACIAD2607 | enoyl-CoA hydratase |  |
| aci:ACIAD2608 | nitrorecductase |  |

  
**Neighborhood Representations for "ecl:EcolC\_1658"**  

| ID | Annotation | EC number |
| --- | --- | --- |
| ecl:EcolC\_1648 | ribulose-phosphate 3-epimerase (EC:5.1.3.1); K01783 ribulose-phosphate 3-epimerase [EC:5.1.3.1] | ec:5.1.3.1 |
| ecl:EcolC\_1649 | short-chain dehydrogenase/reductase SDR |  |
| ecl:EcolC\_1650 | sugar isomerase (SIS); K08094 6-phospho-3-hexuloisomerase [EC:5.3.1.27] | ec:5.3.1.27 |
| ecl:EcolC\_1651 | hypothetical protein |  |
| ecl:EcolC\_1652 | hypothetical protein |  |
| ecl:EcolC\_1653 | YD repeat-containing protein |  |
| ecl:EcolC\_1654 | hypothetical protein |  |
| ecl:EcolC\_1655 | hypothetical protein |  |
| ecl:EcolC\_1656 | pseudogene |  |
| ecl:EcolC\_1657 | nitrogen assimilation transcriptional regulator |  |
| ecl:EcolC\_1658 | cbl; transcriptional regulator Cbl; K13635 LysR family transcriptional regulator, cys regulon transcriptional activator |  |
| ecl:EcolC\_1659 | hypothetical protein |  |
| ecl:EcolC\_1660 | hypothetical protein |  |
| ecl:EcolC\_1661 | AMP nucleosidase (EC:3.2.2.4); K01241 AMP nucleosidase [EC:3.2.2.4] | ec:3.2.2.4 |
| ecl:EcolC\_1662 | shikimate transporter; K08172 MFS transporter, MHS family, shikimate and dehydroshikimate transport protein |  |
| ecl:EcolC\_1663 | ADP-heptose--LPS heptosyltransferase-like protein |  |
| ecl:EcolC\_1664 | pseudogene |  |
| ecl:EcolC\_1667 | YD repeat-containing protein |  |
| ecl:EcolC\_1668 | hypothetical protein |  |
| ecl:EcolC\_1669 | pseudogene |  |
| ecl:EcolC\_1670 | hypothetical protein; K09933 hypothetical protein |  |

  
**Neighborhood Representations for "sdy:SDY\_2246"**  

| ID | Annotation | EC number |
| --- | --- | --- |
| sdy:SDY\_2236 | yeeA; hypothetical protein |  |
| sdy:SDY\_2237 | yeeX; hypothetical protein; K09802 hypothetical protein |  |
| sdy:SDY\_2238 | iso-IS1 ORF2; K07480 insertion element IS1 protein InsB |  |
| sdy:SDY\_2239 | hypothetical protein |  |
| sdy:SDY\_2240 | cobU; adenosylcobinamide kinase (EC:2.7.1.156 2.7.7.62); K02231 adenosylcobinamide kinase / adenosylcobinamide-phosphate guanylyltransferase [EC:2.7.1.156 2.7.7.62] | ec:2.7.7.62 ec:2.7.1.156 |
| sdy:SDY\_2241 | cobS; cobalamin synthase; K02233 adenosylcobinamide-GDP ribazoletransferase [EC:2.7.8.26] | ec:2.7.8.26 |
| sdy:SDY\_2242 | cobT; nicotinate-nucleotide--dimethylbenzimidazole phosphoribosyltransferase (EC:2.4.2.21); K00768 nicotinate-nucleotide--dimethylbenzimidazole phosphoribosyltransferase [EC:2.4.2.21] | ec:2.4.2.21 |
| sdy:SDY\_2243 | erfK; hypothetical protein; K16291 L,D-transpeptidase |  |
| sdy:SDY\_2244 | tRNA-Asn; K14220 tRNA Asn |  |
| sdy:SDY\_2245 | nac; nitrogen assimilation transcriptional regulator |  |
| sdy:SDY\_2246 | cbl; transcriptional regulator Cbl; K13635 LysR family transcriptional regulator, cys regulon transcriptional activator |  |
| sdy:SDY\_2247 | tRNA-Asn; K14220 tRNA Asn |  |
| sdy:SDY\_2248 | hypothetical protein |  |
| sdy:SDY\_2249 | hypothetical protein |  |
| sdy:SDY\_2250 | tRNA-Asn; K14220 tRNA Asn |  |
| sdy:SDY\_2251 | insA; IS1 ORF1 |  |
| sdy:SDY\_2252 | insB; IS1 ORF2; K07480 insertion element IS1 protein InsB |  |
| sdy:SDY\_2253 | amn; AMP nucleosidase (EC:3.2.2.4); K01241 AMP nucleosidase [EC:3.2.2.4] | ec:3.2.2.4 |
| sdy:SDY\_2254 | shiA; shikimate transporter; K08172 MFS transporter, MHS family, shikimate and dehydroshikimate transport protein |  |
| sdy:SDY\_2255 | iso-IS1 ORF2 |  |
| sdy:SDY\_2256 | iso-IS1 ORF2 |  |

  
**Neighborhood Representations for "abb:ABBFA\_000909"**  

| ID | Annotation | EC number |
| --- | --- | --- |
| abb:ABBFA\_000899 | Enoyl-CoA hydratase/isomerase family protein |  |
| abb:ABBFA\_000900 | nicotinamide-nucleotide adenylyltransferase (EC:2.7.7.1) |  |
| abb:ABBFA\_000901 | SCP-like extracellular family protein |  |
| abb:ABBFA\_000902 | AhpC/TSA family protein; K03564 peroxiredoxin Q/BCP [EC:1.11.1.15] | ec:1.11.1.15 |
| abb:ABBFA\_000903 | hypothetical protein |  |
| abb:ABBFA\_000904 | hypothetical protein |  |
| abb:ABBFA\_000905 | Queuosine biosynthesis protein queC; K06920 7-cyano-7-deazaguanine synthase [EC:6.3.4.20] | ec:6.3.4.20 |
| abb:ABBFA\_000906 | Radical SAM superfamily protein; K10026 7-carboxy-7-deazaguanine synthase [EC:4.3.99.3] | ec:4.3.99.3 |
| abb:ABBFA\_000907 | dapD; 2,3,4,5-tetrahydropyridine-2,6-carboxylate N-succinyltransferase (EC:2.3.1.117); K00674 2,3,4,5-tetrahydropyridine-2-carboxylate N-succinyltransferase [EC:2.3.1.117] | ec:2.3.1.117 |
| abb:ABBFA\_000908 | hypothetical protein |  |
| abb:ABBFA\_000909 | CysB family transcriptional regulator; K13635 LysR family transcriptional regulator, cys regulon transcriptional activator |  |
| abb:ABBFA\_000910 | Sulfate/thiosulfate import ATP-binding protein cysA(Sulfate-transporting ATPase) (EC:3.6.3.25); K02045 sulfate transport system ATP-binding protein [EC:3.6.3.25] | ec:3.6.3.25 |
| abb:ABBFA\_000911 | cysW; sulfate ABC transporter, permease protein CysW; K02047 sulfate transport system permease protein |  |
| abb:ABBFA\_000912 | cysT; sulfate ABC transporter, permease protein CysT; K02046 sulfate transport system permease protein |  |
| abb:ABBFA\_000913 | hypothetical protein; K07002 |  |
| abb:ABBFA\_000914 | Sulfate-binding protein precursor; K02048 sulfate transport system substrate-binding protein |  |
| abb:ABBFA\_000915 | pabC; aminodeoxychorismate lyase (EC:4.1.3.38); K02619 4-amino-4-deoxychorismate lyase [EC:4.1.3.38] | ec:4.1.3.38 |
| abb:ABBFA\_000916 | aminodeoxychorismate lyase family protein; K07082 UPF0755 protein |  |
| abb:ABBFA\_000917 | tmk; thymidylate kinase (EC:2.7.4.9); K00943 dTMP kinase [EC:2.7.4.9] | ec:2.7.4.9 |
| abb:ABBFA\_000918 | Thioesterase superfamily protein |  |
| abb:ABBFA\_000919 | nadB; L-aspartate oxidase (EC:1.4.3.16); K00278 L-aspartate oxidase [EC:1.4.3.16] | ec:1.4.3.16 |

  
**Neighborhood Representations for "abc:ACICU\_02812"**  

| ID | Annotation | EC number |
| --- | --- | --- |
| abc:ACICU\_02802 | L-aspartate oxidase; K00278 L-aspartate oxidase [EC:1.4.3.16] | ec:1.4.3.16 |
| abc:ACICU\_02803 | hypothetical protein |  |
| abc:ACICU\_02804 | tmk; thymidylate kinase; K00943 dTMP kinase [EC:2.7.4.9] | ec:2.7.4.9 |
| abc:ACICU\_02805 | periplasmic solute-binding protein; K07082 UPF0755 protein |  |
| abc:ACICU\_02806 | branched-chain amino acid aminotransferase/4-amino-4-deoxychorismate lyase; K02619 4-amino-4-deoxychorismate lyase [EC:4.1.3.38] | ec:4.1.3.38 |
| abc:ACICU\_02807 | sulfate ABC transporter periplasmic protein; K02048 sulfate transport system substrate-binding protein |  |
| abc:ACICU\_02808 | esterase of the alpha/beta hydrolase fold; K07002 |  |
| abc:ACICU\_02809 | sulfate ABC transporter permease; K02046 sulfate transport system permease protein |  |
| abc:ACICU\_02810 | sulfate ABC transporter permease; K02047 sulfate transport system permease protein |  |
| abc:ACICU\_02811 | sulfate/molybdate ABC transporter ATPase; K02045 sulfate transport system ATP-binding protein [EC:3.6.3.25] | ec:3.6.3.25 |
| abc:ACICU\_02812 | transcriptional regulator CysB-like protein; K13635 LysR family transcriptional regulator, cys regulon transcriptional activator |  |
| abc:ACICU\_02813 | carO; putative porin protein associated with imipenem resistance |  |
| abc:ACICU\_02814 | dapD; 2,3,4,5-tetrahydropyridine-2,6-carboxylate N-succinyltransferase; K00674 2,3,4,5-tetrahydropyridine-2-carboxylate N-succinyltransferase [EC:2.3.1.117] | ec:2.3.1.117 |
| abc:ACICU\_02815 | organic radical activating protein; K10026 7-carboxy-7-deazaguanine synthase [EC:4.3.99.3] | ec:4.3.99.3 |
| abc:ACICU\_02816 | PP-loop superfamily ATPase; K06920 7-cyano-7-deazaguanine synthase [EC:6.3.4.20] | ec:6.3.4.20 |
| abc:ACICU\_02817 | hypothetical protein |  |
| abc:ACICU\_02818 | ATPase |  |
| abc:ACICU\_02819 | peroxiredoxin; K03564 peroxiredoxin Q/BCP [EC:1.11.1.15] | ec:1.11.1.15 |
| abc:ACICU\_02820 | SCP/PR1 domain-containing proteins |  |
| abc:ACICU\_02821 | nicotinamide-nucleotide adenylyltransferase |  |
| abc:ACICU\_02822 | enoyl-CoA hydratase/carnithine racemase |  |

  
**Neighborhood Representations for "abm:ABSDF0935"**  

| ID | Annotation | EC number |
| --- | --- | --- |
| abm:ABSDF0925 | signal peptide |  |
| abm:ABSDF0926 | bcp; bacterioferritin comigratory protein; K03564 peroxiredoxin Q/BCP [EC:1.11.1.15] | ec:1.11.1.15 |
| abm:ABSDF0927 | pseudogene |  |
| abm:ABSDF0928 | IS982 family transposase |  |
| abm:ABSDF0929 | pseudogene |  |
| abm:ABSDF0930 | hypothetical protein |  |
| abm:ABSDF0931 | hypothetical protein; K06920 7-cyano-7-deazaguanine synthase [EC:6.3.4.20] | ec:6.3.4.20 |
| abm:ABSDF0932 | radical activating enzyme; K10026 7-carboxy-7-deazaguanine synthase [EC:4.3.99.3] | ec:4.3.99.3 |
| abm:ABSDF0933 | dapD; 2,3,4,5-tetrahydropyridine-2,6-carboxylate N-succinyltransferase (EC:2.3.1.117); K00674 2,3,4,5-tetrahydropyridine-2-carboxylate N-succinyltransferase [EC:2.3.1.117] | ec:2.3.1.117 |
| abm:ABSDF0934 | porin protein associated with imipenem resistance |  |
| abm:ABSDF0935 | cbl; CysB family transcriptional regulator; K13635 LysR family transcriptional regulator, cys regulon transcriptional activator |  |
| abm:ABSDF0936 | cysA; sulfate permease; K02045 sulfate transport system ATP-binding protein [EC:3.6.3.25] | ec:3.6.3.25 |
| abm:ABSDF0937 | cysW; sulfate ABC transporter membrane protein; K02047 sulfate transport system permease protein |  |
| abm:ABSDF0938 | cysT; sulfate ABC transporter membrane protein; K02046 sulfate transport system permease protein |  |
| abm:ABSDF0939 | hypothetical protein; K07002 |  |
| abm:ABSDF0940 | cysP; sulfate ABC transporter substrate-binding protein; K02048 sulfate transport system substrate-binding protein |  |
| abm:ABSDF0941 | pabC; 4-amino-4-deoxychorismate lyase (EC:4.-.-.-); K02619 4-amino-4-deoxychorismate lyase [EC:4.1.3.38] | ec:4.1.3.38 |
| abm:ABSDF0942 | hypothetical protein; K07082 UPF0755 protein |  |
| abm:ABSDF0943 | tmk; thymidylate kinase (EC:2.7.4.9); K00943 dTMP kinase [EC:2.7.4.9] | ec:2.7.4.9 |
| abm:ABSDF0944 | thioesterase |  |
| abm:ABSDF0945 | nadB; L-aspartate oxidase (EC:1.4.3.16); K00278 L-aspartate oxidase [EC:1.4.3.16] | ec:1.4.3.16 |

  
**Neighborhood Representations for "abn:AB57\_2978"**  

| ID | Annotation | EC number |
| --- | --- | --- |
| abn:AB57\_2967 | nadB; L-aspartate oxidase (EC:1.4.3.16); K00278 L-aspartate oxidase [EC:1.4.3.16] | ec:1.4.3.16 |
| abn:AB57\_2968 | thioesterase family protein |  |
| abn:AB57\_2969 | tmk; thymidylate kinase (EC:2.7.4.9); K00943 dTMP kinase [EC:2.7.4.9] | ec:2.7.4.9 |
| abn:AB57\_2970 | aminodeoxychorismate lyase; K07082 UPF0755 protein |  |
| abn:AB57\_2971 | pabC; aminodeoxychorismate lyase (EC:4.1.3.38); K02619 4-amino-4-deoxychorismate lyase [EC:4.1.3.38] | ec:4.1.3.38 |
| abn:AB57\_2972 | thiosulfate-binding protein; K02048 sulfate transport system substrate-binding protein |  |
| abn:AB57\_2974 | alpha/beta hydrolase fold protein; K07002 |  |
| abn:AB57\_2975 | cysT; sulfate ABC transporter, permease protein CysT; K02046 sulfate transport system permease protein |  |
| abn:AB57\_2976 | cysW; sulfate ABC transporter, permease protein CysW; K02047 sulfate transport system permease protein |  |
| abn:AB57\_2977 | cysA; sulfate ABC transporter, ATP-binding protein CysA (EC:3.6.3.25); K02045 sulfate transport system ATP-binding protein [EC:3.6.3.25] | ec:3.6.3.25 |
| abn:AB57\_2978 | transcriptional regulator CysB-like protein; K13635 LysR family transcriptional regulator, cys regulon transcriptional activator |  |
| abn:AB57\_2979 | outer membrane protein CarO |  |
| abn:AB57\_2980 | dapD; 2,3,4,5-tetrahydropyridine-2,6-carboxylate N-succinyltransferase (EC:2.3.1.117); K00674 2,3,4,5-tetrahydropyridine-2-carboxylate N-succinyltransferase [EC:2.3.1.117] | ec:2.3.1.117 |
| abn:AB57\_2981 | radical SAM domain protein; K10026 7-carboxy-7-deazaguanine synthase [EC:4.3.99.3] | ec:4.3.99.3 |
| abn:AB57\_2982 | ExsB protein; K06920 7-cyano-7-deazaguanine synthase [EC:6.3.4.20] | ec:6.3.4.20 |
| abn:AB57\_2983 | hypothetical protein |  |
| abn:AB57\_2984 | hypothetical protein |  |
| abn:AB57\_2985 | bacterioferritin comigratory protein; K03564 peroxiredoxin Q/BCP [EC:1.11.1.15] | ec:1.11.1.15 |
| abn:AB57\_2986 | hypothetical protein |  |
| abn:AB57\_2987 | nicotinamide-nucleotide adenylyltransferase |  |
| abn:AB57\_2989 | enoyl-CoA hydratase/isomerase |  |

  
**Neighborhood Representations for "aby:ABAYE0925"**  

| ID | Annotation | EC number |
| --- | --- | --- |
| aby:ABAYE0915 | enoyl-CoA hydratase/isomerase |  |
| aby:ABAYE0916 | nicotinamide-nucleotide adenylyltransferase (EC:2.7.7.18 2.7.7.1) |  |
| aby:ABAYE0917 | signal peptide |  |
| aby:ABAYE0918 | bcp; bacterioferritin comigratory protein; K03564 peroxiredoxin Q/BCP [EC:1.11.1.15] | ec:1.11.1.15 |
| aby:ABAYE0919 | hypothetical protein |  |
| aby:ABAYE0920 | hypothetical protein |  |
| aby:ABAYE0921 | hypothetical protein; K06920 7-cyano-7-deazaguanine synthase [EC:6.3.4.20] | ec:6.3.4.20 |
| aby:ABAYE0922 | radical activating enzyme; K10026 7-carboxy-7-deazaguanine synthase [EC:4.3.99.3] | ec:4.3.99.3 |
| aby:ABAYE0923 | dapD; 2,3,4,5-tetrahydropyridine-2,6-carboxylate N-succinyltransferase (EC:2.3.1.117); K00674 2,3,4,5-tetrahydropyridine-2-carboxylate N-succinyltransferase [EC:2.3.1.117] | ec:2.3.1.117 |
| aby:ABAYE0924 | porin protein associated with imipenem resistance |  |
| aby:ABAYE0925 | cbl; transcriptional regulator CysB-like protein; K13635 LysR family transcriptional regulator, cys regulon transcriptional activator |  |
| aby:ABAYE0926 | cysA; sulfate permease; K02045 sulfate transport system ATP-binding protein [EC:3.6.3.25] | ec:3.6.3.25 |
| aby:ABAYE0927 | cysW; sulfate ABC transporter membrane protein; K02047 sulfate transport system permease protein |  |
| aby:ABAYE0928 | cysT; sulfate ABC transporter membrane protein; K02046 sulfate transport system permease protein |  |
| aby:ABAYE0929 | hypothetical protein; K07002 |  |
| aby:ABAYE0930 | cysP; sulfate ABC transporter substrate-binding protein; K02048 sulfate transport system substrate-binding protein |  |
| aby:ABAYE0931 | pabC; 4-amino-4-deoxychorismate lyase (EC:4.-.-.-); K02619 4-amino-4-deoxychorismate lyase [EC:4.1.3.38] | ec:4.1.3.38 |
| aby:ABAYE0932 | hypothetical protein; K07082 UPF0755 protein |  |
| aby:ABAYE0933 | tmk; thymidylate kinase (EC:2.7.4.9); K00943 dTMP kinase [EC:2.7.4.9] | ec:2.7.4.9 |
| aby:ABAYE0934 | thioesterase |  |
| aby:ABAYE0935 | nadB; L-aspartate oxidase (EC:1.4.3.16); K00278 L-aspartate oxidase [EC:1.4.3.16] | ec:1.4.3.16 |

  
**Neighborhood Representations for "acd:AOLE\_04485"**  

| ID | Annotation | EC number |
| --- | --- | --- |
| acd:AOLE\_04435 | hypothetical protein |  |
| acd:AOLE\_04440 | hypothetical protein |  |
| acd:AOLE\_04445 | SCP-like extracellular family protein |  |
| acd:AOLE\_04450 | AhpC/TSA family protein; K03564 peroxiredoxin Q/BCP [EC:1.11.1.15] | ec:1.11.1.15 |
| acd:AOLE\_04455 | hypothetical protein |  |
| acd:AOLE\_04460 | hypothetical protein |  |
| acd:AOLE\_04465 | hypothetical protein; K06920 7-cyano-7-deazaguanine synthase [EC:6.3.4.20] | ec:6.3.4.20 |
| acd:AOLE\_04470 | radical SAM protein; K10026 7-carboxy-7-deazaguanine synthase [EC:4.3.99.3] | ec:4.3.99.3 |
| acd:AOLE\_04475 | dapD; 2,3,4,5-tetrahydropyridine-2,6-carboxylate N-succinyltransferase (EC:2.3.1.117); K00674 2,3,4,5-tetrahydropyridine-2-carboxylate N-succinyltransferase [EC:2.3.1.117] | ec:2.3.1.117 |
| acd:AOLE\_04480 | putative porin protein associated with imipenem resistance |  |
| acd:AOLE\_04485 | transcriptional regulator CysB-like protein; K13635 LysR family transcriptional regulator, cys regulon transcriptional activator |  |
| acd:AOLE\_04490 | sulfate/thiosulfate import ATP-binding protein cysA(Sulfate-transporting ATPase); K02045 sulfate transport system ATP-binding protein [EC:3.6.3.25] | ec:3.6.3.25 |
| acd:AOLE\_04495 | ABC-type sulfate transport system permease; K02047 sulfate transport system permease protein |  |
| acd:AOLE\_04500 | ABC-type sulfate transport system permease; K02046 sulfate transport system permease protein |  |
| acd:AOLE\_04505 | alpha/beta hydrolase fold protein; K07002 |  |
| acd:AOLE\_04510 | sulfate-binding protein; K02048 sulfate transport system substrate-binding protein |  |
| acd:AOLE\_04515 | 4-amino-4-deoxychorismate lyase; K02619 4-amino-4-deoxychorismate lyase [EC:4.1.3.38] | ec:4.1.3.38 |
| acd:AOLE\_04520 | Aminodeoxychorismate lyase family protein; K07082 UPF0755 protein |  |
| acd:AOLE\_04525 | tmk; thymidylate kinase (EC:2.7.4.9); K00943 dTMP kinase [EC:2.7.4.9] | ec:2.7.4.9 |
| acd:AOLE\_04530 | hypothetical protein |  |
| acd:AOLE\_04535 | L-aspartate oxidase (EC:1.4.3.16); K00278 L-aspartate oxidase [EC:1.4.3.16] | ec:1.4.3.16 |

  
**Over-represented Enzyme Summary**: Table of E.C. identified protein in the "Neighborhood Representation" ranked by frequency of occurrence  

| EC number | Frequency | Annotation | Reactions |
| --- | --- | --- | --- |
| ec:3.6.3.25 | 59 | sulfate-transporting ATPase | ATP + H2O + sulfateout = ADP + phosphate + sulfatein [RN:R00086] |
| ec:3.4.21.88 | 27 | repressor LexA; LexA repressor | Hydrolysis of Ala84!Gly bond in repressor LexA |
| ec:3.2.2.4 | 21 | AMP nucleosidase; adenylate nucleosidase; adenosine monophosphate nucleosidase | AMP + H2O = D-ribose 5-phosphate + adenine [RN:R00182] |
| ec:2.4.2.21 | 21 | nicotinate-nucleotide---dimethylbenzimidazole phosphoribosyltransferase; nicotinate mononucleotide-dimethylbenzimidazole phosphoribosyltransferase; nicotinate ribonucleotide:benzimidazole (adenine) phosphoribosyltransferase; nicotinate-nucleotide:dimethylbenzimidazole phospho-D-ribosyltransferase; CobT; nicotinate mononucleotide (NaMN):5,6-dimethylbenzimidazole phosphoribosyltransferase | beta-nicotinate D-ribonucleotide + 5,6-dimethylbenzimidazole = nicotinate + alpha-ribazole 5'-phosphate [RN:R04148] |
| ec:2.7.8.26 | 20 | adenosylcobinamide-GDP ribazoletransferase; CobS; cobalamin synthase; cobalamin-5'-phosphate synthase; cobalamin (5'-phosphate) synthase | (1) adenosylcobinamide-GDP + alpha-ribazole = GMP + adenosylcobalamin [RN:R05223]; (2) adenosylcobinamide-GDP + alpha-ribazole 5'-phosphate = GMP + adenosylcobalamin 5'-phosphate |
| ec:2.7.7.62 | 19 | adenosylcobinamide-phosphate guanylyltransferase; CobU; adenosylcobinamide kinase/adenosylcobinamide-phosphate guanylyltransferase; AdoCbi kinase/AdoCbi-phosphate guanylyltransferase | GTP + adenosylcobinamide phosphate = diphosphate + adenosylcobinamide-GDP [RN:R05222] |
| ec:2.7.1.156 | 19 | adenosylcobinamide kinase; CobU; adenosylcobinamide kinase/adenosylcobinamide-phosphate guanylyltransferase; AdoCbi kinase/AdoCbi-phosphate guanylyltransferase | RTP + adenosylcobinamide = adenosylcobinamide phosphate + RDP [where RTP is either ATP or GTP (for symbol definitions, click here)] [RN:R05221 R06558] |
| ec:3.6.3.17 | 18 | monosaccharide-transporting ATPase | ATP + H2O + monosaccharideout = ADP + phosphate + monosaccharidein [RN:R00086] |
| ec:2.7.1.15 | 17 | ribokinase; deoxyribokinase; ribokinase (phosphorylating); D-ribokinase | ATP + D-ribose = ADP + D-ribose 5-phosphate [RN:R01051] |
| ec:1.14.14.5 | 11 | alkanesulfonate monooxygenase; SsuD; sulfate starvation-induced protein 6; alkanesulfonate,reduced-FMN:oxygen oxidoreductase | an alkanesulfonate + FMNH2 + O2 = an aldehyde + FMN + sulfite + H2O [RN:R07210] |
| ec:1.4.3.16 | 8 | L-aspartate oxidase; NadB; Laspo; AO | L-aspartate + O2 = iminosuccinate + H2O2 [RN:R00481] |
| ec:4.1.3.1 | 8 | isocitrate lyase; isocitrase; isocitritase; isocitratase; threo-Ds-isocitrate glyoxylate-lyase; isocitrate glyoxylate-lyase | isocitrate = succinate + glyoxylate [RN:R00479] |
| ec:2.7.7.4 | 8 | sulfate adenylyltransferase; ATP-sulfurylase; adenosine-5'-triphosphate sulfurylase; adenosinetriphosphate sulfurylase; adenylylsulfate pyrophosphorylase; ATP sulfurylase; ATP-sulfurylase; sulfurylase | ATP + sulfate = diphosphate + adenylyl sulfate [RN:R00529] |
| ec:2.3.1.117 | 7 | 2,3,4,5-tetrahydropyridine-2,6-dicarboxylate N-succinyltransferase; tetrahydropicolinate succinylase; tetrahydrodipicolinate N-succinyltransferase; tetrahydrodipicolinate succinyltransferase; succinyl-CoA:tetrahydrodipicolinate N-succinyltransferase; succinyl-CoA:2,3,4,5-tetrahydropyridine-2,6-dicarboxylate N-succinyltransferase | succinyl-CoA + (S)-2,3,4,5-tetrahydropyridine-2,6-dicarboxylate + H2O = CoA + N-succinyl-L-2-amino-6-oxoheptanedioate [RN:R04365] |
| ec:6.3.4.20 | 7 | 7-cyano-7-deazaguanine synthase; preQ0 synthase; 7-cyano-7-carbaguanine synthase; queC (gene name) | 7-carboxy-7-carbaguanine + NH3 + ATP = 7-cyano-7-carbaguanine + ADP + phosphate + H2O [RN:R09978] |
| ec:1.11.1.15 | 7 | peroxiredoxin; thioredoxin peroxidase; tryparedoxin peroxidase; alkyl hydroperoxide reductase C22; AhpC; TrxPx; TXNPx; Prx; PRDX | 2 R'-SH + ROOH = R'-S-S-R' + H2O + ROH [RN:R07180] |
| ec:2.7.4.9 | 7 | dTMP kinase; thymidine monophosphate kinase; thymidylate kinase; thymidylate monophosphate kinase; thymidylic acid kinase; thymidylic kinase; deoxythymidine 5'-monophosphate kinase; TMPK; thymidine 5'-monophosphate kinase | ATP + dTMP = ADP + dTDP [RN:R02094] |
| ec:2.2.1.6 | 7 | acetolactate synthase; alpha-acetohydroxy acid synthetase; alpha-acetohydroxyacid synthase; alpha-acetolactate synthase; alpha-acetolactate synthetase; acetohydroxy acid synthetase; acetohydroxyacid synthase; acetolactate pyruvate-lyase (carboxylating); acetolactic synthetase | 2 pyruvate = 2-acetolactate + CO2 [RN:R00006] |
| ec:4.3.99.3 | 7 | 7-carboxy-7-deazaguanine synthase; 7-carboxy-7-carbaguanine synthase; queE (gene name) | 6-carboxy-5,6,7,8-tetrahydropterin = 7-carboxy-7-carbaguanine + NH3 [RN:R10002] |
| ec:4.1.3.38 | 7 | aminodeoxychorismate lyase; enzyme X; 4-amino-4-deoxychorismate lyase; 4-amino-4-deoxychorismate pyruvate-lyase | 4-amino-4-deoxychorismate = 4-aminobenzoate + pyruvate [RN:R05553] |
| ec:5.3.1.22 | 6 | hydroxypyruvate isomerase | hydroxypyruvate = 2-hydroxy-3-oxopropanoate [RN:R01394] |
| ec:4.1.1.47 | 5 | tartronate-semialdehyde synthase; tartronate semialdehyde carboxylase; glyoxylate carbo-ligase; glyoxylic carbo-ligase; hydroxymalonic semialdehyde carboxylase; tartronic semialdehyde carboxylase; glyoxalate carboligase; glyoxylate carboxy-lyase (dimerizing); glyoxylate carboxy-lyase (dimerizing; tartronate-semialdehyde-forming) | 2 glyoxylate = 2-hydroxy-3-oxopropanoate + CO2 [RN:R00013] |
| ec:1.8.1.2 | 5 | sulfite reductase (NADPH); sulfite (reduced nicotinamide adenine dinucleotide phosphate) reductase; NADPH-sulfite reductase; NADPH-dependent sulfite reductase; H2S-NADP oxidoreductase; sulfite reductase (NADPH2) | hydrogen sulfide + 3 NADP+ + 3 H2O = sulfite + 3 NADPH + 3 H+ [RN:R00858] |
| ec:1.1.1.60 | 5 | 2-hydroxy-3-oxopropionate reductase; tartronate semialdehyde reductase; (R)-glycerate:NAD(P)+ oxidoreductase | D-glycerate + NAD(P)+ = 2-hydroxy-3-oxopropanoate + NAD(P)H + H+ [RN:R01745 R01747] |
| ec:3.5.1.1 | 5 | asparaginase; asparaginase II; L-asparaginase; colaspase; elspar; leunase; crasnitin; alpha-asparaginase | L-asparagine + H2O = L-aspartate + NH3 [RN:R00485] |
| ec:1.9.3.1 | 4 | cytochrome-c oxidase; cytochrome oxidase; cytochrome a3; cytochrome aa3; Warburg's respiratory enzyme; indophenol oxidase; indophenolase; complex IV (mitochondrial electron transport); ferrocytochrome c oxidase; NADH cytochrome c oxidase | 4 ferrocytochrome c + O2 + 4 H+ = 4 ferricytochrome c + 2 H2O [RN:R00081] |
| ec:2.3.2.2 | 4 | gamma-glutamyltransferase; glutamyl transpeptidase; alpha-glutamyl transpeptidase; gamma-glutamyl peptidyltransferase; gamma-glutamyl transpeptidase (ambiguous); gamma-GPT; gamma-GT; gamma-GTP; L-gamma-glutamyl transpeptidase; L-gamma-glutamyltransferase; L-glutamyltransferase; GGT (ambiguous); gamma-glutamyltranspeptidase (ambiguous) | a (5-L-glutamyl)-peptide + an amino acid = a peptide + a 5-L-glutamyl amino acid [RN:R04159] |
| ec:1.8.4.8 | 4 | phosphoadenylyl-sulfate reductase (thioredoxin); PAPS reductase, thioredoxin-dependent; PAPS reductase; thioredoxin:adenosine 3'-phosphate 5'-phosphosulfate reductase; 3'-phosphoadenylylsulfate reductase; thioredoxin:3'-phospho-adenylylsulfate reductase; phosphoadenosine-phosphosulfate reductase; adenosine 3',5'-bisphosphate,sulfite:oxidized-thioredoxin oxidoreductase (3'-phosphoadenosine-5'-phosphosulfate-forming) | adenosine 3',5'-bisphosphate + sulfite + thioredoxin disulfide = 3'-phosphoadenylyl sulfate + thioredoxin [RN:R02021] |
| ec:1.12.99.6 | 4 | hydrogenase (acceptor); H2 producing hydrogenase[ambiguous]; hydrogen-lyase[ambiguous]; hydrogenlyase[ambiguous]; uptake hydrogenase[ambiguous]; hydrogen:(acceptor) oxidoreductase | H2 + A = AH2 [RN:R07182] |
| ec:1.6.1.2 | 3 | NAD(P)+ transhydrogenase (Re/Si-specific); pyridine nucleotide transhydrogenase; transhydrogenase; NAD(P)+ transhydrogenase; nicotinamide adenine dinucleotide (phosphate) transhydrogenase; NAD+ transhydrogenase; NADH transhydrogenase; nicotinamide nucleotide transhydrogenase; NADPH-NAD+ transhydrogenase; pyridine nucleotide transferase; NADPH-NAD+ oxidoreductase; NADH-NADP+-transhydrogenase; NADPH:NAD+ transhydrogenase; H+-Thase; energy-linked transhydrogenase; NAD(P) transhydrogenase (AB-specific); NAD(P)+ transhydrogenase (AB-specific); NADPH:NAD+ oxidoreductase (AB-specific) | NADPH + NAD+ = NADP+ + NADH [RN:R00112] |
| ec:1.2.99.5 | 3 | formylmethanofuran dehydrogenase; formylmethanofuran:(acceptor) oxidoreductase | formylmethanofuran + H2O + acceptor = CO2 + methanofuran + reduced acceptor [RN:R03015] |
| ec:3.5.1.5 | 3 | urease | urea + H2O = CO2 + 2 NH3 [RN:R00131] |
| ec:1.5.1.38 | 3 | FMN reductase (NADPH); FRP; flavin reductase P; SsuE | FMNH2 + NADP+ = FMN + NADPH + H+ [RN:R05706] |
| ec:1.1.1.6 | 2 | glycerol dehydrogenase; glycerin dehydrogenase; NAD+-linked glycerol dehydrogenase | glycerol + NAD+ = glycerone + NADH + H+ [RN:R01034] |
| ec:1.1.1.1 | 2 | alcohol dehydrogenase; aldehyde reductase; ADH; alcohol dehydrogenase (NAD); aliphatic alcohol dehydrogenase; ethanol dehydrogenase; NAD-dependent alcohol dehydrogenase; NAD-specific aromatic alcohol dehydrogenase; NADH-alcohol dehydrogenase; NADH-aldehyde dehydrogenase; primary alcohol dehydrogenase; yeast alcohol dehydrogenase | (1) a primary alcohol + NAD+ = an aldehyde + NADH + H+ [RN:R07326]; (2) a secondary alcohol + NAD+ = a ketone + NADH + H+ [RN:R07327] |
| ec:3.6.3.21 | 2 | polar-amino-acid-transporting ATPase; histidine permease | ATP + H2O + polar amino acidout = ADP + phosphate + polar amino acidin [RN:R00086] |
| ec:4.4.1.22 | 2 | S-(hydroxymethyl)glutathione synthase; glutathione-dependent formaldehyde-activating enzyme; Gfa; S-(hydroxymethyl)glutathione formaldehyde-lyase | S-(hydroxymethyl)glutathione = glutathione + formaldehyde [RN:R06982] |
| ec:1.3.8.7 | 2 | medium-chain acyl-CoA dehydrogenase; fatty acyl coenzyme A dehydrogenase (ambiguous); acyl coenzyme A dehydrogenase (ambiguous); acyl dehydrogenase (ambiguous); fatty-acyl-CoA dehydrogenase (ambiguous); acyl CoA dehydrogenase (ambiguous); general acyl CoA dehydrogenase (ambiguous); medium-chain acyl-coenzyme A dehydrogenase; acyl-CoA:(acceptor) 2,3-oxidoreductase (ambiguous); ACADM (gene name). | a medium-chain acyl-CoA + electron-transfer flavoprotein = a medium-chain trans-2,3-dehydroacyl-CoA + reduced electron-transfer flavoprotein [RN:R00392] |
| ec:2.7.13.3 | 2 | histidine kinase; EnvZ; histidine kinase (ambiguous); histidine protein kinase (ambiguous); protein histidine kinase (ambiguous); protein kinase (histidine) (ambiguous); HK1; HP165; Sln1p | ATP + protein L-histidine = ADP + protein N-phospho-L-histidine |
| ec:2.3.1.79 | 2 | maltose O-acetyltransferase; maltose transacetylase; maltose O-acetyltransferase; MAT | acetyl-CoA + maltose = CoA + 6-O-acetyl-alpha-D-glucopyranosyl-(1->4)-D-glucose [RN:R01556 R06251] |
| ec:3.1.2.12 | 2 | S-formylglutathione hydrolase | S-formylglutathione + H2O = glutathione + formate [RN:R00527] |
| ec:2.5.1.17 | 2 | cob(I)yrinic acid a,c-diamide adenosyltransferase; CobA; CobO; ATP:corrinoid adenosyltransferase; cob(I)alamin adenosyltransferase; aquacob(I)alamin adenosyltransferase; aquocob(I)alamin vitamin B12s adenosyltransferase; ATP:cob(I)alamin Cobeta-adenosyltransferase | (1) ATP + cob(I)yrinic acid a,c-diamide = triphosphate + adenosylcob(III)yrinic acid a,c-diamide [RN:R05220]; (2) ATP + cobinamide = triphosphate + adenosylcobinamide [RN:R07268] |
| ec:1.2.1.2 | 2 | formate dehydrogenase; formate-NAD+ oxidoreductase; FDH I; FDH II; N-FDH; formic hydrogen-lyase; formate hydrogenlyase; hydrogenlyase; NAD+-linked formate dehydrogenase; NAD+-dependent formate dehydrogenase; formate dehydrogenase (NAD+); NAD+-formate dehydrogenase; formate benzyl-viologen oxidoreductase; formic acid dehydrogenase | formate + NAD+ = CO2 + NADH [RN:R00519] |
| ec:1.1.1.284 | 2 | S-(hydroxymethyl)glutathione dehydrogenase; NAD-linked formaldehyde dehydrogenase (incorrect); formaldehyde dehydrogenase (incorrect); formic dehydrogenase (incorrect); class III alcohol dehydrogenase; ADH3; chi-ADH; FDH (incorrect); formaldehyde dehydrogenase (glutathione) (incorrect); GS-FDH (incorrect); glutathione-dependent formaldehyde dehydrogenase (incorrect); NAD-dependent formaldehyde dehydrogenase; GD-FALDH; NAD- and glutathione-dependent formaldehyde dehydrogenase | S-(hydroxymethyl)glutathione + NAD(P)+ = S-formylglutathione + NAD(P)H + H+ [RN:R06983 R07140] |
| ec:6.1.1.14 | 2 | glycine---tRNA ligase; glycyl-tRNA synthetase; glycyl-transfer ribonucleate synthetase; glycyl-transfer RNA synthetase; glycyl-transfer ribonucleic acid synthetase; glycyl translase | ATP + glycine + tRNAGly = AMP + diphosphate + glycyl-tRNAGly [RN:R03654] |
| ec:1.1.2.4 | 2 | D-lactate dehydrogenase (cytochrome); lactic acid dehydrogenase; D-lactate (cytochrome) dehydrogenase; cytochrome-dependent D-(-)-lactate dehydrogenase; D-lactate-cytochrome c reductase; D-(-)-lactic cytochrome c reductase | (R)-lactate + 2 ferricytochrome c = pyruvate + 2 ferrocytochrome c + 2 H+ [RN:R00197] |
| ec:2.6.1.11 | 1 | acetylornithine transaminase; acetylornithine delta-transaminase; ACOAT; acetylornithine 5-aminotransferase; acetylornithine aminotransferase; N-acetylornithine aminotransferase; N-acetylornithine-delta-transaminase; N2-acetylornithine 5-transaminase; N2-acetyl-L-ornithine:2-oxoglutarate aminotransferase; succinylornithine aminotransferase; 2-N-acetyl-L-ornithine:2-oxoglutarate 5-aminotransferase | N2-acetyl-L-ornithine + 2-oxoglutarate = N-acetyl-L-glutamate 5-semialdehyde + L-glutamate [RN:R02283] |
| ec:5.4.2.12 | 1 | phosphoglycerate mutase (2,3-diphosphoglycerate-independent); cofactor independent phosphoglycerate mutase; 2,3-diphosphoglycerate-independent phosphoglycerate mutase; phosphoglycerate phosphomutase (ambiguous); phosphoglyceromutase (ambiguous); monophosphoglycerate mutase (ambiguous); monophosphoglyceromutase (ambiguous); GriP mutase (ambiguous); PGA mutase (ambiguous); iPGM; iPGAM; PGAM-i | 2-phospho-D-glycerate = 3-phospho-D-glycerate [RN:R01518] |
| ec:3.1.26.12 | 1 | ribonuclease E; endoribonuclease E; RNase E; Rne protein | Endonucleolytic cleavage of single-stranded RNA in A- and U-rich regions |
| ec:2.3.1.40 | 1 | acyl-[acyl-carrier-protein]---phospholipid O-acyltransferase; acyl-[acyl-carrier protein]:O-(2-acyl-sn-glycero-3-phospho)-ethanolamine O-acyltransferase | an acyl-[acyl-carrier protein] + O-(2-acyl-sn-glycero-3-phospho)ethanolamine = an [acyl-carrier protein] + O-(1,2-diacyl-sn-glycero-3-phospho)ethanolamine [RN:R04864] |
| ec:5.4.99.24 | 1 | 23S rRNA pseudouridine955/2504/2580 synthase; RluC; pseudouridine synthase RluC | 23S rRNA uridine955/uridine2504/uridine2580 = 23S rRNA pseudouridine955/pseudouridine2504/pseudouridine2580 |
| ec:3.4.23.36 | 1 | signal peptidase II; premurein-leader peptidase; prolipoprotein signal peptidase; leader peptidase II; premurein leader proteinase; leader peptidase II | Release of signal peptides from bacterial membrane prolipoproteins including murein prolipoprotein. Hydrolyses -Xaa-Yaa-Zaa!(S,diacylglyceryl)Cys-, in which Xaa is hydrophobic (preferably Leu), and Yaa (Ala or Ser) and Zaa (Gly or Ala) have small, neutral sidechains |
| ec:3.5.1.88 | 1 | peptide deformylase | formyl-L-methionyl peptide + H2O = formate + methionyl peptide [RN:R05635] |
| ec:2.1.1.197 | 1 | malonyl-[acyl-carrier protein] O-methyltransferase; BioC | S-adenosyl-L-methionine + malonyl-[acyl-carrier protein] = S-adenosyl-L-homocysteine + malonyl-[acyl-carrier protein] methyl ester [RN:R09543] |
| ec:6.1.1.7 | 1 | alanine---tRNA ligase; alanyl-tRNA synthetase; alanyl-transfer ribonucleate synthetase; alanyl-transfer RNA synthetase; alanyl-transfer ribonucleic acid synthetase; alanine-transfer RNA ligase; alanine transfer RNA synthetase; alanine tRNA synthetase; alanine translase; alanyl-transfer ribonucleate synthase; AlaRS; Ala-tRNA synthetase | ATP + L-alanine + tRNAAla = AMP + diphosphate + L-alanyl-tRNAAla [RN:R03038] |
| ec:2.5.1.6 | 1 | methionine adenosyltransferase; adenosylmethionine synthetase; ATP-methionine adenosyltransferase; methionine S-adenosyltransferase; methionine-activating enzyme; S-adenosyl-L-methionine synthetase; S-adenosylmethionine synthase; S-adenosylmethionine synthetase; AdoMet synthetase | ATP + L-methionine + H2O = phosphate + diphosphate + S-adenosyl-L-methionine [RN:R00177] |
| ec:6.1.1.5 | 1 | isoleucine---tRNA ligase; isoleucyl-tRNA synthetase; isoleucyl-transfer ribonucleate synthetase; isoleucyl-transfer RNA synthetase; isoleucine-transfer RNA ligase; isoleucine-tRNA synthetase; isoleucine translase | ATP + L-isoleucine + tRNAIle = AMP + diphosphate + L-isoleucyl-tRNAIle [RN:R03656] |
| ec:2.1.1.193 | 1 | 16S rRNA (uracil1498-N3)-methyltransferase; DUF558 protein; YggJ; RsmE; m3U1498 specific methyltransferase | S-adenosyl-L-methionine + uracil1498 in 16S rRNA = S-adenosyl-L-homocysteine + N3-methyluracil1498 in 16S rRNA |
| ec:1.4.3.19 | 1 | glycine oxidase | glycine + H2O + O2 = glyoxylate + NH3 + H2O2 (overall reaction) [RN:R00366]; (1a) glycine + O2 = 2-iminoacetate + H2O2 [RN:R07463]; (1b) 2-iminoacetate + H2O = glyoxylate + NH3 [RN:R10245] |
| ec:2.5.1.3 | 1 | thiamine-phosphate diphosphorylase; thiamine phosphate pyrophosphorylase; thiamine monophosphate pyrophosphorylase; TMP-PPase | 2-methyl-4-amino-5-hydroxymethylpyrimidine diphosphate + 4-methyl-5-(2-phosphono-oxyethyl)thiazole = diphosphate + thiamine phosphate [RN:R03223] |
| ec:6.1.1.2 | 1 | tryptophan---tRNA ligase; tryptophanyl-tRNA synthetase; L-tryptophan-tRNATrp ligase (AMP-forming); tryptophanyl-transfer ribonucleate synthetase; tryptophanyl-transfer ribonucleic acid synthetase; tryptophanyl-transfer RNA synthetase; tryptophanyl ribonucleic synthetase; tryptophanyl-transfer ribonucleic synthetase; tryptophanyl-tRNA synthase; tryptophan translase; TrpRS | ATP + L-tryptophan + tRNATrp = AMP + diphosphate + L-tryptophyl-tRNATrp [RN:R03664] |
| ec:2.6.1.66 | 1 | valine---pyruvate transaminase; transaminase C; valine-pyruvate aminotransferase; alanine-oxoisovalerate aminotransferase | L-valine + pyruvate = 3-methyl-2-oxobutanoate + L-alanine [RN:R01215] |
| ec:3.5.3.23 | 1 | N-succinylarginine dihydrolase; N2-succinylarginine dihydrolase; arginine succinylhydrolase; SADH; AruB; AstB; 2-N-succinyl-L-arginine iminohydrolase (decarboxylating) | N2-succinyl-L-arginine + 2 H2O = N2-succinyl-L-ornithine + 2 NH3 + CO2 [RN:R04189] |
| ec:2.1.1.79 | 1 | cyclopropane-fatty-acyl-phospholipid synthase; cyclopropane synthetase; unsaturated-phospholipid methyltransferase; cyclopropane synthase; cyclopropane fatty acid synthase; cyclopropane fatty acid synthetase; CFA synthase | S-adenosyl-L-methionine + phospholipid olefinic fatty acid = S-adenosyl-L-homocysteine + phospholipid cyclopropane fatty acid [RN:R03411] |
| ec:2.1.1.13 | 1 | methionine synthase; 5-methyltetrahydrofolate---homocysteine S-methyltransferase; 5-methyltetrahydrofolate---homocysteine transmethylase; N-methyltetrahydrofolate:L-homocysteine methyltransferase; N5-methyltetrahydrofolate methyltransferase; N5-methyltetrahydrofolate-homocysteine cobalamin methyltransferase; N5-methyltetrahydrofolic---homocysteine vitamin B12 transmethylase; B12 N5-methyltetrahydrofolate homocysteine methyltransferase; methyltetrahydrofolate---homocysteine vitamin B12 methyltransferase; tetrahydrofolate methyltransferase; tetrahydropteroylglutamate methyltransferase; tetrahydropteroylglutamic methyltransferase; vitamin B12 methyltransferase; cobalamin-dependent methionine synthase; methionine synthase (cobalamin-dependent); MetH | 5-methyltetrahydrofolate + L-homocysteine = tetrahydrofolate + L-methionine [RN:R00946] |
| ec:2.3.1.30 | 1 | serine O-acetyltransferase; SATase; L-serine acetyltransferase; serine acetyltransferase; serine transacetylase | acetyl-CoA + L-serine = CoA + O-acetyl-L-serine [RN:R00586] |
| ec:5.1.3.1 | 1 | ribulose-phosphate 3-epimerase; phosphoribulose epimerase; erythrose-4-phosphate isomerase; phosphoketopentose 3-epimerase; xylulose phosphate 3-epimerase; phosphoketopentose epimerase; ribulose 5-phosphate 3-epimerase; D-ribulose phosphate-3-epimerase; D-ribulose 5-phosphate epimerase; D-ribulose-5-P 3-epimerase; D-xylulose-5-phosphate 3-epimerase; pentose-5-phosphate 3-epimerase | D-ribulose 5-phosphate = D-xylulose 5-phosphate [RN:R01529] |
| ec:6.3.4.21 | 1 | nicotinate phosphoribosyltransferase; niacin ribonucleotidase; nicotinic acid mononucleotide glycohydrolase; nicotinic acid mononucleotide pyrophosphorylase; nicotinic acid phosphoribosyltransferase; nicotinate-nucleotide:diphosphate phospho-alpha-D-ribosyltransferase | nicotinate + 5-phospho-alpha-D-ribose 1-diphosphate + ATP + H2O = beta-nicotinate D-ribonucleotide + diphosphate + ADP + phosphate [RN:R01724] |
| ec:1.5.1.3 | 1 | dihydrofolate reductase; tetrahydrofolate dehydrogenase; DHFR; pteridine reductase:dihydrofolate reductase; dihydrofolate reductase:thymidylate synthase; thymidylate synthetase-dihydrofolate reductase; folic acid reductase; folic reductase; dihydrofolic acid reductase; dihydrofolic reductase; 7,8-dihydrofolate reductase; NADPH-dihydrofolate reductase | 5,6,7,8-tetrahydrofolate + NADP+ = 7,8-dihydrofolate + NADPH + H+ [RN:R00939] |
| ec:3.6.1.23 | 1 | dUTP diphosphatase; deoxyuridine-triphosphatase; dUTPase; dUTP pyrophosphatase; desoxyuridine 5'-triphosphate nucleotidohydrolase; desoxyuridine 5'-triphosphatase | dUTP + H2O = dUMP + diphosphate [RN:R02100] |
| ec:4.2.3.1 | 1 | threonine synthase; threonine synthetase; O-phospho-L-homoserine phospho-lyase (adding water) | O-phospho-L-homoserine + H2O = L-threonine + phosphate [RN:R01466] |
| ec:2.3.1.109 | 1 | arginine N-succinyltransferase; arginine succinyltransferase; AstA; arginine and ornithine N2-succinyltransferase; AOST; AST; succinyl-CoA:L-arginine 2-N-succinyltransferase | succinyl-CoA + L-arginine = CoA + N2-succinyl-L-arginine [RN:R00832] |
| ec:6.3.1.10 | 1 | adenosylcobinamide-phosphate synthase; CbiB | (1) ATP + adenosylcobyric acid + (R)-1-aminopropan-2-yl phosphate = ADP + phosphate + adenosylcobinamide phosphate [RN:R06529]; (2) ATP + adenosylcobyric acid + (R)-1-aminopropan-2-ol = ADP + phosphate + adenosylcobinamide [RN:R07302] |
| ec:2.5.1.47 | 1 | cysteine synthase; O-acetyl-L-serine sulfhydrylase; O-acetyl-L-serine sulfohydrolase; O-acetylserine (thiol)-lyase; O-acetylserine (thiol)-lyase A; O-acetylserine sulfhydrylase; O3-acetyl-L-serine acetate-lyase (adding hydrogen-sulfide); acetylserine sulfhydrylase; cysteine synthetase; S-sulfocysteine synthase; 3-O-acetyl-L-serine:hydrogen-sulfide 2-amino-2-carboxyethyltransferase | O3-acetyl-L-serine + hydrogen sulfide = L-cysteine + acetate [RN:R00897] |
| ec:3.8.1.2 | 1 | (S)-2-haloacid dehalogenase; 2-haloacid dehalogenase[ambiguous]; 2-haloacid halidohydrolase [ambiguous][ambiguous]; 2-haloalkanoic acid dehalogenase; 2-haloalkanoid acid halidohydrolase; 2-halocarboxylic acid dehalogenase II; DL-2-haloacid dehalogenase[ambiguous]; L-2-haloacid dehalogenase; L-DEX | (S)-2-haloacid + H2O = (R)-2-hydroxyacid + halide [RN:R03830] |
| ec:1.1.1.94 | 1 | glycerol-3-phosphate dehydrogenase [NAD(P)+]; L-glycerol-3-phosphate:NAD(P) oxidoreductase; glycerol phosphate dehydrogenase (nicotinamide adenine dinucleotide (phosphate)); glycerol 3-phosphate dehydrogenase (NADP); glycerol-3-phosphate dehydrogenase [NAD(P)] | sn-glycerol 3-phosphate + NAD(P)+ = glycerone phosphate + NAD(P)H + H+ [RN:R00842 R00844] |
| ec:2.3.1.101 | 1 | formylmethanofuran---tetrahydromethanopterin N-formyltransferase; formylmethanofuran-tetrahydromethanopterin formyltransferase; formylmethanofuran:tetrahydromethanopterin formyltransferase; N-formylmethanofuran(CHO-MFR):tetrahydromethanopterin(H4MPT) formyltransferase; FTR; formylmethanofuran:5,6,7,8-tetrahydromethanopterin N5-formyltransferase | formylmethanofuran + 5,6,7,8-tetrahydromethanopterin = methanofuran + 5-formyl-5,6,7,8-tetrahydromethanopterin [RN:R03390] |
| ec:4.4.1.25 | 1 | L-cysteate sulfo-lyase; L-cysteate sulfo-lyase (deaminating); CuyA | L-cysteate + H2O = pyruvate + bisulfite + NH3 [RN:R07634] |
| ec:2.1.1.61 | 1 | tRNA (5-methylaminomethyl-2-thiouridylate)-methyltransferase; transfer ribonucleate 5-methylaminomethyl-2-thiouridylate 5-methyltransferase; tRNA 5-methylaminomethyl-2-thiouridylate 5'-methyltransferase | S-adenosyl-L-methionine + tRNA containing 5-aminomethyl-2-thiouridine = S-adenosyl-L-homocysteine + tRNA containing 5-methylaminomethyl-2-thiouridylate [RN:R00601] |
| ec:1.2.1.38 | 1 | N-acetyl-gamma-glutamyl-phosphate reductase; reductase, acetyl-gamma-glutamyl phosphate; N-acetylglutamate 5-semialdehyde dehydrogenase; N-acetylglutamic gamma-semialdehyde dehydrogenase; N-acetyl-L-glutamate gamma-semialdehyde:NADP+ oxidoreductase (phosphorylating) | N-acetyl-L-glutamate 5-semialdehyde + NADP+ + phosphate = N-acetyl-L-glutamyl 5-phosphate + NADPH + H+ [RN:R03443] |
| ec:1.16.1.1 | 1 | mercury(II) reductase; mercuric reductase; mercurate(II) reductase; mercuric ion reductase; mercury reductase; reduced NADP:mercuric ion oxidoreductase; mer A | Hg + NADP+ + H+ = Hg2+ + NADPH [RN:R02807] |
| ec:1.3.1.44 | 1 | trans-2-enoyl-CoA reductase (NAD+); trans-2-enoyl-CoA reductase (NAD+) | acyl-CoA + NAD+ = trans-didehydroacyl-CoA + NADH + H+ [RN:R00384] |
| ec:2.1.1.170 | 1 | 16S rRNA (guanine527-N7)-methyltransferase; ribosomal RNA small subunit methyltransferase G; 16S rRNA methyltransferase RsmG; GidB; rsmG (gene name) | S-adenosyl-L-methionine + guanine527 in 16S rRNA = S-adenosyl-L-homocysteine + N7-methylguanine527 in 16S rRNA |
| ec:2.4.1.25 | 1 | 4-alpha-glucanotransferase; disproportionating enzyme; dextrin glycosyltransferase; D-enzyme; debranching enzyme maltodextrin glycosyltransferase; amylomaltase; dextrin transglycosylase; 1,4-alpha-D-glucan:1,4-alpha-D-glucan 4-alpha-D-glycosyltransferase | Transfers a segment of a (1->4)-alpha-D-glucan to a new position in an acceptor, which may be glucose or a (1->4)-alpha-D-glucan |
| ec:2.4.2.28 | 1 | S-methyl-5'-thioadenosine phosphorylase; 5'-methylthioadenosine nucleosidase; 5'-deoxy-5'-methylthioadenosine phosphorylase; MTA phosphorylase; MeSAdo phosphorylase; MeSAdo/Ado phosphorylase; methylthioadenosine phosphorylase; methylthioadenosine nucleoside phosphorylase; 5'-methylthioadenosine:phosphate methylthio-D-ribosyl-transferase; S-methyl-5-thioadenosine phosphorylase; S-methyl-5-thioadenosine:phosphate S-methyl-5-thio-alpha-D-ribosyl-transferase | S-methyl-5'-thioadenosine + phosphate = adenine + S-methyl-5-thio-alpha-D-ribose 1-phosphate [RN:R01402] |
| ec:2.7.9.3 | 1 | selenide, water dikinase; selenophosphate synthase | ATP + selenide + H2O = AMP + selenophosphate + phosphate [RN:R03595] |
| ec:3.6.4.12 | 1 | DNA helicase; 3' to 5' DNA helicase; 3'-5' DNA helicase; 3'-5' PfDH; 5' to 3' DNA helicase; AvDH1; BACH1 helicase; BcMCM; BLM protein; BRCA1-associated C-terminal helicase; CeWRN-1; Dbp9p; DmRECQ5; DNA helicase 120; DNA helicase A; DNA helicase E; DNA helicase II; DNA helicase III; DNA helicase RECQL5beta; DNA helicase VI; dnaB; DnaB helicase E1; helicase HDH IV; Hel E; helicase DnaB; helicase domain of bacteriophage T7 gene 4 protein helicase; PcrA helicase; UvrD; hHcsA; Hmi1p; hPif1; MCM helicase; MCM protein; MER3 helicase; MER3 protein; MPH1; PcrA; PcrA helicase; PDH120; PfDH A; Pfh1p; PIF1 | ATP + H2O = ADP + phosphate [RN:R00086] |
| ec:1.3.5.3 | 1 | protoporphyrinogen IX dehydrogenase (menaquinone); HemG | protoporphyrinogen IX + 3 menaquinone = protoporphyrin IX + 3 menaquinol [RN:R09489] |
| ec:2.8.3.1 | 1 | propionate CoA-transferase; propionate coenzyme A-transferase; propionate-CoA:lactoyl-CoA transferase; propionyl CoA:acetate CoA transferase; propionyl-CoA transferase | acetyl-CoA + propanoate = acetate + propanoyl-CoA [RN:R00928] |
| ec:3.4.11.1 | 1 | leucyl aminopeptidase; leucine aminopeptidase; leucyl peptidase; peptidase S; cytosol aminopeptidase; cathepsin III; L-leucine aminopeptidase; leucinaminopeptidase; leucinamide aminopeptidase; FTBL proteins; proteinates FTBL; aminopeptidase II; aminopeptidase III; aminopeptidase I | Release of an N-terminal amino acid, Xaa!Yaa-, in which Xaa is preferably Leu, but may be other amino acids including Pro although not Arg or Lys, and Yaa may be Pro. Amino acid amides and methyl esters are also readily hydrolysed, but rates on arylamides are exceedingly low |
| ec:2.1.1.207 | 1 | tRNA (cytidine34-2'-O)-methyltransferase; yibK (gene name); methyltransferase yibK; TrmL; tRNA methyltransferase L; tRNA (cytidine34/5-carboxymethylaminomethyluridine34-2'-O)-methyltransferase | (1) S-adenosyl-L-methionine + cytidine34 in tRNA = S-adenosyl-L-homocysteine + 2'-O-methylcytidine34 in tRNA; (2) S-adenosyl-L-methionine + 5-carboxymethylaminomethyluridine34 in tRNALeu = S-adenosyl-L-homocysteine + 5-carboxymethylaminomethyl-2'-O-methyluridine34 in tRNALeu |
| ec:2.8.1.8 | 1 | lipoyl synthase; LS; LipA; lipoate synthase; protein 6-N-(octanoyl)lysine:sulfur sulfurtransferase; protein N6-(octanoyl)lysine:sulfur sulfurtransferase | protein N6-(octanoyl)lysine + 2 sulfur-(sulfur carrier) + 2 S-adenosyl-L-methionine = protein N6-(lipoyl)lysine + 2 (sulfur carrier) + 2 L-methionine + 2 5'-deoxyadenosine [RN:R07767] |
| ec:4.4.1.16 | 1 | selenocysteine lyase; selenocysteine reductase; selenocysteine beta-lyase | L-selenocysteine + reduced acceptor = selenide + L-alanine + acceptor [RN:R03599] |
| ec:2.8.1.7 | 1 | cysteine desulfurase; IscS; NIFS; NifS; SufS; cysteine desulfurylase | L-cysteine + acceptor = L-alanine + S-sulfanyl-acceptor (overall reaction); (1a) L-cysteine + [enzyme]-cysteine = L-alanine + [enzyme]-S-sulfanylcysteine [RN:R07460]; (1b) [enzyme]-S-sulfanylcysteine + acceptor = [enzyme]-cysteine + S-sulfanyl-acceptor |
| ec:2.7.7.7 | 1 | DNA-directed DNA polymerase; DNA polymerase I; DNA polymerase II; DNA polymerase III; DNA polymerase alpha; DNA polymerase beta; DNA polymerase gamma; DNA nucleotidyltransferase (DNA-directed); DNA nucleotidyltransferase (DNA-directed); deoxyribonucleate nucleotidyltransferase; deoxynucleate polymerase; deoxyribonucleic acid duplicase; deoxyribonucleic acid polymerase; deoxyribonucleic duplicase; deoxyribonucleic polymerase; deoxyribonucleic polymerase I; DNA duplicase; DNA nucleotidyltransferase; DNA polymerase; DNA replicase; DNA-dependent DNA polymerase; duplicase; Klenow fragment; sequenase; Taq DNA polymerase; Taq Pol I; Tca DNA polymerase | deoxynucleoside triphosphate + DNAn = diphosphate + DNAn+1 [RN:R00379] |
| ec:2.3.3.13 | 1 | 2-isopropylmalate synthase; 3-carboxy-3-hydroxy-4-methylpentanoate 3-methyl-2-oxobutanoate-lyase (CoA-acetylating); alpha-isopropylmalate synthetase; alpha-isopropylmalate synthase; alpha-isopropylmalic synthetase; isopropylmalate synthase; isopropylmalate synthetase | acetyl-CoA + 3-methyl-2-oxobutanoate + H2O = (2S)-2-isopropylmalate + CoA [RN:R01213] |
| ec:2.7.7.2 | 1 | FAD synthetase; FAD pyrophosphorylase; riboflavin mononucleotide adenylyltransferase; adenosine triphosphate-riboflavin mononucleotide transadenylase; adenosine triphosphate-riboflavine mononucleotide transadenylase; riboflavin adenine dinucleotide pyrophosphorylase; riboflavine adenine dinucleotide adenylyltransferase; flavin adenine dinucleotide synthetase; FADS; FMN adenylyltransferase | ATP + FMN = diphosphate + FAD [RN:R00161] |
| ec:2.8.1.2 | 1 | 3-mercaptopyruvate sulfurtransferase; beta-mercaptopyruvate sulfurtransferase | 3-mercaptopyruvate + cyanide = pyruvate + thiocyanate [RN:R03106] |
| ec:2.8.1.1 | 1 | thiosulfate sulfurtransferase; thiosulfate cyanide transsulfurase; thiosulfate thiotransferase; rhodanese; rhodanase | thiosulfate + cyanide = sulfite + thiocyanate [RN:R01931] |
| ec:2.7.7.1 | 1 | nicotinamide-nucleotide adenylyltransferase; NAD+ pyrophosphorylase; adenosine triphosphate-nicotinamide mononucleotide transadenylase; ATP:NMN adenylyltransferase; diphosphopyridine nucleotide pyrophosphorylase; nicotinamide adenine dinucleotide pyrophosphorylase; nicotinamide mononucleotide adenylyltransferase; NMN adenylyltransferase | ATP + nicotinamide ribonucleotide = diphosphate + NAD+ [RN:R00137] |
| ec:2.7.6.1 | 1 | ribose-phosphate diphosphokinase; ribose-phosphate pyrophosphokinase; PRPP synthetase; phosphoribosylpyrophosphate synthetase; PPRibP synthetase; PP-ribose P synthetase; 5-phosphoribosyl-1-pyrophosphate synthetase; 5-phosphoribose pyrophosphorylase; 5-phosphoribosyl-alpha-1-pyrophosphate synthetase; phosphoribosyl-diphosphate synthetase; phosphoribosylpyrophosphate synthase; pyrophosphoribosylphosphate synthetase; ribophosphate pyrophosphokinase; ribose-5-phosphate pyrophosphokinase | ATP + D-ribose 5-phosphate = AMP + 5-phospho-alpha-D-ribose 1-diphosphate [RN:R01049] |
| ec:3.1.2.1 | 1 | acetyl-CoA hydrolase; acetyl-CoA deacylase; acetyl-CoA acylase; acetyl coenzyme A hydrolase; acetyl coenzyme A deacylase; acetyl coenzyme A acylase; acetyl-CoA thiol esterase | acetyl-CoA + H2O = CoA + acetate [RN:R00227] |
| ec:1.1.1.14 | 1 | L-iditol 2-dehydrogenase; polyol dehydrogenase; sorbitol dehydrogenase; L-iditol:NAD+ 5-oxidoreductase; L-iditol (sorbitol) dehydrogenase; glucitol dehydrogenase; L-iditol:NAD+ oxidoreductase; NAD+-dependent sorbitol dehydrogenase; NAD+-sorbitol dehydrogenase | L-iditol + NAD+ = L-sorbose + NADH + H+ [RN:R07145] |
| ec:3.6.3.4 | 1 | Cu2+-exporting ATPase; CopB | ATP + H2O + Cu2+[side 1] = ADP + phosphate + Cu2+[side 2] [RN:R00086] |
| ec:6.3.5.1 | 1 | NAD+ synthase (glutamine-hydrolysing); NAD synthetase (glutamine-hydrolysing); nicotinamide adenine dinucleotide synthetase (glutamine); desamidonicotinamide adenine dinucleotide amidotransferase; DPN synthetase | ATP + deamido-NAD+ + L-glutamine + H2O = AMP + diphosphate + NAD+ + L-glutamate [RN:R00257] |
| ec:1.15.1.1 | 1 | superoxide dismutase; superoxidase dismutase; copper-zinc superoxide dismutase; Cu-Zn superoxide dismutase; ferrisuperoxide dismutase; superoxide dismutase I; superoxide dismutase II; SOD; Cu,Zn-SOD; Mn-SOD; Fe-SOD; SODF; SODS; SOD-1; SOD-2; SOD-3; SOD-4; hemocuprein; erythrocuprein; cytocuprein; cuprein ; hepatocuprein | 2 O2.- + 2 H+ = O2 + H2O2 [RN:R00275] |
| ec:6.3.4.5 | 1 | argininosuccinate synthase; citrulline---aspartate ligase; argininosuccinate synthetase; arginine succinate synthetase; argininosuccinic acid synthetase; arginosuccinate synthetase | ATP + L-citrulline + L-aspartate = AMP + diphosphate + 2-(Nomega-L-arginino)succinate [RN:R01954] |
| ec:5.3.1.27 | 1 | 6-phospho-3-hexuloisomerase; 3-hexulose-6-phosphate isomerase; phospho-3-hexuloisomerase; PHI; 6-phospho-3-hexulose isomerase; YckF | D-arabino-hex-3-ulose 6-phosphate = D-fructose 6-phosphate [RN:R05339] |
| ec:3.1.3.18 | 1 | phosphoglycolate phosphatase; phosphoglycolate hydrolase; 2-phosphoglycolate phosphatase; P-glycolate phosphatase; phosphoglycollate phosphatase | 2-phosphoglycolate + H2O = glycolate + phosphate [RN:R01334] |
| ec:3.2.2.20 | 1 | DNA-3-methyladenine glycosylase I; deoxyribonucleate 3-methyladenine glycosidase I; 3-methyladenine DNA glycosylase I; DNA-3-methyladenine glycosidase I | Hydrolysis of alkylated DNA, releasing 3-methyladenine |
| ec:2.1.1.45 | 1 | thymidylate synthase; dTMP synthase; thymidylate synthetase; methylenetetrahydrofolate:dUMP C-methyltransferase; TMP synthetase | 5,10-methylenetetrahydrofolate + dUMP = dihydrofolate + dTMP [RN:R02101] |
| ec:3.1.3.11 | 1 | fructose-bisphosphatase; hexose diphosphatase; FBPase; fructose 1,6-diphosphatase; fructose 1,6-diphosphate phosphatase; D-fructose 1,6-diphosphatase; fructose 1,6-bisphosphatase; fructose diphosphatase; fructose diphosphate phosphatase; fructose bisphosphate phosphatase; fructose 1,6-bisphosphate 1-phosphatase; fructose 1,6-bisphosphate phosphatase; hexose bisphosphatase; D-fructose-1,6-bisphosphate phosphatase | D-fructose 1,6-bisphosphate + H2O = D-fructose 6-phosphate + phosphate [RN:R00762] |
| ec:2.2.1.1 | 1 | transketolase; glycolaldehydetransferase | sedoheptulose 7-phosphate + D-glyceraldehyde 3-phosphate = D-ribose 5-phosphate + D-xylulose 5-phosphate [RN:R01641] |
| ec:6.3.2.5 | 1 | phosphopantothenate---cysteine ligase; phosphopantothenoylcysteine synthetase | CTP + (R)-4'-phosphopantothenate + L-cysteine = CMP + diphosphate + N-[(R)-4'-phosphopantothenoyl]-L-cysteine [RN:R04231] |
| ec:4.1.1.37 | 1 | uroporphyrinogen decarboxylase; uroporphyrinogen III decarboxylase; porphyrinogen carboxy-lyase; porphyrinogen decarboxylase; uroporphyrinogen-III carboxy-lyase | uroporphyrinogen III = coproporphyrinogen III + 4 CO2 [RN:R03197] |
| ec:4.1.1.36 | 1 | phosphopantothenoylcysteine decarboxylase; 4-phosphopantotheoylcysteine decarboxylase; 4-phosphopantothenoyl-L-cysteine decarboxylase; PPC-decarboxylase; N-[(R)-4'-phosphopantothenoyl]-L-cysteine carboxy-lyase | N-[(R)-4'-phosphopantothenoyl]-L-cysteine = pantotheine 4'-phosphate + CO2 [RN:R03269] |
| ec:1.2.1.19 | 1 | aminobutyraldehyde dehydrogenase; gamma-guanidinobutyraldehyde dehydrogenase (ambiguous); ABAL dehydrogenase; 4-aminobutyraldehyde dehydrogenase; 4-aminobutanal dehydrogenase; gamma-aminobutyraldehyde dehydroganase; 1-pyrroline dehydrogenase; ABALDH; YdcW | 4-aminobutanal + NAD+ + H2O = 4-aminobutanoate + NADH + 2 H+ [RN:R02549] |
| ec:6.2.1.34 | 1 | trans-feruloyl-CoA synthase; trans-feruloyl-CoA synthetase; trans-ferulate:CoASH ligase (ATP-hydrolysing); ferulate:CoASH ligase (ATP-hydrolysing) | ferulic acid + CoA + ATP = feruloyl-CoA + products of ATP breakdown [RN:R05744] |
| ec:1.2.1.71 | 1 | succinylglutamate-semialdehyde dehydrogenase; succinylglutamic semialdehyde dehydrogenase; N-succinylglutamate 5-semialdehyde dehydrogenase; SGSD; AruD; AstD | N-succinyl-L-glutamate 5-semialdehyde + NAD+ + H2O = N-succinyl-L-glutamate + NADH + 2 H+ [RN:R05049] |
| ec:3.1.1.61 | 1 | protein-glutamate methylesterase; chemotaxis-specific methylesterase; methyl-accepting chemotaxis protein methyl-esterase; CheB methylesterase; methylesterase CheB; protein methyl-esterase; protein carboxyl methylesterase; PME; protein methylesterase; protein-L-glutamate-5-O-methyl-ester acylhydrolase | protein L-glutamate O5-methyl ester + H2O = protein L-glutamate + methanol [RN:R02624] |
| ec:2.6.1.82 | 1 | putrescine aminotransferase; putrescine-alpha-ketoglutarate transaminase; YgjG; putrescine:alpha-ketoglutarate aminotransferase; PAT; putrescine:2-oxoglutarate aminotransferase; putrescine transaminase | putrescine + 2-oxoglutarate = 1-pyrroline + L-glutamate + H2O (overall reaction) [RN:R10064]; (1a) putrescine + 2-oxoglutarate = 4-aminobutanal + L-glutamate [RN:R01155]; (1b) 4-aminobutanal = 1-pyrroline + H2O (spontaneous) [RN:R07408] |
| ec:2.6.1.81 | 1 | succinylornithine transaminase; succinylornithine aminotransferase; N2-succinylornithine 5-aminotransferase; AstC; SOAT; 2-N-succinyl-L-ornithine:2-oxoglutarate 5-aminotransferase | N2-succinyl-L-ornithine + 2-oxoglutarate = N-succinyl-L-glutamate 5-semialdehyde + L-glutamate [RN:R04217] |
| ec:2.1.1.33 | 1 | tRNA (guanine46-N7)-methyltransferase; Trm8/Trm82; TrmB; tRNA (m7G46) methyltransferase; transfer ribonucleate guanine 7-methyltransferase; 7-methylguanine transfer ribonucleate methylase; tRNA guanine 7-methyltransferase; N7-methylguanine methylase; S-adenosyl-L-methionine:tRNA (guanine-7-N-)-methyltransferase | S-adenosyl-L-methionine + guanine46 in tRNA = S-adenosyl-L-homocysteine + N7-methylguanine46 in tRNA [RN:R00600] |
| ec:5.1.99.3 | 1 | allantoin racemase | (S)(+)-allantoin = (R)(-)-allantoin [RN:R03925] |
| ec:5.3.1.1 | 1 | triose-phosphate isomerase; phosphotriose isomerase; triose phosphoisomerase; triose phosphate mutase; D-glyceraldehyde-3-phosphate ketol-isomerase | D-glyceraldehyde 3-phosphate = glycerone phosphate [RN:R01015] |
| ec:4.4.1.8 | 1 | cystathionine beta-lyase; beta-cystathionase; cystine lyase; cystathionine L-homocysteine-lyase (deaminating); L-cystathionine L-homocysteine-lyase (deaminating) | L-cystathionine + H2O = L-homocysteine + NH3 + pyruvate [RN:R01286] |
| ec:4.2.1.96 | 1 | 4a-hydroxytetrahydrobiopterin dehydratase; 4alpha-hydroxy-tetrahydropterin dehydratase; pterin-4alpha-carbinolamine dehydratase; 4a-hydroxytetrahydrobiopterin hydro-lyase | (6R)-6-(L-erythro-1,2-dihydroxypropyl)-5,6,7,8-tetrahydro-4a-hydroxypterin = (6R)-6-(L-erythro-1,2-dihydroxypropyl)-7,8-dihydro-6H-pterin + H2O [RN:R04734] |
| ec:1.6.5.3 | 1 | NADH:ubiquinone reductase (H+-translocating); ubiquinone reductase; type 1 dehydrogenase; complex 1 dehydrogenase; coenzyme Q reductase; complex I (electron transport chain); complex I (mitochondrial electron transport); complex I (NADH:Q1 oxidoreductase); dihydronicotinamide adenine dinucleotide-coenzyme Q reductase; DPNH-coenzyme Q reductase; DPNH-ubiquinone reductase; mitochondrial electron transport complex 1; mitochondrial electron transport complex I; NADH coenzyme Q1 reductase; NADH-coenzyme Q oxidoreductase; NADH-coenzyme Q reductase; NADH-CoQ oxidoreductase; NADH-CoQ reductase; NADH-ubiquinone reductase; NADH-ubiquinone oxidoreductase; NADH-ubiquinone-1 reductase; reduced nicotinamide adenine dinucleotide-coenzyme Q reductase; NADH:ubiquinone oxidoreductase complex; NADH-Q6 oxidoreductase; electron transfer complex I; NADH2 dehydrogenase (ubiquinone) | NADH + ubiquinone + 6 H+[side 1] = NAD+ + ubiquinol + 7 H+[side 2] [RN:R02163] |
| ec:3.5.1.96 | 1 | succinylglutamate desuccinylase; N2-succinylglutamate desuccinylase; SGDS; AstE | N-succinyl-L-glutamate + H2O = succinate + L-glutamate [RN:R00411] |
| ec:2.7.1.26 | 1 | riboflavin kinase; flavokinase; FK; RFK | ATP + riboflavin = ADP + FMN [RN:R00549] |
| ec:6.2.1.20 | 1 | long-chain-fatty-acid---[acyl-carrier-protein] ligase; acyl-[acyl-carrier-protein] synthetase; acyl-[acyl carrier protein] synthetase; acyl-ACP synthetase; acyl-[acyl-carrier-protein]synthetase; stearoyl-ACP synthetase; acyl-acyl carrier protein synthetase; long-chain-fatty-acid:[acyl-carrier-protein] ligase (AMP-forming) | ATP + an acid + an [acyl-carrier protein] = AMP + diphosphate + an acyl-[acyl-carrier protein] [RN:R07325] |

  
**Over-represented Metabolite Summary**: Collection of the metabolites identified as substrates or products of the proteins representaed the "Over-represented Enzyme Summary" ranked by frequency of occurrence  

| ID | Structure | Name | Frequency | EC |
| --- | --- | --- | --- | --- |
| cpd:C00001 |  | H2O; Water | 102 | ec:2.7.9.3 ec:6.3.5.1 ec:2.3.3.13 ec:3.1.2.12 ec:2.5.1.6 ec:3.6.1.23 ec:3.5.1.1 ec:3.5.1.96 ec:3.5.1.5 ec:3.6.3.4 ec:1.4.3.16 ec:3.2.2.4 ec:3.1.2.1 ec:2.3.1.117 ec:3.8.1.2 ec:1.14.14.5 ec:1.2.1.71 ec:1.2.1.19 ec:4.2.3.1 ec:1.1.1.284 ec:3.5.3.23 ec:4.2.1.96 ec:3.1.3.11 ec:2.3.2.2 ec:1.8.1.2 ec:4.4.1.8 ec:6.3.4.21 ec:3.4.11.1 ec:1.9.3.1 ec:4.4.1.25 ec:1.2.99.5 ec:3.1.3.18 ec:1.1.1.1 ec:6.3.4.20 |
| cpd:C00002 |  | ATP; Adenosine 5'-triphosphate | 77 | ec:6.3.1.10 ec:2.7.9.3 ec:6.3.5.1 ec:6.1.1.2 ec:2.5.1.6 ec:2.3.1.40 ec:6.1.1.5 ec:6.1.1.7 ec:2.7.1.26 ec:4.1.1.36 ec:3.6.3.4 ec:2.7.7.62 ec:6.3.2.5 ec:2.5.1.17 ec:6.3.4.5 ec:2.7.7.1 ec:2.7.7.2 ec:2.7.1.156 ec:2.7.6.1 ec:6.2.1.20 ec:2.7.7.4 ec:6.3.4.21 ec:2.7.1.15 ec:2.7.4.9 ec:6.3.4.20 ec:6.1.1.14 |
| cpd:C00080 |  | H+; Hydron | 56 | ec:1.1.1.14 ec:1.5.1.38 ec:1.1.1.6 ec:1.5.1.3 ec:1.8.1.2 ec:1.1.2.4 ec:1.2.1.38 ec:1.1.1.94 ec:1.2.1.2 ec:1.1.1.60 ec:6.3.4.21 ec:2.5.1.47 ec:1.9.3.1 ec:1.3.1.44 ec:1.2.1.71 ec:1.2.1.19 ec:1.1.1.284 ec:1.1.1.1 ec:2.4.2.21 |
| cpd:C00008 |  | ADP; Adenosine 5'-diphosphate | 54 | ec:6.3.1.10 ec:6.3.4.21 ec:2.7.7.62 ec:2.7.1.15 ec:2.7.1.26 ec:2.7.1.156 ec:2.7.7.2 ec:3.6.3.4 ec:2.7.4.9 ec:6.3.4.20 |
| cpd:C00013 |  | Diphosphate; Diphosphoric acid; Pyrophosphate; Pyrophosphoric acid; PPi | 42 | ec:6.3.5.1 ec:6.3.4.5 ec:6.1.1.2 ec:6.1.1.5 ec:2.3.1.40 ec:2.5.1.6 ec:3.6.1.23 ec:2.7.1.26 ec:6.1.1.7 ec:4.1.1.36 ec:2.5.1.3 ec:2.7.7.1 ec:2.7.1.156 ec:2.7.7.2 ec:6.2.1.20 ec:2.7.7.4 ec:6.3.4.21 ec:2.7.7.62 ec:6.3.2.5 ec:6.1.1.14 |
| cpd:C00117 |  | D-Ribose 5-phosphate; Ribose 5-phosphate | 40 | ec:2.2.1.1 ec:3.2.2.4 ec:2.7.1.15 ec:2.7.6.1 |
| cpd:C06510 |  | Adenosine-GDP-cobinamide; Adenosylcobinamide-GDP | 39 | ec:2.7.7.62 ec:2.7.8.26 ec:2.7.1.156 |
| cpd:C00014 |  | Ammonia; NH3 | 33 | ec:4.4.1.25 ec:3.5.1.1 ec:4.3.99.3 ec:3.5.1.5 ec:3.5.3.23 ec:4.4.1.8 ec:6.3.4.20 ec:1.4.3.16 |
| cpd:C00020 |  | AMP; Adenosine 5'-monophosphate; Adenylic acid; Adenylate; 5'-AMP; 5'-Adenylic acid; 5'-Adenosine monophosphate; Adenosine 5'-phosphate | 32 | ec:2.7.9.3 ec:6.3.5.1 ec:3.2.2.4 ec:6.3.2.5 ec:6.3.4.5 ec:6.1.1.2 ec:6.1.1.5 ec:2.3.1.40 ec:6.1.1.7 ec:4.1.1.36 ec:2.7.6.1 ec:6.2.1.20 ec:6.1.1.14 |
| cpd:C00011 |  | CO2; Carbon dioxide | 24 | ec:6.3.2.5 ec:2.2.1.6 ec:1.2.99.5 ec:4.1.1.36 ec:3.5.1.5 ec:3.5.3.23 ec:1.2.1.2 ec:4.1.1.37 ec:4.1.1.47 |
| cpd:C01185 |  | Nicotinate D-ribonucleotide; beta-Nicotinate D-ribonucleotide; Nicotinate ribonucleotide; Nicotinic acid ribonucleotide | 23 | ec:6.3.4.21 ec:2.7.7.1 ec:2.4.2.21 |
| cpd:C00007 |  | Oxygen; O2 | 23 | ec:1.9.3.1 ec:1.14.14.5 ec:1.4.3.19 ec:1.4.3.16 |
| cpd:C00253 |  | Nicotinate; Nicotinic acid; Niacin; 3-Pyridinecarboxylic acid | 22 | ec:6.3.4.21 ec:2.4.2.21 |
| cpd:C00147 |  | Adenine; 6-Aminopurine | 22 | ec:3.2.2.4 ec:2.4.2.28 |
| cpd:C00094 |  | Sulfite; Sulfurous acid | 22 | ec:1.8.1.2 ec:2.5.1.47 ec:2.8.1.1 ec:2.8.1.2 ec:1.14.14.5 ec:1.8.4.8 |
| cpd:C06508 |  | Adenosyl cobinamide | 22 | ec:6.3.1.10 ec:2.7.7.62 ec:2.5.1.17 ec:2.7.1.156 |
| cpd:C00006 |  | NADP+; NADP; Nicotinamide adenine dinucleotide phosphate; beta-Nicotinamide adenine dinucleotide phosphate; TPN; Triphosphopyridine nucleotide | 22 | ec:1.1.1.60 ec:1.5.1.38 ec:1.8.1.2 ec:1.5.1.3 ec:1.6.1.2 ec:1.2.1.38 ec:1.3.1.44 ec:1.1.1.94 |
| cpd:C00005 |  | NADPH; TPNH; Reduced nicotinamide adenine dinucleotide phosphate | 22 | ec:1.1.1.60 ec:1.5.1.38 ec:1.8.1.2 ec:1.5.1.3 ec:1.6.1.2 ec:1.2.1.38 ec:1.3.1.44 ec:1.1.1.94 |
| cpd:C00003 |  | NAD+; NAD; Nicotinamide adenine dinucleotide; DPN; Diphosphopyridine nucleotide; Nadide | 22 | ec:1.1.1.14 ec:6.3.5.1 ec:1.1.1.6 ec:1.5.1.3 ec:1.6.1.2 ec:1.1.1.94 ec:2.7.7.1 ec:1.2.1.2 ec:1.1.1.60 ec:1.3.1.44 ec:1.2.1.71 ec:1.2.1.19 ec:1.1.1.284 ec:1.1.1.1 |
| cpd:C03114 |  | Dimethylbenzimidazole; 5,6-Dimethylbenzimidazole | 21 | ec:2.4.2.21 |
| cpd:C04778 |  | N1-(5-Phospho-alpha-D-ribosyl)-5,6-dimethylbenzimidazole; alpha-Ribazole 5'-phosphate | 21 | ec:2.4.2.21 |
| cpd:C00194 |  | Cobamide coenzyme; Adenosylcobalamin; Adenosylcob(III)alamin; Deoxyadenosylcobalamin; Cobamamide; Vitamin B12 coenzyme; 5,6-Dimethylbenzimidazolyl-5-deoxyadenosyl-cobamide; (5'-Deoxy-5'-adenosyl)cobamide coenzyme; (5,6-Dimethylbenzimidazolyl)cobamide coenzyme; alpha-(5,6-Dimethylbenzimidazolyl)cobamide coenzyme; 5'-Deoxy-5'-adenosylcobalamin; 5'-Deoxy-5'-adenosyl vitamin B12; 5'-Deoxy-5'-adenosyl-5,6-dimethylbenzimidazolylcobamide; 5,6-Dimethylbenzimidazolyl-Co-5'-deoxy-5'-adenosylcobamide; Calomide; Cobalamin coenzyme; Coenzyme B12; DMBC coenzyme; Dibencozide; Funacomide | 20 | ec:2.7.8.26 |
| cpd:C00144 |  | GMP; Guanosine 5'-phosphate; Guanosine monophosphate; Guanosine 5'-monophosphate; Guanylic acid | 20 | ec:2.7.8.26 |
| cpd:C06509 |  | Adenosyl cobinamide phosphate | 20 | ec:6.3.1.10 ec:2.7.7.62 ec:2.7.1.156 |
| cpd:C05775 |  | alpha-Ribazole; N1-(alpha-D-Ribosyl)-5,6-dimethylbenzimidazole | 20 | ec:2.7.8.26 |
| cpd:C00022 |  | Pyruvate; Pyruvic acid; 2-Oxopropanoate; 2-Oxopropanoic acid; Pyroracemic acid | 20 | ec:2.6.1.66 ec:4.1.3.38 ec:4.4.1.25 ec:1.1.2.4 ec:2.2.1.6 ec:2.8.1.1 ec:2.8.1.2 ec:4.4.1.8 |
| cpd:C00004 |  | NADH; DPNH; Reduced nicotinamide adenine dinucleotide | 20 | ec:1.1.1.60 ec:1.1.1.14 ec:1.1.1.6 ec:1.5.1.3 ec:1.6.1.2 ec:1.3.1.44 ec:1.2.1.71 ec:1.1.1.94 ec:1.2.1.19 ec:1.1.1.284 ec:1.1.1.1 ec:1.2.1.2 |
| cpd:C00044 |  | GTP; Guanosine 5'-triphosphate | 19 | ec:2.7.7.62 ec:2.7.1.156 |
| cpd:C00035 |  | GDP; Guanosine 5'-diphosphate; Guanosine diphosphate | 19 | ec:2.7.7.62 ec:2.7.1.156 |
| cpd:C01801 |  | Deoxyribose; 2-Deoxy-D-erythro-pentose; Thyminose; 2-Deoxy-D-ribose | 17 | ec:2.7.1.15 |
| cpd:C00673 |  | 2-Deoxy-D-ribose 5-phosphate | 17 | ec:2.7.1.15 |
| cpd:C00121 |  | D-Ribose | 17 | ec:2.7.1.15 |
| cpd:C00009 |  | Orthophosphate; Phosphate; Phosphoric acid; Orthophosphoric acid | 17 | ec:2.7.9.3 ec:6.3.1.10 ec:6.3.4.21 ec:3.1.3.11 ec:2.5.1.6 ec:2.4.2.28 ec:1.2.1.38 ec:3.1.3.18 ec:4.2.3.1 ec:3.6.3.4 ec:6.3.4.20 |
| cpd:C01146 |  | 2-Hydroxy-3-oxopropanoate; Tartronate semialdehyde | 16 | ec:5.3.1.22 ec:1.1.1.60 ec:4.1.1.47 |
| cpd:C00061 |  | FMN; Riboflavin-5-phosphate; Flavin mononucleotide | 15 | ec:1.5.1.38 ec:2.7.1.26 ec:1.14.14.5 ec:2.7.7.2 |
| cpd:C20248 |  | 7-Carboxy-7-carbaguanine; 7-Carboxy-7-deazaguanine | 14 | ec:4.3.99.3 ec:6.3.4.20 |
| cpd:C00049 |  | L-Aspartate; L-Aspartic acid; 2-Aminosuccinic acid; L-Asp | 14 | ec:6.3.4.5 ec:3.5.1.1 ec:1.4.3.16 |
| cpd:C01847 |  | Reduced FMN; FMNH2 | 14 | ec:1.5.1.38 ec:1.14.14.5 |
| cpd:C00067 |  | Formaldehyde; Methanal; Oxomethane; Oxomethylene; Methylene oxide; Formalin | 13 | ec:1.14.14.5 ec:4.4.1.22 |
| cpd:C00048 |  | Glyoxylate; Glyoxalate; Glyoxylic acid | 13 | ec:4.1.3.1 ec:4.1.1.47 |
| cpd:C11145 |  | Methanesulfonic acid; Methanesulfonate | 11 | ec:1.14.14.5 |
| cpd:C00010 |  | CoA; Coenzyme A; CoA-SH | 11 | ec:2.3.1.30 ec:2.3.3.13 ec:3.1.2.1 ec:2.3.1.117 ec:2.3.1.109 |
| cpd:C00059 |  | Sulfate; Sulfuric acid | 9 | ec:2.7.7.4 |
| cpd:C00042 |  | Succinate; Succinic acid; Butanedionic acid; Ethylenesuccinic acid | 9 | ec:4.1.3.1 ec:3.5.1.96 |
| cpd:C00027 |  | Hydrogen peroxide; H2O2; Oxydol | 9 | ec:1.4.3.19 ec:1.4.3.16 |
| cpd:C00025 |  | L-Glutamate; L-Glutamic acid; L-Glutaminic acid; Glutamate | 9 | ec:6.3.5.1 ec:2.3.2.2 ec:2.6.1.81 ec:2.6.1.82 ec:2.6.1.11 ec:3.5.1.96 |
| cpd:C00365 |  | dUMP; Deoxyuridylic acid; Deoxyuridine monophosphate; Deoxyuridine 5'-phosphate; 2'-Deoxyuridine 5'-phosphate | 9 | ec:2.1.1.45 ec:3.6.1.23 ec:2.7.4.9 |
| cpd:C00311 |  | Isocitrate; Isocitric acid; 1-Hydroxytricarballylic acid; 1-Hydroxypropane-1,2,3-tricarboxylic acid | 8 | ec:4.1.3.1 |
| cpd:C00224 |  | Adenylyl sulfate; Adenosine 5'-phosphosulfate; APS; 5'-Adenylyl sulfate | 8 | ec:2.7.7.4 |
| cpd:C05840 |  | Iminoaspartate; Iminoaspartic acid; Iminosuccinate | 8 | ec:1.4.3.16 |
| cpd:C00091 |  | Succinyl-CoA; Succinyl coenzyme A | 8 | ec:2.3.1.117 ec:2.3.1.109 |
| cpd:C00051 |  | Glutathione; 5-L-Glutamyl-L-cysteinylglycine; N-(N-gamma-L-Glutamyl-L-cysteinyl)glycine; gamma-L-Glutamyl-L-cysteinyl-glycine; GSH; Reduced glutathione | 8 | ec:2.3.2.2 ec:3.1.2.12 ec:4.4.1.22 |
| cpd:C00036 |  | Oxaloacetate; Oxalacetic acid; Oxaloacetic acid; 2-Oxobutanedioic acid; 2-Oxosuccinic acid; keto-Oxaloacetate | 8 | ec:1.4.3.16 |
| cpd:C05697 |  | Selenate; Selenic acid | 8 | ec:2.7.7.4 |
| cpd:C05686 |  | Adenylylselenate; Adenosine-5'-phosphoselenate | 8 | ec:2.7.7.4 |
| cpd:C00364 |  | dTMP; Thymidine 5'-phosphate; Deoxythymidine 5'-phosphate; Thymidylic acid; 5'-Thymidylic acid; Thymidine monophosphate; Deoxythymidylic acid; Thymidylate | 8 | ec:2.1.1.45 ec:2.7.4.9 |
| cpd:C15996 |  | 7-Cyano-7-carbaguanine; 7-Cyano-7-deazaguanine | 7 | ec:6.3.4.20 |
| cpd:C11355 |  | 4-Amino-4-deoxychorismate; ADC | 7 | ec:4.1.3.38 |
| cpd:C00283 |  | Hydrogen sulfide; Hydrogen-sulfide; H2S; Sulfide | 7 | ec:1.8.1.2 ec:2.5.1.47 ec:4.4.1.8 |
| cpd:C20239 |  | 6-Carboxy-5,6,7,8-tetrahydropterin; 6-Carboxytetrahydropterin | 7 | ec:4.3.99.3 |
| cpd:C01346 |  | dUDP; 2'-Deoxyuridine 5'-diphosphate | 7 | ec:2.7.4.9 |
| cpd:C04462 |  | N-Succinyl-2-L-amino-6-oxoheptanedioate; N-Succinyl-L-2-amino-6-oxoheptanedioate; N-Succinyl-L-2-amino-6-oxopimelate; N-Succinyl-2-amino-6-oxo-L-pimelic acid; N-Succinyl-epsilon-keto-L-aminopimelic acid; (S)-2-(Succinylamino)-6-oxoheptanedioate | 7 | ec:2.3.1.117 |
| cpd:C05125 |  | 2-(alpha-Hydroxyethyl)thiamine diphosphate; 2-Hydroxyethyl-ThPP | 7 | ec:2.2.1.6 |
| cpd:C00568 |  | 4-Aminobenzoate; ABEE; 4-Aminobenzoic acid; p-Aminobenzoate | 7 | ec:4.1.3.38 |
| cpd:C00900 |  | 2-Acetolactate | 7 | ec:2.2.1.6 |
| cpd:C00109 |  | 2-Oxobutanoate; 2-Ketobutyric acid; 2-Oxobutyric acid; 2-Oxobutyrate; 2-Oxobutanoic acid; alpha-Ketobutyric acid; alpha-Ketobutyrate | 7 | ec:2.2.1.6 |
| cpd:C00068 |  | Thiamin diphosphate; Thiamine diphosphate; Thiamin pyrophosphate; TPP; ThPP | 7 | ec:2.2.1.6 |
| cpd:C00058 |  | Formate; Methanoic acid; Formic acid | 7 | ec:3.1.2.12 ec:1.2.99.5 ec:1.2.1.2 |
| cpd:C00030 |  | Reduced acceptor; AH2; Hydrogen-donor; Donor | 7 | ec:1.3.8.7 ec:2.8.1.7 ec:1.6.5.3 ec:4.4.1.16 ec:1.2.99.5 |
| cpd:C00028 |  | Acceptor; Hydrogen-acceptor; A; Oxidized donor | 7 | ec:1.3.8.7 ec:2.8.1.7 ec:1.6.5.3 ec:4.4.1.16 ec:1.2.99.5 |
| cpd:C06010 |  | (S)-2-Acetolactate; (S)-2-Hydroxy-2-methyl-3-oxobutanoate | 7 | ec:2.2.1.6 |
| cpd:C03972 |  | 2,3,4,5-Tetrahydrodipicolinate; delta1-Piperidine-2,6-dicarboxylate; L-2,3,4,5-Tetrahydrodipicolinate; (S)-2,3,4,5-Tetrahydropyridine-2,6-dicarboxylate | 7 | ec:2.3.1.117 |
| cpd:C06006 |  | (S)-2-Aceto-2-hydroxybutanoate; (S)-2-Hydroxy-2-ethyl-3-oxobutanoate | 7 | ec:2.2.1.6 |
| cpd:C00363 |  | dTDP; Deoxythymidine 5'-diphosphate | 7 | ec:2.7.4.9 |
| cpd:C00168 |  | Hydroxypyruvate; Hydroxypyruvic acid; 3-Hydroxypyruvate; 3-Hydroxypyruvic acid | 6 | ec:5.3.1.22 |
| cpd:C00126 |  | Ferrocytochrome c; Cytochrome c2+; Reduced cytochrome c | 6 | ec:1.1.2.4 ec:1.9.3.1 |
| cpd:C00125 |  | Ferricytochrome c; Cytochrome c3+ | 6 | ec:1.1.2.4 ec:1.9.3.1 |
| cpd:C00343 |  | Thioredoxin disulfide; Oxidized thioredoxin; Thioredoxin sulfide | 5 | ec:2.5.1.47 ec:1.8.4.8 |
| cpd:C00342 |  | Thioredoxin; Reduced thioredoxin | 5 | ec:2.5.1.47 ec:1.8.4.8 |
| cpd:C01419 |  | Cys-Gly; L-Cysteinylglycine | 5 | ec:2.3.2.2 ec:3.4.11.1 |
| cpd:C00258 |  | D-Glycerate; Glycerate; (R)-Glycerate; Glyceric acid | 5 | ec:1.1.1.60 |
| cpd:C00152 |  | L-Asparagine; 2-Aminosuccinamic acid | 5 | ec:3.5.1.1 |
| cpd:C05729 |  | R-S-Cysteinylglycine | 5 | ec:2.3.2.2 ec:3.4.11.1 |
| cpd:C01001 |  | Formylmethanofuran | 4 | ec:2.3.1.101 ec:1.2.99.5 |
| cpd:C05951 |  | Leukotriene D4; LTD4 | 4 | ec:2.3.2.2 |
| cpd:C00245 |  | Taurine; 2-Aminoethanesulfonic acid; Aminoethylsulfonic acid | 4 | ec:2.3.2.2 |
| cpd:C03363 |  | 5-L-Glutamyl amino acid; L-gamma-Glutamyl amino acid | 4 | ec:2.3.2.2 |
| cpd:C02320 |  | R-S-Glutathione | 4 | ec:2.3.2.2 |
| cpd:C00151 |  | L-Amino acid; L-2-Amino acid | 4 | ec:2.3.2.2 |
| cpd:C03740 |  | (5-L-Glutamyl)-L-amino acid; L-gamma-Glutamyl-L-amino acid | 4 | ec:2.3.2.2 |
| cpd:C05844 |  | 5-L-Glutamyl-taurine; 5-Glutamyl-taurine; Glutaurine | 4 | ec:2.3.2.2 |
| cpd:C00097 |  | L-Cysteine; L-2-Amino-3-mercaptopropionic acid | 4 | ec:6.3.2.5 ec:3.4.11.1 ec:2.5.1.47 ec:4.1.1.36 ec:4.4.1.8 |
| cpd:C06114 |  | gamma-Glutamyl-beta-aminopropiononitrile; gamma-Glutamyl-3-aminopropiononitrile | 4 | ec:2.3.2.2 |
| cpd:C00862 |  | Methanofuran; Carbon dioxide reduction factor | 4 | ec:2.3.1.101 ec:1.2.99.5 |
| cpd:C16399 |  | 2,4-Diamino-6-hydroxylaminotoluene | 4 | ec:1.12.99.6 |
| cpd:C16396 |  | 2,4-Diamino-6-nitrotoluene | 4 | ec:1.12.99.6 |
| cpd:C00054 |  | Adenosine 3',5'-bisphosphate; PAP; 3'-Phosphoadenylate; Phosphoadenosine phosphate | 4 | ec:1.8.4.8 |
| cpd:C00053 |  | 3'-Phosphoadenylyl sulfate; 3'-Phosphoadenosine 5'-phosphosulfate; 3'-Phospho-5'-adenylyl sulfate; PAPS | 4 | ec:1.8.4.8 |
| cpd:C02166 |  | Leukotriene C4; LTC4 | 4 | ec:2.3.2.2 |
| cpd:C00045 |  | Amino acid; Amino acids | 4 | ec:2.3.2.2 |
| cpd:C03193 |  | (5-L-Glutamyl)-peptide | 4 | ec:2.3.2.2 |
| cpd:C00037 |  | Glycine; Aminoacetic acid; Gly | 4 | ec:3.4.11.1 ec:1.4.3.19 ec:6.1.1.14 |
| cpd:C05695 |  | gamma-Glutamyl-Se-methylselenocysteine; 5-L-Glutamyl-Se-methylselenocysteine | 4 | ec:2.3.2.2 |
| cpd:C00024 |  | Acetyl-CoA; Acetyl coenzyme A | 4 | ec:2.3.1.30 ec:2.3.3.13 ec:3.1.2.1 ec:2.8.3.1 |
| cpd:C05689 |  | Se-Methyl-L-selenocysteine | 4 | ec:2.3.2.2 |
| cpd:C00012 |  | Peptide | 4 | ec:2.3.2.2 |
| cpd:C05711 |  | gamma-Glutamyl-beta-cyanoalanine | 4 | ec:2.3.2.2 |
| cpd:C14180 |  | S-(Hydroxymethyl)glutathione | 4 | ec:4.4.1.22 ec:1.1.1.284 ec:1.1.1.1 |
| cpd:C05670 |  | 3-Aminopropiononitrile; beta-Aminopropionitrile | 4 | ec:2.3.2.2 |
| cpd:C02512 |  | 3-Cyano-L-alanine; L-3-Cyanoalanine; L-beta-Cyanoalanine | 4 | ec:2.3.2.2 |
| cpd:C01031 |  | S-Formylglutathione | 4 | ec:3.1.2.12 ec:1.1.1.284 ec:1.1.1.1 |
| cpd:C00320 |  | Thiosulfate; Hyposulfite | 3 | ec:2.5.1.47 ec:2.8.1.1 ec:2.8.1.2 |
| cpd:C00229 |  | Acyl-carrier protein; ACP; [Acyl-carrier protein]; Holo-[acyl-carrier protein] | 3 | ec:2.3.1.40 ec:6.2.1.20 |
| cpd:C00536 |  | Triphosphate; Triphosphoric acid; Tripolyphosphate; Inorganic triphosphate | 3 | ec:2.5.1.6 ec:2.5.1.17 |
| cpd:C00136 |  | Butanoyl-CoA; Butyryl-CoA | 3 | ec:1.3.8.7 ec:1.3.1.44 |
| cpd:C00086 |  | Urea; Carbamide | 3 | ec:3.5.1.5 |
| cpd:C00085 |  | D-Fructose 6-phosphate; D-Fructose 6-phosphoric acid; Neuberg ester | 3 | ec:3.1.3.11 ec:2.2.1.1 ec:5.3.1.27 |
| cpd:C00877 |  | Crotonoyl-CoA; Crotonyl-CoA; 2-Butenoyl-CoA; trans-But-2-enoyl-CoA; But-2-enoyl-CoA; (E)-But-2-enoyl-CoA | 3 | ec:1.3.8.7 ec:1.3.1.44 |
| cpd:C00073 |  | L-Methionine; Methionine; L-2-Amino-4methylthiobutyric acid | 3 | ec:2.8.1.8 ec:2.5.1.6 ec:2.1.1.13 |
| cpd:C00100 |  | Propanoyl-CoA; Propionyl-CoA; Propionyl coenzyme A | 3 | ec:1.3.8.7 ec:2.8.3.1 |
| cpd:C01528 |  | Hydrogen selenide; Selenide | 3 | ec:2.7.9.3 ec:2.8.1.7 ec:2.5.1.47 ec:4.4.1.16 |
| cpd:C00041 |  | L-Alanine; L-2-Aminopropionic acid; L-alpha-Alanine | 3 | ec:2.6.1.66 ec:2.8.1.7 ec:4.4.1.16 ec:6.1.1.7 |
| cpd:C05345 |  | beta-D-Fructose 6-phosphate | 3 | ec:3.1.3.11 ec:2.2.1.1 ec:5.3.1.27 |
| cpd:C00033 |  | Acetate; Acetic acid; Ethanoic acid | 3 | ec:2.5.1.47 ec:3.1.2.1 ec:2.8.3.1 |
| cpd:C00026 |  | 2-Oxoglutarate; Oxoglutaric acid; 2-Ketoglutaric acid; alpha-Ketoglutaric acid | 3 | ec:2.6.1.81 ec:2.6.1.82 ec:2.6.1.11 |
| cpd:C00019 |  | S-Adenosyl-L-methionine; S-Adenosylmethionine; AdoMet; SAM | 3 | ec:2.8.1.8 ec:2.5.1.6 ec:2.1.1.197 |
| cpd:C00016 |  | FAD; Flavin adenine dinucleotide | 3 | ec:1.3.8.7 ec:2.7.1.26 ec:2.7.7.2 |
| cpd:C04570 |  | Reduced electron-transferring flavoprotein; Reduced electron-transfer flavoprotein | 2 | ec:1.3.8.7 |
| cpd:C15980 |  | (S)-2-Methylbutanoyl-CoA | 2 | ec:1.3.8.7 |
| cpd:C14099 |  | 2-Naphthaldehyde; 2-Naphthalenecarboxaldehyde | 2 | ec:1.1.1.284 ec:1.1.1.1 |
| cpd:C01352 |  | FADH2 | 2 | ec:1.3.8.7 |
| cpd:C14090 |  | 1-Naphthaldehyde; 1-Formylnaphthalene | 2 | ec:1.1.1.284 ec:1.1.1.1 |
| cpd:C05223 |  | Dodecanoyl-[acyl-carrier protein]; Dodecanoyl-[acp]; Lauroyl-[acyl-carrier protein] | 2 |  |
| cpd:C03069 |  | 3-Methylcrotonyl-CoA; 3-Methylbut-2-enoyl-CoA; 3-Methylcrotonoyl-CoA; Dimethylacryloyl-CoA | 2 | ec:1.3.8.7 |
| cpd:C03460 |  | 2-Methylprop-2-enoyl-CoA; Methacrylyl-CoA; Methylacrylyl-CoA | 2 | ec:1.3.8.7 |
| cpd:C14089 |  | 1-Hydroxymethylnaphthalene; 1-Naphthalenemethanol | 2 | ec:1.1.1.284 ec:1.1.1.1 |
| cpd:C05577 |  | 3,4-Dihydroxymandelaldehyde; 3,4-Dihydroxyphenylglycolaldehyde | 2 | ec:1.1.1.284 ec:1.1.1.1 |
| cpd:C05576 |  | 3,4-Dihydroxyphenylethyleneglycol | 2 | ec:1.1.1.284 ec:1.1.1.1 |
| cpd:C06251 |  | Lauroyl-KDO2-lipid IV(A) | 2 |  |
| cpd:C16596 |  | 5-Phenyl-1,3-oxazinane-2,4-dione | 2 | ec:1.1.1.284 ec:1.1.1.1 |
| cpd:C16595 |  | 4-Hydroxy-5-phenyltetrahydro-1,3-oxazin-2-one | 2 | ec:1.1.1.284 ec:1.1.1.1 |
| cpd:C02412 |  | Glycyl-tRNA(Gly) | 2 | ec:6.1.1.14 |
| cpd:C00256 |  | (R)-Lactate; D-Lactate; D-Lactic acid; D-2-Hydroxypropanoic acid; D-2-Hydroxypropionic acid | 2 | ec:1.1.2.4 |
| cpd:C16587 |  | 3-Carbamoyl-2-phenylpropionaldehyde | 2 | ec:1.1.1.284 ec:1.1.1.1 |
| cpd:C16586 |  | 2-Phenyl-1,3-propanediol monocarbamate | 2 | ec:1.1.1.284 ec:1.1.1.1 |
| cpd:C00630 |  | 2-Methylpropanoyl-CoA; 2-Methylpropionyl-CoA; Isobutyryl-CoA | 2 | ec:1.3.8.7 |
| cpd:C00231 |  | D-Xylulose 5-phosphate | 2 | ec:2.2.1.1 ec:5.1.3.1 |
| cpd:C06613 |  | trans-3-Chloroallyl aldehyde; trans-3-Chloro-2-propenal | 2 | ec:1.1.1.284 ec:1.1.1.1 |
| cpd:C06612 |  | cis-3-Chloro-2-propene-1-ol; cis-3-Chloroallyl alcohol | 2 | ec:1.1.1.284 ec:1.1.1.1 |
| cpd:C06611 |  | trans-3-Chloro-2-propene-1-ol; trans-3-Chloroallyl alcohol | 2 | ec:1.1.1.284 ec:1.1.1.1 |
| cpd:C05932 |  | N-Succinyl-L-glutamate 5-semialdehyde; (2S)-2-(3-Carboxypropanoylamino)-5-oxopentanoic acid | 2 | ec:2.6.1.81 ec:1.2.1.71 |
| cpd:C05931 |  | N-Succinyl-L-glutamate; (2S)-2-(3-Carboxypropanoylamino)pentanedioic acid | 2 | ec:1.2.1.71 ec:3.5.1.96 |
| cpd:C00226 |  | Primary alcohol; 1-Alcohol | 2 | ec:1.1.1.284 ec:1.1.1.1 |
| cpd:C16551 |  | Alcophosphamide | 2 | ec:1.1.1.284 ec:1.1.1.1 |
| cpd:C00184 |  | Glycerone; Dihydroxyacetone; 1,3-Dihydroxyacetone; 1,3-Dihydroxy-2-propanone; 1,3-Dihydroxypropan-2-one | 2 | ec:1.1.1.6 |
| cpd:C03415 |  | N2-Succinyl-L-ornithine; (2S)-5-Amino-2-(3-carboxypropanoylamino)pentanoic acid | 2 | ec:2.6.1.81 ec:3.5.3.23 |
| cpd:C07645 |  | Aldophosphamide | 2 | ec:1.1.1.284 ec:1.1.1.1 |
| cpd:C00979 |  | O-Acetyl-L-serine; O3-Acetyl-L-serine | 2 | ec:2.3.1.30 ec:2.5.1.47 |
| cpd:C01250 |  | N-Acetyl-L-glutamate 5-semialdehyde; 2-Acetamido-5-oxopentanoate | 2 | ec:1.2.1.38 ec:2.6.1.11 |
| cpd:C01642 |  | tRNA(Gly) | 2 | ec:6.1.1.14 |
| cpd:C00160 |  | Glycolate; Glycolic acid; Hydroxyacetic acid | 2 | ec:3.8.1.2 ec:3.1.3.18 |
| cpd:C00555 |  | 4-Aminobutyraldehyde; 4-Aminobutanal | 2 | ec:2.6.1.82 ec:1.2.1.19 |
| cpd:C00155 |  | L-Homocysteine; L-2-Amino-4-mercaptobutyric acid | 2 | ec:4.4.1.8 ec:2.1.1.13 |
| cpd:C00154 |  | Palmitoyl-CoA; Hexadecanoyl-CoA | 2 | ec:1.3.8.7 |
| cpd:C03345 |  | 2-Methylbut-2-enoyl-CoA; trans-2-Methylbut-2-enoyl-CoA; Tiglyl-CoA; (E)-2-Methylcrotonoyl-CoA; Methylcrotonoyl-CoA; Methylcrotonyl-CoA; Tigloyl-CoA; 2-Methylcrotanoyl-CoA | 2 | ec:1.3.8.7 |
| cpd:C06899 |  | Chloral hydrate | 2 | ec:1.1.1.284 ec:1.1.1.1 |
| cpd:C00141 |  | 3-Methyl-2-oxobutanoic acid; 3-Methyl-2-oxobutyric acid; 3-Methyl-2-oxobutanoate; 2-Oxo-3-methylbutanoate; 2-Oxoisovalerate; 2-Oxoisopentanoate; alpha-Ketovaline; 2-Ketovaline; 2-Keto-3-methylbutyric acid | 2 | ec:2.6.1.66 ec:2.3.3.13 |
| cpd:C03296 |  | N2-Succinyl-L-arginine; (2S)-2-(3-Carboxypropanoylamino)-5-(diaminomethylideneamino)pentanoic acid | 2 | ec:2.3.1.109 ec:3.5.3.23 |
| cpd:C00894 |  | Propenoyl-CoA; Acryloyl-CoA; Acrylyl-CoA | 2 | ec:1.3.8.7 |
| cpd:C00084 |  | Acetaldehyde; Ethanal | 2 | ec:1.1.1.284 ec:1.1.1.1 |
| cpd:C06506 |  | Adenosyl cobyrinate a,c diamide; Adenosyl cobyrinate diamide; Adenosylcob(III)yrinic acid a,c-diamide; Adenosylcobyrinic acid a,c-diamide | 2 | ec:2.5.1.17 |
| cpd:C06505 |  | Cob(I)yrinate a,c diamide; Cob(I)yrinate diamide; Cob(I)yrinic acid a,c-diamide | 2 | ec:2.5.1.17 |
| cpd:C02593 |  | Tetradecanoyl-CoA; Myristoyl-CoA | 2 | ec:1.3.8.7 |
| cpd:C15809 |  | Iminoglycine; Iminoacetic acid; 2-Iminoacetate | 2 | ec:1.4.3.19 |
| cpd:C00119 |  | 5-Phospho-alpha-D-ribose 1-diphosphate; 5-Phosphoribosyl diphosphate; 5-Phosphoribosyl 1-pyrophosphate; PRPP | 2 | ec:6.3.4.21 ec:2.7.6.1 |
| cpd:C00118 |  | D-Glyceraldehyde 3-phosphate; (2R)-2-Hydroxy-3-(phosphonooxy)-propanal; Glyceraldehyde 3-phosphate | 2 | ec:2.2.1.1 ec:5.3.1.1 |
| cpd:C00116 |  | Glycerol; Glycerin; 1,2,3-Trihydroxypropane; 1,2,3-Propanetriol | 2 | ec:1.1.1.6 |
| cpd:C00473 |  | Retinol; all-trans-Retinol; Vitamin A; Vitamin A1 | 2 | ec:1.1.1.284 ec:1.1.1.1 |
| cpd:C00111 |  | Glycerone phosphate; Dihydroxyacetone phosphate | 2 | ec:1.1.1.94 ec:5.3.1.1 |
| cpd:C00071 |  | Aldehyde; RCHO | 2 | ec:1.1.1.284 ec:1.1.1.1 |
| cpd:C01944 |  | Octanoyl-CoA | 2 | ec:1.3.8.7 |
| cpd:C00469 |  | Ethanol; Ethyl alcohol; Methylcarbinol | 2 | ec:1.1.1.284 ec:1.1.1.1 |
| cpd:C05774 |  | Cobinamide; Cob(I)inamide | 2 | ec:2.5.1.17 |
| cpd:C07490 |  | Trichloroethanol; 2,2,2-Trichloroethanol | 2 | ec:1.1.1.284 ec:1.1.1.1 |
| cpd:C00101 |  | Tetrahydrofolate; 5,6,7,8-Tetrahydrofolate; Tetrahydrofolic acid; THF; (6S)-Tetrahydrofolate; (6S)-Tetrahydrofolic acid; (6S)-THFA | 2 | ec:1.5.1.3 ec:2.1.1.13 |
| cpd:C00857 |  | Deamino-NAD+; Deamido-NAD+; Deamido-NAD | 2 | ec:6.3.5.1 ec:2.7.7.1 |
| cpd:C04327 |  | 4-Methyl-5-(2-phosphoethyl)-thiazole; 4-Methyl-5-(2-phosphono-oxyethyl)-thiazole | 2 | ec:2.5.1.3 |
| cpd:C06025 |  | Di[3-deoxy-D-manno-octulosonyl]-lipid IV(A); KDO2-lipid IV(A) | 2 |  |
| cpd:C03221 |  | 2-trans-Dodecenoyl-CoA; (2E)-Dodec-2-enoyl-CoA; (2E)-Dodecenoyl-CoA | 2 | ec:1.3.8.7 |
| cpd:C05698 |  | Selenohomocysteine | 2 | ec:4.4.1.8 ec:2.1.1.13 |
| cpd:C02939 |  | 3-Methylbutanoyl-CoA; Isovaleryl-CoA | 2 | ec:1.3.8.7 |
| cpd:C05335 |  | L-Selenomethionine | 2 | ec:2.5.1.6 ec:2.1.1.13 |
| cpd:C04253 |  | Electron-transferring flavoprotein; Electron-transfer flavoprotein | 2 | ec:1.3.8.7 |
| cpd:C00415 |  | Dihydrofolate; Dihydrofolic acid; 7,8-Dihydrofolate; 7,8-Dihydrofolic acid; 7,8-Dihydropteroylglutamate | 2 | ec:2.1.1.45 ec:1.5.1.3 |
| cpd:C05688 |  | L-Selenocysteine | 2 | ec:2.8.1.7 ec:2.5.1.47 ec:4.4.1.16 |
| cpd:C00376 |  | Retinal; Vitamin A aldehyde; Retinene; all-trans-Retinal; all-trans-Vitamin A aldehyde; all-trans-Retinene | 2 | ec:1.1.1.284 ec:1.1.1.1 |
| cpd:C16348 |  | cis-3-Chloroallyl aldehyde; cis-3-Chloro-2-propenal | 2 | ec:1.1.1.284 ec:1.1.1.1 |
| cpd:C05276 |  | trans-Oct-2-enoyl-CoA; (2E)-Octenoyl-CoA | 2 | ec:1.3.8.7 |
| cpd:C05275 |  | trans-Dec-2-enoyl-CoA; (2E)-Decenoyl-CoA | 2 | ec:1.3.8.7 |
| cpd:C05274 |  | Decanoyl-CoA | 2 | ec:1.3.8.7 |
| cpd:C05273 |  | trans-Tetradec-2-enoyl-CoA; (2E)-Tetradecenoyl-CoA | 2 | ec:1.3.8.7 |
| cpd:C05272 |  | trans-Hexadec-2-enoyl-CoA; trans-2-Hexadecenoyl-CoA; (2E)-Hexadecenoyl-CoA | 2 | ec:1.3.8.7 |
| cpd:C05271 |  | trans-Hex-2-enoyl-CoA; (2E)-Hexenoyl-CoA | 2 | ec:1.3.8.7 |
| cpd:C05270 |  | Hexanoyl-CoA | 2 | ec:1.3.8.7 |
| cpd:C01832 |  | Lauroyl-CoA; Lauroyl coenzyme A; Dodecanoyl-CoA | 2 | ec:1.3.8.7 |
| cpd:C02909 |  | (2-Naphthyl)methanol; 2-Naphthalenemethanol; 2-Hydroxymethylnaphthalene | 2 | ec:1.1.1.284 ec:1.1.1.1 |
| cpd:C03492 |  | D-4'-Phosphopantothenate; (R)-4'-Phosphopantothenate | 1 | ec:6.3.2.5 ec:4.1.1.36 |
| cpd:C00334 |  | 4-Aminobutanoate; 4-Aminobutanoic acid; 4-Aminobutyrate; 4-Aminobutyric acid; gamma-Aminobutyric acid; GABA | 1 | ec:1.2.1.19 |
| cpd:C01013 |  | 3-Hydroxypropanoate; 3-Hydroxypropanoic acid; 3-Hydroxypropionate; 3-Hydroxypropionic acid; Hydracrylic acid | 1 |  |
| cpd:C03127 |  | L-Isoleucyl-tRNA(Ile) | 1 | ec:6.1.1.5 |
| cpd:C00327 |  | L-Citrulline; 2-Amino-5-ureidovaleric acid; Citrulline | 1 | ec:6.3.4.5 |
| cpd:C05198 |  | 5'-Deoxyadenosine | 1 | ec:2.8.1.8 |
| cpd:C03512 |  | L-Tryptophanyl-tRNA(Trp) | 1 | ec:6.1.1.2 |
| cpd:C00718 |  | Amylose; Amylose chain; (1,4-alpha-D-Glucosyl)n; (1,4-alpha-D-Glucosyl)n+1; (1,4-alpha-D-Glucosyl)n-1; 4-{(1,4)-alpha-D-Glucosyl}(n-1)-D-glucose; 1,4-alpha-D-Glucan | 1 | ec:2.4.1.25 |
| cpd:C01755 |  | Thiocyanate; Thiocyanic acid | 1 | ec:2.8.1.1 ec:2.8.1.2 |
| cpd:C00315 |  | Spermidine; N-(3-Aminopropyl)-1,4-butane-diamine | 1 |  |
| cpd:C00279 |  | D-Erythrose 4-phosphate | 1 | ec:2.2.1.1 |
| cpd:C04144 |  | Tetrahydropteroyltri-L-glutamate | 1 | ec:2.1.1.13 |
| cpd:C05973 |  | 2-Acyl-sn-glycero-3-phosphoethanolamine; L-1-Lysophosphatidylethanolamine; O-(2-Acyl-sn-glycero-3-phospho)-ethanolamine | 1 | ec:2.3.1.40 ec:6.2.1.20 |
| cpd:C00268 |  | Dihydrobiopterin; 6,7-Dihydrobiopterin; Quinoid-dihydrobiopterin; (6R)-6-(L-erythro-1,2-Dihydroxypropyl)-7,8-dihydro-6H-pterin | 1 | ec:4.2.1.96 |
| cpd:C16239 |  | Lipoyl-[acp]; Lipoyl-[acyl carrier protein]; Lipoyl-ACP | 1 | ec:2.8.1.8 |
| cpd:C16237 |  | Protein N6-(lipoyl)lysine | 1 | ec:2.8.1.8 |
| cpd:C16236 |  | Protein N6-(octanoyl)lysine | 1 | ec:2.8.1.8 |
| cpd:C05172 |  | Selenophosphoric acid; Selenophosphate | 1 | ec:2.7.9.3 |
| cpd:C04133 |  | N-Acetyl-L-glutamate 5-phosphate; N-Acetyl-L-glutamyl 5-phosphate | 1 | ec:1.2.1.38 |
| cpd:C00255 |  | Riboflavin; Lactoflavin; 7,8-Dimethyl-10-ribitylisoalloxazine; Vitamin B2 | 1 | ec:2.7.1.26 ec:2.7.7.2 |
| cpd:C04489 |  | 5-Methyltetrahydropteroyltri-L-glutamate | 1 | ec:2.1.1.13 |
| cpd:C04122 |  | D-1-Aminopropan-2-ol O-phosphate; (R)-1-Aminopropan-2-yl phosphate | 1 | ec:6.3.1.10 |
| cpd:C01327 |  | Hydrochloric acid; HCl; Hydrogen chloride; Hydrochloride | 1 | ec:3.8.1.2 |
| cpd:C01326 |  | Hydrogen cyanide; HCN | 1 | ec:2.8.1.1 ec:2.8.1.2 |
| cpd:C00638 |  | Long-chain fatty acid; Higher fatty acid | 1 | ec:2.3.1.40 ec:6.2.1.20 |
| cpd:C02350 |  | (S)(+)-Allantoin; (S)-Allantoin | 1 | ec:5.1.99.3 |
| cpd:C00631 |  | 2-Phospho-D-glycerate; D-Glycerate 2-phosphate; 2-Phospho-(R)-glycerate | 1 | ec:5.4.2.12 |
| cpd:C01274 |  | 5-Formyl-5,6,7,8-tetrahydromethanopterin; 5-Formyl-H4MPT | 1 | ec:2.3.1.101 |
| cpd:C00199 |  | D-Ribulose 5-phosphate | 1 | ec:5.1.3.1 |
| cpd:C00197 |  | 3-Phospho-D-glycerate; D-Glycerate 3-phosphate; 3-Phospho-(R)-glycerate; 3-Phosphoglycerate | 1 | ec:5.4.2.12 |
| cpd:C15522 |  | 4a-Hydroxytetrahydrobiopterin; 4a-Hydroxy-5,6,4,8-tetrahydrobiopterin; (6R)-6-(L-erythro-1,2-Dihydroxypropyl)-5,6,7,8-tetrahydro-4a-hydroxypterin | 1 | ec:4.2.1.96 |
| cpd:C02348 |  | (R)(-)-Allantoin; (R)-Allantoin | 1 | ec:5.1.99.3 |
| cpd:C00988 |  | 2-Phosphoglycolate; Phosphoglycolic acid | 1 | ec:3.1.3.18 |
| cpd:C00222 |  | 3-Oxopropanoate; Malonate semialdehyde | 1 |  |
| cpd:C00188 |  | L-Threonine; 2-Amino-3-hydroxybutyric acid | 1 | ec:4.2.3.1 |
| cpd:C00186 |  | (S)-Lactate; L-Lactate; L-Lactic acid | 1 | ec:2.8.3.1 |
| cpd:C00183 |  | L-Valine; 2-Amino-3-methylbutyric acid | 1 | ec:2.6.1.66 |
| cpd:C03375 |  | Norspermidine; Bis(3-aminopropyl)amine; 3,3'-Iminobispropylamine | 1 |  |
| cpd:C01652 |  | tRNA(Trp) | 1 | ec:6.1.1.2 |
| cpd:C02291 |  | L-Cystathionine | 1 | ec:4.4.1.8 |
| cpd:C00177 |  | Cyanide; Prussiate; CN-; Cyano | 1 | ec:2.8.1.1 ec:2.8.1.2 |
| cpd:C03406 |  | N-(L-Arginino)succinate; 2-(Nomega-L-Arginino)succinate; L-Argininosuccinate; L-Argininosuccinic acid; L-Arginosuccinic acid | 1 | ec:6.3.4.5 |
| cpd:C00173 |  | Acyl-[acyl-carrier protein] | 1 | ec:2.3.1.40 ec:6.2.1.20 |
| cpd:C00170 |  | 5'-Methylthioadenosine; Methylthioadenosine; S-Methyl-5'-thioadenosine; 5-Methylthioadenosine; 5'-Deoxy-5'-(methylthio)adenosine; Thiomethyladenosine; MTA | 1 | ec:2.4.2.28 |
| cpd:C01644 |  | tRNA(Ile) | 1 | ec:6.1.1.5 |
| cpd:C00208 |  | Maltose; Malt sugar; alpha-D-Glucopyranosyl-(1->4)-D-glucopyranose | 1 | ec:2.4.1.25 |
| cpd:C00163 |  | Propanoate; Propionate; Propanoic acid; Propionic acid | 1 | ec:2.8.3.1 |
| cpd:C00957 |  | Mercaptopyruvate; 3-Mercaptopyruvic acid; 3-Mercaptopyruvate | 1 | ec:2.8.1.1 ec:2.8.1.2 |
| cpd:C01635 |  | tRNA(Ala) | 1 | ec:6.1.1.7 |
| cpd:C19673 |  | Malonyl-[acp] methyl ester; Malonyl-[acyl-carrier protein] methyl ester | 1 | ec:2.1.1.197 |
| cpd:C00143 |  | 5,10-Methylenetetrahydrofolate; (6R)-5,10-Methylenetetrahydrofolate; 5,10-Methylene-THF | 1 | ec:2.1.1.45 |
| cpd:C04377 |  | 5,10-Methylenetetrahydromethanopterin; N5,N10-Methylenetetrahydromethanopterin | 1 |  |
| cpd:C01217 |  | 5,6,7,8-Tetrahydromethanopterin; H4MPT; THMPT; Tetrahydromethanopterin | 1 | ec:2.3.1.101 |
| cpd:C00134 |  | Putrescine; 1,4-Butanediamine; 1,4-Diaminobutane; Tetramethylenediamine; Butane-1,4-diamine | 1 | ec:2.6.1.82 |
| cpd:C00491 |  | L-Cystine; L-Dicysteine; L-alpha-Diamino-beta-dithiolactic acid | 1 | ec:4.4.1.8 |
| cpd:C00095 |  | D-Fructose; Levulose; Fruit sugar; D-arabino-Hexulose | 1 | ec:1.1.1.14 |
| cpd:C00093 |  | sn-Glycerol 3-phosphate; Glycerophosphoric acid; D-Glycerol 1-phosphate | 1 | ec:1.1.1.94 |
| cpd:C01962 |  | Thiocysteine; S-Mercapto-L-cysteine | 1 | ec:4.4.1.8 |
| cpd:C01209 |  | Malonyl-[acyl-carrier protein]; Malonyl-[acp] | 1 | ec:2.1.1.197 |
| cpd:C00886 |  | L-Alanyl-tRNA; L-Alanyl-tRNA(Ala) | 1 | ec:6.1.1.7 |
| cpd:C15815 |  | C15815; Thiamine biosynthesis intermediate 6 | 1 |  |
| cpd:C04752 |  | 2-Methyl-4-amino-5-hydroxymethylpyrimidine diphosphate; 4-Amino-2-methyl-5-diphosphomethylpyrimidine | 1 | ec:2.5.1.3 |
| cpd:C18174 |  | Carboxynorspermidine | 1 |  |
| cpd:C18172 |  | Carboxyspermidine; 2-Amino-4-[(4-aminobutyl)amino]-butanoic acid | 1 |  |
| cpd:C04352 |  | (R)-4'-Phosphopantothenoyl-L-cysteine; N-[(R)-4'-Phosphopantothenoyl]-L-cysteine | 1 | ec:6.3.2.5 ec:4.1.1.36 |
| cpd:C00082 |  | L-Tyrosine; (S)-3-(p-Hydroxyphenyl)alanine; (S)-2-Amino-3-(p-hydroxyphenyl)propionic acid; Tyrosine | 1 |  |
| cpd:C06507 |  | Adenosyl cobyrinate hexaamide; Adenosylcobyric acid | 1 | ec:6.3.1.10 |
| cpd:C12215 |  | Iminoerythrose 4-phosphate; Imino-D-erythrose 4-phosphate | 1 | ec:2.2.1.1 |
| cpd:C12214 |  | Aminofructose 6-phosphate; Amino-D-fructose 6-phosphate | 1 | ec:2.2.1.1 |
| cpd:C02191 |  | Protoporphyrin; Protoporphyrin IX; Porphyrinogen IX | 1 | ec:1.3.5.3 |
| cpd:C00078 |  | L-Tryptophan; Tryptophan; (S)-alpha-Amino-beta-(3-indolyl)-propionic acid | 1 | ec:6.1.1.2 |
| cpd:C05382 |  | Sedoheptulose 7-phosphate; D-Sedoheptulose 7-phosphate; D-altro-Heptulose 7-phosphate; altro-Heptulose 7-phosphate | 1 | ec:2.2.1.1 |
| cpd:C05819 |  | Menaquinol; Reduced menaquinone; Vitamin K2 hydroquinone; Reduced vitamin K2 | 1 | ec:1.3.5.3 |
| cpd:C03263 |  | Coproporphyrinogen III | 1 | ec:4.1.1.37 |
| cpd:C00506 |  | L-Cysteate; L-Cysteic acid; 3-Sulfoalanine; 2-Amino-3-sulfopropionic acid | 1 | ec:4.4.1.25 |
| cpd:C00504 |  | Folate; Pteroylglutamic acid; Folic acid | 1 | ec:1.5.1.3 |
| cpd:C05378 |  | beta-D-Fructose 1,6-bisphosphate | 1 | ec:3.1.3.11 |
| cpd:C06056 |  | 4-Hydroxy-L-threonine; Hydroxythreonine | 1 | ec:4.2.3.1 |
| cpd:C06055 |  | O-Phospho-4-hydroxy-L-threonine; 4-(Phosphonooxy)-threonine; 4-(Phosphonooxy)-L-threonine | 1 | ec:4.2.3.1 |
| cpd:C00460 |  | dUTP; 2'-Deoxyuridine 5'-triphosphate | 1 | ec:3.6.1.23 |
| cpd:C00065 |  | L-Serine; L-2-Amino-3-hydroxypropionic acid; L-3-Hydroxy-alanine; Serine | 1 | ec:2.3.1.30 |
| cpd:C00064 |  | L-Glutamine; L-2-Aminoglutaramic acid | 1 | ec:6.3.5.1 |
| cpd:C00063 |  | CTP; Cytidine 5'-triphosphate; Cytidine triphosphate | 1 | ec:6.3.2.5 ec:4.1.1.36 |
| cpd:C11481 |  | HSO3-; Hydrogen sulfite; Bisulfite | 1 | ec:4.4.1.25 |
| cpd:C00062 |  | L-Arginine; (S)-2-Amino-5-guanidinovaleric acid; L-Arg | 1 | ec:2.3.1.109 |
| cpd:C04330 |  | 5,10-Methenyltetrahydromethanopterin; 5,10-Methenyl-5,6,7,8-tetrahydromethanopterin; N5,N10-Methenyltetrahydromethanopterin | 1 |  |
| cpd:C05768 |  | Coproporphyrinogen I | 1 | ec:4.1.1.37 |
| cpd:C05766 |  | Uroporphyrinogen I | 1 | ec:4.1.1.37 |
| cpd:C00455 |  | Nicotinamide D-ribonucleotide; NMN; Nicotinamide mononucleotide; Nicotinamide ribonucleotide; Nicotinamide nucleotide; beta-Nicotinamide D-ribonucleotide; beta-Nicotinamide ribonucleotide; beta-Nicotinamide mononucleotide | 1 | ec:2.7.7.1 |
| cpd:C01134 |  | Pantetheine 4'-phosphate; 4'-Phosphopantetheine; Phosphopantetheine; D-Pantetheine 4'-phosphate | 1 | ec:6.3.2.5 ec:4.1.1.36 |
| cpd:C00055 |  | CMP; Cytidine-5'-monophosphate; Cytidylic acid | 1 | ec:6.3.2.5 ec:4.1.1.36 |
| cpd:C05752 |  | Octanoyl-[acp]; Octanoyl-[acyl-carrier protein] | 1 | ec:2.8.1.8 |
| cpd:C00440 |  | 5-Methyltetrahydrofolate | 1 | ec:2.1.1.13 |
| cpd:C01081 |  | Thiamin monophosphate; Thiamine monophosphate; Thiamin phosphate; Thiamine phosphate; TMP | 1 | ec:2.5.1.3 |
| cpd:C03194 |  | (R)-1-Aminopropan-2-ol; (R)-1-Amino-2-propanol | 1 | ec:6.3.1.10 |
| cpd:C05745 |  | Butyryl-[acp]; Butyryl-[acyl-carrier protein]; Butanoyl-[acp] | 1 | ec:1.3.1.44 |
| cpd:C00437 |  | N-Acetylornithine; N2-Acetyl-L-ornithine | 1 | ec:2.6.1.11 |
| cpd:C00794 |  | D-Sorbitol; D-Glucitol; L-Gulitol; Sorbitol | 1 | ec:1.1.1.14 |
| cpd:C01079 |  | Protoporphyrinogen IX | 1 | ec:1.3.5.3 |
| cpd:C00399 |  | Ubiquinone; Coenzyme Q; CoQ; Q | 1 | ec:1.6.5.3 |
| cpd:C00031 |  | D-Glucose; Grape sugar; Dextrose; Glucose; D-Glucopyranose | 1 | ec:2.4.1.25 |
| cpd:C00390 |  | Ubiquinol; QH2; CoQH2 | 1 | ec:1.6.5.3 |
| cpd:C00828 |  | Menaquinone; Vitamin K2 | 1 | ec:1.3.5.3 |
| cpd:C00827 |  | Lactoyl-CoA | 1 | ec:2.8.3.1 |
| cpd:C06019 |  | D-arabino-Hex-3-ulose 6-phosphate; D-arabino-3-Hexulose 6-phosphate; D-arabino-6-Phospho-hex-3-ulose | 1 | ec:5.3.1.27 |
| cpd:C05699 |  | L-Selenocystathionine | 1 | ec:4.4.1.8 |
| cpd:C01102 |  | O-Phospho-L-homoserine | 1 | ec:4.2.3.1 |
| cpd:C05691 |  | Se-Adenosylselenomethionine | 1 | ec:2.5.1.6 |
| cpd:C00021 |  | S-Adenosyl-L-homocysteine; S-Adenosylhomocysteine | 1 | ec:2.1.1.197 |
| cpd:C05726 |  | S-Substituted L-cysteine; R-S-Cysteine | 1 | ec:3.4.11.1 |
| cpd:C01051 |  | Uroporphyrinogen III | 1 | ec:4.1.1.37 |
| cpd:C17023 |  | Sulfur donor; S-donor | 1 | ec:2.8.1.8 |
| cpd:C04246 |  | But-2-enoyl-[acyl-carrier protein] | 1 | ec:1.3.1.44 |
| cpd:C06755 |  | Chloroacetic acid; Chloroethanoic acid | 1 | ec:3.8.1.2 |
| cpd:C00407 |  | L-Isoleucine; 2-Amino-3-methylvaleric acid | 1 | ec:6.1.1.5 |
| cpd:C20683 |  | Long-chain acyl-[acyl-carrier protein] | 1 | ec:2.3.1.40 ec:6.2.1.20 |
| cpd:C00354 |  | D-Fructose 1,6-bisphosphate | 1 | ec:3.1.3.11 |
| cpd:C00350 |  | Phosphatidylethanolamine; (3-Phosphatidyl)ethanolamine; (3-Phosphatidyl)-ethanolamine; Cephalin; O-(1-beta-Acyl-2-acyl-sn-glycero-3-phospho)ethanolamine; 1-Acyl-2-acyl-sn-glycero-3-phosphoethanolamine; L-1-Phosphatidylethanolamine | 1 | ec:2.3.1.40 ec:6.2.1.20 |
| cpd:C02504 |  | alpha-Isopropylmalate; (2S)-2-Isopropylmalate; (2S)-2-Hydroxy-2-isopropylsuccinic acid; 2-Isopropylmalic acid; 3-Carboxy-3-hydroxy-4-methylpentanoate; 3-Carboxy-3-hydroxyisocaproate | 1 | ec:2.3.3.13 |
| cpd:C04188 |  | S-Methyl-5-thio-D-ribose 1-phosphate; S-Methyl-5-thio-alpha-D-ribose 1-phosphate; S-Methyl-5-thio-5-deoxy-D-ribose 1-phosphate | 1 | ec:2.4.2.28 |

  
**Over-represented Pathway Summary**: Collection of the KEGG metabolic pathways containing the proteins identified in the "Over-represented Metabolite Summary" ranked by the highest number of hits per pathway  

| Pathway ID | EC | EC Frequency | Name |
| --- | --- | --- | --- |
| map00920 | ec:2.8.1.1 ec:2.7.7.4 ec:1.8.4.8 ec:3.6.3.25 ec:2.3.1.30 ec:1.14.14.5 ec:1.8.1.2 ec:2.5.1.47 | 90 | path:map00920 Sulfur metabolism |
| map00860 | ec:4.1.1.37 ec:6.3.1.10 ec:2.5.1.17 ec:2.7.1.156 ec:1.3.5.3 ec:2.7.8.26 ec:2.7.7.62 ec:2.4.2.21 | 84 | path:map00860 Porphyrin and chlorophyll metabolism |
| map00230 | ec:2.7.7.4 ec:5.1.99.3 ec:3.5.1.5 ec:3.2.2.4 ec:2.7.6.1 ec:2.7.7.7 | 35 | path:map00230 Purine metabolism |
| map00630 | ec:4.1.3.1 ec:3.1.3.18 ec:5.3.1.22 ec:4.1.1.47 ec:1.2.1.2 ec:1.1.1.60 | 27 | path:map00630 Glyoxylate and dicarboxylate metabolism |
| map00030 | ec:5.1.3.1 ec:2.7.1.15 ec:2.7.6.1 ec:2.2.1.1 ec:3.1.3.11 ec:5.3.1.27 | 22 | path:map00030 Pentose phosphate pathway |
| map00790 | ec:1.5.1.3 ec:4.3.99.3 ec:4.1.3.38 ec:6.3.4.20 | 22 | path:map00790 Folate biosynthesis |
| map00680 | ec:1.2.99.5 ec:5.4.2.12 ec:4.4.1.22 ec:3.1.3.11 ec:1.1.1.284 ec:1.2.1.2 ec:3.1.2.12 ec:5.3.1.27 ec:2.3.1.101 | 15 | path:map00680 Methane metabolism |
| map00250 | ec:1.4.3.16 ec:3.5.1.1 ec:6.3.4.5 | 14 | path:map00250 Alanine, aspartate and glutamate metabolism |
| map00760 | ec:1.4.3.16 ec:2.7.7.1 ec:6.3.4.21 ec:1.6.1.2 ec:6.3.5.1 | 14 | path:map00760 Nicotinate and nicotinamide metabolism |
| map00330 | ec:2.6.1.82 ec:3.5.1.96 ec:2.6.1.81 ec:3.5.3.23 ec:3.5.1.5 ec:2.3.1.109 ec:1.2.1.19 ec:2.6.1.11 ec:6.3.4.5 ec:1.2.1.71 ec:1.2.1.38 | 13 | path:map00330 Arginine and proline metabolism |
| map00450 | ec:2.7.7.4 ec:2.1.1.13 ec:2.7.9.3 ec:4.4.1.16 ec:4.4.1.8 | 12 | path:map00450 Selenocompound metabolism |
| map00480 | ec:3.4.11.1 ec:1.11.1.15 ec:2.3.2.2 | 12 | path:map00480 Glutathione metabolism |
| map00240 | ec:2.1.1.45 ec:2.7.4.9 ec:2.7.7.7 ec:3.6.1.23 | 10 | path:map00240 Pyrimidine metabolism |
| map00770 | ec:4.1.1.36 ec:2.2.1.6 ec:6.3.2.5 | 9 | path:map00770 Pantothenate and CoA biosynthesis |
| map00460 | ec:3.5.1.1 ec:2.3.2.2 | 9 | path:map00460 Cyanoamino acid metabolism |
| map00290 | ec:2.6.1.66 ec:2.2.1.6 ec:2.3.3.13 | 9 | path:map00290 Valine, leucine and isoleucine biosynthesis |
| map00270 | ec:2.1.1.13 ec:2.4.2.28 ec:2.3.1.30 ec:4.4.1.8 ec:4.4.1.25 ec:2.8.1.2 ec:2.5.1.47 ec:2.5.1.6 | 8 | path:map00270 Cysteine and methionine metabolism |
| map00650 | ec:2.2.1.6 ec:1.3.1.44 | 8 | path:map00650 Butanoate metabolism |
| map00660 | ec:2.2.1.6 | 7 | path:map00660 C5-Branched dibasic acid metabolism |
| map00300 | ec:2.3.1.117 | 7 | path:map00300 Lysine biosynthesis |
| map00740 | ec:2.7.7.2 ec:2.7.1.26 ec:1.5.1.38 | 5 | path:map00740 Riboflavin metabolism |
| map00190 | ec:1.6.5.3 ec:1.9.3.1 | 5 | path:map00190 Oxidative phosphorylation |
| map00620 | ec:3.1.2.1 ec:2.8.3.1 ec:2.3.3.13 ec:1.1.2.4 | 5 | path:map00620 Pyruvate metabolism |
| map00970 | ec:6.1.1.14 ec:6.1.1.2 ec:6.1.1.7 ec:6.1.1.5 | 5 | path:map00970 Aminoacyl-tRNA biosynthesis |
| map00010 | ec:1.1.1.1 ec:5.3.1.1 ec:5.4.2.12 ec:3.1.3.11 | 5 | path:map00010 Glycolysis / Gluconeogenesis |
| map00071 | ec:1.3.8.7 ec:1.1.1.1 ec:6.2.1.20 | 5 | path:map00071 Fatty acid degradation |
| map00710 | ec:5.3.1.1 ec:5.1.3.1 ec:2.2.1.1 ec:3.1.3.11 | 4 | path:map00710 Carbon fixation in photosynthetic organisms |
| map00633 | ec:1.12.99.6 | 4 | path:map00633 Nitrotoluene degradation |
| map00590 | ec:2.3.2.2 | 4 | path:map00590 Arachidonic acid metabolism |
| map00430 | ec:2.3.2.2 | 4 | path:map00430 Taurine and hypotaurine metabolism |
| map00260 | ec:1.1.1.1 ec:4.2.3.1 ec:5.4.2.12 | 4 | path:map00260 Glycine, serine and threonine metabolism |
| map00640 | ec:1.3.8.7 ec:2.8.3.1 | 3 | path:map00640 Propanoate metabolism |
| map00670 | ec:1.5.1.3 ec:2.1.1.13 ec:2.1.1.45 | 3 | path:map00670 One carbon pool by folate |
| map00730 | ec:2.5.1.3 ec:2.8.1.7 ec:1.4.3.19 | 3 | path:map00730 Thiamine metabolism |
| map00410 | ec:1.3.8.7 ec:1.2.1.19 | 3 | path:map00410 beta-Alanine metabolism |
| map00791 | ec:3.5.1.5 | 3 | path:map00791 Atrazine degradation |
| map00051 | ec:5.3.1.1 ec:1.1.1.14 ec:3.1.3.11 | 3 | path:map00051 Fructose and mannose metabolism |
| map00625 | ec:1.1.1.1 ec:3.8.1.2 | 3 | path:map00625 Chloroalkane and chloroalkene degradation |
| map00350 | ec:1.1.1.1 | 2 | path:map00350 Tyrosine metabolism |
| map00830 | ec:1.1.1.1 | 2 | path:map00830 Retinol metabolism |
| map00280 | ec:1.3.8.7 | 2 | path:map00280 Valine, leucine and isoleucine degradation |
| map00982 | ec:1.1.1.1 | 2 | path:map00982 Drug metabolism - cytochrome P450 |
| map00980 | ec:1.1.1.1 | 2 | path:map00980 Metabolism of xenobiotics by cytochrome P450 |
| map00564 | ec:1.1.1.94 ec:2.3.1.40 | 2 | path:map00564 Glycerophospholipid metabolism |
| map00561 | ec:1.1.1.6 | 2 | path:map00561 Glycerolipid metabolism |
| map00626 | ec:1.1.1.1 | 2 | path:map00626 Naphthalene degradation |
| map00643 | ec:2.8.3.1 | 1 | path:map00643 Styrene degradation |
| map00500 | ec:2.4.1.25 | 1 | path:map00500 Starch and sucrose metabolism |
| map00562 | ec:5.3.1.1 | 1 | path:map00562 Inositol phosphate metabolism |
| map00785 | ec:2.8.1.8 | 1 | path:map00785 Lipoic acid metabolism |
| map00750 | ec:4.2.3.1 | 1 | path:map00750 Vitamin B6 metabolism |
| map00780 | ec:2.1.1.197 | 1 | path:map00780 Biotin metabolism |
| map00361 | ec:3.8.1.2 | 1 | path:map00361 Chlorocyclohexane and chlorobenzene degradation |
| map00040 | ec:5.1.3.1 | 1 | path:map00040 Pentose and glucuronate interconversions |

  
Analysis performed on 2014/02/14 22:11:46
