# Supplementary material for: Automated genomic context analysis and experimental validation platform for discovery of prokaryote transcriptional regulator functions
Source: BMC Genomics. 2014 Dec 18;15(1):1142. doi: 10.1186/1471-2164-15-1142 (PMC4349456; doi:10.1186/1471-2164-15-1142)
Supplement: Supplementary file 6 — Additional file 6: Result GlpR. Function Discovery V1.0 output (.html format) for the glycerol metabolism regulator (GlpR, Bxe_ A0643). For detailed instructions on how to analyze the results please refer to the Function Discovery V1.0, a gene neighborhood analysis tool section in the Results part of the main text. (HTML 684 KB) [file 12864_2014_6995_MOESM6_ESM.html]

```
ENTRY       Bxe_A0643         CDS       T00340
DEFINITION  DeoR family transcriptional regulator
ORTHOLOGY   K02444  DeoR family transcriptional regulator, glycerol-3-phosphate regulon repressor
ORGANISM    bxe  Burkholderia xenovorans
POSITION    1:4172637..4173416
MOTIF       Pfam: DeoRC HTH_DeoR HTH_11 MarR_2 HTH_38 HTH_Crp_2 MarR HTH_24 GntR HTH_28 HTH_20 Sigma70_r4 HTH_AsnC-type LEDGF
DBLINKS     NCBI-GI: 91785137
            NCBI-GeneID: 4005768
            JGI: BxeA0643
            UniProt: Q13UE8
AASEQ       259
            MTRDPRLTLNARQQELLEWVQRDGFVTVDDLAAHFDVTPQTIRRDVNWLADMNLLRRYHG
            GASLPTSSENVSYTARQRMFHEEKRRIAALVATHIPDQASLFINLGTTTEEVARALNRHR
            GLRVITNNLNVASMMSGYPDCEVLVTGGIVRPWDKGIVGELAIDFIRQFKVDFAIIGTSS
            IETDGTLRDFDTREVRVAEAIIQHARTVFLAADNSKFGRPALVRQGHLDQIDALFTDMAP
            PAAMTETLTAANCQVYVAE
NTSEQ       780
            atgacccgagacccccgcctgactctcaatgcccggcaacaggaactgctggagtgggtg
            caacgcgacggcttcgtgaccgtggacgacctcgccgcccacttcgacgtgacgccgcag
            acgatccgccgcgacgtcaactggctcgccgacatgaatctgctgcgccgttatcacggc
            ggcgccagtcttccgaccagttccgaaaacgtctcctacaccgcgcgtcagcgcatgttc
            cacgaagaaaagcggcgcattgcggcgctagtggccactcacattcccgatcaggcctcg
            ctgttcatcaacctcggcaccaccacggaggaagtggctcgcgcgctcaatcgccaccgc
            gggctgcgcgtgatcaccaacaatctgaacgtcgccagcatgatgagcggctatccggat
            tgcgaggtgctggtgacgggcggcatcgtgcggccgtgggacaaagggatcgtcggcgaa
            ctggcgatcgatttcatccgccagttcaaggtggactttgcgatcatcggcacgtcgagc
            atcgaaacggacggcacgctgcgcgatttcgacacgcgcgaagtgcgcgtggccgaagcg
            atcatccagcacgcgcgcacggttttcctcgccgcggacaactcgaaattcggccgcccc
            gcgctggtccgccaaggtcatctcgatcagatcgacgctctgttcacggacatggcgccg
            cccgccgcgatgaccgaaacgctcactgccgccaactgccaggtgtatgtcgccgaatga
///
```

  
**Homolog ID**: Table of closest homologs  

```
                 Homologs                                       len   identity overlap
---------------------------------------------------------------------------------
bpy:Bphyt_3316 DeoR family transcriptional regulator    K     259     0.977    259 
bgf:BC1003_2900 DeoR family transcriptional regulator   K     259     0.969    258 
bge:BC1002_2585 DeoR family transcriptional regulator   K     259     0.953    258 
bph:Bphy_0423 DeoR family transcriptional regulator     K     259     0.930    258 
bgl:bglu_1g30380 glycerol-3-phosphate regulon repressor K     259     0.876    259 
bte:BTH_I0603 glycerol-3-phosphate regulon repressor    K     278     0.892    259 
bma:BMA0244 glycerol-3-phosphate regulon repressor      K     259     0.896    259 
bml:BMA10229_A2375 glycerol-3-phosphate regulon repress K     259     0.896    259 
bmn:BMA10247_2456 glycerol-3-phosphate regulon represso K     259     0.896    259 
bmv:BMASAVP1_A2702 glycerol-3-phosphate regulon repress K     278     0.896    259 
bpd:BURPS668_0729 glycerol-3-phosphate regulon represso K     259     0.896    259 
bpl:BURPS1106A_0743 glycerol-3-phosphate regulon repres K     259     0.896    259 
bpm:BURPS1710b_0910 glycerol-3-phosphate regulon repres K     278     0.896    259 
bpr:GBP346_A0659 glycerol-3-phosphate regulon repressor K     310     0.896    259 
bps:BPSL0691 DeoR family glycerol-3-phosphate regulon r K     278     0.896    259 
bmj:BMULJ_02637 glycerol-3-phosphate regulon repressor  K     259     0.888    259 
bmu:Bmul_0623 DeoR family transcriptional regulator     K     294     0.888    259 
bvi:Bcep1808_2786 DeoR family transcriptional regulator K     259     0.884    259 
bch:Bcen2424_2674 DeoR family transcriptional regulator K     294     0.880    259 
bcj:BCAL0929 putative DeoR family glycerol-3-phosphate  K     259     0.880    259 
bur:Bcep18194_A6002 DeoR family transcriptional regulat K     259     0.880    259 
bcn:Bcen_2063 DeoR family transcriptional regulator     K     294     0.876    259 
bac:BamMC406_2599 DeoR family transcriptional regulator K     259     0.876    259 
bam:Bamb_2727 DeoR family transcriptional regulator     K     290     0.876    259 
bcm:Bcenmc03_2703 DeoR family transcriptional regulator K     259     0.876    259 
brh:RBRH_01996 glycerol-3-phosphate regulon repressor   K     288     0.814    258 
cvi:CV_0136 glycerol-3-phosphate regulon repressor      K     254     0.648    253 
xcv:XCV0375 DeoR family transcriptional regulator       K     320     0.584    255 
xac:XAC0361 glycerol-3-phosphate regulon repressor      K     256     0.582    251 
xca:xccb100_0387 glycerol-3-phosphate regulon repressor K     273     0.575    254 
xcb:XC_0373 glycerol-3-phosphate regulon repressor      K     259     0.571    254 
xcc:XCC0361 glycerol-3-phosphate regulon repressor      K     259     0.571    254 
bid:Bind_0377 DeoR family transcriptional regulator     K     261     0.578    258 
bpt:Bpet2588 hypothetical protein                       K     268     0.555    256 
dsa:Desal_3154 DeoR family transcriptional regulator    K     275     0.552    252 
vei:Veis_3832 DeoR family transcriptional regulator     K     277     0.552    252 
psa:PST_1602 glycerol-3-phosphate regulon repressor     K     252     0.565    253 
hse:Hsero_0965 glycerol-3-phosphate regulon repressor t K     268     0.562    251 
bpa:BPP3096 glycerol-3-phosphate regulon repressor prot K     269     0.559    256 
dba:Dbac_1439 DeoR family transcriptional regulator     K     275     0.542    253 
bbr:BB3059 glycerol-3-phosphate regulon repressor prote K     269     0.559    256 
pol:Bpro_0477 DeoR family transcriptional regulator     K     259     0.562    256 
rso:RSc3044 glycerol-3-phosphate regulon repressor tran K     261     0.577    253 
bpe:BP2643 glycerol-3-phosphate regulon repressor prote K     262     0.561    253 
vap:Vapar_3398 DeoR family transcriptional regulator    K     255     0.576    250 
azl:AZL_e03100 transcriptional regulator                K     329     0.523    256 
rsc:RCFBP_10402 glycerol-3-phosphate regulon repressor, K     254     0.571    247 
lch:Lcho_3222 DeoR family transcriptional regulator     K     257     0.552    250 
rpf:Rpic12D_2979 DeoR family transcriptional regulator  K     257     0.576    250 
rsl:RPSI07_0452 glycerol-3-phosphate regulon repressor, K     254     0.567    247 
axy:AXYL_03486 glycerol-3-phosphate regulon repressor   K     263     0.549    255 
rpi:Rpic_3326 DeoR family transcriptional regulator     K     257     0.576    250 
hch:HCH_06963 sugar metabolism transcriptional regulato K     258     0.542    249 
ppf:Pput_1115 DeoR family transcriptional regulator     K     286     0.541    255 
cti:RALTA_A2007 glycerol-3-phosphate regulon repressor, K     256     0.549    255 
ppu:PP_1074 DeoR family transcriptional regulator       K     251     0.550    251 
reu:Reut_A2210 DeoR family transcriptional regulator    K     259     0.543    254 
pen:PSEEN1196 glycerol-3-phosphate regulon repressor    K     251     0.566    242 
ppg:PputGB1_4338 DeoR family transcriptional regulator  K     251     0.566    242 
aav:Aave_0603 DeoR family transcriptional regulator     K     254     0.559    254 
psb:Psyr_3906 regulatory protein, DeoR                  K     251     0.568    243 
psp:PSPPH_3900 glycerol-3-phosphate regulon repressor   K     251     0.568    243 
reh:H16_A2504 DeoR family transcriptional regulator     K     256     0.535    254 
dac:Daci_1024 DeoR family transcriptional regulator     K     254     0.555    254 
asa:ASA_2707 glycerol-3-phosphate regulon repressor     K     252     0.541    246 
gpb:HDN1F_32230 transcriptional Regulator, DeoR family  K     268     0.502    259 
aha:AHA_1651 glycerol-3-phosphate regulon repressor     K     252     0.537    246 
bav:BAV2010 glycerol-3-phosphate regulon repressor      K     253     0.545    253 
pae:PA3583 glycerol-3-phosphate regulon repressor       K     251     0.550    242 
pag:PLES_14511 glycerol-3-phosphate regulon repressor   K     251     0.550    242 
pap:PSPA7_1560 glycerol-3-phosphate regulon repressor   K     251     0.550    242 
pau:PA14_17940 glycerol-3-phosphate regulon repressor   K     251     0.550    242 
pfs:PFLU1141 glycerol-3-phosphate regulon repressor     K     251     0.538    251 
avn:Avin_45720 glycerol-3-phosphate regulon repressor p K     251     0.534    251 
ppw:PputW619_1103 DeoR family transcriptional regulator K     251     0.562    242 
rme:Rmet_2235 DNA-binding transcriptional repressor     K     259     0.531    256 
dia:Dtpsy_0374 DeoR family transcriptional regulator    K     262     0.529    255 
pfl:PFL_4869 glycerol-3-phosphate regulon repressor     K     251     0.562    242 
pfo:Pfl01_4533 DeoR family transcriptional regulator    K     251     0.551    243 
pst:PSPTO_4169 glycerol-3-phosphate regulon repressor   K     251     0.562    242 
rfr:Rfer_3677 DeoR family transcriptional regulator     K     254     0.554    249 
pmy:Pmen_3347 DeoR family transcriptional regulator     K     252     0.548    250 
ajs:Ajs_0383 DeoR family transcriptional regulator      K     254     0.548    252 
dat:HRM2_42290 protein GlpR2                            K     249     0.536    248 
vvu:VV1_1786 glycerol-3-phosphate regulon repressor     K     255     0.516    248 
vvy:VV2625 sugar metabolism transcriptional regulator   K     264     0.516    248 
ppr:PBPRA0159 glycerol-3-phosphate repressor protein    K     259     0.546    238 
ctt:CtCNB1_4445 DeoR family transcriptional regulator   K     254     0.542    251 
hel:HELO_3002 DNA-binding transcriptional repressor     K     252     0.530    247 
mmw:Mmwyl1_3954 DeoR family transcriptional regulator   K     256     0.548    239 
vex:VEA_002674 glycerol-3-phosphate regulon repressor D K     263     0.510    249 
csa:Csal_2105 DeoR family transcriptional regulator     K     256     0.514    253 
vha:VIBHAR_03314 transcriptional regulator              K     263     0.506    249 
vpa:VP2387 DeoR family transcriptional regulator        K     263     0.506    249 
vcm:VCM66_A0900 transcriptional regulator, DeoR family  K     253     0.541    231 
vco:VC0395_0299 DeoR family transcriptional regulator   K     253     0.541    231 
vch:VCA0940 DeoR family transcriptional regulator       K     253     0.541    231 
vcj:VCD_000394 glycerol-3-phosphate regulon repressor D K     253     0.541    231 
vsp:VS_II0114 Transcriptional regulator, DeoR family    K     261     0.508    238 
xbo:XBJ1_0140 DeoR family transcriptional regulator     K     252     0.523    237
```

**Neighborhood Representations**: Table of genes in the defined genetic neighborhoods of the entry protein and its closest homologs  
  
**Neighborhood Representations for "bxe:Bxe\_A0643"**  

| ID | Annotation | EC number |
| --- | --- | --- |
| bxe:Bxe\_A0653 | copper-resistance transporter, CopD; K07245 putative copper resistance protein D |  |
| bxe:Bxe\_A0652 | copper resistance protein, CopC; K07156 |  |
| bxe:Bxe\_A0651 | periplasmic cytochrome c |  |
| bxe:Bxe\_A0650 | periplasmic cytochrome c |  |
| bxe:Bxe\_A0649 | hemolysin HylII; K11068 hemolysin III |  |
| bxe:Bxe\_A0648 | ATP-dependent RNA helicase 1; K11927 ATP-dependent RNA helicase RhlE [EC:3.6.4.13] | ec:3.6.4.13 |
| bxe:Bxe\_A0647 | hypothetical protein |  |
| bxe:Bxe\_A0646 | hypothetical protein |  |
| bxe:Bxe\_A0645 | major facilitator superfamily metabolite/H(+) symporter |  |
| bxe:Bxe\_A0644 | gamma-glutamyltransferase 2 (EC:2.3.2.2); K00681 gamma-glutamyltranspeptidase [EC:2.3.2.2] | ec:2.3.2.2 |
| bxe:Bxe\_A0643 | DeoR family transcriptional regulator; K02444 DeoR family transcriptional regulator, glycerol-3-phosphate regulon repressor |  |
| bxe:Bxe\_A0642 | hypothetical protein |  |
| bxe:Bxe\_A0641 | TetR family transcriptional regulator |  |
| bxe:Bxe\_A0640 | hypothetical protein |  |
| bxe:Bxe\_A0639 | glpD; glycerol-3-phosphate dehydrogenase (EC:1.1.5.3); K00111 glycerol-3-phosphate dehydrogenase [EC:1.1.5.3] | ec:1.1.5.3 |
| bxe:Bxe\_A0638 | glpK; glycerol kinase (EC:2.7.1.30); K00864 glycerol kinase [EC:2.7.1.30] | ec:2.7.1.30 |
| bxe:Bxe\_A0637 | major intrinsic protein, glycerol uptake channel; K02440 glycerol uptake facilitator protein |  |
| bxe:Bxe\_A0636 | HAD family hydrolase |  |
| bxe:Bxe\_A0635 | 3,4-dihydroxy-2-butanone 4-phosphate synthase; K02858 3,4-dihydroxy 2-butanone 4-phosphate synthase [EC:4.1.99.12] | ec:4.1.99.12 |
| bxe:Bxe\_A0634 | XRE family transcriptional regulator |  |
| bxe:Bxe\_A0633 | major facilitator transporter; K08156 MFS transporter, DHA1 family, arabinose polymer transporter |  |

  
**Neighborhood Representations for "bpy:Bphyt\_3316"**  

| ID | Annotation | EC number |
| --- | --- | --- |
| bpy:Bphyt\_3306 | copper resistance protein D; K07245 putative copper resistance protein D |  |
| bpy:Bphyt\_3307 | copper resistance protein C; K07156 |  |
| bpy:Bphyt\_3308 | cytochrome C class I |  |
| bpy:Bphyt\_3309 | gluconate 2-dehydrogenase (EC:1.1.99.3) |  |
| bpy:Bphyt\_3310 | hemolysin III family channel protein; K11068 hemolysin III |  |
| bpy:Bphyt\_3311 | DEAD/DEAH box helicase; K11927 ATP-dependent RNA helicase RhlE [EC:3.6.4.13] | ec:3.6.4.13 |
| bpy:Bphyt\_3312 | hypothetical protein |  |
| bpy:Bphyt\_3313 | hypothetical protein |  |
| bpy:Bphyt\_3314 | major facilitator superfamily protein |  |
| bpy:Bphyt\_3315 | gamma-glutamyltransferase (EC:2.3.2.2); K00681 gamma-glutamyltranspeptidase [EC:2.3.2.2] | ec:2.3.2.2 |
| bpy:Bphyt\_3316 | DeoR family transcriptional regulator; K02444 DeoR family transcriptional regulator, glycerol-3-phosphate regulon repressor |  |
| bpy:Bphyt\_3317 | hypothetical protein |  |
| bpy:Bphyt\_3318 | TetR family transcriptional regulator |  |
| bpy:Bphyt\_3319 | hypothetical protein |  |
| bpy:Bphyt\_3320 | glpD; glycerol-3-phosphate dehydrogenase; K00111 glycerol-3-phosphate dehydrogenase [EC:1.1.5.3] | ec:1.1.5.3 |
| bpy:Bphyt\_3321 | glpK; glycerol kinase; K00864 glycerol kinase [EC:2.7.1.30] | ec:2.7.1.30 |
| bpy:Bphyt\_3322 | MIP family channel protein; K02440 glycerol uptake facilitator protein |  |
| bpy:Bphyt\_3323 | HAD family hydrolase |  |
| bpy:Bphyt\_3324 | 3,4-dihydroxy-2-butanone 4-phosphate synthase; K02858 3,4-dihydroxy 2-butanone 4-phosphate synthase [EC:4.1.99.12] | ec:4.1.99.12 |
| bpy:Bphyt\_3325 | XRE family transcriptional regulator |  |
| bpy:Bphyt\_3326 | major facilitator superfamily protein; K08156 MFS transporter, DHA1 family, arabinose polymer transporter |  |

  
**Neighborhood Representations for "bgf:BC1003\_2900"**  

| ID | Annotation | EC number |
| --- | --- | --- |
| bgf:BC1003\_2890 | hypothetical protein |  |
| bgf:BC1003\_2891 | hypothetical protein |  |
| bgf:BC1003\_2892 | copper resistance D domain-containing protein; K07245 putative copper resistance protein D |  |
| bgf:BC1003\_2893 | copper resistance protein CopC; K07156 |  |
| bgf:BC1003\_2894 | cytochrome c class I |  |
| bgf:BC1003\_2895 | gluconate 2-dehydrogenase (acceptor) (EC:1.1.99.3) |  |
| bgf:BC1003\_2896 | hemolysin III family channel protein; K11068 hemolysin III |  |
| bgf:BC1003\_2897 | DEAD/DEAH box helicase; K11927 ATP-dependent RNA helicase RhlE [EC:3.6.4.13] | ec:3.6.4.13 |
| bgf:BC1003\_2898 | hypothetical protein |  |
| bgf:BC1003\_2899 | hypothetical protein |  |
| bgf:BC1003\_2900 | DeoR family transcriptional regulator; K02444 DeoR family transcriptional regulator, glycerol-3-phosphate regulon repressor |  |
| bgf:BC1003\_2901 | hypothetical protein |  |
| bgf:BC1003\_2902 | hypothetical protein |  |
| bgf:BC1003\_2903 | glycerol-3-phosphate dehydrogenase; K00111 glycerol-3-phosphate dehydrogenase [EC:1.1.5.3] | ec:1.1.5.3 |
| bgf:BC1003\_2904 | glycerol kinase; K00864 glycerol kinase [EC:2.7.1.30] | ec:2.7.1.30 |
| bgf:BC1003\_2905 | major intrinsic protein; K02440 glycerol uptake facilitator protein |  |
| bgf:BC1003\_2906 | HAD-superfamily hydrolase |  |
| bgf:BC1003\_2907 | 3,4-dihydroxy-2-butanone 4-phosphate synthase; K02858 3,4-dihydroxy 2-butanone 4-phosphate synthase [EC:4.1.99.12] | ec:4.1.99.12 |
| bgf:BC1003\_2908 | helix-turn-helix domain-containing protein |  |
| bgf:BC1003\_2909 | major facilitator superfamily protein; K08156 MFS transporter, DHA1 family, arabinose polymer transporter |  |
| bgf:BC1003\_2910 | Glyoxalase/bleomycin resistance protein/dioxygenase |  |

  
**Neighborhood Representations for "bge:BC1002\_2585"**  

| ID | Annotation | EC number |
| --- | --- | --- |
| bge:BC1002\_2575 | copper resistance D domain-containing protein; K07245 putative copper resistance protein D |  |
| bge:BC1002\_2576 | copper resistance protein CopC; K07156 |  |
| bge:BC1002\_2577 | periplasmic cytochrome c |  |
| bge:BC1002\_2578 | gluconate 2-dehydrogenase (acceptor) (EC:1.1.99.3) |  |
| bge:BC1002\_2579 | hemolysin III family channel protein; K11068 hemolysin III |  |
| bge:BC1002\_2580 | DEAD/DEAH box helicase; K11927 ATP-dependent RNA helicase RhlE [EC:3.6.4.13] | ec:3.6.4.13 |
| bge:BC1002\_2581 | hypothetical protein |  |
| bge:BC1002\_2582 | hypothetical protein |  |
| bge:BC1002\_2583 | major facilitator superfamily protein |  |
| bge:BC1002\_2584 | gamma-glutamyltransferase (EC:2.3.2.2); K00681 gamma-glutamyltranspeptidase [EC:2.3.2.2] | ec:2.3.2.2 |
| bge:BC1002\_2585 | DeoR family transcriptional regulator; K02444 DeoR family transcriptional regulator, glycerol-3-phosphate regulon repressor |  |
| bge:BC1002\_2586 | hypothetical protein |  |
| bge:BC1002\_2587 | transcriptional regulator, TetR family |  |
| bge:BC1002\_2588 | hypothetical protein |  |
| bge:BC1002\_2589 | FAD dependent oxidoreductase; K00111 glycerol-3-phosphate dehydrogenase [EC:1.1.5.3] | ec:1.1.5.3 |
| bge:BC1002\_2590 | glycerol kinase; K00864 glycerol kinase [EC:2.7.1.30] | ec:2.7.1.30 |
| bge:BC1002\_2591 | HAD-superfamily hydrolase |  |
| bge:BC1002\_2592 | 3,4-dihydroxy-2-butanone 4-phosphate synthase; K02858 3,4-dihydroxy 2-butanone 4-phosphate synthase [EC:4.1.99.12] | ec:4.1.99.12 |
| bge:BC1002\_2593 | transcriptional regulator, XRE family |  |
| bge:BC1002\_2594 | major facilitator superfamily protein; K08156 MFS transporter, DHA1 family, arabinose polymer transporter |  |
| bge:BC1002\_2595 | hypothetical protein |  |

  
**Neighborhood Representations for "bph:Bphy\_0423"**  

| ID | Annotation | EC number |
| --- | --- | --- |
| bph:Bphy\_0413 | LysR family transcriptional regulator |  |
| bph:Bphy\_0414 | hypothetical protein |  |
| bph:Bphy\_0415 | major facilitator transporter; K08156 MFS transporter, DHA1 family, arabinose polymer transporter |  |
| bph:Bphy\_0416 | XRE family transcriptional regulator |  |
| bph:Bphy\_0417 | 3,4-dihydroxy-2-butanone 4-phosphate synthase; K02858 3,4-dihydroxy 2-butanone 4-phosphate synthase [EC:4.1.99.12] | ec:4.1.99.12 |
| bph:Bphy\_0418 | HAD family hydrolase |  |
| bph:Bphy\_0419 | glpK; glycerol kinase; K00864 glycerol kinase [EC:2.7.1.30] | ec:2.7.1.30 |
| bph:Bphy\_0420 | glpD; glycerol-3-phosphate dehydrogenase; K00111 glycerol-3-phosphate dehydrogenase [EC:1.1.5.3] | ec:1.1.5.3 |
| bph:Bphy\_0421 | hypothetical protein |  |
| bph:Bphy\_0422 | hypothetical protein |  |
| bph:Bphy\_0423 | DeoR family transcriptional regulator; K02444 DeoR family transcriptional regulator, glycerol-3-phosphate regulon repressor |  |
| bph:Bphy\_0424 | hypothetical protein |  |
| bph:Bphy\_0425 | hypothetical protein |  |
| bph:Bphy\_0426 | DEAD/DEAH box helicase domain-containing protein; K11927 ATP-dependent RNA helicase RhlE [EC:3.6.4.13] | ec:3.6.4.13 |
| bph:Bphy\_0427 | hemolysin III family channel protein; K11068 hemolysin III |  |
| bph:Bphy\_0428 | gluconate 2-dehydrogenase (EC:1.1.99.3) |  |
| bph:Bphy\_0429 | cytochrome c class I |  |
| bph:Bphy\_0430 | copper resistance protein CopC |  |
| bph:Bphy\_0431 | copper resistance D domain-containing protein; K07245 putative copper resistance protein D |  |
| bph:Bphy\_0432 | hypothetical protein |  |
| bph:Bphy\_0433 | galactonate dehydratase; K01684 galactonate dehydratase [EC:4.2.1.6] | ec:4.2.1.6 |

  
**Neighborhood Representations for "bgl:bglu\_1g30380"**  

| ID | Annotation | EC number |
| --- | --- | --- |
| bgl:bglu\_1g30280 | GntR family transcriptional regulator |  |
| bgl:bglu\_1g30290 | galactonate dehydratase; K01684 galactonate dehydratase [EC:4.2.1.6] | ec:4.2.1.6 |
| bgl:bglu\_1g30300 | hypothetical protein |  |
| bgl:bglu\_1g30310 | copper resistance D domain-containing protein; K07245 putative copper resistance protein D |  |
| bgl:bglu\_1g30320 | cytochrome c4 |  |
| bgl:bglu\_1g30330 | periplasmic cytochrome c containing protein |  |
| bgl:bglu\_1g30340 | ATP-dependent RNA helicase; K11927 ATP-dependent RNA helicase RhlE [EC:3.6.4.13] | ec:3.6.4.13 |
| bgl:bglu\_1g30350 | hypothetical protein |  |
| bgl:bglu\_1g30360 | hypothetical protein |  |
| bgl:bglu\_1g30370 | Gamma-glutamyltransferase 2; K00681 gamma-glutamyltranspeptidase [EC:2.3.2.2] | ec:2.3.2.2 |
| bgl:bglu\_1g30380 | glycerol-3-phosphate regulon repressor; K02444 DeoR family transcriptional regulator, glycerol-3-phosphate regulon repressor |  |
| bgl:bglu\_1g30390 | hypothetical protein |  |
| bgl:bglu\_1g30400 | hypothetical protein |  |
| bgl:bglu\_1g30410 | glycerol-3-phosphate dehydrogenase; K00111 glycerol-3-phosphate dehydrogenase [EC:1.1.5.3] | ec:1.1.5.3 |
| bgl:bglu\_1g30420 | glycerol kinase; K00864 glycerol kinase [EC:2.7.1.30] | ec:2.7.1.30 |
| bgl:bglu\_1g30430 | glycerol uptake facilitator protein; K02440 glycerol uptake facilitator protein |  |
| bgl:bglu\_1g30440 | HAD-superfamily hydrolase |  |
| bgl:bglu\_1g30450 | XRE family transcriptional regulator |  |
| bgl:bglu\_1g30460 | (dimethylallyl)adenosine tRNA methylthiotransferase; K06168 bifunctional enzyme involved in thiolation and methylation of tRNA |  |
| bgl:bglu\_1g30470 | PhoH family protein; K06217 phosphate starvation-inducible protein PhoH and related proteins |  |
| bgl:bglu\_1g30480 | unkown domain/metalloprotease fusion protein; K07042 probable rRNA maturation factor |  |

  
**Neighborhood Representations for "bte:BTH\_I0603"**  

| ID | Annotation | EC number |
| --- | --- | --- |
| bte:BTH\_I0593 | major facilitator family transporter; K08156 MFS transporter, DHA1 family, arabinose polymer transporter |  |
| bte:BTH\_I0594 | DNA-binding protein |  |
| bte:BTH\_I0595 | 3,4-dihydroxy-2-butanone 4-phosphate synthase; K02858 3,4-dihydroxy 2-butanone 4-phosphate synthase [EC:4.1.99.12] | ec:4.1.99.12 |
| bte:BTH\_I0596 | HAD-superfamily hydrolase |  |
| bte:BTH\_I0597 | hypothetical protein |  |
| bte:BTH\_I0598 | glycerol uptake facilitator protein; K02440 glycerol uptake facilitator protein |  |
| bte:BTH\_I0599 | glpK; glycerol kinase (EC:2.7.1.30); K00864 glycerol kinase [EC:2.7.1.30] | ec:2.7.1.30 |
| bte:BTH\_I0600 | glpD; glycerol-3-phosphate dehydrogenase (EC:1.1.5.3); K00111 glycerol-3-phosphate dehydrogenase [EC:1.1.5.3] | ec:1.1.5.3 |
| bte:BTH\_I0601 | hypothetical protein |  |
| bte:BTH\_I0602 | hypothetical protein |  |
| bte:BTH\_I0603 | glycerol-3-phosphate regulon repressor; K02444 DeoR family transcriptional regulator, glycerol-3-phosphate regulon repressor |  |
| bte:BTH\_I0604 | gamma-glutamyltransferase; K00681 gamma-glutamyltranspeptidase [EC:2.3.2.2] | ec:2.3.2.2 |
| bte:BTH\_I0605 | hypothetical protein |  |
| bte:BTH\_I0606 | hypothetical protein |  |
| bte:BTH\_I0607 | ATP-dependent RNA helicase RhlE; K11927 ATP-dependent RNA helicase RhlE [EC:3.6.4.13] | ec:3.6.4.13 |
| bte:BTH\_I0608 | cytochrome c family protein |  |
| bte:BTH\_I0609 | cytochrome c4 |  |
| bte:BTH\_I0610 | copper resistance protein; K07245 putative copper resistance protein D |  |
| bte:BTH\_I0611 | galactonate dehydratase; K01684 galactonate dehydratase [EC:4.2.1.6] | ec:4.2.1.6 |
| bte:BTH\_I0612 | hypothetical protein |  |
| bte:BTH\_I0613 | Ser/Thr protein phosphatase family protein |  |

  
**Neighborhood Representations for "bma:BMA0244"**  

| ID | Annotation | EC number |
| --- | --- | --- |
| bma:BMA0234 | asparagine synthase (EC:6.3.5.4); K01953 asparagine synthase (glutamine-hydrolysing) [EC:6.3.5.4] | ec:6.3.5.4 |
| bma:BMA0235 | 3-phosphoshikimate 1-carboxyvinyltransferase (EC:2.5.1.19); K00800 3-phosphoshikimate 1-carboxyvinyltransferase [EC:2.5.1.19] | ec:2.5.1.19 |
| bma:BMA0236 | pseudogene |  |
| bma:BMA0237 | hypothetical protein |  |
| bma:BMA0238 | hypothetical protein |  |
| bma:BMA0239 | glpF; glycerol uptake facilitator protein; K02440 glycerol uptake facilitator protein |  |
| bma:BMA0240 | glpK; glycerol kinase (EC:2.7.1.30); K00864 glycerol kinase [EC:2.7.1.30] | ec:2.7.1.30 |
| bma:BMA0241 | glpD; glycerol-3-phosphate dehydrogenase (EC:1.1.5.3); K00111 glycerol-3-phosphate dehydrogenase [EC:1.1.5.3] | ec:1.1.5.3 |
| bma:BMA0242 | pseudogene |  |
| bma:BMA0243 | hypothetical protein |  |
| bma:BMA0244 | glpR; glycerol-3-phosphate regulon repressor; K02444 DeoR family transcriptional regulator, glycerol-3-phosphate regulon repressor |  |
| bma:BMA0245 | ggt-1; gamma-glutamyltransferase (EC:2.3.2.2); K00681 gamma-glutamyltranspeptidase [EC:2.3.2.2] | ec:2.3.2.2 |
| bma:BMA0246 | hypothetical protein |  |
| bma:BMA0247 | hypothetical protein |  |
| bma:BMA0248 | rhlE-2; ATP-dependent RNA helicase RhlE; K11927 ATP-dependent RNA helicase RhlE [EC:3.6.4.13] | ec:3.6.4.13 |
| bma:BMA0249 | cytochrome c family protein |  |
| bma:BMA0250 | cytochrome c4 |  |
| bma:BMA0251 | hypothetical protein |  |
| bma:BMA0252 | copper resistance protein; K07245 putative copper resistance protein D |  |
| bma:BMA0253 | hypothetical protein |  |
| bma:BMA0254 | dgoA; galactonate dehydratase; K01684 galactonate dehydratase [EC:4.2.1.6] | ec:4.2.1.6 |

  
**Neighborhood Representations for "bml:BMA10229\_A2375"**  

| ID | Annotation | EC number |
| --- | --- | --- |
| bml:BMA10229\_A2365 | hypothetical protein |  |
| bml:BMA10229\_A2366 | asparagine synthase; K01953 asparagine synthase (glutamine-hydrolysing) [EC:6.3.5.4] | ec:6.3.5.4 |
| bml:BMA10229\_A2367 | aroA; 3-phosphoshikimate 1-carboxyvinyltransferase (EC:2.5.1.19); K00800 3-phosphoshikimate 1-carboxyvinyltransferase [EC:2.5.1.19] | ec:2.5.1.19 |
| bml:BMA10229\_A2368 | 3-phosphoshikimate 1-carboxyvinyltransferase; K00800 3-phosphoshikimate 1-carboxyvinyltransferase [EC:2.5.1.19] | ec:2.5.1.19 |
| bml:BMA10229\_A2369 | endoribonuclease L-PSP |  |
| bml:BMA10229\_A2370 | glpF; glycerol uptake facilitator protein; K02440 glycerol uptake facilitator protein |  |
| bml:BMA10229\_A2371 | glpK; glycerol kinase (EC:2.7.1.30); K00864 glycerol kinase [EC:2.7.1.30] | ec:2.7.1.30 |
| bml:BMA10229\_A2372 | glpD; glycerol-3-phosphate dehydrogenase (EC:1.1.5.3); K00111 glycerol-3-phosphate dehydrogenase [EC:1.1.5.3] | ec:1.1.5.3 |
| bml:BMA10229\_A2373 | lipoprotein |  |
| bml:BMA10229\_A2374 | hypothetical protein |  |
| bml:BMA10229\_A2375 | glpR; glycerol-3-phosphate regulon repressor; K02444 DeoR family transcriptional regulator, glycerol-3-phosphate regulon repressor |  |
| bml:BMA10229\_A2376 | ggt-1; gamma-glutamyltransferase (EC:2.3.2.2); K00681 gamma-glutamyltranspeptidase [EC:2.3.2.2] | ec:2.3.2.2 |
| bml:BMA10229\_A2377 | hypothetical protein |  |
| bml:BMA10229\_A2378 | hypothetical protein |  |
| bml:BMA10229\_A2379 | rhlE-2; ATP-dependent RNA helicase RhlE; K11927 ATP-dependent RNA helicase RhlE [EC:3.6.4.13] | ec:3.6.4.13 |
| bml:BMA10229\_A2380 | cytochrome c family protein |  |
| bml:BMA10229\_A2381 | cytochrome c4 |  |
| bml:BMA10229\_A2382 | copper resistance protein; K07245 putative copper resistance protein D |  |
| bml:BMA10229\_A2383 | dgoA; galactonate dehydratase; K01684 galactonate dehydratase [EC:4.2.1.6] | ec:4.2.1.6 |
| bml:BMA10229\_A2384 | hypothetical protein |  |
| bml:BMA10229\_A2385 | hypothetical protein |  |

  
**Neighborhood Representations for "bmn:BMA10247\_2456"**  

| ID | Annotation | EC number |
| --- | --- | --- |
| bmn:BMA10247\_2446 | asparagine synthase; K01953 asparagine synthase (glutamine-hydrolysing) [EC:6.3.5.4] | ec:6.3.5.4 |
| bmn:BMA10247\_2447 | aroA; 3-phosphoshikimate 1-carboxyvinyltransferase (EC:2.5.1.19); K00800 3-phosphoshikimate 1-carboxyvinyltransferase [EC:2.5.1.19] | ec:2.5.1.19 |
| bmn:BMA10247\_2448 | 3-phosphoshikimate 1-carboxyvinyltransferase; K00800 3-phosphoshikimate 1-carboxyvinyltransferase [EC:2.5.1.19] | ec:2.5.1.19 |
| bmn:BMA10247\_2449 | endoribonuclease L-PSP |  |
| bmn:BMA10247\_2450 | hypothetical protein |  |
| bmn:BMA10247\_2451 | glpF; glycerol uptake facilitator protein; K02440 glycerol uptake facilitator protein |  |
| bmn:BMA10247\_2452 | glpK; glycerol kinase (EC:2.7.1.30); K00864 glycerol kinase [EC:2.7.1.30] | ec:2.7.1.30 |
| bmn:BMA10247\_2453 | glpD; glycerol-3-phosphate dehydrogenase (EC:1.1.5.3); K00111 glycerol-3-phosphate dehydrogenase [EC:1.1.5.3] | ec:1.1.5.3 |
| bmn:BMA10247\_2454 | lipoprotein |  |
| bmn:BMA10247\_2455 | hypothetical protein |  |
| bmn:BMA10247\_2456 | glpR; glycerol-3-phosphate regulon repressor; K02444 DeoR family transcriptional regulator, glycerol-3-phosphate regulon repressor |  |
| bmn:BMA10247\_2457 | ggt-1; gamma-glutamyltransferase (EC:2.3.2.2); K00681 gamma-glutamyltranspeptidase [EC:2.3.2.2] | ec:2.3.2.2 |
| bmn:BMA10247\_2458 | hypothetical protein |  |
| bmn:BMA10247\_2459 | hypothetical protein |  |
| bmn:BMA10247\_2460 | ATP-dependent RNA helicase rhlE (EC:3.6.1.-); K11927 ATP-dependent RNA helicase RhlE [EC:3.6.4.13] | ec:3.6.4.13 |
| bmn:BMA10247\_2461 | cytochrome c family protein |  |
| bmn:BMA10247\_2462 | cytochrome c4 |  |
| bmn:BMA10247\_2463 | copper resistance protein; K07245 putative copper resistance protein D |  |
| bmn:BMA10247\_2464 | dgoA; galactonate dehydratase; K01684 galactonate dehydratase [EC:4.2.1.6] | ec:4.2.1.6 |
| bmn:BMA10247\_2465 | hypothetical protein |  |
| bmn:BMA10247\_2466 | hypothetical protein |  |

  
**Neighborhood Representations for "bmv:BMASAVP1\_A2702"**  

| ID | Annotation | EC number |
| --- | --- | --- |
| bmv:BMASAVP1\_A2692 | hypothetical protein |  |
| bmv:BMASAVP1\_A2693 | hypothetical protein |  |
| bmv:BMASAVP1\_A2694 | dgoA; galactonate dehydratase; K01684 galactonate dehydratase [EC:4.2.1.6] | ec:4.2.1.6 |
| bmv:BMASAVP1\_A2695 | putative copper resistance protein; K07245 putative copper resistance protein D |  |
| bmv:BMASAVP1\_A2696 | putative cytochrome c4 |  |
| bmv:BMASAVP1\_A2697 | cytochrome c family protein |  |
| bmv:BMASAVP1\_A2698 | rhlE; putative ATP-dependent RNA helicase rhlE (EC:3.6.1.-); K11927 ATP-dependent RNA helicase RhlE [EC:3.6.4.13] | ec:3.6.4.13 |
| bmv:BMASAVP1\_A2699 | hypothetical protein |  |
| bmv:BMASAVP1\_A2700 | hypothetical protein |  |
| bmv:BMASAVP1\_A2701 | ggt-1; gamma-glutamyltransferase (EC:2.3.2.2); K00681 gamma-glutamyltranspeptidase [EC:2.3.2.2] | ec:2.3.2.2 |
| bmv:BMASAVP1\_A2702 | glpR; glycerol-3-phosphate regulon repressor; K02444 DeoR family transcriptional regulator, glycerol-3-phosphate regulon repressor |  |
| bmv:BMASAVP1\_A2703 | hypothetical protein |  |
| bmv:BMASAVP1\_A2704 | putative lipoprotein |  |
| bmv:BMASAVP1\_A2705 | glpD; glycerol-3-phosphate dehydrogenase (EC:1.1.5.3); K00111 glycerol-3-phosphate dehydrogenase [EC:1.1.5.3] | ec:1.1.5.3 |
| bmv:BMASAVP1\_A2706 | glpK; glycerol kinase (EC:2.7.1.30); K00864 glycerol kinase [EC:2.7.1.30] | ec:2.7.1.30 |
| bmv:BMASAVP1\_A2707 | glpF; glycerol uptake facilitator protein; K02440 glycerol uptake facilitator protein |  |
| bmv:BMASAVP1\_A2708 | hypothetical protein |  |
| bmv:BMASAVP1\_A2709 | endoribonuclease L-PSP |  |
| bmv:BMASAVP1\_A2710 | putative 3-phosphoshikimate-1-carboxyvinyltransferase; K00800 3-phosphoshikimate 1-carboxyvinyltransferase [EC:2.5.1.19] | ec:2.5.1.19 |
| bmv:BMASAVP1\_A2711 | aroA; 3-phosphoshikimate 1-carboxyvinyltransferase (EC:2.5.1.19); K00800 3-phosphoshikimate 1-carboxyvinyltransferase [EC:2.5.1.19] | ec:2.5.1.19 |
| bmv:BMASAVP1\_A2712 | putative asparagine synthase; K01953 asparagine synthase (glutamine-hydrolysing) [EC:6.3.5.4] | ec:6.3.5.4 |

  
**Neighborhood Representations for "bpd:BURPS668\_0729"**  

| ID | Annotation | EC number |
| --- | --- | --- |
| bpd:BURPS668\_0720 | aroA; 3-phosphoshikimate 1-carboxyvinyltransferase (EC:2.5.1.19); K00800 3-phosphoshikimate 1-carboxyvinyltransferase [EC:2.5.1.19] | ec:2.5.1.19 |
| bpd:BURPS668\_0721 | aroA; 3-phosphoshikimate 1-carboxyvinyltransferase (EC:2.5.1.19); K00800 3-phosphoshikimate 1-carboxyvinyltransferase [EC:2.5.1.19] | ec:2.5.1.19 |
| bpd:BURPS668\_0722 | endoribonuclease L-PSP |  |
| bpd:BURPS668\_0723 | hypothetical protein |  |
| bpd:BURPS668\_0724 | glpF; glycerol uptake facilitator protein; K02440 glycerol uptake facilitator protein |  |
| bpd:BURPS668\_0725 | glpK; glycerol kinase (EC:2.7.1.30); K00864 glycerol kinase [EC:2.7.1.30] | ec:2.7.1.30 |
| bpd:BURPS668\_0726 | glpD; glycerol-3-phosphate dehydrogenase (EC:1.1.5.3); K00111 glycerol-3-phosphate dehydrogenase [EC:1.1.5.3] | ec:1.1.5.3 |
| bpd:BURPS668\_0727 | lipoprotein |  |
| bpd:BURPS668\_0728 | hypothetical protein |  |
| bpd:BURPS668\_0730 | hypothetical protein |  |
| bpd:BURPS668\_0729 | glpR; glycerol-3-phosphate regulon repressor; K02444 DeoR family transcriptional regulator, glycerol-3-phosphate regulon repressor |  |
| bpd:BURPS668\_0731 | gamma-glutamyltransferase |  |
| bpd:BURPS668\_0732 | hypothetical protein |  |
| bpd:BURPS668\_0733 | hypothetical protein |  |
| bpd:BURPS668\_0734 | hypothetical protein |  |
| bpd:BURPS668\_0735 | ATP-dependent RNA helicase rhlE; K11927 ATP-dependent RNA helicase RhlE [EC:3.6.4.13] | ec:3.6.4.13 |
| bpd:BURPS668\_0736 | cytochrome c family protein |  |
| bpd:BURPS668\_0737 | cytochrome |  |
| bpd:BURPS668\_0738 | hypothetical protein |  |
| bpd:BURPS668\_0739 | copper resistance family protein; K07245 putative copper resistance protein D |  |
| bpd:BURPS668\_0740 | galactonate dehydratase; K01684 galactonate dehydratase [EC:4.2.1.6] | ec:4.2.1.6 |

  
**Neighborhood Representations for "bpl:BURPS1106A\_0743"**  

| ID | Annotation | EC number |
| --- | --- | --- |
| bpl:BURPS1106A\_0733 | asparagine synthase; K01953 asparagine synthase (glutamine-hydrolysing) [EC:6.3.5.4] | ec:6.3.5.4 |
| bpl:BURPS1106A\_0734 | aroA; 3-phosphoshikimate 1-carboxyvinyltransferase (EC:2.5.1.19); K00800 3-phosphoshikimate 1-carboxyvinyltransferase [EC:2.5.1.19] | ec:2.5.1.19 |
| bpl:BURPS1106A\_0735 | aroA; 3-phosphoshikimate 1-carboxyvinyltransferase (EC:2.5.1.19); K00800 3-phosphoshikimate 1-carboxyvinyltransferase [EC:2.5.1.19] | ec:2.5.1.19 |
| bpl:BURPS1106A\_0736 | endoribonuclease L-PSP |  |
| bpl:BURPS1106A\_0737 | hypothetical protein |  |
| bpl:BURPS1106A\_0738 | glpF; glycerol uptake facilitator protein; K02440 glycerol uptake facilitator protein |  |
| bpl:BURPS1106A\_0739 | glpK; glycerol kinase (EC:2.7.1.30); K00864 glycerol kinase [EC:2.7.1.30] | ec:2.7.1.30 |
| bpl:BURPS1106A\_0740 | glpD; glycerol-3-phosphate dehydrogenase (EC:1.1.5.3); K00111 glycerol-3-phosphate dehydrogenase [EC:1.1.5.3] | ec:1.1.5.3 |
| bpl:BURPS1106A\_0741 | lipoprotein |  |
| bpl:BURPS1106A\_0742 | hypothetical protein |  |
| bpl:BURPS1106A\_0743 | glpR; glycerol-3-phosphate regulon repressor; K02444 DeoR family transcriptional regulator, glycerol-3-phosphate regulon repressor |  |
| bpl:BURPS1106A\_0744 | gamma-glutamyltransferase; K00681 gamma-glutamyltranspeptidase [EC:2.3.2.2] | ec:2.3.2.2 |
| bpl:BURPS1106A\_0745 | hypothetical protein |  |
| bpl:BURPS1106A\_0746 | hypothetical protein |  |
| bpl:BURPS1106A\_0747 | hypothetical protein |  |
| bpl:BURPS1106A\_0748 | ATP-dependent RNA helicase rhlE; K11927 ATP-dependent RNA helicase RhlE [EC:3.6.4.13] | ec:3.6.4.13 |
| bpl:BURPS1106A\_0749 | cytochrome c family protein |  |
| bpl:BURPS1106A\_0750 | cytochrome |  |
| bpl:BURPS1106A\_0751 | copper resistance family protein; K07245 putative copper resistance protein D |  |
| bpl:BURPS1106A\_0752 | galactonate dehydratase; K01684 galactonate dehydratase [EC:4.2.1.6] | ec:4.2.1.6 |
| bpl:BURPS1106A\_0753 | hypothetical protein |  |

  
**Neighborhood Representations for "bpm:BURPS1710b\_0910"**  

| ID | Annotation | EC number |
| --- | --- | --- |
| bpm:BURPS1710b\_0900 | asnB; asparagine synthase (EC:6.3.5.4); K01953 asparagine synthase (glutamine-hydrolysing) [EC:6.3.5.4] | ec:6.3.5.4 |
| bpm:BURPS1710b\_0901 | hypothetical protein |  |
| bpm:BURPS1710b\_0902 | aroA; 3-phosphoshikimate 1-carboxyvinyltransferase (EC:2.5.1.19); K00800 3-phosphoshikimate 1-carboxyvinyltransferase [EC:2.5.1.19] | ec:2.5.1.19 |
| bpm:BURPS1710b\_0903 | 3-phosphoskimimate 1-carboxyvinyltransferase (EC:2.5.1.19); K00800 3-phosphoshikimate 1-carboxyvinyltransferase [EC:2.5.1.19] | ec:2.5.1.19 |
| bpm:BURPS1710b\_0904 | hypothetical protein |  |
| bpm:BURPS1710b\_0905 | glpF; glycerol uptake facilitator protein; K02440 glycerol uptake facilitator protein |  |
| bpm:BURPS1710b\_0906 | glpK; glycerol kinase (EC:2.7.1.30); K00864 glycerol kinase [EC:2.7.1.30] | ec:2.7.1.30 |
| bpm:BURPS1710b\_0907 | glpD; glycerol-3-phosphate dehydrogenase (EC:1.1.5.3); K00111 glycerol-3-phosphate dehydrogenase [EC:1.1.5.3] | ec:1.1.5.3 |
| bpm:BURPS1710b\_0908 | hypothetical protein |  |
| bpm:BURPS1710b\_0909 | hypothetical protein |  |
| bpm:BURPS1710b\_0910 | glpR; glycerol-3-phosphate regulon repressor; K02444 DeoR family transcriptional regulator, glycerol-3-phosphate regulon repressor |  |
| bpm:BURPS1710b\_0911 | ggt-1; gamma-glutamyltransferase 2 (EC:2.3.2.2); K00681 gamma-glutamyltranspeptidase [EC:2.3.2.2] | ec:2.3.2.2 |
| bpm:BURPS1710b\_0912 | hypothetical protein |  |
| bpm:BURPS1710b\_0913 | hypothetical protein |  |
| bpm:BURPS1710b\_0914 | rhlE1; ATP-dependent RNA helicase; K11927 ATP-dependent RNA helicase RhlE [EC:3.6.4.13] | ec:3.6.4.13 |
| bpm:BURPS1710b\_0915 | cytochrome C (EC:1.1.-.-) |  |
| bpm:BURPS1710b\_0916 | cytC4; cytochrome c |  |
| bpm:BURPS1710b\_0917 | copD; transmembrane transporter protein; K07245 putative copper resistance protein D |  |
| bpm:BURPS1710b\_0918 | dgoA; galactonate dehydratase (EC:4.2.1.6); K01684 galactonate dehydratase [EC:4.2.1.6] | ec:4.2.1.6 |
| bpm:BURPS1710b\_0919 | hypothetical protein |  |
| bpm:BURPS1710b\_0920 | hypothetical protein |  |

  
**Neighborhood Representations for "bpr:GBP346\_A0659"**  

| ID | Annotation | EC number |
| --- | --- | --- |
| bpr:GBP346\_A0650 | 3-phosphoshikimate 1-carboxyvinyltransferase (EC:2.5.1.19); K00800 3-phosphoshikimate 1-carboxyvinyltransferase [EC:2.5.1.19] | ec:2.5.1.19 |
| bpr:GBP346\_A0651 | 3-phosphoshikimate 1-carboxyvinyltransferase; K00800 3-phosphoshikimate 1-carboxyvinyltransferase [EC:2.5.1.19] | ec:2.5.1.19 |
| bpr:GBP346\_A0652 | endoribonuclease L-PSP |  |
| bpr:GBP346\_A0653 | hypothetical protein |  |
| bpr:GBP346\_A0654 | glycerol uptake facilitator protein; K02440 glycerol uptake facilitator protein |  |
| bpr:GBP346\_A0655 | glpK; glycerol kinase (EC:2.7.1.30); K00864 glycerol kinase [EC:2.7.1.30] | ec:2.7.1.30 |
| bpr:GBP346\_A0656 | glpD; glycerol-3-phosphate dehydrogenase (EC:1.1.5.3); K00111 glycerol-3-phosphate dehydrogenase [EC:1.1.5.3] | ec:1.1.5.3 |
| bpr:GBP346\_A0657 | putative lipoprotein |  |
| bpr:GBP346\_A0658 | hypothetical protein |  |
| bpr:GBP346\_A0660 | hypothetical protein |  |
| bpr:GBP346\_A0659 | glpR; glycerol-3-phosphate regulon repressor; K02444 DeoR family transcriptional regulator, glycerol-3-phosphate regulon repressor |  |
| bpr:GBP346\_A0661 | putative gamma-glutamyltransferase; K00681 gamma-glutamyltranspeptidase [EC:2.3.2.2] | ec:2.3.2.2 |
| bpr:GBP346\_A0662 | hypothetical protein |  |
| bpr:GBP346\_A0663 | hypothetical protein |  |
| bpr:GBP346\_A0664 | hypothetical protein |  |
| bpr:GBP346\_A0665 | rhlE; ATP-dependent RNA helicase RhlE; K11927 ATP-dependent RNA helicase RhlE [EC:3.6.4.13] | ec:3.6.4.13 |
| bpr:GBP346\_A0666 | cytochrome c family protein |  |
| bpr:GBP346\_A0667 | cytochrome c, class I |  |
| bpr:GBP346\_A0668 | hypothetical protein |  |
| bpr:GBP346\_A0669 | copper resistance D; K07245 putative copper resistance protein D |  |
| bpr:GBP346\_A0670 | galactonate dehydratase; K01684 galactonate dehydratase [EC:4.2.1.6] | ec:4.2.1.6 |

  
**Neighborhood Representations for "bps:BPSL0691"**  

| ID | Annotation | EC number |
| --- | --- | --- |
| bps:BPSL0681 | hypothetical protein |  |
| bps:BPSL0682 | asparagine synthetase B (EC:6.3.5.4); K01953 asparagine synthase (glutamine-hydrolysing) [EC:6.3.5.4] | ec:6.3.5.4 |
| bps:BPSL0683 | 3-phosphoshikimate 1-carboxyvinyltransferase (EC:2.5.1.19); K00800 3-phosphoshikimate 1-carboxyvinyltransferase [EC:2.5.1.19] | ec:2.5.1.19 |
| bps:BPSL0684 | 3-phosphoskimimate 1-carboxyvinyltransferase (EC:2.5.1.19); K00800 3-phosphoshikimate 1-carboxyvinyltransferase [EC:2.5.1.19] | ec:2.5.1.19 |
| bps:BPSL0685 | hypothetical protein |  |
| bps:BPSL0686 | glpF; glycerol uptake facilitator protein; K02440 glycerol uptake facilitator protein |  |
| bps:BPSL0687 | glpK; glycerol kinase (EC:2.7.1.30); K00864 glycerol kinase [EC:2.7.1.30] | ec:2.7.1.30 |
| bps:BPSL0688 | glpD; glycerol-3-phosphate dehydrogenase (EC:1.1.5.3); K00111 glycerol-3-phosphate dehydrogenase [EC:1.1.5.3] | ec:1.1.5.3 |
| bps:BPSL0689 | hypothetical protein |  |
| bps:BPSL0690 | hypothetical protein |  |
| bps:BPSL0691 | glpR; DeoR family glycerol-3-phosphate regulon repressor; K02444 DeoR family transcriptional regulator, glycerol-3-phosphate regulon repressor |  |
| bps:BPSL0692 | gamma-glutamyltransferase 2; K00681 gamma-glutamyltranspeptidase [EC:2.3.2.2] | ec:2.3.2.2 |
| bps:BPSL0693 | hypothetical protein |  |
| bps:BPSL0694 | hypothetical protein |  |
| bps:BPSL0695 | rhlE1; ATP-dependent RNA helicase 1; K11927 ATP-dependent RNA helicase RhlE [EC:3.6.4.13] | ec:3.6.4.13 |
| bps:BPSL0696 | periplasmic cytochrome c containing protein |  |
| bps:BPSL0697 | periplasmic cytochrome c protein |  |
| bps:BPSL0698 | transmembrane transporter protein; K07245 putative copper resistance protein D |  |
| bps:BPSL0699 | galactonate dehydratase; K01684 galactonate dehydratase [EC:4.2.1.6] | ec:4.2.1.6 |
| bps:BPSL0700 | hypothetical protein |  |
| bps:BPSL0701 | hypothetical protein |  |

  
**Neighborhood Representations for "bmj:BMULJ\_02637"**  

| ID | Annotation | EC number |
| --- | --- | --- |
| bmj:BMULJ\_02627 | hypothetical protein; K09984 hypothetical protein |  |
| bmj:BMULJ\_02628 | GntR family transcriptional regulator |  |
| bmj:BMULJ\_02629 | dgoA; galactonate dehydratase (EC:4.2.1.6); K01684 galactonate dehydratase [EC:4.2.1.6] | ec:4.2.1.6 |
| bmj:BMULJ\_02630 | pcoD; putative copper resistance protein D; K07245 putative copper resistance protein D |  |
| bmj:BMULJ\_02631 | cytochrome c553 |  |
| bmj:BMULJ\_02632 | cccA; putative periplasmic cytochrome c-containing protein |  |
| bmj:BMULJ\_02633 | deaD; ATP-dependent RNA helicase; K11927 ATP-dependent RNA helicase RhlE [EC:3.6.4.13] | ec:3.6.4.13 |
| bmj:BMULJ\_02634 | hypothetical protein |  |
| bmj:BMULJ\_02635 | hypothetical protein |  |
| bmj:BMULJ\_02636 | ggt; gamma-glutamyltranspeptidase (EC:2.3.2.2); K00681 gamma-glutamyltranspeptidase [EC:2.3.2.2] | ec:2.3.2.2 |
| bmj:BMULJ\_02637 | glpR; glycerol-3-phosphate regulon repressor; K02444 DeoR family transcriptional regulator, glycerol-3-phosphate regulon repressor |  |
| bmj:BMULJ\_02638 | hypothetical protein |  |
| bmj:BMULJ\_02639 | hypothetical protein |  |
| bmj:BMULJ\_02640 | glpD; glycerol-3-phosphate dehydrogenase (EC:1.1.5.3); K00111 glycerol-3-phosphate dehydrogenase [EC:1.1.5.3] | ec:1.1.5.3 |
| bmj:BMULJ\_02641 | glpK; glycerol kinase (EC:2.7.1.30); K00864 glycerol kinase [EC:2.7.1.30] | ec:2.7.1.30 |
| bmj:BMULJ\_02642 | glpF; glycerol uptake facilitator protein; K02440 glycerol uptake facilitator protein |  |
| bmj:BMULJ\_02643 | hcaD; ferredoxin-NAD+ reductase (EC:1.18.1.3); K00529 ferredoxin--NAD+ reductase [EC:1.18.1.3] | ec:1.18.1.3 |
| bmj:BMULJ\_02644 | hcaC; ferredoxin subunit of phenylpropionate dioxygenase; K05710 dioxygenase ferredoxin subunit |  |
| bmj:BMULJ\_02645 | putative fatty acid desaturase |  |
| bmj:BMULJ\_02646 | ABC-type sugar transporter periplasmic component; K02529 LacI family transcriptional regulator |  |
| bmj:BMULJ\_02647 | hypothetical protein |  |

  
**Neighborhood Representations for "bmu:Bmul\_0623"**  

| ID | Annotation | EC number |
| --- | --- | --- |
| bmu:Bmul\_0613 | hypothetical protein |  |
| bmu:Bmul\_0614 | LacI family transcriptional regulator; K02529 LacI family transcriptional regulator |  |
| bmu:Bmul\_0615 | fatty acid desaturase |  |
| bmu:Bmul\_0616 | Rieske (2Fe-2S) domain-containing protein; K05710 dioxygenase ferredoxin subunit |  |
| bmu:Bmul\_0617 | FAD-dependent pyridine nucleotide-disulfide oxidoreductase; K00529 ferredoxin--NAD+ reductase [EC:1.18.1.3] | ec:1.18.1.3 |
| bmu:Bmul\_0618 | MIP family channel protein; K02440 glycerol uptake facilitator protein |  |
| bmu:Bmul\_0619 | glpK; glycerol kinase; K00864 glycerol kinase [EC:2.7.1.30] | ec:2.7.1.30 |
| bmu:Bmul\_0620 | glpD; glycerol-3-phosphate dehydrogenase; K00111 glycerol-3-phosphate dehydrogenase [EC:1.1.5.3] | ec:1.1.5.3 |
| bmu:Bmul\_0621 | hypothetical protein |  |
| bmu:Bmul\_0622 | hypothetical protein |  |
| bmu:Bmul\_0623 | DeoR family transcriptional regulator; K02444 DeoR family transcriptional regulator, glycerol-3-phosphate regulon repressor |  |
| bmu:Bmul\_0624 | gamma-glutamyltransferase (EC:2.3.2.2); K00681 gamma-glutamyltranspeptidase [EC:2.3.2.2] | ec:2.3.2.2 |
| bmu:Bmul\_0625 | hypothetical protein |  |
| bmu:Bmul\_0626 | hypothetical protein |  |
| bmu:Bmul\_0627 | DEAD/DEAH box helicase; K11927 ATP-dependent RNA helicase RhlE [EC:3.6.4.13] | ec:3.6.4.13 |
| bmu:Bmul\_0628 | gluconate 2-dehydrogenase (EC:1.1.99.3) |  |
| bmu:Bmul\_0629 | cytochrome c, class I |  |
| bmu:Bmul\_0630 | copper resistance D domain-containing protein; K07245 putative copper resistance protein D |  |
| bmu:Bmul\_0631 | galactonate dehydratase; K01684 galactonate dehydratase [EC:4.2.1.6] | ec:4.2.1.6 |
| bmu:Bmul\_0632 | GntR family transcriptional regulator |  |
| bmu:Bmul\_0633 | hypothetical protein; K09984 hypothetical protein |  |

  
**Neighborhood Representations for "bvi:Bcep1808\_2786"**  

| ID | Annotation | EC number |
| --- | --- | --- |
| bvi:Bcep1808\_2776 | glutathione-dependent formaldehyde-activating protein |  |
| bvi:Bcep1808\_2777 | GntR family transcriptional regulator |  |
| bvi:Bcep1808\_2778 | galactonate dehydratase (EC:4.2.1.6); K01684 galactonate dehydratase [EC:4.2.1.6] | ec:4.2.1.6 |
| bvi:Bcep1808\_2779 | copper resistance D domain-containing protein; K07245 putative copper resistance protein D |  |
| bvi:Bcep1808\_2780 | class I cytochrome c |  |
| bvi:Bcep1808\_2781 | class I cytochrome c |  |
| bvi:Bcep1808\_2782 | DEAD/DEAH box helicase domain-containing protein; K11927 ATP-dependent RNA helicase RhlE [EC:3.6.4.13] | ec:3.6.4.13 |
| bvi:Bcep1808\_2783 | hypothetical protein |  |
| bvi:Bcep1808\_2784 | hypothetical protein |  |
| bvi:Bcep1808\_2785 | gamma-glutamyltransferase 2 (EC:2.3.2.2); K00681 gamma-glutamyltranspeptidase [EC:2.3.2.2] | ec:2.3.2.2 |
| bvi:Bcep1808\_2786 | DeoR family transcriptional regulator; K02444 DeoR family transcriptional regulator, glycerol-3-phosphate regulon repressor |  |
| bvi:Bcep1808\_2787 | hypothetical protein |  |
| bvi:Bcep1808\_2788 | hypothetical protein |  |
| bvi:Bcep1808\_2789 | glpD; glycerol-3-phosphate dehydrogenase (EC:1.1.5.3); K00111 glycerol-3-phosphate dehydrogenase [EC:1.1.5.3] | ec:1.1.5.3 |
| bvi:Bcep1808\_2790 | glpK; glycerol kinase (EC:2.7.1.30); K00864 glycerol kinase [EC:2.7.1.30] | ec:2.7.1.30 |
| bvi:Bcep1808\_2791 | MIP family channel protein; K02440 glycerol uptake facilitator protein |  |
| bvi:Bcep1808\_2792 | HAD family hydrolase |  |
| bvi:Bcep1808\_2793 | 3,4-dihydroxy-2-butanone 4-phosphate synthase; K02858 3,4-dihydroxy 2-butanone 4-phosphate synthase [EC:4.1.99.12] | ec:4.1.99.12 |
| bvi:Bcep1808\_2794 | XRE family transcriptional regulator |  |
| bvi:Bcep1808\_2795 | (dimethylallyl)adenosine tRNA methylthiotransferase; K06168 bifunctional enzyme involved in thiolation and methylation of tRNA |  |
| bvi:Bcep1808\_2796 | PhoH family protein; K06217 phosphate starvation-inducible protein PhoH and related proteins |  |

  
**Neighborhood Representations for "bch:Bcen2424\_2674"**  

| ID | Annotation | EC number |
| --- | --- | --- |
| bch:Bcen2424\_2664 | hypothetical protein; K09984 hypothetical protein |  |
| bch:Bcen2424\_2665 | GntR domain-containing protein |  |
| bch:Bcen2424\_2666 | galactonate dehydratase; K01684 galactonate dehydratase [EC:4.2.1.6] | ec:4.2.1.6 |
| bch:Bcen2424\_2667 | copper resistance D domain-containing protein; K07245 putative copper resistance protein D |  |
| bch:Bcen2424\_2668 | cytochrome c, class I |  |
| bch:Bcen2424\_2669 | cytochrome c, class I |  |
| bch:Bcen2424\_2670 | DEAD/DEAH box helicase domain-containing protein; K11927 ATP-dependent RNA helicase RhlE [EC:3.6.4.13] | ec:3.6.4.13 |
| bch:Bcen2424\_2671 | hypothetical protein |  |
| bch:Bcen2424\_2672 | hypothetical protein |  |
| bch:Bcen2424\_2673 | gamma-glutamyltransferase (EC:2.3.2.2); K00681 gamma-glutamyltranspeptidase [EC:2.3.2.2] | ec:2.3.2.2 |
| bch:Bcen2424\_2674 | DeoR family transcriptional regulator; K02444 DeoR family transcriptional regulator, glycerol-3-phosphate regulon repressor |  |
| bch:Bcen2424\_2675 | hypothetical protein |  |
| bch:Bcen2424\_2676 | hypothetical protein |  |
| bch:Bcen2424\_2677 | glpD; glycerol-3-phosphate dehydrogenase (EC:1.1.5.3); K00111 glycerol-3-phosphate dehydrogenase [EC:1.1.5.3] | ec:1.1.5.3 |
| bch:Bcen2424\_2678 | glpK; glycerol kinase (EC:2.7.1.30); K00864 glycerol kinase [EC:2.7.1.30] | ec:2.7.1.30 |
| bch:Bcen2424\_2679 | MIP family channel protein; K02440 glycerol uptake facilitator protein |  |
| bch:Bcen2424\_2680 | FAD-dependent pyridine nucleotide-disulfide oxidoreductase; K00529 ferredoxin--NAD+ reductase [EC:1.18.1.3] | ec:1.18.1.3 |
| bch:Bcen2424\_2681 | Rieske (2Fe-2S) domain-containing protein; K05710 dioxygenase ferredoxin subunit |  |
| bch:Bcen2424\_2682 | fatty acid desaturase |  |
| bch:Bcen2424\_2683 | LacI family transcription regulator; K02529 LacI family transcriptional regulator |  |
| bch:Bcen2424\_2684 | hypothetical protein |  |

  
**Neighborhood Representations for "bcj:BCAL0929"**  

| ID | Annotation | EC number |
| --- | --- | --- |
| bcj:BCAL0919 | hypothetical protein |  |
| bcj:BCAL0920 | LacI family regulatory protein; K02529 LacI family transcriptional regulator |  |
| bcj:BCAL0921 | fatty acid desaturase |  |
| bcj:BCAL0922 | 2Fe-2S ferredoxin; K05710 dioxygenase ferredoxin subunit |  |
| bcj:BCAL0923 | putative oxidoreductases; K00529 ferredoxin--NAD+ reductase [EC:1.18.1.3] | ec:1.18.1.3 |
| bcj:BCAL0924 | glpF; putative glycerol uptake facilitator protein; K02440 glycerol uptake facilitator protein |  |
| bcj:BCAL0925 | glpK; glycerol kinase (EC:2.7.1.30); K00864 glycerol kinase [EC:2.7.1.30] | ec:2.7.1.30 |
| bcj:BCAL0926 | glpD; glycerol-3-phosphate dehydrogenase (EC:1.1.5.3); K00111 glycerol-3-phosphate dehydrogenase [EC:1.1.5.3] | ec:1.1.5.3 |
| bcj:BCAL0927 | hypothetical protein |  |
| bcj:BCAL0928 | hypothetical protein |  |
| bcj:BCAL0929 | glpR; putative DeoR family glycerol-3-phosphate regulon repressor; K02444 DeoR family transcriptional regulator, glycerol-3-phosphate regulon repressor |  |
| bcj:BCAL0930 | putative gamma-glutamyltransferase; K00681 gamma-glutamyltranspeptidase [EC:2.3.2.2] | ec:2.3.2.2 |
| bcj:BCAL0931 | hypothetical protein |  |
| bcj:BCAL0932 | hypothetical protein |  |
| bcj:BCAL0933 | rhlE1; putative ATP-dependent RNA helicase 1; K11927 ATP-dependent RNA helicase RhlE [EC:3.6.4.13] | ec:3.6.4.13 |
| bcj:BCAL0934 | putative periplasmic cytochrome c containing protein |  |
| bcj:BCAL0935 | putative periplasmic cytochrome c protein |  |
| bcj:BCAL0936 | putative transmembrane transporter protein; K07245 putative copper resistance protein D |  |
| bcj:BCAL0937 | galactonate dehydratase; K01684 galactonate dehydratase [EC:4.2.1.6] | ec:4.2.1.6 |
| bcj:BCAL0938 | pseudogene |  |
| bcj:BCAL0939 | hypothetical protein |  |

  
**Neighborhood Representations for "bur:Bcep18194\_A6002"**  

| ID | Annotation | EC number |
| --- | --- | --- |
| bur:Bcep18194\_A5992 | hypothetical protein; K09984 hypothetical protein |  |
| bur:Bcep18194\_A5993 | GntR family transcriptional regulator |  |
| bur:Bcep18194\_A5994 | galactonate dehydratase (EC:4.2.1.6); K01684 galactonate dehydratase [EC:4.2.1.6] | ec:4.2.1.6 |
| bur:Bcep18194\_A5995 | copper resistance D; K07245 putative copper resistance protein D |  |
| bur:Bcep18194\_A5996 | cytochrome c, class I |  |
| bur:Bcep18194\_A5997 | cytochrome c, class I |  |
| bur:Bcep18194\_A5998 | DEAD/DEAH box helicase; K11927 ATP-dependent RNA helicase RhlE [EC:3.6.4.13] | ec:3.6.4.13 |
| bur:Bcep18194\_A5999 | hypothetical protein |  |
| bur:Bcep18194\_A6000 | hypothetical protein |  |
| bur:Bcep18194\_A6001 | gamma-glutamyltransferase 2 (EC:2.3.2.2); K00681 gamma-glutamyltranspeptidase [EC:2.3.2.2] | ec:2.3.2.2 |
| bur:Bcep18194\_A6002 | DeoR family transcriptional regulator; K02444 DeoR family transcriptional regulator, glycerol-3-phosphate regulon repressor |  |
| bur:Bcep18194\_A6003 | hypothetical protein |  |
| bur:Bcep18194\_A6004 | hypothetical protein |  |
| bur:Bcep18194\_A6005 | glpD; glycerol-3-phosphate dehydrogenase (EC:1.1.5.3); K00111 glycerol-3-phosphate dehydrogenase [EC:1.1.5.3] | ec:1.1.5.3 |
| bur:Bcep18194\_A6006 | glpK; glycerol kinase (EC:2.7.1.30); K00864 glycerol kinase [EC:2.7.1.30] | ec:2.7.1.30 |
| bur:Bcep18194\_A6007 | Aquaporin; K02440 glycerol uptake facilitator protein |  |
| bur:Bcep18194\_A6008 | FAD-dependent pyridine nucleotide-disulfide oxidoreductase (EC:1.18.1.3); K00529 ferredoxin--NAD+ reductase [EC:1.18.1.3] | ec:1.18.1.3 |
| bur:Bcep18194\_A6009 | Rieske (2Fe-2S) protein; K05710 dioxygenase ferredoxin subunit |  |
| bur:Bcep18194\_A6010 | fatty acid desaturase |  |
| bur:Bcep18194\_A6011 | LacI family transcriptional regulator; K02529 LacI family transcriptional regulator |  |
| bur:Bcep18194\_A6012 | hypothetical protein |  |

  
**Neighborhood Representations for "bcn:Bcen\_2063"**  

| ID | Annotation | EC number |
| --- | --- | --- |
| bcn:Bcen\_2053 | hypothetical protein; K09984 hypothetical protein |  |
| bcn:Bcen\_2054 | GntR family transcriptional regulator |  |
| bcn:Bcen\_2055 | galactonate dehydratase; K01684 galactonate dehydratase [EC:4.2.1.6] | ec:4.2.1.6 |
| bcn:Bcen\_2056 | copper resistance D; K07245 putative copper resistance protein D |  |
| bcn:Bcen\_2057 | cytochrome c, class I |  |
| bcn:Bcen\_2058 | cytochrome c, class I |  |
| bcn:Bcen\_2059 | DEAD/DEAH box helicase; K11927 ATP-dependent RNA helicase RhlE [EC:3.6.4.13] | ec:3.6.4.13 |
| bcn:Bcen\_2060 | hypothetical protein |  |
| bcn:Bcen\_2061 | hypothetical protein |  |
| bcn:Bcen\_2062 | gamma-glutamyltransferase (EC:2.3.2.2); K00681 gamma-glutamyltranspeptidase [EC:2.3.2.2] | ec:2.3.2.2 |
| bcn:Bcen\_2063 | DeoR family transcriptional regulator; K02444 DeoR family transcriptional regulator, glycerol-3-phosphate regulon repressor |  |
| bcn:Bcen\_2064 | hypothetical protein |  |
| bcn:Bcen\_2065 | hypothetical protein |  |
| bcn:Bcen\_2066 | glpD; glycerol-3-phosphate dehydrogenase (EC:1.1.5.3); K00111 glycerol-3-phosphate dehydrogenase [EC:1.1.5.3] | ec:1.1.5.3 |
| bcn:Bcen\_2067 | glpK; glycerol kinase (EC:2.7.1.30); K00864 glycerol kinase [EC:2.7.1.30] | ec:2.7.1.30 |
| bcn:Bcen\_2068 | MIP family channel protein; K02440 glycerol uptake facilitator protein |  |
| bcn:Bcen\_2069 | FAD-dependent pyridine nucleotide-disulfide oxidoreductase; K00529 ferredoxin--NAD+ reductase [EC:1.18.1.3] | ec:1.18.1.3 |
| bcn:Bcen\_2070 | MocE Rieske (2Fe-2S); K05710 dioxygenase ferredoxin subunit |  |
| bcn:Bcen\_2071 | fatty acid desaturase |  |
| bcn:Bcen\_2072 | LacI family transcriptional regulator; K02529 LacI family transcriptional regulator |  |
| bcn:Bcen\_2073 | hypothetical protein |  |

  
**Neighborhood Representations for "bac:BamMC406\_2599"**  

| ID | Annotation | EC number |
| --- | --- | --- |
| bac:BamMC406\_2589 | hypothetical protein; K09984 hypothetical protein |  |
| bac:BamMC406\_2590 | GntR family transcriptional regulator |  |
| bac:BamMC406\_2591 | galactonate dehydratase; K01684 galactonate dehydratase [EC:4.2.1.6] | ec:4.2.1.6 |
| bac:BamMC406\_2592 | copper resistance D domain-containing protein; K07245 putative copper resistance protein D |  |
| bac:BamMC406\_2593 | cytochrome c, class I |  |
| bac:BamMC406\_2594 | gluconate 2-dehydrogenase (acceptor) (EC:1.1.99.3) |  |
| bac:BamMC406\_2595 | DEAD/DEAH box helicase; K11927 ATP-dependent RNA helicase RhlE [EC:3.6.4.13] | ec:3.6.4.13 |
| bac:BamMC406\_2596 | hypothetical protein |  |
| bac:BamMC406\_2597 | hypothetical protein |  |
| bac:BamMC406\_2598 | gamma-glutamyltransferase (EC:2.3.2.2); K00681 gamma-glutamyltranspeptidase [EC:2.3.2.2] | ec:2.3.2.2 |
| bac:BamMC406\_2599 | DeoR family transcriptional regulator; K02444 DeoR family transcriptional regulator, glycerol-3-phosphate regulon repressor |  |
| bac:BamMC406\_2600 | hypothetical protein |  |
| bac:BamMC406\_2601 | hypothetical protein |  |
| bac:BamMC406\_2602 | glpD; glycerol-3-phosphate dehydrogenase; K00111 glycerol-3-phosphate dehydrogenase [EC:1.1.5.3] | ec:1.1.5.3 |
| bac:BamMC406\_2603 | glpK; glycerol kinase; K00864 glycerol kinase [EC:2.7.1.30] | ec:2.7.1.30 |
| bac:BamMC406\_2604 | MIP family channel protein; K02440 glycerol uptake facilitator protein |  |
| bac:BamMC406\_2605 | HAD family hydrolase |  |
| bac:BamMC406\_2606 | 3,4-dihydroxy-2-butanone 4-phosphate synthase; K02858 3,4-dihydroxy 2-butanone 4-phosphate synthase [EC:4.1.99.12] | ec:4.1.99.12 |
| bac:BamMC406\_2607 | XRE family transcriptional regulator |  |
| bac:BamMC406\_2608 | major facilitator transporter; K08156 MFS transporter, DHA1 family, arabinose polymer transporter |  |
| bac:BamMC406\_2609 | LysR family transcriptional regulator |  |

  
**Neighborhood Representations for "bam:Bamb\_2727"**  

| ID | Annotation | EC number |
| --- | --- | --- |
| bam:Bamb\_2717 | hypothetical protein; K09984 hypothetical protein |  |
| bam:Bamb\_2718 | GntR family transcriptional regulator |  |
| bam:Bamb\_2719 | galactonate dehydratase; K01684 galactonate dehydratase [EC:4.2.1.6] | ec:4.2.1.6 |
| bam:Bamb\_2720 | copper resistance D domain-containing protein; K07245 putative copper resistance protein D |  |
| bam:Bamb\_2721 | class I cytochrome c |  |
| bam:Bamb\_2722 | class I cytochrome c |  |
| bam:Bamb\_2723 | DEAD/DEAH box helicase; K11927 ATP-dependent RNA helicase RhlE [EC:3.6.4.13] | ec:3.6.4.13 |
| bam:Bamb\_2724 | hypothetical protein |  |
| bam:Bamb\_2725 | hypothetical protein |  |
| bam:Bamb\_2726 | gamma-glutamyltransferase (EC:2.3.2.2); K00681 gamma-glutamyltranspeptidase [EC:2.3.2.2] | ec:2.3.2.2 |
| bam:Bamb\_2727 | DeoR family transcriptional regulator; K02444 DeoR family transcriptional regulator, glycerol-3-phosphate regulon repressor |  |
| bam:Bamb\_2728 | hypothetical protein |  |
| bam:Bamb\_2729 | hypothetical protein |  |
| bam:Bamb\_2730 | glpD; glycerol-3-phosphate dehydrogenase (EC:1.1.5.3); K00111 glycerol-3-phosphate dehydrogenase [EC:1.1.5.3] | ec:1.1.5.3 |
| bam:Bamb\_2731 | glpK; glycerol kinase (EC:2.7.1.30); K00864 glycerol kinase [EC:2.7.1.30] | ec:2.7.1.30 |
| bam:Bamb\_2732 | MIP family channel protein; K02440 glycerol uptake facilitator protein |  |
| bam:Bamb\_2733 | FAD-dependent pyridine nucleotide-disulfide oxidoreductase; K00529 ferredoxin--NAD+ reductase [EC:1.18.1.3] | ec:1.18.1.3 |
| bam:Bamb\_2734 | Rieske (2Fe-2S) domain-containing protein; K05710 dioxygenase ferredoxin subunit |  |
| bam:Bamb\_2735 | fatty acid desaturase |  |
| bam:Bamb\_2736 | LacI family transcriptional regulator; K02529 LacI family transcriptional regulator |  |
| bam:Bamb\_2737 | hypothetical protein |  |

  
**Neighborhood Representations for "bcm:Bcenmc03\_2703"**  

| ID | Annotation | EC number |
| --- | --- | --- |
| bcm:Bcenmc03\_2693 | hypothetical protein; K09984 hypothetical protein |  |
| bcm:Bcenmc03\_2694 | GntR family transcriptional regulator |  |
| bcm:Bcenmc03\_2695 | galactonate dehydratase; K01684 galactonate dehydratase [EC:4.2.1.6] | ec:4.2.1.6 |
| bcm:Bcenmc03\_2696 | copper resistance D domain-containing protein; K07245 putative copper resistance protein D |  |
| bcm:Bcenmc03\_2697 | cytochrome c, class I |  |
| bcm:Bcenmc03\_2698 | gluconate 2-dehydrogenase (acceptor) (EC:1.1.99.3) |  |
| bcm:Bcenmc03\_2699 | DEAD/DEAH box helicase; K11927 ATP-dependent RNA helicase RhlE [EC:3.6.4.13] | ec:3.6.4.13 |
| bcm:Bcenmc03\_2700 | hypothetical protein |  |
| bcm:Bcenmc03\_2701 | hypothetical protein |  |
| bcm:Bcenmc03\_2702 | gamma-glutamyltransferase (EC:2.3.2.2); K00681 gamma-glutamyltranspeptidase [EC:2.3.2.2] | ec:2.3.2.2 |
| bcm:Bcenmc03\_2703 | DeoR family transcriptional regulator; K02444 DeoR family transcriptional regulator, glycerol-3-phosphate regulon repressor |  |
| bcm:Bcenmc03\_2704 | hypothetical protein |  |
| bcm:Bcenmc03\_2705 | hypothetical protein |  |
| bcm:Bcenmc03\_2706 | glpD; glycerol-3-phosphate dehydrogenase; K00111 glycerol-3-phosphate dehydrogenase [EC:1.1.5.3] | ec:1.1.5.3 |
| bcm:Bcenmc03\_2707 | glpK; glycerol kinase; K00864 glycerol kinase [EC:2.7.1.30] | ec:2.7.1.30 |
| bcm:Bcenmc03\_2708 | MIP family channel protein; K02440 glycerol uptake facilitator protein |  |
| bcm:Bcenmc03\_2709 | FAD-dependent pyridine nucleotide-disulfide oxidoreductase; K00529 ferredoxin--NAD+ reductase [EC:1.18.1.3] | ec:1.18.1.3 |
| bcm:Bcenmc03\_2710 | Rieske (2Fe-2S) domain-containing protein; K05710 dioxygenase ferredoxin subunit |  |
| bcm:Bcenmc03\_2711 | fatty acid desaturase |  |
| bcm:Bcenmc03\_2712 | LacI family transcriptional regulator; K02529 LacI family transcriptional regulator |  |
| bcm:Bcenmc03\_2713 | xylose isomerase domain-containing protein |  |

  
**Neighborhood Representations for "brh:RBRH\_01996"**  

| ID | Annotation | EC number |
| --- | --- | --- |
| brh:RBRH\_01986 | manganese catalase (EC:1.11.1.6); K07217 Mn-containing catalase |  |
| brh:RBRH\_01987 | Purine/pyrimidine phosphoribosyl transferase (EC:2.4.2.-); K07100 |  |
| brh:RBRH\_04100 | hypothetical protein |  |
| brh:RBRH\_01988 | hypothetical protein |  |
| brh:RBRH\_01989 | hypothetical protein |  |
| brh:RBRH\_01990 | cytochrome c4 |  |
| brh:RBRH\_01991 | cytochrome c |  |
| brh:RBRH\_01992 | ATP-dependent RNA helicase; K11927 ATP-dependent RNA helicase RhlE [EC:3.6.4.13] | ec:3.6.4.13 |
| brh:RBRH\_01993 | Vanillate decarboxylase VdcD protein (EC:4.1.1.-) |  |
| brh:RBRH\_01994 | hypothetical protein |  |
| brh:RBRH\_01996 | glycerol-3-phosphate regulon repressor; K02444 DeoR family transcriptional regulator, glycerol-3-phosphate regulon repressor |  |
| brh:RBRH\_01998 | ferric siderophore receptor; K02014 iron complex outermembrane recepter protein |  |
| brh:RBRH\_01999 | glycerol-3-phosphate dehydrogenase (EC:1.1.5.3); K00111 glycerol-3-phosphate dehydrogenase [EC:1.1.5.3] | ec:1.1.5.3 |
| brh:RBRH\_02000 | glycerol kinase (EC:2.7.1.30) |  |
| brh:RBRH\_04099 | hypothetical protein |  |
| brh:RBRH\_02002 | 3,4-dihydroxy-2-butanone 4-phosphate synthase (EC:4.1.99.12); K02858 3,4-dihydroxy 2-butanone 4-phosphate synthase [EC:4.1.99.12] | ec:4.1.99.12 |
| brh:RBRH\_02003 | hypothetical protein |  |
| brh:RBRH\_02004 | tRNA 2-methylthioadenosine synthase; K06168 bifunctional enzyme involved in thiolation and methylation of tRNA |  |
| brh:RBRH\_04098 | tRNA 2-methylthioadenosine synthase |  |
| brh:RBRH\_02005 | PhoH protein; K06217 phosphate starvation-inducible protein PhoH and related proteins |  |
| brh:RBRH\_02006 | hypothetical protein; K07042 probable rRNA maturation factor |  |

  
**Neighborhood Representations for "cvi:CV\_0136"**  

| ID | Annotation | EC number |
| --- | --- | --- |
| cvi:CV\_0126 | hypothetical protein; K07491 putative transposase |  |
| cvi:CV\_0127 | cytidine deaminase (EC:3.5.4.5); K01489 cytidine deaminase [EC:3.5.4.5] | ec:3.5.4.5 |
| cvi:CV\_0128 | hypothetical protein |  |
| cvi:CV\_0129 | hypothetical protein |  |
| cvi:CV\_0130 | Xaa-Pro aminopeptidase (EC:3.4.11.9); K01262 Xaa-Pro aminopeptidase [EC:3.4.11.9] | ec:3.4.11.9 |
| cvi:CV\_0131 | benzoate membrane transport protein; K05782 benzoate membrane transport protein |  |
| cvi:CV\_0132 | hypothetical protein |  |
| cvi:CV\_0133 | transcriptional regulator |  |
| cvi:CV\_0134 | acetyltransferase |  |
| cvi:CV\_0135 | hypothetical protein |  |
| cvi:CV\_0136 | glpR2; glycerol-3-phosphate regulon repressor; K02444 DeoR family transcriptional regulator, glycerol-3-phosphate regulon repressor |  |
| cvi:CV\_0137 | hypothetical protein |  |
| cvi:CV\_0138 | hypothetical protein |  |
| cvi:CV\_0139 | hypothetical protein |  |
| cvi:CV\_0140 | hypothetical protein |  |
| cvi:CV\_0141 | transmembrane efflux protein |  |
| cvi:CV\_0142 | transcriptional regulator |  |
| cvi:CV\_0143 | eda; 2-dehydro-3-deoxyphosphooctonate aldolase (EC:4.1.2.14 4.1.3.16); K01625 2-dehydro-3-deoxyphosphogluconate aldolase / 4-hydroxy-2-oxoglutarate aldolase [EC:4.1.2.14 4.1.3.16] | ec:4.1.3.16 ec:4.1.2.14 |
| cvi:CV\_0144 | edd; phosphogluconate dehydratase (EC:4.2.1.12); K01690 phosphogluconate dehydratase [EC:4.2.1.12] | ec:4.2.1.12 |
| cvi:CV\_0145 | zwf; glucose-6-phosphate 1-dehydrogenase (EC:1.1.1.49); K00036 glucose-6-phosphate 1-dehydrogenase [EC:1.1.1.49] | ec:1.1.1.49 |
| cvi:CV\_0146 | pgl; 6-phosphogluconolactonase (EC:3.1.1.31); K01057 6-phosphogluconolactonase [EC:3.1.1.31] | ec:3.1.1.31 |

  
**Neighborhood Representations for "xcv:XCV0375"**  

| ID | Annotation | EC number |
| --- | --- | --- |
| xcv:XCV0365 | hypothetical protein |  |
| xcv:XCV0366 | 3-alpha-hydroxysteroid dehydrogenase (EC:1.1.1.50) |  |
| xcv:XCV0367 | aldehyde dehydrogenase; K00141 benzaldehyde dehydrogenase (NAD) [EC:1.2.1.28] | ec:1.2.1.28 |
| xcv:XCV0368 | pobR; transcriptional regulator PobR |  |
| xcv:XCV0369 | pobA; 4-hydroxybenzoate 3-monooxygenase (EC:1.14.13.2); K00481 p-hydroxybenzoate 3-monooxygenase [EC:1.14.13.2] | ec:1.14.13.2 |
| xcv:XCV0370 | hypothetical protein |  |
| xcv:XCV0371 | hypothetical protein |  |
| xcv:XCV0372 | glpK; glycerol kinase (EC:2.7.1.30); K00864 glycerol kinase [EC:2.7.1.30] | ec:2.7.1.30 |
| xcv:XCV0373 | glpF; glycerol uptake facilitator protein; K02440 glycerol uptake facilitator protein |  |
| xcv:XCV0374 | glpD; glycerol-3-phosphate dehydrogenase (EC:1.1.5.3); K00111 glycerol-3-phosphate dehydrogenase [EC:1.1.5.3] | ec:1.1.5.3 |
| xcv:XCV0375 | glpR; DeoR family transcriptional regulator; K02444 DeoR family transcriptional regulator, glycerol-3-phosphate regulon repressor |  |
| xcv:XCV0376 | vanillate o-demethylase oxidoreductase; K03863 vanillate monooxygenase [EC:1.14.13.82] | ec:1.14.13.82 |
| xcv:XCV0377 | vanillate O-demethylase oxygenase subunit (EC:1.14.13.82); K03862 vanillate monooxygenase [EC:1.14.13.82] | ec:1.14.13.82 |
| xcv:XCV0378 | gctA; glutaconate CoA transferase subunit A; K01039 glutaconate CoA-transferase, subunit A [EC:2.8.3.12] | ec:2.8.3.12 |
| xcv:XCV0379 | gctB; glutaconate CoA transferase subunit B; K01040 glutaconate CoA-transferase, subunit B [EC:2.8.3.12] | ec:2.8.3.12 |
| xcv:XCV0380 | pcaF; beta-ketoadipyl CoA thiolase (EC:2.3.1.16); K07823 3-oxoadipyl-CoA thiolase [EC:2.3.1.174] | ec:2.3.1.174 |
| xcv:XCV0381 | pcaH; protocatechuate 3,4-dioxygenase subunit beta (EC:1.13.11.3); K00449 protocatechuate 3,4-dioxygenase, beta subunit [EC:1.13.11.3] | ec:1.13.11.3 |
| xcv:XCV0382 | pcaG; protocatechuate 3,4-dioxygenase alpha chain (EC:1.13.11.3); K00448 protocatechuate 3,4-dioxygenase, alpha subunit [EC:1.13.11.3] | ec:1.13.11.3 |
| xcv:XCV0383 | pcaB; 3-carboxy-cis,cis-muconate cycloisomerase (EC:5.5.1.2); K01857 3-carboxy-cis,cis-muconate cycloisomerase [EC:5.5.1.2] | ec:5.5.1.2 |
| xcv:XCV0384 | 3-oxoadipate enol-lactonase (EC:3.1.1.24); K01055 3-oxoadipate enol-lactonase [EC:3.1.1.24] | ec:3.1.1.24 |
| xcv:XCV0385 | pcaC; 4-carboxymuconolactone decarboxylase (EC:4.1.1.44); K01607 4-carboxymuconolactone decarboxylase [EC:4.1.1.44] | ec:4.1.1.44 |

  
**Neighborhood Representations for "xac:XAC0361"**  

| ID | Annotation | EC number |
| --- | --- | --- |
| xac:XAC0351 | MarR family transcriptional regulator |  |
| xac:XAC0352 | hypothetical protein |  |
| xac:XAC0353 | drb0080; 3-alpha-hydroxysteroid dehydrogenase (EC:1.1.1.50) |  |
| xac:XAC0354 | xylC; benzaldehyde dehydrogenase II; K00141 benzaldehyde dehydrogenase (NAD) [EC:1.2.1.28] | ec:1.2.1.28 |
| xac:XAC0355 | pobR; PobR regulator |  |
| xac:XAC0356 | pobA; 4-hydroxybenzoate 3-monooxygenase (EC:1.14.13.2); K00481 p-hydroxybenzoate 3-monooxygenase [EC:1.14.13.2] | ec:1.14.13.2 |
| xac:XAC0357 | hypothetical protein |  |
| xac:XAC0358 | glpK; glycerol kinase (EC:2.7.1.30); K00864 glycerol kinase [EC:2.7.1.30] | ec:2.7.1.30 |
| xac:XAC0359 | glpF; glycerol uptake facilitator protein; K02440 glycerol uptake facilitator protein |  |
| xac:XAC0360 | glpD; glycerol-3-phosphate dehydrogenase (EC:1.1.5.3); K00111 glycerol-3-phosphate dehydrogenase [EC:1.1.5.3] | ec:1.1.5.3 |
| xac:XAC0361 | glpR; glycerol-3-phosphate regulon repressor; K02444 DeoR family transcriptional regulator, glycerol-3-phosphate regulon repressor |  |
| xac:XAC0362 | pobB; phenoxybenzoate dioxygenase subunit beta; K03863 vanillate monooxygenase [EC:1.14.13.82] | ec:1.14.13.82 |
| xac:XAC0363 | vanA; vanillate O-demethylase oxygenase; K03862 vanillate monooxygenase [EC:1.14.13.82] | ec:1.14.13.82 |
| xac:XAC0364 | gctA; glutaconate CoA transferase subunit A; K01039 glutaconate CoA-transferase, subunit A [EC:2.8.3.12] | ec:2.8.3.12 |
| xac:XAC0365 | gctB; glutaconate CoA transferase subunit B; K01040 glutaconate CoA-transferase, subunit B [EC:2.8.3.12] | ec:2.8.3.12 |
| xac:XAC0366 | pcaF; beta-ketoadipyl CoA thiolase; K07823 3-oxoadipyl-CoA thiolase [EC:2.3.1.174] | ec:2.3.1.174 |
| xac:XAC0367 | pcaH; protocatechuate 3,4-dioxygenase subunit beta; K00449 protocatechuate 3,4-dioxygenase, beta subunit [EC:1.13.11.3] | ec:1.13.11.3 |
| xac:XAC0368 | pcaG; protocatechuate 3,4-dioxygenase subunit alpha; K00448 protocatechuate 3,4-dioxygenase, alpha subunit [EC:1.13.11.3] | ec:1.13.11.3 |
| xac:XAC0369 | pcaB; 3-carboxy-cis,cis-muconate cycloisomerase (EC:5.5.1.2); K01857 3-carboxy-cis,cis-muconate cycloisomerase [EC:5.5.1.2] | ec:5.5.1.2 |
| xac:XAC0370 | catD; b-ketoadipate enol-lactone hydrolase; K01055 3-oxoadipate enol-lactonase [EC:3.1.1.24] | ec:3.1.1.24 |
| xac:XAC0371 | pcaC; 4-carboxymuconolactone decarboxylase; K01607 4-carboxymuconolactone decarboxylase [EC:4.1.1.44] | ec:4.1.1.44 |

  
**Neighborhood Representations for "xca:xccb100\_0387"**  

| ID | Annotation | EC number |
| --- | --- | --- |
| xca:xccb100\_0377 | transcriptional regulator, MarR family |  |
| xca:xccb100\_0378 | hypothetical protein |  |
| xca:xccb100\_0379 | 3-alpha-hydroxysteroid dehydrogenase |  |
| xca:xccb100\_0380 | benzaldehyde dehydrogenase (NAD(+)) (EC:1.2.1.28); K00141 benzaldehyde dehydrogenase (NAD) [EC:1.2.1.28] | ec:1.2.1.28 |
| xca:xccb100\_0381 | AraC family transcriptional regulator |  |
| xca:xccb100\_0382 | 4-hydroxybenzoate 3-monooxygenase; K00481 p-hydroxybenzoate 3-monooxygenase [EC:1.14.13.2] | ec:1.14.13.2 |
| xca:xccb100\_0383 | hypothetical protein |  |
| xca:xccb100\_0384 | glpK; glycerol kinase; K00864 glycerol kinase [EC:2.7.1.30] | ec:2.7.1.30 |
| xca:xccb100\_0385 | glpF; glycerol uptake facilitator; K02440 glycerol uptake facilitator protein |  |
| xca:xccb100\_0386 | glpD; glycerol-3-phosphate dehydrogenase; K00111 glycerol-3-phosphate dehydrogenase [EC:1.1.5.3] | ec:1.1.5.3 |
| xca:xccb100\_0387 | glpR; glycerol-3-phosphate regulon repressor; K02444 DeoR family transcriptional regulator, glycerol-3-phosphate regulon repressor |  |
| xca:xccb100\_0388 | oxygenase subunit (EC:1.14.-.-); K03863 vanillate monooxygenase [EC:1.14.13.82] | ec:1.14.13.82 |
| xca:xccb100\_0389 | oxygenase subunit (EC:1.14.13.82); K03862 vanillate monooxygenase [EC:1.14.13.82] | ec:1.14.13.82 |
| xca:xccb100\_0390 | gctA; glutaconate CoA transferase subunit A (EC:2.8.3.12); K01039 glutaconate CoA-transferase, subunit A [EC:2.8.3.12] | ec:2.8.3.12 |
| xca:xccb100\_0391 | gctB; glutaconate CoA transferase subunit B (EC:2.8.3.12); K01040 glutaconate CoA-transferase, subunit B [EC:2.8.3.12] | ec:2.8.3.12 |
| xca:xccb100\_0392 | pcaF; beta-ketoadipyl CoA thiolase (EC:2.3.1.16); K07823 3-oxoadipyl-CoA thiolase [EC:2.3.1.174] | ec:2.3.1.174 |
| xca:xccb100\_0393 | pcaH; protocatechuate 3,4-dioxygenase subunit beta (EC:1.13.11.3); K00449 protocatechuate 3,4-dioxygenase, beta subunit [EC:1.13.11.3] | ec:1.13.11.3 |
| xca:xccb100\_0394 | pcaG; protocatechuate 3,4-dioxygenase alpha chain (EC:1.13.11.3); K00448 protocatechuate 3,4-dioxygenase, alpha subunit [EC:1.13.11.3] | ec:1.13.11.3 |
| xca:xccb100\_0395 | pcaB; 3-carboxy-cis,cis-muconate cycloisomerase (EC:5.5.1.2); K01857 3-carboxy-cis,cis-muconate cycloisomerase [EC:5.5.1.2] | ec:5.5.1.2 |
| xca:xccb100\_0396 | pcaD; 3-oxoadipate enol-lactonase (EC:3.1.1.24); K01055 3-oxoadipate enol-lactonase [EC:3.1.1.24] | ec:3.1.1.24 |
| xca:xccb100\_0397 | pcaC; 4-carboxymuconolactone decarboxylase (EC:4.1.1.44); K01607 4-carboxymuconolactone decarboxylase [EC:4.1.1.44] | ec:4.1.1.44 |

  
**Neighborhood Representations for "xcb:XC\_0373"**  

| ID | Annotation | EC number |
| --- | --- | --- |
| xcb:XC\_0363 | MarR family transcriptional regulator |  |
| xcb:XC\_0364 | hypothetical protein |  |
| xcb:XC\_0365 | 3-alpha-hydroxysteroid dehydrogenase (EC:1.1.1.50) |  |
| xcb:XC\_0366 | benzaldehyde dehydrogenase II; K00141 benzaldehyde dehydrogenase (NAD) [EC:1.2.1.28] | ec:1.2.1.28 |
| xcb:XC\_0367 | PobR regulator |  |
| xcb:XC\_0368 | 4-hydroxybenzoate 3-monooxygenase (EC:1.14.13.2); K00481 p-hydroxybenzoate 3-monooxygenase [EC:1.14.13.2] | ec:1.14.13.2 |
| xcb:XC\_0369 | hypothetical protein |  |
| xcb:XC\_0370 | glpK; glycerol kinase (EC:2.7.1.30); K00864 glycerol kinase [EC:2.7.1.30] | ec:2.7.1.30 |
| xcb:XC\_0371 | glycerol uptake facilitator protein; K02440 glycerol uptake facilitator protein |  |
| xcb:XC\_0372 | glpD; glycerol-3-phosphate dehydrogenase (EC:1.1.5.3); K00111 glycerol-3-phosphate dehydrogenase [EC:1.1.5.3] | ec:1.1.5.3 |
| xcb:XC\_0373 | glycerol-3-phosphate regulon repressor; K02444 DeoR family transcriptional regulator, glycerol-3-phosphate regulon repressor |  |
| xcb:XC\_0374 | phenoxybenzoate dioxygenase subunit beta; K03863 vanillate monooxygenase [EC:1.14.13.82] | ec:1.14.13.82 |
| xcb:XC\_0375 | vanillate O-demethylase oxygenase; K03862 vanillate monooxygenase [EC:1.14.13.82] | ec:1.14.13.82 |
| xcb:XC\_0376 | glutaconate CoA transferase subunit A; K01039 glutaconate CoA-transferase, subunit A [EC:2.8.3.12] | ec:2.8.3.12 |
| xcb:XC\_0377 | glutaconate CoA transferase subunit B; K01040 glutaconate CoA-transferase, subunit B [EC:2.8.3.12] | ec:2.8.3.12 |
| xcb:XC\_0378 | beta-ketoadipyl CoA thiolase; K07823 3-oxoadipyl-CoA thiolase [EC:2.3.1.174] | ec:2.3.1.174 |
| xcb:XC\_0379 | protocatechuate 3,4-dioxygenase subunit beta; K00449 protocatechuate 3,4-dioxygenase, beta subunit [EC:1.13.11.3] | ec:1.13.11.3 |
| xcb:XC\_0380 | protocatechuate 3,4-dioxygenase alpha chain; K00448 protocatechuate 3,4-dioxygenase, alpha subunit [EC:1.13.11.3] | ec:1.13.11.3 |
| xcb:XC\_0381 | 3-carboxy-cis,cis-muconate cycloisomerase (EC:5.5.1.2); K01857 3-carboxy-cis,cis-muconate cycloisomerase [EC:5.5.1.2] | ec:5.5.1.2 |
| xcb:XC\_0382 | b-ketoadipate enol-lactone hydrolase; K01055 3-oxoadipate enol-lactonase [EC:3.1.1.24] | ec:3.1.1.24 |
| xcb:XC\_0383 | 4-carboxymuconolactone decarboxylase; K01607 4-carboxymuconolactone decarboxylase [EC:4.1.1.44] | ec:4.1.1.44 |

  
**Neighborhood Representations for "xcc:XCC0361"**  

| ID | Annotation | EC number |
| --- | --- | --- |
| xcc:XCC0351 | MarR family transcriptional regulator |  |
| xcc:XCC0352 | hypothetical protein |  |
| xcc:XCC0353 | drb0080; 3-alpha-hydroxysteroid dehydrogenase (EC:1.1.1.50) |  |
| xcc:XCC0354 | xylC; benzaldehyde dehydrogenase II; K00141 benzaldehyde dehydrogenase (NAD) [EC:1.2.1.28] | ec:1.2.1.28 |
| xcc:XCC0355 | pobR; PobR regulator |  |
| xcc:XCC0356 | pobA; 4-hydroxybenzoate 3-monooxygenase (EC:1.14.13.2); K00481 p-hydroxybenzoate 3-monooxygenase [EC:1.14.13.2] | ec:1.14.13.2 |
| xcc:XCC0357 | hypothetical protein |  |
| xcc:XCC0358 | glpK; glycerol kinase (EC:2.7.1.30); K00864 glycerol kinase [EC:2.7.1.30] | ec:2.7.1.30 |
| xcc:XCC0359 | glpF; glycerol uptake facilitator protein; K02440 glycerol uptake facilitator protein |  |
| xcc:XCC0360 | glpD; glycerol-3-phosphate dehydrogenase (EC:1.1.5.3); K00111 glycerol-3-phosphate dehydrogenase [EC:1.1.5.3] | ec:1.1.5.3 |
| xcc:XCC0361 | glpR; glycerol-3-phosphate regulon repressor; K02444 DeoR family transcriptional regulator, glycerol-3-phosphate regulon repressor |  |
| xcc:XCC0362 | pobB; phenoxybenzoate dioxygenase subunit beta; K03863 vanillate monooxygenase [EC:1.14.13.82] | ec:1.14.13.82 |
| xcc:XCC0363 | vanA; vanillate O-demethylase oxygenase; K03862 vanillate monooxygenase [EC:1.14.13.82] | ec:1.14.13.82 |
| xcc:XCC0364 | gctA; glutaconate CoA transferase subunit A; K01039 glutaconate CoA-transferase, subunit A [EC:2.8.3.12] | ec:2.8.3.12 |
| xcc:XCC0365 | gctB; glutaconate CoA transferase subunit B; K01040 glutaconate CoA-transferase, subunit B [EC:2.8.3.12] | ec:2.8.3.12 |
| xcc:XCC0366 | pcaF; beta-ketoadipyl CoA thiolase; K07823 3-oxoadipyl-CoA thiolase [EC:2.3.1.174] | ec:2.3.1.174 |
| xcc:XCC0367 | pcaH; protocatechuate 3,4-dioxygenase subunit beta; K00449 protocatechuate 3,4-dioxygenase, beta subunit [EC:1.13.11.3] | ec:1.13.11.3 |
| xcc:XCC0368 | pcaG; protocatechuate 3,4-dioxygenase alpha chain; K00448 protocatechuate 3,4-dioxygenase, alpha subunit [EC:1.13.11.3] | ec:1.13.11.3 |
| xcc:XCC0369 | pcaB; 3-carboxy-cis,cis-muconate cycloisomerase (EC:5.5.1.2); K01857 3-carboxy-cis,cis-muconate cycloisomerase [EC:5.5.1.2] | ec:5.5.1.2 |
| xcc:XCC0370 | catD; b-ketoadipate enol-lactone hydrolase; K01055 3-oxoadipate enol-lactonase [EC:3.1.1.24] | ec:3.1.1.24 |
| xcc:XCC0371 | pcaC; 4-carboxymuconolactone decarboxylase; K01607 4-carboxymuconolactone decarboxylase [EC:4.1.1.44] | ec:4.1.1.44 |

  
**Neighborhood Representations for "bid:Bind\_0377"**  

| ID | Annotation | EC number |
| --- | --- | --- |
| bid:Bind\_0367 | transposase |  |
| bid:Bind\_0368 | N-acetyltransferase GCN5 |  |
| bid:Bind\_0369 | TonB-dependent receptor; K02014 iron complex outermembrane recepter protein |  |
| bid:Bind\_0370 | pepF/M3 family oligoendopeptidase; K08602 oligoendopeptidase F [EC:3.4.24.-] |  |
| bid:Bind\_0371 | alcohol dehydrogenase |  |
| bid:Bind\_0372 | DEAD/DEAH box helicase; K03724 ATP-dependent helicase Lhr and Lhr-like helicase [EC:3.6.4.-] |  |
| bid:Bind\_0373 | hypothetical protein; K06953 |  |
| bid:Bind\_0374 | hypothetical protein |  |
| bid:Bind\_0375 | pseudogene |  |
| bid:Bind\_0376 | histone family protein DNA-binding protein; K04764 integration host factor subunit alpha |  |
| bid:Bind\_0377 | DeoR family transcriptional regulator; K02444 DeoR family transcriptional regulator, glycerol-3-phosphate regulon repressor |  |
| bid:Bind\_0378 | FAD dependent oxidoreductase; K00111 glycerol-3-phosphate dehydrogenase [EC:1.1.5.3] | ec:1.1.5.3 |
| bid:Bind\_0379 | MIP family channel protein; K02440 glycerol uptake facilitator protein |  |
| bid:Bind\_0380 | glycerol kinase; K00864 glycerol kinase [EC:2.7.1.30] | ec:2.7.1.30 |
| bid:Bind\_0381 | Ku protein; K10979 DNA end-binding protein Ku |  |
| bid:Bind\_0382 | DNA ligase D; K01971 DNA ligase (ATP) [EC:6.5.1.1] | ec:6.5.1.1 |
| bid:Bind\_0383 | Ku protein; K10979 DNA end-binding protein Ku |  |
| bid:Bind\_0384 | filamentation induced by cAMP protein fic; K04095 cell filamentation protein |  |
| bid:Bind\_0385 | hypothetical protein |  |
| bid:Bind\_0386 | cob(I)alamin adenosyltransferase (EC:2.5.1.17); K00798 cob(I)alamin adenosyltransferase [EC:2.5.1.17] | ec:2.5.1.17 |
| bid:Bind\_0387 | cobyric acid synthase CobQ; K02232 adenosylcobyric acid synthase [EC:6.3.5.10] | ec:6.3.5.10 |

  
**Neighborhood Representations for "bpt:Bpet2588"**  

| ID | Annotation | EC number |
| --- | --- | --- |
| bpt:Bpet2578 | D-amino acid dehydrogenase small subunit (EC:1.4.99.1); K00285 D-amino-acid dehydrogenase [EC:1.4.99.1] | ec:1.4.99.1 |
| bpt:Bpet2579 | AsnC family transcriptional regulator; K03719 Lrp/AsnC family transcriptional regulator, leucine-responsive regulatory protein |  |
| bpt:Bpet2580 | hypothetical protein |  |
| bpt:Bpet2581 | lipoprotein |  |
| bpt:Bpet2582 | sugar ABC transporter substrate-binding protein; K17321 glycerol transport system substrate-binding protein |  |
| bpt:Bpet2583 | hypothetical protein |  |
| bpt:Bpet2584 | hypothetical protein; K17323 glycerol transport system permease protein |  |
| bpt:Bpet2585 | ABC transporter inner membrane protein; K17322 glycerol transport system permease protein |  |
| bpt:Bpet2586 | sugar ABC transporter ATP-binding protein; K17325 glycerol transport system ATP-binding protein |  |
| bpt:Bpet2587 | ABC transporter ATP-binding protein; K17324 glycerol transport system ATP-binding protein |  |
| bpt:Bpet2588 | glpR; hypothetical protein; K02444 DeoR family transcriptional regulator, glycerol-3-phosphate regulon repressor |  |
| bpt:Bpet2589 | glpA; hypothetical protein; K00111 glycerol-3-phosphate dehydrogenase [EC:1.1.5.3] | ec:1.1.5.3 |
| bpt:Bpet2590 | TetR family transcriptional regulator |  |
| bpt:Bpet2591 | hlyD; HlyD family secretion protein; K01993 HlyD family secretion protein |  |
| bpt:Bpet2592 | ATP-binding component of a transport system; K01990 ABC-2 type transport system ATP-binding protein |  |
| bpt:Bpet2593 | multidrug ABC transporter permease; K01992 ABC-2 type transport system permease protein |  |
| bpt:Bpet2594 | outer membrane exporter protein |  |
| bpt:Bpet2595 | hypothetical protein |  |
| bpt:Bpet2596 | Ser/Thr-rich protein T10 |  |
| bpt:Bpet2597 | hypothetical protein |  |
| bpt:Bpet2598 | hypothetical protein; K07052 |  |

  
**Neighborhood Representations for "dsa:Desal\_3154"**  

| ID | Annotation | EC number |
| --- | --- | --- |
| dsa:Desal\_3144 | hypothetical protein |  |
| dsa:Desal\_3145 | hypothetical protein |  |
| dsa:Desal\_3146 | PAS/PAC sensor signal transduction histidine kinase |  |
| dsa:Desal\_3147 | PAS/PAC sensor signal transduction histidine kinase |  |
| dsa:Desal\_3148 | extracellular ligand-binding receptor; K01999 branched-chain amino acid transport system substrate-binding protein |  |
| dsa:Desal\_3149 | hypothetical protein |  |
| dsa:Desal\_3150 | signal transduction histidine kinase, nitrogen specific, NtrB |  |
| dsa:Desal\_3151 | glycerol kinase; K00864 glycerol kinase [EC:2.7.1.30] | ec:2.7.1.30 |
| dsa:Desal\_3152 | MIP family channel protein; K02440 glycerol uptake facilitator protein |  |
| dsa:Desal\_3153 | FAD dependent oxidoreductase; K00111 glycerol-3-phosphate dehydrogenase [EC:1.1.5.3] | ec:1.1.5.3 |
| dsa:Desal\_3154 | DeoR family transcriptional regulator; K02444 DeoR family transcriptional regulator, glycerol-3-phosphate regulon repressor |  |
| dsa:Desal\_3155 | radical SAM domain protein |  |
| dsa:Desal\_3156 | hypothetical protein |  |
| dsa:Desal\_3157 | hypothetical protein |  |
| dsa:Desal\_3158 | hypothetical protein |  |
| dsa:Desal\_3159 | DSBA oxidoreductase |  |
| dsa:Desal\_3160 | glycerophosphoryl diester phosphodiesterase; K01126 glycerophosphoryl diester phosphodiesterase [EC:3.1.4.46] | ec:3.1.4.46 |
| dsa:Desal\_3161 | iron-containing alcohol dehydrogenase |  |
| dsa:Desal\_3162 | signal transduction protein with EFhand domain-containing protein |  |
| dsa:Desal\_3163 | hypothetical protein |  |
| dsa:Desal\_3164 | hypothetical protein |  |

  
**Neighborhood Representations for "vei:Veis\_3832"**  

| ID | Annotation | EC number |
| --- | --- | --- |
| vei:Veis\_3822 | fumarylacetoacetate (FAA) hydrolase; K16165 fumarylpyruvate hydrolase [EC:3.7.1.20] | ec:3.7.1.20 |
| vei:Veis\_3823 | NUDIX hydrolase |  |
| vei:Veis\_3824 | fimbrial protein pilin; K02655 type IV pilus assembly protein PilE |  |
| vei:Veis\_3825 | Tfp pilus assembly protein tip-associated adhesin PilY1-like protein; K02674 type IV pilus assembly protein PilY1 |  |
| vei:Veis\_3826 | Tfp pilus assembly protein PilX-like protein; K02673 type IV pilus assembly protein PilX |  |
| vei:Veis\_3827 | hypothetical protein |  |
| vei:Veis\_3828 | type IV pilus modification protein PilV; K02671 type IV pilus assembly protein PilV |  |
| vei:Veis\_3829 | Tfp pilus assembly protein FimT-like protein; K08084 type IV fimbrial biogenesis protein FimT |  |
| vei:Veis\_3830 | hypothetical protein |  |
| vei:Veis\_3831 | hypothetical protein |  |
| vei:Veis\_3832 | DeoR family transcriptional regulator; K02444 DeoR family transcriptional regulator, glycerol-3-phosphate regulon repressor |  |
| vei:Veis\_3833 | hypothetical protein |  |
| vei:Veis\_3834 | ABC transporter-like protein |  |
| vei:Veis\_3835 | ABC transporter-like protein; K01996 branched-chain amino acid transport system ATP-binding protein |  |
| vei:Veis\_3836 | extracellular ligand-binding receptor |  |
| vei:Veis\_3837 | inner-membrane translocator |  |
| vei:Veis\_3838 | inner-membrane translocator |  |
| vei:Veis\_3839 | AMP-dependent synthetase and ligase; K01897 long-chain acyl-CoA synthetase [EC:6.2.1.3] | ec:6.2.1.3 |
| vei:Veis\_3840 | hypothetical protein |  |
| vei:Veis\_3841 | enoyl-CoA hydratase (EC:4.2.1.17) |  |
| vei:Veis\_3842 | hypothetical protein |  |

  
**Neighborhood Representations for "psa:PST\_1602"**  

| ID | Annotation | EC number |
| --- | --- | --- |
| psa:PST\_1592 | oxidoreductase |  |
| psa:PST\_1593 | inositol-1-monophosphatase; K01092 myo-inositol-1(or 4)-monophosphatase [EC:3.1.3.25] | ec:3.1.3.25 |
| psa:PST\_1594 | hypothetical protein |  |
| psa:PST\_1595 | ribosomal subunit interface protein |  |
| psa:PST\_1596 | hypothetical protein |  |
| psa:PST\_1597 | methyl-accepting chemotaxis transducer |  |
| psa:PST\_1598 | peptidyl-prolyl cis-trans isomerase C; K03769 peptidyl-prolyl cis-trans isomerase C [EC:5.2.1.8] | ec:5.2.1.8 |
| psa:PST\_1599 | phosphate ABC transporter substrate-binding protein; K02040 phosphate transport system substrate-binding protein |  |
| psa:PST\_1600 | Na+/Pi-cotransporter; K14683 solute carrier family 34 (sodium-dependent phosphate cotransporter) |  |
| psa:PST\_1601 | glpD; glycerol-3-phosphate dehydrogenase (EC:1.1.5.3); K00111 glycerol-3-phosphate dehydrogenase [EC:1.1.5.3] | ec:1.1.5.3 |
| psa:PST\_1602 | glpR; glycerol-3-phosphate regulon repressor; K02444 DeoR family transcriptional regulator, glycerol-3-phosphate regulon repressor |  |
| psa:PST\_1603 | glpK; glycerol kinase; K00864 glycerol kinase [EC:2.7.1.30] | ec:2.7.1.30 |
| psa:PST\_1604 | glpF; glycerol uptake facilitator protein; K02440 glycerol uptake facilitator protein |  |
| psa:PST\_1605 | hypothetical protein |  |
| psa:PST\_1606 | ATP-dependent RNA helicase; K17675 ATP-dependent RNA helicase SUPV3L1/SUV3 [EC:3.6.4.13] | ec:3.6.4.13 |
| psa:PST\_1607 | ion transporter; K10716 voltage-gated potassium channel |  |
| psa:PST\_1608 | hypothetical protein |  |
| psa:PST\_1609 | hypothetical protein |  |
| psa:PST\_1610 | flavin monoamine oxidase-related protein; K00274 monoamine oxidase [EC:1.4.3.4] | ec:1.4.3.4 |
| psa:PST\_1611 | cytochrome C6 |  |
| psa:PST\_1612 | putative endoribonuclease L-PSP; K07567 TdcF protein |  |

  
**Neighborhood Representations for "hse:Hsero\_0965"**  

| ID | Annotation | EC number |
| --- | --- | --- |
| hse:Hsero\_0955 | hypothetical protein; K01999 branched-chain amino acid transport system substrate-binding protein |  |
| hse:Hsero\_0956 | dibenzothiophene desulfurization flavin reductase (EC:1.3.1.60) |  |
| hse:Hsero\_0957 | livF; branched-chain amino acid ABC transporter ATPase; K01996 branched-chain amino acid transport system ATP-binding protein |  |
| hse:Hsero\_0958 | LysR family transcription regulator protein |  |
| hse:Hsero\_0959 | mmsB; 3-hydroxyisobutyrate dehydrogenase (EC:1.1.1.31 1.1.1.44); K00020 3-hydroxyisobutyrate dehydrogenase [EC:1.1.1.31] | ec:1.1.1.31 |
| hse:Hsero\_0960 | NAD-dependent aldehyde dehydrogenase (EC:1.1.1.1 1.2.1.3) |  |
| hse:Hsero\_0961 | membrane protein |  |
| hse:Hsero\_0962 | C4-dicarboxylate transporter; K03319 divalent anion:Na+ symporter, DASS family |  |
| hse:Hsero\_0963 | helix-turn-helix family protein |  |
| hse:Hsero\_0964 | adhA; alcohol dehydrogenase (EC:1.1.1.1 1.1.1.2); K13953 alcohol dehydrogenase, propanol-preferring [EC:1.1.1.1] | ec:1.1.1.1 |
| hse:Hsero\_0965 | glpR; glycerol-3-phosphate regulon repressor transcription regulator protein; K02444 DeoR family transcriptional regulator, glycerol-3-phosphate regulon repressor |  |
| hse:Hsero\_0966 | glpD2; glycerol-3-phosphate dehydrogenase (EC:1.1.5.3); K00111 glycerol-3-phosphate dehydrogenase [EC:1.1.5.3] | ec:1.1.5.3 |
| hse:Hsero\_0967 | sugar ABC transporter ATPase; K17324 glycerol transport system ATP-binding protein |  |
| hse:Hsero\_0968 | sugar ABC transporter ATPase; K17325 glycerol transport system ATP-binding protein |  |
| hse:Hsero\_0969 | sugar ABC transporter permease; K17322 glycerol transport system permease protein |  |
| hse:Hsero\_0970 | sugar ABC transporter permease; K17323 glycerol transport system permease protein |  |
| hse:Hsero\_0971 | integral transmembrane protein |  |
| hse:Hsero\_0972 | sugar ABC transporter periplasmic protein; K17321 glycerol transport system substrate-binding protein |  |
| hse:Hsero\_0973 | glpK; glycerol kinase (EC:2.7.1.30); K00864 glycerol kinase [EC:2.7.1.30] | ec:2.7.1.30 |
| hse:Hsero\_0974 | HD-GYP domain metal-dependent phosphohydrolase (EC:3.1.3.-) |  |
| hse:Hsero\_0975 | hypothetical protein |  |

  
**Neighborhood Representations for "bpa:BPP3096"**  

| ID | Annotation | EC number |
| --- | --- | --- |
| bpa:BPP3086 | dadA; D-amino acid dehydrogenase small subunit (EC:1.4.99.1); K00285 D-amino-acid dehydrogenase [EC:1.4.99.1] | ec:1.4.99.1 |
| bpa:BPP3087 | lrp; leucine-responsive regulatory protein; K03719 Lrp/AsnC family transcriptional regulator, leucine-responsive regulatory protein |  |
| bpa:BPP3088 | hypothetical protein |  |
| bpa:BPP3089 | lipoprotein |  |
| bpa:BPP3090 | hypothetical protein; K17321 glycerol transport system substrate-binding protein |  |
| bpa:BPP3091 | integral membrane protein |  |
| bpa:BPP3092 | ABC transporter inner membrane protein; K17323 glycerol transport system permease protein |  |
| bpa:BPP3093 | ABC transport protein, inner membrane component; K17322 glycerol transport system permease protein |  |
| bpa:BPP3094 | ABC transporter ATP-binding protein; K17325 glycerol transport system ATP-binding protein |  |
| bpa:BPP3095 | ABC transporter ATP-binding protein; K17324 glycerol transport system ATP-binding protein |  |
| bpa:BPP3096 | glpR; glycerol-3-phosphate regulon repressor protein; K02444 DeoR family transcriptional regulator, glycerol-3-phosphate regulon repressor |  |
| bpa:BPP3097 | glycerol-3-phosphate dehydrogenase; K00111 glycerol-3-phosphate dehydrogenase [EC:1.1.5.3] | ec:1.1.5.3 |
| bpa:BPP3098 | IclR family regulatory protein |  |
| bpa:BPP3099 | oxidoreductase |  |
| bpa:BPP3100 | hypothetical protein |  |
| bpa:BPP3101 | hypothetical protein |  |
| bpa:BPP3102 | hypothetical protein; K07045 |  |
| bpa:BPP3103 | hypothetical protein |  |
| bpa:BPP3104 | hypothetical protein |  |
| bpa:BPP3105 | hypothetical protein |  |
| bpa:BPP3106 | pseudogene |  |

  
**Neighborhood Representations for "dba:Dbac\_1439"**  

| ID | Annotation | EC number |
| --- | --- | --- |
| dba:Dbac\_1429 | chemotaxis protein CheR |  |
| dba:Dbac\_1430 | chemotaxis protein CheD; K03411 chemotaxis protein CheD [EC:3.5.1.44] | ec:3.5.1.44 |
| dba:Dbac\_1431 | Fis family sigma-54 specific transcriptional regulator with PAS/PAC sensor |  |
| dba:Dbac\_1432 | PAS/PAC sensor hybrid histidine kinase |  |
| dba:Dbac\_1433 | transposase IS204/IS1001/IS1096/IS1165 family protein |  |
| dba:Dbac\_1434 | glycerol kinase; K00864 glycerol kinase [EC:2.7.1.30] | ec:2.7.1.30 |
| dba:Dbac\_1435 | CoB--CoM heterodisulfide reductase (EC:1.8.98.1); K03389 heterodisulfide reductase subunit B [EC:1.8.98.1] | ec:1.8.98.1 |
| dba:Dbac\_1436 | Heterodisulfide reductase subunit C-like protein |  |
| dba:Dbac\_1437 | glycerol-3-phosphate dehydrogenase (EC:1.1.5.3); K00112 glycerol-3-phosphate dehydrogenase subunit B [EC:1.1.5.3] | ec:1.1.5.3 |
| dba:Dbac\_1438 | FAD dependent oxidoreductase; K00111 glycerol-3-phosphate dehydrogenase [EC:1.1.5.3] | ec:1.1.5.3 |
| dba:Dbac\_1439 | DeoR family transcriptional regulator; K02444 DeoR family transcriptional regulator, glycerol-3-phosphate regulon repressor |  |
| dba:Dbac\_1440 | ABC transporter; K17324 glycerol transport system ATP-binding protein |  |
| dba:Dbac\_1441 | ABC transporter; K17325 glycerol transport system ATP-binding protein |  |
| dba:Dbac\_1442 | binding-protein-dependent transport system inner membrane protein; K17322 glycerol transport system permease protein |  |
| dba:Dbac\_1443 | binding-protein-dependent transport system inner membrane protein; K17323 glycerol transport system permease protein |  |
| dba:Dbac\_1444 | hypothetical protein |  |
| dba:Dbac\_1445 | sugar ABC transporter substrate-binding protein; K17321 glycerol transport system substrate-binding protein |  |
| dba:Dbac\_1446 | molybdopterin binding domain-containing protein; K03742 competence/damage-inducible protein CinA |  |
| dba:Dbac\_1447 | hypothetical protein; K00783 23S rRNA (pseudouridine1915-N3)-methyltransferase [EC:2.1.1.177] | ec:2.1.1.177 |
| dba:Dbac\_1448 | type IV pilus assembly PilZ |  |
| dba:Dbac\_1449 | hypothetical protein |  |

  
**Neighborhood Representations for "bbr:BB3059"**  

| ID | Annotation | EC number |
| --- | --- | --- |
| bbr:BB3049 | dadA; D-amino acid dehydrogenase small subunit (EC:1.4.99.1); K00285 D-amino-acid dehydrogenase [EC:1.4.99.1] | ec:1.4.99.1 |
| bbr:BB3050 | lrp; leucine-responsive regulatory protein; K03719 Lrp/AsnC family transcriptional regulator, leucine-responsive regulatory protein |  |
| bbr:BB3051 | hypothetical protein |  |
| bbr:BB3052 | lipoprotein |  |
| bbr:BB3053 | hypothetical protein; K17321 glycerol transport system substrate-binding protein |  |
| bbr:BB3054 | integral membrane protein |  |
| bbr:BB3055 | ABC transporter; K17323 glycerol transport system permease protein |  |
| bbr:BB3056 | ABC transporter; K17322 glycerol transport system permease protein |  |
| bbr:BB3057 | ABC transporter ATP-binding protein; K17325 glycerol transport system ATP-binding protein |  |
| bbr:BB3058 | ABC transporter ATP-binding protein; K17324 glycerol transport system ATP-binding protein |  |
| bbr:BB3059 | glpR; glycerol-3-phosphate regulon repressor protein; K02444 DeoR family transcriptional regulator, glycerol-3-phosphate regulon repressor |  |
| bbr:BB3060 | glycerol-3-phosphate dehydrogenase; K00111 glycerol-3-phosphate dehydrogenase [EC:1.1.5.3] | ec:1.1.5.3 |
| bbr:BB3061 | IclR family transcriptional regulator |  |
| bbr:BB3062 | oxidoreductase |  |
| bbr:BB3063 | hypothetical protein |  |
| bbr:BB3064 | hypothetical protein |  |
| bbr:BB3065 | hypothetical protein; K07045 |  |
| bbr:BB3066 | hypothetical protein |  |
| bbr:BB3067 | hypothetical protein |  |
| bbr:BB3068 | hypothetical protein |  |
| bbr:BB3069 | hypothetical protein |  |

  
**Neighborhood Representations for "pol:Bpro\_0477"**  

| ID | Annotation | EC number |
| --- | --- | --- |
| pol:Bpro\_0467 | S-adenosylmethionine; K07568 S-adenosylmethionine:tRNA ribosyltransferase-isomerase [EC:2.4.99.17] | ec:2.4.99.17 |
| pol:Bpro\_0468 | ATP-dependent DNA helicase RecG; K03655 ATP-dependent DNA helicase RecG [EC:3.6.4.12] | ec:3.6.4.12 |
| pol:Bpro\_0469 | hypothetical protein |  |
| pol:Bpro\_0470 | hypothetical protein |  |
| pol:Bpro\_0471 | putative transcriptional regulator; K07726 putative transcriptional regulator |  |
| pol:Bpro\_0472 | LysR family transcriptional regulator; K04761 LysR family transcriptional regulator, hydrogen peroxide-inducible genes activator |  |
| pol:Bpro\_0473 | Dyp-type peroxidase; K07223 putative iron-dependent peroxidase |  |
| pol:Bpro\_0474 | 4-hydroxybenzoate octaprenyltransferase (EC:2.5.1.-); K03179 4-hydroxybenzoate octaprenyltransferase [EC:2.5.1.-] |  |
| pol:Bpro\_0475 | pyrroline-5-carboxylate reductase (EC:1.5.1.2); K00286 pyrroline-5-carboxylate reductase [EC:1.5.1.2] | ec:1.5.1.2 |
| pol:Bpro\_0476 | glycerol kinase; K00864 glycerol kinase [EC:2.7.1.30] | ec:2.7.1.30 |
| pol:Bpro\_0477 | DeoR family transcriptional regulator; K02444 DeoR family transcriptional regulator, glycerol-3-phosphate regulon repressor |  |
| pol:Bpro\_0478 | ABC transporter-like protein; K17324 glycerol transport system ATP-binding protein |  |
| pol:Bpro\_0479 | ABC transporter-like protein; K17325 glycerol transport system ATP-binding protein |  |
| pol:Bpro\_0480 | binding-protein-dependent transport systems inner membrane component; K17322 glycerol transport system permease protein |  |
| pol:Bpro\_0481 | binding-protein-dependent transport systems inner membrane component; K17323 glycerol transport system permease protein |  |
| pol:Bpro\_0482 | putative integral membrane protein |  |
| pol:Bpro\_0483 | twin-arginine translocation pathway signal protein; K17321 glycerol transport system substrate-binding protein |  |
| pol:Bpro\_0484 | FAD dependent oxidoreductase; K00111 glycerol-3-phosphate dehydrogenase [EC:1.1.5.3] | ec:1.1.5.3 |
| pol:Bpro\_0485 | rplN; 50S ribosomal protein L14; K02874 large subunit ribosomal protein L14 |  |
| pol:Bpro\_0486 | rplX; 50S ribosomal protein L24; K02895 large subunit ribosomal protein L24 |  |
| pol:Bpro\_0487 | rplE; 50S ribosomal protein L5; K02931 large subunit ribosomal protein L5 |  |

  
**Neighborhood Representations for "rso:RSc3044"**  

| ID | Annotation | EC number |
| --- | --- | --- |
| rso:RSc3034 | rpoB; DNA-directed RNA polymerase subunit beta (EC:2.7.7.6); K03043 DNA-directed RNA polymerase subunit beta [EC:2.7.7.6] | ec:2.7.7.6 |
| rso:RSc3035 | rplL; 50S ribosomal protein L7/L12; K02935 large subunit ribosomal protein L7/L12 |  |
| rso:RSc3036 | rplJ; 50S ribosomal protein L10; K02864 large subunit ribosomal protein L10 |  |
| rso:RSc3037 | rplA; 50S ribosomal protein L1; K02863 large subunit ribosomal protein L1 |  |
| rso:RSc3038 | rplK; 50S ribosomal protein L11; K02867 large subunit ribosomal protein L11 |  |
| rso:RSc3039 | nusG; transcription antitermination protein NusG; K02601 transcriptional antiterminator NusG |  |
| rso:RSc3040 | secE; preprotein translocase subunit SecE; K03073 preprotein translocase subunit SecE |  |
| rso:RSc3041 | tuf; elongation factor Tu (EC:3.6.5.3); K02358 elongation factor Tu |  |
| rso:RSc3042 | hypothetical protein |  |
| rso:RSc3043 | hypothetical protein |  |
| rso:RSc3044 | glpR; glycerol-3-phosphate regulon repressor transcription regulator protein; K02444 DeoR family transcriptional regulator, glycerol-3-phosphate regulon repressor |  |
| rso:RSc3045 | glpD; glycerol-3-phosphate dehydrogenase (EC:1.1.5.3); K00111 glycerol-3-phosphate dehydrogenase [EC:1.1.5.3] | ec:1.1.5.3 |
| rso:RSc3046 | sugar-phosphate ATP-binding ABC transporter protein; K17324 glycerol transport system ATP-binding protein |  |
| rso:RSc3047 | ABC transporter ATP-binding protein; K17325 glycerol transport system ATP-binding protein |  |
| rso:RSc3048 | sugar-phosphate transmembrane ABC transporter protein; K17322 glycerol transport system permease protein |  |
| rso:RSc3049 | transmembrane ABC transporter protein; K17323 glycerol transport system permease protein |  |
| rso:RSc3050 | hypothetical protein |  |
| rso:RSc3051 | signal peptide protein; K17321 glycerol transport system substrate-binding protein |  |
| rso:RSc3052 | glpK; ATP:glycerol 3-phosphotransferase (EC:2.7.1.30); K00864 glycerol kinase [EC:2.7.1.30] | ec:2.7.1.30 |
| rso:RSc3053 | signal peptide protein |  |
| rso:RSc3054 | hypothetical protein; K09941 hypothetical protein |  |

  
**Neighborhood Representations for "bpe:BP2643"**  

| ID | Annotation | EC number |
| --- | --- | --- |
| bpe:BP2633 | exonuclease |  |
| bpe:BP2634 | dadA; pseudogene |  |
| bpe:BP2635 | lrp; leucine-responsive regulatory protein; K03719 Lrp/AsnC family transcriptional regulator, leucine-responsive regulatory protein |  |
| bpe:BP2636 | hypothetical protein |  |
| bpe:BP2637 | lipoprotein |  |
| bpe:BP2638 | ABC transporter substrate-binding protein; K17321 glycerol transport system substrate-binding protein |  |
| bpe:BP2639 | hypothetical protein |  |
| bpe:BP2640 | ABC transporter permease; K17323 glycerol transport system permease protein |  |
| bpe:BP2641 | pseudogene |  |
| bpe:BP2642 | ABC transporter ATP-binding protein; K17325 glycerol transport system ATP-binding protein |  |
| bpe:BP2643 | glpR; glycerol-3-phosphate regulon repressor protein; K02444 DeoR family transcriptional regulator, glycerol-3-phosphate regulon repressor |  |
| bpe:BP2644 | glycerol-3-phosphate dehydrogenase; K00111 glycerol-3-phosphate dehydrogenase [EC:1.1.5.3] | ec:1.1.5.3 |
| bpe:BP2645 | IclR family transcriptional regulator |  |
| bpe:BP2646 | pseudogene |  |
| bpe:BP2647 | hypothetical protein |  |
| bpe:BP2648 | hypothetical protein |  |
| bpe:BP2649 | hypothetical protein; K07045 |  |
| bpe:BP2650 | pseudogene |  |
| bpe:BP2651 | hypothetical protein |  |
| bpe:BP2652 | hypothetical protein |  |
| bpe:BP2653 | hypothetical protein |  |

  
**Neighborhood Representations for "vap:Vapar\_3398"**  

| ID | Annotation | EC number |
| --- | --- | --- |
| vap:Vapar\_3388 | hypothetical protein |  |
| vap:Vapar\_3389 | 3-oxoacyl-(acyl carrier protein) synthase III; K00648 3-oxoacyl-[acyl-carrier-protein] synthase III [EC:2.3.1.180] | ec:2.3.1.180 |
| vap:Vapar\_3390 | hypothetical protein |  |
| vap:Vapar\_3391 | extracellular solute-binding protein; K17321 glycerol transport system substrate-binding protein |  |
| vap:Vapar\_3392 | integral membrane protein |  |
| vap:Vapar\_3393 | binding-protein-dependent transport systems inner membrane component; K17323 glycerol transport system permease protein |  |
| vap:Vapar\_3394 | binding-protein-dependent transport systems inner membrane component; K17322 glycerol transport system permease protein |  |
| vap:Vapar\_3395 | ABC transporter; K17325 glycerol transport system ATP-binding protein |  |
| vap:Vapar\_3396 | ABC transporter; K17324 glycerol transport system ATP-binding protein |  |
| vap:Vapar\_3397 | glpD; glycerol-3-phosphate dehydrogenase; K00111 glycerol-3-phosphate dehydrogenase [EC:1.1.5.3] | ec:1.1.5.3 |
| vap:Vapar\_3398 | DeoR family transcriptional regulator; K02444 DeoR family transcriptional regulator, glycerol-3-phosphate regulon repressor |  |
| vap:Vapar\_3399 | glycerol kinase; K00864 glycerol kinase [EC:2.7.1.30] | ec:2.7.1.30 |
| vap:Vapar\_3400 | hypothetical protein |  |
| vap:Vapar\_3401 | sulfatase |  |
| vap:Vapar\_3402 | hypothetical protein |  |
| vap:Vapar\_3403 | hypothetical protein |  |
| vap:Vapar\_3404 | DNA internalization-related competence protein ComEC/Rec2; K02238 competence protein ComEC |  |
| vap:Vapar\_3405 | transglutaminase |  |
| vap:Vapar\_3406 | hypothetical protein |  |
| vap:Vapar\_3407 | transglutaminase |  |
| vap:Vapar\_3408 | endoribonuclease L-PSP |  |

  
**Neighborhood Representations for "azl:AZL\_e03100"**  

| ID | Annotation | EC number |
| --- | --- | --- |
| azl:AZL\_e03000 | acyl-CoA synthetase; K00666 fatty-acyl-CoA synthase [EC:6.2.1.-] |  |
| azl:AZL\_e03010 | glpK; glycerol kinase (EC:2.7.1.30); K00864 glycerol kinase [EC:2.7.1.30] | ec:2.7.1.30 |
| azl:AZL\_e03020 | multiple sugar transport system substrate-binding protein; K17321 glycerol transport system substrate-binding protein |  |
| azl:AZL\_e03030 | hypothetical protein |  |
| azl:AZL\_e03040 | multiple sugar transport system permease protein; K17323 glycerol transport system permease protein |  |
| azl:AZL\_e03050 | multiple sugar transport system permease protein; K17322 glycerol transport system permease protein |  |
| azl:AZL\_e03060 | multiple sugar transport system ATP-binding protein; K17325 glycerol transport system ATP-binding protein |  |
| azl:AZL\_e03070 | multiple sugar transport system ATP-binding protein; K17324 glycerol transport system ATP-binding protein |  |
| azl:AZL\_e03080 | glpD; glycerol-3-phosphate dehydrogenase (EC:1.1.99.5); K00111 glycerol-3-phosphate dehydrogenase [EC:1.1.5.3] | ec:1.1.5.3 |
| azl:AZL\_e03090 | mcp; methyl-accepting chemotaxis protein |  |
| azl:AZL\_e03100 | glpR; transcriptional regulator; K02444 DeoR family transcriptional regulator, glycerol-3-phosphate regulon repressor |  |
| azl:AZL\_e03110 | guaC; GMP reductase (EC:1.7.1.7); K00364 GMP reductase [EC:1.7.1.7] | ec:1.7.1.7 |
| azl:AZL\_e03120 | hypothetical protein |  |
| azl:AZL\_e03130 | hemerythrin HHE cation binding region |  |
| azl:AZL\_e03140 | hypothetical protein |  |
| azl:AZL\_e03150 | uspA-like nucleotide-binding protein |  |
| azl:AZL\_e03160 | hypothetical protein; K07028 |  |
| azl:AZL\_e03170 | hypothetical protein |  |
| azl:AZL\_e03180 | hypothetical protein |  |
| azl:AZL\_e03190 | permease of the drug/metabolite transporter superfamily |  |
| azl:AZL\_e03200 | hypothetical protein |  |

  
**Neighborhood Representations for "rsc:RCFBP\_10402"**  

| ID | Annotation | EC number |
| --- | --- | --- |
| rsc:RCFBP\_10391 | hypothetical protein; K09941 hypothetical protein |  |
| rsc:RCFBP\_10392 | hypothetical protein |  |
| rsc:RCFBP\_10393 | glpK; glycerol kinase (ATP:glycerol 3-phosphotransferase) (EC:2.7.1.30); K00864 glycerol kinase [EC:2.7.1.30] | ec:2.7.1.30 |
| rsc:RCFBP\_10394 | sugar ABC transporter sugar binding; K17321 glycerol transport system substrate-binding protein |  |
| rsc:RCFBP\_10395 | hypothetical protein |  |
| rsc:RCFBP\_10396 | sugar ABC transporter permease; K17323 glycerol transport system permease protein |  |
| rsc:RCFBP\_10397 | ABC transporter permease; K17322 glycerol transport system permease protein |  |
| rsc:RCFBP\_10398 | ugpC; sn-glycerol-3-phosphate transport ATP-binding protein (EC:3.6.3.-); K17325 glycerol transport system ATP-binding protein |  |
| rsc:RCFBP\_10399 | ugpC; sn-glycerol-3-phosphate transport ATP-binding protein (EC:3.6.3.-); K17324 glycerol transport system ATP-binding protein |  |
| rsc:RCFBP\_10400 | glpD; glycerol-3-phosphate dehydrogenase (EC:1.1.5.3); K00111 glycerol-3-phosphate dehydrogenase [EC:1.1.5.3] | ec:1.1.5.3 |
| rsc:RCFBP\_10402 | glpR; glycerol-3-phosphate regulon repressor, deor family; K02444 DeoR family transcriptional regulator, glycerol-3-phosphate regulon repressor |  |
| rsc:RCFBP\_10404 | hypothetical protein |  |
| rsc:RCFBP\_tRNA2 | tRNA-Ile; K14227 tRNA Ile |  |
| rsc:RCFBP\_tRNA3 | tRNA-Ala; K14218 tRNA Ala |  |
| rsc:RCFBP\_10406 | hypothetical protein |  |
| rsc:RCFBP\_tRNA4 | tRNA-Tyr; K14236 tRNA Tyr |  |
| rsc:RCFBP\_tRNA5 | tRNA-Gly; K14225 tRNA Gly |  |
| rsc:RCFBP\_tRNA6 | tRNA-Thr; K14234 tRNA Thr |  |
| rsc:RCFBP\_10407 | tufB; protein chain elongation factor ef-tu; K02358 elongation factor Tu |  |
| rsc:RCFBP\_tRNA7 | tRNA-Trp; K14235 tRNA Trp |  |
| rsc:RCFBP\_10408 | secE; preprotein translocase membrane subunit; K03073 preprotein translocase subunit SecE |  |

  
**Neighborhood Representations for "lch:Lcho\_3222"**  

| ID | Annotation | EC number |
| --- | --- | --- |
| lch:Lcho\_3212 | coenzyme F390 synthetase-like protein |  |
| lch:Lcho\_3213 | poly-beta-hydroxybutyrate polymerase domain-containing protein; K03821 polyhydroxyalkanoate synthase [EC:2.3.1.-] |  |
| lch:Lcho\_3214 | 5'-nucleotidase domain-containing protein |  |
| lch:Lcho\_3215 | hypothetical protein |  |
| lch:Lcho\_3216 | methyl-accepting chemotaxis sensory transducer; K05874 methyl-accepting chemotaxis protein I, serine sensor receptor |  |
| lch:Lcho\_3217 | Alpha-glucosidase (EC:3.2.1.20); K01187 alpha-glucosidase [EC:3.2.1.20] | ec:3.2.1.20 |
| lch:Lcho\_3218 | TRAP dicarboxylate transporter subunit DctM |  |
| lch:Lcho\_3219 | tripartite ATP-independent periplasmic transporter DctQ |  |
| lch:Lcho\_3220 | TRAP dicarboxylate transporter subunit Dctp |  |
| lch:Lcho\_3221 | glpD; glycerol-3-phosphate dehydrogenase; K00111 glycerol-3-phosphate dehydrogenase [EC:1.1.5.3] | ec:1.1.5.3 |
| lch:Lcho\_3222 | DeoR family transcriptional regulator; K02444 DeoR family transcriptional regulator, glycerol-3-phosphate regulon repressor |  |
| lch:Lcho\_3223 | glycerol kinase; K00864 glycerol kinase [EC:2.7.1.30] | ec:2.7.1.30 |
| lch:Lcho\_3224 | sodium/hydrogen exchanger |  |
| lch:Lcho\_3225 | carboxylate-amine ligase; K06048 carboxylate-amine ligase [EC:6.3.-.-] |  |
| lch:Lcho\_3226 | peptidase C26; K07010 putative glutamine amidotransferase |  |
| lch:Lcho\_3227 | LacI family transcriptional regulator (EC:5.1.1.1); K02529 LacI family transcriptional regulator |  |
| lch:Lcho\_3228 | tryptophan halogenase; K14266 FADH2 O2-dependent halogenase I [EC:1.14.14.7] | ec:1.14.14.7 |
| lch:Lcho\_3229 | TonB-dependent receptor |  |
| lch:Lcho\_3230 | sodium/hydrogen exchanger |  |
| lch:Lcho\_3231 | hypothetical protein |  |
| lch:Lcho\_3232 | biopolymer transport protein ExbD/TolR; K03559 biopolymer transport protein ExbD |  |

  
**Neighborhood Representations for "rpf:Rpic12D\_2979"**  

| ID | Annotation | EC number |
| --- | --- | --- |
| rpf:Rpic12D\_2969 | rplJ; 50S ribosomal protein L10; K02864 large subunit ribosomal protein L10 |  |
| rpf:Rpic12D\_2970 | rplA; 50S ribosomal protein L1; K02863 large subunit ribosomal protein L1 |  |
| rpf:Rpic12D\_2971 | rplK; 50S ribosomal protein L11; K02867 large subunit ribosomal protein L11 |  |
| rpf:Rpic12D\_2972 | nusG; transcription antitermination protein NusG; K02601 transcriptional antiterminator NusG |  |
| rpf:Rpic12D\_2973 | secE; preprotein translocase subunit SecE; K03073 preprotein translocase subunit SecE |  |
| rpf:Rpic12D\_2974 | elongation factor Tu; K02358 elongation factor Tu |  |
| rpf:Rpic12D\_2975 | transposase IS66 |  |
| rpf:Rpic12D\_2976 | IS66 Orf2 family protein |  |
| rpf:Rpic12D\_2977 | transposase IS3/IS911 family protein |  |
| rpf:Rpic12D\_2978 | CHAD domain-containing protein |  |
| rpf:Rpic12D\_2979 | DeoR family transcriptional regulator; K02444 DeoR family transcriptional regulator, glycerol-3-phosphate regulon repressor |  |
| rpf:Rpic12D\_2980 | glpD; glycerol-3-phosphate dehydrogenase; K00111 glycerol-3-phosphate dehydrogenase [EC:1.1.5.3] | ec:1.1.5.3 |
| rpf:Rpic12D\_2981 | ABC transporter; K17324 glycerol transport system ATP-binding protein |  |
| rpf:Rpic12D\_2982 | ABC transporter; K17325 glycerol transport system ATP-binding protein |  |
| rpf:Rpic12D\_2983 | binding-protein-dependent transport system inner membrane protein; K17322 glycerol transport system permease protein |  |
| rpf:Rpic12D\_2984 | binding-protein-dependent transport system inner membrane protein; K17323 glycerol transport system permease protein |  |
| rpf:Rpic12D\_2985 | hypothetical protein |  |
| rpf:Rpic12D\_2986 | signal peptide protein; K17321 glycerol transport system substrate-binding protein |  |
| rpf:Rpic12D\_2987 | glycerol kinase; K00864 glycerol kinase [EC:2.7.1.30] | ec:2.7.1.30 |
| rpf:Rpic12D\_2988 | signal peptide protein |  |
| rpf:Rpic12D\_2989 | hypothetical protein; K09941 hypothetical protein |  |

  
**Neighborhood Representations for "rsl:RPSI07\_0452"**  

| ID | Annotation | EC number |
| --- | --- | --- |
| rsl:RPSI07\_0442 | hypothetical protein; K09941 hypothetical protein |  |
| rsl:RPSI07\_0443 | hypothetical protein |  |
| rsl:RPSI07\_0444 | glpK; glycerol kinase (EC:2.7.1.30); K00864 glycerol kinase [EC:2.7.1.30] | ec:2.7.1.30 |
| rsl:RPSI07\_0445 | sugar ABC transporter substrate-binding protein; K17321 glycerol transport system substrate-binding protein |  |
| rsl:RPSI07\_0446 | hypothetical protein |  |
| rsl:RPSI07\_0447 | sugar ABC transporter permease; K17323 glycerol transport system permease protein |  |
| rsl:RPSI07\_0448 | ABC transporter permease; K17322 glycerol transport system permease protein |  |
| rsl:RPSI07\_0449 | ugpC; sn-glycerol-3-phosphate transport ATP-binding protein (EC:3.6.3.-); K17325 glycerol transport system ATP-binding protein |  |
| rsl:RPSI07\_0450 | ugpC; sn-glycerol-3-phosphate transport ATP-binding protein (EC:3.6.3.-); K17324 glycerol transport system ATP-binding protein |  |
| rsl:RPSI07\_0451 | glpD; glycerol-3-phosphate dehydrogenase (EC:1.1.5.3); K00111 glycerol-3-phosphate dehydrogenase [EC:1.1.5.3] | ec:1.1.5.3 |
| rsl:RPSI07\_0452 | glpR; glycerol-3-phosphate regulon repressor, deoR family; K02444 DeoR family transcriptional regulator, glycerol-3-phosphate regulon repressor |  |
| rsl:RPSI07\_0453 | hypothetical protein |  |
| rsl:RPSI07\_0455 | hypothetical protein |  |
| rsl:RPSI07\_0456 | tufB; protein chain elongation factor EF-Tu; K02358 elongation factor Tu |  |
| rsl:RPSI07\_0457 | secE; preprotein translocase membrane subunit; K03073 preprotein translocase subunit SecE |  |
| rsl:RPSI07\_0458 | nusG; transcription termination factor; K02601 transcriptional antiterminator NusG |  |
| rsl:RPSI07\_0459 | rplK; 50S ribosomal subunit protein L11; K02867 large subunit ribosomal protein L11 |  |
| rsl:RPSI07\_0460 | rplA; 50S ribosomal subunit protein L1; K02863 large subunit ribosomal protein L1 |  |
| rsl:RPSI07\_0461 | rplJ; 50S ribosomal subunit protein L10; K02864 large subunit ribosomal protein L10 |  |
| rsl:RPSI07\_0462 | rplL; 50S ribosomal subunit protein L7/L12; K02935 large subunit ribosomal protein L7/L12 |  |
| rsl:RPSI07\_0463 | rpoB; RNA polymerase subunit beta (EC:2.7.7.6); K03043 DNA-directed RNA polymerase subunit beta [EC:2.7.7.6] | ec:2.7.7.6 |

  
**Neighborhood Representations for "axy:AXYL\_03486"**  

| ID | Annotation | EC number |
| --- | --- | --- |
| axy:AXYL\_03476 | malate/L-lactate dehydrogenase (EC:1.1.1.-) |  |
| axy:AXYL\_03477 | extra-cytoplasmic solute receptor family protein 76 |  |
| axy:AXYL\_03478 | LysR family transcriptional regulator |  |
| axy:AXYL\_03479 | hypothetical protein |  |
| axy:AXYL\_03480 | extracellular solute-binding family protein; K17321 glycerol transport system substrate-binding protein |  |
| axy:AXYL\_03481 | hypothetical protein |  |
| axy:AXYL\_03482 | binding-protein-dependent transporter inner membrane component family protein 40; K17323 glycerol transport system permease protein |  |
| axy:AXYL\_03483 | binding-protein-dependent transporter inner membrane component family protein 41; K17322 glycerol transport system permease protein |  |
| axy:AXYL\_03484 | ABC transporter (EC:3.6.3.-); K17325 glycerol transport system ATP-binding protein |  |
| axy:AXYL\_03485 | ABC transporter (EC:3.6.3.-); K17324 glycerol transport system ATP-binding protein |  |
| axy:AXYL\_03486 | glycerol-3-phosphate regulon repressor; K02444 DeoR family transcriptional regulator, glycerol-3-phosphate regulon repressor |  |
| axy:AXYL\_03487 | glpD2; glycerol-3-phosphate dehydrogenase 2 (EC:1.1.5.3); K00111 glycerol-3-phosphate dehydrogenase [EC:1.1.5.3] | ec:1.1.5.3 |
| axy:AXYL\_03488 | HD domain-containing protein 1 |  |
| axy:AXYL\_03489 | DJ-1/PfpI family protein 1 |  |
| axy:AXYL\_03490 | oxidoreductase, zinc-binding dehydrogenase family protein (EC:1.-.-.-); K07119 |  |
| axy:AXYL\_03491 | TetR family transcriptional regulator |  |
| axy:AXYL\_03492 | hemolysin D; K01993 HlyD family secretion protein |  |
| axy:AXYL\_03493 | ABC transporter (EC:3.6.3.-); K01990 ABC-2 type transport system ATP-binding protein |  |
| axy:AXYL\_03494 | ABC transporter; K01992 ABC-2 type transport system permease protein |  |
| axy:AXYL\_03495 | efflux transporter outer membrane factor lipoprotein 5 |  |
| axy:AXYL\_03496 | response regulator 11 |  |

  
**Neighborhood Representations for "rpi:Rpic\_3326"**  

| ID | Annotation | EC number |
| --- | --- | --- |
| rpi:Rpic\_3316 | rplJ; 50S ribosomal protein L10; K02864 large subunit ribosomal protein L10 |  |
| rpi:Rpic\_3317 | rplA; 50S ribosomal protein L1; K02863 large subunit ribosomal protein L1 |  |
| rpi:Rpic\_3318 | rplK; 50S ribosomal protein L11; K02867 large subunit ribosomal protein L11 |  |
| rpi:Rpic\_3319 | nusG; transcription antitermination protein NusG; K02601 transcriptional antiterminator NusG |  |
| rpi:Rpic\_3320 | secE; preprotein translocase subunit SecE; K03073 preprotein translocase subunit SecE |  |
| rpi:Rpic\_3321 | elongation factor Tu; K02358 elongation factor Tu |  |
| rpi:Rpic\_3322 | transposase IS66 |  |
| rpi:Rpic\_3323 | IS66 Orf2 family protein |  |
| rpi:Rpic\_3324 | hypothetical protein |  |
| rpi:Rpic\_3325 | CHAD domain-containing protein |  |
| rpi:Rpic\_3326 | DeoR family transcriptional regulator; K02444 DeoR family transcriptional regulator, glycerol-3-phosphate regulon repressor |  |
| rpi:Rpic\_3327 | glpD; glycerol-3-phosphate dehydrogenase; K00111 glycerol-3-phosphate dehydrogenase [EC:1.1.5.3] | ec:1.1.5.3 |
| rpi:Rpic\_3328 | ABC transporter-like protein; K17324 glycerol transport system ATP-binding protein |  |
| rpi:Rpic\_3329 | ABC transporter-like protein; K17325 glycerol transport system ATP-binding protein |  |
| rpi:Rpic\_3330 | binding-protein-dependent transport system inner membrane protein; K17322 glycerol transport system permease protein |  |
| rpi:Rpic\_3331 | binding-protein-dependent transport system inner membrane protein; K17323 glycerol transport system permease protein |  |
| rpi:Rpic\_3332 | hypothetical protein |  |
| rpi:Rpic\_3333 | hypothetical protein; K17321 glycerol transport system substrate-binding protein |  |
| rpi:Rpic\_3334 | glycerol kinase; K00864 glycerol kinase [EC:2.7.1.30] | ec:2.7.1.30 |
| rpi:Rpic\_3335 | hypothetical protein |  |
| rpi:Rpic\_3336 | hypothetical protein; K09941 hypothetical protein |  |

  
**Neighborhood Representations for "hch:HCH\_06963"**  

| ID | Annotation | EC number |
| --- | --- | --- |
| hch:HCH\_06951 | metalloendopeptidase-like membrane protein |  |
| hch:HCH\_06953 | hypothetical protein |  |
| hch:HCH\_06954 | hypothetical protein |  |
| hch:HCH\_06955 | integral membrane protein |  |
| hch:HCH\_06956 | nucleoside-diphosphate-sugar epimerase |  |
| hch:HCH\_06957 | transcriptional regulator |  |
| hch:HCH\_06958 | trpB2; tryptophan synthase subunit beta (EC:4.2.1.20); K01696 tryptophan synthase beta chain [EC:4.2.1.20] | ec:4.2.1.20 |
| hch:HCH\_06959 | trpA2; tryptophan synthase subunit alpha (EC:4.2.1.20); K01695 tryptophan synthase alpha chain [EC:4.2.1.20] | ec:4.2.1.20 |
| hch:HCH\_06960 | hypothetical protein; K09768 hypothetical protein |  |
| hch:HCH\_06962 | flagellin-like protein; K02406 flagellin |  |
| hch:HCH\_06963 | sugar metabolism transcriptional regulator; K02444 DeoR family transcriptional regulator, glycerol-3-phosphate regulon repressor |  |
| hch:HCH\_06964 | glpD; glycerol-3-phosphate dehydrogenase (EC:1.1.5.3); K00111 glycerol-3-phosphate dehydrogenase [EC:1.1.5.3] | ec:1.1.5.3 |
| hch:HCH\_06965 | glpK; glycerol kinase (EC:2.7.1.30); K00864 glycerol kinase [EC:2.7.1.30] | ec:2.7.1.30 |
| hch:HCH\_06966 | acoR; transcriptional activator of acetoin/glycerol metabolism |  |
| hch:HCH\_06967 | sugar ABC transporter ATPase; K17324 glycerol transport system ATP-binding protein |  |
| hch:HCH\_06968 | sugar ABC transporter ATPase; K17325 glycerol transport system ATP-binding protein |  |
| hch:HCH\_06969 | sugar ABC transporter permease; K17322 glycerol transport system permease protein |  |
| hch:HCH\_06970 | sugar ABC transporter permease; K17323 glycerol transport system permease protein |  |
| hch:HCH\_06971 | hypothetical protein |  |
| hch:HCH\_06972 | hypothetical protein |  |
| hch:HCH\_06973 | sugar ABC transporter periplasmic protein; K17321 glycerol transport system substrate-binding protein |  |

  
**Neighborhood Representations for "ppf:Pput\_1115"**  

| ID | Annotation | EC number |
| --- | --- | --- |
| ppf:Pput\_1105 | alpha/beta hydrolase fold family protein |  |
| ppf:Pput\_1106 | GlpM family protein; K02442 membrane protein GlpM |  |
| ppf:Pput\_1107 | Fis family two component sigma-54 specific transcriptional regulator; K17061 two-component system, response regulator AauR |  |
| ppf:Pput\_1108 | integral membrane sensor signal transduction histidine kinase; K17060 two-component system, sensor histidine kinase AauS [EC:2.7.13.3] | ec:2.7.13.3 |
| ppf:Pput\_1109 | ABC transporter-like protein; K10004 glutamate/aspartate transport system ATP-binding protein [EC:3.6.3.-] |  |
| ppf:Pput\_1110 | polar amino acid ABC transporter inner membrane subunit; K10002 glutamate/aspartate transport system permease protein |  |
| ppf:Pput\_1111 | polar amino acid ABC transporter inner membrane subunit; K10003 glutamate/aspartate transport system permease protein |  |
| ppf:Pput\_1112 | extracellular solute-binding protein; K10001 glutamate/aspartate transport system substrate-binding protein |  |
| ppf:Pput\_1113 | hypothetical protein |  |
| ppf:Pput\_1114 | glpD; glycerol-3-phosphate dehydrogenase; K00111 glycerol-3-phosphate dehydrogenase [EC:1.1.5.3] | ec:1.1.5.3 |
| ppf:Pput\_1115 | DeoR family transcriptional regulator; K02444 DeoR family transcriptional regulator, glycerol-3-phosphate regulon repressor |  |
| ppf:Pput\_1116 | glpK; glycerol kinase; K00864 glycerol kinase [EC:2.7.1.30] | ec:2.7.1.30 |
| ppf:Pput\_1117 | MIP family channel protein; K02440 glycerol uptake facilitator protein |  |
| ppf:Pput\_1118 | ybaK/ebsC protein; K03976 putative transcription regulator |  |
| ppf:Pput\_1119 | ABC transporter-like protein; K02010 iron(III) transport system ATP-binding protein [EC:3.6.3.30] | ec:3.6.3.30 |
| ppf:Pput\_1120 | ornithine carbamoyltransferase; K00611 ornithine carbamoyltransferase [EC:2.1.3.3] | ec:2.1.3.3 |
| ppf:Pput\_1121 | molydopterin dinucleotide-binding region |  |
| ppf:Pput\_1122 | glutaredoxin-like protein; K07390 monothiol glutaredoxin |  |
| ppf:Pput\_1123 | bacterioferritin; K03594 bacterioferritin |  |
| ppf:Pput\_1124 | BFD/(2Fe-2S)-binding domain-containing protein; K02192 bacterioferritin-associated ferredoxin |  |
| ppf:Pput\_1125 | alkyl hydroperoxide reductase; K03386 peroxiredoxin (alkyl hydroperoxide reductase subunit C) [EC:1.11.1.15] | ec:1.11.1.15 |

  
**Neighborhood Representations for "cti:RALTA\_A2007"**  

| ID | Annotation | EC number |
| --- | --- | --- |
| cti:RALTA\_A1997 | rnhA; ribonuclease h (EC:3.1.26.4); K03469 ribonuclease HI [EC:3.1.26.4] | ec:3.1.26.4 |
| cti:RALTA\_A1998 | dnaQ; DNA polymerase III subunit epsilon (EC:2.7.7.7); K02342 DNA polymerase III subunit epsilon [EC:2.7.7.7] | ec:2.7.7.7 |
| cti:RALTA\_A1999 | isochorismatase hydrolase |  |
| cti:RALTA\_A2000 | diguanylate cyclase with associated pas domain |  |
| cti:RALTA\_A2001 | ABC transporter substrate-binding protein; K17321 glycerol transport system substrate-binding protein |  |
| cti:RALTA\_A2002 | transmembrane lipoprotein, cog5477 |  |
| cti:RALTA\_A2003 | ABC transporter permease; K17323 glycerol transport system permease protein |  |
| cti:RALTA\_A2004 | ABC transporter permease; K17322 glycerol transport system permease protein |  |
| cti:RALTA\_A2005 | ugpC2; sn-glycerol-3-phosphate transport ATP-binding protein; K17325 glycerol transport system ATP-binding protein |  |
| cti:RALTA\_A2006 | ugpC1; sn-glycerol-3-phosphate transport ATP-binding protein; K17324 glycerol transport system ATP-binding protein |  |
| cti:RALTA\_A2007 | glpR; glycerol-3-phosphate regulon repressor, deor family; K02444 DeoR family transcriptional regulator, glycerol-3-phosphate regulon repressor |  |
| cti:RALTA\_A2008 | purU; formyltetrahydrofolate deformylase (EC:3.5.1.10); K01433 formyltetrahydrofolate deformylase [EC:3.5.1.10] | ec:3.5.1.10 |
| cti:RALTA\_A2009 | hypothetical protein |  |
| cti:RALTA\_A2010 | glpK; glycerol kinase (ATP:glycerol 3-phosphotransferase) (EC:2.7.1.30); K00864 glycerol kinase [EC:2.7.1.30] | ec:2.7.1.30 |
| cti:RALTA\_A2011 | glpD; sn-glycerol-3-phosphate dehydrogenase, fad/NAD(P)-binding protein (EC:1.1.5.3); K00111 glycerol-3-phosphate dehydrogenase [EC:1.1.5.3] | ec:1.1.5.3 |
| cti:RALTA\_A2012 | lipoprotein |  |
| cti:RALTA\_A2013 | hypothetical protein |  |
| cti:RALTA\_A2014 | hypothetical protein |  |
| cti:RALTA\_A2015 | hypothetical protein |  |
| cti:RALTA\_A2016 | hypothetical protein |  |
| cti:RALTA\_A2017 | hypothetical protein |  |

  
**Neighborhood Representations for "ppu:PP\_1074"**  

| ID | Annotation | EC number |
| --- | --- | --- |
| ppu:PP\_1064 | alpha/beta hydrolase |  |
| ppu:PP\_1065 | GlpM family protein; K02442 membrane protein GlpM |  |
| ppu:PP\_1066 | Fis family transcriptional regulator; K17061 two-component system, response regulator AauR |  |
| ppu:PP\_1067 | integral membrane sensor signal transduction histidine kinase; K17060 two-component system, sensor histidine kinase AauS [EC:2.7.13.3] | ec:2.7.13.3 |
| ppu:PP\_1068 | amino acid ABC transporter ATP-binding protein; K10004 glutamate/aspartate transport system ATP-binding protein [EC:3.6.3.-] |  |
| ppu:PP\_1069 | polar amino acid ABC transporter inner membrane subunit; K10002 glutamate/aspartate transport system permease protein |  |
| ppu:PP\_1070 | polar amino acid ABC transporter inner membrane subunit; K10003 glutamate/aspartate transport system permease protein |  |
| ppu:PP\_1071 | amino acid ABC transporter substrate-binding protein; K10001 glutamate/aspartate transport system substrate-binding protein |  |
| ppu:PP\_1072 | hypothetical protein |  |
| ppu:PP\_1073 | glpD; glycerol-3-phosphate dehydrogenase (EC:1.1.5.3); K00111 glycerol-3-phosphate dehydrogenase [EC:1.1.5.3] | ec:1.1.5.3 |
| ppu:PP\_1074 | glpR; DeoR family transcriptional regulator; K02444 DeoR family transcriptional regulator, glycerol-3-phosphate regulon repressor |  |
| ppu:PP\_1075 | glpK; glycerol kinase (EC:2.7.1.30); K00864 glycerol kinase [EC:2.7.1.30] | ec:2.7.1.30 |
| ppu:PP\_1076 | glpF; MIP family channel protein; K02440 glycerol uptake facilitator protein |  |
| ppu:PP\_1077 | ybaK/ebsC protein |  |
| ppu:PP\_1078 | ABC transporter ATP-binding protein; K02010 iron(III) transport system ATP-binding protein [EC:3.6.3.30] | ec:3.6.3.30 |
| ppu:PP\_1079 | argF; ornithine carbamoyltransferase (EC:2.1.3.3); K00611 ornithine carbamoyltransferase [EC:2.1.3.3] | ec:2.1.3.3 |
| ppu:PP\_1080 | molydopterin dinucleotide-binding region |  |
| ppu:PP\_1081 | glutaredoxin-like protein; K07390 monothiol glutaredoxin |  |
| ppu:PP\_1082 | bfr; bacterioferritin; K03594 bacterioferritin |  |
| ppu:PP\_1083 | BFD (2Fe-2S)-binding domain-containing protein; K02192 bacterioferritin-associated ferredoxin |  |
| ppu:PP\_1084 | anti-oxidant AhpCTSA family protein; K03386 peroxiredoxin (alkyl hydroperoxide reductase subunit C) [EC:1.11.1.15] | ec:1.11.1.15 |

  
**Neighborhood Representations for "reu:Reut\_A2210"**  

| ID | Annotation | EC number |
| --- | --- | --- |
| reu:Reut\_A2200 | transposase |  |
| reu:Reut\_A2201 | KAP P-loop protein |  |
| reu:Reut\_A2202 | hypothetical protein |  |
| reu:Reut\_A2203 | hypothetical protein |  |
| reu:Reut\_A2204 | extracellular solute-binding protein; K17321 glycerol transport system substrate-binding protein |  |
| reu:Reut\_A2205 | integral membrane protein |  |
| reu:Reut\_A2206 | binding-protein dependent transport system inner membrane protein; K17323 glycerol transport system permease protein |  |
| reu:Reut\_A2207 | binding-protein dependent transport system inner membrane protein; K17322 glycerol transport system permease protein |  |
| reu:Reut\_A2208 | ABC transporter; K17325 glycerol transport system ATP-binding protein |  |
| reu:Reut\_A2209 | ABC transporter; K17324 glycerol transport system ATP-binding protein |  |
| reu:Reut\_A2210 | DeoR family transcriptional regulator; K02444 DeoR family transcriptional regulator, glycerol-3-phosphate regulon repressor |  |
| reu:Reut\_A2211 | purU; formyltetrahydrofolate deformylase (EC:3.5.1.10); K01433 formyltetrahydrofolate deformylase [EC:3.5.1.10] | ec:3.5.1.10 |
| reu:Reut\_A2212 | NUDIX hydrolase |  |
| reu:Reut\_A2213 | glycerol kinase; K00864 glycerol kinase [EC:2.7.1.30] | ec:2.7.1.30 |
| reu:Reut\_A2214 | FAD dependent oxidoreductase; K00111 glycerol-3-phosphate dehydrogenase [EC:1.1.5.3] | ec:1.1.5.3 |
| reu:Reut\_A2215 | hypothetical protein |  |
| reu:Reut\_A2216 | hypothetical protein |  |
| reu:Reut\_A2217 | hypothetical protein |  |
| reu:Reut\_A2218 | signal peptide protein |  |
| reu:Reut\_A2219 | hypothetical protein |  |
| reu:Reut\_A2220 | integral membrane protein |  |

  
**Neighborhood Representations for "pen:PSEEN1196"**  

| ID | Annotation | EC number |
| --- | --- | --- |
| pen:PSEEN1185 | alpha/beta fold family hydrolase |  |
| pen:PSEEN1187 | glpM; membrane protein required for efficient alginate biosynthesis; K02442 membrane protein GlpM |  |
| pen:PSEEN1188 | sigma-54 dependent response regulator; K17061 two-component system, response regulator AauR |  |
| pen:PSEEN1189 | sensor histidine kinase; K17060 two-component system, sensor histidine kinase AauS [EC:2.7.13.3] | ec:2.7.13.3 |
| pen:PSEEN1190 | gltL; glutamate-aspartate ABC transporter ATP-binding protein (EC:3.6.3.-); K10004 glutamate/aspartate transport system ATP-binding protein [EC:3.6.3.-] |  |
| pen:PSEEN1191 | gltK; glutamate-aspartate ABC transporter permease; K10002 glutamate/aspartate transport system permease protein |  |
| pen:PSEEN1192 | gltJ; glutamate/aspartate ABC transporter permease; K10003 glutamate/aspartate transport system permease protein |  |
| pen:PSEEN1193 | gltI; glutamate/aspartate ABC transporter periplasmic binding protein; K10001 glutamate/aspartate transport system substrate-binding protein |  |
| pen:PSEEN1194 | hypothetical protein |  |
| pen:PSEEN1195 | glpD; glycerol-3-phosphate dehydrogenase (EC:1.1.5.3); K00111 glycerol-3-phosphate dehydrogenase [EC:1.1.5.3] | ec:1.1.5.3 |
| pen:PSEEN1196 | glpR; glycerol-3-phosphate regulon repressor; K02444 DeoR family transcriptional regulator, glycerol-3-phosphate regulon repressor |  |
| pen:PSEEN1197 | glpK; glycerol kinase (EC:2.7.1.30); K00864 glycerol kinase [EC:2.7.1.30] | ec:2.7.1.30 |
| pen:PSEEN1198 | glpF; glycerol uptake facilitator protein; K02440 glycerol uptake facilitator protein |  |
| pen:PSEEN1199 | hypothetical protein; K03976 putative transcription regulator |  |
| pen:PSEEN1200 | ABC transporter ATP-binding protein (EC:3.6.3.-); K02010 iron(III) transport system ATP-binding protein [EC:3.6.3.30] | ec:3.6.3.30 |
| pen:PSEEN1201 | argI; ornithine carbamoyltransferase (EC:2.1.3.3); K00611 ornithine carbamoyltransferase [EC:2.1.3.3] | ec:2.1.3.3 |
| pen:PSEEN1202 | oxidoreductase |  |
| pen:PSEEN1203 | hypothetical protein; K07390 monothiol glutaredoxin |  |
| pen:PSEEN1204 | bfrB; bacterioferritin; K03594 bacterioferritin |  |
| pen:PSEEN1205 | bacterioferritin-associated ferredoxin BFD; K02192 bacterioferritin-associated ferredoxin |  |
| pen:PSEEN1206 | peroxiredoxin (alkyl hydroperoxide reductase C) (EC:1.11.1.7); K03386 peroxiredoxin (alkyl hydroperoxide reductase subunit C) [EC:1.11.1.15] | ec:1.11.1.15 |

  
**Neighborhood Representations for "ppg:PputGB1\_4338"**  

| ID | Annotation | EC number |
| --- | --- | --- |
| ppg:PputGB1\_4328 | alkyl hydroperoxide reductase; K03386 peroxiredoxin (alkyl hydroperoxide reductase subunit C) [EC:1.11.1.15] | ec:1.11.1.15 |
| ppg:PputGB1\_4329 | BFD/(2Fe-2S)-binding domain-containing protein; K02192 bacterioferritin-associated ferredoxin |  |
| ppg:PputGB1\_4330 | bacterioferritin; K03594 bacterioferritin |  |
| ppg:PputGB1\_4331 | glutaredoxin-like protein; K07390 monothiol glutaredoxin |  |
| ppg:PputGB1\_4332 | molydopterin dinucleotide-binding region |  |
| ppg:PputGB1\_4333 | ornithine carbamoyltransferase; K00611 ornithine carbamoyltransferase [EC:2.1.3.3] | ec:2.1.3.3 |
| ppg:PputGB1\_4334 | ABC transporter-like protein; K02010 iron(III) transport system ATP-binding protein [EC:3.6.3.30] | ec:3.6.3.30 |
| ppg:PputGB1\_4335 | ybaK/ebsC protein |  |
| ppg:PputGB1\_4336 | MIP family channel protein; K02440 glycerol uptake facilitator protein |  |
| ppg:PputGB1\_4337 | glpK; glycerol kinase; K00864 glycerol kinase [EC:2.7.1.30] | ec:2.7.1.30 |
| ppg:PputGB1\_4338 | DeoR family transcriptional regulator; K02444 DeoR family transcriptional regulator, glycerol-3-phosphate regulon repressor |  |
| ppg:PputGB1\_4339 | glpD; glycerol-3-phosphate dehydrogenase; K00111 glycerol-3-phosphate dehydrogenase [EC:1.1.5.3] | ec:1.1.5.3 |
| ppg:PputGB1\_4340 | hypothetical protein |  |
| ppg:PputGB1\_4341 | extracellular solute-binding protein; K10001 glutamate/aspartate transport system substrate-binding protein |  |
| ppg:PputGB1\_4342 | polar amino acid ABC transporter inner membrane subunit; K10003 glutamate/aspartate transport system permease protein |  |
| ppg:PputGB1\_4343 | polar amino acid ABC transporter inner membrane subunit; K10002 glutamate/aspartate transport system permease protein |  |
| ppg:PputGB1\_4344 | ABC transporter-like protein; K10004 glutamate/aspartate transport system ATP-binding protein [EC:3.6.3.-] |  |
| ppg:PputGB1\_4345 | integral membrane sensor signal transduction histidine kinase; K17060 two-component system, sensor histidine kinase AauS [EC:2.7.13.3] | ec:2.7.13.3 |
| ppg:PputGB1\_4346 | Fis family two component sigma-54 specific transcriptional regulator; K17061 two-component system, response regulator AauR |  |
| ppg:PputGB1\_4347 | GlpM family protein; K02442 membrane protein GlpM |  |
| ppg:PputGB1\_4348 | alpha/beta hydrolase fold family protein |  |

  
**Neighborhood Representations for "aav:Aave\_0603"**  

| ID | Annotation | EC number |
| --- | --- | --- |
| aav:Aave\_0593 | ATP-dependent DNA helicase RecG; K03655 ATP-dependent DNA helicase RecG [EC:3.6.4.12] | ec:3.6.4.12 |
| aav:Aave\_0594 | LysR family transcriptional regulator; K04761 LysR family transcriptional regulator, hydrogen peroxide-inducible genes activator |  |
| aav:Aave\_0595 | Ferritin, Dps family protein; K04047 starvation-inducible DNA-binding protein |  |
| aav:Aave\_0596 | 4-hydroxybenzoate octaprenyltransferase (EC:2.5.1.-); K03179 4-hydroxybenzoate octaprenyltransferase [EC:2.5.1.-] |  |
| aav:Aave\_0597 | hypothetical protein; K03478 hypothetical protein |  |
| aav:Aave\_0598 | GtrA family protein |  |
| aav:Aave\_0599 | glycosyl transferase family protein |  |
| aav:Aave\_0600 | PMT family 4-amino-4-deoxy-L-arabinose transferase/glycosyltransferase |  |
| aav:Aave\_0601 | pyrroline-5-carboxylate reductase (EC:1.5.1.2); K00286 pyrroline-5-carboxylate reductase [EC:1.5.1.2] | ec:1.5.1.2 |
| aav:Aave\_0602 | glycerol kinase; K00864 glycerol kinase [EC:2.7.1.30] | ec:2.7.1.30 |
| aav:Aave\_0603 | DeoR family transcriptional regulator; K02444 DeoR family transcriptional regulator, glycerol-3-phosphate regulon repressor |  |
| aav:Aave\_0604 | ABC transporter-like protein; K17324 glycerol transport system ATP-binding protein |  |
| aav:Aave\_0605 | ABC transporter-like protein; K17325 glycerol transport system ATP-binding protein |  |
| aav:Aave\_0606 | binding-protein-dependent transport systems inner membrane component; K17322 glycerol transport system permease protein |  |
| aav:Aave\_0607 | binding-protein-dependent transport system inner membrane protein; K17323 glycerol transport system permease protein |  |
| aav:Aave\_0608 | putative integral membrane protein |  |
| aav:Aave\_0609 | extracellular solute-binding protein; K17321 glycerol transport system substrate-binding protein |  |
| aav:Aave\_0610 | FAD dependent oxidoreductase; K00111 glycerol-3-phosphate dehydrogenase [EC:1.1.5.3] | ec:1.1.5.3 |
| aav:Aave\_0611 | rplN; 50S ribosomal protein L14; K02874 large subunit ribosomal protein L14 |  |
| aav:Aave\_0612 | rplX; 50S ribosomal protein L24; K02895 large subunit ribosomal protein L24 |  |
| aav:Aave\_0613 | rplE; 50S ribosomal protein L5; K02931 large subunit ribosomal protein L5 |  |

  
**Neighborhood Representations for "psb:Psyr\_3906"**  

| ID | Annotation | EC number |
| --- | --- | --- |
| psb:Psyr\_3896 | BFD-like (2Fe-2S)-binding protein; K02192 bacterioferritin-associated ferredoxin |  |
| psb:Psyr\_3897 | bacterioferritin; K03594 bacterioferritin |  |
| psb:Psyr\_3898 | glutaredoxin-like protein; K07390 monothiol glutaredoxin |  |
| psb:Psyr\_3899 | molybdopterin oxidoreductase |  |
| psb:Psyr\_3900 | hypothetical protein |  |
| psb:Psyr\_3901 | ornithine carbamoyltransferase (EC:2.1.3.3); K00611 ornithine carbamoyltransferase [EC:2.1.3.3] | ec:2.1.3.3 |
| psb:Psyr\_3902 | ABC transporter; K02010 iron(III) transport system ATP-binding protein [EC:3.6.3.30] | ec:3.6.3.30 |
| psb:Psyr\_3903 | hypothetical protein |  |
| psb:Psyr\_3904 | major intrinsic protein; K02440 glycerol uptake facilitator protein |  |
| psb:Psyr\_3905 | glpK; glycerol kinase (EC:2.7.1.30); K00864 glycerol kinase [EC:2.7.1.30] | ec:2.7.1.30 |
| psb:Psyr\_3906 | regulatory protein, DeoR; K02444 DeoR family transcriptional regulator, glycerol-3-phosphate regulon repressor |  |
| psb:Psyr\_3907 | glpD; glycerol-3-phosphate dehydrogenase (EC:1.1.5.3); K00111 glycerol-3-phosphate dehydrogenase [EC:1.1.5.3] | ec:1.1.5.3 |
| psb:Psyr\_3908 | extracellular solute-binding protein; K10001 glutamate/aspartate transport system substrate-binding protein |  |
| psb:Psyr\_3909 | amino acid ABC transporter permease; K10003 glutamate/aspartate transport system permease protein |  |
| psb:Psyr\_3910 | amino acid ABC transporter permease; K10002 glutamate/aspartate transport system permease protein |  |
| psb:Psyr\_3911 | ABC transporter; K10004 glutamate/aspartate transport system ATP-binding protein [EC:3.6.3.-] |  |
| psb:Psyr\_3912 | sensor histidine kinase; K17060 two-component system, sensor histidine kinase AauS [EC:2.7.13.3] | ec:2.7.13.3 |
| psb:Psyr\_3913 | helix-turn-helix, Fis-type; K17061 two-component system, response regulator AauR |  |
| psb:Psyr\_3914 | DSBA oxidoreductase |  |
| psb:Psyr\_3915 | Alpha/beta hydrolase fold |  |
| psb:Psyr\_3916 | 5-methyltetrahydropteroyltriglutamate/homocysteine S-methyltransferase (EC:2.1.1.14); K00549 5-methyltetrahydropteroyltriglutamate--homocysteine methyltransferase [EC:2.1.1.14] | ec:2.1.1.14 |

  
**Neighborhood Representations for "psp:PSPPH\_3900"**  

| ID | Annotation | EC number |
| --- | --- | --- |
| psp:PSPPH\_3890 | bacterioferritin-associated ferredoxin; K02192 bacterioferritin-associated ferredoxin |  |
| psp:PSPPH\_3891 | bfr1; bacterioferritin; K03594 bacterioferritin |  |
| psp:PSPPH\_3892 | glutaredoxin-like protein; K07390 monothiol glutaredoxin |  |
| psp:PSPPH\_3893 | molybdopterin-binding oxidoreductase (EC:1.7.99.4) |  |
| psp:PSPPH\_3894 | hypothetical protein |  |
| psp:PSPPH\_3895 | argF; ornithine carbamoyltransferase (EC:2.1.3.3); K00611 ornithine carbamoyltransferase [EC:2.1.3.3] | ec:2.1.3.3 |
| psp:PSPPH\_3896 | ABC transporter ATP-binding protein; K02010 iron(III) transport system ATP-binding protein [EC:3.6.3.30] | ec:3.6.3.30 |
| psp:PSPPH\_3897 | ybaK; ybaK/ebsC protein |  |
| psp:PSPPH\_3898 | glpF; glycerol uptake facilitator protein; K02440 glycerol uptake facilitator protein |  |
| psp:PSPPH\_3899 | glpK; glycerol kinase (EC:2.7.1.30); K00864 glycerol kinase [EC:2.7.1.30] | ec:2.7.1.30 |
| psp:PSPPH\_3900 | glpR; glycerol-3-phosphate regulon repressor; K02444 DeoR family transcriptional regulator, glycerol-3-phosphate regulon repressor |  |
| psp:PSPPH\_3901 | glpD; glycerol-3-phosphate dehydrogenase (EC:1.1.5.3); K00111 glycerol-3-phosphate dehydrogenase [EC:1.1.5.3] | ec:1.1.5.3 |
| psp:PSPPH\_3902 | amino acid ABC transporter substrate-binding protein; K10001 glutamate/aspartate transport system substrate-binding protein |  |
| psp:PSPPH\_3903 | amino acid ABC transporter permease; K10003 glutamate/aspartate transport system permease protein |  |
| psp:PSPPH\_3904 | amino acid ABC transporter permease; K10002 glutamate/aspartate transport system permease protein |  |
| psp:PSPPH\_3905 | amino acid ABC transporter ATP-binding protein; K10004 glutamate/aspartate transport system ATP-binding protein [EC:3.6.3.-] |  |
| psp:PSPPH\_3906 | sensor histidine kinase; K17060 two-component system, sensor histidine kinase AauS [EC:2.7.13.3] | ec:2.7.13.3 |
| psp:PSPPH\_3907 | Fis family transcriptional regulator; K17061 two-component system, response regulator AauR |  |
| psp:PSPPH\_3908 | 2-hydroxychromene-2-carboxylate isomerase |  |
| psp:PSPPH\_3909 | pseudogene |  |
| psp:PSPPH\_3910 | 5-methyltetrahydropteroyltriglutamate/homocysteine S-methyltransferase (EC:2.1.1.14); K00549 5-methyltetrahydropteroyltriglutamate--homocysteine methyltransferase [EC:2.1.1.14] | ec:2.1.1.14 |

  
**Neighborhood Representations for "reh:H16\_A2504"**  

| ID | Annotation | EC number |
| --- | --- | --- |
| reh:H16\_A2494 | h16\_A2494; hypothetical protein |  |
| reh:H16\_A2495 | h16\_A2495; hypothetical protein |  |
| reh:H16\_A2496 | h16\_A2496; amidase (EC:3.-.-.-) |  |
| reh:H16\_A2497 | h16\_A2497; signal transduction protein |  |
| reh:H16\_A2498 | h16\_A2498; ABC-type sugar transporter, periplasmic component; K17321 glycerol transport system substrate-binding protein |  |
| reh:H16\_A2499 | h16\_A2499; small integral membrane protein |  |
| reh:H16\_A2500 | h16\_A2500; ABC transporter permease; K17323 glycerol transport system permease protein |  |
| reh:H16\_A2501 | h16\_A2501; sugar ABC transporter permease; K17322 glycerol transport system permease protein |  |
| reh:H16\_A2502 | h16\_A2502; ABC transporter ATPase (EC:3.6.3.-); K17325 glycerol transport system ATP-binding protein |  |
| reh:H16\_A2503 | h16\_A2503; ABC transporter ATPase (EC:3.6.3.-); K17324 glycerol transport system ATP-binding protein |  |
| reh:H16\_A2504 | h16\_A2504; DeoR family transcriptional regulator; K02444 DeoR family transcriptional regulator, glycerol-3-phosphate regulon repressor |  |
| reh:H16\_A2505 | purU; formyltetrahydrofolate deformylase (EC:3.5.1.10); K01433 formyltetrahydrofolate deformylase [EC:3.5.1.10] | ec:3.5.1.10 |
| reh:H16\_A2506 | h16\_A2506; NTP pyrophosphohydrolase including oxidative damage repair enzymes (EC:2.7.6.2) |  |
| reh:H16\_A2507 | h16\_A2507; glycerol kinase (EC:2.7.1.30); K00864 glycerol kinase [EC:2.7.1.30] | ec:2.7.1.30 |
| reh:H16\_A2508 | h16\_A2508; glycerol-3-phosphate dehydrogenase (EC:1.1.5.3); K00111 glycerol-3-phosphate dehydrogenase [EC:1.1.5.3] | ec:1.1.5.3 |
| reh:H16\_A2509 | h16\_A2509; periplasmic protein |  |
| reh:H16\_A2510 | h16\_A2510; hypothetical protein |  |
| reh:H16\_A2511 | h16\_A2511; hypothetical protein |  |
| reh:H16\_A2512 | h16\_A2512; hypothetical protein |  |
| reh:H16\_A2513 | h16\_A2513; hypothetical protein |  |
| reh:H16\_A2514 | h16\_A2514; hypothetical protein |  |

  
**Neighborhood Representations for "dac:Daci\_1024"**  

| ID | Annotation | EC number |
| --- | --- | --- |
| dac:Daci\_1014 | ferritin Dps family protein; K04047 starvation-inducible DNA-binding protein |  |
| dac:Daci\_1015 | phosphoesterase PA-phosphatase-like protein |  |
| dac:Daci\_1016 | 4-hydroxybenzoate polyprenyltransferase; K03179 4-hydroxybenzoate octaprenyltransferase [EC:2.5.1.-] |  |
| dac:Daci\_1017 | pyrroline-5-carboxylate reductase (EC:1.5.1.2); K00286 pyrroline-5-carboxylate reductase [EC:1.5.1.2] | ec:1.5.1.2 |
| dac:Daci\_1018 | hypothetical protein |  |
| dac:Daci\_1019 | hypothetical protein |  |
| dac:Daci\_1020 | thiamine pyrophosphate-binding domain-containing protein; K01652 acetolactate synthase I/II/III large subunit [EC:2.2.1.6] | ec:2.2.1.6 |
| dac:Daci\_1021 | GntR family transcriptional regulator |  |
| dac:Daci\_1022 | hypothetical protein |  |
| dac:Daci\_1023 | glycerol kinase; K00864 glycerol kinase [EC:2.7.1.30] | ec:2.7.1.30 |
| dac:Daci\_1024 | DeoR family transcriptional regulator; K02444 DeoR family transcriptional regulator, glycerol-3-phosphate regulon repressor |  |
| dac:Daci\_1025 | ABC transporter-like protein; K17324 glycerol transport system ATP-binding protein |  |
| dac:Daci\_1026 | ABC transporter-like protein; K17325 glycerol transport system ATP-binding protein |  |
| dac:Daci\_1027 | binding-protein-dependent transport system inner membrane protein; K17322 glycerol transport system permease protein |  |
| dac:Daci\_1028 | binding-protein-dependent transport system inner membrane protein; K17323 glycerol transport system permease protein |  |
| dac:Daci\_1029 | putative integral membrane protein |  |
| dac:Daci\_1030 | extracellular solute-binding protein; K17321 glycerol transport system substrate-binding protein |  |
| dac:Daci\_1031 | FAD dependent oxidoreductase; K00111 glycerol-3-phosphate dehydrogenase [EC:1.1.5.3] | ec:1.1.5.3 |
| dac:Daci\_1032 | 50S ribosomal protein L14; K02874 large subunit ribosomal protein L14 |  |
| dac:Daci\_1033 | rplX; 50S ribosomal protein L24; K02895 large subunit ribosomal protein L24 |  |
| dac:Daci\_1034 | rplE; 50S ribosomal protein L5; K02931 large subunit ribosomal protein L5 |  |

  
**Neighborhood Representations for "asa:ASA\_2707"**  

| ID | Annotation | EC number |
| --- | --- | --- |
| asa:ASA\_2697 | hypothetical protein |  |
| asa:ASA\_2698 | hypothetical protein |  |
| asa:ASA\_2699 | hypothetical protein |  |
| asa:ASA\_2700 | eco; ecotin; K08276 ecotin |  |
| asa:ASA\_2701 | hypothetical protein; K09897 hypothetical protein |  |
| asa:ASA\_2702 | LysR family transcriptional regulator |  |
| asa:ASA\_2703 | tyrA; bifunctional chorismate mutase/prephenate dehydrogenase (EC:1.3.1.12 5.4.99.5); K14187 chorismate mutase / prephenate dehydrogenase [EC:5.4.99.5 1.3.1.12] | ec:5.4.99.5 ec:1.3.1.12 |
| asa:ASA\_2704 | aroF; phospho-2-dehydro-3-deoxyheptonate aldolase (EC:2.5.1.54); K01626 3-deoxy-7-phosphoheptulonate synthase [EC:2.5.1.54] | ec:2.5.1.54 |
| asa:ASA\_2705 | glpQ; glycerophosphodiester phosphodiesterase (EC:3.1.4.46); K01126 glycerophosphoryl diester phosphodiesterase [EC:3.1.4.46] | ec:3.1.4.46 |
| asa:ASA\_2706 | glpD; glycerol-3-phosphate dehydrogenase (EC:1.1.5.3); K00111 glycerol-3-phosphate dehydrogenase [EC:1.1.5.3] | ec:1.1.5.3 |
| asa:ASA\_2707 | glpR; glycerol-3-phosphate regulon repressor; K02444 DeoR family transcriptional regulator, glycerol-3-phosphate regulon repressor |  |
| asa:ASA\_2708 | glpT; sn-glycerol-3-phosphate transporter; K02445 MFS transporter, OPA family, glycerol-3-phosphate transporter |  |
| asa:ASA\_2709 | glpF; glycerol uptake facilitator; K02440 glycerol uptake facilitator protein |  |
| asa:ASA\_2710 | glpK; glycerol kinase; K00864 glycerol kinase [EC:2.7.1.30] | ec:2.7.1.30 |
| asa:ASA\_2711 | LysR family transcriptional regulator |  |
| asa:ASA\_2712 | hypothetical protein |  |
| asa:ASA\_2713 | hypothetical protein |  |
| asa:ASA\_2714 | intracellular protease/amidase |  |
| asa:ASA\_2715 | hypothetical protein; K06996 |  |
| asa:ASA\_2716 | antibiotic biosynthesis monooxygenase |  |
| asa:ASA\_2717 | dkgB; 2,5-diketo-D-gluconate reductase B (EC:1.1.1.274); K06222 2,5-diketo-D-gluconate reductase B [EC:1.1.1.346] | ec:1.1.1.346 |

  
**Neighborhood Representations for "gpb:HDN1F\_32230"**  

| ID | Annotation | EC number |
| --- | --- | --- |
| gpb:HDN1F\_32130 | hypothetical protein; K09966 hypothetical protein |  |
| gpb:HDN1F\_32140 | hypothetical protein |  |
| gpb:HDN1F\_32150 | Lon protease (S16) |  |
| gpb:HDN1F\_32160 | gltX; glutamyl-tRNA synthetase (EC:6.1.1.17); K01885 glutamyl-tRNA synthetase [EC:6.1.1.17] | ec:6.1.1.17 |
| gpb:HDN1F\_32170 | hypothetical protein |  |
| gpb:HDN1F\_32180 | hypothetical protein |  |
| gpb:HDN1F\_32190 | hypothetical protein |  |
| gpb:HDN1F\_32200 | Serine-pyruvate aminotransferase |  |
| gpb:HDN1F\_32210 | alcohol dehydrogenase |  |
| gpb:HDN1F\_32220 | glpD; glycerol-3-phosphate dehydrogenase; K00111 glycerol-3-phosphate dehydrogenase [EC:1.1.5.3] | ec:1.1.5.3 |
| gpb:HDN1F\_32230 | transcriptional Regulator, DeoR family; K02444 DeoR family transcriptional regulator, glycerol-3-phosphate regulon repressor |  |
| gpb:HDN1F\_32240 | glycerol kinase; K00864 glycerol kinase [EC:2.7.1.30] | ec:2.7.1.30 |
| gpb:HDN1F\_32250 | argF; ornithine carbamoyltransferase (EC:2.1.3.3) |  |
| gpb:HDN1F\_32260 | argD; acetylornithine aminotransferase (EC:2.6.1.11); K00818 acetylornithine aminotransferase [EC:2.6.1.11] | ec:2.6.1.11 |
| gpb:HDN1F\_32270 | hypothetical protein |  |
| gpb:HDN1F\_32280 | glutaredoxin-like protein; K07390 monothiol glutaredoxin |  |
| gpb:HDN1F\_32290 | hypothetical protein |  |
| gpb:HDN1F\_32300 | chemotaxis sensory transducer protein; K03406 methyl-accepting chemotaxis protein |  |
| gpb:HDN1F\_32310 | chemotaxis protein CheR; K00575 chemotaxis protein methyltransferase CheR [EC:2.1.1.80] | ec:2.1.1.80 |
| gpb:HDN1F\_32320 | penicillin amidase, peptidase S45; K01434 penicillin amidase [EC:3.5.1.11] | ec:3.5.1.11 |
| gpb:HDN1F\_32330 | asnS; asparaginyl-tRNA synthetase; K01893 asparaginyl-tRNA synthetase [EC:6.1.1.22] | ec:6.1.1.22 |

  
**Neighborhood Representations for "aha:AHA\_1651"**  

| ID | Annotation | EC number |
| --- | --- | --- |
| aha:AHA\_1641 | cydB-1; cytochrome D ubiquinol oxidase subunit II (EC:1.10.3.-); K00426 cytochrome d ubiquinol oxidase subunit II [EC:1.10.3.-] |  |
| aha:AHA\_1642 | cyd operon protein YbgT |  |
| aha:AHA\_1643 | major facilitator transporter |  |
| aha:AHA\_1644 | dkgB; 2,5-diketo-D-gluconate reductase B (EC:1.1.1.274); K06222 2,5-diketo-D-gluconate reductase B [EC:1.1.1.346] | ec:1.1.1.346 |
| aha:AHA\_1645 | antibiotic biosynthesis monooxygenase family protein |  |
| aha:AHA\_1646 | glyoxalase/dioxygenase superfamily protein; K06996 |  |
| aha:AHA\_1647 | HTH-type transcriptional regulator YafC |  |
| aha:AHA\_1648 | glpK; glycerol kinase (EC:2.7.1.30); K00864 glycerol kinase [EC:2.7.1.30] | ec:2.7.1.30 |
| aha:AHA\_1649 | glpF; glycerol uptake facilitator protein; K02440 glycerol uptake facilitator protein |  |
| aha:AHA\_1650 | glpT; sn-glycerol-3-phosphate transporter; K02445 MFS transporter, OPA family, glycerol-3-phosphate transporter |  |
| aha:AHA\_1651 | glpR; glycerol-3-phosphate regulon repressor; K02444 DeoR family transcriptional regulator, glycerol-3-phosphate regulon repressor |  |
| aha:AHA\_1652 | glpD; glycerol-3-phosphate dehydrogenase (EC:1.1.5.3); K00111 glycerol-3-phosphate dehydrogenase [EC:1.1.5.3] | ec:1.1.5.3 |
| aha:AHA\_1653 | glpQ; glycerophosphodiester phosphodiesterase (EC:3.1.4.46); K01126 glycerophosphoryl diester phosphodiesterase [EC:3.1.4.46] | ec:3.1.4.46 |
| aha:AHA\_1654 | phospho-2-dehydro-3-deoxyheptonate aldolase (EC:2.5.1.54); K01626 3-deoxy-7-phosphoheptulonate synthase [EC:2.5.1.54] | ec:2.5.1.54 |
| aha:AHA\_1655 | tyrA; bifunctional chorismate mutase/prephenate dehydrogenase (EC:1.3.1.12 5.4.99.5); K14187 chorismate mutase / prephenate dehydrogenase [EC:5.4.99.5 1.3.1.12] | ec:5.4.99.5 ec:1.3.1.12 |
| aha:AHA\_1656 | LysR family transcriptional regulator |  |
| aha:AHA\_1657 | hypothetical protein; K09897 hypothetical protein |  |
| aha:AHA\_1658 | ecotin; K08276 ecotin |  |
| aha:AHA\_1659 | hypothetical protein |  |
| aha:AHA\_1660 | hypothetical protein |  |
| aha:AHA\_1661 | proprotein convertase P-domain-containing protein |  |

  
**Neighborhood Representations for "bav:BAV2010"**  

| ID | Annotation | EC number |
| --- | --- | --- |
| bav:BAV2000 | phaD; putative monovalent cation/H+ antiporter subunit D; K05561 multicomponent K+:H+ antiporter subunit D |  |
| bav:BAV2001 | phaC; putative monovalent cation/H+ antiporter subunit C; K05560 multicomponent K+:H+ antiporter subunit C |  |
| bav:BAV2002 | phaAB; putative monovalent cation/H+ antiporter subunit A; K05559 multicomponent K+:H+ antiporter subunit A |  |
| bav:BAV2003 | GTP-binding protein |  |
| bav:BAV2004 | exonuclease |  |
| bav:BAV2005 | dadA2; D-amino acid dehydrogenase small subunit (EC:1.4.99.1); K00285 D-amino-acid dehydrogenase [EC:1.4.99.1] | ec:1.4.99.1 |
| bav:BAV2006 | lrp; leucine-responsive regulatory protein; K03719 Lrp/AsnC family transcriptional regulator, leucine-responsive regulatory protein |  |
| bav:BAV2007 | membrane protein |  |
| bav:BAV2008 | hypothetical protein |  |
| bav:BAV2009 | murA; UDP-N-acetylglucosamine 1-carboxyvinyltransferase (EC:2.5.1.7); K00790 UDP-N-acetylglucosamine 1-carboxyvinyltransferase [EC:2.5.1.7] | ec:2.5.1.7 |
| bav:BAV2010 | glpR; glycerol-3-phosphate regulon repressor; K02444 DeoR family transcriptional regulator, glycerol-3-phosphate regulon repressor |  |
| bav:BAV2011 | glpD; glycerol-3-phosphate dehydrogenase (EC:1.1.5.3); K00111 glycerol-3-phosphate dehydrogenase [EC:1.1.5.3] | ec:1.1.5.3 |
| bav:BAV2012 | hypothetical protein |  |
| bav:BAV2013 | hypothetical protein |  |
| bav:BAV2014 | hypothetical protein |  |
| bav:BAV2015 | hypothetical protein |  |
| bav:BAV2016 | membrane-associated protease; K07052 |  |
| bav:BAV2017 | peptidyl-tRNA hydrolase domain-containing protein; K15034 ribosome-associated protein |  |
| bav:BAV2018 | glycosyl transferase; K02843 heptosyltransferase II [EC:2.4.-.-] |  |
| bav:BAV2019 | hypothetical protein |  |
| bav:BAV2020 | hypothetical protein; K01737 6-pyruvoyltetrahydropterin/6-carboxytetrahydropterin synthase [EC:4.2.3.12 4.1.2.50] | ec:4.1.2.50 ec:4.2.3.12 |

  
**Neighborhood Representations for "pae:PA3583"**  

| ID | Annotation | EC number |
| --- | --- | --- |
| pae:PA3573 | major facilitator superfamily (MFS) transporter; K07552 MFS transporter, DHA1 family, bicyclomycin/chloramphenicol resistance protein |  |
| pae:PA3574 | nalD; NalD protein; K03577 TetR/AcrR family transcriptional regulator, acrAB operon repressor |  |
| pae:PA3575 | hypothetical protein |  |
| pae:PA3576 | hypothetical protein |  |
| pae:PA3577 | hypothetical protein |  |
| pae:PA3578 | hypothetical protein |  |
| pae:PA3579 | carbohydrate kinase (EC:2.7.1.30); K00864 glycerol kinase [EC:2.7.1.30] | ec:2.7.1.30 |
| pae:PA3580 | hypothetical protein |  |
| pae:PA3581 | glpF; glycerol uptake facilitator protein; K02440 glycerol uptake facilitator protein |  |
| pae:PA3582 | glpK; glycerol kinase (EC:2.7.1.30); K00864 glycerol kinase [EC:2.7.1.30] | ec:2.7.1.30 |
| pae:PA3583 | glpR; glycerol-3-phosphate regulon repressor; K02444 DeoR family transcriptional regulator, glycerol-3-phosphate regulon repressor |  |
| pae:PA3584 | glpD; glycerol-3-phosphate dehydrogenase (EC:1.1.5.3); K00111 glycerol-3-phosphate dehydrogenase [EC:1.1.5.3] | ec:1.1.5.3 |
| pae:PA3585 | glpM; membrane protein GlpM; K02442 membrane protein GlpM |  |
| pae:PA3586 | hydrolase |  |
| pae:PA3587 | metR; transcriptional regulator MetR; K03576 LysR family transcriptional regulator, regulator for metE and metH |  |
| pae:PA3588 | porin |  |
| pae:PA3589 | acetyl-CoA acetyltransferase (EC:2.3.1.9); K00626 acetyl-CoA C-acetyltransferase [EC:2.3.1.9] | ec:2.3.1.9 |
| pae:PA3590 | 3-hydroxyacyl-CoA dehydrogenase (EC:1.1.1.157); K00074 3-hydroxybutyryl-CoA dehydrogenase [EC:1.1.1.157] | ec:1.1.1.157 |
| pae:PA3591 | enoyl-CoA hydratase (EC:4.2.1.17) |  |
| pae:PA3592 | hypothetical protein |  |
| pae:PA3593 | acyl-CoA dehydrogenase |  |

  
**Neighborhood Representations for "pag:PLES\_14511"**  

| ID | Annotation | EC number |
| --- | --- | --- |
| pag:PLES\_14411 | putative acyl-CoA dehydrogenase |  |
| pag:PLES\_14421 | putative L-carnitine dehydratase |  |
| pag:PLES\_14431 | enoyl-CoA hydratase |  |
| pag:PLES\_14441 | 3-hydroxyacyl-CoA dehydrogenase; K00074 3-hydroxybutyryl-CoA dehydrogenase [EC:1.1.1.157] | ec:1.1.1.157 |
| pag:PLES\_14451 | acetyl-CoA acetyltransferase; K00626 acetyl-CoA C-acetyltransferase [EC:2.3.1.9] | ec:2.3.1.9 |
| pag:PLES\_14461 | putative porin |  |
| pag:PLES\_14471 | metR; transcriptional regulator MetR; K03576 LysR family transcriptional regulator, regulator for metE and metH |  |
| pag:PLES\_14481 | putative hydrolase |  |
| pag:PLES\_14491 | glpM; membrane protein GlpM; K02442 membrane protein GlpM |  |
| pag:PLES\_14501 | glpD; glycerol-3-phosphate dehydrogenase; K00111 glycerol-3-phosphate dehydrogenase [EC:1.1.5.3] | ec:1.1.5.3 |
| pag:PLES\_14511 | glpR; glycerol-3-phosphate regulon repressor; K02444 DeoR family transcriptional regulator, glycerol-3-phosphate regulon repressor |  |
| pag:PLES\_14521 | glpK; glycerol kinase; K00864 glycerol kinase [EC:2.7.1.30] | ec:2.7.1.30 |
| pag:PLES\_14531 | glpF; glycerol uptake facilitator protein; K02440 glycerol uptake facilitator protein |  |
| pag:PLES\_14541 | hypothetical protein |  |
| pag:PLES\_14551 | putative carbohydrate kinase; K00864 glycerol kinase [EC:2.7.1.30] | ec:2.7.1.30 |
| pag:PLES\_14561 | putative phenazine biosynthesis protein, PhzF family |  |
| pag:PLES\_14571 | hypothetical protein |  |
| pag:PLES\_14581 | putative cytochrome B561 |  |
| pag:PLES\_14591 | putative transcriptional regulator; K03577 TetR/AcrR family transcriptional regulator, acrAB operon repressor |  |
| pag:PLES\_14601 | putative major facilitator superfamily transporter; K07552 MFS transporter, DHA1 family, bicyclomycin/chloramphenicol resistance protein |  |
| pag:PLES\_14611 | hypothetical protein |  |

  
**Neighborhood Representations for "pap:PSPA7\_1560"**  

| ID | Annotation | EC number |
| --- | --- | --- |
| pap:PSPA7\_1550 | hypothetical protein |  |
| pap:PSPA7\_1551 | enoyl-CoA hydratase |  |
| pap:PSPA7\_1552 | 3-hydroxyacyl-CoA dehydrogenase; K00074 3-hydroxybutyryl-CoA dehydrogenase [EC:1.1.1.157] | ec:1.1.1.157 |
| pap:PSPA7\_1553 | acetyl-CoA acetyltransferase (EC:2.3.1.-); K00626 acetyl-CoA C-acetyltransferase [EC:2.3.1.9] | ec:2.3.1.9 |
| pap:PSPA7\_1554 | putative porin |  |
| pap:PSPA7\_1555 | metR; transcriptional regulator MetR; K03576 LysR family transcriptional regulator, regulator for metE and metH |  |
| pap:PSPA7\_1556 | metE; 5-methyltetrahydropteroyltriglutamate/homocysteine S-methyltransferase (EC:2.1.1.14); K00549 5-methyltetrahydropteroyltriglutamate--homocysteine methyltransferase [EC:2.1.1.14] | ec:2.1.1.14 |
| pap:PSPA7\_1557 | putative hydrolase |  |
| pap:PSPA7\_1558 | glpM; membrane protein GlpM; K02442 membrane protein GlpM |  |
| pap:PSPA7\_1559 | glpD; glycerol-3-phosphate dehydrogenase; K00111 glycerol-3-phosphate dehydrogenase [EC:1.1.5.3] | ec:1.1.5.3 |
| pap:PSPA7\_1560 | glpR; glycerol-3-phosphate regulon repressor; K02444 DeoR family transcriptional regulator, glycerol-3-phosphate regulon repressor |  |
| pap:PSPA7\_1561 | glpK; glycerol kinase (EC:2.7.1.30); K00864 glycerol kinase [EC:2.7.1.30] | ec:2.7.1.30 |
| pap:PSPA7\_1562 | glpF; glycerol uptake facilitator protein; K02440 glycerol uptake facilitator protein |  |
| pap:PSPA7\_1563 | hypothetical protein |  |
| pap:PSPA7\_1564 | glpK1; glycerol kinase (EC:2.7.1.30); K00864 glycerol kinase [EC:2.7.1.30] | ec:2.7.1.30 |
| pap:PSPA7\_1565 | PhzF family phenazine biosynthesis protein |  |
| pap:PSPA7\_1567 | hypothetical protein |  |
| pap:PSPA7\_1566 | hypothetical protein |  |
| pap:PSPA7\_1568 | hypothetical protein |  |
| pap:PSPA7\_1570 | hypothetical protein; K07213 copper chaperone |  |
| pap:PSPA7\_1569 | putative transcriptional regulator; K03577 TetR/AcrR family transcriptional regulator, acrAB operon repressor |  |

  
**Neighborhood Representations for "pau:PA14\_17940"**  

| ID | Annotation | EC number |
| --- | --- | --- |
| pau:PA14\_17810 | acyl-CoA dehydrogenase |  |
| pau:PA14\_17820 | hypothetical protein |  |
| pau:PA14\_17850 | fadB; enoyl-CoA hydratase (EC:4.2.1.17) |  |
| pau:PA14\_17860 | paaH; 3-hydroxyacyl-CoA dehydrogenase (EC:1.1.1.157); K00074 3-hydroxybutyryl-CoA dehydrogenase [EC:1.1.1.157] | ec:1.1.1.157 |
| pau:PA14\_17880 | paaJ; acetyl-CoA acetyltransferase (EC:2.3.1.9); K00626 acetyl-CoA C-acetyltransferase [EC:2.3.1.9] | ec:2.3.1.9 |
| pau:PA14\_17890 | porin |  |
| pau:PA14\_17900 | metR; transcriptional regulator MetR; K03576 LysR family transcriptional regulator, regulator for metE and metH |  |
| pau:PA14\_17910 | lipG; alpha/beta hydrolase |  |
| pau:PA14\_17920 | glpM; membrane protein GlpM; K02442 membrane protein GlpM |  |
| pau:PA14\_17930 | glpD; glycerol-3-phosphate dehydrogenase (EC:1.1.5.3); K00111 glycerol-3-phosphate dehydrogenase [EC:1.1.5.3] | ec:1.1.5.3 |
| pau:PA14\_17940 | glpR; glycerol-3-phosphate regulon repressor; K02444 DeoR family transcriptional regulator, glycerol-3-phosphate regulon repressor |  |
| pau:PA14\_17960 | glpK; glycerol kinase (EC:2.7.1.30); K00864 glycerol kinase [EC:2.7.1.30] | ec:2.7.1.30 |
| pau:PA14\_17980 | glpF; glycerol uptake facilitator protein; K02440 glycerol uptake facilitator protein |  |
| pau:PA14\_17990 | ybaK; hypothetical protein |  |
| pau:PA14\_18010 | glpK; glycerol kinase; K00864 glycerol kinase [EC:2.7.1.30] | ec:2.7.1.30 |
| pau:PA14\_18020 | PhzF family phenazine biosynthesis protein |  |
| pau:PA14\_18040 | hypothetical protein |  |
| pau:PA14\_18050 | hypothetical protein |  |
| pau:PA14\_18060 | hypothetical protein |  |
| pau:PA14\_18070 | periplasmic metal-binding protein; K07213 copper chaperone |  |
| pau:PA14\_18080 | TetR family transcriptional regulator; K03577 TetR/AcrR family transcriptional regulator, acrAB operon repressor |  |

  
**Neighborhood Representations for "pfs:PFLU1141"**  

| ID | Annotation | EC number |
| --- | --- | --- |
| pfs:PFLU1131 | putative two-component system sensor kinase |  |
| pfs:PFLU1132 | putative two-component system response regulator |  |
| pfs:PFLU1133 | hypothetical protein; K02442 membrane protein GlpM |  |
| pfs:PFLU1134 | putative two-component system response regulator; K17061 two-component system, response regulator AauR |  |
| pfs:PFLU1135 | putative two-component system sensor kinase; K17060 two-component system, sensor histidine kinase AauS [EC:2.7.13.3] | ec:2.7.13.3 |
| pfs:PFLU1136 | gltL; glutamate/aspartate ABC transporter ATP-binding protein; K10004 glutamate/aspartate transport system ATP-binding protein [EC:3.6.3.-] |  |
| pfs:PFLU1137 | gltK; glutamate/aspartate ABC transporter permease; K10002 glutamate/aspartate transport system permease protein |  |
| pfs:PFLU1138 | gltJ; glutamate/aspartate ABC transporter permease; K10003 glutamate/aspartate transport system permease protein |  |
| pfs:PFLU1139 | gltI; glutamate/aspartate ABC transporter periplasmic binding protein; K10001 glutamate/aspartate transport system substrate-binding protein |  |
| pfs:PFLU1140 | glpD; glycerol-3-phosphate dehydrogenase (EC:1.1.5.3); K00111 glycerol-3-phosphate dehydrogenase [EC:1.1.5.3] | ec:1.1.5.3 |
| pfs:PFLU1141 | glpR; glycerol-3-phosphate regulon repressor; K02444 DeoR family transcriptional regulator, glycerol-3-phosphate regulon repressor |  |
| pfs:PFLU1142 | glpK; glycerol kinase (EC:2.7.1.30); K00864 glycerol kinase [EC:2.7.1.30] | ec:2.7.1.30 |
| pfs:PFLU1143 | glpF; glycerol uptake facilitator protein; K02440 glycerol uptake facilitator protein |  |
| pfs:PFLU1144 | hypothetical protein |  |
| pfs:PFLU1145 | putative ABC transporter ATP-binding protein; K02010 iron(III) transport system ATP-binding protein [EC:3.6.3.30] | ec:3.6.3.30 |
| pfs:PFLU1146 | argF; ornithine carbamoyltransferase (EC:2.1.3.3); K00611 ornithine carbamoyltransferase [EC:2.1.3.3] | ec:2.1.3.3 |
| pfs:PFLU1148 | putative formate dehydrogenase |  |
| pfs:PFLU1149 | hypothetical protein; K07390 monothiol glutaredoxin |  |
| pfs:PFLU1150 | bfr1; bacterioferritin; K03594 bacterioferritin |  |
| pfs:PFLU1151 | bfd; bacterioferritin-associated ferredoxin; K02192 bacterioferritin-associated ferredoxin |  |
| pfs:PFLU1152 | putative alkyl hydroperoxide reductase; K03386 peroxiredoxin (alkyl hydroperoxide reductase subunit C) [EC:1.11.1.15] | ec:1.11.1.15 |

  
**Neighborhood Representations for "avn:Avin\_45720"**  

| ID | Annotation | EC number |
| --- | --- | --- |
| avn:Avin\_45620 | lipocalin-like protein; K03098 apolipoprotein D and lipocalin family protein |  |
| avn:Avin\_45630 | hypothetical protein |  |
| avn:Avin\_45640 | fbp; fructose-1,6-bisphosphatase; K03841 fructose-1,6-bisphosphatase I [EC:3.1.3.11] | ec:3.1.3.11 |
| avn:Avin\_45650 | glycogen phosphorylase; K00688 starch phosphorylase [EC:2.4.1.1] | ec:2.4.1.1 |
| avn:Avin\_45660 | hypothetical protein; K06940 |  |
| avn:Avin\_45670 | hypothetical protein |  |
| avn:Avin\_45680 | alkaline phosphatase |  |
| avn:Avin\_45690 | hypothetical protein |  |
| avn:Avin\_45700 | glpF; glycerol uptake facilitator protein; K02440 glycerol uptake facilitator protein |  |
| avn:Avin\_45710 | glpK; glycerol kinase; K00864 glycerol kinase [EC:2.7.1.30] | ec:2.7.1.30 |
| avn:Avin\_45720 | glpR; glycerol-3-phosphate regulon repressor protein GlpR; K02444 DeoR family transcriptional regulator, glycerol-3-phosphate regulon repressor |  |
| avn:Avin\_45730 | glpD; glycerol-3-phosphate dehydrogenase; K00111 glycerol-3-phosphate dehydrogenase [EC:1.1.5.3] | ec:1.1.5.3 |
| avn:Avin\_45740 | pseudogene |  |
| avn:Avin\_45750 | hypothetical protein |  |
| avn:Avin\_45760 | eno-4; enolase; K01689 enolase [EC:4.2.1.11] | ec:4.2.1.11 |
| avn:Avin\_45770 | tpiA-2; triosephosphate isomerase; K01803 triosephosphate isomerase (TIM) [EC:5.3.1.1] | ec:5.3.1.1 |
| avn:Avin\_45780 | aldehyde dehydrogenase |  |
| avn:Avin\_45790 | transposase IS116/IS110/IS902; K07486 transposase |  |
| avn:Avin\_45800 | transposase |  |
| avn:Avin\_45810 | hypothetical protein |  |
| avn:Avin\_65240 | IIC RNA structure |  |

  
**Neighborhood Representations for "ppw:PputW619\_1103"**  

| ID | Annotation | EC number |
| --- | --- | --- |
| ppw:PputW619\_1093 | LysR family transcriptional regulator; K03576 LysR family transcriptional regulator, regulator for metE and metH |  |
| ppw:PputW619\_1094 | alpha/beta hydrolase fold family protein |  |
| ppw:PputW619\_1095 | Fis family two component sigma-54 specific transcriptional regulator; K17061 two-component system, response regulator AauR |  |
| ppw:PputW619\_1096 | integral membrane sensor signal transduction histidine kinase; K17060 two-component system, sensor histidine kinase AauS [EC:2.7.13.3] | ec:2.7.13.3 |
| ppw:PputW619\_1097 | ABC transporter-like protein; K10004 glutamate/aspartate transport system ATP-binding protein [EC:3.6.3.-] |  |
| ppw:PputW619\_1098 | polar amino acid ABC transporter inner membrane subunit; K10002 glutamate/aspartate transport system permease protein |  |
| ppw:PputW619\_1099 | polar amino acid ABC transporter inner membrane subunit; K10003 glutamate/aspartate transport system permease protein |  |
| ppw:PputW619\_1100 | extracellular solute-binding protein; K10001 glutamate/aspartate transport system substrate-binding protein |  |
| ppw:PputW619\_1101 | hypothetical protein |  |
| ppw:PputW619\_1102 | glpD; glycerol-3-phosphate dehydrogenase; K00111 glycerol-3-phosphate dehydrogenase [EC:1.1.5.3] | ec:1.1.5.3 |
| ppw:PputW619\_1103 | DeoR family transcriptional regulator; K02444 DeoR family transcriptional regulator, glycerol-3-phosphate regulon repressor |  |
| ppw:PputW619\_1104 | glpK; glycerol kinase; K00864 glycerol kinase [EC:2.7.1.30] | ec:2.7.1.30 |
| ppw:PputW619\_1105 | MIP family channel protein; K02440 glycerol uptake facilitator protein |  |
| ppw:PputW619\_1106 | ybaK/ebsC protein |  |
| ppw:PputW619\_1107 | ABC transporter-like protein; K02010 iron(III) transport system ATP-binding protein [EC:3.6.3.30] | ec:3.6.3.30 |
| ppw:PputW619\_1108 | ornithine carbamoyltransferase; K00611 ornithine carbamoyltransferase [EC:2.1.3.3] | ec:2.1.3.3 |
| ppw:PputW619\_1109 | molydopterin dinucleotide-binding region |  |
| ppw:PputW619\_1110 | glutaredoxin-like protein; K07390 monothiol glutaredoxin |  |
| ppw:PputW619\_1111 | bacterioferritin; K03594 bacterioferritin |  |
| ppw:PputW619\_1112 | BFD/(2Fe-2S)-binding domain-containing protein; K02192 bacterioferritin-associated ferredoxin |  |
| ppw:PputW619\_1113 | alkyl hydroperoxide reductase; K03386 peroxiredoxin (alkyl hydroperoxide reductase subunit C) [EC:1.11.1.15] | ec:1.11.1.15 |

  
**Neighborhood Representations for "rme:Rmet\_2235"**  

| ID | Annotation | EC number |
| --- | --- | --- |
| rme:Rmet\_2226 | LysR family transcriptional regulator |  |
| rme:Rmet\_2227 | signal transduction protein (GGDEF domain) |  |
| rme:Rmet\_2228 | hypothetical protein |  |
| rme:Rmet\_6513 | hypothetical protein |  |
| rme:Rmet\_2229 | ABC-type sugar transporter periplasmic protein putative sugar binding; K17321 glycerol transport system substrate-binding protein |  |
| rme:Rmet\_2230 | putative small integral membrane protein |  |
| rme:Rmet\_2231 | ABC transporter permease; K17323 glycerol transport system permease protein |  |
| rme:Rmet\_2232 | ABC-type sugar transport systems permease; K17322 glycerol transport system permease protein |  |
| rme:Rmet\_2233 | ABC transporter ATPase; K17325 glycerol transport system ATP-binding protein |  |
| rme:Rmet\_2234 | ABC transporter ATPase; K17324 glycerol transport system ATP-binding protein |  |
| rme:Rmet\_2235 | glpR; DNA-binding transcriptional repressor; K02444 DeoR family transcriptional regulator, glycerol-3-phosphate regulon repressor |  |
| rme:Rmet\_2236 | purU; formyltetrahydrofolate deformylase (EC:3.5.1.10); K01433 formyltetrahydrofolate deformylase [EC:3.5.1.10] | ec:3.5.1.10 |
| rme:Rmet\_2237 | nucleoside diphosphate hydrolase protein (NUDIX) |  |
| rme:Rmet\_2238 | glpK; glycerol kinase (EC:2.7.1.30); K00864 glycerol kinase [EC:2.7.1.30] | ec:2.7.1.30 |
| rme:Rmet\_2239 | glpD; sn-glycerol-3-phosphate dehydrogenase, aerobic, FAD/NAD(P)-binding protein (EC:1.1.5.3); K00111 glycerol-3-phosphate dehydrogenase [EC:1.1.5.3] | ec:1.1.5.3 |
| rme:Rmet\_2240 | hypothetical protein |  |
| rme:Rmet\_2241 | hypothetical protein |  |
| rme:Rmet\_2242 | hypothetical protein |  |
| rme:Rmet\_2243 | hypothetical protein |  |
| rme:Rmet\_2244 | hypothetical protein |  |
| rme:Rmet\_2245 | putative diguanylate cyclase |  |

  
**Neighborhood Representations for "dia:Dtpsy\_0374"**  

| ID | Annotation | EC number |
| --- | --- | --- |
| dia:Dtpsy\_0364 | hypothetical protein |  |
| dia:Dtpsy\_0365 | s-adenosylmethionine/tRNA-ribosyltransferase-isomerase; K07568 S-adenosylmethionine:tRNA ribosyltransferase-isomerase [EC:2.4.99.17] | ec:2.4.99.17 |
| dia:Dtpsy\_0366 | metal dependent phosphohydrolase |  |
| dia:Dtpsy\_0367 | ATP-dependent DNA helicase recg; K03655 ATP-dependent DNA helicase RecG [EC:3.6.4.12] | ec:3.6.4.12 |
| dia:Dtpsy\_0368 | LysR family transcriptional regulator; K04761 LysR family transcriptional regulator, hydrogen peroxide-inducible genes activator |  |
| dia:Dtpsy\_0369 | ferritin dps family protein; K04047 starvation-inducible DNA-binding protein |  |
| dia:Dtpsy\_0370 | chad domain-containing protein |  |
| dia:Dtpsy\_0371 | 4-hydroxybenzoate polyprenyl transferase; K03179 4-hydroxybenzoate octaprenyltransferase [EC:2.5.1.-] |  |
| dia:Dtpsy\_0372 | pyrroline-5-carboxylate reductase (EC:1.5.1.2); K00286 pyrroline-5-carboxylate reductase [EC:1.5.1.2] | ec:1.5.1.2 |
| dia:Dtpsy\_0373 | glycerol kinase; K00864 glycerol kinase [EC:2.7.1.30] | ec:2.7.1.30 |
| dia:Dtpsy\_0374 | DeoR family transcriptional regulator; K02444 DeoR family transcriptional regulator, glycerol-3-phosphate regulon repressor |  |
| dia:Dtpsy\_0375 | ABC transporter; K17324 glycerol transport system ATP-binding protein |  |
| dia:Dtpsy\_0376 | ABC transporter; K17325 glycerol transport system ATP-binding protein |  |
| dia:Dtpsy\_0377 | hypothetical protein |  |
| dia:Dtpsy\_0378 | binding-protein-dependent transport systems inner membrane component; K17322 glycerol transport system permease protein |  |
| dia:Dtpsy\_0379 | binding-protein-dependent transport systems inner membrane component; K17323 glycerol transport system permease protein |  |
| dia:Dtpsy\_0380 | integral membrane protein |  |
| dia:Dtpsy\_0381 | extracellular solute-binding protein; K17321 glycerol transport system substrate-binding protein |  |
| dia:Dtpsy\_0382 | fad dependent oxidoreductase; K00111 glycerol-3-phosphate dehydrogenase [EC:1.1.5.3] | ec:1.1.5.3 |
| dia:Dtpsy\_0383 | rplN; 50S ribosomal protein L14; K02874 large subunit ribosomal protein L14 |  |
| dia:Dtpsy\_0384 | rplX; 50S ribosomal protein l24; K02895 large subunit ribosomal protein L24 |  |

  
**Neighborhood Representations for "pfl:PFL\_4869"**  

| ID | Annotation | EC number |
| --- | --- | --- |
| pfl:PFL\_4859 | bfr\_1; bacterioferritin; K03594 bacterioferritin |  |
| pfl:PFL\_4860 | grxD; monothiol glutaredoxin; K07390 monothiol glutaredoxin |  |
| pfl:PFL\_4861 | molybdopterin oxidoreductase |  |
| pfl:PFL\_4862 | hypothetical protein |  |
| pfl:PFL\_4863 | argF\_2; ornithine carbamoyltransferase (EC:2.1.3.3); K00611 ornithine carbamoyltransferase [EC:2.1.3.3] | ec:2.1.3.3 |
| pfl:PFL\_4864 | ABC transporter ATP-binding protein; K02010 iron(III) transport system ATP-binding protein [EC:3.6.3.30] | ec:3.6.3.30 |
| pfl:PFL\_4865 | PhzF family phenazine biosynthesis protein |  |
| pfl:PFL\_4866 | ybaK; Cys-tRNA[Pro] and Cys-tRNA[Cys] deacylase YbaK; K03976 putative transcription regulator |  |
| pfl:PFL\_4867 | glpF; glycerol uptake facilitator protein GlpF; K02440 glycerol uptake facilitator protein |  |
| pfl:PFL\_4868 | glpK; glycerol kinase (EC:2.7.1.30); K00864 glycerol kinase [EC:2.7.1.30] | ec:2.7.1.30 |
| pfl:PFL\_4869 | glpR; glycerol-3-phosphate regulon repressor; K02444 DeoR family transcriptional regulator, glycerol-3-phosphate regulon repressor |  |
| pfl:PFL\_4870 | glpD; glycerol-3-phosphate dehydrogenase (EC:1.1.5.3); K00111 glycerol-3-phosphate dehydrogenase [EC:1.1.5.3] | ec:1.1.5.3 |
| pfl:PFL\_4871 | gltI; glutamate/aspartate ABC transporter periplasmic glutamate/aspartate-binding protein GltI; K10001 glutamate/aspartate transport system substrate-binding protein |  |
| pfl:PFL\_4872 | gltJ; glutamate/aspartate ABC transporter permease GltJ; K10003 glutamate/aspartate transport system permease protein |  |
| pfl:PFL\_4873 | gltK; glutamate/aspartate ABC transporter permease GltK; K10002 glutamate/aspartate transport system permease protein |  |
| pfl:PFL\_4874 | gltL; glutamate/aspartate ABC transporter ATP-binding protein GltL; K10004 glutamate/aspartate transport system ATP-binding protein [EC:3.6.3.-] |  |
| pfl:PFL\_4875 | sensor histidine kinase; K17060 two-component system, sensor histidine kinase AauS [EC:2.7.13.3] | ec:2.7.13.3 |
| pfl:PFL\_4876 | Fis family transcriptional regulator; K17061 two-component system, response regulator AauR |  |
| pfl:PFL\_4877 | glpM; membrane protein GlpM; K02442 membrane protein GlpM |  |
| pfl:PFL\_4878 | hypothetical protein |  |
| pfl:PFL\_4879 | hypothetical protein |  |

  
**Neighborhood Representations for "pfo:Pfl01\_4533"**  

| ID | Annotation | EC number |
| --- | --- | --- |
| pfo:Pfl01\_4523 | bacterioferritin; K03594 bacterioferritin |  |
| pfo:Pfl01\_4524 | glutaredoxin-like protein; K07390 monothiol glutaredoxin |  |
| pfo:Pfl01\_4525 | molybdopterin oxidoreductase |  |
| pfo:Pfl01\_4526 | hypothetical protein |  |
| pfo:Pfl01\_4527 | ornithine carbamoyltransferase (EC:2.1.3.3); K00611 ornithine carbamoyltransferase [EC:2.1.3.3] | ec:2.1.3.3 |
| pfo:Pfl01\_4528 | ABC transporter-like protein; K02010 iron(III) transport system ATP-binding protein [EC:3.6.3.30] | ec:3.6.3.30 |
| pfo:Pfl01\_4529 | phenazine biosynthesis PhzC/PhzF protein |  |
| pfo:Pfl01\_4530 | hypothetical protein |  |
| pfo:Pfl01\_4531 | Aquaporin; K02440 glycerol uptake facilitator protein |  |
| pfo:Pfl01\_4532 | glpK; glycerol kinase (EC:2.7.1.30); K00864 glycerol kinase [EC:2.7.1.30] | ec:2.7.1.30 |
| pfo:Pfl01\_4533 | DeoR family transcriptional regulator; K02444 DeoR family transcriptional regulator, glycerol-3-phosphate regulon repressor |  |
| pfo:Pfl01\_4534 | glpD; glycerol-3-phosphate dehydrogenase (EC:1.1.5.3); K00111 glycerol-3-phosphate dehydrogenase [EC:1.1.5.3] | ec:1.1.5.3 |
| pfo:Pfl01\_4535 | extracellular solute-binding protein; K10001 glutamate/aspartate transport system substrate-binding protein |  |
| pfo:Pfl01\_4536 | amino acid ABC transporter permease; K10003 glutamate/aspartate transport system permease protein |  |
| pfo:Pfl01\_4537 | amino acid ABC transporter permease; K10002 glutamate/aspartate transport system permease protein |  |
| pfo:Pfl01\_4538 | ABC transporter-like protein; K10004 glutamate/aspartate transport system ATP-binding protein [EC:3.6.3.-] |  |
| pfo:Pfl01\_4539 | periplasmic sensor Signal transduction histidine kinase; K17060 two-component system, sensor histidine kinase AauS [EC:2.7.13.3] | ec:2.7.13.3 |
| pfo:Pfl01\_4540 | two component, sigma-54 specific, Fis family transcriptional regulator; K17061 two-component system, response regulator AauR |  |
| pfo:Pfl01\_4541 | GlpM; K02442 membrane protein GlpM |  |
| pfo:Pfl01\_4542 | hypothetical protein |  |
| pfo:Pfl01\_4543 | alpha/beta hydrolase fold protein |  |

  
**Neighborhood Representations for "pst:PSPTO\_4169"**  

| ID | Annotation | EC number |
| --- | --- | --- |
| pst:PSPTO\_4159 | bacterioferritin-associated ferredoxin; K02192 bacterioferritin-associated ferredoxin |  |
| pst:PSPTO\_4160 | bfr; bacterioferritin; K03594 bacterioferritin |  |
| pst:PSPTO\_4161 | glutaredoxin-like protein; K07390 monothiol glutaredoxin |  |
| pst:PSPTO\_4162 | oxidoreductase, molybdopterin-binding protein |  |
| pst:PSPTO\_4163 | hypothetical protein |  |
| pst:PSPTO\_4164 | argF; ornithine carbamoyltransferase; K00611 ornithine carbamoyltransferase [EC:2.1.3.3] | ec:2.1.3.3 |
| pst:PSPTO\_4165 | ABC transporter ATP-binding protein; K02010 iron(III) transport system ATP-binding protein [EC:3.6.3.30] | ec:3.6.3.30 |
| pst:PSPTO\_4166 | ybaK/ebsC protein |  |
| pst:PSPTO\_4167 | glpF; glycerol uptake facilitator protein; K02440 glycerol uptake facilitator protein |  |
| pst:PSPTO\_4168 | glpK; glycerol kinase; K00864 glycerol kinase [EC:2.7.1.30] | ec:2.7.1.30 |
| pst:PSPTO\_4169 | glpR; glycerol-3-phosphate regulon repressor; K02444 DeoR family transcriptional regulator, glycerol-3-phosphate regulon repressor |  |
| pst:PSPTO\_4170 | glpD; glycerol-3-phosphate dehydrogenase; K00111 glycerol-3-phosphate dehydrogenase [EC:1.1.5.3] | ec:1.1.5.3 |
| pst:PSPTO\_4171 | amino acid ABC transporter substrate-binding protein; K10001 glutamate/aspartate transport system substrate-binding protein |  |
| pst:PSPTO\_4172 | amino acid ABC transporter permease; K10003 glutamate/aspartate transport system permease protein |  |
| pst:PSPTO\_4173 | amino acid ABC transporter permease; K10002 glutamate/aspartate transport system permease protein |  |
| pst:PSPTO\_4174 | amino acid ABC transporter ATP-binding protein; K10004 glutamate/aspartate transport system ATP-binding protein [EC:3.6.3.-] |  |
| pst:PSPTO\_4175 | sensor histidine kinase; K17060 two-component system, sensor histidine kinase AauS [EC:2.7.13.3] | ec:2.7.13.3 |
| pst:PSPTO\_4176 | sigma-54 dependent transcriptional regulator/response regulator; K17061 two-component system, response regulator AauR |  |
| pst:PSPTO\_4177 | 2-hydroxychromene-2-carboxylate isomerase |  |
| pst:PSPTO\_4178 | alpha/beta fold family hydrolase |  |
| pst:PSPTO\_4179 | metE; 5-methyltetrahydropteroyltriglutamate/homocysteine S-methyltransferase; K00549 5-methyltetrahydropteroyltriglutamate--homocysteine methyltransferase [EC:2.1.1.14] | ec:2.1.1.14 |

  
**Neighborhood Representations for "rfr:Rfer\_3677"**  

| ID | Annotation | EC number |
| --- | --- | --- |
| rfr:Rfer\_3667 | ArsR family transcriptional regulator |  |
| rfr:Rfer\_3668 | hypothetical protein |  |
| rfr:Rfer\_3669 | nucleic acid-binding, OB-fold, tRNA/helicase-type |  |
| rfr:Rfer\_3670 | FAD dependent oxidoreductase; K00111 glycerol-3-phosphate dehydrogenase [EC:1.1.5.3] | ec:1.1.5.3 |
| rfr:Rfer\_3671 | extracellular solute-binding protein; K17321 glycerol transport system substrate-binding protein |  |
| rfr:Rfer\_3672 | putative integral membrane protein |  |
| rfr:Rfer\_3673 | binding-protein-dependent transport systems inner membrane component; K17323 glycerol transport system permease protein |  |
| rfr:Rfer\_3674 | binding-protein-dependent transport systems inner membrane component; K17322 glycerol transport system permease protein |  |
| rfr:Rfer\_3675 | ABC transporter-like protein; K17325 glycerol transport system ATP-binding protein |  |
| rfr:Rfer\_3676 | ABC transporter-like protein; K17324 glycerol transport system ATP-binding protein |  |
| rfr:Rfer\_3677 | DeoR family transcriptional regulator; K02444 DeoR family transcriptional regulator, glycerol-3-phosphate regulon repressor |  |
| rfr:Rfer\_3678 | glycerol kinase; K00864 glycerol kinase [EC:2.7.1.30] | ec:2.7.1.30 |
| rfr:Rfer\_3679 | pyrroline-5-carboxylate reductase (EC:1.5.1.2); K00286 pyrroline-5-carboxylate reductase [EC:1.5.1.2] | ec:1.5.1.2 |
| rfr:Rfer\_3680 | 4-hydroxybenzoate polyprenyl transferase; K03179 4-hydroxybenzoate octaprenyltransferase [EC:2.5.1.-] |  |
| rfr:Rfer\_3681 | LysR family transcriptional regulator; K04761 LysR family transcriptional regulator, hydrogen peroxide-inducible genes activator |  |
| rfr:Rfer\_3682 | hypothetical protein |  |
| rfr:Rfer\_3683 | metallophosphoesterase |  |
| rfr:Rfer\_3684 | periplasmic sensor signal transduction histidine kinase; K07637 two-component system, OmpR family, sensor histidine kinase PhoQ [EC:2.7.13.3] | ec:2.7.13.3 |
| rfr:Rfer\_3685 | two component transcriptional regulator; K07660 two-component system, OmpR family, response regulator PhoP |  |
| rfr:Rfer\_3686 | peptidase |  |
| rfr:Rfer\_3687 | 17 kDa surface antigen |  |

  
**Neighborhood Representations for "pmy:Pmen\_3347"**  

| ID | Annotation | EC number |
| --- | --- | --- |
| pmy:Pmen\_3337 | hypothetical protein |  |
| pmy:Pmen\_3338 | hypothetical protein; K09978 hypothetical protein |  |
| pmy:Pmen\_3339 | glutaredoxin-like protein; K07390 monothiol glutaredoxin |  |
| pmy:Pmen\_3340 | molydopterin dinucleotide-binding protein |  |
| pmy:Pmen\_3341 | hypothetical protein |  |
| pmy:Pmen\_3342 | ornithine carbamoyltransferase (EC:2.1.3.3); K00611 ornithine carbamoyltransferase [EC:2.1.3.3] | ec:2.1.3.3 |
| pmy:Pmen\_3343 | ABC transporter-like protein; K02010 iron(III) transport system ATP-binding protein [EC:3.6.3.30] | ec:3.6.3.30 |
| pmy:Pmen\_3344 | PhzF family phenazine biosynthesis protein |  |
| pmy:Pmen\_3345 | glycerol kinase; K00864 glycerol kinase [EC:2.7.1.30] | ec:2.7.1.30 |
| pmy:Pmen\_3346 | ybaK/ebsC protein |  |
| pmy:Pmen\_3347 | DeoR family transcriptional regulator; K02444 DeoR family transcriptional regulator, glycerol-3-phosphate regulon repressor |  |
| pmy:Pmen\_3348 | glpD; glycerol-3-phosphate dehydrogenase (EC:1.1.5.3); K00111 glycerol-3-phosphate dehydrogenase [EC:1.1.5.3] | ec:1.1.5.3 |
| pmy:Pmen\_3349 | bacterioferritin; K03594 bacterioferritin |  |
| pmy:Pmen\_3350 | acriflavin resistance protein |  |
| pmy:Pmen\_3351 | RND family efflux transporter MFP subunit |  |
| pmy:Pmen\_3352 | TetR family transcriptional regulator |  |
| pmy:Pmen\_3353 | hypothetical protein; K15984 16S rRNA (guanine1516-N2)-methyltransferase [EC:2.1.1.242] | ec:2.1.1.242 |
| pmy:Pmen\_3354 | extensin family protein |  |
| pmy:Pmen\_3355 | hypothetical protein |  |
| pmy:Pmen\_3356 | hypothetical protein |  |
| pmy:Pmen\_3357 | peptidase M22, glycoprotease; K14742 tRNA threonylcarbamoyladenosine biosynthesis protein TsaB |  |

  
**Neighborhood Representations for "ajs:Ajs\_0383"**  

| ID | Annotation | EC number |
| --- | --- | --- |
| ajs:Ajs\_0373 | hypothetical protein |  |
| ajs:Ajs\_0374 | S-adenosylmethionine--tRNA-ribosyltransferase-isomerase; K07568 S-adenosylmethionine:tRNA ribosyltransferase-isomerase [EC:2.4.99.17] | ec:2.4.99.17 |
| ajs:Ajs\_0375 | pseudogene |  |
| ajs:Ajs\_0376 | ATP-dependent DNA helicase RecG; K03655 ATP-dependent DNA helicase RecG [EC:3.6.4.12] | ec:3.6.4.12 |
| ajs:Ajs\_0377 | LysR family transcriptional regulator; K04761 LysR family transcriptional regulator, hydrogen peroxide-inducible genes activator |  |
| ajs:Ajs\_0378 | Ferritin, Dps family protein; K04047 starvation-inducible DNA-binding protein |  |
| ajs:Ajs\_0379 | adenylate cyclase |  |
| ajs:Ajs\_0380 | 4-hydroxybenzoate octaprenyltransferase (EC:2.5.1.-); K03179 4-hydroxybenzoate octaprenyltransferase [EC:2.5.1.-] |  |
| ajs:Ajs\_0381 | pyrroline-5-carboxylate reductase (EC:1.5.1.2); K00286 pyrroline-5-carboxylate reductase [EC:1.5.1.2] | ec:1.5.1.2 |
| ajs:Ajs\_0382 | glycerol kinase; K00864 glycerol kinase [EC:2.7.1.30] | ec:2.7.1.30 |
| ajs:Ajs\_0383 | DeoR family transcriptional regulator; K02444 DeoR family transcriptional regulator, glycerol-3-phosphate regulon repressor |  |
| ajs:Ajs\_0384 | ABC transporter-like protein; K17324 glycerol transport system ATP-binding protein |  |
| ajs:Ajs\_0385 | ABC transporter-like protein; K17325 glycerol transport system ATP-binding protein |  |
| ajs:Ajs\_0386 | binding-protein-dependent transport systems inner membrane component; K17322 glycerol transport system permease protein |  |
| ajs:Ajs\_0387 | binding-protein-dependent transport systems inner membrane component; K17323 glycerol transport system permease protein |  |
| ajs:Ajs\_0388 | putative integral membrane protein |  |
| ajs:Ajs\_0389 | extracellular solute-binding protein; K17321 glycerol transport system substrate-binding protein |  |
| ajs:Ajs\_0390 | FAD dependent oxidoreductase; K00111 glycerol-3-phosphate dehydrogenase [EC:1.1.5.3] | ec:1.1.5.3 |
| ajs:Ajs\_0391 | rplN; 50S ribosomal protein L14; K02874 large subunit ribosomal protein L14 |  |
| ajs:Ajs\_0392 | rplX; 50S ribosomal protein L24; K02895 large subunit ribosomal protein L24 |  |
| ajs:Ajs\_0393 | rplE; 50S ribosomal protein L5; K02931 large subunit ribosomal protein L5 |  |

  
**Neighborhood Representations for "dat:HRM2\_42290"**  

| ID | Annotation | EC number |
| --- | --- | --- |
| dat:HRM2\_42190 | pspA2; protein PspA2; K03969 phage shock protein A |  |
| dat:HRM2\_42200 | pspF; protein PspF; K03974 psp operon transcriptional activator |  |
| dat:HRM2\_42210 | dsbD; protein DsbD (EC:1.8.1.8); K06196 cytochrome c-type biogenesis protein |  |
| dat:HRM2\_42220 | putative thioredoxin |  |
| dat:HRM2\_42230 | hypothetical protein |  |
| dat:HRM2\_42240 | ppiB2; protein PpiB1 (EC:5.2.1.8); K03768 peptidyl-prolyl cis-trans isomerase B (cyclophilin B) [EC:5.2.1.8] | ec:5.2.1.8 |
| dat:HRM2\_42250 | hypothetical protein |  |
| dat:HRM2\_42260 | serB; SerB protein (EC:3.1.3.3); K01079 phosphoserine phosphatase [EC:3.1.3.3] | ec:3.1.3.3 |
| dat:HRM2\_42270 | hypothetical protein |  |
| dat:HRM2\_42280 | rocD2; protein RocD2 (EC:2.6.1.13) |  |
| dat:HRM2\_42290 | glpR2; protein GlpR2; K02444 DeoR family transcriptional regulator, glycerol-3-phosphate regulon repressor |  |
| dat:HRM2\_42300 | beta-lactamase-like:RNA-metabolising metallo-beta-lactamase; K07576 metallo-beta-lactamase family protein |  |
| dat:HRM2\_42310 | glpA3; protein GlpA3 (EC:1.1.5.3); K00111 glycerol-3-phosphate dehydrogenase [EC:1.1.5.3] | ec:1.1.5.3 |
| dat:HRM2\_42320 | glpB3; anaerobic glycerol-3-phosphate dehydrogenase subunit B (EC:1.1.5.3); K00112 glycerol-3-phosphate dehydrogenase subunit B [EC:1.1.5.3] | ec:1.1.5.3 |
| dat:HRM2\_42330 | recQ2; protein RecQ2 (EC:3.6.1.-); K03732 ATP-dependent RNA helicase RhlB [EC:3.6.4.13] | ec:3.6.4.13 |
| dat:HRM2\_42340 | tatA; twin arginine translocase protein A; K03117 sec-independent protein translocase protein TatB |  |
| dat:HRM2\_42350 | tatC; TatC; K03118 sec-independent protein translocase protein TatC |  |
| dat:HRM2\_42360 | cytochrome c, class III family protein |  |
| dat:HRM2\_42370 | cbiA2; protein CbiA2 (EC:6.3.5.9); K02224 cobyrinic acid a,c-diamide synthase [EC:6.3.5.9 6.3.5.11] | ec:6.3.5.11 ec:6.3.5.9 |
| dat:HRM2\_42380 | dsrD; protein DsrD |  |
| dat:HRM2\_42390 | dsrB2; protein DsrB2 (EC:1.8.99.3); K11181 sulfite reductase beta subunit [EC:1.8.99.3 1.8.99.1] | ec:1.8.99.1 ec:1.8.99.3 |

  
**Neighborhood Representations for "vvu:VV1\_1786"**  

| ID | Annotation | EC number |
| --- | --- | --- |
| vvu:VV1\_1774 | methyl-accepting chemotaxis protein; K03406 methyl-accepting chemotaxis protein |  |
| vvu:VV1\_1775 | Galactose operon repressor; K02529 LacI family transcriptional regulator |  |
| vvu:VV1\_1777 | tRNA-binding protein; K06878 tRNA-binding protein |  |
| vvu:VV1\_1779 | hypothetical protein |  |
| vvu:VV1\_1780 | hypothetical protein |  |
| vvu:VV1\_1781 | NnrS protein involved in response to NO; K07234 uncharacterized protein involved in response to NO |  |
| vvu:VV1\_1782 | glutathione synthase |  |
| vvu:VV1\_1783 | rimI; ribosomal-protein-alanine acetyltransferase (EC:2.3.1.128 2.3.1.-) |  |
| vvu:VV1\_1784 | Mg2+ and Co2+ transporter; K16074 zinc transporter |  |
| vvu:VV1\_1785 | glycerol-3-phosphate dehydrogenase (EC:1.1.5.3); K00111 glycerol-3-phosphate dehydrogenase [EC:1.1.5.3] | ec:1.1.5.3 |
| vvu:VV1\_1786 | glycerol-3-phosphate regulon repressor; K02444 DeoR family transcriptional regulator, glycerol-3-phosphate regulon repressor |  |
| vvu:VV1\_1787 | glpK; glycerol kinase (EC:2.7.1.30); K00864 glycerol kinase [EC:2.7.1.30] | ec:2.7.1.30 |
| vvu:VV1\_1788 | glycerol uptake facilitator protein; K02440 glycerol uptake facilitator protein |  |
| vvu:VV1\_1790 | 2,3,4,5-tetrahydropyridine-2,6-carboxylate N-succinyltransferase (EC:2.3.1.117); K00674 2,3,4,5-tetrahydropyridine-2-carboxylate N-succinyltransferase [EC:2.3.1.117] | ec:2.3.1.117 |
| vvu:VV1\_1791 | DNA damage-inducible protein; K09918 hypothetical protein |  |
| vvu:VV1\_1792 | LysR family transcriptional regulator |  |
| vvu:VV1\_1793 | Tellurite resistance protein |  |
| vvu:VV1\_1794 | hydrolase; K07014 |  |
| vvu:VV1\_1795 | recC; exonuclease V subunit gamma (EC:3.1.11.5); K03583 exodeoxyribonuclease V gamma subunit [EC:3.1.11.5] | ec:3.1.11.5 |
| vvu:VV1\_1796 | recB; exodeoxyribonuclease V subunit beta (EC:3.1.11.5); K03582 exodeoxyribonuclease V beta subunit [EC:3.1.11.5] | ec:3.1.11.5 |
| vvu:VV1\_1797 | recD; exodeoxyribonuclease V subunit alpha (EC:3.1.11.5); K03581 exodeoxyribonuclease V alpha subunit [EC:3.1.11.5] | ec:3.1.11.5 |

  
**Neighborhood Representations for "vvy:VV2625"**  

| ID | Annotation | EC number |
| --- | --- | --- |
| vvy:VV2615 | recC; exonuclease V subunit gamma (EC:3.1.11.5); K03583 exodeoxyribonuclease V gamma subunit [EC:3.1.11.5] | ec:3.1.11.5 |
| vvy:VV2616 | hydrolase; K07014 |  |
| vvy:VV2617 | tellurite resistance protein |  |
| vvy:VV2618 | transcriptional regulator |  |
| vvy:VV2619 | hypothetical protein; K09918 hypothetical protein |  |
| vvy:VV2620 | hypothetical protein |  |
| vvy:VV2621 | hypothetical protein |  |
| vvy:VV2622 | tetrahydrodipicolinate N-succinyltransferase; K00674 2,3,4,5-tetrahydropyridine-2-carboxylate N-succinyltransferase [EC:2.3.1.117] | ec:2.3.1.117 |
| vvy:VV2623 | glycerol uptake facilitator; K02440 glycerol uptake facilitator protein |  |
| vvy:VV2624 | glpK; glycerol kinase (EC:2.7.1.30); K00864 glycerol kinase [EC:2.7.1.30] | ec:2.7.1.30 |
| vvy:VV2625 | sugar metabolism transcriptional regulator; K02444 DeoR family transcriptional regulator, glycerol-3-phosphate regulon repressor |  |
| vvy:VV2626 | glpD; glycerol-3-phosphate dehydrogenase (EC:1.1.5.3); K00111 glycerol-3-phosphate dehydrogenase [EC:1.1.5.3] | ec:1.1.5.3 |
| vvy:VV2627 | Mg2+ and Co2+ transporter; K16074 zinc transporter |  |
| vvy:VV2628 | acetyltransferase |  |
| vvy:VV2629 | 30S ribosomal protein S6 modification protein |  |
| vvy:VV2630 | hypothetical protein; K07234 uncharacterized protein involved in response to NO |  |
| vvy:VV2631 | hypothetical protein |  |
| vvy:VV2632 | hypothetical protein; K06878 tRNA-binding protein |  |
| vvy:VV2634 | hypothetical protein |  |
| vvy:VV2633 | transcriptional regulator; K02529 LacI family transcriptional regulator |  |
| vvy:VV2635 | methyl-accepting chemotaxis protein; K03406 methyl-accepting chemotaxis protein |  |

  
**Neighborhood Representations for "ppr:PBPRA0159"**  

| ID | Annotation | EC number |
| --- | --- | --- |
| ppr:PBPRA0149 | hypothetical protein |  |
| ppr:PBPRA0150 | hypothetical protein; K05595 multiple antibiotic resistance protein |  |
| ppr:PBPRA0151 | hypothetical protein; K08993 putative membrane protein |  |
| ppr:PBPRA0152 | hypothetical protein; K08316 16S rRNA (guanine966-N2)-methyltransferase [EC:2.1.1.171] | ec:2.1.1.171 |
| ppr:PBPRA0153 | cell division protein FtsY; K03110 fused signal recognition particle receptor |  |
| ppr:PBPRA0154 | cell division protein FtsX; K09811 cell division transport system permease protein |  |
| ppr:PBPRA0155 | RNA polymerase factor sigma-32; K03089 RNA polymerase sigma-32 factor |  |
| ppr:PBPRA0156 | glpE; thiosulfate sulfurtransferase (EC:2.8.1.1); K02439 thiosulfate sulfurtransferase [EC:2.8.1.1] | ec:2.8.1.1 |
| ppr:PBPRA0157 | hypothetical protein; K02441 GlpG protein |  |
| ppr:PBPRA0158 | glpT; sn-glycerol-3-phosphate transporter; K02445 MFS transporter, OPA family, glycerol-3-phosphate transporter |  |
| ppr:PBPRA0159 | glycerol-3-phosphate repressor protein; K02444 DeoR family transcriptional regulator, glycerol-3-phosphate regulon repressor |  |
| ppr:PBPRA0160 | glpD; glycerol-3-phosphate dehydrogenase (EC:1.1.5.3); K00111 glycerol-3-phosphate dehydrogenase [EC:1.1.5.3] | ec:1.1.5.3 |
| ppr:PBPRA0161 | flagellar basal body protein FliL; K02415 flagellar FliL protein |  |
| ppr:PBPRA0162 | chorismate--pyruvate lyase; K03181 chorismate--pyruvate lyase [EC:4.1.3.40] | ec:4.1.3.40 |
| ppr:PBPRA0163 | ubiA; 4-hydroxybenzoate octaprenyltransferase; K03179 4-hydroxybenzoate octaprenyltransferase [EC:2.5.1.-] |  |
| ppr:PBPRA0164 | glycerol-3-phosphate acyltransferase (EC:2.3.1.15); K00631 glycerol-3-phosphate O-acyltransferase [EC:2.3.1.15] | ec:2.3.1.15 |
| ppr:PBPRA0165 | LexA repressor (EC:3.4.21.88); K01356 repressor LexA [EC:3.4.21.88] | ec:3.4.21.88 |
| ppr:PBPRA0166 | O-methyltransferase-relateprotein |  |
| ppr:PBPRA0167 | cytochrome c oxidase, subunit II; K02275 cytochrome c oxidase subunit II [EC:1.9.3.1] | ec:1.9.3.1 |
| ppr:PBPRA0168 | cytochrome c oxidase, subunit I; K02274 cytochrome c oxidase subunit I [EC:1.9.3.1] | ec:1.9.3.1 |
| ppr:PBPRA0169 | cytochrome C oxidase assembly protein; K02258 cytochrome c oxidase assembly protein subunit 11 |  |

  
**Neighborhood Representations for "ctt:CtCNB1\_4445"**  

| ID | Annotation | EC number |
| --- | --- | --- |
| ctt:CtCNB1\_4435 | 50S ribosomal protein L30; K02907 large subunit ribosomal protein L30 |  |
| ctt:CtCNB1\_4436 | 30S ribosomal protein S5; K02988 small subunit ribosomal protein S5 |  |
| ctt:CtCNB1\_4437 | 50S ribosomal protein L18; K02881 large subunit ribosomal protein L18 |  |
| ctt:CtCNB1\_4438 | 50S ribosomal protein L6; K02933 large subunit ribosomal protein L6 |  |
| ctt:CtCNB1\_4439 | 30S ribosomal protein S8; K02994 small subunit ribosomal protein S8 |  |
| ctt:CtCNB1\_4440 | 30S ribosomal protein S14; K02954 small subunit ribosomal protein S14 |  |
| ctt:CtCNB1\_4441 | 50S ribosomal protein L5; K02931 large subunit ribosomal protein L5 |  |
| ctt:CtCNB1\_4442 | 50S ribosomal protein L24; K02895 large subunit ribosomal protein L24 |  |
| ctt:CtCNB1\_4443 | 50S ribosomal protein L14; K02874 large subunit ribosomal protein L14 |  |
| ctt:CtCNB1\_4444 | FAD dependent oxidoreductase; K00111 glycerol-3-phosphate dehydrogenase [EC:1.1.5.3] | ec:1.1.5.3 |
| ctt:CtCNB1\_4445 | DeoR family transcriptional regulator; K02444 DeoR family transcriptional regulator, glycerol-3-phosphate regulon repressor |  |
| ctt:CtCNB1\_4446 | GCN5-like N-acetyltransferase |  |
| ctt:CtCNB1\_4447 | glycerol kinase; K00864 glycerol kinase [EC:2.7.1.30] | ec:2.7.1.30 |
| ctt:CtCNB1\_4448 | MIP family channel protein; K06188 aquaporin Z |  |
| ctt:CtCNB1\_4449 | hypothetical protein |  |
| ctt:CtCNB1\_4450 | hypothetical protein |  |
| ctt:CtCNB1\_4451 | pyrroline-5-carboxylate reductase; K00286 pyrroline-5-carboxylate reductase [EC:1.5.1.2] | ec:1.5.1.2 |
| ctt:CtCNB1\_4452 | 4-hydroxybenzoate polyprenyltransferase; K03179 4-hydroxybenzoate octaprenyltransferase [EC:2.5.1.-] |  |
| ctt:CtCNB1\_4453 | phosphoesterase |  |
| ctt:CtCNB1\_4454 | ferritin; K04047 starvation-inducible DNA-binding protein |  |
| ctt:CtCNB1\_4455 | LysR family transcriptional regulator; K04761 LysR family transcriptional regulator, hydrogen peroxide-inducible genes activator |  |

  
**Neighborhood Representations for "hel:HELO\_3002"**  

| ID | Annotation | EC number |
| --- | --- | --- |
| hel:HELO\_2992 | ung; uracil-DNA glycosylase (EC:3.2.2.-); K03648 uracil-DNA glycosylase [EC:3.2.2.27] | ec:3.2.2.27 |
| hel:HELO\_2993 | gpt; xanthine-guanine phosphoribosyltransferase (EC:2.4.2.22); K00769 xanthine phosphoribosyltransferase [EC:2.4.2.22] | ec:2.4.2.22 |
| hel:HELO\_2994 | apt; adenine phosphoribosyltransferase (EC:2.4.2.7); K00759 adenine phosphoribosyltransferase [EC:2.4.2.7] | ec:2.4.2.7 |
| hel:HELO\_2995 | NCS2 family transporter; K06901 putative MFS transporter, AGZA family, xanthine/uracil permease |  |
| hel:HELO\_2996 | ribE; riboflavin synthase subunit alpha (EC:2.5.1.9); K00793 riboflavin synthase [EC:2.5.1.9] | ec:2.5.1.9 |
| hel:HELO\_2997 | ABC transporter ATP-binding protein; K02071 D-methionine transport system ATP-binding protein |  |
| hel:HELO\_2998 | ABC transporter permease; K02072 D-methionine transport system permease protein |  |
| hel:HELO\_2999 | ABC transporter periplasmic protein; K02073 D-methionine transport system substrate-binding protein |  |
| hel:HELO\_3000 | GAF; GAF domain-containing protein; K07170 GAF domain-containing protein |  |
| hel:HELO\_3001 | glpK; glycerol kinase (EC:2.7.1.30); K00864 glycerol kinase [EC:2.7.1.30] | ec:2.7.1.30 |
| hel:HELO\_3002 | glpR; DNA-binding transcriptional repressor; K02444 DeoR family transcriptional regulator, glycerol-3-phosphate regulon repressor |  |
| hel:HELO\_3003 | glpD; glycerol-3-phosphate dehydrogenase (EC:1.1.5.3); K00111 glycerol-3-phosphate dehydrogenase [EC:1.1.5.3] | ec:1.1.5.3 |
| hel:HELO\_3004 | hypothetical protein; K15634 probable phosphoglycerate mutase [EC:5.4.2.12] | ec:5.4.2.12 |
| hel:HELO\_3005 | ABC transporter periplasmic protein; K02044 phosphonate transport system substrate-binding protein |  |
| hel:HELO\_3006 | ABC transporter ATP-binding protein; K02041 phosphonate transport system ATP-binding protein [EC:3.6.3.28] | ec:3.6.3.28 |
| hel:HELO\_3007 | ABC transporter permease; K02042 phosphonate transport system permease protein |  |
| hel:HELO\_3008 | ABC transporter permease; K02042 phosphonate transport system permease protein |  |
| hel:HELO\_3009 | endonuclease/exonuclease/phosphatase |  |
| hel:HELO\_3010 | ppc; phosphoenolpyruvate carboxylase (EC:4.1.1.31); K01595 phosphoenolpyruvate carboxylase [EC:4.1.1.31] | ec:4.1.1.31 |
| hel:HELO\_3011 | mazG; nucleoside triphosphate pyrophosphohydrolase (EC:3.6.1.19); K04765 ATP diphosphatase [EC:3.6.1.8] | ec:3.6.1.8 |
| hel:HELO\_3012 | relA; GTP pyrophosphokinase (EC:2.7.6.5); K00951 GTP pyrophosphokinase [EC:2.7.6.5] | ec:2.7.6.5 |

  
**Neighborhood Representations for "mmw:Mmwyl1\_3954"**  

| ID | Annotation | EC number |
| --- | --- | --- |
| mmw:Mmwyl1\_3944 | TRAP dicarboxylate transporter subunit DctP |  |
| mmw:Mmwyl1\_3945 | tripartite ATP-independent periplasmic transporter DctQ |  |
| mmw:Mmwyl1\_3946 | TRAP dicarboxylate transporter subunit DctM |  |
| mmw:Mmwyl1\_3947 | redoxin domain-containing protein |  |
| mmw:Mmwyl1\_3948 | methyl-accepting chemotaxis sensory transducer; K03406 methyl-accepting chemotaxis protein |  |
| mmw:Mmwyl1\_3949 | GntR family transcriptional regulator |  |
| mmw:Mmwyl1\_3950 | TonB-like protein |  |
| mmw:Mmwyl1\_3951 | hypothetical protein; K09009 hypothetical protein |  |
| mmw:Mmwyl1\_3952 | hypothetical protein |  |
| mmw:Mmwyl1\_3953 | glycerol kinase; K00864 glycerol kinase [EC:2.7.1.30] | ec:2.7.1.30 |
| mmw:Mmwyl1\_3954 | DeoR family transcriptional regulator; K02444 DeoR family transcriptional regulator, glycerol-3-phosphate regulon repressor |  |
| mmw:Mmwyl1\_3955 | glpD; glycerol-3-phosphate dehydrogenase; K00111 glycerol-3-phosphate dehydrogenase [EC:1.1.5.3] | ec:1.1.5.3 |
| mmw:Mmwyl1\_3956 | hypothetical protein; K07090 |  |
| mmw:Mmwyl1\_3957 | LysR family transcriptional regulator; K03566 LysR family transcriptional regulator, glycine cleavage system transcriptional activator |  |
| mmw:Mmwyl1\_3958 | glyceraldehyde-3-phosphate dehydrogenase (EC:1.2.1.12); K00134 glyceraldehyde 3-phosphate dehydrogenase [EC:1.2.1.12] | ec:1.2.1.12 |
| mmw:Mmwyl1\_3959 | hypothetical protein |  |
| mmw:Mmwyl1\_3960 | hypothetical protein |  |
| mmw:Mmwyl1\_3961 | phosphoribulokinase/uridine kinase; K00876 uridine kinase [EC:2.7.1.48] | ec:2.7.1.48 |
| mmw:Mmwyl1\_3962 | Rieske (2Fe-2S) domain-containing protein; K00479 Rieske 2Fe-2S family protein |  |
| mmw:Mmwyl1\_3963 | chaperone protein DnaJ; K03686 molecular chaperone DnaJ |  |
| mmw:Mmwyl1\_3964 | dnaK; molecular chaperone DnaK; K04043 molecular chaperone DnaK |  |

  
**Neighborhood Representations for "vex:VEA\_002674"**  

| ID | Annotation | EC number |
| --- | --- | --- |
| vex:VEA\_002664 | LacI-family regulatory protein; K02529 LacI family transcriptional regulator |  |
| vex:VEA\_002665 | hypothetical protein |  |
| vex:VEA\_002666 | galactose permease |  |
| vex:VEA\_002667 | Galactose operon repressor; K02529 LacI family transcriptional regulator |  |
| vex:VEA\_002668 | tRNA-binding protein YgjH; K06878 tRNA-binding protein |  |
| vex:VEA\_002669 | hypothetical protein |  |
| vex:VEA\_002670 | hypothetical protein |  |
| vex:VEA\_002671 | NnrS protein; K07234 uncharacterized protein involved in response to NO |  |
| vex:VEA\_002672 | Mg2+ and Co2+ transporter; K16074 zinc transporter |  |
| vex:VEA\_002673 | aerobic glycerol-3-phosphate dehydrogenase (EC:1.1.5.3); K00111 glycerol-3-phosphate dehydrogenase [EC:1.1.5.3] | ec:1.1.5.3 |
| vex:VEA\_002674 | glycerol-3-phosphate regulon repressor DeoR family; K02444 DeoR family transcriptional regulator, glycerol-3-phosphate regulon repressor |  |
| vex:VEA\_002675 | glycerol kinase (EC:2.7.1.30); K00864 glycerol kinase [EC:2.7.1.30] | ec:2.7.1.30 |
| vex:VEA\_002676 | glycerol uptake facilitator protein; K02440 glycerol uptake facilitator protein |  |
| vex:VEA\_002677 | glycerol-3-phosphate transporter; K02445 MFS transporter, OPA family, glycerol-3-phosphate transporter |  |
| vex:VEA\_002678 | glycerophosphoryl diester phosphodiesterase (EC:3.1.4.46); K01126 glycerophosphoryl diester phosphodiesterase [EC:3.1.4.46] | ec:3.1.4.46 |
| vex:VEA\_002679 | 2,3,4,5-tetrahydropyridine-2,6-carboxylate N-succinyltransferase (EC:2.3.1.117); K00674 2,3,4,5-tetrahydropyridine-2-carboxylate N-succinyltransferase [EC:2.3.1.117] | ec:2.3.1.117 |
| vex:VEA\_002680 | hypothetical protein; K09918 hypothetical protein |  |
| vex:VEA\_002681 | LysR family transcriptional regulator |  |
| vex:VEA\_002682 | tellurite resistance protein |  |
| vex:VEA\_002683 | hydrolase; K07014 |  |
| vex:VEA\_002684 | exodeoxyribonuclease V subunit gamma (EC:3.1.11.5); K03583 exodeoxyribonuclease V gamma subunit [EC:3.1.11.5] | ec:3.1.11.5 |

  
**Neighborhood Representations for "csa:Csal\_2105"**  

| ID | Annotation | EC number |
| --- | --- | --- |
| csa:Csal\_2095 | uracil phosphoribosyltransferase; K00761 uracil phosphoribosyltransferase [EC:2.4.2.9] | ec:2.4.2.9 |
| csa:Csal\_2096 | uracil-DNA glycosylase; K03648 uracil-DNA glycosylase [EC:3.2.2.27] | ec:3.2.2.27 |
| csa:Csal\_2097 | xanthine-guanine phosphoribosyltransferase; K00769 xanthine phosphoribosyltransferase [EC:2.4.2.22] | ec:2.4.2.22 |
| csa:Csal\_2098 | adenine phosphoribosyltransferase; K00759 adenine phosphoribosyltransferase [EC:2.4.2.7] | ec:2.4.2.7 |
| csa:Csal\_2099 | riboflavin synthase subunit alpha; K00793 riboflavin synthase [EC:2.5.1.9] | ec:2.5.1.9 |
| csa:Csal\_2100 | ABC transporter-like protein; K02071 D-methionine transport system ATP-binding protein |  |
| csa:Csal\_2101 | binding-protein-dependent transport system inner membrane protein; K02072 D-methionine transport system permease protein |  |
| csa:Csal\_2102 | lipoprotein YaeC; K02072 D-methionine transport system permease protein K02073 D-methionine transport system substrate-binding protein |  |
| csa:Csal\_2103 | putative GAF sensor protein; K07170 GAF domain-containing protein |  |
| csa:Csal\_2104 | glycerol kinase; K00864 glycerol kinase [EC:2.7.1.30] | ec:2.7.1.30 |
| csa:Csal\_2105 | DeoR family transcriptional regulator; K02444 DeoR family transcriptional regulator, glycerol-3-phosphate regulon repressor |  |
| csa:Csal\_2106 | glpD; glycerol-3-phosphate dehydrogenase; K00111 glycerol-3-phosphate dehydrogenase [EC:1.1.5.3] | ec:1.1.5.3 |
| csa:Csal\_2107 | hypothetical protein |  |
| csa:Csal\_2108 | fumC; fumarate hydratase; K01679 fumarate hydratase, class II [EC:4.2.1.2] | ec:4.2.1.2 |
| csa:Csal\_2109 | hypothetical protein |  |
| csa:Csal\_2110 | regulatory inactivation of DnaA Hda protein; K10763 DnaA-homolog protein |  |
| csa:Csal\_2111 | hypothetical protein |  |
| csa:Csal\_2112 | phosphoribosylformylglycinamidine cyclo-ligase; K01933 phosphoribosylformylglycinamidine cyclo-ligase [EC:6.3.3.1] | ec:6.3.3.1 |
| csa:Csal\_2113 | phosphoribosylglycinamide formyltransferase; K11175 phosphoribosylglycinamide formyltransferase 1 [EC:2.1.2.2] | ec:2.1.2.2 |
| csa:Csal\_2114 | dcd; deoxycytidine triphosphate deaminase; K01494 dCTP deaminase [EC:3.5.4.13] | ec:3.5.4.13 |
| csa:Csal\_2115 | ParA family protein; K03593 ATP-binding protein involved in chromosome partitioning |  |

  
**Neighborhood Representations for "vha:VIBHAR\_03314"**  

| ID | Annotation | EC number |
| --- | --- | --- |
| vha:VIBHAR\_03304 | hypothetical protein |  |
| vha:VIBHAR\_03305 | transcriptional regulator |  |
| vha:VIBHAR\_03306 | hypothetical protein; K09918 hypothetical protein |  |
| vha:VIBHAR\_03307 | hypothetical protein |  |
| vha:VIBHAR\_03308 | hypothetical protein |  |
| vha:VIBHAR\_03309 | 2,3,4,5-tetrahydropyridine-2,6-carboxylate N-succinyltransferase; K00674 2,3,4,5-tetrahydropyridine-2-carboxylate N-succinyltransferase [EC:2.3.1.117] | ec:2.3.1.117 |
| vha:VIBHAR\_03310 | glpQ; glycerophosphodiester phosphodiesterase; K01126 glycerophosphoryl diester phosphodiesterase [EC:3.1.4.46] | ec:3.1.4.46 |
| vha:VIBHAR\_03311 | glpT; sn-glycerol-3-phosphate transporter; K02445 MFS transporter, OPA family, glycerol-3-phosphate transporter |  |
| vha:VIBHAR\_03312 | hypothetical protein; K02440 glycerol uptake facilitator protein |  |
| vha:VIBHAR\_03313 | glpK; glycerol kinase; K00864 glycerol kinase [EC:2.7.1.30] | ec:2.7.1.30 |
| vha:VIBHAR\_03314 | transcriptional regulator; K02444 DeoR family transcriptional regulator, glycerol-3-phosphate regulon repressor |  |
| vha:VIBHAR\_03315 | glpD; glycerol-3-phosphate dehydrogenase; K00111 glycerol-3-phosphate dehydrogenase [EC:1.1.5.3] | ec:1.1.5.3 |
| vha:VIBHAR\_03316 | lytic murein transglycosylase; K08306 membrane-bound lytic murein transglycosylase C [EC:3.2.1.-] |  |
| vha:VIBHAR\_03317 | hypothetical protein; K07234 uncharacterized protein involved in response to NO |  |
| vha:VIBHAR\_03318 | hypothetical protein |  |
| vha:VIBHAR\_03319 | hypothetical protein |  |
| vha:VIBHAR\_03320 | hypothetical protein |  |
| vha:VIBHAR\_03321 | hypothetical protein; K06878 tRNA-binding protein |  |
| vha:VIBHAR\_03322 | hypothetical protein |  |
| vha:VIBHAR\_03323 | hypothetical protein |  |
| vha:VIBHAR\_03324 | PTS system, glucose-specific IIBC component |  |

  
**Neighborhood Representations for "vpa:VP2387"**  

| ID | Annotation | EC number |
| --- | --- | --- |
| vpa:VP2377 | hypothetical protein |  |
| vpa:VP2378 | LysR family transcriptional regulator |  |
| vpa:VP2379 | DNA damage-inducible gene in SOS regulon, dependent on cyclic AMP and H-NS; K09918 hypothetical protein |  |
| vpa:VP2380 | 2,3,4,5-tetrahydropyridine-2,6-carboxylate N-succinyltransferase; K00674 2,3,4,5-tetrahydropyridine-2-carboxylate N-succinyltransferase [EC:2.3.1.117] | ec:2.3.1.117 |
| vpa:VP2381 | glpQ; glycerophosphodiester phosphodiesterase (EC:3.1.4.46); K01126 glycerophosphoryl diester phosphodiesterase [EC:3.1.4.46] | ec:3.1.4.46 |
| vpa:VP2382 | glpT; sn-glycerol-3-phosphate transporter; K02445 MFS transporter, OPA family, glycerol-3-phosphate transporter |  |
| vpa:VP2383 | hypothetical protein |  |
| vpa:VP2384 | hypothetical protein |  |
| vpa:VP2385 | glycerol uptake facilitator protein GlpF; K02440 glycerol uptake facilitator protein |  |
| vpa:VP2386 | glpK; glycerol kinase (EC:2.7.1.30); K00864 glycerol kinase [EC:2.7.1.30] | ec:2.7.1.30 |
| vpa:VP2387 | DeoR family transcriptional regulator; K02444 DeoR family transcriptional regulator, glycerol-3-phosphate regulon repressor |  |
| vpa:VP2388 | glpD; glycerol-3-phosphate dehydrogenase (EC:1.1.5.3); K00111 glycerol-3-phosphate dehydrogenase [EC:1.1.5.3] | ec:1.1.5.3 |
| vpa:VP2389 | membrane transport protein; K16074 zinc transporter |  |
| vpa:VP2390 | hypothetical protein; K07234 uncharacterized protein involved in response to NO |  |
| vpa:VP2391 | hypothetical protein |  |
| vpa:VP2392 | hypothetical protein; K06878 tRNA-binding protein |  |
| vpa:VP2393 | LacI family transcription regulator; K02529 LacI family transcriptional regulator |  |
| vpa:VP2394 | sodium:galactoside symporter family protein |  |
| vpa:VP2395 | hypothetical protein |  |
| vpa:VP2396 | LacI-family regulatory protein; K02529 LacI family transcriptional regulator |  |
| vpa:VP2397 | galM; aldose 1-epimerase (EC:5.1.3.3); K01785 aldose 1-epimerase [EC:5.1.3.3] | ec:5.1.3.3 |

  
**Neighborhood Representations for "vcm:VCM66\_A0900"**  

| ID | Annotation | EC number |
| --- | --- | --- |
| vcm:VCM66\_A0888 | hypothetical protein |  |
| vcm:VCM66\_A0889 | rumB; 23S rRNA methyluridine methyltransferase; K03212 23S rRNA (uracil747-C5)-methyltransferase [EC:2.1.1.189] | ec:2.1.1.189 |
| vcm:VCM66\_A0890 | hypothetical protein; K09781 hypothetical protein |  |
| vcm:VCM66\_A0891 | hypothetical protein |  |
| vcm:VCM66\_A0893 | cold shock domain family protein; K03704 cold shock protein (beta-ribbon, CspA family) |  |
| vcm:VCM66\_A0895 | hypothetical protein |  |
| vcm:VCM66\_A0896 | hypothetical protein |  |
| vcm:VCM66\_A0897 | araC family transcriptional regulator |  |
| vcm:VCM66\_A0898 | hypothetical protein |  |
| vcm:VCM66\_A0899 | sensory box/GGDEF family protein |  |
| vcm:VCM66\_A0900 | transcriptional regulator, DeoR family; K02444 DeoR family transcriptional regulator, glycerol-3-phosphate regulon repressor |  |
| vcm:VCM66\_A0901 | acyl-CoA thioester hydrolase-related protein |  |
| vcm:VCM66\_A0902 | hypothetical protein |  |
| vcm:VCM66\_A0903 | malG; maltose transporter permease; K10110 maltose/maltodextrin transport system permease protein |  |
| vcm:VCM66\_A0904 | malF; maltose transporter membrane protein; K10109 maltose/maltodextrin transport system permease protein |  |
| vcm:VCM66\_A0905 | malE; maltose ABC transporter substrate-binding protein; K10108 maltose/maltodextrin transport system substrate-binding protein |  |
| vcm:VCM66\_A0906 | malK; maltose/maltodextrin transporter ATP-binding protein; K10111 multiple sugar transport system ATP-binding protein [EC:3.6.3.-] |  |
| vcm:VCM66\_A0907 | speG; spermidine n1-acetyltransferase (EC:2.3.1.57); K00657 diamine N-acetyltransferase [EC:2.3.1.57] | ec:2.3.1.57 |
| vcm:VCM66\_A0908 | hypothetical protein |  |
| vcm:VCM66\_A0909 | SM-20-like protein; K07394 SM-20-related protein |  |
| vcm:VCM66\_A0911 | hypothetical protein |  |

  
**Neighborhood Representations for "vco:VC0395\_0299"**  

| ID | Annotation | EC number |
| --- | --- | --- |
| vco:VC0395\_0289 | SM-20-related protein; K07394 SM-20-related protein |  |
| vco:VC0395\_0290 | hypothetical protein |  |
| vco:VC0395\_0291 | speG; spermidine n1-acetyltransferase (EC:2.3.1.57); K00657 diamine N-acetyltransferase [EC:2.3.1.57] | ec:2.3.1.57 |
| vco:VC0395\_0292 | malK; maltose/maltodextrin transporter ATP-binding protein; K10111 multiple sugar transport system ATP-binding protein [EC:3.6.3.-] |  |
| vco:VC0395\_0293 | hypothetical protein |  |
| vco:VC0395\_0294 | malE; maltose ABC transporter periplasmic protein; K10108 maltose/maltodextrin transport system substrate-binding protein |  |
| vco:VC0395\_0295 | malF; maltose transporter membrane protein; K10109 maltose/maltodextrin transport system permease protein |  |
| vco:VC0395\_0296 | malG; maltose transporter permease; K10110 maltose/maltodextrin transport system permease protein |  |
| vco:VC0395\_0297 | hypothetical protein |  |
| vco:VC0395\_0298 | acyl-CoA thioester hydrolase-related protein |  |
| vco:VC0395\_0299 | DeoR family transcriptional regulator; K02444 DeoR family transcriptional regulator, glycerol-3-phosphate regulon repressor |  |
| vco:VC0395\_0300 | sensory box/GGDEF family protein |  |
| vco:VC0395\_0301 | hypothetical protein |  |
| vco:VC0395\_0303 | AraC/XylS family transcriptional regulator |  |
| vco:VC0395\_0302 | hypothetical protein |  |
| vco:VC0395\_0304 | hypothetical protein |  |
| vco:VC0395\_0305 | hypothetical protein |  |
| vco:VC0395\_0306 | cold shock domain-contain protein; K03704 cold shock protein (beta-ribbon, CspA family) |  |
| vco:VC0395\_0307 | hypothetical protein |  |
| vco:VC0395\_0308 | hypothetical protein; K09781 hypothetical protein |  |
| vco:VC0395\_0309 | rumB; 23S rRNA methyluridine methyltransferase; K03212 23S rRNA (uracil747-C5)-methyltransferase [EC:2.1.1.189] | ec:2.1.1.189 |

  
**Neighborhood Representations for "vch:VCA0940"**  

| ID | Annotation | EC number |
| --- | --- | --- |
| vch:VCA0928 | hypothetical protein |  |
| vch:VCA0929 | rumB; 23S rRNA methyluridine methyltransferase; K03212 23S rRNA (uracil747-C5)-methyltransferase [EC:2.1.1.189] | ec:2.1.1.189 |
| vch:VCA0930 | hypothetical protein; K09781 hypothetical protein |  |
| vch:VCA0931 | hypothetical protein |  |
| vch:VCA0933 | cold shock domain-contain protein; K03704 cold shock protein (beta-ribbon, CspA family) |  |
| vch:VCA0935 | hypothetical protein |  |
| vch:VCA0936 | hypothetical protein |  |
| vch:VCA0937 | AraC family transcriptional regulator |  |
| vch:VCA0938 | pseudogene |  |
| vch:VCA0939 | sensory box/GGDEF family protein |  |
| vch:VCA0940 | DeoR family transcriptional regulator; K02444 DeoR family transcriptional regulator, glycerol-3-phosphate regulon repressor |  |
| vch:VCA0941 | acyl-CoA thioester hydrolase |  |
| vch:VCA0942 | hypothetical protein |  |
| vch:VCA0943 | malG; maltose transporter permease; K10110 maltose/maltodextrin transport system permease protein |  |
| vch:VCA0944 | malF; maltose transporter membrane protein; K10109 maltose/maltodextrin transport system permease protein |  |
| vch:VCA0945 | malE; maltose ABC transporter substrate-binding protein; K10108 maltose/maltodextrin transport system substrate-binding protein |  |
| vch:VCA0946 | maltose/maltodextrin transporter ATP-binding protein; K10111 multiple sugar transport system ATP-binding protein [EC:3.6.3.-] |  |
| vch:VCA0947 | spermidine n1-acetyltransferase; K00657 diamine N-acetyltransferase [EC:2.3.1.57] | ec:2.3.1.57 |
| vch:VCA0948 | hypothetical protein |  |
| vch:VCA0949 | SM-20-like protein; K07394 SM-20-related protein |  |
| vch:VCA0951 | hypothetical protein |  |

  
**Neighborhood Representations for "vcj:VCD\_000394"**  

| ID | Annotation | EC number |
| --- | --- | --- |
| vcj:VCD\_000384 | hypothetical protein |  |
| vcj:VCD\_000385 | SM-20-related protein; K07394 SM-20-related protein |  |
| vcj:VCD\_000386 | transcriptional antiterminator |  |
| vcj:VCD\_000387 | spermidine N1-acetyltransferase (EC:2.3.1.57); K00657 diamine N-acetyltransferase [EC:2.3.1.57] | ec:2.3.1.57 |
| vcj:VCD\_000388 | maltose/maltodextrin transporter ATP-binding protein (EC:3.6.3.19); K10111 multiple sugar transport system ATP-binding protein [EC:3.6.3.-] |  |
| vcj:VCD\_000389 | malE; maltose ABC transporter periplasmic protein; K10108 maltose/maltodextrin transport system substrate-binding protein |  |
| vcj:VCD\_000390 | malF; maltose transporter membrane protein; K10109 maltose/maltodextrin transport system permease protein |  |
| vcj:VCD\_000391 | malG; maltose transporter permease; K10110 maltose/maltodextrin transport system permease protein |  |
| vcj:VCD\_000392 | hypothetical protein |  |
| vcj:VCD\_000393 | acyl-CoA hydrolase (EC:3.1.2.20) |  |
| vcj:VCD\_000394 | glycerol-3-phosphate regulon repressor DeoR family; K02444 DeoR family transcriptional regulator, glycerol-3-phosphate regulon repressor |  |
| vcj:VCD\_000395 | sensory box/GGDEF family protein |  |
| vcj:VCD\_000396 | hypothetical protein |  |
| vcj:VCD\_000397 | AraC family transcriptional regulator |  |
| vcj:VCD\_000398 | hypothetical protein |  |
| vcj:VCD\_000399 | hypothetical protein |  |
| vcj:VCD\_000400 | cold shock protein CspA; K03704 cold shock protein (beta-ribbon, CspA family) |  |
| vcj:VCD\_000401 | response regulator |  |
| vcj:VCD\_000402 | hypothetical protein; K09781 hypothetical protein |  |
| vcj:VCD\_000403 | rumB; 23S rRNA methyluridine methyltransferase; K03212 23S rRNA (uracil747-C5)-methyltransferase [EC:2.1.1.189] | ec:2.1.1.189 |
| vcj:VCD\_000404 | hypothetical protein |  |

  
**Neighborhood Representations for "vsp:VS\_II0114"**  

| ID | Annotation | EC number |
| --- | --- | --- |
| vsp:VS\_II0104 | plasmid-related protein |  |
| vsp:VS\_II0105 | dihydrofolate reductase |  |
| vsp:VS\_II0106 | transcriptional regulator |  |
| vsp:VS\_II0107 | phospholipase D |  |
| vsp:VS\_II0108 | nitroreductase A; K10678 nitroreductase [EC:1.-.-.-] |  |
| vsp:VS\_II0109 | NADH oxidase |  |
| vsp:VS\_II0110 | LysR family transcriptional regulator |  |
| vsp:VS\_II0111 | hypothetical protein |  |
| vsp:VS\_II0112 | hypothetical protein; K09794 hypothetical protein |  |
| vsp:VS\_II0113 | glpD; glycerol-3-phosphate dehydrogenase; K00111 glycerol-3-phosphate dehydrogenase [EC:1.1.5.3] | ec:1.1.5.3 |
| vsp:VS\_II0114 | Transcriptional regulator, DeoR family; K02444 DeoR family transcriptional regulator, glycerol-3-phosphate regulon repressor |  |
| vsp:VS\_II0115 | glpK; glycerol kinase; K00864 glycerol kinase [EC:2.7.1.30] | ec:2.7.1.30 |
| vsp:VS\_II0116 | Glycerol uptake facilitator; K02440 glycerol uptake facilitator protein |  |
| vsp:VS\_II0117 | hypothetical protein |  |
| vsp:VS\_II0118 | Hypothetical transcriptional regulator |  |
| vsp:VS\_II0119 | heavy metal membrane efflux protein; K03325 arsenite transporter, ACR3 family |  |
| vsp:VS\_II0120 | regulatory protein |  |
| vsp:VS\_II0121 | hydrolase; K07024 |  |
| vsp:VS\_II0122 | DSBA oxidoreductase |  |
| vsp:VS\_II0123 | hypothetical protein; K09927 hypothetical protein |  |
| vsp:VS\_II0124 | LysR family transcriptional regulator |  |

  
**Neighborhood Representations for "xbo:XBJ1\_0140"**  

| ID | Annotation | EC number |
| --- | --- | --- |
| xbo:XBJ1\_0130 | yhfS; PLP-dependent tansferase domain-containing protein |  |
| xbo:XBJ1\_0131 | yhfT; transporter |  |
| xbo:XBJ1\_0132 | hypothetical protein |  |
| xbo:XBJ1\_0133 | phosphotriesterase with metallo-dependent hydrolase domain (EC:3.1.8.1); K07048 phosphotriesterase-related protein |  |
| xbo:XBJ1\_0134 | hypothetical protein |  |
| xbo:XBJ1\_0135 | hypothetical protein |  |
| xbo:XBJ1\_0136 | hypothetical protein |  |
| xbo:XBJ1\_0137 | GNAT family acetyltransferase |  |
| xbo:XBJ1\_0138 | glpD; sn-glycerol-3-phosphate dehydrogenase FAD/NAD(P)-binding (aerobic) (EC:1.1.5.3); K00111 glycerol-3-phosphate dehydrogenase [EC:1.1.5.3] | ec:1.1.5.3 |
| xbo:XBJ1\_0139 | hypothetical protein |  |
| xbo:XBJ1\_0140 | glpR; DeoR family transcriptional regulator; K02444 DeoR family transcriptional regulator, glycerol-3-phosphate regulon repressor |  |
| xbo:XBJ1\_0141 | glpG; hypothetical protein; K02441 GlpG protein |  |
| xbo:XBJ1\_0142 | glpE; thiosulfate:cyanide sulfurtransferase (EC:2.8.1.1); K02439 thiosulfate sulfurtransferase [EC:2.8.1.1] | ec:2.8.1.1 |
| xbo:XBJ1\_0143 | nfuA; Fe/S biogenesis protein; K07400 Fe/S biogenesis protein NfuA |  |
| xbo:XBJ1\_0144 | gntX; periplasmic gluconate-binding protein in GNT I transport system |  |
| xbo:XBJ1\_0145 | bioH; hypothetical protein; K02170 pimeloyl-[acyl-carrier protein] methyl ester esterase [EC:3.1.1.85] | ec:3.1.1.85 |
| xbo:XBJ1\_0146 | hypothetical protein |  |
| xbo:XBJ1\_0147 | Synaptotagmin-1 (Synaptotagmin I) (p65) (fragment) |  |
| xbo:XBJ1\_0148 | insertion element IS1 1/2/3/5/6 protein insA (IS1a/IS1b/IS1c/IS1d) |  |
| xbo:XBJ1\_0149 | insertion element iso-IS1n protein insB; K07480 insertion element IS1 protein InsB |  |
| xbo:XBJ1\_0150 | transposase |  |

  
**Over-represented Enzyme Summary**: Table of E.C. identified protein in the "Neighborhood Representation" ranked by frequency of occurrence  

| EC number | Frequency | Annotation | Reactions |
| --- | --- | --- | --- |
| ec:1.1.5.3 | 97 | glycerol-3-phosphate dehydrogenase; alpha-glycerophosphate dehydrogenase; alpha-glycerophosphate dehydrogenase (acceptor); anaerobic glycerol-3-phosphate dehydrogenase; DL-glycerol 3-phosphate oxidase (misleading); FAD-dependent glycerol-3-phosphate dehydrogenase; FAD-dependent sn-glycerol-3-phosphate dehydrogenase; FAD-GPDH; FAD-linked glycerol 3-phosphate dehydrogenase; FAD-linked L-glycerol-3-phosphate dehydrogenase; flavin-linked glycerol-3-phosphate dehydrogenase; flavoprotein-linked L-glycerol 3-phosphate dehydrogenase; glycerol 3-phosphate cytochrome c reductase (misleading); glycerol phosphate dehydrogenase; glycerol phosphate dehydrogenase (acceptor); glycerol phosphate dehydrogenase (FAD); glycerol-3-phosphate CoQ reductase; glycerol-3-phosphate dehydrogenase (flavin-linked); glycerol-3-phosphate:CoQ reductase; glycerophosphate dehydrogenase; L-3-glycerophosphate-ubiquinone oxidoreductase; L-glycerol-3-phosphate dehydrogenase (ambiguous); L-glycerophosphate dehydrogenase; mGPD; mitochondrial glycerol phosphate dehydrogenase; NAD+-independent glycerol phosphate dehydrogenase; pyridine nucleotide-independent L-glycerol 3-phosphate dehydrogenase; sn-glycerol 3-phosphate oxidase (misleading); sn-glycerol-3-phosphate dehydrogenase; sn-glycerol-3-phosphate:(acceptor) 2-oxidoreductase; sn-glycerol-3-phosphate:acceptor 2-oxidoreductase | sn-glycerol 3-phosphate + a quinone = glycerone phosphate + a quinol [RN:R00849] |
| ec:2.7.1.30 | 89 | glycerol kinase; glycerokinase; GK; ATP:glycerol-3-phosphotransferase; glycerol kinase (phosphorylating); glyceric kinase | ATP + glycerol = ADP + sn-glycerol 3-phosphate [RN:R00847] |
| ec:3.6.4.13 | 29 | RNA helicase; CSFV NS3 helicase; DBP2; DbpA; DDX17; DDX25; DDX3; DDX3X; DDX3Y; DDX4; DDX5; DEAD-box protein DED1; DEAD-box RNA helicase; DEAH-box protein 2; DEAH-box RNA helicase; DED1; Dex(H/D) RNA helicase; EhDEAD1; EhDEAD1 RNA helicase; eIF4A helicase; KOKV helicase; Mtr4p; nonstructural protein 3 helicase; NPH-II; RHA; RNA helicase A; RNA helicase DDX3; RNA helicase Hera; RNA-dependent ATPase; TGBp1 NTPase/helicase domain; VRH1; GRTH/DDX25 | ATP + H2O = ADP + phosphate [RN:R00086] |
| ec:2.3.2.2 | 23 | gamma-glutamyltransferase; glutamyl transpeptidase; alpha-glutamyl transpeptidase; gamma-glutamyl peptidyltransferase; gamma-glutamyl transpeptidase (ambiguous); gamma-GPT; gamma-GT; gamma-GTP; L-gamma-glutamyl transpeptidase; L-gamma-glutamyltransferase; L-glutamyltransferase; GGT (ambiguous); gamma-glutamyltranspeptidase (ambiguous) | a (5-L-glutamyl)-peptide + an amino acid = a peptide + a 5-L-glutamyl amino acid [RN:R04159] |
| ec:4.2.1.6 | 22 | galactonate dehydratase; D-galactonate dehydrase; D-galactonate dehydratase; D-galactonate hydro-lyase | D-galactonate = 2-dehydro-3-deoxy-D-galactonate + H2O [RN:R03033] |
| ec:2.5.1.19 | 17 | 3-phosphoshikimate 1-carboxyvinyltransferase; 5-enolpyruvylshikimate-3-phosphate synthase; 3-enolpyruvylshikimate 5-phosphate synthase; 3-enolpyruvylshikimic acid-5-phosphate synthetase; 5'-enolpyruvylshikimate-3-phosphate synthase; 5-enolpyruvyl-3-phosphoshikimate synthase; 5-enolpyruvylshikimate-3-phosphate synthetase; 5-enolpyruvylshikimate-3-phosphoric acid synthase; enolpyruvylshikimate phosphate synthase; EPSP synthase | phosphoenolpyruvate + 3-phosphoshikimate = phosphate + 5-O-(1-carboxyvinyl)-3-phosphoshikimate [RN:R03460] |
| ec:3.6.3.30 | 12 | Fe3+-transporting ATPase | ATP + H2O + Fe3+out = ADP + phosphate + Fe3+in [RN:R00086] |
| ec:2.7.13.3 | 12 | histidine kinase; EnvZ; histidine kinase (ambiguous); histidine protein kinase (ambiguous); protein histidine kinase (ambiguous); protein kinase (histidine) (ambiguous); HK1; HP165; Sln1p | ATP + protein L-histidine = ADP + protein N-phospho-L-histidine |
| ec:2.1.3.3 | 12 | ornithine carbamoyltransferase; citrulline phosphorylase; ornithine transcarbamylase; OTC; carbamylphosphate-ornithine transcarbamylase; L-ornithine carbamoyltransferase; L-ornithine carbamyltransferase; L-ornithine transcarbamylase; ornithine carbamyltransferase | carbamoyl phosphate + L-ornithine = phosphate + L-citrulline [RN:R01398] |
| ec:2.8.3.12 | 10 | glutaconate CoA-transferase | acetyl-CoA + (E)-glutaconate = acetate + glutaconyl-1-CoA [RN:R03884] |
| ec:1.14.13.82 | 10 | vanillate monooxygenase; 4-hydroxy-3-methoxybenzoate demethylase; vanillate demethylase | vanillate + O2 + NADH + H+ = 3,4-dihydroxybenzoate + NAD+ + H2O + formaldehyde [RN:R05274] |
| ec:1.13.11.3 | 10 | protocatechuate 3,4-dioxygenase; protocatechuate oxygenase; protocatechuic acid oxidase; protocatechuic 3,4-dioxygenase; protocatechuic 3,4-oxygenase | 3,4-dihydroxybenzoate + O2 = 3-carboxy-cis,cis-muconate [RN:R01631] |
| ec:4.1.99.12 | 9 | 3,4-dihydroxy-2-butanone-4-phosphate synthase; DHBP synthase; L-3,4-dihydroxybutan-2-one-4-phosphate synthase | D-ribulose 5-phosphate = formate + L-3,4-dihydroxybutan-2-one 4-phosphate [RN:R07281] |
| ec:1.18.1.3 | 8 | ferredoxin---NAD+ reductase; ferredoxin-nicotinamide adenine dinucleotide reductase; ferredoxin reductase (ambiguous); NAD+-ferredoxin reductase; NADH-ferredoxin oxidoreductase; reductase, reduced nicotinamide adenine dinucleotide-ferredoxin; ferredoxin-NAD+ reductase; NADH-ferredoxin reductase; NADH2-ferredoxin oxidoreductase; NADH flavodoxin oxidoreductase; NADH-ferredoxin NAP reductase (component of naphthalene dioxygenase multicomponent enzyme system); ferredoxin-linked NAD+ reductase; NADH-ferredoxin TOL reductase (component of toluene dioxygenase); ferredoxin---NAD reductase | (1) 2 reduced [2Fe-2S] ferredoxin + NAD+ + H+ = 2 oxidized [2Fe-2S] ferredoxin + NADH [RN:R05875]; (2) reduced 2[4Fe-4S] ferredoxin + NAD+ + H+ = oxidized 2[4Fe-4S] ferredoxin + NADH |
| ec:1.5.1.2 | 7 | pyrroline-5-carboxylate reductase; proline oxidase; L-proline oxidase; 1-pyrroline-5-carboxylate reductase; NADPH-L-Delta1-pyrroline carboxylic acid reductase; L-proline-NAD(P)+ 5-oxidoreductase | L-proline + NAD(P)+ = 1-pyrroline-5-carboxylate + NAD(P)H + H+ [RN:R01248 R01251] |
| ec:6.3.5.4 | 7 | asparagine synthase (glutamine-hydrolysing); asparagine synthetase (glutamine-hydrolysing); glutamine-dependent asparagine synthetase; asparagine synthetase B; AS; AS-B | ATP + L-aspartate + L-glutamine + H2O = AMP + diphosphate + L-asparagine + L-glutamate (overall reaction) [RN:R00578]; (1a) L-glutamine + H2O = L-glutamate + NH3 [RN:R00256]; (1b) ATP + L-aspartate + NH3 = AMP + diphosphate + L-asparagine [RN:R00483] |
| ec:3.1.4.46 | 6 | glycerophosphodiester phosphodiesterase; gene hpd protein; glycerophosphoryl diester phosphodiesterase; IgD-binding protein D | a glycerophosphodiester + H2O = an alcohol + sn-glycerol 3-phosphate [RN:R00857] |
| ec:1.11.1.15 | 6 | peroxiredoxin; thioredoxin peroxidase; tryparedoxin peroxidase; alkyl hydroperoxide reductase C22; AhpC; TrxPx; TXNPx; Prx; PRDX | 2 R'-SH + ROOH = R'-S-S-R' + H2O + ROH [RN:R07180] |
| ec:3.1.11.5 | 5 | exodeoxyribonuclease V; Escherichia coli exonuclease V; E. coli exonuclease V; gene recBC endoenzyme; RecBC deoxyribonuclease; gene recBC DNase; exonuclease V; gene recBCD enzymes | Exonucleolytic cleavage (in the presence of ATP) in either 5'- to 3'- or 3'- to 5'-direction to yield 5'-phosphooligonucleotides |
| ec:1.14.13.2 | 5 | 4-hydroxybenzoate 3-monooxygenase; p-hydroxybenzoate hydrolyase; p-hydroxybenzoate hydroxylase; 4-hydroxybenzoate 3-hydroxylase; 4-hydroxybenzoate monooxygenase; 4-hydroxybenzoic hydroxylase; p-hydroxybenzoate-3-hydroxylase; p-hydroxybenzoic acid hydrolase; p-hydroxybenzoic acid hydroxylase; p-hydroxybenzoic hydroxylase | 4-hydroxybenzoate + NADPH + H+ + O2 = protocatechuate + NADP+ + H2O [RN:R01298] |
| ec:2.3.1.117 | 5 | 2,3,4,5-tetrahydropyridine-2,6-dicarboxylate N-succinyltransferase; tetrahydropicolinate succinylase; tetrahydrodipicolinate N-succinyltransferase; tetrahydrodipicolinate succinyltransferase; succinyl-CoA:tetrahydrodipicolinate N-succinyltransferase; succinyl-CoA:2,3,4,5-tetrahydropyridine-2,6-dicarboxylate N-succinyltransferase | succinyl-CoA + (S)-2,3,4,5-tetrahydropyridine-2,6-dicarboxylate + H2O = CoA + N-succinyl-L-2-amino-6-oxoheptanedioate [RN:R04365] |
| ec:2.3.1.174 | 5 | 3-oxoadipyl-CoA thiolase | succinyl-CoA + acetyl-CoA = CoA + 3-oxoadipyl-CoA [RN:R00829] |
| ec:3.1.1.24 | 5 | 3-oxoadipate enol-lactonase; carboxymethylbutenolide lactonase; beta-ketoadipic enol-lactone hydrolase; 3-ketoadipate enol-lactonase; 3-oxoadipic enol-lactone hydrolase; beta-ketoadipate enol-lactone hydrolase | 3-oxoadipate enol-lactone + H2O = 3-oxoadipate [RN:R02991] |
| ec:5.5.1.2 | 5 | 3-carboxy-cis,cis-muconate cycloisomerase; beta-carboxymuconate lactonizing enzyme; 3-carboxymuconolactone hydrolase | 2-carboxy-2,5-dihydro-5-oxofuran-2-acetate = cis,cis-butadiene-1,2,4-tricarboxylate [RN:R03307] |
| ec:4.1.1.44 | 5 | 4-carboxymuconolactone decarboxylase; gamma-4-carboxymuconolactone decarboxylase; 4-carboxymuconolactone carboxy-lyase; 2-carboxy-2,5-dihydro-5-oxofuran-2-acetate carboxy-lyase (4,5-dihydro-5-oxofuran-2-acetate-forming) | (R)-2-carboxy-2,5-dihydro-5-oxofuran-2-acetate = 4,5-dihydro-5-oxofuran-2-acetate + CO2 [RN:R03470] |
| ec:1.2.1.28 | 5 | benzaldehyde dehydrogenase (NAD+); benzaldehyde (NAD+) dehydrogenase; benzaldehyde dehydrogenase (NAD+) | benzaldehyde + NAD+ + H2O = benzoate + NADH + 2 H+ [RN:R01419] |
| ec:2.1.1.14 | 4 | 5-methyltetrahydropteroyltriglutamate---homocysteine S-methyltransferase; tetrahydropteroyltriglutamate methyltransferase; homocysteine methylase; methyltransferase, tetrahydropteroylglutamate-homocysteine transmethylase; methyltetrahydropteroylpolyglutamate:homocysteine methyltransferase; cobalamin-independent methionine synthase; methionine synthase (cobalamin-independent); MetE | 5-methyltetrahydropteroyltri-L-glutamate + L-homocysteine = tetrahydropteroyltri-L-glutamate + L-methionine [RN:R04405] |
| ec:2.1.1.189 | 4 | 23S rRNA (uracil747-C5)-methyltransferase; YbjF; RumB; RNA uridine methyltransferase B | S-adenosyl-L-methionine + uracil747 in 23S rRNA = S-adenosyl-L-homocysteine + 5-methyluracil747 in 23S rRNA |
| ec:3.5.1.10 | 4 | formyltetrahydrofolate deformylase | 10-formyltetrahydrofolate + H2O = formate + tetrahydrofolate [RN:R00944] |
| ec:1.1.1.157 | 4 | 3-hydroxybutyryl-CoA dehydrogenase; beta-hydroxybutyryl coenzyme A dehydrogenase; L(+)-3-hydroxybutyryl-CoA dehydrogenase; BHBD; dehydrogenase, L-3-hydroxybutyryl coenzyme A (nicotinamide adenine dinucleotide phosphate); L-(+)-3-hydroxybutyryl-CoA dehydrogenase; beta-hydroxybutyryl-CoA dehydrogenase | (S)-3-hydroxybutanoyl-CoA + NADP+ = 3-acetoacetyl-CoA + NADPH + H+ [RN:R01976] |
| ec:1.4.99.1 | 4 | D-amino-acid dehydrogenase; D-amino-acid:(acceptor) oxidoreductase (deaminating) | a D-amino acid + H2O + acceptor = a 2-oxo carboxylate + NH3 + reduced acceptor [RN:R07166] |
| ec:2.3.1.9 | 4 | acetyl-CoA C-acetyltransferase; acetoacetyl-CoA thiolase; beta-acetoacetyl coenzyme A thiolase; 2-methylacetoacetyl-CoA thiolase [misleading]; 3-oxothiolase; acetyl coenzyme A thiolase; acetyl-CoA acetyltransferase; acetyl-CoA:N-acetyltransferase; thiolase II | 2 acetyl-CoA = CoA + acetoacetyl-CoA [RN:R00238] |
| ec:3.6.4.12 | 4 | DNA helicase; 3' to 5' DNA helicase; 3'-5' DNA helicase; 3'-5' PfDH; 5' to 3' DNA helicase; AvDH1; BACH1 helicase; BcMCM; BLM protein; BRCA1-associated C-terminal helicase; CeWRN-1; Dbp9p; DmRECQ5; DNA helicase 120; DNA helicase A; DNA helicase E; DNA helicase II; DNA helicase III; DNA helicase RECQL5beta; DNA helicase VI; dnaB; DnaB helicase E1; helicase HDH IV; Hel E; helicase DnaB; helicase domain of bacteriophage T7 gene 4 protein helicase; PcrA helicase; UvrD; hHcsA; Hmi1p; hPif1; MCM helicase; MCM protein; MER3 helicase; MER3 protein; MPH1; PcrA; PcrA helicase; PDH120; PfDH A; Pfh1p; PIF1 | ATP + H2O = ADP + phosphate [RN:R00086] |
| ec:2.3.1.57 | 4 | diamine N-acetyltransferase; spermidine acetyltransferase; putrescine acetyltransferase; putrescine (diamine)-acetylating enzyme; diamine acetyltransferase; spermidine/spermine N1-acetyltransferase; spermidine N1-acetyltransferase; acetyl-coenzyme A-1,4-diaminobutane N-acetyltransferase; putrescine acetylase; putrescine N-acetyltransferase | acetyl-CoA + an alkane-alpha,omega-diamine = CoA + an N-acetyldiamine [RN:R03910] |
| ec:2.4.99.17 | 3 | S-adenosylmethionine:tRNA ribosyltransferase-isomerase; QueA enzyme; queuosine biosynthesis protein QueA | S-adenosyl-L-methionine + 7-aminomethyl-7-carbaguanosine34 in tRNA = L-methionine + adenine + epoxyqueuosine34 in tRNA |
| ec:5.2.1.8 | 2 | peptidylprolyl isomerase; PPIase; cyclophilin [misleading, see comments]; peptide bond isomerase; peptidyl-prolyl cis-trans isomerase | peptidylproline (omega=180) = peptidylproline (omega=0) [RN:R04273] |
| ec:4.2.1.20 | 2 | tryptophan synthase; L-tryptophan synthetase; indoleglycerol phosphate aldolase; tryptophan desmolase; tryptophan synthetase; L-serine hydro-lyase (adding indoleglycerol-phosphate); L-serine hydro-lyase [adding 1-C-(indol-3-yl)glycerol 3-phosphate, L-tryptophan and glyceraldehyde-3-phosphate-forming] | L-serine + 1-C-(indol-3-yl)glycerol 3-phosphate = L-tryptophan + D-glyceraldehyde 3-phosphate + H2O (overall reaction) [RN:R02722]; (1a) 1-C-(indol-3-yl)glycerol 3-phosphate = indole + D-glyceraldehyde 3-phosphate [RN:R02340]; (1b) L-serine + indole = L-tryptophan + H2O [RN:R00674] |
| ec:2.5.1.9 | 2 | riboflavin synthase; heavy riboflavin synthase; light riboflavin synthase; riboflavin synthetase; riboflavine synthase; riboflavine synthetase | 2 6,7-dimethyl-8-(1-D-ribityl)lumazine = riboflavin + 4-(1-D-ribitylamino)-5-amino-2,6-dihydroxypyrimidine [RN:R00066] |
| ec:2.5.1.54 | 2 | 3-deoxy-7-phosphoheptulonate synthase; 2-dehydro-3-deoxy-phosphoheptonate aldolase; 2-keto-3-deoxy-D-arabino-heptonic acid 7-phosphate synthetase; 3-deoxy-D-arabino-2-heptulosonic acid 7-phosphate synthetase; 3-deoxy-D-arabino-heptolosonate-7-phosphate synthetase; 3-deoxy-D-arabino-heptulosonate 7-phosphate synthetase; 7-phospho-2-keto-3-deoxy-D-arabino-heptonate D-erythrose-4-phosphate lyase (pyruvate-phosphorylating); 7-phospho-2-dehydro-3-deoxy-D-arabino-heptonate D-erythrose-4-phosphate lyase (pyruvate-phosphorylating); D-erythrose-4-phosphate-lyase; D-erythrose-4-phosphate-lyase (pyruvate-phosphorylating); DAH7-P synthase; DAHP synthase; DS-Co; DS-Mn; KDPH synthase; KDPH synthetase; deoxy-D-arabino-heptulosonate-7-phosphate synthetase; phospho-2-dehydro-3-deoxyheptonate aldolase; phospho-2-keto-3-deoxyheptanoate aldolase; phospho-2-keto-3-deoxyheptonate aldolase; phospho-2-keto-3-deoxyheptonic aldolase; phospho-2-oxo-3-deoxyheptonate aldolase | phosphoenolpyruvate + D-erythrose 4-phosphate + H2O = 3-deoxy-D-arabino-hept-2-ulosonate 7-phosphate + phosphate [RN:R01826] |
| ec:2.4.2.7 | 2 | adenine phosphoribosyltransferase; AMP pyrophosphorylase; transphosphoribosidase; APRT; AMP-pyrophosphate phosphoribosyltransferase; adenine phosphoribosylpyrophosphate transferase; adenosine phosphoribosyltransferase; adenylate pyrophosphorylase; adenylic pyrophosphorylase | AMP + diphosphate = adenine + 5-phospho-alpha-D-ribose 1-diphosphate [RN:R00190] |
| ec:5.4.99.5 | 2 | chorismate mutase; hydroxyphenylpyruvate synthase | chorismate = prephenate [RN:R01715] |
| ec:1.9.3.1 | 2 | cytochrome-c oxidase; cytochrome oxidase; cytochrome a3; cytochrome aa3; Warburg's respiratory enzyme; indophenol oxidase; indophenolase; complex IV (mitochondrial electron transport); ferrocytochrome c oxidase; NADH cytochrome c oxidase | 4 ferrocytochrome c + O2 + 4 H+ = 4 ferricytochrome c + 2 H2O [RN:R00081] |
| ec:1.1.1.346 | 2 | 2,5-didehydrogluconate reductase (2-dehydro-L-gulonate-forming); 2,5-diketo-D-gluconate-reductase (ambiguous); YqhE reductase; dkgA (gene name); dkgB (gene name) | 2-dehydro-L-gulonate + NADP+ = 2,5-didehydro-D-gluconate + NADPH + H+ [RN:R08878] |
| ec:2.4.2.22 | 2 | xanthine phosphoribosyltransferase; Xan phosphoribosyltransferase; xanthosine 5'-phosphate pyrophosphorylase; xanthylate pyrophosphorylase; xanthylic pyrophosphorylase; XMP pyrophosphorylase; 5-phospho-alpha-D-ribose-1-diphosphate:xanthine phospho-D-ribosyltransferase; 9-(5-phospho-beta-D-ribosyl)xanthine:diphosphate 5-phospho-alpha-D-ribosyltransferase | XMP + diphosphate = 5-phospho-alpha-D-ribose 1-diphosphate + xanthine [RN:R02142] |
| ec:2.7.7.6 | 2 | DNA-directed RNA polymerase; RNA polymerase; RNA nucleotidyltransferase (DNA-directed); RNA polymerase I; RNA polymerase II; RNA polymerase III; C RNA formation factors; deoxyribonucleic acid-dependent ribonucleic acid polymerase; DNA-dependent ribonucleate nucleotidyltransferase; DNA-dependent RNA nucleotidyltransferase; DNA-dependent RNA polymerase; ribonucleate nucleotidyltransferase; ribonucleate polymerase; C ribonucleic acid formation factors; ribonucleic acid nucleotidyltransferase; ribonucleic acid polymerase; ribonucleic acid transcriptase; ribonucleic polymerase; ribonucleic transcriptase; RNA nucleotidyltransferase; RNA transcriptase; transcriptase; RNA nucleotidyltransferase I | nucleoside triphosphate + RNAn = diphosphate + RNAn+1 [RN:R00444] |
| ec:2.8.1.1 | 2 | thiosulfate sulfurtransferase; thiosulfate cyanide transsulfurase; thiosulfate thiotransferase; rhodanese; rhodanase | thiosulfate + cyanide = sulfite + thiocyanate [RN:R01931] |
| ec:3.2.2.27 | 2 | uracil-DNA glycosylase; UdgB (ambiguous); uracil-DNA N-glycosylase; UDG (ambiguous); uracil DNA glycohydrolase | Hydrolyses single-stranded DNA or mismatched double-stranded DNA and polynucleotides, releasing free uracil |
| ec:1.3.1.12 | 2 | prephenate dehydrogenase; hydroxyphenylpyruvate synthase; chorismate mutase---prephenate dehydrogenase | prephenate + NAD+ = 4-hydroxyphenylpyruvate + CO2 + NADH [RN:R01728] |
| ec:2.6.1.11 | 1 | acetylornithine transaminase; acetylornithine delta-transaminase; ACOAT; acetylornithine 5-aminotransferase; acetylornithine aminotransferase; N-acetylornithine aminotransferase; N-acetylornithine-delta-transaminase; N2-acetylornithine 5-transaminase; N2-acetyl-L-ornithine:2-oxoglutarate aminotransferase; succinylornithine aminotransferase; 2-N-acetyl-L-ornithine:2-oxoglutarate 5-aminotransferase | N2-acetyl-L-ornithine + 2-oxoglutarate = N-acetyl-L-glutamate 5-semialdehyde + L-glutamate [RN:R02283] |
| ec:1.14.14.7 | 1 | tryptophan 7-halogenase; PrnA; RebH | tryptophan + FADH2 + Cl- + O2 + H+ = 7-chloro-L-tryptophan + FAD + 2 H2O [RN:R09570] |
| ec:1.1.1.1 | 1 | alcohol dehydrogenase; aldehyde reductase; ADH; alcohol dehydrogenase (NAD); aliphatic alcohol dehydrogenase; ethanol dehydrogenase; NAD-dependent alcohol dehydrogenase; NAD-specific aromatic alcohol dehydrogenase; NADH-alcohol dehydrogenase; NADH-aldehyde dehydrogenase; primary alcohol dehydrogenase; yeast alcohol dehydrogenase | (1) a primary alcohol + NAD+ = an aldehyde + NADH + H+ [RN:R07326]; (2) a secondary alcohol + NAD+ = a ketone + NADH + H+ [RN:R07327] |
| ec:5.4.2.12 | 1 | phosphoglycerate mutase (2,3-diphosphoglycerate-independent); cofactor independent phosphoglycerate mutase; 2,3-diphosphoglycerate-independent phosphoglycerate mutase; phosphoglycerate phosphomutase (ambiguous); phosphoglyceromutase (ambiguous); monophosphoglycerate mutase (ambiguous); monophosphoglyceromutase (ambiguous); GriP mutase (ambiguous); PGA mutase (ambiguous); iPGM; iPGAM; PGAM-i | 2-phospho-D-glycerate = 3-phospho-D-glycerate [RN:R01518] |
| ec:2.3.1.180 | 1 | beta-ketoacyl-[acyl-carrier-protein] synthase III; 3-oxoacyl:ACP synthase III; 3-ketoacyl-acyl carrier protein synthase III; KASIII; KAS III; FabH; beta-ketoacyl-acyl carrier protein synthase III; beta-ketoacyl-ACP synthase III; beta-ketoacyl (acyl carrier protein) synthase III; acetyl-CoA:malonyl-[acyl-carrier-protein] C-acyltransferase | acetyl-CoA + a malonyl-[acyl-carrier protein] = an acetoacetyl-[acyl-carrier protein] + CoA + CO2 |
| ec:2.1.1.80 | 1 | protein-glutamate O-methyltransferase; methyl-accepting chemotaxis protein O-methyltransferase; S-adenosylmethionine-glutamyl methyltransferase; methyl-accepting chemotaxis protein methyltransferase II; S-adenosylmethionine:protein-carboxyl O-methyltransferase; protein methylase II; MCP methyltransferase I; MCP methyltransferase II; protein O-methyltransferase; protein(aspartate)methyltransferase; protein(carboxyl)methyltransferase; protein carboxyl-methylase; protein carboxyl-O-methyltransferase; protein carboxylmethyltransferase II; protein carboxymethylase; protein carboxymethyltransferase; protein methyltransferase II | S-adenosyl-L-methionine + protein L-glutamate = S-adenosyl-L-homocysteine + protein L-glutamate methyl ester [RN:R02623] |
| ec:1.8.99.3 | 1 | hydrogensulfite reductase; bisulfite reductase; dissimilatory sulfite reductase; desulfoviridin; desulforubidin; desulfofuscidin; dissimilatory-type sulfite reductase; trithionate:(acceptor) oxidoreductase | trithionate + acceptor + 2 H2O + OH- = 3 bisulfite + reduced acceptor [RN:R00295] |
| ec:1.8.99.1 | 1 | sulfite reductase; assimilatory sulfite reductase; assimilatory-type sulfite reductase; hydrogen-sulfide:(acceptor) oxidoreductase | hydrogen sulfide + acceptor + 3 H2O = sulfite + reduced acceptor [RN:R00861] |
| ec:4.1.2.14 | 1 | 2-dehydro-3-deoxy-phosphogluconate aldolase; phospho-2-keto-3-deoxygluconate aldolase; KDPG aldolase; phospho-2-keto-3-deoxygluconic aldolase; 2-keto-3-deoxy-6-phosphogluconic aldolase; 2-keto-3-deoxy-6-phosphogluconate aldolase; 6-phospho-2-keto-3-deoxygluconate aldolase; ODPG aldolase; 2-oxo-3-deoxy-6-phosphogluconate aldolase; 2-keto-3-deoxygluconate-6-P-aldolase; 2-keto-3-deoxygluconate-6-phosphate aldolase; 2-dehydro-3-deoxy-D-gluconate-6-phosphate D-glyceraldehyde-3-phosphate-lyase | 2-dehydro-3-deoxy-D-gluconate 6-phosphate = pyruvate + D-glyceraldehyde 3-phosphate [RN:R05605] |
| ec:2.5.1.7 | 1 | UDP-N-acetylglucosamine 1-carboxyvinyltransferase; MurA transferase; UDP-N-acetylglucosamine 1-carboxyvinyl-transferase; UDP-N-acetylglucosamine enoylpyruvyltransferase; enoylpyruvate transferase; phosphoenolpyruvate-UDP-acetylglucosamine-3-enolpyruvyltransferase; phosphoenolpyruvate:UDP-2-acetamido-2-deoxy-D-glucose 2-enoyl-1-carboxyethyltransferase; phosphoenolpyruvate:uridine diphosphate N-acetylglucosamine enolpyruvyltransferase; phosphoenolpyruvate:uridine-5'-diphospho-N-acetyl-2-amino-2-deoxyglucose 3-enolpyruvyltransferase; phosphopyruvate-uridine diphosphoacetylglucosamine pyruvatetransferase; pyruvate-UDP-acetylglucosamine transferase; pyruvate-uridine diphospho-N-acetylglucosamine transferase; pyruvate-uridine diphospho-N-acetyl-glucosamine transferase; pyruvic-uridine diphospho-N-acetylglucosaminyltransferase; phosphoenolpyruvate:UDP-N-acetyl-D-glucosamine 1-carboxyvinyltransferase | phosphoenolpyruvate + UDP-N-acetyl-alpha-D-glucosamine = phosphate + UDP-N-acetyl-3-O-(1-carboxyvinyl)-alpha-D-glucosamine [RN:R00660] |
| ec:1.1.1.49 | 1 | glucose-6-phosphate dehydrogenase (NADP+); NADP-glucose-6-phosphate dehydrogenase; Zwischenferment; D-glucose 6-phosphate dehydrogenase; glucose 6-phosphate dehydrogenase (NADP); NADP-dependent glucose 6-phosphate dehydrogenase; 6-phosphoglucose dehydrogenase; Entner-Doudoroff enzyme; glucose-6-phosphate 1-dehydrogenase; G6PDH; GPD; glucose-6-phosphate dehydrogenase | D-glucose 6-phosphate + NADP+ = 6-phospho-D-glucono-1,5-lactone + NADPH + H+ [RN:R00835] |
| ec:4.1.3.16 | 1 | 4-hydroxy-2-oxoglutarate aldolase; 2-oxo-4-hydroxyglutarate aldolase; hydroxyketoglutaric aldolase; 4-hydroxy-2-ketoglutaric aldolase; 2-keto-4-hydroxyglutaric aldolase; 4-hydroxy-2-ketoglutarate aldolase; 2-keto-4-hydroxyglutarate aldolase; 2-oxo-4-hydroxyglutaric aldolase; DL-4-hydroxy-2-ketoglutarate aldolase; hydroxyketoglutarate aldolase; 2-keto-4-hydroxybutyrate aldolase; 4-hydroxy-2-oxoglutarate glyoxylate-lyase; KHGA | 4-hydroxy-2-oxoglutarate = pyruvate + glyoxylate [RN:R00470] |
| ec:1.8.98.1 | 1 | CoB---CoM heterodisulfide reductase; heterodisulfide reductase; soluble heterodisulfide reductase | coenzyme B + coenzyme M + methanophenazine = N-{7-[(2-sulfoethyl)dithio]heptanoyl}-O3-phospho-L-threonine + dihydromethanophenazine [RN:R04540] |
| ec:5.1.3.3 | 1 | aldose 1-epimerase; mutarotase; aldose mutarotase; galactose mutarotase; galactose 1-epimerase; D-galactose 1-epimerase | alpha-D-glucose = beta-D-glucose [RN:R01602] |
| ec:3.4.21.88 | 1 | repressor LexA; LexA repressor | Hydrolysis of Ala84!Gly bond in repressor LexA |
| ec:4.2.1.12 | 1 | phosphogluconate dehydratase; 6-phosphogluconate dehydratase; 6-phosphogluconic dehydrase; gluconate-6-phosphate dehydratase; gluconate 6-phosphate dehydratase; 6-phosphogluconate dehydrase; 6-phospho-D-gluconate hydro-lyase | 6-phospho-D-gluconate = 2-dehydro-3-deoxy-6-phospho-D-gluconate + H2O [RN:R02036] |
| ec:4.2.1.11 | 1 | phosphopyruvate hydratase; enolase; 2-phosphoglycerate dehydratase; 14-3-2-protein; nervous-system specific enolase; phosphoenolpyruvate hydratase; 2-phosphoglycerate dehydratase; 2-phosphoglyceric dehydratase; 2-phosphoglycerate enolase; gamma-enolase; 2-phospho-D-glycerate hydro-lyase | 2-phospho-D-glycerate = phosphoenolpyruvate + H2O [RN:R00658] |
| ec:2.4.2.9 | 1 | uracil phosphoribosyltransferase; UMP pyrophosphorylase; UPRTase; UMP:pyrophosphate phosphoribosyltransferase; uridine 5'-phosphate pyrophosphorylase; uridine monophosphate pyrophosphorylase; uridylate pyrophosphorylase; uridylic pyrophosphorylase | UMP + diphosphate = uracil + 5-phospho-alpha-D-ribose 1-diphosphate [RN:R00966] |
| ec:3.5.1.11 | 1 | penicillin amidase; penicillin acylase; benzylpenicillin acylase; novozym 217; semacylase; alpha-acylamino-beta-lactam acylhydrolase; ampicillin acylase | penicillin + H2O = a carboxylate + 6-aminopenicillanate [RN:R02170] |
| ec:3.6.3.28 | 1 | phosphonate-transporting ATPase | ATP + H2O + phosphonateout = ADP + phosphate + phosphonatein [RN:R00086] |
| ec:4.2.3.12 | 1 | 6-pyruvoyltetrahydropterin synthase; 2-amino-4-oxo-6-[(1S,2R)-1,2-dihydroxy-3-triphosphooxypropyl]-7,8-dihydroxypteridine triphosphate lyase; 6-[(1S,2R)-1,2-dihydroxy-3-triphosphooxypropyl]-7,8-dihydropterin triphosphate-lyase (6-pyruvoyl-5,6,7,8-tetrahydropterin-forming) | 7,8-dihydroneopterin 3'-triphosphate = 6-pyruvoyl-5,6,7,8-tetrahydropterin + triphosphate [RN:R04286] |
| ec:1.1.1.31 | 1 | 3-hydroxyisobutyrate dehydrogenase; beta-hydroxyisobutyrate dehydrogenase | 3-hydroxy-2-methylpropanoate + NAD+ = 2-methyl-3-oxopropanoate + NADH + H+ [RN:R02047] |
| ec:4.2.1.2 | 1 | fumarate hydratase; fumarase; L-malate hydro-lyase; (S)-malate hydro-lyase | (S)-malate = fumarate + H2O [RN:R01082] |
| ec:3.1.1.31 | 1 | 6-phosphogluconolactonase; phosphogluconolactonase; 6-PGL | 6-phospho-D-glucono-1,5-lactone + H2O = 6-phospho-D-gluconate [RN:R02035] |
| ec:2.4.1.1 | 1 | glycogen phosphorylase; muscle phosphorylase a and b; amylophosphorylase; polyphosphorylase; amylopectin phosphorylase; glucan phosphorylase; alpha-glucan phosphorylase; 1,4-alpha-glucan phosphorylase; glucosan phosphorylase; granulose phosphorylase; maltodextrin phosphorylase; muscle phosphorylase; myophosphorylase; potato phosphorylase; starch phosphorylase; 1,4-alpha-D-glucan:phosphate alpha-D-glucosyltransferase; phosphorylase (ambiguous) | [(1->4)-alpha-D-glucosyl]n + phosphate = [(1->4)-alpha-D-glucosyl]n-1 + alpha-D-glucose 1-phosphate [RN:R01821 R06050] |
| ec:3.5.4.13 | 1 | dCTP deaminase; deoxycytidine triphosphate deaminase; 5-methyl-dCTP deaminase | dCTP + H2O = dUTP + NH3 [RN:R02325] |
| ec:1.4.3.4 | 1 | monoamine oxidase; adrenalin oxidase; adrenaline oxidase; amine oxidase (ambiguous); amine oxidase (flavin-containing); amine:oxygen oxidoreductase (deaminating) (flavin-containing); epinephrine oxidase; MAO; MAO A; MAO B; MAO-A; MAO-B; monoamine oxidase A; monoamine oxidase B; monoamine:O2 oxidoreductase (deaminating); polyamine oxidase (ambiguous); serotonin deaminase; spermidine oxidase (ambiguous); spermine oxidase (ambiguous); tyraminase; tyramine oxidase | RCH2NHR' + H2O + O2 = RCHO + R'NH2 + H2O2 [RN:R01853] |
| ec:6.5.1.1 | 1 | DNA ligase (ATP); polydeoxyribonucleotide synthase (ATP); polynucleotide ligase; sealase; DNA repair enzyme; DNA joinase; DNA ligase; deoxyribonucleic ligase; deoxyribonucleate ligase; DNA-joining enzyme; deoxyribonucleic-joining enzyme; deoxyribonucleic acid-joining enzyme; deoxyribonucleic repair enzyme; deoxyribonucleic joinase; deoxyribonucleic acid ligase; deoxyribonucleic acid joinase; deoxyribonucleic acid repair enzyme | ATP + (deoxyribonucleotide)n + (deoxyribonucleotide)m = AMP + diphosphate + (deoxyribonucleotide)n+m [RN:R00381] |
| ec:6.3.5.11 | 1 | cobyrinate a,c-diamide synthase; cobyrinic acid a,c-diamide synthetase; CbiA | 2 ATP + cobyrinate + 2 L-glutamine + 2 H2O = 2 ADP + 2 phosphate + cobyrinate a,c-diamide + 2 L-glutamate (overall reaction) [RN:R05815]; (1a) ATP + cobyrinate + L-glutamine + H2O = ADP + phosphate + cobyrinate c-monamide + L-glutamate [RN:R09598]; (1b) ATP + cobyrinate c-monamide + L-glutamine + H2O = ADP + phosphate + cobyrinate a,c-diamide + L-glutamate [RN:R09599] |
| ec:6.3.5.10 | 1 | adenosylcobyric acid synthase (glutamine-hydrolysing); CobQ; cobyric acid synthase; 5'-deoxy-5'-adenosylcobyrinic-acid-a,c-diamide:L-glutamine amido-ligase; Ado-cobyric acid synthase [glutamine hydrolyzing] | 4 ATP + adenosylcobyrinic acid a,c-diamide + 4 L-glutamine + 4 H2O = 4 ADP + 4 phosphate + adenosylcobyric acid + 4 L-glutamate [RN:R05225] |
| ec:2.1.1.177 | 1 | 23S rRNA (pseudouridine1915-N3)-methyltransferase; YbeA; RlmH; pseudouridine methyltransferase; m3Psi methyltransferase; Psi1915-specific methyltransferase; rRNA large subunit methyltransferase H | S-adenosyl-L-methionine + pseudouridine1915 in 23S rRNA = S-adenosyl-L-homocysteine + N3-methylpseudouridine1915 in 23S rRNA |
| ec:3.7.1.20 | 1 | 3-fumarylpyruvate hydrolase; nagK (gene name); naaD (gene name) | 3-fumarylpyruvate + H2O = fumarate + pyruvate [RN:R01085] |
| ec:2.1.1.171 | 1 | 16S rRNA (guanine966-N2)-methyltransferase; yhhF (gene name); rsmD (gene name); m2G966 methyltransferase | S-adenosyl-L-methionine + guanine966 in 16S rRNA = S-adenosyl-L-homocysteine + N2-methylguanine966 in 16S rRNA [RN:R07234] |
| ec:3.4.11.9 | 1 | Xaa-Pro aminopeptidase; proline aminopeptidase; aminopeptidase P; aminoacylproline aminopeptidase; X-Pro aminopeptidase | Release of any N-terminal amino acid, including proline, that is linked to proline, even from a dipeptide or tripeptide |
| ec:4.1.2.50 | 1 | 6-carboxytetrahydropterin synthase; CPH4 synthase; queD (gene name); ToyB; ykvK (gene name) | 7,8-dihydroneopterin 3'-triphosphate + H2O = 6-carboxy-5,6,7,8-tetrahydropterin + acetaldehyde + triphosphate [RN:R09959] |
| ec:3.1.1.85 | 1 | pimelyl-[acyl-carrier protein] methyl ester esterase; BioH | pimelyl-[acyl-carrier protein] methyl ester + H2O = pimelyl-[acyl-carrier protein] + methanol [RN:R09725] |
| ec:2.3.1.15 | 1 | glycerol-3-phosphate 1-O-acyltransferase; alpha-glycerophosphate acyltransferase; 3-glycerophosphate acyltransferase; ACP:sn-glycerol-3-phosphate acyltransferase; glycerol 3-phosphate acyltransferase; glycerol phosphate acyltransferase; glycerol phosphate transacylase; glycerophosphate acyltransferase; glycerophosphate transacylase; sn-glycerol 3-phosphate acyltransferase; sn-glycerol-3-phosphate acyltransferase; glycerol-3-phosphate O-acyltransferase (ambiguous) | acyl-CoA + sn-glycerol 3-phosphate = CoA + 1-acyl-sn-glycerol 3-phosphate [RN:R00851] |
| ec:3.1.3.25 | 1 | inositol-phosphate phosphatase; myo-inositol-1(or 4)-monophosphatase; inositol 1-phosphatase; L-myo-inositol-1-phosphate phosphatase; myo-inositol 1-phosphatase; inositol phosphatase; inositol monophosphate phosphatase; inositol-1(or 4)-monophosphatase; myo-inositol-1(or 4)-phosphate phosphohydrolase; myo-inositol monophosphatase; myo-inositol-1-phosphatase | myo-inositol phosphate + H2O = myo-inositol + phosphate [RN:R07343] |
| ec:2.7.7.7 | 1 | DNA-directed DNA polymerase; DNA polymerase I; DNA polymerase II; DNA polymerase III; DNA polymerase alpha; DNA polymerase beta; DNA polymerase gamma; DNA nucleotidyltransferase (DNA-directed); DNA nucleotidyltransferase (DNA-directed); deoxyribonucleate nucleotidyltransferase; deoxynucleate polymerase; deoxyribonucleic acid duplicase; deoxyribonucleic acid polymerase; deoxyribonucleic duplicase; deoxyribonucleic polymerase; deoxyribonucleic polymerase I; DNA duplicase; DNA nucleotidyltransferase; DNA polymerase; DNA replicase; DNA-dependent DNA polymerase; duplicase; Klenow fragment; sequenase; Taq DNA polymerase; Taq Pol I; Tca DNA polymerase | deoxynucleoside triphosphate + DNAn = diphosphate + DNAn+1 [RN:R00379] |
| ec:3.1.3.3 | 1 | phosphoserine phosphatase | O-phospho-L(or D)-serine + H2O = L(or D)-serine + phosphate [RN:R00582 R02853] |
| ec:2.7.6.5 | 1 | GTP diphosphokinase; stringent factor; guanosine 3',5'-polyphosphate synthase; GTP pyrophosphokinase; ATP-GTP 3'-diphosphotransferase; guanosine 5',3'-polyphosphate synthetase; (p)ppGpp synthetase I; (p)ppGpp synthetase II; guanosine pentaphosphate synthetase; GPSI; GPSII | ATP + GTP = AMP + guanosine 3'-diphosphate 5'-triphosphate [RN:R00429] |
| ec:6.3.5.9 | 1 | hydrogenobyrinic acid a,c-diamide synthase (glutamine-hydrolysing); CobB | 2 ATP + hydrogenobyrinic acid + 2 L-glutamine + 2 H2O = 2 ADP + 2 phosphate + hydrogenobyrinic acid a,c-diamide + 2 L-glutamate [RN:R05224] |
| ec:2.7.1.48 | 1 | uridine kinase; pyrimidine ribonucleoside kinase; uridine-cytidine kinase; uridine kinase (phosphorylating); uridine phosphokinase | ATP + uridine = ADP + UMP [RN:R00964] |
| ec:3.2.1.20 | 1 | alpha-glucosidase; maltase; glucoinvertase; glucosidosucrase; maltase-glucoamylase; alpha-glucopyranosidase; glucosidoinvertase; alpha-D-glucosidase; alpha-glucoside hydrolase; alpha-1,4-glucosidase | Hydrolysis of terminal, non-reducing (1->4)-linked alpha-D-glucose residues with release of D-glucose |
| ec:4.1.3.40 | 1 | chorismate lyase; CL; CPL; UbiC | chorismate = 4-hydroxybenzoate + pyruvate [RN:R01302] |
| ec:3.6.1.8 | 1 | ATP diphosphatase; ATPase (ambiguous); ATP pyrophosphatase; adenosine triphosphate pyrophosphatase; ATP diphosphohydrolase (ambiguous) | ATP + H2O = AMP + diphosphate [RN:R00087] |
| ec:2.2.1.6 | 1 | acetolactate synthase; alpha-acetohydroxy acid synthetase; alpha-acetohydroxyacid synthase; alpha-acetolactate synthase; alpha-acetolactate synthetase; acetohydroxy acid synthetase; acetohydroxyacid synthase; acetolactate pyruvate-lyase (carboxylating); acetolactic synthetase | 2 pyruvate = 2-acetolactate + CO2 [RN:R00006] |
| ec:6.3.3.1 | 1 | phosphoribosylformylglycinamidine cyclo-ligase; phosphoribosylaminoimidazole synthetase; AIR synthetase; 5'-aminoimidazole ribonucleotide synthetase; 2-(formamido)-1-N-(5-phosphoribosyl)acetamidine cyclo-ligase (ADP-forming) | ATP + 2-(formamido)-N1-(5-phospho-D-ribosyl)acetamidine = ADP + phosphate + 5-amino-1-(5-phospho-D-ribosyl)imidazole [RN:R04208] |
| ec:3.1.3.11 | 1 | fructose-bisphosphatase; hexose diphosphatase; FBPase; fructose 1,6-diphosphatase; fructose 1,6-diphosphate phosphatase; D-fructose 1,6-diphosphatase; fructose 1,6-bisphosphatase; fructose diphosphatase; fructose diphosphate phosphatase; fructose bisphosphate phosphatase; fructose 1,6-bisphosphate 1-phosphatase; fructose 1,6-bisphosphate phosphatase; hexose bisphosphatase; D-fructose-1,6-bisphosphate phosphatase | D-fructose 1,6-bisphosphate + H2O = D-fructose 6-phosphate + phosphate [RN:R00762] |
| ec:4.1.1.31 | 1 | phosphoenolpyruvate carboxylase; phosphopyruvate (phosphate) carboxylase; PEP carboxylase; phosphoenolpyruvic carboxylase; PEPC; PEPCase; phosphate:oxaloacetate carboxy-lyase (phosphorylating) | phosphate + oxaloacetate = phosphoenolpyruvate + HCO3- [RN:R00345] |
| ec:3.5.1.44 | 1 | protein-glutamine glutaminase; peptidoglutaminase II; glutaminyl-peptide glutaminase; destabilase; peptidylglutaminase II | protein L-glutamine + H2O = protein L-glutamate + NH3 [RN:R02622] |
| ec:3.5.4.5 | 1 | cytidine deaminase; cytosine nucleoside deaminase; (deoxy)cytidine deaminase; cdd (gene name); CDA (gene name) | (1) cytidine + H2O = uridine + NH3 [RN:R01878]; (2) 2'-deoxycytidine + H2O = 2'-deoxyuridine + NH3 [RN:R02485] |
| ec:1.2.1.12 | 1 | glyceraldehyde-3-phosphate dehydrogenase (phosphorylating); triosephosphate dehydrogenase; dehydrogenase, glyceraldehyde phosphate; phosphoglyceraldehyde dehydrogenase; 3-phosphoglyceraldehyde dehydrogenase; NAD+-dependent glyceraldehyde phosphate dehydrogenase; glyceraldehyde phosphate dehydrogenase (NAD+); glyceraldehyde-3-phosphate dehydrogenase (NAD+); NADH-glyceraldehyde phosphate dehydrogenase; glyceraldehyde-3-P-dehydrogenase | D-glyceraldehyde 3-phosphate + phosphate + NAD+ = 3-phospho-D-glyceroyl phosphate + NADH + H+ [RN:R01061] |
| ec:2.5.1.17 | 1 | cob(I)yrinic acid a,c-diamide adenosyltransferase; CobA; CobO; ATP:corrinoid adenosyltransferase; cob(I)alamin adenosyltransferase; aquacob(I)alamin adenosyltransferase; aquocob(I)alamin vitamin B12s adenosyltransferase; ATP:cob(I)alamin Cobeta-adenosyltransferase | (1) ATP + cob(I)yrinic acid a,c-diamide = triphosphate + adenosylcob(III)yrinic acid a,c-diamide [RN:R05220]; (2) ATP + cobinamide = triphosphate + adenosylcobinamide [RN:R07268] |
| ec:6.1.1.22 | 1 | asparagine---tRNA ligase; asparaginyl-tRNA synthetase; asparaginyl-transfer ribonucleate synthetase; asparaginyl transfer RNA synthetase; asparaginyl transfer ribonucleic acid synthetase; asparagyl-transfer RNA synthetase; asparagine translase | ATP + L-asparagine + tRNAAsn = AMP + diphosphate + L-asparaginyl-tRNAAsn [RN:R03648] |
| ec:2.1.2.2 | 1 | phosphoribosylglycinamide formyltransferase; 2-amino-N-ribosylacetamide 5'-phosphate transformylase; GAR formyltransferase; GAR transformylase; glycinamide ribonucleotide transformylase; GAR TFase; 5,10-methenyltetrahydrofolate:2-amino-N-ribosylacetamide ribonucleotide transformylase | 10-formyltetrahydrofolate + N1-(5-phospho-D-ribosyl)glycinamide = tetrahydrofolate + N2-formyl-N1-(5-phospho-D-ribosyl)glycinamide [RN:R04325] |
| ec:1.7.1.7 | 1 | GMP reductase; guanosine 5'-monophosphate reductase; NADPH:GMP oxidoreductase (deaminating); guanosine monophosphate reductase; guanylate reductase; NADPH2:guanosine-5'-phosphate oxidoreductase (deaminating); guanosine 5'-phosphate reductase | IMP + NH3 + NADP+ = GMP + NADPH + H+ [RN:R01134] |
| ec:5.3.1.1 | 1 | triose-phosphate isomerase; phosphotriose isomerase; triose phosphoisomerase; triose phosphate mutase; D-glyceraldehyde-3-phosphate ketol-isomerase | D-glyceraldehyde 3-phosphate = glycerone phosphate [RN:R01015] |
| ec:3.1.26.4 | 1 | ribonuclease H; endoribonuclease H (calf thymus); RNase H; RNA\*DNA hybrid ribonucleotidohydrolase; hybrid ribonuclease; hybridase; hybridase (ribonuclease H); ribonuclease H; hybrid nuclease; calf thymus ribonuclease H | Endonucleolytic cleavage to 5'-phosphomonoester |
| ec:2.1.1.242 | 1 | 16S rRNA (guanine1516-N2)-methyltransferase; yhiQ (gene name); rsmJ (gene name); m2G1516 methyltransferase | S-adenosyl-L-methionine + guanine1516 in 16S rRNA = S-adenosyl-L-homocysteine + N2-methylguanine1516 in 16S rRNA |
| ec:6.2.1.3 | 1 | long-chain-fatty-acid---CoA ligase; acyl-CoA synthetase; fatty acid thiokinase (long chain); acyl-activating enzyme; palmitoyl-CoA synthase; lignoceroyl-CoA synthase; arachidonyl-CoA synthetase; acyl coenzyme A synthetase; acyl-CoA ligase; palmitoyl coenzyme A synthetase; thiokinase; palmitoyl-CoA ligase; acyl-coenzyme A ligase; fatty acid CoA ligase; long-chain fatty acyl coenzyme A synthetase; oleoyl-CoA synthetase; stearoyl-CoA synthetase; long chain fatty acyl-CoA synthetase; long-chain acyl CoA synthetase; fatty acid elongase; LCFA synthetase; pristanoyl-CoA synthetase; ACS3; long-chain acyl-CoA synthetase I; long-chain acyl-CoA synthetase II; fatty acyl-coenzyme A synthetase; long-chain acyl-coenzyme A synthetase; FAA1 | ATP + a long-chain fatty acid + CoA = AMP + diphosphate + an acyl-CoA [RN:R00390] |
| ec:6.1.1.17 | 1 | glutamate---tRNA ligase; glutamyl-tRNA synthetase; glutamyl-transfer ribonucleate synthetase; glutamyl-transfer RNA synthetase; glutamyl-transfer ribonucleic acid synthetase; glutamate-tRNA synthetase; glutamic acid translase | ATP + L-glutamate + tRNAGlu = AMP + diphosphate + L-glutamyl-tRNAGlu [RN:R05578] |

  
**Over-represented Metabolite Summary**: Collection of the metabolites identified as substrates or products of the proteins representaed the "Over-represented Enzyme Summary" ranked by frequency of occurrence  

| ID | Structure | Name | Frequency | EC |
| --- | --- | --- | --- | --- |
| cpd:C00093 |  | sn-Glycerol 3-phosphate; Glycerophosphoric acid; D-Glycerol 1-phosphate | 193 | ec:2.7.1.30 ec:2.3.1.15 ec:1.1.5.3 ec:3.1.4.46 |
| cpd:C00001 |  | H2O; Water | 141 | ec:3.1.1.85 ec:6.3.5.4 ec:1.13.11.3 ec:2.5.1.54 ec:2.1.2.2 ec:4.2.1.12 ec:3.1.1.24 ec:3.5.4.13 ec:1.8.99.1 ec:4.2.1.11 ec:4.1.1.31 ec:1.8.99.3 ec:1.4.99.1 ec:2.3.1.117 ec:1.14.13.2 ec:1.4.3.4 ec:3.1.4.46 ec:6.3.5.9 ec:3.1.3.25 ec:4.1.2.50 ec:3.5.1.10 ec:3.5.1.11 ec:4.2.1.6 ec:3.1.3.11 ec:2.3.2.2 ec:4.2.1.2 ec:6.3.5.11 ec:1.2.1.28 ec:3.1.1.31 ec:6.3.5.10 ec:3.1.3.3 ec:3.2.1.20 ec:4.2.3.12 ec:3.6.1.8 ec:4.2.1.20 ec:3.5.4.5 ec:1.9.3.1 ec:1.18.1.3 ec:3.7.1.20 ec:1.1.1.1 |
| cpd:C00002 |  | ATP; Adenosine 5'-triphosphate | 108 | ec:6.3.3.1 ec:6.3.5.11 ec:6.3.5.4 ec:6.1.1.22 ec:6.2.1.3 ec:2.7.6.5 ec:6.3.5.10 ec:2.7.1.48 ec:2.7.7.6 ec:2.7.1.30 ec:3.6.1.8 ec:2.5.1.17 ec:6.1.1.17 ec:6.3.5.9 |
| cpd:C00111 |  | Glycerone phosphate; Dihydroxyacetone phosphate | 98 | ec:1.1.5.3 ec:5.3.1.1 |
| cpd:C01352 |  | FADH2 | 97 | ec:1.1.5.3 |
| cpd:C00016 |  | FAD; Flavin adenine dinucleotide | 97 | ec:1.1.5.3 |
| cpd:C00008 |  | ADP; Adenosine 5'-diphosphate | 94 | ec:2.7.1.30 ec:6.3.3.1 ec:3.6.1.8 ec:6.3.5.11 ec:6.3.5.9 ec:6.3.5.10 ec:2.7.1.48 |
| cpd:C00116 |  | Glycerol; Glycerin; 1,2,3-Trihydroxypropane; 1,2,3-Propanetriol | 89 | ec:2.7.1.30 |
| cpd:C00080 |  | H+; Hydron | 47 | ec:1.1.1.49 ec:1.5.1.2 ec:1.2.1.28 ec:5.4.99.5 ec:1.1.1.31 ec:1.1.1.157 ec:1.2.1.12 ec:1.9.3.1 ec:1.18.1.3 ec:1.14.13.2 ec:1.7.1.7 ec:1.1.1.1 ec:1.3.1.12 |
| cpd:C00009 |  | Orthophosphate; Phosphate; Phosphoric acid; Orthophosphoric acid | 42 | ec:3.1.3.11 ec:6.3.3.1 ec:6.3.5.11 ec:2.5.1.7 ec:6.3.5.10 ec:2.1.3.3 ec:2.5.1.54 ec:3.1.3.3 ec:1.2.1.12 ec:3.6.1.8 ec:2.5.1.19 ec:2.4.1.1 ec:4.1.1.31 ec:6.3.5.9 ec:3.1.3.25 |
| cpd:C00006 |  | NADP+; NADP; Nicotinamide adenine dinucleotide phosphate; beta-Nicotinamide adenine dinucleotide phosphate; TPN; Triphosphopyridine nucleotide | 40 | ec:1.1.1.49  ec:1.5.1.2 ec:1.2.1.28 ec:1.18.1.3 ec:1.14.13.2 ec:1.7.1.7 ec:1.1.1.157 |
| cpd:C00005 |  | NADPH; TPNH; Reduced nicotinamide adenine dinucleotide phosphate | 40 | ec:1.1.1.49  ec:1.5.1.2 ec:1.2.1.28 ec:1.18.1.3 ec:1.14.13.2 ec:1.7.1.7 ec:1.1.1.157 |
| cpd:C00004 |  | NADH; DPNH; Reduced nicotinamide adenine dinucleotide | 38 | ec:1.2.1.12 ec:1.5.1.2 ec:1.2.1.28 ec:5.4.99.5 ec:1.18.1.3 ec:1.1.1.31 ec:1.1.1.1 ec:1.1.1.157 ec:1.3.1.12 |
| cpd:C00003 |  | NAD+; NAD; Nicotinamide adenine dinucleotide; DPN; Diphosphopyridine nucleotide; Nadide | 38 | ec:1.2.1.12 ec:1.5.1.2 ec:1.2.1.28 ec:5.4.99.5 ec:1.18.1.3 ec:1.1.1.31 ec:1.1.1.1 ec:1.1.1.157 ec:1.3.1.12 |
| cpd:C00025 |  | L-Glutamate; L-Glutamic acid; L-Glutaminic acid; Glutamate | 34 | ec:2.3.2.2 ec:6.3.5.11 ec:6.3.5.4 ec:2.6.1.11 ec:6.1.1.17 ec:6.3.5.9 ec:6.3.5.10 |
| cpd:C00007 |  | Oxygen; O2 | 34 | ec:1.9.3.1 ec:1.18.1.3 ec:1.14.13.2 ec:1.4.3.4 ec:1.13.11.3 |
| cpd:C00058 |  | Formate; Methanoic acid; Formic acid | 29 | ec:1.18.1.3 ec:4.1.99.12 ec:3.5.1.10 |
| cpd:C00013 |  | Diphosphate; Diphosphoric acid; Pyrophosphate; Pyrophosphoric acid; PPi | 27 | ec:2.4.2.9 ec:2.7.7.7  ec:2.7.7.6 ec:2.4.2.7 ec:3.6.1.8 ec:6.1.1.22 ec:6.3.5.4 ec:6.2.1.3 ec:6.1.1.17 ec:2.4.2.22 |
| cpd:C00011 |  | CO2; Carbon dioxide | 26 | ec:2.3.1.180 ec:4.1.1.31 ec:5.4.99.5 ec:2.2.1.6 ec:1.18.1.3 ec:4.1.1.44 ec:1.3.1.12 |
| cpd:C00024 |  | Acetyl-CoA; Acetyl coenzyme A | 24 | ec:2.3.1.180 ec:2.3.1.9 ec:2.3.1.174 ec:2.3.1.57 ec:2.8.3.12 |
| cpd:C00014 |  | Ammonia; NH3 | 24 | ec:3.5.4.13 ec:1.4.99.1 ec:3.5.4.5 ec:1.18.1.3 ec:1.7.1.7 ec:1.4.3.4 |
| cpd:C01419 |  | Cys-Gly; L-Cysteinylglycine | 23 | ec:2.3.2.2 |
| cpd:C05951 |  | Leukotriene D4; LTD4 | 23 | ec:2.3.2.2 |
| cpd:C00245 |  | Taurine; 2-Aminoethanesulfonic acid; Aminoethylsulfonic acid | 23 | ec:2.3.2.2 |
| cpd:C03363 |  | 5-L-Glutamyl amino acid; L-gamma-Glutamyl amino acid | 23 | ec:2.3.2.2 |
| cpd:C02320 |  | R-S-Glutathione | 23 | ec:2.3.2.2 |
| cpd:C00151 |  | L-Amino acid; L-2-Amino acid | 23 | ec:2.3.2.2 |
| cpd:C03740 |  | (5-L-Glutamyl)-L-amino acid; L-gamma-Glutamyl-L-amino acid | 23 | ec:2.3.2.2 |
| cpd:C05844 |  | 5-L-Glutamyl-taurine; 5-Glutamyl-taurine; Glutaurine | 23 | ec:2.3.2.2 |
| cpd:C06114 |  | gamma-Glutamyl-beta-aminopropiononitrile; gamma-Glutamyl-3-aminopropiononitrile | 23 | ec:2.3.2.2 |
| cpd:C00051 |  | Glutathione; 5-L-Glutamyl-L-cysteinylglycine; N-(N-gamma-L-Glutamyl-L-cysteinyl)glycine; gamma-L-Glutamyl-L-cysteinyl-glycine; GSH; Reduced glutathione | 23 | ec:2.3.2.2 |
| cpd:C02166 |  | Leukotriene C4; LTC4 | 23 | ec:2.3.2.2 |
| cpd:C00045 |  | Amino acid; Amino acids | 23 | ec:2.3.2.2 |
| cpd:C03193 |  | (5-L-Glutamyl)-peptide | 23 | ec:2.3.2.2 |
| cpd:C05695 |  | gamma-Glutamyl-Se-methylselenocysteine; 5-L-Glutamyl-Se-methylselenocysteine | 23 | ec:2.3.2.2 |
| cpd:C05729 |  | R-S-Cysteinylglycine | 23 | ec:2.3.2.2 |
| cpd:C05689 |  | Se-Methyl-L-selenocysteine | 23 | ec:2.3.2.2 |
| cpd:C00012 |  | Peptide | 23 | ec:2.3.2.2 |
| cpd:C05711 |  | gamma-Glutamyl-beta-cyanoalanine | 23 | ec:2.3.2.2 |
| cpd:C05670 |  | 3-Aminopropiononitrile; beta-Aminopropionitrile | 23 | ec:2.3.2.2 |
| cpd:C02512 |  | 3-Cyano-L-alanine; L-3-Cyanoalanine; L-beta-Cyanoalanine | 23 | ec:2.3.2.2 |
| cpd:C01216 |  | 2-Dehydro-3-deoxy-D-galactonate | 22 | ec:4.2.1.6 |
| cpd:C00880 |  | D-Galactonate; D-Galactonic acid | 22 | ec:4.2.1.6 |
| cpd:C00074 |  | Phosphoenolpyruvate; Phosphoenolpyruvic acid; PEP | 22 | ec:4.2.1.11 ec:4.1.1.31 ec:2.5.1.19 ec:2.5.1.7 ec:2.5.1.54 |
| cpd:C00010 |  | CoA; Coenzyme A; CoA-SH | 22 | ec:2.3.1.180 ec:2.3.1.15 ec:2.3.1.9 ec:2.3.1.117 ec:6.2.1.3 ec:2.3.1.174 ec:2.3.1.57 |
| cpd:C00156 |  | 4-Hydroxybenzoate; Hydroxybenzoic acid; 4-Hydroxybenzoic acid; Hydroxybenzenecarboxylic acid | 19 | ec:1.2.1.28 ec:1.14.13.2 ec:4.1.3.40 |
| cpd:C01269 |  | 5-O-(1-Carboxyvinyl)-3-phosphoshikimate; O5-(1-Carboxyvinyl)-3-phosphoshikimate | 17 | ec:2.5.1.19 |
| cpd:C00048 |  | Glyoxylate; Glyoxalate; Glyoxylic acid | 17 | ec:4.1.3.16 ec:1.18.1.3 ec:4.1.2.14 |
| cpd:C03175 |  | Shikimate 3-phosphate; Shikimate 5-phosphate | 17 | ec:2.5.1.19 |
| cpd:C06727 |  | cis-1,2-Dihydro-3-ethylcatechol; cis-2,3-Dihydroxy-2,3-dihydroethylbenzene; cis-3-Ethyl-cyclohexa-3,5-diene-1,2-diol | 16 | ec:1.18.1.3 |
| cpd:C01407 |  | Benzene | 16 | ec:1.18.1.3 |
| cpd:C05629 |  | Phenylpropanoate; 3-Phenyl-propionic acid; 3-Phenylpropanoic acid; 3-Phenylpropionic acid | 16 | ec:1.18.1.3 |
| cpd:C04091 |  | cis-1,2-Dihydrobenzene-1,2-diol; cis-Benzeneglycol; cis-Cyclohexa-3,5-diene-1,2-diol | 16 | ec:1.18.1.3 |
| cpd:C01327 |  | Hydrochloric acid; HCl; Hydrogen chloride; Hydrochloride | 16 | ec:1.18.1.3 |
| cpd:C06589 |  | cis-2,3-Dihydro-2,3-dihydroxybiphenyl; cis-3-Phenylcyclohexa-3,5-diene-1,2-diol; (1S,2R)-3-Phenylcyclohexa-3,5-diene-1,2-diol | 16 | ec:1.18.1.3 |
| cpd:C06588 |  | Biphenyl; Phenylbenzene; 1,1'-Biphenyl; 1,1'-Diphenyl | 16 | ec:1.18.1.3 |
| cpd:C06585 |  | cis-2,3-Dihydro-2,3-dihydroxy-4'-chlorobiphenyl | 16 | ec:1.18.1.3 |
| cpd:C06584 |  | 4-Chlorobiphenyl; 1-Chloro-4-phenyl benzene; 4-Monochloro-biphenyl | 16 | ec:1.18.1.3 |
| cpd:C06579 |  | cis-2,3-Dihydroxy-2,3-dihydro-p-cumate; cis-5,6-Dihydroxy-4-isopropylcyclohexa-1,3-dienecarboxylate | 16 | ec:1.18.1.3 |
| cpd:C06578 |  | p-Cumate | 16 | ec:1.18.1.3 |
| cpd:C11588 |  | cis-3-(Carboxy-ethyl)-3,5-cyclo-hexadiene-1,2-diol; cis-3-(2-Carboxy-ethyl)-3,5-cyclo-hexadiene-1,2-diol; 3-(cis-5,6-Dihydroxycyclohexa-1,3-dien-1-yl)propanoate | 16 | ec:1.18.1.3 |
| cpd:C00090 |  | Catechol; 1,2-Benzenediol; o-Benzenediol; 1,2-Dihydroxybenzene; Brenzcatechin; Pyrocatechol | 16 | ec:1.18.1.3 |
| cpd:C12622 |  | cis-3-(3-Carboxyethenyl)-3,5-cyclohexadiene-1,2-diol; (2E)-3-(cis-5,6-Dihydroxycyclohexa-1,3-dien-1-yl)prop-2-enoate | 16 | ec:1.18.1.3 |
| cpd:C00108 |  | Anthranilate; Anthranilic acid; o-Aminobenzoic acid; Vitamin L1; 2-Aminobenzoate | 16 | ec:1.18.1.3 |
| cpd:C07111 |  | Ethylbenzene; Phenylethane; Ethylbenzol; Ethylenzene | 16 | ec:1.18.1.3 |
| cpd:C06790 |  | Trichloroethene; Trichloroethylene; TCE | 16 | ec:1.18.1.3 |
| cpd:C00423 |  | trans-Cinnamate; trans-Cinnamic acid; (E)-Cinnamate | 16 | ec:1.18.1.3 |
| cpd:C01455 |  | Toluene; Methylbenzene; Toluol | 16 | ec:1.18.1.3 |
| cpd:C04592 |  | Toluene-cis-dihydrodiol; (1S,2R)-3-Methylcyclohexa-3,5-diene-1,2-diol | 16 | ec:1.18.1.3 |
| cpd:C00230 |  | 3,4-Dihydroxybenzoate; 3,4-Dihydroxybenzoic acid; Protocatechuate; Protocatechuic acid | 15 | ec:1.14.13.2 ec:1.13.11.3 |
| cpd:C01163 |  | 3-Carboxy-cis,cis-muconate; beta-Carboxy-cis,cis-muconate; cis,cis-Butadiene-1,2,4-tricarboxylate | 15 | ec:5.5.1.2 ec:1.13.11.3 |
| cpd:C00020 |  | AMP; Adenosine 5'-monophosphate; Adenylic acid; Adenylate; 5'-AMP; 5'-Adenylic acid; 5'-Adenosine monophosphate; Adenosine 5'-phosphate | 14 | ec:2.4.2.7 ec:3.6.1.8 ec:6.1.1.22 ec:6.3.5.4 ec:6.2.1.3 ec:2.7.6.5 ec:6.1.1.17 |
| cpd:C00327 |  | L-Citrulline; 2-Amino-5-ureidovaleric acid; Citrulline | 12 | ec:2.1.3.3 |
| cpd:C00169 |  | Carbamoyl phosphate | 12 | ec:2.1.3.3 |
| cpd:C00077 |  | L-Ornithine; (S)-2,5-Diaminovaleric acid; (S)-2,5-Diaminopentanoic acid; (S)-2,5-Diaminopentanoate | 12 | ec:2.1.3.3 |
| cpd:C03058 |  | 2-Hydroxyglutaryl-CoA | 10 | ec:2.8.3.12 |
| cpd:C01278 |  | 2-Carboxy-2,5-dihydro-5-oxofuran-2-acetate; 5-Carboxy-2,5-dihydro-2-oxofuran-5-acetate; 4-Carboxymuconolactone; gamma-Carboxymuconolactone | 10 | ec:5.5.1.2 ec:4.1.1.44 |
| cpd:C00894 |  | Propenoyl-CoA; Acryloyl-CoA; Acrylyl-CoA | 10 | ec:2.8.3.12 |
| cpd:C00091 |  | Succinyl-CoA; Succinyl coenzyme A | 10 | ec:2.3.1.117 ec:2.3.1.174 |
| cpd:C03671 |  | 2-Pyrone-4,6-dicarboxylate | 10 | ec:1.13.11.3 |
| cpd:C02630 |  | 2-Hydroxyglutarate; alpha-Hydroxyglutarate | 10 | ec:2.8.3.12 |
| cpd:C00511 |  | Acrylic acid; Propenoate; Acrylate; 2-Propenoic acid; Vinylformic acid | 10 | ec:2.8.3.12 |
| cpd:C00033 |  | Acetate; Acetic acid; Ethanoic acid | 10 | ec:2.8.3.12 |
| cpd:C03586 |  | 2-Oxo-2,3-dihydrofuran-5-acetate; 3-Oxoadipate enol-lactone; 4,5-Dihydro-5-oxofuran-2-acetate; 5-Oxo-4,5-dihydrofuran-2-acetate | 10 | ec:3.1.1.24 ec:4.1.1.44 |
| cpd:C01424 |  | Gallate; Gallic acid; 3,4,5-Trihydroxybenzoic acid; 3,4,5-Trihydroxybenzoate; Pyrogallol-5-carboxylic acid | 10 | ec:1.13.11.3 |
| cpd:C15556 |  | L-3,4-Dihydroxybutan-2-one 4-phosphate; 1-Deoxy-L-glycero-tetrulose 4-phosphate; 2-Hydroxy-3-oxobutyl phosphate | 9 | ec:4.1.99.12 |
| cpd:C00199 |  | D-Ribulose 5-phosphate | 9 | ec:4.1.99.12 |
| cpd:C02232 |  | 3-Oxoadipyl-CoA; beta-Ketoadipyl-CoA | 9 | ec:2.3.1.174 ec:1.1.1.157 |
| cpd:C00064 |  | L-Glutamine; L-2-Aminoglutaramic acid | 9 | ec:6.3.5.11 ec:6.3.5.4 ec:6.3.5.9 ec:6.3.5.10 |
| cpd:C00340 |  | Reduced rubredoxin | 8 | ec:1.18.1.3 |
| cpd:C00332 |  | Acetoacetyl-CoA; Acetoacetyl coenzyme A; 3-Acetoacetyl-CoA | 8 | ec:2.3.1.9 ec:1.1.1.157 |
| cpd:C04146 |  | all-trans-Octaprenyl diphosphate; Farnesylfarnesylgeraniol | 8 |  |
| cpd:C00152 |  | L-Asparagine; 2-Aminosuccinamic acid | 8 | ec:6.1.1.22 ec:6.3.5.4 |
| cpd:C05848 |  | 4-Hydroxy-3-polyprenylbenzoate | 8 |  |
| cpd:C05847 |  | all-trans-Polyprenyl diphosphate; Polyisopentenylpyrophosphate; Polyisopentenyldiphosphate; trans-Polyisopentenyldiphosphate; Polyprenyl diphosphate | 8 |  |
| cpd:C05809 |  | 3-Octaprenyl-4-hydroxybenzoate; 4-Hydroxy-3-octaprenylbenzoate | 8 |  |
| cpd:C00435 |  | Oxidized rubredoxin | 8 | ec:1.18.1.3 |
| cpd:C03912 |  | (S)-1-Pyrroline-5-carboxylate; L-1-Pyrroline-5-carboxylate; 1-Pyrroline-5-carboxylate | 7 | ec:1.5.1.2 |
| cpd:C00148 |  | L-Proline; 2-Pyrrolidinecarboxylic acid | 7 | ec:1.5.1.2 |
| cpd:C01157 |  | Hydroxyproline; L-Hydroxyproline; trans-4-Hydroxy-L-proline | 7 | ec:1.5.1.2 |
| cpd:C04281 |  | L-1-Pyrroline-3-hydroxy-5-carboxylate; 3-Hydroxy-L-1-pyrroline-5-carboxylate; (3R,5S)-1-Pyrroline-3-hydroxy-5-carboxylate | 7 | ec:1.5.1.2 |
| cpd:C00049 |  | L-Aspartate; L-Aspartic acid; 2-Aminosuccinic acid; L-Asp | 7 | ec:6.3.5.4 |
| cpd:C00670 |  | sn-Glycero-3-phosphocholine; Glycerophosphocholine | 6 | ec:3.1.4.46 |
| cpd:C00189 |  | Ethanolamine; Aminoethanol; 2-Hydroxyethylamine | 6 | ec:3.1.4.46 |
| cpd:C01233 |  | sn-Glycero-3-phosphoethanolamine; Glycerophosphoethanolamine | 6 | ec:3.1.4.46 |
| cpd:C00144 |  | GMP; Guanosine 5'-phosphate; Guanosine monophosphate; Guanosine 5'-monophosphate; Guanylic acid | 6 | ec:2.4.2.7 ec:3.6.1.8 ec:1.7.1.7 ec:2.4.2.22 |
| cpd:C00114 |  | Choline; Bilineurine | 6 | ec:3.1.4.46 |
| cpd:C03067 |  | 3-Hydroxybenzaldehyde | 5 | ec:1.2.1.28 |
| cpd:C00261 |  | Benzaldehyde; Benzoic aldehyde | 5 | ec:1.2.1.28 |
| cpd:C00633 |  | 4-Hydroxybenzaldehyde; p-Hydroxybenzaldehyde | 5 | ec:1.2.1.28 |
| cpd:C00234 |  | 10-Formyltetrahydrofolate; 10-Formyl-THF | 5 | ec:2.1.2.2 ec:3.5.1.10 |
| cpd:C04462 |  | N-Succinyl-2-L-amino-6-oxoheptanedioate; N-Succinyl-L-2-amino-6-oxoheptanedioate; N-Succinyl-L-2-amino-6-oxopimelate; N-Succinyl-2-amino-6-oxo-L-pimelic acid; N-Succinyl-epsilon-keto-L-aminopimelic acid; (S)-2-(Succinylamino)-6-oxoheptanedioate | 5 | ec:2.3.1.117 |
| cpd:C00587 |  | 3-Hydroxybenzoate; 3-Hydroxybenzoic acid; m-Hydroxybenzoic acid | 5 | ec:1.2.1.28 |
| cpd:C00180 |  | Benzoate; Benzoic acid; Benzenecarboxylic acid; Phenylformic acid; Dracylic acid | 5 | ec:1.2.1.28 |
| cpd:C02714 |  | N-Acetylputrescine | 5 | ec:1.4.3.4 ec:2.3.1.57 |
| cpd:C07215 |  | o-Toluate; o-Methylbenzoate; o-Toluic Acid; 2-Methylbenzoic acid | 5 | ec:1.2.1.28 |
| cpd:C07214 |  | 2-Methylbenzaldehyde; o-Toluic aldehyde; 2-Formyltoluene; o-Tolualdehyde | 5 | ec:1.2.1.28 |
| cpd:C07211 |  | m-Methylbenzoate; m-Toluic Acid; beta-Bethylbenzoic acid; m-Toluylic acid | 5 | ec:1.2.1.28 |
| cpd:C07209 |  | 3-Methylbenzaldehyde; m-Tolualdehyde | 5 | ec:1.2.1.28 |
| cpd:C00119 |  | 5-Phospho-alpha-D-ribose 1-diphosphate; 5-Phosphoribosyl diphosphate; 5-Phosphoribosyl 1-pyrophosphate; PRPP | 5 | ec:2.4.2.9 ec:2.4.2.7 ec:2.4.2.22 |
| cpd:C00118 |  | D-Glyceraldehyde 3-phosphate; (2R)-2-Hydroxy-3-(phosphonooxy)-propanal; Glyceraldehyde 3-phosphate | 5 | ec:1.2.1.12 ec:4.2.1.20 ec:4.1.3.16 ec:5.3.1.1 ec:4.1.2.14 |
| cpd:C00075 |  | UTP; Uridine 5'-triphosphate; Uridine triphosphate | 5 | ec:2.7.7.6 ec:3.5.4.13 ec:3.6.1.8 ec:2.7.1.48 |
| cpd:C00101 |  | Tetrahydrofolate; 5,6,7,8-Tetrahydrofolate; Tetrahydrofolic acid; THF; (6S)-Tetrahydrofolate; (6S)-Tetrahydrofolic acid; (6S)-THFA | 5 | ec:2.1.2.2 ec:3.5.1.10 |
| cpd:C00846 |  | 3-Oxoadipate; 3-Oxoadipic acid; 3-Keto-adipate | 5 | ec:3.1.1.24 |
| cpd:C00044 |  | GTP; Guanosine 5'-triphosphate | 5 | ec:2.7.7.6 ec:3.6.1.8 ec:2.7.6.5 ec:2.7.1.48 |
| cpd:C00030 |  | Reduced acceptor; AH2; Hydrogen-donor; Donor | 5 | ec:1.8.99.1 ec:1.8.99.3 ec:1.4.99.1 |
| cpd:C00028 |  | Acceptor; Hydrogen-acceptor; A; Oxidized donor | 5 | ec:1.8.99.1 ec:1.8.99.3 ec:1.4.99.1 |
| cpd:C03972 |  | 2,3,4,5-Tetrahydrodipicolinate; delta1-Piperidine-2,6-dicarboxylate; L-2,3,4,5-Tetrahydrodipicolinate; (S)-2,3,4,5-Tetrahydropyridine-2,6-dicarboxylate | 5 | ec:2.3.1.117 |
| cpd:C01454 |  | Toluate; p-Toluate; p-Toluic acid; 4-Methylbenzoic acid; Toluenecarboxylic acid; Crithminic acid | 5 | ec:1.2.1.28 |
| cpd:C06758 |  | p-Tolualdehyde; p-Methylbenzaldehyde; 4-Methylbenzaldehyde; 4-Toluylaldehyde; p-Formyltoluene | 5 | ec:1.2.1.28 |
| cpd:C14145 |  | (3S)-3-Hydroxyadipyl-CoA | 4 | ec:1.1.1.157 |
| cpd:C04144 |  | Tetrahydropteroyltri-L-glutamate | 4 | ec:2.1.1.14 |
| cpd:C04489 |  | 5-Methyltetrahydropteroyltri-L-glutamate | 4 | ec:2.1.1.14 |
| cpd:C05167 |  | alpha-Amino acid | 4 | ec:1.4.99.1 |
| cpd:C00242 |  | Guanine; 2-Amino-6-hydroxypurine | 4 | ec:2.4.2.7 ec:2.4.2.22 |
| cpd:C05116 |  | 3-Hydroxybutanoyl-CoA; 3-Hydroxybutyryl-CoA | 4 | ec:1.1.1.157 |
| cpd:C00166 |  | Phenylpyruvate; Phenylpyruvic acid; alpha-Ketohydrocinnamic acid; keto-Phenylpyruvate; 3-Phenyl-2-oxopropanoate; 2-Oxo-3-phenylpropanoate | 4 | ec:1.4.99.1 |
| cpd:C00161 |  | 2-Oxo acid; 2-Oxocarboxylate | 4 | ec:1.4.99.1 |
| cpd:C00155 |  | L-Homocysteine; L-2-Amino-4-mercaptobutyric acid | 4 | ec:2.1.1.14 |
| cpd:C02265 |  | D-Phenylalanine; D-alpha-Amino-beta-phenylpropionic acid | 4 | ec:1.4.99.1 |
| cpd:C00136 |  | Butanoyl-CoA; Butyryl-CoA | 4 | ec:2.3.1.9 |
| cpd:C00134 |  | Putrescine; 1,4-Butanediamine; 1,4-Diaminobutane; Tetramethylenediamine; Butane-1,4-diamine | 4 | ec:2.3.1.57 |
| cpd:C00073 |  | L-Methionine; Methionine; L-2-Amino-4methylthiobutyric acid | 4 | ec:2.1.1.14 |
| cpd:C01144 |  | (S)-3-Hydroxybutanoyl-CoA; (S)-3-Hydroxybutyryl-CoA | 4 | ec:1.1.1.157 |
| cpd:C00063 |  | CTP; Cytidine 5'-triphosphate; Cytidine triphosphate | 4 | ec:2.7.7.6 ec:3.5.4.13 ec:3.6.1.8 |
| cpd:C05698 |  | Selenohomocysteine | 4 | ec:2.1.1.14 |
| cpd:C05335 |  | L-Selenomethionine | 4 | ec:2.1.1.14 |
| cpd:C00022 |  | Pyruvate; Pyruvic acid; 2-Oxopropanoate; 2-Oxopropanoic acid; Pyroracemic acid | 4 | ec:2.2.1.6 ec:4.1.3.16 ec:3.7.1.20 ec:4.1.3.40 ec:4.1.2.14 |
| cpd:C05269 |  | 3-Oxohexanoyl-CoA; 3-Ketohexanoyl-CoA | 4 | ec:2.3.1.9 |
| cpd:C00251 |  | Chorismate; Chorismic acid | 3 | ec:5.4.99.5 ec:4.1.3.40 ec:1.3.1.12 |
| cpd:C00094 |  | Sulfite; Sulfurous acid | 3 | ec:1.8.99.1 ec:1.8.99.3 ec:2.8.1.1 |
| cpd:C00105 |  | UMP; Uridylic acid; Uridine monophosphate; Uridine 5'-monophosphate; 5'Uridylic acid | 3 | ec:2.4.2.9 ec:3.6.1.8 ec:2.7.1.48 |
| cpd:C00065 |  | L-Serine; L-2-Amino-3-hydroxypropionic acid; L-3-Hydroxy-alanine; Serine | 3 | ec:4.2.1.20 ec:3.1.3.3 |
| cpd:C00458 |  | dCTP; Deoxycytidine 5'-triphosphate; Deoxycytidine triphosphate; 2'-Deoxycytidine 5'-triphosphate | 3 | ec:2.7.7.7 ec:3.5.4.13 ec:2.7.1.48 |
| cpd:C00345 |  | 6-Phospho-D-gluconate | 2 | ec:3.1.1.31 ec:4.2.1.12 |
| cpd:C00299 |  | Uridine | 2 | ec:3.5.4.5 ec:2.7.1.48 |
| cpd:C00320 |  | Thiosulfate; Hyposulfite | 2 | ec:2.8.1.1 |
| cpd:C00286 |  | dGTP; 2'-Deoxyguanosine 5'-triphosphate; Deoxyguanosine 5'-triphosphate; Deoxyguanosine triphosphate | 2 | ec:2.7.7.7 ec:2.7.1.48 |
| cpd:C01755 |  | Thiocyanate; Thiocyanic acid | 2 | ec:2.8.1.1 |
| cpd:C00279 |  | D-Erythrose 4-phosphate | 2 | ec:2.5.1.54 |
| cpd:C03506 |  | Indoleglycerol phosphate; 1-C-(Indol-3-yl)glycerol 3-phosphate; (3-Indolyl)-glycerol phosphate; C1-(3-Indolyl)-glycerol 3-phosphate; (1S,2R)-1-C-(Indol-3-yl)glycerol 3-phosphate; Indole-3-glycerol phosphate | 2 | ec:4.2.1.20 |
| cpd:C05577 |  | 3,4-Dihydroxymandelaldehyde; 3,4-Dihydroxyphenylglycolaldehyde | 2 | ec:1.4.3.4 ec:1.1.1.1 |
| cpd:C00267 |  | alpha-D-Glucose | 2 | ec:5.1.3.3 ec:3.2.1.20 |
| cpd:C00655 |  | Xanthosine 5'-phosphate; Xanthylic acid; XMP; (9-D-Ribosylxanthine)-5'-phosphate | 2 | ec:2.4.2.22 |
| cpd:C00255 |  | Riboflavin; Lactoflavin; 7,8-Dimethyl-10-ribitylisoalloxazine; Vitamin B2 | 2 | ec:2.5.1.9 |
| cpd:C00254 |  | Prephenate; Prephenic acid | 2 | ec:5.4.99.5 ec:1.3.1.12 |
| cpd:C00631 |  | 2-Phospho-D-glycerate; D-Glycerate 2-phosphate; 2-Phospho-(R)-glycerate | 2 | ec:5.4.2.12 ec:4.2.1.11 |
| cpd:C00229 |  | Acyl-carrier protein; ACP; [Acyl-carrier protein]; Holo-[acyl-carrier protein] | 2 | ec:2.3.1.180 ec:2.3.1.15 |
| cpd:C04051 |  | 5-Amino-4-imidazolecarboxyamide | 2 | ec:2.4.2.7 |
| cpd:C00177 |  | Cyanide; Prussiate; CN-; Cyano | 2 | ec:2.8.1.1 |
| cpd:C04442 |  | 2-Dehydro-3-deoxy-6-phospho-D-gluconate; 6-Phospho-2-dehydro-3-deoxy-D-gluconate; 2-Keto-3-deoxy-6-phosphogluconate; 2-Dehydro-3-deoxy-D-gluconate 6-phosphate | 2 | ec:4.1.3.16 ec:4.2.1.12 ec:4.1.2.14 |
| cpd:C01236 |  | D-Glucono-1,5-lactone 6-phosphate; 6-Phospho-D-glucono-1,5-lactone | 2 | ec:1.1.1.49 ec:3.1.1.31 |
| cpd:C00147 |  | Adenine; 6-Aminopurine | 2 | ec:2.4.2.7 |
| cpd:C00536 |  | Triphosphate; Triphosphoric acid; Tripolyphosphate; Inorganic triphosphate | 2 | ec:2.5.1.17 ec:4.1.2.50 ec:4.2.3.12 |
| cpd:C01179 |  | 3-(4-Hydroxyphenyl)pyruvate; 4-Hydroxyphenylpyruvate; p-Hydroxyphenylpyruvic acid | 2 | ec:5.4.99.5 ec:1.3.1.12 |
| cpd:C00131 |  | dATP; 2'-Deoxyadenosine 5'-triphosphate; Deoxyadenosine 5'-triphosphate; Deoxyadenosine triphosphate | 2 | ec:2.7.7.7 ec:2.7.1.48 |
| cpd:C00130 |  | IMP; Inosinic acid; Inosine monophosphate; Inosine 5'-monophosphate; Inosine 5'-phosphate; 5'-Inosinate; 5'-Inosinic acid; 5'-Inosine monophosphate; 5'-IMP | 2 | ec:3.6.1.8 ec:1.7.1.7 |
| cpd:C00126 |  | Ferrocytochrome c; Cytochrome c2+; Reduced cytochrome c | 2 | ec:1.9.3.1 |
| cpd:C00125 |  | Ferricytochrome c; Cytochrome c3+ | 2 | ec:1.9.3.1 |
| cpd:C00122 |  | Fumarate; Fumaric acid; trans-Butenedioic acid | 2 | ec:4.2.1.2 ec:3.7.1.20 |
| cpd:C00084 |  | Acetaldehyde; Ethanal | 2 | ec:1.1.1.1 ec:4.1.2.50 ec:4.2.3.12 |
| cpd:C00081 |  | ITP; Inosine 5'-triphosphate; Inosine triphosphate; Inosine tripolyphosphate | 2 | ec:3.6.1.8 ec:2.7.1.48 |
| cpd:C06506 |  | Adenosyl cobyrinate a,c diamide; Adenosyl cobyrinate diamide; Adenosylcob(III)yrinic acid a,c-diamide; Adenosylcobyrinic acid a,c-diamide | 2 | ec:2.5.1.17 ec:6.3.5.10 |
| cpd:C00475 |  | Cytidine | 2 | ec:3.5.4.5 ec:2.7.1.48 |
| cpd:C00078 |  | L-Tryptophan; Tryptophan; (S)-alpha-Amino-beta-(3-indolyl)-propionic acid | 2 | ec:4.2.1.20 |
| cpd:C04732 |  | 5-Amino-6-(1-D-ribitylamino)uracil; 5-Amino-6-(D-ribitylamino)uracil; 6-(1-D-Ribitylamino)-5-amino-2,4-dihydroxypyrimidine; 6-(1-D-Ribitylamino)-5-aminouracil; 4-(1-D-Ribitylamino)-5-amino-2,6-dihydroxypyrimidine | 2 | ec:2.5.1.9 |
| cpd:C00463 |  | Indole; 2,3-Benzopyrrole | 2 | ec:4.2.1.20 |
| cpd:C00103 |  | D-Glucose 1-phosphate; alpha-D-Glucose 1-phosphate; Cori ester; D-Glucose alpha-1-phosphate | 2 | ec:3.6.1.8 ec:2.4.1.1 |
| cpd:C00460 |  | dUTP; 2'-Deoxyuridine 5'-triphosphate | 2 | ec:3.5.4.13 ec:2.7.1.48 |
| cpd:C04332 |  | 6,7-Dimethyl-8-(D-ribityl)lumazine | 2 | ec:2.5.1.9 |
| cpd:C04691 |  | 2-Dehydro-3-deoxy-D-arabino-heptonate 7-phosphate; 3-Deoxy-D-arabino-hept-2-ulosonate 7-phosphate; 3-Deoxy-arabino-heptulonate 7-phosphate; 3-Deoxy-D-arabino-heptulosonic acid 7-phosphate; DAHP; 2-Dahp | 2 | ec:2.5.1.54 |
| cpd:C00459 |  | dTTP; Deoxythymidine triphosphate; Deoxythymidine 5'-triphosphate; TTP | 2 | ec:2.7.7.7 ec:2.7.1.48 |
| cpd:C00055 |  | CMP; Cytidine-5'-monophosphate; Cytidylic acid | 2 | ec:3.6.1.8 ec:2.7.1.48 |
| cpd:C04677 |  | 1-(5'-Phosphoribosyl)-5-amino-4-imidazolecarboxamide; 5'-Phosphoribosyl-5-amino-4-imidazolecarboxamide; 5'-Phospho-ribosyl-5-amino-4-imidazole carboxamide; AICAR; 5-Aminoimidazole-4-carboxamide ribotide; 5-Phosphoribosyl-4-carbamoyl-5-aminoimidazole; 5-Amino-1-(5-phospho-D-ribosyl)imidazole-4-carboxamide | 2 | ec:2.4.2.7 |
| cpd:C00046 |  | RNA; RNAn; RNAn+1; RNA(linear); (Ribonucleotide)n; (Ribonucleotide)m; (Ribonucleotide)n+m; Ribonucleic acid | 2 | ec:2.7.7.6 |
| cpd:C00385 |  | Xanthine | 2 | ec:2.4.2.22 |
| cpd:C05639 |  | 4,6-Dihydroxyquinoline; Quinoline-4,6-diol | 1 | ec:1.4.3.4 |
| cpd:C05638 |  | 5-Hydroxykynurenamine | 1 | ec:1.4.3.4 |
| cpd:C05637 |  | 4,8-Dihydroxyquinoline; Quinoline-4,8-diol | 1 | ec:1.4.3.4 |
| cpd:C05636 |  | 3-Hydroxykynurenamine | 1 | ec:1.4.3.4 |
| cpd:C05634 |  | 5-Hydroxyindoleacetaldehyde | 1 | ec:1.4.3.4 |
| cpd:C01005 |  | O-Phospho-L-serine; L-O-Phosphoserine; 3-Phosphoserine; Dexfosfoserine | 1 | ec:3.1.3.3 |
| cpd:C00681 |  | 1-Acyl-sn-glycerol 3-phosphate; 2-Lysophosphatidate; Lysophosphatidate; Lysophosphatidic acid | 1 | ec:2.3.1.15 |
| cpd:C00283 |  | Hydrogen sulfide; Hydrogen-sulfide; H2S; Sulfide | 1 | ec:1.8.99.1 ec:1.8.99.3 |
| cpd:C19846 |  | Pimeloyl-[acyl-carrier protein] methyl ester; Pimeloyl-[acp] methyl ester; Pimelyl-[acyl-carrier protein] methyl ester; Pimelyl-[acp] methyl ester | 1 | ec:3.1.1.85 |
| cpd:C19845 |  | Pimeloyl-[acyl-carrier protein]; Pimeloyl-[acp]; Pimelyl-[acyl-carrier protein]; Pimelyl-[acp]; 7-Hydroxy-7-oxoheptanoyl-[acyl-carrier protein] | 1 | ec:3.1.1.85 |
| cpd:C04153 |  | rRNA containing N2-methylguanine | 1 | ec:2.1.1.171 |
| cpd:C00718 |  | Amylose; Amylose chain; (1,4-alpha-D-Glucosyl)n; (1,4-alpha-D-Glucosyl)n+1; (1,4-alpha-D-Glucosyl)n-1; 4-{(1,4)-alpha-D-Glucosyl}(n-1)-D-glucose; 1,4-alpha-D-Glucan | 1 | ec:2.4.1.1 |
| cpd:C14099 |  | 2-Naphthaldehyde; 2-Naphthalenecarboxaldehyde | 1 | ec:1.1.1.1 |
| cpd:C05589 |  | L-Normetanephrine | 1 | ec:1.4.3.4 |
| cpd:C05588 |  | L-Metanephrine | 1 | ec:1.4.3.4 |
| cpd:C05587 |  | 3-Methoxytyramine | 1 | ec:1.4.3.4 |
| cpd:C05583 |  | 3-Methoxy-4-hydroxyphenylglycolaldehyde | 1 | ec:1.4.3.4 |
| cpd:C14090 |  | 1-Naphthaldehyde; 1-Formylnaphthalene | 1 | ec:1.1.1.1 |
| cpd:C05581 |  | 3-Methoxy-4-hydroxyphenylacetaldehyde; (4-Hydroxy-3-methoxyphenyl)acetaldehyde; Homovanillin | 1 | ec:1.4.3.4 |
| cpd:C00705 |  | dCDP; 2'-Deoxycytidine diphosphate; 2'-Deoxycytidine 5'-diphosphate | 1 | ec:2.7.1.48 |
| cpd:C20239 |  | 6-Carboxy-5,6,7,8-tetrahydropterin; 6-Carboxytetrahydropterin | 1 | ec:4.1.2.50 ec:4.2.3.12 |
| cpd:C01346 |  | dUDP; 2'-Deoxyuridine 5'-diphosphate | 1 | ec:2.7.1.48 |
| cpd:C14089 |  | 1-Hydroxymethylnaphthalene; 1-Naphthalenemethanol | 1 | ec:1.1.1.1 |
| cpd:C05576 |  | 3,4-Dihydroxyphenylethyleneglycol | 1 | ec:1.1.1.1 |
| cpd:C16635 |  | 5'-Deoxy-5-fluorocytidine | 1 | ec:3.5.4.5 |
| cpd:C16634 |  | 5-Fluorouridine monophosphate | 1 | ec:2.7.1.48 |
| cpd:C16633 |  | 5-Fluorouridine | 1 | ec:2.7.1.48 |
| cpd:C04895 |  | 7,8-Dihydroneopterin 3'-triphosphate; 2-Amino-4-hydroxy-6-(erythro-1,2,3-trihydroxypropyl)dihydropteridine triphosphate; 6-(L-erythro-1,2-Dihydroxypropyl 3-triphosphate)-7,8-dihydropterin; 6-[(1S,2R)-1,2-Dihydroxy-3-triphosphooxypropyl]-7,8-dihydropterin | 1 | ec:4.1.2.50 ec:4.2.3.12 |
| cpd:C16596 |  | 5-Phenyl-1,3-oxazinane-2,4-dione | 1 | ec:1.1.1.1 |
| cpd:C16595 |  | 4-Hydroxy-5-phenyltetrahydro-1,3-oxazin-2-one | 1 | ec:1.1.1.1 |
| cpd:C04494 |  | Guanosine 3'-diphosphate 5'-triphosphate; Guanosine 5'-triphosphate,3'-diphosphate | 1 | ec:2.7.6.5 |
| cpd:C16587 |  | 3-Carbamoyl-2-phenylpropionaldehyde | 1 | ec:1.1.1.1 |
| cpd:C16586 |  | 2-Phenyl-1,3-propanediol monocarbamate | 1 | ec:1.1.1.1 |
| cpd:C01328 |  | HO-; OH-; Hydroxide ion | 1 | ec:1.8.99.1 ec:1.8.99.3 |
| cpd:C00249 |  | Hexadecanoic acid; Hexadecanoate; Hexadecylic acid; Palmitic acid; Palmitate; Cetylic acid | 1 | ec:6.2.1.3 |
| cpd:C03838 |  | 5'-Phosphoribosylglycinamide; GAR; N1-(5-Phospho-D-ribosyl)glycinamide; Glycinamide ribonucleotide | 1 | ec:2.1.2.2 |
| cpd:C16612 |  | Citalopram aldehyde | 1 | ec:1.4.3.4 |
| cpd:C00240 |  | rRNA; Ribosomal RNA | 1 | ec:2.1.1.171 |
| cpd:C12739 |  | 5'-Deoxy-5-fluorouridine; Doxifluridine | 1 | ec:3.5.4.5 |
| cpd:C05946 |  | D-4-Hydroxy-2-oxoglutarate | 1 | ec:4.1.3.16 ec:4.1.2.14 |
| cpd:C00637 |  | Indole-3-acetaldehyde; 2-(Indol-3-yl)acetaldehyde; Indoleacetaldehyde | 1 | ec:1.4.3.4 |
| cpd:C16609 |  | Didemethylcitalopram | 1 | ec:1.4.3.4 |
| cpd:C16608 |  | Demethylcitalopram | 1 | ec:1.4.3.4 |
| cpd:C00236 |  | 3-Phospho-D-glyceroyl phosphate; 1,3-Bisphospho-D-glycerate; (R)-2-Hydroxy-3-(phosphonooxy)-1-monoanhydride with phosphoric propanoic acid; D-Glycerate 1,3-diphosphate | 1 | ec:1.2.1.12 |
| cpd:C00197 |  | 3-Phospho-D-glycerate; D-Glycerate 3-phosphate; 3-Phospho-(R)-glycerate; 3-Phosphoglycerate | 1 | ec:5.4.2.12 |
| cpd:C05936 |  | N4-Acetylaminobutanal; 4-Acetamidobutanal | 1 | ec:1.4.3.4 |
| cpd:C06613 |  | trans-3-Chloroallyl aldehyde; trans-3-Chloro-2-propenal | 1 | ec:1.1.1.1 |
| cpd:C06612 |  | cis-3-Chloro-2-propene-1-ol; cis-3-Chloroallyl alcohol | 1 | ec:1.1.1.1 |
| cpd:C06611 |  | trans-3-Chloro-2-propene-1-ol; trans-3-Chloroallyl alcohol | 1 | ec:1.1.1.1 |
| cpd:C00226 |  | Primary alcohol; 1-Alcohol | 1 | ec:1.1.1.1 |
| cpd:C00221 |  | beta-D-Glucose | 1 | ec:5.1.3.3 |
| cpd:C16551 |  | Alcophosphamide | 1 | ec:1.1.1.1 |
| cpd:C07645 |  | Aldophosphamide | 1 | ec:1.1.1.1 |
| cpd:C02336 |  | beta-D-Fructose; beta-Fruit sugar; beta-D-arabino-Hexulose; beta-Levulose; Fructose | 1 | ec:3.2.1.20 |
| cpd:C03373 |  | Aminoimidazole ribotide; AIR; 1-(5'-Phosphoribosyl)-5-aminoimidazole; 5'-Phosphoribosyl-5-aminoimidazole; 1-(5-Phospho-D-ribosyl)-5-aminoimidazole; 5-Amino-1-(5-phospho-D-ribosyl)imidazole | 1 | ec:6.3.3.1 |
| cpd:C00218 |  | Methylamine; Methanamine | 1 | ec:1.4.3.4 |
| cpd:C01250 |  | N-Acetyl-L-glutamate 5-semialdehyde; 2-Acetamido-5-oxopentanoate | 1 | ec:2.6.1.11 |
| cpd:C05127 |  | N-Methylhistamine; 1-Methylhistamine; 1-Methyl-4-(2-aminoethyl)imidazole; Ntau-Methylhistamine | 1 | ec:1.4.3.4 |
| cpd:C05125 |  | 2-(alpha-Hydroxyethyl)thiamine diphosphate; 2-Hydroxyethyl-ThPP | 1 | ec:2.2.1.6 |
| cpd:C03765 |  | 4-Hydroxyphenylacetaldehyde; 2-(4-Hydroxyphenyl)acetaldehyde | 1 | ec:1.4.3.4 |
| cpd:C00173 |  | Acyl-[acyl-carrier protein] | 1 | ec:2.3.1.15 |
| cpd:C03402 |  | L-Asparaginyl-tRNA(Asn); Asn-tRNA(Asn); Asparaginyl-tRNA(Asn) | 1 | ec:6.1.1.22 |
| cpd:C04043 |  | 3,4-Dihydroxyphenylacetaldehyde; Protocatechuatealdehyde | 1 | ec:1.4.3.4 |
| cpd:C01641 |  | tRNA(Glu) | 1 | ec:6.1.1.17 |
| cpd:C00601 |  | Phenylacetaldehyde; alpha-Tolualdehyde | 1 | ec:1.4.3.4 |
| cpd:C00208 |  | Maltose; Malt sugar; alpha-D-Glucopyranosyl-(1->4)-D-glucopyranose | 1 | ec:3.2.1.20 |
| cpd:C00206 |  | dADP; 2'-Deoxyadenosine 5'-diphosphate | 1 | ec:2.7.1.48 |
| cpd:C04832 |  | Coenzyme M 7-mercaptoheptanoylthreonine-phosphate heterodisulfide; Coenzyme M-HTP heterodisulfide; CoM-S-S-CoB; N-{7-[(2-Sulfoethyl)dithio]heptanoyl}-3-O-phospho-L-threonine | 1 | ec:1.8.98.1 |
| cpd:C03758 |  | Dopamine; 4-(2-Aminoethyl)-1,2-benzenediol; 4-(2-Aminoethyl)benzene-1,2-diol; 3,4-Dihydroxyphenethylamine; 2-(3,4-Dihydroxyphenyl)ethylamine | 1 | ec:1.4.3.4 |
| cpd:C01637 |  | tRNA(Asn) | 1 | ec:6.1.1.22 |
| cpd:C06143 |  | Poly-beta-hydroxybutyrate | 1 |  |
| cpd:C00154 |  | Palmitoyl-CoA; Hexadecanoyl-CoA | 1 | ec:6.2.1.3 |
| cpd:C06899 |  | Chloral hydrate | 1 | ec:1.1.1.1 |
| cpd:C07572 |  | Citalopram | 1 | ec:1.4.3.4 |
| cpd:C00547 |  | L-Noradrenaline; Noradrenaline; Norepinephrine; Arterenol; 4-[(1R)-2-Amino-1-hydroxyethyl]-1,2-benzenediol | 1 | ec:1.4.3.4 |
| cpd:C00546 |  | Methylglyoxal; Pyruvaldehyde; Pyruvic aldehyde; 2-Ketopropionaldehyde; 2-Oxopropanal | 1 | ec:1.4.3.4 |
| cpd:C00543 |  | Dimethylamine; (CH3)2NH | 1 | ec:1.4.3.4 |
| cpd:C00149 |  | (S)-Malate; L-Malate; L-Apple acid; L-Malic acid; L-2-Hydroxybutanedioic acid; Malate; Malic acid | 1 | ec:4.2.1.2 |
| cpd:C04376 |  | 5'-Phosphoribosyl-N-formylglycinamide; N-Formyl-GAR; N-Formylglycinamide ribonucleotide; N2-Formyl-N1-(5-phospho-D-ribosyl)glycinamide | 1 | ec:2.1.2.2 |
| cpd:C01177 |  | Inositol 1-phosphate; myo-Inositol 1-phosphate; 1D-myo-Inositol 1-phosphate; D-myo-Inositol 1-phosphate; 1D-myo-Inositol 1-monophosphate | 1 | ec:3.1.3.25 |
| cpd:C00137 |  | myo-Inositol; D-myo-Inositol; 1D-myo-Inositol; L-myo-Inositol; 1L-myo-Inositol; meso-Inositol; Inositol; Dambose; Cyclohexitol; Meat sugar; Bios I | 1 | ec:3.1.3.25 |
| cpd:C01172 |  | beta-D-Glucose 6-phosphate | 1 | ec:1.1.1.49 |
| cpd:C00132 |  | Methanol; Methyl alcohol; CH3OH | 1 | ec:3.1.1.85 |
| cpd:C04006 |  | 1D-myo-Inositol 3-phosphate; D-myo-Inositol 3-phosphate; myo-Inositol 3-phosphate; Inositol 3-phosphate; 1D-myo-Inositol 3-monophosphate; D-myo-Inositol 3-monophosphate; myo-Inositol 3-monophosphate; Inositol 3-monophosphate; 1L-myo-Inositol 1-phosphate; L-myo-Inositol 1-phosphate | 1 | ec:3.1.3.25 |
| cpd:C00095 |  | D-Fructose; Levulose; Fruit sugar; D-arabino-Hexulose | 1 | ec:3.2.1.20 |
| cpd:C03684 |  | 6-Pyruvoyltetrahydropterin; 6-(1,2-Dioxopropyl)-5,6,7,8-tetrahydropterin; 6-Pyruvoyl-5,6,7,8-tetrahydropterin | 1 | ec:4.1.2.50 ec:4.2.3.12 |
| cpd:C01209 |  | Malonyl-[acyl-carrier protein]; Malonyl-[acp] | 1 | ec:2.3.1.180 |
| cpd:C00526 |  | Deoxyuridine; 2-Deoxyuridine; 2'-Deoxyuridine | 1 | ec:3.5.4.5 |
| cpd:C00881 |  | Deoxycytidine; 2'-Deoxycytidine | 1 | ec:3.5.4.5 |
| cpd:C00483 |  | Tyramine; 2-(p-Hydroxyphenyl)ethylamine | 1 | ec:1.4.3.4 |
| cpd:C00089 |  | Sucrose; Cane sugar; Saccharose; 1-alpha-D-Glucopyranosyl-2-beta-D-fructofuranoside | 1 | ec:3.2.1.20 |
| cpd:C00085 |  | D-Fructose 6-phosphate; D-Fructose 6-phosphoric acid; Neuberg ester | 1 | ec:3.1.3.11 |
| cpd:C06508 |  | Adenosyl cobinamide | 1 | ec:2.5.1.17 |
| cpd:C06507 |  | Adenosyl cobyrinate hexaamide; Adenosylcobyric acid | 1 | ec:6.3.5.10 |
| cpd:C05827 |  | Methylimidazole acetaldehyde; 1-Methylimidazole-4-acetaldehyde; Methylimidazoleacetaldehyde | 1 | ec:1.4.3.4 |
| cpd:C06505 |  | Cob(I)yrinate a,c diamide; Cob(I)yrinate diamide; Cob(I)yrinic acid a,c-diamide | 1 | ec:2.5.1.17 |
| cpd:C06504 |  | Cob(II)yrinate a,c diamide; Cob(II)yrinate diamide; Cob(II)yrinic acid a,c-diamide; Cobyrinate a,c-diamide; Cobyrinic acid a,c-diamide | 1 | ec:6.3.5.11 ec:6.3.5.9 |
| cpd:C06503 |  | Hydrogenobyrinate a,c diamide; Hydrogenobyrinate diamide; Hydrogenobyrinic acid a,c-diamide | 1 | ec:6.3.5.11 ec:6.3.5.9 |
| cpd:C00473 |  | Retinol; all-trans-Retinol; Vitamin A; Vitamin A1 | 1 | ec:1.1.1.1 |
| cpd:C02987 |  | L-Glutamyl-tRNA(Glu) | 1 | ec:6.1.1.17 |
| cpd:C00071 |  | Aldehyde; RCHO | 1 | ec:1.1.1.1 |
| cpd:C00900 |  | 2-Acetolactate | 1 | ec:2.2.1.6 |
| cpd:C00469 |  | Ethanol; Ethyl alcohol; Methylcarbinol | 1 | ec:1.1.1.1 |
| cpd:C00109 |  | 2-Oxobutanoate; 2-Ketobutyric acid; 2-Oxobutyric acid; 2-Oxobutyrate; 2-Oxobutanoic acid; alpha-Ketobutyric acid; alpha-Ketobutyrate | 1 | ec:2.2.1.6 |
| cpd:C05774 |  | Cobinamide; Cob(I)inamide | 1 | ec:2.5.1.17 |
| cpd:C05773 |  | Cobyrinate; Cobyrinic acid; Cob(II)yrinate; Cob(II)yrinic acid | 1 | ec:6.3.5.11 ec:6.3.5.9 |
| cpd:C07490 |  | Trichloroethanol; 2,2,2-Trichloroethanol | 1 | ec:1.1.1.1 |
| cpd:C00106 |  | Uracil | 1 | ec:2.4.2.9 |
| cpd:C00104 |  | IDP; Inosine 5'-diphosphate; Inosine diphosphate | 1 | ec:2.7.1.48 |
| cpd:C05378 |  | beta-D-Fructose 1,6-bisphosphate | 1 | ec:3.1.3.11 |
| cpd:C00068 |  | Thiamin diphosphate; Thiamine diphosphate; Thiamin pyrophosphate; TPP; ThPP | 1 | ec:2.2.1.6 |
| cpd:C16399 |  | 2,4-Diamino-6-hydroxylaminotoluene | 1 | ec:1.8.99.1 ec:1.8.99.3 |
| cpd:C11481 |  | HSO3-; Hydrogen sulfite; Bisulfite | 1 | ec:1.8.99.1 ec:1.8.99.3 |
| cpd:C00060 |  | Carboxylate; R-COOH; Monocarboxylate; Carboxylic acid | 1 | ec:3.5.1.11 |
| cpd:C16393 |  | 2-Hydroxylamino-4,6-dinitrotoluene | 1 |  |
| cpd:C16392 |  | 4-Hydroxylamino-2,6-dinitrotoluene | 1 |  |
| cpd:C16391 |  | Trinitrotoluene; 2,4,6-Trinitrotoluene | 1 |  |
| cpd:C01888 |  | Aminoacetone; 1-Amino-2-propanone | 1 | ec:1.4.3.4 |
| cpd:C11904 |  | Dihydromethanophenazine; 2-(Dihydropentaprenyloxy)-dihydrophenazine | 1 | ec:1.8.98.1 |
| cpd:C11903 |  | Methanophenazine; 2-(2,3-Dihydro-all-trans-pentaprenyloxy)phenazine; 2-(2,3-Dihydropentaprenyloxy)phenazine | 1 | ec:1.8.98.1 |
| cpd:C01127 |  | 4-Hydroxy-2-oxoglutarate; 4-Hydroxy-2-oxoglutaric acid | 1 | ec:4.1.3.16 ec:4.1.2.14 |
| cpd:C00445 |  | 5,10-Methenyltetrahydrofolate | 1 | ec:2.1.2.2 |
| cpd:C06399 |  | Hydrogenobyrinate; Hydrogenobyrinic acid | 1 | ec:6.3.5.11 ec:6.3.5.9 |
| cpd:C00043 |  | UDP-N-acetyl-alpha-D-glucosamine; UDP-N-acetyl-D-glucosamine; UDP-N-acetylglucosamine | 1 | ec:2.5.1.7 |
| cpd:C02954 |  | 6-Aminopenicillanate; 6-Aminopenicillanic acid | 1 | ec:3.5.1.11 |
| cpd:C00040 |  | Acyl-CoA; Acyl coenzyme A | 1 | ec:2.3.1.15 |
| cpd:C05744 |  | Acetoacetyl-[acp]; Acetoacetyl-[acyl-carrier protein] | 1 | ec:2.3.1.180 |
| cpd:C00437 |  | N-Acetylornithine; N2-Acetyl-L-ornithine | 1 | ec:2.6.1.11 |
| cpd:C00039 |  | DNA; DNAn; DNAn+1; (Deoxyribonucleotide)n; (Deoxyribonucleotide)m; (Deoxyribonucleotide)n+m; Deoxyribonucleic acid | 1 | ec:2.7.7.7 |
| cpd:C00398 |  | Tryptamine; 3-(2-Aminoethyl)indole | 1 | ec:1.4.3.4 |
| cpd:C05345 |  | beta-D-Fructose 6-phosphate | 1 | ec:3.1.3.11 |
| cpd:C00036 |  | Oxaloacetate; Oxalacetic acid; Oxaloacetic acid; 2-Oxobutanedioic acid; 2-Oxosuccinic acid; keto-Oxaloacetate | 1 | ec:4.1.1.31 |
| cpd:C00395 |  | Penicillin; Penam | 1 | ec:3.5.1.11 |
| cpd:C00035 |  | GDP; Guanosine 5'-diphosphate; Guanosine diphosphate | 1 | ec:2.7.1.48 |
| cpd:C16400 |  | 2,4,6-Triaminotoluene | 1 | ec:1.8.99.1 ec:1.8.99.3 |
| cpd:C00031 |  | D-Glucose; Grape sugar; Dextrose; Glucose; D-Glucopyranose | 1 | ec:3.2.1.20 |
| cpd:C01861 |  | Trithionate; (O3S.S.SO3)2- | 1 | ec:1.8.99.1 ec:1.8.99.3 |
| cpd:C00788 |  | L-Adrenaline; (R)-(-)-Adrenaline; (R)-(-)-Epinephrine; (R)-(-)-Epirenamine; (R)-(-)-Adnephrine; 4-[(1R)-1-Hydroxy-2-(methylamino)ethyl]-1,2-benzenediol | 1 | ec:1.4.3.4 |
| cpd:C00780 |  | Serotonin; 3-(2-Aminoethyl)-1H-indol-5-ol; 5-Hydroxytryptamine; Enteramine | 1 | ec:1.4.3.4 |
| cpd:C00029 |  | UDP-glucose; UDPglucose; UDP-D-glucose; Uridine diphosphate glucose; UDP-alpha-D-glucose | 1 | ec:3.6.1.8 |
| cpd:C00027 |  | Hydrogen peroxide; H2O2; Oxydol | 1 | ec:1.4.3.4 |
| cpd:C00026 |  | 2-Oxoglutarate; Oxoglutaric acid; 2-Ketoglutaric acid; alpha-Ketoglutaric acid | 1 | ec:2.6.1.11 |
| cpd:C05332 |  | Phenethylamine; 2-Phenylethylamine; beta-Phenylethylamine; Phenylethylamine | 1 | ec:1.4.3.4 |
| cpd:C06010 |  | (S)-2-Acetolactate; (S)-2-Hydroxy-2-methyl-3-oxobutanoate | 1 | ec:2.2.1.6 |
| cpd:C00021 |  | S-Adenosyl-L-homocysteine; S-Adenosylhomocysteine | 1 | ec:2.1.1.171 |
| cpd:C03576 |  | 2-Mercaptoethanesulfonate; Reduced coenzyme M; HS-CoM; CoM | 1 | ec:1.8.98.1 |
| cpd:C06006 |  | (S)-2-Aceto-2-hydroxybutanoate; (S)-2-Hydroxy-2-ethyl-3-oxobutanoate | 1 | ec:2.2.1.6 |
| cpd:C00019 |  | S-Adenosyl-L-methionine; S-Adenosylmethionine; AdoMet; SAM | 1 | ec:2.1.1.171 |
| cpd:C00376 |  | Retinal; Vitamin A aldehyde; Retinene; all-trans-Retinal; all-trans-Vitamin A aldehyde; all-trans-Retinene | 1 | ec:1.1.1.1 |
| cpd:C06002 |  | (S)-Methylmalonate semialdehyde | 1 | ec:1.1.1.31 |
| cpd:C00015 |  | UDP; Uridine 5'-diphosphate | 1 | ec:2.7.1.48 |
| cpd:C06001 |  | (S)-3-Hydroxyisobutyrate | 1 | ec:1.1.1.31 |
| cpd:C16348 |  | cis-3-Chloroallyl aldehyde; cis-3-Chloro-2-propenal | 1 | ec:1.1.1.1 |
| cpd:C04640 |  | 2-(Formamido)-N1-(5'-phosphoribosyl)acetamidine; 1-(5'-Phosphoribosyl)-N-formylglycinamidine; 5'-Phosphoribosyl-N-formylglycinamidine; 5'-Phosphoribosylformylglycinamidine; 2-(Formamido)-N1-(5-phospho-D-ribosyl)acetamidine | 1 | ec:6.3.3.1 |
| cpd:C03561 |  | (R)-3-Hydroxybutanoyl-CoA; (3R)-3-Hydroxybutanoyl-CoA | 1 |  |
| cpd:C00369 |  | Starch | 1 | ec:2.4.1.1 |
| cpd:C00363 |  | dTDP; Deoxythymidine 5'-diphosphate | 1 | ec:2.7.1.48 |
| cpd:C04631 |  | UDP-N-acetyl-3-(1-carboxyvinyl)-D-glucosamine; UDP-N-acetyl-3-O-(1-carboxyvinyl)-D-glucosamine; UDP-N-acetylglucosamine-3-O-pyruvateether; UDP-N-acetylglucosamine enolpyruvate; UDP-N-acetyl-3-O-(1-carboxyvinyl)-alpha-D-glucosamine | 1 | ec:2.5.1.7 |
| cpd:C00361 |  | dGDP; 2'-Deoxyguanosine 5'-diphosphate | 1 | ec:2.7.1.48 |
| cpd:C02514 |  | 3-Fumarylpyruvate | 1 | ec:3.7.1.20 |
| cpd:C04628 |  | Coenzyme B; N-(7-Mercaptoheptanoyl)threonine 3-O-phosphate; N-(7-Mercaptoheptanoyl)threonine O3-phosphate; HTP | 1 | ec:1.8.98.1 |
| cpd:C02909 |  | (2-Naphthyl)methanol; 2-Naphthalenemethanol; 2-Hydroxymethylnaphthalene | 1 | ec:1.1.1.1 |
| cpd:C00354 |  | D-Fructose 1,6-bisphosphate | 1 | ec:3.1.3.11 |
| cpd:C03546 |  | myo-Inositol 4-phosphate; D-myo-Inositol 4-phosphate; 1D-myo-Inositol 4-phosphate; 1D-myo-Inositol 4-monophosphate; Inositol 4-phosphate | 1 | ec:3.1.3.25 |
| cpd:C03939 |  | Acetyl-[acyl-carrier protein] | 1 | ec:2.3.1.180 |

  
**Over-represented Pathway Summary**: Collection of the KEGG metabolic pathways containing the proteins identified in the "Over-represented Metabolite Summary" ranked by the highest number of hits per pathway  

| Pathway ID | EC | EC Frequency | Name |
| --- | --- | --- | --- |
| map00564 | ec:1.1.5.3 ec:3.1.4.46 ec:2.3.1.15 | 104 | path:map00564 Glycerophospholipid metabolism |
| map00561 | ec:2.7.1.30 ec:2.3.1.15 | 90 | path:map00561 Glycerolipid metabolism |
| map00362 | ec:5.5.1.2 ec:3.1.1.24 ec:1.14.13.2 ec:2.3.1.9 ec:4.1.1.44 ec:1.1.1.157 ec:2.3.1.174 ec:1.13.11.3 | 43 | path:map00362 Benzoate degradation |
| map00480 | ec:1.1.1.49 ec:1.11.1.15 ec:2.3.2.2 | 30 | path:map00480 Glutathione metabolism |
| map00330 | ec:1.5.1.2 ec:4.1.3.16 ec:2.3.1.57 ec:2.1.3.3 ec:1.4.3.4 ec:2.6.1.11 | 26 | path:map00330 Arginine and proline metabolism |
| map00400 | ec:1.3.1.12 ec:2.5.1.19 ec:2.5.1.54 ec:4.2.1.20 ec:5.4.99.5 | 25 | path:map00400 Phenylalanine, tyrosine and tryptophan biosynthesis |
| map00460 | ec:2.3.2.2 | 23 | path:map00460 Cyanoamino acid metabolism |
| map00052 | ec:3.2.1.20 ec:4.2.1.6 | 23 | path:map00052 Galactose metabolism |
| map00590 | ec:2.3.2.2 | 23 | path:map00590 Arachidonic acid metabolism |
| map00430 | ec:2.3.2.2 | 23 | path:map00430 Taurine and hypotaurine metabolism |
| map00650 | ec:2.8.3.12 ec:2.2.1.6 ec:2.3.1.9 ec:1.1.1.157 | 19 | path:map00650 Butanoate metabolism |
| map00627 | ec:1.14.13.82 ec:1.2.1.28 | 15 | path:map00627 Aminobenzoate degradation |
| map00071 | ec:1.1.1.1 ec:2.3.1.9 ec:6.2.1.3 ec:1.18.1.3 | 14 | path:map00071 Fatty acid degradation |
| map00622 | ec:1.2.1.28 ec:1.18.1.3 | 13 | path:map00622 Xylene degradation |
| map00230 | ec:1.7.1.7 ec:3.6.1.8 ec:2.1.2.2 ec:2.4.2.22 ec:2.7.6.5 ec:2.7.7.7 ec:6.3.3.1 ec:2.7.7.6 ec:2.4.2.7 | 12 | path:map00230 Purine metabolism |
| map00740 | ec:2.5.1.9 ec:4.1.99.12 | 11 | path:map00740 Riboflavin metabolism |
| map00643 | ec:2.8.3.12 | 10 | path:map00643 Styrene degradation |
| map00624 | ec:1.13.11.3 | 10 | path:map00624 Polycyclic aromatic hydrocarbon degradation |
| map00360 | ec:1.4.99.1 ec:1.4.3.4 ec:1.1.1.157 | 9 | path:map00360 Phenylalanine metabolism |
| map00630 | ec:3.5.1.10 ec:4.1.3.16 ec:2.3.1.9 | 9 | path:map00630 Glyoxylate and dicarboxylate metabolism |
| map00240 | ec:3.5.4.5 ec:3.6.1.8 ec:2.7.1.48 ec:2.7.7.7 ec:2.4.2.9 ec:2.7.7.6 ec:3.5.4.13 | 8 | path:map00240 Pyrimidine metabolism |
| map00010 | ec:1.1.1.1 ec:5.3.1.1 ec:4.2.1.11 ec:5.1.3.3 ec:1.2.1.12 ec:5.4.2.12 ec:3.1.3.11 | 7 | path:map00010 Glycolysis / Gluconeogenesis |
| map00250 | ec:6.3.5.4 | 7 | path:map00250 Alanine, aspartate and glutamate metabolism |
| map00260 | ec:1.1.1.1 ec:4.2.1.20 ec:3.1.3.3 ec:1.4.3.4 ec:5.4.2.12 | 6 | path:map00260 Glycine, serine and threonine metabolism |
| map00720 | ec:4.2.1.2 ec:2.3.1.9 ec:4.1.1.31 | 6 | path:map00720 Carbon fixation pathways in prokaryotes |
| map00680 | ec:4.2.1.11 ec:1.8.98.1 ec:4.1.1.31 ec:3.1.3.3 ec:5.4.2.12 ec:3.1.3.11 | 6 | path:map00680 Methane metabolism |
| map00300 | ec:2.3.1.117 | 5 | path:map00300 Lysine biosynthesis |
| map00623 | ec:1.2.1.28 | 5 | path:map00623 Toluene degradation |
| map00620 | ec:2.3.1.9 ec:4.1.1.31 | 5 | path:map00620 Pyruvate metabolism |
| map00030 | ec:4.2.1.12 ec:1.1.1.49 ec:3.1.1.31 ec:3.1.3.11 ec:4.1.2.14 | 5 | path:map00030 Pentose phosphate pathway |
| map00380 | ec:2.3.1.9 ec:1.4.3.4 | 5 | path:map00380 Tryptophan metabolism |
| map00280 | ec:2.3.1.9 ec:1.1.1.31 | 5 | path:map00280 Valine, leucine and isoleucine degradation |
| map00670 | ec:3.5.1.10 ec:2.1.2.2 | 5 | path:map00670 One carbon pool by folate |
| map00860 | ec:6.3.5.11 ec:6.3.5.10 ec:6.3.5.9 ec:2.5.1.17 ec:6.1.1.17 | 5 | path:map00860 Porphyrin and chlorophyll metabolism |
| map00072 | ec:2.3.1.9 | 4 | path:map00072 Synthesis and degradation of ketone bodies |
| map00450 | ec:2.1.1.14 | 4 | path:map00450 Selenocompound metabolism |
| map00640 | ec:2.3.1.9 | 4 | path:map00640 Propanoate metabolism |
| map00920 | ec:2.8.1.1 ec:1.8.99.1 ec:1.8.99.3 | 4 | path:map00920 Sulfur metabolism |
| map00710 | ec:5.3.1.1 ec:1.2.1.12 ec:4.1.1.31 ec:3.1.3.11 | 4 | path:map00710 Carbon fixation in photosynthetic organisms |
| map00900 | ec:2.3.1.9 | 4 | path:map00900 Terpenoid backbone biosynthesis |
| map00310 | ec:2.3.1.9 | 4 | path:map00310 Lysine degradation |
| map00270 | ec:2.1.1.14 | 4 | path:map00270 Cysteine and methionine metabolism |
| map00350 | ec:3.7.1.20 ec:1.1.1.1 ec:1.4.3.4 | 3 | path:map00350 Tyrosine metabolism |
| map00500 | ec:3.2.1.20 ec:2.4.1.1 | 2 | path:map00500 Starch and sucrose metabolism |
| map00562 | ec:5.3.1.1 ec:3.1.3.25 | 2 | path:map00562 Inositol phosphate metabolism |
| map00401 | ec:1.3.1.12 | 2 | path:map00401 Novobiocin biosynthesis |
| map00051 | ec:5.3.1.1 ec:3.1.3.11 | 2 | path:map00051 Fructose and mannose metabolism |
| map00790 | ec:4.2.3.12 ec:4.1.2.50 | 2 | path:map00790 Folate biosynthesis |
| map00983 | ec:3.5.4.5 ec:2.7.1.48 | 2 | path:map00983 Drug metabolism - other enzymes |
| map00982 | ec:1.1.1.1 ec:1.4.3.4 | 2 | path:map00982 Drug metabolism - cytochrome P450 |
| map00190 | ec:1.9.3.1 | 2 | path:map00190 Oxidative phosphorylation |
| map00970 | ec:6.1.1.22 ec:6.1.1.17 | 2 | path:map00970 Aminoacyl-tRNA biosynthesis |
| map00660 | ec:2.2.1.6 | 1 | path:map00660 C5-Branched dibasic acid metabolism |
| map00950 | ec:1.4.3.4 | 1 | path:map00950 Isoquinoline alkaloid biosynthesis |
| map04070 | ec:3.1.3.25 | 1 | path:map04070 Phosphatidylinositol signaling system |
| map00061 | ec:2.3.1.180 | 1 | path:map00061 Fatty acid biosynthesis |
| map00550 | ec:2.5.1.7 | 1 | path:map00550 Peptidoglycan biosynthesis |
| map00340 | ec:1.4.3.4 | 1 | path:map00340 Histidine metabolism |
| map00830 | ec:1.1.1.1 | 1 | path:map00830 Retinol metabolism |
| map00633 | ec:1.8.99.3 | 1 | path:map00633 Nitrotoluene degradation |
| map00980 | ec:1.1.1.1 | 1 | path:map00980 Metabolism of xenobiotics by cytochrome P450 |
| map00626 | ec:1.1.1.1 | 1 | path:map00626 Naphthalene degradation |
| map00625 | ec:1.1.1.1 | 1 | path:map00625 Chloroalkane and chloroalkene degradation |
| map00290 | ec:2.2.1.6 | 1 | path:map00290 Valine, leucine and isoleucine biosynthesis |
| map00780 | ec:3.1.1.85 | 1 | path:map00780 Biotin metabolism |
| map00130 | ec:4.1.3.40 | 1 | path:map00130 Ubiquinone and other terpenoid-quinone biosynthesis |
| map00521 | ec:3.1.3.25 | 1 | path:map00521 Streptomycin biosynthesis |
| map00520 | ec:2.5.1.7 | 1 | path:map00520 Amino sugar and nucleotide sugar metabolism |
| map00770 | ec:2.2.1.6 | 1 | path:map00770 Pantothenate and CoA biosynthesis |
| map00020 | ec:4.2.1.2 | 1 | path:map00020 Citrate cycle (TCA cycle) |
| map00311 | ec:3.5.1.11 | 1 | path:map00311 Penicillin and cephalosporin biosynthesis |

  
Analysis performed on 2014/02/14 22:49:07
