# Supplementary material for: Automated genomic context analysis and experimental validation platform for discovery of prokaryote transcriptional regulator functions
Source: BMC Genomics. 2014 Dec 18;15(1):1142. doi: 10.1186/1471-2164-15-1142 (PMC4349456; doi:10.1186/1471-2164-15-1142)
Supplement: Supplementary file 7 — Additional file 7: Result HpaR. Function Discovery V1.0 output (.html format) for the hydroxyphenylacetate catabolism regulator (HpaR, Bxe_ B2027). For detailed instructions on how to analyze the results please refer to the Function Discovery V1.0, a gene neighborhood analysis tool section in the Results part of the main text. (HTML 380 KB) [file 12864_2014_6995_MOESM7_ESM.html]

```
ENTRY       Bxe_B2027         CDS       T00340
DEFINITION  MarR family transcriptional regulator
ORGANISM    bxe  Burkholderia xenovorans
POSITION    2:1136177..1136629
MOTIF       Pfam: HTH_27 MarR MarR_2 TrmB HTH_24 Fe_dep_repress HTH_20 HxlR
DBLINKS     NCBI-GI: 91778100
            NCBI-GeneID: 4007477
            JGI: BxeB2027
            UniProt: Q13PN1
AASEQ       150
            MSASASTRVLHRNLPMLLLRAREKMMERFRPLITAHGLTEQQWRVIRALNEHGPMEPRHI
            SDICTISSPSMAGVLARMESMELVTKERFAEDQRRVLVSLTDTSLELVRVISKDLEAHYR
            ELERKVGPEIVERVYRAVDDLLAGLEEEEE
NTSEQ       453
            atgtctgcttccgcttcgacccgagttttgcaccgcaacctgccgatgttgttgctgcgc
            gccagagaaaaaatgatggagcggtttcgtccgttgattaccgcgcatggattgaccgag
            cagcagtggcgcgtgattcgcgcgctgaacgaacacggcccgatggagccgcgccatatc
            tccgatatctgcacgatttcgagcccgagcatggccggcgtgctcgcgcgcatggagagc
            atggaactggtgaccaaggagcggttcgccgaagatcagcggcgcgtgctggtgtcgctc
            acggacacgagcctggaattggtgcgcgtgatttccaaagacctcgaggcgcattaccgc
            gagttggagcgcaaggtggggccggagatcgtcgagcgcgtgtatcgcgcggtggacgat
            ctgctggccggtctggaggaagaagaggagtga
///
```

  
**Homolog ID**: Table of closest homologs  

```
                 Homologs                                       len   identity overlap
---------------------------------------------------------------------------------
bpy:Bphyt_5823 MarR family transcriptional regulator          150     0.993    150 
bgf:BC1003_4084 homoprotocatechuate degradation operon        150     0.947    150 
bge:BC1002_3577 MarR family transcriptional regulator         149     0.973    149 
bph:Bphy_4194 MarR family transcriptional regulator           158     0.615    148 
rfr:Rfer_1215 MarR family transcriptional regulator           174     0.451    142 
reh:H16_A1367 MarR family transcriptional regulator           204     0.483    145 
rsc:RCFBP_11599 homoprotocatechuate degradative operon        154     0.478    136 
mag:amb0811 transcriptional regulator                         163     0.471    138 
axy:AXYL_04170 homoprotocatechuate degradation operon r       154     0.457    138 
brh:RBRH_01410 MarR family transcriptional regulator          168     0.496    137 
cti:RALTA_A1286 marr family transcription regulator           207     0.441    145 
rsl:RPSI07_1682 homoprotocatechuate degradative operon        154     0.452    135 
rso:RSc1592 transcription regulator protein                   154     0.444    135 
rpi:Rpic_1953 MarR family transcriptional regulator           154     0.441    136 
rpf:Rpic12D_1625 MarR family transcriptional regulator        154     0.434    136 
bch:Bcen2424_4230 MarR family transcriptional regulator       155     0.500    134 
bcm:Bcenmc03_3287 MarR family transcriptional regulator       146     0.500    134 
bcn:Bcen_4136 MarR family transcriptional regulator           155     0.500    134 
vap:Vapar_0246 MarR family transcriptional regulator          144     0.455    145 
bcj:BCAM1365 putative homoprotocatechuate degradative o       146     0.493    134 
bur:Bcep18194_B1784 MarR family transcriptional regulat       155     0.493    134 
bac:BamMC406_4126 MarR family transcriptional regulator       146     0.496    135 
bam:Bamb_3652 MarR family transcriptional regulator           155     0.496    135 
bgl:bglu_2g17970 MarR family transcriptional regulator        146     0.470    134 
bte:BTH_II1736 homoprotocatechuate degradation operon r       145     0.478    134 
bmj:BMULJ_04131 MarR family transcriptional regulator         148     0.493    134 
bmu:Bmul_4375 MarR family transcriptional regulator           148     0.493    134 
bpl:BURPS1106A_A0937 homoprotocatechuate degradation op       145     0.478    134 
bpm:BURPS1710b_A2262 homoprotocatechuate degradation op       145     0.478    134 
bps:BPSS0691 MarR family transcriptional regulator            145     0.478    134 
bma:BMAA1141 homoprotocatechuate degradative operon rep       145     0.478    134 
bml:BMA10229_0099 homoprotocatechuate degradative opero       145     0.478    134 
bmn:BMA10247_A1514 homoprotocatechuate degradative oper       145     0.478    134 
bpd:BURPS668_A1022 homoprotocatechuate degradation oper       145     0.478    134 
bvi:Bcep1808_4800 MarR family transcriptional regulator       146     0.481    135 
ppg:PputGB1_3494 MarR family transcriptional regulator        140     0.426    129 
pen:PSEEN3090 homoprotocatechuate degradative operon re       140     0.411    129 
mrb:Mrub_1336 MarR family transcriptional regulator           161     0.409    149 
dac:Daci_0098 MarR family transcriptional regulator           166     0.414    145 
pfs:PFLU3277 putative homoprotocatechuate degradative o       140     0.400    135 
pae:PA4135 transcriptional regulator                          140     0.403    129 
pag:PLES_08361 putative transcriptional regulator             140     0.403    129 
pau:PA14_10480 transcriptional regulator                      140     0.403    129 
rpt:Rpal_4278 MarR family transcriptional regulator           179     0.417    120 
rpa:RPA3757 MarR family transcriptional regulator             179     0.417    120
```

**Neighborhood Representations**: Table of genes in the defined genetic neighborhoods of the entry protein and its closest homologs  
  
**Neighborhood Representations for "bxe:Bxe\_B2027"**  

| ID | Annotation | EC number |
| --- | --- | --- |
| bxe:Bxe\_B2037 | hypothetical protein |  |
| bxe:Bxe\_B2036 | diguanylate cyclase |  |
| bxe:Bxe\_B2035 | diguanylate cyclase/phosphodiesterase |  |
| bxe:Bxe\_B2034 | 2,4-dihydroxyhept-2-ene-1,7-dioic acid aldolase (EC:4.1.2.-); K02510 4-hydroxy-2-oxoheptanedioate aldolase [EC:4.1.2.52] | ec:4.1.2.52 |
| bxe:Bxe\_B2033 | putative 2-oxo-hept-3-ene-1,7-dioate hydratase HpaH (EC:4.2.1.-); K02509 2-oxo-hept-3-ene-1,7-dioate hydratase [EC:4.2.1.-] |  |
| bxe:Bxe\_B2032 | 5-carboxymethyl-2-hydroxymuconate delta-isomerase (EC:5.3.3.10); K01826 5-carboxymethyl-2-hydroxymuconate isomerase [EC:5.3.3.10] | ec:5.3.3.10 |
| bxe:Bxe\_B2031 | 3,4-dihydroxyphenylacetate 2,3-dioxygenase (EC:1.13.11.15); K00455 3,4-dihydroxyphenylacetate 2,3-dioxygenase [EC:1.13.11.15] | ec:1.13.11.15 |
| bxe:Bxe\_B2030 | 5-carboxy-2-hydroxymuconate semialdehyde dehydrogenase HpaE (EC:1.2.1.60); K00151 5-carboxymethyl-2-hydroxymuconic-semialdehyde dehydrogenase [EC:1.2.1.60] | ec:1.2.1.60 |
| bxe:Bxe\_B2029 | putative 5-oxo-1,2,5 tricarboxilic-3-penta acido decarboxylase HpaG2 (EC:4.1.1.68); K05921 5-oxopent-3-ene-1,2,5-tricarboxylate decarboxylase / 2-hydroxyhepta-2,4-diene-1,7-dioate isomerase [EC:4.1.1.68 5.3.3.-] | ec:4.1.1.68 |
| bxe:Bxe\_B2028 | putative 5-oxo-1,2,5-tricarboxilic-3-penten acid decarboxylase/isomerase (HpaG) (EC:4.1.1.68); K05921 5-oxopent-3-ene-1,2,5-tricarboxylate decarboxylase / 2-hydroxyhepta-2,4-diene-1,7-dioate isomerase [EC:4.1.1.68 5.3.3.-] | ec:4.1.1.68 |
| bxe:Bxe\_B2027 | MarR family transcriptional regulator |  |
| bxe:Bxe\_B2026 | metal-activated pyridoxal protein |  |
| bxe:Bxe\_B2025 | HAD family hydrolase |  |
| bxe:Bxe\_B2024 | LysR family transcriptional regulator |  |
| bxe:Bxe\_B2023 | acylating methylmalonate-semialdehyde dehydrogenase (EC:1.2.1.27); K00140 malonate-semialdehyde dehydrogenase (acetylating) / methylmalonate-semialdehyde dehydrogenase [EC:1.2.1.18 1.2.1.27] | ec:1.2.1.18 ec:1.2.1.27 |
| bxe:Bxe\_B2022 | major facilitator superfamily multidrug efflux transporter |  |
| bxe:Bxe\_B2021 | isochorismatase hydrolase |  |
| bxe:Bxe\_B2020 | MarR family transcriptional regulator |  |
| bxe:Bxe\_B2019 | putative acetyltransferase |  |
| bxe:Bxe\_B2018 | putative N-ethylmaleimide reductase |  |
| bxe:Bxe\_B2017 | ArsR family transcriptional regulator; K03892 ArsR family transcriptional regulator |  |

  
**Neighborhood Representations for "bpy:Bphyt\_5823"**  

| ID | Annotation | EC number |
| --- | --- | --- |
| bpy:Bphyt\_5813 | major facilitator superfamily protein |  |
| bpy:Bphyt\_5814 | methylmalonate-semialdehyde dehydrogenase; K00140 malonate-semialdehyde dehydrogenase (acetylating) / methylmalonate-semialdehyde dehydrogenase [EC:1.2.1.18 1.2.1.27] | ec:1.2.1.18 ec:1.2.1.27 |
| bpy:Bphyt\_5815 | LysR family transcriptional regulator; K16135 LysR family transcriptional regulator, transcriptional activator for dmlA |  |
| bpy:Bphyt\_5816 | major facilitator superfamily protein |  |
| bpy:Bphyt\_5817 | oxidoreductase domain-containing protein |  |
| bpy:Bphyt\_5818 | 3-dehydroquinate dehydratase (EC:4.2.1.10); K03786 3-dehydroquinate dehydratase II [EC:4.2.1.10] | ec:4.2.1.10 |
| bpy:Bphyt\_5819 | hypothetical protein |  |
| bpy:Bphyt\_5820 | LysR family transcriptional regulator |  |
| bpy:Bphyt\_5821 | HAD-superfamily hydrolase |  |
| bpy:Bphyt\_5822 | alanine racemase domain-containing protein |  |
| bpy:Bphyt\_5823 | MarR family transcriptional regulator |  |
| bpy:Bphyt\_5824 | 5-oxopent-3-ene-1,2,5-tricarboxylate decarboxylase (EC:4.1.1.68); K05921 5-oxopent-3-ene-1,2,5-tricarboxylate decarboxylase / 2-hydroxyhepta-2,4-diene-1,7-dioate isomerase [EC:4.1.1.68 5.3.3.-] | ec:4.1.1.68 |
| bpy:Bphyt\_5825 | 4-hydroxyphenylacetate degradation bifunctional isomerase/decarboxylase subunit HpaG2 (EC:4.1.1.68); K05921 5-oxopent-3-ene-1,2,5-tricarboxylate decarboxylase / 2-hydroxyhepta-2,4-diene-1,7-dioate isomerase [EC:4.1.1.68 5.3.3.-] | ec:4.1.1.68 |
| bpy:Bphyt\_5826 | 5-carboxymethyl-2-hydroxymuconate semialdehyde dehydrogenase; K00151 5-carboxymethyl-2-hydroxymuconic-semialdehyde dehydrogenase [EC:1.2.1.60] | ec:1.2.1.60 |
| bpy:Bphyt\_5827 | 3,4-dihydroxyphenylacetate 2,3-dioxygenase (EC:1.13.11.15); K00455 3,4-dihydroxyphenylacetate 2,3-dioxygenase [EC:1.13.11.15] | ec:1.13.11.15 |
| bpy:Bphyt\_5828 | 5-carboxymethyl-2-hydroxymuconate isomerase; K01826 5-carboxymethyl-2-hydroxymuconate isomerase [EC:5.3.3.10] | ec:5.3.3.10 |
| bpy:Bphyt\_5829 | 2-oxo-hepta-3-ene-1,7-dioic acid hydratase (EC:4.1.1.77); K02509 2-oxo-hept-3-ene-1,7-dioate hydratase [EC:4.2.1.-] |  |
| bpy:Bphyt\_5830 | 2,4-dihydroxyhept-2-ene-1,7-dioic acid aldolase (EC:4.1.2.20); K02510 4-hydroxy-2-oxoheptanedioate aldolase [EC:4.1.2.52] | ec:4.1.2.52 |
| bpy:Bphyt\_5831 | diguanylate cyclase/phosphodiesterase |  |
| bpy:Bphyt\_5832 | diguanylate cyclase |  |
| bpy:Bphyt\_5833 | hypothetical protein |  |

  
**Neighborhood Representations for "bgf:BC1003\_4084"**  

| ID | Annotation | EC number |
| --- | --- | --- |
| bgf:BC1003\_4074 | hypothetical protein |  |
| bgf:BC1003\_4075 | TonB-dependent receptor plug; K02014 iron complex outermembrane recepter protein |  |
| bgf:BC1003\_4076 | putative transmembrane protein |  |
| bgf:BC1003\_4077 | diguanylate cyclase/phosphodiesterase |  |
| bgf:BC1003\_4078 | regulatory protein MarR |  |
| bgf:BC1003\_4079 | isochorismatase hydrolase |  |
| bgf:BC1003\_4080 | major facilitator superfamily protein |  |
| bgf:BC1003\_4081 | methylmalonate-semialdehyde dehydrogenase; K00140 malonate-semialdehyde dehydrogenase (acetylating) / methylmalonate-semialdehyde dehydrogenase [EC:1.2.1.18 1.2.1.27] | ec:1.2.1.18 ec:1.2.1.27 |
| bgf:BC1003\_4082 | major facilitator superfamily protein |  |
| bgf:BC1003\_4083 | hypothetical protein |  |
| bgf:BC1003\_4084 | homoprotocatechuate degradation operon regulator, HpaR |  |
| bgf:BC1003\_4085 | fumarylacetoacetate (FAA) hydrolase; K05921 5-oxopent-3-ene-1,2,5-tricarboxylate decarboxylase / 2-hydroxyhepta-2,4-diene-1,7-dioate isomerase [EC:4.1.1.68 5.3.3.-] | ec:4.1.1.68 |
| bgf:BC1003\_4086 | 4-hydroxyphenylacetate degradation bifunctional isomerase/decarboxylase,HpaG2 subunit; K05921 5-oxopent-3-ene-1,2,5-tricarboxylate decarboxylase / 2-hydroxyhepta-2,4-diene-1,7-dioate isomerase [EC:4.1.1.68 5.3.3.-] | ec:4.1.1.68 |
| bgf:BC1003\_4087 | 5-carboxymethyl-2-hydroxymuconate semialdehyde dehydrogenase; K00151 5-carboxymethyl-2-hydroxymuconic-semialdehyde dehydrogenase [EC:1.2.1.60] | ec:1.2.1.60 |
| bgf:BC1003\_4088 | 3,4-dihydroxyphenylacetate 2,3-dioxygenase (EC:1.13.11.15); K00455 3,4-dihydroxyphenylacetate 2,3-dioxygenase [EC:1.13.11.15] | ec:1.13.11.15 |
| bgf:BC1003\_4089 | 5-carboxymethyl-2-hydroxymuconate isomerase; K01826 5-carboxymethyl-2-hydroxymuconate isomerase [EC:5.3.3.10] | ec:5.3.3.10 |
| bgf:BC1003\_4090 | 2-oxo-hepta-3-ene-1,7-dioic acid hydratase (EC:4.2.1.80); K02509 2-oxo-hept-3-ene-1,7-dioate hydratase [EC:4.2.1.-] |  |
| bgf:BC1003\_4091 | 2,4-dihydroxyhept-2-ene-1,7-dioic acid aldolase (EC:4.1.3.39); K02510 4-hydroxy-2-oxoheptanedioate aldolase [EC:4.1.2.52] | ec:4.1.2.52 |
| bgf:BC1003\_4092 | diguanylate cyclase/phosphodiesterase |  |
| bgf:BC1003\_4093 | hypothetical protein |  |
| bgf:BC1003\_4094 | hypothetical protein |  |

  
**Neighborhood Representations for "bge:BC1002\_3577"**  

| ID | Annotation | EC number |
| --- | --- | --- |
| bge:BC1002\_3567 | hypothetical protein |  |
| bge:BC1002\_3568 | diguanylate cyclase |  |
| bge:BC1002\_3569 | diguanylate cyclase/phosphodiesterase |  |
| bge:BC1002\_3570 | 2,4-dihydroxyhept-2-ene-1,7-dioic acid aldolase (EC:4.1.3.39); K02510 4-hydroxy-2-oxoheptanedioate aldolase [EC:4.1.2.52] | ec:4.1.2.52 |
| bge:BC1002\_3571 | 2-oxo-hepta-3-ene-1,7-dioic acid hydratase (EC:4.2.1.80); K02509 2-oxo-hept-3-ene-1,7-dioate hydratase [EC:4.2.1.-] |  |
| bge:BC1002\_3572 | 5-carboxymethyl-2-hydroxymuconate isomerase; K01826 5-carboxymethyl-2-hydroxymuconate isomerase [EC:5.3.3.10] | ec:5.3.3.10 |
| bge:BC1002\_3573 | 3,4-dihydroxyphenylacetate 2,3-dioxygenase (EC:1.13.11.15); K00455 3,4-dihydroxyphenylacetate 2,3-dioxygenase [EC:1.13.11.15] | ec:1.13.11.15 |
| bge:BC1002\_3574 | 5-carboxymethyl-2-hydroxymuconate semialdehyde dehydrogenase (EC:1.2.1.8); K00151 5-carboxymethyl-2-hydroxymuconic-semialdehyde dehydrogenase [EC:1.2.1.60] | ec:1.2.1.60 |
| bge:BC1002\_3575 | 4-hydroxyphenylacetate degradation bifunctional isomerase/decarboxylase subunit HpaG2 (EC:5.3.3.10); K05921 5-oxopent-3-ene-1,2,5-tricarboxylate decarboxylase / 2-hydroxyhepta-2,4-diene-1,7-dioate isomerase [EC:4.1.1.68 5.3.3.-] | ec:4.1.1.68 |
| bge:BC1002\_3576 | 5-carboxymethyl-2-hydroxymuconate delta-isomerase (EC:5.3.3.10); K05921 5-oxopent-3-ene-1,2,5-tricarboxylate decarboxylase / 2-hydroxyhepta-2,4-diene-1,7-dioate isomerase [EC:4.1.1.68 5.3.3.-] | ec:4.1.1.68 |
| bge:BC1002\_3577 | MarR family transcriptional regulator |  |
| bge:BC1002\_3578 | alanine racemase |  |
| bge:BC1002\_3579 | HAD-superfamily hydrolase |  |
| bge:BC1002\_3580 | LysR family transcriptional regulator |  |
| bge:BC1002\_3581 | hypothetical protein |  |
| bge:BC1002\_3582 | 3-dehydroquinate dehydratase (EC:4.2.1.10); K03786 3-dehydroquinate dehydratase II [EC:4.2.1.10] | ec:4.2.1.10 |
| bge:BC1002\_3583 | oxidoreductase domain-containing protein |  |
| bge:BC1002\_3584 | major facilitator superfamily protein |  |
| bge:BC1002\_3585 | LysR family transcriptional regulator; K16135 LysR family transcriptional regulator, transcriptional activator for dmlA |  |
| bge:BC1002\_3586 | major facilitator superfamily protein |  |
| bge:BC1002\_3587 | isochorismatase hydrolase |  |

  
**Neighborhood Representations for "bph:Bphy\_4194"**  

| ID | Annotation | EC number |
| --- | --- | --- |
| bph:Bphy\_4184 | hypothetical protein |  |
| bph:Bphy\_4185 | cytochrome B561; K12262 cytochrome b561 |  |
| bph:Bphy\_4186 | zinc-binding CMP/dCMP deaminase |  |
| bph:Bphy\_4187 | basic membrane lipoprotein; K02058 simple sugar transport system substrate-binding protein |  |
| bph:Bphy\_4188 | hypothetical protein |  |
| bph:Bphy\_4189 | selenium-binding protein; K17285 selenium-binding protein 1 |  |
| bph:Bphy\_4190 | LuxR family transcriptional regulator |  |
| bph:Bphy\_4191 | 4-hydroxyphenylacetate transporter; K02511 MFS transporter, ACS family, 4-hydroxyphenylacetate permease |  |
| bph:Bphy\_4192 | FAD-dependent oxidoreductase; K05712 3-(3-hydroxy-phenyl)propionate hydroxylase [EC:1.14.13.127] | ec:1.14.13.127 |
| bph:Bphy\_4193 | hypothetical protein |  |
| bph:Bphy\_4194 | MarR family transcriptional regulator |  |
| bph:Bphy\_4195 | AraC family transcriptional regulator |  |
| bph:Bphy\_4196 | metal dependent phosphohydrolase |  |
| bph:Bphy\_4197 | LysR family transcriptional regulator |  |
| bph:Bphy\_4198 | extracellular solute-binding protein |  |
| bph:Bphy\_4199 | succinylglutamate desuccinylase/aspartoacylase |  |
| bph:Bphy\_4200 | short-chain dehydrogenase/reductase SDR; K07124 |  |
| bph:Bphy\_4201 | AraC family transcriptional regulator |  |
| bph:Bphy\_4202 | hypothetical protein |  |
| bph:Bphy\_4203 | putative lipoprotein |  |
| bph:Bphy\_4204 | OmpA/MotB domain-containing protein; K02557 chemotaxis protein MotB |  |

  
**Neighborhood Representations for "rfr:Rfer\_1215"**  

| ID | Annotation | EC number |
| --- | --- | --- |
| rfr:Rfer\_1205 | hypothetical protein |  |
| rfr:Rfer\_1206 | major facilitator transporter |  |
| rfr:Rfer\_1207 | aspartyl protease-like protein; K06985 aspartyl protease family protein |  |
| rfr:Rfer\_1208 | HNH nuclease |  |
| rfr:Rfer\_1209 | histidine triad (HIT) protein |  |
| rfr:Rfer\_1210 | hypothetical protein |  |
| rfr:Rfer\_1211 | hypothetical protein |  |
| rfr:Rfer\_1212 | DNA/RNA non-specific endonuclease; K01173 endonuclease G, mitochondrial |  |
| rfr:Rfer\_1213 | pseudogene |  |
| rfr:Rfer\_1214 | hypothetical protein |  |
| rfr:Rfer\_1215 | MarR family transcriptional regulator |  |
| rfr:Rfer\_1216 | Acetyl-CoA hydrolase (EC:3.1.2.1); K01067 acetyl-CoA hydrolase [EC:3.1.2.1] | ec:3.1.2.1 |
| rfr:Rfer\_1217 | hypothetical protein |  |
| rfr:Rfer\_1218 | natural resistance-associated macrophage protein |  |
| rfr:Rfer\_1219 | hypothetical protein |  |
| rfr:Rfer\_1220 | Bcr/CflA subfamily drug resistance transporter; K07552 MFS transporter, DHA1 family, bicyclomycin/chloramphenicol resistance protein |  |
| rfr:Rfer\_1221 | LysR family transcriptional regulator |  |
| rfr:Rfer\_1222 | hypothetical protein |  |
| rfr:Rfer\_1223 | ABC-type transport system periplasmic component; K02067 putative ABC transport system substrate-binding protein |  |
| rfr:Rfer\_1224 | ABC-type transport system permease; K02066 putative ABC transport system permease protein |  |
| rfr:Rfer\_1225 | hypothetical protein; K14160 protein ImuA |  |

  
**Neighborhood Representations for "reh:H16\_A1367"**  

| ID | Annotation | EC number |
| --- | --- | --- |
| reh:H16\_A1357 | h16\_A1357; hypothetical protein |  |
| reh:H16\_A1358 | h16\_A1358; Acetyl-CoA hydrolase (EC:3.1.2.1); K01067 acetyl-CoA hydrolase [EC:3.1.2.1] | ec:3.1.2.1 |
| reh:H16\_A1359 | h16\_A1359; hypothetical protein |  |
| reh:H16\_A1360 | h16\_A1360; extra-cytoplasmic solute receptor |  |
| reh:H16\_A1361 | tRNA-Lys\_(CTT); tRNA-Lys; K14229 tRNA Lys |  |
| reh:H16\_A1362 | h16\_A1362; LysR family transcriptional regulator |  |
| reh:H16\_A1363 | h16\_A1363; hydroxydechloroatrazine ethylaminohydrolase (EC:3.5.99.3); K03382 hydroxyatrazine ethylaminohydrolase [EC:3.5.99.3] | ec:3.5.99.3 |
| reh:H16\_A1364 | tRNA-Lys\_(CTT); tRNA-Lys; K14229 tRNA Lys |  |
| reh:H16\_A1365 | h16\_A1365; hypothetical protein |  |
| reh:H16\_A1366 | h16\_A1366; sulfate permease family transporter; K03321 sulfate permease, SulP family |  |
| reh:H16\_A1367 | h16\_A1367; MarR family transcriptional regulator |  |
| reh:H16\_A1368 | h16\_A1368; exonuclease III (EC:3.1.11.2); K01142 exodeoxyribonuclease III [EC:3.1.11.2] | ec:3.1.11.2 |
| reh:H16\_A1369 | dcp; oligopeptidase A (EC:3.4.24.70); K01414 oligopeptidase A [EC:3.4.24.70] | ec:3.4.24.70 |
| reh:H16\_A1370 | folD; bifunctional 5,10-methylene-tetrahydrofolate dehydrogenase/5,10-methylene-tetrahydrofolate cyclohydrolase (EC:1.5.1.5); K01491 methylenetetrahydrofolate dehydrogenase (NADP+) / methenyltetrahydrofolate cyclohydrolase [EC:1.5.1.5 3.5.4.9] | ec:3.5.4.9 ec:1.5.1.5 |
| reh:H16\_A1371 | h16\_A1371; hypothetical protein |  |
| reh:H16\_A1372 | h16\_A1372; response regulator |  |
| reh:H16\_A1373 | h16\_A1373; signal transduction histidine kinase (EC:2.7.3.-); K00936 [EC:2.7.3.-] |  |
| reh:H16\_A1374 | aceE; pyruvate dehydrogenase subunit E1 (EC:1.2.4.1); K00163 pyruvate dehydrogenase E1 component [EC:1.2.4.1] | ec:1.2.4.1 |
| reh:H16\_A1375 | pdhB; dihydrolipoamide acetyltransferase (EC:2.3.1.12); K00627 pyruvate dehydrogenase E2 component (dihydrolipoamide acetyltransferase) [EC:2.3.1.12] | ec:2.3.1.12 |
| reh:H16\_A1376 | hslV; ATP-dependent protease HslVU (ClpYQ), peptidase subunit (EC:3.4.25.1); K01419 ATP-dependent HslUV protease, peptidase subunit HslV [EC:3.4.25.2] | ec:3.4.25.2 |
| reh:H16\_A1377 | pdhL; dihydrolipoamide dehydrogenase (E3) component ofpyruvate dehydrogenase (EC:1.8.1.4); K00382 dihydrolipoamide dehydrogenase [EC:1.8.1.4] | ec:1.8.1.4 |

  
**Neighborhood Representations for "rsc:RCFBP\_11599"**  

| ID | Annotation | EC number |
| --- | --- | --- |
| rsc:RCFBP\_11587 | hypothetical protein |  |
| rsc:RCFBP\_11588 | resolvase, N-:resolvase helix-turN-helix region |  |
| rsc:RCFBP\_11589 | transposase of insertion sequence isrta2, is256 family |  |
| rsc:RCFBP\_11591 | aldose epimerase or galactose mutarotase |  |
| rsc:RCFBP\_11593 | hypothetical protein |  |
| rsc:RCFBP\_11594 | dinB; DNA polymerase iv, devoid of proofreading, damage-inducible protein p (EC:2.7.7.7); K02346 DNA polymerase IV [EC:2.7.7.7] | ec:2.7.7.7 |
| rsc:RCFBP\_11595 | cationic amino acid transporter; K03294 basic amino acid/polyamine antiporter, APA family |  |
| rsc:RCFBP\_11596 | hypothetical protein |  |
| rsc:RCFBP\_11597 | hypothetical protein |  |
| rsc:RCFBP\_11598 | hpaI; 2-dehydro-3-deoxyglucarate aldolase (EC:4.1.2.20); K02510 4-hydroxy-2-oxoheptanedioate aldolase [EC:4.1.2.52] | ec:4.1.2.52 |
| rsc:RCFBP\_11599 | hpcR; homoprotocatechuate degradative operon repressor |  |
| rsc:RCFBP\_11600 | xthA; exodeoxyribonuclease III (EC:3.1.11.2); K01142 exodeoxyribonuclease III [EC:3.1.11.2] | ec:3.1.11.2 |
| rsc:RCFBP\_11601 | codA; cytosine deaminase (EC:3.5.4.1); K01485 cytosine deaminase [EC:3.5.4.1] | ec:3.5.4.1 |
| rsc:RCFBP\_11602 | prlC; oligopeptidase a (EC:3.4.24.70); K01414 oligopeptidase A [EC:3.4.24.70] | ec:3.4.24.70 |
| rsc:RCFBP\_11603 | folD; bifunctional: 5,10-methylene-tetrahydrofolate dehydrogenase; 5,10-methylene-tetrahydrofolate cyclohydrolase (EC:1.5.1.5 3.5.4.9); K01491 methylenetetrahydrofolate dehydrogenase (NADP+) / methenyltetrahydrofolate cyclohydrolase [EC:1.5.1.5 3.5.4.9] | ec:3.5.4.9 ec:1.5.1.5 |
| rsc:RCFBP\_11604 | fixJ; two component transcriptional regulatory protein |  |
| rsc:RCFBP\_11605 | sensor hybrid histidine kinase (EC:2.7.13.3) |  |
| rsc:RCFBP\_11606 | pdhA; pyruvate dehydrogenase, decarboxylase subunit e1, thiamiN-binding (EC:1.2.4.1); K00163 pyruvate dehydrogenase E1 component [EC:1.2.4.1] | ec:1.2.4.1 |
| rsc:RCFBP\_11607 | pdhB; dihydrolipoyllysine-residue succinyltransferase, component of pyruvate dehydrogenase complex (e2) (EC:2.3.1.12); K00627 pyruvate dehydrogenase E2 component (dihydrolipoamide acetyltransferase) [EC:2.3.1.12] | ec:2.3.1.12 |
| rsc:RCFBP\_11608 | hydrolase |  |
| rsc:RCFBP\_11609 | pdhL; dihydrolipoamide dehydrogenase, fad/NAD(P)-binding, component of the 2-oxoglutarate dehydrogenase and the pyruvate dehydrogenase complexes (EC:1.8.1.4); K00382 dihydrolipoamide dehydrogenase [EC:1.8.1.4] | ec:1.8.1.4 |

  
**Neighborhood Representations for "mag:amb0811"**  

| ID | Annotation | EC number |
| --- | --- | --- |
| mag:amb0801 | 2,4-dihydroxyhept-2-ene-1,7-dioic acid aldolase; K02510 4-hydroxy-2-oxoheptanedioate aldolase [EC:4.1.2.52] | ec:4.1.2.52 |
| mag:amb0802 | 2-keto-4-pentenoate hydratase; K02509 2-oxo-hept-3-ene-1,7-dioate hydratase [EC:4.2.1.-] |  |
| mag:amb0803 | 5-carboxymethyl-2-hydroxymuconate isomerase; K01826 5-carboxymethyl-2-hydroxymuconate isomerase [EC:5.3.3.10] | ec:5.3.3.10 |
| mag:amb0804 | hypothetical protein; K00455 3,4-dihydroxyphenylacetate 2,3-dioxygenase [EC:1.13.11.15] | ec:1.13.11.15 |
| mag:amb0805 | NAD-dependent aldehyde dehydrogenase; K00151 5-carboxymethyl-2-hydroxymuconic-semialdehyde dehydrogenase [EC:1.2.1.60] | ec:1.2.1.60 |
| mag:amb0806 | 2-keto-4-pentenoate hydratase/2-oxohepta-3-ene-1,7-dioic acid hydratase; K05921 5-oxopent-3-ene-1,2,5-tricarboxylate decarboxylase / 2-hydroxyhepta-2,4-diene-1,7-dioate isomerase [EC:4.1.1.68 5.3.3.-] | ec:4.1.1.68 |
| mag:amb0807 | 2-keto-4-pentenoate hydratase/2-oxohepta-3-ene-1,7-dioic acid hydratase; K05921 5-oxopent-3-ene-1,2,5-tricarboxylate decarboxylase / 2-hydroxyhepta-2,4-diene-1,7-dioate isomerase [EC:4.1.1.68 5.3.3.-] | ec:4.1.1.68 |
| mag:amb0808 | TRAP-type C4-dicarboxylate transport system |  |
| mag:amb0809 | hypothetical protein |  |
| mag:amb0810 | TRAP-type C4-dicarboxylate transport system |  |
| mag:amb0811 | transcriptional regulator |  |
| mag:amb0812 | ornithine/acetylornithine aminotransferase; K00821 acetylornithine/N-succinyldiaminopimelate aminotransferase [EC:2.6.1.11 2.6.1.17] | ec:2.6.1.17 ec:2.6.1.11 |
| mag:amb0813 | hypothetical protein |  |
| mag:amb0814 | NAD-dependent aldehyde dehydrogenase; K00135 succinate-semialdehyde dehydrogenase / glutarate-semialdehyde dehydrogenase [EC:1.2.1.16 1.2.1.79 1.2.1.20] | ec:1.2.1.79 ec:1.2.1.16 ec:1.2.1.20 |
| mag:amb0815 | transcriptional regulator |  |
| mag:amb0816 | acyl-CoA transferase/carnitine dehydratase |  |
| mag:amb0817 | hypothetical protein |  |
| mag:amb0818 | hypothetical protein |  |
| mag:amb0819 | hypothetical protein |  |
| mag:amb0820 | hypothetical protein |  |
| mag:amb0821 | transglutaminase-like enzyme |  |

  
**Neighborhood Representations for "axy:AXYL\_04170"**  

| ID | Annotation | EC number |
| --- | --- | --- |
| axy:AXYL\_04160 | malate/L-lactate dehydrogenase (EC:1.1.1.-) |  |
| axy:AXYL\_04161 | GntR family transcriptional regulator |  |
| axy:AXYL\_04162 | hypothetical protein |  |
| axy:AXYL\_04163 | extra-cytoplasmic solute receptor family protein 99 |  |
| axy:AXYL\_04164 | extra-cytoplasmic solute receptor family protein 100 |  |
| axy:AXYL\_04165 | gyaR; glyoxylate reductase (EC:1.1.1.26) |  |
| axy:AXYL\_04166 | hypothetical protein |  |
| axy:AXYL\_04167 | hypothetical protein; K07090 |  |
| axy:AXYL\_04168 | radA; DNA repair protein RadA; K04485 DNA repair protein RadA/Sms |  |
| axy:AXYL\_04169 | hypothetical protein |  |
| axy:AXYL\_04170 | hpaR; homoprotocatechuate degradation operon regulator, HpaR |  |
| axy:AXYL\_04171 | hpaD; 3,4-dihydroxyphenylacetate 2,3-dioxygenase (EC:1.13.11.15); K00455 3,4-dihydroxyphenylacetate 2,3-dioxygenase [EC:1.13.11.15] | ec:1.13.11.15 |
| axy:AXYL\_04172 | hpaE; 5-carboxymethyl-2-hydroxymuconate semialdehyde dehydrogenase (EC:1.2.1.60); K00151 5-carboxymethyl-2-hydroxymuconic-semialdehyde dehydrogenase [EC:1.2.1.60] | ec:1.2.1.60 |
| axy:AXYL\_04173 | 4-hydroxyphenylacetate degradation bifunctional isomerase/decarboxylase subunit A (EC:4.1.1.68 5.3.3.10) |  |
| axy:AXYL\_04174 | 4-hydroxyphenylacetate degradation bifunctional isomerase/decarboxylase subunit B (EC:4.1.1.68 5.3.3.10); K05921 5-oxopent-3-ene-1,2,5-tricarboxylate decarboxylase / 2-hydroxyhepta-2,4-diene-1,7-dioate isomerase [EC:4.1.1.68 5.3.3.-] | ec:4.1.1.68 |
| axy:AXYL\_04175 | hpaH; 2-oxo-hepta-3-ene-1,7-dioic acid hydratase (EC:4.2.-.-); K02509 2-oxo-hept-3-ene-1,7-dioate hydratase [EC:4.2.1.-] |  |
| axy:AXYL\_04176 | hpaI; 2,4-dihydroxyhept-2-ene-1,7-dioic acid aldolase (EC:4.1.2.-); K02510 4-hydroxy-2-oxoheptanedioate aldolase [EC:4.1.2.52] | ec:4.1.2.52 |
| axy:AXYL\_04177 | alpha/beta hydrolase |  |
| axy:AXYL\_04178 | extra-cytoplasmic solute receptor family protein 101 |  |
| axy:AXYL\_04179 | metallo-beta-lactamase; K00784 ribonuclease Z [EC:3.1.26.11] | ec:3.1.26.11 |
| axy:AXYL\_04180 | LysR family transcriptional regulator |  |

  
**Neighborhood Representations for "brh:RBRH\_01410"**  

| ID | Annotation | EC number |
| --- | --- | --- |
| brh:RBRH\_01400 | hypothetical protein |  |
| brh:RBRH\_01401 | Osmolarity sensor protein envZ (EC:2.7.3.-); K07638 two-component system, OmpR family, osmolarity sensor histidine kinase EnvZ [EC:2.7.13.3] | ec:2.7.13.3 |
| brh:RBRH\_01402 | transcriptional regulatory protein ompR; K07659 two-component system, OmpR family, phosphate regulon response regulator OmpR |  |
| brh:RBRH\_01403 | hypothetical protein |  |
| brh:RBRH\_01404 | hypothetical protein |  |
| brh:RBRH\_01405 | histidine transport system permease hisQ; K10016 histidine transport system permease protein |  |
| brh:RBRH\_04111 | histidine transport system permease hisM; K10015 histidine transport system permease protein |  |
| brh:RBRH\_01406 | histidine transport ATP-binding protein hisP; K10017 histidine transport system ATP-binding protein [EC:3.6.3.21] | ec:3.6.3.21 |
| brh:RBRH\_01407 | RpiR family transcriptional regulator (EC:2.7.1.2); K00845 glucokinase [EC:2.7.1.2] | ec:2.7.1.2 |
| brh:RBRH\_01408 | glucose-6-phosphate 1-dehydrogenase (EC:1.1.1.49); K00036 glucose-6-phosphate 1-dehydrogenase [EC:1.1.1.49] | ec:1.1.1.49 |
| brh:RBRH\_01410 | MarR family transcriptional regulator |  |
| brh:RBRH\_01411 | multidrug resistance transporter, Bcr family; K07552 MFS transporter, DHA1 family, bicyclomycin/chloramphenicol resistance protein |  |
| brh:RBRH\_01412 | glutamate-1-semialdehyde 2,1-aminomutase (EC:5.4.3.8); K01845 glutamate-1-semialdehyde 2,1-aminomutase [EC:5.4.3.8] | ec:5.4.3.8 |
| brh:RBRH\_01413 | Diaminohydroxyphosphoribosylaminopyrimidine deaminase / 5-amino-6-(5-phosphoribosylamino)uracil reductase (EC:3.5.4.26 1.1.1.193); K11752 diaminohydroxyphosphoribosylaminopyrimidine deaminase / 5-amino-6-(5-phosphoribosylamino)uracil reductase [EC:3.5.4.26 1.1.1.193] | ec:3.5.4.26 ec:1.1.1.193 |
| brh:RBRH\_01414 | riboflavin synthase subunit alpha (EC:2.5.1.9); K00793 riboflavin synthase [EC:2.5.1.9] | ec:2.5.1.9 |
| brh:RBRH\_01415 | hypothetical protein |  |
| brh:RBRH\_04110 | hypothetical protein |  |
| brh:RBRH\_01416 | FMN reductase |  |
| brh:RBRH\_01417 | hypothetical protein |  |
| brh:RBRH\_01418 | GTP cyclohydrolase II / 3,4-dihydroxy-2-butanone-4-phosphate synthase (EC:3.5.4.25 4.1.99.12); K14652 3,4-dihydroxy 2-butanone 4-phosphate synthase / GTP cyclohydrolase II [EC:4.1.99.12 3.5.4.25] | ec:3.5.4.25 ec:4.1.99.12 |
| brh:RBRH\_01419 | 6,7-dimethyl-8-ribityllumazine synthase (EC:2.5.1.9); K00794 6,7-dimethyl-8-ribityllumazine synthase [EC:2.5.1.78] | ec:2.5.1.78 |

  
**Neighborhood Representations for "cti:RALTA\_A1286"**  

| ID | Annotation | EC number |
| --- | --- | --- |
| cti:RALTA\_A1276 | nad-glutamate dehydrogenase (EC:1.4.1.2); K15371 glutamate dehydrogenase [EC:1.4.1.2] | ec:1.4.1.2 |
| cti:RALTA\_A1277 | hypothetical protein |  |
| cti:RALTA\_A1278 | ygfH; propionyl-CoA:succinate-CoA transferase (EC:2.8.3.-) |  |
| cti:RALTA\_A1279 | hypothetical protein |  |
| cti:RALTA\_A1280 | hypothetical protein |  |
| cti:RALTA\_A1281 | hypothetical protein |  |
| cti:RALTA\_A1282 | LysR family transcriptional regulator |  |
| cti:RALTA\_A1283 | hydroxydechloroatrazine ethylaminohydrolase (EC:3.5.99.3); K03382 hydroxyatrazine ethylaminohydrolase [EC:3.5.99.3] | ec:3.5.99.3 |
| cti:RALTA\_A1284 | hypothetical protein |  |
| cti:RALTA\_A1285 | sulfate transporter; K03321 sulfate permease, SulP family |  |
| cti:RALTA\_A1286 | marr family transcription regulator |  |
| cti:RALTA\_A1287 | xthA; exodeoxyribonuclease iii (EC:3.1.11.2); K01142 exodeoxyribonuclease III [EC:3.1.11.2] | ec:3.1.11.2 |
| cti:RALTA\_A1288 | prlC; oligopeptidase a (EC:3.4.24.70); K01414 oligopeptidase A [EC:3.4.24.70] | ec:3.4.24.70 |
| cti:RALTA\_A1289 | folD; bifunctional 5,10-methylene-tetrahydrofolate dehydrogenase/5,10-methylene-tetrahydrofolate cyclohydrolase (EC:1.5.1.5 3.5.4.9); K01491 methylenetetrahydrofolate dehydrogenase (NADP+) / methenyltetrahydrofolate cyclohydrolase [EC:1.5.1.5 3.5.4.9] | ec:3.5.4.9 ec:1.5.1.5 |
| cti:RALTA\_A1290 | hypothetical protein |  |
| cti:RALTA\_A1291 | response regulator |  |
| cti:RALTA\_A1292 | sensor hybrid histidine kinase; K00936 [EC:2.7.3.-] |  |
| cti:RALTA\_A1293 | aceE; pyruvate dehydrogenase subunit e1 (EC:1.2.4.1); K00163 pyruvate dehydrogenase E1 component [EC:1.2.4.1] | ec:1.2.4.1 |
| cti:RALTA\_A1294 | pdhB; dihydrolipoamide acetyltransferase (EC:2.3.1.12); K00627 pyruvate dehydrogenase E2 component (dihydrolipoamide acetyltransferase) [EC:2.3.1.12] | ec:2.3.1.12 |
| cti:RALTA\_A1296 | hydrolase, proteasome hslv type peptidase domain |  |
| cti:RALTA\_A1297 | pdhL; dihydrolipoamide dehydrogenase, fad/NAD(P)-binding, component of the 2-oxoglutarate dehydrogenase and the pyruvate dehydrogenase complexes (EC:1.8.1.4); K00382 dihydrolipoamide dehydrogenase [EC:1.8.1.4] | ec:1.8.1.4 |

  
**Neighborhood Representations for "rsl:RPSI07\_1682"**  

| ID | Annotation | EC number |
| --- | --- | --- |
| rsl:RPSI07\_1672 | MerR family transcriptional regulator |  |
| rsl:RPSI07\_1673 | lipoprotein, Rhodopsin-like GPCR superfamily domain |  |
| rsl:RPSI07\_1674 | hypothetical protein |  |
| rsl:RPSI07\_1675 | aldose epimerase or galactose mutarotase |  |
| rsl:RPSI07\_1676 | hypothetical protein |  |
| rsl:RPSI07\_1677 | dinB; DNA polymerase IV, devoid of proofreading, damage-inducible protein P (EC:2.7.7.7); K02346 DNA polymerase IV [EC:2.7.7.7] | ec:2.7.7.7 |
| rsl:RPSI07\_1678 | cationic amino acid transporter; K03294 basic amino acid/polyamine antiporter, APA family |  |
| rsl:RPSI07\_1679 | hypothetical protein |  |
| rsl:RPSI07\_1680 | hypothetical protein |  |
| rsl:RPSI07\_1681 | hpaI; 2-dehydro-3-deoxyglucarate aldolase (EC:4.1.2.20); K02510 4-hydroxy-2-oxoheptanedioate aldolase [EC:4.1.2.52] | ec:4.1.2.52 |
| rsl:RPSI07\_1682 | hpcR; homoprotocatechuate degradative operon repressor |  |
| rsl:RPSI07\_1683 | xthA; exodeoxyribonuclease III (EC:3.1.11.2); K01142 exodeoxyribonuclease III [EC:3.1.11.2] | ec:3.1.11.2 |
| rsl:RPSI07\_1684 | codA; cytosine deaminase (EC:3.5.4.1); K01485 cytosine deaminase [EC:3.5.4.1] | ec:3.5.4.1 |
| rsl:RPSI07\_1685 | prlC; oligopeptidase A (EC:3.4.24.70); K01414 oligopeptidase A [EC:3.4.24.70] | ec:3.4.24.70 |
| rsl:RPSI07\_1686 | folD; bifunctional 5,10-methylene-tetrahydrofolate dehydrogenase/5,10-methylene-tetrahydrofolate cyclohydrolase (EC:1.5.1.5 3.5.4.9); K01491 methylenetetrahydrofolate dehydrogenase (NADP+) / methenyltetrahydrofolate cyclohydrolase [EC:1.5.1.5 3.5.4.9] | ec:3.5.4.9 ec:1.5.1.5 |
| rsl:RPSI07\_1687 | fixJ; two component transcriptional regulator |  |
| rsl:RPSI07\_1688 | sensor hybrid histidine kinase (EC:2.7.13.3) |  |
| rsl:RPSI07\_1689 | hypothetical protein |  |
| rsl:RPSI07\_1690 | pdhA; pyruvate dehydrogenase, decarboxylase subunit E1, thiamin-binding (EC:1.2.4.1); K00163 pyruvate dehydrogenase E1 component [EC:1.2.4.1] | ec:1.2.4.1 |
| rsl:RPSI07\_1691 | pdhB; dihydrolipoyllysine-residue succinyltransferase, component of pyruvate dehydrogenase complex (E2) (EC:2.3.1.12); K00627 pyruvate dehydrogenase E2 component (dihydrolipoamide acetyltransferase) [EC:2.3.1.12] | ec:2.3.1.12 |
| rsl:RPSI07\_1692 | hydrolase, proteasome hslV type peptidase domain |  |

  
**Neighborhood Representations for "rso:RSc1592"**  

| ID | Annotation | EC number |
| --- | --- | --- |
| rso:RSc1582 | pheT; phenylalanyl-tRNA synthetase subunit beta (EC:6.1.1.20); K01890 phenylalanyl-tRNA synthetase beta chain [EC:6.1.1.20] | ec:6.1.1.20 |
| rso:RSc1583 | ihfA; integration host factor subunit alpha; K04764 integration host factor subunit alpha |  |
| rso:RSc1584 | transcription regulator protein |  |
| rso:RSc1585 | hypothetical protein |  |
| rso:RSc1586 | lipoprotein |  |
| rso:RSc1587 | dinP; DNA polymerase IV (EC:2.7.7.7); K02346 DNA polymerase IV [EC:2.7.7.7] | ec:2.7.7.7 |
| rso:RSc1588 | amino-acid transporter transmembrane protein; K03294 basic amino acid/polyamine antiporter, APA family |  |
| rso:RSc1589 | hypothetical protein |  |
| rso:RSc1590 | hypothetical protein |  |
| rso:RSc1591 | hpaI1; 2,4-dihydroxyhept-2-ene-1,7-dioic acid aldolase (EC:4.1.2.-); K02510 4-hydroxy-2-oxoheptanedioate aldolase [EC:4.1.2.52] | ec:4.1.2.52 |
| rso:RSc1592 | transcription regulator protein |  |
| rso:RSc1593 | xthA; exodeoxyribonuclease III (EC:3.1.11.2); K01142 exodeoxyribonuclease III [EC:3.1.11.2] | ec:3.1.11.2 |
| rso:RSc1594 | codA; cytosine deaminase (EC:3.5.4.1); K01485 cytosine deaminase [EC:3.5.4.1] | ec:3.5.4.1 |
| rso:RSc1595 | prlC; oligopeptidase A; K01414 oligopeptidase A [EC:3.4.24.70] | ec:3.4.24.70 |
| rso:RSc1596 | folD; bifunctional 5,10-methylene-tetrahydrofolate dehydrogenase/5,10-methylene-tetrahydrofolate cyclohydrolase (EC:1.5.1.5 3.5.4.9); K01491 methylenetetrahydrofolate dehydrogenase (NADP+) / methenyltetrahydrofolate cyclohydrolase [EC:1.5.1.5 3.5.4.9] | ec:3.5.4.9 ec:1.5.1.5 |
| rso:RSc1597 | response regulator transcription regulator protein |  |
| rso:RSc1598 | two-component transmembrane sensor histidine kinase transcription regulator protein (EC:2.7.3.-); K00936 [EC:2.7.3.-] |  |
| rso:RSc1599 | hypothetical protein |  |
| rso:RSc1600 | aceE; pyruvate dehydrogenase subunit E1 (EC:1.2.4.1); K00163 pyruvate dehydrogenase E1 component [EC:1.2.4.1] | ec:1.2.4.1 |
| rso:RSc1601 | aceF; dihydrolipoamide acetyltransferase (EC:2.3.1.12); K00627 pyruvate dehydrogenase E2 component (dihydrolipoamide acetyltransferase) [EC:2.3.1.12] | ec:2.3.1.12 |
| rso:RSc1602 | hypothetical protein |  |

  
**Neighborhood Representations for "rpi:Rpic\_1953"**  

| ID | Annotation | EC number |
| --- | --- | --- |
| rpi:Rpic\_1943 | hypothetical protein |  |
| rpi:Rpic\_1944 | dihydrolipoamide dehydrogenase; K00382 dihydrolipoamide dehydrogenase [EC:1.8.1.4] | ec:1.8.1.4 |
| rpi:Rpic\_1945 | 20S proteasome subunits A and B |  |
| rpi:Rpic\_1946 | dihydrolipoamide acetyltransferase; K00627 pyruvate dehydrogenase E2 component (dihydrolipoamide acetyltransferase) [EC:2.3.1.12] | ec:2.3.1.12 |
| rpi:Rpic\_1947 | aceE; pyruvate dehydrogenase subunit E1; K00163 pyruvate dehydrogenase E1 component [EC:1.2.4.1] | ec:1.2.4.1 |
| rpi:Rpic\_1948 | PAS/PAC sensor signal transduction histidine kinase (EC:2.7.13.3) |  |
| rpi:Rpic\_1949 | two component LuxR family transcriptional regulator |  |
| rpi:Rpic\_1950 | bifunctional 5,10-methylene-tetrahydrofolate dehydrogenase/5,10-methylene-tetrahydrofolate cyclohydrolase (EC:1.5.1.5); K01491 methylenetetrahydrofolate dehydrogenase (NADP+) / methenyltetrahydrofolate cyclohydrolase [EC:1.5.1.5 3.5.4.9] | ec:3.5.4.9 ec:1.5.1.5 |
| rpi:Rpic\_1951 | oligopeptidase A (EC:3.4.24.70); K01414 oligopeptidase A [EC:3.4.24.70] | ec:3.4.24.70 |
| rpi:Rpic\_1952 | exodeoxyribonuclease III Xth; K01142 exodeoxyribonuclease III [EC:3.1.11.2] | ec:3.1.11.2 |
| rpi:Rpic\_1953 | MarR family transcriptional regulator |  |
| rpi:Rpic\_1954 | 2,4-dihydroxyhept-2-ene-1,7-dioic acid aldolase (EC:4.1.2.20); K02510 4-hydroxy-2-oxoheptanedioate aldolase [EC:4.1.2.52] | ec:4.1.2.52 |
| rpi:Rpic\_1955 | 2-oxo-hepta-3-ene-1,7-dioic acid hydratase (EC:4.1.1.77); K02509 2-oxo-hept-3-ene-1,7-dioate hydratase [EC:4.2.1.-] |  |
| rpi:Rpic\_1956 | 5-carboxymethyl-2-hydroxymuconate isomerase; K01826 5-carboxymethyl-2-hydroxymuconate isomerase [EC:5.3.3.10] | ec:5.3.3.10 |
| rpi:Rpic\_1957 | 3,4-dihydroxyphenylacetate 2,3-dioxygenase (EC:1.13.11.15); K00455 3,4-dihydroxyphenylacetate 2,3-dioxygenase [EC:1.13.11.15] | ec:1.13.11.15 |
| rpi:Rpic\_1958 | 5-carboxymethyl-2-hydroxymuconate semialdehyde dehydrogenase; K00151 5-carboxymethyl-2-hydroxymuconic-semialdehyde dehydrogenase [EC:1.2.1.60] | ec:1.2.1.60 |
| rpi:Rpic\_1959 | 4-hydroxyphenylacetate degradation bifunctional isomerase/decarboxylase subunit HpaG2 (EC:5.3.3.10); K05921 5-oxopent-3-ene-1,2,5-tricarboxylate decarboxylase / 2-hydroxyhepta-2,4-diene-1,7-dioate isomerase [EC:4.1.1.68 5.3.3.-] | ec:4.1.1.68 |
| rpi:Rpic\_1960 | 4-hydroxyphenylacetate degradation bifunctional isomerase/decarboxylase subunit HpaG1 (EC:4.1.1.68); K05921 5-oxopent-3-ene-1,2,5-tricarboxylate decarboxylase / 2-hydroxyhepta-2,4-diene-1,7-dioate isomerase [EC:4.1.1.68 5.3.3.-] | ec:4.1.1.68 |
| rpi:Rpic\_1961 | hypothetical protein |  |
| rpi:Rpic\_1962 | amino acid permease-associated protein; K03294 basic amino acid/polyamine antiporter, APA family |  |
| rpi:Rpic\_1963 | DNA polymerase IV (EC:2.7.7.7); K02346 DNA polymerase IV [EC:2.7.7.7] | ec:2.7.7.7 |

  
**Neighborhood Representations for "rpf:Rpic12D\_1625"**  

| ID | Annotation | EC number |
| --- | --- | --- |
| rpf:Rpic12D\_1615 | hypothetical protein |  |
| rpf:Rpic12D\_1616 | dihydrolipoamide dehydrogenase; K00382 dihydrolipoamide dehydrogenase [EC:1.8.1.4] | ec:1.8.1.4 |
| rpf:Rpic12D\_1617 | 20S proteasome subunits A and B |  |
| rpf:Rpic12D\_1618 | dihydrolipoamide acetyltransferase; K00627 pyruvate dehydrogenase E2 component (dihydrolipoamide acetyltransferase) [EC:2.3.1.12] | ec:2.3.1.12 |
| rpf:Rpic12D\_1619 | aceE; pyruvate dehydrogenase subunit E1; K00163 pyruvate dehydrogenase E1 component [EC:1.2.4.1] | ec:1.2.4.1 |
| rpf:Rpic12D\_1620 | PAS/PAC sensor signal transduction histidine kinase (EC:2.7.13.3); K00936 [EC:2.7.3.-] |  |
| rpf:Rpic12D\_1621 | two component LuxR family transcriptional regulator |  |
| rpf:Rpic12D\_1622 | bifunctional 5,10-methylene-tetrahydrofolate dehydrogenase/5,10-methylene-tetrahydrofolate cyclohydrolase (EC:1.5.1.5); K01491 methylenetetrahydrofolate dehydrogenase (NADP+) / methenyltetrahydrofolate cyclohydrolase [EC:1.5.1.5 3.5.4.9] | ec:3.5.4.9 ec:1.5.1.5 |
| rpf:Rpic12D\_1623 | oligopeptidase A (EC:3.4.24.70); K01414 oligopeptidase A [EC:3.4.24.70] | ec:3.4.24.70 |
| rpf:Rpic12D\_1624 | exodeoxyribonuclease III Xth; K01142 exodeoxyribonuclease III [EC:3.1.11.2] | ec:3.1.11.2 |
| rpf:Rpic12D\_1625 | MarR family transcriptional regulator |  |
| rpf:Rpic12D\_1626 | 2,4-dihydroxyhept-2-ene-1,7-dioic acid aldolase (EC:4.1.2.20); K02510 4-hydroxy-2-oxoheptanedioate aldolase [EC:4.1.2.52] | ec:4.1.2.52 |
| rpf:Rpic12D\_1627 | 2-oxo-hepta-3-ene-1,7-dioic acid hydratase (EC:4.1.1.77); K02509 2-oxo-hept-3-ene-1,7-dioate hydratase [EC:4.2.1.-] |  |
| rpf:Rpic12D\_1628 | 5-carboxymethyl-2-hydroxymuconate isomerase; K01826 5-carboxymethyl-2-hydroxymuconate isomerase [EC:5.3.3.10] | ec:5.3.3.10 |
| rpf:Rpic12D\_1629 | 3,4-dihydroxyphenylacetate 2,3-dioxygenase (EC:1.13.11.15); K00455 3,4-dihydroxyphenylacetate 2,3-dioxygenase [EC:1.13.11.15] | ec:1.13.11.15 |
| rpf:Rpic12D\_1630 | 5-carboxymethyl-2-hydroxymuconate semialdehyde dehydrogenase; K00151 5-carboxymethyl-2-hydroxymuconic-semialdehyde dehydrogenase [EC:1.2.1.60] | ec:1.2.1.60 |
| rpf:Rpic12D\_1631 | 4-hydroxyphenylacetate degradation bifunctional isomerase/decarboxylase subunit HpaG2 (EC:4.1.1.68); K05921 5-oxopent-3-ene-1,2,5-tricarboxylate decarboxylase / 2-hydroxyhepta-2,4-diene-1,7-dioate isomerase [EC:4.1.1.68 5.3.3.-] | ec:4.1.1.68 |
| rpf:Rpic12D\_1632 | 4-hydroxyphenylacetate degradation bifunctional isomerase/decarboxylase subunit HpaG1 (EC:4.1.1.68); K05921 5-oxopent-3-ene-1,2,5-tricarboxylate decarboxylase / 2-hydroxyhepta-2,4-diene-1,7-dioate isomerase [EC:4.1.1.68 5.3.3.-] | ec:4.1.1.68 |
| rpf:Rpic12D\_1633 | hypothetical protein |  |
| rpf:Rpic12D\_1634 | amino acid permease-associated protein; K03294 basic amino acid/polyamine antiporter, APA family |  |
| rpf:Rpic12D\_1635 | DNA polymerase IV (EC:2.7.7.7); K02346 DNA polymerase IV [EC:2.7.7.7] | ec:2.7.7.7 |

  
**Neighborhood Representations for "bch:Bcen2424\_4230"**  

| ID | Annotation | EC number |
| --- | --- | --- |
| bch:Bcen2424\_4220 | alanine dehydrogenase; K00259 alanine dehydrogenase [EC:1.4.1.1] | ec:1.4.1.1 |
| bch:Bcen2424\_4221 | hypothetical protein |  |
| bch:Bcen2424\_4222 | RNA:NAD 2'-phosphotransferase-like protein; K07559 putative RNA 2'-phosphotransferase [EC:2.7.1.-] |  |
| bch:Bcen2424\_4223 | hypothetical protein; K06152 gluconate 2-dehydrogenase gamma chain [EC:1.1.99.3] | ec:1.1.99.3 |
| bch:Bcen2424\_4224 | glucose-methanol-choline oxidoreductase; K06151 gluconate 2-dehydrogenase alpha chain [EC:1.1.99.3] | ec:1.1.99.3 |
| bch:Bcen2424\_4225 | cytochrome c, class I |  |
| bch:Bcen2424\_4226 | sodium/hydrogen exchanger; K03455 monovalent cation:H+ antiporter-2, CPA2 family |  |
| bch:Bcen2424\_4227 | peptidoglycan glycosyltransferase (EC:2.4.1.129); K03587 cell division protein FtsI (penicillin-binding protein 3) [EC:2.4.1.129] | ec:2.4.1.129 |
| bch:Bcen2424\_4228 | hypothetical protein |  |
| bch:Bcen2424\_4229 | hypothetical protein |  |
| bch:Bcen2424\_4230 | MarR family transcriptional regulator |  |
| bch:Bcen2424\_4231 | 4-hydroxyphenylacetate degradation bifunctional isomerase/decarboxylase subunit HpaG1 (EC:5.3.3.10); K05921 5-oxopent-3-ene-1,2,5-tricarboxylate decarboxylase / 2-hydroxyhepta-2,4-diene-1,7-dioate isomerase [EC:4.1.1.68 5.3.3.-] | ec:4.1.1.68 |
| bch:Bcen2424\_4232 | 4-hydroxyphenylacetate degradation bifunctional isomerase/decarboxylase subunit HpaG2 (EC:5.3.3.10); K05921 5-oxopent-3-ene-1,2,5-tricarboxylate decarboxylase / 2-hydroxyhepta-2,4-diene-1,7-dioate isomerase [EC:4.1.1.68 5.3.3.-] | ec:4.1.1.68 |
| bch:Bcen2424\_4233 | 5-carboxymethyl-2-hydroxymuconate semialdehyde dehydrogenase (EC:1.2.1.8); K00151 5-carboxymethyl-2-hydroxymuconic-semialdehyde dehydrogenase [EC:1.2.1.60] | ec:1.2.1.60 |
| bch:Bcen2424\_4234 | 3,4-dihydroxyphenylacetate 2,3-dioxygenase (EC:1.13.11.15); K00455 3,4-dihydroxyphenylacetate 2,3-dioxygenase [EC:1.13.11.15] | ec:1.13.11.15 |
| bch:Bcen2424\_4235 | 5-carboxymethyl-2-hydroxymuconate isomerase; K01826 5-carboxymethyl-2-hydroxymuconate isomerase [EC:5.3.3.10] | ec:5.3.3.10 |
| bch:Bcen2424\_4236 | 2-oxo-hepta-3-ene-1,7-dioic acid hydratase (EC:4.1.1.77); K02509 2-oxo-hept-3-ene-1,7-dioate hydratase [EC:4.2.1.-] |  |
| bch:Bcen2424\_4237 | 2,4-dihydroxyhept-2-ene-1,7-dioic acid aldolase (EC:4.1.2.20); K02510 4-hydroxy-2-oxoheptanedioate aldolase [EC:4.1.2.52] | ec:4.1.2.52 |
| bch:Bcen2424\_4238 | AraC family transcriptional regulator |  |
| bch:Bcen2424\_4239 | hypothetical protein |  |
| bch:Bcen2424\_4240 | alcohol dehydrogenase |  |

  
**Neighborhood Representations for "bcm:Bcenmc03\_3287"**  

| ID | Annotation | EC number |
| --- | --- | --- |
| bcm:Bcenmc03\_3277 | alcohol dehydrogenase |  |
| bcm:Bcenmc03\_3278 | hypothetical protein |  |
| bcm:Bcenmc03\_3279 | AraC family transcriptional regulator |  |
| bcm:Bcenmc03\_3280 | 2,4-dihydroxyhept-2-ene-1,7-dioic acid aldolase (EC:4.1.2.20); K02510 4-hydroxy-2-oxoheptanedioate aldolase [EC:4.1.2.52] | ec:4.1.2.52 |
| bcm:Bcenmc03\_3281 | 2-oxo-hepta-3-ene-1,7-dioic acid hydratase (EC:4.1.1.77); K02509 2-oxo-hept-3-ene-1,7-dioate hydratase [EC:4.2.1.-] |  |
| bcm:Bcenmc03\_3282 | 5-carboxymethyl-2-hydroxymuconate isomerase; K01826 5-carboxymethyl-2-hydroxymuconate isomerase [EC:5.3.3.10] | ec:5.3.3.10 |
| bcm:Bcenmc03\_3283 | 3,4-dihydroxyphenylacetate 2,3-dioxygenase (EC:1.13.11.15); K00455 3,4-dihydroxyphenylacetate 2,3-dioxygenase [EC:1.13.11.15] | ec:1.13.11.15 |
| bcm:Bcenmc03\_3284 | 5-carboxymethyl-2-hydroxymuconate semialdehyde dehydrogenase; K00151 5-carboxymethyl-2-hydroxymuconic-semialdehyde dehydrogenase [EC:1.2.1.60] | ec:1.2.1.60 |
| bcm:Bcenmc03\_3285 | 4-hydroxyphenylacetate degradation bifunctional isomerase/decarboxylase subunit HpaG2 (EC:4.1.1.68); K05921 5-oxopent-3-ene-1,2,5-tricarboxylate decarboxylase / 2-hydroxyhepta-2,4-diene-1,7-dioate isomerase [EC:4.1.1.68 5.3.3.-] | ec:4.1.1.68 |
| bcm:Bcenmc03\_3286 | 4-hydroxyphenylacetate degradation bifunctional isomerase/decarboxylase subunit HpaG1 (EC:4.1.1.68); K05921 5-oxopent-3-ene-1,2,5-tricarboxylate decarboxylase / 2-hydroxyhepta-2,4-diene-1,7-dioate isomerase [EC:4.1.1.68 5.3.3.-] | ec:4.1.1.68 |
| bcm:Bcenmc03\_3287 | MarR family transcriptional regulator |  |
| bcm:Bcenmc03\_3288 | hypothetical protein |  |
| bcm:Bcenmc03\_3289 | hypothetical protein |  |
| bcm:Bcenmc03\_3290 | peptidoglycan glycosyltransferase (EC:2.4.1.129); K03587 cell division protein FtsI (penicillin-binding protein 3) [EC:2.4.1.129] | ec:2.4.1.129 |
| bcm:Bcenmc03\_3291 | sodium/hydrogen exchanger; K03455 monovalent cation:H+ antiporter-2, CPA2 family |  |
| bcm:Bcenmc03\_3292 | gluconate 2-dehydrogenase (acceptor) (EC:1.1.99.3) |  |
| bcm:Bcenmc03\_3293 | gluconate 2-dehydrogenase (acceptor) (EC:1.1.99.3); K06151 gluconate 2-dehydrogenase alpha chain [EC:1.1.99.3] | ec:1.1.99.3 |
| bcm:Bcenmc03\_3294 | gluconate 2-dehydrogenase (acceptor) (EC:1.1.99.3); K06152 gluconate 2-dehydrogenase gamma chain [EC:1.1.99.3] | ec:1.1.99.3 |
| bcm:Bcenmc03\_3295 | pseudogene |  |
| bcm:Bcenmc03\_3296 | hypothetical protein |  |
| bcm:Bcenmc03\_3297 | alanine dehydrogenase; K00259 alanine dehydrogenase [EC:1.4.1.1] | ec:1.4.1.1 |

  
**Neighborhood Representations for "bcn:Bcen\_4136"**  

| ID | Annotation | EC number |
| --- | --- | --- |
| bcn:Bcen\_4126 | zinc-binding alcohol dehydrogenase |  |
| bcn:Bcen\_4127 | hypothetical protein |  |
| bcn:Bcen\_4128 | AraC family transcriptional regulator |  |
| bcn:Bcen\_4129 | 2,4-dihydroxyhept-2-ene-1,7-dioic acid aldolase (EC:4.1.2.20); K02510 4-hydroxy-2-oxoheptanedioate aldolase [EC:4.1.2.52] | ec:4.1.2.52 |
| bcn:Bcen\_4130 | 2-oxo-hepta-3-ene-1,7-dioic acid hydratase (EC:4.1.1.77); K02509 2-oxo-hept-3-ene-1,7-dioate hydratase [EC:4.2.1.-] |  |
| bcn:Bcen\_4131 | 5-carboxymethyl-2-hydroxymuconate isomerase; K01826 5-carboxymethyl-2-hydroxymuconate isomerase [EC:5.3.3.10] | ec:5.3.3.10 |
| bcn:Bcen\_4132 | 3,4-dihydroxyphenylacetate 2,3-dioxygenase (EC:1.13.11.15); K00455 3,4-dihydroxyphenylacetate 2,3-dioxygenase [EC:1.13.11.15] | ec:1.13.11.15 |
| bcn:Bcen\_4133 | 5-carboxymethyl-2-hydroxymuconate semialdehyde dehydrogenase (EC:1.2.1.8); K00151 5-carboxymethyl-2-hydroxymuconic-semialdehyde dehydrogenase [EC:1.2.1.60] | ec:1.2.1.60 |
| bcn:Bcen\_4134 | 4-hydroxyphenylacetate degradation bifunctional isomerase/decarboxylase (EC:5.3.3.10); K05921 5-oxopent-3-ene-1,2,5-tricarboxylate decarboxylase / 2-hydroxyhepta-2,4-diene-1,7-dioate isomerase [EC:4.1.1.68 5.3.3.-] | ec:4.1.1.68 |
| bcn:Bcen\_4135 | 4-hydroxyphenylacetate degradation bifunctional isomerase/decarboxylase (EC:5.3.3.10); K05921 5-oxopent-3-ene-1,2,5-tricarboxylate decarboxylase / 2-hydroxyhepta-2,4-diene-1,7-dioate isomerase [EC:4.1.1.68 5.3.3.-] | ec:4.1.1.68 |
| bcn:Bcen\_4136 | MarR family transcriptional regulator |  |
| bcn:Bcen\_4137 | Male sterility-like |  |
| bcn:Bcen\_4138 | hypothetical protein |  |
| bcn:Bcen\_4139 | peptidoglycan glycosyltransferase (EC:2.4.1.129); K03587 cell division protein FtsI (penicillin-binding protein 3) [EC:2.4.1.129] | ec:2.4.1.129 |
| bcn:Bcen\_4140 | sodium/hydrogen exchanger; K03455 monovalent cation:H+ antiporter-2, CPA2 family |  |
| bcn:Bcen\_4141 | cytochrome c, class I |  |
| bcn:Bcen\_4142 | glucose-methanol-choline oxidoreductase; K06151 gluconate 2-dehydrogenase alpha chain [EC:1.1.99.3] | ec:1.1.99.3 |
| bcn:Bcen\_4143 | hypothetical protein; K06152 gluconate 2-dehydrogenase gamma chain [EC:1.1.99.3] | ec:1.1.99.3 |
| bcn:Bcen\_4144 | RNA:NAD 2'-phosphotransferase-like; K07559 putative RNA 2'-phosphotransferase [EC:2.7.1.-] |  |
| bcn:Bcen\_4145 | hypothetical protein |  |
| bcn:Bcen\_4146 | alanine dehydrogenase; K00259 alanine dehydrogenase [EC:1.4.1.1] | ec:1.4.1.1 |

  
**Neighborhood Representations for "vap:Vapar\_0246"**  

| ID | Annotation | EC number |
| --- | --- | --- |
| vap:Vapar\_0236 | hypothetical protein |  |
| vap:Vapar\_0237 | dihydroxy-acid dehydratase (EC:4.2.1.9); K01687 dihydroxy-acid dehydratase [EC:4.2.1.9] | ec:4.2.1.9 |
| vap:Vapar\_0238 | hypothetical protein |  |
| vap:Vapar\_0239 | 2,4-dihydroxyhept-2-ene-1,7-dioic acid aldolase (EC:4.1.3.39); K02510 4-hydroxy-2-oxoheptanedioate aldolase [EC:4.1.2.52] | ec:4.1.2.52 |
| vap:Vapar\_0240 | 2-oxo-hepta-3-ene-1,7-dioic acid hydratase (EC:4.2.1.80); K02509 2-oxo-hept-3-ene-1,7-dioate hydratase [EC:4.2.1.-] |  |
| vap:Vapar\_0241 | 5-carboxymethyl-2-hydroxymuconate isomerase; K01826 5-carboxymethyl-2-hydroxymuconate isomerase [EC:5.3.3.10] | ec:5.3.3.10 |
| vap:Vapar\_0242 | 3,4-dihydroxyphenylacetate 2,3-dioxygenase (EC:1.13.11.15); K00455 3,4-dihydroxyphenylacetate 2,3-dioxygenase [EC:1.13.11.15] | ec:1.13.11.15 |
| vap:Vapar\_0243 | 5-carboxymethyl-2-hydroxymuconate semialdehyde dehydrogenase (EC:1.2.1.8); K00151 5-carboxymethyl-2-hydroxymuconic-semialdehyde dehydrogenase [EC:1.2.1.60] | ec:1.2.1.60 |
| vap:Vapar\_0244 | 4-hydroxyphenylacetate degradation bifunctional isomerase/decarboxylase subunit HpaG2 (EC:5.3.3.10); K05921 5-oxopent-3-ene-1,2,5-tricarboxylate decarboxylase / 2-hydroxyhepta-2,4-diene-1,7-dioate isomerase [EC:4.1.1.68 5.3.3.-] | ec:4.1.1.68 |
| vap:Vapar\_0245 | fumarylacetoacetate (FAA) hydrolase; K05921 5-oxopent-3-ene-1,2,5-tricarboxylate decarboxylase / 2-hydroxyhepta-2,4-diene-1,7-dioate isomerase [EC:4.1.1.68 5.3.3.-] | ec:4.1.1.68 |
| vap:Vapar\_0246 | MarR family transcriptional regulator |  |
| vap:Vapar\_0247 | hypothetical protein |  |
| vap:Vapar\_0248 | hypothetical protein |  |
| vap:Vapar\_0249 | hypothetical protein |  |
| vap:Vapar\_0250 | type VI secretion system Vgr family protein |  |
| vap:Vapar\_0251 | DNA repair protein RadA; K04485 DNA repair protein RadA/Sms |  |
| vap:Vapar\_0252 | hypothetical protein |  |
| vap:Vapar\_0253 | rdgC; recombination associated protein; K03554 recombination associated protein RdgC |  |
| vap:Vapar\_0254 | branched-chain amino acid aminotransferase; K00826 branched-chain amino acid aminotransferase [EC:2.6.1.42] | ec:2.6.1.42 |
| vap:Vapar\_0255 | hypothetical protein |  |
| vap:Vapar\_0256 | hypothetical protein |  |

  
**Neighborhood Representations for "bcj:BCAM1365"**  

| ID | Annotation | EC number |
| --- | --- | --- |
| bcj:BCAM1355 | kptA; putative phosphotransferase (EC:2.7.-.-); K07559 putative RNA 2'-phosphotransferase [EC:2.7.1.-] |  |
| bcj:BCAM1356 | putative gluconate 2-dehydrogenase subunit 3 (EC:1.1.99.3); K06152 gluconate 2-dehydrogenase gamma chain [EC:1.1.99.3] | ec:1.1.99.3 |
| bcj:BCAM1357 | gluconate 2-dehydrogenase flavoprotein subunit (EC:1.1.99.3); K06151 gluconate 2-dehydrogenase alpha chain [EC:1.1.99.3] | ec:1.1.99.3 |
| bcj:BCAM1358 | gluconate 2-dehydrogenase cytochrome c subunit (EC:1.1.99.3) |  |
| bcj:BCAM1359 | putative efflux pump/antiporter; K03455 monovalent cation:H+ antiporter-2, CPA2 family |  |
| bcj:BCAM1360 | short chain dehydrogenase |  |
| bcj:BCAM1361 | sugar-binding periplasmic protein precursor; K02058 simple sugar transport system substrate-binding protein |  |
| bcj:BCAM1362 | putative penicillin-binding protein; K03587 cell division protein FtsI (penicillin-binding protein 3) [EC:2.4.1.129] | ec:2.4.1.129 |
| bcj:BCAM1363 | hypothetical protein |  |
| bcj:BCAM1364 | putative NAD dependent epimerase/dehydratase |  |
| bcj:BCAM1365 | hpaR; putative homoprotocatechuate degradative operon repressor |  |
| bcj:BCAM1366 | hpaG; putative 5-carboxymethyl-2-hydroxymuconate delta-isomerase; K05921 5-oxopent-3-ene-1,2,5-tricarboxylate decarboxylase / 2-hydroxyhepta-2,4-diene-1,7-dioate isomerase [EC:4.1.1.68 5.3.3.-] | ec:4.1.1.68 |
| bcj:BCAM1367 | hpaG'; putative 5-carboxymethyl-2-oxo-hex-3-ene-1,7-dioate decarboxylase; K05921 5-oxopent-3-ene-1,2,5-tricarboxylate decarboxylase / 2-hydroxyhepta-2,4-diene-1,7-dioate isomerase [EC:4.1.1.68 5.3.3.-] | ec:4.1.1.68 |
| bcj:BCAM1368 | hpaE; 5-carboxy-2-hydroxymuconate semialdehyde dehydrogenase; K00151 5-carboxymethyl-2-hydroxymuconic-semialdehyde dehydrogenase [EC:1.2.1.60] | ec:1.2.1.60 |
| bcj:BCAM1369 | hpaD; homoprotocatechuate dyoxygenase (EC:1.13.11.15); K00455 3,4-dihydroxyphenylacetate 2,3-dioxygenase [EC:1.13.11.15] | ec:1.13.11.15 |
| bcj:BCAM1370 | hpaF; 5-carboxymethyl-2-hydroxymuconate isomerase; K01826 5-carboxymethyl-2-hydroxymuconate isomerase [EC:5.3.3.10] | ec:5.3.3.10 |
| bcj:BCAM1371 | hpaH; 2-oxo-hept-3-ene-1,7-dioate hydratase; K02509 2-oxo-hept-3-ene-1,7-dioate hydratase [EC:4.2.1.-] |  |
| bcj:BCAM1372 | hpaI; 2,4-dihydroxyhept-2-ene-1,7-dioic acid aldolase (EC:4.1.2.-); K02510 4-hydroxy-2-oxoheptanedioate aldolase [EC:4.1.2.52] | ec:4.1.2.52 |
| bcj:BCAM1373 | AraC family regulatory protein |  |
| bcj:BCAM1374 | hypothetical protein |  |
| bcj:BCAM1375 | putative zinc-binding dehydrogenase |  |

  
**Neighborhood Representations for "bur:Bcep18194\_B1784"**  

| ID | Annotation | EC number |
| --- | --- | --- |
| bur:Bcep18194\_B1774 | hypothetical protein |  |
| bur:Bcep18194\_B1775 | hypothetical protein |  |
| bur:Bcep18194\_B1776 | excisionase |  |
| bur:Bcep18194\_B1777 | 2-dehydro-3-deoxyglucarate aldolase (EC:4.1.2.20); K02510 4-hydroxy-2-oxoheptanedioate aldolase [EC:4.1.2.52] | ec:4.1.2.52 |
| bur:Bcep18194\_B1778 | 4-oxalocrotonate decarboxylase (EC:4.1.1.77); K02509 2-oxo-hept-3-ene-1,7-dioate hydratase [EC:4.2.1.-] |  |
| bur:Bcep18194\_B1779 | 5-carboxymethyl-2-hydroxymuconate isomerase; K01826 5-carboxymethyl-2-hydroxymuconate isomerase [EC:5.3.3.10] | ec:5.3.3.10 |
| bur:Bcep18194\_B1780 | 3,4-dihydroxyphenylacetate 2,3-dioxygenase (EC:1.13.11.15); K00455 3,4-dihydroxyphenylacetate 2,3-dioxygenase [EC:1.13.11.15] | ec:1.13.11.15 |
| bur:Bcep18194\_B1781 | 5-carboxymethyl-2-hydroxymuconate semialdehyde dehydrogenase (EC:1.2.1.8); K00151 5-carboxymethyl-2-hydroxymuconic-semialdehyde dehydrogenase [EC:1.2.1.60] | ec:1.2.1.60 |
| bur:Bcep18194\_B1782 | 5-carboxymethyl-2-hydroxymuconate delta-isomerase (EC:5.3.3.10); K05921 5-oxopent-3-ene-1,2,5-tricarboxylate decarboxylase / 2-hydroxyhepta-2,4-diene-1,7-dioate isomerase [EC:4.1.1.68 5.3.3.-] | ec:4.1.1.68 |
| bur:Bcep18194\_B1783 | 5-carboxymethyl-2-hydroxymuconate delta-isomerase (EC:5.3.3.10); K05921 5-oxopent-3-ene-1,2,5-tricarboxylate decarboxylase / 2-hydroxyhepta-2,4-diene-1,7-dioate isomerase [EC:4.1.1.68 5.3.3.-] | ec:4.1.1.68 |
| bur:Bcep18194\_B1784 | MarR family transcriptional regulator |  |
| bur:Bcep18194\_B1785 | NAD-dependent epimerase/dehydratase |  |
| bur:Bcep18194\_B1786 | hypothetical protein |  |
| bur:Bcep18194\_B1787 | hypothetical protein |  |
| bur:Bcep18194\_B1788 | peptidoglycan synthetase FtsI (EC:2.4.1.129); K03587 cell division protein FtsI (penicillin-binding protein 3) [EC:2.4.1.129] | ec:2.4.1.129 |
| bur:Bcep18194\_B1789 | periplasmic binding protein/LacI transcriptional regulator; K02058 simple sugar transport system substrate-binding protein |  |
| bur:Bcep18194\_B1790 | short chain dehydrogenase (EC:1.1.1.100) |  |
| bur:Bcep18194\_B1791 | TrkA-N family protein; K03455 monovalent cation:H+ antiporter-2, CPA2 family |  |
| bur:Bcep18194\_B1792 | cytochrome c, class I |  |
| bur:Bcep18194\_B1793 | glucose-methanol-choline oxidoreductase; K06151 gluconate 2-dehydrogenase alpha chain [EC:1.1.99.3] | ec:1.1.99.3 |
| bur:Bcep18194\_B1794 | hypothetical protein; K06152 gluconate 2-dehydrogenase gamma chain [EC:1.1.99.3] | ec:1.1.99.3 |

  
**Neighborhood Representations for "bac:BamMC406\_4126"**  

| ID | Annotation | EC number |
| --- | --- | --- |
| bac:BamMC406\_4116 | TraR/DksA family transcriptional regulator |  |
| bac:BamMC406\_4117 | phosphotransferase domain-containing protein; K02347 DNA polymerase (family X) |  |
| bac:BamMC406\_4118 | alanine dehydrogenase (EC:1.4.1.1); K00259 alanine dehydrogenase [EC:1.4.1.1] | ec:1.4.1.1 |
| bac:BamMC406\_4119 | gluconate 2-dehydrogenase (acceptor) (EC:1.1.99.3); K06152 gluconate 2-dehydrogenase gamma chain [EC:1.1.99.3] | ec:1.1.99.3 |
| bac:BamMC406\_4120 | gluconate 2-dehydrogenase (acceptor) (EC:1.1.99.3); K06151 gluconate 2-dehydrogenase alpha chain [EC:1.1.99.3] | ec:1.1.99.3 |
| bac:BamMC406\_4121 | gluconate 2-dehydrogenase (acceptor) (EC:1.1.99.3) |  |
| bac:BamMC406\_4122 | sodium/hydrogen exchanger; K03455 monovalent cation:H+ antiporter-2, CPA2 family |  |
| bac:BamMC406\_4123 | peptidoglycan glycosyltransferase (EC:2.4.1.129); K03587 cell division protein FtsI (penicillin-binding protein 3) [EC:2.4.1.129] | ec:2.4.1.129 |
| bac:BamMC406\_4124 | hypothetical protein |  |
| bac:BamMC406\_4125 | hypothetical protein |  |
| bac:BamMC406\_4126 | MarR family transcriptional regulator |  |
| bac:BamMC406\_4127 | 4-hydroxyphenylacetate degradation bifunctional isomerase/decarboxylase subunit HpaG1 (EC:4.1.1.68); K05921 5-oxopent-3-ene-1,2,5-tricarboxylate decarboxylase / 2-hydroxyhepta-2,4-diene-1,7-dioate isomerase [EC:4.1.1.68 5.3.3.-] | ec:4.1.1.68 |
| bac:BamMC406\_4128 | 4-hydroxyphenylacetate degradation bifunctional isomerase/decarboxylase subunit HpaG2 (EC:4.1.1.68); K05921 5-oxopent-3-ene-1,2,5-tricarboxylate decarboxylase / 2-hydroxyhepta-2,4-diene-1,7-dioate isomerase [EC:4.1.1.68 5.3.3.-] | ec:4.1.1.68 |
| bac:BamMC406\_4129 | 5-carboxymethyl-2-hydroxymuconate semialdehyde dehydrogenase; K00151 5-carboxymethyl-2-hydroxymuconic-semialdehyde dehydrogenase [EC:1.2.1.60] | ec:1.2.1.60 |
| bac:BamMC406\_4130 | 3,4-dihydroxyphenylacetate 2,3-dioxygenase (EC:1.13.11.15); K00455 3,4-dihydroxyphenylacetate 2,3-dioxygenase [EC:1.13.11.15] | ec:1.13.11.15 |
| bac:BamMC406\_4131 | 5-carboxymethyl-2-hydroxymuconate isomerase; K01826 5-carboxymethyl-2-hydroxymuconate isomerase [EC:5.3.3.10] | ec:5.3.3.10 |
| bac:BamMC406\_4132 | 2-oxo-hepta-3-ene-1,7-dioic acid hydratase (EC:4.1.1.77); K02509 2-oxo-hept-3-ene-1,7-dioate hydratase [EC:4.2.1.-] |  |
| bac:BamMC406\_4133 | 2,4-dihydroxyhept-2-ene-1,7-dioic acid aldolase (EC:4.1.2.20); K02510 4-hydroxy-2-oxoheptanedioate aldolase [EC:4.1.2.52] | ec:4.1.2.52 |
| bac:BamMC406\_4134 | AraC family transcriptional regulator |  |
| bac:BamMC406\_4135 | hypothetical protein |  |
| bac:BamMC406\_4136 | alcohol dehydrogenase |  |

  
**Neighborhood Representations for "bam:Bamb\_3652"**  

| ID | Annotation | EC number |
| --- | --- | --- |
| bam:Bamb\_3642 | phosphotransferase domain-containing protein; K02347 DNA polymerase (family X) |  |
| bam:Bamb\_3643 | alanine dehydrogenase; K00259 alanine dehydrogenase [EC:1.4.1.1] | ec:1.4.1.1 |
| bam:Bamb\_3644 | hypothetical protein |  |
| bam:Bamb\_3645 | hypothetical protein; K06152 gluconate 2-dehydrogenase gamma chain [EC:1.1.99.3] | ec:1.1.99.3 |
| bam:Bamb\_3646 | glucose-methanol-choline oxidoreductase; K06151 gluconate 2-dehydrogenase alpha chain [EC:1.1.99.3] | ec:1.1.99.3 |
| bam:Bamb\_3647 | cytochrome c, class I |  |
| bam:Bamb\_3648 | sodium/hydrogen exchanger; K03455 monovalent cation:H+ antiporter-2, CPA2 family |  |
| bam:Bamb\_3649 | peptidoglycan glycosyltransferase (EC:2.4.1.129); K03587 cell division protein FtsI (penicillin-binding protein 3) [EC:2.4.1.129] | ec:2.4.1.129 |
| bam:Bamb\_3650 | hypothetical protein |  |
| bam:Bamb\_3651 | hypothetical protein |  |
| bam:Bamb\_3652 | MarR family transcriptional regulator |  |
| bam:Bamb\_3653 | 4-hydroxyphenylacetate degradation bifunctional isomerase/decarboxylase subunit HpaG1 (EC:5.3.3.10); K05921 5-oxopent-3-ene-1,2,5-tricarboxylate decarboxylase / 2-hydroxyhepta-2,4-diene-1,7-dioate isomerase [EC:4.1.1.68 5.3.3.-] | ec:4.1.1.68 |
| bam:Bamb\_3654 | 4-hydroxyphenylacetate degradation bifunctional isomerase/decarboxylase subunit HpaG2 (EC:4.1.1.68); K05921 5-oxopent-3-ene-1,2,5-tricarboxylate decarboxylase / 2-hydroxyhepta-2,4-diene-1,7-dioate isomerase [EC:4.1.1.68 5.3.3.-] | ec:4.1.1.68 |
| bam:Bamb\_3655 | 5-carboxymethyl-2-hydroxymuconate semialdehyde dehydrogenase (EC:1.2.1.8); K00151 5-carboxymethyl-2-hydroxymuconic-semialdehyde dehydrogenase [EC:1.2.1.60] | ec:1.2.1.60 |
| bam:Bamb\_3656 | 3,4-dihydroxyphenylacetate 2,3-dioxygenase (EC:1.13.11.15); K00455 3,4-dihydroxyphenylacetate 2,3-dioxygenase [EC:1.13.11.15] | ec:1.13.11.15 |
| bam:Bamb\_3657 | 5-carboxymethyl-2-hydroxymuconate isomerase; K01826 5-carboxymethyl-2-hydroxymuconate isomerase [EC:5.3.3.10] | ec:5.3.3.10 |
| bam:Bamb\_3658 | 2-oxo-hepta-3-ene-1,7-dioic acid hydratase (EC:4.1.1.77); K02509 2-oxo-hept-3-ene-1,7-dioate hydratase [EC:4.2.1.-] |  |
| bam:Bamb\_3659 | 2,4-dihydroxyhept-2-ene-1,7-dioic acid aldolase (EC:4.1.2.20); K02510 4-hydroxy-2-oxoheptanedioate aldolase [EC:4.1.2.52] | ec:4.1.2.52 |
| bam:Bamb\_3660 | AraC family transcriptional regulator |  |
| bam:Bamb\_3661 | hypothetical protein |  |
| bam:Bamb\_3662 | alcohol dehydrogenase |  |

  
**Neighborhood Representations for "bgl:bglu\_2g17970"**  

| ID | Annotation | EC number |
| --- | --- | --- |
| bgl:bglu\_2g17870 | pseudogene |  |
| bgl:bglu\_2g17880 | outer membrane hemolysin activator protein; K07326 hemolysin activation/secretion protein |  |
| bgl:bglu\_2g17890 | hypothetical protein |  |
| bgl:bglu\_2g17900 | pseudogene |  |
| bgl:bglu\_2g17910 | pseudogene |  |
| bgl:bglu\_2g17920 | lipoprotein |  |
| bgl:bglu\_2g17930 | hypothetical protein |  |
| bgl:bglu\_2g17940 | filamentous hemagglutinin; K15125 filamentous hemagglutinin |  |
| bgl:bglu\_2g17950 | pseudogene |  |
| bgl:bglu\_2g17960 | MOSC domain-containing protein; K07140 |  |
| bgl:bglu\_2g17970 | MarR family transcriptional regulator |  |
| bgl:bglu\_2g17980 | acyl-CoA dehydrogenase |  |
| bgl:bglu\_2g17990 | hypothetical protein |  |
| bgl:bglu\_2g18000 | winged helix family two component response transcriptional regulator |  |
| bgl:bglu\_2g18010 | Neutral trehalase; K01194 alpha,alpha-trehalase [EC:3.2.1.28] | ec:3.2.1.28 |
| bgl:bglu\_2g18020 | EmrB/QacA family drug resistance transporter |  |
| bgl:bglu\_2g18030 | hypothetical protein; K09927 hypothetical protein |  |
| bgl:bglu\_2g18040 | hypothetical protein |  |
| bgl:bglu\_2g18050 | acetylornithine deacetylase/succinyl-diaminopimelate desuccinylase-like deacylase |  |
| bgl:bglu\_2g18060 | transcriptional activator FtrA; K13633 AraC family transcriptional regulator, transcriptional activator FtrA |  |
| bgl:bglu\_2g18070 | Rhodanese-related sulfurtransferase |  |

  
**Neighborhood Representations for "bte:BTH\_II1736"**  

| ID | Annotation | EC number |
| --- | --- | --- |
| bte:BTH\_II1726 | cobalamin/Fe3+-siderophores ABC transporter ATPase; K02011 iron(III) transport system permease protein |  |
| bte:BTH\_II1727 | ABC transporter ATP-binding protein; K02010 iron(III) transport system ATP-binding protein [EC:3.6.3.30] | ec:3.6.3.30 |
| bte:BTH\_II1728 | porin |  |
| bte:BTH\_II1729 | hpaI; 2,4-dihydroxyhept-2-ene-1,7-dioic acid aldolase (EC:4.1.2.-); K02510 4-hydroxy-2-oxoheptanedioate aldolase [EC:4.1.2.52] | ec:4.1.2.52 |
| bte:BTH\_II1730 | hpaH; 2-oxo-hepta-3-ene-1,7-dioic acid hydratase (EC:4.2.-.-); K02509 2-oxo-hept-3-ene-1,7-dioate hydratase [EC:4.2.1.-] |  |
| bte:BTH\_II1731 | hpcD; 5-carboxymethyl-2-hydroxymuconate delta-isomerase (EC:5.3.3.10); K01826 5-carboxymethyl-2-hydroxymuconate isomerase [EC:5.3.3.10] | ec:5.3.3.10 |
| bte:BTH\_II1732 | hpaD; 3,4-dihydroxyphenylacetate 2,3-dioxygenase (EC:1.13.11.15); K00455 3,4-dihydroxyphenylacetate 2,3-dioxygenase [EC:1.13.11.15] | ec:1.13.11.15 |
| bte:BTH\_II1733 | hpaE; 5-carboxymethyl-2-hydroxymuconate semialdehyde dehydrogenase (EC:1.2.1.60); K00151 5-carboxymethyl-2-hydroxymuconic-semialdehyde dehydrogenase [EC:1.2.1.60] | ec:1.2.1.60 |
| bte:BTH\_II1734 | hpaG-1; 4-hydroxyphenylacetate degradation bifunctional isomerase/decarboxylase, C-terminal subunit (EC:4.1.1.68 5.3.3.10); K05921 5-oxopent-3-ene-1,2,5-tricarboxylate decarboxylase / 2-hydroxyhepta-2,4-diene-1,7-dioate isomerase [EC:4.1.1.68 5.3.3.-] | ec:4.1.1.68 |
| bte:BTH\_II1735 | hpaG-2; 4-hydroxyphenylacetate degradation bifunctional isomerase/decarboxylase, N-terminal subunit (EC:4.1.1.68 5.3.3.10); K05921 5-oxopent-3-ene-1,2,5-tricarboxylate decarboxylase / 2-hydroxyhepta-2,4-diene-1,7-dioate isomerase [EC:4.1.1.68 5.3.3.-] | ec:4.1.1.68 |
| bte:BTH\_II1736 | hpaR; homoprotocatechuate degradation operon regulator HpaR |  |
| bte:BTH\_II1737 | hypothetical protein |  |
| bte:BTH\_II1738 | MipA family MltA-interacting protein |  |
| bte:BTH\_II1739 | DNA-binding response regulator; K02483 two-component system, OmpR family, response regulator |  |
| bte:BTH\_II1740 | sensor histidine kinase; K02484 two-component system, OmpR family, sensor kinase [EC:2.7.13.3] | ec:2.7.13.3 |
| bte:BTH\_II1741 | monoxygenase |  |
| bte:BTH\_II1742 | acyl-CoA dehydrogenase |  |
| bte:BTH\_II1743 | hypothetical protein |  |
| bte:BTH\_II1744 | hypothetical protein |  |
| bte:BTH\_II1745 | AraC family transcriptional regulator |  |
| bte:BTH\_II1746 | isoquinoline 1-oxidoreductase subunit alpha; K07302 isoquinoline 1-oxidoreductase, alpha subunit [EC:1.3.99.16] | ec:1.3.99.16 |

  
**Neighborhood Representations for "bmj:BMULJ\_04131"**  

| ID | Annotation | EC number |
| --- | --- | --- |
| bmj:BMULJ\_04121 | dpx; family X DNA polymerase; K02347 DNA polymerase (family X) |  |
| bmj:BMULJ\_04122 | ald; alanine dehydrogenase (EC:1.4.1.1); K00259 alanine dehydrogenase [EC:1.4.1.1] | ec:1.4.1.1 |
| bmj:BMULJ\_04123 | hypothetical protein |  |
| bmj:BMULJ\_04124 | gluconate 2-dehydrogenase gamma chain (EC:1.1.99.3); K06152 gluconate 2-dehydrogenase gamma chain [EC:1.1.99.3] | ec:1.1.99.3 |
| bmj:BMULJ\_04125 | gluconate 2-dehydrogenase alpha chain (EC:1.1.99.3); K06151 gluconate 2-dehydrogenase alpha chain [EC:1.1.99.3] | ec:1.1.99.3 |
| bmj:BMULJ\_04126 | cccA; gluconate dehydrogenase cytochrome c subunit |  |
| bmj:BMULJ\_04127 | CPA2 family monovalent cation:H+ antiporter-2; K03455 monovalent cation:H+ antiporter-2, CPA2 family |  |
| bmj:BMULJ\_04128 | ftsI; cell division protein; K03587 cell division protein FtsI (penicillin-binding protein 3) [EC:2.4.1.129] | ec:2.4.1.129 |
| bmj:BMULJ\_04129 | hypothetical protein |  |
| bmj:BMULJ\_04130 | nucleoside-diphosphate-sugar epimerase |  |
| bmj:BMULJ\_04131 | hpaR; MarR family transcriptional regulator |  |
| bmj:BMULJ\_04132 | hpaG; 5-carboxymethyl-2-hydroxymuconate isomerase (EC:5.3.3.10); K05921 5-oxopent-3-ene-1,2,5-tricarboxylate decarboxylase / 2-hydroxyhepta-2,4-diene-1,7-dioate isomerase [EC:4.1.1.68 5.3.3.-] | ec:4.1.1.68 |
| bmj:BMULJ\_04133 | 5-oxopent-3-ene-1,2,5-tricarboxylate decarboxylase (EC:4.1.1.68); K05921 5-oxopent-3-ene-1,2,5-tricarboxylate decarboxylase / 2-hydroxyhepta-2,4-diene-1,7-dioate isomerase [EC:4.1.1.68 5.3.3.-] | ec:4.1.1.68 |
| bmj:BMULJ\_04134 | hpaE; 5-carboxymethyl-2-hydroxymuconic-semialdehyde dehydrogenase (EC:1.2.1.60); K00151 5-carboxymethyl-2-hydroxymuconic-semialdehyde dehydrogenase [EC:1.2.1.60] | ec:1.2.1.60 |
| bmj:BMULJ\_04135 | hpaD; 3,4-dihydroxyphenylacetate 2,3-dioxygenase (EC:1.13.11.15); K00455 3,4-dihydroxyphenylacetate 2,3-dioxygenase [EC:1.13.11.15] | ec:1.13.11.15 |
| bmj:BMULJ\_04136 | hpaF; 5-carboxymethyl-2-hydroxymuconate isomerase (EC:5.3.3.10); K01826 5-carboxymethyl-2-hydroxymuconate isomerase [EC:5.3.3.10] | ec:5.3.3.10 |
| bmj:BMULJ\_04137 | hpaH; 2-oxo-hept-3-ene-1,7-dioate hydratase; K02509 2-oxo-hept-3-ene-1,7-dioate hydratase [EC:4.2.1.-] |  |
| bmj:BMULJ\_04138 | hpaI; 2,4-dihydroxyhept-2-ene-1,7-dioic acid aldolase; K02510 4-hydroxy-2-oxoheptanedioate aldolase [EC:4.1.2.52] | ec:4.1.2.52 |
| bmj:BMULJ\_04139 | periplasmic molybdate-binding protein |  |
| bmj:BMULJ\_04140 | AraC family transcriptional regulator |  |
| bmj:BMULJ\_04141 | hypothetical protein |  |

  
**Neighborhood Representations for "bmu:Bmul\_4375"**  

| ID | Annotation | EC number |
| --- | --- | --- |
| bmu:Bmul\_4365 | hypothetical protein |  |
| bmu:Bmul\_4366 | AraC family transcriptional regulator |  |
| bmu:Bmul\_4367 | DNA binding domain-containing protein |  |
| bmu:Bmul\_4368 | 2,4-dihydroxyhept-2-ene-1,7-dioic acid aldolase (EC:4.1.2.20); K02510 4-hydroxy-2-oxoheptanedioate aldolase [EC:4.1.2.52] | ec:4.1.2.52 |
| bmu:Bmul\_4369 | 2-oxo-hepta-3-ene-1,7-dioic acid hydratase (EC:4.1.1.77); K02509 2-oxo-hept-3-ene-1,7-dioate hydratase [EC:4.2.1.-] |  |
| bmu:Bmul\_4370 | 5-carboxymethyl-2-hydroxymuconate isomerase; K01826 5-carboxymethyl-2-hydroxymuconate isomerase [EC:5.3.3.10] | ec:5.3.3.10 |
| bmu:Bmul\_4371 | 3,4-dihydroxyphenylacetate 2,3-dioxygenase (EC:1.13.11.15); K00455 3,4-dihydroxyphenylacetate 2,3-dioxygenase [EC:1.13.11.15] | ec:1.13.11.15 |
| bmu:Bmul\_4372 | 5-carboxymethyl-2-hydroxymuconate semialdehyde dehydrogenase; K00151 5-carboxymethyl-2-hydroxymuconic-semialdehyde dehydrogenase [EC:1.2.1.60] | ec:1.2.1.60 |
| bmu:Bmul\_4373 | 4-hydroxyphenylacetate degradation bifunctional isomerase/decarboxylase subunit HpaG2 (EC:5.3.3.10); K05921 5-oxopent-3-ene-1,2,5-tricarboxylate decarboxylase / 2-hydroxyhepta-2,4-diene-1,7-dioate isomerase [EC:4.1.1.68 5.3.3.-] | ec:4.1.1.68 |
| bmu:Bmul\_4374 | 4-hydroxyphenylacetate degradation bifunctional isomerase/decarboxylase subunit HpaG1 (EC:4.1.1.68); K05921 5-oxopent-3-ene-1,2,5-tricarboxylate decarboxylase / 2-hydroxyhepta-2,4-diene-1,7-dioate isomerase [EC:4.1.1.68 5.3.3.-] | ec:4.1.1.68 |
| bmu:Bmul\_4375 | MarR family transcriptional regulator |  |
| bmu:Bmul\_4376 | hypothetical protein |  |
| bmu:Bmul\_4377 | hypothetical protein |  |
| bmu:Bmul\_4378 | peptidoglycan glycosyltransferase (EC:2.4.1.129); K03587 cell division protein FtsI (penicillin-binding protein 3) [EC:2.4.1.129] | ec:2.4.1.129 |
| bmu:Bmul\_4379 | sodium/hydrogen exchanger; K03455 monovalent cation:H+ antiporter-2, CPA2 family |  |
| bmu:Bmul\_4380 | gluconate 2-dehydrogenase (acceptor) (EC:1.1.99.3) |  |
| bmu:Bmul\_4381 | gluconate 2-dehydrogenase (acceptor) (EC:1.1.99.3); K06151 gluconate 2-dehydrogenase alpha chain [EC:1.1.99.3] | ec:1.1.99.3 |
| bmu:Bmul\_4382 | gluconate 2-dehydrogenase (acceptor) (EC:1.1.99.3); K06152 gluconate 2-dehydrogenase gamma chain [EC:1.1.99.3] | ec:1.1.99.3 |
| bmu:Bmul\_4383 | hypothetical protein |  |
| bmu:Bmul\_4384 | hypothetical protein |  |
| bmu:Bmul\_4385 | alanine dehydrogenase; K00259 alanine dehydrogenase [EC:1.4.1.1] | ec:1.4.1.1 |

  
**Neighborhood Representations for "bpl:BURPS1106A\_A0937"**  

| ID | Annotation | EC number |
| --- | --- | --- |
| bpl:BURPS1106A\_A0927 | hypothetical protein |  |
| bpl:BURPS1106A\_A0928 | acyl-CoA dehydrogenase domain-containing protein |  |
| bpl:BURPS1106A\_A0930 | acyl-CoA dehydrogenase domain-containing protein |  |
| bpl:BURPS1106A\_A0929 | hypothetical protein |  |
| bpl:BURPS1106A\_A0931 | monoxygenase |  |
| bpl:BURPS1106A\_A0932 | hypothetical protein |  |
| bpl:BURPS1106A\_A0933 | sensor histidine kinase (EC:2.7.3.-); K02484 two-component system, OmpR family, sensor kinase [EC:2.7.13.3] | ec:2.7.13.3 |
| bpl:BURPS1106A\_A0934 | DNA-binding response regulator; K02483 two-component system, OmpR family, response regulator |  |
| bpl:BURPS1106A\_A0935 | mipA family protein |  |
| bpl:BURPS1106A\_A0936 | NAD-dependent epimerase/dehydratase family protein |  |
| bpl:BURPS1106A\_A0937 | hpaR; homoprotocatechuate degradation operon regulator, HpaR |  |
| bpl:BURPS1106A\_A0938 | 4-hydroxyphenylacetate degradation bifunctional isomerase/decarboxylase, N-terminal subunit (EC:4.1.1.68 5.3.3.10); K05921 5-oxopent-3-ene-1,2,5-tricarboxylate decarboxylase / 2-hydroxyhepta-2,4-diene-1,7-dioate isomerase [EC:4.1.1.68 5.3.3.-] | ec:4.1.1.68 |
| bpl:BURPS1106A\_A0939 | 4-hydroxyphenylacetate degradation bifunctional isomerase/decarboxylase, C-terminal subunit (EC:4.1.1.68 5.3.3.10); K05921 5-oxopent-3-ene-1,2,5-tricarboxylate decarboxylase / 2-hydroxyhepta-2,4-diene-1,7-dioate isomerase [EC:4.1.1.68 5.3.3.-] | ec:4.1.1.68 |
| bpl:BURPS1106A\_A0940 | hpaE; 5-carboxymethyl-2-hydroxymuconate semialdehyde dehydrogenase (EC:1.2.1.60); K00151 5-carboxymethyl-2-hydroxymuconic-semialdehyde dehydrogenase [EC:1.2.1.60] | ec:1.2.1.60 |
| bpl:BURPS1106A\_A0941 | hpaD; 3,4-dihydroxyphenylacetate 2,3-dioxygenase (EC:1.13.11.15); K00455 3,4-dihydroxyphenylacetate 2,3-dioxygenase [EC:1.13.11.15] | ec:1.13.11.15 |
| bpl:BURPS1106A\_A0942 | hpaF; 5-carboxymethyl-2-hydroxymuconate delta isomerase (EC:5.3.3.10); K01826 5-carboxymethyl-2-hydroxymuconate isomerase [EC:5.3.3.10] | ec:5.3.3.10 |
| bpl:BURPS1106A\_A0943 | hpaH; 2-oxo-hepta-3-ene-1,7-dioic acid hydratase (EC:4.2.-.-); K02509 2-oxo-hept-3-ene-1,7-dioate hydratase [EC:4.2.1.-] |  |
| bpl:BURPS1106A\_A0945 | hpaI; 2,4-dihydroxyhept-2-ene-1,7-dioic acid aldolase (EC:4.1.2.-); K02510 4-hydroxy-2-oxoheptanedioate aldolase [EC:4.1.2.52] | ec:4.1.2.52 |
| bpl:BURPS1106A\_A0944 | AraC family transcriptional regulator |  |
| bpl:BURPS1106A\_A0946 | zinc-binding dehydrogenase family oxidoreductase |  |
| bpl:BURPS1106A\_A0947 | outer membrane porin |  |

  
**Neighborhood Representations for "bpm:BURPS1710b\_A2262"**  

| ID | Annotation | EC number |
| --- | --- | --- |
| bpm:BURPS1710b\_A2252 | hypothetical protein |  |
| bpm:BURPS1710b\_A2253 | hypothetical protein |  |
| bpm:BURPS1710b\_A2254 | acyl-CoA dehydrogenase |  |
| bpm:BURPS1710b\_A2255 | acyl-CoA dehydrogenase |  |
| bpm:BURPS1710b\_A2256 | hypothetical protein |  |
| bpm:BURPS1710b\_A2257 | monoxygenase |  |
| bpm:BURPS1710b\_A2258 | rpeA; sensor kinase protein; K02484 two-component system, OmpR family, sensor kinase [EC:2.7.13.3] | ec:2.7.13.3 |
| bpm:BURPS1710b\_A2259 | response regulator protein; K02483 two-component system, OmpR family, response regulator |  |
| bpm:BURPS1710b\_A2260 | MipA family MltA-interacting protein |  |
| bpm:BURPS1710b\_A2261 | NAD-dependent epimerase/dehydratase |  |
| bpm:BURPS1710b\_A2262 | hpaR; homoprotocatechuate degradation operon regulator HpaR |  |
| bpm:BURPS1710b\_A2263 | hpaG; 4-hydroxyphenylacetate degradation bifunctional isomerase/decarboxylase, N-terminal subunit; K05921 5-oxopent-3-ene-1,2,5-tricarboxylate decarboxylase / 2-hydroxyhepta-2,4-diene-1,7-dioate isomerase [EC:4.1.1.68 5.3.3.-] | ec:4.1.1.68 |
| bpm:BURPS1710b\_A2264 | hpaG; 4-hydroxyphenylacetate degradation bifunctional isomerase/decarboxylase, C-terminal subunit; K05921 5-oxopent-3-ene-1,2,5-tricarboxylate decarboxylase / 2-hydroxyhepta-2,4-diene-1,7-dioate isomerase [EC:4.1.1.68 5.3.3.-] | ec:4.1.1.68 |
| bpm:BURPS1710b\_A2265 | hpaE; 5-carboxymethyl-2-hydroxymuconate semialdehyde dehydrogenase; K00151 5-carboxymethyl-2-hydroxymuconic-semialdehyde dehydrogenase [EC:1.2.1.60] | ec:1.2.1.60 |
| bpm:BURPS1710b\_A2266 | hypothetical protein |  |
| bpm:BURPS1710b\_A2267 | hpaD; 3,4-dihydroxyphenylacetate 2,3-dioxygenase; K00455 3,4-dihydroxyphenylacetate 2,3-dioxygenase [EC:1.13.11.15] | ec:1.13.11.15 |
| bpm:BURPS1710b\_A2268 | hpcD; 5-carboxymethyl-2-hydroxymuconate delta-isomerase; K01826 5-carboxymethyl-2-hydroxymuconate isomerase [EC:5.3.3.10] | ec:5.3.3.10 |
| bpm:BURPS1710b\_A2269 | hpaH; 2-oxo-hepta-3-ene-1,7-dioic acid hydratase; K02509 2-oxo-hept-3-ene-1,7-dioate hydratase [EC:4.2.1.-] |  |
| bpm:BURPS1710b\_A2271 | AraC family transcriptional regulator |  |
| bpm:BURPS1710b\_A2270 | hpaI; 2,4-dihydroxyhept-2-ene-1,7-dioic acid aldolase; K02510 4-hydroxy-2-oxoheptanedioate aldolase [EC:4.1.2.52] | ec:4.1.2.52 |
| bpm:BURPS1710b\_A2272 | zinc-binding dehydrogenase family oxidoreductase |  |

  
**Neighborhood Representations for "bps:BPSS0691"**  

| ID | Annotation | EC number |
| --- | --- | --- |
| bps:BPSS0681 | AraC family transcriptional regulator |  |
| bps:BPSS0682 | hypothetical protein |  |
| bps:BPSS0683 | hypothetical protein |  |
| bps:BPSS0684 | hypothetical protein |  |
| bps:BPSS0685 | hypothetical protein |  |
| bps:BPSS0686 | hypothetical protein |  |
| bps:BPSS0687 | sensor kinase (EC:2.7.3.-); K02484 two-component system, OmpR family, sensor kinase [EC:2.7.13.3] | ec:2.7.13.3 |
| bps:BPSS0688 | response regulator protein; K02483 two-component system, OmpR family, response regulator |  |
| bps:BPSS0689 | hypothetical protein |  |
| bps:BPSS0690 | hypothetical protein |  |
| bps:BPSS0691 | MarR family transcriptional regulator |  |
| bps:BPSS0692 | fumarylacetoacetate (FAA) hydrolase; K05921 5-oxopent-3-ene-1,2,5-tricarboxylate decarboxylase / 2-hydroxyhepta-2,4-diene-1,7-dioate isomerase [EC:4.1.1.68 5.3.3.-] | ec:4.1.1.68 |
| bps:BPSS0693 | fumarylacetoacetate (FAA) hydrolase; K05921 5-oxopent-3-ene-1,2,5-tricarboxylate decarboxylase / 2-hydroxyhepta-2,4-diene-1,7-dioate isomerase [EC:4.1.1.68 5.3.3.-] | ec:4.1.1.68 |
| bps:BPSS0694 | hpcC; 5-carboxymethyl-2-hydroxymuconate semialdehyde dehydrogenase (EC:1.2.1.-); K00151 5-carboxymethyl-2-hydroxymuconic-semialdehyde dehydrogenase [EC:1.2.1.60] | ec:1.2.1.60 |
| bps:BPSS0695 | hpcB; 3,4-dihydroxyphenylacetate 2,3-dioxygenase (EC:1.13.11.15); K00455 3,4-dihydroxyphenylacetate 2,3-dioxygenase [EC:1.13.11.15] | ec:1.13.11.15 |
| bps:BPSS0696 | hpcD; 5-carboxymethyl-2-hydroxymuconate delta-isomerase (EC:5.3.3.10); K01826 5-carboxymethyl-2-hydroxymuconate isomerase [EC:5.3.3.10] | ec:5.3.3.10 |
| bps:BPSS0697 | hpcG; 2-oxo-hepta-3-ene-1,7-dioic acid hydratase (EC:4.2.-.-); K02509 2-oxo-hept-3-ene-1,7-dioate hydratase [EC:4.2.1.-] |  |
| bps:BPSS0698 | hpcH; 2,4-dihydroxyhept-2-ene-1,7-dioic acid aldolase (EC:4.1.2.-); K02510 4-hydroxy-2-oxoheptanedioate aldolase [EC:4.1.2.52] | ec:4.1.2.52 |
| bps:BPSS0699 | AraC family transcriptional regulator |  |
| bps:BPSS0700 | zinc-binding dehydrogenase |  |
| bps:BPSS0701 | outer membrane porin protein |  |

  
**Neighborhood Representations for "bma:BMAA1141"**  

| ID | Annotation | EC number |
| --- | --- | --- |
| bma:BMAA1131 | porin |  |
| bma:BMAA1132 | zinc-binding dehydrogenase family oxidoreductase |  |
| bma:BMAA1133 | AraC family transcriptional regulator |  |
| bma:BMAA1134 | pseudogene |  |
| bma:BMAA1135 | hpcG; 2-oxo-hepta-3-ene-1,7-dioic acid hydratase (EC:4.2.-.-); K02509 2-oxo-hept-3-ene-1,7-dioate hydratase [EC:4.2.1.-] |  |
| bma:BMAA1136 | hpcD; 5-carboxymethyl-2-hydroxymuconate delta-isomerase (EC:5.3.3.10); K01826 5-carboxymethyl-2-hydroxymuconate isomerase [EC:5.3.3.10] | ec:5.3.3.10 |
| bma:BMAA1137 | hpcB; 3,4-dihydroxyphenylacetate 2,3-dioxygenase (EC:1.13.11.15); K00455 3,4-dihydroxyphenylacetate 2,3-dioxygenase [EC:1.13.11.15] | ec:1.13.11.15 |
| bma:BMAA1138 | hpcE; 5-carboxy-2-hydroxymuconate semialdehyde dehydrogenase (EC:1.2.1.-); K00151 5-carboxymethyl-2-hydroxymuconic-semialdehyde dehydrogenase [EC:1.2.1.60] | ec:1.2.1.60 |
| bma:BMAA1139 | 5-carboxymethyl-2-oxo-hex-3-ene-1,7-dioate decarboxylase; K05921 5-oxopent-3-ene-1,2,5-tricarboxylate decarboxylase / 2-hydroxyhepta-2,4-diene-1,7-dioate isomerase [EC:4.1.1.68 5.3.3.-] | ec:4.1.1.68 |
| bma:BMAA1140 | 2-hydroxyhepta-2,4-diene-1,7-dioate isomerase; K05921 5-oxopent-3-ene-1,2,5-tricarboxylate decarboxylase / 2-hydroxyhepta-2,4-diene-1,7-dioate isomerase [EC:4.1.1.68 5.3.3.-] | ec:4.1.1.68 |
| bma:BMAA1141 | hpaR; homoprotocatechuate degradative operon repressor |  |
| bma:BMAA1142 | hypothetical protein |  |
| bma:BMAA1143 | hypothetical protein |  |
| bma:BMAA1144 | DNA-binding response regulator; K02483 two-component system, OmpR family, response regulator |  |
| bma:BMAA1145 | sensor histidine kinase; K02484 two-component system, OmpR family, sensor kinase [EC:2.7.13.3] | ec:2.7.13.3 |
| bma:BMAA1146 | monoxygenase |  |
| bma:BMAA1147 | acyl-CoA dehydrogenase |  |
| bma:BMAA1148 | acyl-CoA dehydrogenase |  |
| bma:BMAA1149 | hypothetical protein |  |
| bma:BMAA1150 | hypothetical protein |  |
| bma:BMAA1151 | hypothetical protein |  |

  
**Neighborhood Representations for "bml:BMA10229\_0099"**  

| ID | Annotation | EC number |
| --- | --- | --- |
| bml:BMA10229\_0089 | hypothetical protein |  |
| bml:BMA10229\_0090 | hypothetical protein |  |
| bml:BMA10229\_0091 | acyl-CoA dehydrogenase |  |
| bml:BMA10229\_0092 | acyl-CoA dehydrogenase |  |
| bml:BMA10229\_0093 | monoxygenase |  |
| bml:BMA10229\_0094 | hypothetical protein |  |
| bml:BMA10229\_0095 | sensor histidine kinase; K02484 two-component system, OmpR family, sensor kinase [EC:2.7.13.3] | ec:2.7.13.3 |
| bml:BMA10229\_0096 | DNA-binding response regulator; K02483 two-component system, OmpR family, response regulator |  |
| bml:BMA10229\_0097 | hypothetical protein |  |
| bml:BMA10229\_0098 | NAD-dependent epimerase/dehydratase |  |
| bml:BMA10229\_0099 | hpaR; homoprotocatechuate degradative operon repressor |  |
| bml:BMA10229\_0100 | 4-hydroxyphenylacetate degradation bifunctional isomerase/decarboxylase, N-terminal subunit; K05921 5-oxopent-3-ene-1,2,5-tricarboxylate decarboxylase / 2-hydroxyhepta-2,4-diene-1,7-dioate isomerase [EC:4.1.1.68 5.3.3.-] | ec:4.1.1.68 |
| bml:BMA10229\_0101 | 4-hydroxyphenylacetate degradation bifunctional isomerase/decarboxylase, C-terminal subunit; K05921 5-oxopent-3-ene-1,2,5-tricarboxylate decarboxylase / 2-hydroxyhepta-2,4-diene-1,7-dioate isomerase [EC:4.1.1.68 5.3.3.-] | ec:4.1.1.68 |
| bml:BMA10229\_0102 | hpaE; 5-carboxymethyl-2-hydroxymuconate semialdehyde dehydrogenase; K00151 5-carboxymethyl-2-hydroxymuconic-semialdehyde dehydrogenase [EC:1.2.1.60] | ec:1.2.1.60 |
| bml:BMA10229\_0103 | hpaD; 3,4-dihydroxyphenylacetate 2,3-dioxygenase; K00455 3,4-dihydroxyphenylacetate 2,3-dioxygenase [EC:1.13.11.15] | ec:1.13.11.15 |
| bml:BMA10229\_0104 | hpaF; 5-carboxymethyl-2-hydroxymuconate delta-isomerase; K01826 5-carboxymethyl-2-hydroxymuconate isomerase [EC:5.3.3.10] | ec:5.3.3.10 |
| bml:BMA10229\_0105 | hpaH; 2-oxo-hepta-3-ene-1,7-dioic acid hydratase; K02509 2-oxo-hept-3-ene-1,7-dioate hydratase [EC:4.2.1.-] |  |
| bml:BMA10229\_0106 | AraC family transcriptional regulator |  |
| bml:BMA10229\_0107 | hypothetical protein |  |
| bml:BMA10229\_0108 | zinc-binding dehydrogenase family oxidoreductase |  |
| bml:BMA10229\_0109 | porin |  |

  
**Neighborhood Representations for "bmn:BMA10247\_A1514"**  

| ID | Annotation | EC number |
| --- | --- | --- |
| bmn:BMA10247\_A1504 | ABC transporter ATP-binding protein; K02010 iron(III) transport system ATP-binding protein [EC:3.6.3.30] | ec:3.6.3.30 |
| bmn:BMA10247\_A1505 | putative outer membrane porin |  |
| bmn:BMA10247\_A1506 | zinc-binding dehydrogenase family oxidoreductase |  |
| bmn:BMA10247\_A1507 | pseudogene |  |
| bmn:BMA10247\_A1508 | hpaH; 2-oxo-hepta-3-ene-1,7-dioic acid hydratase (EC:4.2.-.-); K02509 2-oxo-hept-3-ene-1,7-dioate hydratase [EC:4.2.1.-] |  |
| bmn:BMA10247\_A1509 | hpaF; 5-carboxymethyl-2-hydroxymuconate delta isomerase (EC:5.3.3.10); K01826 5-carboxymethyl-2-hydroxymuconate isomerase [EC:5.3.3.10] | ec:5.3.3.10 |
| bmn:BMA10247\_A1510 | hpaD; 3,4-dihydroxyphenylacetate 2,3-dioxygenase (EC:1.13.11.15); K00455 3,4-dihydroxyphenylacetate 2,3-dioxygenase [EC:1.13.11.15] | ec:1.13.11.15 |
| bmn:BMA10247\_A1511 | hpaE; 5-carboxymethyl-2-hydroxymuconate semialdehyde dehydrogenase (EC:1.2.1.60); K00151 5-carboxymethyl-2-hydroxymuconic-semialdehyde dehydrogenase [EC:1.2.1.60] | ec:1.2.1.60 |
| bmn:BMA10247\_A1512 | 4-hydroxyphenylacetate degradation bifunctional isomerase/decarboxylase, C-terminal subunit (EC:4.1.1.68 5.3.3.10); K05921 5-oxopent-3-ene-1,2,5-tricarboxylate decarboxylase / 2-hydroxyhepta-2,4-diene-1,7-dioate isomerase [EC:4.1.1.68 5.3.3.-] | ec:4.1.1.68 |
| bmn:BMA10247\_A1513 | 4-hydroxyphenylacetate degradation bifunctional isomerase/decarboxylase, N-terminal subunit (EC:4.1.1.68 5.3.3.10); K05921 5-oxopent-3-ene-1,2,5-tricarboxylate decarboxylase / 2-hydroxyhepta-2,4-diene-1,7-dioate isomerase [EC:4.1.1.68 5.3.3.-] | ec:4.1.1.68 |
| bmn:BMA10247\_A1514 | hpaR; homoprotocatechuate degradative operon repressor |  |
| bmn:BMA10247\_A1515 | NAD-dependent epimerase/dehydratase family protein |  |
| bmn:BMA10247\_A1516 | mipA family protein |  |
| bmn:BMA10247\_A1517 | DNA-binding response regulator; K02483 two-component system, OmpR family, response regulator |  |
| bmn:BMA10247\_A1518 | sensor histidine kinase; K02484 two-component system, OmpR family, sensor kinase [EC:2.7.13.3] | ec:2.7.13.3 |
| bmn:BMA10247\_A1519 | pseudogene |  |
| bmn:BMA10247\_A1520 | monooxygenase |  |
| bmn:BMA10247\_A1522 | hypothetical protein |  |
| bmn:BMA10247\_A1521 | acyl-CoA dehydrogenase domain-containing protein |  |
| bmn:BMA10247\_A1523 | acyl-CoA dehydrogenase domain-containing protein |  |
| bmn:BMA10247\_A1524 | hypothetical protein |  |

  
**Neighborhood Representations for "bpd:BURPS668\_A1022"**  

| ID | Annotation | EC number |
| --- | --- | --- |
| bpd:BURPS668\_A1012 | hypothetical protein |  |
| bpd:BURPS668\_A1013 | acyl-CoA dehydrogenase domain-containing protein |  |
| bpd:BURPS668\_A1015 | acyl-CoA dehydrogenase domain-containing protein |  |
| bpd:BURPS668\_A1014 | hypothetical protein |  |
| bpd:BURPS668\_A1016 | monoxygenase |  |
| bpd:BURPS668\_A1017 | hypothetical protein |  |
| bpd:BURPS668\_A1018 | sensor histidine kinase (EC:2.7.3.-); K02484 two-component system, OmpR family, sensor kinase [EC:2.7.13.3] | ec:2.7.13.3 |
| bpd:BURPS668\_A1019 | DNA-binding response regulator; K02483 two-component system, OmpR family, response regulator |  |
| bpd:BURPS668\_A1020 | mipA family protein |  |
| bpd:BURPS668\_A1021 | NAD-dependent epimerase/dehydratase family protein |  |
| bpd:BURPS668\_A1022 | hpaR; homoprotocatechuate degradation operon regulator HpaR |  |
| bpd:BURPS668\_A1023 | 4-hydroxyphenylacetate degradation bifunctional isomerase/decarboxylase, N-terminal subunit (EC:4.1.1.68 5.3.3.10); K05921 5-oxopent-3-ene-1,2,5-tricarboxylate decarboxylase / 2-hydroxyhepta-2,4-diene-1,7-dioate isomerase [EC:4.1.1.68 5.3.3.-] | ec:4.1.1.68 |
| bpd:BURPS668\_A1024 | 4-hydroxyphenylacetate degradation bifunctional isomerase/decarboxylase, C-terminal subunit (EC:4.1.1.68 5.3.3.10); K05921 5-oxopent-3-ene-1,2,5-tricarboxylate decarboxylase / 2-hydroxyhepta-2,4-diene-1,7-dioate isomerase [EC:4.1.1.68 5.3.3.-] | ec:4.1.1.68 |
| bpd:BURPS668\_A1025 | hpaE; 5-carboxymethyl-2-hydroxymuconate semialdehyde dehydrogenase (EC:1.2.1.60); K00151 5-carboxymethyl-2-hydroxymuconic-semialdehyde dehydrogenase [EC:1.2.1.60] | ec:1.2.1.60 |
| bpd:BURPS668\_A1026 | hpaD; 3,4-dihydroxyphenylacetate 2,3-dioxygenase (EC:1.13.11.15); K00455 3,4-dihydroxyphenylacetate 2,3-dioxygenase [EC:1.13.11.15] | ec:1.13.11.15 |
| bpd:BURPS668\_A1027 | hpaF; 5-carboxymethyl-2-hydroxymuconate delta isomerase (EC:5.3.3.10); K01826 5-carboxymethyl-2-hydroxymuconate isomerase [EC:5.3.3.10] | ec:5.3.3.10 |
| bpd:BURPS668\_A1028 | hpaH; 2-oxo-hepta-3-ene-1,7-dioic acid hydratase (EC:4.2.-.-); K02509 2-oxo-hept-3-ene-1,7-dioate hydratase [EC:4.2.1.-] |  |
| bpd:BURPS668\_A1030 | hpaI; 2,4-dihydroxyhept-2-ene-1,7-dioic acid aldolase (EC:4.1.2.-); K02510 4-hydroxy-2-oxoheptanedioate aldolase [EC:4.1.2.52] | ec:4.1.2.52 |
| bpd:BURPS668\_A1029 | AraC family transcriptional regulator |  |
| bpd:BURPS668\_A1031 | zinc-binding dehydrogenase family oxidoreductase |  |
| bpd:BURPS668\_A1032 | outer membrane porin |  |

  
**Neighborhood Representations for "bvi:Bcep1808\_4800"**  

| ID | Annotation | EC number |
| --- | --- | --- |
| bvi:Bcep1808\_4790 | TraR/DksA family transcriptional regulator |  |
| bvi:Bcep1808\_4791 | L-alanine dehydrogenase (EC:1.4.1.1); K00259 alanine dehydrogenase [EC:1.4.1.1] | ec:1.4.1.1 |
| bvi:Bcep1808\_4792 | hypothetical protein |  |
| bvi:Bcep1808\_4793 | hypothetical protein; K06152 gluconate 2-dehydrogenase gamma chain [EC:1.1.99.3] | ec:1.1.99.3 |
| bvi:Bcep1808\_4794 | glucose-methanol-choline oxidoreductase; K06151 gluconate 2-dehydrogenase alpha chain [EC:1.1.99.3] | ec:1.1.99.3 |
| bvi:Bcep1808\_4795 | cytochrome c, class I |  |
| bvi:Bcep1808\_4796 | sodium/hydrogen exchanger; K03455 monovalent cation:H+ antiporter-2, CPA2 family |  |
| bvi:Bcep1808\_4797 | peptidoglycan synthetase FtsI (EC:2.4.1.129); K03587 cell division protein FtsI (penicillin-binding protein 3) [EC:2.4.1.129] | ec:2.4.1.129 |
| bvi:Bcep1808\_4798 | hypothetical protein |  |
| bvi:Bcep1808\_4799 | hypothetical protein |  |
| bvi:Bcep1808\_4800 | MarR family transcriptional regulator |  |
| bvi:Bcep1808\_4801 | 5-carboxymethyl-2-hydroxymuconate delta-isomerase (EC:5.3.3.10); K05921 5-oxopent-3-ene-1,2,5-tricarboxylate decarboxylase / 2-hydroxyhepta-2,4-diene-1,7-dioate isomerase [EC:4.1.1.68 5.3.3.-] | ec:4.1.1.68 |
| bvi:Bcep1808\_4802 | 4-hydroxyphenylacetate degradation bifunctional isomerase/decarboxylase subunit HpaG2 (EC:5.3.3.10); K05921 5-oxopent-3-ene-1,2,5-tricarboxylate decarboxylase / 2-hydroxyhepta-2,4-diene-1,7-dioate isomerase [EC:4.1.1.68 5.3.3.-] | ec:4.1.1.68 |
| bvi:Bcep1808\_4803 | 5-carboxymethyl-2-hydroxymuconate semialdehyde dehydrogenase (EC:1.2.1.8); K00151 5-carboxymethyl-2-hydroxymuconic-semialdehyde dehydrogenase [EC:1.2.1.60] | ec:1.2.1.60 |
| bvi:Bcep1808\_4804 | 3,4-dihydroxyphenylacetate 2,3-dioxygenase (EC:1.13.11.15); K00455 3,4-dihydroxyphenylacetate 2,3-dioxygenase [EC:1.13.11.15] | ec:1.13.11.15 |
| bvi:Bcep1808\_4805 | 5-carboxymethyl-2-hydroxymuconate isomerase; K01826 5-carboxymethyl-2-hydroxymuconate isomerase [EC:5.3.3.10] | ec:5.3.3.10 |
| bvi:Bcep1808\_4806 | 2-oxo-hepta-3-ene-1,7-dioic acid hydratase (EC:4.1.1.77); K02509 2-oxo-hept-3-ene-1,7-dioate hydratase [EC:4.2.1.-] |  |
| bvi:Bcep1808\_4807 | 2,4-dihydroxyhept-2-ene-1,7-dioic acid aldolase (EC:4.1.2.20); K02510 4-hydroxy-2-oxoheptanedioate aldolase [EC:4.1.2.52] | ec:4.1.2.52 |
| bvi:Bcep1808\_4808 | AraC family transcriptional regulator |  |
| bvi:Bcep1808\_4809 | hypothetical protein |  |
| bvi:Bcep1808\_4810 | alcohol dehydrogenase |  |

  
**Neighborhood Representations for "ppg:PputGB1\_3494"**  

| ID | Annotation | EC number |
| --- | --- | --- |
| ppg:PputGB1\_3484 | hypothetical protein |  |
| ppg:PputGB1\_3485 | hypothetical protein |  |
| ppg:PputGB1\_3486 | inosine/uridine-preferring nucleoside hydrolase; K01239 purine nucleosidase [EC:3.2.2.1] | ec:3.2.2.1 |
| ppg:PputGB1\_3487 | D-ribose pyranase; K06726 D-ribose pyranase [EC:5.-.-.-] |  |
| ppg:PputGB1\_3488 | ribokinase; K00852 ribokinase [EC:2.7.1.15] | ec:2.7.1.15 |
| ppg:PputGB1\_3489 | LacI family transcriptional regulator (EC:5.1.1.1); K02529 LacI family transcriptional regulator |  |
| ppg:PputGB1\_3490 | monosaccharide-transporting ATPase (EC:3.6.3.17); K10440 ribose transport system permease protein |  |
| ppg:PputGB1\_3491 | ABC transporter-like protein; K10441 ribose transport system ATP-binding protein [EC:3.6.3.17] | ec:3.6.3.17 |
| ppg:PputGB1\_3492 | monosaccharide-transporting ATPase (EC:3.6.3.17); K10439 ribose transport system substrate-binding protein |  |
| ppg:PputGB1\_3493 | type II L-asparaginase (EC:3.5.1.38); K05597 glutamin-(asparagin-)ase [EC:3.5.1.38] | ec:3.5.1.38 |
| ppg:PputGB1\_3494 | MarR family transcriptional regulator |  |
| ppg:PputGB1\_3495 | AraC family transcriptional regulator; K02508 AraC family transcriptional regulator, 4-hydroxyphenylacetate 3-monooxygenase operon regulatory protein |  |
| ppg:PputGB1\_3496 | 4-hydroxyphenylacetate degradation bifunctional isomerase/decarboxylase subunit HpaG1 (EC:5.3.3.10); K05921 5-oxopent-3-ene-1,2,5-tricarboxylate decarboxylase / 2-hydroxyhepta-2,4-diene-1,7-dioate isomerase [EC:4.1.1.68 5.3.3.-] | ec:4.1.1.68 |
| ppg:PputGB1\_3497 | 4-hydroxyphenylacetate degradation bifunctional isomerase/decarboxylase subunit HpaG2 (EC:4.1.1.68); K05921 5-oxopent-3-ene-1,2,5-tricarboxylate decarboxylase / 2-hydroxyhepta-2,4-diene-1,7-dioate isomerase [EC:4.1.1.68 5.3.3.-] | ec:4.1.1.68 |
| ppg:PputGB1\_3498 | 5-carboxymethyl-2-hydroxymuconate semialdehyde dehydrogenase; K00151 5-carboxymethyl-2-hydroxymuconic-semialdehyde dehydrogenase [EC:1.2.1.60] | ec:1.2.1.60 |
| ppg:PputGB1\_3499 | 3,4-dihydroxyphenylacetate 2,3-dioxygenase (EC:1.13.11.15); K00455 3,4-dihydroxyphenylacetate 2,3-dioxygenase [EC:1.13.11.15] | ec:1.13.11.15 |
| ppg:PputGB1\_3500 | 5-carboxymethyl-2-hydroxymuconate isomerase; K01826 5-carboxymethyl-2-hydroxymuconate isomerase [EC:5.3.3.10] | ec:5.3.3.10 |
| ppg:PputGB1\_3501 | major facilitator superfamily transporter |  |
| ppg:PputGB1\_3502 | 2-oxo-hepta-3-ene-1,7-dioic acid hydratase (EC:4.1.1.77); K02509 2-oxo-hept-3-ene-1,7-dioate hydratase [EC:4.2.1.-] |  |
| ppg:PputGB1\_3503 | 2,4-dihydroxyhept-2-ene-1,7-dioic acid aldolase (EC:4.1.2.20); K02510 4-hydroxy-2-oxoheptanedioate aldolase [EC:4.1.2.52] | ec:4.1.2.52 |
| ppg:PputGB1\_3504 | flavin reductase domain-containing protein |  |

  
**Neighborhood Representations for "pen:PSEEN3090"**  

| ID | Annotation | EC number |
| --- | --- | --- |
| pen:PSEEN3079 | DNase; K06896 |  |
| pen:PSEEN3080 | cardiolipin synthase 2; K06132 putative cardiolipin synthase [EC:2.7.8.-] |  |
| pen:PSEEN3081 | hypothetical protein; K07027 |  |
| pen:PSEEN3082 | hypothetical protein |  |
| pen:PSEEN3084 | carboxylate-amine ligase; K06048 carboxylate-amine ligase [EC:6.3.-.-] |  |
| pen:PSEEN3085 | DNA methylase (EC:2.1.1.-); K00599 [EC:2.1.1.-] |  |
| pen:PSEEN3086 | hypothetical protein |  |
| pen:PSEEN3087 | hypothetical protein; K06884 |  |
| pen:PSEEN3088 | multidrug ABC transporter; K07552 MFS transporter, DHA1 family, bicyclomycin/chloramphenicol resistance protein |  |
| pen:PSEEN3089 | TetR family transcriptional regulator |  |
| pen:PSEEN3090 | hpaR; homoprotocatechuate degradative operon repressor |  |
| pen:PSEEN3091 | hpaA; 4-hydroxyphenylacetate catabolism regulatory protein hpaA; K02508 AraC family transcriptional regulator, 4-hydroxyphenylacetate 3-monooxygenase operon regulatory protein |  |
| pen:PSEEN3092 | hpaG-1; 2-hydroxyhepta-2,4-diene-1,7-dioate isomerase (EC:5.3.3.10); K05921 5-oxopent-3-ene-1,2,5-tricarboxylate decarboxylase / 2-hydroxyhepta-2,4-diene-1,7-dioate isomerase [EC:4.1.1.68 5.3.3.-] | ec:4.1.1.68 |
| pen:PSEEN3093 | hpaG-2; 4-hydroxyphenylacetate degradation protein (EC:4.1.1.68); K05921 5-oxopent-3-ene-1,2,5-tricarboxylate decarboxylase / 2-hydroxyhepta-2,4-diene-1,7-dioate isomerase [EC:4.1.1.68 5.3.3.-] | ec:4.1.1.68 |
| pen:PSEEN3094 | hpaE; 5-carboxymethyl-2-hydroxymuconate semialdehyde dehydrogenase (EC:1.2.1.-); K00151 5-carboxymethyl-2-hydroxymuconic-semialdehyde dehydrogenase [EC:1.2.1.60] | ec:1.2.1.60 |
| pen:PSEEN3095 | hpaD; 3,4-dihydroxyphenylacetate 2,3-dioxygenase (EC:1.13.11.15); K00455 3,4-dihydroxyphenylacetate 2,3-dioxygenase [EC:1.13.11.15] | ec:1.13.11.15 |
| pen:PSEEN3096 | hpaF; 5-carboxymethyl-2-hydroxymuconate delta-isomerase (EC:5.3.3.10); K01826 5-carboxymethyl-2-hydroxymuconate isomerase [EC:5.3.3.10] | ec:5.3.3.10 |
| pen:PSEEN3097 | hpaH; 2-oxo-hepta-3-ene-1,7-dioic acid hydratase (EC:4.2.-.-); K02509 2-oxo-hept-3-ene-1,7-dioate hydratase [EC:4.2.1.-] |  |
| pen:PSEEN3098 | hpaI; 2,4-dihydroxyhept-2-ene-1,7-dioic acid aldolase; K02510 4-hydroxy-2-oxoheptanedioate aldolase [EC:4.1.2.52] | ec:4.1.2.52 |
| pen:PSEEN3099 | aromatic-ring hydroxylase; K09471 gamma-glutamylputrescine oxidase [EC:1.4.3.-] |  |
| pen:PSEEN3100 | hypothetical protein; K06995 |  |

  
**Neighborhood Representations for "mrb:Mrub\_1336"**  

| ID | Annotation | EC number |
| --- | --- | --- |
| mrb:Mrub\_1326 | ABC transporter; K01995 branched-chain amino acid transport system ATP-binding protein K01998 branched-chain amino acid transport system permease protein |  |
| mrb:Mrub\_1327 | inner-membrane translocator; K01997 branched-chain amino acid transport system permease protein |  |
| mrb:Mrub\_1328 | extracellular ligand-binding receptor; K01999 branched-chain amino acid transport system substrate-binding protein |  |
| mrb:Mrub\_1329 | 3-hydroxybutyryl-CoA dehydrogenase (EC:1.1.1.157); K00074 3-hydroxybutyryl-CoA dehydrogenase [EC:1.1.1.157] | ec:1.1.1.157 |
| mrb:Mrub\_1330 | 3,4-dihydroxyphenylacetate 2,3-dioxygenase |  |
| mrb:Mrub\_1331 | flavin reductase domain-containing FMN-binding protein |  |
| mrb:Mrub\_1332 | 4-hydroxyphenylacetate 3-monooxygenase oxygenase subunit (EC:5.3.3.3); K00483 4-hydroxyphenylacetate 3-monooxygenase [EC:1.14.14.9] | ec:1.14.14.9 |
| mrb:Mrub\_1333 | 5-carboxymethyl-2-hydroxymuconate semialdehyde dehydrogenase (EC:1.2.1.8); K00151 5-carboxymethyl-2-hydroxymuconic-semialdehyde dehydrogenase [EC:1.2.1.60] | ec:1.2.1.60 |
| mrb:Mrub\_1334 | 4-hydroxyphenylacetate degradation bifunctional isomerase/decarboxylase subunit HpaG2 (EC:5.3.3.10); K05921 5-oxopent-3-ene-1,2,5-tricarboxylate decarboxylase / 2-hydroxyhepta-2,4-diene-1,7-dioate isomerase [EC:4.1.1.68 5.3.3.-] | ec:4.1.1.68 |
| mrb:Mrub\_1335 | 2,4-dihydroxyhept-2-ene-1,7-dioic acid aldolase (EC:4.2.1.52); K01714 4-hydroxy-tetrahydrodipicolinate synthase [EC:4.3.3.7] | ec:4.3.3.7 |
| mrb:Mrub\_1336 | MarR family transcriptional regulator |  |
| mrb:Mrub\_1337 | N-acetyltransferase GCN5 |  |
| mrb:Mrub\_1338 | ABC transporter |  |
| mrb:Mrub\_1339 | Fe-S metabolism associated SufE; K02426 cysteine desulfuration protein SufE |  |
| mrb:Mrub\_1340 | hypothetical protein |  |
| mrb:Mrub\_1341 | hypothetical protein |  |
| mrb:Mrub\_1342 | RNA polymerase sigma 70 subunit, RpoD subfamily; K03086 RNA polymerase primary sigma factor |  |
| mrb:Mrub\_1343 | methyltransferase small; K00564 16S rRNA (guanine1207-N2)-methyltransferase [EC:2.1.1.172] | ec:2.1.1.172 |
| mrb:Mrub\_1344 | peptidase M22 glycoprotease |  |
| mrb:Mrub\_1345 | pyrroline-5-carboxylate reductase (EC:1.5.1.2); K00286 pyrroline-5-carboxylate reductase [EC:1.5.1.2] | ec:1.5.1.2 |
| mrb:Mrub\_1346 | hypothetical protein |  |

  
**Neighborhood Representations for "dac:Daci\_0098"**  

| ID | Annotation | EC number |
| --- | --- | --- |
| dac:Daci\_0088 | acyl-CoA dehydrogenase domain-containing protein |  |
| dac:Daci\_0089 | acyl-CoA dehydrogenase domain-containing protein; K00249 acyl-CoA dehydrogenase [EC:1.3.8.7] | ec:1.3.8.7 |
| dac:Daci\_0090 | enoyl-CoA hydratase |  |
| dac:Daci\_0091 | septum site-determining protein MinC; K03610 septum site-determining protein MinC |  |
| dac:Daci\_0092 | septum site-determining protein MinD; K03609 septum site-determining protein MinD |  |
| dac:Daci\_0093 | cell division topological specificity factor MinE; K03608 cell division topological specificity factor |  |
| dac:Daci\_0094 | amino acid permease-associated protein; K03293 amino acid transporter, AAT family |  |
| dac:Daci\_0095 | pseudogene |  |
| dac:Daci\_0096 | flavin reductase domain-containing protein |  |
| dac:Daci\_0097 | DNA polymerase III subunit epsilon (EC:2.7.7.7); K02342 DNA polymerase III subunit epsilon [EC:2.7.7.7] | ec:2.7.7.7 |
| dac:Daci\_0098 | MarR family transcriptional regulator |  |
| dac:Daci\_0099 | 2,4-dihydroxyhept-2-ene-1,7-dioic acid aldolase (EC:4.1.2.20); K02510 4-hydroxy-2-oxoheptanedioate aldolase [EC:4.1.2.52] | ec:4.1.2.52 |
| dac:Daci\_0100 | succinate-semialdehyde dehydrogenase; K00135 succinate-semialdehyde dehydrogenase / glutarate-semialdehyde dehydrogenase [EC:1.2.1.16 1.2.1.79 1.2.1.20] | ec:1.2.1.79 ec:1.2.1.16 ec:1.2.1.20 |
| dac:Daci\_0101 | 2-oxo-hepta-3-ene-1,7-dioic acid hydratase (EC:4.1.1.77); K02509 2-oxo-hept-3-ene-1,7-dioate hydratase [EC:4.2.1.-] |  |
| dac:Daci\_0102 | 5-carboxymethyl-2-hydroxymuconate isomerase; K01826 5-carboxymethyl-2-hydroxymuconate isomerase [EC:5.3.3.10] | ec:5.3.3.10 |
| dac:Daci\_0103 | extradiol ring-cleavage dioxygenase class III protein subunit B; K05713 2,3-dihydroxyphenylpropionate 1,2-dioxygenase [EC:1.13.11.16] | ec:1.13.11.16 |
| dac:Daci\_0104 | extradiol ring-cleavage dioxygenase LigAB LigA subunit |  |
| dac:Daci\_0105 | hypothetical protein |  |
| dac:Daci\_0106 | 5-carboxymethyl-2-hydroxymuconate semialdehyde dehydrogenase; K00151 5-carboxymethyl-2-hydroxymuconic-semialdehyde dehydrogenase [EC:1.2.1.60] | ec:1.2.1.60 |
| dac:Daci\_0107 | 4-hydroxyphenylacetate degradation bifunctional isomerase/decarboxylase subunit HpaG2 (EC:4.1.1.68); K05921 5-oxopent-3-ene-1,2,5-tricarboxylate decarboxylase / 2-hydroxyhepta-2,4-diene-1,7-dioate isomerase [EC:4.1.1.68 5.3.3.-] | ec:4.1.1.68 |
| dac:Daci\_0108 | 4-hydroxyphenylacetate degradation bifunctional isomerase/decarboxylase subunit HpaG1 (EC:4.1.1.68); K05921 5-oxopent-3-ene-1,2,5-tricarboxylate decarboxylase / 2-hydroxyhepta-2,4-diene-1,7-dioate isomerase [EC:4.1.1.68 5.3.3.-] | ec:4.1.1.68 |

  
**Neighborhood Representations for "pfs:PFLU3277"**  

| ID | Annotation | EC number |
| --- | --- | --- |
| pfs:PFLU3267 | homoprotocatechuate degradative operon repressor |  |
| pfs:PFLU3268 | 4-hydroxyphenylacetate 3-monooxygenase operon regulatory protein; K02508 AraC family transcriptional regulator, 4-hydroxyphenylacetate 3-monooxygenase operon regulatory protein |  |
| pfs:PFLU3269 | fumarylacetoacetate (FAA) hydrolase family protein; K05921 5-oxopent-3-ene-1,2,5-tricarboxylate decarboxylase / 2-hydroxyhepta-2,4-diene-1,7-dioate isomerase [EC:4.1.1.68 5.3.3.-] | ec:4.1.1.68 |
| pfs:PFLU3270 | putative 5-carboxymethyl-2-oxo-hex-3-ene-1,7-dioate decarboxylase (EC:4.1.1.68); K05921 5-oxopent-3-ene-1,2,5-tricarboxylate decarboxylase / 2-hydroxyhepta-2,4-diene-1,7-dioate isomerase [EC:4.1.1.68 5.3.3.-] | ec:4.1.1.68 |
| pfs:PFLU3271 | 5-carboxymethyl-2-hydroxymuconate semialdehyde dehydrogenase; K00151 5-carboxymethyl-2-hydroxymuconic-semialdehyde dehydrogenase [EC:1.2.1.60] | ec:1.2.1.60 |
| pfs:PFLU3272 | 3,4-dihydroxyphenylacetate 2,3-dioxygenase (EC:1.13.11.15); K00455 3,4-dihydroxyphenylacetate 2,3-dioxygenase [EC:1.13.11.15] | ec:1.13.11.15 |
| pfs:PFLU3273 | 6 5-carboxymethyl-2-hydroxymuconate delta-isomerase; K01826 5-carboxymethyl-2-hydroxymuconate isomerase [EC:5.3.3.10] | ec:5.3.3.10 |
| pfs:PFLU3274 | putative permease transmembrane protein |  |
| pfs:PFLU3275 | 6 2-oxo-hepta-3-ene-1,7-dioic acid hydratase; K02509 2-oxo-hept-3-ene-1,7-dioate hydratase [EC:4.2.1.-] |  |
| pfs:PFLU3276 | 2,4-dihydroxyhept-2-ene-1,7-dioic acid aldolase; K02510 4-hydroxy-2-oxoheptanedioate aldolase [EC:4.1.2.52] | ec:4.1.2.52 |
| pfs:PFLU3277 | putative homoprotocatechuate degradative operon repressor |  |
| pfs:PFLU3278 | putative oxidoreductase; K09471 gamma-glutamylputrescine oxidase [EC:1.4.3.-] |  |
| pfs:PFLU3279 | hypothetical protein; K06995 |  |
| pfs:PFLU3280 | GerR family regulatory protein |  |
| pfs:PFLU3281 | hypothetical protein |  |
| pfs:PFLU3282 | putative short chain dehydrogenase |  |
| pfs:PFLU3283 | 3-ketoacyl-ACP reductase; K00059 3-oxoacyl-[acyl-carrier protein] reductase [EC:1.1.1.100] | ec:1.1.1.100 |
| pfs:PFLU3284 | TetR family transcriptional regulator |  |
| pfs:PFLU3285 | DNA-binding transcriptional activator FeaR |  |
| pfs:PFLU3286 | hypothetical protein |  |
| pfs:PFLU3287 | putative amino acid permease |  |

  
**Neighborhood Representations for "pae:PA4135"**  

| ID | Annotation | EC number |
| --- | --- | --- |
| pae:PA4125 | hpcD; 5-carboxymethyl-2-hydroxymuconate isomerase; K01826 5-carboxymethyl-2-hydroxymuconate isomerase [EC:5.3.3.10] | ec:5.3.3.10 |
| pae:PA4126 | major facilitator superfamily (MFS) transporter |  |
| pae:PA4127 | hpcG; 2-oxo-hepta-3-ene-1,7-dioic acid hydratase; K02509 2-oxo-hept-3-ene-1,7-dioate hydratase [EC:4.2.1.-] |  |
| pae:PA4128 | hypothetical protein; K02510 4-hydroxy-2-oxoheptanedioate aldolase [EC:4.1.2.52] | ec:4.1.2.52 |
| pae:PA4129 | hypothetical protein |  |
| pae:PA4130 | sulfite or nitrite reductase; K00381 sulfite reductase (NADPH) hemoprotein beta-component [EC:1.8.1.2] | ec:1.8.1.2 |
| pae:PA4131 | iron-sulfur protein |  |
| pae:PA4132 | hypothetical protein |  |
| pae:PA4133 | cbb3-type cytochrome C oxidase subunit I; K00404 cytochrome c oxidase cbb3-type subunit I [EC:1.9.3.1] | ec:1.9.3.1 |
| pae:PA4134 | hypothetical protein |  |
| pae:PA4135 | transcriptional regulator |  |
| pae:PA4136 | major facilitator superfamily (MFS) transporter; K07552 MFS transporter, DHA1 family, bicyclomycin/chloramphenicol resistance protein |  |
| pae:PA4137 | porin |  |
| pae:PA4138 | tyrS; tyrosyl-tRNA synthetase; K01866 tyrosyl-tRNA synthetase [EC:6.1.1.1] | ec:6.1.1.1 |
| pae:PA4139 | hypothetical protein |  |
| pae:PA4140 | hypothetical protein |  |
| pae:PA4141 | hypothetical protein |  |
| pae:PA4142 | secretion protein; K13408 membrane fusion protein RaxA |  |
| pae:PA4143 | toxin transporter; K13409 ATP-binding cassette, subfamily B, bacterial RaxB |  |
| pae:PA4144 | hypothetical protein; K12340 outer membrane channel protein |  |
| pae:PA4145 | transcriptional regulator |  |

  
**Neighborhood Representations for "pag:PLES\_08361"**  

| ID | Annotation | EC number |
| --- | --- | --- |
| pag:PLES\_08261 | hypothetical protein |  |
| pag:PLES\_08271 | hypothetical protein |  |
| pag:PLES\_08281 | hypothetical protein |  |
| pag:PLES\_08291 | hypothetical protein |  |
| pag:PLES\_08301 | hypothetical protein |  |
| pag:PLES\_08311 | hypothetical protein |  |
| pag:PLES\_08321 | hypothetical protein |  |
| pag:PLES\_08331 | tyrS; tyrosyl-tRNA synthetase; K01866 tyrosyl-tRNA synthetase [EC:6.1.1.1] | ec:6.1.1.1 |
| pag:PLES\_08341 | putative porin |  |
| pag:PLES\_08351 | putative major facilitator superfamily transporter; K07552 MFS transporter, DHA1 family, bicyclomycin/chloramphenicol resistance protein |  |
| pag:PLES\_08361 | putative transcriptional regulator |  |
| pag:PLES\_08371 | hypothetical protein |  |
| pag:PLES\_08381 | cbb3-type cytochrome c oxidase subunit I; K00404 cytochrome c oxidase cbb3-type subunit I [EC:1.9.3.1] | ec:1.9.3.1 |
| pag:PLES\_08391 | GntR family transcriptional regulator |  |
| pag:PLES\_08401 | putative iron-sulfur protein |  |
| pag:PLES\_08411 | putative sulfite or nitrite reductase; K00381 sulfite reductase (NADPH) hemoprotein beta-component [EC:1.8.1.2] | ec:1.8.1.2 |
| pag:PLES\_08421 | hypothetical protein |  |
| pag:PLES\_08431 | putative 2,4-dihydroxyhept-2-ene-1,7-dioic acid aldolase; K02510 4-hydroxy-2-oxoheptanedioate aldolase [EC:4.1.2.52] | ec:4.1.2.52 |
| pag:PLES\_08441 | hpcG; 2-oxo-hepta-3-ene-1,7-dioic acid hydratase; K02509 2-oxo-hept-3-ene-1,7-dioate hydratase [EC:4.2.1.-] |  |
| pag:PLES\_08451 | putative major facilitator superfamily transporter |  |
| pag:PLES\_08461 | hpcD; 5-carboxymethyl-2-hydroxymuconate isomerase; K01826 5-carboxymethyl-2-hydroxymuconate isomerase [EC:5.3.3.10] | ec:5.3.3.10 |

  
**Neighborhood Representations for "pau:PA14\_10480"**  

| ID | Annotation | EC number |
| --- | --- | --- |
| pau:PA14\_10340 | cvaB; toxin transporter; K13409 ATP-binding cassette, subfamily B, bacterial RaxB |  |
| pau:PA14\_10350 | secretion protein; K13408 membrane fusion protein RaxA |  |
| pau:PA14\_10360 | hypothetical protein |  |
| pau:PA14\_10370 | choS; hypothetical protein |  |
| pau:PA14\_10380 | hypothetical protein |  |
| pau:PA14\_10400 | hypothetical protein |  |
| pau:PA14\_10410 | hypothetical protein |  |
| pau:PA14\_10420 | tyrS; tyrosyl-tRNA synthetase; K01866 tyrosyl-tRNA synthetase [EC:6.1.1.1] | ec:6.1.1.1 |
| pau:PA14\_10440 | opdL; porin |  |
| pau:PA14\_10470 | bcr; MFS transporter; K07552 MFS transporter, DHA1 family, bicyclomycin/chloramphenicol resistance protein |  |
| pau:PA14\_10480 | transcriptional regulator |  |
| pau:PA14\_10490 | hypothetical protein |  |
| pau:PA14\_10500 | ccoN, cytN, fixN; cbb3-type cytochrome c oxidase subunit I; K00404 cytochrome c oxidase cbb3-type subunit I [EC:1.9.3.1] | ec:1.9.3.1 |
| pau:PA14\_10530 | GntR family transcriptional regulator |  |
| pau:PA14\_10540 | fixG; iron-sulfur cluster-binding protein |  |
| pau:PA14\_10550 | cysI; sulfite or nitrite reductas; K00381 sulfite reductase (NADPH) hemoprotein beta-component [EC:1.8.1.2] | ec:1.8.1.2 |
| pau:PA14\_10560 | hypothetical protein |  |
| pau:PA14\_10570 | hpcH; 2,4-dihydroxyhept-2-ene-1,7-dioic acid aldolase; K02510 4-hydroxy-2-oxoheptanedioate aldolase [EC:4.1.2.52] | ec:4.1.2.52 |
| pau:PA14\_10590 | hpcG; 2-oxo-hepta-3-ene-1,7-dioic acid hydratase; K02509 2-oxo-hept-3-ene-1,7-dioate hydratase [EC:4.2.1.-] |  |
| pau:PA14\_10600 | hpaX; MFS transporter |  |
| pau:PA14\_10610 | hpcD; 5-carboxymethyl-2-hydroxymuconate isomerase; K01826 5-carboxymethyl-2-hydroxymuconate isomerase [EC:5.3.3.10] | ec:5.3.3.10 |

  
**Neighborhood Representations for "rpt:Rpal\_4278"**  

| ID | Annotation | EC number |
| --- | --- | --- |
| rpt:Rpal\_4268 | ethanolamine ammonia-lyase small subunit; K03736 ethanolamine ammonia-lyase small subunit [EC:4.3.1.7] | ec:4.3.1.7 |
| rpt:Rpal\_4269 | radical SAM domain-containing protein |  |
| rpt:Rpal\_4270 | ethanolamine ammonia lyase large subunit; K03735 ethanolamine ammonia-lyase large subunit [EC:4.3.1.7] | ec:4.3.1.7 |
| rpt:Rpal\_4271 | methyl-accepting chemotaxis sensory transducer |  |
| rpt:Rpal\_4272 | Cache type 2 domain-containing protein |  |
| rpt:Rpal\_4273 | transglutaminase domain-containing protein |  |
| rpt:Rpal\_4274 | hypothetical protein |  |
| rpt:Rpal\_4275 | transglutaminase domain-containing protein |  |
| rpt:Rpal\_4276 | HpcH/HpaI aldolase; K02510 4-hydroxy-2-oxoheptanedioate aldolase [EC:4.1.2.52] | ec:4.1.2.52 |
| rpt:Rpal\_4277 | malate/L-lactate dehydrogenase; K13574 uncharacterized oxidoreductase [EC:1.1.1.-] |  |
| rpt:Rpal\_4278 | MarR family transcriptional regulator |  |
| rpt:Rpal\_4279 | 2-oxo-hepta-3-ene-1,7-dioic acid hydratase; K02509 2-oxo-hept-3-ene-1,7-dioate hydratase [EC:4.2.1.-] |  |
| rpt:Rpal\_4280 | 5-carboxymethyl-2-hydroxymuconate isomerase; K01826 5-carboxymethyl-2-hydroxymuconate isomerase [EC:5.3.3.10] | ec:5.3.3.10 |
| rpt:Rpal\_4281 | 5-carboxymethyl-2-hydroxymuconate semialdehyde dehydrogenase; K00151 5-carboxymethyl-2-hydroxymuconic-semialdehyde dehydrogenase [EC:1.2.1.60] | ec:1.2.1.60 |
| rpt:Rpal\_4282 | 3,4-dihydroxyphenylacetate 2,3-dioxygenase; K00446 catechol 2,3-dioxygenase [EC:1.13.11.2] | ec:1.13.11.2 |
| rpt:Rpal\_4283 | 5-oxopent-3-ene-1,2,5-tricarboxylate decarboxylase |  |
| rpt:Rpal\_4284 | hypothetical protein; K01652 acetolactate synthase I/II/III large subunit [EC:2.2.1.6] | ec:2.2.1.6 |
| rpt:Rpal\_4285 | phenylacetate-CoA oxygenase/reductase subunit PaaK; K02613 ring-1,2-phenylacetyl-CoA epoxidase subunit PaaE |  |
| rpt:Rpal\_4286 | phenylacetate-CoA oxygenase subunit PaaJ; K02612 ring-1,2-phenylacetyl-CoA epoxidase subunit PaaD |  |
| rpt:Rpal\_4287 | phenylacetate-CoA oxygenase subunit PaaI; K02611 ring-1,2-phenylacetyl-CoA epoxidase subunit PaaC [EC:1.14.13.149] | ec:1.14.13.149 |
| rpt:Rpal\_4288 | paaB; phenylacetate-CoA oxygenase subunit PaaB; K02610 ring-1,2-phenylacetyl-CoA epoxidase subunit PaaB |  |

  
**Neighborhood Representations for "rpa:RPA3757"**  

| ID | Annotation | EC number |
| --- | --- | --- |
| rpa:RPA3747 | eutC; ethanolamine ammonia-lyase small subunit; K03736 ethanolamine ammonia-lyase small subunit [EC:4.3.1.7] | ec:4.3.1.7 |
| rpa:RPA3748 | radical SAM family protein |  |
| rpa:RPA3749 | eutB; ethanolamine ammonia lyase large subunit; K03735 ethanolamine ammonia-lyase large subunit [EC:4.3.1.7] | ec:4.3.1.7 |
| rpa:RPA3750 | methyl-accepting chemotaxis sensory transducer |  |
| rpa:RPA3751 | hypothetical protein |  |
| rpa:RPA3752 | transglutaminase |  |
| rpa:RPA3753 | hypothetical protein |  |
| rpa:RPA3754 | transglutaminase |  |
| rpa:RPA3755 | hpcH; HpcH/HpaI aldolase; K02510 4-hydroxy-2-oxoheptanedioate aldolase [EC:4.1.2.52] | ec:4.1.2.52 |
| rpa:RPA3756 | malate dehydrogenase; K13574 uncharacterized oxidoreductase [EC:1.1.1.-] |  |
| rpa:RPA3757 | MarR family transcriptional regulator |  |
| rpa:RPA3758 | hpcG; 2-oxo-hepta-3-ene-1,7-dioic acid hydratase; K02509 2-oxo-hept-3-ene-1,7-dioate hydratase [EC:4.2.1.-] |  |
| rpa:RPA3759 | hpcD; 5-carboxymethyl-2-hydroxymuconate isomerase; K01826 5-carboxymethyl-2-hydroxymuconate isomerase [EC:5.3.3.10] | ec:5.3.3.10 |
| rpa:RPA3760 | hpcC; 5-carboxy-2-hydroxymuconate semialdehyde dehydrogenase; K00151 5-carboxymethyl-2-hydroxymuconic-semialdehyde dehydrogenase [EC:1.2.1.60] | ec:1.2.1.60 |
| rpa:RPA3761 | catechol 2,3-dioxygenase; K00446 catechol 2,3-dioxygenase [EC:1.13.11.2] | ec:1.13.11.2 |
| rpa:RPA3762 | 2-oxo-3-ene-1,7-dioic acid hydratase |  |
| rpa:RPA3763 | ilvB; hypothetical protein; K01652 acetolactate synthase I/II/III large subunit [EC:2.2.1.6] | ec:2.2.1.6 |
| rpa:RPA3764 | phenylacetate-CoA oxygenase/reductase subunit PaaK; K02613 ring-1,2-phenylacetyl-CoA epoxidase subunit PaaE |  |
| rpa:RPA3765 | paaD; phenylacetate-CoA oxygenase subunit PaaJ; K02612 ring-1,2-phenylacetyl-CoA epoxidase subunit PaaD |  |
| rpa:RPA3766 | paaC; phenylacetate-CoA oxygenase subunit PaaI; K02611 ring-1,2-phenylacetyl-CoA epoxidase subunit PaaC [EC:1.14.13.149] | ec:1.14.13.149 |
| rpa:RPA3767 | paaB; phenylacetate-CoA oxygenase subunit PaaB; K02610 ring-1,2-phenylacetyl-CoA epoxidase subunit PaaB |  |

  
**Over-represented Enzyme Summary**: Table of E.C. identified protein in the "Neighborhood Representation" ranked by frequency of occurrence  

| EC number | Frequency | Annotation | Reactions |
| --- | --- | --- | --- |
| ec:4.1.1.68 | 62 | 5-oxopent-3-ene-1,2,5-tricarboxylate decarboxylase; 5-carboxymethyl-2-oxo-hex-3-ene-1,6-dioate decarboxylase; 5-oxopent-3-ene-1,2,5-tricarboxylate carboxy-lyase | 5-oxopent-3-ene-1,2,5-tricarboxylate = 2-oxohept-3-enedioate + CO2 [RN:R04133] |
| ec:4.1.2.52 | 36 | 4-hydroxy-2-oxoheptanedioate aldolase; 2,4-dihydroxyhept-2-enedioate aldolase; HHED aldolase; 4-hydroxy-2-ketoheptanedioate aldolase; HKHD aldolase; HpcH; HpaI | 4-hydroxy-2-oxoheptanedioate = pyruvate + succinate semialdehyde [RN:R01645] |
| ec:5.3.3.10 | 35 | 5-carboxymethyl-2-hydroxymuconate Delta-isomerase | 5-carboxymethyl-2-hydroxymuconate = 5-carboxy-2-oxohept-3-enedioate [RN:R04379] |
| ec:1.2.1.60 | 34 | 5-carboxymethyl-2-hydroxymuconic-semialdehyde dehydrogenase; carboxymethylhydroxymuconic semialdehyde dehydrogenase | 5-carboxymethyl-2-hydroxymuconate semialdehyde + H2O + NAD+ = 5-carboxymethyl-2-hydroxymuconate + NADH + 2 H+ [RN:R04418] |
| ec:1.13.11.15 | 30 | 3,4-dihydroxyphenylacetate 2,3-dioxygenase; 3,4-dihydroxyphenylacetic acid 2,3-dioxygenase; HPC dioxygenase; homoprotocatechuate 2,3-dioxygenase | 3,4-dihydroxyphenylacetate + O2 = 2-hydroxy-5-carboxymethylmuconate semialdehyde [RN:R03303] |
| ec:1.1.99.3 | 20 | gluconate 2-dehydrogenase (acceptor); gluconate oxidase; gluconate dehydrogenase; gluconic dehydrogenase; D-gluconate dehydrogenase; gluconic acid dehydrogenase; 2-ketogluconate reductase; D-gluconate dehydrogenase, 2-keto-D-gluconate-yielding; D-gluconate:(acceptor) 2-oxidoreductase | D-gluconate + acceptor = 2-dehydro-D-gluconate + reduced acceptor [RN:R01742] |
| ec:2.4.1.129 | 10 | peptidoglycan glycosyltransferase; PG-II; bactoprenyldiphospho-N-acetylmuramoyl-(N-acetyl-D-glucosaminyl)-pentapeptide:peptidoglycan N-acetylmuramoyl-N-acetyl-D-glucosaminyltransferase; penicillin binding protein (3 or 1B); peptidoglycan transglycosylase; undecaprenyldiphospho-(N-acetyl-D-glucosaminyl-(1->4)-N-acetyl-D-muramoylpentapeptide):undecaprenyldiphospho-(N-acetyl-D-glucosaminyl-(1->4)-N-acetyl-D-muramoylpentapeptide) disaccharidetransferase | [GlcNAc-(1->4)-Mur2Ac(oyl-L-Ala-gamma-D-Glu-L-Lys-D-Ala-D-Ala)]n-diphosphoundecaprenol + GlcNAc-(1->4)-Mur2Ac(oyl-L-Ala-gamma-D-Glu-L-Lys-D-Ala-D-Ala)-diphosphoundecaprenol = [GlcNAc-(1->4)-Mur2Ac(oyl-L-Ala-gamma-D-Glu-L-Lys-D-Ala-D-Ala)]n+1-diphosphoundecaprenol + undecaprenyl diphosphate [RN:R06178 R06179] |
| ec:2.7.13.3 | 9 | histidine kinase; EnvZ; histidine kinase (ambiguous); histidine protein kinase (ambiguous); protein histidine kinase (ambiguous); protein kinase (histidine) (ambiguous); HK1; HP165; Sln1p | ATP + protein L-histidine = ADP + protein N-phospho-L-histidine |
| ec:1.4.1.1 | 8 | alanine dehydrogenase; AlaDH; L-alanine dehydrogenase; NAD+-linked alanine dehydrogenase; alpha-alanine dehydrogenase; NAD+-dependent alanine dehydrogenase; alanine oxidoreductase; NADH-dependent alanine dehydrogenase | L-alanine + H2O + NAD+ = pyruvate + NH3 + NADH + H+ [RN:R00396] |
| ec:3.1.11.2 | 7 | exodeoxyribonuclease III; Escherichia coli exonuclease III; E. coli exonuclease III; endoribonuclease III | Exonucleolytic cleavage in the 3'- to 5'-direction to yield nucleoside 5'-phosphates |
| ec:1.2.4.1 | 7 | pyruvate dehydrogenase (acetyl-transferring); MtPDC (mitochondrial pyruvate dehydogenase complex); pyruvate decarboxylase; pyruvate dehydrogenase; pyruvate dehydrogenase (lipoamide); pyruvate dehydrogenase complex; pyruvate:lipoamide 2-oxidoreductase (decarboxylating and acceptor-acetylating); pyruvic acid dehydrogenase; pyruvic dehydrogenase | pyruvate + [dihydrolipoyllysine-residue acetyltransferase] lipoyllysine = [dihydrolipoyllysine-residue acetyltransferase] S-acetyldihydrolipoyllysine + CO2 [RN:R01699] |
| ec:3.5.4.9 | 7 | methenyltetrahydrofolate cyclohydrolase; Citrovorum factor cyclodehydrase; cyclohydrolase; formyl-methenyl-methylenetetrahydrofolate synthetase (combined) | 5,10-methenyltetrahydrofolate + H2O = 10-formyltetrahydrofolate [RN:R01655] |
| ec:2.3.1.12 | 7 | dihydrolipoyllysine-residue acetyltransferase; acetyl-CoA:dihydrolipoamide S-acetyltransferase; dihydrolipoamide S-acetyltransferase; dihydrolipoate acetyltransferase; dihydrolipoic transacetylase; dihydrolipoyl acetyltransferase; lipoate acetyltransferase; lipoate transacetylase; lipoic acetyltransferase; lipoic acid acetyltransferase; lipoic transacetylase; lipoylacetyltransferase; thioltransacetylase A; transacetylase X; enzyme-dihydrolipoyllysine:acetyl-CoA S-acetyltransferase; acetyl-CoA:enzyme 6-N-(dihydrolipoyl)lysine S-acetyltransferase | acetyl-CoA + enzyme N6-(dihydrolipoyl)lysine = CoA + enzyme N6-(S-acetyldihydrolipoyl)lysine [RN:R02569] |
| ec:1.5.1.5 | 7 | methylenetetrahydrofolate dehydrogenase (NADP+); N5,N10-methylenetetrahydrofolate dehydrogenase; 5,10-methylenetetrahydrofolate:NADP oxidoreductase; 5,10-methylenetetrahydrofolate dehydrogenase; methylenetetrahydrofolate dehydrogenase; methylenetetrahydrofolate dehydrogenase (NADP) | 5,10-methylenetetrahydrofolate + NADP+ = 5,10-methenyltetrahydrofolate + NADPH + H+ [RN:R01220] |
| ec:3.4.24.70 | 7 | oligopeptidase A; 68000-M signalpeptide hydrolase | Hydrolysis of oligopeptides, with broad specificity. Gly or Ala commonly occur as P1 or P1' residues, but more distant residues are also important, as is shown by the fact that Z-Gly-Pro-Gly!Gly-Pro-Ala is cleaved, but not Z-(Gly)5 [4] |
| ec:2.7.7.7 | 6 | DNA-directed DNA polymerase; DNA polymerase I; DNA polymerase II; DNA polymerase III; DNA polymerase alpha; DNA polymerase beta; DNA polymerase gamma; DNA nucleotidyltransferase (DNA-directed); DNA nucleotidyltransferase (DNA-directed); deoxyribonucleate nucleotidyltransferase; deoxynucleate polymerase; deoxyribonucleic acid duplicase; deoxyribonucleic acid polymerase; deoxyribonucleic duplicase; deoxyribonucleic polymerase; deoxyribonucleic polymerase I; DNA duplicase; DNA nucleotidyltransferase; DNA polymerase; DNA replicase; DNA-dependent DNA polymerase; duplicase; Klenow fragment; sequenase; Taq DNA polymerase; Taq Pol I; Tca DNA polymerase | deoxynucleoside triphosphate + DNAn = diphosphate + DNAn+1 [RN:R00379] |
| ec:1.8.1.4 | 5 | dihydrolipoyl dehydrogenase; LDP-Glc; LDP-Val; dehydrolipoate dehydrogenase; diaphorase; dihydrolipoamide dehydrogenase; dihydrolipoamide:NAD+ oxidoreductase; dihydrolipoic dehydrogenase; dihydrothioctic dehydrogenase; lipoamide dehydrogenase (NADH); lipoamide oxidoreductase (NADH); lipoamide reductase; lipoamide reductase (NADH); lipoate dehydrogenase; lipoic acid dehydrogenase; lipoyl dehydrogenase; protein-6-N-(dihydrolipoyl)lysine:NAD+ oxidoreductase | protein N6-(dihydrolipoyl)lysine + NAD+ = protein N6-(lipoyl)lysine + NADH + H+ [RN:R08550] |
| ec:4.3.1.7 | 4 | ethanolamine ammonia-lyase; ethanolamine deaminase | ethanolamine = acetaldehyde + NH3 [RN:R00749] |
| ec:3.5.4.1 | 3 | cytosine deaminase; isocytosine deaminase | cytosine + H2O = uracil + NH3 [RN:R00974] |
| ec:1.9.3.1 | 3 | cytochrome-c oxidase; cytochrome oxidase; cytochrome a3; cytochrome aa3; Warburg's respiratory enzyme; indophenol oxidase; indophenolase; complex IV (mitochondrial electron transport); ferrocytochrome c oxidase; NADH cytochrome c oxidase | 4 ferrocytochrome c + O2 + 4 H+ = 4 ferricytochrome c + 2 H2O [RN:R00081] |
| ec:1.2.1.27 | 3 | methylmalonate-semialdehyde dehydrogenase (acylating); MSDH; MMSA dehydrogenase | 2-methyl-3-oxopropanoate + CoA + H2O + NAD+ = propanoyl-CoA + HCO3- + NADH [RN:R00922] |
| ec:1.2.1.18 | 3 | malonate-semialdehyde dehydrogenase (acetylating); malonic semialdehyde oxidative decarboxylase | 3-oxopropanoate + CoA + NAD(P)+ = acetyl-CoA + CO2 + NAD(P)H [RN:R00705 R00706] |
| ec:6.1.1.1 | 3 | tyrosine---tRNA ligase | ATP + L-tyrosine + tRNATyr = AMP + diphosphate + L-tyrosyl-tRNATyr [RN:R02918] |
| ec:1.8.1.2 | 3 | sulfite reductase (NADPH); sulfite (reduced nicotinamide adenine dinucleotide phosphate) reductase; NADPH-sulfite reductase; NADPH-dependent sulfite reductase; H2S-NADP oxidoreductase; sulfite reductase (NADPH2) | hydrogen sulfide + 3 NADP+ + 3 H2O = sulfite + 3 NADPH + 3 H+ [RN:R00858] |
| ec:4.2.1.10 | 2 | 3-dehydroquinate dehydratase; 3-dehydroquinate hydrolase; DHQase; dehydroquinate dehydratase; 3-dehydroquinase; 5-dehydroquinase; dehydroquinase; 5-dehydroquinate dehydratase; 5-dehydroquinate hydro-lyase; 3-dehydroquinate hydro-lyase | 3-dehydroquinate = 3-dehydroshikimate + H2O [RN:R03084] |
| ec:3.6.3.30 | 2 | Fe3+-transporting ATPase | ATP + H2O + Fe3+out = ADP + phosphate + Fe3+in [RN:R00086] |
| ec:1.14.13.149 | 2 | phenylacetyl-CoA 1,2-epoxidase; ring 1,2-phenylacetyl-CoA epoxidase; phenylacetyl-CoA monooxygenase; PaaAC; PaaABC(D)E | phenylacetyl-CoA + NADPH + H+ + O2 = 2-(1,2-epoxy-1,2-dihydrophenyl)acetyl-CoA + NADP+ + H2O [RN:R09838] |
| ec:2.2.1.6 | 2 | acetolactate synthase; alpha-acetohydroxy acid synthetase; alpha-acetohydroxyacid synthase; alpha-acetolactate synthase; alpha-acetolactate synthetase; acetohydroxy acid synthetase; acetohydroxyacid synthase; acetolactate pyruvate-lyase (carboxylating); acetolactic synthetase | 2 pyruvate = 2-acetolactate + CO2 [RN:R00006] |
| ec:3.5.99.3 | 2 | hydroxydechloroatrazine ethylaminohydrolase; AtzB; hydroxyatrazine ethylaminohydrolase | 4-(ethylamino)-2-hydroxy-6-(isopropylamino)-1,3,5-triazine + H2O = N-isopropylammelide + ethylamine [RN:R05559] |
| ec:1.2.1.20 | 2 | glutarate-semialdehyde dehydrogenase; glutarate semialdehyde dehydrogenase | 5-oxopentanoate + NAD+ + H2O = glutarate + NADH + 2 H+ [RN:R02401] |
| ec:3.1.2.1 | 2 | acetyl-CoA hydrolase; acetyl-CoA deacylase; acetyl-CoA acylase; acetyl coenzyme A hydrolase; acetyl coenzyme A deacylase; acetyl coenzyme A acylase; acetyl-CoA thiol esterase | acetyl-CoA + H2O = CoA + acetate [RN:R00227] |
| ec:1.2.1.16 | 2 | succinate-semialdehyde dehydrogenase [NAD(P)+]; succinate semialdehyde dehydrogenase (nicotinamide adenine dinucleotide (phosphate)); succinate-semialdehyde dehydrogenase [NAD(P)] | succinate semialdehyde + NAD(P)+ + H2O = succinate + NAD(P)H + 2 H+ [RN:R00713 R00714] |
| ec:1.2.1.79 | 2 | succinate-semialdehyde dehydrogenase (NADP+); succinic semialdehyde dehydrogenase (NADP+); succinyl semialdehyde dehydrogenase (NADP+); succinate semialdehyde:NADP+ oxidoreductase; NADP-dependent succinate-semialdehyde dehydrogenase; GabD | succinate semialdehyde + NADP+ + H2O = succinate + NADPH + 2 H+ [RN:R00714] |
| ec:1.13.11.2 | 2 | catechol 2,3-dioxygenase; 2,3-pyrocatechase; catechol 2,3-oxygenase; catechol oxygenase; metapyrocatechase; pyrocatechol 2,3-dioxygenase; xylE (gene name) | catechol + O2 = 2-hydroxymuconate-6-semialdehyde [RN:R00816] |
| ec:2.7.1.2 | 1 | glucokinase; glucokinase (phosphorylating) | ATP + D-glucose = ADP + D-glucose 6-phosphate [RN:R00299] |
| ec:1.1.1.49 | 1 | glucose-6-phosphate dehydrogenase (NADP+); NADP-glucose-6-phosphate dehydrogenase; Zwischenferment; D-glucose 6-phosphate dehydrogenase; glucose 6-phosphate dehydrogenase (NADP); NADP-dependent glucose 6-phosphate dehydrogenase; 6-phosphoglucose dehydrogenase; Entner-Doudoroff enzyme; glucose-6-phosphate 1-dehydrogenase; G6PDH; GPD; glucose-6-phosphate dehydrogenase | D-glucose 6-phosphate + NADP+ = 6-phospho-D-glucono-1,5-lactone + NADPH + H+ [RN:R00835] |
| ec:4.1.99.12 | 1 | 3,4-dihydroxy-2-butanone-4-phosphate synthase; DHBP synthase; L-3,4-dihydroxybutan-2-one-4-phosphate synthase | D-ribulose 5-phosphate = formate + L-3,4-dihydroxybutan-2-one 4-phosphate [RN:R07281] |
| ec:3.5.4.26 | 1 | diaminohydroxyphosphoribosylaminopyrimidine deaminase | 2,5-diamino-6-hydroxy-4-(5-phospho-D-ribosylamino)pyrimidine + H2O = 5-amino-6-(5-phospho-D-ribosylamino)uracil + NH3 [RN:R03459] |
| ec:3.5.4.25 | 1 | GTP cyclohydrolase II; guanosine triphosphate cyclohydrolase II; GTP-8-formylhydrolase | GTP + 3 H2O = formate + 2,5-diamino-6-hydroxy-4-(5-phospho-D-ribosylamino)pyrimidine + diphosphate [RN:R00425] |
| ec:6.1.1.20 | 1 | phenylalanine---tRNA ligase; phenylalanyl-tRNA synthetase; phenylalanyl-transfer ribonucleate synthetase; phenylalanine-tRNA synthetase; phenylalanyl-transfer RNA synthetase; phenylalanyl-tRNA ligase; phenylalanyl-transfer RNA ligase; L-phenylalanyl-tRNA synthetase; phenylalanine translase | ATP + L-phenylalanine + tRNAPhe = AMP + diphosphate + L-phenylalanyl-tRNAPhe [RN:R03660] |
| ec:1.3.8.7 | 1 | medium-chain acyl-CoA dehydrogenase; fatty acyl coenzyme A dehydrogenase (ambiguous); acyl coenzyme A dehydrogenase (ambiguous); acyl dehydrogenase (ambiguous); fatty-acyl-CoA dehydrogenase (ambiguous); acyl CoA dehydrogenase (ambiguous); general acyl CoA dehydrogenase (ambiguous); medium-chain acyl-coenzyme A dehydrogenase; acyl-CoA:(acceptor) 2,3-oxidoreductase (ambiguous); ACADM (gene name). | a medium-chain acyl-CoA + electron-transfer flavoprotein = a medium-chain trans-2,3-dehydroacyl-CoA + reduced electron-transfer flavoprotein [RN:R00392] |
| ec:2.5.1.78 | 1 | 6,7-dimethyl-8-ribityllumazine synthase; lumazine synthase; 6,7-dimethyl-8-ribityllumazine synthase 2; 6,7-dimethyl-8-ribityllumazine synthase 1; lumazine synthase 2; lumazine synthase 1; type I lumazine synthase; type II lumazine synthase; RIB4; MJ0303; RibH; Pbls; MbtLS; RibH1 protein; RibH2 protein; RibH1; RibH2 | 1-deoxy-L-glycero-tetrulose 4-phosphate + 5-amino-6-(D-ribitylamino)uracil = 6,7-dimethyl-8-(D-ribityl)lumazine + 2 H2O + phosphate [RN:R04457] |
| ec:2.7.1.15 | 1 | ribokinase; deoxyribokinase; ribokinase (phosphorylating); D-ribokinase | ATP + D-ribose = ADP + D-ribose 5-phosphate [RN:R01051] |
| ec:3.2.2.1 | 1 | purine nucleosidase; nucleosidase (misleading); purine beta-ribosidase; purine nucleoside hydrolase; purine ribonucleosidase; ribonucleoside hydrolase (misleading); nucleoside hydrolase (misleading); N-ribosyl purine ribohydrolase; nucleosidase g; N-D-ribosylpurine ribohydrolase; inosine-adenosine-guanosine preferring nucleoside hydrolase; purine-specific nucleoside N-ribohydrolase; IAG-nucleoside hydrolase; IAG-NH | a purine nucleoside + H2O = D-ribose + a purine base [RN:R02341] |
| ec:4.2.1.9 | 1 | dihydroxy-acid dehydratase; acetohydroxyacid dehydratase; alpha,beta-dihydroxyacid dehydratase; 2,3-dihydroxyisovalerate dehydratase; alpha,beta-dihydroxyisovalerate dehydratase; dihydroxy acid dehydrase; DHAD; 2,3-dihydroxy-acid hydro-lyase | 2,3-dihydroxy-3-methylbutanoate = 3-methyl-2-oxobutanoate + H2O [RN:R01209] |
| ec:3.6.3.21 | 1 | polar-amino-acid-transporting ATPase; histidine permease | ATP + H2O + polar amino acidout = ADP + phosphate + polar amino acidin [RN:R00086] |
| ec:3.1.26.11 | 1 | tRNase Z; 3 tRNase; tRNA 3 endonuclease; RNase Z; 3' tRNase | endonucleolytic cleavage of RNA, removing extra 3' nucleotides from tRNA precursor, generating 3' termini of tRNAs. A 3'-hydroxy group is left at the tRNA terminus and a 5'-phosphoryl group is left at the trailer molecule |
| ec:3.2.1.28 | 1 | alpha,alpha-trehalase; trehalase | alpha,alpha-trehalose + H2O = beta-D-glucose + alpha-D-glucose [RN:R00010 R06103] |
| ec:3.4.25.2 | 1 | HslU---HslV peptidase; HslUV; HslV-HslU; HslV peptidase; ATP-dependent HslV-HslU proteinase; caseinolytic protease X; caseinolytic proteinase X; ClpXP ATP-dependent protease; ClpXP protease; ClpXP serine proteinase; Escherichia coli ClpXP serine proteinase; HslUV protease; HslUV proteinase; HslVU protease; HslVU proteinase; protease HslVU; proteinase HslUV | ATP-dependent cleavage of peptide bonds with broad specificity. |
| ec:2.5.1.9 | 1 | riboflavin synthase; heavy riboflavin synthase; light riboflavin synthase; riboflavin synthetase; riboflavine synthase; riboflavine synthetase | 2 6,7-dimethyl-8-(1-D-ribityl)lumazine = riboflavin + 4-(1-D-ribitylamino)-5-amino-2,6-dihydroxypyrimidine [RN:R00066] |
| ec:1.1.1.100 | 1 | 3-oxoacyl-[acyl-carrier-protein] reductase; beta-ketoacyl-[acyl-carrier protein](ACP) reductase; beta-ketoacyl acyl carrier protein (ACP) reductase; beta-ketoacyl reductase; beta-ketoacyl thioester reductase; beta-ketoacyl-ACP reductase; beta-ketoacyl-acyl carrier protein reductase; 3-ketoacyl acyl carrier protein reductase; NADPH-specific 3-oxoacyl-[acylcarrier protein]reductase; 3-oxoacyl-[ACP]reductase; (3R)-3-hydroxyacyl-[acyl-carrier-protein]:NADP+ oxidoreductase | a (3R)-3-hydroxyacyl-[acyl-carrier protein] + NADP+ = a 3-oxoacyl-[acyl-carrier protein] + NADPH + H+ [RN:R02767] |
| ec:1.1.1.193 | 1 | 5-amino-6-(5-phosphoribosylamino)uracil reductase; aminodioxyphosphoribosylaminopyrimidine reductase | 5-amino-6-(5-phospho-D-ribitylamino)uracil + NADP+ = 5-amino-6-(5-phospho-D-ribosylamino)uracil + NADPH + H+ [RN:R03458] |
| ec:1.5.1.2 | 1 | pyrroline-5-carboxylate reductase; proline oxidase; L-proline oxidase; 1-pyrroline-5-carboxylate reductase; NADPH-L-Delta1-pyrroline carboxylic acid reductase; L-proline-NAD(P)+ 5-oxidoreductase | L-proline + NAD(P)+ = 1-pyrroline-5-carboxylate + NAD(P)H + H+ [RN:R01248 R01251] |
| ec:5.4.3.8 | 1 | glutamate-1-semialdehyde 2,1-aminomutase; glutamate-1-semialdehyde aminotransferase | L-glutamate 1-semialdehyde = 5-aminolevulinate [RN:R02272] |
| ec:1.13.11.16 | 1 | 3-carboxyethylcatechol 2,3-dioxygenase; 2,3-dihydroxy-beta-phenylpropionic dioxygenase; 2,3-dihydroxy-beta-phenylpropionate oxygenase; 3-(2,3-dihydroxyphenyl)propanoate:oxygen 1,2-oxidoreductase | (1) 3-(2,3-dihydroxyphenyl)propanoate + O2 = (2Z,4E)-2-hydroxy-6-oxonona-2,4-diene-1,9-dioate; (2) (2E)-3-(2,3-dihydroxyphenyl)prop-2-enoate + O2 = (2Z,4E,7E)-2-hydroxy-6-oxonona-2,4,7-triene-1,9-dioate |
| ec:2.1.1.172 | 1 | 16S rRNA (guanine1207-N2)-methyltransferase; m2G1207 methyltransferase | S-adenosyl-L-methionine + guanine1207 in 16S rRNA = S-adenosyl-L-homocysteine + N2-methylguanine1207 in 16S rRNA [RN:R07234] |
| ec:3.5.1.38 | 1 | glutamin-(asparagin-)ase; glutaminase-asparaginase; ansB (gene name); L-asparagine/L-glutamine amidohydrolase; L-ASNase/L-GLNase | (1) L-glutamine + H2O = L-glutamate + NH3 [RN:R00256]; (2) L-asparagine + H2O = L-aspartate + NH3 [RN:R00485] |
| ec:3.6.3.17 | 1 | monosaccharide-transporting ATPase | ATP + H2O + monosaccharideout = ADP + phosphate + monosaccharidein [RN:R00086] |
| ec:2.6.1.17 | 1 | succinyldiaminopimelate transaminase; succinyldiaminopimelate aminotransferase; N-succinyl-L-diaminopimelic glutamic transaminase | N-succinyl-L-2,6-diaminoheptanedioate + 2-oxoglutarate = N-succinyl-L-2-amino-6-oxoheptanedioate + L-glutamate [RN:R04475] |
| ec:1.1.1.157 | 1 | 3-hydroxybutyryl-CoA dehydrogenase; beta-hydroxybutyryl coenzyme A dehydrogenase; L(+)-3-hydroxybutyryl-CoA dehydrogenase; BHBD; dehydrogenase, L-3-hydroxybutyryl coenzyme A (nicotinamide adenine dinucleotide phosphate); L-(+)-3-hydroxybutyryl-CoA dehydrogenase; beta-hydroxybutyryl-CoA dehydrogenase | (S)-3-hydroxybutanoyl-CoA + NADP+ = 3-acetoacetyl-CoA + NADPH + H+ [RN:R01976] |
| ec:1.3.99.16 | 1 | isoquinoline 1-oxidoreductase | isoquinoline + acceptor + H2O = isoquinolin-1(2H)-one + reduced acceptor [RN:R05151] |
| ec:2.6.1.11 | 1 | acetylornithine transaminase; acetylornithine delta-transaminase; ACOAT; acetylornithine 5-aminotransferase; acetylornithine aminotransferase; N-acetylornithine aminotransferase; N-acetylornithine-delta-transaminase; N2-acetylornithine 5-transaminase; N2-acetyl-L-ornithine:2-oxoglutarate aminotransferase; succinylornithine aminotransferase; 2-N-acetyl-L-ornithine:2-oxoglutarate 5-aminotransferase | N2-acetyl-L-ornithine + 2-oxoglutarate = N-acetyl-L-glutamate 5-semialdehyde + L-glutamate [RN:R02283] |
| ec:2.6.1.42 | 1 | branched-chain-amino-acid transaminase; transaminase B; branched-chain amino acid aminotransferase; branched-chain amino acid-glutamate transaminase; branched-chain aminotransferase; L-branched chain amino acid aminotransferase; glutamate-branched-chain amino acid transaminase | L-leucine + 2-oxoglutarate = 4-methyl-2-oxopentanoate + L-glutamate [RN:R01090] |
| ec:1.14.13.127 | 1 | 3-(3-hydroxyphenyl)propanoate hydroxylase; mhpA (gene name) | (1) 3-(3-hydroxyphenyl)propanoate + NADH + H+ + O2 = 3-(2,3-dihydroxyphenyl)propanoate + H2O + NAD+ [RN:R06786]; (2) (2E)-3-(3-hydroxyphenyl)prop-2-enoate + NADH + H+ + O2 = (2E)-3-(2,3-dihydroxyphenyl)prop-2-enoate + H2O + NAD+ [RN:R06787] |
| ec:4.3.3.7 | 1 | 4-hydroxy-tetrahydrodipicolinate synthase; dihydrodipicolinate synthase (incorrect); dihydropicolinate synthetase (incorrect); dihydrodipicolinic acid synthase (incorrect); L-aspartate-4-semialdehyde hydro-lyase (adding pyruvate and cyclizing); dapA (gene name). | pyruvate + L-aspartate-4-semialdehyde = (2S,4S)-4-hydroxy-2,3,4,5-tetrahydrodipicolinate + H2O [RN:R10147] |
| ec:1.4.1.2 | 1 | glutamate dehydrogenase; glutamic dehydrogenase; glutamate dehydrogenase (NAD+); glutamate oxidoreductase; glutamic acid dehydrogenase; L-glutamate dehydrogenase; NAD+-dependent glutamate dehydrogenase; NAD+-dependent glutamic dehydrogenase; NAD+-glutamate dehydrogenase; NAD+-linked glutamate dehydrogenase; NAD+-linked glutamic dehydrogenase; NAD+-specific glutamic dehydrogenase; NAD+-specific glutamate dehydrogenase; NAD+:glutamate oxidoreductase; NADH-linked glutamate dehydrogenase | L-glutamate + H2O + NAD+ = 2-oxoglutarate + NH3 + NADH + H+ [RN:R00243] |
| ec:1.14.14.9 | 1 | 4-hydroxyphenylacetate 3-monooxygenase; p-hydroxyphenylacetate 3-hydroxylase; 4-hydroxyphenylacetic acid-3-hydroxylase; p-hydroxyphenylacetate hydroxylase (FAD); 4 HPA 3-hydroxylase; p-hydroxyphenylacetate 3-hydroxylase (FAD); HpaB | 4-hydroxyphenylacetate + FADH2 + O2 = 3,4-dihydroxyphenylacetate + FAD + H2O |

  
**Over-represented Metabolite Summary**: Collection of the metabolites identified as substrates or products of the proteins representaed the "Over-represented Enzyme Summary" ranked by frequency of occurrence  

| ID | Structure | Name | Frequency | EC |
| --- | --- | --- | --- | --- |
| cpd:C00001 |  | H2O; Water | 122 | ec:1.2.1.79 ec:3.5.99.3 ec:3.5.1.38 ec:1.13.11.2 ec:1.14.13.149 ec:2.5.1.78 ec:1.14.13.127 ec:1.4.1.1 ec:4.2.1.10 ec:1.4.1.2 ec:3.2.2.1 ec:1.2.1.16 ec:3.1.2.1 ec:4.1.99.12 ec:4.3.3.7 ec:1.8.1.2 ec:1.5.1.5 ec:1.1.1.193 ec:1.14.14.9 ec:1.2.1.20 ec:4.2.1.9 ec:3.5.4.9 ec:3.5.4.25 ec:3.5.4.26 ec:3.2.1.28 ec:1.2.1.60 ec:3.5.4.1 |
| cpd:C05600 |  | 2-Hydroxyhepta-2,4-dienedioate; 2-Hydroxyhepta-2,4-diene-1,7-dioate | 98 | ec:4.1.1.68 |
| cpd:C03063 |  | 2-Oxohept-3-enedioate; 2-Oxohept-3-ene-1,7-dioate | 98 | ec:4.1.1.68 |
| cpd:C04052 |  | 5-Carboxy-2-oxohept-3-enedioate; 5-Oxopent-3-ene-1,2,5-tricarboxylate | 97 | ec:4.1.1.68 ec:5.3.3.10 |
| cpd:C00011 |  | CO2; Carbon dioxide | 79 | ec:4.1.1.68 ec:1.2.4.1 ec:2.2.1.6 ec:1.2.1.18 ec:1.8.1.4 ec:1.2.1.27 |
| cpd:C00080 |  | H+; Hydron | 78 | ec:1.1.1.49 ec:1.5.1.2 ec:1.2.1.79 ec:1.8.1.2 ec:1.5.1.5 ec:1.1.1.193 ec:1.8.1.4 ec:1.14.13.149 ec:1.14.14.9 ec:1.2.1.27 ec:1.2.1.20 ec:1.1.1.157 ec:1.14.13.127 ec:1.4.1.1 ec:3.5.4.9 ec:1.4.1.2 ec:1.2.1.16 ec:3.5.4.26 ec:1.2.1.60 ec:1.2.1.18 ec:1.1.1.100 |
| cpd:C06201 |  | 2,4-Dihydroxyhept-2-enedioate; 2,4-Dihydroxyhept-2-1,7-dioate; 2,4-Dihydroxyhept-2-enedioic acid | 72 | ec:4.1.2.52 |
| cpd:C04186 |  | 5-Carboxymethyl-2-hydroxymuconate | 69 | ec:1.2.1.60 ec:5.3.3.10 |
| cpd:C04642 |  | 2-Hydroxy-5-carboxymethylmuconate semialdehyde; 5-Carboxymethyl-2-hydroxymuconate semialdehyde; 5-Carboxymethyl-2-hydroxymuconic semialdehyde | 64 | ec:1.13.11.15 ec:1.2.1.60 |
| cpd:C00004 |  | NADH; DPNH; Reduced nicotinamide adenine dinucleotide | 57 | ec:1.5.1.2 ec:1.2.1.79 ec:1.8.1.4 ec:1.2.1.27 ec:1.14.14.9 ec:1.2.1.20 ec:1.1.1.157 ec:1.14.13.127 ec:1.4.1.1 ec:1.4.1.2 ec:1.2.1.16 ec:1.2.1.60 ec:1.2.1.18 |
| cpd:C00003 |  | NAD+; NAD; Nicotinamide adenine dinucleotide; DPN; Diphosphopyridine nucleotide; Nadide | 57 | ec:1.5.1.2 ec:1.2.1.79 ec:1.8.1.4 ec:1.2.1.27 ec:1.14.14.9 ec:1.2.1.20 ec:1.1.1.157 ec:1.14.13.127 ec:1.4.1.1 ec:1.4.1.2 ec:1.2.1.16 ec:1.2.1.60 ec:1.2.1.18 |
| cpd:C00022 |  | Pyruvate; Pyruvic acid; 2-Oxopropanoate; 2-Oxopropanoic acid; Pyroracemic acid | 54 | ec:1.4.1.1 ec:1.2.4.1 ec:2.2.1.6 ec:4.1.2.52 ec:4.3.3.7 |
| cpd:C00007 |  | Oxygen; O2 | 45 | ec:1.13.11.15 ec:1.13.11.2 ec:1.13.11.16 ec:1.14.13.149 ec:1.14.14.9 ec:1.14.13.127 |
| cpd:C00232 |  | Succinate semialdehyde; Succinic semialdehyde; 4-Oxobutanoate | 38 | ec:1.2.1.79 ec:1.2.1.16 ec:4.1.2.52 ec:1.2.1.20 |
| cpd:C05601 |  | 4-Hydroxy-2-oxo-heptanedioate; 4-Hydroxy-2-ketopimelate; 4-Hydroxy-2-oxoheptanedioic acid; 4-Hydroxy-2-oxoheptanedioate | 36 | ec:4.1.2.52 |
| cpd:C05364 |  | 4-Carboxy-2-oxo-3-hexenedioate | 35 | ec:5.3.3.10 |
| cpd:C04324 |  | (1E,3E)-4-Hydroxybuta-1,3-diene-1,2,4-tricarboxylate; 2-Hydroxy-4-carboxyhexa-2,4-dienedioate; 4-Carboxy-2-hydroxy-cis,cis-muconate; 4-Carboxy-2-hydroxyhexa-2,4-cis,cis-dienedioate | 35 | ec:5.3.3.10 |
| cpd:C01161 |  | 3,4-Dihydroxyphenylacetate; 3,4-Dihydroxyphenylacetic acid; Homoprotocatechuate | 31 | ec:1.13.11.15 ec:1.14.14.9 |
| cpd:C00006 |  | NADP+; NADP; Nicotinamide adenine dinucleotide phosphate; beta-Nicotinamide adenine dinucleotide phosphate; TPN; Triphosphopyridine nucleotide | 28 | ec:1.1.1.49 ec:1.2.1.79 ec:1.5.1.2 ec:1.8.1.2 ec:1.5.1.5 ec:1.1.1.193 ec:1.14.13.149 ec:1.2.1.27 ec:1.2.1.20 ec:1.1.1.157 ec:3.5.4.9 ec:1.2.1.16 ec:3.5.4.26 ec:1.2.1.18 ec:1.1.1.100 |
| cpd:C00005 |  | NADPH; TPNH; Reduced nicotinamide adenine dinucleotide phosphate | 28 | ec:1.1.1.49 ec:1.2.1.79 ec:1.5.1.2 ec:1.8.1.2 ec:1.5.1.5 ec:1.1.1.193 ec:1.14.13.149 ec:1.2.1.27 ec:1.2.1.20 ec:1.1.1.157 ec:3.5.4.9 ec:1.2.1.16 ec:3.5.4.26 ec:1.2.1.18 ec:1.1.1.100 |
| cpd:C00014 |  | Ammonia; NH3 | 25 | ec:1.4.1.1 ec:1.4.1.2 ec:3.5.1.38 ec:3.5.4.26 ec:1.1.1.193 ec:3.5.4.1 ec:1.8.1.4 ec:4.3.1.7 |
| cpd:C01352 |  | FADH2 | 21 | ec:1.3.8.7 ec:1.1.99.3 |
| cpd:C00016 |  | FAD; Flavin adenine dinucleotide | 21 | ec:1.3.8.7 ec:1.1.99.3 |
| cpd:C00257 |  | D-Gluconic acid; D-Gluconate; D-gluco-Hexonic acid | 20 | ec:1.1.99.3 |
| cpd:C06473 |  | 2-Keto-D-gluconic acid; 2-Dehydro-D-gluconate; 2-Dehydro-D-gluconic acid; alpha-D-arabino-2-Hexulosonic acid | 20 | ec:1.1.99.3 |
| cpd:C00010 |  | CoA; Coenzyme A; CoA-SH | 17 | ec:2.3.1.12 ec:3.1.2.1 ec:1.2.1.18 ec:1.8.1.4 ec:1.2.1.27 |
| cpd:C16255 |  | [Dihydrolipoyllysine-residue acetyltransferase] S-acetyldihydrolipoyllysine; S-Acetyldihydrolipoamide-E | 14 | ec:2.3.1.12 ec:1.2.4.1 |
| cpd:C15973 |  | Enzyme N6-(dihydrolipoyl)lysine; Dihydrolipoamide-E | 12 | ec:2.3.1.12 ec:1.8.1.4 |
| cpd:C15972 |  | Enzyme N6-(lipoyl)lysine; Lipoamide-E | 12 | ec:1.2.4.1 ec:1.8.1.4 |
| cpd:C00024 |  | Acetyl-CoA; Acetyl coenzyme A | 12 | ec:2.3.1.12 ec:3.1.2.1 ec:1.2.1.18 ec:1.2.1.27 |
| cpd:C00143 |  | 5,10-Methylenetetrahydrofolate; (6R)-5,10-Methylenetetrahydrofolate; 5,10-Methylene-THF | 12 | ec:3.5.4.9 ec:1.5.1.5 ec:1.8.1.4 |
| cpd:C05898 |  | Undecaprenyl-diphospho-N-acetylmuramoyl-(N-acetylglucosamine)-L-alanyl-D-glutamyl-meso-2,6-diaminopimeloyl-D-alanyl-D-alanine | 10 | ec:2.4.1.129 |
| cpd:C11827 |  | [GlcNAc-(1->4)-Mur2Ac(oyl-L-Ala-g-D-Glu-A2pm-D-Ala-D-Ala)]n-diphosphoundecaprenol; [GlcNAc-(1->4)-Mur2Ac(oyl-L-Ala-g-D-Glu-A2pm-D-Ala-D-Ala)]n+1-diphosphoundecaprenol | 10 | ec:2.4.1.129 |
| cpd:C04574 |  | di-trans,poly-cis-Undecaprenyl diphosphate; Undecaprenyl diphosphate; Bactoprenyl diphosphate; ditrans,octacis-Undecaprenyl diphosphate | 10 | ec:2.4.1.129 |
| cpd:C05125 |  | 2-(alpha-Hydroxyethyl)thiamine diphosphate; 2-Hydroxyethyl-ThPP | 9 | ec:1.2.4.1 ec:2.2.1.6 |
| cpd:C00068 |  | Thiamin diphosphate; Thiamine diphosphate; Thiamin pyrophosphate; TPP; ThPP | 9 | ec:1.2.4.1 ec:2.2.1.6 |
| cpd:C00041 |  | L-Alanine; L-2-Aminopropionic acid; L-alpha-Alanine | 8 | ec:1.4.1.1 |
| cpd:C00582 |  | Phenylacetyl-CoA; Phenylacetyl coenzyme A | 8 | ec:1.14.13.149 |
| cpd:C00026 |  | 2-Oxoglutarate; Oxoglutaric acid; 2-Ketoglutaric acid; alpha-Ketoglutaric acid | 8 | ec:2.6.1.42 ec:1.4.1.2 ec:2.6.1.17 ec:2.6.1.11 ec:1.8.1.4 |
| cpd:C20062 |  | 2-(1,2-Epoxy-1,2-dihydrophenyl)acetyl-CoA | 8 | ec:1.14.13.149 |
| cpd:C00445 |  | 5,10-Methenyltetrahydrofolate | 7 | ec:3.5.4.9 ec:1.5.1.5 |
| cpd:C00234 |  | 10-Formyltetrahydrofolate; 10-Formyl-THF | 7 | ec:3.5.4.9 ec:1.5.1.5 |
| cpd:C00101 |  | Tetrahydrofolate; 5,6,7,8-Tetrahydrofolate; Tetrahydrofolic acid; THF; (6S)-Tetrahydrofolate; (6S)-Tetrahydrofolic acid; (6S)-THFA | 6 | ec:1.8.1.4 |
| cpd:C00013 |  | Diphosphate; Diphosphoric acid; Pyrophosphate; Pyrophosphoric acid; PPi | 6 | ec:2.7.7.7 ec:6.1.1.1 ec:3.5.4.25 ec:6.1.1.20 ec:4.1.99.12 |
| cpd:C00002 |  | ATP; Adenosine 5'-triphosphate | 6 | ec:6.1.1.1 ec:2.7.1.15 ec:6.1.1.20 ec:2.7.1.2 |
| cpd:C00094 |  | Sulfite; Sulfurous acid | 5 | ec:1.8.1.2 ec:1.13.11.2 |
| cpd:C00091 |  | Succinyl-CoA; Succinyl coenzyme A | 5 | ec:1.8.1.4 |
| cpd:C02972 |  | Dihydrolipoylprotein; [H Protein]-dihydrolipoyllysine | 5 | ec:1.8.1.4 |
| cpd:C00248 |  | Lipoamide; Thioctic acid amide | 5 | ec:1.8.1.4 |
| cpd:C00037 |  | Glycine; Aminoacetic acid; Gly | 5 | ec:1.8.1.4 |
| cpd:C00579 |  | Dihydrolipoamide; Dihydrothioctamide | 5 | ec:1.8.1.4 |
| cpd:C02051 |  | Lipoylprotein; H-Protein-lipoyllysine | 5 | ec:1.8.1.4 |
| cpd:C00084 |  | Acetaldehyde; Ethanal | 4 | ec:4.3.1.7 |
| cpd:C00100 |  | Propanoyl-CoA; Propionyl-CoA; Propionyl coenzyme A | 4 | ec:1.3.8.7 ec:1.2.1.18 ec:1.2.1.27 |
| cpd:C00189 |  | Ethanolamine; Aminoethanol; 2-Hydroxyethylamine | 4 | ec:4.3.1.7 |
| cpd:C00025 |  | L-Glutamate; L-Glutamic acid; L-Glutaminic acid; Glutamate | 4 | ec:2.6.1.42 ec:1.4.1.2 ec:3.5.1.38 ec:2.6.1.17 ec:2.6.1.11 |
| cpd:C00020 |  | AMP; Adenosine 5'-monophosphate; Adenylic acid; Adenylate; 5'-AMP; 5'-Adenylic acid; 5'-Adenosine monophosphate; Adenosine 5'-phosphate | 4 | ec:6.1.1.1 ec:6.1.1.20 |
| cpd:C06002 |  | (S)-Methylmalonate semialdehyde | 3 | ec:1.2.1.18 ec:1.2.1.27 |
| cpd:C02376 |  | 5-Methylcytosine | 3 | ec:3.5.4.1 |
| cpd:C00283 |  | Hydrogen sulfide; Hydrogen-sulfide; H2S; Sulfide | 3 | ec:1.8.1.2 |
| cpd:C00082 |  | L-Tyrosine; (S)-3-(p-Hydroxyphenyl)alanine; (S)-2-Amino-3-(p-hydroxyphenyl)propionic acid; Tyrosine | 3 | ec:6.1.1.1 |
| cpd:C02565 |  | N-Methylhydantoin; N-Methylimidazolidine-2,4-dione | 3 | ec:3.5.4.1 |
| cpd:C00106 |  | Uracil | 3 | ec:3.5.4.1 |
| cpd:C00791 |  | Creatinine; 1-Methylglycocyamidine | 3 | ec:3.5.4.1 |
| cpd:C00787 |  | tRNA(Tyr) | 3 | ec:6.1.1.1 |
| cpd:C00222 |  | 3-Oxopropanoate; Malonate semialdehyde | 3 | ec:1.2.1.18 ec:1.2.1.27 |
| cpd:C00380 |  | Cytosine | 3 | ec:3.5.4.1 |
| cpd:C00178 |  | Thymine; 5-Methyluracil | 3 | ec:3.5.4.1 |
| cpd:C02839 |  | L-Tyrosyl-tRNA(Tyr) | 3 | ec:6.1.1.1 |
| cpd:C01213 |  | (R)-Methylmalonyl-CoA; L-Methylmalonyl-CoA | 3 | ec:1.2.1.18 ec:1.2.1.27 |
| cpd:C06210 |  | 2-Hydroxy-6-keto-2,4-heptadienoate; 2-Hydroxy-6-oxo-hept-2,4-dienoate | 2 | ec:1.13.11.2 |
| cpd:C06010 |  | (S)-2-Acetolactate; (S)-2-Hydroxy-2-methyl-3-oxobutanoate | 2 | ec:2.2.1.6 |
| cpd:C15700 |  | gamma-Glutamyl-gamma-aminobutyraldehyde | 2 |  |
| cpd:C06760 |  | 2-Hydroxy-5-methyl-cis,cis-muconic semialdehyde; (2E,4Z)-2-Hydroxy-5-methyl-6-oxohexa-2,4-dienoate | 2 | ec:1.13.11.2 |
| cpd:C00090 |  | Catechol; 1,2-Benzenediol; o-Benzenediol; 1,2-Dihydroxybenzene; Brenzcatechin; Pyrocatechol | 2 | ec:1.13.11.2 |
| cpd:C06006 |  | (S)-2-Aceto-2-hydroxybutanoate; (S)-2-Hydroxy-2-ethyl-3-oxobutanoate | 2 | ec:2.2.1.6 |
| cpd:C00682 |  | 2-Hydroxymuconate semialdehyde; 2-Hydroxymuconic semialdehyde; 2-Hydroxymuconate-6-semialdehyde | 2 | ec:1.13.11.2 |
| cpd:C00489 |  | Glutarate; Glutaric acid; Pentanedioic acid; 1,3-Propanedicarboxylic acid | 2 | ec:1.2.1.79 ec:1.2.1.16 ec:1.2.1.20 |
| cpd:C02375 |  | 4-Chlorocatechol | 2 | ec:1.13.11.2 |
| cpd:C00121 |  | D-Ribose | 2 | ec:3.2.2.1 ec:2.7.1.15 |
| cpd:C06553 |  | N-Isopropylammelide | 2 | ec:3.5.99.3 |
| cpd:C06552 |  | Hydroxyatrazine; 4-(Ethylamino)-2-hydroxy-6-(isopropylamino)-1,3,5-triazine | 2 | ec:3.5.99.3 |
| cpd:C00671 |  | (S)-3-Methyl-2-oxopentanoic acid; (S)-3-Methyl-2-oxopentanoate; (3S)-3-Methyl-2-oxopentanoic acid; (3S)-3-Methyl-2-oxopentanoate | 2 | ec:2.6.1.42 ec:4.2.1.9 |
| cpd:C00900 |  | 2-Acetolactate | 2 | ec:2.2.1.6 |
| cpd:C00109 |  | 2-Oxobutanoate; 2-Ketobutyric acid; 2-Oxobutyric acid; 2-Oxobutyrate; 2-Oxobutanoic acid; alpha-Ketobutyric acid; alpha-Ketobutyrate | 2 | ec:2.2.1.6 |
| cpd:C06730 |  | 4-Methylcatechol; 3,4-Dihydroxytoluene; 1,2-Dihydroxy-4-methylbenzene; 4-Methyl-1,2-benzenediol | 2 | ec:1.13.11.2 |
| cpd:C01327 |  | Hydrochloric acid; HCl; Hydrogen chloride; Hydrochloride | 2 | ec:3.5.99.3 |
| cpd:C06336 |  | 3-Sulfocatechol; 2,3-Dihydroxybenzenesulfonate | 2 | ec:1.13.11.2 |
| cpd:C04044 |  | 3-(2,3-Dihydroxyphenyl)propanoate; 2,3-Dihydroxyphenylpropanoate | 2 | ec:1.13.11.16 ec:1.14.13.127 |
| cpd:C00042 |  | Succinate; Succinic acid; Butanedionic acid; Ethylenesuccinic acid | 2 | ec:1.2.1.79 ec:1.2.1.16 ec:1.2.1.20 |
| cpd:C01304 |  | 2,5-Diamino-6-(5-phospho-D-ribosylamino)pyrimidin-4(3H)-one; 2,5-Diamino-6-(1-D-ribosylamino)pyrimidin-4(3H)-one 5'-phosphate | 2 | ec:3.5.4.25 ec:3.5.4.26 ec:4.1.99.12 ec:1.1.1.193 |
| cpd:C02923 |  | 2,3-Dihydroxytoluene; 3-Methylcatechol | 2 | ec:1.13.11.2 |
| cpd:C00797 |  | Ethylamine | 2 | ec:3.5.99.3 |
| cpd:C00033 |  | Acetate; Acetic acid; Ethanoic acid | 2 | ec:3.1.2.1 |
| cpd:C00031 |  | D-Glucose; Grape sugar; Dextrose; Glucose; D-Glucopyranose | 2 | ec:3.2.1.28 ec:2.7.1.2 |
| cpd:C00027 |  | Hydrogen peroxide; H2O2; Oxydol | 2 |  |
| cpd:C00021 |  | S-Adenosyl-L-homocysteine; S-Adenosylhomocysteine | 2 | ec:2.1.1.172 |
| cpd:C15556 |  | L-3,4-Dihydroxybutan-2-one 4-phosphate; 1-Deoxy-L-glycero-tetrulose 4-phosphate; 2-Hydroxy-3-oxobutyl phosphate | 2 | ec:3.5.4.25 ec:4.1.99.12 ec:2.5.1.78 |
| cpd:C07089 |  | 5-Chloro-2-hydroxymuconic semialdehyde | 2 | ec:1.13.11.2 |
| cpd:C07087 |  | 2-Hydroxy-6-oxoocta-2,4,7-trienoate | 2 | ec:1.13.11.2 |
| cpd:C07085 |  | 3-Vinylcatechol | 2 | ec:1.13.11.2 |
| cpd:C02501 |  | 2-Hydroxymuconate | 2 | ec:1.13.11.2 |
| cpd:C00019 |  | S-Adenosyl-L-methionine; S-Adenosylmethionine; AdoMet; SAM | 2 | ec:2.1.1.172 |
| cpd:C08735 |  | 2-Chloro-4-hydroxy-6-amino-1,3,5-triazine | 2 | ec:3.5.99.3 |
| cpd:C08734 |  | Ammelide; 2,4-Dihydroxy-6-amino-1,3,5-triazine | 2 | ec:3.5.99.3 |
| cpd:C12623 |  | trans-2,3-Dihydroxycinnamate; (2E)-3-(2,3-Dihydroxyphenyl)prop-2-enoate | 2 | ec:1.13.11.16 ec:1.14.13.127 |
| cpd:C00008 |  | ADP; Adenosine 5'-diphosphate | 2 | ec:2.7.1.15 ec:2.7.1.2 |
| cpd:C15699 |  | gamma-L-Glutamylputrescine | 2 |  |
| cpd:C04732 |  | 5-Amino-6-(1-D-ribitylamino)uracil; 5-Amino-6-(D-ribitylamino)uracil; 6-(1-D-Ribitylamino)-5-amino-2,4-dihydroxypyrimidine; 6-(1-D-Ribitylamino)-5-aminouracil; 4-(1-D-Ribitylamino)-5-amino-2,6-dihydroxypyrimidine | 2 | ec:2.5.1.9 ec:2.5.1.78 |
| cpd:C03273 |  | 5-Oxopentanoate; Glutarate semialdehyde | 2 | ec:1.2.1.79 ec:1.2.1.16 ec:1.2.1.20 |
| cpd:C00944 |  | 3-Dehydroquinate; 3-Dehydroquinic acid; 5-Dehydroquinate; 5-Dehydroquinic acid | 2 | ec:4.2.1.10 |
| cpd:C04332 |  | 6,7-Dimethyl-8-(D-ribityl)lumazine | 2 | ec:2.5.1.9 ec:2.5.1.78 |
| cpd:C02637 |  | 3-Dehydroshikimate | 2 | ec:4.2.1.10 |
| cpd:C01172 |  | beta-D-Glucose 6-phosphate | 2 | ec:1.1.1.49 ec:2.7.1.2 |
| cpd:C00141 |  | 3-Methyl-2-oxobutanoic acid; 3-Methyl-2-oxobutyric acid; 3-Methyl-2-oxobutanoate; 2-Oxo-3-methylbutanoate; 2-Oxoisovalerate; 2-Oxoisopentanoate; alpha-Ketovaline; 2-Ketovaline; 2-Keto-3-methylbutyric acid | 2 | ec:2.6.1.42 ec:4.2.1.9 |
| cpd:C04281 |  | L-1-Pyrroline-3-hydroxy-5-carboxylate; 3-Hydroxy-L-1-pyrroline-5-carboxylate; (3R,5S)-1-Pyrroline-3-hydroxy-5-carboxylate | 1 | ec:1.5.1.2 |
| cpd:C00332 |  | Acetoacetyl-CoA; Acetoacetyl coenzyme A; 3-Acetoacetyl-CoA | 1 | ec:1.1.1.157 |
| cpd:C00136 |  | Butanoyl-CoA; Butyryl-CoA | 1 | ec:1.3.8.7 |
| cpd:C00135 |  | L-Histidine; (S)-alpha-Amino-1H-imidazole-4-propionic acid | 1 |  |
| cpd:C04479 |  | 2-Hydroxy-6-oxonona-2,4-diene-1,9-dioate | 1 | ec:1.13.11.16 |
| cpd:C00294 |  | Inosine | 1 | ec:3.2.2.1 |
| cpd:C00131 |  | dATP; 2'-Deoxyadenosine 5'-triphosphate; Deoxyadenosine 5'-triphosphate; Deoxyadenosine triphosphate | 1 | ec:2.7.7.7 |
| cpd:C00092 |  | D-Glucose 6-phosphate; Glucose 6-phosphate; Robison ester | 1 | ec:2.7.1.2 |
| cpd:C04272 |  | (R)-2,3-Dihydroxy-3-methylbutanoate; (R)-2,3-Dihydroxy-isovalerate; (R)-2,3-Dihydroxy-isovaleric acid; (2R)-2,3-Dihydroxy-3-methylbutanoate | 1 | ec:4.2.1.9 |
| cpd:C06007 |  | (R)-2,3-Dihydroxy-3-methylpentanoate; (R)-2,3-Dihydroxy-3-methylvalerate; (2R,3R)-2,3-Dihydroxy-3-methylpentanoate | 1 | ec:4.2.1.9 |
| cpd:C01157 |  | Hydroxyproline; L-Hydroxyproline; trans-4-Hydroxy-L-proline | 1 | ec:1.5.1.2 |
| cpd:C05694 |  | CH3-R; Methyl-acceptor | 1 |  |
| cpd:C05693 |  | R; Methyl group acceptor | 1 |  |
| cpd:C05692 |  | Se-Adenosyl-L-selenohomocysteine; Se-Adenosylselenohomocysteine | 1 |  |
| cpd:C05691 |  | Se-Adenosylselenomethionine | 1 |  |
| cpd:C01152 |  | N(pi)-Methyl-L-histidine; N-pros-Methyl-L-histidine; 3-Methylhistidine; 1-Methylhistidine | 1 |  |
| cpd:C01944 |  | Octanoyl-CoA | 1 | ec:1.3.8.7 |
| cpd:C00286 |  | dGTP; 2'-Deoxyguanosine 5'-triphosphate; Deoxyguanosine 5'-triphosphate; Deoxyguanosine triphosphate | 1 | ec:2.7.7.7 |
| cpd:C00123 |  | L-Leucine; 2-Amino-4-methylvaleric acid; (2S)-alpha-2-Amino-4-methylvaleric acid; (2S)-alpha-Leucine | 1 | ec:2.6.1.42 |
| cpd:C20377 |  | 3-Hydroxypimeloyl-[acp] methyl ester; 3-Hydroxypimeloyl-[acyl-carrier protein] methyl ester | 1 | ec:1.1.1.100 |
| cpd:C00122 |  | Fumarate; Fumaric acid; trans-Butenedioic acid | 1 | ec:3.5.1.38 |
| cpd:C20376 |  | 3-Ketopimeloyl-[acp] methyl ester; 3-Ketopimeloyl-[acyl-carrier protein] methyl ester | 1 | ec:1.1.1.100 |
| cpd:C20373 |  | 3-Hydroxyglutaryl-[acp] methyl ester; 3-Hydroxyglutaryl-[acyl-carrier protein] methyl ester | 1 | ec:1.1.1.100 |
| cpd:C20372 |  | 3-Ketoglutaryl-[acp] methyl ester; 3-Ketoglutaryl-[acyl-carrier protein] methyl ester | 1 | ec:1.1.1.100 |
| cpd:C04462 |  | N-Succinyl-2-L-amino-6-oxoheptanedioate; N-Succinyl-L-2-amino-6-oxoheptanedioate; N-Succinyl-L-2-amino-6-oxopimelate; N-Succinyl-2-amino-6-oxo-L-pimelic acid; N-Succinyl-epsilon-keto-L-aminopimelic acid; (S)-2-(Succinylamino)-6-oxoheptanedioate | 1 | ec:2.6.1.17 ec:2.6.1.11 |
| cpd:C00877 |  | Crotonoyl-CoA; Crotonyl-CoA; 2-Butenoyl-CoA; trans-But-2-enoyl-CoA; But-2-enoyl-CoA; (E)-But-2-enoyl-CoA | 1 | ec:1.3.8.7 |
| cpd:C00673 |  | 2-Deoxy-D-ribose 5-phosphate | 1 | ec:2.7.1.15 |
| cpd:C01144 |  | (S)-3-Hydroxybutanoyl-CoA; (S)-3-Hydroxybutyryl-CoA | 1 | ec:1.1.1.157 |
| cpd:C00117 |  | D-Ribose 5-phosphate; Ribose 5-phosphate | 1 | ec:2.7.1.15 |
| cpd:C00116 |  | Glycerol; Glycerin; 1,2,3-Trihydroxypropane; 1,2,3-Propanetriol | 1 |  |
| cpd:C00079 |  | L-Phenylalanine; (S)-alpha-Amino-beta-phenylpropionic acid | 1 | ec:6.1.1.20 |
| cpd:C04454 |  | 5-Amino-6-(5'-phospho-D-ribitylamino)uracil; 5-Amino-2,6-dioxy-4-(5'-phospho-D-ribitylamino)pyrimidine; 5-Amino-6-(5-phospho-D-ribitylamino)uracil | 1 | ec:3.5.4.26 ec:1.1.1.193 |
| cpd:C04253 |  | Electron-transferring flavoprotein; Electron-transfer flavoprotein | 1 | ec:1.3.8.7 |
| cpd:C00668 |  | alpha-D-Glucose 6-phosphate | 1 | ec:2.7.1.2 |
| cpd:C03221 |  | 2-trans-Dodecenoyl-CoA; (2E)-Dodec-2-enoyl-CoA; (2E)-Dodecenoyl-CoA | 1 | ec:1.3.8.7 |
| cpd:C00269 |  | CDP-diacylglycerol; CDP-1,2-diacylglycerol; 1,2-Diacyl-sn-glycero-3-cytidine-5'-diphosphate | 1 |  |
| cpd:C11457 |  | 3-(3-Hydroxyphenyl)propanoic acid; Dihydro-3-coumaric acid; 3-Hydroxyphenylpropanoate; 3-(3-Hydroxyphenyl)propanoate | 1 | ec:1.14.13.127 |
| cpd:C05116 |  | 3-Hydroxybutanoyl-CoA; 3-Hydroxybutyryl-CoA | 1 | ec:1.1.1.157 |
| cpd:C00267 |  | alpha-D-Glucose | 1 | ec:2.7.1.2 |
| cpd:C05276 |  | trans-Oct-2-enoyl-CoA; (2E)-Octenoyl-CoA | 1 | ec:1.3.8.7 |
| cpd:C05275 |  | trans-Dec-2-enoyl-CoA; (2E)-Decenoyl-CoA | 1 | ec:1.3.8.7 |
| cpd:C05274 |  | Decanoyl-CoA | 1 | ec:1.3.8.7 |
| cpd:C05273 |  | trans-Tetradec-2-enoyl-CoA; (2E)-Tetradecenoyl-CoA | 1 | ec:1.3.8.7 |
| cpd:C00262 |  | Hypoxanthine; Purine-6-ol | 1 | ec:3.2.2.1 |
| cpd:C05272 |  | trans-Hexadec-2-enoyl-CoA; trans-2-Hexadecenoyl-CoA; (2E)-Hexadecenoyl-CoA | 1 | ec:1.3.8.7 |
| cpd:C05271 |  | trans-Hex-2-enoyl-CoA; (2E)-Hexenoyl-CoA | 1 | ec:1.3.8.7 |
| cpd:C05270 |  | Hexanoyl-CoA | 1 | ec:1.3.8.7 |
| cpd:C00064 |  | L-Glutamine; L-2-Aminoglutaramic acid | 1 | ec:3.5.1.38 |
| cpd:C00459 |  | dTTP; Deoxythymidine triphosphate; Deoxythymidine 5'-triphosphate; TTP | 1 | ec:2.7.7.7 |
| cpd:C00458 |  | dCTP; Deoxycytidine 5'-triphosphate; Deoxycytidine triphosphate; 2'-Deoxycytidine 5'-triphosphate | 1 | ec:2.7.7.7 |
| cpd:C04633 |  | (3R)-3-Hydroxypalmitoyl-[acyl-carrier protein]; (R)-3-Hydroxypalmitoyl-[acyl-carrier protein]; (3R)-3-Hydroxyhexadecanoyl-[acyl-carrier protein]; (R)-3-Hydroxyhexadecanoyl-[acyl-carrier protein] | 1 | ec:1.1.1.100 |
| cpd:C01083 |  | alpha,alpha-Trehalose; alpha,alpha'-Trehalose; Trehalose | 1 | ec:3.2.1.28 |
| cpd:C00255 |  | Riboflavin; Lactoflavin; 7,8-Dimethyl-10-ribitylisoalloxazine; Vitamin B2 | 1 | ec:2.5.1.9 |
| cpd:C15980 |  | (S)-2-Methylbutanoyl-CoA | 1 | ec:1.3.8.7 |
| cpd:C00058 |  | Formate; Methanoic acid; Formic acid | 1 | ec:3.5.4.25 ec:4.1.99.12 |
| cpd:C00055 |  | CMP; Cytidine-5'-monophosphate; Cytidylic acid | 1 |  |
| cpd:C02939 |  | 3-Methylbutanoyl-CoA; Isovaleryl-CoA | 1 | ec:1.3.8.7 |
| cpd:C05659 |  | 5-Methoxytryptamine; 5-MeOT | 1 |  |
| cpd:C04039 |  | 2,3-Dihydroxy-3-methylbutanoate; 2,3-Dihydroxy-isovalerate; 2,3-Dihydroxy-isovaleric acid | 1 | ec:4.2.1.9 |
| cpd:C00642 |  | 4-Hydroxyphenylacetate; 4-Hydroxyphenylacetic acid | 1 | ec:1.14.14.9 |
| cpd:C00441 |  | L-Aspartate 4-semialdehyde; Aspartate beta-semialdehyde; L-Aspartic 4-semialdehyde | 1 | ec:4.3.3.7 |
| cpd:C00440 |  | 5-Methyltetrahydrofolate | 1 |  |
| cpd:C11433 |  | 1-Methoxyphenanthrene | 1 |  |
| cpd:C04620 |  | (3R)-3-Hydroxyoctanoyl-[acyl-carrier protein]; (R)-3-Hydroxyoctanoyl-[acyl-carrier protein] | 1 | ec:1.1.1.100 |
| cpd:C11432 |  | 1-Phenanthrol; 1-Hydroxyphenanthrene | 1 |  |
| cpd:C00049 |  | L-Aspartate; L-Aspartic acid; 2-Aminosuccinic acid; L-Asp | 1 | ec:3.5.1.38 |
| cpd:C00242 |  | Guanine; 2-Amino-6-hydroxypurine | 1 | ec:3.2.2.1 |
| cpd:C00240 |  | rRNA; Ribosomal RNA | 1 | ec:2.1.1.172 |
| cpd:C04421 |  | N-Succinyl-LL-2,6-diaminoheptanedioate; N-Succinyl-LL-2,6-diaminopimelate; N-Succinyl-L-2,6-diaminoheptanedioate; N-Succinyl-L-2,6-diaminopimelate | 1 | ec:2.6.1.17 ec:2.6.1.11 |
| cpd:C00044 |  | GTP; Guanosine 5'-triphosphate | 1 | ec:3.5.4.25 ec:4.1.99.12 |
| cpd:C01268 |  | 5-Amino-6-(5'-phosphoribosylamino)uracil; 5-Amino-6-(ribosylamino)-2,4-(1H,3H)-pyrimidinedione 5'-phosphate; 5-Amino-6-(5-phosphoribosylamino)uracil | 1 | ec:3.5.4.26 ec:1.1.1.193 |
| cpd:C00632 |  | 3-Hydroxyanthranilate; 3-Hydroxyanthranilic acid | 1 |  |
| cpd:C16639 |  | beta-D-Ribofuranose | 1 |  |
| cpd:C00630 |  | 2-Methylpropanoyl-CoA; 2-Methylpropionyl-CoA; Isobutyryl-CoA | 1 | ec:1.3.8.7 |
| cpd:C00437 |  | N-Acetylornithine; N2-Acetyl-L-ornithine | 1 | ec:2.6.1.17 ec:2.6.1.11 |
| cpd:C04619 |  | (3R)-3-Hydroxydecanoyl-[acyl-carrier protein]; (R)-3-Hydroxydecanoyl-[acyl-carrier protein] | 1 | ec:1.1.1.100 |
| cpd:C04618 |  | (3R)-3-Hydroxybutanoyl-[acyl-carrier protein]; (R)-3-Hydroxybutanoyl-[acyl-carrier protein] | 1 | ec:1.1.1.100 |
| cpd:C00430 |  | 5-Aminolevulinate; 5-Amino-4-oxopentanoate; 5-Amino-4-oxovaleric acid | 1 | ec:5.4.3.8 |
| cpd:C00233 |  | 4-Methyl-2-oxopentanoate; 2-Oxoisocaproate | 1 | ec:2.6.1.42 |
| cpd:C00039 |  | DNA; DNAn; DNAn+1; (Deoxyribonucleotide)n; (Deoxyribonucleotide)m; (Deoxyribonucleotide)n+m; Deoxyribonucleic acid | 1 | ec:2.7.7.7 |
| cpd:C03150 |  | Nicotinamide-beta-riboside; N-Ribosylnicotinamide; 1-(beta-D-Ribofuranosyl)nicotinamide | 1 | ec:3.2.2.1 |
| cpd:C14145 |  | (3S)-3-Hydroxyadipyl-CoA | 1 | ec:1.1.1.157 |
| cpd:C00199 |  | D-Ribulose 5-phosphate | 1 | ec:3.5.4.25 ec:4.1.99.12 |
| cpd:C04570 |  | Reduced electron-transferring flavoprotein; Reduced electron-transfer flavoprotein | 1 | ec:1.3.8.7 |
| cpd:C00030 |  | Reduced acceptor; AH2; Hydrogen-donor; Donor | 1 | ec:1.3.8.7 |
| cpd:C03741 |  | (S)-4-Amino-5-oxopentanoate; L-Glutamate 1-semialdehyde | 1 | ec:5.4.3.8 |
| cpd:C05831 |  | 3-Methoxyanthranilate; 3-Methoxyanthranilic acid | 1 |  |
| cpd:C05830 |  | 8-Methoxykynurenate; Xanthurenic acid 8-methyl ether | 1 |  |
| cpd:C03345 |  | 2-Methylbut-2-enoyl-CoA; trans-2-Methylbut-2-enoyl-CoA; Tiglyl-CoA; (E)-2-Methylcrotonoyl-CoA; Methylcrotonoyl-CoA; Methylcrotonyl-CoA; Tigloyl-CoA; 2-Methylcrotanoyl-CoA | 1 | ec:1.3.8.7 |
| cpd:C00780 |  | Serotonin; 3-(2-Aminoethyl)-1H-indol-5-ol; 5-Hydroxytryptamine; Enteramine | 1 |  |
| cpd:C08353 |  | beta-D-Ribopyranose; Ribose | 1 |  |
| cpd:C01250 |  | N-Acetyl-L-glutamate 5-semialdehyde; 2-Acetamido-5-oxopentanoate | 1 | ec:2.6.1.17 ec:2.6.1.11 |
| cpd:C05593 |  | 3-Hydroxyphenylacetate; 3-Hydroxyphenylacetic acid | 1 | ec:1.14.14.9 |
| cpd:C00387 |  | Guanosine | 1 | ec:3.2.2.1 |
| cpd:C00385 |  | Xanthine | 1 | ec:3.2.2.1 |
| cpd:C00028 |  | Acceptor; Hydrogen-acceptor; A; Oxidized donor | 1 | ec:1.3.8.7 |
| cpd:C00221 |  | beta-D-Glucose | 1 | ec:2.7.1.2 |
| cpd:C01648 |  | tRNA(Phe) | 1 | ec:6.1.1.20 |
| cpd:C00819 |  | D-Glutamine; D-2-Aminoglutaramic acid | 1 | ec:3.5.1.38 |
| cpd:C02470 |  | Xanthurenic acid; Xanthurenate | 1 |  |
| cpd:C00183 |  | L-Valine; 2-Amino-3-methylbutyric acid | 1 | ec:2.6.1.42 |
| cpd:C16220 |  | 3-Hydroxyoctadecanoyl-[acp]; 3-Hydroxystearoyl-[acp] | 1 | ec:1.1.1.100 |
| cpd:C05980 |  | Cardiolipin; Diphosphatidylglycerol; 1',3'-Bis(1,2-diacyl-sn-glycero-3-phospho)-sn-glycerol | 1 |  |
| cpd:C00217 |  | D-Glutamate; D-Glutamic acid; D-Glutaminic acid; D-2-Aminoglutaric acid | 1 | ec:3.5.1.38 |
| cpd:C16219 |  | 3-Oxostearoyl-[acp]; 3-Oxooctadecanoyl-[acp]; beta-Ketostearoyl-[acp]; 3-Ketostearoyl-[acp] | 1 | ec:1.1.1.100 |
| cpd:C01832 |  | Lauroyl-CoA; Lauroyl coenzyme A; Dodecanoyl-CoA | 1 | ec:1.3.8.7 |
| cpd:C00212 |  | Adenosine | 1 | ec:3.2.2.1 |
| cpd:C16217 |  | 3-Hydroxyoctadecanoyl-CoA; 3-Hydroxystearoyl-CoA; beta-Hydroxystearoyl-CoA | 1 | ec:1.1.1.100 |
| cpd:C16216 |  | 3-Oxostearoyl-CoA; 3-Oxooctadecanoyl-CoA; beta-Ketostearoyl-CoA; 3-Ketostearoyl-CoA | 1 | ec:1.1.1.100 |
| cpd:C12624 |  | 2-Hydroxy-6-ketononatrienedioate | 1 | ec:1.13.11.16 |
| cpd:C12621 |  | trans-3-Hydroxycinnamate; (2E)-3-(3-Hydroxyphenyl)prop-2-enoate | 1 | ec:1.14.13.127 |
| cpd:C14519 |  | 1-Hydroxypyrene | 1 |  |
| cpd:C01236 |  | D-Glucono-1,5-lactone 6-phosphate; 6-Phospho-D-glucono-1,5-lactone | 1 | ec:1.1.1.49 |
| cpd:C04153 |  | rRNA containing N2-methylguanine | 1 | ec:2.1.1.172 |
| cpd:C18261 |  | 1-Hydroxy-6-methoxypyrene; 6-Methoxy-1-pyrenol | 1 |  |
| cpd:C00407 |  | L-Isoleucine; 2-Amino-3-methylvaleric acid | 1 | ec:2.6.1.42 |
| cpd:C18260 |  | 1,6-Dimethoxypyrene | 1 |  |
| cpd:C00009 |  | Orthophosphate; Phosphate; Phosphoric acid; Orthophosphoric acid | 1 | ec:2.5.1.78 |
| cpd:C03912 |  | (S)-1-Pyrroline-5-carboxylate; L-1-Pyrroline-5-carboxylate; 1-Pyrroline-5-carboxylate | 1 | ec:1.5.1.2 |
| cpd:C20258 |  | (2S,4S)-4-Hydroxy-2,3,4,5-tetrahydrodipicolinate; (2S,4S)-4-Hydroxy-2,3,4,5-tetrahydropyridine-2,6-dicarboxylate; HTPA | 1 | ec:4.3.3.7 |
| cpd:C18259 |  | 1-Methoxypyrene | 1 |  |
| cpd:C03511 |  | L-Phenylalanyl-tRNA(Phe) | 1 | ec:6.1.1.20 |
| cpd:C05762 |  | 3-Oxohexadecanoyl-[acp]; 3-Oxohexadecanoyl-[acyl-carrier protein] | 1 | ec:1.1.1.100 |
| cpd:C06199 |  | Hordenine; 4-[2-(Dimethylamino)ethyl]phenol | 1 |  |
| cpd:C02442 |  | N-Methyltyramine | 1 |  |
| cpd:C00154 |  | Palmitoyl-CoA; Hexadecanoyl-CoA | 1 | ec:1.3.8.7 |
| cpd:C00153 |  | Nicotinamide; Nicotinic acid amide; Niacinamide; Vitamin PP | 1 | ec:3.2.2.1 |
| cpd:C00152 |  | L-Asparagine; 2-Aminosuccinamic acid | 1 | ec:3.5.1.38 |
| cpd:C05759 |  | 3-Oxotetradecanoyl-[acp]; 3-Oxotetradecanoyl-[acyl-carrier protein] | 1 | ec:1.1.1.100 |
| cpd:C05757 |  | (R)-3-Hydroxydodecanoyl-[acp]; (R)-3-Hydroxydodecanoyl-[acyl-carrier protein]; D-3-Hydroxydodecanoyl-[acp]; D-3-Hydroxydodecanoyl-[acyl-carrier protein] | 1 | ec:1.1.1.100 |
| cpd:C05756 |  | 3-Oxododecanoyl-[acp]; 3-Oxododecanoyl-[acyl-carrier protein] | 1 | ec:1.1.1.100 |
| cpd:C05753 |  | 3-Oxodecanoyl-[acp]; 3-Oxodecanoyl-[acyl-carrier protein] | 1 | ec:1.1.1.100 |
| cpd:C05750 |  | 3-Oxooctanoyl-[acp]; 3-Oxooctanoyl-[acyl-carrier protein] | 1 | ec:1.1.1.100 |
| cpd:C03460 |  | 2-Methylprop-2-enoyl-CoA; Methacrylyl-CoA; Methylacrylyl-CoA | 1 | ec:1.3.8.7 |
| cpd:C00344 |  | Phosphatidylglycerol; 3-(3-sn-Phosphatidyl)glycerol; 3(3-Phosphatidyl-)glycerol; PtdGro | 1 |  |
| cpd:C03069 |  | 3-Methylcrotonyl-CoA; 3-Methylbut-2-enoyl-CoA; 3-Methylcrotonoyl-CoA; Dimethylacryloyl-CoA | 1 | ec:1.3.8.7 |
| cpd:C01801 |  | Deoxyribose; 2-Deoxy-D-erythro-pentose; Thyminose; 2-Deoxy-D-ribose | 1 | ec:2.7.1.15 |
| cpd:C04688 |  | (3R)-3-Hydroxytetradecanoyl-[acyl-carrier protein]; (R)-3-Hydroxytetradecanoyl-[acyl-carrier protein]; beta-Hydroxymyristyl-[acyl-carrier protein]; HMA | 1 | ec:1.1.1.100 |
| cpd:C00148 |  | L-Proline; 2-Pyrrolidinecarboxylic acid | 1 | ec:1.5.1.2 |
| cpd:C00147 |  | Adenine; 6-Aminopurine | 1 | ec:3.2.2.1 |
| cpd:C02593 |  | Tetradecanoyl-CoA; Myristoyl-CoA | 1 | ec:1.3.8.7 |
| cpd:C01762 |  | Xanthosine | 1 | ec:3.2.2.1 |
| cpd:C02232 |  | 3-Oxoadipyl-CoA; beta-Ketoadipyl-CoA | 1 | ec:1.1.1.157 |
| cpd:C05747 |  | (R)-3-Hydroxyhexanoyl-[acp]; (R)-3-Hydroxyhexanoyl-[acyl-carrier protein]; D-3-Hydroxyhexanoyl-[acp]; D-3-Hydroxyhexanoyl-[acyl-carrier protein] | 1 | ec:1.1.1.100 |
| cpd:C05746 |  | 3-Oxohexanoyl-[acp]; 3-Oxohexanoyl-[acyl-carrier protein] | 1 | ec:1.1.1.100 |
| cpd:C05744 |  | Acetoacetyl-[acp]; Acetoacetyl-[acyl-carrier protein] | 1 | ec:1.1.1.100 |
| cpd:C00894 |  | Propenoyl-CoA; Acryloyl-CoA; Acrylyl-CoA | 1 | ec:1.3.8.7 |

  
**Over-represented Pathway Summary**: Collection of the KEGG metabolic pathways containing the proteins identified in the "Over-represented Metabolite Summary" ranked by the highest number of hits per pathway  

| Pathway ID | EC | EC Frequency | Name |
| --- | --- | --- | --- |
| map00350 | ec:1.13.11.15 ec:1.14.14.9 ec:1.2.1.60 ec:4.1.1.68 ec:5.3.3.10 ec:4.1.2.52 ec:1.2.1.16 | 200 | path:map00350 Tyrosine metabolism |
| map00030 | ec:1.1.1.49 ec:2.7.1.15 ec:1.1.99.3 | 22 | path:map00030 Pentose phosphate pathway |
| map00620 | ec:2.3.1.12 ec:3.1.2.1 ec:1.2.4.1 ec:1.8.1.4 | 21 | path:map00620 Pyruvate metabolism |
| map00010 | ec:2.3.1.12 ec:2.7.1.2 ec:1.2.4.1 ec:1.8.1.4 | 20 | path:map00010 Glycolysis / Gluconeogenesis |
| map00020 | ec:2.3.1.12 ec:1.2.4.1 ec:1.8.1.4 | 19 | path:map00020 Citrate cycle (TCA cycle) |
| map00670 | ec:1.5.1.5 ec:3.5.4.9 | 14 | path:map00670 One carbon pool by folate |
| map00250 | ec:1.4.1.2 ec:3.5.1.38 ec:1.4.1.1 ec:1.2.1.79 ec:1.2.1.16 | 14 | path:map00250 Alanine, aspartate and glutamate metabolism |
| map00720 | ec:1.5.1.5 ec:3.5.4.9 | 14 | path:map00720 Carbon fixation pathways in prokaryotes |
| map00280 | ec:1.3.8.7 ec:1.2.1.27 ec:2.6.1.42 ec:1.8.1.4 | 10 | path:map00280 Valine, leucine and isoleucine degradation |
| map00550 | ec:2.4.1.129 | 10 | path:map00550 Peptidoglycan biosynthesis |
| map00240 | ec:3.5.4.1 ec:2.7.7.7 | 9 | path:map00240 Pyrimidine metabolism |
| map00430 | ec:1.4.1.2 ec:1.4.1.1 | 9 | path:map00430 Taurine and hypotaurine metabolism |
| map00640 | ec:1.3.8.7 ec:1.2.1.27 ec:1.2.1.18 | 7 | path:map00640 Propanoate metabolism |
| map00650 | ec:2.2.1.6 ec:1.1.1.157 ec:1.2.1.79 ec:1.2.1.16 | 7 | path:map00650 Butanoate metabolism |
| map00330 | ec:1.5.1.2 ec:1.4.1.2 ec:3.5.1.38 ec:3.5.4.1 ec:2.6.1.11 | 7 | path:map00330 Arginine and proline metabolism |
| map00230 | ec:3.2.2.1 ec:2.7.7.7 | 7 | path:map00230 Purine metabolism |
| map00740 | ec:2.5.1.78 ec:2.5.1.9 ec:1.1.1.193 ec:3.5.4.26 ec:4.1.99.12 ec:3.5.4.25 | 6 | path:map00740 Riboflavin metabolism |
| map00360 | ec:1.14.13.127 ec:1.14.13.149 ec:1.1.1.157 ec:1.13.11.16 | 5 | path:map00360 Phenylalanine metabolism |
| map00260 | ec:1.8.1.4 | 5 | path:map00260 Glycine, serine and threonine metabolism |
| map00770 | ec:2.6.1.42 ec:2.2.1.6 ec:4.2.1.9 | 4 | path:map00770 Pantothenate and CoA biosynthesis |
| map00410 | ec:1.3.8.7 ec:1.2.1.18 | 4 | path:map00410 beta-Alanine metabolism |
| map00564 | ec:4.3.1.7 | 4 | path:map00564 Glycerophospholipid metabolism |
| map00970 | ec:6.1.1.1 ec:6.1.1.20 | 4 | path:map00970 Aminoacyl-tRNA biosynthesis |
| map00290 | ec:2.6.1.42 ec:2.2.1.6 ec:4.2.1.9 | 4 | path:map00290 Valine, leucine and isoleucine biosynthesis |
| map00190 | ec:1.9.3.1 | 3 | path:map00190 Oxidative phosphorylation |
| map00920 | ec:1.8.1.2 | 3 | path:map00920 Sulfur metabolism |
| map00562 | ec:1.2.1.18 | 3 | path:map00562 Inositol phosphate metabolism |
| map00362 | ec:1.13.11.2 ec:1.1.1.157 | 3 | path:map00362 Benzoate degradation |
| map00643 | ec:1.13.11.2 | 2 | path:map00643 Styrene degradation |
| map00791 | ec:3.5.99.3 | 2 | path:map00791 Atrazine degradation |
| map00310 | ec:1.2.1.20 | 2 | path:map00310 Lysine degradation |
| map00660 | ec:2.2.1.6 | 2 | path:map00660 C5-Branched dibasic acid metabolism |
| map00500 | ec:2.7.1.2 ec:3.2.1.28 | 2 | path:map00500 Starch and sucrose metabolism |
| map00400 | ec:4.2.1.10 | 2 | path:map00400 Phenylalanine, tyrosine and tryptophan biosynthesis |
| map00622 | ec:1.13.11.2 | 2 | path:map00622 Xylene degradation |
| map00300 | ec:2.6.1.17 ec:4.3.3.7 | 2 | path:map00300 Lysine biosynthesis |
| map00361 | ec:1.13.11.2 | 2 | path:map00361 Chlorocyclohexane and chlorobenzene degradation |
| map00480 | ec:1.1.1.49 | 1 | path:map00480 Glutathione metabolism |
| map00966 | ec:2.6.1.42 | 1 | path:map00966 Glucosinolate biosynthesis |
| map00061 | ec:1.1.1.100 | 1 | path:map00061 Fatty acid biosynthesis |
| map00860 | ec:5.4.3.8 | 1 | path:map00860 Porphyrin and chlorophyll metabolism |
| map00760 | ec:3.2.2.1 | 1 | path:map00760 Nicotinate and nicotinamide metabolism |
| map00471 | ec:3.5.1.38 | 1 | path:map00471 D-Glutamine and D-glutamate metabolism |
| map00052 | ec:2.7.1.2 | 1 | path:map00052 Galactose metabolism |
| map00780 | ec:1.1.1.100 | 1 | path:map00780 Biotin metabolism |
| map00910 | ec:1.4.1.2 | 1 | path:map00910 Nitrogen metabolism |
| map00071 | ec:1.3.8.7 | 1 | path:map00071 Fatty acid degradation |
| map00524 | ec:2.7.1.2 | 1 | path:map00524 Butirosin and neomycin biosynthesis |
| map00521 | ec:2.7.1.2 | 1 | path:map00521 Streptomycin biosynthesis |
| map00520 | ec:2.7.1.2 | 1 | path:map00520 Amino sugar and nucleotide sugar metabolism |

  
Analysis performed on 2014/02/14 23:06:46
