# Supplementary material for: Automated genomic context analysis and experimental validation platform for discovery of prokaryote transcriptional regulator functions
Source: BMC Genomics. 2014 Dec 18;15(1):1142. doi: 10.1186/1471-2164-15-1142 (PMC4349456; doi:10.1186/1471-2164-15-1142)
Supplement: Supplementary file 8 — Additional file 8: Result KynR. Function Discovery V1.0 output (.html format) for the oxidative tryptophan degradation regulator (KynR, Bxe_ A0736). For detailed instructions on how to analyze the results please refer to the Function Discovery V1.0, a gene neighborhood analysis tool section in the Results part of the main text. (HTML 476 KB) [file 12864_2014_6995_MOESM8_ESM.html]

```
ENTRY       Bxe_A0736         CDS       T00340
DEFINITION  AsnC family transcriptional regulator
ORGANISM    bxe  Burkholderia xenovorans
POSITION    1:complement(4065723..4066238)
MOTIF       Pfam: AsnC_trans_reg HTH_24 HTH_AsnC-type MarR HTH_20 MarR_2 HTH_11 HTH_5 HTH_DeoR HTH_CodY HTH_IclR Fe_dep_repress EAP30 Rrf2 TrmB
DBLINKS     NCBI-GI: 91785044
            NCBI-GeneID: 4005674
            JGI: BxeA0736
            UniProt: Q13UP1
AASEQ       171
            MNAISLDATDCRILTVLQQEGRISNLDLAERISLSPSACLRRLRLLEEQGVIEHYRACLN
            REVLGFELEAFVQVSMRNDQENWHERFADAVRDWPEVVGAFVVTGETHYLLRVLAHNLKH
            YSDFVLQRLYKAPGVMDIRSNIVLQTLKEDSGVPVSLVKKASGHGAAHNDR
NTSEQ       516
            atgaacgcgatctcgctcgacgccaccgattgccgtatcttgacggtgcttcagcaagaa
            ggacggatcagcaatctcgacctcgcggagcgtatctcgctctcgccgtcagcctgtctg
            cgacgcctgcgcctgctcgaagagcagggtgtcatcgaacattaccgcgcgtgtctgaac
            cgcgaagtgctgggattcgagctggaagcgttcgtgcaggtgtccatgcgcaacgaccag
            gagaactggcacgagcgctttgcggacgcggtgcgcgactggccggaagtggtcggcgcg
            ttcgtcgtgaccggcgagacacactatctgctgcgcgttctcgcgcacaacctcaagcac
            tattcggatttcgtgctgcagcggctctacaaggcgccgggcgtgatggatatccgttcg
            aatatcgtgctgcagacactcaaggaagattcgggcgtgcccgtttcgttagtgaagaaa
            gctagcggccacggcgccgcgcacaacgatcgttga
///
```

  
**Homolog ID**: Table of closest homologs  

```
                 Homologs                                       len   identity overlap
---------------------------------------------------------------------------------
bpy:Bphyt_3226 AsnC family transcriptional regulator          171     0.977    171 
bge:BC1002_2483 transcriptional regulator, AsnC family        171     0.953    171 
bgf:BC1003_2812 AsnC family transcriptional regulator         171     0.936    171 
bph:Bphy_0509 AsnC family transcriptional regulator           167     0.956    158 
bgl:bglu_1g29370 AsnC family transcriptional regulator        166     0.870    162 
bac:BamMC406_2495 AsnC family transcriptional regulator       166     0.855    159 
bam:Bamb_2624 AsnC family transcriptional regulator           166     0.855    159 
bch:Bcen2424_2576 AsnC family transcriptional regulator       166     0.855    159 
bcj:BCAL2789 AnsC family regulatory protein                   166     0.855    159 
bcm:Bcenmc03_2600 AsnC family transcriptional regulator       166     0.855    159 
bcn:Bcen_1965 AsnC family transcriptional regulator           166     0.855    159 
bur:Bcep18194_A5907 AsnC family transcriptional regulat       166     0.855    159 
bvi:Bcep1808_2698 AsnC family transcriptional regulator       166     0.855    159 
bte:BTH_I0712 AsnC family transcriptional regulator           167     0.868    159 
bmj:BMULJ_02539 AsnC family transcriptional regulator         166     0.849    159 
bmu:Bmul_0721 AsnC family transcriptional regulator           166     0.849    159 
bma:BMA0354 AsnC family transcriptional regulator             167     0.855    159 
bml:BMA10229_A2489 AsnC family transcriptional regulato       167     0.855    159 
bmn:BMA10247_0102 AsnC family transcriptional regulator       167     0.855    159 
bmv:BMASAVP1_A0654 AsnC family transcriptional regulato       167     0.855    159 
bpd:BURPS668_0896 AsnC family transcriptional regulator       167     0.855    159 
bpl:BURPS1106A_0899 AsnC family transcriptional regulat       167     0.855    159 
bpm:BURPS1710b_1056 transcription regulator AsnC              167     0.855    159 
bps:BPSL0849 transcription regulator AsnC                     167     0.855    159 
bpr:GBP346_A0823 transcriptional regulator, AsnC family       167     0.849    159 
brh:RBRH_01087 AsnC family transcriptional regulator          162     0.801    161 
cti:RALTA_A2302 AsnC family transcriptional regulator         161     0.627    161 
rso:RSc0761 transcription regulator protein                   176     0.631    160 
reh:H16_A2813 AsnC family transcriptional regulator           160     0.631    160 
rme:Rmet_2647 AsnC/Lrp family transcriptional regulator       159     0.625    160 
reu:Reut_A0810 AsnC family transcriptional regulator          162     0.617    162 
rsc:RCFBP_20654 AsnC/lrp family transcriptional regulat       174     0.625    160 
rpf:Rpic12D_0777 AsnC family transcriptional regulator        175     0.619    160 
rpi:Rpic_0711 AsnC family transcriptional regulator           175     0.619    160 
rsl:RPSI07_2579 AsnC family transcriptional regulator         174     0.625    160 
pfl:PFL_0761 AsnC family transcriptional regulator            157     0.592    157 
pae:PA2082 transcriptional regulator                          158     0.655    145 
pag:PLES_32391 putative transcriptional regulator             158     0.655    145 
pau:PA14_37580 leucine-responsive regulatory protein          158     0.655    145 
pfs:PFLU5189 putative AsnC family regulatory protein          157     0.594    155 
pap:PSPA7_3205 AsnC family transcriptional regulator          158     0.641    145 
bav:BAV0200 leucine-responsive regulatory protein             163     0.477    153 
axy:AXYL_05264 AsnC family transcriptional regulator          157     0.487    150 
bbr:BB0245 leucine-responsive regulatory protein              177     0.484    153 
bpa:BPP0241 leucine-responsive regulatory protein             187     0.484    153 
bpe:BP0554 leucine-responsive regulatory protein              177     0.484    153 
dno:DNO_0469 transcription regulator AsnC               K     157     0.440    150 
hdn:Hden_1810 AsnC family transcriptional regulator           162     0.406    155 
abb:ABBFA_001491 AsnC family protein                          149     0.417    144 
abn:AB57_2300 transcriptional regulator, AsnC family          149     0.417    144 
aby:ABAYE1598 AsnC family transcriptional regulator           149     0.417    144 
acd:AOLE_07995 transcriptional regulator                      149     0.417    144 
abc:ACICU_02078 transcriptional regulator                     149     0.410    144 
abm:ABSDF1702 AsnC family transcriptional regulator           149     0.410    144 
acb:A1S_1958 transcriptional regulator                        149     0.410    144 
rlt:Rleg2_2720 AsnC family transcriptional regulator          155     0.400    155 
sme:SMc01223 transcriptional regulator                        156     0.401    152 
ret:RHE_CH02974 AsnC family leucine-responsive regulato       207     0.407    150 
ara:Arad_3141 leucine-responsive transcriptional regula       155     0.410    144 
bcs:BCAN_A1539 leucine-responsive regulatory protein          156     0.407    145 
bme:BMEI0510 leucine-responsive regulatory protein            156     0.407    145 
bmi:BMEA_A1553 leucine-responsive regulatory protein          156     0.407    145 
bmr:BMI_I1516 leucine-responsive regulatory protein           156     0.407    145 
bms:BR1502 leucine-responsive regulatory protein              156     0.407    145 
bmt:BSUIS_A1558 leucine-responsive regulatory protein         156     0.407    145 
bov:BOV_1452 leucine-responsive regulatory protein            156     0.407    145 
bmb:BruAb1_1495 Lrp-1, leucine-responsive regulatory pr       156     0.400    145 
bmc:BAbS19_I14170 AsnC family regulatory protein              156     0.400    145 
bmf:BAB1_1521 AsnC family regulatory protein                  156     0.400    145
```

**Neighborhood Representations**: Table of genes in the defined genetic neighborhoods of the entry protein and its closest homologs  
  
**Neighborhood Representations for "bxe:Bxe\_A0736"**  

| ID | Annotation | EC number |
| --- | --- | --- |
| bxe:Bxe\_A0746 | N-acetylmuramoyl-L-alanine amidase (EC:3.5.1.28); K01448 N-acetylmuramoyl-L-alanine amidase [EC:3.5.1.28] | ec:3.5.1.28 |
| bxe:Bxe\_A0745 | DMT superfamily drug/metabolite efflux pump; K15268 O-acetylserine/cysteine efflux transporter |  |
| bxe:Bxe\_A0744 | hypothetical protein; K06911 |  |
| bxe:Bxe\_A0743 | thioredoxin; K05838 putative thioredoxin |  |
| bxe:Bxe\_A0742 | ThiF family protein |  |
| bxe:Bxe\_A0741 | pyridoxamine 5'-phosphate oxidase (EC:1.4.3.5); K00275 pyridoxamine 5'-phosphate oxidase [EC:1.4.3.5] | ec:1.4.3.5 |
| bxe:Bxe\_A0740 | cyclopropane-fatty-acyl-phospholipid synthase (EC:2.1.1.79); K00574 cyclopropane-fatty-acyl-phospholipid synthase [EC:2.1.1.79] | ec:2.1.1.79 |
| bxe:Bxe\_A0739 | hypothetical protein |  |
| bxe:Bxe\_A0738 | peptide methionine sulfoxide reductase (EC:1.8.4.11); K07304 peptide-methionine (S)-S-oxide reductase [EC:1.8.4.11] | ec:1.8.4.11 |
| bxe:Bxe\_A0737 | flavin reductase-like protein |  |
| bxe:Bxe\_A0736 | AsnC family transcriptional regulator |  |
| bxe:Bxe\_A0735 | kynureninase; K07130 arylformamidase [EC:3.5.1.9] | ec:3.5.1.9 |
| bxe:Bxe\_A0734 | kynureninase (EC:3.7.1.3); K01556 kynureninase [EC:3.7.1.3] | ec:3.7.1.3 |
| bxe:Bxe\_A0733 | tryptophan 2,3-dioxygenase (EC:1.13.11.11); K00453 tryptophan 2,3-dioxygenase [EC:1.13.11.11] | ec:1.13.11.11 |
| bxe:Bxe\_A0732 | hypothetical protein |  |
| bxe:Bxe\_A0731 | AraC family transcriptional regulator |  |
| bxe:Bxe\_A0730 | D-arabinitol 4-dehydrogenase (EC:1.1.1.11); K00007 D-arabinitol 4-dehydrogenase [EC:1.1.1.11] | ec:1.1.1.11 |
| bxe:Bxe\_A0729 | xylulokinase (EC:2.7.1.17); K00854 xylulokinase [EC:2.7.1.17] | ec:2.7.1.17 |
| bxe:Bxe\_A0728 | transcriptional regulator |  |
| bxe:Bxe\_A0727 | hypothetical protein |  |
| bxe:Bxe\_A0726 | hypothetical protein |  |

  
**Neighborhood Representations for "bpy:Bphyt\_3226"**  

| ID | Annotation | EC number |
| --- | --- | --- |
| bpy:Bphyt\_3216 | N-acetylmuramoyl-L-alanine amidase (EC:3.5.1.28); K01448 N-acetylmuramoyl-L-alanine amidase [EC:3.5.1.28] | ec:3.5.1.28 |
| bpy:Bphyt\_3217 | hypothetical protein; K15268 O-acetylserine/cysteine efflux transporter |  |
| bpy:Bphyt\_3218 | pirin; K06911 |  |
| bpy:Bphyt\_3219 | thioredoxin; K05838 putative thioredoxin |  |
| bpy:Bphyt\_3220 | UBA/THIF-type NAD/FAD binding protein |  |
| bpy:Bphyt\_3221 | pyridoxamine 5'-phosphate oxidase (EC:1.4.3.5); K00275 pyridoxamine 5'-phosphate oxidase [EC:1.4.3.5] | ec:1.4.3.5 |
| bpy:Bphyt\_3222 | cyclopropane-fatty-acyl-phospholipid synthase (EC:2.1.1.79); K00574 cyclopropane-fatty-acyl-phospholipid synthase [EC:2.1.1.79] | ec:2.1.1.79 |
| bpy:Bphyt\_3223 | hypothetical protein |  |
| bpy:Bphyt\_3224 | peptide methionine sulfoxide reductase (EC:1.8.4.11); K07304 peptide-methionine (S)-S-oxide reductase [EC:1.8.4.11] | ec:1.8.4.11 |
| bpy:Bphyt\_3225 | flavin reductase domain-containing protein; K00492 [EC:1.14.13.-] |  |
| bpy:Bphyt\_3226 | AsnC family transcriptional regulator |  |
| bpy:Bphyt\_3227 | arylformamidase; K07130 arylformamidase [EC:3.5.1.9] | ec:3.5.1.9 |
| bpy:Bphyt\_3228 | kynureninase; K01556 kynureninase [EC:3.7.1.3] | ec:3.7.1.3 |
| bpy:Bphyt\_3229 | tryptophan 2,3-dioxygenase; K00453 tryptophan 2,3-dioxygenase [EC:1.13.11.11] | ec:1.13.11.11 |
| bpy:Bphyt\_3230 | pseudogene |  |
| bpy:Bphyt\_3231 | short chain dehydrogenase |  |
| bpy:Bphyt\_3232 | xylulokinase (EC:2.7.1.17); K00854 xylulokinase [EC:2.7.1.17] | ec:2.7.1.17 |
| bpy:Bphyt\_3233 | alcohol dehydrogenase GroES domain-containing protein; K00008 L-iditol 2-dehydrogenase [EC:1.1.1.14] | ec:1.1.1.14 |
| bpy:Bphyt\_3234 | sugar ABC transporter ATPase (EC:3.6.3.17); K10440 ribose transport system permease protein |  |
| bpy:Bphyt\_3235 | ABC transporter; K10441 ribose transport system ATP-binding protein [EC:3.6.3.17] | ec:3.6.3.17 |
| bpy:Bphyt\_3236 | periplasmic binding protein/LacI transcriptional regulator; K10439 ribose transport system substrate-binding protein |  |

  
**Neighborhood Representations for "bge:BC1002\_2483"**  

| ID | Annotation | EC number |
| --- | --- | --- |
| bge:BC1002\_2473 | N-acetylmuramoyl-L-alanine amidase (EC:3.5.1.28); K01448 N-acetylmuramoyl-L-alanine amidase [EC:3.5.1.28] | ec:3.5.1.28 |
| bge:BC1002\_2474 | hypothetical protein; K15268 O-acetylserine/cysteine efflux transporter |  |
| bge:BC1002\_2475 | pirin; K06911 |  |
| bge:BC1002\_2476 | thioredoxin; K05838 putative thioredoxin |  |
| bge:BC1002\_2477 | UBA/THIF-type NAD/FAD binding protein |  |
| bge:BC1002\_2478 | pyridoxamine 5'-phosphate oxidase (EC:1.4.3.5); K00275 pyridoxamine 5'-phosphate oxidase [EC:1.4.3.5] | ec:1.4.3.5 |
| bge:BC1002\_2479 | cyclopropane-fatty-acyl-phospholipid synthase (EC:2.1.1.79); K00574 cyclopropane-fatty-acyl-phospholipid synthase [EC:2.1.1.79] | ec:2.1.1.79 |
| bge:BC1002\_2480 | hypothetical protein |  |
| bge:BC1002\_2481 | peptide methionine sulfoxide reductase (EC:1.8.4.11); K07304 peptide-methionine (S)-S-oxide reductase [EC:1.8.4.11] | ec:1.8.4.11 |
| bge:BC1002\_2482 | flavin reductase domain-containing FMN-binding protein |  |
| bge:BC1002\_2483 | transcriptional regulator, AsnC family |  |
| bge:BC1002\_2484 | arylformamidase; K07130 arylformamidase [EC:3.5.1.9] | ec:3.5.1.9 |
| bge:BC1002\_2485 | kynureninase; K01556 kynureninase [EC:3.7.1.3] | ec:3.7.1.3 |
| bge:BC1002\_2486 | tryptophan 2,3-dioxygenase; K00453 tryptophan 2,3-dioxygenase [EC:1.13.11.11] | ec:1.13.11.11 |
| bge:BC1002\_2487 | hypothetical protein |  |
| bge:BC1002\_2488 | hypothetical protein |  |
| bge:BC1002\_2489 | hypothetical protein |  |
| bge:BC1002\_2490 | short-chain dehydrogenase/reductase SDR |  |
| bge:BC1002\_2491 | xylulokinase (EC:2.7.1.17); K00854 xylulokinase [EC:2.7.1.17] | ec:2.7.1.17 |
| bge:BC1002\_2492 | alcohol dehydrogenase GroES domain-containing protein; K00008 L-iditol 2-dehydrogenase [EC:1.1.1.14] | ec:1.1.1.14 |
| bge:BC1002\_2493 | inner-membrane translocator; K10440 ribose transport system permease protein |  |

  
**Neighborhood Representations for "bgf:BC1003\_2812"**  

| ID | Annotation | EC number |
| --- | --- | --- |
| bgf:BC1003\_2802 | cell wall hydrolase/autolysin; K01448 N-acetylmuramoyl-L-alanine amidase [EC:3.5.1.28] | ec:3.5.1.28 |
| bgf:BC1003\_2803 | hypothetical protein; K15268 O-acetylserine/cysteine efflux transporter |  |
| bgf:BC1003\_2804 | Pirin domain-containing protein; K06911 |  |
| bgf:BC1003\_2805 | thioredoxin; K05838 putative thioredoxin |  |
| bgf:BC1003\_2806 | UBA/THIF-type NAD/FAD-binding protein |  |
| bgf:BC1003\_2807 | pyridoxamine 5'-phosphate oxidase (EC:1.4.3.5); K00275 pyridoxamine 5'-phosphate oxidase [EC:1.4.3.5] | ec:1.4.3.5 |
| bgf:BC1003\_2808 | cyclopropane-fatty-acyl-phospholipid synthase (EC:2.1.1.79); K00574 cyclopropane-fatty-acyl-phospholipid synthase [EC:2.1.1.79] | ec:2.1.1.79 |
| bgf:BC1003\_2809 | hypothetical protein |  |
| bgf:BC1003\_2810 | peptide methionine sulfoxide reductase (EC:1.8.4.11); K07304 peptide-methionine (S)-S-oxide reductase [EC:1.8.4.11] | ec:1.8.4.11 |
| bgf:BC1003\_2811 | flavin reductase domain-containing FMN-binding protein |  |
| bgf:BC1003\_2812 | AsnC family transcriptional regulator |  |
| bgf:BC1003\_2813 | arylformamidase (EC:3.5.1.9); K07130 arylformamidase [EC:3.5.1.9] | ec:3.5.1.9 |
| bgf:BC1003\_2814 | kynureninase; K01556 kynureninase [EC:3.7.1.3] | ec:3.7.1.3 |
| bgf:BC1003\_2815 | tryptophan 2,3-dioxygenase; K00453 tryptophan 2,3-dioxygenase [EC:1.13.11.11] | ec:1.13.11.11 |
| bgf:BC1003\_2816 | short-chain dehydrogenase/reductase SDR |  |
| bgf:BC1003\_2817 | Xylulokinase (EC:2.7.1.17); K00854 xylulokinase [EC:2.7.1.17] | ec:2.7.1.17 |
| bgf:BC1003\_2818 | alcohol dehydrogenase GroES domain-containing protein; K00008 L-iditol 2-dehydrogenase [EC:1.1.1.14] | ec:1.1.1.14 |
| bgf:BC1003\_2819 | inner-membrane translocator; K10440 ribose transport system permease protein |  |
| bgf:BC1003\_2820 | ABC transporter-like protein; K10441 ribose transport system ATP-binding protein [EC:3.6.3.17] | ec:3.6.3.17 |
| bgf:BC1003\_2821 | periplasmic-binding protein/LacI transcriptional regulator; K10439 ribose transport system substrate-binding protein |  |
| bgf:BC1003\_2822 | AraC family transcriptional regulator |  |

  
**Neighborhood Representations for "bph:Bphy\_0509"**  

| ID | Annotation | EC number |
| --- | --- | --- |
| bph:Bphy\_0499 | AraC family transcriptional regulator |  |
| bph:Bphy\_0500 | periplasmic binding protein/LacI transcriptional regulator; K10439 ribose transport system substrate-binding protein |  |
| bph:Bphy\_0501 | ABC transporter-like protein; K10441 ribose transport system ATP-binding protein [EC:3.6.3.17] | ec:3.6.3.17 |
| bph:Bphy\_0502 | monosaccharide-transporting ATPase (EC:3.6.3.17); K10440 ribose transport system permease protein |  |
| bph:Bphy\_0503 | alcohol dehydrogenase; K00008 L-iditol 2-dehydrogenase [EC:1.1.1.14] | ec:1.1.1.14 |
| bph:Bphy\_0504 | xylulokinase (EC:2.7.1.17); K00854 xylulokinase [EC:2.7.1.17] | ec:2.7.1.17 |
| bph:Bphy\_0505 | short chain dehydrogenase |  |
| bph:Bphy\_0506 | tryptophan 2,3-dioxygenase; K00453 tryptophan 2,3-dioxygenase [EC:1.13.11.11] | ec:1.13.11.11 |
| bph:Bphy\_0507 | kynureninase; K01556 kynureninase [EC:3.7.1.3] | ec:3.7.1.3 |
| bph:Bphy\_0508 | arylformamidase; K07130 arylformamidase [EC:3.5.1.9] | ec:3.5.1.9 |
| bph:Bphy\_0509 | AsnC family transcriptional regulator |  |
| bph:Bphy\_0510 | flavin reductase domain-containing protein; K00492 [EC:1.14.13.-] |  |
| bph:Bphy\_0511 | peptide methionine sulfoxide reductase (EC:1.8.4.11); K07304 peptide-methionine (S)-S-oxide reductase [EC:1.8.4.11] | ec:1.8.4.11 |
| bph:Bphy\_0512 | hypothetical protein |  |
| bph:Bphy\_0513 | cyclopropane-fatty-acyl-phospholipid synthase (EC:2.1.1.79); K00574 cyclopropane-fatty-acyl-phospholipid synthase [EC:2.1.1.79] | ec:2.1.1.79 |
| bph:Bphy\_0514 | pyridoxamine 5'-phosphate oxidase (EC:1.4.3.5); K00275 pyridoxamine 5'-phosphate oxidase [EC:1.4.3.5] | ec:1.4.3.5 |
| bph:Bphy\_0515 | UBA/THIF-type NAD/FAD binding protein |  |
| bph:Bphy\_0516 | thioredoxin; K05838 putative thioredoxin |  |
| bph:Bphy\_0517 | pirin domain-containing protein; K06911 |  |
| bph:Bphy\_0518 | hypothetical protein; K15268 O-acetylserine/cysteine efflux transporter |  |
| bph:Bphy\_0519 | N-acetylmuramoyl-L-alanine amidase (EC:3.5.1.28); K01448 N-acetylmuramoyl-L-alanine amidase [EC:3.5.1.28] | ec:3.5.1.28 |

  
**Neighborhood Representations for "bgl:bglu\_1g29370"**  

| ID | Annotation | EC number |
| --- | --- | --- |
| bgl:bglu\_1g29270 | N-acetylmuramoyl-L-alanine amidase; K01448 N-acetylmuramoyl-L-alanine amidase [EC:3.5.1.28] | ec:3.5.1.28 |
| bgl:bglu\_1g29280 | DME family drug/metabolite transporter; K15268 O-acetylserine/cysteine efflux transporter |  |
| bgl:bglu\_1g29290 | pirin domain-containing protein; K06911 |  |
| bgl:bglu\_1g29300 | Thioredoxin; K05838 putative thioredoxin |  |
| bgl:bglu\_1g29310 | HesA/MoeB/ThiF family protein |  |
| bgl:bglu\_1g29320 | pyridoxamine 5'-phosphate oxidase; K00275 pyridoxamine 5'-phosphate oxidase [EC:1.4.3.5] | ec:1.4.3.5 |
| bgl:bglu\_1g29330 | cyclopropane-fatty-acyl-phospholipid synthase; K00574 cyclopropane-fatty-acyl-phospholipid synthase [EC:2.1.1.79] | ec:2.1.1.79 |
| bgl:bglu\_1g29340 | hypothetical protein |  |
| bgl:bglu\_1g29350 | peptide methionine sulfoxide reductase; K07304 peptide-methionine (S)-S-oxide reductase [EC:1.8.4.11] | ec:1.8.4.11 |
| bgl:bglu\_1g29360 | flavin reductase domain-containing protein |  |
| bgl:bglu\_1g29370 | AsnC family transcriptional regulator |  |
| bgl:bglu\_1g29380 | arylformamidase; K07130 arylformamidase [EC:3.5.1.9] | ec:3.5.1.9 |
| bgl:bglu\_1g29390 | Kynureninase; K01556 kynureninase [EC:3.7.1.3] | ec:3.7.1.3 |
| bgl:bglu\_1g29400 | Tryptophan 2,3-dioxygenase; K00453 tryptophan 2,3-dioxygenase [EC:1.13.11.11] | ec:1.13.11.11 |
| bgl:bglu\_1g29410 | major facilitator family transporter |  |
| bgl:bglu\_1g29420 | mannitol dehydrogenase-like protein; K00007 D-arabinitol 4-dehydrogenase [EC:1.1.1.11] | ec:1.1.1.11 |
| bgl:bglu\_1g29430 | Xylulokinase; K00854 xylulokinase [EC:2.7.1.17] | ec:2.7.1.17 |
| bgl:bglu\_1g29440 | deoR family transcriptional regulator |  |
| bgl:bglu\_1g29450 | ABC transporter carbohydrate uptake transporter-1 family, ATP-binding protein; K10111 multiple sugar transport system ATP-binding protein [EC:3.6.3.-] |  |
| bgl:bglu\_1g29460 | HAD-superfamily hydrolase |  |
| bgl:bglu\_1g29470 | ABC transporter permease; K10229 sorbitol/mannitol transport system permease protein |  |

  
**Neighborhood Representations for "bac:BamMC406\_2495"**  

| ID | Annotation | EC number |
| --- | --- | --- |
| bac:BamMC406\_2485 | thioredoxin; K05838 putative thioredoxin |  |
| bac:BamMC406\_2486 | UBA/THIF-type NAD/FAD binding protein |  |
| bac:BamMC406\_2487 | pyridoxamine 5'-phosphate oxidase (EC:1.4.3.5); K00275 pyridoxamine 5'-phosphate oxidase [EC:1.4.3.5] | ec:1.4.3.5 |
| bac:BamMC406\_2488 | cyclopropane-fatty-acyl-phospholipid synthase (EC:2.1.1.79); K00574 cyclopropane-fatty-acyl-phospholipid synthase [EC:2.1.1.79] | ec:2.1.1.79 |
| bac:BamMC406\_2489 | hypothetical protein |  |
| bac:BamMC406\_2490 | hypothetical protein |  |
| bac:BamMC406\_2491 | peptide methionine sulfoxide reductase (EC:1.8.4.11); K07304 peptide-methionine (S)-S-oxide reductase [EC:1.8.4.11] | ec:1.8.4.11 |
| bac:BamMC406\_2492 | selenium-binding protein; K17285 selenium-binding protein 1 |  |
| bac:BamMC406\_2493 | hypothetical protein |  |
| bac:BamMC406\_2494 | flavin reductase domain-containing protein |  |
| bac:BamMC406\_2495 | AsnC family transcriptional regulator |  |
| bac:BamMC406\_2496 | arylformamidase; K07130 arylformamidase [EC:3.5.1.9] | ec:3.5.1.9 |
| bac:BamMC406\_2497 | kynureninase; K01556 kynureninase [EC:3.7.1.3] | ec:3.7.1.3 |
| bac:BamMC406\_2498 | tryptophan 2,3-dioxygenase; K00453 tryptophan 2,3-dioxygenase [EC:1.13.11.11] | ec:1.13.11.11 |
| bac:BamMC406\_2499 | major facilitator transporter; K08195 MFS transporter, AAHS family, 4-hydroxybenzoate transporter |  |
| bac:BamMC406\_2500 | 2-dehydropantoate 2-reductase (EC:1.1.1.169); K00077 2-dehydropantoate 2-reductase [EC:1.1.1.169] | ec:1.1.1.169 |
| bac:BamMC406\_2501 | aldehyde dehydrogenase; K00141 benzaldehyde dehydrogenase (NAD) [EC:1.2.1.28] | ec:1.2.1.28 |
| bac:BamMC406\_2502 | benzoylformate decarboxylase; K01576 benzoylformate decarboxylase [EC:4.1.1.7] | ec:4.1.1.7 |
| bac:BamMC406\_2503 | LysR family transcriptional regulator |  |
| bac:BamMC406\_2504 | mannitol dehydrogenase domain-containing protein; K00007 D-arabinitol 4-dehydrogenase [EC:1.1.1.11] | ec:1.1.1.11 |
| bac:BamMC406\_2505 | xylulokinase; K00854 xylulokinase [EC:2.7.1.17] | ec:2.7.1.17 |

  
**Neighborhood Representations for "bam:Bamb\_2624"**  

| ID | Annotation | EC number |
| --- | --- | --- |
| bam:Bamb\_2614 | thioredoxin; K05838 putative thioredoxin |  |
| bam:Bamb\_2615 | UBA/THIF-type NAD/FAD binding protein |  |
| bam:Bamb\_2616 | pyridoxamine 5'-phosphate oxidase (EC:1.4.3.5); K00275 pyridoxamine 5'-phosphate oxidase [EC:1.4.3.5] | ec:1.4.3.5 |
| bam:Bamb\_2617 | cyclopropane-fatty-acyl-phospholipid synthase (EC:2.1.1.79); K00574 cyclopropane-fatty-acyl-phospholipid synthase [EC:2.1.1.79] | ec:2.1.1.79 |
| bam:Bamb\_2618 | hypothetical protein |  |
| bam:Bamb\_2619 | peptide methionine sulfoxide reductase; K07304 peptide-methionine (S)-S-oxide reductase [EC:1.8.4.11] | ec:1.8.4.11 |
| bam:Bamb\_2620 | hypothetical protein |  |
| bam:Bamb\_2621 | selenium-binding protein; K17285 selenium-binding protein 1 |  |
| bam:Bamb\_2622 | hypothetical protein |  |
| bam:Bamb\_2623 | flavin reductase domain-containing protein |  |
| bam:Bamb\_2624 | AsnC family transcriptional regulator |  |
| bam:Bamb\_2625 | cyclase family protein; K07130 arylformamidase [EC:3.5.1.9] | ec:3.5.1.9 |
| bam:Bamb\_2626 | kynureninase; K01556 kynureninase [EC:3.7.1.3] | ec:3.7.1.3 |
| bam:Bamb\_2627 | tryptophan 2,3-dioxygenase; K00453 tryptophan 2,3-dioxygenase [EC:1.13.11.11] | ec:1.13.11.11 |
| bam:Bamb\_2628 | major facilitator superfamily transporter; K08195 MFS transporter, AAHS family, 4-hydroxybenzoate transporter |  |
| bam:Bamb\_2629 | 2-dehydropantoate 2-reductase (EC:1.1.1.169); K00077 2-dehydropantoate 2-reductase [EC:1.1.1.169] | ec:1.1.1.169 |
| bam:Bamb\_2630 | aldehyde dehydrogenase; K00141 benzaldehyde dehydrogenase (NAD) [EC:1.2.1.28] | ec:1.2.1.28 |
| bam:Bamb\_2631 | benzoylformate decarboxylase (EC:4.1.1.7); K01576 benzoylformate decarboxylase [EC:4.1.1.7] | ec:4.1.1.7 |
| bam:Bamb\_2632 | LysR family transcriptional regulator |  |
| bam:Bamb\_2633 | mannitol dehydrogenase domain-containing protein; K00007 D-arabinitol 4-dehydrogenase [EC:1.1.1.11] | ec:1.1.1.11 |
| bam:Bamb\_2634 | xylulokinase; K00854 xylulokinase [EC:2.7.1.17] | ec:2.7.1.17 |

  
**Neighborhood Representations for "bch:Bcen2424\_2576"**  

| ID | Annotation | EC number |
| --- | --- | --- |
| bch:Bcen2424\_2566 | thioredoxin; K05838 putative thioredoxin |  |
| bch:Bcen2424\_2567 | UBA/THIF-type NAD/FAD binding protein |  |
| bch:Bcen2424\_2568 | pyridoxamine 5'-phosphate oxidase (EC:1.4.3.5); K00275 pyridoxamine 5'-phosphate oxidase [EC:1.4.3.5] | ec:1.4.3.5 |
| bch:Bcen2424\_2569 | cyclopropane-fatty-acyl-phospholipid synthase (EC:2.1.1.79); K00574 cyclopropane-fatty-acyl-phospholipid synthase [EC:2.1.1.79] | ec:2.1.1.79 |
| bch:Bcen2424\_2570 | hypothetical protein |  |
| bch:Bcen2424\_2571 | peptide methionine sulfoxide reductase (EC:1.8.4.11); K07304 peptide-methionine (S)-S-oxide reductase [EC:1.8.4.11] | ec:1.8.4.11 |
| bch:Bcen2424\_2572 | hypothetical protein |  |
| bch:Bcen2424\_2573 | selenium-binding protein; K17285 selenium-binding protein 1 |  |
| bch:Bcen2424\_2574 | hypothetical protein |  |
| bch:Bcen2424\_2575 | flavin reductase domain-containing protein |  |
| bch:Bcen2424\_2576 | AsnC family transcriptional regulator |  |
| bch:Bcen2424\_2577 | cyclase family protein; K07130 arylformamidase [EC:3.5.1.9] | ec:3.5.1.9 |
| bch:Bcen2424\_2578 | kynureninase; K01556 kynureninase [EC:3.7.1.3] | ec:3.7.1.3 |
| bch:Bcen2424\_2579 | tryptophan 2,3-dioxygenase; K00453 tryptophan 2,3-dioxygenase [EC:1.13.11.11] | ec:1.13.11.11 |
| bch:Bcen2424\_2580 | major facilitator transporter; K08195 MFS transporter, AAHS family, 4-hydroxybenzoate transporter |  |
| bch:Bcen2424\_2581 | 2-dehydropantoate 2-reductase (EC:1.1.1.169); K00077 2-dehydropantoate 2-reductase [EC:1.1.1.169] | ec:1.1.1.169 |
| bch:Bcen2424\_2582 | aldehyde dehydrogenase; K00141 benzaldehyde dehydrogenase (NAD) [EC:1.2.1.28] | ec:1.2.1.28 |
| bch:Bcen2424\_2583 | benzoylformate decarboxylase (EC:4.1.1.7); K01576 benzoylformate decarboxylase [EC:4.1.1.7] | ec:4.1.1.7 |
| bch:Bcen2424\_2584 | LysR family transcriptional regulator |  |
| bch:Bcen2424\_2585 | mannitol dehydrogenase domain-containing protein; K00007 D-arabinitol 4-dehydrogenase [EC:1.1.1.11] | ec:1.1.1.11 |
| bch:Bcen2424\_2586 | xylulokinase; K00854 xylulokinase [EC:2.7.1.17] | ec:2.7.1.17 |

  
**Neighborhood Representations for "bcj:BCAL2789"**  

| ID | Annotation | EC number |
| --- | --- | --- |
| bcj:BCAL2779 | pirin-like protein; K06911 |  |
| bcj:BCAL2780 | putative thioredoxin protein; K05838 putative thioredoxin |  |
| bcj:BCAL2781 | ThiF family protein |  |
| bcj:BCAL2782 | pdxH; pyridoxamine 5'-phosphate oxidase (EC:1.4.3.5); K00275 pyridoxamine 5'-phosphate oxidase [EC:1.4.3.5] | ec:1.4.3.5 |
| bcj:BCAL2783 | putative cyclopropane-fatty-acyl-phospholipid synthase; K00574 cyclopropane-fatty-acyl-phospholipid synthase [EC:2.1.1.79] | ec:2.1.1.79 |
| bcj:BCAL2784 | hypothetical protein |  |
| bcj:BCAL2785 | putative peptide methionine sulfoxide reductase; K07304 peptide-methionine (S)-S-oxide reductase [EC:1.8.4.11] | ec:1.8.4.11 |
| bcj:BCAL2786 | putative selenium-binding protein; K17285 selenium-binding protein 1 |  |
| bcj:BCAL2787 | hypothetical protein |  |
| bcj:BCAL2788 | flavin reductase family protein |  |
| bcj:BCAL2789 | AnsC family regulatory protein |  |
| bcj:BCAL2790 | putative cyclase; K07130 arylformamidase [EC:3.5.1.9] | ec:3.5.1.9 |
| bcj:BCAL2791 | putative kynureninase; K01556 kynureninase [EC:3.7.1.3] | ec:3.7.1.3 |
| bcj:BCAL2792 | putative tryptophan 2,3-dioxygenase; K00453 tryptophan 2,3-dioxygenase [EC:1.13.11.11] | ec:1.13.11.11 |
| bcj:BCAL2793 | major facilitator superfamily protein; K08195 MFS transporter, AAHS family, 4-hydroxybenzoate transporter |  |
| bcj:BCAL2794 | putative ketopantoate reductase; K00077 2-dehydropantoate 2-reductase [EC:1.1.1.169] | ec:1.1.1.169 |
| bcj:BCAL2795 | aldehyde dehydrogenase family protein; K00141 benzaldehyde dehydrogenase (NAD) [EC:1.2.1.28] | ec:1.2.1.28 |
| bcj:BCAL2796 | benzoylformate decarboxylase; K01576 benzoylformate decarboxylase [EC:4.1.1.7] | ec:4.1.1.7 |
| bcj:BCAL2797 | LysR family regulatory protein |  |
| bcj:BCAL2798 | dalD; putative D-arabinitol 4-dehydrogenase (EC:1.1.1.11); K00007 D-arabinitol 4-dehydrogenase [EC:1.1.1.11] | ec:1.1.1.11 |
| bcj:BCAL2799 | dalK; putative carbohydrate kinase; K00854 xylulokinase [EC:2.7.1.17] | ec:2.7.1.17 |

  
**Neighborhood Representations for "bcm:Bcenmc03\_2600"**  

| ID | Annotation | EC number |
| --- | --- | --- |
| bcm:Bcenmc03\_2590 | thioredoxin; K05838 putative thioredoxin |  |
| bcm:Bcenmc03\_2591 | UBA/THIF-type NAD/FAD binding protein |  |
| bcm:Bcenmc03\_2592 | pyridoxamine 5'-phosphate oxidase (EC:1.4.3.5); K00275 pyridoxamine 5'-phosphate oxidase [EC:1.4.3.5] | ec:1.4.3.5 |
| bcm:Bcenmc03\_2593 | cyclopropane-fatty-acyl-phospholipid synthase (EC:2.1.1.79); K00574 cyclopropane-fatty-acyl-phospholipid synthase [EC:2.1.1.79] | ec:2.1.1.79 |
| bcm:Bcenmc03\_2594 | hypothetical protein |  |
| bcm:Bcenmc03\_2595 | peptide methionine sulfoxide reductase (EC:1.8.4.11); K07304 peptide-methionine (S)-S-oxide reductase [EC:1.8.4.11] | ec:1.8.4.11 |
| bcm:Bcenmc03\_2596 | hypothetical protein |  |
| bcm:Bcenmc03\_2597 | selenium-binding protein; K17285 selenium-binding protein 1 |  |
| bcm:Bcenmc03\_2598 | hypothetical protein |  |
| bcm:Bcenmc03\_2599 | flavin reductase domain-containing protein |  |
| bcm:Bcenmc03\_2600 | AsnC family transcriptional regulator |  |
| bcm:Bcenmc03\_2601 | arylformamidase; K07130 arylformamidase [EC:3.5.1.9] | ec:3.5.1.9 |
| bcm:Bcenmc03\_2602 | kynureninase; K01556 kynureninase [EC:3.7.1.3] | ec:3.7.1.3 |
| bcm:Bcenmc03\_2603 | tryptophan 2,3-dioxygenase; K00453 tryptophan 2,3-dioxygenase [EC:1.13.11.11] | ec:1.13.11.11 |
| bcm:Bcenmc03\_2604 | major facilitator transporter; K08195 MFS transporter, AAHS family, 4-hydroxybenzoate transporter |  |
| bcm:Bcenmc03\_2605 | 2-dehydropantoate 2-reductase (EC:1.1.1.169); K00077 2-dehydropantoate 2-reductase [EC:1.1.1.169] | ec:1.1.1.169 |
| bcm:Bcenmc03\_2606 | aldehyde dehydrogenase; K00141 benzaldehyde dehydrogenase (NAD) [EC:1.2.1.28] | ec:1.2.1.28 |
| bcm:Bcenmc03\_2607 | benzoylformate decarboxylase; K01576 benzoylformate decarboxylase [EC:4.1.1.7] | ec:4.1.1.7 |
| bcm:Bcenmc03\_2608 | LysR family transcriptional regulator |  |
| bcm:Bcenmc03\_2609 | mannitol dehydrogenase domain-containing protein; K00007 D-arabinitol 4-dehydrogenase [EC:1.1.1.11] | ec:1.1.1.11 |
| bcm:Bcenmc03\_2610 | xylulokinase; K00854 xylulokinase [EC:2.7.1.17] | ec:2.7.1.17 |

  
**Neighborhood Representations for "bcn:Bcen\_1965"**  

| ID | Annotation | EC number |
| --- | --- | --- |
| bcn:Bcen\_1955 | thioredoxin; K05838 putative thioredoxin |  |
| bcn:Bcen\_1956 | UBA/THIF-type NAD/FAD binding fold |  |
| bcn:Bcen\_1957 | pyridoxamine 5'-phosphate oxidase (EC:1.4.3.5); K00275 pyridoxamine 5'-phosphate oxidase [EC:1.4.3.5] | ec:1.4.3.5 |
| bcn:Bcen\_1958 | cyclopropane-fatty-acyl-phospholipid synthase (EC:2.1.1.79); K00574 cyclopropane-fatty-acyl-phospholipid synthase [EC:2.1.1.79] | ec:2.1.1.79 |
| bcn:Bcen\_1959 | hypothetical protein |  |
| bcn:Bcen\_1960 | peptide methionine sulfoxide reductase (EC:1.8.4.11); K07304 peptide-methionine (S)-S-oxide reductase [EC:1.8.4.11] | ec:1.8.4.11 |
| bcn:Bcen\_1961 | hypothetical protein |  |
| bcn:Bcen\_1962 | selenium-binding protein; K17285 selenium-binding protein 1 |  |
| bcn:Bcen\_1963 | hypothetical protein |  |
| bcn:Bcen\_1964 | flavin reductase-like, FMN-binding |  |
| bcn:Bcen\_1965 | AsnC family transcriptional regulator |  |
| bcn:Bcen\_1966 | cyclase; K07130 arylformamidase [EC:3.5.1.9] | ec:3.5.1.9 |
| bcn:Bcen\_1967 | kynureninase; K01556 kynureninase [EC:3.7.1.3] | ec:3.7.1.3 |
| bcn:Bcen\_1968 | tryptophan 2,3-dioxygenase; K00453 tryptophan 2,3-dioxygenase [EC:1.13.11.11] | ec:1.13.11.11 |
| bcn:Bcen\_1969 | major facilitator transporter; K08195 MFS transporter, AAHS family, 4-hydroxybenzoate transporter |  |
| bcn:Bcen\_1970 | 2-dehydropantoate 2-reductase (EC:1.1.1.169); K00077 2-dehydropantoate 2-reductase [EC:1.1.1.169] | ec:1.1.1.169 |
| bcn:Bcen\_1971 | aldehyde dehydrogenase; K00141 benzaldehyde dehydrogenase (NAD) [EC:1.2.1.28] | ec:1.2.1.28 |
| bcn:Bcen\_1972 | pseudogene |  |
| bcn:Bcen\_1974 | LysR family transcriptional regulator |  |
| bcn:Bcen\_1975 | mannitol dehydrogenase-like protein; K00007 D-arabinitol 4-dehydrogenase [EC:1.1.1.11] | ec:1.1.1.11 |
| bcn:Bcen\_1976 | xylulokinase; K00854 xylulokinase [EC:2.7.1.17] | ec:2.7.1.17 |

  
**Neighborhood Representations for "bur:Bcep18194\_A5907"**  

| ID | Annotation | EC number |
| --- | --- | --- |
| bur:Bcep18194\_A5897 | pirin; K06911 |  |
| bur:Bcep18194\_A5898 | thioredoxin; K05838 putative thioredoxin |  |
| bur:Bcep18194\_A5899 | UBA/THIF-type NAD/FAD binding protein |  |
| bur:Bcep18194\_A5900 | pyridoxamine 5'-phosphate oxidase (EC:1.4.3.5); K00275 pyridoxamine 5'-phosphate oxidase [EC:1.4.3.5] | ec:1.4.3.5 |
| bur:Bcep18194\_A5901 | cyclopropane-fatty-acyl-phospholipid synthase (EC:2.1.1.79); K00574 cyclopropane-fatty-acyl-phospholipid synthase [EC:2.1.1.79] | ec:2.1.1.79 |
| bur:Bcep18194\_A5902 | hypothetical protein |  |
| bur:Bcep18194\_A5903 | protein-methionine-S-oxide reductase (EC:1.8.4.11); K07304 peptide-methionine (S)-S-oxide reductase [EC:1.8.4.11] | ec:1.8.4.11 |
| bur:Bcep18194\_A5904 | selenium-binding protien; K17285 selenium-binding protein 1 |  |
| bur:Bcep18194\_A5905 | hypothetical protein |  |
| bur:Bcep18194\_A5906 | flavin reductase-like, FMN-binding |  |
| bur:Bcep18194\_A5907 | AsnC family transcriptional regulator |  |
| bur:Bcep18194\_A5908 | cyclase; K07130 arylformamidase [EC:3.5.1.9] | ec:3.5.1.9 |
| bur:Bcep18194\_A5909 | kynureninase (EC:3.7.1.3); K01556 kynureninase [EC:3.7.1.3] | ec:3.7.1.3 |
| bur:Bcep18194\_A5910 | tryptophan 2,3-dioxygenase (EC:1.13.11.11); K00453 tryptophan 2,3-dioxygenase [EC:1.13.11.11] | ec:1.13.11.11 |
| bur:Bcep18194\_A5911 | major facilitator transporter; K08195 MFS transporter, AAHS family, 4-hydroxybenzoate transporter |  |
| bur:Bcep18194\_A5912 | 2-dehydropantoate 2-reductase (EC:1.1.1.169); K00077 2-dehydropantoate 2-reductase [EC:1.1.1.169] | ec:1.1.1.169 |
| bur:Bcep18194\_A5913 | aldehyde dehydrogenase (EC:1.2.1.65); K00141 benzaldehyde dehydrogenase (NAD) [EC:1.2.1.28] | ec:1.2.1.28 |
| bur:Bcep18194\_A5914 | benzoylformate decarboxylase (EC:4.1.1.7); K01576 benzoylformate decarboxylase [EC:4.1.1.7] | ec:4.1.1.7 |
| bur:Bcep18194\_A5915 | LysR family transcriptional regulator |  |
| bur:Bcep18194\_A5916 | mannitol dehydrogenase (EC:1.1.1.11); K00007 D-arabinitol 4-dehydrogenase [EC:1.1.1.11] | ec:1.1.1.11 |
| bur:Bcep18194\_A5917 | xylulokinase (EC:2.7.1.17); K00854 xylulokinase [EC:2.7.1.17] | ec:2.7.1.17 |

  
**Neighborhood Representations for "bvi:Bcep1808\_2698"**  

| ID | Annotation | EC number |
| --- | --- | --- |
| bvi:Bcep1808\_2688 | relaxase/mobilization nuclease family protein |  |
| bvi:Bcep1808\_2689 | hypothetical protein |  |
| bvi:Bcep1808\_2690 | hypothetical protein |  |
| bvi:Bcep1808\_2691 | hypothetical protein |  |
| bvi:Bcep1808\_2692 | bacteriophage replication gene A |  |
| bvi:Bcep1808\_2693 | pseudogene |  |
| bvi:Bcep1808\_2694 | hypothetical protein |  |
| bvi:Bcep1808\_2695 | selenium-binding protein; K17285 selenium-binding protein 1 |  |
| bvi:Bcep1808\_2696 | hypothetical protein |  |
| bvi:Bcep1808\_2697 | flavin reductase domain-containing protein |  |
| bvi:Bcep1808\_2698 | AsnC family transcriptional regulator |  |
| bvi:Bcep1808\_2699 | cyclase family protein; K07130 arylformamidase [EC:3.5.1.9] | ec:3.5.1.9 |
| bvi:Bcep1808\_2700 | kynureninase (EC:3.7.1.3); K01556 kynureninase [EC:3.7.1.3] | ec:3.7.1.3 |
| bvi:Bcep1808\_2701 | tryptophan 2,3-dioxygenase (EC:1.13.11.11); K00453 tryptophan 2,3-dioxygenase [EC:1.13.11.11] | ec:1.13.11.11 |
| bvi:Bcep1808\_2702 | mannitol dehydrogenase domain-containing protein; K00007 D-arabinitol 4-dehydrogenase [EC:1.1.1.11] | ec:1.1.1.11 |
| bvi:Bcep1808\_2703 | xylulokinase; K00854 xylulokinase [EC:2.7.1.17] | ec:2.7.1.17 |
| bvi:Bcep1808\_2704 | DeoR family transcriptional regulator |  |
| bvi:Bcep1808\_2705 | ABC transporter-like protein; K10111 multiple sugar transport system ATP-binding protein [EC:3.6.3.-] |  |
| bvi:Bcep1808\_2706 | HAD family hydrolase |  |
| bvi:Bcep1808\_2707 | binding-protein-dependent transport systems inner membrane component; K10229 sorbitol/mannitol transport system permease protein |  |
| bvi:Bcep1808\_2708 | binding-protein-dependent transport systems inner membrane component; K10228 sorbitol/mannitol transport system permease protein |  |

  
**Neighborhood Representations for "bte:BTH\_I0712"**  

| ID | Annotation | EC number |
| --- | --- | --- |
| bte:BTH\_I0702 | xylB-1; xylulokinase (EC:2.7.1.17); K00854 xylulokinase [EC:2.7.1.17] | ec:2.7.1.17 |
| bte:BTH\_I0703 | mannitol dehydrogenase family protein; K00007 D-arabinitol 4-dehydrogenase [EC:1.1.1.11] | ec:1.1.1.11 |
| bte:BTH\_I0704 | LysR family transcriptional regulator |  |
| bte:BTH\_I0705 | benzoylformate decarboxylase (EC:4.1.1.7); K01576 benzoylformate decarboxylase [EC:4.1.1.7] | ec:4.1.1.7 |
| bte:BTH\_I0706 | aldehyde dehydrogenase family protein; K00141 benzaldehyde dehydrogenase (NAD) [EC:1.2.1.28] | ec:1.2.1.28 |
| bte:BTH\_I0707 | panE-1; 2-dehydropantoate 2-reductase (EC:1.1.1.169); K00077 2-dehydropantoate 2-reductase [EC:1.1.1.169] | ec:1.1.1.169 |
| bte:BTH\_I0708 | 4-hydroxybenzoate transporter |  |
| bte:BTH\_I0709 | tryptophan 2,3-dioxygenase family protein; K00453 tryptophan 2,3-dioxygenase [EC:1.13.11.11] | ec:1.13.11.11 |
| bte:BTH\_I0710 | kynU; kynureninase (EC:3.7.1.3); K01556 kynureninase [EC:3.7.1.3] | ec:3.7.1.3 |
| bte:BTH\_I0711 | cyclase; K07130 arylformamidase [EC:3.5.1.9] | ec:3.5.1.9 |
| bte:BTH\_I0712 | AsnC family transcriptional regulator |  |
| bte:BTH\_I0713 | flavin reductase domain-containing protein |  |
| bte:BTH\_I0714 | peptide methionine sulfoxide reductase; K07304 peptide-methionine (S)-S-oxide reductase [EC:1.8.4.11] | ec:1.8.4.11 |
| bte:BTH\_I0715 | hypothetical protein |  |
| bte:BTH\_I0716 | cyclopropane fatty acid synthase family protein; K00574 cyclopropane-fatty-acyl-phospholipid synthase [EC:2.1.1.79] | ec:2.1.1.79 |
| bte:BTH\_I0717 | pdxH; pyridoxamine 5'-phosphate oxidase (EC:1.4.3.5); K00275 pyridoxamine 5'-phosphate oxidase [EC:1.4.3.5] | ec:1.4.3.5 |
| bte:BTH\_I0718 | HesA/MoeB/ThiF family protein |  |
| bte:BTH\_I0719 | thioredoxin; K05838 putative thioredoxin |  |
| bte:BTH\_I0720 | hypothetical protein; K06911 |  |
| bte:BTH\_I0721 | hypothetical protein; K15268 O-acetylserine/cysteine efflux transporter |  |
| bte:BTH\_I0722 | N-acetylmuramoyl-L-alanine amidase; K01448 N-acetylmuramoyl-L-alanine amidase [EC:3.5.1.28] | ec:3.5.1.28 |

  
**Neighborhood Representations for "bmj:BMULJ\_02539"**  

| ID | Annotation | EC number |
| --- | --- | --- |
| bmj:BMULJ\_02529 | putative chromosome condensation protein; K06911 |  |
| bmj:BMULJ\_02530 | ybbN; putative thioredoxin; K05838 putative thioredoxin |  |
| bmj:BMULJ\_02531 | ThiF family protein |  |
| bmj:BMULJ\_02532 | pdxH; pyridoxamine 5'-phosphate oxidase (EC:1.4.3.5); K00275 pyridoxamine 5'-phosphate oxidase [EC:1.4.3.5] | ec:1.4.3.5 |
| bmj:BMULJ\_02533 | cfa; cyclopropane-fatty-acyl-phospholipid synthase (EC:2.1.1.79); K00574 cyclopropane-fatty-acyl-phospholipid synthase [EC:2.1.1.79] | ec:2.1.1.79 |
| bmj:BMULJ\_02534 | hypothetical protein |  |
| bmj:BMULJ\_02535 | msrA; peptide-methionine (S)-S-oxide reductase (EC:1.8.4.11); K07304 peptide-methionine (S)-S-oxide reductase [EC:1.8.4.11] | ec:1.8.4.11 |
| bmj:BMULJ\_02536 | hypothetical protein; K17285 selenium-binding protein 1 |  |
| bmj:BMULJ\_02537 | putative major facilitator superfamily permease |  |
| bmj:BMULJ\_02538 | flavin reductase domain protein (EC:1.14.13.-) |  |
| bmj:BMULJ\_02539 | AsnC family transcriptional regulator |  |
| bmj:BMULJ\_02540 | kynB; N-formyl-L-kynurenine/N-Formylanthranilate amidohydrolase (EC:3.5.1.9); K07130 arylformamidase [EC:3.5.1.9] | ec:3.5.1.9 |
| bmj:BMULJ\_02541 | kynU; kynureninase (EC:3.7.1.3); K01556 kynureninase [EC:3.7.1.3] | ec:3.7.1.3 |
| bmj:BMULJ\_02542 | kynA; tryptophan 2,3-dioxygenase (EC:1.13.11.11); K00453 tryptophan 2,3-dioxygenase [EC:1.13.11.11] | ec:1.13.11.11 |
| bmj:BMULJ\_02543 | putative 4-hydroxybenzoate transporter; K08195 MFS transporter, AAHS family, 4-hydroxybenzoate transporter |  |
| bmj:BMULJ\_02544 | apbA; 2-dehydropantoate 2-reductase (EC:1.1.1.169); K00077 2-dehydropantoate 2-reductase [EC:1.1.1.169] | ec:1.1.1.169 |
| bmj:BMULJ\_02545 | xylC; NAD-dependent benzaldehyde dehydrogenase (EC:1.2.1.28); K00141 benzaldehyde dehydrogenase (NAD) [EC:1.2.1.28] | ec:1.2.1.28 |
| bmj:BMULJ\_02546 | mdlC; benzoylformate decarboxylase (EC:4.1.1.7); K01576 benzoylformate decarboxylase [EC:4.1.1.7] | ec:4.1.1.7 |
| bmj:BMULJ\_02547 | LysR family transcriptional regulator |  |
| bmj:BMULJ\_02548 | mtlK; mannitol 2-dehydrogenase (EC:1.1.1.67); K00007 D-arabinitol 4-dehydrogenase [EC:1.1.1.11] | ec:1.1.1.11 |
| bmj:BMULJ\_02549 | xylB; xylulokinase (EC:2.7.1.17); K00854 xylulokinase [EC:2.7.1.17] | ec:2.7.1.17 |

  
**Neighborhood Representations for "bmu:Bmul\_0721"**  

| ID | Annotation | EC number |
| --- | --- | --- |
| bmu:Bmul\_0711 | xylulokinase; K00854 xylulokinase [EC:2.7.1.17] | ec:2.7.1.17 |
| bmu:Bmul\_0712 | mannitol dehydrogenase domain-containing protein; K00007 D-arabinitol 4-dehydrogenase [EC:1.1.1.11] | ec:1.1.1.11 |
| bmu:Bmul\_0713 | LysR family transcriptional regulator |  |
| bmu:Bmul\_0714 | benzoylformate decarboxylase; K01576 benzoylformate decarboxylase [EC:4.1.1.7] | ec:4.1.1.7 |
| bmu:Bmul\_0715 | aldehyde dehydrogenase; K00141 benzaldehyde dehydrogenase (NAD) [EC:1.2.1.28] | ec:1.2.1.28 |
| bmu:Bmul\_0716 | 2-dehydropantoate 2-reductase (EC:1.1.1.169); K00077 2-dehydropantoate 2-reductase [EC:1.1.1.169] | ec:1.1.1.169 |
| bmu:Bmul\_0717 | major facilitator transporter; K08195 MFS transporter, AAHS family, 4-hydroxybenzoate transporter |  |
| bmu:Bmul\_0718 | tryptophan 2,3-dioxygenase; K00453 tryptophan 2,3-dioxygenase [EC:1.13.11.11] | ec:1.13.11.11 |
| bmu:Bmul\_0719 | kynureninase; K01556 kynureninase [EC:3.7.1.3] | ec:3.7.1.3 |
| bmu:Bmul\_0720 | arylformamidase; K07130 arylformamidase [EC:3.5.1.9] | ec:3.5.1.9 |
| bmu:Bmul\_0721 | AsnC family transcriptional regulator |  |
| bmu:Bmul\_0722 | flavin reductase domain-containing protein |  |
| bmu:Bmul\_0723 | hypothetical protein |  |
| bmu:Bmul\_0724 | selenium-binding protein; K17285 selenium-binding protein 1 |  |
| bmu:Bmul\_0725 | peptide methionine sulfoxide reductase (EC:1.8.4.11); K07304 peptide-methionine (S)-S-oxide reductase [EC:1.8.4.11] | ec:1.8.4.11 |
| bmu:Bmul\_0726 | hypothetical protein |  |
| bmu:Bmul\_0727 | cyclopropane-fatty-acyl-phospholipid synthase (EC:2.1.1.79); K00574 cyclopropane-fatty-acyl-phospholipid synthase [EC:2.1.1.79] | ec:2.1.1.79 |
| bmu:Bmul\_0728 | pyridoxamine 5'-phosphate oxidase (EC:1.4.3.5); K00275 pyridoxamine 5'-phosphate oxidase [EC:1.4.3.5] | ec:1.4.3.5 |
| bmu:Bmul\_0729 | UBA/THIF-type NAD/FAD binding protein |  |
| bmu:Bmul\_0730 | thioredoxin; K05838 putative thioredoxin |  |
| bmu:Bmul\_0731 | pirin domain-containing protein; K06911 |  |

  
**Neighborhood Representations for "bma:BMA0354"**  

| ID | Annotation | EC number |
| --- | --- | --- |
| bma:BMA0344 | mannitol dehydrogenase; K00007 D-arabinitol 4-dehydrogenase [EC:1.1.1.11] | ec:1.1.1.11 |
| bma:BMA0345 | LysR family transcriptional regulator |  |
| bma:BMA0346 | mdlC; benzoylformate decarboxylase (EC:4.1.1.7); K01576 benzoylformate decarboxylase [EC:4.1.1.7] | ec:4.1.1.7 |
| bma:BMA0347 | aldehyde dehydrogenase; K00141 benzaldehyde dehydrogenase (NAD) [EC:1.2.1.28] | ec:1.2.1.28 |
| bma:BMA0348 | panE-1; 2-dehydropantoate 2-reductase (EC:1.1.1.169); K00077 2-dehydropantoate 2-reductase [EC:1.1.1.169] | ec:1.1.1.169 |
| bma:BMA0349 | hypothetical protein |  |
| bma:BMA0350 | 4-hydroxybenzoate transporter |  |
| bma:BMA0351 | tryptophan 2,3-dioxygenase family protein; K00453 tryptophan 2,3-dioxygenase [EC:1.13.11.11] | ec:1.13.11.11 |
| bma:BMA0352 | kynureninase; K01556 kynureninase [EC:3.7.1.3] | ec:3.7.1.3 |
| bma:BMA0353 | cyclase; K07130 arylformamidase [EC:3.5.1.9] | ec:3.5.1.9 |
| bma:BMA0354 | AsnC family transcriptional regulator |  |
| bma:BMA0355 | flavin reductase domain-containing protein; K00492 [EC:1.14.13.-] |  |
| bma:BMA0356 | msrA; peptide methionine sulfoxide reductase (EC:1.8.4.11); K07304 peptide-methionine (S)-S-oxide reductase [EC:1.8.4.11] | ec:1.8.4.11 |
| bma:BMA0357 | hypothetical protein |  |
| bma:BMA0358 | cyclopropane fatty acid synthase family protein; K00574 cyclopropane-fatty-acyl-phospholipid synthase [EC:2.1.1.79] | ec:2.1.1.79 |
| bma:BMA0359 | pdxH; pyridoxamine 5'-phosphate oxidase (EC:1.4.3.5); K00275 pyridoxamine 5'-phosphate oxidase [EC:1.4.3.5] | ec:1.4.3.5 |
| bma:BMA0360 | HesA/MoeB/ThiF family protein |  |
| bma:BMA0361 | pseudogene |  |
| bma:BMA0362 | hypothetical protein; K06911 |  |
| bma:BMA0363 | hypothetical protein; K15268 O-acetylserine/cysteine efflux transporter |  |
| bma:BMA0364 | hypothetical protein |  |

  
**Neighborhood Representations for "bml:BMA10229\_A2489"**  

| ID | Annotation | EC number |
| --- | --- | --- |
| bml:BMA10229\_A2478 | mannitol dehydrogenase; K00007 D-arabinitol 4-dehydrogenase [EC:1.1.1.11] | ec:1.1.1.11 |
| bml:BMA10229\_A2479 | LysR family transcriptional regulator |  |
| bml:BMA10229\_A2480 | mdlC; benzoylformate decarboxylase (EC:4.1.1.7); K01576 benzoylformate decarboxylase [EC:4.1.1.7] | ec:4.1.1.7 |
| bml:BMA10229\_A2481 | aldehyde dehydrogenase; K00141 benzaldehyde dehydrogenase (NAD) [EC:1.2.1.28] | ec:1.2.1.28 |
| bml:BMA10229\_A2482 | panE-1; 2-dehydropantoate 2-reductase (EC:1.1.1.169); K00077 2-dehydropantoate 2-reductase [EC:1.1.1.169] | ec:1.1.1.169 |
| bml:BMA10229\_A2483 | hypothetical protein |  |
| bml:BMA10229\_A2484 | 4-hydroxybenzoate transporter; K08195 MFS transporter, AAHS family, 4-hydroxybenzoate transporter |  |
| bml:BMA10229\_A2485 | kynA; tryptophan 2,3-dioxygenase (EC:1.13.11.11); K00453 tryptophan 2,3-dioxygenase [EC:1.13.11.11] | ec:1.13.11.11 |
| bml:BMA10229\_A2486 | kynU; kynureninase (EC:3.7.1.3); K01556 kynureninase [EC:3.7.1.3] | ec:3.7.1.3 |
| bml:BMA10229\_A2487 | kynB; arylformamidase (EC:3.5.1.9); K07130 arylformamidase [EC:3.5.1.9] | ec:3.5.1.9 |
| bml:BMA10229\_A2489 | AsnC family transcriptional regulator |  |
| bml:BMA10229\_A2488 | flavin reductase domain-containing protein; K00492 [EC:1.14.13.-] |  |
| bml:BMA10229\_A2490 | hypothetical protein |  |
| bml:BMA10229\_A2491 | msrA; peptide methionine sulfoxide reductase (EC:1.8.4.11); K07304 peptide-methionine (S)-S-oxide reductase [EC:1.8.4.11] | ec:1.8.4.11 |
| bml:BMA10229\_A2492 | hypothetical protein |  |
| bml:BMA10229\_A2493 | cyclopropane fatty acid synthase; K00574 cyclopropane-fatty-acyl-phospholipid synthase [EC:2.1.1.79] | ec:2.1.1.79 |
| bml:BMA10229\_A2494 | pdxH; pyridoxamine 5'-phosphate oxidase (EC:1.4.3.5); K00275 pyridoxamine 5'-phosphate oxidase [EC:1.4.3.5] | ec:1.4.3.5 |
| bml:BMA10229\_A2495 | HesA/MoeB/ThiF family protein |  |
| bml:BMA10229\_A2496 | thioredoxin; K05838 putative thioredoxin |  |
| bml:BMA10229\_A2497 | thioredoxin |  |
| bml:BMA10229\_A2498 | hypothetical protein; K06911 |  |

  
**Neighborhood Representations for "bmn:BMA10247\_0102"**  

| ID | Annotation | EC number |
| --- | --- | --- |
| bmn:BMA10247\_0091 | mannitol dehydrogenase; K00007 D-arabinitol 4-dehydrogenase [EC:1.1.1.11] | ec:1.1.1.11 |
| bmn:BMA10247\_0092 | LysR family transcriptional regulator |  |
| bmn:BMA10247\_0093 | mdlC; benzoylformate decarboxylase (EC:4.1.1.7); K01576 benzoylformate decarboxylase [EC:4.1.1.7] | ec:4.1.1.7 |
| bmn:BMA10247\_0094 | aldehyde dehydrogenase; K00141 benzaldehyde dehydrogenase (NAD) [EC:1.2.1.28] | ec:1.2.1.28 |
| bmn:BMA10247\_0095 | panE-1; 2-dehydropantoate 2-reductase (EC:1.1.1.169); K00077 2-dehydropantoate 2-reductase [EC:1.1.1.169] | ec:1.1.1.169 |
| bmn:BMA10247\_0096 | hypothetical protein |  |
| bmn:BMA10247\_0097 | major facilitator family transporter; K08195 MFS transporter, AAHS family, 4-hydroxybenzoate transporter |  |
| bmn:BMA10247\_0098 | kynA; tryptophan 2,3-dioxygenase (EC:1.13.11.11); K00453 tryptophan 2,3-dioxygenase [EC:1.13.11.11] | ec:1.13.11.11 |
| bmn:BMA10247\_0099 | kynU; kynureninase (EC:3.7.1.3); K01556 kynureninase [EC:3.7.1.3] | ec:3.7.1.3 |
| bmn:BMA10247\_0100 | kynB; arylformamidase (EC:3.5.1.9); K07130 arylformamidase [EC:3.5.1.9] | ec:3.5.1.9 |
| bmn:BMA10247\_0102 | AsnC family transcriptional regulator |  |
| bmn:BMA10247\_0101 | flavin reductase domain-containing protein; K00492 [EC:1.14.13.-] |  |
| bmn:BMA10247\_0103 | msrA; peptide methionine sulfoxide reductase (EC:1.8.4.11); K07304 peptide-methionine (S)-S-oxide reductase [EC:1.8.4.11] | ec:1.8.4.11 |
| bmn:BMA10247\_0104 | hypothetical protein |  |
| bmn:BMA10247\_0105 | hypothetical protein |  |
| bmn:BMA10247\_0106 | cfa; cyclopropane-fatty-acyl-phospholipid synthase (EC:2.1.1.79); K00574 cyclopropane-fatty-acyl-phospholipid synthase [EC:2.1.1.79] | ec:2.1.1.79 |
| bmn:BMA10247\_0107 | pdxH; pyridoxamine 5'-phosphate oxidase (EC:1.4.3.5); K00275 pyridoxamine 5'-phosphate oxidase [EC:1.4.3.5] | ec:1.4.3.5 |
| bmn:BMA10247\_0108 | HesA/MoeB/ThiF family protein |  |
| bmn:BMA10247\_0109 | pseudogene |  |
| bmn:BMA10247\_0110 | pseudogene |  |
| bmn:BMA10247\_0111 | hypothetical protein; K06911 |  |

  
**Neighborhood Representations for "bmv:BMASAVP1\_A0654"**  

| ID | Annotation | EC number |
| --- | --- | --- |
| bmv:BMASAVP1\_A0643 | mannitol dehydrogenase family protein; K00007 D-arabinitol 4-dehydrogenase [EC:1.1.1.11] | ec:1.1.1.11 |
| bmv:BMASAVP1\_A0644 | LysR family transcriptional regulator |  |
| bmv:BMASAVP1\_A0645 | mdlC; benzoylformate decarboxylase (EC:4.1.1.7); K01576 benzoylformate decarboxylase [EC:4.1.1.7] | ec:4.1.1.7 |
| bmv:BMASAVP1\_A0646 | aldehyde dehydrogenase family protein; K00141 benzaldehyde dehydrogenase (NAD) [EC:1.2.1.28] | ec:1.2.1.28 |
| bmv:BMASAVP1\_A0647 | panE-1; 2-dehydropantoate 2-reductase (EC:1.1.1.169); K00077 2-dehydropantoate 2-reductase [EC:1.1.1.169] | ec:1.1.1.169 |
| bmv:BMASAVP1\_A0648 | hypothetical protein |  |
| bmv:BMASAVP1\_A0649 | major facilitator family transporter; K08195 MFS transporter, AAHS family, 4-hydroxybenzoate transporter |  |
| bmv:BMASAVP1\_A0650 | kynA; tryptophan 2,3-dioxygenase (EC:1.13.11.11); K00453 tryptophan 2,3-dioxygenase [EC:1.13.11.11] | ec:1.13.11.11 |
| bmv:BMASAVP1\_A0651 | kynU; kynureninase (EC:3.7.1.3); K01556 kynureninase [EC:3.7.1.3] | ec:3.7.1.3 |
| bmv:BMASAVP1\_A0652 | kynB; arylformamidase (EC:3.5.1.9); K07130 arylformamidase [EC:3.5.1.9] | ec:3.5.1.9 |
| bmv:BMASAVP1\_A0654 | AsnC family transcriptional regulator |  |
| bmv:BMASAVP1\_A0653 | flavin reductase domain-containing protein; K00492 [EC:1.14.13.-] |  |
| bmv:BMASAVP1\_A0655 | msrA; peptide methionine sulfoxide reductase (EC:1.8.4.13); K07304 peptide-methionine (S)-S-oxide reductase [EC:1.8.4.11] | ec:1.8.4.11 |
| bmv:BMASAVP1\_A0656 | hypothetical protein |  |
| bmv:BMASAVP1\_A0657 | hypothetical protein |  |
| bmv:BMASAVP1\_A0658 | cfa; cyclopropane-fatty-acyl-phospholipid synthase (EC:2.1.1.79); K00574 cyclopropane-fatty-acyl-phospholipid synthase [EC:2.1.1.79] | ec:2.1.1.79 |
| bmv:BMASAVP1\_A0659 | pdxH; pyridoxamine 5'-phosphate oxidase (EC:1.4.3.5); K00275 pyridoxamine 5'-phosphate oxidase [EC:1.4.3.5] | ec:1.4.3.5 |
| bmv:BMASAVP1\_A0660 | HesA/MoeB/ThiF family protein |  |
| bmv:BMASAVP1\_A0661 | thioredoxin domain-containing protein; K05838 putative thioredoxin |  |
| bmv:BMASAVP1\_A0662 | hypothetical protein; K06911 |  |
| bmv:BMASAVP1\_A0663 | putative transporter; K15268 O-acetylserine/cysteine efflux transporter |  |

  
**Neighborhood Representations for "bpd:BURPS668\_0896"**  

| ID | Annotation | EC number |
| --- | --- | --- |
| bpd:BURPS668\_0885 | mannitol dehydrogenase; K00007 D-arabinitol 4-dehydrogenase [EC:1.1.1.11] | ec:1.1.1.11 |
| bpd:BURPS668\_0886 | LysR family transcriptional regulator |  |
| bpd:BURPS668\_0887 | mdlC; benzoylformate decarboxylase (EC:4.1.1.7); K01576 benzoylformate decarboxylase [EC:4.1.1.7] | ec:4.1.1.7 |
| bpd:BURPS668\_0888 | vanillin dehydrogenase; K00141 benzaldehyde dehydrogenase (NAD) [EC:1.2.1.28] | ec:1.2.1.28 |
| bpd:BURPS668\_0889 | panE; 2-dehydropantoate 2-reductase (EC:1.1.1.169); K00077 2-dehydropantoate 2-reductase [EC:1.1.1.169] | ec:1.1.1.169 |
| bpd:BURPS668\_0890 | hypothetical protein |  |
| bpd:BURPS668\_0891 | major facilitator family transporter; K08195 MFS transporter, AAHS family, 4-hydroxybenzoate transporter |  |
| bpd:BURPS668\_0892 | kynA; tryptophan 2,3-dioxygenase (EC:1.13.11.11); K00453 tryptophan 2,3-dioxygenase [EC:1.13.11.11] | ec:1.13.11.11 |
| bpd:BURPS668\_0893 | kynU; kynureninase (EC:3.7.1.3); K01556 kynureninase [EC:3.7.1.3] | ec:3.7.1.3 |
| bpd:BURPS668\_0894 | kynB; arylformamidase (EC:3.5.1.9); K07130 arylformamidase [EC:3.5.1.9] | ec:3.5.1.9 |
| bpd:BURPS668\_0896 | AsnC family transcriptional regulator |  |
| bpd:BURPS668\_0895 | flavin reductase; K00492 [EC:1.14.13.-] |  |
| bpd:BURPS668\_0897 | msrA; methionine-S-sulfoxide reductase (EC:1.8.4.11); K07304 peptide-methionine (S)-S-oxide reductase [EC:1.8.4.11] | ec:1.8.4.11 |
| bpd:BURPS668\_0898 | 2'-5' RNA ligase |  |
| bpd:BURPS668\_0899 | hypothetical protein |  |
| bpd:BURPS668\_0900 | cfa; cyclopropane-fatty-acyl-phospholipid synthase (EC:2.1.1.79); K00574 cyclopropane-fatty-acyl-phospholipid synthase [EC:2.1.1.79] | ec:2.1.1.79 |
| bpd:BURPS668\_0901 | pdxH; pyridoxamine 5'-phosphate oxidase (EC:1.4.3.5); K00275 pyridoxamine 5'-phosphate oxidase [EC:1.4.3.5] | ec:1.4.3.5 |
| bpd:BURPS668\_0902 | HesA/MoeB/ThiF family protein |  |
| bpd:BURPS668\_0903 | thioredoxin; K05838 putative thioredoxin |  |
| bpd:BURPS668\_0904 | pirin; K06911 |  |
| bpd:BURPS668\_0905 | transporter; K15268 O-acetylserine/cysteine efflux transporter |  |

  
**Neighborhood Representations for "bpl:BURPS1106A\_0899"**  

| ID | Annotation | EC number |
| --- | --- | --- |
| bpl:BURPS1106A\_0888 | mannitol dehydrogenase; K00007 D-arabinitol 4-dehydrogenase [EC:1.1.1.11] | ec:1.1.1.11 |
| bpl:BURPS1106A\_0889 | LysR family transcriptional regulator |  |
| bpl:BURPS1106A\_0890 | mdlC; benzoylformate decarboxylase (EC:4.1.1.7); K01576 benzoylformate decarboxylase [EC:4.1.1.7] | ec:4.1.1.7 |
| bpl:BURPS1106A\_0891 | vanillin dehydrogenase; K00141 benzaldehyde dehydrogenase (NAD) [EC:1.2.1.28] | ec:1.2.1.28 |
| bpl:BURPS1106A\_0892 | panE; 2-dehydropantoate 2-reductase (EC:1.1.1.169); K00077 2-dehydropantoate 2-reductase [EC:1.1.1.169] | ec:1.1.1.169 |
| bpl:BURPS1106A\_0893 | hypothetical protein |  |
| bpl:BURPS1106A\_0894 | major facilitator family transporter; K08195 MFS transporter, AAHS family, 4-hydroxybenzoate transporter |  |
| bpl:BURPS1106A\_0895 | kynA; tryptophan 2,3-dioxygenase (EC:1.13.11.11); K00453 tryptophan 2,3-dioxygenase [EC:1.13.11.11] | ec:1.13.11.11 |
| bpl:BURPS1106A\_0896 | kynU; kynureninase (EC:3.7.1.3); K01556 kynureninase [EC:3.7.1.3] | ec:3.7.1.3 |
| bpl:BURPS1106A\_0897 | kynB; arylformamidase (EC:3.5.1.9); K07130 arylformamidase [EC:3.5.1.9] | ec:3.5.1.9 |
| bpl:BURPS1106A\_0899 | AsnC family transcriptional regulator |  |
| bpl:BURPS1106A\_0898 | flavin reductase family protein; K00492 [EC:1.14.13.-] |  |
| bpl:BURPS1106A\_0900 | msrA; methionine-S-sulfoxide reductase (EC:1.8.4.11); K07304 peptide-methionine (S)-S-oxide reductase [EC:1.8.4.11] | ec:1.8.4.11 |
| bpl:BURPS1106A\_0901 | hypothetical protein |  |
| bpl:BURPS1106A\_0902 | hypothetical protein |  |
| bpl:BURPS1106A\_0903 | cfa; cyclopropane-fatty-acyl-phospholipid synthase (EC:2.1.1.79); K00574 cyclopropane-fatty-acyl-phospholipid synthase [EC:2.1.1.79] | ec:2.1.1.79 |
| bpl:BURPS1106A\_0904 | pdxH; pyridoxamine 5'-phosphate oxidase (EC:1.4.3.5); K00275 pyridoxamine 5'-phosphate oxidase [EC:1.4.3.5] | ec:1.4.3.5 |
| bpl:BURPS1106A\_0905 | HesA/MoeB/ThiF family protein |  |
| bpl:BURPS1106A\_0906 | thioredoxin; K05838 putative thioredoxin |  |
| bpl:BURPS1106A\_0907 | pirin; K06911 |  |
| bpl:BURPS1106A\_0908 | transporter; K15268 O-acetylserine/cysteine efflux transporter |  |

  
**Neighborhood Representations for "bpm:BURPS1710b\_1056"**  

| ID | Annotation | EC number |
| --- | --- | --- |
| bpm:BURPS1710b\_1046 | xylB; xylulokinase (EC:2.7.1.17); K00854 xylulokinase [EC:2.7.1.17] | ec:2.7.1.17 |
| bpm:BURPS1710b\_1047 | dalD; mannitol dehydrogenase (EC:1.1.1.11); K00007 D-arabinitol 4-dehydrogenase [EC:1.1.1.11] | ec:1.1.1.11 |
| bpm:BURPS1710b\_1048 | mdlC; benzoylformate decarboxylase (EC:4.1.1.7); K01576 benzoylformate decarboxylase [EC:4.1.1.7] | ec:4.1.1.7 |
| bpm:BURPS1710b\_1050 | hypothetical protein |  |
| bpm:BURPS1710b\_1049 | vdh; aldehyde dehydrogenase (EC:1.2.1.28); K00141 benzaldehyde dehydrogenase (NAD) [EC:1.2.1.28] | ec:1.2.1.28 |
| bpm:BURPS1710b\_1051 | panE; 2-dehydropantoate 2-reductase (EC:1.1.1.169); K00077 2-dehydropantoate 2-reductase [EC:1.1.1.169] | ec:1.1.1.169 |
| bpm:BURPS1710b\_1052 | pcaK; 4-hydroxybenzoate transporter; K08195 MFS transporter, AAHS family, 4-hydroxybenzoate transporter |  |
| bpm:BURPS1710b\_1053 | tryptophan 2,3-dioxygenase family protein (EC:1.13.11.11); K00453 tryptophan 2,3-dioxygenase [EC:1.13.11.11] | ec:1.13.11.11 |
| bpm:BURPS1710b\_1054 | kynU; kynureninase (EC:3.7.1.3); K01556 kynureninase [EC:3.7.1.3] | ec:3.7.1.3 |
| bpm:BURPS1710b\_1055 | cyclase; K07130 arylformamidase [EC:3.5.1.9] | ec:3.5.1.9 |
| bpm:BURPS1710b\_1056 | transcription regulator AsnC |  |
| bpm:BURPS1710b\_1057 | tesA; flavin reductase domain-containing protein (EC:1.14.13.-); K00492 [EC:1.14.13.-] |  |
| bpm:BURPS1710b\_1058 | peptide methionine sulfoxide reductase (EC:1.8.4.11); K07304 peptide-methionine (S)-S-oxide reductase [EC:1.8.4.11] | ec:1.8.4.11 |
| bpm:BURPS1710b\_1059 | hypothetical protein |  |
| bpm:BURPS1710b\_1060 | cfa2; cyclopropane fatty acid synthase family protein (EC:2.1.1.79); K00574 cyclopropane-fatty-acyl-phospholipid synthase [EC:2.1.1.79] | ec:2.1.1.79 |
| bpm:BURPS1710b\_1061 | pdxH; pyridoxamine 5'-phosphate oxidase (EC:1.4.3.5); K00275 pyridoxamine 5'-phosphate oxidase [EC:1.4.3.5] | ec:1.4.3.5 |
| bpm:BURPS1710b\_1062 | moeB; HesA/MoeB/ThiF family protein |  |
| bpm:BURPS1710b\_1063 | trx; thioredoxin; K05838 putative thioredoxin |  |
| bpm:BURPS1710b\_1064 | amiC; N-acetylmuramoyl-L-alanine amidase (EC:3.5.1.28); K01448 N-acetylmuramoyl-L-alanine amidase [EC:3.5.1.28] | ec:3.5.1.28 |
| bpm:BURPS1710b\_1065 | hypothetical protein; K06925 tRNA threonylcarbamoyladenosine biosynthesis protein TsaE |  |
| bpm:BURPS1710b\_1066 | (Fe-S)-binding protein |  |

  
**Neighborhood Representations for "bps:BPSL0849"**  

| ID | Annotation | EC number |
| --- | --- | --- |
| bps:BPSL0839 | dalK; carbohydrate kinase; K00854 xylulokinase [EC:2.7.1.17] | ec:2.7.1.17 |
| bps:BPSL0840 | dalD; D-arabinitol 4-dehydrogenase (EC:1.1.1.11); K00007 D-arabinitol 4-dehydrogenase [EC:1.1.1.11] | ec:1.1.1.11 |
| bps:BPSL0841 | LysR family transcriptional regulator |  |
| bps:BPSL0842 | benzoylformate decarboxylase (EC:4.1.1.7); K01576 benzoylformate decarboxylase [EC:4.1.1.7] | ec:4.1.1.7 |
| bps:BPSL0843 | aldehyde dehydrogenase; K00141 benzaldehyde dehydrogenase (NAD) [EC:1.2.1.28] | ec:1.2.1.28 |
| bps:BPSL0844 | ketopantoate reductase; K00077 2-dehydropantoate 2-reductase [EC:1.1.1.169] | ec:1.1.1.169 |
| bps:BPSL0845 | transporter protein; K08195 MFS transporter, AAHS family, 4-hydroxybenzoate transporter |  |
| bps:BPSL0846 | tryptophan 2,3-dioxygenase; K00453 tryptophan 2,3-dioxygenase [EC:1.13.11.11] | ec:1.13.11.11 |
| bps:BPSL0847 | hypothetical protein; K01556 kynureninase [EC:3.7.1.3] | ec:3.7.1.3 |
| bps:BPSL0848 | hypothetical protein; K07130 arylformamidase [EC:3.5.1.9] | ec:3.5.1.9 |
| bps:BPSL0849 | transcription regulator AsnC |  |
| bps:BPSL0850 | hypothetical protein; K00492 [EC:1.14.13.-] |  |
| bps:BPSL0851 | peptide methionine sulfoxide reductase (EC:1.8.4.11); K07304 peptide-methionine (S)-S-oxide reductase [EC:1.8.4.11] | ec:1.8.4.11 |
| bps:BPSL0852 | hypothetical protein |  |
| bps:BPSL0853 | cyclopropane-fatty-acyl-phospholipid synthase; K00574 cyclopropane-fatty-acyl-phospholipid synthase [EC:2.1.1.79] | ec:2.1.1.79 |
| bps:BPSL0854 | pdxH; pyridoxamine 5'-phosphate oxidase (EC:1.4.3.5); K00275 pyridoxamine 5'-phosphate oxidase [EC:1.4.3.5] | ec:1.4.3.5 |
| bps:BPSL0855 | ThiF family protein |  |
| bps:BPSL0856 | thioredoxin protein; K05838 putative thioredoxin |  |
| bps:BPSL0857 | hypothetical protein; K06911 |  |
| bps:BPSL0858 | permease; K15268 O-acetylserine/cysteine efflux transporter |  |
| bps:BPSL0859 | N-acetylmuramoyl-L-alanine amidase (EC:3.5.1.28); K01448 N-acetylmuramoyl-L-alanine amidase [EC:3.5.1.28] | ec:3.5.1.28 |

  
**Neighborhood Representations for "bpr:GBP346\_A0823"**  

| ID | Annotation | EC number |
| --- | --- | --- |
| bpr:GBP346\_A0812 | D-arabinitol 4-dehydrogenase; K00007 D-arabinitol 4-dehydrogenase [EC:1.1.1.11] | ec:1.1.1.11 |
| bpr:GBP346\_A0813 | LysR family transcriptional regulator |  |
| bpr:GBP346\_A0814 | benzoylformate decarboxylase (EC:4.1.1.7); K01576 benzoylformate decarboxylase [EC:4.1.1.7] | ec:4.1.1.7 |
| bpr:GBP346\_A0815 | salicylaldehyde dehydrogenase (EC:1.2.1.65); K00141 benzaldehyde dehydrogenase (NAD) [EC:1.2.1.28] | ec:1.2.1.28 |
| bpr:GBP346\_A0816 | panE\_1; 2-dehydropantoate 2-reductase (EC:1.1.1.169); K00077 2-dehydropantoate 2-reductase [EC:1.1.1.169] | ec:1.1.1.169 |
| bpr:GBP346\_A0817 | hypothetical protein |  |
| bpr:GBP346\_A0818 | major facilitator family transporter; K08195 MFS transporter, AAHS family, 4-hydroxybenzoate transporter |  |
| bpr:GBP346\_A0819 | kynA; tryptophan 2,3-dioxygenase (EC:1.13.11.11); K00453 tryptophan 2,3-dioxygenase [EC:1.13.11.11] | ec:1.13.11.11 |
| bpr:GBP346\_A0820 | kynU; kynureninase (EC:3.7.1.3); K01556 kynureninase [EC:3.7.1.3] | ec:3.7.1.3 |
| bpr:GBP346\_A0821 | kynB; arylformamidase (EC:3.5.1.9); K07130 arylformamidase [EC:3.5.1.9] | ec:3.5.1.9 |
| bpr:GBP346\_A0823 | transcriptional regulator, AsnC family |  |
| bpr:GBP346\_A0822 | flavin reductase domain protein |  |
| bpr:GBP346\_A0824 | peptide-methionine (S)-S-oxide reductase; K07304 peptide-methionine (S)-S-oxide reductase [EC:1.8.4.11] | ec:1.8.4.11 |
| bpr:GBP346\_A0825 | 2',5' RNA ligase |  |
| bpr:GBP346\_A0826 | hypothetical protein |  |
| bpr:GBP346\_A0827 | cyclopropane-fatty-acyl-phospholipid synthase; K00574 cyclopropane-fatty-acyl-phospholipid synthase [EC:2.1.1.79] | ec:2.1.1.79 |
| bpr:GBP346\_A0828 | pdxH; pyridoxamine 5'-phosphate oxidase (EC:1.4.3.5); K00275 pyridoxamine 5'-phosphate oxidase [EC:1.4.3.5] | ec:1.4.3.5 |
| bpr:GBP346\_A0829 | ThiF family protein |  |
| bpr:GBP346\_A0830 | thioredoxin; K05838 putative thioredoxin |  |
| bpr:GBP346\_A0831 | pirin domain protein; K06911 |  |
| bpr:GBP346\_A0832 | amino acid metabolite efflux pump; K15268 O-acetylserine/cysteine efflux transporter |  |

  
**Neighborhood Representations for "brh:RBRH\_01087"**  

| ID | Annotation | EC number |
| --- | --- | --- |
| brh:RBRH\_01098 | phosphoglucosamine mutase (EC:5.4.2.10); K03431 phosphoglucosamine mutase [EC:5.4.2.10] | ec:5.4.2.10 |
| brh:RBRH\_01097 | phosphate-binding protein; K02040 phosphate transport system substrate-binding protein |  |
| brh:RBRH\_01096 | phosphate transport system permease pstC; K02037 phosphate transport system permease protein |  |
| brh:RBRH\_01095 | phosphate transport system permease pstA; K02038 phosphate transport system permease protein |  |
| brh:RBRH\_01094 | phosphate transport ATP-binding protein pstB; K02036 phosphate transport system ATP-binding protein [EC:3.6.3.27] | ec:3.6.3.27 |
| brh:RBRH\_01093 | phosphate transport system protein phoU; K02039 phosphate transport system protein |  |
| brh:RBRH\_01092 | phosphate regulon transcriptional regulatory protein PhoB; K07657 two-component system, OmpR family, phosphate regulon response regulator PhoB |  |
| brh:RBRH\_01091 | phosphate regulon sensor protein phoR (EC:2.7.3.-); K07636 two-component system, OmpR family, phosphate regulon sensor histidine kinase PhoR [EC:2.7.13.3] | ec:2.7.13.3 |
| brh:RBRH\_01089 | exopolyphosphatase (EC:3.6.1.11); K01524 exopolyphosphatase / guanosine-5'-triphosphate,3'-diphosphate pyrophosphatase [EC:3.6.1.11 3.6.1.40] | ec:3.6.1.11 ec:3.6.1.40 |
| brh:RBRH\_01088 | glutaryl-CoA dehydrogenase |  |
| brh:RBRH\_01087 | AsnC family transcriptional regulator |  |
| brh:RBRH\_01086 | FMN reductase |  |
| brh:RBRH\_01085 | peptide methionine sulfoxide reductase MsrA (EC:1.8.4.11); K07304 peptide-methionine (S)-S-oxide reductase [EC:1.8.4.11] | ec:1.8.4.11 |
| brh:RBRH\_01084 | hypothetical protein |  |
| brh:RBRH\_01083 | cyclopropane-fatty-acyl-phospholipid synthase (EC:2.1.1.79); K00574 cyclopropane-fatty-acyl-phospholipid synthase [EC:2.1.1.79] | ec:2.1.1.79 |
| brh:RBRH\_01082 | hypothetical protein |  |
| brh:RBRH\_01081 | Pyridoxamine 5'-phosphate oxidase (EC:1.4.3.5); K00275 pyridoxamine 5'-phosphate oxidase [EC:1.4.3.5] | ec:1.4.3.5 |
| brh:RBRH\_01079 | ThiF/MoeB family protein |  |
| brh:RBRH\_01078 | Thioredoxin; K05838 putative thioredoxin |  |
| brh:RBRH\_04014 | hypothetical protein |  |
| brh:RBRH\_01076 | Pirin |  |

  
**Neighborhood Representations for "cti:RALTA\_A2302"**  

| ID | Annotation | EC number |
| --- | --- | --- |
| cti:RALTA\_A2292 | pdxH; pyridoxamine 5'-phosphate oxidase (EC:1.4.3.5); K00275 pyridoxamine 5'-phosphate oxidase [EC:1.4.3.5] | ec:1.4.3.5 |
| cti:RALTA\_A2293 | cfa; cyclopropane-fatty-acyl-phospholipid synthase (EC:2.1.1.79); K00574 cyclopropane-fatty-acyl-phospholipid synthase [EC:2.1.1.79] | ec:2.1.1.79 |
| cti:RALTA\_A2294 | hypothetical protein |  |
| cti:RALTA\_A2295 | msrA; methionine sulfoxide reductase a (EC:1.8.4.11); K07304 peptide-methionine (S)-S-oxide reductase [EC:1.8.4.11] | ec:1.8.4.11 |
| cti:RALTA\_A2296 | o-succinylbenzoate--CoA ligase (EC:6.2.1.26) |  |
| cti:RALTA\_A2297 | acyl-CoA dehydrogenase (EC:1.3.99.-) |  |
| cti:RALTA\_A2298 | hydrolase; 3-oxoadipate enol-lactonase (EC:3.1.1.- 3.1.1.24 3.1.1.1) |  |
| cti:RALTA\_A2299 | hypothetical protein |  |
| cti:RALTA\_A2300 | monooxygenase oxidoreductase flavin reductase-like fmn-binding domain (EC:1.14.-.-) |  |
| cti:RALTA\_A2301 | marr family transcription regulator |  |
| cti:RALTA\_A2302 | AsnC family transcriptional regulator |  |
| cti:RALTA\_A2303 | metal-dependent hydrolase/cyclase; K07130 arylformamidase [EC:3.5.1.9] | ec:3.5.1.9 |
| cti:RALTA\_A2304 | pyridoxal-5'-phosphate (plp)-dependent kynureninase (EC:3.7.1.3); K01556 kynureninase [EC:3.7.1.3] | ec:3.7.1.3 |
| cti:RALTA\_A2305 | tryptophan 2,3-dioxygenase; exported protein (EC:1.13.11.11); K00453 tryptophan 2,3-dioxygenase [EC:1.13.11.11] | ec:1.13.11.11 |
| cti:RALTA\_A2306 | IclR family transcriptional regulator |  |
| cti:RALTA\_A2307 | gcdH; glutaryl-CoA dehydrogenase (EC:1.3.99.7); K00252 glutaryl-CoA dehydrogenase [EC:1.3.8.6] | ec:1.3.8.6 |
| cti:RALTA\_A2308 | antioxidant oxidoreductase; peroxidase peroxiredoxin (EC:1.11.1.7 1.11.1.15) |  |
| cti:RALTA\_A2309 | hypothetical protein |  |
| cti:RALTA\_A2310 | hypothetical protein |  |
| cti:RALTA\_A2311 | hypothetical protein |  |
| cti:RALTA\_A2312 | signaling diguanylate cyclase (ggdef) with eal and pas domains |  |

  
**Neighborhood Representations for "rso:RSc0761"**  

| ID | Annotation | EC number |
| --- | --- | --- |
| rso:RSc0751 | hypothetical protein |  |
| rso:RSc0752 | hypothetical protein |  |
| rso:RSc0753 | hypothetical protein |  |
| rso:RSc0754 | antioxidant oxidoreductase (EC:1.11.1.7) |  |
| rso:RSc0755 | hypothetical protein; K06901 putative MFS transporter, AGZA family, xanthine/uracil permease |  |
| rso:RSc0756 | gcdH; glutaryl-CoA dehydrogenase (EC:1.3.99.7); K00252 glutaryl-CoA dehydrogenase [EC:1.3.8.6] | ec:1.3.8.6 |
| rso:RSc0757 | transcription regulator protein |  |
| rso:RSc0758 | oxygenase oxidoreductase (EC:1.-.-.-); K00453 tryptophan 2,3-dioxygenase [EC:1.13.11.11] | ec:1.13.11.11 |
| rso:RSc0759 | hydrolase (EC:3.-.-.-); K01556 kynureninase [EC:3.7.1.3] | ec:3.7.1.3 |
| rso:RSc0760 | hypothetical protein; K07130 arylformamidase [EC:3.5.1.9] | ec:3.5.1.9 |
| rso:RSc0761 | transcription regulator protein |  |
| rso:RSc0762 | hypothetical protein |  |
| rso:RSc0763 | monooxygenase oxidoreductase (EC:1.14.-.-) |  |
| rso:RSc0764 | msrA; methionine sulfoxide reductase A (EC:1.8.4.11); K07304 peptide-methionine (S)-S-oxide reductase [EC:1.8.4.11] | ec:1.8.4.11 |
| rso:RSc0765 | hypothetical protein |  |
| rso:RSc0766 | cfa2; cyclopropane-fatty-acyl-phospholipid synthase (EC:2.1.1.79); K00574 cyclopropane-fatty-acyl-phospholipid synthase [EC:2.1.1.79] | ec:2.1.1.79 |
| rso:RSc0767 | pdxH1; pyridoxamine 5'-phosphate oxidase (EC:1.4.3.5); K00275 pyridoxamine 5'-phosphate oxidase [EC:1.4.3.5] | ec:1.4.3.5 |
| rso:RSc0768 | hypothetical protein |  |
| rso:RSc0769 | hydrolase glycosidase (EC:3.-.-.-); K01207 beta-N-acetylhexosaminidase [EC:3.2.1.52] | ec:3.2.1.52 |
| rso:RSc0770 | transmembrane dehydrogenase (small subunit) oxidoreductase (EC:1.-.-.-) |  |
| rso:RSc0771 | transmembrane dehydrogenase (large subunit) oxidoreductase (EC:1.-.-.-) |  |

  
**Neighborhood Representations for "reh:H16\_A2813"**  

| ID | Annotation | EC number |
| --- | --- | --- |
| reh:H16\_A2802 | pdxH; pyridoxamine 5'-phosphate oxidase (EC:1.4.3.5); K00275 pyridoxamine 5'-phosphate oxidase [EC:1.4.3.5] | ec:1.4.3.5 |
| reh:H16\_A2803 | h16\_A2803; cyclopropane fatty acid synthase (EC:2.1.1.79); K00574 cyclopropane-fatty-acyl-phospholipid synthase [EC:2.1.1.79] | ec:2.1.1.79 |
| reh:H16\_A2804 | h16\_A2804; hypothetical protein |  |
| reh:H16\_A2806 | h16\_A2806; methionine sulfoxide reductase A (EC:1.8.4.11); K07304 peptide-methionine (S)-S-oxide reductase [EC:1.8.4.11] | ec:1.8.4.11 |
| reh:H16\_A2807 | h16\_A2807; acyl-CoA synthetase (EC:6.2.1.-); K01913 [EC:6.2.1.-] |  |
| reh:H16\_A2808 | h16\_A2808; BEC protein-catalyzes hydroxylation of indole to indoxyl / Acyl-CoA dehydrogenase (EC:1.3.99.-); K00257 [EC:1.3.99.-] |  |
| reh:H16\_A2809 | h16\_A2809; S33 family peptidase (EC:3.-.-.-) |  |
| reh:H16\_A2810 | h16\_A2810; hypothetical protein |  |
| reh:H16\_A2811 | h16\_A2811; hypothetical protein |  |
| reh:H16\_A2812 | h16\_A2812; MarR family transcriptional regulator |  |
| reh:H16\_A2813 | h16\_A2813; AsnC family transcriptional regulator |  |
| reh:H16\_A2814 | h16\_A2814; metal-dependent hydrolase/cyclase; K07130 arylformamidase [EC:3.5.1.9] | ec:3.5.1.9 |
| reh:H16\_A2815 | kynU; L-kynurenine hydrolase (EC:3.7.1.3); K01556 kynureninase [EC:3.7.1.3] | ec:3.7.1.3 |
| reh:H16\_A2816 | tdo1; tryptophan 2,3-dioxygenase (EC:1.13.11.11); K00453 tryptophan 2,3-dioxygenase [EC:1.13.11.11] | ec:1.13.11.11 |
| reh:H16\_A2817 | h16\_A2817; IclR family transcriptional regulator |  |
| reh:H16\_A2818 | gcdH; glutaryl-CoA dehydrogenase (EC:1.3.99.7); K00252 glutaryl-CoA dehydrogenase [EC:1.3.8.6] | ec:1.3.8.6 |
| reh:H16\_A2819 | h16\_A2819; peroxiredoxin (EC:1.11.1.-) |  |
| reh:H16\_A2820 | h16\_A2820; hypothetical protein |  |
| reh:H16\_A2821 | h16\_A2821; hypothetical protein |  |
| reh:H16\_A2822 | h16\_A2822; hypothetical protein |  |
| reh:H16\_A2823 | h16\_A2823; signal transduction protein |  |

  
**Neighborhood Representations for "rme:Rmet\_2647"**  

| ID | Annotation | EC number |
| --- | --- | --- |
| rme:Rmet\_2637 | cation-binding hemerythrin HHE family protein |  |
| rme:Rmet\_2638 | putative ATP-ase |  |
| rme:Rmet\_2639 | putative dinucleotide-utilizing enzyme involved in molybdopterin and thiamine biosynthesis |  |
| rme:Rmet\_2640 | putative outer membrane biogenesis protein (yhjG-like); K07290 hypothetical protein |  |
| rme:Rmet\_2641 | putative major facilitator superfamily transporter |  |
| rme:Rmet\_2642 | pdxH; pyridoxamine 5'-phosphate oxidase (EC:1.4.3.5); K00275 pyridoxamine 5'-phosphate oxidase [EC:1.4.3.5] | ec:1.4.3.5 |
| rme:Rmet\_2643 | cfa; cyclopropane-fatty-acyl-phospholipid synthase (EC:2.1.1.79); K00574 cyclopropane-fatty-acyl-phospholipid synthase [EC:2.1.1.79] | ec:2.1.1.79 |
| rme:Rmet\_2644 | hypothetical protein |  |
| rme:Rmet\_2645 | msrA; methionine sulfoxide reductase A (EC:1.8.4.11); K07304 peptide-methionine (S)-S-oxide reductase [EC:1.8.4.11] | ec:1.8.4.11 |
| rme:Rmet\_2646 | Flavin reductase-like, FMN-binding protein |  |
| rme:Rmet\_2647 | AsnC/Lrp family transcriptional regulator |  |
| rme:Rmet\_2648 | pseudogene |  |
| rme:Rmet\_6545 | putative cyclase; K07130 arylformamidase [EC:3.5.1.9] | ec:3.5.1.9 |
| rme:Rmet\_2650 | kynU; Kynureninase, L-Kynurenine hydrolase (EC:3.7.1.3); K01556 kynureninase [EC:3.7.1.3] | ec:3.7.1.3 |
| rme:Rmet\_2651 | tdo; Tryptophan 2,3-dioxygenase (EC:1.13.11.11); K00453 tryptophan 2,3-dioxygenase [EC:1.13.11.11] | ec:1.13.11.11 |
| rme:Rmet\_2652 | IclR family transcriptional regulator |  |
| rme:Rmet\_2653 | gcdH; glutaryl-CoA dehydrogenase (EC:1.3.99.7); K00252 glutaryl-CoA dehydrogenase [EC:1.3.8.6] | ec:1.3.8.6 |
| rme:Rmet\_2654 | antioxidant protein LsfA (EC:1.11.1.7) |  |
| rme:Rmet\_6546 | hypothetical protein |  |
| rme:Rmet\_2655 | elaB; hypothetical protein |  |
| rme:Rmet\_2656 | hypothetical protein |  |

  
**Neighborhood Representations for "reu:Reut\_A0810"**  

| ID | Annotation | EC number |
| --- | --- | --- |
| reu:Reut\_A0800 | PAS/PAC sensor-containing diguanylate cyclase/phosphodiesterase |  |
| reu:Reut\_A0801 | hypothetical protein |  |
| reu:Reut\_A0802 | transmembrane protein |  |
| reu:Reut\_A0803 | hypothetical protein |  |
| reu:Reut\_A0804 | pseudogene |  |
| reu:Reut\_A0805 | acyl-CoA dehydrogenase; K00252 glutaryl-CoA dehydrogenase [EC:1.3.8.6] | ec:1.3.8.6 |
| reu:Reut\_A0806 | IclR family transcriptional regulator |  |
| reu:Reut\_A0807 | tryptophan 2,3-dioxygenase (EC:1.13.11.11); K00453 tryptophan 2,3-dioxygenase [EC:1.13.11.11] | ec:1.13.11.11 |
| reu:Reut\_A0808 | kynureninase (EC:3.7.1.3); K01556 kynureninase [EC:3.7.1.3] | ec:3.7.1.3 |
| reu:Reut\_A0809 | cyclase; K07130 arylformamidase [EC:3.5.1.9] | ec:3.5.1.9 |
| reu:Reut\_A0810 | AsnC family transcriptional regulator |  |
| reu:Reut\_A0811 | flavin reductase-like, FMN-binding |  |
| reu:Reut\_A0812 | methionine sulfoxide reductase A (EC:1.8.4.11); K07304 peptide-methionine (S)-S-oxide reductase [EC:1.8.4.11] | ec:1.8.4.11 |
| reu:Reut\_A0813 | hypothetical protein |  |
| reu:Reut\_A0814 | cyclopropane-fatty-acyl-phospholipid synthase (EC:2.1.1.79); K00574 cyclopropane-fatty-acyl-phospholipid synthase [EC:2.1.1.79] | ec:2.1.1.79 |
| reu:Reut\_A0815 | pyridoxamine 5'-phosphate oxidase (EC:1.4.3.5); K00275 pyridoxamine 5'-phosphate oxidase [EC:1.4.3.5] | ec:1.4.3.5 |
| reu:Reut\_A0816 | major facilitator transporter |  |
| reu:Reut\_A0817 | AsmA protein; K07290 hypothetical protein |  |
| reu:Reut\_A0818 | UBA/THIF-type NAD/FAD binding fold |  |
| reu:Reut\_A0819 | ATPase |  |
| reu:Reut\_A0820 | cation-binding hemerythrin HHE family protein |  |

  
**Neighborhood Representations for "rsc:RCFBP\_20654"**  

| ID | Annotation | EC number |
| --- | --- | --- |
| rsc:RCFBP\_20644 | activating enzyme (e1 family) of ubiquitin-like protein |  |
| rsc:RCFBP\_20645 | outer membrane biogenesis protein, asma family; K07290 hypothetical protein |  |
| rsc:RCFBP\_20646 | oxidoreductase dehydrogenase (cytochrome C subunit) (fragment) (EC:1.-.-.-) |  |
| rsc:RCFBP\_20647 | beta-hexosaminidase a (EC:3.2.1.52); K01207 beta-N-acetylhexosaminidase [EC:3.2.1.52] | ec:3.2.1.52 |
| rsc:RCFBP\_20648 | pdxH; pyridoxine 5'-phosphate oxidase (EC:1.4.3.5); K00275 pyridoxamine 5'-phosphate oxidase [EC:1.4.3.5] | ec:1.4.3.5 |
| rsc:RCFBP\_20649 | cfa; cyclopropane-fatty-acyl-phospholipid synthase (EC:2.1.1.79); K00574 cyclopropane-fatty-acyl-phospholipid synthase [EC:2.1.1.79] | ec:2.1.1.79 |
| rsc:RCFBP\_20650 | hypothetical protein |  |
| rsc:RCFBP\_20651 | msrA; protein-methionine-s-oxide reductase (EC:1.8.4.11); K07304 peptide-methionine (S)-S-oxide reductase [EC:1.8.4.11] | ec:1.8.4.11 |
| rsc:RCFBP\_20652 | monooxygenase oxidoreducatse, flavin reductase-like, fmN-binding domain (EC:1.14.-.-) |  |
| rsc:RCFBP\_20653 | hypothetical protein |  |
| rsc:RCFBP\_20654 | Irp; AsnC/lrp family transcriptional regulator |  |
| rsc:RCFBP\_20655 | metal-dependent hydrolase cyclase (arylformamidase) (EC:3.5.1.9); K07130 arylformamidase [EC:3.5.1.9] | ec:3.5.1.9 |
| rsc:RCFBP\_20656 | pyridoxal-5'-phosphate (plp)-dependent kynureninase (EC:3.7.1.3); K01556 kynureninase [EC:3.7.1.3] | ec:3.7.1.3 |
| rsc:RCFBP\_20657 | dioxygenase (EC:1.13.11.-); K00453 tryptophan 2,3-dioxygenase [EC:1.13.11.11] | ec:1.13.11.11 |
| rsc:RCFBP\_20658 | iclr family transcriptional regulator |  |
| rsc:RCFBP\_20659 | dehydrogenase (EC:1.3.99.-); K00252 glutaryl-CoA dehydrogenase [EC:1.3.8.6] | ec:1.3.8.6 |
| rsc:RCFBP\_20661 | permease; K06901 putative MFS transporter, AGZA family, xanthine/uracil permease |  |
| rsc:RCFBP\_20662 | peroxidase (EC:1.11.1.7) |  |
| rsc:RCFBP\_20663 | hypothetical protein |  |
| rsc:RCFBP\_20664 | hypothetical protein |  |
| rsc:RCFBP\_20665 | hypothetical protein |  |

  
**Neighborhood Representations for "rpf:Rpic12D\_0777"**  

| ID | Annotation | EC number |
| --- | --- | --- |
| rpf:Rpic12D\_0767 | hypothetical protein |  |
| rpf:Rpic12D\_0768 | hypothetical protein |  |
| rpf:Rpic12D\_0769 | hypothetical protein |  |
| rpf:Rpic12D\_0770 | peroxidase (EC:1.11.1.7) |  |
| rpf:Rpic12D\_0771 | xanthine/uracil/vitamin C permease; K06901 putative MFS transporter, AGZA family, xanthine/uracil permease |  |
| rpf:Rpic12D\_0772 | acyl-CoA dehydrogenase domain-containing protein; K00252 glutaryl-CoA dehydrogenase [EC:1.3.8.6] | ec:1.3.8.6 |
| rpf:Rpic12D\_0773 | IclR family transcriptional regulator |  |
| rpf:Rpic12D\_0774 | tryptophan 2,3-dioxygenase; K00453 tryptophan 2,3-dioxygenase [EC:1.13.11.11] | ec:1.13.11.11 |
| rpf:Rpic12D\_0775 | kynureninase; K01556 kynureninase [EC:3.7.1.3] | ec:3.7.1.3 |
| rpf:Rpic12D\_0776 | arylformamidase; K07130 arylformamidase [EC:3.5.1.9] | ec:3.5.1.9 |
| rpf:Rpic12D\_0777 | AsnC family transcriptional regulator |  |
| rpf:Rpic12D\_0778 | hypothetical protein |  |
| rpf:Rpic12D\_0779 | flavin reductase domain-containing protein |  |
| rpf:Rpic12D\_0780 | TrmB family transcriptional regulator |  |
| rpf:Rpic12D\_0781 | relaxase/mobilization nuclease |  |
| rpf:Rpic12D\_0782 | mobilisation protein |  |
| rpf:Rpic12D\_0783 | integrase family protein |  |
| rpf:Rpic12D\_0784 | methionine sulfoxide reductase A; K07304 peptide-methionine (S)-S-oxide reductase [EC:1.8.4.11] | ec:1.8.4.11 |
| rpf:Rpic12D\_0785 | hypothetical protein |  |
| rpf:Rpic12D\_0786 | cyclopropane-fatty-acyl-phospholipid synthase (EC:2.1.1.79); K00574 cyclopropane-fatty-acyl-phospholipid synthase [EC:2.1.1.79] | ec:2.1.1.79 |
| rpf:Rpic12D\_0787 | pyridoxamine 5'-phosphate oxidase (EC:1.4.3.5); K00275 pyridoxamine 5'-phosphate oxidase [EC:1.4.3.5] | ec:1.4.3.5 |

  
**Neighborhood Representations for "rpi:Rpic\_0711"**  

| ID | Annotation | EC number |
| --- | --- | --- |
| rpi:Rpic\_0701 | hypothetical protein |  |
| rpi:Rpic\_0702 | hypothetical protein |  |
| rpi:Rpic\_0703 | hypothetical protein |  |
| rpi:Rpic\_0704 | peroxidase (EC:1.11.1.7) |  |
| rpi:Rpic\_0705 | xanthine/uracil/vitamin C permease; K06901 putative MFS transporter, AGZA family, xanthine/uracil permease |  |
| rpi:Rpic\_0706 | acyl-CoA dehydrogenase domain-containing protein; K00252 glutaryl-CoA dehydrogenase [EC:1.3.8.6] | ec:1.3.8.6 |
| rpi:Rpic\_0707 | IclR family transcriptional regulator |  |
| rpi:Rpic\_0708 | tryptophan 2,3-dioxygenase; K00453 tryptophan 2,3-dioxygenase [EC:1.13.11.11] | ec:1.13.11.11 |
| rpi:Rpic\_0709 | kynureninase; K01556 kynureninase [EC:3.7.1.3] | ec:3.7.1.3 |
| rpi:Rpic\_0710 | arylformamidase; K07130 arylformamidase [EC:3.5.1.9] | ec:3.5.1.9 |
| rpi:Rpic\_0711 | AsnC family transcriptional regulator |  |
| rpi:Rpic\_0712 | hypothetical protein |  |
| rpi:Rpic\_0713 | flavin reductase domain-containing protein |  |
| rpi:Rpic\_0714 | methionine sulfoxide reductase A (EC:1.8.4.11); K07304 peptide-methionine (S)-S-oxide reductase [EC:1.8.4.11] | ec:1.8.4.11 |
| rpi:Rpic\_0715 | hypothetical protein |  |
| rpi:Rpic\_0716 | cyclopropane-fatty-acyl-phospholipid synthase (EC:2.1.1.79); K00574 cyclopropane-fatty-acyl-phospholipid synthase [EC:2.1.1.79] | ec:2.1.1.79 |
| rpi:Rpic\_0717 | pyridoxamine 5'-phosphate oxidase (EC:1.4.3.5); K00275 pyridoxamine 5'-phosphate oxidase [EC:1.4.3.5] | ec:1.4.3.5 |
| rpi:Rpic\_0718 | putative transmembrane dehydrogenase (small subunit) oxidoreductase protein |  |
| rpi:Rpic\_0719 | glucose-methanol-choline oxidoreductase |  |
| rpi:Rpic\_0720 | gluconate 2-dehydrogenase (EC:1.1.99.3) |  |
| rpi:Rpic\_0721 | AsmA family protein; K07290 hypothetical protein |  |

  
**Neighborhood Representations for "rsl:RPSI07\_2579"**  

| ID | Annotation | EC number |
| --- | --- | --- |
| rsl:RPSI07\_2569 | oxidoreductase dehydrogenase (cytochrome C subunit) (EC:1.-.-.-) |  |
| rsl:RPSI07\_2570 | transmembrane dehydrogenase (Large subunit) oxidoreductase (EC:1.-.-.-) |  |
| rsl:RPSI07\_2571 | transmembrane dehydrogenase (Small subunit) oxidoreductase protein (EC:1.-.-.-) |  |
| rsl:RPSI07\_2572 | beta-hexosaminidase A (EC:3.2.1.52); K01207 beta-N-acetylhexosaminidase [EC:3.2.1.52] | ec:3.2.1.52 |
| rsl:RPSI07\_2573 | pdxH; pyridoxine 5'-phosphate oxidase (EC:1.4.3.5); K00275 pyridoxamine 5'-phosphate oxidase [EC:1.4.3.5] | ec:1.4.3.5 |
| rsl:RPSI07\_2574 | cfa; cyclopropane-fatty-acyl-phospholipid synthase (EC:2.1.1.79); K00574 cyclopropane-fatty-acyl-phospholipid synthase [EC:2.1.1.79] | ec:2.1.1.79 |
| rsl:RPSI07\_2575 | hypothetical protein |  |
| rsl:RPSI07\_2576 | msrA; protein-methionine-S-oxide reductase (EC:1.8.4.11); K07304 peptide-methionine (S)-S-oxide reductase [EC:1.8.4.11] | ec:1.8.4.11 |
| rsl:RPSI07\_2577 | monooxygenase oxidoreducatse, Flavin reductase-like, FMN-binding domain (EC:1.14.-.-) |  |
| rsl:RPSI07\_2578 | hypothetical protein |  |
| rsl:RPSI07\_2579 | Irp; AsnC family transcriptional regulator |  |
| rsl:RPSI07\_2580 | metal-dependent hydrolase cyclase (arylformamidase) (EC:3.5.1.9); K07130 arylformamidase [EC:3.5.1.9] | ec:3.5.1.9 |
| rsl:RPSI07\_2581 | pyridoxal-5'-phosphate (PLP)-dependent kynureninase (EC:3.7.1.3); K01556 kynureninase [EC:3.7.1.3] | ec:3.7.1.3 |
| rsl:RPSI07\_2582 | dioxygenase (EC:1.13.11.-); K00453 tryptophan 2,3-dioxygenase [EC:1.13.11.11] | ec:1.13.11.11 |
| rsl:RPSI07\_2583 | IclR family transcriptional regulator |  |
| rsl:RPSI07\_2584 | dehydrogenase (EC:1.3.99.-); K00252 glutaryl-CoA dehydrogenase [EC:1.3.8.6] | ec:1.3.8.6 |
| rsl:RPSI07\_2585 | permease; K06901 putative MFS transporter, AGZA family, xanthine/uracil permease |  |
| rsl:RPSI07\_2586 | peroxidase (EC:1.11.1.7) |  |
| rsl:RPSI07\_2587 | hypothetical protein |  |
| rsl:RPSI07\_2588 | hypothetical protein |  |
| rsl:RPSI07\_2589 | hypothetical protein |  |

  
**Neighborhood Representations for "pfl:PFL\_0761"**  

| ID | Annotation | EC number |
| --- | --- | --- |
| pfl:PFL\_0751 | hypothetical protein; K06995 |  |
| pfl:PFL\_0752 | kynB; arylformamidase (EC:3.5.1.9); K07130 arylformamidase [EC:3.5.1.9] | ec:3.5.1.9 |
| pfl:PFL\_0753 | kynA; tryptophan 2,3-dioxygenase (EC:1.13.11.11); K00453 tryptophan 2,3-dioxygenase [EC:1.13.11.11] | ec:1.13.11.11 |
| pfl:PFL\_0754 | GntR family transcriptional regulator; K00375 GntR family transcriptional regulator / MocR family aminotransferase |  |
| pfl:PFL\_0755 | benzoate transporter family protein; K05782 benzoate membrane transport protein |  |
| pfl:PFL\_0756 | aroP\_2; aromatic amino acid transport protein AroP; K11734 aromatic amino acid transport protein AroP |  |
| pfl:PFL\_0757 | antC; anthranilate dioxygenase reductase (EC:1.14.12.1); K11311 anthranilate dioxygenase reductase |  |
| pfl:PFL\_0758 | antB; anthranilate 1,2-dioxygenase, small subunit (EC:1.14.12.1); K05600 anthranilate 1,2-dioxygenase (deaminating, decarboxylating) small subunit [EC:1.14.12.1] | ec:1.14.12.1 |
| pfl:PFL\_0759 | antA; anthranilate 1,2-dioxygenase, large subunit (EC:1.14.12.1); K05599 anthranilate 1,2-dioxygenase (deaminating, decarboxylating) large subunit [EC:1.14.12.1] | ec:1.14.12.1 |
| pfl:PFL\_0760 | AraC family transcriptional regulator |  |
| pfl:PFL\_0761 | AsnC family transcriptional regulator |  |
| pfl:PFL\_0762 | kynU; kynureninase (EC:3.7.1.3); K01556 kynureninase [EC:3.7.1.3] | ec:3.7.1.3 |
| pfl:PFL\_0763 | APC family amino acid permease |  |
| pfl:PFL\_0764 | MerR family transcriptional regulator |  |
| pfl:PFL\_0765 | NAD(P)H dehydrogenase |  |
| pfl:PFL\_0766 | LysR family transcriptional regulator |  |
| pfl:PFL\_0767 | hypothetical protein; K07090 |  |
| pfl:PFL\_0768 | antibiotic biosynthesis monooxygenase domain-containing protein |  |
| pfl:PFL\_0769 | mdaB; NAD(P)H dehydrogenase; K03923 modulator of drug activity B |  |
| pfl:PFL\_0770 | LysR family transcriptional regulator |  |
| pfl:PFL\_0771 | ggt\_2; gamma-glutamyltransferase (EC:2.3.2.2); K00681 gamma-glutamyltranspeptidase [EC:2.3.2.2] | ec:2.3.2.2 |

  
**Neighborhood Representations for "pae:PA2082"**  

| ID | Annotation | EC number |
| --- | --- | --- |
| pae:PA2072 | hypothetical protein |  |
| pae:PA2073 | transporter membrane subunit |  |
| pae:PA2074 | hypothetical protein |  |
| pae:PA2075 | hypothetical protein |  |
| pae:PA2076 | transcriptional regulator |  |
| pae:PA2077 | hypothetical protein |  |
| pae:PA2078 | hypothetical protein |  |
| pae:PA2079 | amino acid permease |  |
| pae:PA2080 | kynU; kynureninase KynU; K01556 kynureninase [EC:3.7.1.3] | ec:3.7.1.3 |
| pae:PA2081 | kynB; kynurenine formamidase, KynB; K07130 arylformamidase [EC:3.5.1.9] | ec:3.5.1.9 |
| pae:PA2082 | transcriptional regulator |  |
| pae:PA2083 | ring-hydroxylating dioxygenase subunit |  |
| pae:PA2084 | asparagine synthetase; K01953 asparagine synthase (glutamine-hydrolysing) [EC:6.3.5.4] | ec:6.3.5.4 |
| pae:PA2085 | ring-hydroxylating dioxygenase small subunit |  |
| pae:PA2086 | epoxide hydrolase; K01561 haloacetate dehalogenase [EC:3.8.1.3] | ec:3.8.1.3 |
| pae:PA2087 | hypothetical protein |  |
| pae:PA2088 | hypothetical protein |  |
| pae:PA2089 | hypothetical protein; K02014 iron complex outermembrane recepter protein |  |
| pae:PA2090 | hypothetical protein |  |
| pae:PA2091 | hypothetical protein |  |
| pae:PA2092 | major facilitator superfamily (MFS) transporter; K08164 MFS transporter, DHA1 family, chloramphenicol resistance protein |  |

  
**Neighborhood Representations for "pag:PLES\_32391"**  

| ID | Annotation | EC number |
| --- | --- | --- |
| pag:PLES\_32291 | putative major facilitator superfamily transporter; K08164 MFS transporter, DHA1 family, chloramphenicol resistance protein |  |
| pag:PLES\_32301 | putative permease |  |
| pag:PLES\_32311 | putative flavin-dependent oxidoreductase |  |
| pag:PLES\_32321 | putative TonB-dependent receptor; K02014 iron complex outermembrane recepter protein |  |
| pag:PLES\_32331 | hypothetical protein |  |
| pag:PLES\_32341 | hypothetical protein |  |
| pag:PLES\_32351 | putative epoxide hydrolase; K01561 haloacetate dehalogenase [EC:3.8.1.3] | ec:3.8.1.3 |
| pag:PLES\_32361 | putative ring-hydroxylating dioxygenase small subunit |  |
| pag:PLES\_32371 | putative asparagine synthetase; K01953 asparagine synthase (glutamine-hydrolysing) [EC:6.3.5.4] | ec:6.3.5.4 |
| pag:PLES\_32381 | putative ring-hydroxylating dioxygenase subunit |  |
| pag:PLES\_32391 | putative transcriptional regulator |  |
| pag:PLES\_32401 | kynB; kynurenine formamidase, KynB; K07130 arylformamidase [EC:3.5.1.9] | ec:3.5.1.9 |
| pag:PLES\_32411 | putative kynureninase; K01556 kynureninase [EC:3.7.1.3] | ec:3.7.1.3 |
| pag:PLES\_32421 | putative amino acid permease |  |
| pag:PLES\_32431 | hypothetical protein |  |
| pag:PLES\_32441 | hypothetical protein |  |
| pag:PLES\_32451 | putative transcriptional regulator |  |
| pag:PLES\_32461 | hypothetical protein |  |
| pag:PLES\_32471 | hypothetical protein |  |
| pag:PLES\_32481 | hypothetical protein |  |
| pag:PLES\_32491 | putative transporter (membrane subunit) |  |

  
**Neighborhood Representations for "pau:PA14\_37580"**  

| ID | Annotation | EC number |
| --- | --- | --- |
| pau:PA14\_37440 | MFS transporter |  |
| pau:PA14\_37460 | permease |  |
| pau:PA14\_37470 | flavin-dependent oxidoreductase |  |
| pau:PA14\_37490 | TonB-dependent receptor; K02014 iron complex outermembrane recepter protein |  |
| pau:PA14\_37510 | hypothetical protein |  |
| pau:PA14\_37520 | hypothetical protein |  |
| pau:PA14\_37530 | hydrolase; K01561 haloacetate dehalogenase [EC:3.8.1.3] | ec:3.8.1.3 |
| pau:PA14\_37550 | ring-hydroxylating dioxygenase small subunit |  |
| pau:PA14\_37560 | asnB; asparagine synthetase, glutamine-hydrolysing; K01953 asparagine synthase (glutamine-hydrolysing) [EC:6.3.5.4] | ec:6.3.5.4 |
| pau:PA14\_37570 | ring-hydroxylating dioxygenase, large terminal subunit |  |
| pau:PA14\_37580 | lrp; leucine-responsive regulatory protein |  |
| pau:PA14\_37590 | kynB; kynurenine formamidase, KynB; K07130 arylformamidase [EC:3.5.1.9] | ec:3.5.1.9 |
| pau:PA14\_37610 | kynureninase; K01556 kynureninase [EC:3.7.1.3] | ec:3.7.1.3 |
| pau:PA14\_37630 | amino acid permease |  |
| pau:PA14\_37640 | hypothetical protein |  |
| pau:PA14\_37650 | hypothetical protein |  |
| pau:PA14\_37660 | transcriptional regulator |  |
| pau:PA14\_37670 | hypothetical protein |  |
| pau:PA14\_37680 | hypothetical protein |  |
| pau:PA14\_37690 | sensory box protein |  |
| pau:PA14\_37710 | fusA2; elongation factor G; K02355 elongation factor G |  |

  
**Neighborhood Representations for "pfs:PFLU5189"**  

| ID | Annotation | EC number |
| --- | --- | --- |
| pfs:PFLU5179 | RpiR family regulatory protein |  |
| pfs:PFLU5180 | putative gluconate permease; K03299 gluconate:H+ symporter, GntP family |  |
| pfs:PFLU5181 | hypothetical protein |  |
| pfs:PFLU5182 | integral membrane sulfate transporter; K03321 sulfate permease, SulP family |  |
| pfs:PFLU5183 | putative polysaccharide synthesis protein |  |
| pfs:PFLU5184 | LysR family transcriptional regulator |  |
| pfs:PFLU5185 | hypothetical protein; K07090 |  |
| pfs:PFLU5186 | LysR family transcriptional regulator |  |
| pfs:PFLU5187 | putative amino acid transporter membrane protein |  |
| pfs:PFLU5188 | hypothetical protein; K01556 kynureninase [EC:3.7.1.3] | ec:3.7.1.3 |
| pfs:PFLU5189 | putative AsnC family regulatory protein |  |
| pfs:PFLU5190 | catechol 1,2-dioxygenase (EC:1.13.11.1); K03381 catechol 1,2-dioxygenase [EC:1.13.11.1] | ec:1.13.11.1 |
| pfs:PFLU5191 | muconolactone delta-isomerase (EC:5.3.3.4); K03464 muconolactone D-isomerase [EC:5.3.3.4] | ec:5.3.3.4 |
| pfs:PFLU5192 | muconate cycloisomerase 1 (EC:5.5.1.1); K01856 muconate cycloisomerase [EC:5.5.1.1] | ec:5.5.1.1 |
| pfs:PFLU5193 | AraC family transcriptional regulator |  |
| pfs:PFLU5194 | benzoate 1,2-dioxygenase subunit alpha (EC:1.14.12.10); K05599 anthranilate 1,2-dioxygenase (deaminating, decarboxylating) large subunit [EC:1.14.12.1] | ec:1.14.12.1 |
| pfs:PFLU5195 | benzoate 1,2-dioxygenase subunit beta (EC:1.14.12.10); K05600 anthranilate 1,2-dioxygenase (deaminating, decarboxylating) small subunit [EC:1.14.12.1] | ec:1.14.12.1 |
| pfs:PFLU5196 | antC; anthranilate dioxygenase reductase (EC:1.18.1.3); K11311 anthranilate dioxygenase reductase |  |
| pfs:PFLU5197 | aromatic amino acid transport membrane protein; K03293 amino acid transporter, AAT family |  |
| pfs:PFLU5198 | putative dioxygenase; K00453 tryptophan 2,3-dioxygenase [EC:1.13.11.11] | ec:1.13.11.11 |
| pfs:PFLU5199 | hypothetical protein; K07130 arylformamidase [EC:3.5.1.9] | ec:3.5.1.9 |

  
**Neighborhood Representations for "pap:PSPA7\_3205"**  

| ID | Annotation | EC number |
| --- | --- | --- |
| pap:PSPA7\_3195 | putative transcriptional regulator |  |
| pap:PSPA7\_3196 | hypothetical protein |  |
| pap:PSPA7\_3197 | MFS family transporter |  |
| pap:PSPA7\_3198 | putative porin |  |
| pap:PSPA7\_3199 | LamB/YcsF family protein; K07160 UPF0271 protein |  |
| pap:PSPA7\_3200 | hypothetical protein |  |
| pap:PSPA7\_3201 | hypothetical protein |  |
| pap:PSPA7\_3202 | hypothetical protein |  |
| pap:PSPA7\_3203 | thiamine pyrophosphate protein; K00156 pyruvate dehydrogenase (quinone) [EC:1.2.5.1] | ec:1.2.5.1 |
| pap:PSPA7\_3204 | hypothetical protein |  |
| pap:PSPA7\_3205 | AsnC family transcriptional regulator |  |
| pap:PSPA7\_3206 | kynB; arylformamidase (EC:3.5.1.9); K07130 arylformamidase [EC:3.5.1.9] | ec:3.5.1.9 |
| pap:PSPA7\_3208 | kynU; kynureninase (EC:3.7.1.3); K01556 kynureninase [EC:3.7.1.3] | ec:3.7.1.3 |
| pap:PSPA7\_3207 | hypothetical protein |  |
| pap:PSPA7\_3209 | putative amino acid permease |  |
| pap:PSPA7\_3210 | hypothetical protein |  |
| pap:PSPA7\_3211 | hypothetical protein |  |
| pap:PSPA7\_3212 | hypothetical protein |  |
| pap:PSPA7\_3213 | putative transcriptional regulator |  |
| pap:PSPA7\_3214 | hypothetical protein |  |
| pap:PSPA7\_3215 | hypothetical protein |  |

  
**Neighborhood Representations for "bav:BAV0200"**  

| ID | Annotation | EC number |
| --- | --- | --- |
| bav:BAV0190 | lysG; chromosome replication initiation inhibitor protein; K05596 LysR family transcriptional regulator, chromosome initiation inhibitor |  |
| bav:BAV0191 | transferrin/hemoglobin-binding protein; K16087 hemoglobin/transferrin/lactoferrin receptor protein |  |
| bav:BAV0192 | hypothetical protein |  |
| bav:BAV0193 | hypothetical protein; K06997 |  |
| bav:BAV0194 | aminotransferase |  |
| bav:BAV0195 | amino acid-binding periplasmic protein; K09969 general L-amino acid transport system substrate-binding protein |  |
| bav:BAV0196 | membrane protein |  |
| bav:BAV0197 | hmgcL; hydroxymethylglutaryl-CoA lyase (EC:4.1.3.4); K01640 hydroxymethylglutaryl-CoA lyase [EC:4.1.3.4] | ec:4.1.3.4 |
| bav:BAV0198 | smf; Smf protein; K04096 DNA processing protein |  |
| bav:BAV0199 | two-component system response regulator |  |
| bav:BAV0200 | alsB; leucine-responsive regulatory protein |  |
| bav:BAV0201 | kynureninase (EC:3.7.1.3); K01556 kynureninase [EC:3.7.1.3] | ec:3.7.1.3 |
| bav:BAV0202 | def; peptide deformylase (EC:3.5.1.88); K01462 peptide deformylase [EC:3.5.1.88] | ec:3.5.1.88 |
| bav:BAV0203 | fmt; methionyl-tRNA formyltransferase (EC:2.1.2.9); K00604 methionyl-tRNA formyltransferase [EC:2.1.2.9] | ec:2.1.2.9 |
| bav:BAV0204 | pyridoxamine 5'-phosphate oxidase; K07006 |  |
| bav:BAV0205 | phosphatase; K01091 phosphoglycolate phosphatase [EC:3.1.3.18] | ec:3.1.3.18 |
| bav:BAV0206 | hypothetical protein; K06966 |  |
| bav:BAV0207 | CoA-transferase family III |  |
| bav:BAV0208 | rimO; ribosomal protein S12 methylthiotransferase; K14441 ribosomal protein S12 methylthiotransferase [EC:2.-.-.-] |  |
| bav:BAV0209 | DNA-binding protein |  |
| bav:BAV0210 | hypothetical protein |  |

  
**Neighborhood Representations for "axy:AXYL\_05264"**  

| ID | Annotation | EC number |
| --- | --- | --- |
| axy:AXYL\_05254 | hmuS; hemin transporter HmuS; K07225 putative hemin transport protein |  |
| axy:AXYL\_05255 | TonB-dependent siderophore receptor family protein 17; K16087 hemoglobin/transferrin/lactoferrin receptor protein |  |
| axy:AXYL\_05256 | FecR family protein |  |
| axy:AXYL\_05257 | RNA polymerase sigma factor FecI; K03088 RNA polymerase sigma-70 factor, ECF subfamily |  |
| axy:AXYL\_05258 | integral membrane protein 4 |  |
| axy:AXYL\_05259 | hypothetical protein |  |
| axy:AXYL\_05260 | HTH domain-containing protein 1 |  |
| axy:AXYL\_05261 | glyoxalase/bleomycin resistance protein/dioxygenase superfamily protein 22 |  |
| axy:AXYL\_05262 | amino acid permease family protein 2 |  |
| axy:AXYL\_05263 | kynU; kynureninase (EC:3.7.1.3); K01556 kynureninase [EC:3.7.1.3] | ec:3.7.1.3 |
| axy:AXYL\_05264 | AsnC family transcriptional regulator |  |
| axy:AXYL\_05265 | hemolysin-III related family protein; K11068 hemolysin III |  |
| axy:AXYL\_05266 | Tat pathway signal sequence domain-containing protein 22 |  |
| axy:AXYL\_05267 | tcyA; L-cystine-binding protein TcyA; K02424 cystine transport system substrate-binding protein |  |
| axy:AXYL\_05268 | L-cystine transporter permease TcyB; K10009 cystine transport system permease protein |  |
| axy:AXYL\_05269 | amino-acid ABC transporter ATP-binding protein 2; K10010 cystine transport system ATP-binding protein [EC:3.6.3.-] |  |
| axy:AXYL\_05270 | hypothetical protein |  |
| axy:AXYL\_05271 | TonB-dependent Receptor Plug domain-containing protein 5; K16087 hemoglobin/transferrin/lactoferrin receptor protein |  |
| axy:AXYL\_05272 | hypothetical protein |  |
| axy:AXYL\_05273 | hypothetical protein |  |
| axy:AXYL\_05274 | ABC transporter (EC:3.6.3.-); K02013 iron complex transport system ATP-binding protein [EC:3.6.3.34] | ec:3.6.3.34 |

  
**Neighborhood Representations for "bbr:BB0245"**  

| ID | Annotation | EC number |
| --- | --- | --- |
| bbr:BB0235 | transporter; K06895 L-lysine exporter family protein LysE/ArgO |  |
| bbr:BB0236 | chromosome replication initiation inhibitor protein; K05596 LysR family transcriptional regulator, chromosome initiation inhibitor |  |
| bbr:BB0237 | hypothetical protein; K09796 hypothetical protein |  |
| bbr:BB0238 | hypothetical protein |  |
| bbr:BB0239 | hypothetical protein; K06997 |  |
| bbr:BB0240 | aminotransferase |  |
| bbr:BB0241 | amino acid ABC transporter substrate-binding protein; K09969 general L-amino acid transport system substrate-binding protein |  |
| bbr:BB0242 | hypothetical protein |  |
| bbr:BB0243 | hmgcL; hydroxymethylglutaryl-CoA lyase (EC:4.1.3.4); K01640 hydroxymethylglutaryl-CoA lyase [EC:4.1.3.4] | ec:4.1.3.4 |
| bbr:BB0244 | smf; hypothetical protein; K04096 DNA processing protein |  |
| bbr:BB0245 | lrp; leucine-responsive regulatory protein |  |
| bbr:BB0246 | hypothetical protein; K01556 kynureninase [EC:3.7.1.3] | ec:3.7.1.3 |
| bbr:BB0247 | def; peptide deformylase (EC:3.5.1.88); K01462 peptide deformylase [EC:3.5.1.88] | ec:3.5.1.88 |
| bbr:BB0248 | fmt; methionyl-tRNA formyltransferase (EC:2.1.2.9); K00604 methionyl-tRNA formyltransferase [EC:2.1.2.9] | ec:2.1.2.9 |
| bbr:BB0249 | hypothetical protein; K07006 |  |
| bbr:BB0250 | haloacid dehalogenase-like hydrolase; K01091 phosphoglycolate phosphatase [EC:3.1.3.18] | ec:3.1.3.18 |
| bbr:BB0251 | hypothetical protein |  |
| bbr:BB0252 | lysine decarboxylase; K06966 |  |
| bbr:BB0253 | bacterioferritin; K03594 bacterioferritin |  |
| bbr:BB0254 | rimO; ribosomal protein S12 methylthiotransferase; K14441 ribosomal protein S12 methylthiotransferase [EC:2.-.-.-] |  |
| bbr:BB0255 | 4-oxalocrotonate tautomerase; K01821 4-oxalocrotonate tautomerase [EC:5.3.2.6] | ec:5.3.2.6 |

  
**Neighborhood Representations for "bpa:BPP0241"**  

| ID | Annotation | EC number |
| --- | --- | --- |
| bpa:BPP0231 | transporter; K06895 L-lysine exporter family protein LysE/ArgO |  |
| bpa:BPP0232 | chromosome replication initiation inhibitor protein; K05596 LysR family transcriptional regulator, chromosome initiation inhibitor |  |
| bpa:BPP0233 | hypothetical protein; K09796 hypothetical protein |  |
| bpa:BPP0234 | hypothetical protein |  |
| bpa:BPP0235 | hypothetical protein; K06997 |  |
| bpa:BPP0236 | aminotransferase |  |
| bpa:BPP0237 | amino acid-binding periplasmic protein; K09969 general L-amino acid transport system substrate-binding protein |  |
| bpa:BPP0238 | hypothetical protein |  |
| bpa:BPP0239 | hmgcL; hydroxymethylglutaryl-CoA lyase (EC:4.1.3.4); K01640 hydroxymethylglutaryl-CoA lyase [EC:4.1.3.4] | ec:4.1.3.4 |
| bpa:BPP0240 | smf; hypothetical protein; K04096 DNA processing protein |  |
| bpa:BPP0241 | lrp; leucine-responsive regulatory protein |  |
| bpa:BPP0242 | hypothetical protein; K01556 kynureninase [EC:3.7.1.3] | ec:3.7.1.3 |
| bpa:BPP0243 | def; peptide deformylase (EC:3.5.1.88); K01462 peptide deformylase [EC:3.5.1.88] | ec:3.5.1.88 |
| bpa:BPP0244 | fmt; methionyl-tRNA formyltransferase (EC:2.1.2.9); K00604 methionyl-tRNA formyltransferase [EC:2.1.2.9] | ec:2.1.2.9 |
| bpa:BPP0245 | hypothetical protein; K07006 |  |
| bpa:BPP0246 | haloacid dehalogenase-like hydrolase; K01091 phosphoglycolate phosphatase [EC:3.1.3.18] | ec:3.1.3.18 |
| bpa:BPP0247 | hypothetical protein |  |
| bpa:BPP0248 | lysine decarboxylase; K06966 |  |
| bpa:BPP0249 | bacterioferritin; K03594 bacterioferritin |  |
| bpa:BPP0250 | rimO; ribosomal protein S12 methylthiotransferase; K14441 ribosomal protein S12 methylthiotransferase [EC:2.-.-.-] |  |
| bpa:BPP0251 | 4-oxalocrotonate tautomerase; K01821 4-oxalocrotonate tautomerase [EC:5.3.2.6] | ec:5.3.2.6 |

  
**Neighborhood Representations for "bpe:BP0554"**  

| ID | Annotation | EC number |
| --- | --- | --- |
| bpe:BP0544 | 4-oxalocrotonate tautomerase; K01821 4-oxalocrotonate tautomerase [EC:5.3.2.6] | ec:5.3.2.6 |
| bpe:BP0545 | rimO; ribosomal protein S12 methylthiotransferase; K14441 ribosomal protein S12 methylthiotransferase [EC:2.-.-.-] |  |
| bpe:BP0546 | bacterioferritin; K03594 bacterioferritin |  |
| bpe:BP0547 | lysine decarboxylase; K06966 |  |
| bpe:BP0548 | hypothetical protein |  |
| bpe:BP0549 | haloacid dehalogenase; K01091 phosphoglycolate phosphatase [EC:3.1.3.18] | ec:3.1.3.18 |
| bpe:BP0550 | hypothetical protein; K07006 |  |
| bpe:BP0551 | fmt; methionyl-tRNA formyltransferase (EC:2.1.2.9); K00604 methionyl-tRNA formyltransferase [EC:2.1.2.9] | ec:2.1.2.9 |
| bpe:BP0552 | def; peptide deformylase (EC:3.5.1.88); K01462 peptide deformylase [EC:3.5.1.88] | ec:3.5.1.88 |
| bpe:BP0553 | hypothetical protein; K01556 kynureninase [EC:3.7.1.3] | ec:3.7.1.3 |
| bpe:BP0554 | lrp; leucine-responsive regulatory protein |  |
| bpe:BP0555 | smf; hypothetical protein; K04096 DNA processing protein |  |
| bpe:BP0556 | hmgcL; hydroxymethylglutaryl-CoA lyase (EC:4.1.3.4); K01640 hydroxymethylglutaryl-CoA lyase [EC:4.1.3.4] | ec:4.1.3.4 |
| bpe:BP0557 | hypothetical protein |  |
| bpe:BP0558 | amino acid ABC transporter substrate-binding protein; K09969 general L-amino acid transport system substrate-binding protein |  |
| bpe:BP0559 | aminotransferase |  |
| bpe:BP0560 | pseudogene |  |
| bpe:BP0561 | hypothetical protein |  |
| bpe:BP0562 | hypothetical protein; K09796 hypothetical protein |  |
| bpe:BP0563 | chromosome replication initiation inhibitor protein; K05596 LysR family transcriptional regulator, chromosome initiation inhibitor |  |
| bpe:BP0564 | transporter; K06895 L-lysine exporter family protein LysE/ArgO |  |

  
**Neighborhood Representations for "dno:DNO\_0469"**  

| ID | Annotation | EC number |
| --- | --- | --- |
| dno:DNO\_0459 | DNO\_0459 |  |
| dno:DNO\_0460 | lipoprotein |  |
| dno:DNO\_0461 | hypothetical protein |  |
| dno:DNO\_0462 | lipoprotein |  |
| dno:DNO\_0463 | tRNA-Ser; K14233 tRNA Ser |  |
| dno:DNO\_0464 | hypothetical protein |  |
| dno:DNO\_0466 | ispZ; intracellular septation protein A; K06190 intracellular septation protein |  |
| dno:DNO\_0465 | hypothetical protein |  |
| dno:DNO\_0467 | hypothetical protein; K09710 ribosome-associated protein |  |
| dno:DNO\_0468 | fbp; fructose-1,6-bisphosphatase (EC:3.1.3.11); K03841 fructose-1,6-bisphosphatase I [EC:3.1.3.11] | ec:3.1.3.11 |
| dno:DNO\_0469 | transcription regulator AsnC; K03719 Lrp/AsnC family transcriptional regulator, leucine-responsive regulatory protein |  |
| dno:DNO\_0470 | nusB; transcription antitermination factor NusB; K03625 N utilization substance protein B |  |
| dno:DNO\_0471 | ribH; 6,7-dimethyl-8-ribityllumazine synthase (EC:2.5.1.9); K00794 6,7-dimethyl-8-ribityllumazine synthase [EC:2.5.1.78] | ec:2.5.1.78 |
| dno:DNO\_0472 | ribB; 3,4-dihydroxy-2-butanone 4-phosphate synthase (EC:3.5.4.25); K14652 3,4-dihydroxy 2-butanone 4-phosphate synthase / GTP cyclohydrolase II [EC:4.1.99.12 3.5.4.25] | ec:3.5.4.25 ec:4.1.99.12 |
| dno:DNO\_0473 | ribE; riboflavin synthase subunit alpha (EC:2.5.1.9); K00793 riboflavin synthase [EC:2.5.1.9] | ec:2.5.1.9 |
| dno:DNO\_0474 | ribD; riboflavin biosynthesis protein RibD (EC:1.1.1.193); K11752 diaminohydroxyphosphoribosylaminopyrimidine deaminase / 5-amino-6-(5-phosphoribosylamino)uracil reductase [EC:3.5.4.26 1.1.1.193] | ec:3.5.4.26 ec:1.1.1.193 |
| dno:DNO\_0475 | nrdR; hypothetical protein; K07738 transcriptional repressor NrdR |  |
| dno:DNO\_0476 | glyA; serine hydroxymethyltransferase (EC:2.1.2.1); K00600 glycine hydroxymethyltransferase [EC:2.1.2.1] | ec:2.1.2.1 |
| dno:DNO\_0477 | scpA; hypothetical protein; K05896 segregation and condensation protein A |  |
| dno:DNO\_0478 | hypothetical protein; K06024 segregation and condensation protein B |  |
| dno:DNO\_0479 | rluB; ribosomal large subunit pseudouridine synthase, RluB (EC:4.2.1.70); K06178 23S rRNA pseudouridine2605 synthase [EC:5.4.99.22] | ec:5.4.99.22 |

  
**Neighborhood Representations for "hdn:Hden\_1810"**  

| ID | Annotation | EC number |
| --- | --- | --- |
| hdn:Hden\_1800 | methionine synthase (EC:2.1.1.13); K00548 5-methyltetrahydrofolate--homocysteine methyltransferase [EC:2.1.1.13] | ec:2.1.1.13 |
| hdn:Hden\_1801 | homocysteine S-methyltransferase |  |
| hdn:Hden\_1802 | 5,10-methylenetetrahydrofolate reductase (EC:1.5.1.20); K00297 methylenetetrahydrofolate reductase (NADPH) [EC:1.5.1.20] | ec:1.5.1.20 |
| hdn:Hden\_1803 | ArsR family transcriptional regulator; K03892 ArsR family transcriptional regulator |  |
| hdn:Hden\_1804 | hypothetical protein |  |
| hdn:Hden\_1805 | toluene tolerance family protein; K07323 putative toluene tolerance protein |  |
| hdn:Hden\_1806 | hypothetical protein |  |
| hdn:Hden\_1807 | hypothetical protein |  |
| hdn:Hden\_1808 | ABC transporter |  |
| hdn:Hden\_1809 | hypothetical protein; K07276 hypothetical protein |  |
| hdn:Hden\_1810 | AsnC family transcriptional regulator |  |
| hdn:Hden\_1811 | hypothetical protein |  |
| hdn:Hden\_1812 | transcription elongation factor GreA; K03624 transcription elongation factor GreA |  |
| hdn:Hden\_1813 | carbamoyl-phosphate synthase, large subunit; K01955 carbamoyl-phosphate synthase large subunit [EC:6.3.5.5] | ec:6.3.5.5 |
| hdn:Hden\_1814 | major facilitator superfamily protein; K08223 MFS transporter, FSR family, fosmidomycin resistance protein |  |
| hdn:Hden\_1815 | hypothetical protein |  |
| hdn:Hden\_1816 | hypothetical protein |  |
| hdn:Hden\_1817 | carbamoyl-phosphate synthase, small subunit; K01956 carbamoyl-phosphate synthase small subunit [EC:6.3.5.5] | ec:6.3.5.5 |
| hdn:Hden\_1818 | hypothetical protein; K09117 hypothetical protein |  |
| hdn:Hden\_1819 | DNA primase; K02316 DNA primase [EC:2.7.7.-] |  |
| hdn:Hden\_1820 | RNA polymerase, sigma 70 subunit, RpoD; K03086 RNA polymerase primary sigma factor |  |

  
**Neighborhood Representations for "abb:ABBFA\_001491"**  

| ID | Annotation | EC number |
| --- | --- | --- |
| abb:ABBFA\_001481 | lpxA; UDP-N-acetylglucosamine acyltransferase (EC:2.3.1.129); K00677 UDP-N-acetylglucosamine acyltransferase [EC:2.3.1.129] | ec:2.3.1.129 |
| abb:ABBFA\_001482 | hypothetical protein |  |
| abb:ABBFA\_001483 | Regulatory protein recX; K03565 regulatory protein |  |
| abb:ABBFA\_001484 | recA; recombinase A; K03553 recombination protein RecA |  |
| abb:ABBFA\_001485 | Heat shock protein 15 (HSP15); K04762 ribosome-associated heat shock protein Hsp15 |  |
| abb:ABBFA\_001486 | HAD-superfamily hydrolase, subfamily IA, variant 3 family protein; K07025 putative hydrolase of the HAD superfamily |  |
| abb:ABBFA\_001487 | Fels-1 Prophage protein-like family protein |  |
| abb:ABBFA\_001488 | hypothetical protein |  |
| abb:ABBFA\_001489 | hypothetical protein |  |
| abb:ABBFA\_001490 | acetyltransferase (GNAT) family protein |  |
| abb:ABBFA\_001491 | AsnC family protein |  |
| abb:ABBFA\_001492 | kynU; kynureninase (EC:3.7.1.3); K01556 kynureninase [EC:3.7.1.3] | ec:3.7.1.3 |
| abb:ABBFA\_001493 | Proline-specific permease proY |  |
| abb:ABBFA\_001494 | hypothetical protein |  |
| abb:ABBFA\_001495 | Extracellular serine proteinase precursor |  |
| abb:ABBFA\_001496 | Sulfate transporter family protein; K03321 sulfate permease, SulP family |  |
| abb:ABBFA\_001497 | ACR protein |  |
| abb:ABBFA\_001498 | Quinoprotein glucose dehydrogenase-B precursor (EC:1.1.5.2); K00117 quinoprotein glucose dehydrogenase [EC:1.1.5.2] | ec:1.1.5.2 |
| abb:ABBFA\_001499 | Universal stress family protein |  |
| abb:ABBFA\_001500 | diguanylate cyclase (GGDEF) domain protein |  |
| abb:ABBFA\_001501 | hypothetical protein |  |

  
**Neighborhood Representations for "abn:AB57\_2300"**  

| ID | Annotation | EC number |
| --- | --- | --- |
| abn:AB57\_2290 | hypothetical protein |  |
| abn:AB57\_2291 | diguanylate cyclase |  |
| abn:AB57\_2292 | UspA domain-containing protein |  |
| abn:AB57\_2293 | quinoprotein glucose dehydrogenase (EC:1.1.5.2); K00117 quinoprotein glucose dehydrogenase [EC:1.1.5.2] | ec:1.1.5.2 |
| abn:AB57\_2294 | hypothetical protein |  |
| abn:AB57\_2295 | sulfate permease; K03321 sulfate permease, SulP family |  |
| abn:AB57\_2296 | extracellular serine protease (EC:3.4.21.111) |  |
| abn:AB57\_2297 | hypothetical protein |  |
| abn:AB57\_2298 | proline-specific permease ProY |  |
| abn:AB57\_2299 | kynU; kynureninase (EC:3.7.1.3); K01556 kynureninase [EC:3.7.1.3] | ec:3.7.1.3 |
| abn:AB57\_2300 | transcriptional regulator, AsnC family |  |
| abn:AB57\_2301 | acetyltransferase gnat family |  |
| abn:AB57\_2302 | hypothetical protein |  |
| abn:AB57\_2303 | hypothetical protein |  |
| abn:AB57\_2304 | putative prophage protein |  |
| abn:AB57\_2305 | HAD-superfamily hydrolase; K07025 putative hydrolase of the HAD superfamily |  |
| abn:AB57\_2306 | heat shock protein 15; K04762 ribosome-associated heat shock protein Hsp15 |  |
| abn:AB57\_2307 | recA; recombinase A; K03553 recombination protein RecA |  |
| abn:AB57\_2308 | recX; regulatory protein RecX; K03565 regulatory protein |  |
| abn:AB57\_2309 | hypothetical protein |  |
| abn:AB57\_2310 | lpxA; UDP-N-acetylglucosamine acyltransferase (EC:2.3.1.129); K00677 UDP-N-acetylglucosamine acyltransferase [EC:2.3.1.129] | ec:2.3.1.129 |

  
**Neighborhood Representations for "aby:ABAYE1598"**  

| ID | Annotation | EC number |
| --- | --- | --- |
| aby:ABAYE1587 | lpxA; UDP-N-acetylglucosamine acyltransferase (EC:2.3.1.129); K00677 UDP-N-acetylglucosamine acyltransferase [EC:2.3.1.129] | ec:2.3.1.129 |
| aby:ABAYE1588 | hypothetical protein |  |
| aby:ABAYE1589 | recX; regulatory protein; K03565 regulatory protein |  |
| aby:ABAYE1590 | recA; recombinase A; K03553 recombination protein RecA |  |
| aby:ABAYE1591 | hslR; heat shock protein 15; K04762 ribosome-associated heat shock protein Hsp15 |  |
| aby:ABAYE1592 | haloacid dehalogenase-like hydrolase; K07025 putative hydrolase of the HAD superfamily |  |
| aby:ABAYE1594 | signal peptide |  |
| aby:ABAYE1595 | hypothetical protein |  |
| aby:ABAYE1596 | hypothetical protein |  |
| aby:ABAYE1597 | acetyltransferase |  |
| aby:ABAYE1598 | AsnC family transcriptional regulator |  |
| aby:ABAYE1599 | L-kynurenine hydrolase (EC:3.7.1.3); K01556 kynureninase [EC:3.7.1.3] | ec:3.7.1.3 |
| aby:ABAYE1600 | amino acid permease |  |
| aby:ABAYE1601 | hypothetical protein |  |
| aby:ABAYE1602 | extracellular serine proteinase (EC:3.4.21.-); K01362 [EC:3.4.21.-] |  |
| aby:ABAYE1603 | sulfate transporter; K03321 sulfate permease, SulP family |  |
| aby:ABAYE1604 | hypothetical protein |  |
| aby:ABAYE1605 | gdhB; quinoprotein glucose dehydrogenase (EC:1.1.5.2); K00117 quinoprotein glucose dehydrogenase [EC:1.1.5.2] | ec:1.1.5.2 |
| aby:ABAYE1606 | stress protein |  |
| aby:ABAYE1607 | diguanylate cyclase/phosphodiesterase |  |
| aby:ABAYE1608 | hypothetical protein |  |

  
**Neighborhood Representations for "acd:AOLE\_07995"**  

| ID | Annotation | EC number |
| --- | --- | --- |
| acd:AOLE\_07945 | hypothetical protein |  |
| acd:AOLE\_07950 | hypothetical protein |  |
| acd:AOLE\_07955 | hypothetical protein |  |
| acd:AOLE\_07960 | hypothetical protein |  |
| acd:AOLE\_07965 | DNA-directed DNA polymerase UmuC; K03502 DNA polymerase V |  |
| acd:AOLE\_07970 | DNA polymerase V component; K03503 DNA polymerase V [EC:3.4.21.-] |  |
| acd:AOLE\_07975 | hypothetical protein |  |
| acd:AOLE\_07980 | hypothetical protein |  |
| acd:AOLE\_07985 | hypothetical protein |  |
| acd:AOLE\_07990 | acetyltransferase (GNAT) family protein |  |
| acd:AOLE\_07995 | transcriptional regulator |  |
| acd:AOLE\_08000 | kynureninase; K01556 kynureninase [EC:3.7.1.3] | ec:3.7.1.3 |
| acd:AOLE\_08005 | gamma-aminobutyrate permease |  |
| acd:AOLE\_08010 | hypothetical protein |  |
| acd:AOLE\_08015 | hypothetical protein |  |
| acd:AOLE\_08020 | Extracellular serine proteinase |  |
| acd:AOLE\_08025 | sulfate permease; K03321 sulfate permease, SulP family |  |
| acd:AOLE\_08030 | hypothetical protein |  |
| acd:AOLE\_08035 | quinoprotein glucose dehydrogenase; K00117 quinoprotein glucose dehydrogenase [EC:1.1.5.2] | ec:1.1.5.2 |
| acd:AOLE\_08040 | putative stress protein |  |
| acd:AOLE\_08045 | diguanylate cyclase (GGDEF) domain-containing protein |  |

  
**Neighborhood Representations for "abc:ACICU\_02078"**  

| ID | Annotation | EC number |
| --- | --- | --- |
| abc:ACICU\_02068 | signal transduction protein |  |
| abc:ACICU\_02069 | universal stress protein UspA |  |
| abc:ACICU\_02070 | glucose/sorbosone dehydrogenase; K00117 quinoprotein glucose dehydrogenase [EC:1.1.5.2] | ec:1.1.5.2 |
| abc:ACICU\_02071 | hypothetical protein |  |
| abc:ACICU\_02072 | Sulfate permease; K03321 sulfate permease, SulP family |  |
| abc:ACICU\_02073 | subtilisin-like serine protease |  |
| abc:ACICU\_02074 | esterase/lipase |  |
| abc:ACICU\_02075 | IS30 family transposase |  |
| abc:ACICU\_02076 | gamma-aminobutyrate permease |  |
| abc:ACICU\_02077 | kynureninase; K01556 kynureninase [EC:3.7.1.3] | ec:3.7.1.3 |
| abc:ACICU\_02078 | transcriptional regulator |  |
| abc:ACICU\_02079 | acetyltransferase |  |
| abc:ACICU\_02080 | hypothetical protein |  |
| abc:ACICU\_02081 | hypothetical protein |  |
| abc:ACICU\_02082 | putative prophage protein |  |
| abc:ACICU\_02083 | HAD superfamily hydrolase; K07025 putative hydrolase of the HAD superfamily |  |
| abc:ACICU\_02084 | ribosome-associated heat shock protein; K04762 ribosome-associated heat shock protein Hsp15 |  |
| abc:ACICU\_02085 | recA; recombinase A; K03553 recombination protein RecA |  |
| abc:ACICU\_02086 | hypothetical protein; K03565 regulatory protein |  |
| abc:ACICU\_02087 | hypothetical protein |  |
| abc:ACICU\_02088 | UDP-N-acetylglucosamine acyltransferase; K00677 UDP-N-acetylglucosamine acyltransferase [EC:2.3.1.129] | ec:2.3.1.129 |

  
**Neighborhood Representations for "abm:ABSDF1702"**  

| ID | Annotation | EC number |
| --- | --- | --- |
| abm:ABSDF1691 | hypothetical protein |  |
| abm:ABSDF1693 | recX; regulatory protein; K03565 regulatory protein |  |
| abm:ABSDF1694 | recA; recombinase A; K03553 recombination protein RecA |  |
| abm:ABSDF1695 | hslR; heat shock protein 15; K04762 ribosome-associated heat shock protein Hsp15 |  |
| abm:ABSDF1696 | pseudogene |  |
| abm:ABSDF1697 | pseudogene |  |
| abm:ABSDF1698 | signal peptide |  |
| abm:ABSDF1699 | hypothetical protein |  |
| abm:ABSDF1700 | IS982 family transposase |  |
| abm:ABSDF1701 | acetyltransferase |  |
| abm:ABSDF1702 | AsnC family transcriptional regulator |  |
| abm:ABSDF1703 | L-kynurenine hydrolase (EC:3.7.1.3); K01556 kynureninase [EC:3.7.1.3] | ec:3.7.1.3 |
| abm:ABSDF1704 | amino acid permease |  |
| abm:ABSDF1705 | pseudogene |  |
| abm:ABSDF1706 | IS5 family transposase |  |
| abm:ABSDF1707 | hypothetical protein |  |
| abm:ABSDF1708 | hypothetical protein |  |
| abm:ABSDF1709 | hypothetical protein; K09966 hypothetical protein |  |
| abm:ABSDF1710 | pgk; phosphoglycerate kinase (EC:2.7.2.3); K00927 phosphoglycerate kinase [EC:2.7.2.3] | ec:2.7.2.3 |
| abm:ABSDF1711 | hypothetical protein |  |
| abm:ABSDF1712 | fda; fructose-1,6-bisphosphate aldolase (EC:4.1.2.13); K01624 fructose-bisphosphate aldolase, class II [EC:4.1.2.13] | ec:4.1.2.13 |

  
**Neighborhood Representations for "acb:A1S\_1958"**  

| ID | Annotation | EC number |
| --- | --- | --- |
| acb:A1S\_1948 | MarR family multidrug resistance pump transcriptional regulator |  |
| acb:A1S\_1949 | diguanylate cyclase |  |
| acb:A1S\_1950 | universal stress protein |  |
| acb:A1S\_1951 | hypothetical protein; K00117 quinoprotein glucose dehydrogenase [EC:1.1.5.2] | ec:1.1.5.2 |
| acb:A1S\_1952 | hypothetical protein |  |
| acb:A1S\_1953 | sulfate transporter; K03321 sulfate permease, SulP family |  |
| acb:A1S\_1954 | serine proteinase |  |
| acb:A1S\_1955 | hypothetical protein |  |
| acb:A1S\_1956 | amino acid permease |  |
| acb:A1S\_1957 | L-kynurenine hydrolase; K01556 kynureninase [EC:3.7.1.3] | ec:3.7.1.3 |
| acb:A1S\_1958 | transcriptional regulator |  |
| acb:A1S\_1959 | acetyltransferase |  |
| acb:A1S\_1960 | HAD superfamily hydrolase; K07025 putative hydrolase of the HAD superfamily |  |
| acb:A1S\_1961 | heat shock protein 15; K04762 ribosome-associated heat shock protein Hsp15 |  |
| acb:A1S\_1962 | recA; recombinase A; K03553 recombination protein RecA |  |
| acb:A1S\_1963 | regulatory protein; K03565 regulatory protein |  |
| acb:A1S\_1964 | signal peptide |  |
| acb:A1S\_1965 | UDP-N-acetylglucosamine acyltransferase (EC:2.3.1.129); K00677 UDP-N-acetylglucosamine acyltransferase [EC:2.3.1.129] | ec:2.3.1.129 |
| acb:A1S\_1966 | fabZ; (3R)-hydroxymyristoyl-ACP dehydratase; K02372 3-hydroxyacyl-[acyl-carrier-protein] dehydratase [EC:4.2.1.59] | ec:4.2.1.59 |
| acb:A1S\_1967 | lpxD; UDP-3-O-[3-hydroxymyristoyl] glucosamine N-acyltransferase; K02536 UDP-3-O-[3-hydroxymyristoyl] glucosamine N-acyltransferase [EC:2.3.1.191] | ec:2.3.1.191 |
| acb:A1S\_1968 | outer membrane protein (OmpH); K06142 outer membrane protein |  |

  
**Neighborhood Representations for "rlt:Rleg2\_2720"**  

| ID | Annotation | EC number |
| --- | --- | --- |
| rlt:Rleg2\_2710 | aldo/keto reductase |  |
| rlt:Rleg2\_2711 | hypothetical protein; K07054 |  |
| rlt:Rleg2\_2712 | carB; carbamoyl phosphate synthase large subunit; K01955 carbamoyl-phosphate synthase large subunit [EC:6.3.5.5] | ec:6.3.5.5 |
| rlt:Rleg2\_2713 | phage SPO1 DNA polymerase-like protein; K02334 DNA polymerase bacteriophage-type [EC:2.7.7.7] | ec:2.7.7.7 |
| rlt:Rleg2\_2714 | radical SAM protein |  |
| rlt:Rleg2\_2715 | greA; transcription elongation factor GreA; K03624 transcription elongation factor GreA |  |
| rlt:Rleg2\_2716 | group 1 glycosyl transferase; K12989 mannosyltransferase [EC:2.4.1.-] |  |
| rlt:Rleg2\_2717 | C4-dicarboxylate transporter DctA; K11103 aerobic C4-dicarboxylate transport protein |  |
| rlt:Rleg2\_2718 | histidine kinase; K10125 two-component system, NtrC family, C4-dicarboxylate transport sensor histidine kinase DctB [EC:2.7.13.3] | ec:2.7.13.3 |
| rlt:Rleg2\_2719 | Fis family transcriptional regulator; K10126 two-component system, NtrC family, C4-dicarboxylate transport response regulator DctD |  |
| rlt:Rleg2\_2720 | AsnC family transcriptional regulator |  |
| rlt:Rleg2\_2721 | thioredoxin reductase; K00384 thioredoxin reductase (NADPH) [EC:1.8.1.9] | ec:1.8.1.9 |
| rlt:Rleg2\_2722 | LysR family transcriptional regulator |  |
| rlt:Rleg2\_2723 | ArsR family transcriptional regulator; K03892 ArsR family transcriptional regulator |  |
| rlt:Rleg2\_2724 | major facilitator superfamily protein; K08156 MFS transporter, DHA1 family, arabinose polymer transporter |  |
| rlt:Rleg2\_2725 | NADH:flavin oxidoreductase; K10680 N-ethylmaleimide reductase [EC:1.-.-.-] |  |
| rlt:Rleg2\_2726 | aminoglycoside/hydroxyurea antibiotic resistance kinase; K04343 streptomycin 6-kinase [EC:2.7.1.72] | ec:2.7.1.72 |
| rlt:Rleg2\_2727 | NAD-dependent epimerase/dehydratase |  |
| rlt:Rleg2\_2728 | LysR family transcriptional regulator |  |
| rlt:Rleg2\_2729 | hypothetical protein |  |
| rlt:Rleg2\_2730 | hypothetical protein |  |

  
**Neighborhood Representations for "sme:SMc01223"**  

| ID | Annotation | EC number |
| --- | --- | --- |
| sme:SMc01233 | ssb; single-stranded DNA-binding protein; K03111 single-strand DNA-binding protein |  |
| sme:SMc01232 | hypothetical protein; K05595 multiple antibiotic resistance protein |  |
| sme:SMc01231 | gyrA; DNA gyrase subunit A (EC:5.99.1.3); K02469 DNA gyrase subunit A [EC:5.99.1.3] | ec:5.99.1.3 |
| sme:SMc01230 | transglycosylase transmembrane protein (EC:3.2.1.-) |  |
| sme:SMc01229 | acetyltransferase; K03823 phosphinothricin acetyltransferase [EC:2.3.1.183] | ec:2.3.1.183 |
| sme:SMc01228 | antibiotic resistance (kinase) protein (EC:2.7.1.-); K04343 streptomycin 6-kinase [EC:2.7.1.72] | ec:2.7.1.72 |
| sme:SMc01227 | nerA; glycerol trinitrate (GTN) reductase (EC:1.-.-.-); K10680 N-ethylmaleimide reductase [EC:1.-.-.-] |  |
| sme:SMc01226 | transcriptional regulator; K03892 ArsR family transcriptional regulator |  |
| sme:SMc01225 | transcriptional regulator |  |
| sme:SMc01224 | trxB; thioredoxin reductase; K00384 thioredoxin reductase (NADPH) [EC:1.8.1.9] | ec:1.8.1.9 |
| sme:SMc01223 | transcriptional regulator |  |
| sme:SMc01222 | lpsC; lipopolysaccharide core biosynthesis glycosyl transferase (EC:2.-.-.-) |  |
| sme:SMc01221 | lpsD; lipopolysaccharide core biosynthesis glycosyl transferase (EC:2.-.-.-) |  |
| sme:SMc01220 | lpsE; lipopolysaccharide core biosynthesis glycosyl transferase (EC:2.-.-.-) |  |
| sme:SMc01219 | lpsB; lipopolysaccharide core biosynthesis mannosyltransferase (EC:2.7.-.-); K12989 mannosyltransferase [EC:2.4.1.-] |  |
| sme:SMc01218 | greA; transcription elongation factor GreA; K03624 transcription elongation factor GreA |  |
| sme:SMc01217 | transport transmembrane protein |  |
| sme:SMc01216 | hypothetical protein |  |
| sme:SMc01215 | carB; carbamoyl phosphate synthase large subunit (EC:6.3.5.5); K01955 carbamoyl-phosphate synthase large subunit [EC:6.3.5.5] | ec:6.3.5.5 |
| sme:SMc01214 | zinc-containing alcohol dehydrogenase (EC:1.1.1.-) |  |
| sme:SMc01213 | hypothetical protein; K07054 |  |

  
**Neighborhood Representations for "ret:RHE\_CH02974"**  

| ID | Annotation | EC number |
| --- | --- | --- |
| ret:RHE\_CH02964 | zinc protease; K07054 |  |
| ret:RHE\_CH02965 | L-iditol 2-dehydrogenase (EC:1.1.1.14); K00008 L-iditol 2-dehydrogenase [EC:1.1.1.14] | ec:1.1.1.14 |
| ret:RHE\_CH02966 | carB; carbamoyl phosphate synthase large subunit (EC:6.3.5.5); K01955 carbamoyl-phosphate synthase large subunit [EC:6.3.5.5] | ec:6.3.5.5 |
| ret:RHE\_CH02967 | uracil DNA glycosylase (EC:2.7.7.7); K02334 DNA polymerase bacteriophage-type [EC:2.7.7.7] | ec:2.7.7.7 |
| ret:RHE\_CH02968 | hypothetical protein |  |
| ret:RHE\_CH02969 | greA; transcription elongation factor GreA; K03624 transcription elongation factor GreA |  |
| ret:RHE\_CH02970 | lpcC; lipopolysaccharide core biosynthesis mannosyltransferase; K12989 mannosyltransferase [EC:2.4.1.-] |  |
| ret:RHE\_CH02971 | dctA; C4-dicarboxylate transporter DctA; K11103 aerobic C4-dicarboxylate transport protein |  |
| ret:RHE\_CH02972 | dctB; two-component sensor histidine kinase regulating C4-dicarboxylate transport system; K10125 two-component system, NtrC family, C4-dicarboxylate transport sensor histidine kinase DctB [EC:2.7.13.3] | ec:2.7.13.3 |
| ret:RHE\_CH02973 | dctD; two-component response regulator protein regulating C4-dicarboxylate transport system; K10126 two-component system, NtrC family, C4-dicarboxylate transport response regulator DctD |  |
| ret:RHE\_CH02974 | lrp; AsnC family leucine-responsive regulatory protein |  |
| ret:RHE\_CH02975 | trxBch; thioredoxin reductase (NADPH) protein (EC:1.8.1.9); K00384 thioredoxin reductase (NADPH) [EC:1.8.1.9] | ec:1.8.1.9 |
| ret:RHE\_CH02976 | LysR family transcriptional regulator |  |
| ret:RHE\_CH02977 | ArsR family transcriptional regulator; K03892 ArsR family transcriptional regulator |  |
| ret:RHE\_CH02978 | MFS family transporter; K08156 MFS transporter, DHA1 family, arabinose polymer transporter |  |
| ret:RHE\_CH02979 | nerA; glycerol trinitrate reductase; K10680 N-ethylmaleimide reductase [EC:1.-.-.-] |  |
| ret:RHE\_CH02980 | antibiotic resistance (streptomycin kinase) protein; K04343 streptomycin 6-kinase [EC:2.7.1.72] | ec:2.7.1.72 |
| ret:RHE\_CH02981 | LacI family transcriptional regulator; K02529 LacI family transcriptional regulator |  |
| ret:RHE\_CH02982 | sugar ABC transporter, substrate-binding protein; K02027 multiple sugar transport system substrate-binding protein |  |
| ret:RHE\_CH02983 | sugar ABC transporter, permease; K02025 multiple sugar transport system permease protein |  |
| ret:RHE\_CH02984 | sugar ABC transporter, permease; K02026 multiple sugar transport system permease protein |  |

  
**Neighborhood Representations for "ara:Arad\_3141"**  

| ID | Annotation | EC number |
| --- | --- | --- |
| ara:Arad\_3129 | multidrug efflux protein; K03327 multidrug resistance protein, MATE family |  |
| ara:Arad\_3131 | transporter; K08151 MFS transporter, DHA1 family, tetracycline resistance protein |  |
| ara:Arad\_3132 | zinc protease; K07054 |  |
| ara:Arad\_3133 | hypothetical protein |  |
| ara:Arad\_3134 | hypothetical protein |  |
| ara:Arad\_3135 | carB; carbamoyl phosphate synthase large subunit; K01955 carbamoyl-phosphate synthase large subunit [EC:6.3.5.5] | ec:6.3.5.5 |
| ara:Arad\_3136 | greA; transcription elongation factor GreA; K03624 transcription elongation factor GreA |  |
| ara:Arad\_3138 | lpcC; lipopolysaccharide core biosynthesis mannosyltransferase; K12989 mannosyltransferase [EC:2.4.1.-] |  |
| ara:Arad\_3139 | hypothetical protein |  |
| ara:Arad\_3140 | glycosyltransferase |  |
| ara:Arad\_3141 | lrp; leucine-responsive transcriptional regulator protein |  |
| ara:Arad\_3143 | trxB; thioredoxin-disulfide reductase; K00384 thioredoxin reductase (NADPH) [EC:1.8.1.9] | ec:1.8.1.9 |
| ara:Arad\_3145 | transcriptional regulator |  |
| ara:Arad\_5113 | suhB; suhB |  |
| ara:Arad\_3147 | transcriptional regulator; K03892 ArsR family transcriptional regulator |  |
| ara:Arad\_3148 | nerA; glycerol trinitrate reductase; K10680 N-ethylmaleimide reductase [EC:1.-.-.-] |  |
| ara:Arad\_3150 | antibiotic resistance (streptomycin kinase) protein; K04343 streptomycin 6-kinase [EC:2.7.1.72] | ec:2.7.1.72 |
| ara:Arad\_3151 | hypothetical protein |  |
| ara:Arad\_3152 | transcriptional regulator |  |
| ara:Arad\_3153 | hypothetical protein |  |
| ara:Arad\_3154 | chaA; LPS-associated cation exporter (Ca2+/H+ antiporter) protein; K07300 Ca2+:H+ antiporter |  |

  
**Neighborhood Representations for "bcs:BCAN\_A1539"**  

| ID | Annotation | EC number |
| --- | --- | --- |
| bcs:BCAN\_A1529 | NUDIX hydrolase |  |
| bcs:BCAN\_A1530 | hypothetical protein; K03704 cold shock protein (beta-ribbon, CspA family) |  |
| bcs:BCAN\_A1531 | gstA; protein gstA; K00799 glutathione S-transferase [EC:2.5.1.18] | ec:2.5.1.18 |
| bcs:BCAN\_A1532 | aspC; aspartate aminotransferase; K00812 aspartate aminotransferase [EC:2.6.1.1] | ec:2.6.1.1 |
| bcs:BCAN\_A1533 | hypothetical protein |  |
| bcs:BCAN\_A1534 | hypothetical protein |  |
| bcs:BCAN\_A1535 | hypothetical protein |  |
| bcs:BCAN\_A1536 | trxB; thioredoxin-disulfide reductase; K00384 thioredoxin reductase (NADPH) [EC:1.8.1.9] | ec:1.8.1.9 |
| bcs:BCAN\_A1537 | cation transporter; K03498 trk system potassium uptake protein TrkH |  |
| bcs:BCAN\_A1538 | hypothetical protein |  |
| bcs:BCAN\_A1539 | lrp; leucine-responsive regulatory protein |  |
| bcs:BCAN\_A1540 | lpcC; lipopolysaccharide core biosynthesis mannosyltransferase lpcC; K12989 mannosyltransferase [EC:2.4.1.-] |  |
| bcs:BCAN\_A1541 | greA; transcription elongation factor GreA; K03624 transcription elongation factor GreA |  |
| bcs:BCAN\_A1542 | hypothetical protein |  |
| bcs:BCAN\_A1543 | hypothetical protein |  |
| bcs:BCAN\_A1544 | hypothetical protein |  |
| bcs:BCAN\_A1545 | hypothetical protein; K09922 hypothetical protein |  |
| bcs:BCAN\_A1546 | extensin family protein |  |
| bcs:BCAN\_A1547 | thioesterase superfamily protein; K10806 acyl-CoA thioesterase YciA [EC:3.1.2.-] |  |
| bcs:BCAN\_A1548 | SH3 type 3 domain-containing protein |  |
| bcs:BCAN\_A1549 | uvrB; excinuclease ABC subunit B; K03702 excinuclease ABC subunit B |  |

  
**Neighborhood Representations for "bme:BMEI0510"**  

| ID | Annotation | EC number |
| --- | --- | --- |
| bme:BMEI0500 | soluble lytic murein transglycosylase (EC:3.2.1.-); K01238 [EC:3.2.1.-] |  |
| bme:BMEI0501 | excinuclease ABC subunit B; K03702 excinuclease ABC subunit B |  |
| bme:BMEI0502 | hypothetical protein |  |
| bme:BMEI0503 | acyl-CoA hydrolase (EC:3.1.2.20); K10806 acyl-CoA thioesterase YciA [EC:3.1.2.-] |  |
| bme:BMEI0504 | extensin |  |
| bme:BMEI0505 | hypothetical protein; K09922 hypothetical protein |  |
| bme:BMEI0506 | transporter, DME family |  |
| bme:BMEI0507 | hypothetical protein |  |
| bme:BMEI0508 | greA; transcription elongation factor GreA; K03624 transcription elongation factor GreA |  |
| bme:BMEI0509 | lipopolysaccharide core biosynthesis mannosyltransferase LPCC (EC:2.-.-.-); K12989 mannosyltransferase [EC:2.4.1.-] |  |
| bme:BMEI0510 | leucine-responsive regulatory protein |  |
| bme:BMEI0511 | TRK system potassium uptake protein TRKH; K03498 trk system potassium uptake protein TrkH |  |
| bme:BMEI0512 | thioredoxin reductase (EC:1.8.1.9); K00384 thioredoxin reductase (NADPH) [EC:1.8.1.9] | ec:1.8.1.9 |
| bme:BMEI0513 | LysR family transcriptional regulator |  |
| bme:BMEI0514 | hypothetical protein |  |
| bme:BMEI0515 | hypothetical protein |  |
| bme:BMEI0516 | aspartate aminotransferase (EC:2.6.1.1); K00812 aspartate aminotransferase [EC:2.6.1.1] | ec:2.6.1.1 |
| bme:BMEI0517 | glutathione S-transferase (EC:2.5.1.18); K00799 glutathione S-transferase [EC:2.5.1.18] | ec:2.5.1.18 |
| bme:BMEI0518 | cold shock protein CSPA; K03704 cold shock protein (beta-ribbon, CspA family) |  |
| bme:BMEI0519 | phosphohydrolase |  |
| bme:BMEI0520 | ABC transporter ATP-binding protein; K02471 putative ATP-binding cassette transporter |  |

  
**Neighborhood Representations for "bmi:BMEA\_A1553"**  

| ID | Annotation | EC number |
| --- | --- | --- |
| bmi:BMEA\_A1543 | ABC transporter; K02471 putative ATP-binding cassette transporter |  |
| bmi:BMEA\_A1544 | NUDIX hydrolase |  |
| bmi:BMEA\_A1545 | hypothetical protein; K03704 cold shock protein (beta-ribbon, CspA family) |  |
| bmi:BMEA\_A1546 | protein GstA; K00799 glutathione S-transferase [EC:2.5.1.18] | ec:2.5.1.18 |
| bmi:BMEA\_A1547 | aspartate aminotransferase; K00812 aspartate aminotransferase [EC:2.6.1.1] | ec:2.6.1.1 |
| bmi:BMEA\_A1548 | hypothetical protein |  |
| bmi:BMEA\_A1549 | hypothetical protein |  |
| bmi:BMEA\_A1550 | trxB; thioredoxin-disulfide reductase (EC:2.1.1.83); K00384 thioredoxin reductase (NADPH) [EC:1.8.1.9] | ec:1.8.1.9 |
| bmi:BMEA\_A1551 | cation transporter; K03498 trk system potassium uptake protein TrkH |  |
| bmi:BMEA\_A1552 | hypothetical protein |  |
| bmi:BMEA\_A1553 | leucine-responsive regulatory protein |  |
| bmi:BMEA\_A1554 | lipopolysaccharide core biosynthesis mannosyltransferase lpcC; K12989 mannosyltransferase [EC:2.4.1.-] |  |
| bmi:BMEA\_A1555 | greA; transcription elongation factor GreA; K03624 transcription elongation factor GreA |  |
| bmi:BMEA\_A1556 | hypothetical protein |  |
| bmi:BMEA\_A1557 | hypothetical protein |  |
| bmi:BMEA\_A1558 | hypothetical protein |  |
| bmi:BMEA\_A1559 | hypothetical protein; K09922 hypothetical protein |  |
| bmi:BMEA\_A1560 | extensin family protein |  |
| bmi:BMEA\_A1561 | thioesterase superfamily protein; K10806 acyl-CoA thioesterase YciA [EC:3.1.2.-] |  |
| bmi:BMEA\_A1562 | pseudogene |  |
| bmi:BMEA\_A1563 | uvrB; excinuclease ABC subunit B; K03702 excinuclease ABC subunit B |  |

  
**Neighborhood Representations for "bmr:BMI\_I1516"**  

| ID | Annotation | EC number |
| --- | --- | --- |
| bmr:BMI\_I1506 | MutT/nudix family protein |  |
| bmr:BMI\_I1507 | cold-shock family protein; K03704 cold shock protein (beta-ribbon, CspA family) |  |
| bmr:BMI\_I1508 | glutathione S-transferase; K00799 glutathione S-transferase [EC:2.5.1.18] | ec:2.5.1.18 |
| bmr:BMI\_I1509 | hypothetical protein |  |
| bmr:BMI\_I1510 | aspC; aspartate aminotransferase (EC:2.6.1.1); K00812 aspartate aminotransferase [EC:2.6.1.1] | ec:2.6.1.1 |
| bmr:BMI\_I1511 | hypothetical protein |  |
| bmr:BMI\_I1512 | LysR family transcriptional regulator |  |
| bmr:BMI\_I1513 | trxB; thioredoxin reductase (EC:1.8.1.9); K00384 thioredoxin reductase (NADPH) [EC:1.8.1.9] | ec:1.8.1.9 |
| bmr:BMI\_I1514 | cation transport protein; K03498 trk system potassium uptake protein TrkH |  |
| bmr:BMI\_I1515 | hypothetical protein |  |
| bmr:BMI\_I1516 | lrp-1; leucine-responsive regulatory protein |  |
| bmr:BMI\_I1517 | lpcC; lipopolysaccharide core biosynthesis mannosyltransferase LpcC (EC:2.-.-.-); K12989 mannosyltransferase [EC:2.4.1.-] |  |
| bmr:BMI\_I1518 | greA; transcription elongation factor GreA; K03624 transcription elongation factor GreA |  |
| bmr:BMI\_I1519 | hypothetical protein |  |
| bmr:BMI\_I1520 | hypothetical protein |  |
| bmr:BMI\_I1521 | hypothetical protein |  |
| bmr:BMI\_I1522 | hypothetical protein; K09922 hypothetical protein |  |
| bmr:BMI\_I1523 | hypothetical protein |  |
| bmr:BMI\_I1524 | long-chain acyl-CoA thioester hydrolase, putative; K10806 acyl-CoA thioesterase YciA [EC:3.1.2.-] |  |
| bmr:BMI\_I1525 | SH3 type 3 domain-containing protein |  |
| bmr:BMI\_I1526 | uvrB; excinuclease ABC subunit B; K03702 excinuclease ABC subunit B |  |

  
**Neighborhood Representations for "bms:BR1502"**  

| ID | Annotation | EC number |
| --- | --- | --- |
| bms:BR1492 | cold-shock family protein; K03704 cold shock protein (beta-ribbon, CspA family) |  |
| bms:BR1493 | glutathione S-transferase; K00799 glutathione S-transferase [EC:2.5.1.18] | ec:2.5.1.18 |
| bms:BR1494 | hypothetical protein |  |
| bms:BR1495 | aspC; aspartate aminotransferase (EC:2.6.1.1); K00812 aspartate aminotransferase [EC:2.6.1.1] | ec:2.6.1.1 |
| bms:BR1496 | hypothetical protein |  |
| bms:BR1497 | hypothetical protein |  |
| bms:BR1498 | LysR family transcriptional regulator |  |
| bms:BR1499 | trxB; thioredoxin reductase (EC:1.8.1.9); K00384 thioredoxin reductase (NADPH) [EC:1.8.1.9] | ec:1.8.1.9 |
| bms:BR1500 | cation transport protein; K03498 trk system potassium uptake protein TrkH |  |
| bms:BR1501 | hypothetical protein |  |
| bms:BR1502 | lrp-1; leucine-responsive regulatory protein |  |
| bms:BR1503 | lpcC; lipopolysaccharide core biosynthesis mannosyltransferase LpcC (EC:2.-.-.-); K12989 mannosyltransferase [EC:2.4.1.-] |  |
| bms:BR1504 | greA; transcription elongation factor GreA; K03624 transcription elongation factor GreA |  |
| bms:BR1505 | hypothetical protein |  |
| bms:BR1506 | hypothetical protein |  |
| bms:BR1507 | hypothetical protein |  |
| bms:BR1508 | hypothetical protein; K09922 hypothetical protein |  |
| bms:BR1509 | hypothetical protein |  |
| bms:BR1510 | long-chain acyl-CoA thioester hydrolase; K10806 acyl-CoA thioesterase YciA [EC:3.1.2.-] |  |
| bms:BR1511 | hypothetical protein |  |
| bms:BR1512 | uvrB; excinuclease ABC subunit B; K03702 excinuclease ABC subunit B |  |

  
**Neighborhood Representations for "bmt:BSUIS\_A1558"**  

| ID | Annotation | EC number |
| --- | --- | --- |
| bmt:BSUIS\_A1548 | NUDIX hydrolase |  |
| bmt:BSUIS\_A1549 | hypothetical protein; K03704 cold shock protein (beta-ribbon, CspA family) |  |
| bmt:BSUIS\_A1550 | gstA; protein gstA; K00799 glutathione S-transferase [EC:2.5.1.18] | ec:2.5.1.18 |
| bmt:BSUIS\_A1551 | hypothetical protein |  |
| bmt:BSUIS\_A1552 | aspC; aspartate aminotransferase; K00812 aspartate aminotransferase [EC:2.6.1.1] | ec:2.6.1.1 |
| bmt:BSUIS\_A1553 | hypothetical protein |  |
| bmt:BSUIS\_A1554 | hypothetical protein |  |
| bmt:BSUIS\_A1555 | trxB; thioredoxin-disulfide reductase; K00384 thioredoxin reductase (NADPH) [EC:1.8.1.9] | ec:1.8.1.9 |
| bmt:BSUIS\_A1556 | cation transporter; K03498 trk system potassium uptake protein TrkH |  |
| bmt:BSUIS\_A1557 | hypothetical protein |  |
| bmt:BSUIS\_A1558 | lrp; leucine-responsive regulatory protein |  |
| bmt:BSUIS\_A1559 | lpcC; lipopolysaccharide core biosynthesis mannosyltransferase lpcC; K12989 mannosyltransferase [EC:2.4.1.-] |  |
| bmt:BSUIS\_A1560 | greA; transcription elongation factor GreA; K03624 transcription elongation factor GreA |  |
| bmt:BSUIS\_A1561 | hypothetical protein |  |
| bmt:BSUIS\_A1562 | hypothetical protein |  |
| bmt:BSUIS\_A1563 | hypothetical protein |  |
| bmt:BSUIS\_A1564 | hypothetical protein; K09922 hypothetical protein |  |
| bmt:BSUIS\_A1565 | extensin family protein |  |
| bmt:BSUIS\_A1566 | thioesterase superfamily protein; K10806 acyl-CoA thioesterase YciA [EC:3.1.2.-] |  |
| bmt:BSUIS\_A1567 | SH3 type 3 domain-containing protein |  |
| bmt:BSUIS\_A1568 | uvrB; excinuclease ABC subunit B; K03702 excinuclease ABC subunit B |  |

  
**Neighborhood Representations for "bov:BOV\_1452"**  

| ID | Annotation | EC number |
| --- | --- | --- |
| bov:BOV\_1442 | hypothetical protein |  |
| bov:BOV\_1443 | carB; carbamoyl phosphate synthase large subunit (EC:6.3.5.5); K01955 carbamoyl-phosphate synthase large subunit [EC:6.3.5.5] | ec:6.3.5.5 |
| bov:BOV\_1444 | putative transporter; K02471 putative ATP-binding cassette transporter |  |
| bov:BOV\_1445 | MutT/nudix family protein |  |
| bov:BOV\_1446 | cold-shock family protein; K03704 cold shock protein (beta-ribbon, CspA family) |  |
| bov:BOV\_1447 | glutathione S-transferase family protein; K00799 glutathione S-transferase [EC:2.5.1.18] | ec:2.5.1.18 |
| bov:BOV\_1448 | aspC; aspartate aminotransferase (EC:2.6.1.1); K00812 aspartate aminotransferase [EC:2.6.1.1] | ec:2.6.1.1 |
| bov:BOV\_1449 | LysR family transcriptional regulator |  |
| bov:BOV\_1450 | trxB; thioredoxin-disulfide reductase (EC:1.8.1.9); K00384 thioredoxin reductase (NADPH) [EC:1.8.1.9] | ec:1.8.1.9 |
| bov:BOV\_1451 | cation transport protein; K03498 trk system potassium uptake protein TrkH |  |
| bov:BOV\_1452 | lrp-1; leucine-responsive regulatory protein |  |
| bov:BOV\_1453 | lpcC; lipopolysaccharide core biosynthesis mannosyltransferase LpcC (EC:2.-.-.-); K12989 mannosyltransferase [EC:2.4.1.-] |  |
| bov:BOV\_1454 | greA; transcription elongation factor GreA; K03624 transcription elongation factor GreA |  |
| bov:BOV\_1455 | hypothetical protein |  |
| bov:BOV\_1456 | hypothetical protein |  |
| bov:BOV\_1457 | hypothetical protein |  |
| bov:BOV\_1458 | hypothetical protein; K09922 hypothetical protein |  |
| bov:BOV\_1459 | hypothetical protein |  |
| bov:BOV\_1460 | putative long-chain acyl-CoA thioester hydrolase; K10806 acyl-CoA thioesterase YciA [EC:3.1.2.-] |  |
| bov:BOV\_1461 | hypothetical protein |  |
| bov:BOV\_1462 | excinuclease ABC subunit B; K03702 excinuclease ABC subunit B |  |

  
**Neighborhood Representations for "bmb:BruAb1\_1495"**  

| ID | Annotation | EC number |
| --- | --- | --- |
| bmb:BruAb1\_1485 | MutT/nudix family protein |  |
| bmb:BruAb1\_1486 | cold-shock family protein; K03704 cold shock protein (beta-ribbon, CspA family) |  |
| bmb:BruAb1\_1487 | glutathione S-transferase family protein; K00799 glutathione S-transferase [EC:2.5.1.18] | ec:2.5.1.18 |
| bmb:BruAb1\_1488 | aspC; aspartate aminotransferase (EC:2.6.1.1); K00812 aspartate aminotransferase [EC:2.6.1.1] | ec:2.6.1.1 |
| bmb:BruAb1\_1489 | hypothetical protein |  |
| bmb:BruAb1\_1490 | hypothetical protein |  |
| bmb:BruAb1\_1491 | LysR family transcriptional regulator |  |
| bmb:BruAb1\_1492 | trxB; thioredoxin reductase; K00384 thioredoxin reductase (NADPH) [EC:1.8.1.9] | ec:1.8.1.9 |
| bmb:BruAb1\_1493 | cation transport protein; K03498 trk system potassium uptake protein TrkH |  |
| bmb:BruAb1\_1494 | hypothetical protein |  |
| bmb:BruAb1\_1495 | lrp-1; Lrp-1, leucine-responsive regulatory protein |  |
| bmb:BruAb1\_1496 | lpcC; lipopolysaccharide core biosynthesis mannosyltransferase; K12989 mannosyltransferase [EC:2.4.1.-] |  |
| bmb:BruAb1\_1497 | greA; transcription elongation factor GreA; K03624 transcription elongation factor GreA |  |
| bmb:BruAb1\_1498 | hypothetical protein |  |
| bmb:BruAb1\_1499 | hypothetical protein |  |
| bmb:BruAb1\_1500 | hypothetical protein; K09922 hypothetical protein |  |
| bmb:BruAb1\_1501 | hypothetical protein |  |
| bmb:BruAb1\_1502 | long-chain acyl-CoA thioester hydrolase; K10806 acyl-CoA thioesterase YciA [EC:3.1.2.-] |  |
| bmb:BruAb1\_1503 | hypothetical protein |  |
| bmb:BruAb1\_1504 | uvrB; excinuclease ABC subunit B; K03702 excinuclease ABC subunit B |  |
| bmb:BruAb1\_1505 | transglycosylase |  |

  
**Neighborhood Representations for "bmc:BAbS19\_I14170"**  

| ID | Annotation | EC number |
| --- | --- | --- |
| bmc:BAbS19\_I14070 | carB; carbamoyl phosphate synthase large subunit; K01955 carbamoyl-phosphate synthase large subunit [EC:6.3.5.5] | ec:6.3.5.5 |
| bmc:BAbS19\_I14080 | ABC transporter; K02471 putative ATP-binding cassette transporter |  |
| bmc:BAbS19\_I14090 | NUDIX hydrolase |  |
| bmc:BAbS19\_I14100 | Cold-shock DNA-binding domain protein; K03704 cold shock protein (beta-ribbon, CspA family) |  |
| bmc:BAbS19\_I14110 | glutathione S-transferase; K00799 glutathione S-transferase [EC:2.5.1.18] | ec:2.5.1.18 |
| bmc:BAbS19\_I14120 | aspartate aminotransferase; K00812 aspartate aminotransferase [EC:2.6.1.1] | ec:2.6.1.1 |
| bmc:BAbS19\_I14130 | hypothetical protein |  |
| bmc:BAbS19\_I14140 | LysR family transcriptional regulator |  |
| bmc:BAbS19\_I14150 | TrxB, thioredoxin reductase; K00384 thioredoxin reductase (NADPH) [EC:1.8.1.9] | ec:1.8.1.9 |
| bmc:BAbS19\_I14160 | Pyrokinin; K03498 trk system potassium uptake protein TrkH |  |
| bmc:BAbS19\_I14170 | AsnC family regulatory protein |  |
| bmc:BAbS19\_I14180 | group 1 glycosyl transferase; K12989 mannosyltransferase [EC:2.4.1.-] |  |
| bmc:BAbS19\_I14190 | greA; transcription elongation factor GreA; K03624 transcription elongation factor GreA |  |
| bmc:BAbS19\_I14200 | hypothetical protein |  |
| bmc:BAbS19\_I14210 | hypothetical protein |  |
| bmc:BAbS19\_I14220 | hypothetical protein |  |
| bmc:BAbS19\_I14230 | hypothetical protein; K09922 hypothetical protein |  |
| bmc:BAbS19\_I14240 | hypothetical protein |  |
| bmc:BAbS19\_I14250 | thioesterase superfamily protein; K10806 acyl-CoA thioesterase YciA [EC:3.1.2.-] |  |
| bmc:BAbS19\_I14260 | SH3 domain-containing protein |  |
| bmc:BAbS19\_I14270 | excinuclease ABC subunit B; K03702 excinuclease ABC subunit B |  |

  
**Neighborhood Representations for "bmf:BAB1\_1521"**  

| ID | Annotation | EC number |
| --- | --- | --- |
| bmf:BAB1\_1511 | NUDIX hydrolase |  |
| bmf:BAB1\_1512 | cold shock DNA-binding domain-containing protein; K03704 cold shock protein (beta-ribbon, CspA family) |  |
| bmf:BAB1\_1513 | glutathione S-transferase; K00799 glutathione S-transferase [EC:2.5.1.18] | ec:2.5.1.18 |
| bmf:BAB1\_1514 | aspC; aspartate aminotransferase (EC:2.6.1.1); K00812 aspartate aminotransferase [EC:2.6.1.1] | ec:2.6.1.1 |
| bmf:BAB1\_1515 | hypothetical protein |  |
| bmf:BAB1\_1516 | hypothetical protein |  |
| bmf:BAB1\_1517 | transcriptional regulator LysR |  |
| bmf:BAB1\_1518 | trxB; pyridine nucleotide-disulfide oxidoreductase (EC:1.8.1.9); K00384 thioredoxin reductase (NADPH) [EC:1.8.1.9] | ec:1.8.1.9 |
| bmf:BAB1\_1519 | cation transporter; K03498 trk system potassium uptake protein TrkH |  |
| bmf:BAB1\_1520 | hypothetical protein |  |
| bmf:BAB1\_1521 | lrp-1; AsnC family regulatory protein |  |
| bmf:BAB1\_1522 | lpcC; group 1 glycosyl transferase (EC:2.-.-.-); K12989 mannosyltransferase [EC:2.4.1.-] |  |
| bmf:BAB1\_1523 | greA; transcription elongation factor GreA; K03624 transcription elongation factor GreA |  |
| bmf:BAB1\_1524 | hypothetical protein |  |
| bmf:BAB1\_1525 | hypothetical protein |  |
| bmf:BAB1\_1526 | hypothetical protein; K09922 hypothetical protein |  |
| bmf:BAB1\_1527 | hypothetical protein |  |
| bmf:BAB1\_1528 | thioesterase superfamily protein; K10806 acyl-CoA thioesterase YciA [EC:3.1.2.-] |  |
| bmf:BAB1\_1529 | hypothetical protein |  |
| bmf:BAB1\_1530 | uvrB; excinuclease ABC subunit B; K03702 excinuclease ABC subunit B |  |
| bmf:BAB1\_1531 | SLT domain-containing protein (EC:3.2.1.-); K01238 [EC:3.2.1.-] |  |

  
**Over-represented Enzyme Summary**: Table of E.C. identified protein in the "Neighborhood Representation" ranked by frequency of occurrence  

| EC number | Frequency | Annotation | Reactions |
| --- | --- | --- | --- |
| ec:3.7.1.3 | 53 | kynureninase | L-kynurenine + H2O = anthranilate + L-alanine [RN:R00987] |
| ec:3.5.1.9 | 41 | arylformamidase; kynurenine formamidase; formylase; formylkynureninase; formylkynurenine formamidase; formamidase I; formamidase II | N-formyl-L-kynurenine + H2O = formate + L-kynurenine [RN:R01959] |
| ec:1.13.11.11 | 37 | tryptophan 2,3-dioxygenase; tryptophan pyrrolase (ambiguous); tryptophanase; tryptophan oxygenase; tryptamine 2,3-dioxygenase; tryptophan peroxidase; indoleamine 2,3-dioxygenase (ambiguous); indolamine 2,3-dioxygenase (ambiguous); L-tryptophan pyrrolase; TDO; L-tryptophan 2,3-dioxygenase | L-tryptophan + O2 = N-formyl-L-kynurenine [RN:R00678] |
| ec:1.8.4.11 | 35 | peptide-methionine (S)-S-oxide reductase; MsrA; methionine sulfoxide reductase (ambiguous); methionine sulphoxide reductase A; methionine S-oxide reductase (ambiguous); methionine S-oxide reductase (S-form oxidizing); methionine sulfoxide reductase A; peptide methionine sulfoxide reductase | (1) peptide-L-methionine + thioredoxin disulfide + H2O = peptide-L-methionine (S)-S-oxide + thioredoxin [RN:R04120]; (2) L-methionine + thioredoxin disulfide + H2O = L-methionine (S)-S-oxide + thioredoxin [RN:R07606] |
| ec:2.1.1.79 | 35 | cyclopropane-fatty-acyl-phospholipid synthase; cyclopropane synthetase; unsaturated-phospholipid methyltransferase; cyclopropane synthase; cyclopropane fatty acid synthase; cyclopropane fatty acid synthetase; CFA synthase | S-adenosyl-L-methionine + phospholipid olefinic fatty acid = S-adenosyl-L-homocysteine + phospholipid cyclopropane fatty acid [RN:R03411] |
| ec:1.4.3.5 | 35 | pyridoxal 5'-phosphate synthase; pyridoxamine 5'-phosphate oxidase; pyridoxamine phosphate oxidase; pyridoxine (pyridoxamine)phosphate oxidase; pyridoxine (pyridoxamine) 5'-phosphate oxidase; pyridoxaminephosphate oxidase (EC 1.4.3.5: deaminating); PMP oxidase; pyridoxol-5'-phosphate:oxygen oxidoreductase (deaminating) (incorrect); pyridoxamine-phosphate oxidase; PdxH | (1) pyridoxamine 5'-phosphate + H2O + O2 = pyridoxal 5'-phosphate + NH3 + H2O2 [RN:R00277]; (2) pyridoxine 5'-phosphate + O2 = pyridoxal 5'-phosphate + H2O2 [RN:R00278] |
| ec:1.1.1.11 | 22 | D-arabinitol 4-dehydrogenase; D-arabitol dehydrogenase; arabitol dehydrogenase | D-arabinitol + NAD+ = D-xylulose + NADH + H+ [RN:R05604] |
| ec:2.7.1.17 | 19 | xylulokinase; xylulokinase (phosphorylating); D-xylulokinase | ATP + D-xylulose = ADP + D-xylulose 5-phosphate [RN:R01639] |
| ec:1.2.1.28 | 19 | benzaldehyde dehydrogenase (NAD+); benzaldehyde (NAD+) dehydrogenase; benzaldehyde dehydrogenase (NAD+) | benzaldehyde + NAD+ + H2O = benzoate + NADH + 2 H+ [RN:R01419] |
| ec:1.1.1.169 | 19 | 2-dehydropantoate 2-reductase; 2-oxopantoate reductase; 2-ketopantoate reductase; 2-ketopantoic acid reductase; ketopantoate reductase; ketopantoic acid reductase | (R)-pantoate + NADP+ = 2-dehydropantoate + NADPH + H+ [RN:R02472] |
| ec:4.1.1.7 | 18 | benzoylformate decarboxylase; phenylglyoxylate decarboxylase; benzoylformate carboxy-lyase; benzoylformate carboxy-lyase (benzaldehyde-forming) | phenylglyoxylate = benzaldehyde + CO2 [RN:R01764] |
| ec:1.8.1.9 | 14 | thioredoxin-disulfide reductase; NADP-thioredoxin reductase; NADPH-thioredoxin reductase; thioredoxin reductase (NADPH); NADPH2:oxidized thioredoxin oxidoreductase | thioredoxin + NADP+ = thioredoxin disulfide + NADPH + H+ [RN:R02016] |
| ec:2.5.1.18 | 10 | glutathione transferase; glutathione S-transferase; glutathione S-alkyltransferase; glutathione S-aryltransferase; S-(hydroxyalkyl)glutathione lyase; glutathione S-aralkyltransferase; glutathione S-alkyl transferase; GST | RX + glutathione = HX + R-S-glutathione [RN:R03522 R08511 R08512] |
| ec:2.6.1.1 | 10 | aspartate transaminase; glutamic-oxaloacetic transaminase; glutamic-aspartic transaminase; transaminase A; AAT; AspT; 2-oxoglutarate-glutamate aminotransferase; aspartate alpha-ketoglutarate transaminase; aspartate aminotransferase; aspartate-2-oxoglutarate transaminase; aspartic acid aminotransferase; aspartic aminotransferase; aspartyl aminotransferase; AST; glutamate-oxalacetate aminotransferase; glutamate-oxalate transaminase; glutamic-aspartic aminotransferase; glutamic-oxalacetic transaminase; glutamic oxalic transaminase; GOT (enzyme) [ambiguous]; L-aspartate transaminase; L-aspartate-alpha-ketoglutarate transaminase; L-aspartate-2-ketoglutarate aminotransferase; L-aspartate-2-oxoglutarate aminotransferase; L-aspartate-2-oxoglutarate-transaminase; L-aspartic aminotransferase; oxaloacetate-aspartate aminotransferase; oxaloacetate transferase; aspartate:2-oxoglutarate aminotransferase; glutamate oxaloacetate transaminase | L-aspartate + 2-oxoglutarate = oxaloacetate + L-glutamate [RN:R00355] |
| ec:3.5.1.28 | 9 | N-acetylmuramoyl-L-alanine amidase; acetylmuramyl-L-alanine amidase; N-acetylmuramyl-L-alanine amidase; N-acylmuramyl-L-alanine amidase; acetylmuramoyl-alanine amidase; N-acetylmuramic acid L-alanine amidase; acetylmuramyl-alanine amidase; N-acetylmuramylalanine amidase; murein hydrolase; N-acetylmuramoyl-L-alanine amidase type I; N-acetylmuramoyl-L-alanine amidase type II | Hydrolyses the link between N-acetylmuramoyl residues and L-amino acid residues in certain cell-wall glycopeptides |
| ec:1.3.8.6 | 9 | glutaryl-CoA dehydrogenase (ETF); glutaryl coenzyme A dehydrogenase; glutaryl-CoA:(acceptor) 2,3-oxidoreductase (decarboxylating); glutaryl-CoA dehydrogenase | glutaryl-CoA + electron-transfer flavoprotein = crotonyl-CoA + CO2 + reduced electron-transfer flavoprotein (overall reaction) [RN:R02488]; (1a) glutaryl-CoA + electron-transfer flavoprotein = (E)-glutaconyl-CoA + reduced electron-transfer flavoprotein [RN:R10074]; (1b) (E)-glutaconyl-CoA = crotonyl-CoA + CO2 [RN:R03028] |
| ec:6.3.5.5 | 8 | carbamoyl-phosphate synthase (glutamine-hydrolysing); carbamoyl-phosphate synthetase (glutamine-hydrolysing); carbamyl phosphate synthetase (glutamine); carbamoylphosphate synthetase II; glutamine-dependent carbamyl phosphate synthetase; carbamoyl phosphate synthetase; CPS; carbon-dioxide:L-glutamine amido-ligase (ADP-forming, carbamate-phosphorylating) | 2 ATP + L-glutamine + HCO3- + H2O = 2 ADP + phosphate + L-glutamate + carbamoyl phosphate (overall reaction) [RN:R00575]; (1a) L-glutamine + H2O = L-glutamate + NH3 [RN:R00256]; (1b) 2 ATP + HCO3- + NH3 = 2 ADP + phosphate + carbamoyl phosphate [RN:R07641] |
| ec:1.1.5.2 | 6 | glucose 1-dehydrogenase (PQQ, quinone); quinoprotein glucose dehydrogenase; membrane-bound glucose dehydrogenase; mGDH; glucose dehydrogenase (PQQ-dependent); glucose dehydrogenase (pyrroloquinoline-quinone); quinoprotein D-glucose dehydrogenase | D-glucose + ubiquinone = D-glucono-1,5-lactone + ubiquinol [RN:R06620] |
| ec:1.1.1.14 | 5 | L-iditol 2-dehydrogenase; polyol dehydrogenase; sorbitol dehydrogenase; L-iditol:NAD+ 5-oxidoreductase; L-iditol (sorbitol) dehydrogenase; glucitol dehydrogenase; L-iditol:NAD+ oxidoreductase; NAD+-dependent sorbitol dehydrogenase; NAD+-sorbitol dehydrogenase | L-iditol + NAD+ = L-sorbose + NADH + H+ [RN:R07145] |
| ec:2.3.1.129 | 5 | acyl-[acyl-carrier-protein]---UDP-N-acetylglucosamine O-acyltransferase; UDP-N-acetylglucosamine acyltransferase; uridine diphosphoacetylglucosamine acyltransferase; acyl-[acyl-carrier-protein]-UDP-N-acetylglucosamine O-acyltransferase; (R)-3-hydroxytetradecanoyl-[acyl-carrier-protein]:UDP-N-acetylglucosamine 3-O-(3-hydroxytetradecanoyl)transferase | (R)-3-hydroxytetradecanoyl-[acyl-carrier protein] + UDP-N-acetyl-alpha-D-glucosamine = an [acyl-carrier protein] + UDP-3-O-[(3R)-3-hydroxytetradecanoyl]-N-acetyl-alpha-D-glucosamine [RN:R04567] |
| ec:3.5.1.88 | 4 | peptide deformylase | formyl-L-methionyl peptide + H2O = formate + methionyl peptide [RN:R05635] |
| ec:3.1.3.18 | 4 | phosphoglycolate phosphatase; phosphoglycolate hydrolase; 2-phosphoglycolate phosphatase; P-glycolate phosphatase; phosphoglycollate phosphatase | 2-phosphoglycolate + H2O = glycolate + phosphate [RN:R01334] |
| ec:1.14.12.1 | 4 | anthranilate 1,2-dioxygenase (deaminating, decarboxylating); anthranilate hydroxylase; anthranilic hydroxylase; anthranilic acid hydroxylase | anthranilate + NAD(P)H + 2 H+ + O2 = catechol + CO2 + NAD(P)+ + NH3 [RN:R00823 R00825] |
| ec:2.7.1.72 | 4 | streptomycin 6-kinase; streptidine kinase; SM 6-kinase; streptomycin 6-kinase (phosphorylating); streptidine kinase (phosphorylating); streptomycin 6-O-phosphotransferase; streptomycin 6-phosphotransferase | ATP + streptomycin = ADP + streptomycin 6-phosphate [RN:R02225] |
| ec:4.1.3.4 | 4 | hydroxymethylglutaryl-CoA lyase; hydroxymethylglutaryl coenzyme A-cleaving enzyme; hydroxymethylglutaryl coenzyme A lyase; 3-hydroxy-3-methylglutaryl coenzyme A lyase; 3-hydroxy-3-methylglutaryl CoA cleaving enzyme; 3-hydroxy-3-methylglutaryl-CoA lyase; (S)-3-hydroxy-3-methylglutaryl-CoA acetoacetate-lyase | (S)-3-hydroxy-3-methylglutaryl-CoA = acetyl-CoA + acetoacetate [RN:R01360] |
| ec:2.1.2.9 | 4 | methionyl-tRNA formyltransferase; N10-formyltetrahydrofolic-methionyl-transfer ribonucleic transformylase; formylmethionyl-transfer ribonucleic synthetase; methionyl ribonucleic formyltransferase; methionyl-tRNA Met formyltransferase; methionyl-tRNA transformylase; methionyl-transfer RNA transformylase; methionyl-transfer ribonucleate methyltransferase; methionyl-transfer ribonucleic transformylase | 10-formyltetrahydrofolate + L-methionyl-tRNAfMet = tetrahydrofolate + N-formylmethionyl-tRNAfMet [RN:R03940] |
| ec:5.3.2.6 | 3 | 2-hydroxymuconate tautomerase; 4-oxalocrotonate tautomerase (misleading); 4-oxalocrotonate isomerase (misleading); cnbG (gene name); praC (gene name); xylH (gene name) | (2Z,4E)-2-hydroxyhexa-2,4-dienedioate = (3E)-2-oxohex-3-enedioate |
| ec:3.2.1.52 | 3 | beta-N-acetylhexosaminidase; hexosaminidase; beta-acetylaminodeoxyhexosidase; N-acetyl-beta-D-hexosaminidase; N-acetyl-beta-hexosaminidase; beta-hexosaminidase; beta-acetylhexosaminidinase; beta-D-N-acetylhexosaminidase; beta-N-acetyl-D-hexosaminidase; beta-N-acetylglucosaminidase; hexosaminidase A; N-acetylhexosaminidase; beta-D-hexosaminidase | Hydrolysis of terminal non-reducing N-acetyl-D-hexosamine residues in N-acetyl-beta-D-hexosaminides |
| ec:6.3.5.4 | 3 | asparagine synthase (glutamine-hydrolysing); asparagine synthetase (glutamine-hydrolysing); glutamine-dependent asparagine synthetase; asparagine synthetase B; AS; AS-B | ATP + L-aspartate + L-glutamine + H2O = AMP + diphosphate + L-asparagine + L-glutamate (overall reaction) [RN:R00578]; (1a) L-glutamine + H2O = L-glutamate + NH3 [RN:R00256]; (1b) ATP + L-aspartate + NH3 = AMP + diphosphate + L-asparagine [RN:R00483] |
| ec:3.6.3.17 | 3 | monosaccharide-transporting ATPase | ATP + H2O + monosaccharideout = ADP + phosphate + monosaccharidein [RN:R00086] |
| ec:2.7.13.3 | 3 | histidine kinase; EnvZ; histidine kinase (ambiguous); histidine protein kinase (ambiguous); protein histidine kinase (ambiguous); protein kinase (histidine) (ambiguous); HK1; HP165; Sln1p | ATP + protein L-histidine = ADP + protein N-phospho-L-histidine |
| ec:3.8.1.3 | 3 | haloacetate dehalogenase; monohaloacetate dehalogenase | haloacetate + H2O = glycolate + halide [RN:R02336] |
| ec:2.7.7.7 | 2 | DNA-directed DNA polymerase; DNA polymerase I; DNA polymerase II; DNA polymerase III; DNA polymerase alpha; DNA polymerase beta; DNA polymerase gamma; DNA nucleotidyltransferase (DNA-directed); DNA nucleotidyltransferase (DNA-directed); deoxyribonucleate nucleotidyltransferase; deoxynucleate polymerase; deoxyribonucleic acid duplicase; deoxyribonucleic acid polymerase; deoxyribonucleic duplicase; deoxyribonucleic polymerase; deoxyribonucleic polymerase I; DNA duplicase; DNA nucleotidyltransferase; DNA polymerase; DNA replicase; DNA-dependent DNA polymerase; duplicase; Klenow fragment; sequenase; Taq DNA polymerase; Taq Pol I; Tca DNA polymerase | deoxynucleoside triphosphate + DNAn = diphosphate + DNAn+1 [RN:R00379] |
| ec:2.7.2.3 | 1 | phosphoglycerate kinase; PGK; 3-PGK; ATP-3-phospho-D-glycerate-1-phosphotransferase; ATP:D-3-phosphoglycerate 1-phosphotransferase; 3-phosphoglycerate kinase; 3-phosphoglycerate phosphokinase; 3-phosphoglyceric acid kinase; 3-phosphoglyceric acid phosphokinase; 3-phosphoglyceric kinase; glycerate 3-phosphate kinase; glycerophosphate kinase; phosphoglyceric acid kinase; phosphoglyceric kinase; phosphoglycerokinase | ATP + 3-phospho-D-glycerate = ADP + 3-phospho-D-glyceroyl phosphate [RN:R01512] |
| ec:5.3.3.4 | 1 | muconolactone Delta-isomerase; muconolactone isomerase | (S)-5-oxo-2,5-dihydrofuran-2-acetate = 5-oxo-4,5-dihydrofuran-2-acetate [RN:R06990] |
| ec:3.6.1.11 | 1 | exopolyphosphatase; metaphosphatase; acid phosphoanhydride phosphohydrolase; Gra-Pase | (polyphosphate)n + H2O = (polyphosphate)n-1 + phosphate [RN:R03042] |
| ec:1.5.1.20 | 1 | methylenetetrahydrofolate reductase [NAD(P)H]; methylenetetrahydrofolate (reduced nicotinamide adenine dinucleotide phosphate) reductase; 5,10-methylenetetrahydrofolate reductase (NADPH); 5,10-methylenetetrahydrofolic acid reductase; 5,10-CH2-H4folate reductase; methylenetetrahydrofolate reductase (NADPH2); 5-methyltetrahydrofolate:NAD+ oxidoreductase; 5-methyltetrahydrofolate:NAD+ oxidoreductase; methylenetetrahydrofolate (reduced riboflavin adenine dinucleotide) reductase; 5,10-methylenetetrahydrofolate reductase; methylenetetrahydrofolate reductase; N5,10-methylenetetrahydrofolate reductase; 5,10-methylenetetrahydropteroylglutamate reductase; N5,N10-methylenetetrahydrofolate reductase; methylenetetrahydrofolic acid reductase; 5-methyltetrahydrofolate:(acceptor) oxidoreductase (incorrect); 5,10-methylenetetrahydrofolate reductase (FADH2); MetF; methylenetetrahydrofolate reductase (NADPH); 5-methyltetrahydrofolate:NADP+ oxidoreductase | 5-methyltetrahydrofolate + NAD(P)+ = 5,10-methylenetetrahydrofolate + NAD(P)H + H+ [RN:R01224 R07168] |
| ec:3.6.1.40 | 1 | guanosine-5'-triphosphate,3'-diphosphate phosphatase; pppGpp 5'-phosphohydrolase; guanosine 5'-triphosphate-3'-diphosphate 5'-phosphohydrolase; guanosine pentaphosphatase; guanosine pentaphosphate phosphatase; guanosine 5'-triphosphate 3'-diphosphate 5'-phosphatase; guanosine pentaphosphate phosphohydrolase | guanosine 5'-triphosphate 3'-diphosphate + H2O = guanosine 3',5'-bis(diphosphate) + phosphate [RN:R03409] |
| ec:1.2.5.1 | 1 | pyruvate dehydrogenase (quinone); pyruvate dehydrogenase; pyruvic dehydrogenase; pyruvic (cytochrome b1) dehydrogenase; pyruvate:ubiquinone-8-oxidoreductase; pyruvate oxidase (ambiguous); pyruvate dehydrogenase (cytochrome) (incorrect) | pyruvate + ubiquinone + H2O = acetate + CO2 + ubiquinol |
| ec:5.99.1.3 | 1 | DNA topoisomerase (ATP-hydrolysing); type II DNA topoisomerase; DNA-gyrase; deoxyribonucleate topoisomerase; deoxyribonucleic topoisomerase; topoisomerase; DNA topoisomerase II | ATP-dependent breakage, passage and rejoining of double-stranded DNA |
| ec:2.3.1.191 | 1 | UDP-3-O-(3-hydroxymyristoyl)glucosamine N-acyltransferase; UDP-3-O-acyl-glucosamine N-acyltransferase; UDP-3-O-(R-3-hydroxymyristoyl)-glucosamine N-acyltransferase; acyltransferase LpxD; acyl-ACP:UDP-3-O-(3-hydroxyacyl)-GlcN N-acyltransferase; firA (gene name); lpxD (gene name) | (3R)-3-hydroxymyristoyl-[acyl-carrier protein] + UDP-3-O-[(3R)-3-hydroxymyristoyl]-alpha-D-glucosamine = UDP-2,3-bis[O-(3R)-3-hydroxymyristoyl]-alpha-D-glucosamine + a holo-[acyl-carrier protein] [RN:R04550] |
| ec:2.3.2.2 | 1 | gamma-glutamyltransferase; glutamyl transpeptidase; alpha-glutamyl transpeptidase; gamma-glutamyl peptidyltransferase; gamma-glutamyl transpeptidase (ambiguous); gamma-GPT; gamma-GT; gamma-GTP; L-gamma-glutamyl transpeptidase; L-gamma-glutamyltransferase; L-glutamyltransferase; GGT (ambiguous); gamma-glutamyltranspeptidase (ambiguous) | a (5-L-glutamyl)-peptide + an amino acid = a peptide + a 5-L-glutamyl amino acid [RN:R04159] |
| ec:3.6.3.34 | 1 | iron-chelate-transporting ATPase | ATP + H2O + iron chelateout = ADP + phosphate + iron chelatein [RN:R00086] |
| ec:4.1.99.12 | 1 | 3,4-dihydroxy-2-butanone-4-phosphate synthase; DHBP synthase; L-3,4-dihydroxybutan-2-one-4-phosphate synthase | D-ribulose 5-phosphate = formate + L-3,4-dihydroxybutan-2-one 4-phosphate [RN:R07281] |
| ec:3.1.3.11 | 1 | fructose-bisphosphatase; hexose diphosphatase; FBPase; fructose 1,6-diphosphatase; fructose 1,6-diphosphate phosphatase; D-fructose 1,6-diphosphatase; fructose 1,6-bisphosphatase; fructose diphosphatase; fructose diphosphate phosphatase; fructose bisphosphate phosphatase; fructose 1,6-bisphosphate 1-phosphatase; fructose 1,6-bisphosphate phosphatase; hexose bisphosphatase; D-fructose-1,6-bisphosphate phosphatase | D-fructose 1,6-bisphosphate + H2O = D-fructose 6-phosphate + phosphate [RN:R00762] |
| ec:2.1.1.13 | 1 | methionine synthase; 5-methyltetrahydrofolate---homocysteine S-methyltransferase; 5-methyltetrahydrofolate---homocysteine transmethylase; N-methyltetrahydrofolate:L-homocysteine methyltransferase; N5-methyltetrahydrofolate methyltransferase; N5-methyltetrahydrofolate-homocysteine cobalamin methyltransferase; N5-methyltetrahydrofolic---homocysteine vitamin B12 transmethylase; B12 N5-methyltetrahydrofolate homocysteine methyltransferase; methyltetrahydrofolate---homocysteine vitamin B12 methyltransferase; tetrahydrofolate methyltransferase; tetrahydropteroylglutamate methyltransferase; tetrahydropteroylglutamic methyltransferase; vitamin B12 methyltransferase; cobalamin-dependent methionine synthase; methionine synthase (cobalamin-dependent); MetH | 5-methyltetrahydrofolate + L-homocysteine = tetrahydrofolate + L-methionine [RN:R00946] |
| ec:5.4.99.22 | 1 | 23S rRNA pseudouridine2605 synthase; RluB; YciL | 23S rRNA uridine2605 = 23S rRNA pseudouridine2605 |
| ec:3.5.4.26 | 1 | diaminohydroxyphosphoribosylaminopyrimidine deaminase | 2,5-diamino-6-hydroxy-4-(5-phospho-D-ribosylamino)pyrimidine + H2O = 5-amino-6-(5-phospho-D-ribosylamino)uracil + NH3 [RN:R03459] |
| ec:3.5.4.25 | 1 | GTP cyclohydrolase II; guanosine triphosphate cyclohydrolase II; GTP-8-formylhydrolase | GTP + 3 H2O = formate + 2,5-diamino-6-hydroxy-4-(5-phospho-D-ribosylamino)pyrimidine + diphosphate [RN:R00425] |
| ec:2.5.1.78 | 1 | 6,7-dimethyl-8-ribityllumazine synthase; lumazine synthase; 6,7-dimethyl-8-ribityllumazine synthase 2; 6,7-dimethyl-8-ribityllumazine synthase 1; lumazine synthase 2; lumazine synthase 1; type I lumazine synthase; type II lumazine synthase; RIB4; MJ0303; RibH; Pbls; MbtLS; RibH1 protein; RibH2 protein; RibH1; RibH2 | 1-deoxy-L-glycero-tetrulose 4-phosphate + 5-amino-6-(D-ribitylamino)uracil = 6,7-dimethyl-8-(D-ribityl)lumazine + 2 H2O + phosphate [RN:R04457] |
| ec:2.3.1.183 | 1 | phosphinothricin acetyltransferase; PAT; PPT acetyltransferase; Pt-N-acetyltransferase; ac-Pt | acetyl-CoA + phosphinothricin = CoA + N-acetylphosphinothricin [RN:R08938] |
| ec:3.6.3.27 | 1 | phosphate-transporting ATPase; ABC phosphate transporter | ATP + H2O + phosphateout = ADP + phosphate + phosphatein [RN:R00086] |
| ec:5.4.2.10 | 1 | phosphoglucosamine mutase | alpha-D-glucosamine 1-phosphate = D-glucosamine 6-phosphate [RN:R02060] |
| ec:2.5.1.9 | 1 | riboflavin synthase; heavy riboflavin synthase; light riboflavin synthase; riboflavin synthetase; riboflavine synthase; riboflavine synthetase | 2 6,7-dimethyl-8-(1-D-ribityl)lumazine = riboflavin + 4-(1-D-ribitylamino)-5-amino-2,6-dihydroxypyrimidine [RN:R00066] |
| ec:4.2.1.59 | 1 | 3-hydroxyacyl-[acyl-carrier-protein] dehydratase; fabZ (gene name); fabA (gene name); D-3-hydroxyoctanoyl-[acyl carrier protein] dehydratase; D-3-hydroxyoctanoyl-acyl carrier protein dehydratase; beta-hydroxyoctanoyl-acyl carrier protein dehydrase; beta-hydroxyoctanoyl thioester dehydratase; beta-hydroxyoctanoyl-ACP-dehydrase; (3R)-3-hydroxyoctanoyl-[acyl-carrier-protein] hydro-lyase; (3R)-3-hydroxyoctanoyl-[acyl-carrier-protein] hydro-lyase (oct-2-enoyl-[acyl-carrier protein]-forming); 3-hydroxyoctanoyl-[acyl-carrier-protein] dehydratase | a (3R)-3-hydroxyacyl-[acyl-carrier protein] = a trans-2-enoyl-[acyl-carrier protein] + H2O [RN:R10208] |
| ec:1.1.1.193 | 1 | 5-amino-6-(5-phosphoribosylamino)uracil reductase; aminodioxyphosphoribosylaminopyrimidine reductase | 5-amino-6-(5-phospho-D-ribitylamino)uracil + NADP+ = 5-amino-6-(5-phospho-D-ribosylamino)uracil + NADPH + H+ [RN:R03458] |
| ec:2.1.2.1 | 1 | glycine hydroxymethyltransferase; serine aldolase; threonine aldolase; serine hydroxymethylase; serine hydroxymethyltransferase; allothreonine aldolase; L-serine hydroxymethyltransferase; L-threonine aldolase; serine hydroxymethyltransferase; serine transhydroxymethylase | 5,10-methylenetetrahydrofolate + glycine + H2O = tetrahydrofolate + L-serine [RN:R00945] |
| ec:5.5.1.1 | 1 | muconate cycloisomerase; muconate cycloisomerase I; cis,cis-muconate-lactonizing enzyme; cis,cis-muconate cycloisomerase; muconate lactonizing enzyme; 4-carboxymethyl-4-hydroxyisocrotonolactone lyase (decyclizing); CatB; MCI | 2,5-dihydro-5-oxofuran-2-acetate = cis,cis-hexadienedioate [RN:R03959] |
| ec:1.13.11.1 | 1 | catechol 1,2-dioxygenase; catechol-oxygen 1,2-oxidoreductase; 1,2-pyrocatechase; catechase; catechol 1,2-oxygenase; catechol dioxygenase; pyrocatechase; pyrocatechol 1,2-dioxygenase; CD I; CD II | catechol + O2 = cis,cis-muconate [RN:R00817] |
| ec:4.1.2.13 | 1 | fructose-bisphosphate aldolase; aldolase; fructose-1,6-bisphosphate triosephosphate-lyase; fructose diphosphate aldolase; diphosphofructose aldolase; fructose 1,6-diphosphate aldolase; ketose 1-phosphate aldolase; phosphofructoaldolase; zymohexase; fructoaldolase; fructose 1-phosphate aldolase; fructose 1-monophosphate aldolase; 1,6-Diphosphofructose aldolase; SMALDO; D-fructose-1,6-bisphosphate D-glyceraldehyde-3-phosphate-lyase | D-fructose 1,6-bisphosphate = glycerone phosphate + D-glyceraldehyde 3-phosphate [RN:R01068] |

  
**Over-represented Metabolite Summary**: Collection of the metabolites identified as substrates or products of the proteins representaed the "Over-represented Enzyme Summary" ranked by frequency of occurrence  

| ID | Structure | Name | Frequency | EC |
| --- | --- | --- | --- | --- |
| cpd:C00001 |  | H2O; Water | 226 | ec:3.6.1.11 ec:3.1.3.11 ec:3.5.1.28 ec:3.7.1.3 ec:2.3.2.2 ec:1.2.1.28 ec:6.3.5.5 ec:6.3.5.4 ec:4.2.1.59 ec:1.1.1.193 ec:2.1.2.1 ec:2.5.1.78 ec:3.6.1.40 ec:1.8.1.9 ec:3.5.1.9 ec:3.2.1.52 ec:3.5.4.25 ec:2.5.1.18 ec:3.5.4.26 ec:1.4.3.5 ec:3.8.1.3 ec:4.1.99.12 ec:3.1.3.18 ec:1.2.5.1 |
| cpd:C02700 |  | L-Formylkynurenine; N-Formyl-L-kynurenine; N-Formylkynurenine | 131 | ec:1.13.11.11 ec:3.7.1.3 ec:3.5.1.9 |
| cpd:C00080 |  | H+; Hydron | 111 | ec:1.1.1.14 ec:1.2.1.28 ec:1.1.1.11 ec:2.5.1.18 ec:1.5.1.20 ec:3.5.4.26 ec:1.1.1.193 ec:1.14.12.1 ec:1.1.1.169 ec:1.8.1.9 |
| cpd:C00108 |  | Anthranilate; Anthranilic acid; o-Aminobenzoic acid; Vitamin L1; 2-Aminobenzoate | 100 | ec:3.7.1.3 ec:1.14.12.1 ec:3.5.1.9 |
| cpd:C00328 |  | L-Kynurenine; 3-Anthraniloyl-L-alanine | 94 | ec:3.7.1.3 ec:3.5.1.9 |
| cpd:C05653 |  | Formylanthranilate; N-Formylanthranilate; 2-(Formylamino)-benzoic acid | 94 | ec:3.7.1.3 ec:3.5.1.9 |
| cpd:C00007 |  | Oxygen; O2 | 89 | ec:1.13.11.11 ec:1.13.11.1 ec:1.4.3.5 ec:1.14.12.1 |
| cpd:C00006 |  | NADP+; NADP; Nicotinamide adenine dinucleotide phosphate; beta-Nicotinamide adenine dinucleotide phosphate; TPN; Triphosphopyridine nucleotide | 74 | ec:1.2.1.28 ec:1.5.1.20 ec:3.5.4.26 ec:1.1.1.193 ec:1.14.12.1 ec:1.1.1.169 ec:1.8.1.9 |
| cpd:C00005 |  | NADPH; TPNH; Reduced nicotinamide adenine dinucleotide phosphate | 74 | ec:1.2.1.28 ec:1.5.1.20 ec:3.5.4.26 ec:1.1.1.193 ec:1.14.12.1 ec:1.1.1.169 ec:1.8.1.9 |
| cpd:C00004 |  | NADH; DPNH; Reduced nicotinamide adenine dinucleotide | 67 | ec:1.1.1.14 ec:1.2.1.28 ec:1.1.1.11 ec:1.5.1.20 ec:1.14.12.1 |
| cpd:C00003 |  | NAD+; NAD; Nicotinamide adenine dinucleotide; DPN; Diphosphopyridine nucleotide; Nadide | 67 | ec:1.1.1.14 ec:1.2.1.28 ec:1.1.1.11 ec:1.5.1.20 ec:1.14.12.1 |
| cpd:C00041 |  | L-Alanine; L-2-Aminopropionic acid; L-alpha-Alanine | 62 | ec:3.5.1.28 ec:3.7.1.3 |
| cpd:C00632 |  | 3-Hydroxyanthranilate; 3-Hydroxyanthranilic acid | 53 | ec:3.7.1.3 |
| cpd:C03227 |  | 3-Hydroxy-L-kynurenine | 53 | ec:3.7.1.3 |
| cpd:C00027 |  | Hydrogen peroxide; H2O2; Oxydol | 45 | ec:1.4.3.5 |
| cpd:C00058 |  | Formate; Methanoic acid; Formic acid | 42 | ec:3.5.4.25 ec:4.1.99.12 ec:3.5.1.9 |
| cpd:C00014 |  | Ammonia; NH3 | 42 | ec:3.5.4.26 ec:1.4.3.5 ec:1.1.1.193 ec:1.14.12.1 |
| cpd:C05651 |  | 5-Hydroxykynurenine; 5-Hydroxy-L-kynurenine | 41 | ec:3.5.1.9 |
| cpd:C05648 |  | 5-Hydroxy-N-formylkynurenine; 5-Hydroxy-N-formyl-L-kynurenine | 41 | ec:3.5.1.9 |
| cpd:C00310 |  | D-Xylulose; D-threo-Pentulose; D-Lyxulose | 41 | ec:1.1.1.11 ec:2.7.1.17 |
| cpd:C00261 |  | Benzaldehyde; Benzoic aldehyde | 37 | ec:1.2.1.28 ec:4.1.1.7 |
| cpd:C00633 |  | 4-Hydroxybenzaldehyde; p-Hydroxybenzaldehyde | 37 | ec:1.2.1.28 ec:4.1.1.7 |
| cpd:C00078 |  | L-Tryptophan; Tryptophan; (S)-alpha-Amino-beta-(3-indolyl)-propionic acid | 37 | ec:1.13.11.11 |
| cpd:C00002 |  | ATP; Adenosine 5'-triphosphate | 36 | ec:2.7.1.72 ec:6.3.5.5 ec:6.3.5.4 ec:2.7.1.17 ec:2.7.2.3 |
| cpd:C00314 |  | Pyridoxine; Pyridoxol | 35 | ec:1.4.3.5 |
| cpd:C00250 |  | Pyridoxal | 35 | ec:1.4.3.5 |
| cpd:C00647 |  | Pyridoxamine phosphate; Pyridoxamine 5-phosphate; Pyridoxamine 5'-phosphate | 35 | ec:1.4.3.5 |
| cpd:C00627 |  | Pyridoxine phosphate; Pyridoxine 5-phosphate; Pyridoxine 5'-phosphate; Pyridoxol 5'-phosphate | 35 | ec:1.4.3.5 |
| cpd:C00534 |  | Pyridoxamine; PM | 35 | ec:1.4.3.5 |
| cpd:C00018 |  | Pyridoxal phosphate; Pyridoxal 5-phosphate; Pyridoxal 5'-phosphate; PLP | 35 | ec:1.4.3.5 |
| cpd:C00011 |  | CO2; Carbon dioxide | 35 | ec:1.3.8.6 ec:5.5.1.1 ec:1.14.12.1 ec:1.2.5.1 ec:4.1.1.7 |
| cpd:C00008 |  | ADP; Adenosine 5'-diphosphate | 33 | ec:2.7.1.72 ec:6.3.5.5 ec:2.7.1.17 ec:2.7.2.3 |
| cpd:C00095 |  | D-Fructose; Levulose; Fruit sugar; D-arabino-Hexulose | 27 | ec:1.1.1.14 ec:1.1.1.11 |
| cpd:C00392 |  | Mannitol; D-Mannitol | 22 | ec:1.1.1.11 |
| cpd:C01904 |  | D-Arabitol; D-Arabinitol; D-Arabinol; D-Lyxitol | 22 | ec:1.1.1.11 |
| cpd:C00025 |  | L-Glutamate; L-Glutamic acid; L-Glutaminic acid; Glutamate | 22 | ec:2.3.2.2 ec:2.6.1.1 ec:6.3.5.5 ec:6.3.5.4 |
| cpd:C03067 |  | 3-Hydroxybenzaldehyde | 19 | ec:1.2.1.28 |
| cpd:C00231 |  | D-Xylulose 5-phosphate | 19 | ec:2.7.1.17 |
| cpd:C00587 |  | 3-Hydroxybenzoate; 3-Hydroxybenzoic acid; m-Hydroxybenzoic acid | 19 | ec:1.2.1.28 |
| cpd:C00180 |  | Benzoate; Benzoic acid; Benzenecarboxylic acid; Phenylformic acid; Dracylic acid | 19 | ec:1.2.1.28 |
| cpd:C00966 |  | 2-Dehydropantoate | 19 | ec:1.1.1.169 |
| cpd:C00156 |  | 4-Hydroxybenzoate; Hydroxybenzoic acid; 4-Hydroxybenzoic acid; Hydroxybenzenecarboxylic acid | 19 | ec:1.2.1.28 |
| cpd:C07215 |  | o-Toluate; o-Methylbenzoate; o-Toluic Acid; 2-Methylbenzoic acid | 19 | ec:1.2.1.28 |
| cpd:C07214 |  | 2-Methylbenzaldehyde; o-Toluic aldehyde; 2-Formyltoluene; o-Tolualdehyde | 19 | ec:1.2.1.28 |
| cpd:C07211 |  | m-Methylbenzoate; m-Toluic Acid; beta-Bethylbenzoic acid; m-Toluylic acid | 19 | ec:1.2.1.28 |
| cpd:C07209 |  | 3-Methylbenzaldehyde; m-Tolualdehyde | 19 | ec:1.2.1.28 |
| cpd:C00522 |  | (R)-Pantoate; Pantoate; Pantoic acid | 19 | ec:1.1.1.169 |
| cpd:C01454 |  | Toluate; p-Toluate; p-Toluic acid; 4-Methylbenzoic acid; Toluenecarboxylic acid; Crithminic acid | 19 | ec:1.2.1.28 |
| cpd:C06758 |  | p-Tolualdehyde; p-Methylbenzaldehyde; 4-Methylbenzaldehyde; 4-Toluylaldehyde; p-Formyltoluene | 19 | ec:1.2.1.28 |
| cpd:C03590 |  | 4-Hydroxyphenylglyoxylate; 4-Hydroxybenzoylformate; 4-Hydroxyphenylglyoxylic acid | 18 | ec:4.1.1.7 |
| cpd:C02137 |  | alpha-Oxo-benzeneacetic acid; Benzoylformate; Benzoylformic acid; Phenylglyoxylic acid; Phenylglyoxylate; 2-Oxo-2-phenylacetate | 18 | ec:4.1.1.7 |
| cpd:C00009 |  | Orthophosphate; Phosphate; Phosphoric acid; Orthophosphoric acid | 16 | ec:3.6.1.11 ec:3.1.3.11 ec:6.3.5.5 ec:3.1.3.18 ec:2.5.1.78 ec:3.6.1.40 |
| cpd:C00343 |  | Thioredoxin disulfide; Oxidized thioredoxin; Thioredoxin sulfide | 14 | ec:1.8.1.9 |
| cpd:C00342 |  | Thioredoxin; Reduced thioredoxin | 14 | ec:1.8.1.9 |
| cpd:C01327 |  | Hydrochloric acid; HCl; Hydrogen chloride; Hydrochloride | 14 | ec:2.5.1.18 ec:5.5.1.1 ec:3.8.1.3 |
| cpd:C01528 |  | Hydrogen selenide; Selenide | 14 | ec:1.8.1.9 |
| cpd:C18902 |  | Methylselenic acid; Methylseleninate | 14 | ec:1.8.1.9 |
| cpd:C05684 |  | Selenite | 14 | ec:1.8.1.9 |
| cpd:C05703 |  | Methaneselenol; Methylselenol | 14 | ec:1.8.1.9 |
| cpd:C00049 |  | L-Aspartate; L-Aspartic acid; 2-Aminosuccinic acid; L-Asp | 13 | ec:2.6.1.1 ec:6.3.5.4 |
| cpd:C02320 |  | R-S-Glutathione | 11 | ec:2.3.2.2 ec:2.5.1.18 |
| cpd:C00064 |  | L-Glutamine; L-2-Aminoglutaramic acid | 11 | ec:6.3.5.5 ec:6.3.5.4 |
| cpd:C00051 |  | Glutathione; 5-L-Glutamyl-L-cysteinylglycine; N-(N-gamma-L-Glutamyl-L-cysteinyl)glycine; gamma-L-Glutamyl-L-cysteinyl-glycine; GSH; Reduced glutathione | 11 | ec:2.3.2.2 ec:2.5.1.18 |
| cpd:C09880 |  | alpha-Pinene | 10 |  |
| cpd:C06677 |  | Toluene-4-sulfonate; Tosylate | 10 |  |
| cpd:C14098 |  | 2-Methylnaphthalene; beta-Methylnaphthalene | 10 |  |
| cpd:C01352 |  | FADH2 | 10 | ec:1.3.8.6 |
| cpd:C14089 |  | 1-Hydroxymethylnaphthalene; 1-Naphthalenemethanol | 10 |  |
| cpd:C00302 |  | DL-Glutamate; DL-Glutaminic acid; 2-Aminoglutaric acid; Glutamate; Glutamic acid | 10 | ec:2.6.1.1 |
| cpd:C14082 |  | 1-Methylnaphthalene; alpha-Methylnaphthalene | 10 |  |
| cpd:C02814 |  | Benzene-1,2,4-triol; Hydroxyhydroquinone; 1,2,4-Benzenetriol; 1,2,4-Trihydroxybenzene; Hydroxyquinol | 10 |  |
| cpd:C14874 |  | Glutathione episulfonium ion | 10 | ec:2.5.1.18 |
| cpd:C14871 |  | S-(Formylmethyl)glutathione | 10 | ec:2.5.1.18 |
| cpd:C14870 |  | 2-Bromoacetaldehyde | 10 | ec:2.5.1.18 |
| cpd:C05565 |  | Hydantoin-5-propionate; Hydantoin-propionate | 10 |  |
| cpd:C11278 |  | Aflatoxin B1exo-8,9-epoxide-GSH; 8,9-Dihydro-8-(S-glutathionyl)-9-hydroxyaflatoxin B1 | 10 | ec:2.5.1.18 |
| cpd:C14868 |  | S-(1,2-Dichlorovinyl)glutathione; DCVG | 10 | ec:2.5.1.18 |
| cpd:C14865 |  | 2-(S-Glutathionyl)acetyl chloride | 10 | ec:2.5.1.18 |
| cpd:C14864 |  | S-(2-Chloroacetyl)glutathione | 10 | ec:2.5.1.18 |
| cpd:C14863 |  | 2-(S-Glutathionyl)acetyl glutathione | 10 | ec:2.5.1.18 |
| cpd:C14861 |  | S-(2,2-Dichloro-1-hydroxy)ethyl glutathione | 10 | ec:2.5.1.18 |
| cpd:C01322 |  | RX; Organic halide | 10 | ec:2.5.1.18 |
| cpd:C14859 |  | Chloroacetyl chloride | 10 | ec:2.5.1.18 |
| cpd:C14858 |  | 2,2-Dichloroacetaldehyde | 10 | ec:2.5.1.18 |
| cpd:C14857 |  | 1,1-Dichloroethylene epoxide; 2,2-Dichlorooxirane | 10 | ec:2.5.1.18 |
| cpd:C14856 |  | 7,8-Dihydro-7-hydroxy-8-S-glutathionyl-benzo[a]pyrene | 10 | ec:2.5.1.18 |
| cpd:C02759 |  | alpha-Pinene-oxide | 10 |  |
| cpd:C14855 |  | 4,5-Dihydro-4-hydroxy-5-S-glutathionyl-benzo[a]pyrene | 10 | ec:2.5.1.18 |
| cpd:C14852 |  | Benzo[a]pyrene-7,8-diol; Benzo[a]pyrene-7,8-dihydrodiol | 10 | ec:2.5.1.18 |
| cpd:C14851 |  | Benzo[a]pyrene-4,5-oxide; Benzo[a]pyrene-4,5-epoxide | 10 | ec:2.5.1.18 |
| cpd:C05947 |  | L-erythro-4-Hydroxyglutamate | 10 | ec:2.6.1.1 |
| cpd:C05946 |  | D-4-Hydroxy-2-oxoglutarate | 10 | ec:2.6.1.1 |
| cpd:C14848 |  | 2,3-Dihydro-2-S-glutathionyl-3-hydroxy bromobenzene | 10 | ec:2.5.1.18 |
| cpd:C14847 |  | 3,4-Dihydro-3-hydroxy-4-S-glutathionyl bromobenzene | 10 | ec:2.5.1.18 |
| cpd:C14840 |  | Bromobenzene-2,3-oxide; Bromobenzene-2,3-epoxide | 10 | ec:2.5.1.18 |
| cpd:C14839 |  | Bromobenzene-3,4-oxide; Bromobenzene-3,4-epoxide | 10 | ec:2.5.1.18 |
| cpd:C07645 |  | Aldophosphamide | 10 | ec:2.5.1.18 |
| cpd:C14793 |  | (1R)-Glutathionyl-(2R)-hydroxy-1,2-dihydronaphthalene | 10 | ec:2.5.1.18 |
| cpd:C14792 |  | (1S)-Hydroxy-(2S)-glutathionyl-1,2-dihydronaphthalene | 10 | ec:2.5.1.18 |
| cpd:C14791 |  | (1R)-Hydroxy-(2R)-glutathionyl-1,2-dihydronaphthalene | 10 | ec:2.5.1.18 |
| cpd:C05528 |  | 3-Sulfopyruvate; 3-Sulfopyruvic acid | 10 | ec:2.6.1.1 |
| cpd:C05527 |  | 3-Sulfinylpyruvate; 3-Sulfinopyruvate | 10 | ec:2.6.1.1 |
| cpd:C14787 |  | (1S,2R)-Naphthalene 1,2-oxide; (1S,2R)-Naphthalene epoxide | 10 | ec:2.5.1.18 |
| cpd:C14786 |  | (1R,2S)-Naphthalene 1,2-oxide; (1R,2S)-Naphthalene epoxide | 10 | ec:2.5.1.18 |
| cpd:C00606 |  | 3-Sulfino-L-alanine; L-Cysteinesulfinic acid; 3-Sulphino-L-alanine; 3-Sulfinoalanine | 10 | ec:2.6.1.1 |
| cpd:C00166 |  | Phenylpyruvate; Phenylpyruvic acid; alpha-Ketohydrocinnamic acid; keto-Phenylpyruvate; 3-Phenyl-2-oxopropanoate; 2-Oxo-3-phenylpropanoate | 10 | ec:2.6.1.1 |
| cpd:C11583 |  | 4-Glutathionyl cyclophosphamide | 10 | ec:2.5.1.18 |
| cpd:C00957 |  | Mercaptopyruvate; 3-Mercaptopyruvic acid; 3-Mercaptopyruvate | 10 | ec:2.6.1.1 |
| cpd:C03351 |  | 3-Hydroxybenzyl alcohol; 3-Hydroxybenzenemethanol | 10 |  |
| cpd:C14806 |  | 1-Nitro-5-glutathionyl-6-hydroxy-5,6-dihydronaphthalene | 10 | ec:2.5.1.18 |
| cpd:C14805 |  | 1-Nitro-5-hydroxy-6-glutathionyl-5,6-dihydronaphthalene | 10 | ec:2.5.1.18 |
| cpd:C14804 |  | 1-Nitro-7-glutathionyl-8-hydroxy-7,8-dihydronaphthalene | 10 | ec:2.5.1.18 |
| cpd:C14803 |  | 1-Nitro-7-hydroxy-8-glutathionyl-7,8-dihydronaphthalene | 10 | ec:2.5.1.18 |
| cpd:C14802 |  | 1-Nitronaphthalene-7,8-oxide | 10 | ec:2.5.1.18 |
| cpd:C14800 |  | 1-Nitronaphthalene-5,6-oxide | 10 | ec:2.5.1.18 |
| cpd:C01179 |  | 3-(4-Hydroxyphenyl)pyruvate; 4-Hydroxyphenylpyruvate; p-Hydroxyphenylpyruvic acid | 10 | ec:2.6.1.1 |
| cpd:C00530 |  | Hydroquinone; p-Benzenediol; 1,4-Benzenediol; 1,4-Dihydroxybenzene; Benzene-1,4-diol; Quinol; 4-Hydroxyphenol | 10 |  |
| cpd:C00097 |  | L-Cysteine; L-2-Amino-3-mercaptopropionic acid | 10 | ec:2.6.1.1 |
| cpd:C03680 |  | 4-Imidazolone-5-propanoate; 4-Imidazolone-5-propionic acid; 4,5-Dihydro-4-oxo-5-imidazolepropanoate | 10 |  |
| cpd:C00082 |  | L-Tyrosine; (S)-3-(p-Hydroxyphenyl)alanine; (S)-2-Amino-3-(p-hydroxyphenyl)propionic acid; Tyrosine | 10 | ec:2.6.1.1 |
| cpd:C00079 |  | L-Phenylalanine; (S)-alpha-Amino-beta-phenylpropionic acid | 10 | ec:2.6.1.1 |
| cpd:C13645 |  | Hydrobromic acid; HBr | 10 | ec:2.5.1.18 |
| cpd:C00506 |  | L-Cysteate; L-Cysteic acid; 3-Sulfoalanine; 2-Amino-3-sulfopropionic acid | 10 | ec:2.6.1.1 |
| cpd:C07097 |  | 2,6-Dichlorohydroquinone | 10 |  |
| cpd:C07096 |  | 2,6-Dichlorophenol | 10 |  |
| cpd:C00462 |  | Halide; Hydrogen halide; HX; Halo acid | 10 | ec:2.5.1.18 |
| cpd:C11088 |  | 1,2-Dibromoethane; Ethylene dibromide | 10 | ec:2.5.1.18 |
| cpd:C13638 |  | 1-(4'-Hydroxyphenyl)ethanol; 4-Hydroxy-alpha-methyl-benzenemethanol; 4-(1-Hydroxyethyl)phenol | 10 |  |
| cpd:C13637 |  | 4-Ethylphenol; p-Ethylphenol | 10 |  |
| cpd:C19586 |  | Aflatoxin B1-exo-8,9-epoxide; 2,3-Epoxyaflatoxin B1 | 10 | ec:2.5.1.18 |
| cpd:C13631 |  | 2,2-Bis(4-hydroxyphenyl)-1-propanol | 10 |  |
| cpd:C00059 |  | Sulfate; Sulfuric acid | 10 |  |
| cpd:C13624 |  | Bisphenol A; 2,2-Bis(4-Hydroxyphenyl)propane | 10 |  |
| cpd:C06790 |  | Trichloroethene; Trichloroethylene; TCE | 10 | ec:2.5.1.18 |
| cpd:C00036 |  | Oxaloacetate; Oxalacetic acid; Oxaloacetic acid; 2-Oxobutanedioic acid; 2-Oxosuccinic acid; keto-Oxaloacetate | 10 | ec:2.6.1.1 |
| cpd:C01468 |  | 4-Cresol; p-Cresol; 4-Hydroxytoluene; 4-Methylphenol | 10 |  |
| cpd:C01467 |  | 3-Cresol; m-Cresol; 3-Hydroxytoluene | 10 |  |
| cpd:C00026 |  | 2-Oxoglutarate; Oxoglutaric acid; 2-Ketoglutaric acid; alpha-Ketoglutaric acid | 10 | ec:2.6.1.1 |
| cpd:C00016 |  | FAD; Flavin adenine dinucleotide | 10 | ec:1.3.8.6 |
| cpd:C11432 |  | 1-Phenanthrol; 1-Hydroxyphenanthrene | 10 |  |
| cpd:C11431 |  | Phenanthrene-1,2-oxide | 10 |  |
| cpd:C11429 |  | Phenanthrene-9,10-oxide | 10 |  |
| cpd:C11422 |  | Phenanthrene; Phenanthracene | 10 |  |
| cpd:C02909 |  | (2-Naphthyl)methanol; 2-Naphthalenemethanol; 2-Hydroxymethylnaphthalene | 10 |  |
| cpd:C04570 |  | Reduced electron-transferring flavoprotein; Reduced electron-transfer flavoprotein | 9 | ec:1.3.8.6 |
| cpd:C02411 |  | Glutaconyl-1-CoA; 4-Carboxybut-2-enoyl-CoA; Glutaconyl-CoA; (E)-Glutaconyl-CoA; (2E)-4-Carboxybut-2-enoyl-CoA | 9 | ec:1.3.8.6 |
| cpd:C02713 |  | N-Acetylmuramate; N-Acetylmuramic acid; N-Acetyl-D-muramoate | 9 | ec:3.5.1.28 |
| cpd:C00527 |  | Glutaryl-CoA | 9 | ec:1.3.8.6 |
| cpd:C02999 |  | N-Acetylmuramoyl-Ala; N-Acetyl-D-muramoyl-L-alanine | 9 | ec:3.5.1.28 |
| cpd:C00877 |  | Crotonoyl-CoA; Crotonyl-CoA; 2-Butenoyl-CoA; trans-But-2-enoyl-CoA; But-2-enoyl-CoA; (E)-But-2-enoyl-CoA | 9 | ec:1.3.8.6 |
| cpd:C04253 |  | Electron-transferring flavoprotein; Electron-transfer flavoprotein | 9 | ec:1.3.8.6 |
| cpd:C00288 |  | HCO3-; Bicarbonate; Hydrogencarbonate; Acid carbonate | 8 | ec:6.3.5.5 |
| cpd:C00169 |  | Carbamoyl phosphate | 8 | ec:6.3.5.5 |
| cpd:C00160 |  | Glycolate; Glycolic acid; Hydroxyacetic acid | 7 | ec:3.8.1.3 ec:3.1.3.18 |
| cpd:C00090 |  | Catechol; 1,2-Benzenediol; o-Benzenediol; 1,2-Dihydroxybenzene; Brenzcatechin; Pyrocatechol | 7 | ec:1.13.11.1 ec:1.14.12.1 |
| cpd:C04688 |  | (3R)-3-Hydroxytetradecanoyl-[acyl-carrier protein]; (R)-3-Hydroxytetradecanoyl-[acyl-carrier protein]; beta-Hydroxymyristyl-[acyl-carrier protein]; HMA | 7 | ec:2.3.1.191 ec:4.2.1.59 ec:2.3.1.129 |
| cpd:C00399 |  | Ubiquinone; Coenzyme Q; CoQ; Q | 7 | ec:1.1.5.2 ec:1.2.5.1 |
| cpd:C00390 |  | Ubiquinol; QH2; CoQH2 | 7 | ec:1.1.5.2 ec:1.2.5.1 |
| cpd:C00198 |  | D-Glucono-1,5-lactone; Gluconic lactone; Gluconic acid lactone; 1,5-Gluconolactone; delta-Gluconolactone; D-Gluconolactone; Gluconolactone | 6 | ec:1.1.5.2 |
| cpd:C00229 |  | Acyl-carrier protein; ACP; [Acyl-carrier protein]; Holo-[acyl-carrier protein] | 6 | ec:2.3.1.191 ec:2.3.1.129 |
| cpd:C00101 |  | Tetrahydrofolate; 5,6,7,8-Tetrahydrofolate; Tetrahydrofolic acid; THF; (6S)-Tetrahydrofolate; (6S)-Tetrahydrofolic acid; (6S)-THFA | 6 | ec:2.1.2.9 ec:2.1.1.13 ec:2.1.2.1 |
| cpd:C00031 |  | D-Glucose; Grape sugar; Dextrose; Glucose; D-Glucopyranose | 6 | ec:1.1.5.2 |
| cpd:C04738 |  | UDP-3-O-(3-hydroxytetradecanoyl)-N-acetylglucosamine; UDP-3-O-(beta-hydroxymyristoyl)-N-acetylglucosamine; UDP-3-O-[(3R)-3-hydroxymyristoyl]-N-acetylglucosamine | 5 | ec:2.3.1.129 |
| cpd:C00043 |  | UDP-N-acetyl-alpha-D-glucosamine; UDP-N-acetyl-D-glucosamine; UDP-N-acetylglucosamine | 5 | ec:2.3.1.129 |
| cpd:C00794 |  | D-Sorbitol; D-Glucitol; L-Gulitol; Sorbitol | 5 | ec:1.1.1.14 |
| cpd:C00033 |  | Acetate; Acetic acid; Ethanoic acid | 5 | ec:4.1.3.4 ec:1.2.5.1 |
| cpd:C00024 |  | Acetyl-CoA; Acetyl coenzyme A | 5 | ec:4.1.3.4 ec:2.3.1.183 |
| cpd:C00013 |  | Diphosphate; Diphosphoric acid; Pyrophosphate; Pyrophosphoric acid; PPi | 5 | ec:3.5.4.25 ec:6.3.5.4 ec:4.1.99.12 |
| cpd:C02430 |  | L-Methionyl-tRNA; L-Methionyl-tRNA(Met) | 4 | ec:2.1.2.9 |
| cpd:C00234 |  | 10-Formyltetrahydrofolate; 10-Formyl-THF | 4 | ec:2.1.2.9 |
| cpd:C00988 |  | 2-Phosphoglycolate; Phosphoglycolic acid | 4 | ec:3.1.3.18 |
| cpd:C00164 |  | Acetoacetate; 3-Oxobutanoic acid; beta-Ketobutyric acid; Acetoacetic acid | 4 | ec:4.1.3.4 |
| cpd:C03294 |  | N-Formylmethionyl-tRNA | 4 | ec:2.1.2.9 |
| cpd:C16466 |  | 7-Methyl-3-oxo-6-octenoyl-CoA | 4 | ec:4.1.3.4 |
| cpd:C16393 |  | 2-Hydroxylamino-4,6-dinitrotoluene | 4 |  |
| cpd:C16392 |  | 4-Hydroxylamino-2,6-dinitrotoluene | 4 |  |
| cpd:C16391 |  | Trinitrotoluene; 2,4,6-Trinitrotoluene | 4 |  |
| cpd:C01138 |  | Streptomycin 6-phosphate | 4 | ec:2.7.1.72 |
| cpd:C04675 |  | 3-Hydroxy-3-(4-methylpent-3-en-1-yl)glutaryl-CoA; 3-Hydroxy-3-isohexeneylglutaryl-CoA | 4 | ec:4.1.3.4 |
| cpd:C00020 |  | AMP; Adenosine 5'-monophosphate; Adenylic acid; Adenylate; 5'-AMP; 5'-Adenylic acid; 5'-Adenosine monophosphate; Adenosine 5'-phosphate | 4 | ec:6.3.5.4 |
| cpd:C00413 |  | Streptomycin | 4 | ec:2.7.1.72 |
| cpd:C00356 |  | (S)-3-Hydroxy-3-methylglutaryl-CoA; Hydroxymethylglutaryl-CoA; Hydroxymethylglutaroyl coenzyme A; HMG-CoA; 3-Hydroxy-3-methylglutaryl-CoA | 4 | ec:4.1.3.4 |
| cpd:C03453 |  | gamma-Oxalocrotonate; (Z)-5-Oxohex-2-enedioate; 4-Oxalocrotonate | 3 | ec:5.3.2.6 |
| cpd:C01674 |  | Chitobiose; Diacetylchitobiose; N,N'-Diacetylchitobiose | 3 | ec:3.2.1.52 |
| cpd:C00152 |  | L-Asparagine; 2-Aminosuccinamic acid | 3 | ec:6.3.5.4 |
| cpd:C00140 |  | N-Acetyl-D-glucosamine; N-Acetylchitosamine; 2-Acetamido-2-deoxy-D-glucose; GlcNAc | 3 | ec:3.2.1.52 |
| cpd:C01132 |  | N-Acetyl-D-galactosamine; N-Acetyl-D-chondrosamine; 2-Acetamido-2-deoxy-D-galactose | 3 | ec:3.2.1.52 |
| cpd:C07479 |  | 2-Oxo-5-methyl-cis-muconate | 3 | ec:5.3.2.6 |
| cpd:C07478 |  | 2-Hydroxy-5-methyl-cis,cis-muconate | 3 | ec:5.3.2.6 |
| cpd:C06755 |  | Chloroacetic acid; Chloroethanoic acid | 3 | ec:3.8.1.3 |
| cpd:C02501 |  | 2-Hydroxymuconate | 3 | ec:5.3.2.6 |
| cpd:C15556 |  | L-3,4-Dihydroxybutan-2-one 4-phosphate; 1-Deoxy-L-glycero-tetrulose 4-phosphate; 2-Hydroxy-3-oxobutyl phosphate | 2 | ec:3.5.4.25 ec:4.1.99.12 ec:2.5.1.78 |
| cpd:C04112 |  | 3-Methyl-cis,cis-hexadienedioate; 3-Methyl-cis,cis-muconate | 2 | ec:5.5.1.1 ec:1.13.11.1 |
| cpd:C01304 |  | 2,5-Diamino-6-(5-phospho-D-ribosylamino)pyrimidin-4(3H)-one; 2,5-Diamino-6-(1-D-ribosylamino)pyrimidin-4(3H)-one 5'-phosphate | 2 | ec:3.5.4.25 ec:3.5.4.26 ec:1.1.1.193 ec:4.1.99.12 |
| cpd:C00143 |  | 5,10-Methylenetetrahydrofolate; (6R)-5,10-Methylenetetrahydrofolate; 5,10-Methylene-THF | 2 | ec:1.5.1.20 ec:2.1.2.1 |
| cpd:C16474 |  | 3-Fluoro-cis,cis-muconate | 2 | ec:5.5.1.1 ec:1.13.11.1 |
| cpd:C04732 |  | 5-Amino-6-(1-D-ribitylamino)uracil; 5-Amino-6-(D-ribitylamino)uracil; 6-(1-D-Ribitylamino)-5-amino-2,4-dihydroxypyrimidine; 6-(1-D-Ribitylamino)-5-aminouracil; 4-(1-D-Ribitylamino)-5-amino-2,6-dihydroxypyrimidine | 2 | ec:2.5.1.9 ec:2.5.1.78 |
| cpd:C05378 |  | beta-D-Fructose 1,6-bisphosphate | 2 | ec:3.1.3.11 ec:4.1.2.13 |
| cpd:C04332 |  | 6,7-Dimethyl-8-(D-ribityl)lumazine | 2 | ec:2.5.1.9 ec:2.5.1.78 |
| cpd:C00440 |  | 5-Methyltetrahydrofolate | 2 | ec:1.5.1.20 ec:2.1.1.13 |
| cpd:C03585 |  | 3-Chloro-cis,cis-muconate | 2 | ec:5.5.1.1 ec:1.13.11.1 |
| cpd:C00010 |  | CoA; Coenzyme A; CoA-SH | 2 | ec:2.3.1.183 |
| cpd:C02480 |  | cis,cis-Muconate; cis,cis-Hexadienedioate; cis,cis-2,4-Hexadienedioic acid | 2 | ec:5.5.1.1 ec:1.13.11.1 |
| cpd:C14610 |  | (S)-5-Oxo-2,5-dihydrofuran-2-acetate; (+)-Muconolactone | 2 | ec:5.5.1.1 ec:5.3.3.4 |
| cpd:C00354 |  | D-Fructose 1,6-bisphosphate | 2 | ec:3.1.3.11 ec:4.1.2.13 |
| cpd:C01419 |  | Cys-Gly; L-Cysteinylglycine | 1 | ec:2.3.2.2 |
| cpd:C12833 |  | 2,3,5-Trichloro-cis,cis-muconate | 1 | ec:1.13.11.1 |
| cpd:C12831 |  | 3,4,6-Trichlorocatechol | 1 | ec:1.13.11.1 |
| cpd:C16268 |  | Cyclopropanecarboxyl-CoA | 1 |  |
| cpd:C16267 |  | Cyclopropanecarboxylate | 1 |  |
| cpd:C14143 |  | Adipyl-CoA; 5-Carboxypentanoyl-CoA | 1 |  |
| cpd:C04559 |  | 4-Methylmuconolactone; 4-Carboxymethyl-4-methylbut-2-en-1,4-olide | 1 | ec:5.5.1.1 |
| cpd:C04558 |  | 3-Methylmuconolactone; 4-Carboxymethyl-3-methylbut-2-en-1,4-olide | 1 | ec:5.5.1.1 |
| cpd:C00279 |  | D-Erythrose 4-phosphate | 1 | ec:4.1.2.13 |
| cpd:C04144 |  | Tetrahydropteroyltri-L-glutamate | 1 | ec:2.1.1.13 |
| cpd:C05618 |  | 3-Chlorocatechol | 1 | ec:1.13.11.1 |
| cpd:C04494 |  | Guanosine 3'-diphosphate 5'-triphosphate; Guanosine 5'-triphosphate,3'-diphosphate | 1 | ec:3.6.1.11 ec:3.6.1.40 |
| cpd:C02375 |  | 4-Chlorocatechol | 1 | ec:1.13.11.1 |
| cpd:C00255 |  | Riboflavin; Lactoflavin; 7,8-Dimethyl-10-ribitylisoalloxazine; Vitamin B2 | 1 | ec:2.5.1.9 |
| cpd:C04489 |  | 5-Methyltetrahydropteroyltri-L-glutamate | 1 | ec:2.1.1.13 |
| cpd:C16221 |  | (2E)-Octadecenoyl-[acp]; trans-Octadec-2-enoyl-[acp] | 1 | ec:4.2.1.59 |
| cpd:C16220 |  | 3-Hydroxyoctadecanoyl-[acp]; 3-Hydroxystearoyl-[acp] | 1 | ec:4.2.1.59 |
| cpd:C05951 |  | Leukotriene D4; LTD4 | 1 | ec:2.3.2.2 |
| cpd:C00245 |  | Taurine; 2-Aminoethanesulfonic acid; Aminoethylsulfonic acid | 1 | ec:2.3.2.2 |
| cpd:C00236 |  | 3-Phospho-D-glyceroyl phosphate; 1,3-Bisphospho-D-glycerate; (R)-2-Hydroxy-3-(phosphonooxy)-1-monoanhydride with phosphoric propanoic acid; D-Glycerate 1,3-diphosphate | 1 | ec:2.7.2.3 |
| cpd:C00199 |  | D-Ribulose 5-phosphate | 1 | ec:3.5.4.25 ec:4.1.99.12 |
| cpd:C00197 |  | 3-Phospho-D-glycerate; D-Glycerate 3-phosphate; 3-Phospho-(R)-glycerate; 3-Phosphoglycerate | 1 | ec:2.7.2.3 |
| cpd:C01268 |  | 5-Amino-6-(5'-phosphoribosylamino)uracil; 5-Amino-6-(ribosylamino)-2,4-(1H,3H)-pyrimidinedione 5'-phosphate; 5-Amino-6-(5-phosphoribosylamino)uracil | 1 | ec:3.5.4.26 ec:1.1.1.193 |
| cpd:C04454 |  | 5-Amino-6-(5'-phospho-D-ribitylamino)uracil; 5-Amino-2,6-dioxy-4-(5'-phospho-D-ribitylamino)pyrimidine; 5-Amino-6-(5-phospho-D-ribitylamino)uracil | 1 | ec:3.5.4.26 ec:1.1.1.193 |
| cpd:C00577 |  | D-Glyceraldehyde | 1 | ec:4.1.2.13 |
| cpd:C03363 |  | 5-L-Glutamyl amino acid; L-gamma-Glutamyl amino acid | 1 | ec:2.3.2.2 |
| cpd:C06156 |  | alpha-D-Glucosamine 1-phosphate; D-Glucosamine 1-phosphate | 1 | ec:5.4.2.10 |
| cpd:C17962 |  | Demethylphosphinothricin; (2S)-2-Amino-4-(hydroxyphosphinyl)butanoic acid | 1 | ec:2.3.1.183 |
| cpd:C18241 |  | Tetrachloro-cis,cis-muconate | 1 | ec:1.13.11.1 |
| cpd:C18240 |  | Tetrachlorocatechol; Tetrachloro-1,2-benzenediol | 1 | ec:1.13.11.1 |
| cpd:C00155 |  | L-Homocysteine; L-2-Amino-4-mercaptobutyric acid | 1 | ec:2.1.1.13 |
| cpd:C05460 |  | 3alpha,7alpha,12alpha-Trihydroxy-5beta-cholest-24-enoyl-CoA | 1 |  |
| cpd:C00151 |  | L-Amino acid; L-2-Amino acid | 1 | ec:2.3.2.2 |
| cpd:C03740 |  | (5-L-Glutamyl)-L-amino acid; L-gamma-Glutamyl-L-amino acid | 1 | ec:2.3.2.2 |
| cpd:C01228 |  | Guanosine 3',5'-bis(diphosphate); Guanosine 3'-diphosphate 5'-diphosphate; Guanosine 5'-diphosphate,3'-diphosphate | 1 | ec:3.6.1.11 ec:3.6.1.40 |
| cpd:C17952 |  | N-Acetylphosphinothricin; L-N-Acetylphosphinothricin; N-Acetyl-L-Glufosinate; N-Acetylphinothricin | 1 | ec:2.3.1.183 |
| cpd:C16476 |  | 4-Fluoromuconolactone | 1 | ec:5.5.1.1 |
| cpd:C16475 |  | 2-Fluoro-cis,cis-muconate | 1 | ec:1.13.11.1 |
| cpd:C04377 |  | 5,10-Methylenetetrahydromethanopterin; N5,N10-Methylenetetrahydromethanopterin | 1 | ec:2.1.2.1 |
| cpd:C16473 |  | 4-Fluorocatechol | 1 | ec:1.13.11.1 |
| cpd:C16472 |  | 3-Fluorocatechol | 1 | ec:1.13.11.1 |
| cpd:C16470 |  | 5-Methylhex-4-enoyl-CoA | 1 |  |
| cpd:C17949 |  | N-Acetyldemethylphosphinothricin; N-Adpt | 1 | ec:2.3.1.183 |
| cpd:C01217 |  | 5,6,7,8-Tetrahydromethanopterin; H4MPT; THMPT; Tetrahydromethanopterin | 1 | ec:2.1.2.1 |
| cpd:C05844 |  | 5-L-Glutamyl-taurine; 5-Glutamyl-taurine; Glutaurine | 1 | ec:2.3.2.2 |
| cpd:C05447 |  | 3alpha,7alpha-Dihydroxy-5beta-cholest-24-enoyl-CoA | 1 |  |
| cpd:C16468 |  | (2E)-5-Methylhexa-2,4-dienoyl-CoA | 1 |  |
| cpd:C04760 |  | 3alpha,7alpha,12alpha-Trihydroxy-5beta-cholestanoyl-CoA | 1 |  |
| cpd:C05042 |  | Glufosinate; Phosphinothricin; 2-Amino-4-(hydroxymethylphosphinyl)butanoic acid | 1 | ec:2.3.1.183 |
| cpd:C11946 |  | cis-2-Methyl-5-isopropylhexa-2,5-dienoyl-CoA | 1 |  |
| cpd:C11945 |  | trans-2-Methyl-5-isopropylhexa-2,5-dienoyl-CoA | 1 |  |
| cpd:C11944 |  | cis-2-Methyl-5-isopropylhexa-2,5-dienoic acid | 1 |  |
| cpd:C11943 |  | trans-2-Methyl-5-isopropylhexa-2,5-dienoic acid | 1 |  |
| cpd:C06114 |  | gamma-Glutamyl-beta-aminopropiononitrile; gamma-Glutamyl-3-aminopropiononitrile | 1 | ec:2.3.2.2 |
| cpd:C00085 |  | D-Fructose 6-phosphate; D-Fructose 6-phosphoric acid; Neuberg ester | 1 | ec:3.1.3.11 |
| cpd:C06104 |  | Adipate; Adipic acid; Hexanedioate; Hexan-1,6-dicarboxylate | 1 |  |
| cpd:C00118 |  | D-Glyceraldehyde 3-phosphate; (2R)-2-Hydroxy-3-(phosphonooxy)-propanal; Glyceraldehyde 3-phosphate | 1 | ec:4.1.2.13 |
| cpd:C00111 |  | Glycerone phosphate; Dihydroxyacetone phosphate | 1 | ec:4.1.2.13 |
| cpd:C00073 |  | L-Methionine; Methionine; L-2-Amino-4methylthiobutyric acid | 1 | ec:2.1.1.13 |
| cpd:C11929 |  | Perillyl-CoA | 1 |  |
| cpd:C11924 |  | Perillic acid | 1 |  |
| cpd:C07090 |  | Protoanemonin; 4-Methylenebut-2-en-4-olide; cis-4-Methylenebut-2-en-4-olide | 1 | ec:5.5.1.1 |
| cpd:C00065 |  | L-Serine; L-2-Amino-3-hydroxypropionic acid; L-3-Hydroxy-alanine; Serine | 1 | ec:2.1.2.1 |
| cpd:C05763 |  | trans-Hexadec-2-enoyl-[acp]; trans-Hexadec-2-enoyl-[acyl-carrier protein]; (2E)-Hexadecenoyl-[acp] | 1 | ec:4.2.1.59 |
| cpd:C05760 |  | trans-Tetradec-2-enoyl-[acp]; trans-Tetradec-2-enoyl-[acyl-carrier protein]; (2E)-Tetradecenoyl-[acp] | 1 | ec:4.2.1.59 |
| cpd:C01094 |  | D-Fructose 1-phosphate | 1 | ec:4.1.2.13 |
| cpd:C20378 |  | Enoylpimeloyl-[acp] methyl ester; Enoylpimeloyl-[acyl-carrier protein] methyl ester | 1 | ec:4.2.1.59 |
| cpd:C20377 |  | 3-Hydroxypimeloyl-[acp] methyl ester; 3-Hydroxypimeloyl-[acyl-carrier protein] methyl ester | 1 | ec:4.2.1.59 |
| cpd:C05758 |  | trans-Dodec-2-enoyl-[acp]; trans-Dodec-2-enoyl-[acyl-carrier protein]; (2E)-Dodecenoyl-[acp] | 1 | ec:4.2.1.59 |
| cpd:C05757 |  | (R)-3-Hydroxydodecanoyl-[acp]; (R)-3-Hydroxydodecanoyl-[acyl-carrier protein]; D-3-Hydroxydodecanoyl-[acp]; D-3-Hydroxydodecanoyl-[acyl-carrier protein] | 1 | ec:4.2.1.59 |
| cpd:C02166 |  | Leukotriene C4; LTC4 | 1 | ec:2.3.2.2 |
| cpd:C20374 |  | Enoylglutaryl-[acp] methyl ester; Enoylglutaryl-[acyl-carrier protein] methyl ester | 1 | ec:4.2.1.59 |
| cpd:C20373 |  | 3-Hydroxyglutaryl-[acp] methyl ester; 3-Hydroxyglutaryl-[acyl-carrier protein] methyl ester | 1 | ec:4.2.1.59 |
| cpd:C05754 |  | trans-Dec-2-enoyl-[acp]; trans-Dec-2-enoyl-[acyl-carrier protein]; trans-2-Decenoyl-[acyl-carrier protein]; (2E)-Decenoyl-[acp] | 1 | ec:4.2.1.59 |
| cpd:C00447 |  | Sedoheptulose 1,7-bisphosphate; D-Sedoheptulose 1,7-bisphosphate; D-altro-Heptulose 1,7-biphosphate | 1 | ec:4.1.2.13 |
| cpd:C05751 |  | trans-Oct-2-enoyl-[acp]; trans-Oct-2-enoyl-[acyl-carrier protein]; Oct-2-enoyl-[acyl-carrier protein]; 2-Octenoyl-[acyl-carrier protein]; (2E)-Octenoyl-[acp] | 1 | ec:4.2.1.59 |
| cpd:C00045 |  | Amino acid; Amino acids | 1 | ec:2.3.2.2 |
| cpd:C00044 |  | GTP; Guanosine 5'-triphosphate | 1 | ec:3.5.4.25 ec:4.1.99.12 |
| cpd:C05748 |  | trans-Hex-2-enoyl-[acp]; trans-Hex-2-enoyl-[acyl-carrier protein]; (2E)-Hexenoyl-[acp] | 1 | ec:4.2.1.59 |
| cpd:C05747 |  | (R)-3-Hydroxyhexanoyl-[acp]; (R)-3-Hydroxyhexanoyl-[acyl-carrier protein]; D-3-Hydroxyhexanoyl-[acp]; D-3-Hydroxyhexanoyl-[acyl-carrier protein] | 1 | ec:4.2.1.59 |
| cpd:C03193 |  | (5-L-Glutamyl)-peptide | 1 | ec:2.3.2.2 |
| cpd:C05345 |  | beta-D-Fructose 6-phosphate | 1 | ec:3.1.3.11 |
| cpd:C00037 |  | Glycine; Aminoacetic acid; Gly | 1 | ec:2.1.2.1 |
| cpd:C06022 |  | UDP-3-O-(3-hydroxytetradecanoyl)-D-glucosamine; UDP-3-O-(beta-hydroxymyristoyl)-D-glucosamine; UDP-3-O-[(3R)-3-hydroxymyristoyl]-D-glucosamine; UDP-3-O-[(3R)-3-hydroxymyristoyl]-alpha-D-glucosamine | 1 | ec:2.3.1.191 |
| cpd:C03586 |  | 2-Oxo-2,3-dihydrofuran-5-acetate; 3-Oxoadipate enol-lactone; 4,5-Dihydro-5-oxofuran-2-acetate; 5-Oxo-4,5-dihydrofuran-2-acetate | 1 | ec:5.3.3.4 |
| cpd:C05698 |  | Selenohomocysteine | 1 | ec:2.1.1.13 |
| cpd:C05695 |  | gamma-Glutamyl-Se-methylselenocysteine; 5-L-Glutamyl-Se-methylselenocysteine | 1 | ec:2.3.2.2 |
| cpd:C05335 |  | L-Selenomethionine | 1 | ec:2.1.1.13 |
| cpd:C04652 |  | UDP-2,3-bis(3-hydroxytetradecanoyl)glucosamine; UDP-2,3-bis(beta-hydroxymyristoyl)-D-glucosamine; UDP-2,3-bis(3-hydroxytetradecanoyl)-D-glucosamine; UDP-2,3-bis[O-(3R)-3-hydroxymyristoyl]-alpha-D-glucosamine | 1 | ec:2.3.1.191 |
| cpd:C00022 |  | Pyruvate; Pyruvic acid; 2-Oxopropanoate; 2-Oxopropanoic acid; Pyroracemic acid | 1 | ec:1.2.5.1 |
| cpd:C03572 |  | 2-Chloro-cis,cis-muconate | 1 | ec:1.13.11.1 |
| cpd:C05729 |  | R-S-Cysteinylglycine | 1 | ec:2.3.2.2 |
| cpd:C05689 |  | Se-Methyl-L-selenocysteine | 1 | ec:2.3.2.2 |
| cpd:C04644 |  | 3alpha,7alpha-Dihydroxy-5beta-cholestanoyl-CoA | 1 |  |
| cpd:C00012 |  | Peptide | 1 | ec:2.3.2.2 |
| cpd:C04246 |  | But-2-enoyl-[acyl-carrier protein] | 1 | ec:4.2.1.59 |
| cpd:C05711 |  | gamma-Glutamyl-beta-cyanoalanine | 1 | ec:2.3.2.2 |
| cpd:C04633 |  | (3R)-3-Hydroxypalmitoyl-[acyl-carrier protein]; (R)-3-Hydroxypalmitoyl-[acyl-carrier protein]; (3R)-3-Hydroxyhexadecanoyl-[acyl-carrier protein]; (R)-3-Hydroxyhexadecanoyl-[acyl-carrier protein] | 1 | ec:4.2.1.59 |
| cpd:C05670 |  | 3-Aminopropiononitrile; beta-Aminopropionitrile | 1 | ec:2.3.2.2 |
| cpd:C11421 |  | (3S)-3-Isopropenyl-6-oxoheptanoyl-CoA | 1 |  |
| cpd:C02512 |  | 3-Cyano-L-alanine; L-3-Cyanoalanine; L-beta-Cyanoalanine | 1 | ec:2.3.2.2 |
| cpd:C11419 |  | (3S)-3-Isopropenyl-6-oxoheptanoate; (3S)-3-Isopropenyl-6-oxoheptanoic acid | 1 |  |
| cpd:C12450 |  | Pseudoecgonyl-CoA | 1 |  |
| cpd:C00352 |  | D-Glucosamine 6-phosphate; D-Glucosamine phosphate | 1 | ec:5.4.2.10 |
| cpd:C04620 |  | (3R)-3-Hydroxyoctanoyl-[acyl-carrier protein]; (R)-3-Hydroxyoctanoyl-[acyl-carrier protein] | 1 | ec:4.2.1.59 |
| cpd:C06730 |  | 4-Methylcatechol; 3,4-Dihydroxytoluene; 1,2-Dihydroxy-4-methylbenzene; 4-Methyl-1,2-benzenediol | 1 | ec:1.13.11.1 |
| cpd:C12449 |  | Pseudoecgonine | 1 |  |
| cpd:C04619 |  | (3R)-3-Hydroxydecanoyl-[acyl-carrier protein]; (R)-3-Hydroxydecanoyl-[acyl-carrier protein] | 1 | ec:4.2.1.59 |
| cpd:C04618 |  | (3R)-3-Hydroxybutanoyl-[acyl-carrier protein]; (R)-3-Hydroxybutanoyl-[acyl-carrier protein] | 1 | ec:4.2.1.59 |
| cpd:C11407 |  | (3R)-3-Isopropenyl-6-oxoheptanoyl-CoA | 1 |  |
| cpd:C11405 |  | (3R)-3-Isopropenyl-6-oxoheptanoate; (3R)-3-Isopropenyl-6-oxoheptanoic acid | 1 |  |

  
**Over-represented Pathway Summary**: Collection of the KEGG metabolic pathways containing the proteins identified in the "Over-represented Metabolite Summary" ranked by the highest number of hits per pathway  

| Pathway ID | EC | EC Frequency | Name |
| --- | --- | --- | --- |
| map00380 | ec:3.5.1.9 ec:1.3.8.6 ec:1.13.11.11 ec:3.7.1.3 | 140 | path:map00380 Tryptophan metabolism |
| map00630 | ec:3.5.1.9 ec:3.1.3.18 ec:2.1.2.1 | 46 | path:map00630 Glyoxylate and dicarboxylate metabolism |
| map00627 | ec:4.1.1.7 ec:1.14.12.1 ec:1.2.1.28 | 41 | path:map00627 Aminobenzoate degradation |
| map00040 | ec:2.7.1.17 ec:1.1.1.11 | 41 | path:map00040 Pentose and glucuronate interconversions |
| map00750 | ec:1.4.3.5 | 35 | path:map00750 Vitamin B6 metabolism |
| map00051 | ec:1.1.1.14 ec:1.1.1.11 ec:3.1.3.11 ec:4.1.2.13 | 29 | path:map00051 Fructose and mannose metabolism |
| map00240 | ec:1.8.1.9 ec:6.3.5.5 ec:2.7.7.7 | 24 | path:map00240 Pyrimidine metabolism |
| map00622 | ec:5.3.2.6 ec:1.2.1.28 | 22 | path:map00622 Xylene degradation |
| map00250 | ec:6.3.5.5 ec:6.3.5.4 ec:2.6.1.1 | 21 | path:map00250 Alanine, aspartate and glutamate metabolism |
| map00623 | ec:1.13.11.1 ec:5.5.1.1 ec:1.2.1.28 | 21 | path:map00623 Toluene degradation |
| map00770 | ec:1.1.1.169 | 19 | path:map00770 Pantothenate and CoA biosynthesis |
| map00450 | ec:2.1.1.13 ec:1.8.1.9 | 15 | path:map00450 Selenocompound metabolism |
| map00710 | ec:3.1.3.11 ec:4.1.2.13 ec:2.7.2.3 ec:2.6.1.1 | 13 | path:map00710 Carbon fixation in photosynthetic organisms |
| map00480 | ec:2.5.1.18 ec:2.3.2.2 | 11 | path:map00480 Glutathione metabolism |
| map00270 | ec:2.1.1.13 ec:2.6.1.1 | 11 | path:map00270 Cysteine and methionine metabolism |
| map00960 | ec:2.6.1.1 | 10 | path:map00960 Tropane, piperidine and pyridine alkaloid biosynthesis |
| map00350 | ec:2.6.1.1 | 10 | path:map00350 Tyrosine metabolism |
| map00950 | ec:2.6.1.1 | 10 | path:map00950 Isoquinoline alkaloid biosynthesis |
| map00982 | ec:2.5.1.18 | 10 | path:map00982 Drug metabolism - cytochrome P450 |
| map00980 | ec:2.5.1.18 | 10 | path:map00980 Metabolism of xenobiotics by cytochrome P450 |
| map00401 | ec:2.6.1.1 | 10 | path:map00401 Novobiocin biosynthesis |
| map00400 | ec:2.6.1.1 | 10 | path:map00400 Phenylalanine, tyrosine and tryptophan biosynthesis |
| map00330 | ec:2.6.1.1 | 10 | path:map00330 Arginine and proline metabolism |
| map00360 | ec:2.6.1.1 | 10 | path:map00360 Phenylalanine metabolism |
| map00310 | ec:1.3.8.6 | 9 | path:map00310 Lysine degradation |
| map00071 | ec:1.3.8.6 | 9 | path:map00071 Fatty acid degradation |
| map00030 | ec:1.1.5.2 ec:3.1.3.11 ec:4.1.2.13 | 8 | path:map00030 Pentose phosphate pathway |
| map00670 | ec:2.1.1.13 ec:2.1.2.9 ec:1.5.1.20 ec:2.1.2.1 | 7 | path:map00670 One carbon pool by folate |
| map00740 | ec:2.5.1.78 ec:2.5.1.9 ec:1.1.1.193 ec:3.5.4.26 ec:4.1.99.12 ec:3.5.4.25 | 6 | path:map00740 Riboflavin metabolism |
| map00540 | ec:2.3.1.191 ec:2.3.1.129 | 6 | path:map00540 Lipopolysaccharide biosynthesis |
| map00362 | ec:1.13.11.1 ec:5.3.3.4 ec:5.5.1.1 ec:5.3.2.6 | 6 | path:map00362 Benzoate degradation |
| map00361 | ec:1.13.11.1 ec:3.8.1.3 ec:5.5.1.1 | 5 | path:map00361 Chlorocyclohexane and chlorobenzene degradation |
| map00281 | ec:4.1.3.4 | 4 | path:map00281 Geraniol degradation |
| map00280 | ec:4.1.3.4 | 4 | path:map00280 Valine, leucine and isoleucine degradation |
| map00970 | ec:2.1.2.9 | 4 | path:map00970 Aminoacyl-tRNA biosynthesis |
| map00650 | ec:4.1.3.4 | 4 | path:map00650 Butanoate metabolism |
| map00072 | ec:4.1.3.4 | 4 | path:map00072 Synthesis and degradation of ketone bodies |
| map00521 | ec:2.7.1.72 | 4 | path:map00521 Streptomycin biosynthesis |
| map00520 | ec:5.4.2.10 ec:3.2.1.52 | 4 | path:map00520 Amino sugar and nucleotide sugar metabolism |
| map00230 | ec:3.6.1.40 ec:2.7.7.7 ec:3.6.1.11 | 4 | path:map00230 Purine metabolism |
| map00513 | ec:3.2.1.52 | 3 | path:map00513 Various types of N-glycan biosynthesis |
| map00511 | ec:3.2.1.52 | 3 | path:map00511 Other glycan degradation |
| map00604 | ec:3.2.1.52 | 3 | path:map00604 Glycosphingolipid biosynthesis - ganglio series |
| map00603 | ec:3.2.1.52 | 3 | path:map00603 Glycosphingolipid biosynthesis - globo series |
| map00531 | ec:3.2.1.52 | 3 | path:map00531 Glycosaminoglycan degradation |
| map00625 | ec:3.8.1.3 | 3 | path:map00625 Chloroalkane and chloroalkene degradation |
| map00621 | ec:5.3.2.6 | 3 | path:map00621 Dioxin degradation |
| map00010 | ec:3.1.3.11 ec:4.1.2.13 ec:2.7.2.3 | 3 | path:map00010 Glycolysis / Gluconeogenesis |
| map00680 | ec:3.1.3.11 ec:4.1.2.13 ec:2.1.2.1 | 3 | path:map00680 Methane metabolism |
| map00460 | ec:2.1.2.1 ec:2.3.2.2 | 2 | path:map00460 Cyanoamino acid metabolism |
| map00364 | ec:1.13.11.1 ec:5.5.1.1 | 2 | path:map00364 Fluorobenzoate degradation |
| map00061 | ec:4.2.1.59 | 1 | path:map00061 Fatty acid biosynthesis |
| map00590 | ec:2.3.2.2 | 1 | path:map00590 Arachidonic acid metabolism |
| map00720 | ec:1.5.1.20 | 1 | path:map00720 Carbon fixation pathways in prokaryotes |
| map00430 | ec:2.3.2.2 | 1 | path:map00430 Taurine and hypotaurine metabolism |
| map00780 | ec:4.2.1.59 | 1 | path:map00780 Biotin metabolism |
| map00620 | ec:1.2.5.1 | 1 | path:map00620 Pyruvate metabolism |
| map00260 | ec:2.1.2.1 | 1 | path:map00260 Glycine, serine and threonine metabolism |

  
Analysis performed on 2014/02/19 17:10:51
