# Supplementary material for: Automated genomic context analysis and experimental validation platform for discovery of prokaryote transcriptional regulator functions
Source: BMC Genomics. 2014 Dec 18;15(1):1142. doi: 10.1186/1471-2164-15-1142 (PMC4349456; doi:10.1186/1471-2164-15-1142)
Supplement: Supplementary file 9 — Additional file 9: Result HutC. Function Discovery V1.0 output (.html format) for the histidine degradation regulator (HutC, Bxe_ A2946). For detailed instructions on how to analyze the results please refer to the Function Discovery V1.0, a gene neighborhood analysis tool section in the Results part of the main text. (HTML 726 KB) [file 12864_2014_6995_MOESM9_ESM.html]

```
ENTRY       Bxe_A2946         CDS       T00340
DEFINITION  histidine utilization repressor
ORTHOLOGY   K05836  GntR family transcriptional regulator, histidine utilization repressor
ORGANISM    bxe  Burkholderia xenovorans
POSITION    1:1666475..1667170
MOTIF       Pfam: UTRA GntR HTH_DeoR HTH_41 MarR_2 HTH_24 HTH_Crp_2 HTH_11 MarR HTH_7 CENP-B_N Rrf2 TrmB Act-Frag_cataly
DBLINKS     NCBI-GI: 91782877
            NCBI-GeneID: 4003960
            JGI: BxeA2946
            UniProt: Q141F8
AASEQ       231
            MNAPAYQGIKDFILARIHAGEWGEGDQVPSENELAREFNVARMTVNRALRELTSEQVLTR
            VQGSGTFVARPKYESTLVAIRSISDEIVARGHRYQAKVLHIGASIADEALAGEMQVSAGS
            PVFHSRVLHFENDEPVQLEERWVNPAVAPEYARQDFTNTTPNQYLVRVAPLQRVEYRIEA
            LAADADTRELLTMDELEPCLVLHRRTWSQSQVASIANLWHPGSRYRFTGHF
NTSEQ       696
            atgaacgcaccggcctatcagggcatcaaggacttcatcctcgcgcgcatccatgcgggc
            gaatggggtgaaggcgaccaggtgccctccgaaaacgagctcgcgcgcgaattcaacgtg
            gcgcgcatgacggtcaaccgcgcgttgcgcgagctgacctcggagcaggtgctcacgcgt
            gtgcaaggttcgggcacgttcgtggcccgtcccaagtacgaatcgacgctggtggcgatc
            cgcagcatctccgacgaaatcgtcgcgcgcggtcatcgttatcaggcgaaggtgctgcat
            atcggcgcgagcatcgccgacgaagcgctcgccggggaaatgcaggtgagcgcgggcagc
            ccggtgtttcattcgcgcgtgctgcatttcgaaaacgacgagccggtgcagctcgaagag
            cgttgggtcaatccggcggtcgcgcccgagtacgcgcggcaggacttcaccaacaccacg
            ccgaaccagtatctcgtgcgcgtcgcgccgttgcagcgggtcgagtaccgcatcgaggcg
            ctggccgccgacgccgatacgcgcgaactgttgacgatggacgaactggagccctgcctc
            gtgctgcatcggcgcacgtggtcgcaaagccaggtcgcatcgatcgccaatctctggcat
            cccggcagccgctatcgcttcaccggacatttctga
///
```

  
**Homolog ID**: Table of closest homologs  

```
                 Homologs                                       len   identity overlap
---------------------------------------------------------------------------------
bpy:Bphyt_1531 transcriptional regulator, histidine uti K     231     0.983    231 
bgf:BC1003_2185 GntR family transcriptional regulator   K     231     0.970    231 
bge:BC1002_1092 GntR family transcriptional regulator   K     231     0.926    231 
bph:Bphy_1813 histidine utilization repressor           K     234     0.900    231 
bvi:Bcep1808_4120 histidine utilization repressor       K     231     0.814    231 
bch:Bcen2424_3722 histidine utilization repressor       K     231     0.810    231 
bcn:Bcen_4641 histidine utilization repressor           K     231     0.810    231 
bcj:BCAM0756 GntR family regulatory protein             K     231     0.810    231 
bcm:Bcenmc03_3799 histidine utilization repressor       K     231     0.805    231 
bam:Bamb_5462 histidine utilization repressor           K     231     0.810    231 
bmj:BMULJ_03508 GntR family transcriptional regulator   K     231     0.810    231 
bmu:Bmul_5005 histidine utilization repressor           K     231     0.810    231 
bur:Bcep18194_B2386 histidine utilization repressor     K     231     0.801    231 
bac:BamMC406_3606 histidine utilization repressor       K     231     0.801    231 
bgl:bglu_1g25200 histidine utilization repressor        K     231     0.801    231 
bma:BMA0646 histidine utilization repressor             K     231     0.775    231 
bml:BMA10229_A2920 histidine utilization repressor      K     231     0.775    231 
bmn:BMA10247_1680 histidine utilization repressor       K     231     0.775    231 
bmv:BMASAVP1_A2365 histidine utilization repressor      K     231     0.775    231 
bpd:BURPS668_2666 histidine utilization repressor       K     231     0.775    231 
bpl:BURPS1106A_2722 histidine utilization repressor     K     231     0.775    231 
bpr:GBP346_A2845 histidine utilization repressor        K     231     0.775    231 
bps:BPSL2343 histidine utilization repressor            K     231     0.775    231 
bpm:BURPS1710b_2795 histidine utilization repressor     K     231     0.771    231 
bte:BTH_I1821 histidine utilization repressor           K     231     0.762    231 
rpi:Rpic_2882 transcriptional regulator, histidine util K     237     0.596    230 
rsl:RPSI07_0869 GntR family transcriptional regulator   K     237     0.594    229 
rso:RSc2648 histidine utilization repressor transcripti K     237     0.588    228 
rpf:Rpic12D_2476 GntR family transcriptional regulator  K     237     0.591    230 
rsc:RCFBP_10799 GntR family transcriptional regulator   K     237     0.585    229 
ctt:CtCNB1_0165 transcriptional regulator, histidine    K     238     0.583    228 
ddd:Dda3937_01666 histidine utilization repressor       K     285     0.520    229 
ddc:Dd586_2950 GntR family transcriptional regulator    K     249     0.520    229 
dze:Dd1591_1261 GntR family transcriptional regulator   K     247     0.515    229 
dda:Dd703_2647 GntR family transcriptional regulator    K     251     0.498    229 
sen:SACE_3198 histidine utilization genes repressor pro K     246     0.502    229 
xne:XNC1_3188 Histidine utilization repressor           K     244     0.487    228 
pva:Pvag_0646 histidine utilization repressor           K     251     0.485    229 
plu:plu3195 histidine utilization repressor protein     K     243     0.474    228 
axy:AXYL_00282 histidine utilization repressor          K     247     0.480    229 
pap:PSPA7_5838 histidine utilization repressor          K     250     0.489    229 
ent:Ent638_1262 GntR family transcriptional regulator   K     244     0.515    229 
pam:PANA_1266 HutC                                      K     272     0.472    229 
pae:PA5105 histidine utilization repressor HutC         K     250     0.485    229 
pag:PLES_54951 histidine utilization repressor HutC     K     250     0.485    229 
pau:PA14_67420 histidine utilization genes repressor pr K     250     0.485    229 
rer:RER_51240 putative GntR family transcriptional regu K     276     0.483    230 
kpe:KPK_3779 histidine utilization repressor            K     241     0.489    229 
kva:Kvar_3588 GntR family transcriptional regulator     K     241     0.489    229 
azl:AZL_c00100 transcriptional regulator                K     242     0.513    228 
reu:Reut_A0895 GntR family transcriptional regulator    K     251     0.485    231 
pfl:PFL_0399 histidine utilization repressor            K     249     0.485    229 
pst:PSPTO_5172 histidine utilization repressor          K     249     0.498    229 
psp:PSPPH_0349 histidine utilization repressor          K     249     0.498    229 
eam:EAMY_1256 histidine utilization repressor           K     272     0.476    229 
eay:EAM_1255 histidine utilization repressor            K     251     0.476    229 
psb:Psyr_0366 histidine utilization repressor           K     273     0.493    229 
req:REQ_41890 GntR family transcriptional regulator     K     251     0.474    230 
cko:CKO_02358 hypothetical protein                      K     239     0.491    228 
rha:RHA1_ro04645 histidine utilization repressor        K     255     0.487    230 
eta:ETA_22230 GntR family transcriptional regulator     K     251     0.467    229 
pfs:PFLU0359 GntR family transcriptional regulator      K     249     0.489    229 
rop:ROP_47420 GntR family transcriptional regulator     K     253     0.487    230 
pay:PAU_01422 hutc protein (putative gntr-family transc K     243     0.469    228 
nfa:nfa12250 transcriptional regulator                  K     251     0.489    229 
ebi:EbC_14200 GntR family transcriptional regulator     K     251     0.480    229 
cti:RALTA_A0655 GntR family transcriptional regulator   K     240     0.481    231 
ypb:YPTS_2022 histidine utilization repressor           K     255     0.467    229 
yps:YPTB1967 GntR family transcriptional regulator      K     255     0.467    229 
ypy:YPK_2221 histidine utilization repressor            K     255     0.467    229 
enc:ECL_02966 GntR family transcriptional regulator     K     244     0.485    229 
vch:VC1206 histidine utilization repressor              K     236     0.496    228 
vcm:VCM66_1161 histidine utilization repressor          K     236     0.496    228 
vco:VC0395_A0826 histidine utilization repressor        K     236     0.496    228 
spl:Spea_4170 histidine utilization repressor           K     234     0.489    231 
yen:YE2460 GntR family transcriptional regulator        K     255     0.463    229 
ypa:YPA_1355 GntR family transcriptional regulator      K     255     0.467    229 
ype:YPO1973 GntR family transcriptional regulator       K     255     0.467    229 
ypg:YpAngola_A2511 histidine utilization repressor      K     255     0.467    229 
ypi:YpsIP31758_2112 histidine utilization repressor     K     255     0.467    229 
ypk:y2339 repressor                                     K     255     0.467    229 
ypn:YPN_1453 GntR family transcriptional regulator      K     255     0.467    229 
ypp:YPDSF_1150 GntR family transcriptional regulator    K     255     0.467    229 
ypz:YPZ3_1845 GntR family transcriptional regulator     K     255     0.467    229 
swd:Swoo_4839 histidine utilization repressor           K     233     0.481    231 
shn:Shewana3_0099 histidine utilization repressor       K     235     0.476    231 
she:Shewmr4_0098 histidine utilization repressor        K     235     0.476    231 
shm:Shewmr7_0093 histidine utilization repressor        K     235     0.476    231 
slo:Shew_3758 histidine utilization repressor           K     235     0.485    231 
svo:SVI_4276 histidine utilization repressor            K     233     0.481    231 
shl:Shal_0072 histidine utilization repressor           K     234     0.485    231 
xbo:XBJ1_2986 histidine utilization repressor           K     243     0.456    228 
son:SO_0096 transcriptional repressor of histidine util K     235     0.476    231 
vex:VEA_003719 histidine utilization repressor          K     235     0.470    230 
vsa:VSAL_II0704 histidine utilization repressor         K     234     0.461    230 
sdn:Sden_0082 histidine utilization repressor           K     235     0.450    231 
sse:Ssed_4448 histidine utilization repressor           K     233     0.468    231 
swp:swp_0132 histidine utilization repressor            K     234     0.481    231 
hch:HCH_04168 histidine utilization repressor           K     235     0.461    230 
vfm:VFMJ11_A0504 histidine utilization repressor        K     233     0.439    228
```

**Neighborhood Representations**: Table of genes in the defined genetic neighborhoods of the entry protein and its closest homologs  
  
**Neighborhood Representations for "bxe:Bxe\_A2946"**  

| ID | Annotation | EC number |
| --- | --- | --- |
| bxe:Bxe\_A2956 | LysR family transcriptional regulator |  |
| bxe:Bxe\_A2955 | peptidase M20D, amidohydrolase; K01451 hippurate hydrolase [EC:3.5.1.32] | ec:3.5.1.32 |
| bxe:Bxe\_A2954 | major facilitator transporter |  |
| bxe:Bxe\_A2953 | major facilitator transporter |  |
| bxe:Bxe\_A2952 | hypothetical protein |  |
| bxe:Bxe\_A2951 | nucleobase/cation symporter, (NCS2) family; K06901 putative MFS transporter, AGZA family, xanthine/uracil permease |  |
| bxe:Bxe\_A2950 | hypothetical protein |  |
| bxe:Bxe\_A2949 | NAD dependent epimerase/dehydratase |  |
| bxe:Bxe\_A2948 | LysR family transcriptional regulator |  |
| bxe:Bxe\_A2947 | histidine ammonia-lyase (EC:4.3.1.3); K01745 histidine ammonia-lyase [EC:4.3.1.3] | ec:4.3.1.3 |
| bxe:Bxe\_A2946 | histidine utilization repressor; K05836 GntR family transcriptional regulator, histidine utilization repressor |  |
| bxe:Bxe\_A2945 | urocanate hydratase (EC:4.2.1.49); K01712 urocanate hydratase [EC:4.2.1.49] | ec:4.2.1.49 |
| bxe:Bxe\_A2944 | hypothetical protein; K09975 hypothetical protein |  |
| bxe:Bxe\_A2943 | imidazolonepropionase (EC:3.5.2.7); K01468 imidazolonepropionase [EC:3.5.2.7] | ec:3.5.2.7 |
| bxe:Bxe\_A2942 | N-formimino-L-glutamate deiminase (EC:3.5.3.13); K05603 formimidoylglutamate deiminase [EC:3.5.3.13] | ec:3.5.3.13 |
| bxe:Bxe\_A2941 | N-formylglutamate deformylase (EC:3.5.1.68); K01458 N-formylglutamate deformylase [EC:3.5.1.68] | ec:3.5.1.68 |
| bxe:Bxe\_A2940 | hypothetical protein |  |
| bxe:Bxe\_A2939 | transmembrane protein |  |
| bxe:Bxe\_A2938 | branched chain amino acid ABC transporter periplasmic ligand-binding protein; K01999 branched-chain amino acid transport system substrate-binding protein |  |
| bxe:Bxe\_A2937 | hypothetical protein |  |
| bxe:Bxe\_A2936 | pyridoxal kinase (EC:2.7.1.35); K00868 pyridoxine kinase [EC:2.7.1.35] | ec:2.7.1.35 |

  
**Neighborhood Representations for "bpy:Bphyt\_1531"**  

| ID | Annotation | EC number |
| --- | --- | --- |
| bpy:Bphyt\_1521 | major facilitator superfamily protein |  |
| bpy:Bphyt\_1522 | hypothetical protein |  |
| bpy:Bphyt\_1523 | xanthine/uracil/vitamin C permease; K06901 putative MFS transporter, AGZA family, xanthine/uracil permease |  |
| bpy:Bphyt\_1524 | pseudogene |  |
| bpy:Bphyt\_1525 | activator of Hsp90 ATPase 1 family protein |  |
| bpy:Bphyt\_1526 | hypothetical protein |  |
| bpy:Bphyt\_1527 | hypothetical protein |  |
| bpy:Bphyt\_1528 | NAD-dependent epimerase/dehydratase |  |
| bpy:Bphyt\_1529 | LysR family transcriptional regulator |  |
| bpy:Bphyt\_1530 | histidine ammonia-lyase (EC:4.3.1.3); K01745 histidine ammonia-lyase [EC:4.3.1.3] | ec:4.3.1.3 |
| bpy:Bphyt\_1531 | transcriptional regulator, histidine utilization repressor, GntR family; K05836 GntR family transcriptional regulator, histidine utilization repressor |  |
| bpy:Bphyt\_1532 | urocanate hydratase (EC:4.2.1.49); K01712 urocanate hydratase [EC:4.2.1.49] | ec:4.2.1.49 |
| bpy:Bphyt\_1533 | hypothetical protein; K09975 hypothetical protein |  |
| bpy:Bphyt\_1534 | imidazolonepropionase (EC:3.5.2.7); K01468 imidazolonepropionase [EC:3.5.2.7] | ec:3.5.2.7 |
| bpy:Bphyt\_1535 | N-formimino-L-glutamate deiminase; K05603 formimidoylglutamate deiminase [EC:3.5.3.13] | ec:3.5.3.13 |
| bpy:Bphyt\_1536 | N-formylglutamate amidohydrolase; K01458 N-formylglutamate deformylase [EC:3.5.1.68] | ec:3.5.1.68 |
| bpy:Bphyt\_1537 | hypothetical protein |  |
| bpy:Bphyt\_1538 | transmembrane protein |  |
| bpy:Bphyt\_1539 | branched chain amino acid ABC transporter substrate-binding protein; K01999 branched-chain amino acid transport system substrate-binding protein |  |
| bpy:Bphyt\_1540 | hypothetical protein |  |
| bpy:Bphyt\_1541 | pyridoxal kinase (EC:2.7.1.35); K00868 pyridoxine kinase [EC:2.7.1.35] | ec:2.7.1.35 |

  
**Neighborhood Representations for "bgf:BC1003\_2185"**  

| ID | Annotation | EC number |
| --- | --- | --- |
| bgf:BC1003\_2175 | pyridoxal kinase (EC:2.7.1.35); K00868 pyridoxine kinase [EC:2.7.1.35] | ec:2.7.1.35 |
| bgf:BC1003\_2176 | hypothetical protein |  |
| bgf:BC1003\_2177 | extracellular ligand-binding receptor; K01999 branched-chain amino acid transport system substrate-binding protein |  |
| bgf:BC1003\_2178 | transglutaminase domain-containing protein |  |
| bgf:BC1003\_2179 | hypothetical protein |  |
| bgf:BC1003\_2180 | N-formylglutamate amidohydrolase; K01458 N-formylglutamate deformylase [EC:3.5.1.68] | ec:3.5.1.68 |
| bgf:BC1003\_2181 | formiminoglutamate deiminase; K05603 formimidoylglutamate deiminase [EC:3.5.3.13] | ec:3.5.3.13 |
| bgf:BC1003\_2182 | imidazolonepropionase (EC:3.5.2.7); K01468 imidazolonepropionase [EC:3.5.2.7] | ec:3.5.2.7 |
| bgf:BC1003\_2183 | hypothetical protein; K09975 hypothetical protein |  |
| bgf:BC1003\_2184 | urocanate hydratase (EC:4.2.1.49); K01712 urocanate hydratase [EC:4.2.1.49] | ec:4.2.1.49 |
| bgf:BC1003\_2185 | GntR family transcriptional regulator; K05836 GntR family transcriptional regulator, histidine utilization repressor |  |
| bgf:BC1003\_2186 | histidine ammonia-lyase (EC:4.3.1.3); K01745 histidine ammonia-lyase [EC:4.3.1.3] | ec:4.3.1.3 |
| bgf:BC1003\_2187 | LysR family transcriptional regulator |  |
| bgf:BC1003\_2188 | NmrA family protein |  |
| bgf:BC1003\_2189 | hypothetical protein |  |
| bgf:BC1003\_2190 | hypothetical protein |  |
| bgf:BC1003\_2191 | xanthine/uracil/vitamin C permease; K06901 putative MFS transporter, AGZA family, xanthine/uracil permease |  |
| bgf:BC1003\_2192 | PRC-barrel domain-containing protein |  |
| bgf:BC1003\_2193 | major facilitator superfamily protein |  |
| bgf:BC1003\_2194 | LysR family transcriptional regulator; K05596 LysR family transcriptional regulator, chromosome initiation inhibitor |  |
| bgf:BC1003\_2195 | lysine exporter protein LysE/YggA; K06895 L-lysine exporter family protein LysE/ArgO |  |

  
**Neighborhood Representations for "bge:BC1002\_1092"**  

| ID | Annotation | EC number |
| --- | --- | --- |
| bge:BC1002\_1082 | cytochrome c oxidase subunit II; K02275 cytochrome c oxidase subunit II [EC:1.9.3.1] | ec:1.9.3.1 |
| bge:BC1002\_1083 | hypothetical protein |  |
| bge:BC1002\_1084 | major facilitator superfamily protein |  |
| bge:BC1002\_1085 | hypothetical protein |  |
| bge:BC1002\_1086 | xanthine/uracil/vitamin C permease; K06901 putative MFS transporter, AGZA family, xanthine/uracil permease |  |
| bge:BC1002\_1087 | hypothetical protein |  |
| bge:BC1002\_1088 | hypothetical protein |  |
| bge:BC1002\_1089 | NmrA family protein |  |
| bge:BC1002\_1090 | transcriptional regulator, LysR family |  |
| bge:BC1002\_1091 | histidine ammonia-lyase (EC:4.3.1.3); K01745 histidine ammonia-lyase [EC:4.3.1.3] | ec:4.3.1.3 |
| bge:BC1002\_1092 | GntR family transcriptional regulator; K05836 GntR family transcriptional regulator, histidine utilization repressor |  |
| bge:BC1002\_1093 | urocanate hydratase (EC:4.2.1.49); K01712 urocanate hydratase [EC:4.2.1.49] | ec:4.2.1.49 |
| bge:BC1002\_1094 | hypothetical protein; K09975 hypothetical protein |  |
| bge:BC1002\_1095 | imidazolonepropionase (EC:3.5.2.7); K01468 imidazolonepropionase [EC:3.5.2.7] | ec:3.5.2.7 |
| bge:BC1002\_1096 | formiminoglutamate deiminase; K05603 formimidoylglutamate deiminase [EC:3.5.3.13] | ec:3.5.3.13 |
| bge:BC1002\_1097 | N-formylglutamate amidohydrolase; K01458 N-formylglutamate deformylase [EC:3.5.1.68] | ec:3.5.1.68 |
| bge:BC1002\_1098 | hypothetical protein |  |
| bge:BC1002\_1099 | hypothetical protein |  |
| bge:BC1002\_1100 | transglutaminase domain protein |  |
| bge:BC1002\_1101 | branched-chain amino acid ABC transporter substrate-binding protein; K01999 branched-chain amino acid transport system substrate-binding protein |  |
| bge:BC1002\_1102 | hypothetical protein |  |

  
**Neighborhood Representations for "bph:Bphy\_1813"**  

| ID | Annotation | EC number |
| --- | --- | --- |
| bph:Bphy\_1803 | hypothetical protein |  |
| bph:Bphy\_1804 | major facilitator transporter |  |
| bph:Bphy\_1805 | GntR family transcriptional regulator |  |
| bph:Bphy\_1806 | LysR family transcriptional regulator |  |
| bph:Bphy\_1807 | hypothetical protein |  |
| bph:Bphy\_1808 | N-formylglutamate amidohydrolase; K01458 N-formylglutamate deformylase [EC:3.5.1.68] | ec:3.5.1.68 |
| bph:Bphy\_1809 | N-formimino-L-glutamate deiminase; K05603 formimidoylglutamate deiminase [EC:3.5.3.13] | ec:3.5.3.13 |
| bph:Bphy\_1810 | imidazolonepropionase (EC:3.5.2.7); K01468 imidazolonepropionase [EC:3.5.2.7] | ec:3.5.2.7 |
| bph:Bphy\_1811 | hypothetical protein; K09975 hypothetical protein |  |
| bph:Bphy\_1812 | urocanate hydratase (EC:4.2.1.49); K01712 urocanate hydratase [EC:4.2.1.49] | ec:4.2.1.49 |
| bph:Bphy\_1813 | histidine utilization repressor; K05836 GntR family transcriptional regulator, histidine utilization repressor |  |
| bph:Bphy\_1814 | histidine ammonia-lyase (EC:4.3.1.3); K01745 histidine ammonia-lyase [EC:4.3.1.3] | ec:4.3.1.3 |
| bph:Bphy\_1815 | hypothetical protein |  |
| bph:Bphy\_1816 | NAD-dependent epimerase/dehydratase |  |
| bph:Bphy\_1817 | hypothetical protein |  |
| bph:Bphy\_1818 | hypothetical protein |  |
| bph:Bphy\_1819 | activator of Hsp90 ATPase 1 family protein |  |
| bph:Bphy\_1820 | pseudogene |  |
| bph:Bphy\_1821 | xanthine/uracil/vitamin C permease; K06901 putative MFS transporter, AGZA family, xanthine/uracil permease |  |
| bph:Bphy\_1822 | hypothetical protein |  |
| bph:Bphy\_1823 | major facilitator transporter |  |

  
**Neighborhood Representations for "bvi:Bcep1808\_4120"**  

| ID | Annotation | EC number |
| --- | --- | --- |
| bvi:Bcep1808\_4110 | hypothetical protein |  |
| bvi:Bcep1808\_4111 | hypothetical protein |  |
| bvi:Bcep1808\_4112 | argininosuccinate synthase (EC:6.3.4.5); K01940 argininosuccinate synthase [EC:6.3.4.5] | ec:6.3.4.5 |
| bvi:Bcep1808\_4113 | membrane protein |  |
| bvi:Bcep1808\_4114 | diguanylate cyclase |  |
| bvi:Bcep1808\_4115 | amino acid permease; K11733 lysine-specific permease |  |
| bvi:Bcep1808\_4116 | hypothetical protein |  |
| bvi:Bcep1808\_4117 | LysR family transcriptional regulator |  |
| bvi:Bcep1808\_4118 | alpha/beta hydrolase |  |
| bvi:Bcep1808\_4119 | hypothetical protein |  |
| bvi:Bcep1808\_4120 | histidine utilization repressor; K05836 GntR family transcriptional regulator, histidine utilization repressor |  |
| bvi:Bcep1808\_4121 | hypothetical protein |  |
| bvi:Bcep1808\_4122 | hypothetical protein |  |
| bvi:Bcep1808\_4123 | transposase IS3/IS911 family protein; K07483 transposase |  |
| bvi:Bcep1808\_4124 | integrase catalytic subunit; K07497 putative transposase |  |
| bvi:Bcep1808\_4125 | integrase catalytic subunit; K07497 putative transposase |  |
| bvi:Bcep1808\_4126 | transposase, mutator type |  |
| bvi:Bcep1808\_4127 | hypothetical protein |  |
| bvi:Bcep1808\_4128 | hypothetical protein |  |
| bvi:Bcep1808\_4129 | methyltransferase type 11 |  |
| bvi:Bcep1808\_4130 | transposase, IS4 family protein |  |

  
**Neighborhood Representations for "bch:Bcen2424\_3722"**  

| ID | Annotation | EC number |
| --- | --- | --- |
| bch:Bcen2424\_3712 | argininosuccinate synthase (EC:6.3.4.5); K01940 argininosuccinate synthase [EC:6.3.4.5] | ec:6.3.4.5 |
| bch:Bcen2424\_3713 | membrane protein |  |
| bch:Bcen2424\_3714 | diguanylate cyclase |  |
| bch:Bcen2424\_3715 | amino acid permease-associated region; K11733 lysine-specific permease |  |
| bch:Bcen2424\_3716 | hypothetical protein |  |
| bch:Bcen2424\_3717 | LysR family transcriptional regulator |  |
| bch:Bcen2424\_3718 | alpha/beta hydrolase |  |
| bch:Bcen2424\_3719 | hypothetical protein |  |
| bch:Bcen2424\_3720 | TetR family transcriptional regulator |  |
| bch:Bcen2424\_3721 | glycosyl transferase family protein |  |
| bch:Bcen2424\_3722 | histidine utilization repressor; K05836 GntR family transcriptional regulator, histidine utilization repressor |  |
| bch:Bcen2424\_3723 | porin |  |
| bch:Bcen2424\_3724 | hypothetical protein |  |
| bch:Bcen2424\_3725 | polar amino acid ABC transporter inner membrane subunit; K10016 histidine transport system permease protein |  |
| bch:Bcen2424\_3726 | polar amino acid ABC transporter inner membrane subunit; K10015 histidine transport system permease protein |  |
| bch:Bcen2424\_3727 | ABC transporter; K10017 histidine transport system ATP-binding protein [EC:3.6.3.21] | ec:3.6.3.21 |
| bch:Bcen2424\_3728 | cupin |  |
| bch:Bcen2424\_3729 | hypothetical protein |  |
| bch:Bcen2424\_3730 | hypothetical protein |  |
| bch:Bcen2424\_3731 | polyhydroxyalkanoate depolymerase |  |
| bch:Bcen2424\_3732 | glutathione S-transferase domain-containing protein; K04097 glutathione S-transferase [EC:2.5.1.18] | ec:2.5.1.18 |

  
**Neighborhood Representations for "bcn:Bcen\_4641"**  

| ID | Annotation | EC number |
| --- | --- | --- |
| bcn:Bcen\_4631 | cyclic nucleotide-binding protein |  |
| bcn:Bcen\_4632 | glutathione S-transferase; K04097 glutathione S-transferase [EC:2.5.1.18] | ec:2.5.1.18 |
| bcn:Bcen\_4633 | polyhydroxyalkanoate depolymerase |  |
| bcn:Bcen\_4634 | hypothetical protein |  |
| bcn:Bcen\_4635 | cupin |  |
| bcn:Bcen\_4636 | ABC transporter; K10017 histidine transport system ATP-binding protein [EC:3.6.3.21] | ec:3.6.3.21 |
| bcn:Bcen\_4637 | amino acid ABC transporter permease; K10015 histidine transport system permease protein |  |
| bcn:Bcen\_4638 | amino acid ABC transporter permease; K10016 histidine transport system permease protein |  |
| bcn:Bcen\_4639 | hypothetical protein |  |
| bcn:Bcen\_4640 | porin |  |
| bcn:Bcen\_4641 | histidine utilization repressor; K05836 GntR family transcriptional regulator, histidine utilization repressor |  |
| bcn:Bcen\_4642 | glycosyl transferase family protein |  |
| bcn:Bcen\_4643 | TetR family transcriptional regulator |  |
| bcn:Bcen\_4644 | hypothetical protein |  |
| bcn:Bcen\_4645 | alpha/beta hydrolase |  |
| bcn:Bcen\_4646 | LysR family transcriptional regulator |  |
| bcn:Bcen\_4647 | hypothetical protein |  |
| bcn:Bcen\_4648 | amino acid permease-associated region; K11733 lysine-specific permease |  |
| bcn:Bcen\_4649 | diguanylate cyclase |  |
| bcn:Bcen\_4650 | membrane protein |  |
| bcn:Bcen\_4651 | argininosuccinate synthase (EC:6.3.4.5); K01940 argininosuccinate synthase [EC:6.3.4.5] | ec:6.3.4.5 |

  
**Neighborhood Representations for "bcj:BCAM0756"**  

| ID | Annotation | EC number |
| --- | --- | --- |
| bcj:BCAM0746 | argG; argininosuccinate synthase (EC:6.3.4.5); K01940 argininosuccinate synthase [EC:6.3.4.5] | ec:6.3.4.5 |
| bcj:BCAM0747 | hypothetical protein |  |
| bcj:BCAM0748 | putative diguanylate cyclase |  |
| bcj:BCAM0749 | lysP; lysine-specific permease; K11733 lysine-specific permease |  |
| bcj:BCAM0750 | hypothetical protein |  |
| bcj:BCAM0751 | LysR family regulatory protein |  |
| bcj:BCAM0752 | putative hydrolase |  |
| bcj:BCAM0753 | hypothetical protein |  |
| bcj:BCAM0754 | TetR family regulatory protein |  |
| bcj:BCAM0755 | putative glycosyltransferase |  |
| bcj:BCAM0756 | GntR family regulatory protein; K05836 GntR family transcriptional regulator, histidine utilization repressor |  |
| bcj:BCAM0757 | putative porin |  |
| bcj:BCAM0758 | hypothetical protein |  |
| bcj:BCAM0759 | argT; periplasmic lysine-arginine-ornithine-binding protein; K10014 histidine transport system substrate-binding protein |  |
| bcj:BCAM0760 | hisQ; histidine transport system permease; K10016 histidine transport system permease protein |  |
| bcj:BCAM0761 | hisM; histidine transport system permease; K10015 histidine transport system permease protein |  |
| bcj:BCAM0762 | hisP; histidine ABC transporter ATP-binding protein; K10017 histidine transport system ATP-binding protein [EC:3.6.3.21] | ec:3.6.3.21 |
| bcj:BCAM0763 | putative carbohydrate-binding lipoprotein |  |
| bcj:BCAM0764 | LacI family regulatory protein; K02529 LacI family transcriptional regulator |  |
| bcj:BCAM0765 | aldose epimerase family protein |  |
| bcj:BCAM0766 | D-ribose-binding periplasmic protein precursor; K10439 ribose transport system substrate-binding protein |  |

  
**Neighborhood Representations for "bcm:Bcenmc03\_3799"**  

| ID | Annotation | EC number |
| --- | --- | --- |
| bcm:Bcenmc03\_3789 | glutathione S-transferase domain-containing protein; K04097 glutathione S-transferase [EC:2.5.1.18] | ec:2.5.1.18 |
| bcm:Bcenmc03\_3790 | polyhydroxyalkanoate depolymerase, intracellular |  |
| bcm:Bcenmc03\_3791 | hypothetical protein |  |
| bcm:Bcenmc03\_3792 | hypothetical protein |  |
| bcm:Bcenmc03\_3793 | cupin 2 domain-containing protein |  |
| bcm:Bcenmc03\_3794 | ABC transporter-like protein; K10017 histidine transport system ATP-binding protein [EC:3.6.3.21] | ec:3.6.3.21 |
| bcm:Bcenmc03\_3795 | polar amino acid ABC transporter inner membrane subunit; K10015 histidine transport system permease protein |  |
| bcm:Bcenmc03\_3796 | polar amino acid ABC transporter inner membrane subunit; K10016 histidine transport system permease protein |  |
| bcm:Bcenmc03\_3797 | hypothetical protein |  |
| bcm:Bcenmc03\_3798 | porin |  |
| bcm:Bcenmc03\_3799 | histidine utilization repressor; K05836 GntR family transcriptional regulator, histidine utilization repressor |  |
| bcm:Bcenmc03\_3800 | sterol 3-beta-glucosyltransferase (EC:2.4.1.173); K05841 sterol 3beta-glucosyltransferase [EC:2.4.1.173] | ec:2.4.1.173 |
| bcm:Bcenmc03\_3801 | TetR family transcriptional regulator |  |
| bcm:Bcenmc03\_3802 | hypothetical protein |  |
| bcm:Bcenmc03\_3803 | alpha/beta hydrolase fold protein |  |
| bcm:Bcenmc03\_3804 | LysR family transcriptional regulator |  |
| bcm:Bcenmc03\_3805 | hypothetical protein |  |
| bcm:Bcenmc03\_3806 | amino acid permease-associated protein; K11733 lysine-specific permease |  |
| bcm:Bcenmc03\_3807 | diguanylate cyclase |  |
| bcm:Bcenmc03\_3808 | membrane protein |  |
| bcm:Bcenmc03\_3809 | argininosuccinate synthase (EC:6.3.4.5); K01940 argininosuccinate synthase [EC:6.3.4.5] | ec:6.3.4.5 |

  
**Neighborhood Representations for "bam:Bamb\_5462"**  

| ID | Annotation | EC number |
| --- | --- | --- |
| bam:Bamb\_5452 | argininosuccinate synthase (EC:6.3.4.5); K01940 argininosuccinate synthase [EC:6.3.4.5] | ec:6.3.4.5 |
| bam:Bamb\_5453 | membrane protein |  |
| bam:Bamb\_5454 | diguanylate cyclase |  |
| bam:Bamb\_5455 | amino acid permease-associated region; K11733 lysine-specific permease |  |
| bam:Bamb\_5456 | hypothetical protein |  |
| bam:Bamb\_5457 | LysR family transcriptional regulator |  |
| bam:Bamb\_5458 | alpha/beta hydrolase |  |
| bam:Bamb\_5459 | hypothetical protein |  |
| bam:Bamb\_5460 | TetR family transcriptional regulator |  |
| bam:Bamb\_5461 | glycosyl transferase family protein |  |
| bam:Bamb\_5462 | histidine utilization repressor; K05836 GntR family transcriptional regulator, histidine utilization repressor |  |
| bam:Bamb\_5463 | cationic amino acid ABC transporter, periplasmic binding protein; K10014 histidine transport system substrate-binding protein |  |
| bam:Bamb\_5464 | polar amino acid ABC transporter inner membrane subunit; K10016 histidine transport system permease protein |  |
| bam:Bamb\_5465 | polar amino acid ABC transporter inner membrane subunit; K10015 histidine transport system permease protein |  |
| bam:Bamb\_5466 | ABC transporter; K10017 histidine transport system ATP-binding protein [EC:3.6.3.21] | ec:3.6.3.21 |
| bam:Bamb\_5467 | hypothetical protein |  |
| bam:Bamb\_5468 | cyclic nucleotide-binding protein |  |
| bam:Bamb\_5469 | MotA/TolQ/ExbB proton channel; K02556 chemotaxis protein MotA |  |
| bam:Bamb\_5470 | OmpA/MotB domain-containing protein |  |
| bam:Bamb\_5471 | methyl-accepting chemotaxis sensory transducer; K03406 methyl-accepting chemotaxis protein |  |
| bam:Bamb\_5472 | hypothetical protein |  |

  
**Neighborhood Representations for "bmj:BMULJ\_03508"**  

| ID | Annotation | EC number |
| --- | --- | --- |
| bmj:BMULJ\_03498 | hypothetical protein |  |
| bmj:BMULJ\_03499 | argG; argininosuccinate synthase (EC:6.3.4.5); K01940 argininosuccinate synthase [EC:6.3.4.5] | ec:6.3.4.5 |
| bmj:BMULJ\_03500 | hypothetical protein |  |
| bmj:BMULJ\_03501 | diguanylate cyclase |  |
| bmj:BMULJ\_03502 | AAT family amino acid transporter; K11733 lysine-specific permease |  |
| bmj:BMULJ\_03503 | hypothetical protein |  |
| bmj:BMULJ\_03504 | pip; proline iminopeptidase (EC:3.4.11.5) |  |
| bmj:BMULJ\_03505 | hypothetical protein |  |
| bmj:BMULJ\_03506 | TetR family transcriptional regulator |  |
| bmj:BMULJ\_03507 | glycosyl transferase |  |
| bmj:BMULJ\_03508 | hutC; GntR family transcriptional regulator; K05836 GntR family transcriptional regulator, histidine utilization repressor |  |
| bmj:BMULJ\_03509 | polar amino acid transporter substrate-binding protein; K10014 histidine transport system substrate-binding protein |  |
| bmj:BMULJ\_03510 | polar amino acid transporter permease; K10016 histidine transport system permease protein |  |
| bmj:BMULJ\_03511 | polar amino acid transporter permease; K10015 histidine transport system permease protein |  |
| bmj:BMULJ\_03512 | polar amino acid transporter ATP-binding protein; K10017 histidine transport system ATP-binding protein [EC:3.6.3.21] | ec:3.6.3.21 |
| bmj:BMULJ\_03513 | hypothetical protein |  |
| bmj:BMULJ\_03514 | cAMP-binding protein |  |
| bmj:BMULJ\_03515 | motA; chemotaxis protein MotA; K02556 chemotaxis protein MotA |  |
| bmj:BMULJ\_03516 | motB; chemotaxis protein MotB; K02557 chemotaxis protein MotB |  |
| bmj:BMULJ\_03517 | methyl-accepting chemotaxis protein; K03406 methyl-accepting chemotaxis protein |  |
| bmj:BMULJ\_03518 | threonine efflux protein |  |

  
**Neighborhood Representations for "bmu:Bmul\_5005"**  

| ID | Annotation | EC number |
| --- | --- | --- |
| bmu:Bmul\_4995 | LysR family transcriptional regulator |  |
| bmu:Bmul\_4996 | lysine exporter protein LysE/YggA |  |
| bmu:Bmul\_4997 | methyl-accepting chemotaxis sensory transducer; K03406 methyl-accepting chemotaxis protein |  |
| bmu:Bmul\_4998 | OmpA/MotB domain-containing protein; K02557 chemotaxis protein MotB |  |
| bmu:Bmul\_4999 | MotA/TolQ/ExbB proton channel; K02556 chemotaxis protein MotA |  |
| bmu:Bmul\_5000 | cyclic nucleotide-binding protein |  |
| bmu:Bmul\_5001 | ABC transporter; K10017 histidine transport system ATP-binding protein [EC:3.6.3.21] | ec:3.6.3.21 |
| bmu:Bmul\_5002 | polar amino acid ABC transporter inner membrane subunit; K10015 histidine transport system permease protein |  |
| bmu:Bmul\_5003 | polar amino acid ABC transporter, inner membrane subunit; K10016 histidine transport system permease protein |  |
| bmu:Bmul\_5004 | cationic amino acid ABC transporter, periplasmic binding protein; K10014 histidine transport system substrate-binding protein |  |
| bmu:Bmul\_5005 | histidine utilization repressor; K05836 GntR family transcriptional regulator, histidine utilization repressor |  |
| bmu:Bmul\_5006 | sterol 3-beta-glucosyltransferase (EC:2.4.1.173); K05841 sterol 3beta-glucosyltransferase [EC:2.4.1.173] | ec:2.4.1.173 |
| bmu:Bmul\_5007 | TetR family transcriptional regulator |  |
| bmu:Bmul\_5008 | hypothetical protein |  |
| bmu:Bmul\_5009 | alpha/beta hydrolase |  |
| bmu:Bmul\_5010 | hypothetical protein |  |
| bmu:Bmul\_5011 | amino acid permease; K11733 lysine-specific permease |  |
| bmu:Bmul\_5012 | diguanylate cyclase |  |
| bmu:Bmul\_5013 | membrane protein |  |
| bmu:Bmul\_5014 | argininosuccinate synthase (EC:6.3.4.5); K01940 argininosuccinate synthase [EC:6.3.4.5] | ec:6.3.4.5 |
| bmu:Bmul\_5015 | hypothetical protein |  |

  
**Neighborhood Representations for "bur:Bcep18194\_B2386"**  

| ID | Annotation | EC number |
| --- | --- | --- |
| bur:Bcep18194\_B2376 | ABC His/Glu/Gln/Arg/opine transporter, inner membrane subunit; K10015 histidine transport system permease protein |  |
| bur:Bcep18194\_B2377 | ABC His/Glu/Gln/Arg/opine transporter, inner membrane subunit; K10016 histidine transport system permease protein |  |
| bur:Bcep18194\_B2378 | lysine/arginine/ornithine ABC transporter periplasmic-binding protein; K10014 histidine transport system substrate-binding protein |  |
| bur:Bcep18194\_B2379 | two component transcriptional regulator; K07667 two-component system, OmpR family, KDP operon response regulator KdpE |  |
| bur:Bcep18194\_B2380 | periplasmic sensor signal transduction histidine kinase; K07646 two-component system, OmpR family, sensor histidine kinase KdpD [EC:2.7.13.3] | ec:2.7.13.3 |
| bur:Bcep18194\_B2381 | potassium-transporting ATPase subunit B (EC:3.6.3.4); K01547 K+-transporting ATPase ATPase B chain [EC:3.6.3.12] | ec:3.6.3.12 |
| bur:Bcep18194\_B2382 | potassium-transporting ATPase (EC:3.6.3.12); K01548 K+-transporting ATPase ATPase C chain [EC:3.6.3.12] | ec:3.6.3.12 |
| bur:Bcep18194\_B2383 | potassium-transporting ATPase subunit A (EC:3.6.3.12); K01546 K+-transporting ATPase ATPase A chain [EC:3.6.3.12] | ec:3.6.3.12 |
| bur:Bcep18194\_B2384 | hypothetical protein |  |
| bur:Bcep18194\_B2385 | porin |  |
| bur:Bcep18194\_B2386 | histidine utilization repressor; K05836 GntR family transcriptional regulator, histidine utilization repressor |  |
| bur:Bcep18194\_B2387 | glycosyl transferase |  |
| bur:Bcep18194\_B2388 | TetR family transcriptional regulator |  |
| bur:Bcep18194\_B2389 | TetR family transcriptional regulator |  |
| bur:Bcep18194\_B2390 | hypothetical protein |  |
| bur:Bcep18194\_B2391 | hypothetical protein |  |
| bur:Bcep18194\_B2392 | hypothetical protein |  |
| bur:Bcep18194\_B2393 | alpha/beta hydrolase (EC:3.4.11.5) |  |
| bur:Bcep18194\_B2394 | LysR family transcriptional regulator |  |
| bur:Bcep18194\_B2395 | hypothetical protein |  |
| bur:Bcep18194\_B2396 | amino acid transporter; K11733 lysine-specific permease |  |

  
**Neighborhood Representations for "bac:BamMC406\_3606"**  

| ID | Annotation | EC number |
| --- | --- | --- |
| bac:BamMC406\_3596 | argininosuccinate synthase (EC:6.3.4.5); K01940 argininosuccinate synthase [EC:6.3.4.5] | ec:6.3.4.5 |
| bac:BamMC406\_3597 | membrane protein |  |
| bac:BamMC406\_3598 | diguanylate cyclase |  |
| bac:BamMC406\_3599 | amino acid permease-associated protein; K11733 lysine-specific permease |  |
| bac:BamMC406\_3600 | hypothetical protein |  |
| bac:BamMC406\_3601 | LysR family transcriptional regulator |  |
| bac:BamMC406\_3602 | alpha/beta hydrolase fold protein |  |
| bac:BamMC406\_3603 | hypothetical protein |  |
| bac:BamMC406\_3604 | TetR family transcriptional regulator |  |
| bac:BamMC406\_3605 | sterol 3-beta-glucosyltransferase (EC:2.4.1.173); K05841 sterol 3beta-glucosyltransferase [EC:2.4.1.173] | ec:2.4.1.173 |
| bac:BamMC406\_3606 | histidine utilization repressor; K05836 GntR family transcriptional regulator, histidine utilization repressor |  |
| bac:BamMC406\_3607 | polar amino acid ABC transporter inner membrane subunit; K10016 histidine transport system permease protein |  |
| bac:BamMC406\_3608 | polar amino acid ABC transporter inner membrane subunit; K10015 histidine transport system permease protein |  |
| bac:BamMC406\_3609 | ABC transporter-like protein; K10017 histidine transport system ATP-binding protein [EC:3.6.3.21] | ec:3.6.3.21 |
| bac:BamMC406\_3610 | cupin 2 domain-containing protein |  |
| bac:BamMC406\_3611 | hypothetical protein |  |
| bac:BamMC406\_3612 | hypothetical protein |  |
| bac:BamMC406\_3613 | polyhydroxyalkanoate depolymerase |  |
| bac:BamMC406\_3614 | glutathione S-transferase domain-containing protein; K04097 glutathione S-transferase [EC:2.5.1.18] | ec:2.5.1.18 |
| bac:BamMC406\_3615 | cyclic nucleotide-binding protein |  |
| bac:BamMC406\_3616 | MotA/TolQ/ExbB proton channel; K02556 chemotaxis protein MotA |  |

  
**Neighborhood Representations for "bgl:bglu\_1g25200"**  

| ID | Annotation | EC number |
| --- | --- | --- |
| bgl:bglu\_1g25100 | aminotransferase; K12256 putrescine aminotransferase [EC:2.6.1.-] |  |
| bgl:bglu\_1g25110 | glutamine synthetase; K01915 glutamine synthetase [EC:6.3.1.2] | ec:6.3.1.2 |
| bgl:bglu\_1g25120 | peptidase C26; K07010 putative glutamine amidotransferase |  |
| bgl:bglu\_1g25130 | hypothetical protein |  |
| bgl:bglu\_1g25140 | aldehyde dehydrogenase; K09472 gamma-glutamyl-gamma-aminobutyraldehyde dehydrogenase [EC:1.2.1.-] |  |
| bgl:bglu\_1g25150 | HutG protein; K01458 N-formylglutamate deformylase [EC:3.5.1.68] | ec:3.5.1.68 |
| bgl:bglu\_1g25160 | N-formimino-L-glutamate deiminase; K05603 formimidoylglutamate deiminase [EC:3.5.3.13] | ec:3.5.3.13 |
| bgl:bglu\_1g25170 | imidazolonepropionase; K01468 imidazolonepropionase [EC:3.5.2.7] | ec:3.5.2.7 |
| bgl:bglu\_1g25180 | hypothetical protein; K09975 hypothetical protein |  |
| bgl:bglu\_1g25190 | urocanate hydratase; K01712 urocanate hydratase [EC:4.2.1.49] | ec:4.2.1.49 |
| bgl:bglu\_1g25200 | histidine utilization repressor; K05836 GntR family transcriptional regulator, histidine utilization repressor |  |
| bgl:bglu\_1g25210 | histidine ammonia-lyase; K01745 histidine ammonia-lyase [EC:4.3.1.3] | ec:4.3.1.3 |
| bgl:bglu\_1g25220 | 4'-phosphopantetheinyl transferase; K06133 4'-phosphopantetheinyl transferase [EC:2.7.8.-] |  |
| bgl:bglu\_1g25230 | deoxyribodipyrimidine photolyase; K01669 deoxyribodipyrimidine photo-lyase [EC:4.1.99.3] | ec:4.1.99.3 |
| bgl:bglu\_1g25240 | alkane-1 monooxygenase; K00496 alkane 1-monooxygenase [EC:1.14.15.3] | ec:1.14.15.3 |
| bgl:bglu\_1g25250 | NAD-dependent aldehyde dehydrogenase; K00135 succinate-semialdehyde dehydrogenase / glutarate-semialdehyde dehydrogenase [EC:1.2.1.16 1.2.1.79 1.2.1.20] | ec:1.2.1.79 ec:1.2.1.16 ec:1.2.1.20 |
| bgl:bglu\_1g25260 | AraC-type DNA-binding domain-containing protein |  |
| bgl:bglu\_1g25270 | methyl-accepting chemotaxis sensory transducer |  |
| bgl:bglu\_1g25280 | hypothetical protein |  |
| bgl:bglu\_1g25290 | PAAR motif-containing protein |  |
| bgl:bglu\_1g25300 | hypothetical protein |  |

  
**Neighborhood Representations for "bma:BMA0646"**  

| ID | Annotation | EC number |
| --- | --- | --- |
| bma:BMA0636 | phrB; deoxyribodipyrimidine photolyase (EC:4.1.99.3); K01669 deoxyribodipyrimidine photo-lyase [EC:4.1.99.3] | ec:4.1.99.3 |
| bma:BMA0637 | cysC-1; adenylylsulfate kinase (EC:2.7.1.25); K00860 adenylylsulfate kinase [EC:2.7.1.25] | ec:2.7.1.25 |
| bma:BMA0638 | hypothetical protein |  |
| bma:BMA0639 | pseudogene |  |
| bma:BMA0640 | pseudogene |  |
| bma:BMA0641 | LuxR family transcriptional regulator; K07782 LuxR family transcriptional regulator |  |
| bma:BMA0642 | pseudogene |  |
| bma:BMA0643 | hypothetical protein |  |
| bma:BMA0644 | pseudogene |  |
| bma:BMA0645 | hutH; histidine ammonia-lyase (EC:4.3.1.3); K01745 histidine ammonia-lyase [EC:4.3.1.3] | ec:4.3.1.3 |
| bma:BMA0646 | hutC; histidine utilization repressor; K05836 GntR family transcriptional regulator, histidine utilization repressor |  |
| bma:BMA0647 | hutU; urocanate hydratase (EC:4.2.1.49); K01712 urocanate hydratase [EC:4.2.1.49] | ec:4.2.1.49 |
| bma:BMA0648 | hypothetical protein; K09975 hypothetical protein |  |
| bma:BMA0649 | hutI; imidazolonepropionase (EC:3.5.2.7); K01468 imidazolonepropionase [EC:3.5.2.7] | ec:3.5.2.7 |
| bma:BMA0650 | hutF; N-formimino-L-glutamate deiminase (EC:3.5.3.13); K05603 formimidoylglutamate deiminase [EC:3.5.3.13] | ec:3.5.3.13 |
| bma:BMA0651 | hypothetical protein |  |
| bma:BMA0652 | hutG; N-formylglutamate amidohydrolase (EC:3.5.1.68); K01458 N-formylglutamate deformylase [EC:3.5.1.68] | ec:3.5.1.68 |
| bma:BMA0653 | hypothetical protein |  |
| bma:BMA0654 | pseudogene |  |
| bma:BMA0655 | glutamine amidotransferase; K07010 putative glutamine amidotransferase |  |
| bma:BMA0656 | glutamine synthetase; K01915 glutamine synthetase [EC:6.3.1.2] | ec:6.3.1.2 |

  
**Neighborhood Representations for "bml:BMA10229\_A2920"**  

| ID | Annotation | EC number |
| --- | --- | --- |
| bml:BMA10229\_A2910 | alkB; alkane-1 monooxygenase (EC:1.14.15.3); K00496 alkane 1-monooxygenase [EC:1.14.15.3] | ec:1.14.15.3 |
| bml:BMA10229\_A2911 | phrB; deoxyribodipyrimidine photolyase (EC:4.1.99.3); K01669 deoxyribodipyrimidine photo-lyase [EC:4.1.99.3] | ec:4.1.99.3 |
| bml:BMA10229\_A2912 | cysC-1; adenylylsulfate kinase (EC:2.7.1.25); K00860 adenylylsulfate kinase [EC:2.7.1.25] | ec:2.7.1.25 |
| bml:BMA10229\_A2913 | hypothetical protein |  |
| bml:BMA10229\_A2914 | hypothetical protein |  |
| bml:BMA10229\_A2915 | LuxR family transcriptional regulator; K07782 LuxR family transcriptional regulator |  |
| bml:BMA10229\_A2916 | hypothetical protein |  |
| bml:BMA10229\_A2917 | hypothetical protein; K06133 4'-phosphopantetheinyl transferase [EC:2.7.8.-] |  |
| bml:BMA10229\_A2918 | hypothetical protein |  |
| bml:BMA10229\_A2919 | hutH; histidine ammonia-lyase (EC:4.3.1.3); K01745 histidine ammonia-lyase [EC:4.3.1.3] | ec:4.3.1.3 |
| bml:BMA10229\_A2920 | hutC; histidine utilization repressor; K05836 GntR family transcriptional regulator, histidine utilization repressor |  |
| bml:BMA10229\_A2921 | hutU; urocanate hydratase (EC:4.2.1.49); K01712 urocanate hydratase [EC:4.2.1.49] | ec:4.2.1.49 |
| bml:BMA10229\_A2922 | hypothetical protein; K09975 hypothetical protein |  |
| bml:BMA10229\_A2923 | hutI; imidazolonepropionase (EC:3.5.2.7); K01468 imidazolonepropionase [EC:3.5.2.7] | ec:3.5.2.7 |
| bml:BMA10229\_A2924 | hutF; N-formimino-L-glutamate deiminase (EC:3.5.3.13); K05603 formimidoylglutamate deiminase [EC:3.5.3.13] | ec:3.5.3.13 |
| bml:BMA10229\_A2925 | hutG; N-formylglutamate amidohydrolase (EC:3.5.1.68); K01458 N-formylglutamate deformylase [EC:3.5.1.68] | ec:3.5.1.68 |
| bml:BMA10229\_A2926 | hypothetical protein |  |
| bml:BMA10229\_A2927 | hypothetical protein |  |
| bml:BMA10229\_A2928 | hypothetical protein |  |
| bml:BMA10229\_A2929 | glutamine amidotransferase; K07010 putative glutamine amidotransferase |  |
| bml:BMA10229\_A2930 | glutamine synthetase; K01915 glutamine synthetase [EC:6.3.1.2] | ec:6.3.1.2 |

  
**Neighborhood Representations for "bmn:BMA10247\_1680"**  

| ID | Annotation | EC number |
| --- | --- | --- |
| bmn:BMA10247\_1670 | glutamine amidotransferase; K07010 putative glutamine amidotransferase |  |
| bmn:BMA10247\_1671 | hypothetical protein |  |
| bmn:BMA10247\_1672 | hypothetical protein |  |
| bmn:BMA10247\_1673 | hypothetical protein |  |
| bmn:BMA10247\_1674 | hutG; N-formylglutamate amidohydrolase (EC:3.5.1.68); K01458 N-formylglutamate deformylase [EC:3.5.1.68] | ec:3.5.1.68 |
| bmn:BMA10247\_1675 | hypothetical protein |  |
| bmn:BMA10247\_1676 | hutF; N-formimino-L-glutamate deiminase (EC:3.5.3.13); K05603 formimidoylglutamate deiminase [EC:3.5.3.13] | ec:3.5.3.13 |
| bmn:BMA10247\_1677 | hutI; imidazolonepropionase (EC:3.5.2.7); K01468 imidazolonepropionase [EC:3.5.2.7] | ec:3.5.2.7 |
| bmn:BMA10247\_1678 | hypothetical protein; K09975 hypothetical protein |  |
| bmn:BMA10247\_1679 | hutU; urocanate hydratase (EC:4.2.1.49); K01712 urocanate hydratase [EC:4.2.1.49] | ec:4.2.1.49 |
| bmn:BMA10247\_1680 | hutC; histidine utilization repressor; K05836 GntR family transcriptional regulator, histidine utilization repressor |  |
| bmn:BMA10247\_1681 | hutH; histidine ammonia-lyase (EC:4.3.1.3); K01745 histidine ammonia-lyase [EC:4.3.1.3] | ec:4.3.1.3 |
| bmn:BMA10247\_1682 | pseudogene |  |
| bmn:BMA10247\_1683 | hypothetical protein; K06133 4'-phosphopantetheinyl transferase [EC:2.7.8.-] |  |
| bmn:BMA10247\_1684 | hypothetical protein |  |
| bmn:BMA10247\_1685 | hypothetical protein |  |
| bmn:BMA10247\_1686 | LuxR family transcriptional regulator; K07782 LuxR family transcriptional regulator |  |
| bmn:BMA10247\_1688 | hypothetical protein |  |
| bmn:BMA10247\_1687 | hypothetical protein |  |
| bmn:BMA10247\_1689 | hypothetical protein |  |
| bmn:BMA10247\_1690 | cysC-1; adenylylsulfate kinase (EC:2.7.1.25); K00860 adenylylsulfate kinase [EC:2.7.1.25] | ec:2.7.1.25 |

  
**Neighborhood Representations for "bmv:BMASAVP1\_A2365"**  

| ID | Annotation | EC number |
| --- | --- | --- |
| bmv:BMASAVP1\_A2355 | glutamine synthetase; K01915 glutamine synthetase [EC:6.3.1.2] | ec:6.3.1.2 |
| bmv:BMASAVP1\_A2356 | glutamine amidotransferase; K07010 putative glutamine amidotransferase |  |
| bmv:BMASAVP1\_A2357 | hypothetical protein |  |
| bmv:BMASAVP1\_A2358 | hypothetical protein |  |
| bmv:BMASAVP1\_A2359 | hypothetical protein |  |
| bmv:BMASAVP1\_A2360 | hutG; N-formylglutamate amidohydrolase (EC:3.5.1.68); K01458 N-formylglutamate deformylase [EC:3.5.1.68] | ec:3.5.1.68 |
| bmv:BMASAVP1\_A2361 | hutF; N-formimino-L-glutamate deiminase (EC:3.5.3.13); K05603 formimidoylglutamate deiminase [EC:3.5.3.13] | ec:3.5.3.13 |
| bmv:BMASAVP1\_A2362 | hutI; imidazolonepropionase (EC:3.5.2.7); K01468 imidazolonepropionase [EC:3.5.2.7] | ec:3.5.2.7 |
| bmv:BMASAVP1\_A2363 | hypothetical protein; K09975 hypothetical protein |  |
| bmv:BMASAVP1\_A2364 | hutU; urocanate hydratase (EC:4.2.1.49); K01712 urocanate hydratase [EC:4.2.1.49] | ec:4.2.1.49 |
| bmv:BMASAVP1\_A2365 | hutC; histidine utilization repressor; K05836 GntR family transcriptional regulator, histidine utilization repressor |  |
| bmv:BMASAVP1\_A2366 | hutH; histidine ammonia-lyase (EC:4.3.1.3); K01745 histidine ammonia-lyase [EC:4.3.1.3] | ec:4.3.1.3 |
| bmv:BMASAVP1\_A2367 | hypothetical protein |  |
| bmv:BMASAVP1\_A2368 | hypothetical protein; K06133 4'-phosphopantetheinyl transferase [EC:2.7.8.-] |  |
| bmv:BMASAVP1\_A2369 | hypothetical protein |  |
| bmv:BMASAVP1\_A2370 | pseudogene |  |
| bmv:BMASAVP1\_A2371 | ATP-dependent transcription regulator LuxR; K07782 LuxR family transcriptional regulator |  |
| bmv:BMASAVP1\_A2373 | hypothetical protein |  |
| bmv:BMASAVP1\_A2372 | hypothetical protein |  |
| bmv:BMASAVP1\_A2374 | hypothetical protein |  |
| bmv:BMASAVP1\_A2375 | cysC-1; adenylylsulfate kinase (EC:2.7.1.25); K00860 adenylylsulfate kinase [EC:2.7.1.25] | ec:2.7.1.25 |

  
**Neighborhood Representations for "bpd:BURPS668\_2666"**  

| ID | Annotation | EC number |
| --- | --- | --- |
| bpd:BURPS668\_2657 | hypothetical protein |  |
| bpd:BURPS668\_2656 | hypothetical protein |  |
| bpd:BURPS668\_2658 | hypothetical protein |  |
| bpd:BURPS668\_2659 | hypothetical protein |  |
| bpd:BURPS668\_2660 | hutG; N-formylglutamate amidohydrolase (EC:3.5.1.68); K01458 N-formylglutamate deformylase [EC:3.5.1.68] | ec:3.5.1.68 |
| bpd:BURPS668\_2661 | hypothetical protein |  |
| bpd:BURPS668\_2662 | hutF; N-formimino-L-glutamate deiminase (EC:3.5.3.13); K05603 formimidoylglutamate deiminase [EC:3.5.3.13] | ec:3.5.3.13 |
| bpd:BURPS668\_2663 | hutI; imidazolonepropionase (EC:3.5.2.7); K01468 imidazolonepropionase [EC:3.5.2.7] | ec:3.5.2.7 |
| bpd:BURPS668\_2664 | hypothetical protein; K09975 hypothetical protein |  |
| bpd:BURPS668\_2665 | hutU; urocanate hydratase (EC:4.2.1.49); K01712 urocanate hydratase [EC:4.2.1.49] | ec:4.2.1.49 |
| bpd:BURPS668\_2666 | hutC; histidine utilization repressor; K05836 GntR family transcriptional regulator, histidine utilization repressor |  |
| bpd:BURPS668\_2667 | hutH; histidine ammonia-lyase (EC:4.3.1.3); K01745 histidine ammonia-lyase [EC:4.3.1.3] | ec:4.3.1.3 |
| bpd:BURPS668\_2668 | hypothetical protein |  |
| bpd:BURPS668\_2669 | hypothetical protein; K06133 4'-phosphopantetheinyl transferase [EC:2.7.8.-] |  |
| bpd:BURPS668\_2670 | hypothetical protein |  |
| bpd:BURPS668\_2671 | hypothetical protein |  |
| bpd:BURPS668\_2672 | LuxR family transcriptional regulator; K07782 LuxR family transcriptional regulator |  |
| bpd:BURPS668\_2673 | hypothetical protein |  |
| bpd:BURPS668\_2674 | hypothetical protein |  |
| bpd:BURPS668\_2675 | hypothetical protein |  |
| bpd:BURPS668\_2676 | cysC; adenylylsulfate kinase (EC:2.7.1.25); K00860 adenylylsulfate kinase [EC:2.7.1.25] | ec:2.7.1.25 |

  
**Neighborhood Representations for "bpl:BURPS1106A\_2722"**  

| ID | Annotation | EC number |
| --- | --- | --- |
| bpl:BURPS1106A\_2712 | glutamine amidotransferase; K07010 putative glutamine amidotransferase |  |
| bpl:BURPS1106A\_2713 | hypothetical protein |  |
| bpl:BURPS1106A\_2714 | hypothetical protein |  |
| bpl:BURPS1106A\_2715 | hypothetical protein |  |
| bpl:BURPS1106A\_2716 | hutG; N-formylglutamate amidohydrolase (EC:3.5.1.68); K01458 N-formylglutamate deformylase [EC:3.5.1.68] | ec:3.5.1.68 |
| bpl:BURPS1106A\_2717 | hypothetical protein |  |
| bpl:BURPS1106A\_2718 | hutF; N-formimino-L-glutamate deiminase (EC:3.5.3.13); K05603 formimidoylglutamate deiminase [EC:3.5.3.13] | ec:3.5.3.13 |
| bpl:BURPS1106A\_2719 | hutI; imidazolonepropionase (EC:3.5.2.7); K01468 imidazolonepropionase [EC:3.5.2.7] | ec:3.5.2.7 |
| bpl:BURPS1106A\_2720 | hypothetical protein; K09975 hypothetical protein |  |
| bpl:BURPS1106A\_2721 | hutU; urocanate hydratase (EC:4.2.1.49); K01712 urocanate hydratase [EC:4.2.1.49] | ec:4.2.1.49 |
| bpl:BURPS1106A\_2722 | hutC; histidine utilization repressor; K05836 GntR family transcriptional regulator, histidine utilization repressor |  |
| bpl:BURPS1106A\_2723 | hutH; histidine ammonia-lyase (EC:4.3.1.3); K01745 histidine ammonia-lyase [EC:4.3.1.3] | ec:4.3.1.3 |
| bpl:BURPS1106A\_2724 | hypothetical protein |  |
| bpl:BURPS1106A\_2725 | hypothetical protein |  |
| bpl:BURPS1106A\_2726 | transposase, IS4 |  |
| bpl:BURPS1106A\_2727 | 4'-phosphopantetheinyl transferase; K06133 4'-phosphopantetheinyl transferase [EC:2.7.8.-] |  |
| bpl:BURPS1106A\_2728 | hypothetical protein |  |
| bpl:BURPS1106A\_2729 | ATP-dependent transcription regulator LuxR; K07782 LuxR family transcriptional regulator |  |
| bpl:BURPS1106A\_2730 | hypothetical protein |  |
| bpl:BURPS1106A\_2731 | hypothetical protein |  |
| bpl:BURPS1106A\_2732 | hypothetical protein |  |

  
**Neighborhood Representations for "bpr:GBP346\_A2845"**  

| ID | Annotation | EC number |
| --- | --- | --- |
| bpr:GBP346\_A2835 | glutamine synthetase family protein; K01915 glutamine synthetase [EC:6.3.1.2] | ec:6.3.1.2 |
| bpr:GBP346\_A2836 | glutamine amidotransferase, class I; K07010 putative glutamine amidotransferase |  |
| bpr:GBP346\_A2837 | hypothetical protein |  |
| bpr:GBP346\_A2839 | hypothetical protein |  |
| bpr:GBP346\_A2838 | hutG; N-formylglutamate deformylase (EC:3.5.1.68); K01458 N-formylglutamate deformylase [EC:3.5.1.68] | ec:3.5.1.68 |
| bpr:GBP346\_A2840 | hypothetical protein |  |
| bpr:GBP346\_A2841 | hutF; N-formimino-L-glutamate deiminase (EC:3.5.3.13); K05603 formimidoylglutamate deiminase [EC:3.5.3.13] | ec:3.5.3.13 |
| bpr:GBP346\_A2842 | hutI; imidazolonepropionase (EC:3.5.2.7); K01468 imidazolonepropionase [EC:3.5.2.7] | ec:3.5.2.7 |
| bpr:GBP346\_A2843 | hypothetical protein; K09975 hypothetical protein |  |
| bpr:GBP346\_A2844 | hutU\_2; urocanate hydratase (EC:4.2.1.49); K01712 urocanate hydratase [EC:4.2.1.49] | ec:4.2.1.49 |
| bpr:GBP346\_A2845 | hutC; histidine utilization repressor; K05836 GntR family transcriptional regulator, histidine utilization repressor |  |
| bpr:GBP346\_A2846 | hutH; histidine ammonia-lyase (EC:4.3.1.3); K01745 histidine ammonia-lyase [EC:4.3.1.3] | ec:4.3.1.3 |
| bpr:GBP346\_A2847 | hypothetical protein |  |
| bpr:GBP346\_A2848 | 4'-phosphopantetheinyl transferase family protein; K06133 4'-phosphopantetheinyl transferase [EC:2.7.8.-] |  |
| bpr:GBP346\_A2849 | hypothetical protein |  |
| bpr:GBP346\_A2850 | 4'-phosphopantetheinyl transferase family protein |  |
| bpr:GBP346\_A2851 | autoinducer-binding transcriptional regulator, LuxR family; K07782 LuxR family transcriptional regulator |  |
| bpr:GBP346\_A2852 | hypothetical protein |  |
| bpr:GBP346\_A2853 | hypothetical protein |  |
| bpr:GBP346\_A2854 | hypothetical protein |  |
| bpr:GBP346\_A2855 | cysC\_2; adenylyl-sulfate kinase (EC:2.7.1.25); K00860 adenylylsulfate kinase [EC:2.7.1.25] | ec:2.7.1.25 |

  
**Neighborhood Representations for "bps:BPSL2343"**  

| ID | Annotation | EC number |
| --- | --- | --- |
| bps:BPSL2333 | hypothetical protein |  |
| bps:BPSL2334 | hypothetical protein |  |
| bps:BPSL2335 | aminotransferase; K12256 putrescine aminotransferase [EC:2.6.1.-] |  |
| bps:BPSL2336 | glutamine synthetase; K01915 glutamine synthetase [EC:6.3.1.2] | ec:6.3.1.2 |
| bps:BPSL2337 | glutamine amidotransferase class-I; K07010 putative glutamine amidotransferase |  |
| bps:BPSL2338 | HutG protein; K01458 N-formylglutamate deformylase [EC:3.5.1.68] | ec:3.5.1.68 |
| bps:BPSL2339 | N-formimino-L-glutamate deiminase; K05603 formimidoylglutamate deiminase [EC:3.5.3.13] | ec:3.5.3.13 |
| bps:BPSL2340 | imidazolonepropionase; K01468 imidazolonepropionase [EC:3.5.2.7] | ec:3.5.2.7 |
| bps:BPSL2341 | hypothetical protein; K09975 hypothetical protein |  |
| bps:BPSL2342 | hutU; urocanate hydratase (EC:4.2.1.49); K01712 urocanate hydratase [EC:4.2.1.49] | ec:4.2.1.49 |
| bps:BPSL2343 | histidine utilization repressor; K05836 GntR family transcriptional regulator, histidine utilization repressor |  |
| bps:BPSL2344 | hutH; histidine ammonia-lyase (EC:4.3.1.3); K01745 histidine ammonia-lyase [EC:4.3.1.3] | ec:4.3.1.3 |
| bps:BPSL2345 | hypothetical protein; K06133 4'-phosphopantetheinyl transferase [EC:2.7.8.-] |  |
| bps:BPSL2346 | hypothetical protein |  |
| bps:BPSL2347 | LuxR family transcriptional regulator; K07782 LuxR family transcriptional regulator |  |
| bps:BPSL2348 | pseudogene |  |
| bps:BPSL2349 | DNA photolyase; K01669 deoxyribodipyrimidine photo-lyase [EC:4.1.99.3] | ec:4.1.99.3 |
| bps:BPSL2350 | alkane monooxygenase; K00496 alkane 1-monooxygenase [EC:1.14.15.3] | ec:1.14.15.3 |
| bps:BPSL2351 | hypothetical protein; K04561 nitric oxide reductase subunit B [EC:1.7.2.5] | ec:1.7.2.5 |
| bps:BPSL2352 | pseudogene |  |
| bps:BPSL2354 | hypothetical protein |  |

  
**Neighborhood Representations for "bpm:BURPS1710b\_2795"**  

| ID | Annotation | EC number |
| --- | --- | --- |
| bpm:BURPS1710b\_2785 | staB; StaB protein |  |
| bpm:BURPS1710b\_2786 | hypothetical protein |  |
| bpm:BURPS1710b\_2787 | aminotransferase; K12256 putrescine aminotransferase [EC:2.6.1.-] |  |
| bpm:BURPS1710b\_2788 | glutamine synthetase family protein (EC:6.3.1.2); K01915 glutamine synthetase [EC:6.3.1.2] | ec:6.3.1.2 |
| bpm:BURPS1710b\_2789 | glutamine amidotransferase, class I (EC:4.1.3.27); K07010 putative glutamine amidotransferase |  |
| bpm:BURPS1710b\_2790 | hutG; N-formylglutamate amidohydrolase (EC:3.5.1.68); K01458 N-formylglutamate deformylase [EC:3.5.1.68] | ec:3.5.1.68 |
| bpm:BURPS1710b\_2791 | hutF; N-formimino-L-glutamate deiminase (EC:3.5.3.13); K05603 formimidoylglutamate deiminase [EC:3.5.3.13] | ec:3.5.3.13 |
| bpm:BURPS1710b\_2792 | hutI; imidazolonepropionase (EC:3.5.2.7); K01468 imidazolonepropionase [EC:3.5.2.7] | ec:3.5.2.7 |
| bpm:BURPS1710b\_2793 | hypothetical protein; K09975 hypothetical protein |  |
| bpm:BURPS1710b\_2794 | hutU; urocanate hydratase (EC:4.2.1.49); K01712 urocanate hydratase [EC:4.2.1.49] | ec:4.2.1.49 |
| bpm:BURPS1710b\_2795 | hutC; histidine utilization repressor; K05836 GntR family transcriptional regulator, histidine utilization repressor |  |
| bpm:BURPS1710b\_2796 | hutH; histidine ammonia-lyase (EC:4.3.1.3); K01745 histidine ammonia-lyase [EC:4.3.1.3] | ec:4.3.1.3 |
| bpm:BURPS1710b\_2797 | 4'-phosphopantetheinyl transferase; K06133 4'-phosphopantetheinyl transferase [EC:2.7.8.-] |  |
| bpm:BURPS1710b\_2798 | LuxR family transcriptional regulator; K07782 LuxR family transcriptional regulator |  |
| bpm:BURPS1710b\_2799 | cysC; adenylylsulfate kinase (EC:2.7.1.25); K00860 adenylylsulfate kinase [EC:2.7.1.25] | ec:2.7.1.25 |
| bpm:BURPS1710b\_2800 | phrB; deoxyribodipyrimidine photolyase (EC:4.1.99.3); K01669 deoxyribodipyrimidine photo-lyase [EC:4.1.99.3] | ec:4.1.99.3 |
| bpm:BURPS1710b\_2801 | alkane-1 monooxygenase (EC:1.14.15.3); K00496 alkane 1-monooxygenase [EC:1.14.15.3] | ec:1.14.15.3 |
| bpm:BURPS1710b\_2802 | norB; nitric oxide reductase subunit B; K04561 nitric oxide reductase subunit B [EC:1.7.2.5] | ec:1.7.2.5 |
| bpm:BURPS1710b\_2803 | hypothetical protein |  |
| bpm:BURPS1710b\_2804 | phenylacetic acid degradation protein paaD |  |
| bpm:BURPS1710b\_2806 | hypothetical protein |  |

  
**Neighborhood Representations for "bte:BTH\_I1821"**  

| ID | Annotation | EC number |
| --- | --- | --- |
| bte:BTH\_I1811 | lipoprotein |  |
| bte:BTH\_I1812 | mrp protein |  |
| bte:BTH\_I1813 | nitric oxide reductase; K04561 nitric oxide reductase subunit B [EC:1.7.2.5] | ec:1.7.2.5 |
| bte:BTH\_I1814 | alkane-1 monooxygenase; K00496 alkane 1-monooxygenase [EC:1.14.15.3] | ec:1.14.15.3 |
| bte:BTH\_I1815 | deoxyribodipyrimidine photolyase; K01669 deoxyribodipyrimidine photo-lyase [EC:4.1.99.3] | ec:4.1.99.3 |
| bte:BTH\_I1816 | cysC; adenylylsulfate kinase (EC:2.7.1.25); K00860 adenylylsulfate kinase [EC:2.7.1.25] | ec:2.7.1.25 |
| bte:BTH\_I1817 | ATP-dependent transcription regulator LuxR; K07782 LuxR family transcriptional regulator |  |
| bte:BTH\_I1818 | hypothetical protein |  |
| bte:BTH\_I1819 | phosphopantetheinyltransferase family protein (EC:2.7.8.-); K06133 4'-phosphopantetheinyl transferase [EC:2.7.8.-] |  |
| bte:BTH\_I1820 | hutH; histidine ammonia-lyase (EC:4.3.1.3); K01745 histidine ammonia-lyase [EC:4.3.1.3] | ec:4.3.1.3 |
| bte:BTH\_I1821 | histidine utilization repressor; K05836 GntR family transcriptional regulator, histidine utilization repressor |  |
| bte:BTH\_I1822 | hutU; urocanate hydratase (EC:4.2.1.49); K01712 urocanate hydratase [EC:4.2.1.49] | ec:4.2.1.49 |
| bte:BTH\_I1823 | hypothetical protein; K09975 hypothetical protein |  |
| bte:BTH\_I1824 | hutI; imidazolonepropionase (EC:3.5.2.7); K01468 imidazolonepropionase [EC:3.5.2.7] | ec:3.5.2.7 |
| bte:BTH\_I1825 | hutF; N-formimino-L-glutamate deiminase (EC:3.5.3.13); K05603 formimidoylglutamate deiminase [EC:3.5.3.13] | ec:3.5.3.13 |
| bte:BTH\_I1826 | hutG; N-formylglutamate amidohydrolase (EC:3.5.1.68); K01458 N-formylglutamate deformylase [EC:3.5.1.68] | ec:3.5.1.68 |
| bte:BTH\_I1827 | glutamine amidotransferase, class I; K07010 putative glutamine amidotransferase |  |
| bte:BTH\_I1828 | glutamine synthetase family protein; K01915 glutamine synthetase [EC:6.3.1.2] | ec:6.3.1.2 |
| bte:BTH\_I1829 | aminotransferase; K12256 putrescine aminotransferase [EC:2.6.1.-] |  |
| bte:BTH\_I1830 | hypothetical protein |  |
| bte:BTH\_I1831 | tRNA-Val; K14237 tRNA Val |  |

  
**Neighborhood Representations for "rpi:Rpic\_2882"**  

| ID | Annotation | EC number |
| --- | --- | --- |
| rpi:Rpic\_2872 | GreA/GreB family elongation factor; K06140 regulator of nucleoside diphosphate kinase |  |
| rpi:Rpic\_2873 | putative thioredoxin protein |  |
| rpi:Rpic\_2874 | heat shock protein GrpE; K03687 molecular chaperone GrpE |  |
| rpi:Rpic\_2875 | hypothetical protein |  |
| rpi:Rpic\_2876 | RNA-binding S4 domain-containing protein; K04762 ribosome-associated heat shock protein Hsp15 |  |
| rpi:Rpic\_2877 | hemH; ferrochelatase (EC:4.99.1.1); K01772 ferrochelatase [EC:4.99.1.1] | ec:4.99.1.1 |
| rpi:Rpic\_2878 | imidazolonepropionase (EC:3.5.2.7); K01468 imidazolonepropionase [EC:3.5.2.7] | ec:3.5.2.7 |
| rpi:Rpic\_2879 | formimidoylglutamase; K01479 formiminoglutamase [EC:3.5.3.8] | ec:3.5.3.8 |
| rpi:Rpic\_2880 | histidine ammonia-lyase (EC:4.3.1.3); K01745 histidine ammonia-lyase [EC:4.3.1.3] | ec:4.3.1.3 |
| rpi:Rpic\_2881 | urocanate hydratase (EC:4.2.1.49); K01712 urocanate hydratase [EC:4.2.1.49] | ec:4.2.1.49 |
| rpi:Rpic\_2882 | transcriptional regulator, histidine utilization repressor, GntR family; K05836 GntR family transcriptional regulator, histidine utilization repressor |  |
| rpi:Rpic\_2883 | binding-protein-dependent transport system inner membrane protein; K15552 taurine transport system permease protein |  |
| rpi:Rpic\_2884 | ABC transporter-like protein; K10831 taurine transport system ATP-binding protein [EC:3.6.3.36] | ec:3.6.3.36 |
| rpi:Rpic\_2885 | taurine ABC transporter periplasmic binding protein; K15551 taurine transport system substrate-binding protein |  |
| rpi:Rpic\_2886 | hrcA; heat-inducible transcription repressor; K03705 heat-inducible transcriptional repressor |  |
| rpi:Rpic\_2887 | ppnK; NAD(+)/NADH kinase family protein; K00858 NAD+ kinase [EC:2.7.1.23] | ec:2.7.1.23 |
| rpi:Rpic\_2888 | DNA repair protein RecN; K03631 DNA repair protein RecN (Recombination protein N) |  |
| rpi:Rpic\_2889 | putative lipoprotein |  |
| rpi:Rpic\_2890 | peptidase S8/S53 subtilisin kexin sedolisin; K14645 serine protease [EC:3.4.21.-] |  |
| rpi:Rpic\_2891 | peptidase S8/S53 subtilisin kexin sedolisin; K14645 serine protease [EC:3.4.21.-] |  |
| rpi:Rpic\_2892 | hypothetical protein |  |

  
**Neighborhood Representations for "rsl:RPSI07\_0869"**  

| ID | Annotation | EC number |
| --- | --- | --- |
| rsl:RPSI07\_0859 | hypothetical protein |  |
| rsl:RPSI07\_0860 | glnE; glutamate-ammonia-ligase adenylyltransferase (EC:2.7.7.42); K00982 glutamate-ammonia-ligase adenylyltransferase [EC:2.7.7.42] | ec:2.7.7.42 |
| rsl:RPSI07\_0861 | hypothetical protein |  |
| rsl:RPSI07\_0862 | extracellular subtilisin-like protease (EC:3.4.21.-); K14645 serine protease [EC:3.4.21.-] |  |
| rsl:RPSI07\_0863 | extracellular protease , subtilisin-like protein (EC:3.4.21.-); K14645 serine protease [EC:3.4.21.-] |  |
| rsl:RPSI07\_0864 | hypothetical protein |  |
| rsl:RPSI07\_0865 | hypothetical protein |  |
| rsl:RPSI07\_0866 | recN; DNA repair protein recN (Recombination protein N); K03631 DNA repair protein RecN (Recombination protein N) |  |
| rsl:RPSI07\_0867 | ppnK; inorganic polyphosphate/ATP-NAD kinase (EC:2.7.1.23); K00858 NAD+ kinase [EC:2.7.1.23] | ec:2.7.1.23 |
| rsl:RPSI07\_0868 | hrcA; heat-inducible transcription repressor, negative regulator of class I heat shock protein; K03705 heat-inducible transcriptional repressor |  |
| rsl:RPSI07\_0869 | hutC; GntR family transcriptional regulator; K05836 GntR family transcriptional regulator, histidine utilization repressor |  |
| rsl:RPSI07\_0870 | hutU; urocanate hydratase (EC:4.2.1.49); K01712 urocanate hydratase [EC:4.2.1.49] | ec:4.2.1.49 |
| rsl:RPSI07\_0871 | hutH; histidine ammonia-lyase (EC:4.3.1.3); K01745 histidine ammonia-lyase [EC:4.3.1.3] | ec:4.3.1.3 |
| rsl:RPSI07\_0872 | hutG; formimidoylglutamase (EC:3.5.3.8); K01479 formiminoglutamase [EC:3.5.3.8] | ec:3.5.3.8 |
| rsl:RPSI07\_0873 | hutI; imidazolonepropionase (EC:3.5.2.7); K01468 imidazolonepropionase [EC:3.5.2.7] | ec:3.5.2.7 |
| rsl:RPSI07\_0874 | hemH; ferrochelatase (EC:4.99.1.1); K01772 ferrochelatase [EC:4.99.1.1] | ec:4.99.1.1 |
| rsl:RPSI07\_0875 | hslR; ribosome-associated heat shock protein 15; K04762 ribosome-associated heat shock protein Hsp15 |  |
| rsl:RPSI07\_0876 | hypothetical protein |  |
| rsl:RPSI07\_0877 | transposase |  |
| rsl:RPSI07\_0878 | grpE; Hsp 24 nucleotide exchange factor, Ribulose-phosphate 3-epimerase activity (EC:5.1.3.1); K03687 molecular chaperone GrpE |  |
| rsl:RPSI07\_0879 | pseudogene |  |

  
**Neighborhood Representations for "rso:RSc2648"**  

| ID | Annotation | EC number |
| --- | --- | --- |
| rso:RSc2638 | thioredoxin protein |  |
| rso:RSc2639 | grpE; heat shock protein GrpE; K03687 molecular chaperone GrpE |  |
| rso:RSc2640 | transposase |  |
| rso:RSc2641 | transmembrane protein |  |
| rso:RSc2642 | hslR; heat shock protein 15; K04762 ribosome-associated heat shock protein Hsp15 |  |
| rso:RSc2643 | hemH; ferrochelatase (EC:4.99.1.1); K01772 ferrochelatase [EC:4.99.1.1] | ec:4.99.1.1 |
| rso:RSc2644 | hutI; imidazolonepropionase (EC:3.5.2.7); K01468 imidazolonepropionase [EC:3.5.2.7] | ec:3.5.2.7 |
| rso:RSc2645 | formimidoylglutamase (EC:3.5.3.8 3.5.3.-); K01479 formiminoglutamase [EC:3.5.3.8] | ec:3.5.3.8 |
| rso:RSc2646 | hutH; histidine ammonia-lyase (EC:4.3.1.3); K01745 histidine ammonia-lyase [EC:4.3.1.3] | ec:4.3.1.3 |
| rso:RSc2647 | hutU; urocanate hydratase (EC:4.2.1.49); K01712 urocanate hydratase [EC:4.2.1.49] | ec:4.2.1.49 |
| rso:RSc2648 | hutC; histidine utilization repressor transcription regulator protein; K05836 GntR family transcriptional regulator, histidine utilization repressor |  |
| rso:RSc2649 | hrcA; heat-inducible transcription repressor; K03705 heat-inducible transcriptional repressor |  |
| rso:RSc2650 | ppnK; NAD(+)/NADH kinase family protein (EC:2.7.1.23); K00858 NAD+ kinase [EC:2.7.1.23] | ec:2.7.1.23 |
| rso:RSc2651 | recN; DNA repair protein; K03631 DNA repair protein RecN (Recombination protein N) |  |
| rso:RSc2652 | lipoprotein |  |
| rso:RSc2653 | extracellular protease signal peptide protein (EC:3.4.-.-); K14645 serine protease [EC:3.4.21.-] |  |
| rso:RSc2654 | serine protease (EC:3.4.21.-); K14645 serine protease [EC:3.4.21.-] |  |
| rso:RSc2655 | hypothetical protein |  |
| rso:RSc2656 | glnE; bifunctional glutamine-synthetase adenylyltransferase/deadenyltransferase (EC:2.7.7.42); K00982 glutamate-ammonia-ligase adenylyltransferase [EC:2.7.7.42] | ec:2.7.7.42 |
| rso:RSc2657 | hypothetical protein |  |
| rso:RSc2658 | nitrilase (EC:3.5.5.1); K01501 nitrilase [EC:3.5.5.1] | ec:3.5.5.1 |

  
**Neighborhood Representations for "rpf:Rpic12D\_2476"**  

| ID | Annotation | EC number |
| --- | --- | --- |
| rpf:Rpic12D\_2466 | GreA/GreB family elongation factor; K06140 regulator of nucleoside diphosphate kinase |  |
| rpf:Rpic12D\_2467 | thioredoxin protein |  |
| rpf:Rpic12D\_2468 | heat shock protein GrpE; K03687 molecular chaperone GrpE |  |
| rpf:Rpic12D\_2469 | hypothetical protein |  |
| rpf:Rpic12D\_2470 | RNA-binding S4 domain-containing protein; K04762 ribosome-associated heat shock protein Hsp15 |  |
| rpf:Rpic12D\_2471 | hemH; ferrochelatase (EC:4.99.1.1); K01772 ferrochelatase [EC:4.99.1.1] | ec:4.99.1.1 |
| rpf:Rpic12D\_2472 | imidazolonepropionase (EC:3.5.2.7); K01468 imidazolonepropionase [EC:3.5.2.7] | ec:3.5.2.7 |
| rpf:Rpic12D\_2473 | formimidoylglutamase; K01479 formiminoglutamase [EC:3.5.3.8] | ec:3.5.3.8 |
| rpf:Rpic12D\_2474 | histidine ammonia-lyase (EC:4.3.1.3); K01745 histidine ammonia-lyase [EC:4.3.1.3] | ec:4.3.1.3 |
| rpf:Rpic12D\_2475 | urocanate hydratase (EC:4.2.1.49); K01712 urocanate hydratase [EC:4.2.1.49] | ec:4.2.1.49 |
| rpf:Rpic12D\_2476 | GntR family transcriptional regulator; K05836 GntR family transcriptional regulator, histidine utilization repressor |  |
| rpf:Rpic12D\_2477 | binding-protein-dependent transport system inner membrane protein; K15552 taurine transport system permease protein |  |
| rpf:Rpic12D\_2478 | ABC transporter; K10831 taurine transport system ATP-binding protein [EC:3.6.3.36] | ec:3.6.3.36 |
| rpf:Rpic12D\_2479 | taurine ABC transporter periplasmic binding protein; K15551 taurine transport system substrate-binding protein |  |
| rpf:Rpic12D\_2480 | hrcA; heat-inducible transcription repressor; K03705 heat-inducible transcriptional repressor |  |
| rpf:Rpic12D\_2481 | ppnK; NAD(+)/NADH kinase family protein; K00858 NAD+ kinase [EC:2.7.1.23] | ec:2.7.1.23 |
| rpf:Rpic12D\_2482 | DNA repair protein RecN; K03631 DNA repair protein RecN (Recombination protein N) |  |
| rpf:Rpic12D\_2483 | lipoprotein |  |
| rpf:Rpic12D\_2484 | peptidase S8/S53 subtilisin kexin sedolisin; K14645 serine protease [EC:3.4.21.-] |  |
| rpf:Rpic12D\_2485 | peptidase S8/S53 subtilisin kexin sedolisin; K14645 serine protease [EC:3.4.21.-] |  |
| rpf:Rpic12D\_2486 | hypothetical protein |  |

  
**Neighborhood Representations for "rsc:RCFBP\_10799"**  

| ID | Annotation | EC number |
| --- | --- | --- |
| rsc:RCFBP\_10789 | nitrilase (EC:3.5.5.1); K01501 nitrilase [EC:3.5.5.1] | ec:3.5.5.1 |
| rsc:RCFBP\_10790 | hypothetical protein |  |
| rsc:RCFBP\_10791 | glnE; glutamate-ammonia-ligase adenylyltransferase (EC:2.7.7.42); K00982 glutamate-ammonia-ligase adenylyltransferase [EC:2.7.7.42] | ec:2.7.7.42 |
| rsc:RCFBP\_10792 | hypothetical protein |  |
| rsc:RCFBP\_10793 | extracellular subtilisiN-like protease (EC:3.4.21.-); K14645 serine protease [EC:3.4.21.-] |  |
| rsc:RCFBP\_10794 | extracellular protease , subtilisiN-like protein (EC:3.4.21.-); K14645 serine protease [EC:3.4.21.-] |  |
| rsc:RCFBP\_10795 | hypothetical protein |  |
| rsc:RCFBP\_10796 | recN; DNA repair protein recn (recombination protein n); K03631 DNA repair protein RecN (Recombination protein N) |  |
| rsc:RCFBP\_10797 | ppnK; inorganic polyphosphate/ATP-nad kinase (poly(p)/ATP nad kinase) (EC:2.7.1.23); K00858 NAD+ kinase [EC:2.7.1.23] | ec:2.7.1.23 |
| rsc:RCFBP\_10798 | hrcA; heat-inducible transcription repressor, negative regulator of class i heat shock protein; K03705 heat-inducible transcriptional repressor |  |
| rsc:RCFBP\_10799 | hutC; GntR family transcriptional regulator; K05836 GntR family transcriptional regulator, histidine utilization repressor |  |
| rsc:RCFBP\_10800 | hutU; urocanate hydratase (urocanase) (imidazolonepropionate hydrolase) (EC:4.2.1.49); K01712 urocanate hydratase [EC:4.2.1.49] | ec:4.2.1.49 |
| rsc:RCFBP\_10801 | hutH; histidine ammonia-lyase (histidase) (EC:4.3.1.3); K01745 histidine ammonia-lyase [EC:4.3.1.3] | ec:4.3.1.3 |
| rsc:RCFBP\_10802 | hutG; formimidoylglutamase (EC:3.5.3.8); K01479 formiminoglutamase [EC:3.5.3.8] | ec:3.5.3.8 |
| rsc:RCFBP\_10803 | hutI; imidazolonepropionase (imidazolone-5-propionate hydrolase) (EC:3.5.2.7); K01468 imidazolonepropionase [EC:3.5.2.7] | ec:3.5.2.7 |
| rsc:RCFBP\_10804 | hemH; ferrochelatase (EC:4.99.1.1); K01772 ferrochelatase [EC:4.99.1.1] | ec:4.99.1.1 |
| rsc:RCFBP\_10805 | hslR; ribosome-associated heat shock protein 15; K04762 ribosome-associated heat shock protein Hsp15 |  |
| rsc:RCFBP\_10806 | hypothetical protein |  |
| rsc:RCFBP\_10807 | transposase |  |
| rsc:RCFBP\_10808 | grpE; hsp 24 nucleotide exchange factor, ribulose-phosphate 3-epimerase activity (EC:5.1.3.1); K03687 molecular chaperone GrpE |  |
| rsc:RCFBP\_10809 | thioredoxin |  |

  
**Neighborhood Representations for "ctt:CtCNB1\_0165"**  

| ID | Annotation | EC number |
| --- | --- | --- |
| ctt:CtCNB1\_0155 | transcriptional regulator, histidine; K05836 GntR family transcriptional regulator, histidine utilization repressor |  |
| ctt:CtCNB1\_0156 | acyl-CoA dehydrogenase |  |
| ctt:CtCNB1\_0158 | LysR family transcriptional regulator |  |
| ctt:CtCNB1\_0157 | bile acid-inducible L-carnitine dehydratase |  |
| ctt:CtCNB1\_0159 | phytanoyl-CoA dioxygenase |  |
| ctt:CtCNB1\_0160 | family 3 extracellular solute-binding protein; K02051 NitT/TauT family transport system substrate-binding protein |  |
| ctt:CtCNB1\_0161 | taurine transporter permeaseprotein tauC; K02050 NitT/TauT family transport system permease protein |  |
| ctt:CtCNB1\_0162 | taurine transport ATP-binding protein tauB; K02049 NitT/TauT family transport system ATP-binding protein |  |
| ctt:CtCNB1\_0163 | transcriptional regulator, histidine; K05836 GntR family transcriptional regulator, histidine utilization repressor |  |
| ctt:CtCNB1\_0164 | histidine ammonia-lyase; K01745 histidine ammonia-lyase [EC:4.3.1.3] | ec:4.3.1.3 |
| ctt:CtCNB1\_0165 | transcriptional regulator, histidine; K05836 GntR family transcriptional regulator, histidine utilization repressor |  |
| ctt:CtCNB1\_0166 | cytosine/purines/uracil permease; K03457 nucleobase:cation symporter-1, NCS1 family |  |
| ctt:CtCNB1\_0167 | urocanate hydratase; K01712 urocanate hydratase [EC:4.2.1.49] | ec:4.2.1.49 |
| ctt:CtCNB1\_0168 | amidohydrolase; K01451 hippurate hydrolase [EC:3.5.1.32] | ec:3.5.1.32 |
| ctt:CtCNB1\_0169 | hypothetical protein; K09975 hypothetical protein |  |
| ctt:CtCNB1\_0170 | imidazolonepropionase; K01468 imidazolonepropionase [EC:3.5.2.7] | ec:3.5.2.7 |
| ctt:CtCNB1\_0171 | FAD linked oxidase-like protein |  |
| ctt:CtCNB1\_0172 | histidinol-phosphate aminotransferase; K00817 histidinol-phosphate aminotransferase [EC:2.6.1.9] | ec:2.6.1.9 |
| ctt:CtCNB1\_0173 | major facilitator superfamily protein |  |
| ctt:CtCNB1\_0174 | formiminoglutamate deiminase; K05603 formimidoylglutamate deiminase [EC:3.5.3.13] | ec:3.5.3.13 |
| ctt:CtCNB1\_0175 | N-formylglutamate amidohydrolase; K01458 N-formylglutamate deformylase [EC:3.5.1.68] | ec:3.5.1.68 |

  
**Neighborhood Representations for "ddd:Dda3937\_01666"**  

| ID | Annotation | EC number |
| --- | --- | --- |
| ddd:Dda3937\_01657 | ydfJ; MFS superfamily transporter; K08173 MFS transporter, MHS family, metabolite:H+ symporter |  |
| ddd:Dda3937\_01658 | ydfI; D-mannonate oxidoreductase; K00040 fructuronate reductase [EC:1.1.1.57] | ec:1.1.1.57 |
| ddd:Dda3937\_01659 | ydfH; GntR family transcriptional regulator |  |
| ddd:Dda3937\_01660 | hutU; urocanate hydratase; K01712 urocanate hydratase [EC:4.2.1.49] | ec:4.2.1.49 |
| ddd:Dda3937\_01661 | hutH; histidine ammonia-lyase; K01745 histidine ammonia-lyase [EC:4.3.1.3] | ec:4.3.1.3 |
| ddd:Dda3937\_01662 | tauB; Urea carboxylase-related ABC transporter ATPase; K02049 NitT/TauT family transport system ATP-binding protein |  |
| ddd:Dda3937\_01663 | Urea carboxylase-related ABC transporter permease; K02050 NitT/TauT family transport system permease protein |  |
| ddd:Dda3937\_01664 | Urea carboxylase-related ABC transporter substrate-binding protein; K02051 NitT/TauT family transport system substrate-binding protein |  |
| ddd:Dda3937\_04368 | hypothetical protein |  |
| ddd:Dda3937\_01665 | hypothetical protein |  |
| ddd:Dda3937\_01666 | yhfR; histidine utilization repressor; K05836 GntR family transcriptional regulator, histidine utilization repressor |  |
| ddd:Dda3937\_01667 | histidine ammonia-lyase; K01745 histidine ammonia-lyase [EC:4.3.1.3] | ec:4.3.1.3 |
| ddd:Dda3937\_01668 | histidine ABC transporter ATP-binding protein; K10021 octopine/nopaline transport system ATP-binding protein [EC:3.6.3.-] |  |
| ddd:Dda3937\_01669 | Octopine transport system permease occM; K10019 octopine/nopaline transport system permease protein |  |
| ddd:Dda3937\_01670 | Octopine transport system permease occQ; K10020 octopine/nopaline transport system permease protein |  |
| ddd:Dda3937\_01671 | ABC transporter substrate-binding protein; K10018 octopine/nopaline transport system substrate-binding protein |  |
| ddd:Dda3937\_01672 | ydjR; hypothetical protein; K09975 hypothetical protein |  |
| ddd:Dda3937\_01673 | Formiminoglutamic iminohydrolase; K05603 formimidoylglutamate deiminase [EC:3.5.3.13] | ec:3.5.3.13 |
| ddd:Dda3937\_01674 | Imidazolonepropionase; K01468 imidazolonepropionase [EC:3.5.2.7] | ec:3.5.2.7 |
| ddd:Dda3937\_01675 | N-formylglutamate deformylase; K01479 formiminoglutamase [EC:3.5.3.8] | ec:3.5.3.8 |
| ddd:Dda3937\_04369 | methyl-accepting chemotaxis protein |  |

  
**Neighborhood Representations for "ddc:Dd586\_2950"**  

| ID | Annotation | EC number |
| --- | --- | --- |
| ddc:Dd586\_2940 | glucuronate isomerase (EC:5.3.1.12); K01812 glucuronate isomerase [EC:5.3.1.12] | ec:5.3.1.12 |
| ddc:Dd586\_2941 | hypothetical protein; K16210 oligogalacturonide transporter |  |
| ddc:Dd586\_2942 | mannitol dehydrogenase domain-containing protein; K00040 fructuronate reductase [EC:1.1.1.57] | ec:1.1.1.57 |
| ddc:Dd586\_2943 | GntR family transcriptional regulator |  |
| ddc:Dd586\_2944 | urocanate hydratase (EC:4.2.1.49); K01712 urocanate hydratase [EC:4.2.1.49] | ec:4.2.1.49 |
| ddc:Dd586\_2945 | histidine ammonia-lyase (EC:4.3.1.3); K01745 histidine ammonia-lyase [EC:4.3.1.3] | ec:4.3.1.3 |
| ddc:Dd586\_2946 | ABC transporter; K02049 NitT/TauT family transport system ATP-binding protein |  |
| ddc:Dd586\_2947 | binding-protein-dependent transporter inner membrane component; K02050 NitT/TauT family transport system permease protein |  |
| ddc:Dd586\_2948 | glycine/betaine ABC transporter substrate-binding protein; K02051 NitT/TauT family transport system substrate-binding protein |  |
| ddc:Dd586\_2949 | hypothetical protein |  |
| ddc:Dd586\_2950 | GntR family transcriptional regulator; K05836 GntR family transcriptional regulator, histidine utilization repressor |  |
| ddc:Dd586\_2951 | putative sugar-specific permease SgaT/UlaA; K03475 PTS system, ascorbate-specific IIC component |  |
| ddc:Dd586\_2952 | signal transduction histidine kinase regulating citrate/malate metabolism |  |
| ddc:Dd586\_2953 | response regulator receiver |  |
| ddc:Dd586\_2954 | histidine ammonia-lyase (EC:4.3.1.3); K01745 histidine ammonia-lyase [EC:4.3.1.3] | ec:4.3.1.3 |
| ddc:Dd586\_2955 | ABC transporter; K10021 octopine/nopaline transport system ATP-binding protein [EC:3.6.3.-] |  |
| ddc:Dd586\_2956 | polar amino acid ABC transporter permease; K10019 octopine/nopaline transport system permease protein |  |
| ddc:Dd586\_2957 | polar amino acid ABC transporter permease; K10020 octopine/nopaline transport system permease protein |  |
| ddc:Dd586\_2958 | family 3 extracellular solute-binding protein; K10018 octopine/nopaline transport system substrate-binding protein |  |
| ddc:Dd586\_2959 | hypothetical protein; K09975 hypothetical protein |  |
| ddc:Dd586\_2960 | formiminoglutamate deiminase; K05603 formimidoylglutamate deiminase [EC:3.5.3.13] | ec:3.5.3.13 |

  
**Neighborhood Representations for "dze:Dd1591\_1261"**  

| ID | Annotation | EC number |
| --- | --- | --- |
| dze:Dd1591\_1251 | methyl-accepting chemotaxis sensory transducer |  |
| dze:Dd1591\_1252 | N-formylglutamate amidohydrolase; K01479 formiminoglutamase [EC:3.5.3.8] | ec:3.5.3.8 |
| dze:Dd1591\_1253 | imidazolonepropionase (EC:3.5.2.7); K01468 imidazolonepropionase [EC:3.5.2.7] | ec:3.5.2.7 |
| dze:Dd1591\_1254 | N-formimino-L-glutamate deiminase; K05603 formimidoylglutamate deiminase [EC:3.5.3.13] | ec:3.5.3.13 |
| dze:Dd1591\_1255 | hypothetical protein; K09975 hypothetical protein |  |
| dze:Dd1591\_1256 | extracellular solute-binding protein family 3; K10018 octopine/nopaline transport system substrate-binding protein |  |
| dze:Dd1591\_1257 | polar amino acid ABC transporter permease; K10020 octopine/nopaline transport system permease protein |  |
| dze:Dd1591\_1258 | polar amino acid ABC transporter permease; K10019 octopine/nopaline transport system permease protein |  |
| dze:Dd1591\_1259 | ABC transporter; K10021 octopine/nopaline transport system ATP-binding protein [EC:3.6.3.-] |  |
| dze:Dd1591\_1260 | histidine ammonia-lyase (EC:4.3.1.3); K01745 histidine ammonia-lyase [EC:4.3.1.3] | ec:4.3.1.3 |
| dze:Dd1591\_1261 | GntR family transcriptional regulator; K05836 GntR family transcriptional regulator, histidine utilization repressor |  |
| dze:Dd1591\_1262 | hypothetical protein |  |
| dze:Dd1591\_1263 | pseudogene |  |
| dze:Dd1591\_1264 | histidine ammonia-lyase (EC:4.3.1.3); K01745 histidine ammonia-lyase [EC:4.3.1.3] | ec:4.3.1.3 |
| dze:Dd1591\_1265 | urocanate hydratase (EC:4.2.1.49); K01712 urocanate hydratase [EC:4.2.1.49] | ec:4.2.1.49 |
| dze:Dd1591\_1266 | GntR family transcriptional regulator |  |
| dze:Dd1591\_1267 | Fructuronate reductase (EC:1.1.1.57); K00040 fructuronate reductase [EC:1.1.1.57] | ec:1.1.1.57 |
| dze:Dd1591\_1268 | major facilitator superfamily protein; K08173 MFS transporter, MHS family, metabolite:H+ symporter |  |
| dze:Dd1591\_1269 | alcohol dehydrogenase GroES domain-containing protein; K08322 starvation sensing protein RspB [EC:1.1.1.-] |  |
| dze:Dd1591\_1270 | mandelate racemase/muconate lactonizing protein; K08323 starvation sensing protein RspA |  |
| dze:Dd1591\_1271 | hypothetical protein |  |

  
**Neighborhood Representations for "dda:Dd703\_2647"**  

| ID | Annotation | EC number |
| --- | --- | --- |
| dda:Dd703\_2637 | major facilitator superfamily protein; K08173 MFS transporter, MHS family, metabolite:H+ symporter |  |
| dda:Dd703\_2638 | mannitol dehydrogenase; K00040 fructuronate reductase [EC:1.1.1.57] | ec:1.1.1.57 |
| dda:Dd703\_2639 | GntR family transcriptional regulator |  |
| dda:Dd703\_2640 | hypothetical protein |  |
| dda:Dd703\_2641 | urocanate hydratase (EC:4.2.1.49); K01712 urocanate hydratase [EC:4.2.1.49] | ec:4.2.1.49 |
| dda:Dd703\_2642 | histidine ammonia-lyase (EC:4.3.1.3); K01745 histidine ammonia-lyase [EC:4.3.1.3] | ec:4.3.1.3 |
| dda:Dd703\_2643 | ABC transporter; K02049 NitT/TauT family transport system ATP-binding protein |  |
| dda:Dd703\_2644 | binding-protein-dependent transporters inner membrane component; K02050 NitT/TauT family transport system permease protein |  |
| dda:Dd703\_2645 | glycine/betaine ABC transporter substrate-binding protein; K02051 NitT/TauT family transport system substrate-binding protein |  |
| dda:Dd703\_2646 | peptidase S14 ClpP |  |
| dda:Dd703\_2647 | GntR family transcriptional regulator; K05836 GntR family transcriptional regulator, histidine utilization repressor |  |
| dda:Dd703\_2648 | histidine ammonia-lyase (EC:4.3.1.3); K01745 histidine ammonia-lyase [EC:4.3.1.3] | ec:4.3.1.3 |
| dda:Dd703\_2649 | ABC transporter; K10021 octopine/nopaline transport system ATP-binding protein [EC:3.6.3.-] |  |
| dda:Dd703\_2650 | polar amino acid ABC transporter permease; K10019 octopine/nopaline transport system permease protein |  |
| dda:Dd703\_2651 | polar amino acid ABC transporter permease; K10020 octopine/nopaline transport system permease protein |  |
| dda:Dd703\_2652 | family 3 extracellular solute-binding protein; K10018 octopine/nopaline transport system substrate-binding protein |  |
| dda:Dd703\_2653 | hypothetical protein; K09975 hypothetical protein |  |
| dda:Dd703\_2654 | N-formimino-L-glutamate deiminase; K05603 formimidoylglutamate deiminase [EC:3.5.3.13] | ec:3.5.3.13 |
| dda:Dd703\_2655 | imidazolonepropionase (EC:3.5.2.7); K01468 imidazolonepropionase [EC:3.5.2.7] | ec:3.5.2.7 |
| dda:Dd703\_2656 | N-formylglutamate amidohydrolase; K01479 formiminoglutamase [EC:3.5.3.8] | ec:3.5.3.8 |
| dda:Dd703\_2657 | methyl-accepting chemotaxis sensory transducer |  |

  
**Neighborhood Representations for "sen:SACE\_3198"**  

| ID | Annotation | EC number |
| --- | --- | --- |
| sen:SACE\_3187 | terpene synthase metal-binding domain-containing protein; K10187 germacradienol/geosmin synthase [EC:4.2.3.22 4.2.3.75 4.1.99.16] | ec:4.1.99.16 ec:4.2.3.75 ec:4.2.3.22 |
| sen:SACE\_3188 | hypothetical protein |  |
| sen:SACE\_3189 | cytochrome P450 |  |
| sen:SACE\_3190 | hypothetical protein |  |
| sen:SACE\_3192 | pknD; serine/threonine-protein kinase (EC:2.7.11.1); K08884 serine/threonine protein kinase, bacterial [EC:2.7.11.1] | ec:2.7.11.1 |
| sen:SACE\_3193 | peptide ABC transporter permease; K02004 putative ABC transport system permease protein |  |
| sen:SACE\_3194 | peptide ABC transporter ATPase; K02003 putative ABC transport system ATP-binding protein |  |
| sen:SACE\_3195 | hypothetical protein |  |
| sen:SACE\_3196 | leuB; 3-isopropylmalate dehydrogenase (EC:1.1.1.85); K00052 3-isopropylmalate dehydrogenase [EC:1.1.1.85] | ec:1.1.1.85 |
| sen:SACE\_3197 | proY; aromatic amino acid permease; K11736 proline-specific permease ProY |  |
| sen:SACE\_3198 | hutC; histidine utilization genes repressor protein; K05836 GntR family transcriptional regulator, histidine utilization repressor |  |
| sen:SACE\_3199 | hypothetical protein |  |
| sen:SACE\_3200 | hypothetical protein |  |
| sen:SACE\_3201 | hypothetical protein |  |
| sen:SACE\_3202 | hypothetical protein |  |
| sen:SACE\_3203 | UDP-glucose/GDP-mannose dehydrogenase |  |
| sen:SACE\_3204 | calcium binding protein |  |
| sen:SACE\_3205 | transmembrane efflux protein |  |
| sen:SACE\_3206 | hypothetical protein |  |
| sen:SACE\_3207 | oxidoreductase domain-containing protein |  |
| sen:SACE\_3208 | malate/L-lactate dehydrogenase |  |

  
**Neighborhood Representations for "xne:XNC1\_3188"**  

| ID | Annotation | EC number |
| --- | --- | --- |
| xne:XNC1\_3178 | hypothetical protein |  |
| xne:XNC1\_3179 | hypothetical protein |  |
| xne:XNC1\_3180 | hypothetical protein |  |
| xne:XNC1\_3181 | hypothetical protein; K09906 hypothetical protein |  |
| xne:XNC1\_3182 | yfcA; hypothetical protein; K07090 |  |
| xne:XNC1\_3183 | aroC; chorismate synthase (EC:4.2.3.5); K01736 chorismate synthase [EC:4.2.3.5] | ec:4.2.3.5 |
| xne:XNC1\_3184 | prmB; N5-glutamine methyltransferase (EC:1.3.3.-); K07320 putative adenine-specific DNA-methyltransferase [EC:2.1.1.72] | ec:2.1.1.72 |
| xne:XNC1\_3185 | proY; proline transport protein; K11736 proline-specific permease ProY |  |
| xne:XNC1\_3186 | Histidine ammonia-lyase (EC:4.3.1.3); K01745 histidine ammonia-lyase [EC:4.3.1.3] | ec:4.3.1.3 |
| xne:XNC1\_3187 | urocanate hydratase (EC:4.2.1.49); K01712 urocanate hydratase [EC:4.2.1.49] | ec:4.2.1.49 |
| xne:XNC1\_3188 | Histidine utilization repressor; K05836 GntR family transcriptional regulator, histidine utilization repressor |  |
| xne:XNC1\_3189 | formimidoylglutamase (EC:3.5.3.8); K01479 formiminoglutamase [EC:3.5.3.8] | ec:3.5.3.8 |
| xne:XNC1\_3190 | imidazolonepropionase (EC:3.5.2.7); K01468 imidazolonepropionase [EC:3.5.2.7] | ec:3.5.2.7 |
| xne:XNC1\_3191 | yfcN; phage-like protein |  |
| xne:XNC1\_3192 | sixA; phosphohistidine phosphatase (EC:3.1.3.-); K08296 phosphohistidine phosphatase [EC:3.1.3.-] |  |
| xne:XNC1\_3193 | hypothetical protein |  |
| xne:XNC1\_3194 | fadJ; bifunctional anaerobic fatty acid oxidation complex protein enoyl-CoA hydratase/epimerase/isomerase/3-hydroxyacyl-CoA dehydrogenase (EC:1.1.1.35 4.2.1.17 5.3.3.8); K01782 3-hydroxyacyl-CoA dehydrogenase / enoyl-CoA hydratase / 3-hydroxybutyryl-CoA epimerase [EC:1.1.1.35 4.2.1.17 5.1.2.3] | ec:5.1.2.3 ec:4.2.1.17 ec:1.1.1.35 |
| xne:XNC1\_3195 | fadI; beta-keto thiolase (EC:2.3.1.16); K00632 acetyl-CoA acyltransferase [EC:2.3.1.16] | ec:2.3.1.16 |
| xne:XNC1\_3196 | hypothetical protein |  |
| xne:XNC1\_3197 | fadL; outer membrane porin (EC:3.1.1.35); K06076 long-chain fatty acid transport protein |  |
| xne:XNC1\_3198 | vacJ; lipoprotein; K04754 lipoprotein |  |

  
**Neighborhood Representations for "pva:Pvag\_0646"**  

| ID | Annotation | EC number |
| --- | --- | --- |
| pva:Pvag\_0636 | yehW; ABC transporter permease; K05846 osmoprotectant transport system permease protein |  |
| pva:Pvag\_0637 | hypothetical protein |  |
| pva:Pvag\_0638 | yehV; hypothetical protein |  |
| pva:Pvag\_0639 | hypothetical protein |  |
| pva:Pvag\_0640 | ttr; GNAT family acetyltransferase (EC:2.3.1.-) |  |
| pva:Pvag\_0641 | dinG; ATP-dependent helicase dinG (EC:3.6.1.-); K03722 ATP-dependent DNA helicase DinG [EC:3.6.4.12] | ec:3.6.4.12 |
| pva:Pvag\_0642 | ybiB; hypothetical protein |  |
| pva:Pvag\_0643 | hipo1; peptidase M20D, amidohydrolase (EC:3.5.1.32); K01451 hippurate hydrolase [EC:3.5.1.32] | ec:3.5.1.32 |
| pva:Pvag\_0644 | hutU; urocanate hydratase (EC:4.2.1.49); K01712 urocanate hydratase [EC:4.2.1.49] | ec:4.2.1.49 |
| pva:Pvag\_0645 | huth1; histidine ammonia-lyase (EC:4.3.1.3); K01745 histidine ammonia-lyase [EC:4.3.1.3] | ec:4.3.1.3 |
| pva:Pvag\_0646 | hutC; histidine utilization repressor; K05836 GntR family transcriptional regulator, histidine utilization repressor |  |
| pva:Pvag\_0647 | ydjR; hypothetical protein; K09975 hypothetical protein |  |
| pva:Pvag\_0648 | hutF; formiminoglutamate deiminase (EC:3.5.3.13); K05603 formimidoylglutamate deiminase [EC:3.5.3.13] | ec:3.5.3.13 |
| pva:Pvag\_0649 | hutI; imidazolonepropionase (EC:3.5.2.7); K01468 imidazolonepropionase [EC:3.5.2.7] | ec:3.5.2.7 |
| pva:Pvag\_0650 | hutG; N-formylglutamate amidohydrolase (EC:3.5.3.8); K01479 formiminoglutamase [EC:3.5.3.8] | ec:3.5.3.8 |
| pva:Pvag\_0651 | ABC transporter substrate-binding protein; K10018 octopine/nopaline transport system substrate-binding protein |  |
| pva:Pvag\_0652 | ABC transporter permease; K10020 octopine/nopaline transport system permease protein |  |
| pva:Pvag\_0653 | ABC transporter permease; K10019 octopine/nopaline transport system permease protein |  |
| pva:Pvag\_0654 | ABC transporter ATPase (EC:3.6.3.-); K10021 octopine/nopaline transport system ATP-binding protein [EC:3.6.3.-] |  |
| pva:Pvag\_0655 | huth3; histidine ammonia-lyase (EC:4.3.1.3); K01745 histidine ammonia-lyase [EC:4.3.1.3] | ec:4.3.1.3 |
| pva:Pvag\_0656 | hypothetical protein |  |

  
**Neighborhood Representations for "plu:plu3195"**  

| ID | Annotation | EC number |
| --- | --- | --- |
| plu:plu3185 | mnmC; 5-methylaminomethyl-2-thiouridine methyltransferase; K15461 tRNA 5-methylaminomethyl-2-thiouridine biosynthesis bifunctional protein [EC:2.1.1.61 1.5.-.-] | ec:2.1.1.61 |
| plu:plu3186 | hypothetical protein |  |
| plu:plu3187 | hypothetical protein; K09906 hypothetical protein |  |
| plu:plu3188 | hypothetical protein; K07090 |  |
| plu:plu3189 | aroC; chorismate synthase (EC:4.2.3.5); K01736 chorismate synthase [EC:4.2.3.5] | ec:4.2.3.5 |
| plu:plu3190 | N5-glutamine S-adenosyl-L-methionine-dependent methyltransferase; K07320 putative adenine-specific DNA-methyltransferase [EC:2.1.1.72] | ec:2.1.1.72 |
| plu:plu3191 | hypothetical protein; K11736 proline-specific permease ProY |  |
| plu:plu3192 | hutH; histidine ammonia-lyase (EC:4.3.1.3); K01745 histidine ammonia-lyase [EC:4.3.1.3] | ec:4.3.1.3 |
| plu:plu3193 | hutU; urocanate hydratase (EC:4.2.1.49); K01712 urocanate hydratase [EC:4.2.1.49] | ec:4.2.1.49 |
| plu:plu3194 | hypothetical protein |  |
| plu:plu3195 | hutC; histidine utilization repressor protein; K05836 GntR family transcriptional regulator, histidine utilization repressor |  |
| plu:plu3196 | hutG; formimidoylglutamase (EC:3.5.3.8); K01479 formiminoglutamase [EC:3.5.3.8] | ec:3.5.3.8 |
| plu:plu3197 | hutI; imidazolonepropionase (EC:3.5.2.7); K01468 imidazolonepropionase [EC:3.5.2.7] | ec:3.5.2.7 |
| plu:plu3198 | hypothetical protein |  |
| plu:plu3199 | sixA; phosphohistidine phosphatase; K08296 phosphohistidine phosphatase [EC:3.1.3.-] |  |
| plu:plu3200 | fadJ; multifunctional fatty acid oxidation complex subunit alpha (EC:1.1.1.35 4.2.1.17 5.1.2.3); K01782 3-hydroxyacyl-CoA dehydrogenase / enoyl-CoA hydratase / 3-hydroxybutyryl-CoA epimerase [EC:1.1.1.35 4.2.1.17 5.1.2.3] | ec:5.1.2.3 ec:4.2.1.17 ec:1.1.1.35 |
| plu:plu3201 | fadI; 3-ketoacyl-CoA thiolase (EC:2.3.1.16); K00632 acetyl-CoA acyltransferase [EC:2.3.1.16] | ec:2.3.1.16 |
| plu:plu3202 | fadL; long-chain fatty acid outer membrane transporter; K06076 long-chain fatty acid transport protein |  |
| plu:plu3203 | vacJ; VacJ lipoprotein precursor; K04754 lipoprotein |  |
| plu:plut058 | tRNA-Arg; tRNA-Arg; K14219 tRNA Arg |  |
| plu:plu3204 | hypothetical protein |  |

  
**Neighborhood Representations for "axy:AXYL\_00282"**  

| ID | Annotation | EC number |
| --- | --- | --- |
| axy:AXYL\_00272 | AsnC family transcriptional regulator |  |
| axy:AXYL\_00273 | arcB; ornithine cyclodeaminase (EC:4.3.1.12); K01750 ornithine cyclodeaminase [EC:4.3.1.12] | ec:4.3.1.12 |
| axy:AXYL\_00274 | hypothetical protein |  |
| axy:AXYL\_00275 | GTPase |  |
| axy:AXYL\_00276 | panB; methyltransferase (EC:2.1.2.11); K00606 3-methyl-2-oxobutanoate hydroxymethyltransferase [EC:2.1.2.11] | ec:2.1.2.11 |
| axy:AXYL\_00277 | hypothetical protein |  |
| axy:AXYL\_00278 | glycosyl transferase group 1 |  |
| axy:AXYL\_00279 | ushA; 5'-nucleotidase (EC:3.1.3.5); K01081 5'-nucleotidase [EC:3.1.3.5] | ec:3.1.3.5 |
| axy:AXYL\_00280 | adhC; alcohol dehydrogenase (EC:1.1.1.1 1.1.1.284); K00121 S-(hydroxymethyl)glutathione dehydrogenase / alcohol dehydrogenase [EC:1.1.1.284 1.1.1.1] | ec:1.1.1.284 ec:1.1.1.1 |
| axy:AXYL\_00281 | frmR; regulator protein FrmR |  |
| axy:AXYL\_00282 | hutC; histidine utilization repressor; K05836 GntR family transcriptional regulator, histidine utilization repressor |  |
| axy:AXYL\_00283 | hypothetical protein; K09975 hypothetical protein |  |
| axy:AXYL\_00284 | hutU1; urocanate hydratase 1 (EC:4.2.1.49); K01712 urocanate hydratase [EC:4.2.1.49] | ec:4.2.1.49 |
| axy:AXYL\_00285 | permease for cytosine/purines, uracil, thiamine, allantoin family protein; K03457 nucleobase:cation symporter-1, NCS1 family |  |
| axy:AXYL\_00286 | hutH; histidine ammonia-lyase (EC:4.3.1.3); K01745 histidine ammonia-lyase [EC:4.3.1.3] | ec:4.3.1.3 |
| axy:AXYL\_00287 | hutI; imidazolonepropionase (EC:3.5.2.7); K01468 imidazolonepropionase [EC:3.5.2.7] | ec:3.5.2.7 |
| axy:AXYL\_00288 | amidohydrolase |  |
| axy:AXYL\_00289 | major facilitator protein; K08169 MFS transporter, DHA2 family, multidrug resistance protein |  |
| axy:AXYL\_00290 | tripartite tricarboxylate transporter family receptor 1 |  |
| axy:AXYL\_00291 | major facilitator protein |  |
| axy:AXYL\_00292 | sulfite:cytochrome C oxidoreductase subunit B |  |

  
**Neighborhood Representations for "pap:PSPA7\_5838"**  

| ID | Annotation | EC number |
| --- | --- | --- |
| pap:PSPA7\_5828 | ABC transporter ATP-binding protein; K02000 glycine betaine/proline transport system ATP-binding protein [EC:3.6.3.32] | ec:3.6.3.32 |
| pap:PSPA7\_5829 | ABC transporter permease; K02001 glycine betaine/proline transport system permease protein |  |
| pap:PSPA7\_5830 | ABC transporter substrate-binding protein; K02002 glycine betaine/proline transport system substrate-binding protein |  |
| pap:PSPA7\_5831 | hutH1; histidine ammonia-lyase (EC:4.3.1.3); K01745 histidine ammonia-lyase [EC:4.3.1.3] | ec:4.3.1.3 |
| pap:PSPA7\_5832 | cytosine/purines/uracil/thiamine/allantoin permease family protein; K03457 nucleobase:cation symporter-1, NCS1 family |  |
| pap:PSPA7\_5833 | hutU; urocanate hydratase (EC:4.2.1.49); K01712 urocanate hydratase [EC:4.2.1.49] | ec:4.2.1.49 |
| pap:PSPA7\_5834 | hypothetical protein |  |
| pap:PSPA7\_5835 | fatty acid desaturase domain-containing protein |  |
| pap:PSPA7\_5836 | hypothetical protein; K02002 glycine betaine/proline transport system substrate-binding protein |  |
| pap:PSPA7\_5837 | hypothetical protein; K09975 hypothetical protein |  |
| pap:PSPA7\_5838 | hutC; histidine utilization repressor; K05836 GntR family transcriptional regulator, histidine utilization repressor |  |
| pap:PSPA7\_5839 | hutF; N-formimino-L-glutamate deiminase (EC:3.5.3.13); K05603 formimidoylglutamate deiminase [EC:3.5.3.13] | ec:3.5.3.13 |
| pap:PSPA7\_5840 | blc; outer membrane lipoprotein Blc; K03098 apolipoprotein D and lipocalin family protein |  |
| pap:PSPA7\_5841 | putative lipoprotein |  |
| pap:PSPA7\_5842 | hypothetical protein |  |
| pap:PSPA7\_5843 | fbp; fructose-1,6-bisphosphatase; K03841 fructose-1,6-bisphosphatase I [EC:3.1.3.11] | ec:3.1.3.11 |
| pap:PSPA7\_5844 | gloA1; lactoylglutathione lyase (EC:4.4.1.5); K01759 lactoylglutathione lyase [EC:4.4.1.5] | ec:4.4.1.5 |
| pap:PSPA7\_5845 | estA; esterase EstA; K12686 outer membrane lipase/esterase |  |
| pap:PSPA7\_5846 | hypothetical protein |  |
| pap:PSPA7\_5847 | hypothetical protein |  |
| pap:PSPA7\_5848 | hypothetical protein |  |

  
**Neighborhood Representations for "ent:Ent638\_1262"**  

| ID | Annotation | EC number |
| --- | --- | --- |
| ent:Ent638\_1252 | DNA-binding transcriptional regulator ModE; K02019 molybdate transport system regulatory protein |  |
| ent:Ent638\_1253 | hypothetical protein |  |
| ent:Ent638\_1254 | modA; molybdate transporter periplasmic protein; K02020 molybdate transport system substrate-binding protein |  |
| ent:Ent638\_1255 | modB; molybdate ABC transporter permease; K02018 molybdate transport system permease protein |  |
| ent:Ent638\_1256 | modC; molybdate transporter ATP-binding protein (EC:3.6.3.29); K02017 molybdate transport system ATP-binding protein [EC:3.6.3.29] | ec:3.6.3.29 |
| ent:Ent638\_1257 | phosphotransferase; K07024 |  |
| ent:Ent638\_1258 | 6-phosphogluconolactonase (EC:3.1.1.31); K07404 6-phosphogluconolactonase [EC:3.1.1.31] | ec:3.1.1.31 |
| ent:Ent638\_1259 | pectinesterase; K01051 pectinesterase [EC:3.1.1.11] | ec:3.1.1.11 |
| ent:Ent638\_1260 | imidazolonepropionase (EC:3.5.2.7); K01468 imidazolonepropionase [EC:3.5.2.7] | ec:3.5.2.7 |
| ent:Ent638\_1261 | formimidoylglutamase (EC:3.5.3.8); K01479 formiminoglutamase [EC:3.5.3.8] | ec:3.5.3.8 |
| ent:Ent638\_1262 | GntR family transcriptional regulator; K05836 GntR family transcriptional regulator, histidine utilization repressor |  |
| ent:Ent638\_1263 | urocanate hydratase (EC:4.2.1.49); K01712 urocanate hydratase [EC:4.2.1.49] | ec:4.2.1.49 |
| ent:Ent638\_1264 | histidine ammonia-lyase (EC:4.3.1.3); K01745 histidine ammonia-lyase [EC:4.3.1.3] | ec:4.3.1.3 |
| ent:Ent638\_1265 | adenosylmethionine-8-amino-7-oxononanoate aminotransferase (EC:2.6.1.62); K00833 adenosylmethionine-8-amino-7-oxononanoate aminotransferase [EC:2.6.1.62] | ec:2.6.1.62 |
| ent:Ent638\_1266 | biotin synthase (EC:2.8.1.6); K01012 biotin synthase [EC:2.8.1.6] | ec:2.8.1.6 |
| ent:Ent638\_1267 | 8-amino-7-oxononanoate synthase (EC:2.3.1.47); K00652 8-amino-7-oxononanoate synthase [EC:2.3.1.47] | ec:2.3.1.47 |
| ent:Ent638\_1268 | biotin biosynthesis protein BioC; K02169 malonyl-CoA O-methyltransferase [EC:2.1.1.197] | ec:2.1.1.197 |
| ent:Ent638\_1269 | bioD; dithiobiotin synthetase (EC:6.3.3.3); K01935 dethiobiotin synthetase [EC:6.3.3.3] | ec:6.3.3.3 |
| ent:Ent638\_1270 | ABC transporter |  |
| ent:Ent638\_1271 | excinuclease ABC subunit B; K03702 excinuclease ABC subunit B |  |
| ent:Ent638\_1272 | hypothetical protein |  |

  
**Neighborhood Representations for "pam:PANA\_1266"**  

| ID | Annotation | EC number |
| --- | --- | --- |
| pam:PANA\_1256 | ydcN; hypothetical protein |  |
| pam:PANA\_1257 | ttr; Ttr |  |
| pam:PANA\_1258 | dinG; DinG; K03722 ATP-dependent DNA helicase DinG [EC:3.6.4.12] | ec:3.6.4.12 |
| pam:PANA\_1259 | ybiB; hypothetical protein |  |
| pam:PANA\_1260 | hipO; HipO; K01451 hippurate hydrolase [EC:3.5.1.32] | ec:3.5.1.32 |
| pam:PANA\_1261 | idnR; IdnR; K06145 LacI family transcriptional regulator, gluconate utilization system Gnt-I transcriptional repressor |  |
| pam:PANA\_1262 | mdlA; MdlA |  |
| pam:PANA\_1263 | gudP; GudP; K03535 MFS transporter, ACS family, glucarate transporter |  |
| pam:PANA\_1264 | hypothetical Protein; K01712 urocanate hydratase [EC:4.2.1.49] | ec:4.2.1.49 |
| pam:PANA\_1265 | hutH; HutH; K01745 histidine ammonia-lyase [EC:4.3.1.3] | ec:4.3.1.3 |
| pam:PANA\_1266 | hutC; HutC; K05836 GntR family transcriptional regulator, histidine utilization repressor |  |
| pam:PANA\_1267 | ydjR; hypothetical protein; K09975 hypothetical protein |  |
| pam:PANA\_1268 | hypothetical Protein; K05603 formimidoylglutamate deiminase [EC:3.5.3.13] | ec:3.5.3.13 |
| pam:PANA\_1269 | hutI; HutI; K01468 imidazolonepropionase [EC:3.5.2.7] | ec:3.5.2.7 |
| pam:PANA\_1270 | hypothetical Protein; K01479 formiminoglutamase [EC:3.5.3.8] | ec:3.5.3.8 |
| pam:PANA\_1271 | nocT; NocT |  |
| pam:PANA\_1272 | occT; OccT; K10018 octopine/nopaline transport system substrate-binding protein |  |
| pam:PANA\_1273 | occQ; OccQ; K10020 octopine/nopaline transport system permease protein |  |
| pam:PANA\_1274 | occM; OccM; K10019 octopine/nopaline transport system permease protein |  |
| pam:PANA\_1275 | hisP; HisP; K10021 octopine/nopaline transport system ATP-binding protein [EC:3.6.3.-] |  |
| pam:PANA\_1276 | hutH; HutH; K01745 histidine ammonia-lyase [EC:4.3.1.3] | ec:4.3.1.3 |

  
**Neighborhood Representations for "pae:PA5105"**  

| ID | Annotation | EC number |
| --- | --- | --- |
| pae:PA5095 | ABC transporter permease; K02001 glycine betaine/proline transport system permease protein |  |
| pae:PA5096 | ABC transporter; K02002 glycine betaine/proline transport system substrate-binding protein |  |
| pae:PA5097 | amino acid permease; K11736 proline-specific permease ProY |  |
| pae:PA5098 | hutH; histidine ammonia-lyase (EC:4.3.1.3); K01745 histidine ammonia-lyase [EC:4.3.1.3] | ec:4.3.1.3 |
| pae:PA5099 | transporter; K03457 nucleobase:cation symporter-1, NCS1 family |  |
| pae:PA5100 | hutU; urocanate hydratase (EC:4.2.1.49); K01712 urocanate hydratase [EC:4.2.1.49] | ec:4.2.1.49 |
| pae:PA5101 | hypothetical protein |  |
| pae:PA5102 | hypothetical protein |  |
| pae:PA5103 | hypothetical protein; K02002 glycine betaine/proline transport system substrate-binding protein |  |
| pae:PA5104 | hypothetical protein; K09975 hypothetical protein |  |
| pae:PA5105 | hutC; histidine utilization repressor HutC; K05836 GntR family transcriptional regulator, histidine utilization repressor |  |
| pae:PA5106 | N-formimino-L-glutamate deiminase; K05603 formimidoylglutamate deiminase [EC:3.5.3.13] | ec:3.5.3.13 |
| pae:PA5107 | blc; outer membrane lipoprotein Blc; K03098 apolipoprotein D and lipocalin family protein |  |
| pae:PA5108 | hypothetical protein |  |
| pae:PA5109 | hypothetical protein |  |
| pae:PA5110 | fbp; fructose-1,6-bisphosphatase (EC:3.1.3.11); K03841 fructose-1,6-bisphosphatase I [EC:3.1.3.11] | ec:3.1.3.11 |
| pae:PA5111 | gloA3; lactoylglutathione lyase; K01759 lactoylglutathione lyase [EC:4.4.1.5] | ec:4.4.1.5 |
| pae:PA5112 | estA; esterase; K12686 outer membrane lipase/esterase |  |
| pae:PA5113 | hypothetical protein |  |
| pae:PA5114 | hypothetical protein |  |
| pae:PA5115 | hypothetical protein |  |

  
**Neighborhood Representations for "pag:PLES\_54951"**  

| ID | Annotation | EC number |
| --- | --- | --- |
| pag:PLES\_54851 | ABC transporter permease; K02001 glycine betaine/proline transport system permease protein |  |
| pag:PLES\_54861 | putative binding protein component of ABC transporter; K02002 glycine betaine/proline transport system substrate-binding protein |  |
| pag:PLES\_54871 | putative amino acid permease; K11736 proline-specific permease ProY |  |
| pag:PLES\_54881 | hutH; histidine ammonia-lyase; K01745 histidine ammonia-lyase [EC:4.3.1.3] | ec:4.3.1.3 |
| pag:PLES\_54891 | putative transporter; K03457 nucleobase:cation symporter-1, NCS1 family |  |
| pag:PLES\_54901 | hutU; urocanate hydratase; K01712 urocanate hydratase [EC:4.2.1.49] | ec:4.2.1.49 |
| pag:PLES\_54911 | putative ABC-type phosphate/phosphonate transport system, periplasmic component |  |
| pag:PLES\_54921 | putative fatty acid desaturase |  |
| pag:PLES\_54931 | putative ABC transporter periplasmic substrate-binding protein; K02002 glycine betaine/proline transport system substrate-binding protein |  |
| pag:PLES\_54941 | hypothetical protein; K09975 hypothetical protein |  |
| pag:PLES\_54951 | hutC; histidine utilization repressor HutC; K05836 GntR family transcriptional regulator, histidine utilization repressor |  |
| pag:PLES\_54961 | N-formimino-L-glutamate deiminase; K05603 formimidoylglutamate deiminase [EC:3.5.3.13] | ec:3.5.3.13 |
| pag:PLES\_54971 | blc; outer membrane lipoprotein Blc; K03098 apolipoprotein D and lipocalin family protein |  |
| pag:PLES\_54981 | putative lipoprotein |  |
| pag:PLES\_54991 | hypothetical protein |  |
| pag:PLES\_55001 | fbp; fructose-1,6-bisphosphatase; K03841 fructose-1,6-bisphosphatase I [EC:3.1.3.11] | ec:3.1.3.11 |
| pag:PLES\_55011 | gloA3; lactoylglutathione lyase; K01759 lactoylglutathione lyase [EC:4.4.1.5] | ec:4.4.1.5 |
| pag:PLES\_55021 | estA; esterase EstA; K12686 outer membrane lipase/esterase |  |
| pag:PLES\_55031 | hypothetical protein |  |
| pag:PLES\_55041 | hypothetical protein |  |
| pag:PLES\_55051 | hypothetical protein |  |

  
**Neighborhood Representations for "pau:PA14\_67420"**  

| ID | Annotation | EC number |
| --- | --- | --- |
| pau:PA14\_67280 | proW; ABC transporter permease; K02001 glycine betaine/proline transport system permease protein |  |
| pau:PA14\_67300 | proX; ABC transporter substrate-binding protein; K02002 glycine betaine/proline transport system substrate-binding protein |  |
| pau:PA14\_67310 | hutT; amino acid permease; K11736 proline-specific permease ProY |  |
| pau:PA14\_67320 | hutH; histidine ammonia-lyase (EC:4.3.1.3); K01745 histidine ammonia-lyase [EC:4.3.1.3] | ec:4.3.1.3 |
| pau:PA14\_67340 | codB; cytosine/purines uracil thiamine allantoin permease; K03457 nucleobase:cation symporter-1, NCS1 family |  |
| pau:PA14\_67350 | hutU; urocanate hydratase (EC:4.2.1.49); K01712 urocanate hydratase [EC:4.2.1.49] | ec:4.2.1.49 |
| pau:PA14\_67370 | hypothetical protein |  |
| pau:PA14\_67380 | fatty acid desaturase |  |
| pau:PA14\_67400 | ABC transporter substrate-binding protein; K02002 glycine betaine/proline transport system substrate-binding protein |  |
| pau:PA14\_67410 | hypothetical protein; K09975 hypothetical protein |  |
| pau:PA14\_67420 | hutC; histidine utilization genes repressor protein; K05836 GntR family transcriptional regulator, histidine utilization repressor |  |
| pau:PA14\_67440 | N-formimino-L-glutamate deiminase; K05603 formimidoylglutamate deiminase [EC:3.5.3.13] | ec:3.5.3.13 |
| pau:PA14\_67450 | blc; outer membrane lipoprotein Blc; K03098 apolipoprotein D and lipocalin family protein |  |
| pau:PA14\_67460 | lipoprotein |  |
| pau:PA14\_67470 | hypothetical protein |  |
| pau:PA14\_67490 | fbp; fructose-1,6-bisphosphatase (EC:3.1.3.11); K03841 fructose-1,6-bisphosphatase I [EC:3.1.3.11] | ec:3.1.3.11 |
| pau:PA14\_67500 | gloA3; lactoylglutathione lyase; K01759 lactoylglutathione lyase [EC:4.4.1.5] | ec:4.4.1.5 |
| pau:PA14\_67510 | estA; esterase EstA; K12686 outer membrane lipase/esterase |  |
| pau:PA14\_67520 | hypothetical protein |  |
| pau:PA14\_67530 | hypothetical protein |  |
| pau:PA14\_67540 | hypothetical protein |  |

  
**Neighborhood Representations for "rer:RER\_51240"**  

| ID | Annotation | EC number |
| --- | --- | --- |
| rer:RER\_51140 | hypothetical protein |  |
| rer:RER\_51150 | putative oxidoreductase |  |
| rer:RER\_51160 | hypothetical protein |  |
| rer:RER\_51170 | putative oxidoreductase |  |
| rer:RER\_51180 | N-acetyltransferase (EC:2.3.1.-) |  |
| rer:RER\_51190 | hypothetical protein |  |
| rer:RER\_51200 | transposase |  |
| rer:RER\_51210 | hypothetical protein |  |
| rer:RER\_51220 | probable dipeptidase (EC:3.4.13.-); K01273 membrane dipeptidase [EC:3.4.13.19] | ec:3.4.13.19 |
| rer:RER\_51230 | putative penicillin-binding protein |  |
| rer:RER\_51240 | putative GntR family transcriptional regulator; K05836 GntR family transcriptional regulator, histidine utilization repressor |  |
| rer:RER\_51250 | gdh; NAD-dependent glutamate dehydrogenase (EC:1.4.1.2); K15371 glutamate dehydrogenase [EC:1.4.1.2] | ec:1.4.1.2 |
| rer:RER\_51260 | fadE; probable acyl-CoA dehydrogenase (EC:1.3.99.-) |  |
| rer:RER\_51270 | hypothetical protein |  |
| rer:RER\_51280 | putative IclR family transcriptional regulator |  |
| rer:RER\_51290 | putative IclR family transcriptional regulator |  |
| rer:RER\_51300 | hutH; histidine ammonia-lyase (EC:4.3.1.3); K01745 histidine ammonia-lyase [EC:4.3.1.3] | ec:4.3.1.3 |
| rer:RER\_51310 | aminotransferase (EC:2.6.1.-) |  |
| rer:RER\_51320 | putative FAD-linked oxidase; K00104 glycolate oxidase [EC:1.1.3.15] | ec:1.1.3.15 |
| rer:RER\_51330 | putative NCS1 family transporter; K03457 nucleobase:cation symporter-1, NCS1 family |  |
| rer:RER\_51340 | hutU; urocanate hydratase (EC:4.2.1.49); K01712 urocanate hydratase [EC:4.2.1.49] | ec:4.2.1.49 |

  
**Neighborhood Representations for "kpe:KPK\_3779"**  

| ID | Annotation | EC number |
| --- | --- | --- |
| kpe:KPK\_3769 | hypothetical protein |  |
| kpe:KPK\_3770 | bioD; dithiobiotin synthetase; K01935 dethiobiotin synthetase [EC:6.3.3.3] | ec:6.3.3.3 |
| kpe:KPK\_3771 | bioC; biotin biosynthesis protein BioC; K02169 malonyl-CoA O-methyltransferase [EC:2.1.1.197] | ec:2.1.1.197 |
| kpe:KPK\_3772 | bioF; 8-amino-7-oxononanoate synthase; K00652 8-amino-7-oxononanoate synthase [EC:2.3.1.47] | ec:2.3.1.47 |
| kpe:KPK\_3773 | bioB; biotin synthase; K01012 biotin synthase [EC:2.8.1.6] | ec:2.8.1.6 |
| kpe:KPK\_3774 | bioA; adenosylmethionine-8-amino-7-oxononanoate aminotransferase; K00833 adenosylmethionine-8-amino-7-oxononanoate aminotransferase [EC:2.6.1.62] | ec:2.6.1.62 |
| kpe:KPK\_3775 | kinase inhibitor protein; K06910 |  |
| kpe:KPK\_3776 | amino acid permease; K11736 proline-specific permease ProY |  |
| kpe:KPK\_3777 | hutH; histidine ammonia-lyase; K01745 histidine ammonia-lyase [EC:4.3.1.3] | ec:4.3.1.3 |
| kpe:KPK\_3778 | hutU; urocanate hydratase; K01712 urocanate hydratase [EC:4.2.1.49] | ec:4.2.1.49 |
| kpe:KPK\_3779 | hutC; histidine utilization repressor; K05836 GntR family transcriptional regulator, histidine utilization repressor |  |
| kpe:KPK\_3780 | hutG; formimidoylglutamase; K01479 formiminoglutamase [EC:3.5.3.8] | ec:3.5.3.8 |
| kpe:KPK\_3781 | hutI; imidazolonepropionase; K01468 imidazolonepropionase [EC:3.5.2.7] | ec:3.5.2.7 |
| kpe:KPK\_3782 | pectinesterase; K01051 pectinesterase [EC:3.1.1.11] | ec:3.1.1.11 |
| kpe:KPK\_3783 | cation transporting ATPase, E1-E2 family |  |
| kpe:KPK\_3784 | universal stress family protein |  |
| kpe:KPK\_3785 | RND family efflux transporter MFP subunit |  |
| kpe:KPK\_3786 | RND transporter hydrophobe/amphiphile efflux-1 (HAE1) family |  |
| kpe:KPK\_3787 | hypothetical protein |  |
| kpe:KPK\_3788 | hypothetical protein |  |
| kpe:KPK\_3789 | hypothetical protein |  |

  
**Neighborhood Representations for "kva:Kvar\_3588"**  

| ID | Annotation | EC number |
| --- | --- | --- |
| kva:Kvar\_3578 | ABC transporter |  |
| kva:Kvar\_3579 | dethiobiotin synthase (EC:6.3.3.3); K01935 dethiobiotin synthetase [EC:6.3.3.3] | ec:6.3.3.3 |
| kva:Kvar\_3580 | biotin biosynthesis protein BioC; K02169 malonyl-CoA O-methyltransferase [EC:2.1.1.197] | ec:2.1.1.197 |
| kva:Kvar\_3581 | 8-amino-7-oxononanoate synthase (EC:2.3.1.47); K00652 8-amino-7-oxononanoate synthase [EC:2.3.1.47] | ec:2.3.1.47 |
| kva:Kvar\_3582 | biotin synthase (EC:2.8.1.6); K01012 biotin synthase [EC:2.8.1.6] | ec:2.8.1.6 |
| kva:Kvar\_3583 | adenosylmethionine-8-amino-7-oxononanoate aminotransferase; K00833 adenosylmethionine-8-amino-7-oxononanoate aminotransferase [EC:2.6.1.62] | ec:2.6.1.62 |
| kva:Kvar\_3584 | PEBP family protein; K06910 |  |
| kva:Kvar\_3585 | amino acid permease-associated protein; K11736 proline-specific permease ProY |  |
| kva:Kvar\_3586 | histidine ammonia-lyase (EC:4.3.1.3); K01745 histidine ammonia-lyase [EC:4.3.1.3] | ec:4.3.1.3 |
| kva:Kvar\_3587 | urocanate hydratase (EC:4.2.1.49); K01712 urocanate hydratase [EC:4.2.1.49] | ec:4.2.1.49 |
| kva:Kvar\_3588 | GntR family transcriptional regulator; K05836 GntR family transcriptional regulator, histidine utilization repressor |  |
| kva:Kvar\_3589 | formiminoglutamase; K01479 formiminoglutamase [EC:3.5.3.8] | ec:3.5.3.8 |
| kva:Kvar\_3590 | imidazolonepropionase (EC:3.5.2.7); K01468 imidazolonepropionase [EC:3.5.2.7] | ec:3.5.2.7 |
| kva:Kvar\_3591 | pectinesterase; K01051 pectinesterase [EC:3.1.1.11] | ec:3.1.1.11 |
| kva:Kvar\_3592 | ATPase P |  |
| kva:Kvar\_3593 | UspA domain-containing protein |  |
| kva:Kvar\_3594 | hypothetical protein |  |
| kva:Kvar\_3595 | RND family efflux transporter MFP subunit |  |
| kva:Kvar\_3596 | acriflavin resistance protein |  |
| kva:Kvar\_3597 | hypothetical protein |  |
| kva:Kvar\_3598 | hypothetical protein |  |

  
**Neighborhood Representations for "azl:AZL\_c00100"**  

| ID | Annotation | EC number |
| --- | --- | --- |
| azl:AZL\_c05330 | putative cobyrinic acid ac-diamide synthase; K03496 chromosome partitioning protein |  |
| azl:AZL\_c00010 | hypothetical protein |  |
| azl:AZL\_c00020 | hypothetical protein |  |
| azl:AZL\_c00030 | plasmid replication protein |  |
| azl:AZL\_c00040 | carbon-nitrogen family hydrolase |  |
| azl:AZL\_c00050 | hypothetical protein |  |
| azl:AZL\_c00060 | hutG; formiminoglutamase (EC:3.5.3.8); K01479 formiminoglutamase [EC:3.5.3.8] | ec:3.5.3.8 |
| azl:AZL\_c00070 | hutH; histidine ammonia-lyase (EC:4.3.1.3); K01745 histidine ammonia-lyase [EC:4.3.1.3] | ec:4.3.1.3 |
| azl:AZL\_c00080 | N-formimino-L-glutamate deiminase; K05603 formimidoylglutamate deiminase [EC:3.5.3.13] | ec:3.5.3.13 |
| azl:AZL\_c00090 | hutI; imidazolonepropionase (EC:3.5.2.7); K01468 imidazolonepropionase [EC:3.5.2.7] | ec:3.5.2.7 |
| azl:AZL\_c00100 | hutC; transcriptional regulator; K05836 GntR family transcriptional regulator, histidine utilization repressor |  |
| azl:AZL\_c00110 | hutU; urocanate hydratase (EC:4.2.1.49); K01712 urocanate hydratase [EC:4.2.1.49] | ec:4.2.1.49 |
| azl:AZL\_c00120 | beta-glucosidase (EC:3.2.1.21); K05350 beta-glucosidase [EC:3.2.1.21] | ec:3.2.1.21 |
| azl:AZL\_c00130 | hypothetical protein |  |
| azl:AZL\_c00140 | transglycosylase SLT family protein |  |
| azl:AZL\_c00150 | hypothetical protein |  |
| azl:AZL\_c00160 | cat; catalase; K03781 catalase [EC:1.11.1.6] | ec:1.11.1.6 |
| azl:AZL\_c00170 | oxyR; transcriptional regulator; K04761 LysR family transcriptional regulator, hydrogen peroxide-inducible genes activator |  |
| azl:AZL\_c00180 | glgX; glycogen operon protein; K02438 glycogen operon protein [EC:3.2.1.-] |  |
| azl:AZL\_c00190 | hypothetical protein |  |
| azl:AZL\_c00200 | TRAP-type mannitol/chloroaromatic compound transport system |  |

  
**Neighborhood Representations for "reu:Reut\_A0895"**  

| ID | Annotation | EC number |
| --- | --- | --- |
| reu:Reut\_A0885 | hypothetical protein; K07007 |  |
| reu:Reut\_A0886 | hypothetical protein; K09117 hypothetical protein |  |
| reu:Reut\_A0887 | dnaG; DNA primase; K02316 DNA primase [EC:2.7.7.-] |  |
| reu:Reut\_A0888 | RNA polymerase sigma factor RpoD; K03086 RNA polymerase primary sigma factor |  |
| reu:Reut\_A0889 | major facilitator transporter |  |
| reu:Reut\_A0890 | amidohydrolase |  |
| reu:Reut\_A0891 | LysR family transcriptional regulator |  |
| reu:Reut\_A0892 | 6-phosphogluconate dehydrogenase |  |
| reu:Reut\_A0893 | aldolase |  |
| reu:Reut\_A0894 | cyclic nucleotide-binding protein |  |
| reu:Reut\_A0895 | GntR family transcriptional regulator; K05836 GntR family transcriptional regulator, histidine utilization repressor |  |
| reu:Reut\_A0896 | urocanate hydratase (EC:4.2.1.49); K01712 urocanate hydratase [EC:4.2.1.49] | ec:4.2.1.49 |
| reu:Reut\_A0897 | major facilitator transporter |  |
| reu:Reut\_A0898 | hypothetical protein |  |
| reu:Reut\_A0899 | helicase |  |
| reu:Reut\_A0900 | glutamyl-Q tRNA(Asp) synthetase (EC:6.1.1.17); K01894 glutamyl-Q tRNA(Asp) synthetase [EC:6.1.1.-] |  |
| reu:Reut\_A0901 | LysR family transcriptional regulator |  |
| reu:Reut\_A0902 | pirin; K06911 |  |
| reu:Reut\_A0903 | CobD/CbiB family protein; K02227 adenosylcobinamide-phosphate synthase [EC:6.3.1.10] | ec:6.3.1.10 |
| reu:Reut\_A0904 | hypothetical protein |  |
| reu:Reut\_A0905 | ribosome-associated GTPase; K06949 ribosome biogenesis GTPase [EC:3.6.1.-] |  |

  
**Neighborhood Representations for "pfl:PFL\_0399"**  

| ID | Annotation | EC number |
| --- | --- | --- |
| pfl:PFL\_0389 | hypothetical protein; K06940 |  |
| pfl:PFL\_0390 | glgP; glycogen phosphorylase (EC:2.4.1.1); K00688 starch phosphorylase [EC:2.4.1.1] | ec:2.4.1.1 |
| pfl:PFL\_0391 | hypothetical protein |  |
| pfl:PFL\_0392 | hypothetical protein |  |
| pfl:PFL\_0393 | fbp; fructose-1,6-bisphosphatase (EC:3.1.3.11); K03841 fructose-1,6-bisphosphatase I [EC:3.1.3.11] | ec:3.1.3.11 |
| pfl:PFL\_0394 | hypothetical protein |  |
| pfl:PFL\_0395 | pctC; chemotactic transducer PctC; K03406 methyl-accepting chemotaxis protein |  |
| pfl:PFL\_0396 | lipoprotein |  |
| pfl:PFL\_0397 | blc; outer membrane lipoprotein Blc; K03098 apolipoprotein D and lipocalin family protein |  |
| pfl:PFL\_0398 | hutF; N-formimino-L-glutamate deiminase (EC:3.5.3.13); K05603 formimidoylglutamate deiminase [EC:3.5.3.13] | ec:3.5.3.13 |
| pfl:PFL\_0399 | hutC; histidine utilization repressor; K05836 GntR family transcriptional regulator, histidine utilization repressor |  |
| pfl:PFL\_0400 | hypothetical protein; K09975 hypothetical protein |  |
| pfl:PFL\_0401 | hutU; urocanate hydratase (EC:4.2.1.49); K01712 urocanate hydratase [EC:4.2.1.49] | ec:4.2.1.49 |
| pfl:PFL\_0402 | cytosine/purines uracil thiamine allantoin permease; K03457 nucleobase:cation symporter-1, NCS1 family |  |
| pfl:PFL\_0403 | hisX; histidine ABC transporter periplasmic histidine-binding protein; K02002 glycine betaine/proline transport system substrate-binding protein |  |
| pfl:PFL\_0404 | hisW; histidine ABC transporter permease; K02001 glycine betaine/proline transport system permease protein |  |
| pfl:PFL\_0405 | hisV; histidine ABC transporter ATP-binding protein; K02000 glycine betaine/proline transport system ATP-binding protein [EC:3.6.3.32] | ec:3.6.3.32 |
| pfl:PFL\_0406 | hutH\_1; histidine ammonia-lyase (EC:4.3.1.3); K01745 histidine ammonia-lyase [EC:4.3.1.3] | ec:4.3.1.3 |
| pfl:PFL\_0407 | hutH\_2; histidine ammonia-lyase (EC:4.3.1.3); K01745 histidine ammonia-lyase [EC:4.3.1.3] | ec:4.3.1.3 |
| pfl:PFL\_0408 | proY; permease; K11736 proline-specific permease ProY |  |
| pfl:PFL\_0409 | hutI; imidazolonepropionase (EC:3.5.2.7); K01468 imidazolonepropionase [EC:3.5.2.7] | ec:3.5.2.7 |

  
**Neighborhood Representations for "pst:PSPTO\_5172"**  

| ID | Annotation | EC number |
| --- | --- | --- |
| pst:PSPTO\_5162 | mdoG; periplasmic glucan biosynthesis protein; K03670 periplasmic glucans biosynthesis protein |  |
| pst:PSPTO\_5163 | dtd; D-tyrosyl-tRNA(Tyr) deacylase; K07560 D-tyrosyl-tRNA(Tyr) deacylase [EC:3.1.-.-] |  |
| pst:PSPTO\_5164 | pip; proline iminopeptidase; K01259 proline iminopeptidase [EC:3.4.11.5] | ec:3.4.11.5 |
| pst:PSPTO\_5165 | glgP; glycogen phosphorylase; K00688 starch phosphorylase [EC:2.4.1.1] | ec:2.4.1.1 |
| pst:PSPTO\_5166 | membrane protein |  |
| pst:PSPTO\_5167 | hypothetical protein |  |
| pst:PSPTO\_5168 | fbp; fructose-1,6-bisphosphatase; K03841 fructose-1,6-bisphosphatase I [EC:3.1.3.11] | ec:3.1.3.11 |
| pst:PSPTO\_5169 | lipoprotein |  |
| pst:PSPTO\_5170 | blc; lipoprotein Blc; K03098 apolipoprotein D and lipocalin family protein |  |
| pst:PSPTO\_5171 | Atz/Trz family protein; K05603 formimidoylglutamate deiminase [EC:3.5.3.13] | ec:3.5.3.13 |
| pst:PSPTO\_5172 | hutC; histidine utilization repressor; K05836 GntR family transcriptional regulator, histidine utilization repressor |  |
| pst:PSPTO\_5173 | hypothetical protein; K09975 hypothetical protein |  |
| pst:PSPTO\_5174 | nhaA-2; sodium-proton antiporter NhaA; K03313 Na+:H+ antiporter, NhaA family |  |
| pst:PSPTO\_5175 | hypothetical protein |  |
| pst:PSPTO\_5176 | RNA polymerase sigma-70 family protein; K03088 RNA polymerase sigma-70 factor, ECF subfamily |  |
| pst:PSPTO\_5177 | hypothetical protein |  |
| pst:PSPTO\_5178 | serine O-acetyltransferase; K00640 serine O-acetyltransferase [EC:2.3.1.30] | ec:2.3.1.30 |
| pst:PSPTO\_5179 | D-cysteine desulfhydrase; K05396 D-cysteine desulfhydrase [EC:4.4.1.15] | ec:4.4.1.15 |
| pst:PSPTO\_5180 | cystine transporter subunit; K02424 cystine transport system substrate-binding protein |  |
| pst:PSPTO\_5181 | amino acid ABC transporter permease; K10009 cystine transport system permease protein |  |
| pst:PSPTO\_5182 | putative amino-acid ABC transporter ATP-binding protein YecC; K10010 cystine transport system ATP-binding protein [EC:3.6.3.-] |  |

  
**Neighborhood Representations for "psp:PSPPH\_0349"**  

| ID | Annotation | EC number |
| --- | --- | --- |
| psp:PSPPH\_0339 | ISPsy18, transposase |  |
| psp:PSPPH\_0340 | glnQ1; amino-acid ABC transporter ATP-binding protein YecC; K10010 cystine transport system ATP-binding protein [EC:3.6.3.-] |  |
| psp:PSPPH\_0341 | cystine ABC transporter permease; K10009 cystine transport system permease protein |  |
| psp:PSPPH\_0342 | cystine transporter subunit; K02424 cystine transport system substrate-binding protein |  |
| psp:PSPPH\_0343 | dcyD; D-cysteine desulfhydrase (EC:4.4.1.15); K05396 D-cysteine desulfhydrase [EC:4.4.1.15] | ec:4.4.1.15 |
| psp:PSPPH\_0344 | serine O-acetyltransferase; K00640 serine O-acetyltransferase [EC:2.3.1.30] | ec:2.3.1.30 |
| psp:PSPPH\_0345 | RNA polymerase sigma-70 family protein |  |
| psp:PSPPH\_0346 | hypothetical protein |  |
| psp:PSPPH\_0347 | nhaA; pH-dependent sodium/proton antiporter; K03313 Na+:H+ antiporter, NhaA family |  |
| psp:PSPPH\_0348 | hypothetical protein; K09975 hypothetical protein |  |
| psp:PSPPH\_0349 | hutC; histidine utilization repressor; K05836 GntR family transcriptional regulator, histidine utilization repressor |  |
| psp:PSPPH\_0350 | hutF; N-formimino-L-glutamate deiminase (EC:3.5.3.13); K05603 formimidoylglutamate deiminase [EC:3.5.3.13] | ec:3.5.3.13 |
| psp:PSPPH\_0351 | blc; outer membrane lipoprotein Blc; K03098 apolipoprotein D and lipocalin family protein |  |
| psp:PSPPH\_0352 | lipoprotein |  |
| psp:PSPPH\_0353 | fbp; fructose-1,6-bisphosphatase (EC:3.1.3.11); K03841 fructose-1,6-bisphosphatase I [EC:3.1.3.11] | ec:3.1.3.11 |
| psp:PSPPH\_0354 | hypothetical protein |  |
| psp:PSPPH\_0355 | hypothetical protein |  |
| psp:PSPPH\_0356 | glgP; glycogen phosphorylase (EC:2.4.1.1); K00688 starch phosphorylase [EC:2.4.1.1] | ec:2.4.1.1 |
| psp:PSPPH\_0357 | pip; proline iminopeptidase (EC:3.4.11.5); K01259 proline iminopeptidase [EC:3.4.11.5] | ec:3.4.11.5 |
| psp:PSPPH\_0358 | dtd; D-tyrosyl-tRNA(Tyr) deacylase (EC:3.1.-.-); K07560 D-tyrosyl-tRNA(Tyr) deacylase [EC:3.1.-.-] |  |
| psp:PSPPH\_0359 | mdoG; glucan biosynthesis protein G; K03670 periplasmic glucans biosynthesis protein |  |

  
**Neighborhood Representations for "eam:EAMY\_1256"**  

| ID | Annotation | EC number |
| --- | --- | --- |
| eam:EAMY\_1246 | hypothetical protein |  |
| eam:EAMY\_1247 | yehV; HTH-type transcriptional regulator MlrA |  |
| eam:EAMY\_1248 | hypothetical protein |  |
| eam:EAMY\_1249 | DNA-binding protein |  |
| eam:EAMY\_1250 | acetyltransferase |  |
| eam:EAMY\_1251 | dinG; ATP-dependent helicase dinG; K03722 ATP-dependent DNA helicase DinG [EC:3.6.4.12] | ec:3.6.4.12 |
| eam:EAMY\_1252 | ybiB; anthranilate phosphoribosyltransferase |  |
| eam:EAMY\_1253 | hipO; hydrolase; K01451 hippurate hydrolase [EC:3.5.1.32] | ec:3.5.1.32 |
| eam:EAMY\_1254 | hutU; urocanate hydratase; K01712 urocanate hydratase [EC:4.2.1.49] | ec:4.2.1.49 |
| eam:EAMY\_1255 | hutH; histidine ammonia-lyase; K01745 histidine ammonia-lyase [EC:4.3.1.3] | ec:4.3.1.3 |
| eam:EAMY\_1256 | hutC; histidine utilization repressor; K05836 GntR family transcriptional regulator, histidine utilization repressor |  |
| eam:EAMY\_1257 | ydjR; hypothetical protein; K09975 hypothetical protein |  |
| eam:EAMY\_1258 | hutF; formiminoglutamate deiminase; K05603 formimidoylglutamate deiminase [EC:3.5.3.13] | ec:3.5.3.13 |
| eam:EAMY\_1259 | hutI; imidazolonepropionase; K01468 imidazolonepropionase [EC:3.5.2.7] | ec:3.5.2.7 |
| eam:EAMY\_1260 | hutG; N-formylglutamate amidohydrolase; K01479 formiminoglutamase [EC:3.5.3.8] | ec:3.5.3.8 |
| eam:EAMY\_1261 | hypothetical protein |  |
| eam:EAMY\_1262 | hypothetical protein |  |
| eam:EAMY\_1263 | hypothetical protein |  |
| eam:EAMY\_1264 | hypothetical protein |  |
| eam:EAMY\_1265 | syrB; chondroitin sulfate/heparin utilization regulation protein; K06871 uncharacterized protein |  |
| eam:EAMY\_1266 | hypothetical protein |  |

  
**Neighborhood Representations for "eay:EAM\_1255"**  

| ID | Annotation | EC number |
| --- | --- | --- |
| eay:EAM\_1245 | short-chain dehydrogenase |  |
| eay:EAM\_1246 | hypothetical protein |  |
| eay:EAM\_1247 | mlrA; MerR family transcriptional regulator |  |
| eay:EAM\_1248 | regulatory protein |  |
| eay:EAM\_1249 | acetyltransferase |  |
| eay:EAM\_1250 | dinG; ATP-dependent helicase; K03722 ATP-dependent DNA helicase DinG [EC:3.6.4.12] | ec:3.6.4.12 |
| eay:EAM\_1251 | glycosyl transferase family protein |  |
| eay:EAM\_1252 | amidohydrolase; K01451 hippurate hydrolase [EC:3.5.1.32] | ec:3.5.1.32 |
| eay:EAM\_1253 | hutU; urocanate hydratase; K01712 urocanate hydratase [EC:4.2.1.49] | ec:4.2.1.49 |
| eay:EAM\_1254 | hutH; histidine ammonia-lyase; K01745 histidine ammonia-lyase [EC:4.3.1.3] | ec:4.3.1.3 |
| eay:EAM\_1255 | hutC; histidine utilization repressor; K05836 GntR family transcriptional regulator, histidine utilization repressor |  |
| eay:EAM\_1256 | hypothetical protein; K09975 hypothetical protein |  |
| eay:EAM\_1257 | chlorohydrolase; K05603 formimidoylglutamate deiminase [EC:3.5.3.13] | ec:3.5.3.13 |
| eay:EAM\_1258 | hutI; imidazolonepropionase; K01468 imidazolonepropionase [EC:3.5.2.7] | ec:3.5.2.7 |
| eay:EAM\_1259 | hutG; N-formylglutamate amidohydrolase; K01479 formiminoglutamase [EC:3.5.3.8] | ec:3.5.3.8 |
| eay:EAM\_1260 | 5'(3')-deoxyribonucleotidase |  |
| eay:EAM\_1261 | hypothetical protein |  |
| eay:EAM\_1262 | hypothetical protein |  |
| eay:EAM\_1263 | hypothetical protein |  |
| eay:EAM\_1264 | pseudogene |  |
| eay:EAM\_1265 | hypothetical protein; K06970 23S rRNA (adenine1618-N6)-methyltransferase [EC:2.1.1.181] | ec:2.1.1.181 |

  
**Neighborhood Representations for "psb:Psyr\_0366"**  

| ID | Annotation | EC number |
| --- | --- | --- |
| psb:Psyr\_0356 | amino-acid ABC transporter ATP-binding protein YecC; K10010 cystine transport system ATP-binding protein [EC:3.6.3.-] |  |
| psb:Psyr\_0357 | amino acid ABC transporter permease; K10009 cystine transport system permease protein |  |
| psb:Psyr\_0358 | cystine transporter subunit; K02424 cystine transport system substrate-binding protein |  |
| psb:Psyr\_0359 | D-cysteine desulfhydrase (EC:4.4.1.15); K05396 D-cysteine desulfhydrase [EC:4.4.1.15] | ec:4.4.1.15 |
| psb:Psyr\_0360 | Serine O-acetyltransferase (EC:2.3.1.30); K00640 serine O-acetyltransferase [EC:2.3.1.30] | ec:2.3.1.30 |
| psb:Psyr\_0361 | hypothetical protein |  |
| psb:Psyr\_0362 | RNA polymerase sigma factor; K03088 RNA polymerase sigma-70 factor, ECF subfamily |  |
| psb:Psyr\_0363 | hypothetical protein |  |
| psb:Psyr\_0364 | nhaA; pH-dependent sodium/proton antiporter; K03313 Na+:H+ antiporter, NhaA family |  |
| psb:Psyr\_0365 | hypothetical protein; K09975 hypothetical protein |  |
| psb:Psyr\_0366 | histidine utilization repressor; K05836 GntR family transcriptional regulator, histidine utilization repressor |  |
| psb:Psyr\_0367 | N-formimino-L-glutamate deiminase; K05603 formimidoylglutamate deiminase [EC:3.5.3.13] | ec:3.5.3.13 |
| psb:Psyr\_0368 | lipoprotein Blc; K03098 apolipoprotein D and lipocalin family protein |  |
| psb:Psyr\_0369 | lipoprotein |  |
| psb:Psyr\_0370 | fructose-1,6-bisphosphatase (EC:3.1.3.11); K03841 fructose-1,6-bisphosphatase I [EC:3.1.3.11] | ec:3.1.3.11 |
| psb:Psyr\_0371 | hypothetical protein |  |
| psb:Psyr\_0372 | pseudogene |  |
| psb:Psyr\_0373 | pseudogene |  |
| psb:Psyr\_0374 | phosphorylase (EC:2.4.1.1); K00688 starch phosphorylase [EC:2.4.1.1] | ec:2.4.1.1 |
| psb:Psyr\_0375 | peptidase S33, proline iminopeptidase 1 (EC:3.4.11.5); K01259 proline iminopeptidase [EC:3.4.11.5] | ec:3.4.11.5 |
| psb:Psyr\_0376 | D-tyrosyl-tRNA(Tyr) deacylase; K07560 D-tyrosyl-tRNA(Tyr) deacylase [EC:3.1.-.-] |  |

  
**Neighborhood Representations for "req:REQ\_41890"**  

| ID | Annotation | EC number |
| --- | --- | --- |
| req:REQ\_41790 | fe-s cluster assembly protein nifu-like |  |
| req:REQ\_41800 | hydrogenase large subunit; K06281 hydrogenase large subunit [EC:1.12.99.6] | ec:1.12.99.6 |
| req:REQ\_41810 | hydrogenase small subunit; K06282 hydrogenase small subunit [EC:1.12.99.6] | ec:1.12.99.6 |
| req:REQ\_41820 | hypB; hydrogenase accessory protein hypb; K04652 hydrogenase nickel incorporation protein HypB |  |
| req:REQ\_41830 | hypA; hydrogenase accessory protein hypa; K04651 hydrogenase nickel incorporation protein HypA/HybF |  |
| req:REQ\_41840 | tetr family transcriptional regulator |  |
| req:REQ\_41850 | taurine dioxygenase |  |
| req:REQ\_41860 | high affinity substrate-binding lipoprotein |  |
| req:REQ\_41870 | ArsR family transcriptional regulator |  |
| req:REQ\_41880 | alpha/beta hydrolase |  |
| req:REQ\_41890 | GntR family transcriptional regulator; K05836 GntR family transcriptional regulator, histidine utilization repressor |  |
| req:REQ\_41900 | citrate lyase beta chain |  |
| req:REQ\_41910 | glutamate dehydrogenase; K15371 glutamate dehydrogenase [EC:1.4.1.2] | ec:1.4.1.2 |
| req:REQ\_41930 | hutU; urocanate hydratase; K01712 urocanate hydratase [EC:4.2.1.49] | ec:4.2.1.49 |
| req:REQ\_41940 | acyl-CoA dehydrogenase |  |
| req:REQ\_41950 | CoA-transferase |  |
| req:REQ\_41960 | IclR family transcriptional regulator |  |
| req:REQ\_41970 | ArsR family transcriptional regulator |  |
| req:REQ\_41980 | hutH; histidine ammonia-lyase huth; K01745 histidine ammonia-lyase [EC:4.3.1.3] | ec:4.3.1.3 |
| req:REQ\_41990 | hisC2; histidinol-phosphate transaminase hisc2 |  |
| req:REQ\_42000 | FAD-dependent oxidoreductase; K00104 glycolate oxidase [EC:1.1.3.15] | ec:1.1.3.15 |

  
**Neighborhood Representations for "cko:CKO\_02358"**  

| ID | Annotation | EC number |
| --- | --- | --- |
| cko:CKO\_02348 | excinuclease ABC subunit B; K03702 excinuclease ABC subunit B |  |
| cko:CKO\_02349 | hypothetical protein |  |
| cko:CKO\_02350 | bioD; dithiobiotin synthetase; K01935 dethiobiotin synthetase [EC:6.3.3.3] | ec:6.3.3.3 |
| cko:CKO\_02351 | biotin biosynthesis protein BioC; K02169 malonyl-CoA O-methyltransferase [EC:2.1.1.197] | ec:2.1.1.197 |
| cko:CKO\_02352 | 8-amino-7-oxononanoate synthase; K00652 8-amino-7-oxononanoate synthase [EC:2.3.1.47] | ec:2.3.1.47 |
| cko:CKO\_02353 | hypothetical protein; K01012 biotin synthase [EC:2.8.1.6] | ec:2.8.1.6 |
| cko:CKO\_02354 | adenosylmethionine--8-amino-7-oxononanoate transaminase; K00833 adenosylmethionine-8-amino-7-oxononanoate aminotransferase [EC:2.6.1.62] | ec:2.6.1.62 |
| cko:CKO\_02355 | putative kinase inhibitor protein; K06910 |  |
| cko:CKO\_02356 | histidine ammonia-lyase; K01745 histidine ammonia-lyase [EC:4.3.1.3] | ec:4.3.1.3 |
| cko:CKO\_02357 | urocanate hydratase; K01712 urocanate hydratase [EC:4.2.1.49] | ec:4.2.1.49 |
| cko:CKO\_02358 | hypothetical protein; K05836 GntR family transcriptional regulator, histidine utilization repressor |  |
| cko:CKO\_02359 | formimidoylglutamase; K01479 formiminoglutamase [EC:3.5.3.8] | ec:3.5.3.8 |
| cko:CKO\_02360 | imidazolonepropionase; K01468 imidazolonepropionase [EC:3.5.2.7] | ec:3.5.2.7 |
| cko:CKO\_02361 | hypothetical protein |  |
| cko:CKO\_02362 | putative pectinesterase; K01051 pectinesterase [EC:3.1.1.11] | ec:3.1.1.11 |
| cko:CKO\_02363 | hypothetical protein; K01681 aconitate hydratase [EC:4.2.1.3] | ec:4.2.1.3 |
| cko:CKO\_02364 | hypothetical protein; K03319 divalent anion:Na+ symporter, DASS family |  |
| cko:CKO\_02365 | hypothetical protein |  |
| cko:CKO\_02366 | hypothetical protein; K09788 hypothetical protein |  |
| cko:CKO\_02367 | hypothetical protein |  |
| cko:CKO\_02368 | 6-phosphogluconolactonase; K07404 6-phosphogluconolactonase [EC:3.1.1.31] | ec:3.1.1.31 |

  
**Neighborhood Representations for "rha:RHA1\_ro04645"**  

| ID | Annotation | EC number |
| --- | --- | --- |
| rha:RHA1\_ro04635 | CoA transferase; K07749 formyl-CoA transferase [EC:2.8.3.16] | ec:2.8.3.16 |
| rha:RHA1\_ro04636 | acyl-CoA dehydrogenase (EC:1.3.99.3); K00249 acyl-CoA dehydrogenase [EC:1.3.8.7] | ec:1.3.8.7 |
| rha:RHA1\_ro04637 | hypothetical protein |  |
| rha:RHA1\_ro04638 | cold shock protein; K03704 cold shock protein (beta-ribbon, CspA family) |  |
| rha:RHA1\_ro04639 | hydrolase |  |
| rha:RHA1\_ro04640 | hutG; formimidoylglutamase (EC:3.5.3.8); K01479 formiminoglutamase [EC:3.5.3.8] | ec:3.5.3.8 |
| rha:RHA1\_ro04641 | hutI; imidazolonepropionase (EC:3.5.2.7); K01468 imidazolonepropionase [EC:3.5.2.7] | ec:3.5.2.7 |
| rha:RHA1\_ro04642 | hutU; urocanate hydratase (EC:4.2.1.49); K01712 urocanate hydratase [EC:4.2.1.49] | ec:4.2.1.49 |
| rha:RHA1\_ro04643 | cytosine/purines, uracil, thiamine, allantoin permease; K03457 nucleobase:cation symporter-1, NCS1 family |  |
| rha:RHA1\_ro04644 | NAD-specific glutamate dehydrogenase; K15371 glutamate dehydrogenase [EC:1.4.1.2] | ec:1.4.1.2 |
| rha:RHA1\_ro04645 | hutC; histidine utilization repressor; K05836 GntR family transcriptional regulator, histidine utilization repressor |  |
| rha:RHA1\_ro04646 | transcriptional regulator |  |
| rha:RHA1\_ro04647 | hypothetical protein; K13671 alpha-1,2-mannosyltransferase [EC:2.4.1.-] |  |
| rha:RHA1\_ro04648 | hypothetical protein |  |
| rha:RHA1\_ro04649 | 2-nitropropane dioxygenase (EC:1.13.12.16); K00459 nitronate monooxygenase [EC:1.13.12.16] | ec:1.13.12.16 |
| rha:RHA1\_ro04650 | CoA-transferase subunit beta; K01041 [EC:2.8.3.-] |  |
| rha:RHA1\_ro04651 | CoA-transferase subunit alpha; K01041 [EC:2.8.3.-] |  |
| rha:RHA1\_ro04652 | enoyl-CoA hydratase (EC:4.2.1.17); K01692 enoyl-CoA hydratase [EC:4.2.1.17] | ec:4.2.1.17 |
| rha:RHA1\_ro04653 | short chain dehydrogenase |  |
| rha:RHA1\_ro04654 | short chain dehydrogenase (EC:1.1.1.100) |  |
| rha:RHA1\_ro04655 | monooxygenase |  |

  
**Neighborhood Representations for "eta:ETA\_22230"**  

| ID | Annotation | EC number |
| --- | --- | --- |
| eta:ETA\_22130 | 5'(3')-deoxyribonucleotidase |  |
| eta:ETA\_22140 | hypothetical protein |  |
| eta:ETA\_22150 | RNA-directed DNA polymerase |  |
| eta:ETA\_22160 | phage-related reverse transcriptase/maturase family protein |  |
| eta:ETA\_22170 | integrase, fragment |  |
| eta:ETA\_22180 | hypothetical protein |  |
| eta:ETA\_22190 | hutG; N-formylglutamate amidohydrolase (EC:3.5.3.8); K01479 formiminoglutamase [EC:3.5.3.8] | ec:3.5.3.8 |
| eta:ETA\_22200 | hutI; imidazolonepropionase (EC:3.5.2.7); K01468 imidazolonepropionase [EC:3.5.2.7] | ec:3.5.2.7 |
| eta:ETA\_22210 | N-formimino-L-glutamate deiminase; K05603 formimidoylglutamate deiminase [EC:3.5.3.13] | ec:3.5.3.13 |
| eta:ETA\_22220 | ydjR; hypothetical protein; K09975 hypothetical protein |  |
| eta:ETA\_22230 | GntR family transcriptional regulator; K05836 GntR family transcriptional regulator, histidine utilization repressor |  |
| eta:ETA\_22240 | hutH; histidine ammonia-lyase (EC:4.3.1.3); K01745 histidine ammonia-lyase [EC:4.3.1.3] | ec:4.3.1.3 |
| eta:ETA\_22250 | hutU; urocanate hydratase (EC:4.2.1.49); K01712 urocanate hydratase [EC:4.2.1.49] | ec:4.2.1.49 |
| eta:ETA\_22260 | amidohydrolase; K01451 hippurate hydrolase [EC:3.5.1.32] | ec:3.5.1.32 |
| eta:ETA\_22270 | ybiB; glycosyl transferase family protein |  |
| eta:ETA\_22280 | dinG; ATP-dependent DNA helicase DinG (EC:3.6.1.-); K03722 ATP-dependent DNA helicase DinG [EC:3.6.4.12] | ec:3.6.4.12 |
| eta:ETA\_22290 | Acetyltransferase |  |
| eta:ETA\_22300 | hipB; transcriptional regulator |  |
| eta:ETA\_22310 | mlrA; MerR family transcriptional regulator |  |
| eta:ETA\_22320 | transcriptional regulator |  |
| eta:ETA\_22330 | short-chain dehydrogenase (EC:1.1.1.-) |  |

  
**Neighborhood Representations for "pfs:PFLU0359"**  

| ID | Annotation | EC number |
| --- | --- | --- |
| pfs:PFLU0349 | thiI; thiamine biosynthesis protein ThiI; K03151 thiamine biosynthesis protein ThiI |  |
| pfs:PFLU0350 | typA; putative elongation facotr/GTP-binding protein TypA/BipA; K06207 GTP-binding protein |  |
| pfs:PFLU0351 | hypothetical protein; K06940 |  |
| pfs:PFLU0352 | glgP; glycogen phosphorylase (EC:2.4.1.1); K00688 starch phosphorylase [EC:2.4.1.1] | ec:2.4.1.1 |
| pfs:PFLU0353 | hypothetical protein |  |
| pfs:PFLU0354 | hypothetical protein |  |
| pfs:PFLU0355 | fbp; fructose-1,6-bisphosphatase (EC:3.1.3.11); K03841 fructose-1,6-bisphosphatase I [EC:3.1.3.11] | ec:3.1.3.11 |
| pfs:PFLU0356 | putative lipoprotein |  |
| pfs:PFLU0357 | putative lipoprotein; K03098 apolipoprotein D and lipocalin family protein |  |
| pfs:PFLU0358 | N-formimino-L-glutamate deiminase; K05603 formimidoylglutamate deiminase [EC:3.5.3.13] | ec:3.5.3.13 |
| pfs:PFLU0359 | hutC; GntR family transcriptional regulator; K05836 GntR family transcriptional regulator, histidine utilization repressor |  |
| pfs:PFLU0360 | hypothetical protein; K09975 hypothetical protein |  |
| pfs:PFLU0361 | hutU; urocanate hydratase (EC:4.2.1.49); K01712 urocanate hydratase [EC:4.2.1.49] | ec:4.2.1.49 |
| pfs:PFLU0362 | putative histidine ABC transporter membrane protein; K03457 nucleobase:cation symporter-1, NCS1 family |  |
| pfs:PFLU0363 | putative histidine transporter substrate-binding protein; K02002 glycine betaine/proline transport system substrate-binding protein |  |
| pfs:PFLU0364 | putative histidine ABC transporter membrane protein; K02001 glycine betaine/proline transport system permease protein |  |
| pfs:PFLU0365 | putative histidine ABC transporter ATP-binding protein; K02000 glycine betaine/proline transport system ATP-binding protein [EC:3.6.3.32] | ec:3.6.3.32 |
| pfs:PFLU0366 | putative histidine lyase; K01745 histidine ammonia-lyase [EC:4.3.1.3] | ec:4.3.1.3 |
| pfs:PFLU0367 | hutH; histidine ammonia-lyase (EC:4.3.1.3); K01745 histidine ammonia-lyase [EC:4.3.1.3] | ec:4.3.1.3 |
| pfs:PFLU0368 | putative histidine ABC transporter permease; K11736 proline-specific permease ProY |  |
| pfs:PFLU0369 | imidazolonepropionase; K01468 imidazolonepropionase [EC:3.5.2.7] | ec:3.5.2.7 |

  
**Neighborhood Representations for "rop:ROP\_47420"**  

| ID | Annotation | EC number |
| --- | --- | --- |
| rop:ROP\_47320 | CaiB/BaiF family protein |  |
| rop:ROP\_47330 | fadE; acyl-CoA dehydrogenase (EC:1.3.99.-) |  |
| rop:ROP\_47340 | hypothetical protein |  |
| rop:ROP\_47350 | cold shock protein; K03704 cold shock protein (beta-ribbon, CspA family) |  |
| rop:ROP\_47360 | hydrolase |  |
| rop:ROP\_47370 | hutG; formimidoylglutamase (EC:3.5.3.8); K01479 formiminoglutamase [EC:3.5.3.8] | ec:3.5.3.8 |
| rop:ROP\_47380 | hutI; imidazolonepropionase (EC:3.5.2.7); K01468 imidazolonepropionase [EC:3.5.2.7] | ec:3.5.2.7 |
| rop:ROP\_47390 | hutU; urocanate hydratase (EC:4.2.1.49); K01712 urocanate hydratase [EC:4.2.1.49] | ec:4.2.1.49 |
| rop:ROP\_47400 | NCS1 family transporter; K03457 nucleobase:cation symporter-1, NCS1 family |  |
| rop:ROP\_47410 | gdh; NAD-dependent glutamate dehydrogenase (EC:1.4.1.2); K15371 glutamate dehydrogenase [EC:1.4.1.2] | ec:1.4.1.2 |
| rop:ROP\_47420 | GntR family transcriptional regulator; K05836 GntR family transcriptional regulator, histidine utilization repressor |  |
| rop:ROP\_47430 | glycosyltransferase (EC:2.4.-.-) |  |
| rop:ROP\_47440 | hypothetical protein |  |
| rop:ROP\_47450 | AraC family transcriptional regulator |  |
| rop:ROP\_47460 | esterase |  |
| rop:ROP\_47470 | oxidoreductase |  |
| rop:ROP\_47480 | CoA-transferase beta subunit (EC:2.8.3.-); K01041 [EC:2.8.3.-] |  |
| rop:ROP\_47490 | CoA-transferase alpha subunit (EC:2.8.3.-); K01041 [EC:2.8.3.-] |  |
| rop:ROP\_47500 | enoyl-CoA hydratase (EC:4.2.1.17); K01692 enoyl-CoA hydratase [EC:4.2.1.17] | ec:4.2.1.17 |
| rop:ROP\_47510 | short chain dehydrogenase |  |
| rop:ROP\_47520 | short chain dehydrogenase |  |

  
**Neighborhood Representations for "pay:PAU\_01422"**  

| ID | Annotation | EC number |
| --- | --- | --- |
| pay:PAU\_01412 | glk; glucokinase (EC:2.7.1.2); K00845 glucokinase [EC:2.7.1.2] | ec:2.7.1.2 |
| pay:PAU\_01413 | vacJ; vacj lipoprotein; K04754 lipoprotein |  |
| pay:PAU\_01414 | fadL; outer membrane protein; K06076 long-chain fatty acid transport protein |  |
| pay:PAU\_01415 | hypothetical protein |  |
| pay:PAU\_01416 | fadI; 3-ketoacyl-CoA thiolase (EC:2.3.1.16); K00632 acetyl-CoA acyltransferase [EC:2.3.1.16] | ec:2.3.1.16 |
| pay:PAU\_01417 | fadJ; fatty acid oxidation complex subunit alpha (EC:1.1.1.35); K01782 3-hydroxyacyl-CoA dehydrogenase / enoyl-CoA hydratase / 3-hydroxybutyryl-CoA epimerase [EC:1.1.1.35 4.2.1.17 5.1.2.3] | ec:5.1.2.3 ec:4.2.1.17 ec:1.1.1.35 |
| pay:PAU\_01418 | sixA; phosphohistidine phosphatase sixa (rx6) (EC:3.1.3.-); K08296 phosphohistidine phosphatase [EC:3.1.3.-] |  |
| pay:PAU\_01419 | yfcN; hypothetical protein |  |
| pay:PAU\_01420 | hutI; imidazolonepropionase (EC:3.5.2.7); K01468 imidazolonepropionase [EC:3.5.2.7] | ec:3.5.2.7 |
| pay:PAU\_01421 | hutG; formimidoylglutamase (EC:3.5.3.8); K01479 formiminoglutamase [EC:3.5.3.8] | ec:3.5.3.8 |
| pay:PAU\_01422 | hutC; hutc protein (putative gntr-family transcriptional regulatory protein (putative repressor); K05836 GntR family transcriptional regulator, histidine utilization repressor |  |
| pay:PAU\_01423 | hutU; urocanate hydratase (EC:4.2.1.49); K01712 urocanate hydratase [EC:4.2.1.49] | ec:4.2.1.49 |
| pay:PAU\_01424 | hutH; histidine ammonia-lyase (EC:4.3.1.3); K01745 histidine ammonia-lyase [EC:4.3.1.3] | ec:4.3.1.3 |
| pay:PAU\_01425 | amino acid permease; K11736 proline-specific permease ProY |  |
| pay:PAU\_01426 | hypothetical protein; K07320 putative adenine-specific DNA-methyltransferase [EC:2.1.1.72] | ec:2.1.1.72 |
| pay:PAU\_01427 | aroC; chorismate synthase (EC:4.2.3.5); K01736 chorismate synthase [EC:4.2.3.5] | ec:4.2.3.5 |
| pay:PAU\_01428 | putative permease (putative membrane protein); K07090 |  |
| pay:PAU\_01429 | hypothetical protein; K09906 hypothetical protein |  |
| pay:PAU\_01430 | hypothetical protein |  |
| pay:PAU\_01431 | hypothetical protein |  |
| pay:PAU\_01432 | dinJ; damage-inducible protein j (damage-inducible protein dinj); K07473 DNA-damage-inducible protein J |  |

  
**Neighborhood Representations for "nfa:nfa12250"**  

| ID | Annotation | EC number |
| --- | --- | --- |
| nfa:nfa12150 | ferredoxin reductase; K00529 ferredoxin--NAD+ reductase [EC:1.18.1.3] | ec:1.18.1.3 |
| nfa:nfa12160 | cytochrome P450 monooxygenase |  |
| nfa:nfa12170 | transporter |  |
| nfa:nfa12180 | regulator |  |
| nfa:nfa12190 | oxygenase |  |
| nfa:nfa12200 | oxidoreductase |  |
| nfa:nfa12210 | aldehyde dehydrogenase; K00128 aldehyde dehydrogenase (NAD+) [EC:1.2.1.3] | ec:1.2.1.3 |
| nfa:nfa12220 | hypothetical protein |  |
| nfa:nfa12230 | dehydrogenase; K00153 S-(hydroxymethyl)mycothiol dehydrogenase [EC:1.1.1.306] | ec:1.1.1.306 |
| nfa:nfa12240 | transcriptional regulator |  |
| nfa:nfa12250 | transcriptional regulator; K05836 GntR family transcriptional regulator, histidine utilization repressor |  |
| nfa:nfa12260 | hypothetical protein; K15371 glutamate dehydrogenase [EC:1.4.1.2] | ec:1.4.1.2 |
| nfa:nfa12270 | hutU; urocanate hydratase (EC:4.2.1.49); K01712 urocanate hydratase [EC:4.2.1.49] | ec:4.2.1.49 |
| nfa:nfa12280 | hutH; histidine ammonia-lyase (EC:4.3.1.3); K01745 histidine ammonia-lyase [EC:4.3.1.3] | ec:4.3.1.3 |
| nfa:nfa12290 | amino acid transporter; K11736 proline-specific permease ProY |  |
| nfa:nfa12300 | fadE15; acyl-CoA dehydrogenase |  |
| nfa:nfa12310 | CoA-transferase |  |
| nfa:nfa12320 | transcriptional regulator |  |
| nfa:nfa12330 | transporter; K03457 nucleobase:cation symporter-1, NCS1 family |  |
| nfa:nfa12340 | hutI; imidazolonepropionase; K01468 imidazolonepropionase [EC:3.5.2.7] | ec:3.5.2.7 |
| nfa:nfa12350 | formimidoylglutamase; K01479 formiminoglutamase [EC:3.5.3.8] | ec:3.5.3.8 |

  
**Neighborhood Representations for "ebi:EbC\_14200"**  

| ID | Annotation | EC number |
| --- | --- | --- |
| ebi:EbC\_14100 | dinG; DNA-damage-inducible protein G; K03722 ATP-dependent DNA helicase DinG [EC:3.6.4.12] | ec:3.6.4.12 |
| ebi:EbC\_14110 | ybiB; hypothetical protein |  |
| ebi:EbC\_14120 | amidohydrolase; K01451 hippurate hydrolase [EC:3.5.1.32] | ec:3.5.1.32 |
| ebi:EbC\_14130 | hutU; urocanate hydratase; K01712 urocanate hydratase [EC:4.2.1.49] | ec:4.2.1.49 |
| ebi:EbC\_14140 | hutH; histidine ammonia-lyase; K01745 histidine ammonia-lyase [EC:4.3.1.3] | ec:4.3.1.3 |
| ebi:EbC\_14150 | GntR family transcriptional regulator; K05836 GntR family transcriptional regulator, histidine utilization repressor |  |
| ebi:EbC\_14160 | ydjR; hypothetical protein; K09975 hypothetical protein |  |
| ebi:EbC\_14170 | chlorohydrolase; K05603 formimidoylglutamate deiminase [EC:3.5.3.13] | ec:3.5.3.13 |
| ebi:EbC\_14180 | hutI; imidazolonepropionase; K01468 imidazolonepropionase [EC:3.5.2.7] | ec:3.5.2.7 |
| ebi:EbC\_14190 | hutG; N-formylglutamate amidohydrolase; K01479 formiminoglutamase [EC:3.5.3.8] | ec:3.5.3.8 |
| ebi:EbC\_14200 | GntR family transcriptional regulator; K05836 GntR family transcriptional regulator, histidine utilization repressor |  |
| ebi:EbC\_14210 | Extracellular substrate-binding protein, family 3; K10018 octopine/nopaline transport system substrate-binding protein |  |
| ebi:EbC\_14220 | Polar amino acid ABC transporter inner membrane subunit; K10020 octopine/nopaline transport system permease protein |  |
| ebi:EbC\_14230 | Polar amino acid ABC transporter inner membrane subunit; K10019 octopine/nopaline transport system permease protein |  |
| ebi:EbC\_14240 | ABC transporter; K10021 octopine/nopaline transport system ATP-binding protein [EC:3.6.3.-] |  |
| ebi:EbC\_14250 | hutH; histidine ammonia-lyase; K01745 histidine ammonia-lyase [EC:4.3.1.3] | ec:4.3.1.3 |
| ebi:EbC\_14260 | hypothetical protein |  |
| ebi:EbC\_14270 | hypothetical protein; K06970 23S rRNA (adenine1618-N6)-methyltransferase [EC:2.1.1.181] | ec:2.1.1.181 |
| ebi:EbC\_14280 | ybiO; transporter |  |
| ebi:EbC\_14290 | glnQ; glutamine transport ATP-binding protein; K10038 glutamine transport system ATP-binding protein [EC:3.6.3.-] |  |
| ebi:EbC\_14300 | hypothetical protein; K10037 glutamine transport system permease protein |  |

  
**Neighborhood Representations for "cti:RALTA\_A0655"**  

| ID | Annotation | EC number |
| --- | --- | --- |
| cti:RALTA\_A0644 | AsnC family transcriptional regulator |  |
| cti:RALTA\_A0645 | amidinotransferase |  |
| cti:RALTA\_A0646 | ornithine cyclodeaminase (EC:4.3.1.12); K01750 ornithine cyclodeaminase [EC:4.3.1.12] | ec:4.3.1.12 |
| cti:RALTA\_A0647 | hypothetical protein |  |
| cti:RALTA\_A0649 | dctD2; c4-dicarboxylate transport response regulator; K10126 two-component system, NtrC family, C4-dicarboxylate transport response regulator DctD |  |
| cti:RALTA\_A0650 | dctB2; c4-dicarboxylate transport sensor kinase; K10125 two-component system, NtrC family, C4-dicarboxylate transport sensor histidine kinase DctB [EC:2.7.13.3] | ec:2.7.13.3 |
| cti:RALTA\_A0651 | dctA2; citrate and c4-dicarboxylic acids transporter (daacs family) |  |
| cti:RALTA\_A0652 | hutG2; formimidoylglutamase (EC:3.5.3.8); K01479 formiminoglutamase [EC:3.5.3.8] | ec:3.5.3.8 |
| cti:RALTA\_A0653 | histidine permease |  |
| cti:RALTA\_A0654 | hutU2; urocanate hydratase (EC:4.2.1.49); K01712 urocanate hydratase [EC:4.2.1.49] | ec:4.2.1.49 |
| cti:RALTA\_A0655 | hutC2; GntR family transcriptional regulator; K05836 GntR family transcriptional regulator, histidine utilization repressor |  |
| cti:RALTA\_A0656 | sensor protein |  |
| cti:RALTA\_A0657 | hypothetical protein |  |
| cti:RALTA\_A0658 | di-heme cytochrome C-signal peptide |  |
| cti:RALTA\_A0659 | acyltransferase (EC:2.3.-.-) |  |
| cti:RALTA\_A0660 | isoquinoline 1-oxidoreductase (subunit alpha) oxidoreductase (iora2); 2fe-2S ferredoxin domain (EC:1.3.99.16); K07302 isoquinoline 1-oxidoreductase, alpha subunit [EC:1.3.99.16] | ec:1.3.99.16 |
| cti:RALTA\_A0661 | isoquinoline 1-oxidoreductase subunit beta (large chain) (iorb) (EC:1.3.99.16); K07303 isoquinoline 1-oxidoreductase, beta subunit [EC:1.3.99.16] | ec:1.3.99.16 |
| cti:RALTA\_A0662 | hypothetical protein |  |
| cti:RALTA\_A0663 | lipoprotein |  |
| cti:RALTA\_A0665 | hypothetical protein |  |
| cti:RALTA\_A0666 | TetR family transcriptional regulator |  |

  
**Neighborhood Representations for "ypb:YPTS\_2022"**  

| ID | Annotation | EC number |
| --- | --- | --- |
| ypb:YPTS\_2012 | arginine succinyltransferase; K00673 arginine N-succinyltransferase [EC:2.3.1.109] | ec:2.3.1.109 |
| ypb:YPTS\_2013 | astD; succinylglutamic semialdehyde dehydrogenase; K06447 succinylglutamic semialdehyde dehydrogenase [EC:1.2.1.71] | ec:1.2.1.71 |
| ypb:YPTS\_2014 | succinylarginine dihydrolase; K01484 succinylarginine dihydrolase [EC:3.5.3.23] | ec:3.5.3.23 |
| ypb:YPTS\_2015 | succinylglutamate desuccinylase; K05526 succinylglutamate desuccinylase [EC:3.5.1.96] | ec:3.5.1.96 |
| ypb:YPTS\_2016 | porin |  |
| ypb:YPTS\_2017 | transposase/IS protein |  |
| ypb:YPTS\_2018 | Integrase catalytic subunit |  |
| ypb:YPTS\_2019 | porin |  |
| ypb:YPTS\_2020 | N-formylglutamate amidohydrolase; K01479 formiminoglutamase [EC:3.5.3.8] | ec:3.5.3.8 |
| ypb:YPTS\_2021 | imidazolonepropionase; K01468 imidazolonepropionase [EC:3.5.2.7] | ec:3.5.2.7 |
| ypb:YPTS\_2022 | histidine utilization repressor; K05836 GntR family transcriptional regulator, histidine utilization repressor |  |
| ypb:YPTS\_2023 | N-formimino-L-glutamate deiminase; K05603 formimidoylglutamate deiminase [EC:3.5.3.13] | ec:3.5.3.13 |
| ypb:YPTS\_2024 | hypothetical protein; K09975 hypothetical protein |  |
| ypb:YPTS\_2025 | chorismate binding-like protein |  |
| ypb:YPTS\_2026 | 3-oxoacyl-ACP synthase (EC:2.3.1.41); K00648 3-oxoacyl-[acyl-carrier-protein] synthase III [EC:2.3.1.180] | ec:2.3.1.180 |
| ypb:YPTS\_2027 | NAD-dependent epimerase/dehydratase |  |
| ypb:YPTS\_2028 | beta-lactamase domain-containing protein |  |
| ypb:YPTS\_2029 | adenylate-forming protein |  |
| ypb:YPTS\_2030 | NAD-dependent epimerase/dehydratase |  |
| ypb:YPTS\_2031 | putative glycosyl transferase; K00720 ceramide glucosyltransferase [EC:2.4.1.80] | ec:2.4.1.80 |
| ypb:YPTS\_2032 | hypothetical protein |  |

  
**Neighborhood Representations for "yps:YPTB1967"**  

| ID | Annotation | EC number |
| --- | --- | --- |
| yps:YPTB1957 | narX; nitrate/nitrite sensor protein NarX (EC:2.7.3.-); K07673 two-component system, NarL family, nitrate/nitrite sensor histidine kinase NarX [EC:2.7.13.3] | ec:2.7.13.3 |
| yps:YPTB1958 | GntR family transcriptional regulator |  |
| yps:YPTB1959 | argD; bifunctional succinylornithine transaminase/acetylornithine transaminase (EC:2.6.1.-); K00840 succinylornithine aminotransferase [EC:2.6.1.81] | ec:2.6.1.81 |
| yps:YPTB1960 | astA; arginine succinyltransferase (EC:2.3.1.109); K00673 arginine N-succinyltransferase [EC:2.3.1.109] | ec:2.3.1.109 |
| yps:YPTB1961 | astD; succinylglutamic semialdehyde dehydrogenase (EC:1.2.1.-); K06447 succinylglutamic semialdehyde dehydrogenase [EC:1.2.1.71] | ec:1.2.1.71 |
| yps:YPTB1962 | astB; succinylarginine dihydrolase (EC:3.5.3.23); K01484 succinylarginine dihydrolase [EC:3.5.3.23] | ec:3.5.3.23 |
| yps:YPTB1963 | astE; succinylglutamate desuccinylase (EC:3.1.-.-); K05526 succinylglutamate desuccinylase [EC:3.5.1.96] | ec:3.5.1.96 |
| yps:YPTB1964 | ompC2; outer membrane protein OmpC |  |
| yps:YPTB1965 | hutG; N-formylglutamate amidohydrolase (EC:3.5.3.8); K01479 formiminoglutamase [EC:3.5.3.8] | ec:3.5.3.8 |
| yps:YPTB1966 | hutI; imidazolonepropionase (EC:3.5.2.7); K01468 imidazolonepropionase [EC:3.5.2.7] | ec:3.5.2.7 |
| yps:YPTB1967 | hutC; GntR family transcriptional regulator; K05836 GntR family transcriptional regulator, histidine utilization repressor |  |
| yps:YPTB1968 | N-formimino-L-glutamate deiminase; K05603 formimidoylglutamate deiminase [EC:3.5.3.13] | ec:3.5.3.13 |
| yps:YPTB1969 | hypothetical protein; K09975 hypothetical protein |  |
| yps:YPTB1970 | hypothetical protein |  |
| yps:YPTB1971 | 3-oxoacyl-ACP synthase (EC:2.3.1.41); K00648 3-oxoacyl-[acyl-carrier-protein] synthase III [EC:2.3.1.180] | ec:2.3.1.180 |
| yps:YPTB1972 | dehydrogenase |  |
| yps:YPTB1973 | hypothetical protein |  |
| yps:YPTB1974 | coenzyme synthetase |  |
| yps:YPTB1975 | dehydrogenase |  |
| yps:YPTB1976 | glycosyl transferase family protein (EC:2.4.1.80); K00720 ceramide glucosyltransferase [EC:2.4.1.80] | ec:2.4.1.80 |
| yps:YPTB1977 | hypothetical protein |  |

  
**Neighborhood Representations for "ypy:YPK\_2221"**  

| ID | Annotation | EC number |
| --- | --- | --- |
| ypy:YPK\_2211 | hypothetical protein |  |
| ypy:YPK\_2212 | putative glycosyl transferase; K00720 ceramide glucosyltransferase [EC:2.4.1.80] | ec:2.4.1.80 |
| ypy:YPK\_2213 | NAD-dependent epimerase/dehydratase |  |
| ypy:YPK\_2214 | adenylate-forming protein |  |
| ypy:YPK\_2215 | beta-lactamase domain-containing protein |  |
| ypy:YPK\_2216 | NAD-dependent epimerase/dehydratase |  |
| ypy:YPK\_2217 | beta-ketoacyl-acyl-carrier-protein synthase I; K00648 3-oxoacyl-[acyl-carrier-protein] synthase III [EC:2.3.1.180] | ec:2.3.1.180 |
| ypy:YPK\_2218 | chorismate binding-like protein |  |
| ypy:YPK\_2219 | hypothetical protein; K09975 hypothetical protein |  |
| ypy:YPK\_2220 | N-formimino-L-glutamate deiminase; K05603 formimidoylglutamate deiminase [EC:3.5.3.13] | ec:3.5.3.13 |
| ypy:YPK\_2221 | histidine utilization repressor; K05836 GntR family transcriptional regulator, histidine utilization repressor |  |
| ypy:YPK\_2222 | imidazolonepropionase; K01468 imidazolonepropionase [EC:3.5.2.7] | ec:3.5.2.7 |
| ypy:YPK\_2223 | N-formylglutamate amidohydrolase; K01479 formiminoglutamase [EC:3.5.3.8] | ec:3.5.3.8 |
| ypy:YPK\_2224 | porin |  |
| ypy:YPK\_2225 | succinylglutamate desuccinylase; K05526 succinylglutamate desuccinylase [EC:3.5.1.96] | ec:3.5.1.96 |
| ypy:YPK\_2226 | succinylarginine dihydrolase; K01484 succinylarginine dihydrolase [EC:3.5.3.23] | ec:3.5.3.23 |
| ypy:YPK\_2227 | astD; succinylglutamic semialdehyde dehydrogenase; K06447 succinylglutamic semialdehyde dehydrogenase [EC:1.2.1.71] | ec:1.2.1.71 |
| ypy:YPK\_2228 | arginine succinyltransferase; K00673 arginine N-succinyltransferase [EC:2.3.1.109] | ec:2.3.1.109 |
| ypy:YPK\_2229 | bifunctional succinylornithine transaminase/acetylornithine transaminase; K00840 succinylornithine aminotransferase [EC:2.6.1.81] | ec:2.6.1.81 |
| ypy:YPK\_2230 | hypothetical protein |  |
| ypy:YPK\_2231 | GntR family transcriptional regulator |  |

  
**Neighborhood Representations for "enc:ECL\_02966"**  

| ID | Annotation | EC number |
| --- | --- | --- |
| enc:ECL\_02956 | hypothetical protein |  |
| enc:ECL\_02957 | putative amino acid transporter |  |
| enc:ECL\_02958 | dithiobiotin synthetase; K01935 dethiobiotin synthetase [EC:6.3.3.3] | ec:6.3.3.3 |
| enc:ECL\_02959 | bioC; biotin biosynthesis protein BioC; K02169 malonyl-CoA O-methyltransferase [EC:2.1.1.197] | ec:2.1.1.197 |
| enc:ECL\_02960 | bioF; 8-amino-7-oxononanoate synthase; K00652 8-amino-7-oxononanoate synthase [EC:2.3.1.47] | ec:2.3.1.47 |
| enc:ECL\_02961 | bioB; biotin synthase; K01012 biotin synthase [EC:2.8.1.6] | ec:2.8.1.6 |
| enc:ECL\_02962 | bioA; adenosylmethionine-8-amino-7-oxononanoate aminotransferase; K00833 adenosylmethionine-8-amino-7-oxononanoate aminotransferase [EC:2.6.1.62] | ec:2.6.1.62 |
| enc:ECL\_02963 | hypothetical protein; K06910 |  |
| enc:ECL\_02964 | histidine ammonia-lyase; K01745 histidine ammonia-lyase [EC:4.3.1.3] | ec:4.3.1.3 |
| enc:ECL\_02965 | urocanate hydratase; K01712 urocanate hydratase [EC:4.2.1.49] | ec:4.2.1.49 |
| enc:ECL\_02966 | GntR family transcriptional regulator; K05836 GntR family transcriptional regulator, histidine utilization repressor |  |
| enc:ECL\_02967 | formimidoylglutamase; K01479 formiminoglutamase [EC:3.5.3.8] | ec:3.5.3.8 |
| enc:ECL\_02968 | imidazolonepropionase; K01468 imidazolonepropionase [EC:3.5.2.7] | ec:3.5.2.7 |
| enc:ECL\_02969 | putative pectinesterase; K01051 pectinesterase [EC:3.1.1.11] | ec:3.1.1.11 |
| enc:ECL\_02970 | 6-phosphogluconolactonase; K07404 6-phosphogluconolactonase [EC:3.1.1.31] | ec:3.1.1.31 |
| enc:ECL\_02971 | putative phosphotransferase; K07024 |  |
| enc:ECL\_02972 | molybdate ABC transporter ATP-binding protein; K02017 molybdate transport system ATP-binding protein [EC:3.6.3.29] | ec:3.6.3.29 |
| enc:ECL\_02973 | molybdate ABC transporter permease; K02018 molybdate transport system permease protein |  |
| enc:ECL\_02974 | molybdate ABC transporter periplasmic protein; K02020 molybdate transport system substrate-binding protein |  |
| enc:ECL\_02975 | hypothetical protein |  |
| enc:ECL\_02976 | DNA-binding transcriptional regulator ModE; K02019 molybdate transport system regulatory protein |  |

  
**Neighborhood Representations for "vch:VC1206"**  

| ID | Annotation | EC number |
| --- | --- | --- |
| vch:VC1196 | hypothetical protein |  |
| vch:VC1197 | hypothetical protein |  |
| vch:VC1198 | hypothetical protein; K06911 |  |
| vch:VC1199 | hypothetical protein |  |
| vch:VC1200 | serine protease |  |
| vch:VC1201 | hypothetical protein |  |
| vch:VC1202 | hutH; histidine ammonia-lyase (EC:4.3.1.3); K01745 histidine ammonia-lyase [EC:4.3.1.3] | ec:4.3.1.3 |
| vch:VC1203 | urocanate hydratase (EC:4.2.1.49); K01712 urocanate hydratase [EC:4.2.1.49] | ec:4.2.1.49 |
| vch:VC1204 | formimidoylglutamase (EC:3.5.3.8); K01479 formiminoglutamase [EC:3.5.3.8] | ec:3.5.3.8 |
| vch:VC1205 | imidazolonepropionase (EC:3.5.2.7); K01468 imidazolonepropionase [EC:3.5.2.7] | ec:3.5.2.7 |
| vch:VC1206 | histidine utilization repressor; K05836 GntR family transcriptional regulator, histidine utilization repressor |  |
| vch:VC1207 | hypothetical protein |  |
| vch:VC1208 | dsDNA-mimic protein; K09901 hypothetical protein |  |
| vch:VC1209 | elongation factor P; K02356 elongation factor P |  |
| vch:VC1210 | hypothetical protein |  |
| vch:VC1211 | hypothetical protein |  |
| vch:VC1212 | DNA polymerase II (EC:2.7.7.7); K02336 DNA polymerase II [EC:2.7.7.7] | ec:2.7.7.7 |
| vch:VC1213 | sirA, uvrY; response regulator; K07689 two-component system, NarL family, invasion response regulator UvrY |  |
| vch:VC1214 | uvrC; excinuclease ABC subunit C; K03703 excinuclease ABC subunit C |  |
| vch:VC1215 | CDP-diacylglycerol--glycerol-3-phosphate 3-phosphatidyltransferase; K00995 CDP-diacylglycerol--glycerol-3-phosphate 3-phosphatidyltransferase [EC:2.7.8.5] | ec:2.7.8.5 |
| vch:VCt056 | tRNA-Cys-1; tRNA-Cys; K14222 tRNA Cys |  |

  
**Neighborhood Representations for "vcm:VCM66\_1161"**  

| ID | Annotation | EC number |
| --- | --- | --- |
| vcm:VCM66\_1151 | hypothetical protein |  |
| vcm:VCM66\_1152 | hypothetical protein |  |
| vcm:VCM66\_1153 | hypothetical protein; K06911 |  |
| vcm:VCM66\_1154 | hypothetical protein |  |
| vcm:VCM66\_1155 | putative trypsin (EC:3.4.21.4) |  |
| vcm:VCM66\_1156 | hypothetical protein |  |
| vcm:VCM66\_1157 | hutH; histidine ammonia-lyase (EC:4.3.1.3); K01745 histidine ammonia-lyase [EC:4.3.1.3] | ec:4.3.1.3 |
| vcm:VCM66\_1158 | hutU; urocanate hydratase (EC:4.2.1.49); K01712 urocanate hydratase [EC:4.2.1.49] | ec:4.2.1.49 |
| vcm:VCM66\_1159 | hutG; formimidoylglutamase (EC:3.5.3.8); K01479 formiminoglutamase [EC:3.5.3.8] | ec:3.5.3.8 |
| vcm:VCM66\_1160 | hutI; imidazolonepropionase (EC:3.5.2.7); K01468 imidazolonepropionase [EC:3.5.2.7] | ec:3.5.2.7 |
| vcm:VCM66\_1161 | histidine utilization repressor; K05836 GntR family transcriptional regulator, histidine utilization repressor |  |
| vcm:VCM66\_1162 | hypothetical protein |  |
| vcm:VCM66\_1163 | dsDNA-mimic protein; K09901 hypothetical protein |  |
| vcm:VCM66\_1164 | elongation factor P; K02356 elongation factor P |  |
| vcm:VCM66\_1165 | hypothetical protein |  |
| vcm:VCM66\_1166 | EAL family protein |  |
| vcm:VCM66\_1167 | polB; DNA polymerase II (EC:2.7.7.7); K02336 DNA polymerase II [EC:2.7.7.7] | ec:2.7.7.7 |
| vcm:VCM66\_1168 | response regulator; K07689 two-component system, NarL family, invasion response regulator UvrY |  |
| vcm:VCM66\_1169 | uvrC; excinuclease ABC subunit C; K03703 excinuclease ABC subunit C |  |
| vcm:VCM66\_1170 | pgsA; CDP-diacylglycerol--glycerol-3-phosphate 3-phosphatidyltransferase (EC:2.7.8.5); K00995 CDP-diacylglycerol--glycerol-3-phosphate 3-phosphatidyltransferase [EC:2.7.8.5] | ec:2.7.8.5 |
| vcm:VCM66\_1171 | GGDEF family protein; K07212 GGDEF domain K07216 hemerythrin |  |

  
**Neighborhood Representations for "vco:VC0395\_A0826"**  

| ID | Annotation | EC number |
| --- | --- | --- |
| vco:VC0395\_A0816 | hypothetical protein |  |
| vco:VC0395\_A0817 | hypothetical protein |  |
| vco:VC0395\_A0818 | hypothetical protein; K06911 |  |
| vco:VC0395\_A0819 | hypothetical protein |  |
| vco:VC0395\_A0820 | trypsin |  |
| vco:VC0395\_A0821 | hypothetical protein |  |
| vco:VC0395\_A0822 | hutH; histidine ammonia-lyase (EC:4.3.1.3); K01745 histidine ammonia-lyase [EC:4.3.1.3] | ec:4.3.1.3 |
| vco:VC0395\_A0823 | hutU; urocanate hydratase (EC:4.2.1.49); K01712 urocanate hydratase [EC:4.2.1.49] | ec:4.2.1.49 |
| vco:VC0395\_A0824 | hutG; formimidoylglutamase (EC:3.5.3.8); K01479 formiminoglutamase [EC:3.5.3.8] | ec:3.5.3.8 |
| vco:VC0395\_A0825 | hutI; imidazolonepropionase (EC:3.5.2.7); K01468 imidazolonepropionase [EC:3.5.2.7] | ec:3.5.2.7 |
| vco:VC0395\_A0826 | hutC; histidine utilization repressor; K05836 GntR family transcriptional regulator, histidine utilization repressor |  |
| vco:VC0395\_A0827 | hypothetical protein |  |
| vco:VC0395\_A0828 | dsDNA-mimic protein; K09901 hypothetical protein |  |
| vco:VC0395\_A0829 | elongation factor P; K02356 elongation factor P |  |
| vco:VC0395\_A0830 | hypothetical protein |  |
| vco:VC0395\_A0832 | hypothetical protein |  |
| vco:VC0395\_A0831 | polB; DNA polymerase II (EC:2.7.7.7); K02336 DNA polymerase II [EC:2.7.7.7] | ec:2.7.7.7 |
| vco:VC0395\_A0833 | sirA, uvrY; response regulator; K07689 two-component system, NarL family, invasion response regulator UvrY |  |
| vco:VC0395\_A0834 | uvrC; excinuclease ABC subunit C; K03703 excinuclease ABC subunit C |  |
| vco:VC0395\_A0835 | pgsA; CDP-diacylglycerol--glycerol-3-phosphate 3-phosphatidyltransferase (EC:2.7.8.5); K00995 CDP-diacylglycerol--glycerol-3-phosphate 3-phosphatidyltransferase [EC:2.7.8.5] | ec:2.7.8.5 |
| vco:VC0395\_A0836 | tRNA-Cys; K14222 tRNA Cys |  |

  
**Neighborhood Representations for "spl:Spea\_4170"**  

| ID | Annotation | EC number |
| --- | --- | --- |
| spl:Spea\_4160 | iron-containing alcohol dehydrogenase |  |
| spl:Spea\_4161 | hypothetical protein |  |
| spl:Spea\_4162 | hypothetical protein |  |
| spl:Spea\_4163 | hypothetical protein |  |
| spl:Spea\_4164 | 1-aminocyclopropane-1-carboxylate deaminase (EC:3.5.99.7); K05396 D-cysteine desulfhydrase [EC:4.4.1.15] | ec:4.4.1.15 |
| spl:Spea\_4165 | Na+/H+ antiporter NhaC |  |
| spl:Spea\_4166 | putative endoribonuclease L-PSP; K09022 UPF0076 protein YjgF |  |
| spl:Spea\_4167 | LysR family transcriptional regulator |  |
| spl:Spea\_4168 | histidine ammonia-lyase (EC:4.3.1.3); K01745 histidine ammonia-lyase [EC:4.3.1.3] | ec:4.3.1.3 |
| spl:Spea\_4169 | urocanate hydratase (EC:4.2.1.49); K01712 urocanate hydratase [EC:4.2.1.49] | ec:4.2.1.49 |
| spl:Spea\_4170 | histidine utilization repressor; K05836 GntR family transcriptional regulator, histidine utilization repressor |  |
| spl:Spea\_4171 | imidazolonepropionase (EC:3.5.2.7); K01468 imidazolonepropionase [EC:3.5.2.7] | ec:3.5.2.7 |
| spl:Spea\_4172 | hypothetical protein |  |
| spl:Spea\_4173 | ATPase AAA |  |
| spl:Spea\_4174 | hypothetical protein |  |
| spl:Spea\_4175 | ABC transporter-like protein; K02003 putative ABC transport system ATP-binding protein |  |
| spl:Spea\_4176 | hypothetical protein |  |
| spl:Spea\_4177 | hypothetical protein |  |
| spl:Spea\_4178 | lytic transglycosylase; K08306 membrane-bound lytic murein transglycosylase C [EC:3.2.1.-] |  |
| spl:Spea\_4179 | hypothetical protein; K07017 |  |
| spl:Spea\_4180 | 2,3-diketo-5-methylthio-1-phosphopentane phosphatase; K09880 enolase-phosphatase E1 [EC:3.1.3.77] | ec:3.1.3.77 |

  
**Neighborhood Representations for "yen:YE2460"**  

| ID | Annotation | EC number |
| --- | --- | --- |
| yen:YE2448 | agaR; DNA-binding transcriptional regulator AgaR; K02081 DeoR family transcriptional regulator, aga operon transcriptional repressor |  |
| yen:YE2449 | agaS; pseudogene |  |
| yen:YE2450 | agaY; pseudogene |  |
| yen:YE2451 | lipoprotein; K12943 lipoprotein YgeR |  |
| yen:YE2452 | pseudogene |  |
| yen:YE2454 | hypothetical protein |  |
| yen:YE2455 | hypothetical protein |  |
| yen:YE2457 | hypothetical protein |  |
| yen:YE2458 | hypothetical protein; K09975 hypothetical protein |  |
| yen:YE2459 | N-formimino-L-glutamate deiminase; K05603 formimidoylglutamate deiminase [EC:3.5.3.13] | ec:3.5.3.13 |
| yen:YE2460 | hutC; GntR family transcriptional regulator; K05836 GntR family transcriptional regulator, histidine utilization repressor |  |
| yen:YE2461 | hutI; imidazolonepropionase (EC:3.5.2.7); K01468 imidazolonepropionase [EC:3.5.2.7] | ec:3.5.2.7 |
| yen:YE2462 | hutG; N-formylglutamate amidohydrolase; K01479 formiminoglutamase [EC:3.5.3.8] | ec:3.5.3.8 |
| yen:YE2463 | outer membrane porin protein |  |
| yen:YE2465 | astE; succinylglutamate desuccinylase; K05526 succinylglutamate desuccinylase [EC:3.5.1.96] | ec:3.5.1.96 |
| yen:YE2466 | astB; succinylarginine dihydrolase (EC:3.5.3.23); K01484 succinylarginine dihydrolase [EC:3.5.3.23] | ec:3.5.3.23 |
| yen:YE2467 | astD; succinylglutamic semialdehyde dehydrogenase; K06447 succinylglutamic semialdehyde dehydrogenase [EC:1.2.1.71] | ec:1.2.1.71 |
| yen:YE2468 | astA; arginine succinyltransferase; K00673 arginine N-succinyltransferase [EC:2.3.1.109] | ec:2.3.1.109 |
| yen:YE2469 | argM; bifunctional succinylornithine transaminase/acetylornithine transaminase; K00840 succinylornithine aminotransferase [EC:2.6.1.81] | ec:2.6.1.81 |
| yen:YE2470 | GntR family transcriptional regulator |  |
| yen:YE2471 | cytochrome oxidase subunit; K00425 cytochrome d ubiquinol oxidase subunit I [EC:1.10.3.-] |  |

  
**Neighborhood Representations for "ypa:YPA\_1355"**  

| ID | Annotation | EC number |
| --- | --- | --- |
| ypa:YPA\_1345 | arginine succinyltransferase (EC:2.3.1.109); K00673 arginine N-succinyltransferase [EC:2.3.1.109] | ec:2.3.1.109 |
| ypa:YPA\_1346 | astD; succinylglutamic semialdehyde dehydrogenase; K06447 succinylglutamic semialdehyde dehydrogenase [EC:1.2.1.71] | ec:1.2.1.71 |
| ypa:YPA\_1347 | succinylarginine dihydrolase (EC:3.5.3.23); K01484 succinylarginine dihydrolase [EC:3.5.3.23] | ec:3.5.3.23 |
| ypa:YPA\_1348 | succinylglutamate desuccinylase; K05526 succinylglutamate desuccinylase [EC:3.5.1.96] | ec:3.5.1.96 |
| ypa:YPA\_1349 | hypothetical protein |  |
| ypa:YPA\_1350 | transposase for insertion sequence IS100 |  |
| ypa:YPA\_1351 | transposase/IS protein |  |
| ypa:YPA\_1352 | outer membrane protein C2, porin |  |
| ypa:YPA\_1353 | putative N-formylglutamate amidohydrolase; K01479 formiminoglutamase [EC:3.5.3.8] | ec:3.5.3.8 |
| ypa:YPA\_1354 | imidazolonepropionase (EC:3.5.2.7); K01468 imidazolonepropionase [EC:3.5.2.7] | ec:3.5.2.7 |
| ypa:YPA\_1355 | GntR family transcriptional regulator; K05836 GntR family transcriptional regulator, histidine utilization repressor |  |
| ypa:YPA\_1356 | N-formimino-L-glutamate deiminase; K05603 formimidoylglutamate deiminase [EC:3.5.3.13] | ec:3.5.3.13 |
| ypa:YPA\_1357 | hypothetical protein; K09975 hypothetical protein |  |
| ypa:YPA\_1358 | hypothetical protein |  |
| ypa:YPA\_1359 | hypothetical protein; K00648 3-oxoacyl-[acyl-carrier-protein] synthase III [EC:2.3.1.180] | ec:2.3.1.180 |
| ypa:YPA\_1360 | 3-oxoacyl-ACP synthase; K00648 3-oxoacyl-[acyl-carrier-protein] synthase III [EC:2.3.1.180] | ec:2.3.1.180 |
| ypa:YPA\_1361 | putative dehydrogenase |  |
| ypa:YPA\_1362 | hypothetical protein |  |
| ypa:YPA\_1363 | putative coenzyme synthetase |  |
| ypa:YPA\_1364 | putative dehydrogenase |  |
| ypa:YPA\_1365 | putative glycosyl transferase; K00720 ceramide glucosyltransferase [EC:2.4.1.80] | ec:2.4.1.80 |

  
**Neighborhood Representations for "ype:YPO1973"**  

| ID | Annotation | EC number |
| --- | --- | --- |
| ype:YPO1960 | GntR family transcriptional regulator |  |
| ype:YPO1962 | argD; bifunctional succinylornithine transaminase/acetylornithine transaminase (EC:2.6.1.-); K00840 succinylornithine aminotransferase [EC:2.6.1.81] | ec:2.6.1.81 |
| ype:YPO1963 | astA; arginine succinyltransferase (EC:2.3.1.109); K00673 arginine N-succinyltransferase [EC:2.3.1.109] | ec:2.3.1.109 |
| ype:YPO1964 | astD; succinylglutamic semialdehyde dehydrogenase (EC:1.2.1.-); K06447 succinylglutamic semialdehyde dehydrogenase [EC:1.2.1.71] | ec:1.2.1.71 |
| ype:YPO1965 | astB; succinylarginine dihydrolase (EC:3.5.3.23); K01484 succinylarginine dihydrolase [EC:3.5.3.23] | ec:3.5.3.23 |
| ype:YPO1966 | astE; succinylglutamate desuccinylase (EC:3.1.-.-); K05526 succinylglutamate desuccinylase [EC:3.5.1.96] | ec:3.5.1.96 |
| ype:YPO1968 | y1093; transposase for insertion sequence IS100 |  |
| ype:YPO1969 | transposase/IS protein |  |
| ype:YPO1971 | hutG; N-formylglutamate amidohydrolase; K01479 formiminoglutamase [EC:3.5.3.8] | ec:3.5.3.8 |
| ype:YPO1972 | hutI; imidazolonepropionase (EC:3.5.2.7); K01468 imidazolonepropionase [EC:3.5.2.7] | ec:3.5.2.7 |
| ype:YPO1973 | hutC; GntR family transcriptional regulator; K05836 GntR family transcriptional regulator, histidine utilization repressor |  |
| ype:YPO1974 | N-formimino-L-glutamate deiminase; K05603 formimidoylglutamate deiminase [EC:3.5.3.13] | ec:3.5.3.13 |
| ype:YPO1975 | hypothetical protein; K09975 hypothetical protein |  |
| ype:YPO1976 | hypothetical protein |  |
| ype:YPO1977 | pseudogene |  |
| ype:YPO1979 | dehydrogenase |  |
| ype:YPO1980 | hypothetical protein |  |
| ype:YPO1981 | coenzyme synthetase |  |
| ype:YPO1982 | dehydrogenase |  |
| ype:YPO1983 | glycosyl transferase (EC:2.4.1.80); K00720 ceramide glucosyltransferase [EC:2.4.1.80] | ec:2.4.1.80 |
| ype:YPO1984 | hypothetical protein |  |

  
**Neighborhood Representations for "ypg:YpAngola\_A2511"**  

| ID | Annotation | EC number |
| --- | --- | --- |
| ypg:YpAngola\_A2501 | pseudogene |  |
| ypg:YpAngola\_A2502 | NAD dependent epimerase/dehydratase family protein |  |
| ypg:YpAngola\_A2503 | hypothetical protein |  |
| ypg:YpAngola\_A2504 | pseudogene |  |
| ypg:YpAngola\_A2505 | NAD-dependent epimerase/dehydratase family protein |  |
| ypg:YpAngola\_A2506 | pseudogene |  |
| ypg:YpAngola\_A2507 | hypothetical protein |  |
| ypg:YpAngola\_A2508 | chorismate-binding domain-containing protein |  |
| ypg:YpAngola\_A2509 | hypothetical protein; K09975 hypothetical protein |  |
| ypg:YpAngola\_A2510 | hutF; N-formimino-L-glutamate deiminase (EC:3.5.3.13); K05603 formimidoylglutamate deiminase [EC:3.5.3.13] | ec:3.5.3.13 |
| ypg:YpAngola\_A2511 | hutC; histidine utilization repressor; K05836 GntR family transcriptional regulator, histidine utilization repressor |  |
| ypg:YpAngola\_A2512 | hutI; imidazolonepropionase (EC:3.5.2.7); K01468 imidazolonepropionase [EC:3.5.2.7] | ec:3.5.2.7 |
| ypg:YpAngola\_A2513 | hutG; N-formylglutamate amidohydrolase (EC:3.5.1.68); K01479 formiminoglutamase [EC:3.5.3.8] | ec:3.5.3.8 |
| ypg:YpAngola\_A2514 | outer membrane protein N |  |
| ypg:YpAngola\_A2515 | hypothetical protein |  |
| ypg:YpAngola\_A2516 | astE; succinylglutamate desuccinylase; K05526 succinylglutamate desuccinylase [EC:3.5.1.96] | ec:3.5.1.96 |
| ypg:YpAngola\_A2517 | astB; succinylarginine dihydrolase; K01484 succinylarginine dihydrolase [EC:3.5.3.23] | ec:3.5.3.23 |
| ypg:YpAngola\_A2518 | astD; succinylglutamic semialdehyde dehydrogenase (EC:1.2.1.-); K06447 succinylglutamic semialdehyde dehydrogenase [EC:1.2.1.71] | ec:1.2.1.71 |
| ypg:YpAngola\_A2519 | astA; arginine succinyltransferase (EC:2.3.1.109); K00673 arginine N-succinyltransferase [EC:2.3.1.109] | ec:2.3.1.109 |
| ypg:YpAngola\_A2520 | argM; bifunctional succinylornithine transaminase/acetylornithine transaminase (EC:2.6.1.-); K00840 succinylornithine aminotransferase [EC:2.6.1.81] | ec:2.6.1.81 |
| ypg:YpAngola\_A2521 | aminotransferase |  |

  
**Neighborhood Representations for "ypi:YpsIP31758\_2112"**  

| ID | Annotation | EC number |
| --- | --- | --- |
| ypi:YpsIP31758\_2102 | hypothetical protein; K00720 ceramide glucosyltransferase [EC:2.4.1.80] | ec:2.4.1.80 |
| ypi:YpsIP31758\_2103 | NAD dependent epimerase/dehydratase family protein |  |
| ypi:YpsIP31758\_2105 | hypothetical protein |  |
| ypi:YpsIP31758\_2104 | hypothetical protein |  |
| ypi:YpsIP31758\_2106 | metallo-beta-lactamase family protein |  |
| ypi:YpsIP31758\_2107 | NAD-dependent epimerase/dehydratase family protein |  |
| ypi:YpsIP31758\_2108 | hypothetical protein; K00648 3-oxoacyl-[acyl-carrier-protein] synthase III [EC:2.3.1.180] | ec:2.3.1.180 |
| ypi:YpsIP31758\_2109 | chorismate-binding domain-containing protein |  |
| ypi:YpsIP31758\_2110 | cold inducible protein Ves; K09975 hypothetical protein |  |
| ypi:YpsIP31758\_2111 | hutF; N-formimino-L-glutamate deiminase (EC:3.5.3.13); K05603 formimidoylglutamate deiminase [EC:3.5.3.13] | ec:3.5.3.13 |
| ypi:YpsIP31758\_2112 | hutC; histidine utilization repressor; K05836 GntR family transcriptional regulator, histidine utilization repressor |  |
| ypi:YpsIP31758\_2113 | hutI; imidazolonepropionase (EC:3.5.2.7); K01468 imidazolonepropionase [EC:3.5.2.7] | ec:3.5.2.7 |
| ypi:YpsIP31758\_2114 | hutG; N-formylglutamate amidohydrolase (EC:3.5.1.68); K01479 formiminoglutamase [EC:3.5.3.8] | ec:3.5.3.8 |
| ypi:YpsIP31758\_2115 | outer membrane protein N |  |
| ypi:YpsIP31758\_2116 | hypothetical protein |  |
| ypi:YpsIP31758\_2117 | astE; succinylglutamate desuccinylase; K05526 succinylglutamate desuccinylase [EC:3.5.1.96] | ec:3.5.1.96 |
| ypi:YpsIP31758\_2118 | astB; succinylarginine dihydrolase; K01484 succinylarginine dihydrolase [EC:3.5.3.23] | ec:3.5.3.23 |
| ypi:YpsIP31758\_2119 | astD; succinylglutamic semialdehyde dehydrogenase (EC:1.2.1.-); K06447 succinylglutamic semialdehyde dehydrogenase [EC:1.2.1.71] | ec:1.2.1.71 |
| ypi:YpsIP31758\_2120 | astA; arginine succinyltransferase (EC:2.3.1.109); K00673 arginine N-succinyltransferase [EC:2.3.1.109] | ec:2.3.1.109 |
| ypi:YpsIP31758\_2121 | argM; bifunctional succinylornithine transaminase/acetylornithine transaminase (EC:2.6.1.-); K00840 succinylornithine aminotransferase [EC:2.6.1.81] | ec:2.6.1.81 |
| ypi:YpsIP31758\_2122 | GntR family transcriptional regulator |  |

  
**Neighborhood Representations for "ypk:y2339"**  

| ID | Annotation | EC number |
| --- | --- | --- |
| ypk:y2329 | oxidoreductase |  |
| ypk:y2330 | hypothetical protein |  |
| ypk:y2331 | hypothetical protein |  |
| ypk:y2332 | hypothetical protein |  |
| ypk:y2333 | nucleotide di-P-sugar epimerase or dehydratase |  |
| ypk:y2334 | hypothetical protein; K00648 3-oxoacyl-[acyl-carrier-protein] synthase III [EC:2.3.1.180] | ec:2.3.1.180 |
| ypk:y2335 | hypothetical protein; K00648 3-oxoacyl-[acyl-carrier-protein] synthase III [EC:2.3.1.180] | ec:2.3.1.180 |
| ypk:y2336 | hypothetical protein |  |
| ypk:y2337 | hypothetical protein; K09975 hypothetical protein |  |
| ypk:y2338 | N-formimino-L-glutamate deiminase; K05603 formimidoylglutamate deiminase [EC:3.5.3.13] | ec:3.5.3.13 |
| ypk:y2339 | repressor; K05836 GntR family transcriptional regulator, histidine utilization repressor |  |
| ypk:y2340 | imidazolonepropionase (EC:3.5.2.7); K01468 imidazolonepropionase [EC:3.5.2.7] | ec:3.5.2.7 |
| ypk:y2341 | histidine degradation enzyme; K01479 formiminoglutamase [EC:3.5.3.8] | ec:3.5.3.8 |
| ypk:y2344 | pseudogene |  |
| ypk:y2342 | transposase/IS protein |  |
| ypk:y2343 | transposase, N end of IS100 transframe protein |  |
| ypk:y2345 | succinylglutamate desuccinylase; K05526 succinylglutamate desuccinylase [EC:3.5.1.96] | ec:3.5.1.96 |
| ypk:y2346 | succinylarginine dihydrolase (EC:3.5.3.23); K01484 succinylarginine dihydrolase [EC:3.5.3.23] | ec:3.5.3.23 |
| ypk:y2347 | astD; succinylglutamic semialdehyde dehydrogenase; K06447 succinylglutamic semialdehyde dehydrogenase [EC:1.2.1.71] | ec:1.2.1.71 |
| ypk:y2348 | arginine succinyltransferase; K00673 arginine N-succinyltransferase [EC:2.3.1.109] | ec:2.3.1.109 |
| ypk:y2349 | argD; bifunctional succinylornithine transaminase/acetylornithine transaminase; K00840 succinylornithine aminotransferase [EC:2.6.1.81] | ec:2.6.1.81 |

  
**Neighborhood Representations for "ypn:YPN\_1453"**  

| ID | Annotation | EC number |
| --- | --- | --- |
| ypn:YPN\_1443 | hypothetical protein; K11900 type VI secretion system protein ImpC |  |
| ypn:YPN\_1444 | hypothetical protein; K11903 type VI secretion system secreted protein Hcp |  |
| ypn:YPN\_1445 | hypothetical protein; K11906 type VI secretion system protein VasD |  |
| ypn:YPN\_1446 | hypothetical protein; K11893 type VI secretion system protein ImpJ |  |
| ypn:YPN\_1447 | hypothetical protein |  |
| ypn:YPN\_1448 | transposase for insertion sequence IS100 |  |
| ypn:YPN\_1449 | transposase/IS protein |  |
| ypn:YPN\_1450 | outer membrane protein C2, porin |  |
| ypn:YPN\_1451 | N-formylglutamate amidohydrolase; K01479 formiminoglutamase [EC:3.5.3.8] | ec:3.5.3.8 |
| ypn:YPN\_1452 | imidazolonepropionase (EC:3.5.2.7); K01468 imidazolonepropionase [EC:3.5.2.7] | ec:3.5.2.7 |
| ypn:YPN\_1453 | GntR family transcriptional regulator; K05836 GntR family transcriptional regulator, histidine utilization repressor |  |
| ypn:YPN\_1454 | N-formimino-L-glutamate deiminase; K05603 formimidoylglutamate deiminase [EC:3.5.3.13] | ec:3.5.3.13 |
| ypn:YPN\_1455 | hypothetical protein; K09975 hypothetical protein |  |
| ypn:YPN\_1456 | hypothetical protein |  |
| ypn:YPN\_1457 | hypothetical protein; K00648 3-oxoacyl-[acyl-carrier-protein] synthase III [EC:2.3.1.180] | ec:2.3.1.180 |
| ypn:YPN\_1458 | 3-oxoacyl-ACP synthase; K00648 3-oxoacyl-[acyl-carrier-protein] synthase III [EC:2.3.1.180] | ec:2.3.1.180 |
| ypn:YPN\_1459 | dehydrogenase |  |
| ypn:YPN\_1460 | hypothetical protein |  |
| ypn:YPN\_1461 | coenzyme synthetase |  |
| ypn:YPN\_1462 | dehydrogenase |  |
| ypn:YPN\_1463 | glycosyl transferase family protein; K00720 ceramide glucosyltransferase [EC:2.4.1.80] | ec:2.4.1.80 |

  
**Neighborhood Representations for "ypp:YPDSF\_1150"**  

| ID | Annotation | EC number |
| --- | --- | --- |
| ypp:YPDSF\_1140 | glycosyl transferase; K00720 ceramide glucosyltransferase [EC:2.4.1.80] | ec:2.4.1.80 |
| ypp:YPDSF\_1141 | dehydrogenase |  |
| ypp:YPDSF\_1142 | coenzyme synthetase |  |
| ypp:YPDSF\_1143 | hypothetical protein |  |
| ypp:YPDSF\_1144 | dehydrogenase |  |
| ypp:YPDSF\_1145 | 3-oxoacyl-ACP synthase; K00648 3-oxoacyl-[acyl-carrier-protein] synthase III [EC:2.3.1.180] | ec:2.3.1.180 |
| ypp:YPDSF\_1146 | hypothetical protein; K00648 3-oxoacyl-[acyl-carrier-protein] synthase III [EC:2.3.1.180] | ec:2.3.1.180 |
| ypp:YPDSF\_1147 | hypothetical protein |  |
| ypp:YPDSF\_1148 | hypothetical protein; K09975 hypothetical protein |  |
| ypp:YPDSF\_1149 | N-formimino-L-glutamate deiminase; K05603 formimidoylglutamate deiminase [EC:3.5.3.13] | ec:3.5.3.13 |
| ypp:YPDSF\_1150 | GntR family transcriptional regulator; K05836 GntR family transcriptional regulator, histidine utilization repressor |  |
| ypp:YPDSF\_1151 | imidazolonepropionase (EC:3.5.2.7); K01468 imidazolonepropionase [EC:3.5.2.7] | ec:3.5.2.7 |
| ypp:YPDSF\_1152 | transposase |  |
| ypp:YPDSF\_1153 | transposase/IS protein |  |
| ypp:YPDSF\_1154 | N-formylglutamate amidohydrolase; K01479 formiminoglutamase [EC:3.5.3.8] | ec:3.5.3.8 |
| ypp:YPDSF\_1155 | transposase for the IS285 insertion element; K07493 putative transposase |  |
| ypp:YPDSF\_1156 | hypothetical protein |  |
| ypp:YPDSF\_1157 | succinylglutamate desuccinylase; K05526 succinylglutamate desuccinylase [EC:3.5.1.96] | ec:3.5.1.96 |
| ypp:YPDSF\_1158 | succinylarginine dihydrolase (EC:3.5.3.23); K01484 succinylarginine dihydrolase [EC:3.5.3.23] | ec:3.5.3.23 |
| ypp:YPDSF\_1159 | astD; succinylglutamic semialdehyde dehydrogenase; K06447 succinylglutamic semialdehyde dehydrogenase [EC:1.2.1.71] | ec:1.2.1.71 |
| ypp:YPDSF\_1160 | arginine succinyltransferase (EC:2.3.1.109); K00673 arginine N-succinyltransferase [EC:2.3.1.109] | ec:2.3.1.109 |

  
**Neighborhood Representations for "ypz:YPZ3\_1845"**  

| ID | Annotation | EC number |
| --- | --- | --- |
| ypz:YPZ3\_1835 | putative glycosyl transferase; K00720 ceramide glucosyltransferase [EC:2.4.1.80] | ec:2.4.1.80 |
| ypz:YPZ3\_1836 | putative dehydrogenase |  |
| ypz:YPZ3\_1837 | putative coenzyme synthetase |  |
| ypz:YPZ3\_1838 | hypothetical protein |  |
| ypz:YPZ3\_1839 | putative dehydrogenase |  |
| ypz:YPZ3\_1840 | 3-oxoacyl-(acyl-carrier-protein) synthase III; K00648 3-oxoacyl-[acyl-carrier-protein] synthase III [EC:2.3.1.180] | ec:2.3.1.180 |
| ypz:YPZ3\_1841 | hypothetical protein; K00648 3-oxoacyl-[acyl-carrier-protein] synthase III [EC:2.3.1.180] | ec:2.3.1.180 |
| ypz:YPZ3\_1842 | hypothetical protein |  |
| ypz:YPZ3\_1843 | hypothetical protein; K09975 hypothetical protein |  |
| ypz:YPZ3\_1844 | atrazine chlorohydrolase; K05603 formimidoylglutamate deiminase [EC:3.5.3.13] | ec:3.5.3.13 |
| ypz:YPZ3\_1845 | hutC; GntR family transcriptional regulator; K05836 GntR family transcriptional regulator, histidine utilization repressor |  |
| ypz:YPZ3\_1846 | hutI; imidazolonepropionase; K01468 imidazolonepropionase [EC:3.5.2.7] | ec:3.5.2.7 |
| ypz:YPZ3\_1847 | hutG; putative N-formylglutamate amidohydrolase; K01479 formiminoglutamase [EC:3.5.3.8] | ec:3.5.3.8 |
| ypz:YPZ3\_1848 | insertion sequence IS100, ATP-binding protein |  |
| ypz:YPZ3\_1849 | transposase for insertion sequence IS100 |  |
| ypz:YPZ3\_1850 | putative binding-protein-dependent transport system, ATP-binding component; K10111 multiple sugar transport system ATP-binding protein [EC:3.6.3.-] |  |
| ypz:YPZ3\_1851 | putative binding-protein-dependent transport system, inner membrane component; K02026 multiple sugar transport system permease protein |  |
| ypz:YPZ3\_1852 | putative binding-protein-dependent transport system, inner membrane component; K02025 multiple sugar transport system permease protein |  |
| ypz:YPZ3\_1853 | putative exported solute-binding protein; K02027 multiple sugar transport system substrate-binding protein |  |
| ypz:YPZ3\_1854 | putative oxidoreductase |  |
| ypz:YPZ3\_1855 | GntR family transcriptional regulator; K03710 GntR family transcriptional regulator |  |

  
**Neighborhood Representations for "swd:Swoo\_4839"**  

| ID | Annotation | EC number |
| --- | --- | --- |
| swd:Swoo\_4829 | hypothetical protein |  |
| swd:Swoo\_4830 | hypothetical protein |  |
| swd:Swoo\_4831 | hypothetical protein |  |
| swd:Swoo\_4832 | phospholipid/glycerol acyltransferase |  |
| swd:Swoo\_4833 | lysine exporter protein LysE/YggA |  |
| swd:Swoo\_4834 | mechanosensitive ion channel protein MscS; K16053 miniconductance mechanosensitive channel |  |
| swd:Swoo\_4835 | hypothetical protein |  |
| swd:Swoo\_4836 | hypothetical protein |  |
| swd:Swoo\_4837 | histidine ammonia-lyase (EC:4.3.1.3); K01745 histidine ammonia-lyase [EC:4.3.1.3] | ec:4.3.1.3 |
| swd:Swoo\_4838 | urocanate hydratase (EC:4.2.1.49); K01712 urocanate hydratase [EC:4.2.1.49] | ec:4.2.1.49 |
| swd:Swoo\_4839 | histidine utilization repressor; K05836 GntR family transcriptional regulator, histidine utilization repressor |  |
| swd:Swoo\_4840 | imidazolonepropionase (EC:3.5.2.7); K01468 imidazolonepropionase [EC:3.5.2.7] | ec:3.5.2.7 |
| swd:Swoo\_4841 | hypothetical protein |  |
| swd:Swoo\_4842 | hypothetical protein |  |
| swd:Swoo\_4843 | OmpA domain-containing protein |  |
| swd:Swoo\_4844 | hypothetical protein |  |
| swd:Swoo\_4845 | hypothetical protein; K07017 |  |
| swd:Swoo\_4846 | LytTR family two component transcriptional regulator; K02477 two-component system, LytT family, response regulator |  |
| swd:Swoo\_4847 | signal transduction histidine kinase LytS |  |
| swd:Swoo\_4848 | hypothetical protein; K02004 putative ABC transport system permease protein |  |
| swd:Swoo\_4849 | ABC transporter-like protein; K02003 putative ABC transport system ATP-binding protein |  |

  
**Neighborhood Representations for "shn:Shewana3\_0099"**  

| ID | Annotation | EC number |
| --- | --- | --- |
| shn:Shewana3\_0089 | 2,3-diketo-5-methylthio-1-phosphopentane phosphatase; K09880 enolase-phosphatase E1 [EC:3.1.3.77] | ec:3.1.3.77 |
| shn:Shewana3\_0090 | hypothetical protein |  |
| shn:Shewana3\_0091 | hypothetical protein |  |
| shn:Shewana3\_0092 | integrase catalytic subunit |  |
| shn:Shewana3\_0093 | transposase |  |
| shn:Shewana3\_0094 | hypothetical protein |  |
| shn:Shewana3\_0095 | hypothetical protein |  |
| shn:Shewana3\_0096 | MarR family transcriptional regulator |  |
| shn:Shewana3\_0097 | FAD-binding 9, siderophore-interacting domain-containing protein |  |
| shn:Shewana3\_0098 | imidazolonepropionase (EC:3.5.2.7); K01468 imidazolonepropionase [EC:3.5.2.7] | ec:3.5.2.7 |
| shn:Shewana3\_0099 | histidine utilization repressor; K05836 GntR family transcriptional regulator, histidine utilization repressor |  |
| shn:Shewana3\_0100 | urocanate hydratase (EC:4.2.1.49); K01712 urocanate hydratase [EC:4.2.1.49] | ec:4.2.1.49 |
| shn:Shewana3\_0101 | histidine ammonia-lyase (EC:4.3.1.3); K01745 histidine ammonia-lyase [EC:4.3.1.3] | ec:4.3.1.3 |
| shn:Shewana3\_0102 | pseudogene |  |
| shn:Shewana3\_0103 | formate dehydrogenase subunit beta (EC:1.2.1.2); K00124 formate dehydrogenase iron-sulfur subunit |  |
| shn:Shewana3\_0104 | formate dehydrogenase subunit gamma (EC:1.2.1.2); K00127 formate dehydrogenase subunit gamma |  |
| shn:Shewana3\_0105 | formate dehydrogenase accessory protein FdhE; K02380 FdhE protein |  |
| shn:Shewana3\_0106 | selenocysteine synthase (EC:2.9.1.1); K01042 L-seryl-tRNA(Ser) seleniumtransferase [EC:2.9.1.1] | ec:2.9.1.1 |
| shn:Shewana3\_0107 | selenocysteine-specific translation elongation factor SelB; K03833 selenocysteine-specific elongation factor |  |
| shn:Shewana3\_0108 | formate dehydrogenase accessory protein; K02379 FdhD protein |  |
| shn:Shewana3\_0109 | putative inner membrane protein; K07112 |  |

  
**Neighborhood Representations for "she:Shewmr4\_0098"**  

| ID | Annotation | EC number |
| --- | --- | --- |
| she:Shewmr4\_0088 | MerR family transcriptional regulator |  |
| she:Shewmr4\_0089 | carboxymuconolactone decarboxylase; K01607 4-carboxymuconolactone decarboxylase [EC:4.1.1.44] | ec:4.1.1.44 |
| she:Shewmr4\_0090 | 2,3-diketo-5-methylthio-1-phosphopentane phosphatase; K09880 enolase-phosphatase E1 [EC:3.1.3.77] | ec:3.1.3.77 |
| she:Shewmr4\_0091 | hypothetical protein |  |
| she:Shewmr4\_0092 | hypothetical protein |  |
| she:Shewmr4\_0093 | hypothetical protein |  |
| she:Shewmr4\_0094 | hypothetical protein |  |
| she:Shewmr4\_0095 | MarR family transcriptional regulator |  |
| she:Shewmr4\_0096 | FAD-binding 9, siderophore-interacting domain-containing protein |  |
| she:Shewmr4\_0097 | imidazolonepropionase (EC:3.5.2.7); K01468 imidazolonepropionase [EC:3.5.2.7] | ec:3.5.2.7 |
| she:Shewmr4\_0098 | histidine utilization repressor; K05836 GntR family transcriptional regulator, histidine utilization repressor |  |
| she:Shewmr4\_0099 | urocanate hydratase (EC:4.2.1.49); K01712 urocanate hydratase [EC:4.2.1.49] | ec:4.2.1.49 |
| she:Shewmr4\_0100 | histidine ammonia-lyase (EC:4.3.1.3); K01745 histidine ammonia-lyase [EC:4.3.1.3] | ec:4.3.1.3 |
| she:Shewmr4\_0101 | twin-arginine translocation pathway signal (EC:1.2.1.2); K00123 formate dehydrogenase major subunit [EC:1.2.1.2] | ec:1.2.1.2 |
| she:Shewmr4\_0102 | formate dehydrogenase subunit alpha (EC:1.2.1.2); K00123 formate dehydrogenase major subunit [EC:1.2.1.2] | ec:1.2.1.2 |
| she:Shewmr4\_0103 | formate dehydrogenase subunit beta (EC:1.2.1.2); K00124 formate dehydrogenase iron-sulfur subunit |  |
| she:Shewmr4\_0104 | formate dehydrogenase subunit gamma (EC:1.2.1.2); K00127 formate dehydrogenase subunit gamma |  |
| she:Shewmr4\_0105 | formate dehydrogenase accessory protein FdhE; K02380 FdhE protein |  |
| she:Shewmr4\_0106 | selenocysteine synthase (EC:2.9.1.1); K01042 L-seryl-tRNA(Ser) seleniumtransferase [EC:2.9.1.1] | ec:2.9.1.1 |
| she:Shewmr4\_0107 | selenocysteine-specific translation elongation factor SelB; K03833 selenocysteine-specific elongation factor |  |
| she:Shewmr4\_0108 | formate dehydrogenase accessory protein; K02379 FdhD protein |  |

  
**Neighborhood Representations for "shm:Shewmr7\_0093"**  

| ID | Annotation | EC number |
| --- | --- | --- |
| shm:Shewmr7\_0083 | NAD(P)H dehydrogenase (quinone) |  |
| shm:Shewmr7\_0084 | glutathione-dependent formaldehyde-activating, GFA |  |
| shm:Shewmr7\_0085 | 2,3-diketo-5-methylthio-1-phosphopentane phosphatase; K09880 enolase-phosphatase E1 [EC:3.1.3.77] | ec:3.1.3.77 |
| shm:Shewmr7\_0086 | hypothetical protein |  |
| shm:Shewmr7\_0087 | hypothetical protein |  |
| shm:Shewmr7\_0088 | hypothetical protein |  |
| shm:Shewmr7\_0089 | hypothetical protein |  |
| shm:Shewmr7\_0090 | MarR family transcriptional regulator |  |
| shm:Shewmr7\_0091 | FAD-binding 9, siderophore-interacting domain-containing protein |  |
| shm:Shewmr7\_0092 | imidazolonepropionase (EC:3.5.2.7); K01468 imidazolonepropionase [EC:3.5.2.7] | ec:3.5.2.7 |
| shm:Shewmr7\_0093 | histidine utilization repressor; K05836 GntR family transcriptional regulator, histidine utilization repressor |  |
| shm:Shewmr7\_0094 | urocanate hydratase (EC:4.2.1.49); K01712 urocanate hydratase [EC:4.2.1.49] | ec:4.2.1.49 |
| shm:Shewmr7\_0095 | histidine ammonia-lyase (EC:4.3.1.3); K01745 histidine ammonia-lyase [EC:4.3.1.3] | ec:4.3.1.3 |
| shm:Shewmr7\_0096 | twin-arginine translocation pathway signal (EC:1.2.1.2); K00123 formate dehydrogenase major subunit [EC:1.2.1.2] | ec:1.2.1.2 |
| shm:Shewmr7\_0097 | formate dehydrogenase subunit alpha (EC:1.2.1.2); K00123 formate dehydrogenase major subunit [EC:1.2.1.2] | ec:1.2.1.2 |
| shm:Shewmr7\_0098 | formate dehydrogenase subunit beta (EC:1.2.1.2); K00124 formate dehydrogenase iron-sulfur subunit |  |
| shm:Shewmr7\_0099 | formate dehydrogenase subunit gamma (EC:1.2.1.2); K00127 formate dehydrogenase subunit gamma |  |
| shm:Shewmr7\_0100 | formate dehydrogenase accessory protein FdhE; K02380 FdhE protein |  |
| shm:Shewmr7\_0101 | selenocysteine synthase (EC:2.9.1.1); K01042 L-seryl-tRNA(Ser) seleniumtransferase [EC:2.9.1.1] | ec:2.9.1.1 |
| shm:Shewmr7\_0102 | selenocysteine-specific translation elongation factor SelB; K03833 selenocysteine-specific elongation factor |  |
| shm:Shewmr7\_0103 | formate dehydrogenase accessory protein; K02379 FdhD protein |  |

  
**Neighborhood Representations for "slo:Shew\_3758"**  

| ID | Annotation | EC number |
| --- | --- | --- |
| slo:Shew\_3748 | hypothetical protein |  |
| slo:Shew\_3749 | hypothetical protein |  |
| slo:Shew\_3750 | phospholipid/glycerol acyltransferase |  |
| slo:Shew\_3751 | lysine exporter protein LysE/YggA |  |
| slo:Shew\_3752 | mechanosensitive ion channel protein MscS; K16053 miniconductance mechanosensitive channel |  |
| slo:Shew\_3753 | hypothetical protein |  |
| slo:Shew\_3754 | hypothetical protein |  |
| slo:Shew\_3755 | thioesterase superfamily protein |  |
| slo:Shew\_3756 | histidine ammonia-lyase (EC:4.3.1.3); K01745 histidine ammonia-lyase [EC:4.3.1.3] | ec:4.3.1.3 |
| slo:Shew\_3757 | urocanate hydratase (EC:4.2.1.49); K01712 urocanate hydratase [EC:4.2.1.49] | ec:4.2.1.49 |
| slo:Shew\_3758 | histidine utilization repressor; K05836 GntR family transcriptional regulator, histidine utilization repressor |  |
| slo:Shew\_3759 | imidazolonepropionase (EC:3.5.2.7); K01468 imidazolonepropionase [EC:3.5.2.7] | ec:3.5.2.7 |
| slo:Shew\_3760 | KAP P-loop domain-containing protein |  |
| slo:Shew\_3761 | hypothetical protein |  |
| slo:Shew\_3762 | hypothetical protein |  |
| slo:Shew\_3763 | LuxR family transcriptional regulator |  |
| slo:Shew\_3764 | hypothetical protein |  |
| slo:Shew\_3765 | hypothetical protein |  |
| slo:Shew\_3766 | CzcA family heavy metal efflux protein; K15726 cobalt-zinc-cadmium resistance protein CzcA |  |
| slo:Shew\_3767 | hypothetical protein |  |
| slo:Shew\_3768 | hypothetical protein |  |

  
**Neighborhood Representations for "svo:SVI\_4276"**  

| ID | Annotation | EC number |
| --- | --- | --- |
| svo:SVI\_4266 | hypothetical protein |  |
| svo:SVI\_4267 | hypothetical protein |  |
| svo:SVI\_4268 | acyltransferase family protein |  |
| svo:SVI\_4269 | LysE family transporter |  |
| svo:SVI\_4270 | small-conductance mechanosensitive channel; K16053 miniconductance mechanosensitive channel |  |
| svo:SVI\_4271 | hypothetical protein |  |
| svo:SVI\_4272 | hypothetical protein |  |
| svo:SVI\_4273 | hypothetical protein |  |
| svo:SVI\_4274 | hutH; histidine ammonia-lyase; K01745 histidine ammonia-lyase [EC:4.3.1.3] | ec:4.3.1.3 |
| svo:SVI\_4275 | hutU; urocanate hydratase; K01712 urocanate hydratase [EC:4.2.1.49] | ec:4.2.1.49 |
| svo:SVI\_4276 | hutC; histidine utilization repressor; K05836 GntR family transcriptional regulator, histidine utilization repressor |  |
| svo:SVI\_4277 | hutI; imidazolonepropionase; K01468 imidazolonepropionase [EC:3.5.2.7] | ec:3.5.2.7 |
| svo:SVI\_4278 | hypothetical protein |  |
| svo:SVI\_4279 | hypothetical protein |  |
| svo:SVI\_4280 | hypothetical protein |  |
| svo:SVI\_4281 | AraC family transcriptional regulator |  |
| svo:SVI\_4282 | outer membrane iron(III) dicitrate receptor; K16091 Fe(3+) dicitrate transport protein |  |
| svo:SVI\_4283 | hypothetical protein |  |
| svo:SVI\_4284 | hypothetical protein; K07017 |  |
| svo:SVI\_4285 | HAD-superfamily hydrolase; K09880 enolase-phosphatase E1 [EC:3.1.3.77] | ec:3.1.3.77 |
| svo:SVI\_4286 | thioesterase superfamily; K07107 acyl-CoA thioester hydrolase [EC:3.1.2.-] |  |

  
**Neighborhood Representations for "shl:Shal\_0072"**  

| ID | Annotation | EC number |
| --- | --- | --- |
| shl:Shal\_0062 | TAP domain-containing protein |  |
| shl:Shal\_0063 | AMP-dependent synthetase and ligase; K01897 long-chain acyl-CoA synthetase [EC:6.2.1.3] | ec:6.2.1.3 |
| shl:Shal\_0064 | thioesterase superfamily protein; K07107 acyl-CoA thioester hydrolase [EC:3.1.2.-] |  |
| shl:Shal\_0065 | hypothetical protein; K06975 |  |
| shl:Shal\_0066 | thioesterase superfamily protein; K07107 acyl-CoA thioester hydrolase [EC:3.1.2.-] |  |
| shl:Shal\_0067 | 2,3-diketo-5-methylthio-1-phosphopentane phosphatase; K09880 enolase-phosphatase E1 [EC:3.1.3.77] | ec:3.1.3.77 |
| shl:Shal\_0068 | lytic transglycosylase; K08306 membrane-bound lytic murein transglycosylase C [EC:3.2.1.-] |  |
| shl:Shal\_0069 | ATPase AAA |  |
| shl:Shal\_0070 | hypothetical protein |  |
| shl:Shal\_0071 | imidazolonepropionase; K01468 imidazolonepropionase [EC:3.5.2.7] | ec:3.5.2.7 |
| shl:Shal\_0072 | histidine utilization repressor; K05836 GntR family transcriptional regulator, histidine utilization repressor |  |
| shl:Shal\_0073 | urocanate hydratase; K01712 urocanate hydratase [EC:4.2.1.49] | ec:4.2.1.49 |
| shl:Shal\_0074 | histidine ammonia-lyase; K01745 histidine ammonia-lyase [EC:4.3.1.3] | ec:4.3.1.3 |
| shl:Shal\_0075 | N-acetyltransferase GCN5 |  |
| shl:Shal\_0076 | LysR family transcriptional regulator |  |
| shl:Shal\_0077 | putative endoribonuclease L-PSP; K09022 UPF0076 protein YjgF |  |
| shl:Shal\_0078 | Na+/H+ antiporter NhaC |  |
| shl:Shal\_0079 | 1-aminocyclopropane-1-carboxylate deaminase; K05396 D-cysteine desulfhydrase [EC:4.4.1.15] | ec:4.4.1.15 |
| shl:Shal\_0080 | hypothetical protein |  |
| shl:Shal\_0081 | hypothetical protein |  |
| shl:Shal\_0082 | hypothetical protein |  |

  
**Neighborhood Representations for "xbo:XBJ1\_2986"**  

| ID | Annotation | EC number |
| --- | --- | --- |
| xbo:XBJ1\_2976 | peptidase; K15461 tRNA 5-methylaminomethyl-2-thiouridine biosynthesis bifunctional protein [EC:2.1.1.61 1.5.-.-] | ec:2.1.1.61 |
| xbo:XBJ1\_2977 | N-acetyltransferase GCN5 |  |
| xbo:XBJ1\_2978 | hypothetical protein |  |
| xbo:XBJ1\_2979 | transporting ATPase; K09906 hypothetical protein |  |
| xbo:XBJ1\_2980 | yfcA; hypothetical protein; K07090 |  |
| xbo:XBJ1\_2981 | aroC; chorismate synthase (EC:4.2.3.5); K01736 chorismate synthase [EC:4.2.3.5] | ec:4.2.3.5 |
| xbo:XBJ1\_2982 | prmB; N5-glutamine methyltransferase, modifies ribosomal protein L3 (EC:1.3.3.-); K07320 putative adenine-specific DNA-methyltransferase [EC:2.1.1.72] | ec:2.1.1.72 |
| xbo:XBJ1\_2983 | proY; proline transporter; K11736 proline-specific permease ProY |  |
| xbo:XBJ1\_2984 | histidine ammonia-lyase (EC:4.3.1.3); K01745 histidine ammonia-lyase [EC:4.3.1.3] | ec:4.3.1.3 |
| xbo:XBJ1\_2985 | urocanate hydratase (EC:4.2.1.49); K01712 urocanate hydratase [EC:4.2.1.49] | ec:4.2.1.49 |
| xbo:XBJ1\_2986 | histidine utilization repressor; K05836 GntR family transcriptional regulator, histidine utilization repressor |  |
| xbo:XBJ1\_2987 | Formimidoylglutamase (EC:3.5.3.8); K01479 formiminoglutamase [EC:3.5.3.8] | ec:3.5.3.8 |
| xbo:XBJ1\_2988 | imidazolonepropionase (EC:3.5.2.7); K01468 imidazolonepropionase [EC:3.5.2.7] | ec:3.5.2.7 |
| xbo:XBJ1\_2989 | yfcN; phage-like protein |  |
| xbo:XBJ1\_2990 | sixA; phosphohistidine phosphatase (EC:3.1.3.-); K08296 phosphohistidine phosphatase [EC:3.1.3.-] |  |
| xbo:XBJ1\_2991 | hypothetical protein |  |
| xbo:XBJ1\_2992 | fadJ; bifunctional anaerobic fatty acid oxidation complex protein: enoyl-CoA hydratase/epimerase/isomerase (N-terminal); 3-hydroxyacyl-CoA dehydrogenase (C-terminal) (EC:1.1.1.35 4.2.1.17 5.3.3.8); K01782 3-hydroxyacyl-CoA dehydrogenase / enoyl-CoA hydratase / 3-hydroxybutyryl-CoA epimerase [EC:1.1.1.35 4.2.1.17 5.1.2.3] | ec:5.1.2.3 ec:4.2.1.17 ec:1.1.1.35 |
| xbo:XBJ1\_2993 | fadI; beta-keto thiolase (EC:2.3.1.16); K00632 acetyl-CoA acyltransferase [EC:2.3.1.16] | ec:2.3.1.16 |
| xbo:XBJ1\_2994 | hypothetical protein |  |
| xbo:XBJ1\_2995 | fadL; porin (EC:3.1.1.35); K06076 long-chain fatty acid transport protein |  |
| xbo:XBJ1\_2996 | vacJ; lipoprotein; K04754 lipoprotein |  |

  
**Neighborhood Representations for "son:SO\_0096"**  

| ID | Annotation | EC number |
| --- | --- | --- |
| son:SO\_0083 | carboxymuconolactone decarboxylase family protein; K01607 4-carboxymuconolactone decarboxylase [EC:4.1.1.44] | ec:4.1.1.44 |
| son:SO\_0084 | mtnC; 2,3-diketo-5-methylthio-1-phosphopentane phosphatase MtnC (EC:3.1.3.77); K09880 enolase-phosphatase E1 [EC:3.1.3.77] | ec:3.1.3.77 |
| son:SO\_0085 | predicted inner membrane protein |  |
| son:SO\_0086 | predicted membrane protein |  |
| son:SO\_0088 | predicted periplasmic protein |  |
| son:SO\_0090 | periplasmic protein of unknown function DUF442 |  |
| son:SO\_0091 | cyclic nucleotide binding domain protein |  |
| son:SO\_0092 | deoD; purine nucleoside phosphorylase DeoD (EC:2.4.2.1); K03784 purine-nucleoside phosphorylase [EC:2.4.2.1] | ec:2.4.2.1 |
| son:SO\_0093 | Na+ dependent nucleoside transporter NupC family; K03317 concentrative nucleoside transporter, CNT family |  |
| son:SO\_0095 | hutI; imidazolonepropionase HutI (EC:3.5.2.7); K01468 imidazolonepropionase [EC:3.5.2.7] | ec:3.5.2.7 |
| son:SO\_0096 | hutC; transcriptional repressor of histidine utilization genes HutC; K05836 GntR family transcriptional regulator, histidine utilization repressor |  |
| son:SO\_0097 | hutU; urocanate hydratase HutU (EC:4.2.1.49); K01712 urocanate hydratase [EC:4.2.1.49] | ec:4.2.1.49 |
| son:SO\_0098 | hutH; histidine ammonia-lyase HutH (EC:4.3.1.3); K01745 histidine ammonia-lyase [EC:4.3.1.3] | ec:4.3.1.3 |
| son:SO\_0101 | fdnG; nitrate-inducible formate dehydrogenase molybdopterin-binding subunit FdnG (EC:1.1.5.6); K00123 formate dehydrogenase major subunit [EC:1.2.1.2] | ec:1.2.1.2 |
| son:SO\_0102 | fdnH; nitrate-inducible formate dehydrogenase iron-sulfur subunit FdnH (EC:1.1.5.6); K00124 formate dehydrogenase iron-sulfur subunit |  |
| son:SO\_0103 | fdnI; nitrate-inducible formate dehydrogenase cytochrome b subunit FdnI (EC:1.1.5.6); K00127 formate dehydrogenase subunit gamma |  |
| son:SO\_0104 | fdhE; nitrate-inducible formate dehydrogenase chaperone FdhE; K02380 FdhE protein |  |
| son:SO\_0105 | selA; L-seryl-tRNA selenium transferase SelA (EC:2.9.1.1); K01042 L-seryl-tRNA(Ser) seleniumtransferase [EC:2.9.1.1] | ec:2.9.1.1 |
| son:SO\_0106 | selB; selenocysteine-specific translation elongation factor SelB; K03833 selenocysteine-specific elongation factor |  |
| son:SO\_t002 | tRNA-Sec; K14238 tRNA Sec |  |
| son:SO\_0107 | fdhD; formate dehydrogenase assembly/maturation protein FdhD; K02379 FdhD protein |  |

  
**Neighborhood Representations for "vex:VEA\_003719"**  

| ID | Annotation | EC number |
| --- | --- | --- |
| vex:VEA\_003709 | exporter of the RND superfamily; K07003 |  |
| vex:VEA\_003710 | transcriptional regulator |  |
| vex:VEA\_003711 | hypothetical protein |  |
| vex:VEA\_003712 | hypothetical protein |  |
| vex:VEA\_003713 | 50S ribosomal protein L20; K02887 large subunit ribosomal protein L20 |  |
| vex:VEA\_003714 | 50S ribosomal protein L35; K02916 large subunit ribosomal protein L35 |  |
| vex:VEA\_003715 | translation initiation factor 3; K02520 translation initiation factor IF-3 |  |
| vex:VEA\_003716 | threonyl-tRNA synthetase (EC:6.1.1.3); K01868 threonyl-tRNA synthetase [EC:6.1.1.3] | ec:6.1.1.3 |
| vex:VEA\_003717 | hypothetical protein |  |
| vex:VEA\_003718 | SpoOM-like protein; K06377 sporulation-control protein |  |
| vex:VEA\_003719 | histidine utilization repressor; K05836 GntR family transcriptional regulator, histidine utilization repressor |  |
| vex:VEA\_003720 | imidazolonepropionase (EC:3.5.2.7); K01468 imidazolonepropionase [EC:3.5.2.7] | ec:3.5.2.7 |
| vex:VEA\_003721 | formiminoglutamase (EC:3.5.3.8); K01479 formiminoglutamase [EC:3.5.3.8] | ec:3.5.3.8 |
| vex:VEA\_003722 | urocanate hydratase (EC:4.2.1.49); K01712 urocanate hydratase [EC:4.2.1.49] | ec:4.2.1.49 |
| vex:VEA\_003723 | histidine ammonia-lyase (EC:4.3.1.3); K01745 histidine ammonia-lyase [EC:4.3.1.3] | ec:4.3.1.3 |
| vex:VEA\_003724 | hypothetical protein |  |
| vex:VEA\_003725 | protein-S-isoprenylcysteine methyltransferase |  |
| vex:VEA\_003726 | Fe-S oxidoreductase; K06911 |  |
| vex:VEA\_003727 | hypothetical protein |  |
| vex:VEA\_003728 | hypothetical protein |  |
| vex:VEA\_003729 | hypothetical protein |  |

  
**Neighborhood Representations for "vsa:VSAL\_II0704"**  

| ID | Annotation | EC number |
| --- | --- | --- |
| vsa:VSAL\_II0694 | lolC; lipoprotein releasing system, transmembrane protein; K09808 lipoprotein-releasing system permease protein |  |
| vsa:VSAL\_II0695 | hypothetical protein |  |
| vsa:VSAL\_II0696 | mfd; transcription-repair coupling factor (EC:3.6.1.-); K03723 transcription-repair coupling factor (superfamily II helicase) [EC:3.6.4.-] |  |
| vsa:VSAL\_II0697 | hypothetical protein |  |
| vsa:VSAL\_II0698 | pseudogene |  |
| vsa:VSAL\_II0699 | transposase |  |
| vsa:VSAL\_II0700 | pseudogene |  |
| vsa:VSAL\_II0701 | putative lipoprotein |  |
| vsa:VSAL\_II0702 | fkpA; FKBP-type peptidyl-prolyl cis-trans isomerase FkpA (EC:5.2.1.8); K01802 peptidylprolyl isomerase [EC:5.2.1.8] | ec:5.2.1.8 |
| vsa:VSAL\_II0703 | hypothetical protein |  |
| vsa:VSAL\_II0704 | hutC; histidine utilization repressor; K05836 GntR family transcriptional regulator, histidine utilization repressor |  |
| vsa:VSAL\_II0705 | hutI; imidazolonepropionase (EC:3.5.2.7); K01468 imidazolonepropionase [EC:3.5.2.7] | ec:3.5.2.7 |
| vsa:VSAL\_II0706 | formimidoylglutamase; K01479 formiminoglutamase [EC:3.5.3.8] | ec:3.5.3.8 |
| vsa:VSAL\_II0707 | hutU; urocanate hydratase (EC:4.2.1.49); K01712 urocanate hydratase [EC:4.2.1.49] | ec:4.2.1.49 |
| vsa:VSAL\_II0708 | hutH; histidine ammonia-lyase (EC:4.3.1.3); K01745 histidine ammonia-lyase [EC:4.3.1.3] | ec:4.3.1.3 |
| vsa:VSAL\_II0709 | vcmN; pseudogene |  |
| vsa:VSAL\_II0710 | hypothetical protein |  |
| vsa:VSAL\_II0711 | hypothetical protein |  |
| vsa:VSAL\_II0712 | methyl-accepting chemotaxis citrate transducer; K03406 methyl-accepting chemotaxis protein |  |
| vsa:VSAL\_II0713 | hypothetical protein |  |
| vsa:VSAL\_II0714 | cusA; cation efflux system protein; K07787 Cu(I)/Ag(I) efflux system membrane protein CusA/SilA |  |

  
**Neighborhood Representations for "sdn:Sden\_0082"**  

| ID | Annotation | EC number |
| --- | --- | --- |
| sdn:Sden\_0072 | AMP-dependent synthetase and ligase; K01897 long-chain acyl-CoA synthetase [EC:6.2.1.3] | ec:6.2.1.3 |
| sdn:Sden\_0073 | thioesterase superfamily protein; K07107 acyl-CoA thioester hydrolase [EC:3.1.2.-] |  |
| sdn:Sden\_0074 | thioesterase superfamily protein; K07107 acyl-CoA thioester hydrolase [EC:3.1.2.-] |  |
| sdn:Sden\_0075 | hypothetical protein |  |
| sdn:Sden\_0076 | GGDEF domain-containing protein |  |
| sdn:Sden\_0077 | 2,3-diketo-5-methylthio-1-phosphopentane phosphatase; K09880 enolase-phosphatase E1 [EC:3.1.3.77] | ec:3.1.3.77 |
| sdn:Sden\_0078 | CMP/dCMP deaminase, zinc-binding; K01493 dCMP deaminase [EC:3.5.4.12] | ec:3.5.4.12 |
| sdn:Sden\_0079 | hypothetical protein; K07017 |  |
| sdn:Sden\_0080 | hypothetical protein |  |
| sdn:Sden\_0081 | imidazolonepropionase (EC:3.5.2.7); K01468 imidazolonepropionase [EC:3.5.2.7] | ec:3.5.2.7 |
| sdn:Sden\_0082 | histidine utilization repressor; K05836 GntR family transcriptional regulator, histidine utilization repressor |  |
| sdn:Sden\_0083 | urocanate hydratase (EC:4.2.1.49); K01712 urocanate hydratase [EC:4.2.1.49] | ec:4.2.1.49 |
| sdn:Sden\_0084 | histidine ammonia-lyase (EC:4.3.1.3); K01745 histidine ammonia-lyase [EC:4.3.1.3] | ec:4.3.1.3 |
| sdn:Sden\_0085 | hypothetical protein |  |
| sdn:Sden\_0086 | MscS mechanosensitive ion channel; K16053 miniconductance mechanosensitive channel |  |
| sdn:Sden\_0087 | glyoxalase/bleomycin resistance protein/dioxygenase |  |
| sdn:Sden\_0088 | phospholipid/glycerol acyltransferase |  |
| sdn:Sden\_0089 | hypothetical protein |  |
| sdn:Sden\_0090 | hypothetical protein |  |
| sdn:Sden\_0091 | hypothetical protein |  |
| sdn:Sden\_0092 | multiple antibiotic resistance (MarC)-related proteins; K05595 multiple antibiotic resistance protein |  |

  
**Neighborhood Representations for "sse:Ssed\_4448"**  

| ID | Annotation | EC number |
| --- | --- | --- |
| sse:Ssed\_4438 | hypothetical protein |  |
| sse:Ssed\_4439 | hypothetical protein |  |
| sse:Ssed\_4440 | phospholipid/glycerol acyltransferase |  |
| sse:Ssed\_4441 | lysine exporter protein (LysE/YggA) |  |
| sse:Ssed\_4442 | mechanosensitive ion channel protein MscS; K16053 miniconductance mechanosensitive channel |  |
| sse:Ssed\_4443 | hypothetical protein |  |
| sse:Ssed\_4444 | hypothetical protein |  |
| sse:Ssed\_4445 | thioesterase superfamily protein |  |
| sse:Ssed\_4446 | histidine ammonia-lyase; K01745 histidine ammonia-lyase [EC:4.3.1.3] | ec:4.3.1.3 |
| sse:Ssed\_4447 | urocanate hydratase; K01712 urocanate hydratase [EC:4.2.1.49] | ec:4.2.1.49 |
| sse:Ssed\_4448 | histidine utilization repressor; K05836 GntR family transcriptional regulator, histidine utilization repressor |  |
| sse:Ssed\_4449 | imidazolonepropionase; K01468 imidazolonepropionase [EC:3.5.2.7] | ec:3.5.2.7 |
| sse:Ssed\_4450 | hypothetical protein |  |
| sse:Ssed\_4451 | outer membrane protein |  |
| sse:Ssed\_4452 | hypothetical protein; K07017 |  |
| sse:Ssed\_4453 | Sel1 domain-containing protein |  |
| sse:Ssed\_4454 | 2,3-diketo-5-methylthio-1-phosphopentane phosphatase; K09880 enolase-phosphatase E1 [EC:3.1.3.77] | ec:3.1.3.77 |
| sse:Ssed\_4455 | hypothetical protein |  |
| sse:Ssed\_4456 | thioesterase superfamily protein; K07107 acyl-CoA thioester hydrolase [EC:3.1.2.-] |  |
| sse:Ssed\_4457 | hypothetical protein; K06975 |  |
| sse:Ssed\_4458 | thioesterase superfamily protein; K07107 acyl-CoA thioester hydrolase [EC:3.1.2.-] |  |

  
**Neighborhood Representations for "swp:swp\_0132"**  

| ID | Annotation | EC number |
| --- | --- | --- |
| swp:swp\_0122 | hypothetical protein |  |
| swp:swp\_0123 | HAD-superfamily hydrolase; K09880 enolase-phosphatase E1 [EC:3.1.3.77] | ec:3.1.3.77 |
| swp:swp\_0124 | hypothetical protein |  |
| swp:swp\_0125 | hypothetical protein |  |
| swp:swp\_0126 | hypothetical protein |  |
| swp:swp\_0127 | RND family efflux transporter MFP subunit |  |
| swp:swp\_0128 | ATPase; K02003 putative ABC transport system ATP-binding protein |  |
| swp:swp\_0129 | hypothetical protein |  |
| swp:swp\_0130 | hypothetical protein |  |
| swp:swp\_0131 | imidazolonepropionase (EC:3.5.2.7); K01468 imidazolonepropionase [EC:3.5.2.7] | ec:3.5.2.7 |
| swp:swp\_0132 | histidine utilization repressor; K05836 GntR family transcriptional regulator, histidine utilization repressor |  |
| swp:swp\_0133 | urocanate hydratase (EC:4.2.1.49); K01712 urocanate hydratase [EC:4.2.1.49] | ec:4.2.1.49 |
| swp:swp\_0134 | histidine ammonia-lyase (EC:4.3.1.3); K01745 histidine ammonia-lyase [EC:4.3.1.3] | ec:4.3.1.3 |
| swp:swp\_0135 | dipeptidylaminopeptidase/acylaminoacyl-peptidase |  |
| swp:swp\_0136 | histidine ammonia-lyase (EC:4.3.1.3) |  |
| swp:swp\_0137 | thioesterase superfamily protein |  |
| swp:swp\_0138 | regulatory protein MerR |  |
| swp:swp\_0139 | hypothetical protein |  |
| swp:swp\_0140 | hypothetical protein |  |
| swp:swp\_0141 | hypothetical protein |  |
| swp:swp\_0142 | hypothetical protein |  |

  
**Neighborhood Representations for "hch:HCH\_04168"**  

| ID | Annotation | EC number |
| --- | --- | --- |
| hch:HCH\_04156 | hypothetical protein |  |
| hch:HCH\_04157 | hypothetical protein |  |
| hch:HCH\_04158 | hypothetical protein |  |
| hch:HCH\_04159 | hypothetical protein |  |
| hch:HCH\_04160 | hypothetical protein |  |
| hch:HCH\_04161 | amino acid ABC transporter periplasmic protein |  |
| hch:HCH\_04162 | lhr; Lhr-like helicase (EC:3.6.1.-); K03724 ATP-dependent helicase Lhr and Lhr-like helicase [EC:3.6.4.-] |  |
| hch:HCH\_04165 | hypothetical protein |  |
| hch:HCH\_04166 | hypothetical protein |  |
| hch:HCH\_04167 | D-alanine export protein |  |
| hch:HCH\_04168 | hutC; histidine utilization repressor; K05836 GntR family transcriptional regulator, histidine utilization repressor |  |
| hch:HCH\_04169 | hutI; imidazolonepropionase (EC:3.5.2.7); K01468 imidazolonepropionase [EC:3.5.2.7] | ec:3.5.2.7 |
| hch:HCH\_04170 | hutG; formimidoylglutamase (EC:3.5.3.8); K01479 formiminoglutamase [EC:3.5.3.8] | ec:3.5.3.8 |
| hch:HCH\_04171 | hutU; urocanate hydratase (EC:4.2.1.49); K01712 urocanate hydratase [EC:4.2.1.49] | ec:4.2.1.49 |
| hch:HCH\_04172 | hutH; histidine ammonia-lyase (EC:4.3.1.3); K01745 histidine ammonia-lyase [EC:4.3.1.3] | ec:4.3.1.3 |
| hch:HCH\_04173 | amino acid ABC transporter periplasmic protein; K02030 polar amino acid transport system substrate-binding protein |  |
| hch:HCH\_04174 | amino acid ABC transporter periplasmic protein |  |
| hch:HCH\_04175 | hypothetical protein |  |
| hch:HCH\_04176 | Zn-dependent hydrolase |  |
| hch:HCH\_04177 | transcriptional regulator |  |
| hch:HCH\_04178 | hypothetical protein |  |

  
**Neighborhood Representations for "vfm:VFMJ11\_A0504"**  

| ID | Annotation | EC number |
| --- | --- | --- |
| vfm:VFMJ11\_A0494 | heavy metal efflux pump, CzcA family; K07787 Cu(I)/Ag(I) efflux system membrane protein CusA/SilA |  |
| vfm:VFMJ11\_A0495 | copper-binding protein |  |
| vfm:VFMJ11\_A0496 | methyl-accepting chemotaxis protein |  |
| vfm:VFMJ11\_A0497 | methyl-accepting chemotaxis protein; K03406 methyl-accepting chemotaxis protein |  |
| vfm:VFMJ11\_A0498 | methyl-accepting chemotaxis protein; K03406 methyl-accepting chemotaxis protein |  |
| vfm:VFMJ11\_A0499 | hypothetical protein |  |
| vfm:VFMJ11\_A0500 | hutH; histidine ammonia-lyase (EC:4.3.1.3); K01745 histidine ammonia-lyase [EC:4.3.1.3] | ec:4.3.1.3 |
| vfm:VFMJ11\_A0501 | hutU; urocanate hydratase (EC:4.2.1.49); K01712 urocanate hydratase [EC:4.2.1.49] | ec:4.2.1.49 |
| vfm:VFMJ11\_A0502 | hutG; formimidoylglutamase (EC:3.5.3.8); K01479 formiminoglutamase [EC:3.5.3.8] | ec:3.5.3.8 |
| vfm:VFMJ11\_A0503 | hutI; imidazolonepropionase (EC:3.5.2.7); K01468 imidazolonepropionase [EC:3.5.2.7] | ec:3.5.2.7 |
| vfm:VFMJ11\_A0504 | hutC; histidine utilization repressor; K05836 GntR family transcriptional regulator, histidine utilization repressor |  |
| vfm:VFMJ11\_A0505 | Methyltransferase domain family protein |  |
| vfm:VFMJ11\_A0506 | fkbp-type peptidyl-prolyl cis-trans isomerase fkpa (EC:5.2.1.8); K01802 peptidylprolyl isomerase [EC:5.2.1.8] | ec:5.2.1.8 |
| vfm:VFMJ11\_A0507 | thiol-disulfide isomerase |  |
| vfm:VFMJ11\_A0508 | hypothetical protein |  |
| vfm:VFMJ11\_A0509 | mfd; transcription-repair coupling factor (EC:3.6.1.-); K03723 transcription-repair coupling factor (superfamily II helicase) [EC:3.6.4.-] |  |
| vfm:VFMJ11\_A0510 | hypothetical protein |  |
| vfm:VFMJ11\_A0511 | lipoprotein releasing system transmembrane protein LolE; K09808 lipoprotein-releasing system permease protein |  |
| vfm:VFMJ11\_A0512 | lolD; lipoprotein releasing system, ATP-binding protein; K09810 lipoprotein-releasing system ATP-binding protein [EC:3.6.3.-] |  |
| vfm:VFMJ11\_A0513 | lolE; outer membrane-specific lipoprotein transporter subunit LolE; K09808 lipoprotein-releasing system permease protein |  |
| vfm:VFMJ11\_A0514 | hypothetical protein |  |

  
**Over-represented Enzyme Summary**: Table of E.C. identified protein in the "Neighborhood Representation" ranked by frequency of occurrence  

| EC number | Frequency | Annotation | Reactions |
| --- | --- | --- | --- |
| ec:4.3.1.3 | 80 | histidine ammonia-lyase; histidase; histidinase; histidine alpha-deaminase; L-histidine ammonia-lyase | L-histidine = urocanate + NH3 [RN:R01168] |
| ec:3.5.2.7 | 78 | imidazolonepropionase; 4(5)-imidazolone-5(4)-propionic acid hydrolase; imidazolone propionic acid hydrolase | (S)-3-(5-oxo-4,5-dihydro-3H-imidazol-4-yl)propanoate + H2O = N-formimidoyl-L-glutamate + H+ [RN:R02288] |
| ec:4.2.1.49 | 75 | urocanate hydratase; urocanase; 3-(5-oxo-4,5-dihydro-3H-imidazol-4-yl)propanoate hydro-lyase | 3-(5-oxo-4,5-dihydro-3H-imidazol-4-yl)propanoate = urocanate + H2O [RN:R02914] |
| ec:3.5.3.13 | 49 | formimidoylglutamate deiminase; formiminoglutamate deiminase; formiminoglutamic iminohydrolase | N-formimidoyl-L-glutamate + H2O = N-formyl-L-glutamate + NH3 [RN:R02286] |
| ec:3.5.3.8 | 47 | formimidoylglutamase; formiminoglutamase; N-formiminoglutamate hydrolase; N-formimino-L-glutamate formiminohydrolase | N-formimidoyl-L-glutamate + H2O = L-glutamate + formamide [RN:R02285] |
| ec:3.5.1.68 | 17 | N-formylglutamate deformylase; beta-citryl-L-glutamate hydrolase; formylglutamate deformylase; N-formylglutamate hydrolase; beta-citrylglutamate amidase; beta-citryl-L-glutamate amidohydrolase; beta-citryl-L-glutamate amidase; beta-citryl-L-glutamate-hydrolyzing enzyme | N-formyl-L-glutamate + H2O = formate + L-glutamate [RN:R00525] |
| ec:2.3.1.180 | 14 | beta-ketoacyl-[acyl-carrier-protein] synthase III; 3-oxoacyl:ACP synthase III; 3-ketoacyl-acyl carrier protein synthase III; KASIII; KAS III; FabH; beta-ketoacyl-acyl carrier protein synthase III; beta-ketoacyl-ACP synthase III; beta-ketoacyl (acyl carrier protein) synthase III; acetyl-CoA:malonyl-[acyl-carrier-protein] C-acyltransferase | acetyl-CoA + a malonyl-[acyl-carrier protein] = an acetoacetyl-[acyl-carrier protein] + CoA + CO2 |
| ec:3.5.3.23 | 10 | N-succinylarginine dihydrolase; N2-succinylarginine dihydrolase; arginine succinylhydrolase; SADH; AruB; AstB; 2-N-succinyl-L-arginine iminohydrolase (decarboxylating) | N2-succinyl-L-arginine + 2 H2O = N2-succinyl-L-ornithine + 2 NH3 + CO2 [RN:R04189] |
| ec:2.3.1.109 | 10 | arginine N-succinyltransferase; arginine succinyltransferase; AstA; arginine and ornithine N2-succinyltransferase; AOST; AST; succinyl-CoA:L-arginine 2-N-succinyltransferase | succinyl-CoA + L-arginine = CoA + N2-succinyl-L-arginine [RN:R00832] |
| ec:3.1.3.77 | 10 | acireductone synthase; E1; E-1 enolase-phosphatase | 5-(methylthio)-2,3-dioxopentyl phosphate + H2O = 1,2-dihydroxy-5-(methylthio)pent-1-en-3-one + phosphate (overall reaction) [RN:R07395]; (1a) 5-(methylthio)-2,3-dioxopentyl phosphate = 2-hydroxy-5-(methylthio)-3-oxopent-1-enyl phosphate (probably spontaneous) [RN:R07393]; (1b) 2-hydroxy-5-(methylthio)-3-oxopent-1-enyl phosphate + H2O = 1,2-dihydroxy-5-(methylthio)pent-1-en-3-one + phosphate [RN:R07394] |
| ec:1.2.1.71 | 10 | succinylglutamate-semialdehyde dehydrogenase; succinylglutamic semialdehyde dehydrogenase; N-succinylglutamate 5-semialdehyde dehydrogenase; SGSD; AruD; AstD | N-succinyl-L-glutamate 5-semialdehyde + NAD+ + H2O = N-succinyl-L-glutamate + NADH + 2 H+ [RN:R05049] |
| ec:3.5.1.96 | 10 | succinylglutamate desuccinylase; N2-succinylglutamate desuccinylase; SGDS; AstE | N-succinyl-L-glutamate + H2O = succinate + L-glutamate [RN:R00411] |
| ec:2.4.1.80 | 9 | ceramide glucosyltransferase; UDP-glucose:ceramide glucosyltransferase; ceramide:UDP-Glc glucosyltransferase; uridine diphosphoglucose-ceramide glucosyltransferase; ceramide:UDP-glucose glucosyltransferase; glucosylceramide synthase | UDP-glucose + an N-acylsphingosine = UDP + a D-glucosyl-N-acylsphingosine [RN:R01497 R06275] |
| ec:6.3.4.5 | 9 | argininosuccinate synthase; citrulline---aspartate ligase; argininosuccinate synthetase; arginine succinate synthetase; argininosuccinic acid synthetase; arginosuccinate synthetase | ATP + L-citrulline + L-aspartate = AMP + diphosphate + 2-(Nomega-L-arginino)succinate [RN:R01954] |
| ec:3.1.3.11 | 9 | fructose-bisphosphatase; hexose diphosphatase; FBPase; fructose 1,6-diphosphatase; fructose 1,6-diphosphate phosphatase; D-fructose 1,6-diphosphatase; fructose 1,6-bisphosphatase; fructose diphosphatase; fructose diphosphate phosphatase; fructose bisphosphate phosphatase; fructose 1,6-bisphosphate 1-phosphatase; fructose 1,6-bisphosphate phosphatase; hexose bisphosphatase; D-fructose-1,6-bisphosphate phosphatase | D-fructose 1,6-bisphosphate + H2O = D-fructose 6-phosphate + phosphate [RN:R00762] |
| ec:3.6.3.21 | 8 | polar-amino-acid-transporting ATPase; histidine permease | ATP + H2O + polar amino acidout = ADP + phosphate + polar amino acidin [RN:R00086] |
| ec:6.3.1.2 | 8 | glutamate---ammonia ligase; glutamine synthetase; glutamylhydroxamic synthetase; L-glutamine synthetase | ATP + L-glutamate + NH3 = ADP + phosphate + L-glutamine [RN:R00253] |
| ec:3.5.1.32 | 8 | hippurate hydrolase | hippurate + H2O = benzoate + glycine [RN:R01424] |
| ec:2.7.1.25 | 8 | adenylyl-sulfate kinase; adenylylsulfate kinase (phosphorylating); 5'-phosphoadenosine sulfate kinase; adenosine 5'-phosphosulfate kinase; adenosine phosphosulfate kinase; adenosine phosphosulfokinase; adenosine-5'-phosphosulfate-3'-phosphokinase; APS kinase | ATP + adenylyl sulfate = ADP + 3'-phosphoadenylyl sulfate [RN:R00509] |
| ec:2.6.1.81 | 7 | succinylornithine transaminase; succinylornithine aminotransferase; N2-succinylornithine 5-aminotransferase; AstC; SOAT; 2-N-succinyl-L-ornithine:2-oxoglutarate 5-aminotransferase | N2-succinyl-L-ornithine + 2-oxoglutarate = N-succinyl-L-glutamate 5-semialdehyde + L-glutamate [RN:R04217] |
| ec:4.2.1.17 | 6 | enoyl-CoA hydratase; enoyl hydrase; unsaturated acyl-CoA hydratase; beta-hydroxyacyl-CoA dehydrase; beta-hydroxyacid dehydrase; hydratase, enoyl coenzyme A; acyl coenzyme A hydrase; crotonase; crotonyl hydrase; 2-octenoyl coenzyme A hydrase; enoyl coenzyme A hydratase; 2-enoyl-CoA hydratase; short-chain enoyl-CoA hydratase; ECH; trans-2-enoyl-CoA hydratase; enoyl coenzyme A hydrase (D); enoyl coenzyme A hydrase (L); short chain enoyl coenzyme A hydratase; D-3-hydroxyacyl-CoA dehydratase; enol-CoA hydratase | (3S)-3-hydroxyacyl-CoA = trans-2(or 3)-enoyl-CoA + H2O [RN:R02685 R07314] |
| ec:4.1.99.3 | 6 | deoxyribodipyrimidine photo-lyase; photoreactivating enzyme; DNA photolyase; DNA-photoreactivating enzyme; DNA cyclobutane dipyrimidine photolyase; DNA photolyase; deoxyribonucleic photolyase; deoxyribodipyrimidine photolyase; photolyase; PRE; PhrB photolyase; deoxyribonucleic cyclobutane dipyrimidine photolyase; phr A photolyase; dipyrimidine photolyase (photosensitive); deoxyribonucleate pyrimidine dimer lyase (photosensitive) | cyclobutadipyrimidine (in DNA) = 2 pyrimidine residues (in DNA) [RN:R00034] |
| ec:3.6.4.12 | 6 | DNA helicase; 3' to 5' DNA helicase; 3'-5' DNA helicase; 3'-5' PfDH; 5' to 3' DNA helicase; AvDH1; BACH1 helicase; BcMCM; BLM protein; BRCA1-associated C-terminal helicase; CeWRN-1; Dbp9p; DmRECQ5; DNA helicase 120; DNA helicase A; DNA helicase E; DNA helicase II; DNA helicase III; DNA helicase RECQL5beta; DNA helicase VI; dnaB; DnaB helicase E1; helicase HDH IV; Hel E; helicase DnaB; helicase domain of bacteriophage T7 gene 4 protein helicase; PcrA helicase; UvrD; hHcsA; Hmi1p; hPif1; MCM helicase; MCM protein; MER3 helicase; MER3 protein; MPH1; PcrA; PcrA helicase; PDH120; PfDH A; Pfh1p; PIF1 | ATP + H2O = ADP + phosphate [RN:R00086] |
| ec:1.14.15.3 | 5 | alkane 1-monooxygenase; alkane 1-hydroxylase; omega-hydroxylase; fatty acid omega-hydroxylase; alkane monooxygenase; 1-hydroxylase; alkane hydroxylase | octane + reduced rubredoxin + O2 = 1-octanol + oxidized rubredoxin + H2O [RN:R02879] |
| ec:2.3.1.47 | 5 | 8-amino-7-oxononanoate synthase; 7-keto-8-aminopelargonic acid synthetase; 7-keto-8-aminopelargonic synthetase; 8-amino-7-oxopelargonate synthase; bioF (gene name) | pimeloyl-[acyl-carrier protein] + L-alanine = 8-amino-7-oxononanoate + CO2 + holo-[acyl-carrier protein] [RN:R03210] |
| ec:2.1.1.197 | 5 | malonyl-[acyl-carrier protein] O-methyltransferase; BioC | S-adenosyl-L-methionine + malonyl-[acyl-carrier protein] = S-adenosyl-L-homocysteine + malonyl-[acyl-carrier protein] methyl ester [RN:R09543] |
| ec:2.6.1.62 | 5 | adenosylmethionine---8-amino-7-oxononanoate transaminase; 7,8-diaminonanoate transaminase; 7,8-diaminononanoate transaminase; DAPA transaminase (ambiguous); 7,8-diaminopelargonic acid aminotransferase; DAPA aminotransferase (ambiguous); 7-keto-8-aminopelargonic acid; diaminopelargonate synthase; 7-keto-8-aminopelargonic acid aminotransferase | S-adenosyl-L-methionine + 8-amino-7-oxononanoate = S-adenosyl-4-methylthio-2-oxobutanoate + 7,8-diaminononanoate [RN:R03231] |
| ec:2.4.1.1 | 5 | glycogen phosphorylase; muscle phosphorylase a and b; amylophosphorylase; polyphosphorylase; amylopectin phosphorylase; glucan phosphorylase; alpha-glucan phosphorylase; 1,4-alpha-glucan phosphorylase; glucosan phosphorylase; granulose phosphorylase; maltodextrin phosphorylase; muscle phosphorylase; myophosphorylase; potato phosphorylase; starch phosphorylase; 1,4-alpha-D-glucan:phosphate alpha-D-glucosyltransferase; phosphorylase (ambiguous) | [(1->4)-alpha-D-glucosyl]n + phosphate = [(1->4)-alpha-D-glucosyl]n-1 + alpha-D-glucose 1-phosphate [RN:R01821 R06050] |
| ec:1.4.1.2 | 5 | glutamate dehydrogenase; glutamic dehydrogenase; glutamate dehydrogenase (NAD+); glutamate oxidoreductase; glutamic acid dehydrogenase; L-glutamate dehydrogenase; NAD+-dependent glutamate dehydrogenase; NAD+-dependent glutamic dehydrogenase; NAD+-glutamate dehydrogenase; NAD+-linked glutamate dehydrogenase; NAD+-linked glutamic dehydrogenase; NAD+-specific glutamic dehydrogenase; NAD+-specific glutamate dehydrogenase; NAD+:glutamate oxidoreductase; NADH-linked glutamate dehydrogenase | L-glutamate + H2O + NAD+ = 2-oxoglutarate + NH3 + NADH + H+ [RN:R00243] |
| ec:4.4.1.15 | 5 | D-cysteine desulfhydrase; D-cysteine lyase; D-cysteine sulfide-lyase (deaminating) | D-cysteine + H2O = sulfide + NH3 + pyruvate [RN:R01874] |
| ec:2.8.1.6 | 5 | biotin synthase; dethiobiotin:sulfur sulfurtransferase | dethiobiotin + sulfur-(sulfur carrier) + 2 S-adenosyl-L-methionine = biotin + (sulfur carrier) + 2 L-methionine + 2 5'-deoxyadenosine [RN:R01078] |
| ec:4.99.1.1 | 5 | ferrochelatase; ferro-protoporphyrin chelatase; iron chelatase; heme synthetase; heme synthase; protoheme ferro-lyase | protoheme + 2 H+ = protoporphyrin + Fe2+ [RN:R00310] |
| ec:3.1.1.11 | 5 | pectinesterase; pectin demethoxylase; pectin methoxylase; pectin methylesterase; pectase; pectin methyl esterase; pectinoesterase | pectin + n H2O = n methanol + pectate [RN:R02362 R06250] |
| ec:6.3.3.3 | 5 | dethiobiotin synthase; desthiobiotin synthase | ATP + 7,8-diaminononanoate + CO2 = ADP + phosphate + dethiobiotin [RN:R03182] |
| ec:1.2.1.2 | 5 | formate dehydrogenase; formate-NAD+ oxidoreductase; FDH I; FDH II; N-FDH; formic hydrogen-lyase; formate hydrogenlyase; hydrogenlyase; NAD+-linked formate dehydrogenase; NAD+-dependent formate dehydrogenase; formate dehydrogenase (NAD+); NAD+-formate dehydrogenase; formate benzyl-viologen oxidoreductase; formic acid dehydrogenase | formate + NAD+ = CO2 + NADH [RN:R00519] |
| ec:2.7.1.23 | 5 | NAD+ kinase; DPN kinase; nicotinamide adenine dinucleotide kinase (phosphorylating); nicotinamide adenine dinucleotide kinase; NAD kinase; NADK | ATP + NAD+ = ADP + NADP+ [RN:R00104] |
| ec:2.1.1.72 | 4 | site-specific DNA-methyltransferase (adenine-specific); modification methylase; restriction-modification system | S-adenosyl-L-methionine + DNA adenine = S-adenosyl-L-homocysteine + DNA 6-methylaminopurine [RN:R02961] |
| ec:5.1.2.3 | 4 | 3-hydroxybutyryl-CoA epimerase; 3-hydroxybutyryl coenzyme A epimerase; 3-hydroxyacyl-CoA epimerase | (S)-3-hydroxybutanoyl-CoA = (R)-3-hydroxybutanoyl-CoA [RN:R03276] |
| ec:4.2.3.5 | 4 | chorismate synthase; 5-O-(1-carboxyvinyl)-3-phosphoshikimate phosphate-lyase | 5-O-(1-carboxyvinyl)-3-phosphoshikimate = chorismate + phosphate [RN:R01714] |
| ec:1.1.1.35 | 4 | 3-hydroxyacyl-CoA dehydrogenase; beta-hydroxyacyl dehydrogenase; beta-keto-reductase; 3-keto reductase; 3-hydroxyacyl coenzyme A dehydrogenase; beta-hydroxyacyl-coenzyme A synthetase; beta-hydroxyacylcoenzyme A dehydrogenase; beta-hydroxybutyrylcoenzyme A dehydrogenase; 3-hydroxyacetyl-coenzyme A dehydrogenase; L-3-hydroxyacyl coenzyme A dehydrogenase; L-3-hydroxyacyl CoA dehydrogenase; beta-hydroxyacyl CoA dehydrogenase; 3beta-hydroxyacyl coenzyme A dehydrogenase; 3-hydroxybutyryl-CoA dehydrogenase; beta-ketoacyl-CoA reductase; beta-hydroxy acid dehydrogenase; 3-L-hydroxyacyl-CoA dehydrogenase; 3-hydroxyisobutyryl-CoA dehydrogenase; 1-specific DPN-linked beta-hydroxybutyric dehydrogenase | (S)-3-hydroxyacyl-CoA + NAD+ = 3-oxoacyl-CoA + NADH + H+ [RN:R01778] |
| ec:2.9.1.1 | 4 | L-seryl-tRNASec selenium transferase; L-selenocysteinyl-tRNASel synthase; L-selenocysteinyl-tRNASec synthase selenocysteine synthase; cysteinyl-tRNASec-selenium transferase; cysteinyl-tRNASec-selenium transferase | L-seryl-tRNASec + selenophosphate = L-selenocysteinyl-tRNASec + phosphate [RN:R08219] |
| ec:2.3.1.16 | 4 | acetyl-CoA C-acyltransferase; beta-ketothiolase; 3-ketoacyl-CoA thiolase; KAT; beta-ketoacyl coenzyme A thiolase; beta-ketoacyl-CoA thiolase; beta-ketoadipyl coenzyme A thiolase; beta-ketoadipyl-CoA thiolase; 3-ketoacyl CoA thiolase; 3-ketoacyl coenzyme A thiolase; 3-ketoacyl thiolase; 3-ketothiolase; 3-oxoacyl-CoA thiolase; 3-oxoacyl-coenzyme A thiolase; 6-oxoacyl-CoA thiolase; acetoacetyl-CoA beta-ketothiolase; acetyl-CoA acyltransferase; ketoacyl-CoA acyltransferase; ketoacyl-coenzyme A thiolase; long-chain 3-oxoacyl-CoA thiolase; oxoacyl-coenzyme A thiolase; pro-3-ketoacyl-CoA thiolase; thiolase I; 2-methylacetoacetyl-CoA thiolase [misleading] | acyl-CoA + acetyl-CoA = CoA + 3-oxoacyl-CoA [RN:R00391] |
| ec:2.5.1.18 | 4 | glutathione transferase; glutathione S-transferase; glutathione S-alkyltransferase; glutathione S-aryltransferase; S-(hydroxyalkyl)glutathione lyase; glutathione S-aralkyltransferase; glutathione S-alkyl transferase; GST | RX + glutathione = HX + R-S-glutathione [RN:R03522 R08511 R08512] |
| ec:4.4.1.5 | 4 | lactoylglutathione lyase; methylglyoxalase; aldoketomutase; ketone-aldehyde mutase; glyoxylase I; (R)-S-lactoylglutathione methylglyoxal-lyase (isomerizing) | (R)-S-lactoylglutathione = glutathione + methylglyoxal [RN:R02530] |
| ec:1.1.1.57 | 4 | fructuronate reductase; mannonate oxidoreductase; mannonic dehydrogenase; D-mannonate dehydrogenase; D-mannonate:NAD+ oxidoreductase | D-mannonate + NAD+ = D-fructuronate + NADH + H+ [RN:R02454] |
| ec:3.6.3.32 | 3 | quaternary-amine-transporting ATPase | ATP + H2O + quaternary amineout = ADP + phosphate + quaternary aminein [RN:R00086] |
| ec:2.3.1.30 | 3 | serine O-acetyltransferase; SATase; L-serine acetyltransferase; serine acetyltransferase; serine transacetylase | acetyl-CoA + L-serine = CoA + O-acetyl-L-serine [RN:R00586] |
| ec:3.1.1.31 | 3 | 6-phosphogluconolactonase; phosphogluconolactonase; 6-PGL | 6-phospho-D-glucono-1,5-lactone + H2O = 6-phospho-D-gluconate [RN:R02035] |
| ec:2.7.13.3 | 3 | histidine kinase; EnvZ; histidine kinase (ambiguous); histidine protein kinase (ambiguous); protein histidine kinase (ambiguous); protein kinase (histidine) (ambiguous); HK1; HP165; Sln1p | ATP + protein L-histidine = ADP + protein N-phospho-L-histidine |
| ec:3.6.3.12 | 3 | K+-transporting ATPase; K+-translocating Kdp-ATPase; multi-subunit K+-transport ATPase | ATP + H2O + K+out = ADP + phosphate + K+in [RN:R00086] |
| ec:3.4.11.5 | 3 | prolyl aminopeptidase; proline aminopeptidase; Pro-X aminopeptidase; cytosol aminopeptidase V; proline iminopeptidase | Release of N-terminal proline from a peptide |
| ec:2.7.8.5 | 3 | CDP-diacylglycerol---glycerol-3-phosphate 3-phosphatidyltransferase; glycerophosphate phosphatidyltransferase; 3-phosphatidyl-1'-glycerol-3'-phosphate synthase; CDPdiacylglycerol:glycerol-3-phosphate phosphatidyltransferase; cytidine 5'-diphospho-1,2-diacyl-sn-glycerol (CDPdiglyceride):sn-glycerol-3-phosphate phosphatidyltransferase; phosphatidylglycerophosphate synthase; phosphatidylglycerolphosphate synthase; PGP synthase; CDPdiacylglycerol-sn-glycerol-3-phosphate 3-phosphatidyltransferase; CDPdiacylglycerol:sn-glycero-3-phosphate phosphatidyltransferase; glycerol phosphate phosphatidyltransferase; glycerol 3-phosphate phosphatidyltransferase; phosphatidylglycerol phosphate synthase; phosphatidylglycerol phosphate synthetase; phosphatidylglycerophosphate synthetase; sn-glycerol-3-phosphate phosphatidyltransferase | CDP-diacylglycerol + sn-glycerol 3-phosphate = CMP + 3(3-sn-phosphatidyl)-sn-glycerol 1-phosphate [RN:R01801] |
| ec:2.7.7.7 | 3 | DNA-directed DNA polymerase; DNA polymerase I; DNA polymerase II; DNA polymerase III; DNA polymerase alpha; DNA polymerase beta; DNA polymerase gamma; DNA nucleotidyltransferase (DNA-directed); DNA nucleotidyltransferase (DNA-directed); deoxyribonucleate nucleotidyltransferase; deoxynucleate polymerase; deoxyribonucleic acid duplicase; deoxyribonucleic acid polymerase; deoxyribonucleic duplicase; deoxyribonucleic polymerase; deoxyribonucleic polymerase I; DNA duplicase; DNA nucleotidyltransferase; DNA polymerase; DNA replicase; DNA-dependent DNA polymerase; duplicase; Klenow fragment; sequenase; Taq DNA polymerase; Taq Pol I; Tca DNA polymerase | deoxynucleoside triphosphate + DNAn = diphosphate + DNAn+1 [RN:R00379] |
| ec:2.7.1.35 | 3 | pyridoxal kinase; pyridoxal kinase (phosphorylating); pyridoxal 5-phosphate-kinase; pyridoxal phosphokinase; pyridoxine kinase | ATP + pyridoxal = ADP + pyridoxal 5'-phosphate [RN:R00174] |
| ec:2.7.7.42 | 3 | [glutamate---ammonia-ligase] adenylyltransferase; glutamine-synthetase adenylyltransferase; ATP:glutamine synthetase adenylyltransferase; adenosine triphosphate:glutamine synthetase adenylyltransferase | ATP + [L-glutamate:ammonia ligase (ADP-forming)] = diphosphate + adenylyl-[L-glutamate:ammonia ligase (ADP-forming)] [RN:R03473] |
| ec:1.7.2.5 | 3 | nitric oxide reductase (cytochrome c) | nitrous oxide + 2 ferricytochrome c + H2O = 2 nitric oxide + 2 ferrocytochrome c + 2 H+ [RN:R00294] |
| ec:2.4.1.173 | 3 | sterol 3beta-glucosyltransferase; UDPG:sterol glucosyltransferase; UDP-glucose-sterol beta-glucosyltransferase; sterol:UDPG glucosyltransferase; UDPG-SGTase; uridine diphosphoglucose-poriferasterol glucosyltransferase; uridine diphosphoglucose-sterol glucosyltransferase; sterol glucosyltransferase; sterol-beta-D-glucosyltransferase; UDP-glucose-sterol glucosyltransferase | UDP-glucose + a sterol = UDP + a sterol 3-beta-D-glucoside [RN:R02113] |
| ec:5.2.1.8 | 2 | peptidylprolyl isomerase; PPIase; cyclophilin [misleading, see comments]; peptide bond isomerase; peptidyl-prolyl cis-trans isomerase | peptidylproline (omega=180) = peptidylproline (omega=0) [RN:R04273] |
| ec:3.6.3.36 | 2 | taurine-transporting ATPase | ATP + H2O + taurineout = ADP + phosphate + taurinein [RN:R00086] |
| ec:3.6.3.29 | 2 | molybdate-transporting ATPase | ATP + H2O + molybdateout = ADP + phosphate + molybdatein [RN:R00086] |
| ec:2.1.1.181 | 2 | 23S rRNA (adenine1618-N6)-methyltransferase; rRNA large subunit methyltransferase F; YbiN protein; rlmF (gene name); m6A1618 methyltransferase | S-adenosyl-L-methionine + adenine1618 in 23S rRNA = S-adenosyl-L-homocysteine + N6-methyladenine1618 in 23S rRNA [RN:R07232] |
| ec:2.1.1.61 | 2 | tRNA (5-methylaminomethyl-2-thiouridylate)-methyltransferase; transfer ribonucleate 5-methylaminomethyl-2-thiouridylate 5-methyltransferase; tRNA 5-methylaminomethyl-2-thiouridylate 5'-methyltransferase | S-adenosyl-L-methionine + tRNA containing 5-aminomethyl-2-thiouridine = S-adenosyl-L-homocysteine + tRNA containing 5-methylaminomethyl-2-thiouridylate [RN:R00601] |
| ec:4.3.1.12 | 2 | ornithine cyclodeaminase; ornithine cyclase; ornithine cyclase (deaminating); L-ornithine ammonia-lyase (cyclizing) | L-ornithine = L-proline + NH3 [RN:R00671] |
| ec:1.3.99.16 | 2 | isoquinoline 1-oxidoreductase | isoquinoline + acceptor + H2O = isoquinolin-1(2H)-one + reduced acceptor [RN:R05151] |
| ec:4.1.1.44 | 2 | 4-carboxymuconolactone decarboxylase; gamma-4-carboxymuconolactone decarboxylase; 4-carboxymuconolactone carboxy-lyase; 2-carboxy-2,5-dihydro-5-oxofuran-2-acetate carboxy-lyase (4,5-dihydro-5-oxofuran-2-acetate-forming) | (R)-2-carboxy-2,5-dihydro-5-oxofuran-2-acetate = 4,5-dihydro-5-oxofuran-2-acetate + CO2 [RN:R03470] |
| ec:1.1.3.15 | 2 | (S)-2-hydroxy-acid oxidase; hydroxy-acid oxidase A; hydroxy-acid oxidase B; glycolate oxidase; L-2-hydroxy acid oxidase; hydroxyacid oxidase A; L-alpha-hydroxy acid oxidase | an (S)-2-hydroxy carboxylate + O2 = a 2-oxo carboxylate + H2O2 [RN:R01341] |
| ec:1.12.99.6 | 2 | hydrogenase (acceptor); H2 producing hydrogenase[ambiguous]; hydrogen-lyase[ambiguous]; hydrogenlyase[ambiguous]; uptake hydrogenase[ambiguous]; hydrogen:(acceptor) oxidoreductase | H2 + A = AH2 [RN:R07182] |
| ec:3.5.5.1 | 2 | nitrilase; acetonitrilase; benzonitrilase | a nitrile + 2 H2O = a carboxylate + NH3 [RN:R00540] |
| ec:6.2.1.3 | 2 | long-chain-fatty-acid---CoA ligase; acyl-CoA synthetase; fatty acid thiokinase (long chain); acyl-activating enzyme; palmitoyl-CoA synthase; lignoceroyl-CoA synthase; arachidonyl-CoA synthetase; acyl coenzyme A synthetase; acyl-CoA ligase; palmitoyl coenzyme A synthetase; thiokinase; palmitoyl-CoA ligase; acyl-coenzyme A ligase; fatty acid CoA ligase; long-chain fatty acyl coenzyme A synthetase; oleoyl-CoA synthetase; stearoyl-CoA synthetase; long chain fatty acyl-CoA synthetase; long-chain acyl CoA synthetase; fatty acid elongase; LCFA synthetase; pristanoyl-CoA synthetase; ACS3; long-chain acyl-CoA synthetase I; long-chain acyl-CoA synthetase II; fatty acyl-coenzyme A synthetase; long-chain acyl-coenzyme A synthetase; FAA1 | ATP + a long-chain fatty acid + CoA = AMP + diphosphate + an acyl-CoA [RN:R00390] |
| ec:1.1.1.1 | 1 | alcohol dehydrogenase; aldehyde reductase; ADH; alcohol dehydrogenase (NAD); aliphatic alcohol dehydrogenase; ethanol dehydrogenase; NAD-dependent alcohol dehydrogenase; NAD-specific aromatic alcohol dehydrogenase; NADH-alcohol dehydrogenase; NADH-aldehyde dehydrogenase; primary alcohol dehydrogenase; yeast alcohol dehydrogenase | (1) a primary alcohol + NAD+ = an aldehyde + NADH + H+ [RN:R07326]; (2) a secondary alcohol + NAD+ = a ketone + NADH + H+ [RN:R07327] |
| ec:1.18.1.3 | 1 | ferredoxin---NAD+ reductase; ferredoxin-nicotinamide adenine dinucleotide reductase; ferredoxin reductase (ambiguous); NAD+-ferredoxin reductase; NADH-ferredoxin oxidoreductase; reductase, reduced nicotinamide adenine dinucleotide-ferredoxin; ferredoxin-NAD+ reductase; NADH-ferredoxin reductase; NADH2-ferredoxin oxidoreductase; NADH flavodoxin oxidoreductase; NADH-ferredoxin NAP reductase (component of naphthalene dioxygenase multicomponent enzyme system); ferredoxin-linked NAD+ reductase; NADH-ferredoxin TOL reductase (component of toluene dioxygenase); ferredoxin---NAD reductase | (1) 2 reduced [2Fe-2S] ferredoxin + NAD+ + H+ = 2 oxidized [2Fe-2S] ferredoxin + NADH [RN:R05875]; (2) reduced 2[4Fe-4S] ferredoxin + NAD+ + H+ = oxidized 2[4Fe-4S] ferredoxin + NADH |
| ec:6.1.1.3 | 1 | threonine---tRNA ligase; threonyl-tRNA synthetase; threonyl-transfer ribonucleate synthetase; threonyl-transfer RNA synthetase; threonyl-transfer ribonucleic acid synthetase; threonyl ribonucleic synthetase; threonine-transfer ribonucleate synthetase; threonine translase; threonyl-tRNA synthetase; TRS | ATP + L-threonine + tRNAThr = AMP + diphosphate + L-threonyl-tRNAThr [RN:R03663] |
| ec:1.1.1.306 | 1 | S-(hydroxymethyl)mycothiol dehydrogenase; NAD/factor-dependent formaldehyde dehydrogenase; mycothiol-dependent formaldehyde dehydrogenase | S-(hydroxymethyl)mycothiol + NAD+ = S-formylmycothiol + NADH + H+ [RN:R09129] |
| ec:4.2.3.22 | 1 | germacradienol synthase; germacradienol/germacrene-D synthase; 2-trans,6-trans-farnesyl-diphosphate diphosphate-lyase [(1E,4S,5E,7R)-germacra-1(10),5-dien-11-ol-forming] | (2E,6E)-farnesyl diphosphate + H2O = (1E,4S,5E,7R)-germacra-1(10),5-dien-11-ol + diphosphate [RN:R07647] |
| ec:1.13.12.16 | 1 | nitronate monooxygenase; NMO; 2-nitropropane dioxygenase (incorrect) | ethylnitronate + O2 = acetaldehyde + nitrite + other products [RN:R00025] |
| ec:2.1.2.11 | 1 | 3-methyl-2-oxobutanoate hydroxymethyltransferase; alpha-ketoisovalerate hydroxymethyltransferase; dehydropantoate hydroxymethyltransferase; ketopantoate hydroxymethyltransferase; oxopantoate hydroxymethyltransferase; 5,10-methylene tetrahydrofolate:alpha-ketoisovalerate hydroxymethyltransferase | 5,10-methylenetetrahydrofolate + 3-methyl-2-oxobutanoate + H2O = tetrahydrofolate + 2-dehydropantoate [RN:R01226] |
| ec:4.1.99.16 | 1 | geosmin synthase | (1E,4S,5E,7R)-germacra-1(10),5-dien-11-ol + H2O = (-)-geosmin + acetone [RN:R09487] |
| ec:2.4.2.1 | 1 | purine-nucleoside phosphorylase; inosine phosphorylase; PNPase; PUNPI; PUNPII; inosine-guanosine phosphorylase; nucleotide phosphatase; purine deoxynucleoside phosphorylase; purine deoxyribonucleoside phosphorylase; purine nucleoside phosphorylase; purine ribonucleoside phosphorylase | (1) purine ribonucleoside + phosphate = purine + alpha-D-ribose 1-phosphate [RN:R08368]; (2) purine deoxyribonucleoside + phosphate = purine + 2'-deoxy-alpha-D-ribose 1-phosphate [RN:R10244] |
| ec:6.3.1.10 | 1 | adenosylcobinamide-phosphate synthase; CbiB | (1) ATP + adenosylcobyric acid + (R)-1-aminopropan-2-yl phosphate = ADP + phosphate + adenosylcobinamide phosphate [RN:R06529]; (2) ATP + adenosylcobyric acid + (R)-1-aminopropan-2-ol = ADP + phosphate + adenosylcobinamide [RN:R07302] |
| ec:4.2.3.75 | 1 | (-)-germacrene D synthase | (2E,6E)-farnesyl diphosphate = (-)-germacrene D + diphosphate [RN:R07648] |
| ec:4.2.1.3 | 1 | aconitate hydratase; cis-aconitase; aconitase; AcnB; 2-methylaconitate hydratase; citrate(isocitrate) hydro-lyase | citrate = isocitrate (overall reaction) [RN:R01324]; (1a) citrate = cis-aconitate + H2O [RN:R01325]; (1b) cis-aconitate + H2O = isocitrate [RN:R01900] |
| ec:2.8.3.16 | 1 | formyl-CoA transferase; formyl-coenzyme A transferase; formyl-CoA oxalate CoA-transferase | formyl-CoA + oxalate = formate + oxalyl-CoA [RN:R07290] |
| ec:3.5.4.12 | 1 | dCMP deaminase; deoxycytidylate deaminase; deoxy-CMP-deaminase; deoxycytidylate aminohydrolase; deoxycytidine monophosphate deaminase; deoxycytidine-5'-phosphate deaminase; deoxycytidine-5'-monophosphate aminohydrolase | dCMP + H2O = dUMP + NH3 [RN:R01663] |
| ec:1.11.1.6 | 1 | catalase; equilase; caperase; optidase; catalase-peroxidase; CAT | 2 H2O2 = O2 + 2 H2O [RN:R00009] |
| ec:1.3.8.7 | 1 | medium-chain acyl-CoA dehydrogenase; fatty acyl coenzyme A dehydrogenase (ambiguous); acyl coenzyme A dehydrogenase (ambiguous); acyl dehydrogenase (ambiguous); fatty-acyl-CoA dehydrogenase (ambiguous); acyl CoA dehydrogenase (ambiguous); general acyl CoA dehydrogenase (ambiguous); medium-chain acyl-coenzyme A dehydrogenase; acyl-CoA:(acceptor) 2,3-oxidoreductase (ambiguous); ACADM (gene name). | a medium-chain acyl-CoA + electron-transfer flavoprotein = a medium-chain trans-2,3-dehydroacyl-CoA + reduced electron-transfer flavoprotein [RN:R00392] |
| ec:1.9.3.1 | 1 | cytochrome-c oxidase; cytochrome oxidase; cytochrome a3; cytochrome aa3; Warburg's respiratory enzyme; indophenol oxidase; indophenolase; complex IV (mitochondrial electron transport); ferrocytochrome c oxidase; NADH cytochrome c oxidase | 4 ferrocytochrome c + O2 + 4 H+ = 4 ferricytochrome c + 2 H2O [RN:R00081] |
| ec:1.1.1.85 | 1 | 3-isopropylmalate dehydrogenase; beta-isopropylmalic enzyme; beta-isopropylmalate dehydrogenase; threo-Ds-3-isopropylmalate dehydrogenase; 3-carboxy-2-hydroxy-4-methylpentanoate:NAD+ oxidoreductase | (2R,3S)-3-isopropylmalate + NAD+ = 4-methyl-2-oxopentanoate + CO2 + NADH + H+ (overall reaction) [RN:R10052]; (1a) (2R,3S)-3-isopropylmalate + NAD+ = (2S)-2-isopropyl-3-oxosuccinate + NADH + H+ [RN:R04426]; (1b) (2S)-2-isopropyl-3-oxosuccinate = 4-methyl-2-oxopentanoate + CO2 (spontaneous) [RN:R01652] |
| ec:2.7.11.1 | 1 | non-specific serine/threonine protein kinase; A-kinase; AP50 kinase; ATP-protein transphosphorylase; calcium-dependent protein kinase C; calcium/phospholipid-dependent protein kinase; cAMP-dependent protein kinase; cAMP-dependent protein kinase A; casein kinase; casein kinase (phosphorylating); casein kinase 2; casein kinase I; casein kinase II; cGMP-dependent protein kinase; CK-2; CKI; CKII; cyclic AMP-dependent protein kinase; cyclic AMP-dependent protein kinase A; cyclic monophosphate-dependent protein kinase; cyclic nucleotide-dependent protein kinase; cyclin-dependent kinase; cytidine 3',5'-cyclic monophosphate-responsive protein kinase; dsk1; glycogen synthase a kinase; glycogen synthase kinase; HIPK2; Hpr kinase; hydroxyalkyl-protein kinase; hydroxyalkyl-protein kinase; M phase-specific cdc2 kinase; mitogen-activated S6 kinase; p82 kinase; phosphorylase b kinase kinase; PKA; protein glutamyl kinase; protein kinase (phosphorylating); protein kinase A; protein kinase CK2; protein kinase p58; protein phosphokinase; protein serine kinase; protein serine-threonine kinase; protein-aspartyl kinase; protein-cysteine kinase; protein-serine kinase; Prp4 protein kinase; Raf kinase; Raf-1; ribosomal protein S6 kinase II; ribosomal S6 protein kinase; serine kinase; serine protein kinase; serine-specific protein kinase; serine(threonine) protein kinase; serine/threonine protein kinase; STK32; T-antigen kinase; threonine-specific protein kinase; twitchin kinase; type-2 casein kinase; betaIIPKC; epsilon PKC; Wee 1-like kinase; Wee-kinase; WEE1Hu | ATP + a protein = ADP + a phosphoprotein [RN:R00162] |
| ec:3.1.3.5 | 1 | 5'-nucleotidase; uridine 5'-nucleotidase; 5'-adenylic phosphatase; adenosine 5'-phosphatase; AMP phosphatase; adenosine monophosphatase; 5'-mononucleotidase; AMPase; UMPase; snake venom 5'-nucleotidase; thimidine monophosphate nucleotidase; 5'-AMPase; 5'-AMP nucleotidase; AMP phosphohydrolase; IMP 5'-nucleotidase | a 5'-ribonucleotide + H2O = a ribonucleoside + phosphate [RN:R07297] |
| ec:3.4.13.19 | 1 | membrane dipeptidase; renal dipeptidase; dehydropeptidase I (DPH I); dipeptidase (ambiguous); aminodipeptidase; dipeptide hydrolase (ambiguous); dipeptidyl hydrolase (ambiguous); nonspecific dipeptidase; glycosyl-phosphatidylinositol-anchored renal dipeptidase; MDP | Hydrolysis of dipeptides |
| ec:3.2.1.21 | 1 | beta-glucosidase; gentiobiase; cellobiase; emulsin; elaterase; aryl-beta-glucosidase; beta-D-glucosidase; beta-glucoside glucohydrolase; arbutinase; amygdalinase; p-nitrophenyl beta-glucosidase; primeverosidase; amygdalase; linamarase; salicilinase; beta-1,6-glucosidase | Hydrolysis of terminal, non-reducing beta-D-glucosyl residues with release of beta-D-glucose |
| ec:1.2.1.20 | 1 | glutarate-semialdehyde dehydrogenase; glutarate semialdehyde dehydrogenase | 5-oxopentanoate + NAD+ + H2O = glutarate + NADH + 2 H+ [RN:R02401] |
| ec:2.7.1.2 | 1 | glucokinase; glucokinase (phosphorylating) | ATP + D-glucose = ADP + D-glucose 6-phosphate [RN:R00299] |
| ec:1.2.1.16 | 1 | succinate-semialdehyde dehydrogenase [NAD(P)+]; succinate semialdehyde dehydrogenase (nicotinamide adenine dinucleotide (phosphate)); succinate-semialdehyde dehydrogenase [NAD(P)] | succinate semialdehyde + NAD(P)+ + H2O = succinate + NAD(P)H + 2 H+ [RN:R00713 R00714] |
| ec:1.2.1.79 | 1 | succinate-semialdehyde dehydrogenase (NADP+); succinic semialdehyde dehydrogenase (NADP+); succinyl semialdehyde dehydrogenase (NADP+); succinate semialdehyde:NADP+ oxidoreductase; NADP-dependent succinate-semialdehyde dehydrogenase; GabD | succinate semialdehyde + NADP+ + H2O = succinate + NADPH + 2 H+ [RN:R00714] |
| ec:1.2.1.3 | 1 | aldehyde dehydrogenase (NAD+); CoA-independent aldehyde dehydrogenase; m-methylbenzaldehyde dehydrogenase; NAD-aldehyde dehydrogenase; NAD-dependent 4-hydroxynonenal dehydrogenase; NAD-dependent aldehyde dehydrogenase; NAD-linked aldehyde dehydrogenase; propionaldehyde dehydrogenase; aldehyde dehydrogenase (NAD) | an aldehyde + NAD+ + H2O = a carboxylate + NADH + H+ [RN:R00538] |
| ec:5.3.1.12 | 1 | glucuronate isomerase; uronic isomerase; uronate isomerase; D-glucuronate isomerase; uronic acid isomerase; D-glucuronate ketol-isomerase | D-glucuronate = D-fructuronate [RN:R01482] |
| ec:1.1.1.284 | 1 | S-(hydroxymethyl)glutathione dehydrogenase; NAD-linked formaldehyde dehydrogenase (incorrect); formaldehyde dehydrogenase (incorrect); formic dehydrogenase (incorrect); class III alcohol dehydrogenase; ADH3; chi-ADH; FDH (incorrect); formaldehyde dehydrogenase (glutathione) (incorrect); GS-FDH (incorrect); glutathione-dependent formaldehyde dehydrogenase (incorrect); NAD-dependent formaldehyde dehydrogenase; GD-FALDH; NAD- and glutathione-dependent formaldehyde dehydrogenase | S-(hydroxymethyl)glutathione + NAD(P)+ = S-formylglutathione + NAD(P)H + H+ [RN:R06983 R07140] |
| ec:2.6.1.9 | 1 | histidinol-phosphate transaminase; imidazolylacetolphosphate transaminase; glutamic-imidazoleacetol phosphate transaminase; histidinol phosphate aminotransferase; imidazoleacetol phosphate transaminase; L-histidinol phosphate aminotransferase; histidine:imidazoleacetol phosphate transaminase; IAP transaminase; imidazolylacetolphosphate aminotransferase | L-histidinol phosphate + 2-oxoglutarate = 3-(imidazol-4-yl)-2-oxopropyl phosphate + L-glutamate [RN:R03243] |

  
**Over-represented Metabolite Summary**: Collection of the metabolites identified as substrates or products of the proteins representaed the "Over-represented Enzyme Summary" ranked by frequency of occurrence  

| ID | Structure | Name | Frequency | EC |
| --- | --- | --- | --- | --- |
| cpd:C00001 |  | H2O; Water | 379 | ec:1.2.1.79 ec:4.2.1.17 ec:3.5.1.96 ec:1.2.1.3 ec:4.2.3.22 ec:1.1.1.35 ec:1.4.1.2 ec:3.5.4.12 ec:4.4.1.15 ec:1.2.1.16 ec:1.13.12.16 ec:1.2.1.71 ec:3.5.1.32 ec:3.5.3.23 ec:1.1.1.284 ec:3.1.3.11 ec:2.1.2.11 ec:4.2.1.3 ec:1.14.15.3 ec:5.1.2.3 ec:3.5.1.68 ec:4.1.99.16 ec:3.1.1.31 ec:1.2.1.20 ec:3.1.3.5 ec:1.1.1.306 ec:3.2.1.21 ec:4.2.1.49 ec:3.1.1.11 ec:3.5.2.7 ec:3.1.3.77 ec:1.18.1.3 ec:1.9.3.1 ec:3.4.11.5 ec:3.5.3.8 ec:3.5.5.1 ec:1.11.1.6 ec:4.2.3.75 ec:1.1.1.1 ec:1.7.2.5 ec:3.5.3.13 |
| cpd:C00439 |  | N-Formimino-L-glutamate; N-Formimidoyl-L-glutamate | 174 | ec:3.5.2.7 ec:3.5.3.8 ec:3.5.3.13 |
| cpd:C00014 |  | Ammonia; NH3 | 163 | ec:1.4.1.2 ec:3.5.4.12 ec:4.4.1.15 ec:6.3.1.2 ec:1.18.1.3 ec:4.3.1.3 ec:4.3.1.12 ec:3.5.5.1 ec:3.5.3.23 ec:3.5.3.13 |
| cpd:C00785 |  | Urocanate; Urocanic acid | 155 | ec:4.3.1.3 ec:4.2.1.49 |
| cpd:C03680 |  | 4-Imidazolone-5-propanoate; 4-Imidazolone-5-propionic acid; 4,5-Dihydro-4-oxo-5-imidazolepropanoate | 153 | ec:3.5.2.7 ec:4.2.1.49 |
| cpd:C00025 |  | L-Glutamate; L-Glutamic acid; L-Glutaminic acid; Glutamate | 95 | ec:1.4.1.2 ec:6.3.1.2 ec:3.5.1.68 ec:2.6.1.81 ec:3.5.3.8 ec:3.5.1.96 ec:2.6.1.9 |
| cpd:C00135 |  | L-Histidine; (S)-alpha-Amino-1H-imidazole-4-propionic acid | 80 | ec:4.3.1.3 |
| cpd:C01045 |  | N-Formyl-L-glutamate | 66 | ec:3.5.1.68 ec:3.5.3.13 |
| cpd:C00080 |  | H+; Hydron | 59 | ec:1.2.1.79 ec:5.1.2.3 ec:2.8.1.6 ec:2.4.2.1 ec:4.2.1.17 ec:1.1.1.57 ec:1.2.1.3 ec:1.2.1.20 ec:1.2.1.2 ec:1.1.1.306 ec:1.1.1.35 ec:4.99.1.1 ec:1.4.1.2 ec:1.1.1.85 ec:1.2.1.16 ec:1.18.1.3 ec:1.9.3.1 ec:1.2.1.71 ec:1.11.1.6 ec:1.1.1.284 ec:1.1.1.1 ec:1.7.2.5 |
| cpd:C00010 |  | CoA; Coenzyme A; CoA-SH | 52 | ec:2.3.1.180 ec:2.3.1.30 ec:2.3.1.16 ec:2.3.1.47 ec:6.2.1.3 ec:2.3.1.109 |
| cpd:C00011 |  | CO2; Carbon dioxide | 51 | ec:2.3.1.180 ec:1.1.1.85 ec:1.18.1.3 ec:2.3.1.47 ec:4.1.1.44 ec:3.5.3.23 ec:1.2.1.2 ec:6.3.3.3 |
| cpd:C00009 |  | Orthophosphate; Phosphate; Phosphoric acid; Orthophosphoric acid | 48 | ec:6.3.1.10 ec:3.1.3.11 ec:2.9.1.1 ec:3.1.3.77 ec:6.3.1.2 ec:2.4.1.1 ec:4.2.3.5 ec:2.4.2.1 ec:6.3.3.3 ec:3.1.3.5 |
| cpd:C00003 |  | NAD+; NAD; Nicotinamide adenine dinucleotide; DPN; Diphosphopyridine nucleotide; Nadide | 48 | ec:1.2.1.79 ec:5.1.2.3 ec:4.2.1.17 ec:1.1.1.57 ec:1.2.1.3 ec:1.2.1.20 ec:1.2.1.2 ec:1.1.1.306 ec:1.1.1.35 ec:1.4.1.2 ec:1.1.1.85 ec:2.7.1.23 ec:1.2.1.16 ec:1.18.1.3 ec:1.2.1.71 ec:1.1.1.284 ec:1.1.1.1 |
| cpd:C00488 |  | Formamide; Methanamide | 47 | ec:3.5.3.8 |
| cpd:C00004 |  | NADH; DPNH; Reduced nicotinamide adenine dinucleotide | 43 | ec:1.2.1.79 ec:5.1.2.3 ec:4.2.1.17 ec:1.1.1.57 ec:1.2.1.3 ec:1.2.1.20 ec:1.2.1.2 ec:1.1.1.306 ec:1.1.1.35 ec:1.1.1.85 ec:1.4.1.2 ec:1.2.1.16 ec:1.18.1.3 ec:1.2.1.71 ec:1.1.1.284 ec:1.1.1.1 |
| cpd:C00002 |  | ATP; Adenosine 5'-triphosphate | 43 | ec:6.3.1.10 ec:6.1.1.3 ec:2.7.1.23 ec:6.3.1.2 ec:6.3.4.5 ec:2.7.1.35 ec:2.7.1.25 ec:6.2.1.3 ec:2.7.1.2 ec:6.3.3.3 |
| cpd:C00058 |  | Formate; Methanoic acid; Formic acid | 32 | ec:3.5.1.68 ec:1.18.1.3 ec:1.2.1.2 ec:1.1.1.306 |
| cpd:C00008 |  | ADP; Adenosine 5'-diphosphate | 31 | ec:6.3.1.10 ec:2.7.1.23 ec:6.3.1.2 ec:2.7.1.35 ec:2.7.1.25 ec:2.7.1.2 ec:6.3.3.3 |
| cpd:C00229 |  | Acyl-carrier protein; ACP; [Acyl-carrier protein]; Holo-[acyl-carrier protein] | 29 | ec:2.3.1.180 ec:2.3.1.47 |
| cpd:C00024 |  | Acetyl-CoA; Acetyl coenzyme A | 21 | ec:2.3.1.180 ec:2.3.1.30 ec:2.3.1.16 |
| cpd:C05931 |  | N-Succinyl-L-glutamate; (2S)-2-(3-Carboxypropanoylamino)pentanedioic acid | 20 | ec:3.5.1.96 ec:1.2.1.71 |
| cpd:C03296 |  | N2-Succinyl-L-arginine; (2S)-2-(3-Carboxypropanoylamino)-5-(diaminomethylideneamino)pentanoic acid | 20 | ec:3.5.3.23 ec:2.3.1.109 |
| cpd:C01209 |  | Malonyl-[acyl-carrier protein]; Malonyl-[acp] | 19 | ec:2.3.1.180 ec:2.1.1.197 |
| cpd:C05932 |  | N-Succinyl-L-glutamate 5-semialdehyde; (2S)-2-(3-Carboxypropanoylamino)-5-oxopentanoic acid | 17 | ec:2.6.1.81 ec:1.2.1.71 |
| cpd:C03415 |  | N2-Succinyl-L-ornithine; (2S)-5-Amino-2-(3-carboxypropanoylamino)pentanoic acid | 17 | ec:2.6.1.81 ec:3.5.3.23 |
| cpd:C00019 |  | S-Adenosyl-L-methionine; S-Adenosylmethionine; AdoMet; SAM | 17 | ec:2.1.1.181 ec:2.8.1.6 ec:2.6.1.62 ec:2.1.1.197 |
| cpd:C00091 |  | Succinyl-CoA; Succinyl coenzyme A | 14 | ec:2.3.1.16 ec:2.3.1.109 |
| cpd:C05744 |  | Acetoacetyl-[acp]; Acetoacetyl-[acyl-carrier protein] | 14 | ec:2.3.1.180 |
| cpd:C00026 |  | 2-Oxoglutarate; Oxoglutaric acid; 2-Ketoglutaric acid; alpha-Ketoglutaric acid | 14 | ec:1.4.1.2 ec:2.6.1.81 ec:1.2.1.3 ec:2.6.1.9 |
| cpd:C03939 |  | Acetyl-[acyl-carrier protein] | 14 | ec:2.3.1.180 |
| cpd:C00020 |  | AMP; Adenosine 5'-monophosphate; Adenylic acid; Adenylate; 5'-AMP; 5'-Adenylic acid; 5'-Adenosine monophosphate; Adenosine 5'-phosphate | 13 | ec:6.1.1.3 ec:6.3.4.5 ec:6.2.1.3 ec:3.1.3.5 |
| cpd:C00013 |  | Diphosphate; Diphosphoric acid; Pyrophosphate; Pyrophosphoric acid; PPi | 13 | ec:6.1.1.3 ec:6.3.4.5 ec:4.1.99.16 ec:6.2.1.3 ec:4.2.3.75 ec:4.2.3.22 |
| cpd:C03069 |  | 3-Methylcrotonyl-CoA; 3-Methylbut-2-enoyl-CoA; 3-Methylcrotonoyl-CoA; Dimethylacryloyl-CoA | 11 | ec:1.3.8.7 ec:5.1.2.3 ec:2.3.1.16 ec:4.2.1.17 ec:1.1.1.35 |
| cpd:C00042 |  | Succinate; Succinic acid; Butanedionic acid; Ethylenesuccinic acid | 11 | ec:1.2.1.79 ec:1.2.1.16 ec:3.5.1.96 ec:1.2.1.20 |
| cpd:C00007 |  | Oxygen; O2 | 11 | ec:1.14.15.3 ec:1.18.1.3 ec:1.9.3.1 ec:1.13.12.16 ec:1.11.1.6 ec:1.1.3.15 |
| cpd:C15606 |  | 1,2-Dihydroxy-5-(methylthio)pent-1-en-3-one | 10 | ec:3.1.3.77 |
| cpd:C00180 |  | Benzoate; Benzoic acid; Benzenecarboxylic acid; Phenylformic acid; Dracylic acid | 10 | ec:3.5.5.1 ec:3.5.1.32 |
| cpd:C03688 |  | Apo-[acyl-carrier-protein] | 10 |  |
| cpd:C00062 |  | L-Arginine; (S)-2-Amino-5-guanidinovaleric acid; L-Arg | 10 | ec:2.3.1.109 |
| cpd:C00054 |  | Adenosine 3',5'-bisphosphate; PAP; 3'-Phosphoadenylate; Phosphoadenosine phosphate | 10 |  |
| cpd:C01092 |  | 8-Amino-7-oxononanoate; 8-Amino-7-oxononanoic acid | 10 | ec:2.6.1.62 ec:2.3.1.47 |
| cpd:C01909 |  | Dethiobiotin; Desthiobiotin | 10 | ec:2.8.1.6 ec:6.3.3.3 |
| cpd:C15650 |  | 2,3-Diketo-5-methylthiopentyl-1-phosphate; 5-(Methylthio)-2,3-dioxopentyl phosphate | 10 | ec:3.1.3.77 |
| cpd:C01037 |  | 7,8-Diaminononanoate | 10 | ec:2.6.1.62 ec:6.3.3.3 |
| cpd:C00327 |  | L-Citrulline; 2-Amino-5-ureidovaleric acid; Citrulline | 9 | ec:6.3.4.5 |
| cpd:C00195 |  | N-Acylsphingosine; Ceramide | 9 | ec:2.4.1.80 |
| cpd:C03406 |  | N-(L-Arginino)succinate; 2-(Nomega-L-Arginino)succinate; L-Argininosuccinate; L-Argininosuccinic acid; L-Arginosuccinic acid | 9 | ec:6.3.4.5 |
| cpd:C01190 |  | beta-D-Glucosyl-(1<->1)-ceramide; Glucosylceramide; Glucocerebroside; D-Glucosyl-N-acylsphingosine | 9 | ec:2.4.1.80 |
| cpd:C00085 |  | D-Fructose 6-phosphate; D-Fructose 6-phosphoric acid; Neuberg ester | 9 | ec:3.1.3.11 |
| cpd:C05378 |  | beta-D-Fructose 1,6-bisphosphate | 9 | ec:3.1.3.11 |
| cpd:C00049 |  | L-Aspartate; L-Aspartic acid; 2-Aminosuccinic acid; L-Asp | 9 | ec:6.3.4.5 |
| cpd:C00041 |  | L-Alanine; L-2-Aminopropionic acid; L-alpha-Alanine | 9 | ec:2.3.1.47 |
| cpd:C05345 |  | beta-D-Fructose 6-phosphate | 9 | ec:3.1.3.11 |
| cpd:C00029 |  | UDP-glucose; UDPglucose; UDP-D-glucose; Uridine diphosphate glucose; UDP-alpha-D-glucose | 9 | ec:2.4.1.80 |
| cpd:C00022 |  | Pyruvate; Pyruvic acid; 2-Oxopropanoate; 2-Oxopropanoic acid; Pyroracemic acid | 9 | ec:4.4.1.15 |
| cpd:C00015 |  | UDP; Uridine 5'-diphosphate | 9 | ec:2.4.1.80 |
| cpd:C00006 |  | NADP+; NADP; Nicotinamide adenine dinucleotide phosphate; beta-Nicotinamide adenine dinucleotide phosphate; TPN; Triphosphopyridine nucleotide | 9 | ec:1.2.1.79 ec:2.7.1.23 ec:1.2.1.16 ec:1.18.1.3 ec:1.2.1.3 ec:1.2.1.20 |
| cpd:C00354 |  | D-Fructose 1,6-bisphosphate | 9 | ec:3.1.3.11 |
| cpd:C05259 |  | 3-Oxopalmitoyl-CoA; 3-Ketopalmitoyl-CoA; 3-Oxohexadecanoyl-CoA | 8 | ec:5.1.2.3 ec:2.3.1.16 ec:4.2.1.17 ec:1.1.1.35 |
| cpd:C00224 |  | Adenylyl sulfate; Adenosine 5'-phosphosulfate; APS; 5'-Adenylyl sulfate | 8 | ec:2.7.1.25 |
| cpd:C03344 |  | 2-Methylacetoacetyl-CoA; 2-Methyl-3-acetoacetyl-CoA | 8 | ec:5.1.2.3 ec:2.3.1.16 ec:4.2.1.17 ec:1.1.1.35 |
| cpd:C01586 |  | Hippurate; Hippuric acid; N-Benzoylglycine; Benzoylaminoacetic acid | 8 | ec:3.5.1.32 |
| cpd:C16471 |  | 5-Methyl-3-oxo-4-hexenoyl-CoA | 8 | ec:5.1.2.3 ec:2.3.1.16 ec:4.2.1.17 ec:1.1.1.35 |
| cpd:C02232 |  | 3-Oxoadipyl-CoA; beta-Ketoadipyl-CoA | 8 | ec:5.1.2.3 ec:2.3.1.16 ec:4.2.1.17 ec:1.1.1.35 |
| cpd:C00064 |  | L-Glutamine; L-2-Aminoglutaramic acid | 8 | ec:6.3.1.2 |
| cpd:C00053 |  | 3'-Phosphoadenylyl sulfate; 3'-Phosphoadenosine 5'-phosphosulfate; 3'-Phospho-5'-adenylyl sulfate; PAPS | 8 | ec:2.7.1.25 |
| cpd:C00051 |  | Glutathione; 5-L-Glutamyl-L-cysteinylglycine; N-(N-gamma-L-Glutamyl-L-cysteinyl)glycine; gamma-L-Glutamyl-L-cysteinyl-glycine; GSH; Reduced glutathione | 8 | ec:2.5.1.18 ec:4.4.1.5 |
| cpd:C00037 |  | Glycine; Aminoacetic acid; Gly | 8 | ec:3.5.1.32 |
| cpd:C05696 |  | 3'-Phosphoadenylylselenate; 3'-Phosphoadenosine-5'-phosphoselenate | 8 | ec:2.7.1.25 |
| cpd:C05686 |  | Adenylylselenate; Adenosine-5'-phosphoselenate | 8 | ec:2.7.1.25 |
| cpd:C05269 |  | 3-Oxohexanoyl-CoA; 3-Ketohexanoyl-CoA | 8 | ec:5.1.2.3 ec:2.3.1.16 ec:4.2.1.17 ec:1.1.1.35 |
| cpd:C05267 |  | 3-Oxooctanoyl-CoA | 8 | ec:5.1.2.3 ec:2.3.1.16 ec:4.2.1.17 ec:1.1.1.35 |
| cpd:C05263 |  | 3-Oxododecanoyl-CoA | 8 | ec:5.1.2.3 ec:2.3.1.16 ec:4.2.1.17 ec:1.1.1.35 |
| cpd:C05261 |  | 3-Oxotetradecanoyl-CoA | 8 | ec:5.1.2.3 ec:2.3.1.16 ec:4.2.1.17 ec:1.1.1.35 |
| cpd:C03460 |  | 2-Methylprop-2-enoyl-CoA; Methacrylyl-CoA; Methylacrylyl-CoA | 7 | ec:1.3.8.7 ec:5.1.2.3 ec:4.2.1.17 ec:1.1.1.35 |
| cpd:C03345 |  | 2-Methylbut-2-enoyl-CoA; trans-2-Methylbut-2-enoyl-CoA; Tiglyl-CoA; (E)-2-Methylcrotonoyl-CoA; Methylcrotonoyl-CoA; Methylcrotonyl-CoA; Tigloyl-CoA; 2-Methylcrotanoyl-CoA | 7 | ec:1.3.8.7 ec:5.1.2.3 ec:4.2.1.17 ec:1.1.1.35 |
| cpd:C00894 |  | Propenoyl-CoA; Acryloyl-CoA; Acrylyl-CoA | 7 | ec:1.3.8.7 ec:5.1.2.3 ec:4.2.1.17 ec:1.1.1.35 |
| cpd:C00877 |  | Crotonoyl-CoA; Crotonyl-CoA; 2-Butenoyl-CoA; trans-But-2-enoyl-CoA; But-2-enoyl-CoA; (E)-But-2-enoyl-CoA | 7 | ec:1.3.8.7 ec:5.1.2.3 ec:4.2.1.17 ec:1.1.1.35 |
| cpd:C03221 |  | 2-trans-Dodecenoyl-CoA; (2E)-Dodec-2-enoyl-CoA; (2E)-Dodecenoyl-CoA | 7 | ec:1.3.8.7 ec:5.1.2.3 ec:4.2.1.17 ec:1.1.1.35 |
| cpd:C00021 |  | S-Adenosyl-L-homocysteine; S-Adenosylhomocysteine | 7 | ec:2.1.1.181 ec:2.1.1.197 |
| cpd:C05276 |  | trans-Oct-2-enoyl-CoA; (2E)-Octenoyl-CoA | 7 | ec:1.3.8.7 ec:5.1.2.3 ec:4.2.1.17 ec:1.1.1.35 |
| cpd:C05275 |  | trans-Dec-2-enoyl-CoA; (2E)-Decenoyl-CoA | 7 | ec:1.3.8.7 ec:5.1.2.3 ec:4.2.1.17 ec:1.1.1.35 |
| cpd:C05273 |  | trans-Tetradec-2-enoyl-CoA; (2E)-Tetradecenoyl-CoA | 7 | ec:1.3.8.7 ec:5.1.2.3 ec:4.2.1.17 ec:1.1.1.35 |
| cpd:C05272 |  | trans-Hexadec-2-enoyl-CoA; trans-2-Hexadecenoyl-CoA; (2E)-Hexadecenoyl-CoA | 7 | ec:1.3.8.7 ec:5.1.2.3 ec:4.2.1.17 ec:1.1.1.35 |
| cpd:C05271 |  | trans-Hex-2-enoyl-CoA; (2E)-Hexenoyl-CoA | 7 | ec:1.3.8.7 ec:5.1.2.3 ec:4.2.1.17 ec:1.1.1.35 |
| cpd:C05258 |  | (S)-3-Hydroxyhexadecanoyl-CoA | 6 | ec:5.1.2.3 ec:4.2.1.17 ec:1.1.1.35 |
| cpd:C00340 |  | Reduced rubredoxin | 6 | ec:1.14.15.3 ec:1.18.1.3 |
| cpd:C05998 |  | 3-Hydroxyisovaleryl-CoA; 3-Hydroxyisovaleryl coenzyme A | 6 | ec:5.1.2.3 ec:4.2.1.17 ec:1.1.1.35 |
| cpd:C14145 |  | (3S)-3-Hydroxyadipyl-CoA | 6 | ec:5.1.2.3 ec:4.2.1.17 ec:1.1.1.35 |
| cpd:C14144 |  | 5-Carboxy-2-pentenoyl-CoA; 2,3-Dehydroadipyl-CoA | 6 | ec:5.1.2.3 ec:4.2.1.17 ec:1.1.1.35 |
| cpd:C00226 |  | Primary alcohol; 1-Alcohol | 6 | ec:1.14.15.3 ec:1.1.1.284 ec:1.1.1.1 |
| cpd:C00162 |  | Fatty acid | 6 | ec:1.14.15.3 ec:1.2.1.3 |
| cpd:C04405 |  | (2S,3S)-3-Hydroxy-2-methylbutanoyl-CoA; (S)-3-Hydroxy-2-methylbutyryl-CoA | 6 | ec:5.1.2.3 ec:4.2.1.17 ec:1.1.1.35 |
| cpd:C16469 |  | 3-Hydroxy-5-methylhex-4-enoyl-CoA | 6 | ec:5.1.2.3 ec:4.2.1.17 ec:1.1.1.35 |
| cpd:C16468 |  | (2E)-5-Methylhexa-2,4-dienoyl-CoA | 6 | ec:5.1.2.3 ec:4.2.1.17 ec:1.1.1.35 |
| cpd:C00132 |  | Methanol; Methyl alcohol; CH3OH | 6 | ec:3.1.1.11 ec:1.11.1.6 |
| cpd:C11947 |  | 3-Hydroxy-2,6-dimethyl-5-methylene-heptanoyl-CoA | 6 | ec:5.1.2.3 ec:4.2.1.17 ec:1.1.1.35 |
| cpd:C11946 |  | cis-2-Methyl-5-isopropylhexa-2,5-dienoyl-CoA | 6 | ec:5.1.2.3 ec:4.2.1.17 ec:1.1.1.35 |
| cpd:C11945 |  | trans-2-Methyl-5-isopropylhexa-2,5-dienoyl-CoA | 6 | ec:5.1.2.3 ec:4.2.1.17 ec:1.1.1.35 |
| cpd:C01144 |  | (S)-3-Hydroxybutanoyl-CoA; (S)-3-Hydroxybutyryl-CoA | 6 | ec:5.1.2.3 ec:4.2.1.17 ec:1.1.1.35 |
| cpd:C00435 |  | Oxidized rubredoxin | 6 | ec:1.14.15.3 ec:1.18.1.3 |
| cpd:C06000 |  | (S)-3-Hydroxyisobutyryl-CoA | 6 | ec:5.1.2.3 ec:4.2.1.17 ec:1.1.1.35 |
| cpd:C00369 |  | Starch | 6 | ec:2.4.1.1 |
| cpd:C05668 |  | 3-Hydroxypropionyl-CoA; 3-Hydroxypropionyl coenzyme A; 3-Hydroxypropanoyl-CoA; 3-Hydroxypropanoyl coenzymeA; beta-Hydroxypropionyl-CoA | 6 | ec:5.1.2.3 ec:4.2.1.17 ec:1.1.1.35 |
| cpd:C05268 |  | (S)-Hydroxyhexanoyl-CoA; (S)-3-Hydroxyhexanoyl-CoA | 6 | ec:5.1.2.3 ec:4.2.1.17 ec:1.1.1.35 |
| cpd:C05266 |  | (S)-3-Hydroxyoctanoyl-CoA; (S)-3-Hydroxycapryloyl-CoA; (S)-Hydroxyoctanoyl-CoA | 6 | ec:5.1.2.3 ec:4.2.1.17 ec:1.1.1.35 |
| cpd:C05264 |  | (S)-Hydroxydecanoyl-CoA; (S)-3-Hydroxydecanoyl-CoA | 6 | ec:5.1.2.3 ec:4.2.1.17 ec:1.1.1.35 |
| cpd:C05262 |  | (S)-3-Hydroxydodecanoyl-CoA | 6 | ec:5.1.2.3 ec:4.2.1.17 ec:1.1.1.35 |
| cpd:C05260 |  | (S)-3-Hydroxytetradecanoyl-CoA | 6 | ec:5.1.2.3 ec:4.2.1.17 ec:1.1.1.35 |
| cpd:C01371 |  | Alkane; RH | 5 | ec:1.14.15.3 |
| cpd:C00283 |  | Hydrogen sulfide; Hydrogen-sulfide; H2S; Sulfide | 5 | ec:4.4.1.15 |
| cpd:C05198 |  | 5'-Deoxyadenosine | 5 | ec:2.8.1.6 |
| cpd:C19845 |  | Pimeloyl-[acyl-carrier protein]; Pimeloyl-[acp]; Pimelyl-[acyl-carrier protein]; Pimelyl-[acp]; 7-Hydroxy-7-oxoheptanoyl-[acyl-carrier protein] | 5 | ec:2.3.1.47 |
| cpd:C00718 |  | Amylose; Amylose chain; (1,4-alpha-D-Glucosyl)n; (1,4-alpha-D-Glucosyl)n+1; (1,4-alpha-D-Glucosyl)n-1; 4-{(1,4)-alpha-D-Glucosyl}(n-1)-D-glucose; 1,4-alpha-D-Glucan | 5 | ec:2.4.1.1 |
| cpd:C00714 |  | Pectin; Poly(1,4-alpha-D-galacturonide) | 5 | ec:3.1.1.11 |
| cpd:C03024 |  | Reduced flavoprotein | 5 | ec:1.14.15.3 |
| cpd:C14818 |  | Fe2+; Fe(II); Ferrous ion; Iron(2+) | 5 | ec:4.99.1.1 |
| cpd:C00555 |  | 4-Aminobutyraldehyde; 4-Aminobutanal | 5 | ec:1.2.1.3 |
| cpd:C04425 |  | S-Adenosyl-4-methylthio-2-oxobutanoate | 5 | ec:2.6.1.62 |
| cpd:C19673 |  | Malonyl-[acp] methyl ester; Malonyl-[acyl-carrier protein] methyl ester | 5 | ec:2.1.1.197 |
| cpd:C00148 |  | L-Proline; 2-Pyrrolidinecarboxylic acid | 5 | ec:3.4.11.5 ec:4.3.1.12 |
| cpd:C00136 |  | Butanoyl-CoA; Butyryl-CoA | 5 | ec:1.3.8.7 ec:2.3.1.16 |
| cpd:C00120 |  | Biotin; D-Biotin; Vitamin H; Coenzyme R | 5 | ec:2.8.1.6 |
| cpd:C02593 |  | Tetradecanoyl-CoA; Myristoyl-CoA | 5 | ec:1.3.8.7 ec:2.3.1.16 |
| cpd:C02191 |  | Protoporphyrin; Protoporphyrin IX; Porphyrinogen IX | 5 | ec:4.99.1.1 |
| cpd:C00470 |  | Pectate; Pectic acid; alpha-D-Polygalacturonic acid; Poly(1,4-alpha-D-galacturonate); Poly(1,4-alpha-D-galacturonate)(n); (1,4-alpha-D-Galacturonide)n; (1,4-alpha-D-Galacturonosyl)n; De-esterified pectin | 5 | ec:3.1.1.11 |
| cpd:C00073 |  | L-Methionine; Methionine; L-2-Amino-4methylthiobutyric acid | 5 | ec:2.8.1.6 |
| cpd:C01944 |  | Octanoyl-CoA | 5 | ec:1.3.8.7 ec:2.3.1.16 |
| cpd:C00905 |  | D-Fructuronate; D-Fructuronic acid | 5 | ec:5.3.1.12 ec:1.1.1.57 |
| cpd:C00103 |  | D-Glucose 1-phosphate; alpha-D-Glucose 1-phosphate; Cori ester; D-Glucose alpha-1-phosphate | 5 | ec:2.4.1.1 |
| cpd:C00100 |  | Propanoyl-CoA; Propionyl-CoA; Propionyl coenzyme A | 5 | ec:1.3.8.7 ec:2.3.1.16 |
| cpd:C05359 |  | e-; Electron | 5 | ec:2.8.1.6 |
| cpd:C00793 |  | D-Cysteine; D-Amino-3-mercaptopropionic acid | 5 | ec:4.4.1.15 |
| cpd:C00032 |  | Heme; Haem; Protoheme; Heme B; Protoheme IX | 5 | ec:4.99.1.1 |
| cpd:C01063 |  | 6-Carboxyhexanoyl-CoA; Pimeloyl-CoA | 5 | ec:2.3.1.47 |
| cpd:C06002 |  | (S)-Methylmalonate semialdehyde | 5 | ec:5.1.2.3 ec:4.2.1.17 ec:1.2.1.3 ec:1.1.1.35 |
| cpd:C17023 |  | Sulfur donor; S-donor | 5 | ec:2.8.1.6 |
| cpd:C03161 |  | Oxidized flavoprotein | 5 | ec:1.14.15.3 |
| cpd:C05274 |  | Decanoyl-CoA | 5 | ec:1.3.8.7 ec:2.3.1.16 |
| cpd:C05270 |  | Hexanoyl-CoA | 5 | ec:1.3.8.7 ec:2.3.1.16 |
| cpd:C01832 |  | Lauroyl-CoA; Lauroyl coenzyme A; Dodecanoyl-CoA | 5 | ec:1.3.8.7 ec:2.3.1.16 |
| cpd:C03547 |  | omega-Hydroxy fatty acid | 5 | ec:1.14.15.3 |
| cpd:C00332 |  | Acetoacetyl-CoA; Acetoacetyl coenzyme A; 3-Acetoacetyl-CoA | 4 | ec:5.1.2.3 ec:4.2.1.17 ec:1.1.1.35 |
| cpd:C14120 |  | 2-Naphthoyl-CoA | 4 |  |
| cpd:C05172 |  | Selenophosphoric acid; Selenophosphate | 4 | ec:2.9.1.1 |
| cpd:C03451 |  | (R)-S-Lactoylglutathione; S-D-Lactoylglutathione | 4 | ec:4.4.1.5 |
| cpd:C00251 |  | Chorismate; Chorismic acid | 4 | ec:4.2.3.5 |
| cpd:C14101 |  | 2-Naphthoic acid; 2-Naphthalenecarboxylic acid; beta-Naphthoic acid | 4 |  |
| cpd:C01322 |  | RX; Organic halide | 4 | ec:2.5.1.18 |
| cpd:C01269 |  | 5-O-(1-Carboxyvinyl)-3-phosphoshikimate; O5-(1-Carboxyvinyl)-3-phosphoshikimate | 4 | ec:4.2.3.5 |
| cpd:C02320 |  | R-S-Glutathione | 4 | ec:2.5.1.18 |
| cpd:C00546 |  | Methylglyoxal; Pyruvaldehyde; Pyruvic aldehyde; 2-Ketopropionaldehyde; 2-Oxopropanal | 4 | ec:4.4.1.5 |
| cpd:C16470 |  | 5-Methylhex-4-enoyl-CoA | 4 | ec:2.3.1.16 |
| cpd:C06482 |  | L-Selenocysteinyl-tRNA(Sec) | 4 | ec:2.9.1.1 |
| cpd:C06481 |  | L-Seryl-tRNA(Sec) | 4 | ec:2.9.1.1 |
| cpd:C00134 |  | Putrescine; 1,4-Butanediamine; 1,4-Diaminobutane; Tetramethylenediamine; Butane-1,4-diamine | 4 |  |
| cpd:C16466 |  | 7-Methyl-3-oxo-6-octenoyl-CoA | 4 | ec:2.3.1.16 |
| cpd:C16465 |  | trans-Geranyl-CoA | 4 |  |
| cpd:C16461 |  | Geranic acid; 3,7-Dimethylocta-2,6-dienoate; Geranate | 4 |  |
| cpd:C00126 |  | Ferrocytochrome c; Cytochrome c2+; Reduced cytochrome c | 4 | ec:1.9.3.1 ec:1.7.2.5 |
| cpd:C00125 |  | Ferricytochrome c; Cytochrome c3+ | 4 | ec:1.9.3.1 ec:1.7.2.5 |
| cpd:C00514 |  | D-Mannonate | 4 | ec:1.1.1.57 |
| cpd:C00512 |  | S-Benzoate coenzyme A; Benzoyl-CoA | 4 | ec:2.3.1.16 |
| cpd:C00462 |  | Halide; Hydrogen halide; HX; Halo acid | 4 | ec:2.5.1.18 |
| cpd:C00055 |  | CMP; Cytidine-5'-monophosphate; Cytidylic acid | 4 | ec:2.7.8.5 ec:3.1.3.5 |
| cpd:C16388 |  | (6Z,9Z,12Z,15Z,18Z)-3-Hydroxytetracosapenta-6,9,12,15,18-enoyl-CoA | 4 | ec:5.1.2.3 ec:4.2.1.17 ec:1.1.1.35 |
| cpd:C16387 |  | (2E,6Z,9Z,12Z,15Z,18Z)-Tetracosahexa-2,6,9,12,15,18-enoyl-CoA | 4 | ec:5.1.2.3 ec:4.2.1.17 ec:1.1.1.35 |
| cpd:C07118 |  | Benzoyl acetyl-CoA; Benzoyl acetyl coenzyme A | 4 | ec:2.3.1.16 |
| cpd:C05337 |  | Chenodeoxycholoyl-CoA; 3alpha,7alpha-Dihydroxy-5beta-cholanoyl-CoA | 4 | ec:2.3.1.16 |
| cpd:C04644 |  | 3alpha,7alpha-Dihydroxy-5beta-cholestanoyl-CoA | 4 | ec:2.3.1.16 |
| cpd:C06001 |  | (S)-3-Hydroxyisobutyrate | 4 | ec:5.1.2.3 ec:4.2.1.17 ec:1.1.1.35 |
| cpd:C03561 |  | (R)-3-Hydroxybutanoyl-CoA; (3R)-3-Hydroxybutanoyl-CoA | 4 | ec:5.1.2.3 ec:4.2.1.17 ec:1.1.1.35 |
| cpd:C00005 |  | NADPH; TPNH; Reduced nicotinamide adenine dinucleotide phosphate | 4 | ec:1.2.1.79 ec:1.2.1.16 ec:1.18.1.3 ec:1.2.1.3 ec:1.2.1.20 |
| cpd:C16339 |  | (+)-7-Isojasmonic acid CoA | 4 | ec:2.3.1.16 |
| cpd:C16338 |  | 3-Oxo-OPC4-CoA | 4 | ec:2.3.1.16 |
| cpd:C16335 |  | OPC4-CoA | 4 | ec:2.3.1.16 |
| cpd:C16334 |  | 3-Oxo-OPC6-CoA | 4 | ec:2.3.1.16 |
| cpd:C16331 |  | OPC6-CoA | 4 | ec:2.3.1.16 |
| cpd:C16330 |  | 3-Oxo-OPC8-CoA | 4 | ec:2.3.1.16 |
| cpd:C05265 |  | 3-Oxodecanoyl-CoA | 4 | ec:2.3.1.16 |
| cpd:C00345 |  | 6-Phospho-D-gluconate | 3 | ec:3.1.1.31 |
| cpd:C03892 |  | Phosphatidylglycerophosphate; 3(3-sn-Phosphatidyl)-sn-glycerol 1-phosphate; 3(3-Phosphatidyl-)L-glycerol 1-phosphate; 1,2-Diacyl-sn-glycero-3-phospho-sn-glycerol 3'-phosphate | 3 | ec:2.7.8.5 |
| cpd:C00314 |  | Pyridoxine; Pyridoxol | 3 | ec:2.7.1.35 |
| cpd:C00269 |  | CDP-diacylglycerol; CDP-1,2-diacylglycerol; 1,2-Diacyl-sn-glycero-3-cytidine-5'-diphosphate | 3 | ec:2.7.8.5 |
| cpd:C00250 |  | Pyridoxal | 3 | ec:2.7.1.35 |
| cpd:C00647 |  | Pyridoxamine phosphate; Pyridoxamine 5-phosphate; Pyridoxamine 5'-phosphate | 3 | ec:2.7.1.35 |
| cpd:C00627 |  | Pyridoxine phosphate; Pyridoxine 5-phosphate; Pyridoxine 5'-phosphate; Pyridoxol 5'-phosphate | 3 | ec:2.7.1.35 |
| cpd:C00979 |  | O-Acetyl-L-serine; O3-Acetyl-L-serine | 3 | ec:2.3.1.30 |
| cpd:C00954 |  | Indole-3-acetate; Indole-3-acetic acid; (Indol-3-yl)acetate; Indoleacetate; Indoleacetic acid; IAA | 3 | ec:3.5.5.1 ec:1.2.1.3 |
| cpd:C01236 |  | D-Glucono-1,5-lactone 6-phosphate; 6-Phospho-D-glucono-1,5-lactone | 3 | ec:3.1.1.31 |
| cpd:C00154 |  | Palmitoyl-CoA; Hexadecanoyl-CoA | 3 | ec:1.3.8.7 ec:6.2.1.3 |
| cpd:C00534 |  | Pyridoxamine; PM | 3 | ec:2.7.1.35 |
| cpd:C00533 |  | Nitric oxide; NO; Nitrogen monoxide | 3 | ec:1.7.2.5 |
| cpd:C00093 |  | sn-Glycerol 3-phosphate; Glycerophosphoric acid; D-Glycerol 1-phosphate | 3 | ec:2.7.8.5 |
| cpd:C00887 |  | Nitrous oxide; Dinitrogen monoxide; Dinitrogen oxide; N2O | 3 | ec:1.7.2.5 |
| cpd:C00084 |  | Acetaldehyde; Ethanal | 3 | ec:1.13.12.16 ec:1.2.1.3 ec:1.1.1.284 ec:1.1.1.1 |
| cpd:C00065 |  | L-Serine; L-2-Amino-3-hydroxypropionic acid; L-3-Hydroxy-alanine; Serine | 3 | ec:2.3.1.30 |
| cpd:C00048 |  | Glyoxylate; Glyoxalate; Glyoxylic acid | 3 | ec:1.18.1.3 ec:1.1.3.15 |
| cpd:C00027 |  | Hydrogen peroxide; H2O2; Oxydol | 3 | ec:1.11.1.6 ec:1.1.3.15 |
| cpd:C00018 |  | Pyridoxal phosphate; Pyridoxal 5-phosphate; Pyridoxal 5'-phosphate; PLP | 3 | ec:2.7.1.35 |
| cpd:C00012 |  | Peptide | 3 | ec:3.4.11.5 |
| cpd:C00330 |  | Deoxyguanosine; 2'-Deoxyguanosine | 2 | ec:2.4.2.1 ec:3.1.3.5 |
| cpd:C00294 |  | Inosine | 2 | ec:2.4.2.1 ec:3.1.3.5 |
| cpd:C00726 |  | Nitrile; R-CN | 2 | ec:3.5.5.1 |
| cpd:C01762 |  | Xanthosine | 2 | ec:2.4.2.1 ec:3.1.3.5 |
| cpd:C01401 |  | Alanine; 2-Aminopropionic acid; 2-Aminopropanoic acid | 2 | ec:3.5.5.1 |
| cpd:C04154 |  | rRNA containing N6-methyladenine; rRNA(N6-methyladenine) | 2 | ec:2.1.1.181 |
| cpd:C00302 |  | DL-Glutamate; DL-Glutaminic acid; 2-Aminoglutaric acid; Glutamate; Glutamic acid | 2 | ec:3.5.5.1 |
| cpd:C00267 |  | alpha-D-Glucose | 2 | ec:2.7.1.2 ec:3.2.1.21 |
| cpd:C00249 |  | Hexadecanoic acid; Hexadecanoate; Hexadecylic acid; Palmitic acid; Palmitate; Cetylic acid | 2 | ec:6.2.1.3 |
| cpd:C00240 |  | rRNA; Ribosomal RNA | 2 | ec:2.1.1.181 |
| cpd:C01278 |  | 2-Carboxy-2,5-dihydro-5-oxofuran-2-acetate; 5-Carboxy-2,5-dihydro-2-oxofuran-5-acetate; 4-Carboxymuconolactone; gamma-Carboxymuconolactone | 2 | ec:4.1.1.44 |
| cpd:C00239 |  | dCMP; Deoxycytidylic acid; Deoxycytidine monophosphate; Deoxycytidylate; 2'-Deoxycytidine 5'-monophosphate | 2 | ec:3.5.4.12 ec:3.1.3.5 |
| cpd:C09814 |  | Benzonitrile; Phenyl cyanide; Cyanobenzene | 2 | ec:3.5.5.1 |
| cpd:C06613 |  | trans-3-Chloroallyl aldehyde; trans-3-Chloro-2-propenal | 2 | ec:1.2.1.3 ec:1.1.1.284 ec:1.1.1.1 |
| cpd:C00221 |  | beta-D-Glucose | 2 | ec:2.7.1.2 ec:3.2.1.21 |
| cpd:C00212 |  | Adenosine | 2 | ec:2.4.2.1 ec:3.1.3.5 |
| cpd:C05116 |  | 3-Hydroxybutanoyl-CoA; 3-Hydroxybutyryl-CoA | 2 | ec:4.2.1.17 |
| cpd:C00160 |  | Glycolate; Glycolic acid; Hydroxyacetic acid | 2 | ec:1.1.3.15 |
| cpd:C00559 |  | Deoxyadenosine; 2'-Deoxyadenosine | 2 | ec:2.4.2.1 ec:3.1.3.5 |
| cpd:C16074 |  | Phenylacetonitrile; Benzyl cyanide | 2 | ec:3.5.5.1 |
| cpd:C05841 |  | Nicotinate D-ribonucleoside; beta-D-Ribosylnicotinate | 2 | ec:2.4.2.1 ec:3.1.3.5 |
| cpd:C00077 |  | L-Ornithine; (S)-2,5-Diaminovaleric acid; (S)-2,5-Diaminopentanoic acid; (S)-2,5-Diaminopentanoate | 2 | ec:4.3.1.12 |
| cpd:C00071 |  | Aldehyde; RCHO | 2 | ec:1.2.1.3 ec:1.1.1.284 ec:1.1.1.1 |
| cpd:C16399 |  | 2,4-Diamino-6-hydroxylaminotoluene | 2 | ec:1.12.99.6 |
| cpd:C16396 |  | 2,4-Diamino-6-nitrotoluene | 2 | ec:1.12.99.6 |
| cpd:C00060 |  | Carboxylate; R-COOH; Monocarboxylate; Carboxylic acid | 2 | ec:3.5.5.1 |
| cpd:C07086 |  | Phenylacetic acid; Benzylformic acid; Phenylacetate; Benzeneacetic acid | 2 | ec:3.5.5.1 |
| cpd:C00031 |  | D-Glucose; Grape sugar; Dextrose; Glucose; D-Glucopyranose | 2 | ec:2.7.1.2 ec:3.2.1.21 |
| cpd:C03586 |  | 2-Oxo-2,3-dihydrofuran-5-acetate; 3-Oxoadipate enol-lactone; 4,5-Dihydro-5-oxofuran-2-acetate; 5-Oxo-4,5-dihydrofuran-2-acetate | 2 | ec:4.1.1.44 |
| cpd:C00387 |  | Guanosine | 2 | ec:2.4.2.1 ec:3.1.3.5 |
| cpd:C02938 |  | 3-Indoleacetonitrile; Indol-3-ylacetonitrile; Indole-3-acetonitrile; (Indol-3-yl)acetonitrile | 2 | ec:3.5.5.1 |
| cpd:C16348 |  | cis-3-Chloroallyl aldehyde; cis-3-Chloro-2-propenal | 2 | ec:1.2.1.3 ec:1.1.1.284 ec:1.1.1.1 |
| cpd:C05715 |  | gamma-Amino-gamma-cyanobutanoate; 4-Amino-4-cyanobutanoic acid | 2 | ec:3.5.5.1 |
| cpd:C05714 |  | alpha-Aminopropiononitrile | 2 | ec:3.5.5.1 |
| cpd:C03150 |  | Nicotinamide-beta-riboside; N-Ribosylnicotinamide; 1-(beta-D-Ribofuranosyl)nicotinamide | 2 | ec:2.4.2.1 ec:3.1.3.5 |
| cpd:C04570 |  | Reduced electron-transferring flavoprotein; Reduced electron-transfer flavoprotein | 1 | ec:1.3.8.7 |
| cpd:C06727 |  | cis-1,2-Dihydro-3-ethylcatechol; cis-2,3-Dihydroxy-2,3-dihydroethylbenzene; cis-3-Ethyl-cyclohexa-3,5-diene-1,2-diol | 1 | ec:1.18.1.3 |
| cpd:C00334 |  | 4-Aminobutanoate; 4-Aminobutanoic acid; 4-Aminobutyrate; 4-Aminobutyric acid; gamma-Aminobutyric acid; GABA | 1 | ec:1.2.1.3 |
| cpd:C05640 |  | Cinnavalininate; Cinnabarinic acid | 1 | ec:1.11.1.6 |
| cpd:C00333 |  | D-Galacturonate; D-Galacturonic acid | 1 | ec:5.3.1.12 |
| cpd:C00299 |  | Uridine | 1 | ec:3.1.3.5 |
| cpd:C15980 |  | (S)-2-Methylbutanoyl-CoA | 1 | ec:1.3.8.7 |
| cpd:C15587 |  | Purine; Purine base | 1 | ec:2.4.2.1 |
| cpd:C15586 |  | N-D-Ribosylpurine; Purine nucleoside; Purine ribonucleoside | 1 | ec:2.4.2.1 |
| cpd:C06718 |  | S-Formylmycothiol | 1 | ec:1.1.1.306 |
| cpd:C06717 |  | Mycothiol | 1 | ec:1.1.1.306 |
| cpd:C01407 |  | Benzene | 1 | ec:1.18.1.3 |
| cpd:C05635 |  | 5-Hydroxyindoleacetate | 1 | ec:1.2.1.3 |
| cpd:C05634 |  | 5-Hydroxyindoleacetaldehyde | 1 | ec:1.2.1.3 |
| cpd:C04554 |  | 3alpha,7alpha-Dihydroxy-5beta-cholestanate; 3alpha,7alpha-Dihydroxy-5beta-cholestanoate | 1 | ec:1.2.1.3 |
| cpd:C02835 |  | Imidazole-4-acetate; Imidazoleacetic acid; 4-Imidazoleacetate | 1 | ec:1.2.1.3 |
| cpd:C05629 |  | Phenylpropanoate; 3-Phenyl-propionic acid; 3-Phenylpropanoic acid; 3-Phenylpropionic acid | 1 | ec:1.18.1.3 |
| cpd:C05985 |  | 2-Propynal; 2-Propyn-1-al; Propiolaldehyde | 1 | ec:1.2.1.3 |
| cpd:C14099 |  | 2-Naphthaldehyde; 2-Naphthalenecarboxaldehyde | 1 | ec:1.1.1.284 ec:1.1.1.1 |
| cpd:C01352 |  | FADH2 | 1 | ec:1.3.8.7 |
| cpd:C00672 |  | 2-Deoxy-D-ribose 1-phosphate; 2-Deoxy-alpha-D-ribose 1-phosphate | 1 | ec:2.4.2.1 |
| cpd:C00311 |  | Isocitrate; Isocitric acid; 1-Hydroxytricarballylic acid; 1-Hydroxypropane-1,2,3-tricarboxylic acid | 1 | ec:4.2.1.3 |
| cpd:C14090 |  | 1-Naphthaldehyde; 1-Formylnaphthalene | 1 | ec:1.1.1.284 ec:1.1.1.1 |
| cpd:C00704 |  | O2.-; Superoxide anion; O2- | 1 | ec:1.11.1.6 |
| cpd:C00668 |  | alpha-D-Glucose 6-phosphate | 1 | ec:2.7.1.2 |
| cpd:C14089 |  | 1-Hydroxymethylnaphthalene; 1-Naphthalenemethanol | 1 | ec:1.1.1.284 ec:1.1.1.1 |
| cpd:C05577 |  | 3,4-Dihydroxymandelaldehyde; 3,4-Dihydroxyphenylglycolaldehyde | 1 | ec:1.1.1.284 ec:1.1.1.1 |
| cpd:C05576 |  | 3,4-Dihydroxyphenylethyleneglycol | 1 | ec:1.1.1.284 ec:1.1.1.1 |
| cpd:C00262 |  | Hypoxanthine; Purine-6-ol | 1 | ec:2.4.2.1 |
| cpd:C16596 |  | 5-Phenyl-1,3-oxazinane-2,4-dione | 1 | ec:1.1.1.284 ec:1.1.1.1 |
| cpd:C16595 |  | 4-Hydroxy-5-phenyltetrahydro-1,3-oxazin-2-one | 1 | ec:1.1.1.284 ec:1.1.1.1 |
| cpd:C04091 |  | cis-1,2-Dihydrobenzene-1,2-diol; cis-Benzeneglycol; cis-Cyclohexa-3,5-diene-1,2-diol | 1 | ec:1.18.1.3 |
| cpd:C01335 |  | ROH | 1 | ec:3.2.1.21 |
| cpd:C00655 |  | Xanthosine 5'-phosphate; Xanthylic acid; XMP; (9-D-Ribosylxanthine)-5'-phosphate | 1 | ec:3.1.3.5 |
| cpd:C00258 |  | D-Glycerate; Glycerate; (R)-Glycerate; Glyceric acid | 1 | ec:1.2.1.3 |
| cpd:C00253 |  | Nicotinate; Nicotinic acid; Niacin; 3-Pyridinecarboxylic acid | 1 | ec:2.4.2.1 |
| cpd:C16587 |  | 3-Carbamoyl-2-phenylpropionaldehyde | 1 | ec:1.1.1.284 ec:1.1.1.1 |
| cpd:C16586 |  | 2-Phenyl-1,3-propanediol monocarbamate | 1 | ec:1.1.1.284 ec:1.1.1.1 |
| cpd:C04122 |  | D-1-Aminopropan-2-ol O-phosphate; (R)-1-Aminopropan-2-yl phosphate | 1 | ec:6.3.1.10 |
| cpd:C16186 |  | L-Ascorbate 6-phosphate | 1 |  |
| cpd:C01327 |  | Hydrochloric acid; HCl; Hydrogen chloride; Hydrochloride | 1 | ec:1.18.1.3 |
| cpd:C00242 |  | Guanine; 2-Amino-6-hydroxypurine | 1 | ec:2.4.2.1 |
| cpd:C00637 |  | Indole-3-acetaldehyde; 2-(Indol-3-yl)acetaldehyde; Indoleacetaldehyde | 1 | ec:1.2.1.3 |
| cpd:C06589 |  | cis-2,3-Dihydro-2,3-dihydroxybiphenyl; cis-3-Phenylcyclohexa-3,5-diene-1,2-diol; (1S,2R)-3-Phenylcyclohexa-3,5-diene-1,2-diol | 1 | ec:1.18.1.3 |
| cpd:C06588 |  | Biphenyl; Phenylbenzene; 1,1'-Biphenyl; 1,1'-Diphenyl | 1 | ec:1.18.1.3 |
| cpd:C00632 |  | 3-Hydroxyanthranilate; 3-Hydroxyanthranilic acid | 1 | ec:1.11.1.6 |
| cpd:C06585 |  | cis-2,3-Dihydro-2,3-dihydroxy-4'-chlorobiphenyl | 1 | ec:1.18.1.3 |
| cpd:C06584 |  | 4-Chlorobiphenyl; 1-Chloro-4-phenyl benzene; 4-Monochloro-biphenyl | 1 | ec:1.18.1.3 |
| cpd:C00630 |  | 2-Methylpropanoyl-CoA; 2-Methylpropionyl-CoA; Isobutyryl-CoA | 1 | ec:1.3.8.7 |
| cpd:C00233 |  | 4-Methyl-2-oxopentanoate; 2-Oxoisocaproate | 1 | ec:1.1.1.85 |
| cpd:C00232 |  | Succinate semialdehyde; Succinic semialdehyde; 4-Oxobutanoate | 1 | ec:1.2.1.79 ec:1.2.1.16 ec:1.2.1.20 |
| cpd:C05143 |  | Dhurrin; (S)-4-Hydroxymandelonitrile beta-D-glucoside | 1 | ec:3.2.1.21 |
| cpd:C00191 |  | D-Glucuronate; Glucuronic acid; Glucuronate | 1 | ec:5.3.1.12 |
| cpd:C08334 |  | Lotaustralin | 1 | ec:3.2.1.21 |
| cpd:C05936 |  | N4-Acetylaminobutanal; 4-Acetamidobutanal | 1 | ec:1.2.1.3 |
| cpd:C06615 |  | cis-3-Chloroacrylic acid | 1 | ec:1.2.1.3 |
| cpd:C06614 |  | trans-3-Chloroacrylic acid | 1 | ec:1.2.1.3 |
| cpd:C06612 |  | cis-3-Chloro-2-propene-1-ol; cis-3-Chloroallyl alcohol | 1 | ec:1.1.1.284 ec:1.1.1.1 |
| cpd:C06579 |  | cis-2,3-Dihydroxy-2,3-dihydro-p-cumate; cis-5,6-Dihydroxy-4-isopropylcyclohexa-1,3-dienecarboxylate | 1 | ec:1.18.1.3 |
| cpd:C06611 |  | trans-3-Chloro-2-propene-1-ol; trans-3-Chloroallyl alcohol | 1 | ec:1.1.1.284 ec:1.1.1.1 |
| cpd:C06578 |  | p-Cumate | 1 | ec:1.18.1.3 |
| cpd:C00620 |  | alpha-D-Ribose 1-phosphate; Ribose 1-phosphate; D-Ribose 1-phosphate | 1 | ec:2.4.2.1 |
| cpd:C01267 |  | 3-(Imidazol-4-yl)-2-oxopropyl phosphate; Imidazole-acetol phosphate | 1 | ec:2.6.1.9 |
| cpd:C00188 |  | L-Threonine; 2-Amino-3-hydroxybutyric acid | 1 | ec:6.1.1.3 |
| cpd:C16551 |  | Alcophosphamide | 1 | ec:1.1.1.284 ec:1.1.1.1 |
| cpd:C00185 |  | Cellobiose; 1-beta-D-Glucopyranosyl-4-D-glucopyranose | 1 | ec:3.2.1.21 |
| cpd:C05130 |  | Imidazole-4-acetaldehyde; Imidazole acetaldehyde | 1 | ec:1.2.1.3 |
| cpd:C08325 |  | Amygdalin; (R)-Amygdalin; (R)-Amygdaloside; (R)-Laenitrile | 1 | ec:3.2.1.21 |
| cpd:C07645 |  | Aldophosphamide | 1 | ec:1.1.1.284 ec:1.1.1.1 |
| cpd:C00615 |  | Protein histidine; Protein L-histidine; [Protein]-L-histidine | 1 |  |
| cpd:C01651 |  | tRNA(Thr) | 1 | ec:6.1.1.3 |
| cpd:C00577 |  | D-Glyceraldehyde | 1 | ec:1.2.1.3 |
| cpd:C00214 |  | Thymidine; Deoxythymidine | 1 | ec:3.1.3.5 |
| cpd:C16143 |  | (1E,4S,5E,7R)-Germacra-1(10),5-dien-11-ol; Germacradienol | 1 | ec:4.1.99.16 ec:4.2.3.75 ec:4.2.3.22 |
| cpd:C16142 |  | (-)-Germacrene D | 1 | ec:4.1.99.16 ec:4.2.3.75 ec:4.2.3.22 |
| cpd:C00966 |  | 2-Dehydropantoate | 1 | ec:2.1.2.11 |
| cpd:C00207 |  | Acetone; Dimethyl ketone; 2-Propanone | 1 | ec:4.1.99.16 ec:4.2.3.75 ec:4.2.3.22 |
| cpd:C05512 |  | Deoxyinosine | 1 | ec:2.4.2.1 |
| cpd:C11588 |  | cis-3-(Carboxy-ethyl)-3,5-cyclo-hexadiene-1,2-diol; cis-3-(2-Carboxy-ethyl)-3,5-cyclo-hexadiene-1,2-diol; 3-(cis-5,6-Dihydroxycyclohexa-1,3-dien-1-yl)propanoate | 1 | ec:1.18.1.3 |
| cpd:C00561 |  | Mandelonitrile; Benzaldehyde cyanohydrin; (R)-Mandelonitrile | 1 | ec:3.2.1.21 |
| cpd:C00166 |  | Phenylpyruvate; Phenylpyruvic acid; alpha-Ketohydrocinnamic acid; keto-Phenylpyruvate; 3-Phenyl-2-oxopropanoate; 2-Oxo-3-phenylpropanoate | 1 | ec:2.6.1.9 |
| cpd:C02670 |  | D-Glucuronolactone; Glucurone; D-Glucurono-3,6-lactone; D-Glucurone | 1 | ec:1.2.1.3 |
| cpd:C00558 |  | D-Tagaturonate; D-Tagaturonic acid | 1 | ec:5.3.1.12 |
| cpd:C01594 |  | Linamarin; Phaseolunatin | 1 | ec:3.2.1.21 |
| cpd:C00158 |  | Citrate; Citric acid; 2-Hydroxy-1,2,3-propanetricarboxylic acid; 2-Hydroxytricarballylic acid | 1 | ec:4.2.1.3 |
| cpd:C00153 |  | Nicotinamide; Nicotinic acid amide; Niacinamide; Vitamin PP | 1 | ec:2.4.2.1 |
| cpd:C03742 |  | (S)-4-Hydroxymandelonitrile | 1 | ec:3.2.1.21 |
| cpd:C06899 |  | Chloral hydrate | 1 | ec:1.1.1.284 ec:1.1.1.1 |
| cpd:C18235 |  | S-(Hydroxymethyl)mycothiol | 1 | ec:1.1.1.306 |
| cpd:C00147 |  | Adenine; 6-Aminopurine | 1 | ec:2.4.2.1 |
| cpd:C01185 |  | Nicotinate D-ribonucleotide; beta-Nicotinate D-ribonucleotide; Nicotinate ribonucleotide; Nicotinic acid ribonucleotide | 1 | ec:3.1.3.5 |
| cpd:C00144 |  | GMP; Guanosine 5'-phosphate; Guanosine monophosphate; Guanosine 5'-monophosphate; Guanylic acid | 1 | ec:3.1.3.5 |
| cpd:C01181 |  | 4-Trimethylammoniobutanoate; Butyro-betaine; gamma-Butyrobetaine | 1 | ec:1.2.1.3 |
| cpd:C00143 |  | 5,10-Methylenetetrahydrofolate; (6R)-5,10-Methylenetetrahydrofolate; 5,10-Methylene-THF | 1 | ec:2.1.2.11 |
| cpd:C04411 |  | (2R,3S)-3-Isopropylmalate; 3-Isopropylmalate; 3-Carboxy-2-hydroxy-4-methylpentanoate; 2-D-threo-Hydroxy-3-carboxy-isocaproate | 1 | ec:1.1.1.85 |
| cpd:C00141 |  | 3-Methyl-2-oxobutanoic acid; 3-Methyl-2-oxobutyric acid; 3-Methyl-2-oxobutanoate; 2-Oxo-3-methylbutanoate; 2-Oxoisovalerate; 2-Oxoisopentanoate; alpha-Ketovaline; 2-Ketovaline; 2-Keto-3-methylbutyric acid | 1 | ec:2.1.2.11 |
| cpd:C02659 |  | Acetone cyanohydrin; alpha-Hydroxyisobutyronitrile; 2-Hydroxy-2-methylpropanenitrile; 2-Methyllactonitrile; Acetone cyanhydrin; 2-Hydroxyisobutyronitrile | 1 | ec:3.2.1.21 |
| cpd:C20463 |  | Purine deoxyribonucleoside | 1 | ec:2.4.2.1 |
| cpd:C01179 |  | 3-(4-Hydroxyphenyl)pyruvate; 4-Hydroxyphenylpyruvate; p-Hydroxyphenylpyruvic acid | 1 | ec:2.6.1.9 |
| cpd:C05445 |  | 3alpha,7alpha-Dihydroxy-5beta-cholestan-26-al | 1 | ec:1.2.1.3 |
| cpd:C01172 |  | beta-D-Glucose 6-phosphate | 1 | ec:2.7.1.2 |
| cpd:C00099 |  | beta-Alanine; 3-Aminopropionic acid; 3-Aminopropanoate | 1 | ec:1.2.1.3 |
| cpd:C00130 |  | IMP; Inosinic acid; Inosine monophosphate; Inosine 5'-monophosphate; Inosine 5'-phosphate; 5'-Inosinate; 5'-Inosinic acid; 5'-Inosine monophosphate; 5'-IMP | 1 | ec:3.1.3.5 |
| cpd:C00092 |  | D-Glucose 6-phosphate; Glucose 6-phosphate; Robison ester | 1 | ec:2.7.1.2 |
| cpd:C00090 |  | Catechol; 1,2-Benzenediol; o-Benzenediol; 1,2-Dihydroxybenzene; Brenzcatechin; Pyrocatechol | 1 | ec:1.18.1.3 |
| cpd:C05839 |  | cis-beta-D-Glucosyl-2-hydroxycinnamate; beta-D-Glucosyl-2-coumarinate | 1 | ec:3.2.1.21 |
| cpd:C05838 |  | cis-2-Hydroxycinnamate; 2-Coumarinate | 1 | ec:3.2.1.21 |
| cpd:C00526 |  | Deoxyuridine; 2-Deoxyuridine; 2'-Deoxyuridine | 1 | ec:2.4.2.1 |
| cpd:C12622 |  | cis-3-(3-Carboxyethenyl)-3,5-cyclohexadiene-1,2-diol; (2E)-3-(cis-5,6-Dihydroxycyclohexa-1,3-dien-1-yl)prop-2-enoate | 1 | ec:1.18.1.3 |
| cpd:C00881 |  | Deoxycytidine; 2'-Deoxycytidine | 1 | ec:3.1.3.5 |
| cpd:C00489 |  | Glutarate; Glutaric acid; Pentanedioic acid; 1,3-Propanedicarboxylic acid | 1 | ec:1.2.1.79 ec:1.2.1.16 ec:1.2.1.20 |
| cpd:C15814 |  | C15814; Thiamine biosynthesis intermediate 5 | 1 |  |
| cpd:C15813 |  | C15813; Thiamine biosynthesis intermediate 4 | 1 |  |
| cpd:C15812 |  | [Enzyme]-S-sulfanylcysteine; Thiamine biosynthesis intermediate 3 | 1 |  |
| cpd:C00088 |  | Nitrite | 1 | ec:1.13.12.16 |
| cpd:C00082 |  | L-Tyrosine; (S)-3-(p-Hydroxyphenyl)alanine; (S)-2-Amino-3-(p-hydroxyphenyl)propionic acid; Tyrosine | 1 | ec:2.6.1.9 |
| cpd:C02992 |  | L-Threonyl-tRNA(Thr) | 1 | ec:6.1.1.3 |
| cpd:C06509 |  | Adenosyl cobinamide phosphate | 1 | ec:6.3.1.10 |
| cpd:C06508 |  | Adenosyl cobinamide | 1 | ec:6.3.1.10 |
| cpd:C06507 |  | Adenosyl cobyrinate hexaamide; Adenosylcobyric acid | 1 | ec:6.3.1.10 |
| cpd:C03273 |  | 5-Oxopentanoate; Glutarate semialdehyde | 1 | ec:1.2.1.79 ec:1.2.1.16 ec:1.2.1.20 |
| cpd:C00475 |  | Cytidine | 1 | ec:3.1.3.5 |
| cpd:C00473 |  | Retinol; all-trans-Retinol; Vitamin A; Vitamin A1 | 1 | ec:1.1.1.284 ec:1.1.1.1 |
| cpd:C15767 |  | 4-(L-gamma-Glutamylamino)butanoate; gamma-Glutamyl-gamma-aminobutyrate; 4-(Glutamylamino)butanoate; gamma-Glutamyl-GABA | 1 |  |
| cpd:C00079 |  | L-Phenylalanine; (S)-alpha-Amino-beta-phenylpropionic acid | 1 | ec:2.6.1.9 |
| cpd:C00072 |  | Ascorbate; Ascorbic acid; L-Ascorbate; L-Ascorbic acid; Vitamin C | 1 |  |
| cpd:C11924 |  | Perillic acid | 1 | ec:1.2.1.3 |
| cpd:C01149 |  | 4-Trimethylammoniobutanal | 1 | ec:1.2.1.3 |
| cpd:C00469 |  | Ethanol; Ethyl alcohol; Methylcarbinol | 1 | ec:1.1.1.284 ec:1.1.1.1 |
| cpd:C00109 |  | 2-Oxobutanoate; 2-Ketobutyric acid; 2-Oxobutyric acid; 2-Oxobutyrate; 2-Oxobutanoic acid; alpha-Ketobutyric acid; alpha-Ketobutyrate | 1 | ec:1.1.1.85 |
| cpd:C00108 |  | Anthranilate; Anthranilic acid; o-Aminobenzoic acid; Vitamin L1; 2-Aminobenzoate | 1 | ec:1.18.1.3 |
| cpd:C07490 |  | Trichloroethanol; 2,2,2-Trichloroethanol | 1 | ec:1.1.1.284 ec:1.1.1.1 |
| cpd:C00106 |  | Uracil | 1 | ec:2.4.2.1 |
| cpd:C00105 |  | UMP; Uridylic acid; Uridine monophosphate; Uridine 5'-monophosphate; 5'Uridylic acid | 1 | ec:3.1.3.5 |
| cpd:C00101 |  | Tetrahydrofolate; 5,6,7,8-Tetrahydrofolate; Tetrahydrofolic acid; THF; (6S)-Tetrahydrofolate; (6S)-Tetrahydrofolic acid; (6S)-THFA | 1 | ec:2.1.2.11 |
| cpd:C00067 |  | Formaldehyde; Methanal; Oxomethane; Oxomethylene; Methylene oxide; Formalin | 1 | ec:1.11.1.6 |
| cpd:C00061 |  | FMN; Riboflavin-5-phosphate; Flavin mononucleotide | 1 | ec:1.13.12.16 |
| cpd:C02576 |  | Perillyl aldehyde; Perillaldehyde | 1 | ec:1.2.1.3 |
| cpd:C00455 |  | Nicotinamide D-ribonucleotide; NMN; Nicotinamide mononucleotide; Nicotinamide ribonucleotide; Nicotinamide nucleotide; beta-Nicotinamide D-ribonucleotide; beta-Nicotinamide ribonucleotide; beta-Nicotinamide mononucleotide | 1 | ec:3.1.3.5 |
| cpd:C02170 |  | Methylmalonate; Methylmalonic acid | 1 | ec:1.2.1.3 |
| cpd:C00844 |  | Prunasin; (R)-Prunasin | 1 | ec:3.2.1.21 |
| cpd:C00448 |  | trans,trans-Farnesyl diphosphate; Farnesyl diphosphate; Farnesyl pyrophosphate; 2-trans,6-trans-Farnesyl diphosphate; (2E,6E)-Farnesyl diphosphate | 1 | ec:4.1.99.16 ec:4.2.3.75 ec:4.2.3.22 |
| cpd:C07111 |  | Ethylbenzene; Phenylethane; Ethylbenzol; Ethylenzene | 1 | ec:1.18.1.3 |
| cpd:C06790 |  | Trichloroethene; Trichloroethylene; TCE | 1 | ec:1.18.1.3 |
| cpd:C06032 |  | D-erythro-3-Methylmalate; (2R,3S)-3-Methylmalate | 1 | ec:1.1.1.85 |
| cpd:C01083 |  | alpha,alpha-Trehalose; alpha,alpha'-Trehalose; Trehalose | 1 |  |
| cpd:C18091 |  | Ethylnitronate | 1 | ec:1.13.12.16 |
| cpd:C03194 |  | (R)-1-Aminopropan-2-ol; (R)-1-Amino-2-propanol | 1 | ec:6.3.1.10 |
| cpd:C00433 |  | 2,5-Dioxopentanoate; 2-Oxoglutarate semialdehyde | 1 | ec:1.2.1.3 |
| cpd:C02946 |  | 4-Acetamidobutanoate; N4-Acetylaminobutanoate | 1 | ec:1.2.1.3 |
| cpd:C00033 |  | Acetate; Acetic acid; Ethanoic acid | 1 | ec:1.2.1.3 |
| cpd:C00030 |  | Reduced acceptor; AH2; Hydrogen-donor; Donor | 1 | ec:1.3.8.7 |
| cpd:C04261 |  | Protein N(pi)-phospho-L-histidine; Protein N-pros-phospho-L-histidine; Protein N-pros-phosphohistidine; Protein Npi-phospho-L-histidine | 1 |  |
| cpd:C00423 |  | trans-Cinnamate; trans-Cinnamic acid; (E)-Cinnamate | 1 | ec:1.18.1.3 |
| cpd:C01100 |  | L-Histidinol phosphate | 1 | ec:2.6.1.9 |
| cpd:C02939 |  | 3-Methylbutanoyl-CoA; Isovaleryl-CoA | 1 | ec:1.3.8.7 |
| cpd:C00028 |  | Acceptor; Hydrogen-acceptor; A; Oxidized donor | 1 | ec:1.3.8.7 |
| cpd:C00385 |  | Xanthine | 1 | ec:2.4.2.1 |
| cpd:C04253 |  | Electron-transferring flavoprotein; Electron-transfer flavoprotein | 1 | ec:1.3.8.7 |
| cpd:C00818 |  | D-Glucarate; D-Glucaric acid; L-Gularic acid; D-Saccharic acid; D-Glucosaccharic acid; Glucaric acid; Glucarate | 1 | ec:1.2.1.3 |
| cpd:C01455 |  | Toluene; Methylbenzene; Toluol | 1 | ec:1.18.1.3 |
| cpd:C00417 |  | cis-Aconitate; cis-Aconitic acid | 1 | ec:4.2.1.3 |
| cpd:C00376 |  | Retinal; Vitamin A aldehyde; Retinene; all-trans-Retinal; all-trans-Vitamin A aldehyde; all-trans-Retinene | 1 | ec:1.1.1.284 ec:1.1.1.1 |
| cpd:C00016 |  | FAD; Flavin adenine dinucleotide | 1 | ec:1.3.8.7 |
| cpd:C15700 |  | gamma-Glutamyl-gamma-aminobutyraldehyde | 1 |  |
| cpd:C01847 |  | Reduced FMN; FMNH2 | 1 | ec:1.13.12.16 |
| cpd:C00804 |  | Propynoate; Propiolic acid; Acetylenecarboxylic acid; Acetylenemonocarboxylate | 1 | ec:1.2.1.3 |
| cpd:C06755 |  | Chloroacetic acid; Chloroethanoic acid | 1 | ec:1.2.1.3 |
| cpd:C06754 |  | Chloroacetaldehyde; 2-Chloroethanal | 1 | ec:1.2.1.3 |
| cpd:C05713 |  | Cyanoglycoside; Cyanoglucoside | 1 | ec:3.2.1.21 |
| cpd:C05712 |  | Cyanohydrin | 1 | ec:3.2.1.21 |
| cpd:C00760 |  | Cellulose; (1,4-beta-D-Glucosyl)n; (1,4-beta-D-Glucosyl)n+1; (1,4-beta-D-Glucosyl)n-1; 1,4-beta-D-Glucan; Microcrystalline cellulose | 1 | ec:3.2.1.21 |
| cpd:C14180 |  | S-(Hydroxymethyl)glutathione | 1 | ec:1.1.1.284 ec:1.1.1.1 |
| cpd:C00365 |  | dUMP; Deoxyuridylic acid; Deoxyuridine monophosphate; Deoxyuridine 5'-phosphate; 2'-Deoxyuridine 5'-phosphate | 1 | ec:3.5.4.12 |
| cpd:C00364 |  | dTMP; Thymidine 5'-phosphate; Deoxythymidine 5'-phosphate; Thymidylic acid; 5'-Thymidylic acid; Thymidine monophosphate; Deoxythymidylic acid; Thymidylate | 1 | ec:3.1.3.5 |
| cpd:C00362 |  | dGMP; 2'-Deoxyguanosine 5'-monophosphate; 2'-Deoxyguanosine 5'-phosphate; Deoxyguanylic acid; Deoxyguanosine monophosphate | 1 | ec:3.1.3.5 |
| cpd:C00360 |  | dAMP; 2'-Deoxyadenosine 5'-phosphate; 2'-Deoxyadenosine 5'-monophosphate; Deoxyadenylic acid; Deoxyadenosine monophosphate | 1 | ec:3.1.3.5 |
| cpd:C04236 |  | (2S)-2-Isopropyl-3-oxosuccinate; 3-Carboxy-4-methyl-2-oxopentanoate; 2-Oxo-4-methyl-3-carboxypentanoate | 1 | ec:1.1.1.85 |
| cpd:C04592 |  | Toluene-cis-dihydrodiol; (1S,2R)-3-Methylcyclohexa-3,5-diene-1,2-diol | 1 | ec:1.18.1.3 |
| cpd:C01798 |  | D-Glucoside | 1 | ec:3.2.1.21 |
| cpd:C05665 |  | 3-Aminopropanal; beta-Aminopropion aldehyde | 1 | ec:1.2.1.3 |
| cpd:C02909 |  | (2-Naphthyl)methanol; 2-Naphthalenemethanol; 2-Hydroxymethylnaphthalene | 1 | ec:1.1.1.284 ec:1.1.1.1 |
| cpd:C01031 |  | S-Formylglutathione | 1 | ec:1.1.1.284 ec:1.1.1.1 |
| cpd:C16286 |  | Geosmin; (-)-Geosmin; trans-1,10-Dimethyl-trans-9-decalol | 1 | ec:4.1.99.16 ec:4.2.3.75 ec:4.2.3.22 |
| cpd:C18796 |  | (2R)-2-Hydroxy-2-methylbutanenitrile; 2-Hydroxy-2-methylbutanenitrile | 1 | ec:3.2.1.21 |

  
**Over-represented Pathway Summary**: Collection of the KEGG metabolic pathways containing the proteins identified in the "Over-represented Metabolite Summary" ranked by the highest number of hits per pathway  

| Pathway ID | EC | EC Frequency | Name |
| --- | --- | --- | --- |
| map00340 | ec:4.2.1.49 ec:2.6.1.9 ec:3.5.1.68 ec:3.5.3.13 ec:1.2.1.3 ec:3.5.3.8 ec:4.3.1.3 ec:3.5.2.7 | 348 | path:map00340 Histidine metabolism |
| map00330 | ec:3.5.1.96 ec:6.3.1.2 ec:2.6.1.81 ec:3.5.3.23 ec:4.3.1.12 ec:3.4.11.5 ec:1.4.1.2 ec:2.3.1.109 ec:1.2.1.3 ec:6.3.4.5 ec:1.2.1.71 | 75 | path:map00330 Arginine and proline metabolism |
| map00630 | ec:4.2.1.3 ec:3.5.1.68 ec:6.3.1.2 ec:1.2.1.2 ec:1.1.3.15 ec:1.11.1.6 | 34 | path:map00630 Glyoxylate and dicarboxylate metabolism |
| map00071 | ec:1.18.1.3 ec:1.14.15.3 ec:5.1.2.3 ec:6.2.1.3 ec:2.3.1.16 ec:1.2.1.3 ec:1.1.1.35 ec:1.1.1.1 ec:1.3.8.7 ec:4.2.1.17 | 29 | path:map00071 Fatty acid degradation |
| map00780 | ec:2.3.1.47 ec:6.3.3.3 ec:2.8.1.6 ec:2.1.1.197 ec:2.6.1.62 | 25 | path:map00780 Biotin metabolism |
| map00250 | ec:6.3.1.2 ec:1.4.1.2 ec:1.2.1.79 ec:1.2.1.16 ec:6.3.4.5 | 24 | path:map00250 Alanine, aspartate and glutamate metabolism |
| map00910 | ec:3.5.5.1 ec:6.3.1.2 ec:1.7.2.5 ec:1.4.1.2 ec:1.13.12.16 | 19 | path:map00910 Nitrogen metabolism |
| map00270 | ec:3.1.3.77 ec:2.3.1.30 ec:4.4.1.15 | 18 | path:map00270 Cysteine and methionine metabolism |
| map00650 | ec:1.1.1.35 ec:5.1.2.3 ec:4.2.1.17 ec:1.2.1.79 ec:1.2.1.16 | 16 | path:map00650 Butanoate metabolism |
| map00280 | ec:1.3.8.7 ec:1.2.1.3 ec:1.1.1.35 ec:2.3.1.16 ec:4.2.1.17 | 16 | path:map00280 Valine, leucine and isoleucine degradation |
| map00360 | ec:2.6.1.9 ec:3.5.1.32 ec:4.2.1.17 | 15 | path:map00360 Phenylalanine metabolism |
| map00680 | ec:1.1.1.284 ec:1.2.1.2 ec:3.1.3.11 | 15 | path:map00680 Methane metabolism |
| map00062 | ec:1.1.1.35 ec:2.3.1.16 ec:4.2.1.17 | 14 | path:map00062 Fatty acid elongation |
| map00061 | ec:2.3.1.180 | 14 | path:map00061 Fatty acid biosynthesis |
| map00380 | ec:3.5.5.1 ec:1.2.1.3 ec:1.1.1.35 ec:4.2.1.17 ec:1.11.1.6 | 14 | path:map00380 Tryptophan metabolism |
| map00281 | ec:1.1.1.35 ec:2.3.1.16 ec:4.2.1.17 | 14 | path:map00281 Geraniol degradation |
| map00920 | ec:3.6.3.36 ec:2.3.1.30 ec:2.7.1.25 | 13 | path:map00920 Sulfur metabolism |
| map00230 | ec:3.1.3.5 ec:2.4.2.1 ec:2.7.1.25 ec:2.7.7.7 | 13 | path:map00230 Purine metabolism |
| map00010 | ec:1.1.1.1 ec:1.2.1.3 ec:2.7.1.2 ec:3.1.3.11 | 12 | path:map00010 Glycolysis / Gluconeogenesis |
| map00500 | ec:3.1.1.11 ec:2.7.1.2 ec:3.2.1.21 ec:2.4.1.1 | 12 | path:map00500 Starch and sucrose metabolism |
| map00362 | ec:4.1.1.44 ec:2.3.1.16 ec:4.2.1.17 | 12 | path:map00362 Benzoate degradation |
| map00030 | ec:3.1.1.31 ec:3.1.3.11 | 12 | path:map00030 Pentose phosphate pathway |
| map00310 | ec:1.2.1.3 ec:1.1.1.35 ec:1.2.1.20 ec:4.2.1.17 | 12 | path:map00310 Lysine degradation |
| map00040 | ec:5.3.1.12 ec:3.1.1.11 ec:1.2.1.3 ec:1.1.1.57 | 11 | path:map00040 Pentose and glucuronate interconversions |
| map00720 | ec:4.2.1.3 ec:1.1.1.35 ec:4.2.1.17 | 11 | path:map00720 Carbon fixation pathways in prokaryotes |
| map00930 | ec:1.1.1.35 ec:4.2.1.17 | 10 | path:map00930 Caprolactam degradation |
| map00592 | ec:2.3.1.16 ec:4.2.1.17 | 10 | path:map00592 alpha-Linolenic acid metabolism |
| map00051 | ec:3.1.3.11 | 9 | path:map00051 Fructose and mannose metabolism |
| map00710 | ec:3.1.3.11 | 9 | path:map00710 Carbon fixation in photosynthetic organisms |
| map00600 | ec:2.4.1.80 | 9 | path:map00600 Sphingolipid metabolism |
| map00640 | ec:1.3.8.7 ec:1.2.1.3 ec:4.2.1.17 | 8 | path:map00640 Propanoate metabolism |
| map00627 | ec:3.5.5.1 ec:4.2.1.17 | 8 | path:map00627 Aminobenzoate degradation |
| map00410 | ec:1.3.8.7 ec:1.2.1.3 ec:4.2.1.17 | 8 | path:map00410 beta-Alanine metabolism |
| map00903 | ec:1.2.1.3 ec:4.2.1.17 | 7 | path:map00903 Limonene and pinene degradation |
| map00760 | ec:3.1.3.5 ec:2.4.2.1 ec:2.7.1.23 | 7 | path:map00760 Nicotinate and nicotinamide metabolism |
| map00830 | ec:1.1.1.1 ec:1.14.15.3 | 6 | path:map00830 Retinol metabolism |
| map00240 | ec:3.5.4.12 ec:3.1.3.5 ec:2.4.2.1 ec:2.7.7.7 | 6 | path:map00240 Pyrimidine metabolism |
| map00860 | ec:6.3.1.10 ec:4.99.1.1 | 6 | path:map00860 Porphyrin and chlorophyll metabolism |
| map00400 | ec:2.6.1.9 ec:4.2.3.5 | 5 | path:map00400 Phenylalanine, tyrosine and tryptophan biosynthesis |
| map00590 | ec:1.14.15.3 | 5 | path:map00590 Arachidonic acid metabolism |
| map00430 | ec:1.4.1.2 | 5 | path:map00430 Taurine and hypotaurine metabolism |
| map00982 | ec:1.1.1.1 ec:2.5.1.18 | 5 | path:map00982 Drug metabolism - cytochrome P450 |
| map00980 | ec:1.1.1.1 ec:2.5.1.18 | 5 | path:map00980 Metabolism of xenobiotics by cytochrome P450 |
| map00620 | ec:4.4.1.5 ec:1.2.1.3 | 5 | path:map00620 Pyruvate metabolism |
| map00970 | ec:6.1.1.3 ec:2.9.1.1 | 5 | path:map00970 Aminoacyl-tRNA biosynthesis |
| map00450 | ec:2.9.1.1 | 4 | path:map00450 Selenocompound metabolism |
| map00642 | ec:2.3.1.16 | 4 | path:map00642 Ethylbenzene degradation |
| map00623 | ec:1.1.1.35 | 4 | path:map00623 Toluene degradation |
| map00480 | ec:2.5.1.18 | 4 | path:map00480 Glutathione metabolism |
| map00120 | ec:1.1.1.35 | 4 | path:map00120 Primary bile acid biosynthesis |
| map00564 | ec:2.7.8.5 | 3 | path:map00564 Glycerophospholipid metabolism |
| map00460 | ec:3.5.5.1 ec:3.2.1.21 | 3 | path:map00460 Cyanoamino acid metabolism |
| map00750 | ec:2.7.1.35 | 3 | path:map00750 Vitamin B6 metabolism |
| map00350 | ec:1.1.1.1 ec:2.6.1.9 ec:1.2.1.16 | 3 | path:map00350 Tyrosine metabolism |
| map00909 | ec:4.2.3.75 ec:4.1.99.16 ec:4.2.3.22 | 3 | path:map00909 Sesquiterpenoid and triterpenoid biosynthesis |
| map00643 | ec:3.5.5.1 | 2 | path:map00643 Styrene degradation |
| map00633 | ec:1.12.99.6 | 2 | path:map00633 Nitrotoluene degradation |
| map00625 | ec:1.1.1.1 ec:1.2.1.3 | 2 | path:map00625 Chloroalkane and chloroalkene degradation |
| map00561 | ec:1.2.1.3 | 1 | path:map00561 Glycerolipid metabolism |
| map00401 | ec:2.6.1.9 | 1 | path:map00401 Novobiocin biosynthesis |
| map00260 | ec:1.1.1.1 | 1 | path:map00260 Glycine, serine and threonine metabolism |
| map00940 | ec:3.2.1.21 | 1 | path:map00940 Phenylpropanoid biosynthesis |
| map00053 | ec:1.2.1.3 | 1 | path:map00053 Ascorbate and aldarate metabolism |
| map00052 | ec:2.7.1.2 | 1 | path:map00052 Galactose metabolism |
| map04151 | ec:2.7.11.1 | 1 | path:map04151 PI3K-Akt signaling pathway |
| map04150 | ec:2.7.11.1 | 1 | path:map04150 mTOR signaling pathway |
| map00626 | ec:1.1.1.1 | 1 | path:map00626 Naphthalene degradation |
| map00290 | ec:1.1.1.85 | 1 | path:map00290 Valine, leucine and isoleucine biosynthesis |
| map00622 | ec:1.18.1.3 | 1 | path:map00622 Xylene degradation |
| map00524 | ec:2.7.1.2 | 1 | path:map00524 Butirosin and neomycin biosynthesis |
| map00190 | ec:1.9.3.1 | 1 | path:map00190 Oxidative phosphorylation |
| map00521 | ec:2.7.1.2 | 1 | path:map00521 Streptomycin biosynthesis |
| map00520 | ec:2.7.1.2 | 1 | path:map00520 Amino sugar and nucleotide sugar metabolism |
| map00770 | ec:2.1.2.11 | 1 | path:map00770 Pantothenate and CoA biosynthesis |
| map00020 | ec:4.2.1.3 | 1 | path:map00020 Citrate cycle (TCA cycle) |
| map00960 | ec:2.6.1.9 | 1 | path:map00960 Tropane, piperidine and pyridine alkaloid biosynthesis |

  
Analysis performed on 2014/02/15 00:03:53
