# Supplementary material for: Automated genomic context analysis and experimental validation platform for discovery of prokaryote transcriptional regulator functions
Source: BMC Genomics. 2014 Dec 18;15(1):1142. doi: 10.1186/1471-2164-15-1142 (PMC4349456; doi:10.1186/1471-2164-15-1142)
Supplement: Supplementary file 10 — Additional file 10: Result RcoM. Function Discovery V1.0 output (.html format) for the carbon monoxide oxidation regulator (RcoM, Bxe_ A2142). For detailed instructions on how to analyze the results please refer to the Function Discovery V1.0, a gene neighborhood analysis tool section in the Results part of the main text. (HTML 36 KB) [file 12864_2014_6995_MOESM10_ESM.html]

```
ENTRY       Bxe_A2142         CDS       T00340
DEFINITION  CO metabolism transcriptional regulator RcoM
ORGANISM    bxe  Burkholderia xenovorans
POSITION    1:2552861..2553664
MOTIF       Pfam: LytTR PAS_4 PAS_8 PAS_9 PAS_7
DBLINKS     NCBI-GI: 91783672
            NCBI-GeneID: 4003299
            JGI: BxeA2142
            UniProt: Q13YL3
AASEQ       267
            MKSSEPASVSAAERRAETFQHKLEQFNPGIVWLDQHGRVTAFNDVALQILGPAGEQSLGV
            AQDSLFGIDVVQLHPEKSRDKLRFLLQSKDVGGCPVKSPPPVAMMINIPDRILMIKVSSM
            IAAGGACGTCMIFYDVTDLTTEPSGLPAGGSAPSPRRLFKIPVYRKNRVILLDLKDIVRF
            QGDGHYTTIVTRDDRYLSNLSLADLELRLDSSIYLRVHRSHIVSLQYAVELVKLDESVNL
            VMDDAEQTQVPVSRSRTAQLKELLGVV
NTSEQ       804
            atgaaatcgtctgagcccgcttccgtatcggcggccgagcgccgcgcggagacgttccag
            cacaagctcgagcagttcaatccggggatcgtctggctggaccagcacggccgcgtcacg
            gcattcaacgacgtcgcgctgcaaattctcgggccggccggcgagcaatcgctaggcgtg
            gcgcaggatagtctgttcggcatcgacgtggtgcaactgcacccggagaaaagccgcgac
            aagctgcgcttcctgctgcagtcgaaagacgtgggtggctgcccggtcaagtcgccgccg
            ccggtggcgatgatgatcaatattcccgaccggatcctgatgatcaaggtgtccagcatg
            atcgccgccggcggcgcgtgcggcacctgcatgatcttctacgacgtcaccgatctgacc
            accgagccctccggcctgccggcgggcggcagcgcgccctcgccgaggcgcctcttcaag
            attcccgtgtaccggaagaaccgcgtgatcctgctcgatctgaaagacatcgtgcgcttt
            cagggcgacggccactacacgacgatcgtgacgcgggacgaccgctacctgtccaatctt
            tcactggccgatctggagctgcgtctcgacagcagcatttatctgcgcgttcatcgcagc
            catatcgtgagcctgcaatatgcggtggagctggtcaaactggacgagagcgtcaatctc
            gtcatggacgacgcggaacagacccaggtgccggtcagccgctcgcgaacggcgcaactg
            aaggagcttctcggcgtggtttga
///
```

  
**Homolog ID**: Table of closest homologs  

```
                 Homologs                                       len   identity overlap
---------------------------------------------------------------------------------
aeh:Mlg_1561 PAS/PAC sensor protein                           249     0.482    257 
ret:RHE_CH01718 hypothetical protein                           96     0.471    51
```

**Neighborhood Representations**: Table of genes in the defined genetic neighborhoods of the entry protein and its closest homologs  
  
**Neighborhood Representations for "bxe:Bxe\_A2142"**  

| ID | Annotation | EC number |
| --- | --- | --- |
| bxe:Bxe\_A2152 | glycosyl transferase family protein |  |
| bxe:Bxe\_A2151 | pseudogene |  |
| bxe:Bxe\_A2150 | sulfurylase large subunit, molybdopterin cytosine dinucleotide biosynthesis / sulfurylase small subunit, molybdopterin cytosine dinucleotide biosynthesis |  |
| bxe:Bxe\_A2149 | carbon monoxide dehydrogenase, CoxE subunit |  |
| bxe:Bxe\_A2148 | MoxR-like ATPase, CoxD |  |
| bxe:Bxe\_A2147 | carbon monoxide dehydrogenase, CoxG subunit; K09386 hypothetical protein |  |
| bxe:Bxe\_A2146 | coxL2; carbon monoxide dehydrogenase, large subunit apoprotein (EC:1.2.99.2); K03520 carbon-monoxide dehydrogenase large subunit [EC:1.2.99.2] | ec:1.2.99.2 |
| bxe:Bxe\_A2145 | carbon monoxide dehydrogenase, small subunit (EC:1.2.99.2); K03518 carbon-monoxide dehydrogenase small subunit [EC:1.2.99.2] | ec:1.2.99.2 |
| bxe:Bxe\_A2144 | carbon monoxide dehydrogenase, medium subunit (EC:1.2.99.2); K03519 carbon-monoxide dehydrogenase medium subunit [EC:1.2.99.2] | ec:1.2.99.2 |
| bxe:Bxe\_A2143 | hypothetical protein |  |
| bxe:Bxe\_A2142 | CO metabolism transcriptional regulator RcoM |  |
| bxe:Bxe\_A2141 | hypothetical protein |  |
| bxe:Bxe\_A2140 | hypothetical protein |  |
| bxe:Bxe\_A2139 | hypothetical protein; K07114 Ca-activated chloride channel homolog |  |
| bxe:Bxe\_A2138 | hypothetical protein; K07114 Ca-activated chloride channel homolog |  |
| bxe:Bxe\_A2137 | transmembrane protein |  |
| bxe:Bxe\_A2136 | hypothetical protein |  |
| bxe:Bxe\_A2135 | MoxR-like ATPase, transcriptional regulator, C1 metabolism; K03924 MoxR-like ATPase [EC:3.6.3.-] |  |
| bxe:Bxe\_A2134 | hypothetical protein |  |
| bxe:Bxe\_A2133 | nonspecific acid phosphatase, NapD-like |  |
| bxe:Bxe\_A2132 | arylsulfatase (EC:3.1.6.1); K01130 arylsulfatase [EC:3.1.6.1] | ec:3.1.6.1 |

  
**Neighborhood Representations for "aeh:Mlg\_1561"**  

| ID | Annotation | EC number |
| --- | --- | --- |
| aeh:Mlg\_1551 | hypothetical protein |  |
| aeh:Mlg\_1552 | hypothetical protein; K02742 SprT protein |  |
| aeh:Mlg\_1553 | dihydroxy-acid dehydratase (EC:4.2.1.9); K01687 dihydroxy-acid dehydratase [EC:4.2.1.9] | ec:4.2.1.9 |
| aeh:Mlg\_1554 | hypothetical protein |  |
| aeh:Mlg\_1555 | phosphate-selective porin O and P; K07221 phosphate-selective porin OprO and OprP |  |
| aeh:Mlg\_1556 | hypothetical protein |  |
| aeh:Mlg\_1557 | hypothetical protein; K03593 ATP-binding protein involved in chromosome partitioning |  |
| aeh:Mlg\_1558 | hypothetical protein |  |
| aeh:Mlg\_1559 | molybdopterin synthase subunit MoaE; K03635 molybdopterin synthase catalytic subunit [EC:2.-.-.-] |  |
| aeh:Mlg\_1560 | PAS/PAC sensor-containing diguanylate cyclase/phosphodiesterase |  |
| aeh:Mlg\_1561 | PAS/PAC sensor protein |  |
| aeh:Mlg\_1562 | carbon monoxide dehydrogenase, medium subunit; K03519 carbon-monoxide dehydrogenase medium subunit [EC:1.2.99.2] | ec:1.2.99.2 |
| aeh:Mlg\_1563 | carbon monoxide dehydrogenase, small subunit; K03518 carbon-monoxide dehydrogenase small subunit [EC:1.2.99.2] | ec:1.2.99.2 |
| aeh:Mlg\_1564 | carbon monoxide dehydrogenase, large subunit apoprotein; K03520 carbon-monoxide dehydrogenase large subunit [EC:1.2.99.2] | ec:1.2.99.2 |
| aeh:Mlg\_1565 | ATPase |  |
| aeh:Mlg\_1566 | von Willebrand factor A |  |
| aeh:Mlg\_1567 | hypothetical protein; K07402 xanthine dehydrogenase accessory factor |  |
| aeh:Mlg\_1568 | molybdenum cofactor cytidylyltransferase; K07141 molybdenum cofactor cytidylyltransferase [EC:2.7.7.76] | ec:2.7.7.76 |
| aeh:Mlg\_1569 | hypothetical protein |  |
| aeh:Mlg\_1570 | NifU domain-containing protein |  |
| aeh:Mlg\_1571 | type II secretion system protein E |  |

  
**Neighborhood Representations for "ret:RHE\_CH01718"**  

| ID | Annotation | EC number |
| --- | --- | --- |
| ret:RHE\_CH01708 | recombination factor protein RarA; K07478 putative ATPase |  |
| ret:RHE\_CH01709 | hypothetical protein |  |
| ret:RHE\_CH01710 | 2-dehydropantoate 2-reductase (EC:1.1.1.169); K00077 2-dehydropantoate 2-reductase [EC:1.1.1.169] | ec:1.1.1.169 |
| ret:RHE\_CH01711 | dehydrogenase |  |
| ret:RHE\_CH01712 | LysR family transcriptional regulator |  |
| ret:RHE\_CH01713 | nucleoside 2-deoxyribosyltransferase |  |
| ret:RHE\_CH01714 | esterase/lipase/thioesterase family protein; K01066 esterase / lipase [EC:3.1.1.-] |  |
| ret:RHE\_CH01715 | hypothetical protein |  |
| ret:RHE\_CH01716 | two-component sensor histidine kinase |  |
| ret:RHE\_CH01717 | two-component sensor histidine kinase |  |
| ret:RHE\_CH01718 | hypothetical protein |  |
| ret:RHE\_CH01719 | LacI family transcription regulator; K02529 LacI family transcriptional regulator |  |
| ret:RHE\_CH01720 | acyl-CoA transferase; K01041 [EC:2.8.3.-] |  |
| ret:RHE\_CH01721 | enoyl-CoA hydratase (EC:4.2.1.17) |  |
| ret:RHE\_CH01722 | NADP-dependent L-sorbosone dehydrogenase (EC:1.2.1.8); K00130 betaine-aldehyde dehydrogenase [EC:1.2.1.8] | ec:1.2.1.8 |
| ret:RHE\_CH01723 | sugar ABC transporter, substrate-binding protein; K17315 glucose/mannose transport system substrate-binding protein |  |
| ret:RHE\_CH01724 | sugar ABC transporter, permease; K17316 glucose/mannose transport system permease protein |  |
| ret:RHE\_CH01725 | sugar ABC transporter, permease; K17317 glucose/mannose transport system permease protein |  |
| ret:RHE\_CH01726 | sugar ABC transporter, ATP-binding protein; K10112 multiple sugar transport system ATP-binding protein |  |
| ret:RHE\_CH01727 | dehydrogenase; K00119 [EC:1.1.99.-] |  |
| ret:RHE\_CH01728 | FAD-dependent L-sorbose dehydrogenase; K00119 [EC:1.1.99.-] |  |

  
**Over-represented Enzyme Summary**: Table of E.C. identified protein in the "Neighborhood Representation" ranked by frequency of occurrence  

| EC number | Frequency | Annotation | Reactions |
| --- | --- | --- | --- |
| ec:1.2.99.2 | 6 | carbon-monoxide dehydrogenase (acceptor); anaerobic carbon monoxide dehydrogenase; carbon monoxide oxygenase; carbon-monoxide dehydrogenase; carbon-monoxide:(acceptor) oxidoreductase | CO + H2O + A = CO2 + AH2 [RN:R00296] |
| ec:4.2.1.9 | 1 | dihydroxy-acid dehydratase; acetohydroxyacid dehydratase; alpha,beta-dihydroxyacid dehydratase; 2,3-dihydroxyisovalerate dehydratase; alpha,beta-dihydroxyisovalerate dehydratase; dihydroxy acid dehydrase; DHAD; 2,3-dihydroxy-acid hydro-lyase | 2,3-dihydroxy-3-methylbutanoate = 3-methyl-2-oxobutanoate + H2O [RN:R01209] |
| ec:1.1.1.169 | 1 | 2-dehydropantoate 2-reductase; 2-oxopantoate reductase; 2-ketopantoate reductase; 2-ketopantoic acid reductase; ketopantoate reductase; ketopantoic acid reductase | (R)-pantoate + NADP+ = 2-dehydropantoate + NADPH + H+ [RN:R02472] |
| ec:2.7.7.76 | 1 | molybdenum cofactor cytidylyltransferase; MocA; CTP:molybdopterin cytidylyltransferase; MoCo cytidylyltransferase; Mo-MPT cytidyltransferase | CTP + molybdenum cofactor = diphosphate + cytidylyl molybdenum cofactor |
| ec:3.1.6.1 | 1 | arylsulfatase; sulfatase; nitrocatechol sulfatase; phenolsulfatase; phenylsulfatase; p-nitrophenyl sulfatase; arylsulfohydrolase; 4-methylumbelliferyl sulfatase; estrogen sulfatase | a phenol sulfate + H2O = a phenol + sulfate [RN:R01243] |
| ec:1.2.1.8 | 1 | betaine-aldehyde dehydrogenase; betaine aldehyde oxidase; BADH; betaine aldehyde dehydrogenase; BetB | betaine aldehyde + NAD+ + H2O = betaine + NADH + 2 H+ [RN:R02565] |

  
**Over-represented Metabolite Summary**: Collection of the metabolites identified as substrates or products of the proteins representaed the "Over-represented Enzyme Summary" ranked by frequency of occurrence  

| ID | Structure | Name | Frequency | EC |
| --- | --- | --- | --- | --- |
| cpd:C00001 |  | H2O; Water | 11 | ec:3.1.6.1 ec:1.2.1.8 ec:1.2.99.2 ec:4.2.1.9 |
| cpd:C00011 |  | CO2; Carbon dioxide | 6 | ec:1.2.99.2 |
| cpd:C00237 |  | CO; Carbon monoxide | 6 | ec:1.2.99.2 |
| cpd:C16399 |  | 2,4-Diamino-6-hydroxylaminotoluene | 6 | ec:1.2.99.2 |
| cpd:C00139 |  | Oxidized ferredoxin | 6 | ec:1.2.99.2 |
| cpd:C00138 |  | Reduced ferredoxin | 6 | ec:1.2.99.2 |
| cpd:C16396 |  | 2,4-Diamino-6-nitrotoluene | 6 | ec:1.2.99.2 |
| cpd:C00080 |  | H+; Hydron | 3 | ec:1.2.1.8 ec:1.1.1.169 |
| cpd:C00006 |  | NADP+; NADP; Nicotinamide adenine dinucleotide phosphate; beta-Nicotinamide adenine dinucleotide phosphate; TPN; Triphosphopyridine nucleotide | 2 | ec:1.2.1.8 ec:1.1.1.169 |
| cpd:C03557 |  | 2-Aminoethylphosphonate; (2-Aminoethyl)phosphonate; Ciliatine | 2 |  |
| cpd:C00005 |  | NADPH; TPNH; Reduced nicotinamide adenine dinucleotide phosphate | 2 | ec:1.2.1.8 ec:1.1.1.169 |
| cpd:C05678 |  | 1-Hydroxy-2-aminoethylphosphonate | 2 |  |
| cpd:C18216 |  | 4-Hydroxyphenyl-4-hydroxybenzoate | 1 |  |
| cpd:C06007 |  | (R)-2,3-Dihydroxy-3-methylpentanoate; (R)-2,3-Dihydroxy-3-methylvalerate; (2R,3R)-2,3-Dihydroxy-3-methylpentanoate | 1 | ec:4.2.1.9 |
| cpd:C06676 |  | 4-Sulfolactone; 4-Carboxymethyl-4-sulfobut-2-en-4-olide; 4-Sulfomuconolactone | 1 |  |
| cpd:C00530 |  | Hydroquinone; p-Benzenediol; 1,4-Benzenediol; 1,4-Dihydroxybenzene; Benzene-1,4-diol; Quinol; 4-Hydroxyphenol | 1 |  |
| cpd:C02686 |  | Galactosylceramide; Galactocerebroside; D-Galactosyl-N-acylsphingosine; Cerebroside; D-Galactosylceramide | 1 | ec:3.1.6.1 |
| cpd:C00468 |  | Estrone; 3-Hydroxy-1,3,5(10)-estratrien-17-one | 1 | ec:3.1.6.1 |
| cpd:C00141 |  | 3-Methyl-2-oxobutanoic acid; 3-Methyl-2-oxobutyric acid; 3-Methyl-2-oxobutanoate; 2-Oxo-3-methylbutanoate; 2-Oxoisovalerate; 2-Oxoisopentanoate; alpha-Ketovaline; 2-Ketovaline; 2-Keto-3-methylbutyric acid | 1 | ec:4.2.1.9 |
| cpd:C04039 |  | 2,3-Dihydroxy-3-methylbutanoate; 2,3-Dihydroxy-isovalerate; 2,3-Dihydroxy-isovaleric acid | 1 | ec:4.2.1.9 |
| cpd:C14120 |  | 2-Naphthoyl-CoA | 1 |  |
| cpd:C06125 |  | Sulfatide; Galactosylceramidesulfate; Cerebroside 3-sulfate | 1 | ec:3.1.6.1 |
| cpd:C00010 |  | CoA; Coenzyme A; CoA-SH | 1 |  |
| cpd:C12448 |  | Ecgonine methyl ester; Methyl ecgonine | 1 |  |
| cpd:C18239 |  | Precursor Z; Cyclic pyranopterin monophosphate; Cyclic pyranopterin phosphate; cPMP | 1 |  |
| cpd:C16465 |  | trans-Geranyl-CoA | 1 |  |
| cpd:C00522 |  | (R)-Pantoate; Pantoate; Pantoic acid | 1 | ec:1.1.1.169 |
| cpd:C16461 |  | Geranic acid; 3,7-Dimethylocta-2,6-dienoate; Geranate | 1 |  |
| cpd:C00719 |  | Betaine; Trimethylaminoacetate; Glycine betaine; N,N,N-Trimethylglycine; Trimethylammonioacetate | 1 | ec:1.2.1.8 |
| cpd:C00132 |  | Methanol; Methyl alcohol; CH3OH | 1 |  |
| cpd:C02222 |  | 2-Maleylacetate; 4-Oxohex-2-enedioate; Maleylacetate | 1 |  |
| cpd:C17023 |  | Sulfur donor; S-donor | 1 |  |
| cpd:C00966 |  | 2-Dehydropantoate | 1 | ec:1.1.1.169 |
| cpd:C00004 |  | NADH; DPNH; Reduced nicotinamide adenine dinucleotide | 1 | ec:1.2.1.8 |
| cpd:C00003 |  | NAD+; NAD; Nicotinamide adenine dinucleotide; DPN; Diphosphopyridine nucleotide; Nadide | 1 | ec:1.2.1.8 |
| cpd:C00671 |  | (S)-3-Methyl-2-oxopentanoic acid; (S)-3-Methyl-2-oxopentanoate; (3S)-3-Methyl-2-oxopentanoic acid; (3S)-3-Methyl-2-oxopentanoate | 1 | ec:4.2.1.9 |
| cpd:C11481 |  | HSO3-; Hydrogen sulfite; Bisulfite | 1 |  |
| cpd:C00576 |  | Betaine aldehyde | 1 | ec:1.2.1.8 |
| cpd:C10858 |  | Ecgonine | 1 |  |
| cpd:C04272 |  | (R)-2,3-Dihydroxy-3-methylbutanoate; (R)-2,3-Dihydroxy-isovalerate; (R)-2,3-Dihydroxy-isovaleric acid; (2R)-2,3-Dihydroxy-3-methylbutanoate | 1 | ec:4.2.1.9 |
| cpd:C02538 |  | Estrone 3-sulfate | 1 | ec:3.1.6.1 |
| cpd:C00156 |  | 4-Hydroxybenzoate; Hydroxybenzoic acid; 4-Hydroxybenzoic acid; Hydroxybenzenecarboxylic acid | 1 |  |
| cpd:C14101 |  | 2-Naphthoic acid; 2-Naphthalenecarboxylic acid; beta-Naphthoic acid | 1 |  |
| cpd:C00059 |  | Sulfate; Sulfuric acid | 1 | ec:3.1.6.1 |
| cpd:C05924 |  | Molybdopterin; Pyranopterin; H2Dtpp-mP | 1 |  |

  
**Over-represented Pathway Summary**: Collection of the KEGG metabolic pathways containing the proteins identified in the "Over-represented Metabolite Summary" ranked by the highest number of hits per pathway  

| Pathway ID | EC | EC Frequency | Name |
| --- | --- | --- | --- |
| map00720 | ec:1.2.99.2 | 6 | path:map00720 Carbon fixation pathways in prokaryotes |
| map00633 | ec:1.2.99.2 | 6 | path:map00633 Nitrotoluene degradation |
| map00680 | ec:1.2.99.2 | 6 | path:map00680 Methane metabolism |
| map00770 | ec:4.2.1.9 ec:1.1.1.169 | 2 | path:map00770 Pantothenate and CoA biosynthesis |
| map00140 | ec:3.1.6.1 | 1 | path:map00140 Steroid hormone biosynthesis |
| map00600 | ec:3.1.6.1 | 1 | path:map00600 Sphingolipid metabolism |
| map00290 | ec:4.2.1.9 | 1 | path:map00290 Valine, leucine and isoleucine biosynthesis |
| map00260 | ec:1.2.1.8 | 1 | path:map00260 Glycine, serine and threonine metabolism |

  
Analysis performed on 2014/02/15 00:40:25
