# Supplementary material for: Automated genomic context analysis and experimental validation platform for discovery of prokaryote transcriptional regulator functions
Source: BMC Genomics. 2014 Dec 18;15(1):1142. doi: 10.1186/1471-2164-15-1142 (PMC4349456; doi:10.1186/1471-2164-15-1142)
Supplement: Supplementary file 11 — Additional file 11: Result ModE. Function Discovery V1.0 output (.html format) for the molybdate transport regulator (ModE, Bxe_ B2851). For detailed instructions on how to analyze the results please refer to the Function Discovery V1.0, a gene neighborhood analysis tool section in the Results part of the main text. (HTML 732 KB) [file 12864_2014_6995_MOESM11_ESM.html]

```
ENTRY       Bxe_B2851         CDS       T00340
DEFINITION  ModE family transcriptional regulator
ORTHOLOGY   K02019  molybdate transport system regulatory protein
ORGANISM    bxe  Burkholderia xenovorans
POSITION    2:211062..211898
MOTIF       Pfam: TOBE HTH_1
DBLINKS     NCBI-GI: 91777286
            NCBI-GeneID: 4006784
            JGI: BxeB2851
            UniProt: Q13RZ5
AASEQ       278
            MTLHRTDPSRETLELGGSVWFQAGAQTLGGASRIALLAAIGETGSITSAAKAVGMSYKGA
            WDAIDTMNNLAGEPLVVRLTGGKGGGGTTLTPRAVKLIETFRAVEREHRRFLERAGAAIE
            GFATDWDLIGRIGVKTSARNQLYGTVSAITRGTVNDEVTLVLPGGHAIVSVITHESTETL
            GLVEGAAAFALIKASWVVLFVDNQSGVPLKLSARNQLRGTVQSVKRGAVNAEVSLVLAGG
            AVITAVVTNESVDTLGLVEGGNAVAVFKASSVILGVKD
NTSEQ       837
            atgacattgcaccgcaccgatccctcccgcgaaaccctcgaactcggcggctccgtgtgg
            tttcaggcgggcgcgcagacgctcggcggcgcgtcgcggatcgcgctgctcgcggcgatc
            ggcgagacgggttcgatcaccagcgcggccaaagcggtcggcatgagctacaaaggcgcg
            tgggacgcgatcgacacgatgaacaacctcgccggtgagccgctcgtggtgcgcctgacc
            ggcggcaagggcggcggcggcaccacgctgacgccgcgtgccgtgaagctgatcgagacg
            tttcgcgcggtggaacgcgagcaccggcgctttctcgaacgcgcgggcgcggcgatcgaa
            gggttcgcgaccgactgggatctgatcggccgcatcggcgtgaagacgagcgcgcgcaat
            caactgtatggcacggtgtcggcgatcacgcgcggcaccgtcaacgacgaagtcacactc
            gtgctgcccggcggccatgcgatcgtctccgtcatcacgcatgaaagcaccgaaacgctc
            ggcctcgtcgagggcgcggcggcgttcgcgttgatcaaggcgtcgtgggtcgtgctgttc
            gtcgacaaccaaagcggcgtgccgctcaaactctcggcgcgcaatcagttgcgcggcacg
            gtgcaaagcgtgaagcgcggcgcggtcaatgccgaggtgtcgctggtgttggcgggcggc
            gcggtgatcaccgctgtcgtcaccaatgaaagcgtagatacgctcgggctcgtggaaggc
            ggcaacgcagtggccgtgttcaaggcgtcgagtgtgatactcggcgtgaaggactga
///
```

  
**Homolog ID**: Table of closest homologs  

```
                 Homologs                                       len   identity overlap
---------------------------------------------------------------------------------
bpy:Bphyt_6807 ModE family transcriptional regulator    K     278     0.921    278 
bgf:BC1003_4563 putative transcriptional regulator, Mod K     278     0.860    278 
bge:BC1002_5248 ModE family transcriptional regulator   K     281     0.865    281 
bph:Bphy_4402 ModE family transcriptional regulator     K     276     0.707    276 
bur:Bcep18194_B2239 ModE family transcriptional regulat K     278     0.702    272 
bml:BMA10229_1671 transcriptional regulator ModE        K     282     0.690    274 
bmn:BMA10247_A0326 transcriptional regulator ModE       K     282     0.690    274 
bmv:BMASAVP1_1474 transcriptional regulator ModE        K     282     0.690    274 
bpd:BURPS668_A2569 transcriptional regulator ModE       K     282     0.690    274 
bpl:BURPS1106A_A2433 molybdenum transporter/regulator p K     282     0.690    274 
bch:Bcen2424_3857 ModE family transcriptional regulator K     281     0.712    264 
bcm:Bcenmc03_3670 ModE family transcriptional regulator K     278     0.712    264 
bcn:Bcen_4507 ModE family transcriptional regulator     K     281     0.712    264 
bpm:BURPS1710b_A0871 transcriptional regulator ModE     K     282     0.686    274 
bcj:BCAM0889 putative molybdenum transport protein      K     279     0.697    271 
bps:BPSS1789 molybdenum transport protein               K     262     0.698    262 
bma:BMAA0294 transcriptional regulator ModE             K     262     0.698    262 
bte:BTH_II0590 transcriptional regulator ModE           K     282     0.686    274 
bac:BamMC406_3754 ModE family transcriptional regulator K     279     0.705    264 
bam:Bamb_3225 ModE family transcriptional regulator     K     279     0.701    264 
bvi:Bcep1808_4233 ModE family transcriptional regulator K     283     0.684    272 
bmj:BMULJ_03627 molybdate transporter regulatory protei K     278     0.675    274 
bmu:Bmul_4888 ModE family transcriptional regulator     K     278     0.675    274 
cti:RALTA_A0628 transcriptional repressor for molybdate K     294     0.610    264 
reh:H16_A0672 transcriptional repressor-molybdenum-bind K     264     0.614    264 
reu:Reut_A0630 ModE family transcriptional regulator    K     290     0.606    264 
rme:Rmet_0567 DNA-binding transcriptional dual regulato K     268     0.599    267 
axy:AXYL_04387 molybdenum transporter ModE              K     264     0.595    269 
gpb:HDN1F_09840 molybdopterin-binding protein           K     282     0.537    272 
bpt:Bpet3267 molybdenum transporter                     K     315     0.606    269 
rpf:Rpic12D_4329 ModE family transcriptional regulator  K     270     0.565    271 
rpi:Rpic_4219 ModE family transcriptional regulator     K     270     0.565    271 
rso:RS05434 molybdenum-pterin-binding protein           K     269     0.567    270 
rsl:RPSI07_mp1183 transcriptional repressor for molybda K     269     0.559    270 
bbr:BB3353 molybdenum-binding protein                   K     268     0.537    270 
bpe:BP2071 molybdenum-binding protein                   K     268     0.537    270 
bav:BAV2465 regulator of molybdenum transport genes     K     267     0.544    270 
bpa:BPP1755 molybdenum-binding protein                  K     269     0.531    271 
gem:GM21_2529 ModE family transcriptional regulator     K     263     0.502    265 
alv:Alvin_0888 ModE family transcriptional regulator    K     270     0.504    268 
gbm:Gbem_1692 molybdate transport regulatory protein Mo K     271     0.498    265 
cli:Clim_0673 ModE family transcriptional regulator     K     270     0.494    267 
cch:Cag_1230 molybdenum-pterin binding protein          K     281     0.498    267 
gsu:GSU2964 molybdate transport regulatory protein ModE K     271     0.474    268 
glo:Glov_0447 ModE family transcriptional regulator     K     268     0.491    265 
pph:Ppha_1986 ModE family transcriptional regulator     K     269     0.478    268 
wsu:WS1811 molybdenum-pterin-binding protein            K     258     0.481    264 
cpc:Cpar_1630 ModE family transcriptional regulator     K     269     0.455    268 
lhk:LHK_02456 ModE                                      K     266     0.485    266 
cts:Ctha_1482 ModE family transcriptional regulator     K     283     0.493    268 
cte:CT1543 molybdenum transport protein ModE            K     269     0.451    268 
pvi:Cvib_1354 ModE family transcriptional regulator     K     270     0.470    266 
cph:Cpha266_0735 ModE family transcriptional regulator  K     269     0.466    266 
paa:Paes_1638 ModE family transcriptional regulator     K     269     0.470    264 
plt:Plut_1543 molybdenum-binding protein-like protein   K     270     0.468    267 
hch:HCH_02455 molybdenum-binding protein                K     272     0.467    270 
ppd:Ppro_1529 ModE family transcriptional regulator     K     269     0.454    271 
slt:Slit_0896 ModE family transcriptional regulator     K     268     0.473    243 
pca:Pcar_0662 molybdate transport regulatory protein Mo K     270     0.437    263 
psa:PST_1348 putative molybdenum-binding protein        K     268     0.445    265 
har:HEAR3440 transcriptional repressor for molybdate up K     300     0.469    243 
mmw:Mmwyl1_0976 ModE family transcriptional regulator   K     270     0.457    269 
mms:mma_3659 molybdenum transport regulatory protein Mo K     264     0.453    243 
tgr:Tgr7_1573 molybdenum-binding protein                K     265     0.428    264 
dar:Daro_1477 molybdenum-binding protein, N-terminal:mo K     272     0.478    253 
azo:azo3840 putative molybdenum transport protein       K     275     0.475    255 
tau:Tola_1199 ModE family transcriptional regulator     K     288     0.452    261 
tcx:Tcr_1928 ModE family transcriptional regulator      K     274     0.421    259 
pna:Pnap_2036 ModE family transcriptional regulator     K     276     0.443    271 
hna:Hneap_0810 ModE family transcriptional regulator    K     267     0.454    249 
rsq:Rsph17025_3358 molybdenum ABC transporter, periplas K     265     0.433    263 
avn:Avin_50680 Mo regulation, Mo processing homeostasis K     270     0.470    253 
rsp:RSP_3874 transcriptional repressor, ModE            K     262     0.432    264 
rsh:Rsph17029_4166 ModE family transcriptional regulato K     262     0.428    264 
rsk:RSKD131_4480 transcriptional regulator, ModE family K     262     0.428    264 
sdl:Sdel_0810 LysR family transcriptional regulator     K     260     0.440    243 
mgm:Mmc1_1731 ModE family transcriptional regulator     K     268     0.440    257 
rpc:RPC_0705 ModE family transcriptional regulator      K     263     0.415    248 
pag:PLES_04831 putative molybdenum transport regulator  K     252     0.439    244 
abu:Abu_0013 molybdenum-binding protein                 K     260     0.403    243 
pae:PA0487 molybdenum transport regulator               K     252     0.439    244 
pau:PA14_06340 molybdenum transport regulator           K     252     0.439    244 
rpa:RPA4718 ModE family transcriptional regulator       K     263     0.435    248 
rpt:Rpal_5199 ModE family transcriptional regulator     K     263     0.435    248 
mag:amb2949 molybdenum-pterin binding protein mopA      K     264     0.419    260 
pmy:Pmen_4091 ModE family transcriptional regulator     K     254     0.427    246 
cak:Caul_2731 ModE family transcriptional regulator     K     266     0.426    270 
pmx:PERMA_1919 molybdenum-pterin-binding protein        K     261     0.423    246 
rcp:RCAP_rcc00561 molybdenum transport operon repressor K     265     0.444    259 
cco:CCC13826_2203 molybdenum-pterin binding domain-cont K     260     0.404    255 
pap:PSPA7_0590 molybdenum transport regulator           K     252     0.434    244 
rpd:RPD_0962 molybdate transport repressor              K     263     0.403    248 
ppg:PputGB1_0390 ModE family transcriptional regulator  K     254     0.420    245 
ppw:PputW619_4842 ModE family transcriptional regulator K     254     0.424    245
```

**Neighborhood Representations**: Table of genes in the defined genetic neighborhoods of the entry protein and its closest homologs  
  
**Neighborhood Representations for "bxe:Bxe\_B2851"**  

| ID | Annotation | EC number |
| --- | --- | --- |
| bxe:Bxe\_B2861 | putative alpha-amylase-like protein; K16147 starch synthase (maltosyl-transferring) [EC:2.4.99.16] | ec:2.4.99.16 |
| bxe:Bxe\_B2860 | hypothetical protein |  |
| bxe:Bxe\_B2859 | hypothetical protein |  |
| bxe:Bxe\_B2858 | putative serine/threonine protein phosphatase (EC:3.1.3.16); K07313 serine/threonine protein phosphatase 1 [EC:3.1.3.16] | ec:3.1.3.16 |
| bxe:Bxe\_B2857 | hypothetical protein |  |
| bxe:Bxe\_B2856 | hypothetical protein |  |
| bxe:Bxe\_B2855 | hypothetical protein |  |
| bxe:Bxe\_B2854 | LysR family transcriptional regulator |  |
| bxe:Bxe\_B2853 | hypothetical protein |  |
| bxe:Bxe\_B2852 | putative short-chain dehydrogenase/reductase |  |
| bxe:Bxe\_B2851 | ModE family transcriptional regulator; K02019 molybdate transport system regulatory protein |  |
| bxe:Bxe\_B2850 | molybdate ABC transporter ATPase; K02017 molybdate transport system ATP-binding protein [EC:3.6.3.29] | ec:3.6.3.29 |
| bxe:Bxe\_B2849 | molybdate ABC transporter inner membrane subunit; K02018 molybdate transport system permease protein |  |
| bxe:Bxe\_B2848 | molybdate ABC transporter periplasmic ligand binding protein; K02020 molybdate transport system substrate-binding protein |  |
| bxe:Bxe\_B2847 | putative phosphoesterase, PA-phosphatase related |  |
| bxe:Bxe\_B2846 | esterase |  |
| bxe:Bxe\_B2845 | hypothetical protein |  |
| bxe:Bxe\_B2844 | deoxyribodipyrimidine photo-lyase type I (EC:4.1.99.3); K01669 deoxyribodipyrimidine photo-lyase [EC:4.1.99.3] | ec:4.1.99.3 |
| bxe:Bxe\_B2843 | organic hydroperoxide resistance protein |  |
| bxe:Bxe\_B2842 | MarR family transcriptional regulator |  |
| bxe:Bxe\_B2841 | putative transmembrane protein |  |

  
**Neighborhood Representations for "bpy:Bphyt\_6807"**  

| ID | Annotation | EC number |
| --- | --- | --- |
| bpy:Bphyt\_6797 | MarR family transcriptional regulator |  |
| bpy:Bphyt\_6798 | OsmC family protein |  |
| bpy:Bphyt\_6799 | deoxyribodipyrimidine photo-lyase (EC:4.1.99.3); K01669 deoxyribodipyrimidine photo-lyase [EC:4.1.99.3] | ec:4.1.99.3 |
| bpy:Bphyt\_6800 | TspO and MBR-like protein; K07185 tryptophan-rich sensory protein |  |
| bpy:Bphyt\_6801 | hypothetical protein |  |
| bpy:Bphyt\_6802 | esterase, PHB depolymerase family |  |
| bpy:Bphyt\_6803 | PA-phosphatase-like phosphoesterase |  |
| bpy:Bphyt\_6804 | molybdenum ABC transporter periplasmic molybdate-binding protein; K02020 molybdate transport system substrate-binding protein |  |
| bpy:Bphyt\_6805 | molybdate ABC transporter inner membrane subunit; K02018 molybdate transport system permease protein |  |
| bpy:Bphyt\_6806 | ABC transporter-like protein; K02017 molybdate transport system ATP-binding protein [EC:3.6.3.29] | ec:3.6.3.29 |
| bpy:Bphyt\_6807 | ModE family transcriptional regulator; K02019 molybdate transport system regulatory protein |  |
| bpy:Bphyt\_6808 | short-chain dehydrogenase/reductase SDR |  |
| bpy:Bphyt\_6809 | porin |  |
| bpy:Bphyt\_6810 | pseudogene |  |
| bpy:Bphyt\_6811 | salicylate esterase |  |
| bpy:Bphyt\_6812 | LysR family transcriptional regulator |  |
| bpy:Bphyt\_6813 | hypothetical protein |  |
| bpy:Bphyt\_6814 | hypothetical protein |  |
| bpy:Bphyt\_6815 | RES domain-containing protein |  |
| bpy:Bphyt\_6816 | metallophosphoesterase; K07313 serine/threonine protein phosphatase 1 [EC:3.1.3.16] | ec:3.1.3.16 |
| bpy:Bphyt\_6817 | gluconate 2-dehydrogenase (EC:1.1.99.3) |  |

  
**Neighborhood Representations for "bgf:BC1003\_4563"**  

| ID | Annotation | EC number |
| --- | --- | --- |
| bgf:BC1003\_4553 | hypothetical protein |  |
| bgf:BC1003\_4554 | homoserine kinase (EC:2.7.1.39); K02204 homoserine kinase type II [EC:2.7.1.39] | ec:2.7.1.39 |
| bgf:BC1003\_4555 | putative transmembrane protein |  |
| bgf:BC1003\_4556 | Deoxyribodipyrimidine photo-lyase (EC:4.1.99.3); K01669 deoxyribodipyrimidine photo-lyase [EC:4.1.99.3] | ec:4.1.99.3 |
| bgf:BC1003\_4557 | hypothetical protein |  |
| bgf:BC1003\_4558 | esterase, PHB depolymerase family |  |
| bgf:BC1003\_4559 | phosphoesterase PA-phosphatase related protein |  |
| bgf:BC1003\_4560 | molybdenum ABC transporter periplasmic molybdate-binding protein; K02020 molybdate transport system substrate-binding protein |  |
| bgf:BC1003\_4561 | molybdate ABC transporter inner membrane subunit; K02018 molybdate transport system permease protein |  |
| bgf:BC1003\_4562 | ABC transporter-like protein; K02017 molybdate transport system ATP-binding protein [EC:3.6.3.29] | ec:3.6.3.29 |
| bgf:BC1003\_4563 | putative transcriptional regulator, ModE family; K02019 molybdate transport system regulatory protein |  |
| bgf:BC1003\_4564 | 4-oxalocrotonate tautomerase |  |
| bgf:BC1003\_4565 | LysR family transcriptional regulator |  |
| bgf:BC1003\_4566 | hypothetical protein |  |
| bgf:BC1003\_4567 | hypothetical protein |  |
| bgf:BC1003\_4568 | RES domain-containing protein |  |
| bgf:BC1003\_4569 | metallophosphoesterase; K07313 serine/threonine protein phosphatase 1 [EC:3.1.3.16] | ec:3.1.3.16 |
| bgf:BC1003\_4570 | hypothetical protein |  |
| bgf:BC1003\_4571 | alpha amylase catalytic region; K16147 starch synthase (maltosyl-transferring) [EC:2.4.99.16] | ec:2.4.99.16 |
| bgf:BC1003\_4572 | trehalose synthase; K05343 maltose alpha-D-glucosyltransferase/ alpha-amylase [EC:5.4.99.16 3.2.1.1] | ec:3.2.1.1 ec:5.4.99.16 |
| bgf:BC1003\_4573 | 1,4-alpha-glucan branching enzyme; K00700 1,4-alpha-glucan branching enzyme [EC:2.4.1.18] | ec:2.4.1.18 |

  
**Neighborhood Representations for "bge:BC1002\_5248"**  

| ID | Annotation | EC number |
| --- | --- | --- |
| bge:BC1002\_5238 | hypothetical protein |  |
| bge:BC1002\_5239 | LysR family transcriptional regulator |  |
| bge:BC1002\_5240 | 4-oxalocrotonate tautomerase |  |
| bge:BC1002\_5241 | ArsR family transcriptional regulator |  |
| bge:BC1002\_5242 | protein-tyrosine phosphatase, low molecular weight; K03741 arsenate reductase [EC:1.20.4.1] | ec:1.20.4.1 |
| bge:BC1002\_5243 | arsenic resistance protein; K03325 arsenite transporter, ACR3 family |  |
| bge:BC1002\_5244 | pseudogene |  |
| bge:BC1002\_5245 | FAD-dependent pyridine nucleotide-disulfide oxidoreductase |  |
| bge:BC1002\_5246 | major facilitator superfamily protein |  |
| bge:BC1002\_5247 | hypothetical protein |  |
| bge:BC1002\_5248 | ModE family transcriptional regulator; K02019 molybdate transport system regulatory protein |  |
| bge:BC1002\_5249 | ABC transporter; K02017 molybdate transport system ATP-binding protein [EC:3.6.3.29] | ec:3.6.3.29 |
| bge:BC1002\_5250 | molybdate ABC transporter inner membrane subunit; K02018 molybdate transport system permease protein |  |
| bge:BC1002\_5251 | molybdenum ABC transporter periplasmic molybdate-binding protein; K02020 molybdate transport system substrate-binding protein |  |
| bge:BC1002\_5252 | phosphoesterase PA-phosphatase-like protein |  |
| bge:BC1002\_5253 | esterase, PHB depolymerase family |  |
| bge:BC1002\_5254 | hypothetical protein |  |
| bge:BC1002\_5255 | TspO and MBR-like protein; K07185 tryptophan-rich sensory protein |  |
| bge:BC1002\_5256 | deoxyribodipyrimidine photo-lyase (EC:4.1.99.3); K01669 deoxyribodipyrimidine photo-lyase [EC:4.1.99.3] | ec:4.1.99.3 |
| bge:BC1002\_5257 | Ohr subfamily peroxiredoxin |  |
| bge:BC1002\_5258 | MarR family transcriptional regulator |  |

  
**Neighborhood Representations for "bph:Bphy\_4402"**  

| ID | Annotation | EC number |
| --- | --- | --- |
| bph:Bphy\_4392 | trehalose synthase; K05343 maltose alpha-D-glucosyltransferase/ alpha-amylase [EC:5.4.99.16 3.2.1.1] | ec:3.2.1.1 ec:5.4.99.16 |
| bph:Bphy\_4393 | alpha amylase; K16147 starch synthase (maltosyl-transferring) [EC:2.4.99.16] | ec:2.4.99.16 |
| bph:Bphy\_4394 | hypothetical protein |  |
| bph:Bphy\_4395 | pseudogene |  |
| bph:Bphy\_4396 | metallophosphoesterase; K07313 serine/threonine protein phosphatase 1 [EC:3.1.3.16] | ec:3.1.3.16 |
| bph:Bphy\_4397 | RES domain-containing protein |  |
| bph:Bphy\_4398 | hypothetical protein |  |
| bph:Bphy\_4399 | hypothetical protein |  |
| bph:Bphy\_4400 | LysR family transcriptional regulator |  |
| bph:Bphy\_4401 | 4-oxalocrotonate tautomerase |  |
| bph:Bphy\_4402 | ModE family transcriptional regulator; K02019 molybdate transport system regulatory protein |  |
| bph:Bphy\_4403 | ABC transporter; K02017 molybdate transport system ATP-binding protein [EC:3.6.3.29] | ec:3.6.3.29 |
| bph:Bphy\_4404 | molybdate ABC transporter inner membrane subunit; K02018 molybdate transport system permease protein |  |
| bph:Bphy\_4405 | molybdenum ABC transporter periplasmic molybdate-binding protein; K02020 molybdate transport system substrate-binding protein |  |
| bph:Bphy\_4406 | PA-phosphatase like phosphoesterase |  |
| bph:Bphy\_4407 | PHB depolymerase family esterase |  |
| bph:Bphy\_4408 | hypothetical protein |  |
| bph:Bphy\_4409 | deoxyribodipyrimidine photo-lyase (EC:4.1.99.3); K01669 deoxyribodipyrimidine photo-lyase [EC:4.1.99.3] | ec:4.1.99.3 |
| bph:Bphy\_4410 | MarR family transcriptional regulator |  |
| bph:Bphy\_4411 | putative transmembrane protein |  |
| bph:Bphy\_4412 | homoserine kinase (EC:2.7.1.39); K02204 homoserine kinase type II [EC:2.7.1.39] | ec:2.7.1.39 |

  
**Neighborhood Representations for "bur:Bcep18194\_B2239"**  

| ID | Annotation | EC number |
| --- | --- | --- |
| bur:Bcep18194\_B2229 | homoserine kinase (EC:2.7.1.39); K02204 homoserine kinase type II [EC:2.7.1.39] | ec:2.7.1.39 |
| bur:Bcep18194\_B2230 | hypothetical protein |  |
| bur:Bcep18194\_B2231 | MarR family transcriptional regulator |  |
| bur:Bcep18194\_B2232 | OsmC-like protein |  |
| bur:Bcep18194\_B2233 | hypothetical protein |  |
| bur:Bcep18194\_B2234 | esterase (EC:3.1.1.73); K09252 feruloyl esterase [EC:3.1.1.73] | ec:3.1.1.73 |
| bur:Bcep18194\_B2235 | phosphoesterase, PA-phosphatase related |  |
| bur:Bcep18194\_B2236 | molybdenum ABC transporter periplasmic-binding protein; K02020 molybdate transport system substrate-binding protein |  |
| bur:Bcep18194\_B2237 | molybdate ABC transporter inner membrane protein; K02018 molybdate transport system permease protein |  |
| bur:Bcep18194\_B2238 | ABC molybdate transporter, ATPase subunit (EC:3.6.3.25); K02017 molybdate transport system ATP-binding protein [EC:3.6.3.29] | ec:3.6.3.29 |
| bur:Bcep18194\_B2239 | ModE family transcriptional regulator; K02019 molybdate transport system regulatory protein |  |
| bur:Bcep18194\_B2240 | hypothetical protein |  |
| bur:Bcep18194\_B2241 | 4-oxalocrotonate tautomerase |  |
| bur:Bcep18194\_B2242 | LysR family transcriptional regulator |  |
| bur:Bcep18194\_B2243 | hypothetical protein |  |
| bur:Bcep18194\_B2244 | histidine kinase (EC:2.7.3.-) |  |
| bur:Bcep18194\_B2245 | hypothetical protein |  |
| bur:Bcep18194\_B2246 | thiamine monophosphate synthase (EC:2.5.1.3); K00788 thiamine-phosphate pyrophosphorylase [EC:2.5.1.3] | ec:2.5.1.3 |
| bur:Bcep18194\_B2247 | glycoside hydrolase (EC:3.2.1.141); K01236 maltooligosyltrehalose trehalohydrolase [EC:3.2.1.141] | ec:3.2.1.141 |
| bur:Bcep18194\_B2248 | hypothetical protein; K15256 tRNA (cmo5U34)-methyltransferase [EC:2.1.1.-] |  |
| bur:Bcep18194\_B2249 | glutathione S-transferase-like protein (EC:2.5.1.18); K00799 glutathione S-transferase [EC:2.5.1.18] | ec:2.5.1.18 |

  
**Neighborhood Representations for "bml:BMA10229\_1671"**  

| ID | Annotation | EC number |
| --- | --- | --- |
| bml:BMA10229\_1661 | ebsC protein |  |
| bml:BMA10229\_1662 | hypothetical protein |  |
| bml:BMA10229\_1663 | alpha amylase |  |
| bml:BMA10229\_1664 | thiamine-phosphate pyrophosphorylase ThiE; K00788 thiamine-phosphate pyrophosphorylase [EC:2.5.1.3] | ec:2.5.1.3 |
| bml:BMA10229\_1665 | hypothetical protein |  |
| bml:BMA10229\_1666 | putatitve sensor histidine kinase |  |
| bml:BMA10229\_1667 | hypothetical protein |  |
| bml:BMA10229\_1668 | LysR family transcriptional regulator |  |
| bml:BMA10229\_1669 | 4-oxalocrotonate tautomerase; K01821 4-oxalocrotonate tautomerase [EC:5.3.2.6] | ec:5.3.2.6 |
| bml:BMA10229\_1670 | hypothetical protein |  |
| bml:BMA10229\_1671 | modE; transcriptional regulator ModE; K02019 molybdate transport system regulatory protein |  |
| bml:BMA10229\_1672 | modC; molybdate ABC transporter ATP-binding protein; K02017 molybdate transport system ATP-binding protein [EC:3.6.3.29] | ec:3.6.3.29 |
| bml:BMA10229\_1673 | modB; molybdenum ABC transporter permease; K02018 molybdate transport system permease protein |  |
| bml:BMA10229\_1674 | modA; molybdenum ABC transporter periplasmic molybdate-binding protein; K02020 molybdate transport system substrate-binding protein |  |
| bml:BMA10229\_1675 | PAP2 family protein |  |
| bml:BMA10229\_1676 | polyhydroxybutyrate depolymerase domain-containing protein; K01066 esterase / lipase [EC:3.1.1.-] |  |
| bml:BMA10229\_1677 | hypothetical protein |  |
| bml:BMA10229\_1678 | ohr; organic hydroperoxide resistance protein |  |
| bml:BMA10229\_1679 | MarR family transcriptional regulator |  |
| bml:BMA10229\_1680 | hypothetical protein |  |
| bml:BMA10229\_1681 | hypothetical protein |  |

  
**Neighborhood Representations for "bmn:BMA10247\_A0326"**  

| ID | Annotation | EC number |
| --- | --- | --- |
| bmn:BMA10247\_A0316 | putative ebsC protein |  |
| bmn:BMA10247\_A0317 | pseudogene |  |
| bmn:BMA10247\_A0318 | alpha amylase family protein |  |
| bmn:BMA10247\_A0319 | putative thiamine-phosphate pyrophosphorylase ThiE; K00788 thiamine-phosphate pyrophosphorylase [EC:2.5.1.3] | ec:2.5.1.3 |
| bmn:BMA10247\_A0320 | CBS domain-containing protein |  |
| bmn:BMA10247\_A0321 | putatitve sensor histidine kinase |  |
| bmn:BMA10247\_A0322 | hypothetical protein |  |
| bmn:BMA10247\_A0323 | LysR family transcriptional regulator |  |
| bmn:BMA10247\_A0324 | 4-oxalocrotonate tautomerase (EC:5.3.2.-); K01821 4-oxalocrotonate tautomerase [EC:5.3.2.6] | ec:5.3.2.6 |
| bmn:BMA10247\_A0325 | CHAD domain-containing protein |  |
| bmn:BMA10247\_A0326 | modE; transcriptional regulator ModE; K02019 molybdate transport system regulatory protein |  |
| bmn:BMA10247\_A0327 | hypothetical protein |  |
| bmn:BMA10247\_A0328 | modC; putative molybdate ABC transporter ATP-binding protein; K02017 molybdate transport system ATP-binding protein [EC:3.6.3.29] | ec:3.6.3.29 |
| bmn:BMA10247\_A0329 | modB; molybdenum ABC transporter permease; K02018 molybdate transport system permease protein |  |
| bmn:BMA10247\_A0330 | modA; molybdenum ABC transporter periplasmic molybdate-binding protein; K02020 molybdate transport system substrate-binding protein |  |
| bmn:BMA10247\_A0331 | PAP2 family membrane protein |  |
| bmn:BMA10247\_A0332 | polyhydroxybutyrate depolymerase domain-containing protein; K01066 esterase / lipase [EC:3.1.1.-] |  |
| bmn:BMA10247\_A0333 | hypothetical protein |  |
| bmn:BMA10247\_A0334 | ohr; organic hydroperoxide resistance protein |  |
| bmn:BMA10247\_A0335 | MarR family transcriptional regulator |  |
| bmn:BMA10247\_A0336 | hypothetical protein |  |

  
**Neighborhood Representations for "bmv:BMASAVP1\_1474"**  

| ID | Annotation | EC number |
| --- | --- | --- |
| bmv:BMASAVP1\_1464 | ebsC protein |  |
| bmv:BMASAVP1\_1465 | SAM-dependent methyltransferase |  |
| bmv:BMASAVP1\_1466 | alpha amylase |  |
| bmv:BMASAVP1\_1467 | thiamine-phosphate pyrophosphorylase ThiE; K00788 thiamine-phosphate pyrophosphorylase [EC:2.5.1.3] | ec:2.5.1.3 |
| bmv:BMASAVP1\_1468 | hypothetical protein |  |
| bmv:BMASAVP1\_1469 | putatitve sensor histidine kinase |  |
| bmv:BMASAVP1\_1470 | hypothetical protein |  |
| bmv:BMASAVP1\_1471 | LysR family transcriptional regulator |  |
| bmv:BMASAVP1\_1472 | 4-oxalocrotonate tautomerase; K01821 4-oxalocrotonate tautomerase [EC:5.3.2.6] | ec:5.3.2.6 |
| bmv:BMASAVP1\_1473 | CHAD domain-containing protein |  |
| bmv:BMASAVP1\_1474 | modE; transcriptional regulator ModE; K02019 molybdate transport system regulatory protein |  |
| bmv:BMASAVP1\_1475 | hypothetical protein |  |
| bmv:BMASAVP1\_1476 | modC; molybdate ABC transporter ATP-binding protein; K02017 molybdate transport system ATP-binding protein [EC:3.6.3.29] | ec:3.6.3.29 |
| bmv:BMASAVP1\_1477 | modB; molybdenum ABC transporter permease; K02018 molybdate transport system permease protein |  |
| bmv:BMASAVP1\_1478 | modA; molybdenum ABC transporter periplasmic molybdate-binding protein; K02020 molybdate transport system substrate-binding protein |  |
| bmv:BMASAVP1\_1479 | PAP2 (2 phosphatidic acid phosphatase) family protein |  |
| bmv:BMASAVP1\_1480 | polyhydroxybutyrate depolymerase domain-containing protein; K01066 esterase / lipase [EC:3.1.1.-] |  |
| bmv:BMASAVP1\_1481 | hypothetical protein |  |
| bmv:BMASAVP1\_1482 | ohr; organic hydroperoxide resistance protein |  |
| bmv:BMASAVP1\_1483 | MarR family transcriptional regulator |  |
| bmv:BMASAVP1\_1484 | hypothetical protein |  |

  
**Neighborhood Representations for "bpd:BURPS668\_A2569"**  

| ID | Annotation | EC number |
| --- | --- | --- |
| bpd:BURPS668\_A2558 | hypothetical protein |  |
| bpd:BURPS668\_A2559 | transcriptional regulator |  |
| bpd:BURPS668\_A2560 | hypothetical protein |  |
| bpd:BURPS668\_A2561 | hypothetical protein |  |
| bpd:BURPS668\_A2562 | depolymerase; K01066 esterase / lipase [EC:3.1.1.-] |  |
| bpd:BURPS668\_A2563 | major facilitator superfamily permease |  |
| bpd:BURPS668\_A2564 | modA; molybdate ABC transporter periplasmic molybdate-binding protein; K02020 molybdate transport system substrate-binding protein |  |
| bpd:BURPS668\_A2565 | modB; molybdate ABC transporter permease; K02018 molybdate transport system permease protein |  |
| bpd:BURPS668\_A2566 | molybdenum ABC transporter ATP-binding protein; K02017 molybdate transport system ATP-binding protein [EC:3.6.3.29] | ec:3.6.3.29 |
| bpd:BURPS668\_A2567 | hypothetical protein |  |
| bpd:BURPS668\_A2569 | modE; transcriptional regulator ModE; K02019 molybdate transport system regulatory protein |  |
| bpd:BURPS668\_A2570 | CHAD domain-contain protein |  |
| bpd:BURPS668\_A2571 | tautomerase family protein; K01821 4-oxalocrotonate tautomerase [EC:5.3.2.6] | ec:5.3.2.6 |
| bpd:BURPS668\_A2572 | transcriptional regulator |  |
| bpd:BURPS668\_A2573 | multidrug ABC transporter permease |  |
| bpd:BURPS668\_A2574 | Signal transduction histidine kinase |  |
| bpd:BURPS668\_A2575 | CBS domain-containing protein |  |
| bpd:BURPS668\_A2576 | thiamine monophosphate synthase; K00788 thiamine-phosphate pyrophosphorylase [EC:2.5.1.3] | ec:2.5.1.3 |
| bpd:BURPS668\_A2577 | alpha amylase family protein |  |
| bpd:BURPS668\_A2578 | SAM-dependent methyltransferases; K15256 tRNA (cmo5U34)-methyltransferase [EC:2.1.1.-] |  |
| bpd:BURPS668\_A2579 | YbaK protein |  |

  
**Neighborhood Representations for "bpl:BURPS1106A\_A2433"**  

| ID | Annotation | EC number |
| --- | --- | --- |
| bpl:BURPS1106A\_A2423 | organic hydroperoxide resistance protein |  |
| bpl:BURPS1106A\_A2424 | hypothetical protein |  |
| bpl:BURPS1106A\_A2425 | PHB depolymerase family esterase (EC:3.1.1.-); K01066 esterase / lipase [EC:3.1.1.-] |  |
| bpl:BURPS1106A\_A2426 | PAP2 family membrane protein |  |
| bpl:BURPS1106A\_A2427 | modA; molybdate ABC transporter periplasmic molybdate-binding protein; K02020 molybdate transport system substrate-binding protein |  |
| bpl:BURPS1106A\_A2428 | modB; molybdate ABC transporter permease; K02018 molybdate transport system permease protein |  |
| bpl:BURPS1106A\_A2429 | putative molybdate ABC transporter ATP-binding protein; K02017 molybdate transport system ATP-binding protein [EC:3.6.3.29] | ec:3.6.3.29 |
| bpl:BURPS1106A\_A2431 | hypothetical protein |  |
| bpl:BURPS1106A\_A2430 | hypothetical protein |  |
| bpl:BURPS1106A\_A2432 | hypothetical protein |  |
| bpl:BURPS1106A\_A2433 | modE; molybdenum transporter/regulator protein ModE; K02019 molybdate transport system regulatory protein |  |
| bpl:BURPS1106A\_A2434 | CHAD domain-containing protein |  |
| bpl:BURPS1106A\_A2435 | 4-oxalocrotonate tautomerase (EC:5.3.2.-); K01821 4-oxalocrotonate tautomerase [EC:5.3.2.6] | ec:5.3.2.6 |
| bpl:BURPS1106A\_A2436 | LysR family transcriptional regulator |  |
| bpl:BURPS1106A\_A2437 | hypothetical protein |  |
| bpl:BURPS1106A\_A2438 | putative sensor histidine kinase |  |
| bpl:BURPS1106A\_A2439 | putative transporter |  |
| bpl:BURPS1106A\_A2440 | putative thiamine-phosphate pyrophosphorylase ThiE; K00788 thiamine-phosphate pyrophosphorylase [EC:2.5.1.3] | ec:2.5.1.3 |
| bpl:BURPS1106A\_A2441 | putative glycosyl hydrolase |  |
| bpl:BURPS1106A\_A2442 | hypothetical protein; K15256 tRNA (cmo5U34)-methyltransferase [EC:2.1.1.-] |  |
| bpl:BURPS1106A\_A2443 | YbaK/prolyl-tRNA synthetase domain-containing protein |  |

  
**Neighborhood Representations for "bch:Bcen2424\_3857"**  

| ID | Annotation | EC number |
| --- | --- | --- |
| bch:Bcen2424\_3847 | glutathione S-transferase domain-containing protein; K00799 glutathione S-transferase [EC:2.5.1.18] | ec:2.5.1.18 |
| bch:Bcen2424\_3848 | methyltransferase type 11; K15256 tRNA (cmo5U34)-methyltransferase [EC:2.1.1.-] |  |
| bch:Bcen2424\_3849 | glycoside hydrolase family protein |  |
| bch:Bcen2424\_3850 | thiamine monophosphate synthase; K00788 thiamine-phosphate pyrophosphorylase [EC:2.5.1.3] | ec:2.5.1.3 |
| bch:Bcen2424\_3851 | hypothetical protein |  |
| bch:Bcen2424\_3852 | histidine kinase |  |
| bch:Bcen2424\_3853 | hypothetical protein |  |
| bch:Bcen2424\_3854 | LysR family transcriptional regulator |  |
| bch:Bcen2424\_3855 | 4-oxalocrotonate tautomerase |  |
| bch:Bcen2424\_3856 | CHAD domain-containing protein |  |
| bch:Bcen2424\_3857 | ModE family transcriptional regulator; K02019 molybdate transport system regulatory protein |  |
| bch:Bcen2424\_3858 | ABC transporter; K02017 molybdate transport system ATP-binding protein [EC:3.6.3.29] | ec:3.6.3.29 |
| bch:Bcen2424\_3859 | molybdate ABC transporter inner membrane subunit; K02018 molybdate transport system permease protein |  |
| bch:Bcen2424\_3860 | molybdenum ABC transporter, periplasmic molybdate-binding protein; K02020 molybdate transport system substrate-binding protein |  |
| bch:Bcen2424\_3861 | PA-phosphatase-like phosphoesterase |  |
| bch:Bcen2424\_3862 | PHB depolymerase family esterase |  |
| bch:Bcen2424\_3863 | hypothetical protein |  |
| bch:Bcen2424\_3864 | OsmC family protein |  |
| bch:Bcen2424\_3865 | MarR family transcriptional regulator |  |
| bch:Bcen2424\_3866 | hypothetical protein |  |
| bch:Bcen2424\_3867 | homoserine kinase (EC:2.7.1.39); K02204 homoserine kinase type II [EC:2.7.1.39] | ec:2.7.1.39 |

  
**Neighborhood Representations for "bcm:Bcenmc03\_3670"**  

| ID | Annotation | EC number |
| --- | --- | --- |
| bcm:Bcenmc03\_3660 | homoserine kinase (EC:2.7.1.39); K02204 homoserine kinase type II [EC:2.7.1.39] | ec:2.7.1.39 |
| bcm:Bcenmc03\_3661 | hypothetical protein |  |
| bcm:Bcenmc03\_3662 | MarR family transcriptional regulator |  |
| bcm:Bcenmc03\_3663 | OsmC family protein |  |
| bcm:Bcenmc03\_3664 | hypothetical protein |  |
| bcm:Bcenmc03\_3665 | PHB depolymerase family esterase |  |
| bcm:Bcenmc03\_3666 | PA-phosphatase-like protein |  |
| bcm:Bcenmc03\_3667 | molybdenum ABC transporter periplasmic molybdate-binding protein; K02020 molybdate transport system substrate-binding protein |  |
| bcm:Bcenmc03\_3668 | molybdate ABC transporter inner membrane subunit; K02018 molybdate transport system permease protein |  |
| bcm:Bcenmc03\_3669 | ABC transporter-like protein; K02017 molybdate transport system ATP-binding protein [EC:3.6.3.29] | ec:3.6.3.29 |
| bcm:Bcenmc03\_3670 | ModE family transcriptional regulator; K02019 molybdate transport system regulatory protein |  |
| bcm:Bcenmc03\_3671 | CHAD domain-containing protein |  |
| bcm:Bcenmc03\_3672 | 4-oxalocrotonate tautomerase |  |
| bcm:Bcenmc03\_3673 | LysR family transcriptional regulator |  |
| bcm:Bcenmc03\_3674 | hypothetical protein |  |
| bcm:Bcenmc03\_3675 | histidine kinase |  |
| bcm:Bcenmc03\_3676 | hypothetical protein |  |
| bcm:Bcenmc03\_3677 | thiamine monophosphate synthase; K00788 thiamine-phosphate pyrophosphorylase [EC:2.5.1.3] | ec:2.5.1.3 |
| bcm:Bcenmc03\_3678 | glycoside hydrolase family 13 protein |  |
| bcm:Bcenmc03\_3679 | type 11 methyltransferase; K15256 tRNA (cmo5U34)-methyltransferase [EC:2.1.1.-] |  |
| bcm:Bcenmc03\_3680 | glutathione S-transferase domain-containing protein; K00799 glutathione S-transferase [EC:2.5.1.18] | ec:2.5.1.18 |

  
**Neighborhood Representations for "bcn:Bcen\_4507"**  

| ID | Annotation | EC number |
| --- | --- | --- |
| bcn:Bcen\_4497 | homoserine kinase (EC:2.7.1.39); K02204 homoserine kinase type II [EC:2.7.1.39] | ec:2.7.1.39 |
| bcn:Bcen\_4498 | hypothetical protein |  |
| bcn:Bcen\_4499 | MarR family transcriptional regulator |  |
| bcn:Bcen\_4500 | OsmC-like protein |  |
| bcn:Bcen\_4501 | hypothetical protein |  |
| bcn:Bcen\_4502 | esterase |  |
| bcn:Bcen\_4503 | PA-phosphatase-like phosphoesterase |  |
| bcn:Bcen\_4504 | molybdenum ABC transporter, periplasmic molybdate-binding protein; K02020 molybdate transport system substrate-binding protein |  |
| bcn:Bcen\_4505 | molybdate ABC transporter permease; K02018 molybdate transport system permease protein |  |
| bcn:Bcen\_4506 | ABC transporter; K02017 molybdate transport system ATP-binding protein [EC:3.6.3.29] | ec:3.6.3.29 |
| bcn:Bcen\_4507 | ModE family transcriptional regulator; K02019 molybdate transport system regulatory protein |  |
| bcn:Bcen\_4508 | CHAD domain-containing protein |  |
| bcn:Bcen\_4509 | 4-oxalocrotonate tautomerase |  |
| bcn:Bcen\_4510 | LysR family transcriptional regulator |  |
| bcn:Bcen\_4511 | hypothetical protein |  |
| bcn:Bcen\_4512 | histidine kinase |  |
| bcn:Bcen\_4513 | hypothetical protein |  |
| bcn:Bcen\_4514 | thiamine monophosphate synthase; K00788 thiamine-phosphate pyrophosphorylase [EC:2.5.1.3] | ec:2.5.1.3 |
| bcn:Bcen\_4515 | glycoside hydrolase family protein |  |
| bcn:Bcen\_4516 | methyltransferase type 11; K15256 tRNA (cmo5U34)-methyltransferase [EC:2.1.1.-] |  |
| bcn:Bcen\_4517 | glutathione S-transferase; K00799 glutathione S-transferase [EC:2.5.1.18] | ec:2.5.1.18 |

  
**Neighborhood Representations for "bpm:BURPS1710b\_A0871"**  

| ID | Annotation | EC number |
| --- | --- | --- |
| bpm:BURPS1710b\_A0860 | hypothetical protein |  |
| bpm:BURPS1710b\_A0862 | MarR family transcriptional regulator |  |
| bpm:BURPS1710b\_A0863 | ohr; organic hydroperoxide resistance protein |  |
| bpm:BURPS1710b\_A0864 | hypothetical protein |  |
| bpm:BURPS1710b\_A0865 | phbZ; polyhydroxybutyrate depolymerase domain-containing protein; K01066 esterase / lipase [EC:3.1.1.-] |  |
| bpm:BURPS1710b\_A0866 | PAP2 family protein |  |
| bpm:BURPS1710b\_A0867 | modA; molybdenum ABC transporter periplasmic molybdate-binding protein; K02020 molybdate transport system substrate-binding protein |  |
| bpm:BURPS1710b\_A0868 | modB; molybdate ABC transporter permease; K02018 molybdate transport system permease protein |  |
| bpm:BURPS1710b\_A0869 | modC; molybdenum ABC transporter ATP-binding protein; K02017 molybdate transport system ATP-binding protein [EC:3.6.3.29] | ec:3.6.3.29 |
| bpm:BURPS1710b\_A0870 | hypothetical protein |  |
| bpm:BURPS1710b\_A0871 | modE; transcriptional regulator ModE; K02019 molybdate transport system regulatory protein |  |
| bpm:BURPS1710b\_A0872 | CHAD domain-contain protein |  |
| bpm:BURPS1710b\_A0873 | tautomerase family protein; K01821 4-oxalocrotonate tautomerase [EC:5.3.2.6] | ec:5.3.2.6 |
| bpm:BURPS1710b\_A0874 | LysR family transcriptional regulator |  |
| bpm:BURPS1710b\_A0875 | hypothetical protein |  |
| bpm:BURPS1710b\_A0876 | sensor histidine kinase |  |
| bpm:BURPS1710b\_A0877 | hypothetical protein |  |
| bpm:BURPS1710b\_A0878 | thiE2; dGTP-pyrophosphohydrolase; thiamine phosphate synthase; K00788 thiamine-phosphate pyrophosphorylase [EC:2.5.1.3] | ec:2.5.1.3 |
| bpm:BURPS1710b\_A0879 | alpha amylase; K00700 1,4-alpha-glucan branching enzyme [EC:2.4.1.18] | ec:2.4.1.18 |
| bpm:BURPS1710b\_A0880 | hypothetical protein; K15256 tRNA (cmo5U34)-methyltransferase [EC:2.1.1.-] |  |
| bpm:BURPS1710b\_A0881 | prolyl-tRNA synthetase |  |

  
**Neighborhood Representations for "bcj:BCAM0889"**  

| ID | Annotation | EC number |
| --- | --- | --- |
| bcj:BCAM0879 | putative glutathione S-transferase; K00799 glutathione S-transferase [EC:2.5.1.18] | ec:2.5.1.18 |
| bcj:BCAM0880 | putative methyltransferase; K15256 tRNA (cmo5U34)-methyltransferase [EC:2.1.1.-] |  |
| bcj:BCAM0881 | putative alpha amylase-family protein |  |
| bcj:BCAM0882 | hypothetical protein; K00788 thiamine-phosphate pyrophosphorylase [EC:2.5.1.3] | ec:2.5.1.3 |
| bcj:BCAM0883 | putative ion transporter |  |
| bcj:BCAM0884 | two-component regulatory system sensor kinase |  |
| bcj:BCAM0885 | hypothetical protein |  |
| bcj:BCAM0886 | LysR family regulatory protein |  |
| bcj:BCAM0887 | putative tautomerase |  |
| bcj:BCAM0888 | hypothetical protein |  |
| bcj:BCAM0889 | modE; putative molybdenum transport protein; K02019 molybdate transport system regulatory protein |  |
| bcj:BCAM0890 | ABC transporter ATP-binding protein; K02017 molybdate transport system ATP-binding protein [EC:3.6.3.29] | ec:3.6.3.29 |
| bcj:BCAM0891 | putative molybdenum transport system permease; K02018 molybdate transport system permease protein |  |
| bcj:BCAM0892 | putative molybdate-binding periplasmic protein precursor; K02020 molybdate transport system substrate-binding protein |  |
| bcj:BCAM0893 | PAP2 superfamily protein |  |
| bcj:BCAM0894 | poly(3-hydroxyalkanoate) depolymerase C precursor |  |
| bcj:BCAM0895 | hypothetical protein |  |
| bcj:BCAM0896 | putative organic hydroperoxide resistance protein |  |
| bcj:BCAM0897 | MarR family regulatory protein |  |
| bcj:BCAM0898 | hypothetical protein |  |
| bcj:BCAM0899 | homoserine kinase; K02204 homoserine kinase type II [EC:2.7.1.39] | ec:2.7.1.39 |

  
**Neighborhood Representations for "bps:BPSS1789"**  

| ID | Annotation | EC number |
| --- | --- | --- |
| bps:BPSS1779 | thrB; homoserine kinase (EC:2.7.1.39); K02204 homoserine kinase type II [EC:2.7.1.39] | ec:2.7.1.39 |
| bps:BPSS1780 | hypothetical protein |  |
| bps:BPSS1781 | MarR family transcriptional regulator |  |
| bps:BPSS1782 | ohr; organic hydroperoxide resistance protein |  |
| bps:BPSS1783 | hypothetical protein |  |
| bps:BPSS1784 | depolymerase; K01066 esterase / lipase [EC:3.1.1.-] |  |
| bps:BPSS1785 | hypothetical protein |  |
| bps:BPSS1786 | molybdenum transport-related, exported protein; K02020 molybdate transport system substrate-binding protein |  |
| bps:BPSS1787 | molybdenum transport-related membrane protein; K02018 molybdate transport system permease protein |  |
| bps:BPSS1788 | molybdenum transport-related, ATP-binding protein; K02017 molybdate transport system ATP-binding protein [EC:3.6.3.29] | ec:3.6.3.29 |
| bps:BPSS1789 | modE; molybdenum transport protein; K02019 molybdate transport system regulatory protein |  |
| bps:BPSS1790 | hypothetical protein |  |
| bps:BPSS1791 | hypothetical protein; K01821 4-oxalocrotonate tautomerase [EC:5.3.2.6] | ec:5.3.2.6 |
| bps:BPSS1792 | LysR family transcriptional regulator |  |
| bps:BPSS1793 | hypothetical protein |  |
| bps:BPSS1794 | hypothetical protein |  |
| bps:BPSS1795 | hypothetical protein |  |
| bps:BPSS1796 | hypothetical protein; K00788 thiamine-phosphate pyrophosphorylase [EC:2.5.1.3] | ec:2.5.1.3 |
| bps:BPSS1797 | trehalose trehalohydrolase |  |
| bps:BPSS1798 | hypothetical protein; K15256 tRNA (cmo5U34)-methyltransferase [EC:2.1.1.-] |  |
| bps:BPSS1799 | hypothetical protein |  |

  
**Neighborhood Representations for "bma:BMAA0294"**  

| ID | Annotation | EC number |
| --- | --- | --- |
| bma:BMAA0284 | ebsC protein |  |
| bma:BMAA0285 | pseudogene |  |
| bma:BMAA0286 | alpha amylase |  |
| bma:BMAA0287 | thiamine-phosphate pyrophosphorylase ThiE; K00788 thiamine-phosphate pyrophosphorylase [EC:2.5.1.3] | ec:2.5.1.3 |
| bma:BMAA0288 | hypothetical protein |  |
| bma:BMAA0289 | sensor histidine kinase |  |
| bma:BMAA0290 | hypothetical protein |  |
| bma:BMAA0291 | LysR family transcriptional regulator |  |
| bma:BMAA0292 | tautomerase enzyme family protein; K01821 4-oxalocrotonate tautomerase [EC:5.3.2.6] | ec:5.3.2.6 |
| bma:BMAA0293 | hypothetical protein |  |
| bma:BMAA0294 | modE; transcriptional regulator ModE; K02019 molybdate transport system regulatory protein |  |
| bma:BMAA0295 | hypothetical protein |  |
| bma:BMAA0296 | hypothetical protein |  |
| bma:BMAA0297 | modC; molybdenum ABC transporter ATP-binding protein; K02017 molybdate transport system ATP-binding protein [EC:3.6.3.29] | ec:3.6.3.29 |
| bma:BMAA0298 | modB; molybdenum ABC transporter permease; K02018 molybdate transport system permease protein |  |
| bma:BMAA0299 | modA; molybdenum ABC transporter periplasmic molybdate-binding protein; K02020 molybdate transport system substrate-binding protein |  |
| bma:BMAA0300 | PAP2 family protein |  |
| bma:BMAA0301 | polyhydroxybutyrate depolymerase domain-containing protein; K01066 esterase / lipase [EC:3.1.1.-] |  |
| bma:BMAA0302 | hypothetical protein |  |
| bma:BMAA0303 | ohr; organic hydroperoxide resistance protein |  |
| bma:BMAA0304 | MarR family transcriptional regulator |  |

  
**Neighborhood Representations for "bte:BTH\_II0590"**  

| ID | Annotation | EC number |
| --- | --- | --- |
| bte:BTH\_II0580 | ebsC protein |  |
| bte:BTH\_II0581 | hypothetical protein; K15256 tRNA (cmo5U34)-methyltransferase [EC:2.1.1.-] |  |
| bte:BTH\_II0582 | alpha amylase |  |
| bte:BTH\_II0583 | thiamine-phosphate pyrophosphorylase ThiE; K00788 thiamine-phosphate pyrophosphorylase [EC:2.5.1.3] | ec:2.5.1.3 |
| bte:BTH\_II0584 | hypothetical protein |  |
| bte:BTH\_II0585 | sensor histidine kinase |  |
| bte:BTH\_II0586 | hypothetical protein |  |
| bte:BTH\_II0587 | LysR family transcriptional regulator |  |
| bte:BTH\_II0588 | tautomerase enzyme family protein; K01821 4-oxalocrotonate tautomerase [EC:5.3.2.6] | ec:5.3.2.6 |
| bte:BTH\_II0589 | CHAD domain-contain protein |  |
| bte:BTH\_II0590 | transcriptional regulator ModE; K02019 molybdate transport system regulatory protein |  |
| bte:BTH\_II0591 | molybdenum ABC transporter ATP-binding protein; K02017 molybdate transport system ATP-binding protein [EC:3.6.3.29] | ec:3.6.3.29 |
| bte:BTH\_II0592 | modB; molybdate ABC transporter permease; K02018 molybdate transport system permease protein |  |
| bte:BTH\_II0593 | molybdenum ABC transporter periplasmic molybdate-binding protein; K02020 molybdate transport system substrate-binding protein |  |
| bte:BTH\_II0594 | PAP2 family protein |  |
| bte:BTH\_II0595 | polyhydroxybutyrate depolymerase domain-containing protein |  |
| bte:BTH\_II0596 | hypothetical protein |  |
| bte:BTH\_II0597 | organic hydroperoxide resistance protein |  |
| bte:BTH\_II0598 | MarR family transcriptional regulator |  |
| bte:BTH\_II0599 | hypothetical protein |  |
| bte:BTH\_II0600 | thrB; homoserine kinase (EC:2.7.1.39); K02204 homoserine kinase type II [EC:2.7.1.39] | ec:2.7.1.39 |

  
**Neighborhood Representations for "bac:BamMC406\_3754"**  

| ID | Annotation | EC number |
| --- | --- | --- |
| bac:BamMC406\_3744 | glutathione S-transferase domain-containing protein; K00799 glutathione S-transferase [EC:2.5.1.18] | ec:2.5.1.18 |
| bac:BamMC406\_3745 | type 11 methyltransferase; K15256 tRNA (cmo5U34)-methyltransferase [EC:2.1.1.-] |  |
| bac:BamMC406\_3746 | glycoside hydrolase family 13 protein |  |
| bac:BamMC406\_3747 | thiamine monophosphate synthase; K00788 thiamine-phosphate pyrophosphorylase [EC:2.5.1.3] | ec:2.5.1.3 |
| bac:BamMC406\_3748 | hypothetical protein |  |
| bac:BamMC406\_3749 | histidine kinase |  |
| bac:BamMC406\_3750 | hypothetical protein |  |
| bac:BamMC406\_3751 | LysR family transcriptional regulator |  |
| bac:BamMC406\_3752 | 4-oxalocrotonate tautomerase |  |
| bac:BamMC406\_3753 | CHAD domain-containing protein |  |
| bac:BamMC406\_3754 | ModE family transcriptional regulator; K02019 molybdate transport system regulatory protein |  |
| bac:BamMC406\_3755 | ABC transporter-like protein; K02017 molybdate transport system ATP-binding protein [EC:3.6.3.29] | ec:3.6.3.29 |
| bac:BamMC406\_3756 | molybdate ABC transporter inner membrane subunit; K02018 molybdate transport system permease protein |  |
| bac:BamMC406\_3757 | molybdenum ABC transporter periplasmic molybdate-binding protein; K02020 molybdate transport system substrate-binding protein |  |
| bac:BamMC406\_3758 | PA-phosphatase-like phosphoesterase |  |
| bac:BamMC406\_3759 | PHB depolymerase family esterase |  |
| bac:BamMC406\_3760 | hypothetical protein |  |
| bac:BamMC406\_3761 | OsmC family protein |  |
| bac:BamMC406\_3762 | MarR family transcriptional regulator |  |
| bac:BamMC406\_3763 | hypothetical protein |  |
| bac:BamMC406\_3764 | homoserine kinase (EC:2.7.1.39); K02204 homoserine kinase type II [EC:2.7.1.39] | ec:2.7.1.39 |

  
**Neighborhood Representations for "bam:Bamb\_3225"**  

| ID | Annotation | EC number |
| --- | --- | --- |
| bam:Bamb\_3215 | inner membrane protein translocase component YidC; K03217 YidC/Oxa1 family membrane protein insertase |  |
| bam:Bamb\_3216 | hypothetical protein; K08998 hypothetical protein |  |
| bam:Bamb\_3217 | ribonuclease P protein component; K03536 ribonuclease P protein component [EC:3.1.26.5] | ec:3.1.26.5 |
| bam:Bamb\_3218 | rpmH; 50S ribosomal protein L34; K02914 large subunit ribosomal protein L34 |  |
| bam:Bamb\_3219 | hypothetical protein |  |
| bam:Bamb\_3220 | histidine kinase |  |
| bam:Bamb\_3221 | hypothetical protein |  |
| bam:Bamb\_3222 | LysR family transcriptional regulator |  |
| bam:Bamb\_3223 | 4-oxalocrotonate tautomerase; K01821 4-oxalocrotonate tautomerase [EC:5.3.2.6] | ec:5.3.2.6 |
| bam:Bamb\_3224 | CHAD domain-containing protein |  |
| bam:Bamb\_3225 | ModE family transcriptional regulator; K02019 molybdate transport system regulatory protein |  |
| bam:Bamb\_3226 | ABC transporter; K02017 molybdate transport system ATP-binding protein [EC:3.6.3.29] | ec:3.6.3.29 |
| bam:Bamb\_3227 | molybdate ABC transporter inner membrane subunit; K02018 molybdate transport system permease protein |  |
| bam:Bamb\_3228 | molybdenum ABC transporter, periplasmic molybdate-binding protein; K02020 molybdate transport system substrate-binding protein |  |
| bam:Bamb\_3229 | PA-phosphatase-like phosphoesterase |  |
| bam:Bamb\_3230 | PHB depolymerase family esterase |  |
| bam:Bamb\_3231 | hypothetical protein |  |
| bam:Bamb\_3232 | OsmC family protein |  |
| bam:Bamb\_3233 | MarR family transcriptional regulator |  |
| bam:Bamb\_3234 | hypothetical protein |  |
| bam:Bamb\_3235 | homoserine kinase (EC:2.7.1.39); K02204 homoserine kinase type II [EC:2.7.1.39] | ec:2.7.1.39 |

  
**Neighborhood Representations for "bvi:Bcep1808\_4233"**  

| ID | Annotation | EC number |
| --- | --- | --- |
| bvi:Bcep1808\_4223 | hypothetical protein |  |
| bvi:Bcep1808\_4224 | methyltransferase type 11; K15256 tRNA (cmo5U34)-methyltransferase [EC:2.1.1.-] |  |
| bvi:Bcep1808\_4225 | glycoside hydrolase family protein |  |
| bvi:Bcep1808\_4226 | thiamine monophosphate synthase; K00788 thiamine-phosphate pyrophosphorylase [EC:2.5.1.3] | ec:2.5.1.3 |
| bvi:Bcep1808\_4227 | hypothetical protein |  |
| bvi:Bcep1808\_4228 | histidine kinase (EC:2.7.3.-) |  |
| bvi:Bcep1808\_4229 | hypothetical protein |  |
| bvi:Bcep1808\_4230 | LysR family transcriptional regulator |  |
| bvi:Bcep1808\_4231 | 4-oxalocrotonate tautomerase |  |
| bvi:Bcep1808\_4232 | CHAD domain-containing protein |  |
| bvi:Bcep1808\_4233 | ModE family transcriptional regulator; K02019 molybdate transport system regulatory protein |  |
| bvi:Bcep1808\_4234 | ABC transporter; K02017 molybdate transport system ATP-binding protein [EC:3.6.3.29] | ec:3.6.3.29 |
| bvi:Bcep1808\_4235 | molybdate ABC transporter inner membrane subunit; K02018 molybdate transport system permease protein |  |
| bvi:Bcep1808\_4236 | molybdenum ABC transporter, periplasmic molybdate-binding protein; K02020 molybdate transport system substrate-binding protein |  |
| bvi:Bcep1808\_4237 | PA-phosphatase-like phosphoesterase |  |
| bvi:Bcep1808\_4238 | PHB depolymerase family esterase |  |
| bvi:Bcep1808\_4239 | hypothetical protein |  |
| bvi:Bcep1808\_4240 | OsmC family protein |  |
| bvi:Bcep1808\_4241 | MarR family transcriptional regulator |  |
| bvi:Bcep1808\_4242 | hypothetical protein |  |
| bvi:Bcep1808\_4243 | homoserine kinase (EC:2.7.1.39); K02204 homoserine kinase type II [EC:2.7.1.39] | ec:2.7.1.39 |

  
**Neighborhood Representations for "bmj:BMULJ\_03627"**  

| ID | Annotation | EC number |
| --- | --- | --- |
| bmj:BMULJ\_03617 | hypothetical protein |  |
| bmj:BMULJ\_03618 | SAM-dependent methyltransferase; K15256 tRNA (cmo5U34)-methyltransferase [EC:2.1.1.-] |  |
| bmj:BMULJ\_03619 | treZ; maltooligosyltrehalose trehalohydrolase (EC:3.2.1.141) |  |
| bmj:BMULJ\_03620 | thiE; thiamine-phosphate pyrophosphorylase (EC:2.5.1.3); K00788 thiamine-phosphate pyrophosphorylase [EC:2.5.1.3] | ec:2.5.1.3 |
| bmj:BMULJ\_03621 | hypothetical protein |  |
| bmj:BMULJ\_03622 | signal transduction histidine kinase |  |
| bmj:BMULJ\_03623 | drug/metabolite transporter (DMT) superfamily permease |  |
| bmj:BMULJ\_03624 | LysR family transcriptional regulator |  |
| bmj:BMULJ\_03625 | tautomerase enzyme family protein |  |
| bmj:BMULJ\_03626 | hypothetical protein |  |
| bmj:BMULJ\_03627 | modE; molybdate transporter regulatory protein; K02019 molybdate transport system regulatory protein |  |
| bmj:BMULJ\_03628 | modC; molybdate transporter ATP-binding protein; K02017 molybdate transport system ATP-binding protein [EC:3.6.3.29] | ec:3.6.3.29 |
| bmj:BMULJ\_03629 | modB; molybdate transporter permease; K02018 molybdate transport system permease protein |  |
| bmj:BMULJ\_03630 | modA; molybdate transporter substrate-binding protein; K02020 molybdate transport system substrate-binding protein |  |
| bmj:BMULJ\_03631 | PAP2 family PA-phosphatase-related phosphoesterase |  |
| bmj:BMULJ\_03632 | poly(3-hydroxybutyrate) depolymerase |  |
| bmj:BMULJ\_03633 | hypothetical protein |  |
| bmj:BMULJ\_03634 | osmC; osmotically inducible protein |  |
| bmj:BMULJ\_03635 | MarR family transcriptional regulator |  |
| bmj:BMULJ\_03636 | hypothetical protein |  |
| bmj:BMULJ\_03637 | thrB; homoserine kinase (EC:2.7.1.39); K02204 homoserine kinase type II [EC:2.7.1.39] | ec:2.7.1.39 |

  
**Neighborhood Representations for "bmu:Bmul\_4888"**  

| ID | Annotation | EC number |
| --- | --- | --- |
| bmu:Bmul\_4878 | homoserine kinase (EC:2.7.1.39); K02204 homoserine kinase type II [EC:2.7.1.39] | ec:2.7.1.39 |
| bmu:Bmul\_4879 | hypothetical protein |  |
| bmu:Bmul\_4880 | MarR family transcriptional regulator |  |
| bmu:Bmul\_4881 | OsmC family protein |  |
| bmu:Bmul\_4882 | hypothetical protein |  |
| bmu:Bmul\_4883 | PHB depolymerase family esterase |  |
| bmu:Bmul\_4884 | PA-phosphatase like phosphoesterase |  |
| bmu:Bmul\_4885 | molybdenum ABC transporter, periplasmic molybdate-binding protein; K02020 molybdate transport system substrate-binding protein |  |
| bmu:Bmul\_4886 | molybdate ABC transporter inner membrane subunit; K02018 molybdate transport system permease protein |  |
| bmu:Bmul\_4887 | ABC transporter; K02017 molybdate transport system ATP-binding protein [EC:3.6.3.29] | ec:3.6.3.29 |
| bmu:Bmul\_4888 | ModE family transcriptional regulator; K02019 molybdate transport system regulatory protein |  |
| bmu:Bmul\_4889 | CHAD domain-containing protein |  |
| bmu:Bmul\_4890 | 4-oxalocrotonate tautomerase |  |
| bmu:Bmul\_4891 | LysR family transcriptional regulator |  |
| bmu:Bmul\_4892 | hypothetical protein |  |
| bmu:Bmul\_4893 | histidine kinase |  |
| bmu:Bmul\_4894 | hypothetical protein |  |
| bmu:Bmul\_4895 | thiamine monophosphate synthase; K00788 thiamine-phosphate pyrophosphorylase [EC:2.5.1.3] | ec:2.5.1.3 |
| bmu:Bmul\_4896 | glycoside hydrolase family protein |  |
| bmu:Bmul\_4897 | methyltransferase type 11; K15256 tRNA (cmo5U34)-methyltransferase [EC:2.1.1.-] |  |
| bmu:Bmul\_4898 | hypothetical protein |  |

  
**Neighborhood Representations for "cti:RALTA\_A0628"**  

| ID | Annotation | EC number |
| --- | --- | --- |
| cti:RALTA\_A0617 | hypothetical protein |  |
| cti:RALTA\_A0618 | sciN; replication/virulence associated protein; exported protein; K11906 type VI secretion system protein VasD |  |
| cti:RALTA\_A0619 | sciO; replication/virulence associated protein; K11893 type VI secretion system protein ImpJ |  |
| cti:RALTA\_A0620 | sciP; replication/virulence associated protein; outer membrane protein, ompa family membrane protein; K11892 type VI secretion system protein ImpK |  |
| cti:RALTA\_A0621 | sciS; replication related protein; K11891 type VI secretion system protein ImpL |  |
| cti:RALTA\_A0622 | sciT; replication/virulence associated protein; K11890 type VI secretion system protein ImpM |  |
| cti:RALTA\_A0623 | transglycosylase (EC:2.4.2.-) |  |
| cti:RALTA\_A0624 | sodium:sulfate symporter |  |
| cti:RALTA\_A0626 | hypothetical protein |  |
| cti:RALTA\_A0627 | hypothetical protein; K01104 protein-tyrosine phosphatase [EC:3.1.3.48] | ec:3.1.3.48 |
| cti:RALTA\_A0628 | modE; transcriptional repressor for molybdate uptake; K02019 molybdate transport system regulatory protein |  |
| cti:RALTA\_A0629 | l-lysine exporter; K06895 L-lysine exporter family protein LysE/ArgO |  |
| cti:RALTA\_A0630 | adenylate cyclase; cyth family (EC:4.6.1.1) |  |
| cti:RALTA\_A0631 | modC; molybdate transporter; ABC transporter ATP-binding protein; K02017 molybdate transport system ATP-binding protein [EC:3.6.3.29] | ec:3.6.3.29 |
| cti:RALTA\_A0632 | modB; molybdate transporter; ABC transporter; K02018 molybdate transport system permease protein |  |
| cti:RALTA\_A0633 | modA; molybdate transporter; ABC transporter substrate-binding protein; K02020 molybdate transport system substrate-binding protein |  |
| cti:RALTA\_A0634 | hypothetical protein |  |
| cti:RALTA\_A0635 | glyoxylate reductase / 2-ketogluconate reductase (glycolate reductase) (EC:1.1.1.26 1.1.1.215) |  |
| cti:RALTA\_A0636 | nucleoside 2-deoxyribosyltransferase (EC:2.4.2.6) |  |
| cti:RALTA\_A0637 | lipoprotein; K09857 hypothetical protein |  |
| cti:RALTA\_A0638 | pqiB; paraquat-inducible protein b; K06192 paraquat-inducible protein B |  |

  
**Neighborhood Representations for "reh:H16\_A0672"**  

| ID | Annotation | EC number |
| --- | --- | --- |
| reh:H16\_A0662 | h16\_A0662; hypothetical protein; K11892 type VI secretion system protein ImpK |  |
| reh:H16\_A0663 | h16\_A0663; hypothetical protein; K11891 type VI secretion system protein ImpL |  |
| reh:H16\_A0664 | h16\_A0664; hypothetical protein; K11890 type VI secretion system protein ImpM |  |
| reh:H16\_A0665 | h16\_A0665; bifunctional transglycosylase and transpeptidase |  |
| reh:H16\_A0666 | ldh; L-lactate dehydrogenase (EC:1.1.1.27); K00016 L-lactate dehydrogenase [EC:1.1.1.27] | ec:1.1.1.27 |
| reh:H16\_A0667 | h16\_A0667; Sodium:sulfate symporter transmembrane region |  |
| reh:H16\_A0668 | h16\_A0668; hypothetical protein |  |
| reh:H16\_A0669 | h16\_A0669; protein tyrosine/serine phosphatase (EC:3.1.3.48); K01104 protein-tyrosine phosphatase [EC:3.1.3.48] | ec:3.1.3.48 |
| reh:H16\_A0670 | ackA2; acetate kinase (EC:2.7.2.1); K00925 acetate kinase [EC:2.7.2.1] | ec:2.7.2.1 |
| reh:H16\_A0671 | h16\_A0671; hypothetical protein |  |
| reh:H16\_A0672 | h16\_A0672; transcriptional repressor-molybdenum-binding protein; K02019 molybdate transport system regulatory protein |  |
| reh:H16\_A0673 | lysE; lysine efflux permease; K06895 L-lysine exporter family protein LysE/ArgO |  |
| reh:H16\_A0674 | h16\_A0674; adenylate cyclase (EC:4.6.1.1); K01768 adenylate cyclase [EC:4.6.1.1] | ec:4.6.1.1 |
| reh:H16\_A0675 | modC; ABC transporter ATPase (EC:3.6.3.-); K02017 molybdate transport system ATP-binding protein [EC:3.6.3.29] | ec:3.6.3.29 |
| reh:H16\_A0676 | modB; ABC transporter permease; K02018 molybdate transport system permease protein |  |
| reh:H16\_A0677 | modA; ABC transporter periplasmic protein; K02020 molybdate transport system substrate-binding protein |  |
| reh:H16\_A0678 | h16\_A0678; hypothetical protein |  |
| reh:H16\_A0679 | h16\_A0679; lactate dehydrogenase or related dehydrogenase (EC:1.1.1.-); K00100 [EC:1.1.1.-] |  |
| reh:H16\_A0680 | h16\_A0680; nucleoside 2-deoxyribosyltransferase (EC:2.4.2.6); K08728 nucleoside deoxyribosyltransferase [EC:2.4.2.6] | ec:2.4.2.6 |
| reh:H16\_A0681 | h16\_A0681; hypothetical protein; K09857 hypothetical protein |  |
| reh:H16\_A0682 | pqiB; paraquat-inducible protein B; K06192 paraquat-inducible protein B |  |

  
**Neighborhood Representations for "reu:Reut\_A0630"**  

| ID | Annotation | EC number |
| --- | --- | --- |
| reu:Reut\_A0620 | formate dehydrogenase accessory protein; K02379 FdhD protein |  |
| reu:Reut\_A0621 | formate dehydrogenase delta subunit (EC:1.2.1.2); K00126 formate dehydrogenase subunit delta [EC:1.2.1.2] | ec:1.2.1.2 |
| reu:Reut\_A0622 | glycosyl transferase family protein |  |
| reu:Reut\_A0623 | hypothetical protein |  |
| reu:Reut\_A0624 | protein tyrosine/serine phosphatase; K01104 protein-tyrosine phosphatase [EC:3.1.3.48] | ec:3.1.3.48 |
| reu:Reut\_A0625 | acetate kinase (EC:2.7.2.1); K00925 acetate kinase [EC:2.7.2.1] | ec:2.7.2.1 |
| reu:Reut\_A0626 | phosphate acetyltransferase (EC:2.3.1.8); K00625 phosphate acetyltransferase [EC:2.3.1.8] | ec:2.3.1.8 |
| reu:Reut\_A0627 | hypothetical protein |  |
| reu:Reut\_A0628 | enoyl-(acyl carrier protein) reductase (EC:1.3.1.9); K00208 enoyl-[acyl-carrier protein] reductase I [EC:1.3.1.9 1.3.1.10] | ec:1.3.1.10 ec:1.3.1.9 |
| reu:Reut\_A0629 | hypothetical protein |  |
| reu:Reut\_A0630 | ModE family transcriptional regulator; K02019 molybdate transport system regulatory protein |  |
| reu:Reut\_A0631 | adenylate cyclase |  |
| reu:Reut\_A0632 | ABC transporter; K02017 molybdate transport system ATP-binding protein [EC:3.6.3.29] | ec:3.6.3.29 |
| reu:Reut\_A0633 | molybdate ABC transporter permease; K02018 molybdate transport system permease protein |  |
| reu:Reut\_A0634 | molybdenum ABC transporter periplasmic-binding protein; K02020 molybdate transport system substrate-binding protein |  |
| reu:Reut\_A0635 | hypothetical protein |  |
| reu:Reut\_A0636 | D-isomer specific 2-hydroxyacid dehydrogenase catalytic subunit |  |
| reu:Reut\_A0637 | nucleoside 2-deoxyribosyltransferase |  |
| reu:Reut\_A0638 | transposase |  |
| reu:Reut\_A0639 | dihydroxy-acid dehydratase (EC:4.2.1.9); K01687 dihydroxy-acid dehydratase [EC:4.2.1.9] | ec:4.2.1.9 |
| reu:Reut\_A0640 | LysR family transcriptional regulator |  |

  
**Neighborhood Representations for "rme:Rmet\_0567"**  

| ID | Annotation | EC number |
| --- | --- | --- |
| rme:Rmet\_0557 | fdsD; NAD-dependent formate dehydrogenase delta subunit (EC:1.2.1.2); K00126 formate dehydrogenase subunit delta [EC:1.2.1.2] | ec:1.2.1.2 |
| rme:Rmet\_0558 | putative bifunctional transglycosylase and transpeptidase |  |
| rme:Rmet\_0559 | ldh; L-Lactate dehydrogenase (EC:1.1.1.27); K00016 L-lactate dehydrogenase [EC:1.1.1.27] | ec:1.1.1.27 |
| rme:Rmet\_0560 | Sodium:sulfate symporter transmembrane region |  |
| rme:Rmet\_0561 | putative tyrosine/serine phosphatase (EC:3.1.3.48); K01104 protein-tyrosine phosphatase [EC:3.1.3.48] | ec:3.1.3.48 |
| rme:Rmet\_0562 | ackA2; acetate kinase (EC:2.7.2.1); K00925 acetate kinase [EC:2.7.2.1] | ec:2.7.2.1 |
| rme:Rmet\_0563 | ptb; phosphate acetyltransferase (EC:2.3.1.19); K00625 phosphate acetyltransferase [EC:2.3.1.8] | ec:2.3.1.8 |
| rme:Rmet\_0564 | hypothetical protein |  |
| rme:Rmet\_0565 | fabI; enoyl-(acyl carrier protein) reductase (EC:1.3.1.9); K00208 enoyl-[acyl-carrier protein] reductase I [EC:1.3.1.9 1.3.1.10] | ec:1.3.1.10 ec:1.3.1.9 |
| rme:Rmet\_0566 | moaA1; Molybdenum cofactor biosynthesis protein A 1 |  |
| rme:Rmet\_0567 | modE; DNA-binding transcriptional dual regulator; K02019 molybdate transport system regulatory protein |  |
| rme:Rmet\_0568 | adenylate cyclase, class 2 (EC:4.6.1.1) |  |
| rme:Rmet\_0569 | modC; molybdate transporter subunit, ABC transporter ATPase: MolT family (EC:3.6.3.29); K02017 molybdate transport system ATP-binding protein [EC:3.6.3.29] | ec:3.6.3.29 |
| rme:Rmet\_0570 | modB; molybdate ABC transporter membrane protein; K02018 molybdate transport system permease protein |  |
| rme:Rmet\_0571 | modA; molybdate ABC transporter periplasmic-binding protein; K02020 molybdate transport system substrate-binding protein |  |
| rme:Rmet\_0572 | cpdB; 2':3'-cyclic-nucleotide 2'-phosphodiesterase (EC:3.1.4.16); K01119 2',3'-cyclic-nucleotide 2'-phosphodiesterase [EC:3.1.4.16] | ec:3.1.4.16 |
| rme:Rmet\_0573 | hypothetical protein |  |
| rme:Rmet\_0574 | htrE; Fimbrial biogenesis outer membrane usher; K07347 outer membrane usher protein |  |
| rme:Rmet\_0575 | fimC; periplasmic chaperone |  |
| rme:Rmet\_0576 | fimA; Type-1 fimbrial protein subunit precursor; K07345 major type 1 subunit fimbrin (pilin) |  |
| rme:Rmet\_0577 | putative nucleoside 2-deoxyribosyltransferase |  |

  
**Neighborhood Representations for "axy:AXYL\_04387"**  

| ID | Annotation | EC number |
| --- | --- | --- |
| axy:AXYL\_04377 | uvrC; excinuclease ABC subunit C; K03703 excinuclease ABC subunit C |  |
| axy:AXYL\_04378 | nagZ; beta-hexosaminidase (EC:3.2.1.52); K01207 beta-N-acetylhexosaminidase [EC:3.2.1.52] | ec:3.2.1.52 |
| axy:AXYL\_04379 | acpS; holo-[acyl-carrier-protein] synthase (EC:2.7.8.7); K00997 holo-[acyl-carrier protein] synthase [EC:2.7.8.7] | ec:2.7.8.7 |
| axy:AXYL\_04380 | pdxJ; pyridoxal phosphate biosynthetic protein PdxJ (EC:2.6.99.2); K03474 pyridoxine 5-phosphate synthase [EC:2.6.99.2] | ec:2.6.99.2 |
| axy:AXYL\_04381 | outer membrane protein OprM 4 |  |
| axy:AXYL\_04382 | acrB; acriflavine resistance protein B; K03296 hydrophobic/amphiphilic exporter-1 (mainly G- bacteria), HAE1 family |  |
| axy:AXYL\_04383 | bpeA3; periplasmic linker protein 3; K03585 membrane fusion protein |  |
| axy:AXYL\_04384 | transcriptional regulator family 2 |  |
| axy:AXYL\_04385 | patatin-like phospholipase family protein 1; K07001 NTE family protein |  |
| axy:AXYL\_04386 | hypothetical protein |  |
| axy:AXYL\_04387 | modE; molybdenum transporter ModE; K02019 molybdate transport system regulatory protein |  |
| axy:AXYL\_04388 | ABC transporter (EC:3.6.3.-); K02017 molybdate transport system ATP-binding protein [EC:3.6.3.29] | ec:3.6.3.29 |
| axy:AXYL\_04389 | modB; molybdate ABC transporter permease; K02018 molybdate transport system permease protein |  |
| axy:AXYL\_04390 | modA; molybdate ABC transporter periplasmic molybdate-binding protein; K02020 molybdate transport system substrate-binding protein |  |
| axy:AXYL\_04391 | speD; S-adenosylmethionine decarboxylase proenzyme (EC:4.1.1.50); K01611 S-adenosylmethionine decarboxylase [EC:4.1.1.50] | ec:4.1.1.50 |
| axy:AXYL\_04392 | hypothetical protein |  |
| axy:AXYL\_04393 | hypothetical protein |  |
| axy:AXYL\_04394 | SPFH domain/Band 7 family protein 2 |  |
| axy:AXYL\_04395 | hypothetical protein; K08989 putative membrane protein |  |
| axy:AXYL\_04396 | spermine/spermidine synthase family protein 3; K00797 spermidine synthase [EC:2.5.1.16] | ec:2.5.1.16 |
| axy:AXYL\_04397 | twin-arginine translocation pathway signal 3 |  |

  
**Neighborhood Representations for "gpb:HDN1F\_09840"**  

| ID | Annotation | EC number |
| --- | --- | --- |
| gpb:HDN1F\_09740 | mopI; molybdopterin-binding protein; K02019 molybdate transport system regulatory protein |  |
| gpb:HDN1F\_09750 | modA; molybdenum ABC transporter periplasmic molybdenum-binding protein; K02020 molybdate transport system substrate-binding protein |  |
| gpb:HDN1F\_09760 | modB; molybdenum ABC transporter permease; K02018 molybdate transport system permease protein |  |
| gpb:HDN1F\_09770 | modC; molybdenum import ABC transporter ATP-binding protein; K02017 molybdate transport system ATP-binding protein [EC:3.6.3.29] | ec:3.6.3.29 |
| gpb:HDN1F\_09780 | TonB-dependent outer membrane receptor; K02014 iron complex outermembrane recepter protein |  |
| gpb:HDN1F\_09790 | oxidoreductase, molybdopterin binding |  |
| gpb:HDN1F\_09800 | transcriptional regulator |  |
| gpb:HDN1F\_09810 | short-chain dehydrogenase |  |
| gpb:HDN1F\_09820 | NADH:flavin oxidoreductase |  |
| gpb:HDN1F\_09830 | hypothetical protein |  |
| gpb:HDN1F\_09840 | molybdopterin-binding protein; K02019 molybdate transport system regulatory protein |  |
| gpb:HDN1F\_09850 | hemN2; Oxygen-independent coproporphyrinogen III oxidase |  |
| gpb:HDN1F\_09860 | fnr; transcriptional regulatory protein; K01420 CRP/FNR family transcriptional regulator, anaerobic regulatory protein |  |
| gpb:HDN1F\_09870 | hypothetical protein |  |
| gpb:HDN1F\_09880 | ddlA; D-alanine-D-alanine ligase (EC:6.3.2.4); K01921 D-alanine-D-alanine ligase [EC:6.3.2.4] | ec:6.3.2.4 |
| gpb:HDN1F\_09890 | hypothetical protein |  |
| gpb:HDN1F\_09900 | phospholipid/glycerol acyltransferase |  |
| gpb:HDN1F\_09910 | Integral membrane protein DUF6 |  |
| gpb:HDN1F\_09920 | hypothetical protein; K07003 |  |
| gpb:HDN1F\_09930 | thioesterase |  |
| gpb:HDN1F\_09940 | AMP-dependent synthetase/ligase |  |

  
**Neighborhood Representations for "bpt:Bpet3267"**  

| ID | Annotation | EC number |
| --- | --- | --- |
| bpt:Bpet3257 | nagZ; beta-hexosaminidase (EC:3.2.1.52); K01207 beta-N-acetylhexosaminidase [EC:3.2.1.52] | ec:3.2.1.52 |
| bpt:Bpet3258 | acpS; 4'-phosphopantetheinyl transferase (EC:2.7.8.7); K00997 holo-[acyl-carrier protein] synthase [EC:2.7.8.7] | ec:2.7.8.7 |
| bpt:Bpet3259 | pdxJ; pyridoxine 5'-phosphate synthase; K03474 pyridoxine 5-phosphate synthase [EC:2.6.99.2] | ec:2.6.99.2 |
| bpt:Bpet3260 | outer membrane efflux protein |  |
| bpt:Bpet3261 | AcrB/AcrD/AcrF family protein |  |
| bpt:Bpet3262 | AcrB/AcrD/AcrF family protein; K03296 hydrophobic/amphiphilic exporter-1 (mainly G- bacteria), HAE1 family |  |
| bpt:Bpet3263 | acridine efflux pump constituent; K03585 membrane fusion protein |  |
| bpt:Bpet3264 | transcriptional regulator |  |
| bpt:Bpet3265 | hypothetical protein; K07001 NTE family protein |  |
| bpt:Bpet3266 | lipoprotein |  |
| bpt:Bpet3267 | modE; molybdenum transporter; K02019 molybdate transport system regulatory protein |  |
| bpt:Bpet3268 | Sulfate/thiosulfate import ATP-binding protein cysA (EC:3.6.3.25); K05833 putative ABC transport system ATP-binding protein |  |
| bpt:Bpet3269 | transmembrane component of ABC transporter; K05832 putative ABC transport system permease protein |  |
| bpt:Bpet3270 | hypothetical protein; K01989 putative ABC transport system substrate-binding protein |  |
| bpt:Bpet3271 | TRAP-type C4-dicarboxylate transport system, large permease component |  |
| bpt:Bpet3272 | TRAP-type C4-dicarboxylate transport system, small permease component |  |
| bpt:Bpet3273 | TRAP-type C4-dicarboxylate transport system, periplasmic component |  |
| bpt:Bpet3274 | malonyl-CoA synthase |  |
| bpt:Bpet3275 | hypothetical protein |  |
| bpt:Bpet3276 | malonyl-CoA decarboxylase; K01578 malonyl-CoA decarboxylase [EC:4.1.1.9] | ec:4.1.1.9 |
| bpt:Bpet3277 | GntR family transcriptional regulator |  |

  
**Neighborhood Representations for "rpf:Rpic12D\_4329"**  

| ID | Annotation | EC number |
| --- | --- | --- |
| rpf:Rpic12D\_4319 | AraC family transcriptional regulator |  |
| rpf:Rpic12D\_4320 | histidine kinase (EC:2.7.13.3); K07642 two-component system, OmpR family, sensor histidine kinase BaeS [EC:2.7.13.3] | ec:2.7.13.3 |
| rpf:Rpic12D\_4321 | transcriptional regulator; K07664 two-component system, OmpR family, response regulator BaeR |  |
| rpf:Rpic12D\_4322 | lipoprotein signal peptide |  |
| rpf:Rpic12D\_4323 | UspA domain-containing protein |  |
| rpf:Rpic12D\_4324 | AraC family transcriptional regulator; K07506 AraC family transcriptional regulator |  |
| rpf:Rpic12D\_4325 | signal peptide protein |  |
| rpf:Rpic12D\_4326 | hypothetical protein |  |
| rpf:Rpic12D\_4327 | PAS/PAC and GAF sensor-containing diguanylate cyclase/phosphodiesterase |  |
| rpf:Rpic12D\_4328 | hypothetical protein |  |
| rpf:Rpic12D\_4329 | ModE family transcriptional regulator; K02019 molybdate transport system regulatory protein |  |
| rpf:Rpic12D\_4330 | flavodoxin/nitric oxide synthase |  |
| rpf:Rpic12D\_4331 | ABC transporter; K02017 molybdate transport system ATP-binding protein [EC:3.6.3.29] | ec:3.6.3.29 |
| rpf:Rpic12D\_4332 | molybdate ABC transporter inner membrane subunit; K02018 molybdate transport system permease protein |  |
| rpf:Rpic12D\_4333 | NodT family RND efflux system outer membrane lipoprotein |  |
| rpf:Rpic12D\_4334 | hydrophobe/amphiphile efflux-1 (HAE1) family transporter; K03296 hydrophobic/amphiphilic exporter-1 (mainly G- bacteria), HAE1 family |  |
| rpf:Rpic12D\_4335 | RND family efflux transporter MFP subunit |  |
| rpf:Rpic12D\_4336 | alpha/beta hydrolase |  |
| rpf:Rpic12D\_4337 | LysR family transcriptional regulator |  |
| rpf:Rpic12D\_4338 | hypothetical protein |  |
| rpf:Rpic12D\_4339 | hypothetical protein; K09122 hypothetical protein |  |

  
**Neighborhood Representations for "rpi:Rpic\_4219"**  

| ID | Annotation | EC number |
| --- | --- | --- |
| rpi:Rpic\_4209 | AraC family transcriptional regulator |  |
| rpi:Rpic\_4210 | integral membrane sensor signal transduction histidine kinase (EC:2.7.13.3); K07642 two-component system, OmpR family, sensor histidine kinase BaeS [EC:2.7.13.3] | ec:2.7.13.3 |
| rpi:Rpic\_4211 | transcriptional regulator; K07664 two-component system, OmpR family, response regulator BaeR |  |
| rpi:Rpic\_4212 | lipoprotein signal peptide |  |
| rpi:Rpic\_4213 | UspA domain-containing protein |  |
| rpi:Rpic\_4214 | AraC family transcriptional regulator; K07506 AraC family transcriptional regulator |  |
| rpi:Rpic\_4215 | signal peptide protein |  |
| rpi:Rpic\_4216 | glycoprotein |  |
| rpi:Rpic\_4217 | PAS/PAC and GAF sensor-containing diguanylate cyclase/phosphodiesterase |  |
| rpi:Rpic\_4218 | hypothetical protein |  |
| rpi:Rpic\_4219 | ModE family transcriptional regulator; K02019 molybdate transport system regulatory protein |  |
| rpi:Rpic\_4220 | flavodoxin/nitric oxide synthase |  |
| rpi:Rpic\_4221 | ABC transporter; K02017 molybdate transport system ATP-binding protein [EC:3.6.3.29] | ec:3.6.3.29 |
| rpi:Rpic\_4222 | molybdate ABC transporter inner membrane subunit; K02018 molybdate transport system permease protein |  |
| rpi:Rpic\_4223 | NodT family RND efflux system outer membrane lipoprotein |  |
| rpi:Rpic\_4224 | hydrophobe/amphiphile efflux-1 (HAE1) family transporter; K03296 hydrophobic/amphiphilic exporter-1 (mainly G- bacteria), HAE1 family |  |
| rpi:Rpic\_4225 | RND family efflux transporter MFP subunit |  |
| rpi:Rpic\_4226 | Alpha/beta hydrolase fold-3 domain-containing protein |  |
| rpi:Rpic\_4227 | LysR family transcriptional regulator |  |
| rpi:Rpic\_4228 | hypothetical protein |  |
| rpi:Rpic\_4229 | hypothetical protein; K09122 hypothetical protein |  |

  
**Neighborhood Representations for "rso:RS05434"**  

| ID | Annotation | EC number |
| --- | --- | --- |
| rso:RSp1143 | glsA; glutaminase (EC:3.5.1.2); K01425 glutaminase [EC:3.5.1.2] | ec:3.5.1.2 |
| rso:RS05467 | RSp1144; hypothetical protein; K02018 molybdate transport system permease protein |  |
| rso:RS05462 | RSp1145; ABC transporter ATP-binding protein; K02017 molybdate transport system ATP-binding protein [EC:3.6.3.29] | ec:3.6.3.29 |
| rso:RS05458 | RSp1146; transcription regulator protein |  |
| rso:RS05455 | RSp1147; transmemembrane reductasease oxidoreductase; K06988 |  |
| rso:RS05450 | RSp1148; hydrolase signal peptide protein |  |
| rso:RSp1149 | tISRso13; transposase IS13; K07495 putative transposase |  |
| rso:RS05437 | RSp1150; hypothetical protein |  |
| rso:RS05436 | RSp1151; hypothetical protein; K01821 4-oxalocrotonate tautomerase [EC:5.3.2.6] | ec:5.3.2.6 |
| rso:RSp1152 | tISRso5; transposase ISRSO5; K07494 putative transposase |  |
| rso:RS05434 | RSp1153; molybdenum-pterin-binding protein; K02019 molybdate transport system regulatory protein |  |
| rso:RS05433 | RSp1154; hypothetical protein |  |
| rso:RS05432 | RSp1155; hypothetical protein |  |
| rso:RS05430 | RSp1156; signal peptide protein |  |
| rso:RS05428 | RSp1157; transcription regulator protein; K07506 AraC family transcriptional regulator |  |
| rso:RS05427 | RSp1158; hypothetical protein; K09984 hypothetical protein |  |
| rso:RS05426 | RSp1159; hypothetical protein |  |
| rso:RS05425 | RSp1160; lipoprotein signal peptidase |  |
| rso:RS05646 | RSp1161; two-component response regulator transcription regulator protein; K07664 two-component system, OmpR family, response regulator BaeR |  |
| rso:RS05645 | RSp1162; two-component sensor histidine kinase transcription regulator protein (EC:2.7.3.-); K07642 two-component system, OmpR family, sensor histidine kinase BaeS [EC:2.7.13.3] | ec:2.7.13.3 |
| rso:RS05644 | RSp1163; hypothetical protein |  |

  
**Neighborhood Representations for "rsl:RPSI07\_mp1183"**  

| ID | Annotation | EC number |
| --- | --- | --- |
| rsl:RPSI07\_mp1173 | substrate-binding periplasmic (pbp) ABC transporter protein; K10001 glutamate/aspartate transport system substrate-binding protein |  |
| rsl:RPSI07\_mp1174 | LuxR family transcriptional regulator |  |
| rsl:RPSI07\_mp1175 | LuxR family transcriptional regulator |  |
| rsl:RPSI07\_mp1176 | muconolactone transporter |  |
| rsl:RPSI07\_mp1177 | hypothetical protein |  |
| rsl:RPSI07\_mp1178 | glsA; glutaminase (EC:3.5.1.2); K01425 glutaminase [EC:3.5.1.2] | ec:3.5.1.2 |
| rsl:RPSI07\_mp1179 | LysR family transcriptional regulator |  |
| rsl:RPSI07\_mp1180 | modB; molybdate ABC transporter permease; K02018 molybdate transport system permease protein |  |
| rsl:RPSI07\_mp1181 | modC; molybdate ABC transporter ATP-binding protein (EC:3.6.3.29); K02017 molybdate transport system ATP-binding protein [EC:3.6.3.29] | ec:3.6.3.29 |
| rsl:RPSI07\_mp1182 | pseudogene |  |
| rsl:RPSI07\_mp1183 | modE; transcriptional repressor for molybdate uptake; K02019 molybdate transport system regulatory protein |  |
| rsl:RPSI07\_mp1184 | hypothetical protein |  |
| rsl:RPSI07\_mp1185 | signal transduction protein eal-ggdef domains |  |
| rsl:RPSI07\_mp1186 | hypothetical protein |  |
| rsl:RPSI07\_mp1187 | hypothetical protein |  |
| rsl:RPSI07\_mp1188 | AraC family transcriptional regulator; K07506 AraC family transcriptional regulator |  |
| rsl:RPSI07\_mp1189 | hypothetical protein; K09984 hypothetical protein |  |
| rsl:RPSI07\_mp1190 | hypothetical protein |  |
| rsl:RPSI07\_mp1191 | universal stress protein related nucleotide-binding protein; uspa family |  |
| rsl:RPSI07\_mp1192 | hypothetical protein |  |
| rsl:RPSI07\_mp1193 | baeR; transcriptional regulator; K07664 two-component system, OmpR family, response regulator BaeR |  |

  
**Neighborhood Representations for "bbr:BB3353"**  

| ID | Annotation | EC number |
| --- | --- | --- |
| bbr:BB3343 | uvrC; excinuclease ABC subunit C; K03703 excinuclease ABC subunit C |  |
| bbr:BB3344 | nagZ; beta-hexosaminidase (EC:3.2.1.52); K01207 beta-N-acetylhexosaminidase [EC:3.2.1.52] | ec:3.2.1.52 |
| bbr:BB3345 | acpS; 4'-phosphopantetheinyl transferase (EC:2.7.8.7); K00997 holo-[acyl-carrier protein] synthase [EC:2.7.8.7] | ec:2.7.8.7 |
| bbr:BB3346 | pdxJ; pyridoxine 5'-phosphate synthase; K03474 pyridoxine 5-phosphate synthase [EC:2.6.99.2] | ec:2.6.99.2 |
| bbr:BB3347 | efflux system outer membrane component |  |
| bbr:BB3348 | efflux system transmembrane protein; K03296 hydrophobic/amphiphilic exporter-1 (mainly G- bacteria), HAE1 family |  |
| bbr:BB3349 | efflux system inner membrane protein; K03585 membrane fusion protein |  |
| bbr:BB3350 | transcriptional regulator |  |
| bbr:BB3351 | hypothetical protein; K07001 NTE family protein |  |
| bbr:BB3352 | pseudogene |  |
| bbr:BB3353 | molybdenum-binding protein; K02019 molybdate transport system regulatory protein |  |
| bbr:BB3354 | ABC transporter ATP-binding protein; K05833 putative ABC transport system ATP-binding protein |  |
| bbr:BB3355 | transmembrane component of ABC transporter; K05832 putative ABC transport system permease protein |  |
| bbr:BB3356 | hypothetical protein; K01989 putative ABC transport system substrate-binding protein |  |
| bbr:BB3357 | superoxide dismutase (EC:1.15.1.1); K04565 superoxide dismutase, Cu-Zn family [EC:1.15.1.1] | ec:1.15.1.1 |
| bbr:BB3358 | hypothetical protein |  |
| bbr:BB3359 | malonyl-CoA synthase |  |
| bbr:BB3360 | hypothetical protein |  |
| bbr:BB3361 | malonyl-CoA decarboxylase; K01578 malonyl-CoA decarboxylase [EC:4.1.1.9] | ec:4.1.1.9 |
| bbr:BB3362 | GntR family transcriptional regulator |  |
| bbr:BB3363 | hypothetical protein |  |

  
**Neighborhood Representations for "bpe:BP2071"**  

| ID | Annotation | EC number |
| --- | --- | --- |
| bpe:BP2060 | hypothetical protein |  |
| bpe:BP2061 | GntR family transcriptional regulator |  |
| bpe:BP2062 | malonyl-CoA decarboxylase; K01578 malonyl-CoA decarboxylase [EC:4.1.1.9] | ec:4.1.1.9 |
| bpe:BP2063 | hypothetical protein |  |
| bpe:BP2064 | pseudogene |  |
| bpe:BP2066 | hypothetical protein |  |
| bpe:BP2067 | superoxide dismutase (EC:1.15.1.1); K04565 superoxide dismutase, Cu-Zn family [EC:1.15.1.1] | ec:1.15.1.1 |
| bpe:BP2068 | hypothetical protein; K01989 putative ABC transport system substrate-binding protein |  |
| bpe:BP2069 | pseudogene |  |
| bpe:BP2070 | ABC transporter ATP-binding protein; K05833 putative ABC transport system ATP-binding protein |  |
| bpe:BP2071 | molybdenum-binding protein; K02019 molybdate transport system regulatory protein |  |
| bpe:BP2072 | lipoprotein |  |
| bpe:BP2073 | hypothetical protein; K07001 NTE family protein |  |
| bpe:BP2074 | DNA-binding protein |  |
| bpe:BP2075 | efflux system inner membrane protein; K03585 membrane fusion protein |  |
| bpe:BP2076 | efflux system transmembrane protein; K03296 hydrophobic/amphiphilic exporter-1 (mainly G- bacteria), HAE1 family |  |
| bpe:BP2077 | pseudogene |  |
| bpe:BP2078 | pdxJ; pyridoxine 5'-phosphate synthase; K03474 pyridoxine 5-phosphate synthase [EC:2.6.99.2] | ec:2.6.99.2 |
| bpe:BP2079 | acpS; 4'-phosphopantetheinyl transferase (EC:2.7.8.7); K00997 holo-[acyl-carrier protein] synthase [EC:2.7.8.7] | ec:2.7.8.7 |
| bpe:BP2080 | nagZ; beta-hexosaminidase (EC:3.2.1.52); K01207 beta-N-acetylhexosaminidase [EC:3.2.1.52] | ec:3.2.1.52 |
| bpe:BP2081 | uvrC; excinuclease ABC subunit C; K03703 excinuclease ABC subunit C |  |

  
**Neighborhood Representations for "bav:BAV2465"**  

| ID | Annotation | EC number |
| --- | --- | --- |
| bav:BAV2455 | pgsA; CDP-diacylglycerol--glycerol-3-phosphate 3-phosphatidyltransferase (EC:2.7.8.5); K00995 CDP-diacylglycerol--glycerol-3-phosphate 3-phosphatidyltransferase [EC:2.7.8.5] | ec:2.7.8.5 |
| bav:BAV2456 | uvrC; excinuclease ABC subunit C; K03703 excinuclease ABC subunit C |  |
| bav:BAV2457 | nagZ; beta-hexosaminidase (EC:3.2.1.52); K01207 beta-N-acetylhexosaminidase [EC:3.2.1.52] | ec:3.2.1.52 |
| bav:BAV2458 | acpS; 4'-phosphopantetheinyl transferase (EC:2.7.8.7); K00997 holo-[acyl-carrier protein] synthase [EC:2.7.8.7] | ec:2.7.8.7 |
| bav:BAV2459 | pdxJ; pyridoxine 5'-phosphate synthase; K03474 pyridoxine 5-phosphate synthase [EC:2.6.99.2] | ec:2.6.99.2 |
| bav:BAV2460 | multidrug efflux system outer membrane protein |  |
| bav:BAV2461 | multidrug efflux system transmembrane protein; K03296 hydrophobic/amphiphilic exporter-1 (mainly G- bacteria), HAE1 family |  |
| bav:BAV2462 | multidrug efflux system inner membrane protein; K03585 membrane fusion protein |  |
| bav:BAV2463 | transcriptional regulator |  |
| bav:BAV2464 | lipoprotein |  |
| bav:BAV2465 | modE; regulator of molybdenum transport genes; K02019 molybdate transport system regulatory protein |  |
| bav:BAV2466 | modC; molybdenum ABC transporter ATP-binding protein; K02017 molybdate transport system ATP-binding protein [EC:3.6.3.29] | ec:3.6.3.29 |
| bav:BAV2467 | modB; molybdenum ABC transporter permease; K02018 molybdate transport system permease protein |  |
| bav:BAV2468 | modA; molybdenum ABC transporter molybdate-binding periplasmic protein; K02020 molybdate transport system substrate-binding protein |  |
| bav:BAV2469 | ABC transporter ATP-binding protein; K05833 putative ABC transport system ATP-binding protein |  |
| bav:BAV2470 | ABC transporter permease; K05832 putative ABC transport system permease protein |  |
| bav:BAV2471 | hypothetical protein; K01989 putative ABC transport system substrate-binding protein |  |
| bav:BAV2472 | hypothetical protein |  |
| bav:BAV2473 | sodC; superoxide dismutase (EC:1.15.1.1); K04565 superoxide dismutase, Cu-Zn family [EC:1.15.1.1] | ec:1.15.1.1 |
| bav:BAV2474 | hypothetical protein |  |
| bav:BAV2475 | acylase (EC:2.3.2.2 3.5.1.-); K00681 gamma-glutamyltranspeptidase [EC:2.3.2.2] | ec:2.3.2.2 |

  
**Neighborhood Representations for "bpa:BPP1755"**  

| ID | Annotation | EC number |
| --- | --- | --- |
| bpa:BPP1745 | pseudogene |  |
| bpa:BPP1746 | GntR family transcriptional regulator |  |
| bpa:BPP1747 | malonyl-CoA decarboxylase; K01578 malonyl-CoA decarboxylase [EC:4.1.1.9] | ec:4.1.1.9 |
| bpa:BPP1748 | hypothetical protein |  |
| bpa:BPP1749 | malonyl-CoA synthase |  |
| bpa:BPP1750 | hypothetical protein |  |
| bpa:BPP1751 | superoxide dismutase (EC:1.15.1.1); K04565 superoxide dismutase, Cu-Zn family [EC:1.15.1.1] | ec:1.15.1.1 |
| bpa:BPP1752 | hypothetical protein; K01989 putative ABC transport system substrate-binding protein |  |
| bpa:BPP1753 | transmembrane component of ABC transporter; K05832 putative ABC transport system permease protein |  |
| bpa:BPP1754 | ABC-transporter ATP-binding component; K05833 putative ABC transport system ATP-binding protein |  |
| bpa:BPP1755 | molybdenum-binding protein; K02019 molybdate transport system regulatory protein |  |
| bpa:BPP1756 | lipoprotein |  |
| bpa:BPP1757 | hypothetical protein; K07001 NTE family protein |  |
| bpa:BPP1758 | pseudogene |  |
| bpa:BPP1759 | efflux system inner membrane protein; K03585 membrane fusion protein |  |
| bpa:BPP1760 | pseudogene |  |
| bpa:BPP1761 | efflux system outer membrane component |  |
| bpa:BPP1762 | pdxJ; pyridoxine 5'-phosphate synthase; K03474 pyridoxine 5-phosphate synthase [EC:2.6.99.2] | ec:2.6.99.2 |
| bpa:BPP1763 | acpS; 4'-phosphopantetheinyl transferase (EC:2.7.8.7); K00997 holo-[acyl-carrier protein] synthase [EC:2.7.8.7] | ec:2.7.8.7 |
| bpa:BPP1764 | nagZ; beta-hexosaminidase (EC:3.2.1.52); K01207 beta-N-acetylhexosaminidase [EC:3.2.1.52] | ec:3.2.1.52 |
| bpa:BPP1765 | uvrC; excinuclease ABC subunit C; K03703 excinuclease ABC subunit C |  |

  
**Neighborhood Representations for "gem:GM21\_2529"**  

| ID | Annotation | EC number |
| --- | --- | --- |
| gem:GM21\_2519 | hypothetical protein |  |
| gem:GM21\_2520 | hypothetical protein |  |
| gem:GM21\_2521 | PAS/PAC sensor hybrid histidine kinase |  |
| gem:GM21\_2522 | hypothetical protein |  |
| gem:GM21\_2523 | radical SAM protein |  |
| gem:GM21\_2524 | hypothetical protein |  |
| gem:GM21\_2525 | alkylphosphonate utilization operon protein PhnA; K06193 phosphonoacetate hydrolase [EC:3.11.1.2] | ec:3.11.1.2 |
| gem:GM21\_2526 | ABC transporter; K06857 tungstate transport system ATP-binding protein [EC:3.6.3.55] | ec:3.6.3.55 |
| gem:GM21\_2527 | binding-protein-dependent transporters inner membrane component; K05773 tungstate transport system permease protein |  |
| gem:GM21\_2528 | tungstate ABC transporter permease; K05772 tungstate transport system substrate-binding protein |  |
| gem:GM21\_2529 | ModE family transcriptional regulator; K02019 molybdate transport system regulatory protein |  |
| gem:GM21\_2530 | methyltransferase type 11; K15257 tRNA (mo5U34)-methyltransferase [EC:2.1.1.-] |  |
| gem:GM21\_2531 | hypothetical protein |  |
| gem:GM21\_2532 | hypothetical protein |  |
| gem:GM21\_2533 | hypothetical protein |  |
| gem:GM21\_2534 | group 1 glycosyl transferase |  |
| gem:GM21\_2535 | oxidoreductase domain-containing protein |  |
| gem:GM21\_2536 | alcohol dehydrogenase GroES domain protein |  |
| gem:GM21\_2537 | NAD-dependent epimerase/dehydratase; K12454 CDP-paratose 2-epimerase [EC:5.1.3.10] | ec:5.1.3.10 |
| gem:GM21\_2538 | NAD-dependent epimerase/dehydratase; K17947 dTDP-L-rhamnose 4-epimerase [EC:5.1.3.25] | ec:5.1.3.25 |
| gem:GM21\_2539 | malto-oligosyltrehalose trehalohydrolase; K01236 maltooligosyltrehalose trehalohydrolase [EC:3.2.1.141] | ec:3.2.1.141 |

  
**Neighborhood Representations for "alv:Alvin\_0888"**  

| ID | Annotation | EC number |
| --- | --- | --- |
| alv:Alvin\_0878 | MotA/TolQ/ExbB proton channel; K03561 biopolymer transport protein ExbB |  |
| alv:Alvin\_0879 | hypothetical protein |  |
| alv:Alvin\_0880 | TonB-dependent receptor; K02014 iron complex outermembrane recepter protein |  |
| alv:Alvin\_0881 | dual specificity protein phosphatase |  |
| alv:Alvin\_0882 | hypothetical protein |  |
| alv:Alvin\_0883 | diguanylate phosphodiesterase |  |
| alv:Alvin\_0884 | methyl-accepting chemotaxis sensory transducer |  |
| alv:Alvin\_0885 | molybdate ABC transporter ATPase subunit; K02017 molybdate transport system ATP-binding protein [EC:3.6.3.29] | ec:3.6.3.29 |
| alv:Alvin\_0886 | molybdate ABC transporter inner membrane subunit; K02018 molybdate transport system permease protein |  |
| alv:Alvin\_0887 | molybdenum ABC transporter substrate-binding protein; K02020 molybdate transport system substrate-binding protein |  |
| alv:Alvin\_0888 | ModE family transcriptional regulator; K02019 molybdate transport system regulatory protein |  |
| alv:Alvin\_0889 | Exonuclease RNase T and DNA polymerase III; K13288 oligoribonuclease [EC:3.1.-.-] |  |
| alv:Alvin\_0890 | apolipoprotein N-acyltransferase; K03820 apolipoprotein N-acyltransferase [EC:2.3.1.-] |  |
| alv:Alvin\_0891 | integrase family protein |  |
| alv:Alvin\_0892 | hypothetical protein |  |
| alv:Alvin\_0893 | hypothetical protein |  |
| alv:Alvin\_0894 | hypothetical protein |  |
| alv:Alvin\_0895 | hypothetical protein |  |
| alv:Alvin\_0896 | hypothetical protein |  |
| alv:Alvin\_0897 | single-strand binding protein; K03111 single-strand DNA-binding protein |  |
| alv:Alvin\_0898 | hypothetical protein |  |

  
**Neighborhood Representations for "gbm:Gbem\_1692"**  

| ID | Annotation | EC number |
| --- | --- | --- |
| gbm:Gbem\_1682 | alpha-glucan branching enzyme; K01236 maltooligosyltrehalose trehalohydrolase [EC:3.2.1.141] | ec:3.2.1.141 |
| gbm:Gbem\_1683 | NAD-dependent nucleoside diphosphate-sugar epimerase/dehydratase; K17947 dTDP-L-rhamnose 4-epimerase [EC:5.1.3.25] | ec:5.1.3.25 |
| gbm:Gbem\_1684 | NAD-dependent nucleoside diphosphate-sugar epimerase/dehydratase; K12454 CDP-paratose 2-epimerase [EC:5.1.3.10] | ec:5.1.3.10 |
| gbm:Gbem\_1685 | zinc-dependent alcohol dehydrogenase |  |
| gbm:Gbem\_1686 | NAD(P)-dependent oxidoreductase |  |
| gbm:Gbem\_1687 | glycosyltransferase |  |
| gbm:Gbem\_1688 | glycosyltransferase |  |
| gbm:Gbem\_1689 | glycosyltransferase |  |
| gbm:Gbem\_1690 | glycosyltransferase |  |
| gbm:Gbem\_1691 | SAM-dependent methyltransferase; K15257 tRNA (mo5U34)-methyltransferase [EC:2.1.1.-] |  |
| gbm:Gbem\_1692 | modE; molybdate transport regulatory protein ModE; K02019 molybdate transport system regulatory protein |  |
| gbm:Gbem\_1693 | tupA; tungstate ABC transporter periplasmic tungstate-binding protein; K05772 tungstate transport system substrate-binding protein |  |
| gbm:Gbem\_1694 | tupB; tungstate ABC transporter permease; K05773 tungstate transport system permease protein |  |
| gbm:Gbem\_1695 | tupC; tungstate ABC transporter ATP-binding protein; K06857 tungstate transport system ATP-binding protein [EC:3.6.3.55] | ec:3.6.3.55 |
| gbm:Gbem\_1696 | RNA polymerase sigma factor; K03088 RNA polymerase sigma-70 factor, ECF subfamily |  |
| gbm:Gbem\_1697 | hypothetical protein |  |
| gbm:Gbem\_1698 | phnA; phosphonoacetate hydrolase; K06193 phosphonoacetate hydrolase [EC:3.11.1.2] | ec:3.11.1.2 |
| gbm:Gbem\_1699 | cobalamin-binding radical SAM domain-containing iron-sulfur cluster-binding oxidoreductase |  |
| gbm:Gbem\_1700 | hypothetical protein |  |
| gbm:Gbem\_1701 | PAS/PAC domain-containing sensor histidine kinase response regulator |  |
| gbm:Gbem\_1702 | hypothetical protein |  |

  
**Neighborhood Representations for "cli:Clim\_0673"**  

| ID | Annotation | EC number |
| --- | --- | --- |
| cli:Clim\_0663 | UBA/THIF-type NAD/FAD binding protein |  |
| cli:Clim\_0664 | thiH; thiamine biosynthesis protein ThiH; K03150 thiamine biosynthesis ThiH |  |
| cli:Clim\_0665 | thiG; thiazole synthase; K03149 thiamine biosynthesis ThiG |  |
| cli:Clim\_0666 | thiamine biosynthesis protein ThiS; K03154 sulfur carrier protein |  |
| cli:Clim\_0667 | cystathionine gamma-synthase (EC:2.5.1.48); K01739 cystathionine gamma-synthase [EC:2.5.1.48] | ec:2.5.1.48 |
| cli:Clim\_0668 | O-acetylhomoserine/O-acetylserine sulfhydrylase (EC:2.5.1.49); K01740 O-acetylhomoserine (thiol)-lyase [EC:2.5.1.49] | ec:2.5.1.49 |
| cli:Clim\_0669 | hypothetical protein |  |
| cli:Clim\_0670 | hypothetical protein |  |
| cli:Clim\_0671 | MOSC domain-containing protein |  |
| cli:Clim\_0672 | molybdenum cofactor biosynthesis protein A; K03639 molybdenum cofactor biosynthesis protein |  |
| cli:Clim\_0673 | ModE family transcriptional regulator; K02019 molybdate transport system regulatory protein |  |
| cli:Clim\_0674 | binding-protein-dependent transport system inner membrane protein; K02018 molybdate transport system permease protein |  |
| cli:Clim\_0675 | molybdenum ABC transporter periplasmic molybdate-binding protein; K02020 molybdate transport system substrate-binding protein |  |
| cli:Clim\_0676 | TonB-dependent receptor; K02014 iron complex outermembrane recepter protein |  |
| cli:Clim\_0677 | ferredoxin, 2Fe-2S |  |
| cli:Clim\_0678 | nitrogenase cofactor biosynthesis protein NifB; K02585 nitrogen fixation protein NifB |  |
| cli:Clim\_0679 | nitrogenase (EC:1.18.6.1); K02592 nitrogenase molybdenum-iron protein NifN |  |
| cli:Clim\_0680 | nitrogenase MoFe cofactor biosynthesis protein NifE (EC:1.18.6.1); K02587 nitrogenase molybdenum-cofactor synthesis protein NifE |  |
| cli:Clim\_0681 | nitrogenase molybdenum-iron protein subunit beta (EC:1.18.6.1); K02591 nitrogenase molybdenum-iron protein beta chain [EC:1.18.6.1] | ec:1.18.6.1 |
| cli:Clim\_0682 | nitrogenase molybdenum-iron protein subunit alpha (EC:1.18.6.1); K02586 nitrogenase molybdenum-iron protein alpha chain [EC:1.18.6.1] | ec:1.18.6.1 |
| cli:Clim\_0683 | nitrogen regulatory protein P-II; K02590 nitrogen regulatory protein PII 2 |  |

  
**Neighborhood Representations for "cch:Cag\_1230"**  

| ID | Annotation | EC number |
| --- | --- | --- |
| cch:Cag\_1219 | aroB; 3-dehydroquinate synthase (EC:4.2.3.4); K01735 3-dehydroquinate synthase [EC:4.2.3.4] | ec:4.2.3.4 |
| cch:Cag\_1220 | aroK; shikimate kinase (EC:2.7.1.71); K00891 shikimate kinase [EC:2.7.1.71] | ec:2.7.1.71 |
| cch:Cag\_1221 | transcriptional regulator |  |
| cch:Cag\_1222 | methylmalonyl-CoA mutase N-terminal domain-containing protein (EC:5.4.99.2); K01847 methylmalonyl-CoA mutase [EC:5.4.99.2] | ec:5.4.99.2 |
| cch:Cag\_1223 | methylmalonyl-CoA mutase (EC:5.4.99.2); K01847 methylmalonyl-CoA mutase [EC:5.4.99.2] | ec:5.4.99.2 |
| cch:Cag\_1224 | hypothetical protein |  |
| cch:Cag\_1226 | hypothetical protein |  |
| cch:Cag\_1227 | hypothetical protein |  |
| cch:Cag\_1228 | Fis family transcriptional regulator; K02584 Nif-specific regulatory protein |  |
| cch:Cag\_1229 | homocitrate synthase (EC:2.3.3.13); K02594 homocitrate synthase NifV [EC:2.3.3.14] | ec:2.3.3.14 |
| cch:Cag\_1230 | molybdenum-pterin binding protein; K02019 molybdate transport system regulatory protein |  |
| cch:Cag\_1231 | hypothetical protein |  |
| cch:Cag\_1232 | aldo/keto reductase (EC:1.1.1.274) |  |
| cch:Cag\_1233 | hypothetical protein |  |
| cch:Cag\_1234 | universal stress protein |  |
| cch:Cag\_1235 | hypothetical protein |  |
| cch:Cag\_1236 | hypothetical protein |  |
| cch:Cag\_1237 | arginine/ornithine transport system ATPase; K07588 LAO/AO transport system kinase [EC:2.7.-.-] |  |
| cch:Cag\_1238 | hypothetical protein |  |
| cch:Cag\_1239 | hypothetical protein |  |
| cch:Cag\_1240 | hypothetical protein |  |

  
**Neighborhood Representations for "gsu:GSU2964"**  

| ID | Annotation | EC number |
| --- | --- | --- |
| gsu:GSU2954 | arsB; arsenite efflux pump protein; K03325 arsenite transporter, ACR3 family |  |
| gsu:GSU2955 | hypothetical protein; K07089 |  |
| gsu:GSU2956 | redox-active disulfide protein 2 |  |
| gsu:GSU2957 | trx-2; thioredoxin family protein; K03671 thioredoxin 1 |  |
| gsu:GSU2958 | dsbD; thiol:disulfide interchange protein |  |
| gsu:GSU2959 | hypothetical protein |  |
| gsu:GSU2960 | modC; molybdate ABC transporter ATP-binding protein; K02017 molybdate transport system ATP-binding protein [EC:3.6.3.29] | ec:3.6.3.29 |
| gsu:GSU2961 | modB; molybdate ABC transporter permease; K02018 molybdate transport system permease protein |  |
| gsu:GSU2962 | modA; molybdate ABC transporter substrate-binding protein; K02020 molybdate transport system substrate-binding protein |  |
| gsu:GSU2963 | modD; ModD protein; K03813 molybdenum transport protein [EC:2.4.2.-] |  |
| gsu:GSU2964 | modE; molybdate transport regulatory protein ModE; K02019 molybdate transport system regulatory protein |  |
| gsu:GSU2965 | redox-active protein, C\_GCAxxG\_C\_C family |  |
| gsu:GSU2967 | ferritin-like domain-containing protein |  |
| gsu:GSU2968 | hypothetical protein |  |
| gsu:GSU2969 | sensor diguanylate cyclase, PAS domain-containing |  |
| gsu:GSU2970 | stress-responsive alpha/beta-barrel domain-containing protein |  |
| gsu:GSU2971 | hypothetical protein |  |
| gsu:GSU2973 | lipoprotein |  |
| gsu:GSU2974 | metF-2; bifunctional homocysteine S-methyltransferase/5,10-methylenetetrahydrofolate reductase; K00547 homocysteine S-methyltransferase [EC:2.1.1.10] | ec:2.1.1.10 |
| gsu:GSU2975 | manganese-dependent inorganic pyrophosphatase; K15986 manganese-dependent inorganic pyrophosphatase [EC:3.6.1.1] | ec:3.6.1.1 |
| gsu:GSU2976 | membrane protein DedA |  |

  
**Neighborhood Representations for "glo:Glov\_0447"**  

| ID | Annotation | EC number |
| --- | --- | --- |
| glo:Glov\_0437 | hypothetical protein |  |
| glo:Glov\_0438 | hypothetical protein |  |
| glo:Glov\_0439 | transposase IS116/IS110/IS902 family protein |  |
| glo:Glov\_0440 | transposase IS4 family protein |  |
| glo:Glov\_0441 | hypothetical protein |  |
| glo:Glov\_0442 | type 11 methyltransferase |  |
| glo:Glov\_0443 | hypothetical protein |  |
| glo:Glov\_0444 | iron ABC transporter periplasmic protein |  |
| glo:Glov\_0445 | cobalamin synthesis protein P47K |  |
| glo:Glov\_0446 | ABC transporter |  |
| glo:Glov\_0447 | ModE family transcriptional regulator; K02019 molybdate transport system regulatory protein |  |
| glo:Glov\_0448 | modD protein; K03813 molybdenum transport protein [EC:2.4.2.-] |  |
| glo:Glov\_0449 | molybdenum ABC transporter periplasmic protein; K02020 molybdate transport system substrate-binding protein |  |
| glo:Glov\_0450 | molybdenum ABC transporter periplasmic protein; K02020 molybdate transport system substrate-binding protein |  |
| glo:Glov\_0451 | molybdate ABC transporter inner membrane subunit; K02018 molybdate transport system permease protein |  |
| glo:Glov\_0452 | molybdate ABC transporter ATPase; K02017 molybdate transport system ATP-binding protein [EC:3.6.3.29] | ec:3.6.3.29 |
| glo:Glov\_0453 | ion transport 2 domain-containing protein; K10716 voltage-gated potassium channel |  |
| glo:Glov\_0454 | nuclease |  |
| glo:Glov\_0455 | hypothetical protein |  |
| glo:Glov\_0456 | engB; ribosome biogenesis GTP-binding protein YsxC; K03978 GTP-binding protein |  |
| glo:Glov\_0457 | GTP cyclohydrolase; K09007 GTP cyclohydrolase I [EC:3.5.4.16] | ec:3.5.4.16 |

  
**Neighborhood Representations for "pph:Ppha\_1986"**  

| ID | Annotation | EC number |
| --- | --- | --- |
| pph:Ppha\_1976 | hypothetical protein |  |
| pph:Ppha\_1977 | hypothetical protein |  |
| pph:Ppha\_1978 | hypothetical protein |  |
| pph:Ppha\_1979 | type III restriction protein res subunit |  |
| pph:Ppha\_1980 | DNA methylase N-4/N-6 domain-containing protein |  |
| pph:Ppha\_1981 | hypothetical protein |  |
| pph:Ppha\_1982 | XRE family plasmid maintenance system antidote protein |  |
| pph:Ppha\_1983 | SNF2-like protein |  |
| pph:Ppha\_1984 | hypothetical protein |  |
| pph:Ppha\_1985 | PAS/PAC sensor hybrid histidine kinase |  |
| pph:Ppha\_1986 | ModE family transcriptional regulator; K02019 molybdate transport system regulatory protein |  |
| pph:Ppha\_1987 | TonB family protein; K03832 periplasmic protein TonB |  |
| pph:Ppha\_1988 | ABC transporter-like protein |  |
| pph:Ppha\_1989 | cobalamin synthesis protein P47K |  |
| pph:Ppha\_1990 | hypothetical protein |  |
| pph:Ppha\_1991 | periplasmic binding protein; K02016 iron complex transport system substrate-binding protein |  |
| pph:Ppha\_1992 | TonB-dependent receptor plug; K02014 iron complex outermembrane recepter protein |  |
| pph:Ppha\_1993 | ABC transporter-like protein; K02013 iron complex transport system ATP-binding protein [EC:3.6.3.34] | ec:3.6.3.34 |
| pph:Ppha\_1994 | transport system permease; K02015 iron complex transport system permease protein |  |
| pph:Ppha\_1995 | ABC transporter-like protein; K02017 molybdate transport system ATP-binding protein [EC:3.6.3.29] | ec:3.6.3.29 |
| pph:Ppha\_1996 | TOBE domain-containing protein |  |

  
**Neighborhood Representations for "wsu:WS1811"**  

| ID | Annotation | EC number |
| --- | --- | --- |
| wsu:WS1799 | hypothetical protein |  |
| wsu:WS1801 | flagellar basal-body rod protein FlgG; K02392 flagellar basal-body rod protein FlgG |  |
| wsu:WS1802 | flgG; flagellar basal body rod protein FlgG; K02392 flagellar basal-body rod protein FlgG |  |
| wsu:WS1803 | MODC; molybdenum ABC transporter ATP-binding protein; K02017 molybdate transport system ATP-binding protein [EC:3.6.3.29] | ec:3.6.3.29 |
| wsu:WS1804 | MODB; molybdenum ABC transporter permease; K02018 molybdate transport system permease protein |  |
| wsu:WS1805 | hypothetical protein |  |
| wsu:WS1807 | MODD; molybdenum transport system protein; K03813 molybdenum transport protein [EC:2.4.2.-] |  |
| wsu:WS1808 | modB; transmembrane protein; K02018 molybdate transport system permease protein |  |
| wsu:WS1809 | MODA; molybdate periplasmic binding protein; K02020 molybdate transport system substrate-binding protein |  |
| wsu:WS1810 | MODA; molybdate-binding lipoprotein; K02020 molybdate transport system substrate-binding protein |  |
| wsu:WS1811 | molybdenum-pterin-binding protein; K02019 molybdate transport system regulatory protein |  |
| wsu:WS1812 | leuD; 3-isopropylmalate dehydratase small subunit; K01704 3-isopropylmalate/(R)-2-methylmalate dehydratase small subunit [EC:4.2.1.33 4.2.1.35] | ec:4.2.1.33 ec:4.2.1.35 |
| wsu:WS1813 | 3-isopropylmalate dehydrogenase; K00052 3-isopropylmalate dehydrogenase [EC:1.1.1.85] | ec:1.1.1.85 |
| wsu:WS1814 | hypothetical protein |  |
| wsu:WS1815 | hypothetical protein |  |
| wsu:WS1817 | response regulator OMPR; K02483 two-component system, OmpR family, response regulator |  |
| wsu:WS1818 | histidine kinase sensor protein; K02484 two-component system, OmpR family, sensor kinase [EC:2.7.13.3] | ec:2.7.13.3 |
| wsu:WS1819 | RNA nucleotidyltransferase; K00974 tRNA nucleotidyltransferase (CCA-adding enzyme) [EC:2.7.7.72 3.1.3.- 3.1.4.-] | ec:2.7.7.72 |
| wsu:WS1820 | hypothetical protein |  |
| wsu:WS1821 | HEMB; delta-aminolevulinic acid dehydratase (EC:4.2.1.24); K01698 porphobilinogen synthase [EC:4.2.1.24] | ec:4.2.1.24 |
| wsu:WS1822 | hypothetical protein |  |

  
**Neighborhood Representations for "cpc:Cpar\_1630"**  

| ID | Annotation | EC number |
| --- | --- | --- |
| cpc:Cpar\_1620 | nitrogen regulatory protein P-II; K02589 nitrogen regulatory protein PII 1 |  |
| cpc:Cpar\_1621 | nitrogen regulatory protein P-II; K02590 nitrogen regulatory protein PII 2 |  |
| cpc:Cpar\_1622 | nitrogenase molybdenum-iron protein alpha chain (EC:1.18.6.1); K02586 nitrogenase molybdenum-iron protein alpha chain [EC:1.18.6.1] | ec:1.18.6.1 |
| cpc:Cpar\_1623 | nitrogenase molybdenum-iron protein beta chain (EC:1.18.6.1); K02591 nitrogenase molybdenum-iron protein beta chain [EC:1.18.6.1] | ec:1.18.6.1 |
| cpc:Cpar\_1624 | nitrogenase MoFe cofactor biosynthesis protein NifE (EC:1.18.6.1); K02587 nitrogenase molybdenum-cofactor synthesis protein NifE |  |
| cpc:Cpar\_1625 | nitrogenase (EC:1.18.6.1); K02592 nitrogenase molybdenum-iron protein NifN |  |
| cpc:Cpar\_1626 | nitrogenase cofactor biosynthesis protein NifB; K02585 nitrogen fixation protein NifB |  |
| cpc:Cpar\_1627 | ferredoxin, 2Fe-2S |  |
| cpc:Cpar\_1628 | cytochrome c |  |
| cpc:Cpar\_1629 | TonB-dependent receptor; K02014 iron complex outermembrane recepter protein |  |
| cpc:Cpar\_1630 | ModE family transcriptional regulator; K02019 molybdate transport system regulatory protein |  |
| cpc:Cpar\_1631 | molybdenum ABC transporter periplasmic molybdate-binding protein; K02020 molybdate transport system substrate-binding protein |  |
| cpc:Cpar\_1632 | radical SAM domain-containing protein |  |
| cpc:Cpar\_1633 | hydrogenase expression/formation protein HypE; K04655 hydrogenase expression/formation protein HypE |  |
| cpc:Cpar\_1634 | Sel1 domain-containing protein repeat-containing protein; K07126 |  |
| cpc:Cpar\_1635 | hydrogenase expression/formation protein HypD; K04654 hydrogenase expression/formation protein HypD |  |
| cpc:Cpar\_1636 | hydrogenase assembly chaperone HypC/HupF; K04653 hydrogenase expression/formation protein HypC |  |
| cpc:Cpar\_1637 | hypothetical protein |  |
| cpc:Cpar\_1638 | (NiFe) hydrogenase maturation protein HypF; K04656 hydrogenase maturation protein HypF |  |
| cpc:Cpar\_1639 | hydrogenase accessory protein HypB; K04652 hydrogenase nickel incorporation protein HypB |  |
| cpc:Cpar\_1640 | hydrogenase nickel insertion protein HypA; K04651 hydrogenase nickel incorporation protein HypA/HybF |  |

  
**Neighborhood Representations for "lhk:LHK\_02456"**  

| ID | Annotation | EC number |
| --- | --- | --- |
| lhk:LHK\_02446 | hypothetical protein |  |
| lhk:LHK\_02447 | xthA; XthA (EC:3.1.11.2); K01142 exodeoxyribonuclease III [EC:3.1.11.2] | ec:3.1.11.2 |
| lhk:LHK\_02448 | aspS; aspartyl-tRNA synthetase (EC:6.1.1.12); K01876 aspartyl-tRNA synthetase [EC:6.1.1.12] | ec:6.1.1.12 |
| lhk:LHK\_02449 | hypothetical protein |  |
| lhk:LHK\_02450 | Type I antifreeze protein |  |
| lhk:LHK\_02451 | hypothetical protein |  |
| lhk:LHK\_02452 | ptb; phosphate acetyltransferase (EC:2.3.1.8); K00625 phosphate acetyltransferase [EC:2.3.1.8] | ec:2.3.1.8 |
| lhk:LHK\_02453 | member of the acetate kinase family (EC:2.7.2.1); K00925 acetate kinase [EC:2.7.2.1] | ec:2.7.2.1 |
| lhk:LHK\_02454 | ABC transporter; K02471 putative ATP-binding cassette transporter |  |
| lhk:LHK\_02455 | Methyl-accepting chemotaxis protein |  |
| lhk:LHK\_02456 | modE; ModE; K02019 molybdate transport system regulatory protein |  |
| lhk:LHK\_02457 | modA; ModA; K02020 molybdate transport system substrate-binding protein |  |
| lhk:LHK\_02458 | molibdenum binding protein; K02019 molybdate transport system regulatory protein |  |
| lhk:LHK\_02459 | modB; ModB; K02018 molybdate transport system permease protein |  |
| lhk:LHK\_02460 | Molybdenum transport ATP-binding protein (EC:3.6.3.29); K02017 molybdate transport system ATP-binding protein [EC:3.6.3.29] | ec:3.6.3.29 |
| lhk:LHK\_02461 | hypothetical protein |  |
| lhk:LHK\_02462 | hypothetical protein |  |
| lhk:LHK\_02463 | Integrase |  |
| lhk:LHK\_02464 | hypothetical protein |  |
| lhk:LHK\_02465 | hypothetical protein |  |
| lhk:LHK\_02466 | hypothetical protein |  |

  
**Neighborhood Representations for "cts:Ctha\_1482"**  

| ID | Annotation | EC number |
| --- | --- | --- |
| cts:Ctha\_1472 | hypothetical protein |  |
| cts:Ctha\_1473 | alkyl hydroperoxide reductase; K03564 peroxiredoxin Q/BCP [EC:1.11.1.15] | ec:1.11.1.15 |
| cts:Ctha\_1474 | hypothetical protein |  |
| cts:Ctha\_1475 | ABC transporter-like protein; K01990 ABC-2 type transport system ATP-binding protein |  |
| cts:Ctha\_1476 | hypothetical protein |  |
| cts:Ctha\_1477 | hypothetical protein |  |
| cts:Ctha\_1478 | mammalian cell entry related domain-containing protein; K02067 putative ABC transport system substrate-binding protein |  |
| cts:Ctha\_1479 | tyrosine recombinase XerD; K04763 integrase/recombinase XerD |  |
| cts:Ctha\_1480 | hypothetical protein |  |
| cts:Ctha\_1481 | adenylate/guanylate cyclase with integral membrane sensor; K01768 adenylate cyclase [EC:4.6.1.1] | ec:4.6.1.1 |
| cts:Ctha\_1482 | ModE family transcriptional regulator; K02019 molybdate transport system regulatory protein |  |
| cts:Ctha\_1483 | multi-sensor signal transduction histidine kinase |  |
| cts:Ctha\_1484 | TonB-dependent receptor; K02014 iron complex outermembrane recepter protein |  |
| cts:Ctha\_1485 | chaperonin Cpn10 |  |
| cts:Ctha\_1486 | hypothetical protein |  |
| cts:Ctha\_1487 | quinolinate synthetase complex subunit A; K03517 quinolinate synthase [EC:2.5.1.72] | ec:2.5.1.72 |
| cts:Ctha\_1488 | AIG2 family protein |  |
| cts:Ctha\_1489 | hypothetical protein; K03975 membrane-associated protein |  |
| cts:Ctha\_1490 | hypothetical protein; K08974 putative membrane protein |  |
| cts:Ctha\_1491 | 2-oxoglutarate ferredoxin oxidoreductase subunit beta; K00175 2-oxoglutarate ferredoxin oxidoreductase subunit beta [EC:1.2.7.3] | ec:1.2.7.3 |
| cts:Ctha\_1492 | pyruvate flavodoxin/ferredoxin oxidoreductase domain-containing protein; K00174 2-oxoglutarate ferredoxin oxidoreductase subunit alpha [EC:1.2.7.3] | ec:1.2.7.3 |

  
**Neighborhood Representations for "cte:CT1543"**  

| ID | Annotation | EC number |
| --- | --- | --- |
| cte:CT1533 | nifH; nitrogenase reductase; K02588 nitrogenase iron protein NifH [EC:1.18.6.1] | ec:1.18.6.1 |
| cte:CT1534 | P-II family nitrogen regulator; K02589 nitrogen regulatory protein PII 1 |  |
| cte:CT1535 | P-II family nitrogen regulator; K02590 nitrogen regulatory protein PII 2 |  |
| cte:CT1536 | nifD; nitrogenase molybdenum-iron protein subunit alpha; K02586 nitrogenase molybdenum-iron protein alpha chain [EC:1.18.6.1] | ec:1.18.6.1 |
| cte:CT1537 | nifK; nitrogenase molybdenum-iron protein subunit beta; K02591 nitrogenase molybdenum-iron protein beta chain [EC:1.18.6.1] | ec:1.18.6.1 |
| cte:CT1538 | nifE; nitrogenase iron-molybdenum cofactor biosynthesis protein NifE; K02587 nitrogenase molybdenum-cofactor synthesis protein NifE |  |
| cte:CT1539 | nifN; nitrogenase iron-molybdenum cofactor biosynthesis protein NifN; K02592 nitrogenase molybdenum-iron protein NifN |  |
| cte:CT1540 | nifB; NifB protein; K02585 nitrogen fixation protein NifB |  |
| cte:CT1541 | ferredoxin, 2Fe-2S |  |
| cte:CT1542 | TonB-dependent receptor; K02014 iron complex outermembrane recepter protein |  |
| cte:CT1543 | modE; molybdenum transport protein ModE; K02019 molybdate transport system regulatory protein |  |
| cte:CT1544 | modA-2; molybdenum ABC transporter periplasmic molybdenum-binding protein; K02020 molybdate transport system substrate-binding protein |  |
| cte:CT1545 | relA; GTP pyrophosphokinase; K00951 GTP pyrophosphokinase [EC:2.7.6.5] | ec:2.7.6.5 |
| cte:CT1546 | uvrB; excinuclease ABC subunit B; K03702 excinuclease ABC subunit B |  |
| cte:CT1547 | hypothetical protein |  |
| cte:CT1548 | M16 family peptidase |  |
| cte:CT1549 | hypothetical protein; K11720 lipopolysaccharide export system permease protein |  |
| cte:CT1550 | hypothetical protein; K09134 hypothetical protein |  |
| cte:CT1551 | sigA; this region contains an authentic frame shift and is not the result of a sequencing artifact. |  |
| cte:CT1552 | valS; valyl-tRNA synthetase (EC:6.1.1.9); K01873 valyl-tRNA synthetase [EC:6.1.1.9] | ec:6.1.1.9 |
| cte:CT1553 | clpP; ATP-dependent Clp protease, proteolytic subunit ClpP; K01358 ATP-dependent Clp protease, protease subunit [EC:3.4.21.92] | ec:3.4.21.92 |

  
**Neighborhood Representations for "pvi:Cvib\_1354"**  

| ID | Annotation | EC number |
| --- | --- | --- |
| pvi:Cvib\_1344 | nitrogen regulatory protein P-II; K02589 nitrogen regulatory protein PII 1 |  |
| pvi:Cvib\_1345 | nitrogen regulatory protein P-II; K02590 nitrogen regulatory protein PII 2 |  |
| pvi:Cvib\_1346 | nitrogenase molybdenum-iron protein subunit alpha (EC:1.18.6.1); K02586 nitrogenase molybdenum-iron protein alpha chain [EC:1.18.6.1] | ec:1.18.6.1 |
| pvi:Cvib\_1347 | nitrogenase molybdenum-iron protein subunit beta (EC:1.18.6.1); K02591 nitrogenase molybdenum-iron protein beta chain [EC:1.18.6.1] | ec:1.18.6.1 |
| pvi:Cvib\_1348 | nitrogenase MoFe cofactor biosynthesis protein NifE (EC:1.18.6.1); K02587 nitrogenase molybdenum-cofactor synthesis protein NifE |  |
| pvi:Cvib\_1349 | nitrogenase (EC:1.18.6.1); K02592 nitrogenase molybdenum-iron protein NifN |  |
| pvi:Cvib\_1350 | nitrogenase cofactor biosynthesis protein NifB; K02585 nitrogen fixation protein NifB |  |
| pvi:Cvib\_1351 | ferredoxin, 2Fe-2S |  |
| pvi:Cvib\_1352 | cytochrome c |  |
| pvi:Cvib\_1353 | TonB-dependent receptor; K02014 iron complex outermembrane recepter protein |  |
| pvi:Cvib\_1354 | ModE family transcriptional regulator; K02019 molybdate transport system regulatory protein |  |
| pvi:Cvib\_1355 | GTP cyclohydrolase subunit MoaA; K03639 molybdenum cofactor biosynthesis protein |  |
| pvi:Cvib\_1356 | MOSC domain-containing protein |  |
| pvi:Cvib\_1357 | pseudogene |  |
| pvi:Cvib\_1358 | cystathionine gamma-synthase (EC:2.5.1.48); K01739 cystathionine gamma-synthase [EC:2.5.1.48] | ec:2.5.1.48 |
| pvi:Cvib\_1359 | thiamine biosynthesis protein ThiS; K03154 sulfur carrier protein |  |
| pvi:Cvib\_1360 | thiG; thiazole synthase; K03149 thiamine biosynthesis ThiG |  |
| pvi:Cvib\_1361 | thiH; thiamine biosynthesis protein ThiH; K03150 thiamine biosynthesis ThiH |  |
| pvi:Cvib\_1362 | UBA/THIF-type NAD/FAD binding protein |  |
| pvi:Cvib\_1363 | hypothetical protein |  |
| pvi:Cvib\_1364 | cytochrome c assembly protein |  |

  
**Neighborhood Representations for "cph:Cpha266\_0735"**  

| ID | Annotation | EC number |
| --- | --- | --- |
| cph:Cpha266\_0725 | hydrogenase expression/formation protein HypE; K04655 hydrogenase expression/formation protein HypE |  |
| cph:Cpha266\_0726 | pyridine nucleotide-disulfide oxidoreductase dimerisation region; K00382 dihydrolipoamide dehydrogenase [EC:1.8.1.4] | ec:1.8.1.4 |
| cph:Cpha266\_0727 | hypothetical protein |  |
| cph:Cpha266\_0728 | UBA/THIF-type NAD/FAD binding protein |  |
| cph:Cpha266\_0729 | thiH; thiamine biosynthesis protein ThiH; K03150 thiamine biosynthesis ThiH |  |
| cph:Cpha266\_0730 | thiG; thiazole synthase; K03149 thiamine biosynthesis ThiG |  |
| cph:Cpha266\_0731 | thiamine biosynthesis protein ThiS; K03154 sulfur carrier protein |  |
| cph:Cpha266\_0732 | cystathionine gamma-synthase (EC:2.5.1.48); K01739 cystathionine gamma-synthase [EC:2.5.1.48] | ec:2.5.1.48 |
| cph:Cpha266\_0733 | MOSC domain-containing protein |  |
| cph:Cpha266\_0734 | GTP cyclohydrolase subunit MoaA; K03639 molybdenum cofactor biosynthesis protein |  |
| cph:Cpha266\_0735 | ModE family transcriptional regulator; K02019 molybdate transport system regulatory protein |  |
| cph:Cpha266\_0736 | ferredoxin, 2Fe-2S |  |
| cph:Cpha266\_0737 | nitrogenase cofactor biosynthesis protein NifB; K02585 nitrogen fixation protein NifB |  |
| cph:Cpha266\_0738 | nitrogenase (EC:1.18.6.1); K02592 nitrogenase molybdenum-iron protein NifN |  |
| cph:Cpha266\_0739 | pseudogene |  |
| cph:Cpha266\_0740 | nitrogenase molybdenum-iron protein subunit beta (EC:1.18.6.1); K02591 nitrogenase molybdenum-iron protein beta chain [EC:1.18.6.1] | ec:1.18.6.1 |
| cph:Cpha266\_0741 | nitrogenase molybdenum-iron protein subunit alpha (EC:1.18.6.1); K02586 nitrogenase molybdenum-iron protein alpha chain [EC:1.18.6.1] | ec:1.18.6.1 |
| cph:Cpha266\_0742 | nitrogen regulatory protein P-II; K02590 nitrogen regulatory protein PII 2 |  |
| cph:Cpha266\_0743 | nitrogen regulatory protein P-II; K02589 nitrogen regulatory protein PII 1 |  |
| cph:Cpha266\_0744 | nifH; nitrogenase reductase (EC:1.18.6.1); K02588 nitrogenase iron protein NifH [EC:1.18.6.1] | ec:1.18.6.1 |
| cph:Cpha266\_0745 | cation diffusion facilitator family transporter |  |

  
**Neighborhood Representations for "paa:Paes\_1638"**  

| ID | Annotation | EC number |
| --- | --- | --- |
| paa:Paes\_1628 | nitrogen regulatory protein P-II; K02589 nitrogen regulatory protein PII 1 |  |
| paa:Paes\_1629 | nitrogen regulatory protein P-II; K02590 nitrogen regulatory protein PII 2 |  |
| paa:Paes\_1630 | nitrogenase molybdenum-iron protein subunit alpha (EC:1.18.6.1); K02586 nitrogenase molybdenum-iron protein alpha chain [EC:1.18.6.1] | ec:1.18.6.1 |
| paa:Paes\_1631 | nitrogenase molybdenum-iron protein subunit beta (EC:1.18.6.1); K02591 nitrogenase molybdenum-iron protein beta chain [EC:1.18.6.1] | ec:1.18.6.1 |
| paa:Paes\_1632 | nitrogenase MoFe cofactor biosynthesis protein NifE (EC:1.18.6.1); K02587 nitrogenase molybdenum-cofactor synthesis protein NifE |  |
| paa:Paes\_1633 | nitrogenase (EC:1.18.6.1); K02592 nitrogenase molybdenum-iron protein NifN |  |
| paa:Paes\_1634 | nitrogenase cofactor biosynthesis protein NifB; K02585 nitrogen fixation protein NifB |  |
| paa:Paes\_1635 | ferredoxin, 2Fe-2S |  |
| paa:Paes\_1636 | molybdenum ABC transporter periplasmic molybdate-binding protein; K02020 molybdate transport system substrate-binding protein |  |
| paa:Paes\_1637 | binding-protein-dependent transport system inner membrane protein; K02018 molybdate transport system permease protein |  |
| paa:Paes\_1638 | ModE family transcriptional regulator; K02019 molybdate transport system regulatory protein |  |
| paa:Paes\_1639 | histidine kinase |  |
| paa:Paes\_1640 | hydrogenase expression/formation protein HypE; K04655 hydrogenase expression/formation protein HypE |  |
| paa:Paes\_1641 | hydrogenase expression/formation protein HypD; K04654 hydrogenase expression/formation protein HypD |  |
| paa:Paes\_1642 | hydrogenase assembly chaperone HypC/HupF; K04653 hydrogenase expression/formation protein HypC |  |
| paa:Paes\_1643 | (NiFe) hydrogenase maturation protein HypF; K04656 hydrogenase maturation protein HypF |  |
| paa:Paes\_1644 | hydrogenase nickel incorporation protein HypB; K04652 hydrogenase nickel incorporation protein HypB |  |
| paa:Paes\_1645 | hydrogenase nickel incorporation protein HypA; K04651 hydrogenase nickel incorporation protein HypA/HybF |  |
| paa:Paes\_1646 | pyrB; aspartate carbamoyltransferase catalytic subunit (EC:2.1.3.2); K00609 aspartate carbamoyltransferase catalytic subunit [EC:2.1.3.2] | ec:2.1.3.2 |
| paa:Paes\_1647 | hypothetical protein |  |
| paa:Paes\_1648 | NUDIX hydrolase; K03574 8-oxo-dGTP diphosphatase [EC:3.6.1.55] | ec:3.6.1.55 |

  
**Neighborhood Representations for "plt:Plut\_1543"**  

| ID | Annotation | EC number |
| --- | --- | --- |
| plt:Plut\_1533 | nitrogenase MoFe cofactor biosynthesis protein NifE (EC:1.18.6.1); K02587 nitrogenase molybdenum-cofactor synthesis protein NifE |  |
| plt:Plut\_1534 | nitrogenase (EC:1.18.6.1); K02592 nitrogenase molybdenum-iron protein NifN |  |
| plt:Plut\_1535 | nitrogenase cofactor biosynthesis protein NifB; K02585 nitrogen fixation protein NifB |  |
| plt:Plut\_1536 | (2Fe-2S) ferredoxin |  |
| plt:Plut\_1537 | cytochrome c |  |
| plt:Plut\_1538 | cytochrome c |  |
| plt:Plut\_1539 | receptor; K16089 outer membrane receptor for ferrienterochelin and colicins |  |
| plt:Plut\_1540 | TonB-dependent receptor; K02014 iron complex outermembrane recepter protein |  |
| plt:Plut\_1541 | molybdenum ABC transporter periplasmic-binding protein; K02020 molybdate transport system substrate-binding protein |  |
| plt:Plut\_1542 | transmembrane protein; K02018 molybdate transport system permease protein |  |
| plt:Plut\_1543 | molybdenum-binding protein-like protein; K02019 molybdate transport system regulatory protein |  |
| plt:Plut\_1544 | Elongator protein 3/MiaB/NifB; K03639 molybdenum cofactor biosynthesis protein |  |
| plt:Plut\_1545 | hypothetical protein |  |
| plt:Plut\_1546 | hypothetical protein |  |
| plt:Plut\_1547 | hypothetical protein |  |
| plt:Plut\_1548 | cystathionine gamma-synthase (EC:2.5.1.48); K01739 cystathionine gamma-synthase [EC:2.5.1.48] | ec:2.5.1.48 |
| plt:Plut\_1549 | O-acetylhomoserine/O-acetylserine sulfhydrylase (EC:2.5.1.49); K01740 O-acetylhomoserine (thiol)-lyase [EC:2.5.1.49] | ec:2.5.1.49 |
| plt:Plut\_1550 | LuxR family transcriptional regulator |  |
| plt:Plut\_1551 | cysteine synthase K/M/A; K01738 cysteine synthase A [EC:2.5.1.47] | ec:2.5.1.47 |
| plt:Plut\_1552 | thiosulfate-binding protein; K02048 sulfate transport system substrate-binding protein |  |
| plt:Plut\_1553 | Sulfate ABC transporter permease CysT; K02046 sulfate transport system permease protein |  |

  
**Neighborhood Representations for "hch:HCH\_02455"**  

| ID | Annotation | EC number |
| --- | --- | --- |
| hch:HCH\_02446 | lolA; outer-membrane lipoprotein carrier protein; K03634 outer membrane lipoprotein carrier protein |  |
| hch:HCH\_02447 | recombination factor protein RarA; K07478 putative ATPase |  |
| hch:HCH\_02448 | crcB; hypothetical protein; K06199 CrcB protein |  |
| hch:HCH\_02449 | serS; seryl-tRNA synthetase (EC:6.1.1.11); K01875 seryl-tRNA synthetase [EC:6.1.1.11] | ec:6.1.1.11 |
| hch:HCH\_02450 | cysG; uroporphyrinogen-III methylase; K02302 uroporphyrin-III C-methyltransferase / precorrin-2 dehydrogenase / sirohydrochlorin ferrochelatase [EC:2.1.1.107 1.3.1.76 4.99.1.4] | ec:4.99.1.4 ec:1.3.1.76 ec:2.1.1.107 |
| hch:HCH\_02451 | hypothetical protein |  |
| hch:HCH\_02452 | modC; molybdate ABC transporter ATP-binding protein (EC:3.6.3.29); K02017 molybdate transport system ATP-binding protein [EC:3.6.3.29] | ec:3.6.3.29 |
| hch:HCH\_02453 | modB; molybdate ABC transporter permease; K02018 molybdate transport system permease protein |  |
| hch:HCH\_02454 | modA; molybdate ABC transporter periplasmic molybdate-binding protein; K02020 molybdate transport system substrate-binding protein |  |
| hch:HCH\_02456 | hypothetical protein |  |
| hch:HCH\_02455 | modE; molybdenum-binding protein; K02019 molybdate transport system regulatory protein |  |
| hch:HCH\_02457 | FHA domain-containing protein; K11894 type VI secretion system protein ImpI |  |
| hch:HCH\_02458 | methyl-accepting chemotaxis protein; K03406 methyl-accepting chemotaxis protein |  |
| hch:HCH\_02459 | HD-GYP domain-containing protein |  |
| hch:HCH\_02460 | hypothetical protein |  |
| hch:HCH\_02461 | hypothetical protein |  |
| hch:HCH\_02462 | response regulator; K07667 two-component system, OmpR family, KDP operon response regulator KdpE |  |
| hch:HCH\_02463 | hypothetical protein |  |
| hch:HCH\_02464 | cellulase |  |
| hch:HCH\_02465 | Ca2+-binding protein |  |
| hch:HCH\_02466 | transcriptional regulator; K02529 LacI family transcriptional regulator |  |

  
**Neighborhood Representations for "ppd:Ppro\_1529"**  

| ID | Annotation | EC number |
| --- | --- | --- |
| ppd:Ppro\_1519 | molybdopterin binding aldehyde oxidase and xanthine dehydrogenase |  |
| ppd:Ppro\_1520 | histidinol-phosphate aminotransferase; K00817 histidinol-phosphate aminotransferase [EC:2.6.1.9] | ec:2.6.1.9 |
| ppd:Ppro\_1521 | hypothetical protein |  |
| ppd:Ppro\_1522 | hypothetical protein |  |
| ppd:Ppro\_1523 | phosphopantetheine-binding protein |  |
| ppd:Ppro\_1524 | benzoate-CoA ligase family |  |
| ppd:Ppro\_1525 | putative anti-sigma regulatory factor |  |
| ppd:Ppro\_1526 | anti-sigma-factor antagonist |  |
| ppd:Ppro\_1527 | serine phosphatase; K07315 sigma-B regulation protein RsbU (phosphoserine phosphatase) |  |
| ppd:Ppro\_1528 | ABC transporter substrate-binding protein |  |
| ppd:Ppro\_1529 | ModE family transcriptional regulator; K02019 molybdate transport system regulatory protein |  |
| ppd:Ppro\_1530 | hypothetical protein |  |
| ppd:Ppro\_1531 | aspartate/glutamate/uridylate kinase |  |
| ppd:Ppro\_1532 | hypothetical protein |  |
| ppd:Ppro\_1533 | hypothetical protein |  |
| ppd:Ppro\_1534 | hypothetical protein |  |
| ppd:Ppro\_1535 | nitrate reductase subunit gamma |  |
| ppd:Ppro\_1536 | DsrC family protein; K11179 tRNA 2-thiouridine synthesizing protein E [EC:2.8.1.-] |  |
| ppd:Ppro\_1537 | TonB family protein; K03832 periplasmic protein TonB |  |
| ppd:Ppro\_1538 | hypothetical protein |  |
| ppd:Ppro\_1539 | cobalt transport protein; K16785 energy-coupling factor transport system permease protein |  |

  
**Neighborhood Representations for "slt:Slit\_0896"**  

| ID | Annotation | EC number |
| --- | --- | --- |
| slt:Slit\_0886 | dinitrogenase iron-molybdenum cofactor biosynthesis protein |  |
| slt:Slit\_0887 | hypothetical protein |  |
| slt:Slit\_0888 | hypothetical protein |  |
| slt:Slit\_0889 | metal dependent phosphohydrolase |  |
| slt:Slit\_0890 | O-methyltransferase family 3 |  |
| slt:Slit\_0891 | hypothetical protein |  |
| slt:Slit\_0892 | hypothetical protein |  |
| slt:Slit\_0893 | hypothetical protein |  |
| slt:Slit\_0894 | hypothetical protein |  |
| slt:Slit\_0895 | molybdenum ABC transporter substrate-binding protein; K02020 molybdate transport system substrate-binding protein |  |
| slt:Slit\_0896 | ModE family transcriptional regulator; K02019 molybdate transport system regulatory protein |  |
| slt:Slit\_0897 | molybdenum ABC transporter, periplasmic molybdate-binding protein; K02020 molybdate transport system substrate-binding protein |  |
| slt:Slit\_0898 | molybdate ABC transporter permease; K02018 molybdate transport system permease protein |  |
| slt:Slit\_0899 | molybdate ABC transporter, ATPase subunit; K02017 molybdate transport system ATP-binding protein [EC:3.6.3.29] | ec:3.6.3.29 |
| slt:Slit\_0900 | GCN5-related N-acetyltransferase |  |
| slt:Slit\_0901 | LRV FeS4 cluster domain protein |  |
| slt:Slit\_0902 | hypothetical protein |  |
| slt:Slit\_0903 | nitrogenase MoFe cofactor biosynthesis protein NifE; K02587 nitrogenase molybdenum-cofactor synthesis protein NifE |  |
| slt:Slit\_0904 | nitrogenase molybdenum-iron cofactor biosynthesis protein NifN (EC:1.18.6.1); K02592 nitrogenase molybdenum-iron protein NifN |  |
| slt:Slit\_0905 | dinitrogenase iron-molybdenum cofactor biosynthesis protein; K02596 nitrogen fixation protein NifX |  |
| slt:Slit\_0906 | positive regulator of sigma E, RseC/MucC; K03803 sigma-E factor negative regulatory protein RseC |  |

  
**Neighborhood Representations for "pca:Pcar\_0662"**  

| ID | Annotation | EC number |
| --- | --- | --- |
| pca:Pcar\_0652 | outer membrane channel OmpJ-like protein |  |
| pca:Pcar\_0653 | phoB; winged-helix phosphate transcriptional response regulator; K07657 two-component system, OmpR family, phosphate regulon response regulator PhoB |  |
| pca:Pcar\_0654 | phoR; phosphate sensor histidine kinase, HAMP and PAS domain-containing; K07636 two-component system, OmpR family, phosphate regulon sensor histidine kinase PhoR [EC:2.7.13.3] | ec:2.7.13.3 |
| pca:Pcar\_0655 | pstS-1; phosphate ABC transporter substrate-binding protein; K02040 phosphate transport system substrate-binding protein |  |
| pca:Pcar\_0656 | pstC-1; phosphate ABC transporter membrane protein PstC; K02037 phosphate transport system permease protein |  |
| pca:Pcar\_0657 | pstA-1; phosphate ABC transporter membrane protein PstA; K02038 phosphate transport system permease protein |  |
| pca:Pcar\_0658 | pstB-1; phosphate transporter ATP-binding protein; K02036 phosphate transport system ATP-binding protein [EC:3.6.3.27] | ec:3.6.3.27 |
| pca:Pcar\_0659 | phoU; phosphate transport system regulatory protein PhoU; K02039 phosphate transport system protein |  |
| pca:Pcar\_0660 | cytosine/adenosine deaminase |  |
| pca:Pcar\_0661 | FRG domain-containing protein |  |
| pca:Pcar\_0662 | modE; molybdate transport regulatory protein ModE; K02019 molybdate transport system regulatory protein |  |
| pca:Pcar\_0663 | sensor diguanylate cyclase/phosphoesterase, PAS domain-containing |  |
| pca:Pcar\_0664 | sensor helix-turn-helix transcriptional regulator, LuxR family, PAS domain-containing |  |
| pca:Pcar\_0665 | aorA-3; aldehyde:ferredoxin oxidoreductase, tungsten-containing |  |
| pca:Pcar\_0666 | TetR family transcriptional regulator |  |
| pca:Pcar\_0667 | efflux pump, outer membrane protein, NodT family |  |
| pca:Pcar\_0668 | RND family efflux pump membrane fusion protein |  |
| pca:Pcar\_0669 | RND family efflux pump inner membrane protein |  |
| pca:Pcar\_0670 | hypothetical protein |  |
| pca:Pcar\_0671 | ttcA; tRNA 2-thiocytidine biosynthesis protein TtcA; K14058 tRNA 2-thiocytidine biosynthesis protein TtcA |  |
| pca:Pcar\_0672 | serine phosphatase, SpoIIE domain-containing |  |

  
**Neighborhood Representations for "psa:PST\_1348"**  

| ID | Annotation | EC number |
| --- | --- | --- |
| psa:PST\_1338 | ferredoxin, 4Fe-4S |  |
| psa:PST\_1339 | ferredoxin, 2Fe-2S |  |
| psa:PST\_1340 | hypothetical protein |  |
| psa:PST\_1341 | hypothetical protein |  |
| psa:PST\_1342 | hypothetical protein; K09138 hypothetical protein |  |
| psa:PST\_1343 | hypothetical protein |  |
| psa:PST\_1344 | hypothetical protein |  |
| psa:PST\_1345 | modC; molybdenum transport protein ModC; K02017 molybdate transport system ATP-binding protein [EC:3.6.3.29] | ec:3.6.3.29 |
| psa:PST\_1346 | modB; molybdate ABC transporter permease; K02018 molybdate transport system permease protein |  |
| psa:PST\_1347 | modA; molybdenum ABC transporter periplasmic binding protein; K02020 molybdate transport system substrate-binding protein |  |
| psa:PST\_1348 | putative molybdenum-binding protein; K02019 molybdate transport system regulatory protein |  |
| psa:PST\_1349 | hesB; Fe-S cluster assembly protein |  |
| psa:PST\_1350 | nifU; Fe-S cluster assembly protein NifU; K13819 NifU-like protein |  |
| psa:PST\_1351 | nifS; nitrogenase metalloclusters biosynthesis protein NifS; K04487 cysteine desulfurase [EC:2.8.1.7] | ec:2.8.1.7 |
| psa:PST\_1352 | nifV; NifV protein, encodes a homocitrate synthase; K02594 homocitrate synthase NifV [EC:2.3.3.14] | ec:2.3.3.14 |
| psa:PST\_1353 | cysE; serine acetyltransferase (cysE-like); K00640 serine O-acetyltransferase [EC:2.3.1.30] | ec:2.3.1.30 |
| psa:PST\_1354 | hypothetical protein |  |
| psa:PST\_1355 | nifW; nitrogenase stabilizing/protective protein nifW; K02595 nitrogenase-stabilizing/protective protein |  |
| psa:PST\_1356 | nifZ; Fe-S cofactor synthesis protein; K02597 nitrogen fixation protein NifZ |  |
| psa:PST\_1357 | nifM; NifM protein a peptidyl-prolyl cis/trans isomerase |  |
| psa:PST\_1358 | clpX; ATP-dependent protease ATP-binding subunit ClpX; K03544 ATP-dependent Clp protease ATP-binding subunit ClpX |  |

  
**Neighborhood Representations for "har:HEAR3440"**  

| ID | Annotation | EC number |
| --- | --- | --- |
| har:HEAR3430 | NAD-dependent formate dehydrogenase subunit delta FdsD; K00126 formate dehydrogenase subunit delta [EC:1.2.1.2] | ec:1.2.1.2 |
| har:HEAR3431 | fdsC; formate dehydrogenase formation protein subunit FdsC; K02379 FdhD protein |  |
| har:HEAR3432 | NAD dependent formate dehydrogenase subunit alpha fdsA (EC:1.12.1.2 1.2.1.2); K00123 formate dehydrogenase major subunit [EC:1.2.1.2] | ec:1.2.1.2 |
| har:HEAR3433 | NAD dependent formate dehydrogenase subunit beta: fdsB (EC:1.6.99.5); K00124 formate dehydrogenase iron-sulfur subunit |  |
| har:HEAR3435 | NAD-dependent formate dehydrogenase subunit gamma: FdsG (EC:1.6.5.3); K00127 formate dehydrogenase subunit gamma |  |
| har:HEAR3434 | hypothetical protein |  |
| har:HEAR3436 | molybdenum-binding protein |  |
| har:HEAR3437 | modC; molybdate ABC transporter ATP-binding protein (EC:3.6.3.29); K02017 molybdate transport system ATP-binding protein [EC:3.6.3.29] | ec:3.6.3.29 |
| har:HEAR3438 | modB; molybdenum ABC transporter permease; K02018 molybdate transport system permease protein |  |
| har:HEAR3439 | modA; molybdate-binding periplasmic protein; K02020 molybdate transport system substrate-binding protein |  |
| har:HEAR3440 | modE; transcriptional repressor for molybdate uptake; K02019 molybdate transport system regulatory protein |  |
| har:HEAR3441 | transcriptional regulator |  |
| har:HEAR3442 | formate dehydrogenase (EC:1.2.2.1); K00122 formate dehydrogenase [EC:1.2.1.2] | ec:1.2.1.2 |
| har:HEAR3443 | hypothetical protein |  |
| har:HEAR3444 | hypothetical protein |  |
| har:HEAR3445 | signal peptide |  |
| har:HEAR3446 | regulatory lipoprotein |  |
| har:HEAR3447 | TonB-dependent outer membrane receptor; K02014 iron complex outermembrane recepter protein |  |
| har:HEAR3448 | hypothetical protein |  |
| har:HEAR3449 | major facilitator superfamily permease |  |
| har:HEAR3450 | oxalate/formate antiporter, (partial) |  |

  
**Neighborhood Representations for "mmw:Mmwyl1\_0976"**  

| ID | Annotation | EC number |
| --- | --- | --- |
| mmw:Mmwyl1\_0966 | sulfate ABC transporter permease; K02046 sulfate transport system permease protein |  |
| mmw:Mmwyl1\_0967 | sulfate ABC transporter periplasmic sulfate-binding protein; K02048 sulfate transport system substrate-binding protein |  |
| mmw:Mmwyl1\_0968 | N-acetyltransferase GCN5 |  |
| mmw:Mmwyl1\_0969 | methyl-accepting chemotaxis sensory transducer; K03406 methyl-accepting chemotaxis protein |  |
| mmw:Mmwyl1\_0970 | dicarboxylate carrier MatC domain-containing protein |  |
| mmw:Mmwyl1\_0971 | amidohydrolase 2; K07046 |  |
| mmw:Mmwyl1\_0972 | GntR family transcriptional regulator; K03710 GntR family transcriptional regulator |  |
| mmw:Mmwyl1\_0973 | hypothetical protein |  |
| mmw:Mmwyl1\_0974 | helix-turn-helix domain-containing protein |  |
| mmw:Mmwyl1\_0975 | isochorismatase hydrolase |  |
| mmw:Mmwyl1\_0976 | ModE family transcriptional regulator; K02019 molybdate transport system regulatory protein |  |
| mmw:Mmwyl1\_0977 | molybdate ABC transporter ATPase; K02017 molybdate transport system ATP-binding protein [EC:3.6.3.29] | ec:3.6.3.29 |
| mmw:Mmwyl1\_0978 | molybdate ABC transporter inner membrane subunit; K02018 molybdate transport system permease protein |  |
| mmw:Mmwyl1\_0979 | molybdenum ABC transporter periplasmic molybdate-binding protein; K02020 molybdate transport system substrate-binding protein |  |
| mmw:Mmwyl1\_0980 | metal dependent phosphohydrolase |  |
| mmw:Mmwyl1\_0981 | sodium:dicarboxylate symporter; K06956 |  |
| mmw:Mmwyl1\_0982 | methyl-accepting chemotaxis sensory transducer |  |
| mmw:Mmwyl1\_0983 | hypothetical protein |  |
| mmw:Mmwyl1\_0984 | hypothetical protein |  |
| mmw:Mmwyl1\_0985 | DEAD/DEAH box helicase; K11927 ATP-dependent RNA helicase RhlE [EC:3.6.4.13] | ec:3.6.4.13 |
| mmw:Mmwyl1\_0986 | transglutaminase domain-containing protein |  |

  
**Neighborhood Representations for "mms:mma\_3659"**  

| ID | Annotation | EC number |
| --- | --- | --- |
| mms:mma\_3649 | tupB; ABC-type tungstate transport system permease; K05772 tungstate transport system substrate-binding protein |  |
| mms:mma\_3650 | fdsD; NAD-dependent formate dehydrogenase subunit delta; K00126 formate dehydrogenase subunit delta [EC:1.2.1.2] | ec:1.2.1.2 |
| mms:mma\_3651 | fdsC; formate dehydrogenase associated protein; K02379 FdhD protein |  |
| mms:mma\_3652 | fdsA; NAD-dependent formate dehydrogenase subunit alpha (EC:1.2.1.2); K00123 formate dehydrogenase major subunit [EC:1.2.1.2] | ec:1.2.1.2 |
| mms:mma\_3653 | fdsB; NAD-dependent formate dehydrogenase subunit beta; K00124 formate dehydrogenase iron-sulfur subunit |  |
| mms:mma\_3654 | fdnI; formate dehydrogenase, cytochrome b556 subunit (EC:1.2.1.2); K00127 formate dehydrogenase subunit gamma |  |
| mms:mma\_3655 | LysR family transcriptional regulator |  |
| mms:mma\_3656 | modC; molybdate ABC transporter ATP-binding protein; K02017 molybdate transport system ATP-binding protein [EC:3.6.3.29] | ec:3.6.3.29 |
| mms:mma\_3657 | modB; molybdate transport system permease; K02018 molybdate transport system permease protein |  |
| mms:mma\_3658 | modA2; molybdate ABC transporter substrate-binding protein; K02020 molybdate transport system substrate-binding protein |  |
| mms:mma\_3659 | modE1; molybdenum transport regulatory protein ModE; K02019 molybdate transport system regulatory protein |  |
| mms:mma\_3660 | LysR family transcriptional regulator |  |
| mms:mma\_3661 | fdh; formate dehydrogenase (EC:1.2.1.2); K00122 formate dehydrogenase [EC:1.2.1.2] | ec:1.2.1.2 |
| mms:mma\_3662 | hypothetical protein |  |
| mms:mma\_3663 | hypothetical protein |  |
| mms:mma\_3664 | modE2; molybdenum-binding transcriptional regulator; K02019 molybdate transport system regulatory protein |  |
| mms:mma\_3665 | iclR2; IclR family transcriptional regulator |  |
| mms:mma\_3666 | hypothetical protein |  |
| mms:mma\_3667 | ansA2; L-asparaginase (EC:3.5.1.1); K01424 L-asparaginase [EC:3.5.1.1] | ec:3.5.1.1 |
| mms:mma\_3668 | major facilitator transporter |  |
| mms:mma\_3669 | thiol oxidoreductase |  |

  
**Neighborhood Representations for "tgr:Tgr7\_1573"**  

| ID | Annotation | EC number |
| --- | --- | --- |
| tgr:Tgr7\_1563 | NusB antitermination factor; K03625 N utilization substance protein B |  |
| tgr:Tgr7\_1564 | thiamine-monophosphate kinase (EC:2.7.4.16); K00946 thiamine-monophosphate kinase [EC:2.7.4.16] | ec:2.7.4.16 |
| tgr:Tgr7\_1565 | phosphatidylglycerophosphatase A; K01095 phosphatidylglycerophosphatase A [EC:3.1.3.27] | ec:3.1.3.27 |
| tgr:Tgr7\_1566 | aspartyl protease-like protein; K06985 aspartyl protease family protein |  |
| tgr:Tgr7\_1567 | flavodoxin oxidoreductase |  |
| tgr:Tgr7\_1568 | acriflavin resistance protein |  |
| tgr:Tgr7\_1569 | RND family efflux transporter MFP subunit |  |
| tgr:Tgr7\_1570 | molybdate ABC transporter ATPase; K02017 molybdate transport system ATP-binding protein [EC:3.6.3.29] | ec:3.6.3.29 |
| tgr:Tgr7\_1571 | molybdenum ABC transporter permease ModB; K02018 molybdate transport system permease protein |  |
| tgr:Tgr7\_1572 | molybdenum ABC transporter substrate-binding protein; K02020 molybdate transport system substrate-binding protein |  |
| tgr:Tgr7\_1573 | molybdenum-binding protein; K02019 molybdate transport system regulatory protein |  |
| tgr:Tgr7\_1574 | NAD(P)H dehydrogenase (quinone) |  |
| tgr:Tgr7\_1575 | serine-type D-Ala-D-Ala carboxypeptidase (EC:3.4.16.4); K07259 D-alanyl-D-alanine carboxypeptidase / D-alanyl-D-alanine-endopeptidase (penicillin-binding protein 4) [EC:3.4.16.4 3.4.21.-] | ec:3.4.16.4 |
| tgr:Tgr7\_1576 | ABC transporter; K02013 iron complex transport system ATP-binding protein [EC:3.6.3.34] | ec:3.6.3.34 |
| tgr:Tgr7\_1577 | transporter permease; K02015 iron complex transport system permease protein |  |
| tgr:Tgr7\_1578 | vitamin B12 transport protein; K02016 iron complex transport system substrate-binding protein |  |
| tgr:Tgr7\_1579 | gltX; glutamyl-tRNA synthetase; K01885 glutamyl-tRNA synthetase [EC:6.1.1.17] | ec:6.1.1.17 |
| tgr:Tgr7\_1580 | hypothetical protein |  |
| tgr:Tgr7\_1581 | hypothetical protein |  |
| tgr:Tgr7\_1582 | outer membrane efflux protein; K15725 cobalt-zinc-cadmium resistance protein CzcC |  |
| tgr:Tgr7\_1583 | RND family efflux transporter MFP subunit |  |

  
**Neighborhood Representations for "dar:Daro\_1477"**  

| ID | Annotation | EC number |
| --- | --- | --- |
| dar:Daro\_1467 | cytochrome c oxidase, subunit III; K02276 cytochrome c oxidase subunit III [EC:1.9.3.1] | ec:1.9.3.1 |
| dar:Daro\_1468 | cytochrome C oxidase assembly protein; K02258 cytochrome c oxidase assembly protein subunit 11 |  |
| dar:Daro\_1469 | hypothetical protein |  |
| dar:Daro\_1470 | cytochrome c oxidase, subunit I; K02274 cytochrome c oxidase subunit I [EC:1.9.3.1] | ec:1.9.3.1 |
| dar:Daro\_1471 | cytochrome c oxidase, subunit II:cytochrome c, class I:cytochrome C oxidase subunit II, transmembrane region; K02275 cytochrome c oxidase subunit II [EC:1.9.3.1] | ec:1.9.3.1 |
| dar:Daro\_1472 | electron transport protein SCO1/SenC; K07152 protein SCO1/2 |  |
| dar:Daro\_1473 | antibiotic biosynthesis monooxygenase |  |
| dar:Daro\_1474 | hypothetical protein |  |
| dar:Daro\_1475 | molybdenum utilization protein ModD; K03813 molybdenum transport protein [EC:2.4.2.-] |  |
| dar:Daro\_1476 | molybdenum ABC transporter periplasmic-binding protein; K02020 molybdate transport system substrate-binding protein |  |
| dar:Daro\_1477 | molybdenum-binding protein, N-terminal:molybdenum-pterin binding protein; K02019 molybdate transport system regulatory protein |  |
| dar:Daro\_1478 | molybdenum-pterin binding protein; K02019 molybdate transport system regulatory protein |  |
| dar:Daro\_1479 | hypothetical protein |  |
| dar:Daro\_1480 | helix-hairpin-helix DNA-binding motif-containing protein |  |
| dar:Daro\_1481 | hypothetical protein |  |
| dar:Daro\_1482 | hypothetical protein |  |
| dar:Daro\_1483 | flavodoxin FldA; K03839 flavodoxin I |  |
| dar:Daro\_1484 | hypothetical protein |  |
| dar:Daro\_1485 | (2Fe-2S) ferredoxin |  |
| dar:Daro\_1486 | hypothetical protein |  |
| dar:Daro\_1487 | Fis family transcriptional regulator; K02584 Nif-specific regulatory protein |  |

  
**Neighborhood Representations for "azo:azo3840"**  

| ID | Annotation | EC number |
| --- | --- | --- |
| azo:azo3830 | eutC; putative ethanolamine ammonia-lyase small subunit (EC:4.3.1.7); K03736 ethanolamine ammonia-lyase small subunit [EC:4.3.1.7] | ec:4.3.1.7 |
| azo:azo3831 | hypothetical protein |  |
| azo:azo3832 | tungstate ABC transporter permease; K05772 tungstate transport system substrate-binding protein |  |
| azo:azo3833 | putative molybdenum-binding protein |  |
| azo:azo3834 | hypothetical protein |  |
| azo:azo3835 | pgtC; putative regulatory lipoprotein |  |
| azo:azo3836 | TonB-dependent receptor; K02014 iron complex outermembrane recepter protein |  |
| azo:azo3837 | modC1; putative molybdenum transport system ATP-binding protein (EC:3.6.3.29); K02017 molybdate transport system ATP-binding protein [EC:3.6.3.29] | ec:3.6.3.29 |
| azo:azo3838 | modB1; putative molybdenum transport system permease; K02018 molybdate transport system permease protein |  |
| azo:azo3839 | modA1; putative molybdate transport system periplasmic-binding protein; K02020 molybdate transport system substrate-binding protein |  |
| azo:azo3840 | modE; putative molybdenum transport protein; K02019 molybdate transport system regulatory protein |  |
| azo:azo3841 | modG; putative molybdenum-pterin-binding-protein; K02019 molybdate transport system regulatory protein |  |
| azo:azo3842 | modC2; putative molybdate transport system ATP-binding protein (EC:3.6.3.29); K02017 molybdate transport system ATP-binding protein [EC:3.6.3.29] | ec:3.6.3.29 |
| azo:azo3843 | hypothetical protein; K05772 tungstate transport system substrate-binding protein |  |
| azo:azo3844 | putative TonB-dependent receptor; K02014 iron complex outermembrane recepter protein |  |
| azo:azo3845 | modB2; putative molybdenum ABC transporter permease; K02018 molybdate transport system permease protein |  |
| azo:azo3846 | modA2; putative molybdate transport system substrate-binding protein; K02020 molybdate transport system substrate-binding protein |  |
| azo:azo3847 | hypothetical protein |  |
| azo:azo3848 | yhjG; outer membrane protein; K07290 hypothetical protein |  |
| azo:azo3849 | diguanylate cyclase |  |
| azo:azo3850 | recD; DNA helicase (EC:3.1.11.5); K03581 exodeoxyribonuclease V alpha subunit [EC:3.1.11.5] | ec:3.1.11.5 |

  
**Neighborhood Representations for "tau:Tola\_1199"**  

| ID | Annotation | EC number |
| --- | --- | --- |
| tau:Tola\_1189 | pseudogene |  |
| tau:Tola\_1190 | filamentation induced by cAMP protein fic |  |
| tau:Tola\_1191 | GAF sensor-containing diguanylate cyclase |  |
| tau:Tola\_1192 | potassium-transporting ATPase subunit A (EC:3.6.3.12); K01546 K+-transporting ATPase ATPase A chain [EC:3.6.3.12] | ec:3.6.3.12 |
| tau:Tola\_1193 | K+-transporting ATPase subunit B; K01547 K+-transporting ATPase ATPase B chain [EC:3.6.3.12] | ec:3.6.3.12 |
| tau:Tola\_1194 | potassium-transporting ATPase, C subunit (EC:3.6.3.12); K01548 K+-transporting ATPase ATPase C chain [EC:3.6.3.12] | ec:3.6.3.12 |
| tau:Tola\_1195 | sensor protein KdpD; K07646 two-component system, OmpR family, sensor histidine kinase KdpD [EC:2.7.13.3] | ec:2.7.13.3 |
| tau:Tola\_1196 | two component transcriptional regulator, winged helix family; K07667 two-component system, OmpR family, KDP operon response regulator KdpE |  |
| tau:Tola\_1197 | pseudogene |  |
| tau:Tola\_1198 | pseudogene |  |
| tau:Tola\_1199 | ModE family transcriptional regulator; K02019 molybdate transport system regulatory protein |  |
| tau:Tola\_1200 | molybdenum ABC transporter periplasmic molybdate-binding protein; K02020 molybdate transport system substrate-binding protein |  |
| tau:Tola\_1201 | TOBE domain-containing protein; K02019 molybdate transport system regulatory protein |  |
| tau:Tola\_1202 | Fis family sigma-54 specific transcriptional regulator |  |
| tau:Tola\_1203 | Disulfide bond formation protein DsbB; K03611 disulfide bond formation protein DsbB |  |
| tau:Tola\_1204 | hypothetical protein |  |
| tau:Tola\_1205 | ferric uptake regulator, Fur family |  |
| tau:Tola\_1206 | hypothetical protein |  |
| tau:Tola\_1207 | thioesterase superfamily protein |  |
| tau:Tola\_1208 | beta-lactamase domain-containing protein; K06897 7,8-dihydropterin-6-yl-methyl-4-(beta-D-ribofuranosyl)aminobenzene 5'-phosphate synthase [EC:2.5.1.105] | ec:2.5.1.105 |
| tau:Tola\_1209 | pyridine nucleotide-disulfide oxidoreductase dimerisation region; K00382 dihydrolipoamide dehydrogenase [EC:1.8.1.4] | ec:1.8.1.4 |

  
**Neighborhood Representations for "tcx:Tcr\_1928"**  

| ID | Annotation | EC number |
| --- | --- | --- |
| tcx:Tcr\_1918 | 50S ribosomal protein L28; K02902 large subunit ribosomal protein L28 |  |
| tcx:Tcr\_1919 | 50S ribosomal protein L33; K02913 large subunit ribosomal protein L33 |  |
| tcx:Tcr\_1920 | diguanylate cyclase |  |
| tcx:Tcr\_1921 | formamidopyrimidine-DNA glycosylase (EC:3.2.2.23); K10563 formamidopyrimidine-DNA glycosylase [EC:3.2.2.23 4.2.99.18] | ec:3.2.2.23 ec:4.2.99.18 |
| tcx:Tcr\_1922 | hypothetical protein |  |
| tcx:Tcr\_1923 | ATPase; K01537 Ca2+-transporting ATPase [EC:3.6.3.8] | ec:3.6.3.8 |
| tcx:Tcr\_1924 | multi-sensor hybrid histidine kinase |  |
| tcx:Tcr\_1925 | molybdate ABC transporter ATP-binding protein; K02017 molybdate transport system ATP-binding protein [EC:3.6.3.29] | ec:3.6.3.29 |
| tcx:Tcr\_1926 | molybdate ABC transporter permease; K02018 molybdate transport system permease protein |  |
| tcx:Tcr\_1927 | molybdenum ABC transporter, periplasmic molybdate-binding protein; K02020 molybdate transport system substrate-binding protein |  |
| tcx:Tcr\_1928 | ModE family transcriptional regulator; K02019 molybdate transport system regulatory protein |  |
| tcx:Tcr\_1929 | hypothetical protein |  |
| tcx:Tcr\_1930 | coenzyme A biosynthesis protein (EC:2.7.7.3); K00954 pantetheine-phosphate adenylyltransferase [EC:2.7.7.3] | ec:2.7.7.3 |
| tcx:Tcr\_1931 | diguanylate cyclase/phosphodiesterase |  |
| tcx:Tcr\_1932 | hypothetical protein; K08316 16S rRNA (guanine966-N2)-methyltransferase [EC:2.1.1.171] | ec:2.1.1.171 |
| tcx:Tcr\_1933 | hypothetical protein |  |
| tcx:Tcr\_1934 | hypothetical protein |  |
| tcx:Tcr\_1935 | hypothetical protein; K09700 hypothetical protein |  |
| tcx:Tcr\_1936 | hypothetical protein |  |
| tcx:Tcr\_1937 | diguanylate cyclase |  |
| tcx:Tcr\_1938 | LrgB-like protein |  |

  
**Neighborhood Representations for "pna:Pnap\_2036"**  

| ID | Annotation | EC number |
| --- | --- | --- |
| pna:Pnap\_2026 | oxidoreductase domain-containing protein; K10219 2-hydroxy-4-carboxymuconate semialdehyde hemiacetal dehydrogenase [EC:1.1.1.312] | ec:1.1.1.312 |
| pna:Pnap\_2027 | protocatechuate 4,5-dioxygenase subunit beta (EC:1.13.11.8); K04101 protocatechuate 4,5-dioxygenase, beta chain [EC:1.13.11.8] | ec:1.13.11.8 |
| pna:Pnap\_2028 | protocatechuate 4,5-dioxygenase subunit alpha (EC:1.13.11.8); K04100 protocatechuate 4,5-dioxygenase, alpha chain [EC:1.13.11.8] | ec:1.13.11.8 |
| pna:Pnap\_2029 | amidohydrolase 2; K10221 2-pyrone-4,6-dicarboxylate lactonase [EC:3.1.1.57] | ec:3.1.1.57 |
| pna:Pnap\_2030 | hypothetical protein |  |
| pna:Pnap\_2031 | hypothetical protein; K10218 4-hydroxy-4-methyl-2-oxoglutarate aldolase [EC:4.1.3.17] | ec:4.1.3.17 |
| pna:Pnap\_2032 | amidohydrolase 2; K10220 4-oxalmesaconate hydratase [EC:4.2.1.83] | ec:4.2.1.83 |
| pna:Pnap\_2033 | hypothetical protein; K16514 4-oxalomesaconate tautomerase [EC:5.3.2.8] | ec:5.3.2.8 |
| pna:Pnap\_2034 | LysR family transcriptional regulator |  |
| pna:Pnap\_2035 | hypothetical protein |  |
| pna:Pnap\_2036 | ModE family transcriptional regulator; K02019 molybdate transport system regulatory protein |  |
| pna:Pnap\_2037 | molybdenum ABC transporter periplasmic molybdate-binding protein; K02020 molybdate transport system substrate-binding protein |  |
| pna:Pnap\_2038 | hypothetical protein |  |
| pna:Pnap\_2039 | modD protein; K03813 molybdenum transport protein [EC:2.4.2.-] |  |
| pna:Pnap\_2040 | molybdate ABC transporter inner membrane subunit; K02018 molybdate transport system permease protein |  |
| pna:Pnap\_2041 | molybdate ABC transporter ATPase; K02017 molybdate transport system ATP-binding protein [EC:3.6.3.29] | ec:3.6.3.29 |
| pna:Pnap\_2042 | pyridoxamine 5'-phosphate oxidase-like protein |  |
| pna:Pnap\_2043 | hypothetical protein; K06888 |  |
| pna:Pnap\_2044 | hypothetical protein |  |
| pna:Pnap\_2045 | Rieske (2Fe-2S) domain-containing protein; K15762 toluene monooxygenase system ferredoxin subunit |  |
| pna:Pnap\_2046 | hypothetical protein |  |

  
**Neighborhood Representations for "hna:Hneap\_0810"**  

| ID | Annotation | EC number |
| --- | --- | --- |
| hna:Hneap\_0800 | nitrogen regulatory protein P-II; K04751 nitrogen regulatory protein P-II 1 |  |
| hna:Hneap\_0801 | glycine oxidase ThiO; K03153 glycine oxidase [EC:1.4.3.19] | ec:1.4.3.19 |
| hna:Hneap\_0802 | hypothetical protein |  |
| hna:Hneap\_0803 | hypothetical protein |  |
| hna:Hneap\_0804 | RelA/SpoT family protein (EC:2.7.6.5); K00951 GTP pyrophosphokinase [EC:2.7.6.5] | ec:2.7.6.5 |
| hna:Hneap\_0805 | hypothetical protein; K09004 hypothetical protein |  |
| hna:Hneap\_0806 | hypothetical protein |  |
| hna:Hneap\_0807 | molybdate ABC transporter ATPase; K02017 molybdate transport system ATP-binding protein [EC:3.6.3.29] | ec:3.6.3.29 |
| hna:Hneap\_0808 | molybdate ABC transporter inner membrane subunit; K02018 molybdate transport system permease protein |  |
| hna:Hneap\_0809 | molybdenum ABC transporter substrate-binding protein; K02020 molybdate transport system substrate-binding protein |  |
| hna:Hneap\_0810 | ModE family transcriptional regulator; K02019 molybdate transport system regulatory protein |  |
| hna:Hneap\_0811 | pseudogene |  |
| hna:Hneap\_0813 | hypothetical protein |  |
| hna:Hneap\_0814 | deoxyribodipyrimidine photo-lyase (EC:4.1.99.3); K01669 deoxyribodipyrimidine photo-lyase [EC:4.1.99.3] | ec:4.1.99.3 |
| hna:Hneap\_0815 | RND family efflux transporter MFP subunit |  |
| hna:Hneap\_0816 | acriflavin resistance protein |  |
| hna:Hneap\_0817 | pseudogene |  |
| hna:Hneap\_0818 | CRISPR-associated protein Cas2 |  |
| hna:Hneap\_0819 | CRISPR-associated protein Cas1; K15342 CRISP-associated protein Cas1 |  |
| hna:Hneap\_0820 | DNA-binding domain-containing protein |  |
| hna:Hneap\_0821 | MazF family transcriptional regulator; K07171 mRNA interferase [EC:3.1.-.-] |  |

  
**Neighborhood Representations for "rsq:Rsph17025\_3358"**  

| ID | Annotation | EC number |
| --- | --- | --- |
| rsq:Rsph17025\_3348 | hypothetical protein; K00128 aldehyde dehydrogenase (NAD+) [EC:1.2.1.3] | ec:1.2.1.3 |
| rsq:Rsph17025\_3349 | ribokinase; K01619 deoxyribose-phosphate aldolase [EC:4.1.2.4] | ec:4.1.2.4 |
| rsq:Rsph17025\_3350 | hypothetical protein; K00852 ribokinase [EC:2.7.1.15] | ec:2.7.1.15 |
| rsq:Rsph17025\_3351 | monosaccharide-transporting ATPase (EC:3.6.3.17); K10820 monosaccharide-transporting ATPase [EC:3.6.3.17] | ec:3.6.3.17 |
| rsq:Rsph17025\_3352 | hypothetical protein; K02057 simple sugar transport system permease protein |  |
| rsq:Rsph17025\_3353 | hypothetical protein; K02057 simple sugar transport system permease protein |  |
| rsq:Rsph17025\_3354 | ABC-type xylose transport system periplasmic component-like protein; K02056 simple sugar transport system ATP-binding protein [EC:3.6.3.17] | ec:3.6.3.17 |
| rsq:Rsph17025\_3355 | hypothetical protein; K02058 simple sugar transport system substrate-binding protein |  |
| rsq:Rsph17025\_3356 | hypothetical protein; K11534 DeoR family transcriptional regulator, deoxyribose operon repressor |  |
| rsq:Rsph17025\_3357 | putative glutathione S-transferase YghU; K11209 GST-like protein |  |
| rsq:Rsph17025\_3358 | molybdenum ABC transporter, periplasmic molybdate-binding protein; K02019 molybdate transport system regulatory protein |  |
| rsq:Rsph17025\_3359 | hypothetical protein; K02020 molybdate transport system substrate-binding protein |  |
| rsq:Rsph17025\_3360 | pseudogene |  |
| rsq:Rsph17025\_3361 | hypothetical protein; K04655 hydrogenase expression/formation protein HypE |  |
| rsq:Rsph17025\_3362 | hydrogenase assembly chaperone hypC/hupF; K04654 hydrogenase expression/formation protein HypD |  |
| rsq:Rsph17025\_3363 | hypothetical protein; K04653 hydrogenase expression/formation protein HypC |  |
| rsq:Rsph17025\_3364 | hydrogenase accessory protein HypB |  |
| rsq:Rsph17025\_3365 | hydrogenase expression/synthesis, HypA; K04652 hydrogenase nickel incorporation protein HypB |  |
| rsq:Rsph17025\_3366 | hypothetical protein; K04651 hydrogenase nickel incorporation protein HypA/HybF |  |
| rsq:Rsph17025\_3367 | rubredoxin-type Fe(Cys)4 protein |  |
| rsq:Rsph17025\_3368 | HupH hydrogenase expression protein |  |

  
**Neighborhood Representations for "avn:Avin\_50680"**  

| ID | Annotation | EC number |
| --- | --- | --- |
| avn:Avin\_50580 | hoxG; membrane bound nickel-dependent hydrogenase, large subunit, HoxG; K06281 hydrogenase large subunit [EC:1.12.99.6] | ec:1.12.99.6 |
| avn:Avin\_50590 | hoxK; Uptake hydrogenase small subunit , HoxK; K06282 hydrogenase small subunit [EC:1.12.99.6] | ec:1.12.99.6 |
| avn:Avin\_50600 | transposase |  |
| avn:Avin\_50610 | general substrate transporter |  |
| avn:Avin\_50620 | hypothetical protein |  |
| avn:Avin\_50630 | Periplasmic hybrid histidine protein kinase, two-component |  |
| avn:Avin\_50640 | response regulator with metal dependent phosphohydrolase activity (two-component); K07814 putative two-component system response regulator |  |
| avn:Avin\_50650 | modC1; Mo transporter, inner membrane ATP-binding component, ModC1; K02017 molybdate transport system ATP-binding protein [EC:3.6.3.29] | ec:3.6.3.29 |
| avn:Avin\_50660 | modB1; Mo transporter membrane protein, ModB1; K02018 molybdate transport system permease protein |  |
| avn:Avin\_50670 | modA1; molybdenum transporter, periplasmic molybdate-binding protein; K02020 molybdate transport system substrate-binding protein |  |
| avn:Avin\_50680 | modE; Mo regulation, Mo processing homeostasis; K02019 molybdate transport system regulatory protein |  |
| avn:Avin\_50690 | modG; Mo processing, homeostasis; K02019 molybdate transport system regulatory protein |  |
| avn:Avin\_50700 | ABC transporter ATP-binding protein; K02017 molybdate transport system ATP-binding protein [EC:3.6.3.29] | ec:3.6.3.29 |
| avn:Avin\_50710 | ABC type tungstate transporter, permease; K05772 tungstate transport system substrate-binding protein |  |
| avn:Avin\_50720 | ABC transporter permease; K02018 molybdate transport system permease protein |  |
| avn:Avin\_50730 | modA3; molybdate ABC transporter substrate-binding protein; K02020 molybdate transport system substrate-binding protein |  |
| avn:Avin\_50750 | ImpA-like protein; K11902 type VI secretion system protein ImpA |  |
| avn:Avin\_50760 | hypothetical protein; K11891 type VI secretion system protein ImpL |  |
| avn:Avin\_50770 | hypothetical protein; K11892 type VI secretion system protein ImpK |  |
| avn:Avin\_50780 | hypothetical protein; K11893 type VI secretion system protein ImpJ |  |
| avn:Avin\_50790 | hypothetical protein; K11918 type VI secretion system protein |  |

  
**Neighborhood Representations for "rsp:RSP\_3874"**  

| ID | Annotation | EC number |
| --- | --- | --- |
| rsp:RSP\_3862 | chromosome replication initiation inhibitor protein; K05596 LysR family transcriptional regulator, chromosome initiation inhibitor |  |
| rsp:RSP\_3864 | negative transcriptional regulator; K07734 transcriptional regulator |  |
| rsp:RSP\_3865 | BadM/Rrf2 family transcriptional regulator |  |
| rsp:RSP\_3866 | hypothetical protein |  |
| rsp:RSP\_3867 | carboxymuconolactonedecarboxylase/alkylhydropero xidase |  |
| rsp:RSP\_3868 | molybdate ABC transporter inner membrane protein |  |
| rsp:RSP\_3869 | modC; ABC molybdate transporter, ATPase subunit ModC; K02017 molybdate transport system ATP-binding protein [EC:3.6.3.29] | ec:3.6.3.29 |
| rsp:RSP\_3871 | modA; ABC molybdate transporter, periplasmic binding protein ModA; K02020 molybdate transport system substrate-binding protein |  |
| rsp:RSP\_3872 | molybdenum-pterin binding protein |  |
| rsp:RSP\_3873 | modD; quinolinate phosphoribosyl transferase/molybdenum utilization protein ModD (EC:2.4.2.-); K03813 molybdenum transport protein [EC:2.4.2.-] |  |
| rsp:RSP\_3874 | mopB; transcriptional repressor, ModE; K02019 molybdate transport system regulatory protein |  |
| rsp:RSP\_3876 | hypothetical protein; K07090 |  |
| rsp:RSP\_3877 | glycoside hydrolase family protein |  |
| rsp:RSP\_3878 | flaA; flagellin |  |
| rsp:RSP\_3879 | flaF; flagellar biosynthesis regulatory protein FlaF |  |
| rsp:RSP\_3880 | flbT; flagellar biosynthesis repressor FlbT |  |
| rsp:RSP\_3881 | flgF; flagellar basal-body rod protein FLGF |  |
| rsp:RSP\_3882 | ABC di/oligopeptide transporter, ATPase subunit; K02032 peptide/nickel transport system ATP-binding protein |  |
| rsp:RSP\_3883 | ABC di/oligopeptide transporter, ATPase subunit; K02031 peptide/nickel transport system ATP-binding protein |  |
| rsp:RSP\_3884 | mandelate racemase/muconate lactonizing family protein |  |
| rsp:RSP\_3885 | hypothetical protein |  |

  
**Neighborhood Representations for "rsh:Rsph17029\_4166"**  

| ID | Annotation | EC number |
| --- | --- | --- |
| rsh:Rsph17029\_4156 | mandelate racemase/muconate lactonizing protein |  |
| rsh:Rsph17029\_4157 | oligopeptide/dipeptide ABC transporter, ATPase subunit; K02031 peptide/nickel transport system ATP-binding protein |  |
| rsh:Rsph17029\_4158 | oligopeptide/dipeptide ABC transporter, ATPase subunit; K02032 peptide/nickel transport system ATP-binding protein |  |
| rsh:Rsph17029\_4159 | hypothetical protein |  |
| rsh:Rsph17029\_4160 | flbT; flagellar biosynthesis repressor FlbT; K06601 flagellar protein FlbT |  |
| rsh:Rsph17029\_4161 | flaF; flagellar biosynthesis regulatory protein FlaF |  |
| rsh:Rsph17029\_4162 | flagellin domain-containing protein |  |
| rsh:Rsph17029\_4163 | hypothetical protein |  |
| rsh:Rsph17029\_4164 | hypothetical protein; K07090 |  |
| rsh:Rsph17029\_4165 | GntR domain-containing protein |  |
| rsh:Rsph17029\_4166 | ModE family transcriptional regulator; K02019 molybdate transport system regulatory protein |  |
| rsh:Rsph17029\_4167 | modD protein (EC:2.4.2.19); K03813 molybdenum transport protein [EC:2.4.2.-] |  |
| rsh:Rsph17029\_4168 | TOBE domain-containing protein |  |
| rsh:Rsph17029\_4169 | molybdenum ABC transporter, periplasmic molybdate-binding protein; K02020 molybdate transport system substrate-binding protein |  |
| rsh:Rsph17029\_4170 | modB; molybdate ABC transporter permease protein; K02018 molybdate transport system permease protein |  |
| rsh:Rsph17029\_4171 | molybdate ABC transporter, ATPase subunit; K02017 molybdate transport system ATP-binding protein [EC:3.6.3.29] | ec:3.6.3.29 |
| rsh:Rsph17029\_4172 | exopolysaccharide synthesis, ExoD |  |
| rsh:Rsph17029\_4173 | alkylhydroperoxidase |  |
| rsh:Rsph17029\_4174 | hypothetical protein |  |
| rsh:Rsph17029\_4175 | BadM/Rrf2 family transcriptional regulator |  |
| rsh:Rsph17029\_4176 | FMN-binding negative transcriptional regulator; K07734 transcriptional regulator |  |

  
**Neighborhood Representations for "rsk:RSKD131\_4480"**  

| ID | Annotation | EC number |
| --- | --- | --- |
| rsk:RSKD131\_4470 | BadM/Rrf2 family transcriptional regulator |  |
| rsk:RSKD131\_4471 | hypothetical protein |  |
| rsk:RSKD131\_4472 | alkylhydroperoxidase like protein, AhpD family |  |
| rsk:RSKD131\_4473 | molybdate ABC transporter permease |  |
| rsk:RSKD131\_4474 | molybdate ABC transporter, ATPase subunit; K02017 molybdate transport system ATP-binding protein [EC:3.6.3.29] | ec:3.6.3.29 |
| rsk:RSKD131\_4475 | modB; molybdate ABC transporter permease; K02018 molybdate transport system permease protein |  |
| rsk:RSKD131\_4476 | molybdate ABC transporter substrate-binding protein; K02020 molybdate transport system substrate-binding protein |  |
| rsk:RSKD131\_4477 | Molybdenum-pterin binding protein |  |
| rsk:RSKD131\_4478 | hypothetical protein |  |
| rsk:RSKD131\_4479 | modD protein; K03813 molybdenum transport protein [EC:2.4.2.-] |  |
| rsk:RSKD131\_4480 | transcriptional regulator, ModE family; K02019 molybdate transport system regulatory protein |  |
| rsk:RSKD131\_4481 | GntR family transcriptional regulator |  |
| rsk:RSKD131\_4482 | hypothetical protein; K07090 |  |
| rsk:RSKD131\_4483 | Glycoside hydrolase, family 16 |  |
| rsk:RSKD131\_4484 | Flagellin; K02406 flagellin |  |
| rsk:RSKD131\_4485 | flaF; flagellar biosynthesis regulatory protein FlaF; K06602 flagellar protein FlaF |  |
| rsk:RSKD131\_4486 | flbT; flagellar biosynthesis repressor FlbT; K06601 flagellar protein FlbT |  |
| rsk:RSKD131\_4487 | hypothetical protein |  |
| rsk:RSKD131\_4488 | hypothetical protein |  |
| rsk:RSKD131\_4489 | hypothetical protein |  |
| rsk:RSKD131\_4490 | hypothetical protein |  |

  
**Neighborhood Representations for "sdl:Sdel\_0810"**  

| ID | Annotation | EC number |
| --- | --- | --- |
| sdl:Sdel\_0800 | hypothetical protein |  |
| sdl:Sdel\_0801 | ATPase P; K01534 Cd2+/Zn2+-exporting ATPase [EC:3.6.3.3 3.6.3.5] | ec:3.6.3.5 ec:3.6.3.3 |
| sdl:Sdel\_0802 | regulatory protein ArsR; K03892 ArsR family transcriptional regulator |  |
| sdl:Sdel\_0803 | hypothetical protein |  |
| sdl:Sdel\_0804 | hypothetical protein |  |
| sdl:Sdel\_0805 | molybdopterin oxidoreductase Fe4S4 region; K00123 formate dehydrogenase major subunit [EC:1.2.1.2] | ec:1.2.1.2 |
| sdl:Sdel\_0806 | molybdopterin dinucleotide-binding protein; K00123 formate dehydrogenase major subunit [EC:1.2.1.2] | ec:1.2.1.2 |
| sdl:Sdel\_0807 | 4Fe-4S ferredoxin iron-sulfur binding domain-containing protein; K00124 formate dehydrogenase iron-sulfur subunit |  |
| sdl:Sdel\_0808 | formate dehydrogenase subunit gamma; K00127 formate dehydrogenase subunit gamma |  |
| sdl:Sdel\_0809 | modD protein (EC:2.4.2.19); K03813 molybdenum transport protein [EC:2.4.2.-] |  |
| sdl:Sdel\_0810 | LysR family transcriptional regulator; K02019 molybdate transport system regulatory protein |  |
| sdl:Sdel\_0811 | hypothetical protein |  |
| sdl:Sdel\_0812 | CrcB protein; K06199 CrcB protein |  |
| sdl:Sdel\_0813 | formylmethanofuran dehydrogenase subunit E region; K11261 formylmethanofuran dehydrogenase subunit E [EC:1.2.99.5] | ec:1.2.99.5 |
| sdl:Sdel\_0814 | ABC transporter; K02013 iron complex transport system ATP-binding protein [EC:3.6.3.34] | ec:3.6.3.34 |
| sdl:Sdel\_0815 | transporter permease; K02015 iron complex transport system permease protein |  |
| sdl:Sdel\_0816 | periplasmic binding protein; K02016 iron complex transport system substrate-binding protein |  |
| sdl:Sdel\_0817 | methyltransferase |  |
| sdl:Sdel\_0818 | chemotaxis sensory transducer |  |
| sdl:Sdel\_0819 | chemotaxis sensory transducer; K03406 methyl-accepting chemotaxis protein |  |
| sdl:Sdel\_0820 | thiamine-phosphate pyrophosphorylase (EC:2.5.1.3); K00788 thiamine-phosphate pyrophosphorylase [EC:2.5.1.3] | ec:2.5.1.3 |

  
**Neighborhood Representations for "mgm:Mmc1\_1731"**  

| ID | Annotation | EC number |
| --- | --- | --- |
| mgm:Mmc1\_1721 | hypothetical protein |  |
| mgm:Mmc1\_1722 | hypothetical protein |  |
| mgm:Mmc1\_1723 | hypothetical protein |  |
| mgm:Mmc1\_1724 | hypothetical protein |  |
| mgm:Mmc1\_1725 | hypothetical protein |  |
| mgm:Mmc1\_1726 | PAS/PAC sensor protein |  |
| mgm:Mmc1\_1727 | sulfotransferase |  |
| mgm:Mmc1\_1728 | citrate transporter |  |
| mgm:Mmc1\_1729 | ferrous iron transporter B; K04759 ferrous iron transport protein B |  |
| mgm:Mmc1\_1730 | FeoA family protein; K04758 ferrous iron transport protein A |  |
| mgm:Mmc1\_1731 | ModE family transcriptional regulator; K02019 molybdate transport system regulatory protein |  |
| mgm:Mmc1\_1732 | molybdenum ABC transporter periplasmic molybdate-binding protein; K02020 molybdate transport system substrate-binding protein |  |
| mgm:Mmc1\_1733 | diguanylate cyclase |  |
| mgm:Mmc1\_1734 | sulfate transporter; K03321 sulfate permease, SulP family |  |
| mgm:Mmc1\_1735 | cyclic nucleotide-binding protein |  |
| mgm:Mmc1\_1736 | hypothetical protein |  |
| mgm:Mmc1\_1737 | cobalamin synthesis protein, P47K |  |
| mgm:Mmc1\_1738 | DNA polymerase III subunits gamma/tau (EC:2.7.7.7); K02343 DNA polymerase III subunit gamma/tau [EC:2.7.7.7] | ec:2.7.7.7 |
| mgm:Mmc1\_1739 | hypothetical protein; K09747 hypothetical protein |  |
| mgm:Mmc1\_1740 | DNA replication and repair protein RecR; K06187 recombination protein RecR |  |
| mgm:Mmc1\_1741 | pyruvate flavodoxin/ferredoxin oxidoreductase domain-containing protein; K03737 putative pyruvate-flavodoxin oxidoreductase [EC:1.2.7.-] |  |

  
**Neighborhood Representations for "rpc:RPC\_0705"**  

| ID | Annotation | EC number |
| --- | --- | --- |
| rpc:RPC\_0695 | 2-oxoglutarate ferredoxin oxidoreductase subunit alpha (EC:1.2.7.3); K00174 2-oxoglutarate ferredoxin oxidoreductase subunit alpha [EC:1.2.7.3] | ec:1.2.7.3 |
| rpc:RPC\_0696 | 2-oxoglutarate ferredoxin oxidoreductase subunit beta; K00175 2-oxoglutarate ferredoxin oxidoreductase subunit beta [EC:1.2.7.3] | ec:1.2.7.3 |
| rpc:RPC\_0697 | pyruvate ferredoxin/flavodoxin oxidoreductase; K00177 2-oxoglutarate ferredoxin oxidoreductase subunit gamma [EC:1.2.7.3] | ec:1.2.7.3 |
| rpc:RPC\_0698 | phenylacetate--CoA ligase (EC:6.2.1.30); K01912 phenylacetate-CoA ligase [EC:6.2.1.30] | ec:6.2.1.30 |
| rpc:RPC\_0699 | tryptophan synthase subunit beta (EC:4.2.1.20); K06001 tryptophan synthase beta chain [EC:4.2.1.20] | ec:4.2.1.20 |
| rpc:RPC\_0700 | PfkB; K16370 6-phosphofructokinase 2 [EC:2.7.1.11] | ec:2.7.1.11 |
| rpc:RPC\_0701 | autoinducer synthesis protein |  |
| rpc:RPC\_0702 | LuxR family transcriptional regulator; K07782 LuxR family transcriptional regulator |  |
| rpc:RPC\_0703 | putative bifunctional glutamate synthase subunit beta/2-polyprenylphenol hydroxylase; K00266 glutamate synthase (NADPH/NADH) small chain [EC:1.4.1.13 1.4.1.14] | ec:1.4.1.14 ec:1.4.1.13 |
| rpc:RPC\_0704 | pyruvate flavodoxin/ferredoxin oxidoreductase-like; K03737 putative pyruvate-flavodoxin oxidoreductase [EC:1.2.7.-] |  |
| rpc:RPC\_0705 | ModE family transcriptional regulator; K02019 molybdate transport system regulatory protein |  |
| rpc:RPC\_0706 | molybdenum ABC transporter periplasmic molybdate-binding protein; K02020 molybdate transport system substrate-binding protein |  |
| rpc:RPC\_0707 | molybdate ABC transporter permease; K02018 molybdate transport system permease protein |  |
| rpc:RPC\_0708 | molybdate ABC transporter ATP-binding protein; K02017 molybdate transport system ATP-binding protein [EC:3.6.3.29] | ec:3.6.3.29 |
| rpc:RPC\_0709 | hypothetical protein |  |
| rpc:RPC\_0710 | hypothetical protein |  |
| rpc:RPC\_0711 | hypothetical protein |  |
| rpc:RPC\_0712 | hypothetical protein |  |
| rpc:RPC\_0713 | 2-nitropropane dioxygenase; K00459 nitronate monooxygenase [EC:1.13.12.16] | ec:1.13.12.16 |
| rpc:RPC\_0714 | hypothetical protein |  |
| rpc:RPC\_0715 | DedA family |  |

  
**Neighborhood Representations for "pag:PLES\_04831"**  

| ID | Annotation | EC number |
| --- | --- | --- |
| pag:PLES\_04731 | putative transcriptional regulator |  |
| pag:PLES\_04741 | putative N-acetyltransferase |  |
| pag:PLES\_04751 | putative transcriptional regulator |  |
| pag:PLES\_04761 | putative hydrolase; K01055 3-oxoadipate enol-lactonase [EC:3.1.1.24] | ec:3.1.1.24 |
| pag:PLES\_04771 | hypothetical protein |  |
| pag:PLES\_04781 | glcB; malate synthase G; K01638 malate synthase [EC:2.3.3.9] | ec:2.3.3.9 |
| pag:PLES\_04791 | putative acetyltransferase |  |
| pag:PLES\_04801 | putative ACT domain-containing protein |  |
| pag:PLES\_04811 | putative permease; K05786 chloramphenicol-sensitive protein RarD |  |
| pag:PLES\_04821 | serine/threonine protein kinase |  |
| pag:PLES\_04831 | putative molybdenum transport regulator; K02019 molybdate transport system regulatory protein |  |
| pag:PLES\_04841 | hypothetical protein; K05811 putative lipoprotein |  |
| pag:PLES\_04851 | putative phosphoribosyl transferase |  |
| pag:PLES\_04861 | hypothetical protein |  |
| pag:PLES\_04871 | putative transcriptional regulator |  |
| pag:PLES\_04881 | hypothetical protein; K07160 UPF0271 protein |  |
| pag:PLES\_04891 | hypothetical protein |  |
| pag:PLES\_04901 | acetyl-CoA carboxylase biotin carboxylase subunit; K01961 acetyl-CoA carboxylase, biotin carboxylase subunit [EC:6.4.1.2 6.3.4.14] | ec:6.3.4.14 ec:6.4.1.2 |
| pag:PLES\_04911 | putative hydrolase |  |
| pag:PLES\_04921 | putative hydrolase |  |
| pag:PLES\_04931 | hypothetical protein |  |

  
**Neighborhood Representations for "abu:Abu\_0013"**  

| ID | Annotation | EC number |
| --- | --- | --- |
| abu:Abu\_0003 | gyrB; DNA gyrase subunit B (EC:5.99.1.3); K02470 DNA gyrase subunit B [EC:5.99.1.3] | ec:5.99.1.3 |
| abu:Abu\_0004 | thioredoxin reductase (EC:1.8.1.9); K00384 thioredoxin reductase (NADPH) [EC:1.8.1.9] | ec:1.8.1.9 |
| abu:Abu\_0005 | queF; 7-cyano-7-deazaguanine reductase (EC:1.7.1.-); K09457 7-cyano-7-deazaguanine reductase [EC:1.7.1.13] | ec:1.7.1.13 |
| abu:Abu\_0006 | hypothetical protein |  |
| abu:Abu\_0007 | integral membrane protein |  |
| abu:Abu\_0008 | methyltransferase |  |
| abu:Abu\_0009 | modD; molybdenum ABC transporter ATP-binding protein (EC:3.6.3.29); K02017 molybdate transport system ATP-binding protein [EC:3.6.3.29] | ec:3.6.3.29 |
| abu:Abu\_0010 | modB; molybdenum ABC transporter permease; K02018 molybdate transport system permease protein |  |
| abu:Abu\_0011 | hypothetical protein |  |
| abu:Abu\_0012 | modA; molybdenum ABC transporter periplasmic molybdate-binding protein; K02020 molybdate transport system substrate-binding protein |  |
| abu:Abu\_0013 | modE; molybdenum-binding protein; K02019 molybdate transport system regulatory protein |  |
| abu:Abu\_0014 | hypothetical protein |  |
| abu:Abu\_0015 | LysR family transcriptional regulator |  |
| abu:Abu\_0016 | MFS permease |  |
| abu:Abu\_0017 | hypothetical protein |  |
| abu:Abu\_0018 | ribosomal large subunit pseudouridine synthase (EC:4.2.1.70); K06180 23S rRNA pseudouridine1911/1915/1917 synthase [EC:5.4.99.23] | ec:5.4.99.23 |
| abu:Abu\_0019 | purB; adenylosuccinate lyase (EC:4.3.2.2); K01756 adenylosuccinate lyase [EC:4.3.2.2] | ec:4.3.2.2 |
| abu:Abu\_0020 | nrdA; ribonucleotide-diphosphate reductase subunit alpha (EC:1.17.4.1); K00525 ribonucleoside-diphosphate reductase alpha chain [EC:1.17.4.1] | ec:1.17.4.1 |
| abu:Abu\_0021 | nrdF; ribonucleotide-diphosphate reductase subunit beta (EC:1.17.4.1); K00526 ribonucleoside-diphosphate reductase beta chain [EC:1.17.4.1] | ec:1.17.4.1 |
| abu:Abu\_0022 | hypothetical protein |  |
| abu:Abu\_0023 | hypothetical protein; K07107 acyl-CoA thioester hydrolase [EC:3.1.2.-] |  |

  
**Neighborhood Representations for "pae:PA0487"**  

| ID | Annotation | EC number |
| --- | --- | --- |
| pae:PA0477 | transcriptional regulator |  |
| pae:PA0478 | N-acetyltransferase |  |
| pae:PA0479 | transcriptional regulator |  |
| pae:PA0480 | hydrolase; K01055 3-oxoadipate enol-lactonase [EC:3.1.1.24] | ec:3.1.1.24 |
| pae:PA0481 | hypothetical protein |  |
| pae:PA0482 | glcB; malate synthase G (EC:2.3.3.9); K01638 malate synthase [EC:2.3.3.9] | ec:2.3.3.9 |
| pae:PA0483 | acetyltransferase |  |
| pae:PA0484 | hypothetical protein |  |
| pae:PA0485 | hypothetical protein; K05786 chloramphenicol-sensitive protein RarD |  |
| pae:PA0486 | serine/threonine protein kinase |  |
| pae:PA0487 | molybdenum transport regulator; K02019 molybdate transport system regulatory protein |  |
| pae:PA0488 | hypothetical protein; K05811 putative lipoprotein |  |
| pae:PA0489 | phosphoribosyl transferase |  |
| pae:PA0490 | hypothetical protein |  |
| pae:PA0491 | transcriptional regulator |  |
| pae:PA0492 | hypothetical protein; K07160 UPF0271 protein |  |
| pae:PA0493 | hypothetical protein |  |
| pae:PA0494 | acetyl-CoA carboxylase biotin carboxylase subunit; K01961 acetyl-CoA carboxylase, biotin carboxylase subunit [EC:6.4.1.2 6.3.4.14] | ec:6.3.4.14 ec:6.4.1.2 |
| pae:PA0495 | hypothetical protein |  |
| pae:PA0496 | hypothetical protein |  |
| pae:PA0497 | hypothetical protein |  |

  
**Neighborhood Representations for "pau:PA14\_06340"**  

| ID | Annotation | EC number |
| --- | --- | --- |
| pau:PA14\_06240 | LysR family transcriptional regulator |  |
| pau:PA14\_06250 | GNAT family acetyltransferase |  |
| pau:PA14\_06260 | LysR family transcriptional regulator |  |
| pau:PA14\_06270 | catD; hydrolase; K01055 3-oxoadipate enol-lactonase [EC:3.1.1.24] | ec:3.1.1.24 |
| pau:PA14\_06280 | hypothetical protein |  |
| pau:PA14\_06290 | glcB; malate synthase G (EC:2.3.3.9); K01638 malate synthase [EC:2.3.3.9] | ec:2.3.3.9 |
| pau:PA14\_06300 | GNAT family acetyltransferase |  |
| pau:PA14\_06310 | ACT domain-containing protein |  |
| pau:PA14\_06320 | rarD; hypothetical protein; K05786 chloramphenicol-sensitive protein RarD |  |
| pau:PA14\_06330 | serine/threonine protein kinase |  |
| pau:PA14\_06340 | modR; molybdenum transport regulator; K02019 molybdate transport system regulatory protein |  |
| pau:PA14\_06350 | yfiM; hypothetical protein; K05811 putative lipoprotein |  |
| pau:PA14\_06360 | comF; phosphoribosyl transferase |  |
| pau:PA14\_06390 | hypothetical protein |  |
| pau:PA14\_06400 | LysR family transcriptional regulator |  |
| pau:PA14\_06420 | hypothetical protein; K07160 UPF0271 protein |  |
| pau:PA14\_06430 | hypothetical protein |  |
| pau:PA14\_06450 | accC; acetyl-CoA carboxylase biotin carboxylase subunit (EC:6.4.1.2); K01961 acetyl-CoA carboxylase, biotin carboxylase subunit [EC:6.4.1.2 6.3.4.14] | ec:6.3.4.14 ec:6.4.1.2 |
| pau:PA14\_06460 | hypothetical protein |  |
| pau:PA14\_06480 | hydrolase |  |
| pau:PA14\_06500 | bioB; biotin synthase; K01012 biotin synthase [EC:2.8.1.6] | ec:2.8.1.6 |

  
**Neighborhood Representations for "rpa:RPA4718"**  

| ID | Annotation | EC number |
| --- | --- | --- |
| rpa:RPA4708 | 2-nitropropane dioxygenase; K00459 nitronate monooxygenase [EC:1.13.12.16] | ec:1.13.12.16 |
| rpa:RPA4709 | hypothetical protein |  |
| rpa:RPA4710 | hypothetical protein |  |
| rpa:RPA4711 | hypothetical protein |  |
| rpa:RPA4712 | hypothetical protein |  |
| rpa:RPA4713 | hypothetical protein |  |
| rpa:RPA4714 | hypothetical protein |  |
| rpa:RPA4715 | modC; molybdate ABC transporter ATP-binding protein; K02017 molybdate transport system ATP-binding protein [EC:3.6.3.29] | ec:3.6.3.29 |
| rpa:RPA4716 | modB; molybdate ABC transporter permease; K02018 molybdate transport system permease protein |  |
| rpa:RPA4717 | modA; molybdenum ABC transporter periplasmic molybdate-binding protein; K02020 molybdate transport system substrate-binding protein |  |
| rpa:RPA4718 | modE; ModE family transcriptional regulator; K02019 molybdate transport system regulatory protein |  |
| rpa:RPA4719 | molybdo-pterin binding protein |  |
| rpa:RPA4720 | hypothetical protein |  |
| rpa:RPA4721 | pyruvate-flavodoxin oxidoreductase; K03737 putative pyruvate-flavodoxin oxidoreductase [EC:1.2.7.-] |  |
| rpa:RPA4722 | bifunctional glutamate synthase subunit beta/2-polyprenylphenol hydroxylase; K00266 glutamate synthase (NADPH/NADH) small chain [EC:1.4.1.13 1.4.1.14] | ec:1.4.1.14 ec:1.4.1.13 |
| rpa:RPA4723 | polA; DNA polymerase I; K02335 DNA polymerase I [EC:2.7.7.7] | ec:2.7.7.7 |
| rpa:RPA4724 | pyrE; orotate phosphoribosyltransferase; K00762 orotate phosphoribosyltransferase [EC:2.4.2.10] | ec:2.4.2.10 |
| rpa:RPA4725 | hypothetical protein |  |
| rpa:RPA4726 | hypothetical protein |  |
| rpa:RPA4727 | glgA2; glycogen/starch/alpha-glucan phosphorylase; K00688 starch phosphorylase [EC:2.4.1.1] | ec:2.4.1.1 |
| rpa:RPA4728 | hypothetical protein |  |

  
**Neighborhood Representations for "rpt:Rpal\_5199"**  

| ID | Annotation | EC number |
| --- | --- | --- |
| rpt:Rpal\_5189 | 2-nitropropane dioxygenase; K00459 nitronate monooxygenase [EC:1.13.12.16] | ec:1.13.12.16 |
| rpt:Rpal\_5190 | hypothetical protein |  |
| rpt:Rpal\_5191 | hypothetical protein |  |
| rpt:Rpal\_5192 | hypothetical protein |  |
| rpt:Rpal\_5193 | hypothetical protein |  |
| rpt:Rpal\_5194 | peptidase PatA-like protein |  |
| rpt:Rpal\_5195 | hypothetical protein |  |
| rpt:Rpal\_5196 | molybdate ABC transporter ATPase; K02017 molybdate transport system ATP-binding protein [EC:3.6.3.29] | ec:3.6.3.29 |
| rpt:Rpal\_5197 | molybdate ABC transporter inner membrane subunit; K02018 molybdate transport system permease protein |  |
| rpt:Rpal\_5198 | molybdenum ABC transporter periplasmic molybdate-binding protein; K02020 molybdate transport system substrate-binding protein |  |
| rpt:Rpal\_5199 | ModE family transcriptional regulator; K02019 molybdate transport system regulatory protein |  |
| rpt:Rpal\_5200 | TOBE domain-containing protein |  |
| rpt:Rpal\_5201 | hypothetical protein |  |
| rpt:Rpal\_5202 | pyruvate flavodoxin/ferredoxin oxidoreductase domain-containing protein; K03737 putative pyruvate-flavodoxin oxidoreductase [EC:1.2.7.-] |  |
| rpt:Rpal\_5203 | bifunctional glutamate synthase subunit beta/2-polyprenylphenol hydroxylase; K00266 glutamate synthase (NADPH/NADH) small chain [EC:1.4.1.13 1.4.1.14] | ec:1.4.1.14 ec:1.4.1.13 |
| rpt:Rpal\_5204 | DNA polymerase I; K02335 DNA polymerase I [EC:2.7.7.7] | ec:2.7.7.7 |
| rpt:Rpal\_5205 | pyrE; orotate phosphoribosyltransferase; K00762 orotate phosphoribosyltransferase [EC:2.4.2.10] | ec:2.4.2.10 |
| rpt:Rpal\_5206 | hypothetical protein |  |
| rpt:Rpal\_5207 | hypothetical protein |  |
| rpt:Rpal\_5208 | glycogen/starch/alpha-glucan phosphorylase; K00688 starch phosphorylase [EC:2.4.1.1] | ec:2.4.1.1 |
| rpt:Rpal\_5209 | hypothetical protein |  |

  
**Neighborhood Representations for "mag:amb2949"**  

| ID | Annotation | EC number |
| --- | --- | --- |
| mag:amb2939 | hypothetical protein |  |
| mag:amb2940 | hypothetical protein |  |
| mag:amb2941 | Heme/copper-type cytochrome/quinol oxidase, subunit 3; K02164 nitric oxide reductase NorE protein |  |
| mag:amb2942 | nitric oxide reductase activation protein; K02448 nitric oxide reductase NorD protein |  |
| mag:amb2943 | denitrification regulatory protein nirQ; K04748 nitric oxide reductase NorQ protein |  |
| mag:amb2944 | nitric oxide reductase large subunit; K04561 nitric oxide reductase subunit B [EC:1.7.2.5] | ec:1.7.2.5 |
| mag:amb2945 | cytochrome c, mono- and diheme variants; K02305 nitric oxide reductase subunit C |  |
| mag:amb2946 | cAMP-binding protein - catabolite gene activator and regulatory subunit of cAMP-dependent protein kinase |  |
| mag:amb2947 | hypothetical protein |  |
| mag:amb2948 | hypothetical protein |  |
| mag:amb2949 | molybdenum-pterin binding protein mopA; K02019 molybdate transport system regulatory protein |  |
| mag:amb2950 | molybdopterin-binding protein |  |
| mag:amb2951 | ABC-type molybdate transport system, periplasmic component; K02020 molybdate transport system substrate-binding protein |  |
| mag:amb2952 | NifX protein |  |
| mag:amb2953 | nicotinate-nucleotide pyrophosphorylase; K03813 molybdenum transport protein [EC:2.4.2.-] |  |
| mag:amb2954 | Signal transduction histidine kinase |  |
| mag:amb2955 | response regulator |  |
| mag:amb2956 | response regulator |  |
| mag:amb2957 | hypothetical protein |  |
| mag:amb2958 | hypothetical protein |  |
| mag:amb2959 | hypothetical protein |  |

  
**Neighborhood Representations for "pmy:Pmen\_4091"**  

| ID | Annotation | EC number |
| --- | --- | --- |
| pmy:Pmen\_4081 | acyl-CoA dehydrogenase domain-containing protein |  |
| pmy:Pmen\_4082 | hypothetical protein |  |
| pmy:Pmen\_4083 | hypothetical protein |  |
| pmy:Pmen\_4084 | bioD; dithiobiotin synthetase (EC:6.3.3.3); K01935 dethiobiotin synthetase [EC:6.3.3.3] | ec:6.3.3.3 |
| pmy:Pmen\_4085 | biotin biosynthesis protein BioC; K02169 malonyl-CoA O-methyltransferase [EC:2.1.1.197] | ec:2.1.1.197 |
| pmy:Pmen\_4086 | carboxylesterase (EC:3.1.1.1); K02170 pimeloyl-[acyl-carrier protein] methyl ester esterase [EC:3.1.1.85] | ec:3.1.1.85 |
| pmy:Pmen\_4087 | 8-amino-7-oxononanoate synthase (EC:2.3.1.47); K00652 8-amino-7-oxononanoate synthase [EC:2.3.1.47] | ec:2.3.1.47 |
| pmy:Pmen\_4088 | biotin synthase (EC:2.8.1.6); K01012 biotin synthase [EC:2.8.1.6] | ec:2.8.1.6 |
| pmy:Pmen\_4089 | hypothetical protein |  |
| pmy:Pmen\_4090 | amidophosphoribosyltransferase-like protein |  |
| pmy:Pmen\_4091 | ModE family transcriptional regulator; K02019 molybdate transport system regulatory protein |  |
| pmy:Pmen\_4092 | serine/threonine protein kinase |  |
| pmy:Pmen\_4093 | transporter DMT superfamily protein; K05786 chloramphenicol-sensitive protein RarD |  |
| pmy:Pmen\_4094 | amino acid-binding ACT domain-containing protein |  |
| pmy:Pmen\_4095 | N-acetyltransferase GCN5 |  |
| pmy:Pmen\_4096 | malate synthase G (EC:2.3.3.9); K01638 malate synthase [EC:2.3.3.9] | ec:2.3.3.9 |
| pmy:Pmen\_4097 | hypothetical protein; K07182 CBS domain-containing protein |  |
| pmy:Pmen\_4098 | DNA polymerase III subunit epsilon; K02342 DNA polymerase III subunit epsilon [EC:2.7.7.7] | ec:2.7.7.7 |
| pmy:Pmen\_4099 | peptidase U32; K08303 putative protease [EC:3.4.-.-] |  |
| pmy:Pmen\_4100 | hypothetical protein |  |
| pmy:Pmen\_4101 | Na+/solute symporter; K14393 cation/acetate symporter |  |

  
**Neighborhood Representations for "cak:Caul\_2731"**  

| ID | Annotation | EC number |
| --- | --- | --- |
| cak:Caul\_2721 | xylose isomerase domain-containing protein |  |
| cak:Caul\_2722 | short-chain dehydrogenase/reductase SDR |  |
| cak:Caul\_2723 | thiamine pyrophosphate protein central region; K01652 acetolactate synthase I/II/III large subunit [EC:2.2.1.6] | ec:2.2.1.6 |
| cak:Caul\_2724 | hypothetical protein |  |
| cak:Caul\_2725 | glycosyl transferase family protein |  |
| cak:Caul\_2726 | TonB-dependent receptor plug |  |
| cak:Caul\_2727 | anti-FecI sigma factor FecR; K07165 transmembrane sensor |  |
| cak:Caul\_2728 | ECF subfamily RNA polymerase sigma-24 factor; K03088 RNA polymerase sigma-70 factor, ECF subfamily |  |
| cak:Caul\_2729 | hypothetical protein |  |
| cak:Caul\_2730 | hypothetical protein |  |
| cak:Caul\_2731 | ModE family transcriptional regulator; K02019 molybdate transport system regulatory protein |  |
| cak:Caul\_2732 | molybdate ABC transporter inner membrane subunit; K02018 molybdate transport system permease protein |  |
| cak:Caul\_2733 | taurine dioxygenase (EC:1.14.11.17); K03119 taurine dioxygenase [EC:1.14.11.17] | ec:1.14.11.17 |
| cak:Caul\_2734 | adenosylcobinamide kinase (EC:2.7.1.156); K02231 adenosylcobinamide kinase / adenosylcobinamide-phosphate guanylyltransferase [EC:2.7.1.156 2.7.7.62] | ec:2.7.7.62 ec:2.7.1.156 |
| cak:Caul\_2735 | TonB-dependent receptor; K16092 vitamin B12 transporter |  |
| cak:Caul\_2736 | L-threonine-O-3-phosphate decarboxylase; K02225 cobalamin biosynthetic protein CobC |  |
| cak:Caul\_2737 | cob(I)alamin adenosyltransferase (EC:2.5.1.17); K00798 cob(I)alamin adenosyltransferase [EC:2.5.1.17] | ec:2.5.1.17 |
| cak:Caul\_2738 | periplasmic-binding protein; K02016 iron complex transport system substrate-binding protein |  |
| cak:Caul\_2739 | transporter permease; K02015 iron complex transport system permease protein |  |
| cak:Caul\_2740 | ABC transporter-like protein; K02013 iron complex transport system ATP-binding protein [EC:3.6.3.34] | ec:3.6.3.34 |
| cak:Caul\_2741 | cobalamin biosynthesis protein CobD; K02227 adenosylcobinamide-phosphate synthase [EC:6.3.1.10] | ec:6.3.1.10 |

  
**Neighborhood Representations for "pmx:PERMA\_1919"**  

| ID | Annotation | EC number |
| --- | --- | --- |
| pmx:PERMA\_1909 | DNA polymerase beta family; K02347 DNA polymerase (family X) |  |
| pmx:PERMA\_1910 | hypothetical protein |  |
| pmx:PERMA\_1911 | gltX; glutamyl-tRNA synthetase (EC:6.1.1.17); K09698 nondiscriminating glutamyl-tRNA synthetase [EC:6.1.1.24] | ec:6.1.1.24 |
| pmx:PERMA\_1912 | dual specificity protein phosphatase |  |
| pmx:PERMA\_1914 | hypothetical protein |  |
| pmx:PERMA\_1913 | hypothetical protein |  |
| pmx:PERMA\_1915 | spermidine/putrescine import ATP-binding protein PotA (EC:3.6.3.31); K02017 molybdate transport system ATP-binding protein [EC:3.6.3.29] | ec:3.6.3.29 |
| pmx:PERMA\_1916 | modB; molybdate ABC transporter permease; K02018 molybdate transport system permease protein |  |
| pmx:PERMA\_1917 | tobe domain protein |  |
| pmx:PERMA\_1918 | modA; molybdate ABC transporter substrate-binding protein; K02020 molybdate transport system substrate-binding protein |  |
| pmx:PERMA\_1919 | molybdenum-pterin-binding protein; K02019 molybdate transport system regulatory protein |  |
| pmx:PERMA\_1920 | myo-inositol-1-phosphate synthase; K01858 myo-inositol-1-phosphate synthase [EC:5.5.1.4] | ec:5.5.1.4 |
| pmx:PERMA\_1921 | glucose-1-phosphate thymidylyltransferase; K07281 1L-myo-inositol 1-phosphate cytidylyltransferase [EC:2.7.7.74] K07291 CDP-L-myo-inositol myo-inositolphosphotransferase [EC:2.7.8.34] | ec:2.7.8.34 ec:2.7.7.74 |
| pmx:PERMA\_1922 | lipoprotein |  |
| pmx:PERMA\_1923 | smc; chromosome segregation protein SMC; K03529 chromosome segregation protein |  |
| pmx:PERMA\_1924 | long-chain-fatty-acid--CoA ligase (EC:6.2.1.26); K01897 long-chain acyl-CoA synthetase [EC:6.2.1.3] | ec:6.2.1.3 |
| pmx:PERMA\_1925 | membrane protein; K06076 long-chain fatty acid transport protein |  |
| pmx:PERMA\_1926 | hisC\_2; histidinol-phosphate transaminase (EC:2.6.1.9); K00817 histidinol-phosphate aminotransferase [EC:2.6.1.9] | ec:2.6.1.9 |
| pmx:PERMA\_1927 | integral membrane protein |  |
| pmx:PERMA\_1928 | hypothetical protein; K06888 |  |
| pmx:PERMA\_1929 | pgsA\_2; CDP-diacylglycerol--glycerol-3-phosphate 3-phosphatidyltransferase (EC:2.7.8.5); K00995 CDP-diacylglycerol--glycerol-3-phosphate 3-phosphatidyltransferase [EC:2.7.8.5] | ec:2.7.8.5 |

  
**Neighborhood Representations for "rcp:RCAP\_rcc00561"**  

| ID | Annotation | EC number |
| --- | --- | --- |
| rcp:RCAP\_rcc00551 | murI; glutamate racemase (EC:5.1.1.3); K01776 glutamate racemase [EC:5.1.1.3] | ec:5.1.1.3 |
| rcp:RCAP\_rcc00552 | hypothetical protein |  |
| rcp:RCAP\_rcc00553 | argC; N-acetyl-gamma-glutamyl-phosphate reductase (EC:1.2.1.38); K00145 N-acetyl-gamma-glutamyl-phosphate reductase [EC:1.2.1.38] | ec:1.2.1.38 |
| rcp:RCAP\_rcc00554 | ccmE; cytochrome c-type biogenesis protein CcmE; K02197 cytochrome c-type biogenesis protein CcmE |  |
| rcp:RCAP\_rcc00555 | hypothetical protein |  |
| rcp:RCAP\_rcc00556 | hypothetical protein |  |
| rcp:RCAP\_rcc00557 | metG; methionyl-tRNA synthetase (EC:6.1.1.10); K01874 methionyl-tRNA synthetase [EC:6.1.1.10] | ec:6.1.1.10 |
| rcp:RCAP\_rcc00558 | diguanylate cyclase/phosphodiesterase (EC:3.1.4.-) |  |
| rcp:RCAP\_rcc00559 | pmtA; phosphatidylethanolamine N-methyltransferase (EC:2.1.1.17); K00570 phosphatidylethanolamine/phosphatidyl-N-methylethanolamine N-methyltransferase [EC:2.1.1.17 2.1.1.71] | ec:2.1.1.17 ec:2.1.1.71 |
| rcp:RCAP\_rcc00560 | mopB; molybdenum transport operon repressor MopB; K02019 molybdate transport system regulatory protein |  |
| rcp:RCAP\_rcc00561 | mopA; molybdenum transport operon repressor MopA; K02019 molybdate transport system regulatory protein |  |
| rcp:RCAP\_rcc00562 | modA1; molybdenum ABC transporter periplasmic molybdenum-binding protein ModA; K02020 molybdate transport system substrate-binding protein |  |
| rcp:RCAP\_rcc00563 | modB1; molybdenum ABC transporter permease ModB; K02018 molybdate transport system permease protein |  |
| rcp:RCAP\_rcc00564 | modC1; molybdenum ABC transporter ATP-binding protein ModC (EC:3.6.3.29); K02017 molybdate transport system ATP-binding protein [EC:3.6.3.29] | ec:3.6.3.29 |
| rcp:RCAP\_rcc00565 | modD; molybdenum utilization protein ModD (EC:2.4.2.-); K03813 molybdenum transport protein [EC:2.4.2.-] |  |
| rcp:RCAP\_rcc00566 | nifB1; nitrogenase cofactor biosynthesis protein NifB; K02585 nitrogen fixation protein NifB |  |
| rcp:RCAP\_rcc00567 | nifA1; Nif-specific regulatory protein; K02584 Nif-specific regulatory protein |  |
| rcp:RCAP\_rcc00568 | rpoN; RNA polymerase sigma-54 factor; K03092 RNA polymerase sigma-54 factor |  |
| rcp:RCAP\_rcc00569 | nifU1; nitrogen fixation protein NifU |  |
| rcp:RCAP\_rcc00570 | nifK; nitrogenase molybdenum-iron protein subunit beta (EC:1.18.6.1); K02591 nitrogenase molybdenum-iron protein beta chain [EC:1.18.6.1] | ec:1.18.6.1 |
| rcp:RCAP\_rcc00571 | nifD; nitrogenase molybdenum-iron protein subunit alpha (EC:1.18.6.1); K02586 nitrogenase molybdenum-iron protein alpha chain [EC:1.18.6.1] | ec:1.18.6.1 |

  
**Neighborhood Representations for "cco:CCC13826\_2203"**  

| ID | Annotation | EC number |
| --- | --- | --- |
| cco:CCC13826\_2193 | hypothetical protein |  |
| cco:CCC13826\_2194 | leuC; 3-isopropylmalate dehydratase large subunit (EC:4.2.1.33); K01703 3-isopropylmalate/(R)-2-methylmalate dehydratase large subunit [EC:4.2.1.33 4.2.1.35] | ec:4.2.1.33 ec:4.2.1.35 |
| cco:CCC13826\_2195 | hypothetical protein |  |
| cco:CCC13826\_2196 | methyl-accepting chemotaxis protein |  |
| cco:CCC13826\_2197 | Hsp12 variant C |  |
| cco:CCC13826\_2198 | hypothetical protein |  |
| cco:CCC13826\_2199 | transport-associated; K04065 hyperosmotically inducible periplasmic protein |  |
| cco:CCC13826\_2200 | hypothetical protein; K07003 |  |
| cco:CCC13826\_2201 | ttg2D; toluene tolerance protein Ttg2D; K07323 putative toluene tolerance protein |  |
| cco:CCC13826\_2202 | ABC transporter ATPase; K04754 lipoprotein |  |
| cco:CCC13826\_2203 | molybdenum-pterin binding domain-containing protein; K02019 molybdate transport system regulatory protein |  |
| cco:CCC13826\_2204 | modA; molybdate ABC transporter periplasmic molybdate-binding protein; K02020 molybdate transport system substrate-binding protein |  |
| cco:CCC13826\_2205 | beta-lactamase HcpA (EC:3.5.2.6) |  |
| cco:CCC13826\_2281 | RNA pseudouridine synthase (EC:5.4.99.-); K02017 molybdate transport system ATP-binding protein [EC:3.6.3.29] | ec:3.6.3.29 |
| cco:CCC13826\_2282 | modB; molybdate ABC transporter permease; K02018 molybdate transport system permease protein |  |
| cco:CCC13826\_2283 | BcpB |  |
| cco:CCC13826\_2284 | dimethyladenosine transferase (EC:2.1.1.-) |  |
| cco:CCC13826\_2285 | DNA helicase related protein |  |
| cco:CCC13826\_2286 | protein CysQ; K01082 3'(2'), 5'-bisphosphate nucleotidase [EC:3.1.3.7] | ec:3.1.3.7 |
| cco:CCC13826\_2287 | gltA; citrate (Si)-synthase (EC:2.3.3.1); K01647 citrate synthase [EC:2.3.3.1] | ec:2.3.3.1 |
| cco:CCC13826\_2288 | radical SAM protein |  |

  
**Neighborhood Representations for "pap:PSPA7\_0590"**  

| ID | Annotation | EC number |
| --- | --- | --- |
| pap:PSPA7\_0580 | hypothetical protein |  |
| pap:PSPA7\_0581 | putative transcriptional regulator |  |
| pap:PSPA7\_0582 | putative permease; K03457 nucleobase:cation symporter-1, NCS1 family |  |
| pap:PSPA7\_0583 | putative transcriptional regulator |  |
| pap:PSPA7\_0584 | putative N-acetyltransferase |  |
| pap:PSPA7\_0585 | glcB; malate synthase G (EC:2.3.3.9); K01638 malate synthase [EC:2.3.3.9] | ec:2.3.3.9 |
| pap:PSPA7\_0586 | putative acetyltransferase |  |
| pap:PSPA7\_0587 | hypothetical protein |  |
| pap:PSPA7\_0588 | hypothetical protein; K05786 chloramphenicol-sensitive protein RarD |  |
| pap:PSPA7\_0589 | serine/threonine protein kinase |  |
| pap:PSPA7\_0590 | molybdenum transport regulator; K02019 molybdate transport system regulatory protein |  |
| pap:PSPA7\_0591 | hypothetical protein; K05811 putative lipoprotein |  |
| pap:PSPA7\_0592 | putative phosphoribosyl transferase |  |
| pap:PSPA7\_0593 | hypothetical protein |  |
| pap:PSPA7\_0594 | putative transcriptional regulator |  |
| pap:PSPA7\_0595 | hypothetical protein; K07160 UPF0271 protein |  |
| pap:PSPA7\_0596 | hypothetical protein |  |
| pap:PSPA7\_0597 | acetyl-CoA carboxylase biotin carboxylase subunit; K01961 acetyl-CoA carboxylase, biotin carboxylase subunit [EC:6.4.1.2 6.3.4.14] | ec:6.3.4.14 ec:6.4.1.2 |
| pap:PSPA7\_0598 | hypothetical protein |  |
| pap:PSPA7\_0599 | biotin-dependent carboxylase domain-containing protein |  |
| pap:PSPA7\_0600 | bioB; biotin synthase (EC:2.8.1.6); K01012 biotin synthase [EC:2.8.1.6] | ec:2.8.1.6 |

  
**Neighborhood Representations for "rpd:RPD\_0962"**  

| ID | Annotation | EC number |
| --- | --- | --- |
| rpd:RPD\_0952 | glycogen/starch/alpha-glucan phosphorylase (EC:2.4.1.1); K00688 starch phosphorylase [EC:2.4.1.1] | ec:2.4.1.1 |
| rpd:RPD\_0953 | hypothetical protein |  |
| rpd:RPD\_0954 | hypothetical protein |  |
| rpd:RPD\_0955 | pyrE; orotate phosphoribosyltransferase (EC:2.4.2.10); K00762 orotate phosphoribosyltransferase [EC:2.4.2.10] | ec:2.4.2.10 |
| rpd:RPD\_0956 | DNA polymerase I (EC:2.7.7.7); K02335 DNA polymerase I [EC:2.7.7.7] | ec:2.7.7.7 |
| rpd:RPD\_0957 | hypothetical protein |  |
| rpd:RPD\_0958 | putative bifunctional glutamate synthase subunit beta/2-polyprenylphenol hydroxylase; K00266 glutamate synthase (NADPH/NADH) small chain [EC:1.4.1.13 1.4.1.14] | ec:1.4.1.14 ec:1.4.1.13 |
| rpd:RPD\_0959 | pyruvate flavodoxin/ferredoxin oxidoreductase-like; K03737 putative pyruvate-flavodoxin oxidoreductase [EC:1.2.7.-] |  |
| rpd:RPD\_0960 | hypothetical protein |  |
| rpd:RPD\_0961 | hypothetical protein |  |
| rpd:RPD\_0962 | molybdate transport repressor; K02019 molybdate transport system regulatory protein |  |
| rpd:RPD\_0963 | molybdenum ABC transporter periplasmic molybdate-binding protein; K02020 molybdate transport system substrate-binding protein |  |
| rpd:RPD\_0964 | molybdate ABC transporter permease; K02018 molybdate transport system permease protein |  |
| rpd:RPD\_0965 | molybdate ABC transporter ATP-binding protein; K02017 molybdate transport system ATP-binding protein [EC:3.6.3.29] | ec:3.6.3.29 |
| rpd:RPD\_0966 | hypothetical protein |  |
| rpd:RPD\_0967 | hypothetical protein |  |
| rpd:RPD\_0968 | TPR repeat-containing protein |  |
| rpd:RPD\_0969 | cytochrome P450-like protein |  |
| rpd:RPD\_0970 | hypothetical protein |  |
| rpd:RPD\_0971 | sigma-70 region 2; K03088 RNA polymerase sigma-70 factor, ECF subfamily |  |
| rpd:RPD\_0972 | hypothetical protein |  |

  
**Neighborhood Representations for "ppg:PputGB1\_0390"**  

| ID | Annotation | EC number |
| --- | --- | --- |
| ppg:PputGB1\_0380 | RNA polymerase sigma factor; K03088 RNA polymerase sigma-70 factor, ECF subfamily |  |
| ppg:PputGB1\_0381 | DNA polymerase III subunit epsilon; K02342 DNA polymerase III subunit epsilon [EC:2.7.7.7] | ec:2.7.7.7 |
| ppg:PputGB1\_0382 | hypothetical protein; K07182 CBS domain-containing protein |  |
| ppg:PputGB1\_0383 | response regulator receiver protein |  |
| ppg:PputGB1\_0384 | malate synthase G (EC:2.3.3.9); K01638 malate synthase [EC:2.3.3.9] | ec:2.3.3.9 |
| ppg:PputGB1\_0385 | amino acid-binding ACT domain-containing protein |  |
| ppg:PputGB1\_0386 | transporter DMT superfamily protein; K05786 chloramphenicol-sensitive protein RarD |  |
| ppg:PputGB1\_0387 | hypothetical protein |  |
| ppg:PputGB1\_0388 | hypothetical protein |  |
| ppg:PputGB1\_0389 | serine/threonine protein kinase |  |
| ppg:PputGB1\_0390 | ModE family transcriptional regulator; K02019 molybdate transport system regulatory protein |  |
| ppg:PputGB1\_0391 | phosphoribosyltransferase |  |
| ppg:PputGB1\_0392 | biotin synthase (EC:2.8.1.6); K01012 biotin synthase [EC:2.8.1.6] | ec:2.8.1.6 |
| ppg:PputGB1\_0393 | 8-amino-7-oxononanoate synthase (EC:2.3.1.47); K00652 8-amino-7-oxononanoate synthase [EC:2.3.1.47] | ec:2.3.1.47 |
| ppg:PputGB1\_0394 | carboxylesterase (EC:3.1.1.1); K02170 pimeloyl-[acyl-carrier protein] methyl ester esterase [EC:3.1.1.85] | ec:3.1.1.85 |
| ppg:PputGB1\_0395 | biotin biosynthesis protein BioC; K02169 malonyl-CoA O-methyltransferase [EC:2.1.1.197] | ec:2.1.1.197 |
| ppg:PputGB1\_0396 | bioD; dithiobiotin synthetase (EC:6.3.3.3); K01935 dethiobiotin synthetase [EC:6.3.3.3] | ec:6.3.3.3 |
| ppg:PputGB1\_0397 | hypothetical protein |  |
| ppg:PputGB1\_0398 | acyl-CoA dehydrogenase domain-containing protein; K00257 [EC:1.3.99.-] |  |
| ppg:PputGB1\_0399 | diguanylate cyclase |  |
| ppg:PputGB1\_0400 | acyl-CoA dehydrogenase domain-containing protein; K00257 [EC:1.3.99.-] |  |

  
**Neighborhood Representations for "ppw:PputW619\_4842"**  

| ID | Annotation | EC number |
| --- | --- | --- |
| ppw:PputW619\_4832 | acyl-CoA dehydrogenase domain-containing protein; K00257 [EC:1.3.99.-] |  |
| ppw:PputW619\_4833 | diguanylate cyclase |  |
| ppw:PputW619\_4834 | acyl-CoA dehydrogenase domain-containing protein; K00257 [EC:1.3.99.-] |  |
| ppw:PputW619\_4835 | hypothetical protein |  |
| ppw:PputW619\_4836 | bioD; dithiobiotin synthetase (EC:6.3.3.3); K01935 dethiobiotin synthetase [EC:6.3.3.3] | ec:6.3.3.3 |
| ppw:PputW619\_4837 | biotin biosynthesis protein BioC; K02169 malonyl-CoA O-methyltransferase [EC:2.1.1.197] | ec:2.1.1.197 |
| ppw:PputW619\_4838 | carboxylesterase (EC:3.1.1.1); K02170 pimeloyl-[acyl-carrier protein] methyl ester esterase [EC:3.1.1.85] | ec:3.1.1.85 |
| ppw:PputW619\_4839 | 8-amino-7-oxononanoate synthase (EC:2.3.1.47); K00652 8-amino-7-oxononanoate synthase [EC:2.3.1.47] | ec:2.3.1.47 |
| ppw:PputW619\_4840 | biotin synthase (EC:2.8.1.6); K01012 biotin synthase [EC:2.8.1.6] | ec:2.8.1.6 |
| ppw:PputW619\_4841 | competence protein ComF |  |
| ppw:PputW619\_4842 | ModE family transcriptional regulator; K02019 molybdate transport system regulatory protein |  |
| ppw:PputW619\_4843 | serine/threonine protein kinase |  |
| ppw:PputW619\_4844 | transporter DMT superfamily protein; K05786 chloramphenicol-sensitive protein RarD |  |
| ppw:PputW619\_4845 | amino acid-binding ACT domain-containing protein |  |
| ppw:PputW619\_4846 | malate synthase G (EC:2.3.3.9); K01638 malate synthase [EC:2.3.3.9] | ec:2.3.3.9 |
| ppw:PputW619\_4847 | response regulator receiver protein |  |
| ppw:PputW619\_4848 | hypothetical protein; K07182 CBS domain-containing protein |  |
| ppw:PputW619\_4849 | exonuclease RNase T and DNA polymerase III; K02342 DNA polymerase III subunit epsilon [EC:2.7.7.7] | ec:2.7.7.7 |
| ppw:PputW619\_4850 | RNA polymerase sigma factor; K03088 RNA polymerase sigma-70 factor, ECF subfamily |  |
| ppw:PputW619\_4851 | anti-FecI sigma factor FecR |  |
| ppw:PputW619\_4852 | TonB-dependent siderophore receptor; K02014 iron complex outermembrane recepter protein |  |

  
**Over-represented Enzyme Summary**: Table of E.C. identified protein in the "Neighborhood Representation" ranked by frequency of occurrence  

| EC number | Frequency | Annotation | Reactions |
| --- | --- | --- | --- |
| ec:3.6.3.29 | 66 | molybdate-transporting ATPase | ATP + H2O + molybdateout = ADP + phosphate + molybdatein [RN:R00086] |
| ec:2.5.1.3 | 19 | thiamine-phosphate diphosphorylase; thiamine phosphate pyrophosphorylase; thiamine monophosphate pyrophosphorylase; TMP-PPase | 2-methyl-4-amino-5-hydroxymethylpyrimidine diphosphate + 4-methyl-5-(2-phosphono-oxyethyl)thiazole = diphosphate + thiamine phosphate [RN:R03223] |
| ec:1.18.6.1 | 16 | nitrogenase | 8 reduced ferredoxin + 8 H+ + N2 + 16 ATP + 16 H2O = 8 oxidized ferredoxin + H2 + 2 NH3 + 16 ADP + 16 phosphate [RN:R05185] |
| ec:2.7.1.39 | 14 | homoserine kinase; homoserine kinase (phosphorylating); HSK | ATP + L-homoserine = ADP + O-phospho-L-homoserine [RN:R01771] |
| ec:5.3.2.6 | 11 | 2-hydroxymuconate tautomerase; 4-oxalocrotonate tautomerase (misleading); 4-oxalocrotonate isomerase (misleading); cnbG (gene name); praC (gene name); xylH (gene name) | (2Z,4E)-2-hydroxyhexa-2,4-dienedioate = (3E)-2-oxohex-3-enedioate |
| ec:1.2.1.2 | 10 | formate dehydrogenase; formate-NAD+ oxidoreductase; FDH I; FDH II; N-FDH; formic hydrogen-lyase; formate hydrogenlyase; hydrogenlyase; NAD+-linked formate dehydrogenase; NAD+-dependent formate dehydrogenase; formate dehydrogenase (NAD+); NAD+-formate dehydrogenase; formate benzyl-viologen oxidoreductase; formic acid dehydrogenase | formate + NAD+ = CO2 + NADH [RN:R00519] |
| ec:2.7.7.7 | 7 | DNA-directed DNA polymerase; DNA polymerase I; DNA polymerase II; DNA polymerase III; DNA polymerase alpha; DNA polymerase beta; DNA polymerase gamma; DNA nucleotidyltransferase (DNA-directed); DNA nucleotidyltransferase (DNA-directed); deoxyribonucleate nucleotidyltransferase; deoxynucleate polymerase; deoxyribonucleic acid duplicase; deoxyribonucleic acid polymerase; deoxyribonucleic duplicase; deoxyribonucleic polymerase; deoxyribonucleic polymerase I; DNA duplicase; DNA nucleotidyltransferase; DNA polymerase; DNA replicase; DNA-dependent DNA polymerase; duplicase; Klenow fragment; sequenase; Taq DNA polymerase; Taq Pol I; Tca DNA polymerase | deoxynucleoside triphosphate + DNAn = diphosphate + DNAn+1 [RN:R00379] |
| ec:2.3.3.9 | 7 | malate synthase; L-malate glyoxylate-lyase (CoA-acetylating); glyoxylate transacetylase; glyoxylate transacetase; glyoxylic transacetase; malate condensing enzyme; malate synthetase; malic synthetase; malic-condensing enzyme | acetyl-CoA + H2O + glyoxylate = (S)-malate + CoA [RN:R00472] |
| ec:2.6.99.2 | 6 | pyridoxine 5'-phosphate synthase; pyridoxine 5-phosphate phospho lyase; PNP synthase; PdxJ | 1-deoxy-D-xylulose 5-phosphate + 3-amino-2-oxopropyl phosphate = pyridoxine 5'-phosphate + phosphate + 2 H2O [RN:R05838] |
| ec:4.1.99.3 | 6 | deoxyribodipyrimidine photo-lyase; photoreactivating enzyme; DNA photolyase; DNA-photoreactivating enzyme; DNA cyclobutane dipyrimidine photolyase; DNA photolyase; deoxyribonucleic photolyase; deoxyribodipyrimidine photolyase; photolyase; PRE; PhrB photolyase; deoxyribonucleic cyclobutane dipyrimidine photolyase; phr A photolyase; dipyrimidine photolyase (photosensitive); deoxyribonucleate pyrimidine dimer lyase (photosensitive) | cyclobutadipyrimidine (in DNA) = 2 pyrimidine residues (in DNA) [RN:R00034] |
| ec:2.5.1.18 | 6 | glutathione transferase; glutathione S-transferase; glutathione S-alkyltransferase; glutathione S-aryltransferase; S-(hydroxyalkyl)glutathione lyase; glutathione S-aralkyltransferase; glutathione S-alkyl transferase; GST | RX + glutathione = HX + R-S-glutathione [RN:R03522 R08511 R08512] |
| ec:3.2.1.52 | 6 | beta-N-acetylhexosaminidase; hexosaminidase; beta-acetylaminodeoxyhexosidase; N-acetyl-beta-D-hexosaminidase; N-acetyl-beta-hexosaminidase; beta-hexosaminidase; beta-acetylhexosaminidinase; beta-D-N-acetylhexosaminidase; beta-N-acetyl-D-hexosaminidase; beta-N-acetylglucosaminidase; hexosaminidase A; N-acetylhexosaminidase; beta-D-hexosaminidase | Hydrolysis of terminal non-reducing N-acetyl-D-hexosamine residues in N-acetyl-beta-D-hexosaminides |
| ec:2.7.8.7 | 6 | holo-[acyl-carrier-protein] synthase; acyl carrier protein holoprotein (holo-ACP) synthetase; holo-ACP synthetase; coenzyme A:fatty acid synthetase apoenzyme 4'-phosphopantetheine transferase; holosynthase; acyl carrier protein synthetase; holo-ACP synthase; PPTase; AcpS; ACPS; acyl carrier protein synthase; P-pant transferase; CoA:apo-[acyl-carrier-protein] pantetheinephosphotransferase; CoA-[4'-phosphopantetheine]:apo-[acyl-carrier-protein] 4'-pantetheinephosphotransferase | CoA-[4'-phosphopantetheine] + apo-[acyl-carrier protein] = adenosine 3',5'-bisphosphate + holo-[acyl-carrier protein] [RN:R01625] |
| ec:2.7.13.3 | 6 | histidine kinase; EnvZ; histidine kinase (ambiguous); histidine protein kinase (ambiguous); protein histidine kinase (ambiguous); protein kinase (histidine) (ambiguous); HK1; HP165; Sln1p | ATP + protein L-histidine = ADP + protein N-phospho-L-histidine |
| ec:1.2.7.3 | 5 | 2-oxoglutarate synthase; 2-ketoglutarate ferredoxin oxidoreductase; 2-oxoglutarate:ferredoxin oxidoreductase; KGOR; 2-oxoglutarate ferredoxin oxidoreductase; 2-oxoglutarate:ferredoxin 2-oxidoreductase (CoA-succinylating) | 2-oxoglutarate + CoA + 2 oxidized ferredoxin = succinyl-CoA + CO2 + 2 reduced ferredoxin + 2 H+ [RN:R01197] |
| ec:2.8.1.6 | 5 | biotin synthase; dethiobiotin:sulfur sulfurtransferase | dethiobiotin + sulfur-(sulfur carrier) + 2 S-adenosyl-L-methionine = biotin + (sulfur carrier) + 2 L-methionine + 2 5'-deoxyadenosine [RN:R01078] |
| ec:3.1.3.16 | 4 | protein-serine/threonine phosphatase; phosphoprotein phosphatase (ambiguous); protein phosphatase-1; protein phosphatase-2A; protein phosphatase-2B; protein phosphatase-2C; protein D phosphatase; phosphospectrin phosphatase; casein phosphatase; Aspergillus awamori acid protein phosphatase; calcineurin; phosphatase 2A; phosphatase 2B; phosphatase II; phosphatase IB; phosphatase C-II; polycation modulated (PCM-) phosphatase; phosphopyruvate dehydrogenase phosphatase; phosphatase SP; branched-chain alpha-keto acid dehydrogenase phosphatase; BCKDH phosphatase; 3-hydroxy 3-methylglutaryl coenzymeA reductase phosphatase; HMG-CoA reductase phosphatase; phosphatase H-II; phosphatase III; phosphatase I; protein phosphatase; phosphatase IV; phosphoprotein phosphohydrolase | [a protein]-serine/threonine phosphate + H2O = [a protein]-serine/threonine + phosphate [RN:R00164] |
| ec:1.15.1.1 | 4 | superoxide dismutase; superoxidase dismutase; copper-zinc superoxide dismutase; Cu-Zn superoxide dismutase; ferrisuperoxide dismutase; superoxide dismutase I; superoxide dismutase II; SOD; Cu,Zn-SOD; Mn-SOD; Fe-SOD; SODF; SODS; SOD-1; SOD-2; SOD-3; SOD-4; hemocuprein; erythrocuprein; cytocuprein; cuprein ; hepatocuprein | 2 O2.- + 2 H+ = O2 + H2O2 [RN:R00275] |
| ec:2.5.1.48 | 4 | cystathionine gamma-synthase; O-succinyl-L-homoserine succinate-lyase (adding cysteine); O-succinylhomoserine (thiol)-lyase; homoserine O-transsuccinylase; O-succinylhomoserine synthase; O-succinylhomoserine synthetase; cystathionine synthase; cystathionine synthetase; homoserine transsuccinylase; 4-O-succinyl-L-homoserine:L-cysteine S-(3-amino-3-carboxypropyl)transferase | O4-succinyl-L-homoserine + L-cysteine = L-cystathionine + succinate [RN:R03260] |
| ec:3.6.3.34 | 4 | iron-chelate-transporting ATPase | ATP + H2O + iron chelateout = ADP + phosphate + iron chelatein [RN:R00086] |
| ec:6.3.4.14 | 4 | biotin carboxylase; biotin carboxylase (component of acetyl CoA carboxylase) | ATP + biotin-carboxyl-carrier protein + CO2 = ADP + phosphate + carboxybiotin-carboxyl-carrier protein [RN:R04385] |
| ec:2.7.2.1 | 4 | acetate kinase; acetokinase; AckA; AK; acetic kinase; acetate kinase (phosphorylating) | ATP + acetate = ADP + acetyl phosphate [RN:R00315] |
| ec:1.4.1.14 | 4 | glutamate synthase (NADH); glutamate (reduced nicotinamide adenine dinucleotide) synthase; NADH: GOGAT; L-glutamate synthase (NADH); L-glutamate synthetase; NADH-glutamate synthase; NADH-dependent glutamate synthase; glutamate synthase (NADH) | 2 L-glutamate + NAD+ = L-glutamine + 2-oxoglutarate + NADH + H+ [RN:R00093] |
| ec:1.4.1.13 | 4 | glutamate synthase (NADPH); glutamate (reduced nicotinamide adenine dinucleotide phosphate) synthase; L-glutamate synthase; L-glutamate synthetase; glutamate synthetase (NADP); NADPH-dependent glutamate synthase; glutamine-ketoglutaric aminotransferase; NADPH-glutamate synthase; NADPH-linked glutamate synthase; glutamine amide-2-oxoglutarate aminotransferase (oxidoreductase, NADP); L-glutamine:2-oxoglutarate aminotransferase, NADPH oxidizing; GOGAT | 2 L-glutamate + NADP+ = L-glutamine + 2-oxoglutarate + NADPH + H+ (overall reaction) [RN:R00114]; (1a) L-glutamate + NH3 = L-glutamine + H2O [RN:R00256]; (1b) L-glutamate + NADP+ + H2O = NH3 + 2-oxoglutarate + NADPH + H+ [RN:R00248] |
| ec:4.1.1.9 | 4 | malonyl-CoA decarboxylase; malonyl coenzyme A decarboxylase; malonyl-CoA carboxy-lyase | malonyl-CoA = acetyl-CoA + CO2 [RN:R00233] |
| ec:3.1.3.48 | 4 | protein-tyrosine-phosphatase; phosphotyrosine phosphatase; phosphoprotein phosphatase (phosphotyrosine); phosphotyrosine histone phosphatase; protein phosphotyrosine phosphatase; tyrosylprotein phosphatase; phosphotyrosine protein phosphatase; phosphotyrosylprotein phosphatase; tyrosine O-phosphate phosphatase; PPT-phosphatase; PTPase; [phosphotyrosine]protein phosphatase; PTP-phosphatase | protein tyrosine phosphate + H2O = protein tyrosine + phosphate [RN:R02585] |
| ec:6.4.1.2 | 4 | acetyl-CoA carboxylase; acetyl coenzyme A carboxylase | ATP + acetyl-CoA + HCO3- = ADP + phosphate + malonyl-CoA [RN:R00742] |
| ec:2.4.1.1 | 3 | glycogen phosphorylase; muscle phosphorylase a and b; amylophosphorylase; polyphosphorylase; amylopectin phosphorylase; glucan phosphorylase; alpha-glucan phosphorylase; 1,4-alpha-glucan phosphorylase; glucosan phosphorylase; granulose phosphorylase; maltodextrin phosphorylase; muscle phosphorylase; myophosphorylase; potato phosphorylase; starch phosphorylase; 1,4-alpha-D-glucan:phosphate alpha-D-glucosyltransferase; phosphorylase (ambiguous) | [(1->4)-alpha-D-glucosyl]n + phosphate = [(1->4)-alpha-D-glucosyl]n-1 + alpha-D-glucose 1-phosphate [RN:R01821 R06050] |
| ec:3.1.1.85 | 3 | pimelyl-[acyl-carrier protein] methyl ester esterase; BioH | pimelyl-[acyl-carrier protein] methyl ester + H2O = pimelyl-[acyl-carrier protein] + methanol [RN:R09725] |
| ec:2.4.99.16 | 3 | starch synthase (maltosyl-transferring); alpha1,4-glucan:maltose-1-P maltosyltransferase; GMPMT | alpha-maltose 1-phosphate + [(1->4)-alpha-D-glucosyl]n = phosphate + [(1->4)-alpha-D-glucosyl]n+2 [RN:R09994] |
| ec:6.3.3.3 | 3 | dethiobiotin synthase; desthiobiotin synthase | ATP + 7,8-diaminononanoate + CO2 = ADP + phosphate + dethiobiotin [RN:R03182] |
| ec:2.3.1.8 | 3 | phosphate acetyltransferase; phosphotransacetylase; phosphoacylase; PTA | acetyl-CoA + phosphate = CoA + acetyl phosphate [RN:R00230] |
| ec:2.4.2.10 | 3 | orotate phosphoribosyltransferase; orotidylic acid phosphorylase; orotidine-5'-phosphate pyrophosphorylase; OPRTase; orotate phosphoribosyl pyrophosphate transferase; orotic acid phosphoribosyltransferase; orotidine 5'-monophosphate pyrophosphorylase; orotidine monophosphate pyrophosphorylase; orotidine phosphoribosyltransferase; orotidylate phosphoribosyltransferase; orotidylate pyrophosphorylase; orotidylic acid pyrophosphorylase; orotidylic phosphorylase; orotidylic pyrophosphorylase | orotidine 5'-phosphate + diphosphate = orotate + 5-phospho-alpha-D-ribose 1-diphosphate [RN:R01870] |
| ec:1.13.12.16 | 3 | nitronate monooxygenase; NMO; 2-nitropropane dioxygenase (incorrect) | ethylnitronate + O2 = acetaldehyde + nitrite + other products [RN:R00025] |
| ec:3.6.3.12 | 3 | K+-transporting ATPase; K+-translocating Kdp-ATPase; multi-subunit K+-transport ATPase | ATP + H2O + K+out = ADP + phosphate + K+in [RN:R00086] |
| ec:3.1.1.24 | 3 | 3-oxoadipate enol-lactonase; carboxymethylbutenolide lactonase; beta-ketoadipic enol-lactone hydrolase; 3-ketoadipate enol-lactonase; 3-oxoadipic enol-lactone hydrolase; beta-ketoadipate enol-lactone hydrolase | 3-oxoadipate enol-lactone + H2O = 3-oxoadipate [RN:R02991] |
| ec:1.9.3.1 | 3 | cytochrome-c oxidase; cytochrome oxidase; cytochrome a3; cytochrome aa3; Warburg's respiratory enzyme; indophenol oxidase; indophenolase; complex IV (mitochondrial electron transport); ferrocytochrome c oxidase; NADH cytochrome c oxidase | 4 ferrocytochrome c + O2 + 4 H+ = 4 ferricytochrome c + 2 H2O [RN:R00081] |
| ec:3.2.1.141 | 3 | 4-alpha-D-{(1->4)-alpha-D-glucano}trehalose trehalohydrolase; malto-oligosyltrehalose trehalohydrolase | hydrolysis of (1->4)-alpha-D-glucosidic linkage in 4-alpha-D-[(1->4)-alpha-D-glucanosyl]n trehalose to yield trehalose and (1->4)-alpha-D-glucan |
| ec:2.3.1.47 | 3 | 8-amino-7-oxononanoate synthase; 7-keto-8-aminopelargonic acid synthetase; 7-keto-8-aminopelargonic synthetase; 8-amino-7-oxopelargonate synthase; bioF (gene name) | pimeloyl-[acyl-carrier protein] + L-alanine = 8-amino-7-oxononanoate + CO2 + holo-[acyl-carrier protein] [RN:R03210] |
| ec:2.1.1.197 | 3 | malonyl-[acyl-carrier protein] O-methyltransferase; BioC | S-adenosyl-L-methionine + malonyl-[acyl-carrier protein] = S-adenosyl-L-homocysteine + malonyl-[acyl-carrier protein] methyl ester [RN:R09543] |
| ec:4.2.1.35 | 2 | (R)-2-methylmalate dehydratase; citraconate hydratase; citraconase; citramalate hydro-lyase; (-)-citramalate hydro-lyase; (R)-2-methylmalate hydro-lyase | (R)-2-methylmalate = 2-methylmaleate + H2O [RN:R03896] |
| ec:4.2.1.33 | 2 | 3-isopropylmalate dehydratase; (2R,3S)-3-isopropylmalate hydro-lyase; beta-isopropylmalate dehydratase; isopropylmalate isomerase; alpha-isopropylmalate isomerase; 3-isopropylmalate hydro-lyase | (2R,3S)-3-isopropylmalate = (2S)-2-isopropylmalate (overall reaction) [RN:R10170]; (1a) (2R,3S)-3-isopropylmalate = 2-isopropylmaleate + H2O [RN:R04001]; (1b) 2-isopropylmaleate + H2O = (2S)-2-isopropylmalate [RN:R03968] |
| ec:3.6.3.55 | 2 | tungstate-importing ATPase; tungstate transporter; WtpABC; TupABC; tungstate-specific ABC transporter | ATP + H2O + tungstate[side 1] = ADP + phosphate + tungstate[side 2] [RN:R10531] |
| ec:5.4.99.16 | 2 | maltose alpha-D-glucosyltransferase; trehalose synthase; maltose glucosylmutase | maltose = alpha,alpha-trehalose [RN:R01557 R06218] |
| ec:2.4.1.18 | 2 | 1,4-alpha-glucan branching enzyme; branching enzyme; amylo-(1,4->1,6)-transglycosylase; Q-enzyme; alpha-glucan-branching glycosyltransferase; amylose isomerase; enzymatic branching factor; branching glycosyltransferase; enzyme Q; glucosan transglycosylase; glycogen branching enzyme; plant branching enzyme; alpha-1,4-glucan:alpha-1,4-glucan-6-glycosyltransferase; starch branching enzyme; 1,4-alpha-D-glucan:1,4-alpha-D-glucan 6-alpha-D-(1,4-alpha-D-glucano)-transferase | Transfers a segment of a (1->4)-alpha-D-glucan chain to a primary hydroxy group in a similar glucan chain [RN:R02110 R06186] |
| ec:2.5.1.49 | 2 | O-acetylhomoserine aminocarboxypropyltransferase; O-acetyl-L-homoserine acetate-lyase (adding methanethiol); O-acetyl-L-homoserine sulfhydrolase; O-acetylhomoserine (thiol)-lyase; O-acetylhomoserine sulfhydrolase; methionine synthase (misleading) | O-acetyl-L-homoserine + methanethiol = L-methionine + acetate [RN:R00651] |
| ec:1.13.11.8 | 2 | protocatechuate 4,5-dioxygenase; protocatechuate 4,5-oxygenase; protocatechuic 4,5-dioxygenase; protocatechuic 4,5-oxygenase | protocatechuate + O2 = 4-carboxy-2-hydroxymuconate semialdehyde [RN:R01632] |
| ec:1.3.1.9 | 2 | enoyl-[acyl-carrier-protein] reductase (NADH); enoyl-[acyl carrier protein] reductase; enoyl-ACP reductase; NADH-enoyl acyl carrier protein reductase; NADH-specific enoyl-ACP reductase; acyl-[acyl-carrier-protein]:NAD+ oxidoreductase; fabI (gene name); inhA (gene name) | an acyl-[acyl-carrier protein] + NAD+ = a trans-2,3-dehydroacyl-[acyl-carrier protein] + NADH + H+ [RN:R01403] |
| ec:2.3.3.14 | 2 | homocitrate synthase; 2-hydroxybutane-1,2,4-tricarboxylate 2-oxoglutarate-lyase (CoA-acetylating); acetyl-coenzyme A:2-ketoglutarate C-acetyl transferase; homocitrate synthetase; HCS | acetyl-CoA + H2O + 2-oxoglutarate = (2R)-2-hydroxybutane-1,2,4-tricarboxylate + CoA [RN:R00271] |
| ec:3.6.3.17 | 2 | monosaccharide-transporting ATPase | ATP + H2O + monosaccharideout = ADP + phosphate + monosaccharidein [RN:R00086] |
| ec:5.4.99.2 | 2 | methylmalonyl-CoA mutase; methylmalonyl-CoA CoA-carbonyl mutase; methylmalonyl coenzyme A mutase; methylmalonyl coenzyme A carbonylmutase; (S)-methylmalonyl-CoA mutase; (R)-2-methyl-3-oxopropanoyl-CoA CoA-carbonylmutase [incorrect] | (R)-methylmalonyl-CoA = succinyl-CoA [RN:R00833] |
| ec:4.6.1.1 | 2 | adenylate cyclase; adenylylcyclase; adenyl cyclase; 3',5'-cyclic AMP synthetase; ATP diphosphate-lyase (cyclizing) | ATP = 3',5'-cyclic AMP + diphosphate [RN:R00089] |
| ec:2.6.1.9 | 2 | histidinol-phosphate transaminase; imidazolylacetolphosphate transaminase; glutamic-imidazoleacetol phosphate transaminase; histidinol phosphate aminotransferase; imidazoleacetol phosphate transaminase; L-histidinol phosphate aminotransferase; histidine:imidazoleacetol phosphate transaminase; IAP transaminase; imidazolylacetolphosphate aminotransferase | L-histidinol phosphate + 2-oxoglutarate = 3-(imidazol-4-yl)-2-oxopropyl phosphate + L-glutamate [RN:R03243] |
| ec:1.3.1.10 | 2 | enoyl-[acyl-carrier-protein] reductase (NADPH, Si-specific); acyl-ACP dehydrogenase (ambiguous); enoyl-[acyl carrier protein] (reduced nicotinamide adenine dinucleotide phosphate) reductase; NADPH 2-enoyl Co A reductase; enoyl acyl-carrier-protein reductase (ambiguous); enoyl-ACP reductase (ambiguous); acyl-[acyl-carrier-protein]:NADP+ oxidoreductase (B-specific); acyl-[acyl-carrier protein]:NADP+ oxidoreductase (B-specific); enoyl-[acyl-carrier-protein] reductase (NADPH, B-specific) | an acyl-[acyl-carrier protein] + NADP+ = a trans-2,3-dehydroacyl-[acyl-carrier protein] + NADPH + H+ [RN:R01404] |
| ec:3.2.1.1 | 2 | alpha-amylase; glycogenase; alpha amylase, alpha-amylase; endoamylase; Taka-amylase A; 1,4-alpha-D-glucan glucanohydrolase | Endohydrolysis of (1->4)-alpha-D-glucosidic linkages in polysaccharides containing three or more (1->4)-alpha-linked D-glucose units |
| ec:5.1.3.25 | 2 | dTDP-L-rhamnose 4-epimerase; dTDP-4-L-rhamnose 4-epimerase; wbiB (gene name) | dTDP-6-deoxy-beta-L-talose = dTDP-beta-L-rhamnose [RN:R10279] |
| ec:2.7.8.5 | 2 | CDP-diacylglycerol---glycerol-3-phosphate 3-phosphatidyltransferase; glycerophosphate phosphatidyltransferase; 3-phosphatidyl-1'-glycerol-3'-phosphate synthase; CDPdiacylglycerol:glycerol-3-phosphate phosphatidyltransferase; cytidine 5'-diphospho-1,2-diacyl-sn-glycerol (CDPdiglyceride):sn-glycerol-3-phosphate phosphatidyltransferase; phosphatidylglycerophosphate synthase; phosphatidylglycerolphosphate synthase; PGP synthase; CDPdiacylglycerol-sn-glycerol-3-phosphate 3-phosphatidyltransferase; CDPdiacylglycerol:sn-glycero-3-phosphate phosphatidyltransferase; glycerol phosphate phosphatidyltransferase; glycerol 3-phosphate phosphatidyltransferase; phosphatidylglycerol phosphate synthase; phosphatidylglycerol phosphate synthetase; phosphatidylglycerophosphate synthetase; sn-glycerol-3-phosphate phosphatidyltransferase | CDP-diacylglycerol + sn-glycerol 3-phosphate = CMP + 3(3-sn-phosphatidyl)-sn-glycerol 1-phosphate [RN:R01801] |
| ec:2.7.6.5 | 2 | GTP diphosphokinase; stringent factor; guanosine 3',5'-polyphosphate synthase; GTP pyrophosphokinase; ATP-GTP 3'-diphosphotransferase; guanosine 5',3'-polyphosphate synthetase; (p)ppGpp synthetase I; (p)ppGpp synthetase II; guanosine pentaphosphate synthetase; GPSI; GPSII | ATP + GTP = AMP + guanosine 3'-diphosphate 5'-triphosphate [RN:R00429] |
| ec:1.12.99.6 | 2 | hydrogenase (acceptor); H2 producing hydrogenase[ambiguous]; hydrogen-lyase[ambiguous]; hydrogenlyase[ambiguous]; uptake hydrogenase[ambiguous]; hydrogen:(acceptor) oxidoreductase | H2 + A = AH2 [RN:R07182] |
| ec:5.1.3.10 | 2 | CDP-paratose 2-epimerase; CDP-paratose epimerase; cytidine diphosphoabequose epimerase; cytidine diphosphodideoxyglucose epimerase; cytidine diphosphoparatose epimerase; cytidine diphosphate paratose-2-epimerase; CDP-abequose epimerase (incorrect); CDP-D-abequose 2-epimerase (incorrect); CDP-tyvelose 2-epimerase, | CDP-3,6-dideoxy-D-glucose = CDP-3,6-dideoxy-D-mannose [RN:R04266] |
| ec:3.5.1.2 | 2 | glutaminase; glutaminase I; L-glutaminase; glutamine aminohydrolase | L-glutamine + H2O = L-glutamate + NH3 [RN:R00256] |
| ec:3.11.1.2 | 2 | phosphonoacetate hydrolase | phosphonoacetate + H2O = acetate + phosphate [RN:R00318] |
| ec:1.8.1.4 | 2 | dihydrolipoyl dehydrogenase; LDP-Glc; LDP-Val; dehydrolipoate dehydrogenase; diaphorase; dihydrolipoamide dehydrogenase; dihydrolipoamide:NAD+ oxidoreductase; dihydrolipoic dehydrogenase; dihydrothioctic dehydrogenase; lipoamide dehydrogenase (NADH); lipoamide oxidoreductase (NADH); lipoamide reductase; lipoamide reductase (NADH); lipoate dehydrogenase; lipoic acid dehydrogenase; lipoyl dehydrogenase; protein-6-N-(dihydrolipoyl)lysine:NAD+ oxidoreductase | protein N6-(dihydrolipoyl)lysine + NAD+ = protein N6-(lipoyl)lysine + NADH + H+ [RN:R08550] |
| ec:1.1.1.27 | 2 | L-lactate dehydrogenase; lactic acid dehydrogenase; L(+)-nLDH; L-(+)-lactate dehydrogenase; L-lactic dehydrogenase; L-lactic acid dehydrogenase; lactate dehydrogenase; lactate dehydrogenase NAD+-dependent; lactic dehydrogenase; NAD+-lactate dehydrogenase | (S)-lactate + NAD+ = pyruvate + NADH + H+ [RN:R00703] |
| ec:1.17.4.1 | 2 | ribonucleoside-diphosphate reductase; ribonucleotide reductase; CDP reductase; ribonucleoside diphosphate reductase; UDP reductase; ADP reductase; nucleoside diphosphate reductase; ribonucleoside 5'-diphosphate reductase; ribonucleotide diphosphate reductase; 2'-deoxyribonucleoside-diphosphate:oxidized-thioredoxin 2'-oxidoreductase; RR | 2'-deoxyribonucleoside diphosphate + thioredoxin disulfide + H2O = ribonucleoside diphosphate + thioredoxin [RN:R04294] |
| ec:1.11.1.15 | 1 | peroxiredoxin; thioredoxin peroxidase; tryparedoxin peroxidase; alkyl hydroperoxide reductase C22; AhpC; TrxPx; TXNPx; Prx; PRDX | 2 R'-SH + ROOH = R'-S-S-R' + H2O + ROH [RN:R07180] |
| ec:6.3.1.10 | 1 | adenosylcobinamide-phosphate synthase; CbiB | (1) ATP + adenosylcobyric acid + (R)-1-aminopropan-2-yl phosphate = ADP + phosphate + adenosylcobinamide phosphate [RN:R06529]; (2) ATP + adenosylcobyric acid + (R)-1-aminopropan-2-ol = ADP + phosphate + adenosylcobinamide [RN:R07302] |
| ec:3.4.21.92 | 1 | endopeptidase Clp; endopeptidase Ti; caseinolytic protease; protease Ti; ATP-dependent Clp protease; endopeptidase Ti; caseinolytic protease; ClpP; Clp protease | Hydrolysis of proteins to small peptides in the presence of ATP and Mg2+. alpha-Casein is the usual test substrate. In the absence of ATP, only oligopeptides shorter than five residues are hydrolysed (such as succinyl-Leu-Tyr!NHMec; and Leu-Tyr-Leu!Tyr-Trp, in which cleavage of the -Tyr!Leu- and -Tyr!Trp bonds also occurs) |
| ec:3.1.26.5 | 1 | ribonuclease P; RNase P | Endonucleolytic cleavage of RNA, removing 5'-extranucleotides from tRNA precursor |
| ec:5.4.99.23 | 1 | 23S rRNA pseudouridine1911/1915/1917 synthase; RluD; pseudouridine synthase RluD | 23S rRNA uridine1911/uridine1915/uridine1917 = 23S rRNA pseudouridine1911/pseudouridine1915/pseudouridine1917 |
| ec:3.2.2.23 | 1 | DNA-formamidopyrimidine glycosylase; Fapy-DNA glycosylase; deoxyribonucleate glycosidase; 2,6-diamino-4-hydroxy-5N-formamidopyrimidine-DNA glycosylase; 2,6-diamino-4-hydroxy-5(N-methyl)formamidopyrimidine-DNA glycosylase; formamidopyrimidine-DNA glycosylase; DNA-formamidopyrimidine glycosidase; Fpg protein | Hydrolysis of DNA containing ring-opened 7-methylguanine residues, releasing 2,6-diamino-4-hydroxy-5-(N-methyl)formamidopyrimidine |
| ec:2.7.1.156 | 1 | adenosylcobinamide kinase; CobU; adenosylcobinamide kinase/adenosylcobinamide-phosphate guanylyltransferase; AdoCbi kinase/AdoCbi-phosphate guanylyltransferase | RTP + adenosylcobinamide = adenosylcobinamide phosphate + RDP [where RTP is either ATP or GTP (for symbol definitions, click here)] [RN:R05221 R06558] |
| ec:1.14.11.17 | 1 | taurine dioxygenase; 2-aminoethanesulfonate dioxygenase; alpha-ketoglutarate-dependent taurine dioxygenase | taurine + 2-oxoglutarate + O2 = sulfite + aminoacetaldehyde + succinate + CO2 [RN:R05320] |
| ec:1.2.1.38 | 1 | N-acetyl-gamma-glutamyl-phosphate reductase; reductase, acetyl-gamma-glutamyl phosphate; N-acetylglutamate 5-semialdehyde dehydrogenase; N-acetylglutamic gamma-semialdehyde dehydrogenase; N-acetyl-L-glutamate gamma-semialdehyde:NADP+ oxidoreductase (phosphorylating) | N-acetyl-L-glutamate 5-semialdehyde + NADP+ + phosphate = N-acetyl-L-glutamyl 5-phosphate + NADPH + H+ [RN:R03443] |
| ec:4.2.1.24 | 1 | porphobilinogen synthase; aminolevulinate dehydratase; delta-aminolevulinate dehydratase; delta-aminolevulinic acid dehydrase; delta-aminolevulinic acid dehydratase; aminolevulinic dehydratase; delta-aminolevulinic dehydratase; 5-levulinic acid dehydratase; 5-aminolevulinate hydro-lyase (adding 5-aminolevulinate and cyclizing); hemB (gene name) | 2 5-aminolevulinate = porphobilinogen + 2 H2O [RN:R00036] |
| ec:4.2.1.20 | 1 | tryptophan synthase; L-tryptophan synthetase; indoleglycerol phosphate aldolase; tryptophan desmolase; tryptophan synthetase; L-serine hydro-lyase (adding indoleglycerol-phosphate); L-serine hydro-lyase [adding 1-C-(indol-3-yl)glycerol 3-phosphate, L-tryptophan and glyceraldehyde-3-phosphate-forming] | L-serine + 1-C-(indol-3-yl)glycerol 3-phosphate = L-tryptophan + D-glyceraldehyde 3-phosphate + H2O (overall reaction) [RN:R02722]; (1a) 1-C-(indol-3-yl)glycerol 3-phosphate = indole + D-glyceraldehyde 3-phosphate [RN:R02340]; (1b) L-serine + indole = L-tryptophan + H2O [RN:R00674] |
| ec:2.7.7.3 | 1 | pantetheine-phosphate adenylyltransferase; dephospho-CoA pyrophosphorylase; pantetheine phosphate adenylyltransferase; dephospho-coenzyme A pyrophosphorylase; 3'-dephospho-CoA pyrophosphorylase | ATP + pantetheine 4'-phosphate = diphosphate + 3'-dephospho-CoA [RN:R03035] |
| ec:2.5.1.47 | 1 | cysteine synthase; O-acetyl-L-serine sulfhydrylase; O-acetyl-L-serine sulfohydrolase; O-acetylserine (thiol)-lyase; O-acetylserine (thiol)-lyase A; O-acetylserine sulfhydrylase; O3-acetyl-L-serine acetate-lyase (adding hydrogen-sulfide); acetylserine sulfhydrylase; cysteine synthetase; S-sulfocysteine synthase; 3-O-acetyl-L-serine:hydrogen-sulfide 2-amino-2-carboxyethyltransferase | O3-acetyl-L-serine + hydrogen sulfide = L-cysteine + acetate [RN:R00897] |
| ec:3.1.1.73 | 1 | feruloyl esterase; ferulic acid esterase, hydroxycinnamoyl esterase, hemicellulase accessory enzymes; FAE-III, cinnamoyl ester hydrolase, FAEA, cinnAE, FAE-I, FAE-II | feruloyl-polysaccharide + H2O = ferulate + polysaccharide [RN:R07292] |
| ec:1.4.3.19 | 1 | glycine oxidase | glycine + H2O + O2 = glyoxylate + NH3 + H2O2 (overall reaction) [RN:R00366]; (1a) glycine + O2 = 2-iminoacetate + H2O2 [RN:R07463]; (1b) 2-iminoacetate + H2O = glyoxylate + NH3 [RN:R10245] |
| ec:1.1.1.312 | 1 | 2-hydroxy-4-carboxymuconate semialdehyde hemiacetal dehydrogenase; 2-hydroxy-4-carboxymuconate 6-semialdehyde dehydrogenase; 4-carboxy-2-hydroxy-cis,cis-muconate-6-semialdehyde:NADP+ oxidoreductase; alpha-hydroxy-gamma-carboxymuconic epsilon-semialdehyde dehydrogenase; 4-carboxy-2-hydroxymuconate-6-semialdehyde dehydrogenase; LigC; ProD | 4-carboxy-2-hydroxymuconate semialdehyde hemiacetal + NADP+ = 2-oxo-2H-pyran-4,6-dicarboxylate + NADPH + H+ [RN:R04279] |
| ec:1.20.4.1 | 1 | arsenate reductase (glutaredoxin) | arsenate + glutaredoxin = arsenite + glutaredoxin disulfide + H2O [RN:R05747] |
| ec:4.3.1.7 | 1 | ethanolamine ammonia-lyase; ethanolamine deaminase | ethanolamine = acetaldehyde + NH3 [RN:R00749] |
| ec:5.99.1.3 | 1 | DNA topoisomerase (ATP-hydrolysing); type II DNA topoisomerase; DNA-gyrase; deoxyribonucleate topoisomerase; deoxyribonucleic topoisomerase; topoisomerase; DNA topoisomerase II | ATP-dependent breakage, passage and rejoining of double-stranded DNA |
| ec:2.7.8.34 | 1 | CDP-L-myo-inositol myo-inositolphosphotransferase; CDP-inositol:inositol-1-phosphate transferase (bifunctional CTP:inositol-1-phosphate cytidylyltransferase/CDP-inositol:inositol-1-phosphate transferase (IPCT/DIPPS)); DIPPS (bifunctional CTP:inositol-1-phosphate cytidylyltransferase/CDP-inositol:inositol-1-phosphate transferase (IPCT/DIPPS)) | CDP-1L-myo-inositol + 1L-myo-inositol 1-phosphate = CMP + bis(1L-myo-inositol) 3,1'-phosphate 1-phosphate [RN:R09670] |
| ec:3.6.3.27 | 1 | phosphate-transporting ATPase; ABC phosphate transporter | ATP + H2O + phosphateout = ADP + phosphate + phosphatein [RN:R00086] |
| ec:2.3.3.1 | 1 | citrate (Si)-synthase; (R)-citric synthase; citrate condensing enzyme; citrate oxaloacetate-lyase [(pro-3S)-CH2COO-->acetyl-CoA]; citrate oxaloacetate-lyase, CoA-acetylating; citrate synthase; citrate synthetase; citric synthase; citric-condensing enzyme; citrogenase; condensing enzyme; oxaloacetate transacetase; oxalacetic transacetase | acetyl-CoA + H2O + oxaloacetate = citrate + CoA [RN:R00351] |
| ec:3.1.4.16 | 1 | 2',3'-cyclic-nucleotide 2'-phosphodiesterase; ribonucleoside 2',3'-cyclic phosphate diesterase; 2',3 '-cyclic AMP phosphodiesterase; 2',3'-cyclic nucleotidase; cyclic 2',3'-nucleotide 2'-phosphodiesterase; cyclic 2',3'-nucleotide phosphodiesterase; 2',3'-cyclic nucleoside monophosphate phosphodiesterase; 2',3'-cyclic AMP 2'-phosphohydrolase; cyclic phosphodiesterase:3'-nucleotidase; 2',3'-cyclic nucleotide phosphohydrolase; 2':3'-cyclic phosphodiesterase; 2':3'-cyclic nucleotide phosphodiesterase:3'-nucleotidase | nucleoside 2',3'-cyclic phosphate + H2O = nucleoside 3'-phosphate [RN:R03423] |
| ec:4.1.2.4 | 1 | deoxyribose-phosphate aldolase; phosphodeoxyriboaldolase; deoxyriboaldolase; deoxyribose-5-phosphate aldolase; 2-deoxyribose-5-phosphate aldolase; 2-deoxy-D-ribose-5-phosphate acetaldehyde-lyase | 2-deoxy-D-ribose 5-phosphate = D-glyceraldehyde 3-phosphate + acetaldehyde [RN:R01066] |
| ec:3.1.1.57 | 1 | 2-pyrone-4,6-dicarboxylate lactonase; 2-pyrone-4,6-dicarboxylate hydrolase; 2-pyrone-4,6-dicarboxylate lactonohydrolase | 2-oxo-2H-pyran-4,6-dicarboxylate + H2O = (1E)-4-oxobut-1-ene-1,2,4-tricarboxylate [RN:R04277] |
| ec:3.5.4.16 | 1 | GTP cyclohydrolase I; GTP cyclohydrolase; guanosine triphosphate cyclohydrolase; guanosine triphosphate 8-deformylase; dihydroneopterin triphosphate synthase; GTP 8-formylhydrolase | GTP + H2O = formate + 7,8-dihydroneopterin 3'-triphosphate [RN:R00424] |
| ec:2.1.1.17 | 1 | phosphatidylethanolamine N-methyltransferase; PEMT; LMTase; lipid methyl transferase; phosphatidylethanolamine methyltransferase; phosphatidylethanolamine-N-methylase; phosphatidylethanolamine-S-adenosylmethionine methyltransferase | S-adenosyl-L-methionine + phosphatidylethanolamine = S-adenosyl-L-homocysteine + phosphatidyl-N-methylethanolamine [RN:R02056] |
| ec:3.6.3.8 | 1 | Ca2+-transporting ATPase; sarcoplasmic reticulum ATPase; sarco(endo)plasmic reticulum Ca2+-ATPase; calcium pump; Ca2+-pumping ATPase; plasma membrane Ca-ATPase | ATP + H2O + Ca2+[side 1] = ADP + phosphate + Ca2+[side 2] [RN:R00086] |
| ec:4.1.3.17 | 1 | 4-hydroxy-4-methyl-2-oxoglutarate aldolase; pyruvate aldolase; gamma-methyl-gamma-hydroxy-alpha-ketoglutaric aldolase; 4-hydroxy-4-methyl-2-ketoglutarate aldolase; 4-hydroxy-4-methyl-2-oxoglutarate pyruvate-lyase; HMG aldolase; CHA aldolase; 4-carboxy-4-hydroxy-2-oxoadipate aldolase | (1) 4-hydroxy-4-methyl-2-oxoglutarate = 2 pyruvate [RN:R00008]; (2) 2-hydroxy-4-oxobutane-1,2,4-tricarboxylate = oxaloacetate + pyruvate [RN:R00350] |
| ec:3.1.11.5 | 1 | exodeoxyribonuclease V; Escherichia coli exonuclease V; E. coli exonuclease V; gene recBC endoenzyme; RecBC deoxyribonuclease; gene recBC DNase; exonuclease V; gene recBCD enzymes | Exonucleolytic cleavage (in the presence of ATP) in either 5'- to 3'- or 3'- to 5'-direction to yield 5'-phosphooligonucleotides |
| ec:3.6.3.5 | 1 | Zn2+-exporting ATPase; Zn(II)-translocating P-type ATPase; P1B-type ATPase; AtHMA4 | ATP + H2O + Zn2+in = ADP + phosphate + Zn2+out [RN:R00086] |
| ec:3.6.3.3 | 1 | Cd2+-exporting ATPase | ATP + H2O + Cd2+in = ADP + phosphate + Cd2+out [RN:R00086] |
| ec:3.1.11.2 | 1 | exodeoxyribonuclease III; Escherichia coli exonuclease III; E. coli exonuclease III; endoribonuclease III | Exonucleolytic cleavage in the 3'- to 5'-direction to yield nucleoside 5'-phosphates |
| ec:2.1.1.10 | 1 | homocysteine S-methyltransferase; S-adenosylmethionine homocysteine transmethylase; S-methylmethionine homocysteine transmethylase; adenosylmethionine transmethylase; methylmethionine:homocysteine methyltransferase; adenosylmethionine:homocysteine methyltransferase; homocysteine methylase; homocysteine methyltransferase; homocysteine transmethylase; L-homocysteine S-methyltransferase; S-adenosyl-L-methionine:L-homocysteine methyltransferase; S-adenosylmethionine-homocysteine transmethylase; S-adenosylmethionine:homocysteine methyltransferase | S-methyl-L-methionine + L-homocysteine = 2 L-methionine [RN:R00650] |
| ec:2.5.1.17 | 1 | cob(I)yrinic acid a,c-diamide adenosyltransferase; CobA; CobO; ATP:corrinoid adenosyltransferase; cob(I)alamin adenosyltransferase; aquacob(I)alamin adenosyltransferase; aquocob(I)alamin vitamin B12s adenosyltransferase; ATP:cob(I)alamin Cobeta-adenosyltransferase | (1) ATP + cob(I)yrinic acid a,c-diamide = triphosphate + adenosylcob(III)yrinic acid a,c-diamide [RN:R05220]; (2) ATP + cobinamide = triphosphate + adenosylcobinamide [RN:R07268] |
| ec:2.5.1.16 | 1 | spermidine synthase; aminopropyltransferase; putrescine aminopropyltransferase; spermidine synthetase; SpeE; S-adenosylmethioninamine:putrescine 3-aminopropyltransferase | S-adenosyl 3-(methylthio)propylamine + putrescine = S-methyl-5'-thioadenosine + spermidine [RN:R01920] |
| ec:3.6.1.1 | 1 | inorganic diphosphatase | diphosphate + H2O = 2 phosphate [RN:R00004] |
| ec:6.1.1.24 | 1 | glutamate---tRNAGln ligase; nondiscriminating glutamyl-tRNA synthetase | ATP + L-glutamate + tRNAGlx = AMP + diphosphate + glutamyl-tRNAGlx [RN:R03651 R05578] |
| ec:1.1.1.85 | 1 | 3-isopropylmalate dehydrogenase; beta-isopropylmalic enzyme; beta-isopropylmalate dehydrogenase; threo-Ds-3-isopropylmalate dehydrogenase; 3-carboxy-2-hydroxy-4-methylpentanoate:NAD+ oxidoreductase | (2R,3S)-3-isopropylmalate + NAD+ = 4-methyl-2-oxopentanoate + CO2 + NADH + H+ (overall reaction) [RN:R10052]; (1a) (2R,3S)-3-isopropylmalate + NAD+ = (2S)-2-isopropyl-3-oxosuccinate + NADH + H+ [RN:R04426]; (1b) (2S)-2-isopropyl-3-oxosuccinate = 4-methyl-2-oxopentanoate + CO2 (spontaneous) [RN:R01652] |
| ec:2.7.1.71 | 1 | shikimate kinase; shikimate kinase (phosphorylating); shikimate kinase II | ATP + shikimate = ADP + 3-phosphoshikimate [RN:R02412] |
| ec:6.2.1.3 | 1 | long-chain-fatty-acid---CoA ligase; acyl-CoA synthetase; fatty acid thiokinase (long chain); acyl-activating enzyme; palmitoyl-CoA synthase; lignoceroyl-CoA synthase; arachidonyl-CoA synthetase; acyl coenzyme A synthetase; acyl-CoA ligase; palmitoyl coenzyme A synthetase; thiokinase; palmitoyl-CoA ligase; acyl-coenzyme A ligase; fatty acid CoA ligase; long-chain fatty acyl coenzyme A synthetase; oleoyl-CoA synthetase; stearoyl-CoA synthetase; long chain fatty acyl-CoA synthetase; long-chain acyl CoA synthetase; fatty acid elongase; LCFA synthetase; pristanoyl-CoA synthetase; ACS3; long-chain acyl-CoA synthetase I; long-chain acyl-CoA synthetase II; fatty acyl-coenzyme A synthetase; long-chain acyl-coenzyme A synthetase; FAA1 | ATP + a long-chain fatty acid + CoA = AMP + diphosphate + an acyl-CoA [RN:R00390] |
| ec:6.1.1.17 | 1 | glutamate---tRNA ligase; glutamyl-tRNA synthetase; glutamyl-transfer ribonucleate synthetase; glutamyl-transfer RNA synthetase; glutamyl-transfer ribonucleic acid synthetase; glutamate-tRNA synthetase; glutamic acid translase | ATP + L-glutamate + tRNAGlu = AMP + diphosphate + L-glutamyl-tRNAGlu [RN:R05578] |
| ec:6.1.1.12 | 1 | aspartate---tRNA ligase; aspartyl-tRNA synthetase; aspartyl ribonucleic synthetase; aspartyl-transfer RNA synthetase; aspartic acid translase; aspartyl-transfer ribonucleic acid synthetase; aspartyl ribonucleate synthetase | ATP + L-aspartate + tRNAAsp = AMP + diphosphate + L-aspartyl-tRNAAsp [RN:R05577] |
| ec:2.4.2.6 | 1 | nucleoside deoxyribosyltransferase; purine(pyrimidine) nucleoside:purine(pyrimidine) deoxyribosyl transferase; deoxyribose transferase; nucleoside trans-N-deoxyribosylase; trans-deoxyribosylase; trans-N-deoxyribosylase; trans-N-glycosidase; nucleoside deoxyribosyltransferase I (purine nucleoside:purine deoxyribosyltransferase: strictly specific for transfer between purine bases); nucleoside deoxyribosyltransferase II [purine(pyrimidine) nucleoside:purine(pyrimidine) deoxyribosyltransferase] | 2-deoxy-D-ribosyl-base1 + base2 = 2-deoxy-D-ribosyl-base2 + base1 [RN:R04168] |
| ec:4.2.3.4 | 1 | 3-dehydroquinate synthase; 5-dehydroquinate synthase; 5-dehydroquinic acid synthetase; dehydroquinate synthase; 3-dehydroquinate synthetase; 3-deoxy-arabino-heptulosonate-7-phosphate phosphate-lyase (cyclizing); 3-deoxy-arabino-heptulonate-7-phosphate phosphate-lyase (cyclizing); 3-deoxy-arabino-heptulonate-7-phosphate phosphate-lyase (cyclizing; 3-dehydroquinate-forming) | 3-deoxy-D-arabino-hept-2-ulosonate 7-phosphate = 3-dehydroquinate + phosphate [RN:R03083] |
| ec:6.1.1.11 | 1 | serine---tRNA ligase; seryl-tRNA synthetase; SerRS; seryl-transfer ribonucleate synthetase; seryl-transfer RNA synthetase; seryl-transfer ribonucleic acid synthetase; serine translase | ATP + L-serine + tRNASer = AMP + diphosphate + L-seryl-tRNASer [RN:R03662] |
| ec:6.1.1.10 | 1 | methionine---tRNA ligase; methionyl-tRNA synthetase; methionyl-transfer ribonucleic acid synthetase; methionyl-transfer ribonucleate synthetase; methionyl-transfer RNA synthetase; methionine translase; MetRS | ATP + L-methionine + tRNAMet = AMP + diphosphate + L-methionyl-tRNAMet [RN:R03659] |
| ec:4.2.1.9 | 1 | dihydroxy-acid dehydratase; acetohydroxyacid dehydratase; alpha,beta-dihydroxyacid dehydratase; 2,3-dihydroxyisovalerate dehydratase; alpha,beta-dihydroxyisovalerate dehydratase; dihydroxy acid dehydrase; DHAD; 2,3-dihydroxy-acid hydro-lyase | 2,3-dihydroxy-3-methylbutanoate = 3-methyl-2-oxobutanoate + H2O [RN:R01209] |
| ec:2.5.1.105 | 1 | 7,8-dihydropterin-6-yl-methyl-4-(beta-D-ribofuranosyl)aminobenzene 5'-phosphate synthase; MJ0301 (gene name); dihydropteroate synthase (ambiguous) | (7,8-dihydropterin-6-yl)methyl diphosphate + 4-(beta-D-ribofuranosyl)aniline 5'-phosphate = N-[(7,8-dihydropterin-6-yl)methyl]-4-(beta-D-ribofuranosyl)aniline 5'-phosphate + diphosphate [RN:R10339] |
| ec:1.7.1.13 | 1 | preQ1 synthase; YkvM; QueF; preQ0 reductase; preQ0 oxidoreductase; 7-cyano-7-deazaguanine reductase; queuine synthase (incorrect as queuine is not the product); queuine:NADP+ oxidoreductase (incorrect as queuine is not the product) | 7-aminomethyl-7-carbaguanine + 2 NADP+ = 7-cyano-7-carbaguanine + 2 NADPH + 2 H+ [RN:R07605] |
| ec:2.1.1.107 | 1 | uroporphyrinogen-III C-methyltransferase; uroporphyrinogen methyltransferase; uroporphyrinogen-III methyltransferase; adenosylmethionine-uroporphyrinogen III methyltransferase; S-adenosyl-L-methionine-dependent uroporphyrinogen III methylase; uroporphyrinogen-III methylase; SirA; CysG; CobA [ambiguous - see EC 2.5.1.17] SUMT; uroporphyrin-III C-methyltransferase (incorrect); S-adenosyl-L-methionine:uroporphyrin-III C-methyltransferase (incorrect) | 2 S-adenosyl-L-methionine + uroporphyrinogen III = 2 S-adenosyl-L-homocysteine + precorrin-2 (overall reaction) [RN:R03194]; (1a) S-adenosyl-L-methionine + uroporphyrinogen III = S-adenosyl-L-homocysteine + precorrin-1 [RN:R07237]; (1b) S-adenosyl-L-methionine + precorrin-1 = S-adenosyl-L-homocysteine + precorrin-2 [RN:R07238] |
| ec:2.7.4.16 | 1 | thiamine-phosphate kinase; thiamin-monophosphate kinase; thiamin monophosphatase; thiamin monophosphokinase | ATP + thiamine phosphate = ADP + thiamine diphosphate [RN:R00617] |
| ec:1.2.1.3 | 1 | aldehyde dehydrogenase (NAD+); CoA-independent aldehyde dehydrogenase; m-methylbenzaldehyde dehydrogenase; NAD-aldehyde dehydrogenase; NAD-dependent 4-hydroxynonenal dehydrogenase; NAD-dependent aldehyde dehydrogenase; NAD-linked aldehyde dehydrogenase; propionaldehyde dehydrogenase; aldehyde dehydrogenase (NAD) | an aldehyde + NAD+ + H2O = a carboxylate + NADH + H+ [RN:R00538] |
| ec:2.2.1.6 | 1 | acetolactate synthase; alpha-acetohydroxy acid synthetase; alpha-acetohydroxyacid synthase; alpha-acetolactate synthase; alpha-acetolactate synthetase; acetohydroxy acid synthetase; acetohydroxyacid synthase; acetolactate pyruvate-lyase (carboxylating); acetolactic synthetase | 2 pyruvate = 2-acetolactate + CO2 [RN:R00006] |
| ec:3.6.4.13 | 1 | RNA helicase; CSFV NS3 helicase; DBP2; DbpA; DDX17; DDX25; DDX3; DDX3X; DDX3Y; DDX4; DDX5; DEAD-box protein DED1; DEAD-box RNA helicase; DEAH-box protein 2; DEAH-box RNA helicase; DED1; Dex(H/D) RNA helicase; EhDEAD1; EhDEAD1 RNA helicase; eIF4A helicase; KOKV helicase; Mtr4p; nonstructural protein 3 helicase; NPH-II; RHA; RNA helicase A; RNA helicase DDX3; RNA helicase Hera; RNA-dependent ATPase; TGBp1 NTPase/helicase domain; VRH1; GRTH/DDX25 | ATP + H2O = ADP + phosphate [RN:R00086] |
| ec:3.4.16.4 | 1 | serine-type D-Ala-D-Ala carboxypeptidase; DD-peptidase; D-alanyl-D-alanine-carboxypeptidase; D-alanyl-D-alanine-cleaving-peptidase; D-alanyl-D-alanine-cleaving peptidase; DD-transpeptidase; D-alanine carboxypeptidase; DD-carboxypeptidase; D-alanyl carboxypeptidase | Preferential cleavage: (Ac)2-L-Lys-D-Ala!D-Ala. Also transpeptidation of peptidyl-alanyl moieties that are N-acyl substituents of D-alanine |
| ec:5.5.1.4 | 1 | inositol-3-phosphate synthase; myo-inositol-1-phosphate synthase; D-glucose 6-phosphate cycloaldolase; inositol 1-phosphate synthatase; glucose 6-phosphate cyclase; inositol 1-phosphate synthetase; glucose-6-phosphate inositol monophosphate cycloaldolase; glucocycloaldolase; 1L-myo-inositol-1-phosphate lyase (isomerizing) | D-glucose 6-phosphate = 1D-myo-inositol 3-phosphate [RN:R07324] |
| ec:6.2.1.30 | 1 | phenylacetate---CoA ligase; phenacyl coenzyme A synthetase; phenylacetyl-CoA ligase; PA-CoA ligase; phenylacetyl-CoA ligase (AMP-forming) | ATP + phenylacetate + CoA = AMP + diphosphate + phenylacetyl-CoA [RN:R02539] |
| ec:3.6.1.55 | 1 | 8-oxo-dGTP diphosphatase; MutT; 7,8-dihydro-8-oxoguanine triphosphatase; 8-oxo-dGTPase; 7,8-dihydro-8-oxo-dGTP pyrophosphohydrolase | 8-oxo-dGTP + H2O = 8-oxo-dGMP + diphosphate [RN:R09832] |
| ec:4.1.1.50 | 1 | adenosylmethionine decarboxylase; S-adenosylmethionine decarboxylase; S-adenosyl-L-methionine decarboxylase; S-adenosyl-L-methionine carboxy-lyase; S-adenosyl-L-methionine carboxy-lyase [(5-deoxy-5-adenosyl)(3-aminopropyl)methylsulfonium-salt-forming] | S-adenosyl-L-methionine = S-adenosyl 3-(methylthio)propylamine + CO2 [RN:R00178] |
| ec:1.2.99.5 | 1 | formylmethanofuran dehydrogenase; formylmethanofuran:(acceptor) oxidoreductase | formylmethanofuran + H2O + acceptor = CO2 + methanofuran + reduced acceptor [RN:R03015] |
| ec:4.2.1.83 | 1 | 4-oxalomesaconate hydratase; 4-oxalmesaconate hydratase; 4-carboxy-2-oxohexenedioate hydratase; 4-carboxy-2-oxobutane-1,2,4-tricarboxylate 2,3-hydro-lyase; oxalmesaconate hydratase; gamma-oxalmesaconate hydratase; 2-hydroxy-4-oxobutane-1,2,4-tricarboxylate 2,3-hydro-lyase; LigJ; GalB | 2-hydroxy-4-oxobutane-1,2,4-tricarboxylate = (1E,3E)-4-hydroxybuta-1,3-diene-1,2,4-tricarboxylate + H2O [RN:R04478] |
| ec:1.7.2.5 | 1 | nitric oxide reductase (cytochrome c) | nitrous oxide + 2 ferricytochrome c + H2O = 2 nitric oxide + 2 ferrocytochrome c + 2 H+ [RN:R00294] |
| ec:5.3.2.8 | 1 | 4-oxalomesaconate tautomerase; GalD | (1E)-4-oxobut-1-ene-1,2,4-tricarboxylate = (1E,3E)-4-hydroxybuta-1,3-diene-1,2,4-tricarboxylate [RN:R07839] |
| ec:3.5.1.1 | 1 | asparaginase; asparaginase II; L-asparaginase; colaspase; elspar; leunase; crasnitin; alpha-asparaginase | L-asparagine + H2O = L-aspartate + NH3 [RN:R00485] |
| ec:6.3.2.4 | 1 | D-alanine---D-alanine ligase; MurE synthetase [ambiguous]; alanine:alanine ligase (ADP-forming); alanylalanine synthetase | ATP + 2 D-alanine = ADP + phosphate + D-alanyl-D-alanine [RN:R01150] |
| ec:2.7.7.74 | 1 | 1L-myo-inositol 1-phosphate cytidylyltransferase; CTP:inositol-1-phosphate cytidylyltransferase (bifunctional CTP:inositol-1-phosphate cytidylyltransferase/CDP-inositol:inositol-1-phosphate transferase (IPCT/DIPPS)); IPCT (bifunctional CTP:inositol-1-phosphate cytidylyltransferase/CDP-inositol:inositol-1-phosphate transferase (IPCT/DIPPS)); L-myo-inositol-1-phosphate cytidylyltransferase | CTP + 1L-myo-inositol 1-phosphate = diphosphate + CDP-1L-myo-inositol [RN:R09669] |
| ec:2.3.1.30 | 1 | serine O-acetyltransferase; SATase; L-serine acetyltransferase; serine acetyltransferase; serine transacetylase | acetyl-CoA + L-serine = CoA + O-acetyl-L-serine [RN:R00586] |
| ec:2.7.7.72 | 1 | CCA tRNA nucleotidyltransferase; CCA-adding enzyme; tRNA adenylyltransferase; tRNA cytidylyltransferase; tRNA CCA-pyrophosphorylase; tRNA-nucleotidyltransferase; transfer-RNA nucleotidyltransferase; transfer ribonucleic acid nucleotidyl transferase; CTP(ATP):tRNA nucleotidyltransferase; transfer ribonucleate adenylyltransferase; transfer ribonucleate adenyltransferase; transfer RNA adenylyltransferase; transfer ribonucleate nucleotidyltransferase; ATP (CTP):tRNA nucleotidyltransferase; ribonucleic cytidylic cytidylic adenylic pyrophosphorylase; transfer ribonucleic adenylyl (cytidylyl) transferase; transfer ribonucleic-terminal trinucleotide nucleotidyltransferase; transfer ribonucleate cytidylyltransferase; ribonucleic cytidylyltransferase; -C-C-A pyrophosphorylase; ATP(CTP)-tRNA nucleotidyltransferase; tRNA adenylyl(cytidylyl)transferase; CTP:tRNA cytidylyltransferase | a tRNA precursor + 2 CTP + ATP = a tRNA with a 3' CCA end + 3 diphosphate (overall reaction) [RN:R09382]; (1a) a tRNA precursor + CTP = a tRNA with a 3' cytidine end + diphosphate [RN:R09383]; (1b) a tRNA with a 3' cytidine + CTP = a tRNA with a 3' CC end + diphosphate [RN:R09384]; (1c) a tRNA with a 3' CC end + ATP = a tRNA with a 3' CCA end + diphosphate [RN:R09386] |
| ec:6.1.1.9 | 1 | valine---tRNA ligase; valyl-tRNA synthetase; valyl-transfer ribonucleate synthetase; valyl-transfer RNA synthetase; valyl-transfer ribonucleic acid synthetase; valine transfer ribonucleate ligase; valine translase | ATP + L-valine + tRNAVal = AMP + diphosphate + L-valyl-tRNAVal [RN:R03665] |
| ec:5.1.1.3 | 1 | glutamate racemase | L-glutamate = D-glutamate [RN:R00260] |
| ec:4.3.2.2 | 1 | adenylosuccinate lyase; adenylosuccinase; succino AMP-lyase; 6-N-(1,2-dicarboxyethyl)AMP AMP-lyase; 6-N-(1,2-dicarboxyethyl)AMP AMP-lyase (fumarate-forming) | (1) N6-(1,2-dicarboxyethyl)AMP = fumarate + AMP [RN:R01083]; (2) (S)-2-[5-amino-1-(5-phospho-D-ribosyl)imidazole-4-carboxamido]succinate = fumarate + 5-amino-1-(5-phospho-D-ribosyl)imidazole-4-carboxamide [RN:R04559] |
| ec:3.1.3.7 | 1 | 3'(2'),5'-bisphosphate nucleotidase; phosphoadenylate 3'-nucleotidase; 3'-phosphoadenylylsulfate 3'-phosphatase; 3'(2'),5'-bisphosphonucleoside 3'(2')-phosphohydrolase | adenosine 3',5'-bisphosphate + H2O = AMP + phosphate [RN:R00188] |
| ec:2.3.2.2 | 1 | gamma-glutamyltransferase; glutamyl transpeptidase; alpha-glutamyl transpeptidase; gamma-glutamyl peptidyltransferase; gamma-glutamyl transpeptidase (ambiguous); gamma-GPT; gamma-GT; gamma-GTP; L-gamma-glutamyl transpeptidase; L-gamma-glutamyltransferase; L-glutamyltransferase; GGT (ambiguous); gamma-glutamyltranspeptidase (ambiguous) | a (5-L-glutamyl)-peptide + an amino acid = a peptide + a 5-L-glutamyl amino acid [RN:R04159] |
| ec:2.7.7.62 | 1 | adenosylcobinamide-phosphate guanylyltransferase; CobU; adenosylcobinamide kinase/adenosylcobinamide-phosphate guanylyltransferase; AdoCbi kinase/AdoCbi-phosphate guanylyltransferase | GTP + adenosylcobinamide phosphate = diphosphate + adenosylcobinamide-GDP [RN:R05222] |
| ec:4.2.99.18 | 1 | DNA-(apurinic or apyrimidinic site) lyase; AP lyase; AP endonuclease class I; endodeoxyribonuclease (apurinic or apyrimidinic); deoxyribonuclease (apurinic or apyrimidinic); E. coli endonuclease III; phage-T4 UV endonuclease; Micrococcus luteus UV endonuclease; AP site-DNA 5'-phosphomonoester-lyase; X-ray endonuclease III | The C-O-P bond 3' to the apurinic or apyrimidinic site in DNA is broken by a beta-elimination reaction, leaving a 3'-terminal unsaturated sugar and a product with a terminal 5'-phosphate |
| ec:2.1.3.2 | 1 | aspartate carbamoyltransferase; carbamylaspartotranskinase; aspartate transcarbamylase; aspartate carbamyltransferase; aspartic acid transcarbamoylase; aspartic carbamyltransferase; aspartic transcarbamylase; carbamylaspartotranskinase; L-aspartate transcarbamoylase; L-aspartate transcarbamylase; carbamoylaspartotranskinase; aspartate transcarbamylase; aspartate transcarbamoylase; ATCase | carbamoyl phosphate + L-aspartate = phosphate + N-carbamoyl-L-aspartate [RN:R01397] |
| ec:1.8.1.9 | 1 | thioredoxin-disulfide reductase; NADP-thioredoxin reductase; NADPH-thioredoxin reductase; thioredoxin reductase (NADPH); NADPH2:oxidized thioredoxin oxidoreductase | thioredoxin + NADP+ = thioredoxin disulfide + NADPH + H+ [RN:R02016] |
| ec:2.1.1.71 | 1 | phosphatidyl-N-methylethanolamine N-methyltransferase; phosphatidylmonomethylethanolamine methyltransferase; methyltransferase II; phospholipid methyltransferase; PLMT; phosphatidyl-N-methylethanolamine methyltransferase; phosphatidyl-N-monomethylethanolamine methyltransferase; phosphatidylethanolamine methyltransferase I; phosphatidylmonomethylethanolamine methyltransferase | S-adenosyl-L-methionine + phosphatidyl-N-methylethanolamine = S-adenosyl-L-homocysteine + phosphatidyl-N-dimethylethanolamine [RN:R03424] |
| ec:2.8.1.7 | 1 | cysteine desulfurase; IscS; NIFS; NifS; SufS; cysteine desulfurylase | L-cysteine + acceptor = L-alanine + S-sulfanyl-acceptor (overall reaction); (1a) L-cysteine + [enzyme]-cysteine = L-alanine + [enzyme]-S-sulfanylcysteine [RN:R07460]; (1b) [enzyme]-S-sulfanylcysteine + acceptor = [enzyme]-cysteine + S-sulfanyl-acceptor |
| ec:2.7.1.15 | 1 | ribokinase; deoxyribokinase; ribokinase (phosphorylating); D-ribokinase | ATP + D-ribose = ADP + D-ribose 5-phosphate [RN:R01051] |
| ec:2.5.1.72 | 1 | quinolinate synthase; NadA; QS; quinolinate synthetase | glycerone phosphate + iminosuccinate = pyridine-2,3-dicarboxylate + 2 H2O + phosphate [RN:R04292] |
| ec:2.7.1.11 | 1 | 6-phosphofructokinase; phosphohexokinase; phosphofructokinase I; phosphofructokinase (phosphorylating); 6-phosphofructose 1-kinase; ATP-dependent phosphofructokinase; D-fructose-6-phosphate 1-phosphotransferase; fructose 6-phosphate kinase; fructose 6-phosphokinase; nucleotide triphosphate-dependent phosphofructokinase; phospho-1,6-fructokinase; PFK | ATP + D-fructose 6-phosphate = ADP + D-fructose 1,6-bisphosphate [RN:R00756] |
| ec:4.99.1.4 | 1 | sirohydrochlorin ferrochelatase; CysG; Met8P; SirB; sirohydrochlorin ferro-lyase (incorrect) | siroheme + 2 H+ = sirohydrochlorin + Fe2+ [RN:R02864] |
| ec:3.1.3.27 | 1 | phosphatidylglycerophosphatase; phosphatidylglycerol phosphate phosphatase; phosphatidylglycerol phosphatase; PGP phosphatase | phosphatidylglycerophosphate + H2O = phosphatidylglycerol + phosphate [RN:R02029] |
| ec:2.1.1.171 | 1 | 16S rRNA (guanine966-N2)-methyltransferase; yhhF (gene name); rsmD (gene name); m2G966 methyltransferase | S-adenosyl-L-methionine + guanine966 in 16S rRNA = S-adenosyl-L-homocysteine + N2-methylguanine966 in 16S rRNA [RN:R07234] |
| ec:1.3.1.76 | 1 | precorrin-2 dehydrogenase; Met8p; SirC; CysG | precorrin-2 + NAD+ = sirohydrochlorin + NADH + H+ [RN:R03947] |

  
**Over-represented Metabolite Summary**: Collection of the metabolites identified as substrates or products of the proteins representaed the "Over-represented Enzyme Summary" ranked by frequency of occurrence  

| ID | Structure | Name | Frequency | EC |
| --- | --- | --- | --- | --- |
| cpd:C00001 |  | H2O; Water | 110 | ec:1.4.1.14  ec:3.1.1.85 ec:1.4.1.13 ec:2.3.3.14 ec:3.5.1.2 ec:3.5.1.1 ec:4.2.1.35 ec:2.5.1.72 ec:1.2.1.3 ec:1.1.1.312 ec:1.17.4.1 ec:3.1.1.24 ec:3.2.1.52 ec:3.5.4.16 ec:3.2.1.1 ec:2.5.1.18 ec:4.2.1.33 ec:1.13.12.16 ec:2.6.99.2 ec:3.1.3.27 ec:3.1.3.7 ec:2.3.2.2 ec:2.3.3.1 ec:5.4.99.16 ec:4.2.1.9 ec:2.3.3.9 ec:4.2.1.24 ec:1.8.1.9 ec:3.1.4.16 ec:4.2.1.20 ec:3.6.3.55 ec:3.11.1.2 ec:4.2.1.83 ec:2.5.1.48 ec:1.9.3.1 ec:1.2.99.5 ec:3.1.1.57 ec:1.18.6.1 ec:1.7.2.5 |
| cpd:C00080 |  | H+; Hydron | 92 | ec:1.4.1.14 ec:1.4.1.13 ec:2.8.1.6 ec:1.2.1.38 ec:1.2.1.3 ec:1.2.1.2 ec:1.1.1.312 ec:4.99.1.4 ec:4.1.1.50 ec:2.5.1.18 ec:2.1.1.107 ec:1.3.1.10 ec:1.3.1.9 ec:1.3.1.76 ec:1.8.1.4 ec:1.1.1.27 ec:1.8.1.9 ec:1.2.7.3 ec:1.1.1.85 ec:2.5.1.47 ec:2.5.1.49 ec:1.7.1.13 ec:1.9.3.1 ec:1.18.6.1 ec:1.7.2.5 |
| cpd:C00002 |  | ATP; Adenosine 5'-triphosphate | 66 | ec:6.3.1.10 ec:6.2.1.30  ec:6.1.1.9 ec:2.7.2.1 ec:2.7.7.62 ec:6.3.4.14 ec:2.5.1.17 ec:6.3.2.4 ec:6.1.1.24 ec:6.1.1.12 ec:6.1.1.11 ec:6.1.1.10 ec:2.7.1.39 ec:2.7.1.11 ec:6.2.1.3 ec:2.7.6.5 ec:2.7.1.156 ec:2.7.4.16 ec:2.7.7.3 ec:6.3.3.3 ec:4.6.1.1 ec:2.7.7.72 ec:2.7.1.71 ec:3.6.3.55 ec:2.7.1.15 ec:6.1.1.17 ec:1.18.6.1 ec:6.4.1.2 |
| cpd:C00009 |  | Orthophosphate; Phosphate; Phosphoric acid; Orthophosphoric acid | 55 | ec:3.1.3.7 ec:6.3.1.10  ec:1.2.1.38 ec:2.1.3.2 ec:2.5.1.72 ec:6.3.3.3 ec:3.6.3.55 ec:2.4.1.1 ec:2.4.99.16 ec:2.3.1.8 ec:3.11.1.2 ec:6.3.4.14 ec:6.3.2.4 ec:2.5.1.48 ec:2.6.99.2 ec:4.2.3.4 ec:3.1.3.27 ec:1.18.6.1 ec:6.4.1.2 |
| cpd:C00008 |  | ADP; Adenosine 5'-diphosphate | 53 | ec:6.3.1.10  ec:2.7.1.39 ec:2.7.1.11 ec:2.7.1.156 ec:6.3.3.3 ec:2.7.4.16 ec:1.17.4.1 ec:2.7.2.1 ec:2.7.7.62 ec:3.6.3.55 ec:2.7.1.71 ec:2.7.1.15 ec:6.3.4.14 ec:6.3.2.4 ec:1.18.6.1 ec:6.4.1.2 |
| cpd:C00013 |  | Diphosphate; Diphosphoric acid; Pyrophosphate; Pyrophosphoric acid; PPi | 47 | ec:6.1.1.12 ec:6.2.1.30 ec:6.1.1.11 ec:6.1.1.10 ec:6.2.1.3 ec:6.1.1.9 ec:2.5.1.3 ec:2.7.1.156 ec:2.7.7.3 ec:2.7.7.74 ec:2.7.8.34 ec:2.7.7.7 ec:2.4.2.10 ec:4.6.1.1 ec:2.7.7.72 ec:2.7.7.62 ec:6.1.1.24 ec:6.1.1.17 |
| cpd:C00011 |  | CO2; Carbon dioxide | 43 | ec:1.2.7.3 ec:1.1.1.85 ec:4.1.1.50 ec:4.1.1.9 ec:2.2.1.6 ec:2.3.1.47 ec:1.2.99.5 ec:1.8.1.4 ec:1.14.11.17 ec:6.3.3.3 ec:1.2.1.2 |
| cpd:C00010 |  | CoA; Coenzyme A; CoA-SH | 38 | ec:6.2.1.30  ec:2.3.1.30 ec:1.2.7.3 ec:2.3.3.14 ec:2.3.1.8 ec:2.3.3.1 ec:2.3.1.47 ec:6.2.1.3 ec:1.8.1.4 ec:2.3.3.9 ec:2.7.8.7 |
| cpd:C00004 |  | NADH; DPNH; Reduced nicotinamide adenine dinucleotide | 32 | ec:1.4.1.14  ec:1.4.1.13 ec:1.3.1.76 ec:1.8.1.4 ec:1.2.1.3 ec:1.1.1.27 ec:1.2.1.2 ec:1.1.1.312 ec:1.1.1.85 ec:4.99.1.4 ec:2.1.1.107 ec:1.3.1.10 ec:1.3.1.9 |
| cpd:C00003 |  | NAD+; NAD; Nicotinamide adenine dinucleotide; DPN; Diphosphopyridine nucleotide; Nadide | 32 | ec:1.4.1.14  ec:1.4.1.13 ec:1.3.1.76 ec:1.8.1.4 ec:1.2.1.3 ec:1.1.1.27 ec:1.2.1.2 ec:1.1.1.312 ec:1.1.1.85 ec:4.99.1.4 ec:2.1.1.107 ec:1.3.1.10 ec:1.3.1.9 |
| cpd:C00014 |  | Ammonia; NH3 | 30 | ec:1.4.1.14 ec:1.4.1.13 ec:3.5.1.2 ec:3.5.1.1 ec:2.5.1.48 ec:1.8.1.4 ec:4.3.1.7 ec:1.18.6.1 |
| cpd:C00024 |  | Acetyl-CoA; Acetyl coenzyme A | 28 | ec:2.3.1.30 ec:2.3.3.14 ec:4.1.1.9 ec:2.3.1.8 ec:2.3.3.1 ec:6.3.4.14 ec:6.4.1.2 ec:2.3.3.9 |
| cpd:C00139 |  | Oxidized ferredoxin | 27 | ec:1.2.7.3 ec:1.18.6.1 |
| cpd:C00138 |  | Reduced ferredoxin | 27 | ec:1.2.7.3 ec:1.18.6.1 |
| cpd:C04327 |  | 4-Methyl-5-(2-phosphoethyl)-thiazole; 4-Methyl-5-(2-phosphono-oxyethyl)-thiazole | 22 | ec:2.5.1.3 |
| cpd:C01081 |  | Thiamin monophosphate; Thiamine monophosphate; Thiamin phosphate; Thiamine phosphate; TMP | 20 | ec:2.5.1.3 ec:2.7.4.16 |
| cpd:C04752 |  | 2-Methyl-4-amino-5-hydroxymethylpyrimidine diphosphate; 4-Amino-2-methyl-5-diphosphomethylpyrimidine | 19 | ec:2.5.1.3 |
| cpd:C00058 |  | Formate; Methanoic acid; Formic acid | 18 | ec:3.5.4.16 ec:1.2.99.5 ec:1.2.1.2 |
| cpd:C00026 |  | 2-Oxoglutarate; Oxoglutaric acid; 2-Ketoglutaric acid; alpha-Ketoglutaric acid | 17 | ec:1.4.1.14 ec:1.4.1.13 ec:1.2.7.3 ec:2.3.3.14 ec:1.8.1.4 ec:1.14.11.17 ec:1.2.1.3 ec:2.6.1.9 |
| cpd:C00697 |  | Nitrogen; N2 | 16 | ec:1.18.6.1 |
| cpd:C00282 |  | Hydrogen; H2 | 16 | ec:1.18.6.1 |
| cpd:C06547 |  | Ethylene | 16 | ec:1.18.6.1 |
| cpd:C01548 |  | Acetylene; Ethyne | 16 | ec:1.18.6.1 |
| cpd:C00263 |  | L-Homoserine; 2-Amino-4-hydroxybutyric acid | 14 | ec:2.7.1.39 |
| cpd:C00033 |  | Acetate; Acetic acid; Ethanoic acid | 14 | ec:2.7.2.1 ec:2.5.1.47 ec:3.11.1.2 ec:2.5.1.49 ec:2.5.1.48 ec:1.2.1.3 |
| cpd:C01102 |  | O-Phospho-L-homoserine | 14 | ec:2.7.1.39 |
| cpd:C00132 |  | Methanol; Methyl alcohol; CH3OH | 13 | ec:3.1.1.85 ec:1.13.11.8 |
| cpd:C00019 |  | S-Adenosyl-L-methionine; S-Adenosylmethionine; AdoMet; SAM | 13 | ec:2.1.1.17 ec:4.99.1.4 ec:4.1.1.50 ec:2.8.1.6 ec:1.3.1.76 ec:2.1.1.197 ec:2.1.1.71 ec:2.1.1.171 ec:2.1.1.10 ec:2.1.1.107 |
| cpd:C00006 |  | NADP+; NADP; Nicotinamide adenine dinucleotide phosphate; beta-Nicotinamide adenine dinucleotide phosphate; TPN; Triphosphopyridine nucleotide | 13 | ec:1.4.1.14  ec:1.4.1.13 ec:1.7.1.13 ec:1.2.1.38 ec:1.2.1.3 ec:1.3.1.10 ec:1.1.1.312 ec:1.8.1.9 ec:1.3.1.9 |
| cpd:C00005 |  | NADPH; TPNH; Reduced nicotinamide adenine dinucleotide phosphate | 13 | ec:1.4.1.14  ec:1.4.1.13 ec:1.7.1.13 ec:1.2.1.38 ec:1.2.1.3 ec:1.3.1.10 ec:1.1.1.312 ec:1.8.1.9 ec:1.3.1.9 |
| cpd:C00025 |  | L-Glutamate; L-Glutamic acid; L-Glutaminic acid; Glutamate | 12 | ec:1.4.1.14 ec:1.4.1.13 ec:2.3.2.2 ec:3.5.1.2 ec:5.1.1.3 ec:6.1.1.24 ec:6.1.1.17 ec:2.6.1.9 |
| cpd:C03453 |  | gamma-Oxalocrotonate; (Z)-5-Oxohex-2-enedioate; 4-Oxalocrotonate | 11 | ec:5.3.2.6 |
| cpd:C07479 |  | 2-Oxo-5-methyl-cis-muconate | 11 | ec:5.3.2.6 |
| cpd:C07478 |  | 2-Hydroxy-5-methyl-cis,cis-muconate | 11 | ec:5.3.2.6 |
| cpd:C00020 |  | AMP; Adenosine 5'-monophosphate; Adenylic acid; Adenylate; 5'-AMP; 5'-Adenylic acid; 5'-Adenosine monophosphate; Adenosine 5'-phosphate | 11 | ec:6.2.1.30 ec:6.1.1.12 ec:6.1.1.11 ec:6.1.1.10 ec:6.1.1.24 ec:6.2.1.3 ec:6.1.1.17 ec:6.1.1.9 ec:2.7.6.5 ec:4.3.2.2 |
| cpd:C00007 |  | Oxygen; O2 | 11 | ec:1.9.3.1 ec:1.13.12.16 ec:1.4.3.19 ec:1.14.11.17 ec:1.13.11.8 |
| cpd:C02501 |  | 2-Hydroxymuconate | 11 | ec:5.3.2.6 |
| cpd:C00044 |  | GTP; Guanosine 5'-triphosphate | 10 | ec:3.5.4.16  ec:4.6.1.1 ec:2.7.7.62 ec:2.7.6.5 ec:2.7.1.156 |
| cpd:C00369 |  | Starch | 10 | ec:3.2.1.1 ec:2.4.1.18 ec:2.4.1.1 ec:5.4.99.16 ec:3.2.1.141 |
| cpd:C00229 |  | Acyl-carrier protein; ACP; [Acyl-carrier protein]; Holo-[acyl-carrier protein] | 9 | ec:2.3.1.47 ec:2.7.8.7 |
| cpd:C00091 |  | Succinyl-CoA; Succinyl coenzyme A | 9 | ec:1.2.7.3 ec:5.4.99.2 ec:1.8.1.4 |
| cpd:C00022 |  | Pyruvate; Pyruvic acid; 2-Oxopropanoate; 2-Oxopropanoic acid; Pyroracemic acid | 9 | ec:4.1.3.17 ec:2.2.1.6 ec:1.1.1.27 |
| cpd:C06676 |  | 4-Sulfolactone; 4-Carboxymethyl-4-sulfobut-2-en-4-olide; 4-Sulfomuconolactone | 8 |  |
| cpd:C00718 |  | Amylose; Amylose chain; (1,4-alpha-D-Glucosyl)n; (1,4-alpha-D-Glucosyl)n+1; (1,4-alpha-D-Glucosyl)n-1; 4-{(1,4)-alpha-D-Glucosyl}(n-1)-D-glucose; 1,4-alpha-D-Glucan | 8 | ec:2.4.1.18 ec:2.4.1.1 ec:2.4.99.16 |
| cpd:C00156 |  | 4-Hydroxybenzoate; Hydroxybenzoic acid; 4-Hydroxybenzoic acid; Hydroxybenzenecarboxylic acid | 8 |  |
| cpd:C00530 |  | Hydroquinone; p-Benzenediol; 1,4-Benzenediol; 1,4-Dihydroxybenzene; Benzene-1,4-diol; Quinol; 4-Hydroxyphenol | 8 |  |
| cpd:C00097 |  | L-Cysteine; L-2-Amino-3-mercaptopropionic acid | 8 | ec:2.8.1.7 ec:2.5.1.47 ec:2.5.1.49 ec:2.5.1.48 |
| cpd:C18216 |  | 4-Hydroxyphenyl-4-hydroxybenzoate | 8 |  |
| cpd:C00126 |  | Ferrocytochrome c; Cytochrome c2+; Reduced cytochrome c | 8 | ec:1.9.3.1 ec:1.7.2.5 |
| cpd:C00125 |  | Ferricytochrome c; Cytochrome c3+ | 8 | ec:1.9.3.1 ec:1.7.2.5 |
| cpd:C00083 |  | Malonyl-CoA; Malonyl coenzyme A | 8 | ec:4.1.1.9 ec:6.3.4.14 ec:6.4.1.2 |
| cpd:C10858 |  | Ecgonine | 8 |  |
| cpd:C02222 |  | 2-Maleylacetate; 4-Oxohex-2-enedioate; Maleylacetate | 8 |  |
| cpd:C00109 |  | 2-Oxobutanoate; 2-Ketobutyric acid; 2-Oxobutyric acid; 2-Oxobutyrate; 2-Oxobutanoic acid; alpha-Ketobutyric acid; alpha-Ketobutyrate | 8 | ec:1.1.1.85 ec:2.2.1.6 ec:2.5.1.48 ec:1.1.1.27 |
| cpd:C11481 |  | HSO3-; Hydrogen sulfite; Bisulfite | 8 |  |
| cpd:C01909 |  | Dethiobiotin; Desthiobiotin | 8 | ec:2.8.1.6 ec:6.3.3.3 |
| cpd:C12448 |  | Ecgonine methyl ester; Methyl ecgonine | 8 |  |
| cpd:C00286 |  | dGTP; 2'-Deoxyguanosine 5'-triphosphate; Deoxyguanosine 5'-triphosphate; Deoxyguanosine triphosphate | 7 | ec:2.7.7.7 |
| cpd:C00283 |  | Hydrogen sulfide; Hydrogen-sulfide; H2S; Sulfide | 7 | ec:2.5.1.47 ec:2.5.1.49 ec:2.5.1.48 |
| cpd:C00227 |  | Acetyl phosphate | 7 | ec:2.7.2.1 ec:2.3.1.8 |
| cpd:C02320 |  | R-S-Glutathione | 7 | ec:2.3.2.2 ec:2.5.1.18 |
| cpd:C00155 |  | L-Homocysteine; L-2-Amino-4-mercaptobutyric acid | 7 | ec:2.5.1.49 ec:2.5.1.48 ec:2.1.1.10 |
| cpd:C00149 |  | (S)-Malate; L-Malate; L-Apple acid; L-Malic acid; L-2-Hydroxybutanedioic acid; Malate; Malic acid | 7 | ec:2.3.3.9 |
| cpd:C00131 |  | dATP; 2'-Deoxyadenosine 5'-triphosphate; Deoxyadenosine 5'-triphosphate; Deoxyadenosine triphosphate | 7 | ec:2.7.7.7 |
| cpd:C00073 |  | L-Methionine; Methionine; L-2-Amino-4methylthiobutyric acid | 7 | ec:6.1.1.10 ec:2.8.1.6 ec:2.1.1.10 |
| cpd:C00459 |  | dTTP; Deoxythymidine triphosphate; Deoxythymidine 5'-triphosphate; TTP | 7 | ec:2.7.7.7 |
| cpd:C00458 |  | dCTP; Deoxycytidine 5'-triphosphate; Deoxycytidine triphosphate; 2'-Deoxycytidine 5'-triphosphate | 7 | ec:2.7.7.7 |
| cpd:C00051 |  | Glutathione; 5-L-Glutamyl-L-cysteinylglycine; N-(N-gamma-L-Glutamyl-L-cysteinyl)glycine; gamma-L-Glutamyl-L-cysteinyl-glycine; GSH; Reduced glutathione | 7 | ec:2.3.2.2 ec:2.5.1.18 |
| cpd:C00048 |  | Glyoxylate; Glyoxalate; Glyoxylic acid | 7 | ec:2.3.3.9 |
| cpd:C00039 |  | DNA; DNAn; DNAn+1; (Deoxyribonucleotide)n; (Deoxyribonucleotide)m; (Deoxyribonucleotide)n+m; Deoxyribonucleic acid | 7 | ec:2.7.7.7 |
| cpd:C00021 |  | S-Adenosyl-L-homocysteine; S-Adenosylhomocysteine | 7 | ec:2.1.1.17 ec:4.99.1.4 ec:1.3.1.76 ec:2.1.1.197 ec:2.1.1.71 ec:2.1.1.171 ec:2.1.1.10 ec:2.1.1.107 |
| cpd:C02876 |  | Propanoyl phosphate; Propionyl phosphate | 7 | ec:2.7.2.1 ec:2.3.1.8 |
| cpd:C00343 |  | Thioredoxin disulfide; Oxidized thioredoxin; Thioredoxin sulfide | 6 | ec:1.17.4.1 ec:2.5.1.47 ec:2.5.1.49 ec:1.8.1.9 |
| cpd:C00342 |  | Thioredoxin; Reduced thioredoxin | 6 | ec:1.17.4.1 ec:2.5.1.47 ec:2.5.1.49 ec:1.8.1.9 |
| cpd:C19845 |  | Pimeloyl-[acyl-carrier protein]; Pimeloyl-[acp]; Pimelyl-[acyl-carrier protein]; Pimelyl-[acp]; 7-Hydroxy-7-oxoheptanoyl-[acyl-carrier protein] | 6 | ec:3.1.1.85 ec:2.3.1.47 |
| cpd:C14874 |  | Glutathione episulfonium ion | 6 | ec:2.5.1.18 |
| cpd:C14871 |  | S-(Formylmethyl)glutathione | 6 | ec:2.5.1.18 |
| cpd:C14870 |  | 2-Bromoacetaldehyde | 6 | ec:2.5.1.18 |
| cpd:C14868 |  | S-(1,2-Dichlorovinyl)glutathione; DCVG | 6 | ec:2.5.1.18 |
| cpd:C11278 |  | Aflatoxin B1exo-8,9-epoxide-GSH; 8,9-Dihydro-8-(S-glutathionyl)-9-hydroxyaflatoxin B1 | 6 | ec:2.5.1.18 |
| cpd:C14865 |  | 2-(S-Glutathionyl)acetyl chloride | 6 | ec:2.5.1.18 |
| cpd:C14864 |  | S-(2-Chloroacetyl)glutathione | 6 | ec:2.5.1.18 |
| cpd:C14863 |  | 2-(S-Glutathionyl)acetyl glutathione | 6 | ec:2.5.1.18 |
| cpd:C14861 |  | S-(2,2-Dichloro-1-hydroxy)ethyl glutathione | 6 | ec:2.5.1.18 |
| cpd:C01327 |  | Hydrochloric acid; HCl; Hydrogen chloride; Hydrochloride | 6 | ec:2.5.1.18 |
| cpd:C01322 |  | RX; Organic halide | 6 | ec:2.5.1.18 |
| cpd:C14859 |  | Chloroacetyl chloride | 6 | ec:2.5.1.18 |
| cpd:C14858 |  | 2,2-Dichloroacetaldehyde | 6 | ec:2.5.1.18 |
| cpd:C14857 |  | 1,1-Dichloroethylene epoxide; 2,2-Dichlorooxirane | 6 | ec:2.5.1.18 |
| cpd:C14856 |  | 7,8-Dihydro-7-hydroxy-8-S-glutathionyl-benzo[a]pyrene | 6 | ec:2.5.1.18 |
| cpd:C14855 |  | 4,5-Dihydro-4-hydroxy-5-S-glutathionyl-benzo[a]pyrene | 6 | ec:2.5.1.18 |
| cpd:C14852 |  | Benzo[a]pyrene-7,8-diol; Benzo[a]pyrene-7,8-dihydrodiol | 6 | ec:2.5.1.18 |
| cpd:C14851 |  | Benzo[a]pyrene-4,5-oxide; Benzo[a]pyrene-4,5-epoxide | 6 | ec:2.5.1.18 |
| cpd:C01674 |  | Chitobiose; Diacetylchitobiose; N,N'-Diacetylchitobiose | 6 | ec:3.2.1.52 |
| cpd:C14848 |  | 2,3-Dihydro-2-S-glutathionyl-3-hydroxy bromobenzene | 6 | ec:2.5.1.18 |
| cpd:C14847 |  | 3,4-Dihydro-3-hydroxy-4-S-glutathionyl bromobenzene | 6 | ec:2.5.1.18 |
| cpd:C14840 |  | Bromobenzene-2,3-oxide; Bromobenzene-2,3-epoxide | 6 | ec:2.5.1.18 |
| cpd:C00627 |  | Pyridoxine phosphate; Pyridoxine 5-phosphate; Pyridoxine 5'-phosphate; Pyridoxol 5'-phosphate | 6 | ec:2.6.99.2 |
| cpd:C14839 |  | Bromobenzene-3,4-oxide; Bromobenzene-3,4-epoxide | 6 | ec:2.5.1.18 |
| cpd:C07645 |  | Aldophosphamide | 6 | ec:2.5.1.18 |
| cpd:C14793 |  | (1R)-Glutathionyl-(2R)-hydroxy-1,2-dihydronaphthalene | 6 | ec:2.5.1.18 |
| cpd:C14792 |  | (1S)-Hydroxy-(2S)-glutathionyl-1,2-dihydronaphthalene | 6 | ec:2.5.1.18 |
| cpd:C14791 |  | (1R)-Hydroxy-(2R)-glutathionyl-1,2-dihydronaphthalene | 6 | ec:2.5.1.18 |
| cpd:C11638 |  | 3-Amino-2-oxopropyl phosphate; 1-Amino-3-(phosphohydroxy)propan-2-one | 6 | ec:2.6.99.2 |
| cpd:C14787 |  | (1S,2R)-Naphthalene 1,2-oxide; (1S,2R)-Naphthalene epoxide | 6 | ec:2.5.1.18 |
| cpd:C14786 |  | (1R,2S)-Naphthalene 1,2-oxide; (1R,2S)-Naphthalene epoxide | 6 | ec:2.5.1.18 |
| cpd:C11583 |  | 4-Glutathionyl cyclophosphamide | 6 | ec:2.5.1.18 |
| cpd:C14806 |  | 1-Nitro-5-glutathionyl-6-hydroxy-5,6-dihydronaphthalene | 6 | ec:2.5.1.18 |
| cpd:C14805 |  | 1-Nitro-5-hydroxy-6-glutathionyl-5,6-dihydronaphthalene | 6 | ec:2.5.1.18 |
| cpd:C14804 |  | 1-Nitro-7-glutathionyl-8-hydroxy-7,8-dihydronaphthalene | 6 | ec:2.5.1.18 |
| cpd:C14803 |  | 1-Nitro-7-hydroxy-8-glutathionyl-7,8-dihydronaphthalene | 6 | ec:2.5.1.18 |
| cpd:C14802 |  | 1-Nitronaphthalene-7,8-oxide | 6 | ec:2.5.1.18 |
| cpd:C14800 |  | 1-Nitronaphthalene-5,6-oxide | 6 | ec:2.5.1.18 |
| cpd:C00140 |  | N-Acetyl-D-glucosamine; N-Acetylchitosamine; 2-Acetamido-2-deoxy-D-glucose; GlcNAc | 6 | ec:3.2.1.52 |
| cpd:C03688 |  | Apo-[acyl-carrier-protein] | 6 | ec:2.7.8.7 |
| cpd:C00084 |  | Acetaldehyde; Ethanal | 6 | ec:4.1.2.4 ec:1.13.12.16 ec:4.3.1.7 ec:1.2.1.3 |
| cpd:C13645 |  | Hydrobromic acid; HBr | 6 | ec:2.5.1.18 |
| cpd:C00462 |  | Halide; Hydrogen halide; HX; Halo acid | 6 | ec:2.5.1.18 |
| cpd:C00064 |  | L-Glutamine; L-2-Aminoglutaramic acid | 6 | ec:1.4.1.14 ec:1.4.1.13 ec:3.5.1.2 |
| cpd:C11088 |  | 1,2-Dibromoethane; Ethylene dibromide | 6 | ec:2.5.1.18 |
| cpd:C19586 |  | Aflatoxin B1-exo-8,9-epoxide; 2,3-Epoxyaflatoxin B1 | 6 | ec:2.5.1.18 |
| cpd:C01132 |  | N-Acetyl-D-galactosamine; N-Acetyl-D-chondrosamine; 2-Acetamido-2-deoxy-D-galactose | 6 | ec:3.2.1.52 |
| cpd:C00054 |  | Adenosine 3',5'-bisphosphate; PAP; 3'-Phosphoadenylate; Phosphoadenosine phosphate | 6 | ec:2.7.8.7 |
| cpd:C06790 |  | Trichloroethene; Trichloroethylene; TCE | 6 | ec:2.5.1.18 |
| cpd:C01077 |  | O-Acetyl-L-homoserine; O-Acetylhomoserine | 6 | ec:2.5.1.49 ec:2.5.1.48 |
| cpd:C11437 |  | 1-Deoxy-D-xylulose 5-phosphate | 6 | ec:2.6.99.2 |
| cpd:C05198 |  | 5'-Deoxyadenosine | 5 | ec:2.8.1.6 |
| cpd:C19846 |  | Pimeloyl-[acyl-carrier protein] methyl ester; Pimeloyl-[acp] methyl ester; Pimelyl-[acyl-carrier protein] methyl ester; Pimelyl-[acp] methyl ester | 5 | ec:3.1.1.85 ec:1.3.1.10 ec:1.3.1.9 |
| cpd:C04434 |  | (1E)-4-Oxobut-1-ene-1,2,4-tricarboxylate; 4-Oxalomesaconate; 4-Oxalmesaconic acid | 5 | ec:4.2.1.83 ec:5.3.2.8 ec:3.1.1.57 ec:1.13.11.8 |
| cpd:C00533 |  | Nitric oxide; NO; Nitrogen monoxide | 5 | ec:1.7.2.5 |
| cpd:C00094 |  | Sulfite; Sulfurous acid | 5 | ec:2.5.1.47 ec:2.5.1.49 ec:1.14.11.17 |
| cpd:C00887 |  | Nitrous oxide; Dinitrogen monoxide; Dinitrogen oxide; N2O | 5 | ec:1.7.2.5 |
| cpd:C00120 |  | Biotin; D-Biotin; Vitamin H; Coenzyme R | 5 | ec:2.8.1.6 |
| cpd:C00082 |  | L-Tyrosine; (S)-3-(p-Hydroxyphenyl)alanine; (S)-2-Amino-3-(p-hydroxyphenyl)propionic acid; Tyrosine | 5 | ec:2.6.1.9 |
| cpd:C05359 |  | e-; Electron | 5 | ec:2.8.1.6 |
| cpd:C01083 |  | alpha,alpha-Trehalose; alpha,alpha'-Trehalose; Trehalose | 5 | ec:3.2.1.1 ec:5.4.99.16 ec:3.2.1.141 |
| cpd:C00042 |  | Succinate; Succinic acid; Butanedionic acid; Ethylenesuccinic acid | 5 | ec:2.5.1.48 ec:1.14.11.17 |
| cpd:C05688 |  | L-Selenocysteine | 5 | ec:2.5.1.47 ec:2.5.1.48 |
| cpd:C17023 |  | Sulfur donor; S-donor | 5 | ec:2.8.1.6 |
| cpd:C00288 |  | HCO3-; Bicarbonate; Hydrogencarbonate; Acid carbonate | 4 | ec:6.3.4.14 ec:6.4.1.2 |
| cpd:C01352 |  | FADH2 | 4 |  |
| cpd:C06250 |  | Holo-[carboxylase]; Biotin-carboxyl-carrier protein | 4 | ec:6.3.4.14 ec:6.4.1.2 |
| cpd:C00979 |  | O-Acetyl-L-serine; O3-Acetyl-L-serine | 4 | ec:2.3.1.30 ec:2.5.1.47 ec:2.5.1.49 |
| cpd:C02291 |  | L-Cystathionine | 4 | ec:2.5.1.48 |
| cpd:C00163 |  | Propanoate; Propionate; Propanoic acid; Propionic acid | 4 | ec:2.7.2.1 |
| cpd:C05460 |  | 3alpha,7alpha,12alpha-Trihydroxy-5beta-cholest-24-enoyl-CoA | 4 |  |
| cpd:C18239 |  | Precursor Z; Cyclic pyranopterin monophosphate; Cyclic pyranopterin phosphate; cPMP | 4 |  |
| cpd:C00542 |  | Cystathionine | 4 | ec:2.5.1.48 |
| cpd:C04419 |  | Carboxybiotin-carboxyl-carrier protein | 4 | ec:6.3.4.14 ec:6.4.1.2 |
| cpd:C16470 |  | 5-Methylhex-4-enoyl-CoA | 4 |  |
| cpd:C05447 |  | 3alpha,7alpha-Dihydroxy-5beta-cholest-24-enoyl-CoA | 4 |  |
| cpd:C16468 |  | (2E)-5-Methylhexa-2,4-dienoyl-CoA | 4 |  |
| cpd:C04760 |  | 3alpha,7alpha,12alpha-Trihydroxy-5beta-cholestanoyl-CoA | 4 |  |
| cpd:C03671 |  | 2-Pyrone-4,6-dicarboxylate | 4 | ec:3.1.1.57 ec:1.13.11.8 ec:1.1.1.312 |
| cpd:C06508 |  | Adenosyl cobinamide | 4 | ec:6.3.1.10  ec:2.7.7.62 ec:2.5.1.17 ec:2.7.1.156 |
| cpd:C15809 |  | Iminoglycine; Iminoacetic acid; 2-Iminoacetate | 4 | ec:1.4.3.19 |
| cpd:C00041 |  | L-Alanine; L-2-Aminopropionic acid; L-alpha-Alanine | 4 | ec:2.8.1.7 ec:2.3.1.47 |
| cpd:C01118 |  | O-Succinyl-L-homoserine; O4-Succinyl-L-homoserine; O-Succinylhomoserine | 4 | ec:2.5.1.48 |
| cpd:C05699 |  | L-Selenocystathionine | 4 | ec:2.5.1.48 |
| cpd:C04644 |  | 3alpha,7alpha-Dihydroxy-5beta-cholestanoyl-CoA | 4 |  |
| cpd:C00016 |  | FAD; Flavin adenine dinucleotide | 4 |  |
| cpd:C05702 |  | O-Phosphorylhomoserine | 4 | ec:2.5.1.48 |
| cpd:C03892 |  | Phosphatidylglycerophosphate; 3(3-sn-Phosphatidyl)-sn-glycerol 1-phosphate; 3(3-Phosphatidyl-)L-glycerol 1-phosphate; 1,2-Diacyl-sn-glycero-3-phospho-sn-glycerol 3'-phosphate | 3 | ec:3.1.3.27 ec:2.7.8.5 |
| cpd:C00295 |  | Orotate; Orotic acid; Uracil-6-carboxylic acid | 3 | ec:2.4.2.10 |
| cpd:C00320 |  | Thiosulfate; Hyposulfite | 3 | ec:2.5.1.47 ec:2.5.1.49 |
| cpd:C20237 |  | alpha-Maltose 1-phosphate | 3 | ec:2.4.99.16 |
| cpd:C00217 |  | D-Glutamate; D-Glutamic acid; D-Glutaminic acid; D-2-Aminoglutaric acid | 3 | ec:3.5.1.2 ec:5.1.1.3 |
| cpd:C19673 |  | Malonyl-[acp] methyl ester; Malonyl-[acyl-carrier protein] methyl ester | 3 | ec:2.1.1.197 |
| cpd:C04411 |  | (2R,3S)-3-Isopropylmalate; 3-Isopropylmalate; 3-Carboxy-2-hydroxy-4-methylpentanoate; 2-D-threo-Hydroxy-3-carboxy-isocaproate | 3 | ec:1.1.1.85 ec:4.2.1.33 ec:4.2.1.35 |
| cpd:C01209 |  | Malonyl-[acyl-carrier protein]; Malonyl-[acp] | 3 | ec:2.1.1.197 |
| cpd:C15815 |  | C15815; Thiamine biosynthesis intermediate 6 | 3 |  |
| cpd:C00088 |  | Nitrite | 3 | ec:1.13.12.16 |
| cpd:C06509 |  | Adenosyl cobinamide phosphate | 3 | ec:6.3.1.10  ec:2.7.7.62 ec:2.7.1.156 |
| cpd:C00119 |  | 5-Phospho-alpha-D-ribose 1-diphosphate; 5-Phosphoribosyl diphosphate; 5-Phosphoribosyl 1-pyrophosphate; PRPP | 3 | ec:2.4.2.10 |
| cpd:C00112 |  | CDP; Cytidine 5'-diphosphate; Cytidine diphosphate | 3 | ec:1.17.4.1 ec:2.7.1.11 |
| cpd:C00103 |  | D-Glucose 1-phosphate; alpha-D-Glucose 1-phosphate; Cori ester; D-Glucose alpha-1-phosphate | 3 | ec:2.4.1.1 |
| cpd:C00100 |  | Propanoyl-CoA; Propionyl-CoA; Propionyl coenzyme A | 3 | ec:2.3.1.8 |
| cpd:C00065 |  | L-Serine; L-2-Amino-3-hydroxypropionic acid; L-3-Hydroxy-alanine; Serine | 3 | ec:2.3.1.30 ec:6.1.1.11 ec:4.2.1.20 |
| cpd:C00063 |  | CTP; Cytidine 5'-triphosphate; Cytidine triphosphate | 3 | ec:2.7.7.74 ec:2.7.8.34 ec:2.7.7.72 ec:2.7.1.11 |
| cpd:C00061 |  | FMN; Riboflavin-5-phosphate; Flavin mononucleotide | 3 | ec:1.13.12.16 |
| cpd:C04324 |  | (1E,3E)-4-Hydroxybuta-1,3-diene-1,2,4-tricarboxylate; 2-Hydroxy-4-carboxyhexa-2,4-dienedioate; 4-Carboxy-2-hydroxy-cis,cis-muconate; 4-Carboxy-2-hydroxyhexa-2,4-cis,cis-dienedioate | 3 | ec:5.3.2.8 ec:1.13.11.8 |
| cpd:C00055 |  | CMP; Cytidine-5'-monophosphate; Cytidylic acid | 3 | ec:2.7.7.74 ec:2.7.8.34 ec:2.7.8.5 |
| cpd:C01092 |  | 8-Amino-7-oxononanoate; 8-Amino-7-oxononanoic acid | 3 | ec:2.3.1.47 |
| cpd:C00846 |  | 3-Oxoadipate; 3-Oxoadipic acid; 3-Keto-adipate | 3 | ec:3.1.1.24 |
| cpd:C00049 |  | L-Aspartate; L-Aspartic acid; 2-Aminosuccinic acid; L-Asp | 3 | ec:6.1.1.12 ec:3.5.1.1 ec:2.1.3.2 |
| cpd:C06032 |  | D-erythro-3-Methylmalate; (2R,3S)-3-Methylmalate | 3 | ec:1.1.1.85 ec:4.2.1.33 ec:4.2.1.35 |
| cpd:C18091 |  | Ethylnitronate | 3 | ec:1.13.12.16 |
| cpd:C00037 |  | Glycine; Aminoacetic acid; Gly | 3 | ec:1.4.3.19 ec:1.8.1.4 |
| cpd:C00035 |  | GDP; Guanosine 5'-diphosphate; Guanosine diphosphate | 3 | ec:1.17.4.1 ec:2.7.7.62 ec:2.7.1.156 |
| cpd:C03586 |  | 2-Oxo-2,3-dihydrofuran-5-acetate; 3-Oxoadipate enol-lactone; 4,5-Dihydro-5-oxofuran-2-acetate; 5-Oxo-4,5-dihydrofuran-2-acetate | 3 | ec:3.1.1.24 |
| cpd:C01103 |  | Orotidine 5'-phosphate; Orotidylic acid | 3 | ec:2.4.2.10 |
| cpd:C01063 |  | 6-Carboxyhexanoyl-CoA; Pimeloyl-CoA | 3 | ec:2.3.1.47 |
| cpd:C00015 |  | UDP; Uridine 5'-diphosphate | 3 | ec:1.17.4.1 ec:2.7.1.11 |
| cpd:C01847 |  | Reduced FMN; FMNH2 | 3 | ec:1.13.12.16 |
| cpd:C01037 |  | 7,8-Diaminononanoate | 3 | ec:6.3.3.3 |
| cpd:C02051 |  | Lipoylprotein; H-Protein-lipoyllysine | 2 | ec:1.8.1.4 |
| cpd:C00721 |  | Dextrin | 2 | ec:3.2.1.1 ec:5.4.99.16 |
| cpd:C15973 |  | Enzyme N6-(dihydrolipoyl)lysine; Dihydrolipoamide-E | 2 | ec:1.8.1.4 |
| cpd:C15972 |  | Enzyme N6-(lipoyl)lysine; Lipoamide-E | 2 | ec:1.8.1.4 |
| cpd:C05984 |  | 2-Hydroxybutanoic acid; 2-Hydroxybutyrate; 2-Hydroxybutyric acid | 2 | ec:1.1.1.27 |
| cpd:C00673 |  | 2-Deoxy-D-ribose 5-phosphate | 2 | ec:2.7.1.15 ec:4.1.2.4 |
| cpd:C05223 |  | Dodecanoyl-[acyl-carrier protein]; Dodecanoyl-[acp]; Lauroyl-[acyl-carrier protein] | 2 | ec:1.3.1.10 ec:1.3.1.9 |
| cpd:C00705 |  | dCDP; 2'-Deoxycytidine diphosphate; 2'-Deoxycytidine 5'-diphosphate | 2 | ec:1.17.4.1 |
| cpd:C05616 |  | 3-O-Methylgallate; 3-O-Methylgallic acid | 2 | ec:1.13.11.8 |
| cpd:C01346 |  | dUDP; 2'-Deoxyuridine 5'-diphosphate | 2 | ec:1.17.4.1 |
| cpd:C00269 |  | CDP-diacylglycerol; CDP-1,2-diacylglycerol; 1,2-Diacyl-sn-glycero-3-cytidine-5'-diphosphate | 2 | ec:2.7.8.5 |
| cpd:C04494 |  | Guanosine 3'-diphosphate 5'-triphosphate; Guanosine 5'-triphosphate,3'-diphosphate | 2 | ec:2.7.6.5 |
| cpd:C04484 |  | 4-Carboxy-2-hydroxymuconate semialdehyde; 4-Carboxy-2-hydroxy-cis,cis-muconate 6-semialdehyde | 2 | ec:1.13.11.8 |
| cpd:C16221 |  | (2E)-Octadecenoyl-[acp]; trans-Octadec-2-enoyl-[acp] | 2 | ec:1.3.1.10 ec:1.3.1.9 |
| cpd:C04122 |  | D-1-Aminopropan-2-ol O-phosphate; (R)-1-Aminopropan-2-yl phosphate | 2 | ec:6.3.1.10 |
| cpd:C04088 |  | Octadecanoyl-[acyl-carrier protein]; Stearoyl-[acyl-carrier protein] | 2 | ec:1.3.1.10 ec:1.3.1.9 |
| cpd:C00248 |  | Lipoamide; Thioctic acid amide | 2 | ec:1.8.1.4 |
| cpd:C00245 |  | Taurine; 2-Aminoethanesulfonic acid; Aminoethylsulfonic acid | 2 | ec:2.3.2.2 ec:1.14.11.17 |
| cpd:C04115 |  | 4-Carboxy-4-hydroxy-2-oxoadipate; 4-Hydroxy-4-carboxymethyl-2-oxoglutarate; 2-Hydroxy-4-oxobutane-1,2,4-tricarboxylate | 2 | ec:4.1.3.17 ec:4.2.1.83 |
| cpd:C00230 |  | 3,4-Dihydroxybenzoate; 3,4-Dihydroxybenzoic acid; Protocatechuate; Protocatechuic acid | 2 | ec:1.13.11.8 |
| cpd:C01267 |  | 3-(Imidazol-4-yl)-2-oxopropyl phosphate; Imidazole-acetol phosphate | 2 | ec:2.6.1.9 |
| cpd:C00186 |  | (S)-Lactate; L-Lactate; L-Lactic acid | 2 | ec:1.1.1.27 |
| cpd:C00579 |  | Dihydrolipoamide; Dihydrothioctamide | 2 | ec:1.8.1.4 |
| cpd:C00575 |  | 3',5'-Cyclic AMP; Cyclic adenylic acid; Cyclic AMP; Adenosine 3',5'-phosphate; Adenosine 3',5'-cyclic phosphate; cAMP | 2 | ec:4.6.1.1 |
| cpd:C01251 |  | (R)-2-Hydroxybutane-1,2,4-tricarboxylate; Homocitrate; Homocitric acid; 3-Hydroxy-3-carboxyadipic acid; (R)-2-Hydroxy-1,2,4-butanetricarboxylic acid | 2 | ec:2.3.3.14 |
| cpd:C01641 |  | tRNA(Glu) | 2 | ec:6.1.1.24 ec:6.1.1.17 |
| cpd:C00208 |  | Maltose; Malt sugar; alpha-D-Glucopyranosyl-(1->4)-D-glucopyranose | 2 | ec:3.2.1.1 ec:5.4.99.16 |
| cpd:C00206 |  | dADP; 2'-Deoxyadenosine 5'-diphosphate | 2 | ec:1.17.4.1 |
| cpd:C00166 |  | Phenylpyruvate; Phenylpyruvic acid; alpha-Ketohydrocinnamic acid; keto-Phenylpyruvate; 3-Phenyl-2-oxopropanoate; 2-Oxo-3-phenylpropanoate | 2 | ec:2.6.1.9 |
| cpd:C00957 |  | Mercaptopyruvate; 3-Mercaptopyruvic acid; 3-Mercaptopyruvate | 2 | ec:1.1.1.27 |
| cpd:C00942 |  | 3',5'-Cyclic GMP; Guanosine 3',5'-cyclic monophosphate; Guanosine 3',5'-cyclic phosphate; Cyclic GMP; cGMP | 2 | ec:4.6.1.1 |
| cpd:C00143 |  | 5,10-Methylenetetrahydrofolate; (6R)-5,10-Methylenetetrahydrofolate; 5,10-Methylene-THF | 2 | ec:1.8.1.4 |
| cpd:C01213 |  | (R)-Methylmalonyl-CoA; L-Methylmalonyl-CoA | 2 | ec:5.4.99.2 |
| cpd:C01179 |  | 3-(4-Hydroxyphenyl)pyruvate; 4-Hydroxyphenylpyruvate; p-Hydroxyphenylpyruvic acid | 2 | ec:2.6.1.9 |
| cpd:C04006 |  | 1D-myo-Inositol 3-phosphate; D-myo-Inositol 3-phosphate; myo-Inositol 3-phosphate; Inositol 3-phosphate; 1D-myo-Inositol 3-monophosphate; D-myo-Inositol 3-monophosphate; myo-Inositol 3-monophosphate; Inositol 3-monophosphate; 1L-myo-Inositol 1-phosphate; L-myo-Inositol 1-phosphate | 2 | ec:2.7.7.74 ec:2.7.8.34 ec:5.5.1.4 |
| cpd:C00093 |  | sn-Glycerol 3-phosphate; Glycerophosphoric acid; D-Glycerol 1-phosphate | 2 | ec:2.7.8.5 |
| cpd:C03319 |  | dTDP-L-rhamnose; dTDP-6-deoxy-L-mannose; dTDP-6-deoxy-beta-L-mannose; dTDP-beta-L-rhamnose | 2 | ec:5.1.3.25 |
| cpd:C02631 |  | 2-Isopropylmaleate; beta-Isopropylmaleate | 2 | ec:4.2.1.33 ec:4.2.1.35 |
| cpd:C06507 |  | Adenosyl cobyrinate hexaamide; Adenosylcobyric acid | 2 | ec:6.3.1.10 |
| cpd:C05823 |  | 3-Mercaptolactate; L-3-Mercaptolactate; (R)-3-Mercaptolactate | 2 | ec:1.1.1.27 |
| cpd:C00118 |  | D-Glyceraldehyde 3-phosphate; (2R)-2-Hydroxy-3-(phosphonooxy)-propanal; Glyceraldehyde 3-phosphate | 2 | ec:4.2.1.20 ec:4.1.2.4 |
| cpd:C00079 |  | L-Phenylalanine; (S)-alpha-Amino-beta-phenylpropionic acid | 2 | ec:2.6.1.9 |
| cpd:C02987 |  | L-Glutamyl-tRNA(Glu) | 2 | ec:6.1.1.24 ec:6.1.1.17 |
| cpd:C02226 |  | 2-Methylmaleate; Citraconate; Citraconic acid; Methylmaleic acid | 2 | ec:4.2.1.33 ec:4.2.1.35 |
| cpd:C00101 |  | Tetrahydrofolate; 5,6,7,8-Tetrahydrofolate; Tetrahydrofolic acid; THF; (6S)-Tetrahydrofolate; (6S)-Tetrahydrofolic acid; (6S)-THFA | 2 | ec:1.8.1.4 |
| cpd:C00068 |  | Thiamin diphosphate; Thiamine diphosphate; Thiamin pyrophosphate; TPP; ThPP | 2 | ec:2.2.1.6 ec:2.7.4.16 |
| cpd:C16399 |  | 2,4-Diamino-6-hydroxylaminotoluene | 2 | ec:1.12.99.6 |
| cpd:C16396 |  | 2,4-Diamino-6-nitrotoluene | 2 | ec:1.12.99.6 |
| cpd:C02972 |  | Dihydrolipoylprotein; [H Protein]-dihydrolipoyllysine | 2 | ec:1.8.1.4 |
| cpd:C02612 |  | (R)-2-Methylmalate; (R)-2-Methylmalic acid; (3R)-Citramalate; (3R)-Citramalic acid; (3R)-alpha-Hydroxypyrotartaric acid; D-Citramalate; D-Citramalic acid; D-alpha-Hydroxypyrotartaric acid; (2R)-2-Hydroxy-2-methylbutanedioate | 2 | ec:4.2.1.33 ec:4.2.1.35 |
| cpd:C01137 |  | S-Adenosylmethioninamine; (5-Deoxy-5-adenosyl)(3-aminopropyl)methylsulfonium; (5-Deoxy-5-adenosyl)(3-aminopropyl)methylsulfonium cation; S-Adenosyl-(5')-3-methylthiopropylamine; S-Adenosyl 3-(methylthio)propylamine | 2 | ec:2.5.1.16 ec:4.1.1.50 |
| cpd:C05764 |  | Hexadecanoyl-[acp]; Hexadecanoyl-[acyl-carrier protein] | 2 | ec:1.3.1.10 ec:1.3.1.9 |
| cpd:C05763 |  | trans-Hexadec-2-enoyl-[acp]; trans-Hexadec-2-enoyl-[acyl-carrier protein]; (2E)-Hexadecenoyl-[acp] | 2 | ec:1.3.1.10 ec:1.3.1.9 |
| cpd:C05761 |  | Tetradecanoyl-[acp]; Tetradecanoyl-[acyl-carrier protein]; Myristoyl-[acyl-carrier protein] | 2 | ec:1.3.1.10 ec:1.3.1.9 |
| cpd:C05760 |  | trans-Tetradec-2-enoyl-[acp]; trans-Tetradec-2-enoyl-[acyl-carrier protein]; (2E)-Tetradecenoyl-[acp] | 2 | ec:1.3.1.10 ec:1.3.1.9 |
| cpd:C01528 |  | Hydrogen selenide; Selenide | 2 | ec:2.5.1.47 ec:1.8.1.9 |
| cpd:C20378 |  | Enoylpimeloyl-[acp] methyl ester; Enoylpimeloyl-[acyl-carrier protein] methyl ester | 2 | ec:1.3.1.10 ec:1.3.1.9 |
| cpd:C05758 |  | trans-Dodec-2-enoyl-[acp]; trans-Dodec-2-enoyl-[acyl-carrier protein]; (2E)-Dodecenoyl-[acp] | 2 | ec:1.3.1.10 ec:1.3.1.9 |
| cpd:C20375 |  | Glutaryl-[acp] methyl ester; Glutaryl-[acyl-carrier protein] methyl ester | 2 | ec:1.3.1.10 ec:1.3.1.9 |
| cpd:C20374 |  | Enoylglutaryl-[acp] methyl ester; Enoylglutaryl-[acyl-carrier protein] methyl ester | 2 | ec:1.3.1.10 ec:1.3.1.9 |
| cpd:C05755 |  | Decanoyl-[acp]; Decanoyl-[acyl-carrier protein] | 2 | ec:1.3.1.10 ec:1.3.1.9 |
| cpd:C05754 |  | trans-Dec-2-enoyl-[acp]; trans-Dec-2-enoyl-[acyl-carrier protein]; trans-2-Decenoyl-[acyl-carrier protein]; (2E)-Decenoyl-[acp] | 2 | ec:1.3.1.10 ec:1.3.1.9 |
| cpd:C05752 |  | Octanoyl-[acp]; Octanoyl-[acyl-carrier protein] | 2 | ec:1.3.1.10 ec:1.3.1.9 |
| cpd:C05751 |  | trans-Oct-2-enoyl-[acp]; trans-Oct-2-enoyl-[acyl-carrier protein]; Oct-2-enoyl-[acyl-carrier protein]; 2-Octenoyl-[acyl-carrier protein]; (2E)-Octenoyl-[acp] | 2 | ec:1.3.1.10 ec:1.3.1.9 |
| cpd:C03599 |  | CDP-3,6-dideoxy-D-mannose; CDPtyvelose | 2 | ec:5.1.3.10 |
| cpd:C03598 |  | CDP-3,6-dideoxy-D-glucose; CDPparatose; CDP-alpha-D-paratose | 2 | ec:5.1.3.10 |
| cpd:C05749 |  | Hexanoyl-[acp]; Hexanoyl-[acyl-carrier protein] | 2 | ec:1.3.1.10 ec:1.3.1.9 |
| cpd:C05748 |  | trans-Hex-2-enoyl-[acp]; trans-Hex-2-enoyl-[acyl-carrier protein]; (2E)-Hexenoyl-[acp] | 2 | ec:1.3.1.10 ec:1.3.1.9 |
| cpd:C03194 |  | (R)-1-Aminopropan-2-ol; (R)-1-Amino-2-propanol | 2 | ec:6.3.1.10 |
| cpd:C05745 |  | Butyryl-[acp]; Butyryl-[acyl-carrier protein]; Butanoyl-[acp] | 2 | ec:1.3.1.10 ec:1.3.1.9 |
| cpd:C00036 |  | Oxaloacetate; Oxalacetic acid; Oxaloacetic acid; 2-Oxobutanedioic acid; 2-Oxosuccinic acid; keto-Oxaloacetate | 2 | ec:4.1.3.17 ec:2.3.3.1 |
| cpd:C03187 |  | dTDP-6-deoxy-beta-L-talose; dTDP-beta-L-pneumose | 2 | ec:5.1.3.25 |
| cpd:C01100 |  | L-Histidinol phosphate | 2 | ec:2.6.1.9 |
| cpd:C00819 |  | D-Glutamine; D-2-Aminoglutaramic acid | 2 | ec:3.5.1.2 |
| cpd:C05682 |  | Phosphonoacetate; Phosphonoacetic acid; Fosfonet | 2 | ec:3.11.1.2 |
| cpd:C04246 |  | But-2-enoyl-[acyl-carrier protein] | 2 | ec:1.3.1.10 ec:1.3.1.9 |
| cpd:C00361 |  | dGDP; 2'-Deoxyguanosine 5'-diphosphate | 2 | ec:1.17.4.1 |
| cpd:C02504 |  | alpha-Isopropylmalate; (2S)-2-Isopropylmalate; (2S)-2-Hydroxy-2-isopropylsuccinic acid; 2-Isopropylmalic acid; 3-Carboxy-3-hydroxy-4-methylpentanoate; 3-Carboxy-3-hydroxyisocaproate | 2 | ec:4.2.1.33 ec:4.2.1.35 |
| cpd:C20679 |  | Tungstate; Tungstic acid | 2 | ec:3.6.3.55 |
| cpd:C01424 |  | Gallate; Gallic acid; 3,4,5-Trihydroxybenzoic acid; 3,4,5-Trihydroxybenzoate; Pyrogallol-5-carboxylic acid | 2 | ec:1.13.11.8 |
| cpd:C00344 |  | Phosphatidylglycerol; 3-(3-sn-Phosphatidyl)glycerol; 3(3-Phosphatidyl-)glycerol; PtdGro | 1 | ec:3.1.3.27 |
| cpd:C15996 |  | 7-Cyano-7-carbaguanine; 7-Cyano-7-deazaguanine | 1 | ec:1.7.1.13 |
| cpd:C16675 |  | 7-Aminomethyl-7-carbaguanine; 7-Aminomethyl-7-deazaguanine | 1 | ec:1.7.1.13 |
| cpd:C01419 |  | Cys-Gly; L-Cysteinylglycine | 1 | ec:2.3.2.2 |
| cpd:C01412 |  | Butanal; Butyraldehyde | 1 |  |
| cpd:C00334 |  | 4-Aminobutanoate; 4-Aminobutanoic acid; 4-Aminobutyrate; 4-Aminobutyric acid; gamma-Aminobutyric acid; GABA | 1 | ec:1.2.1.3 |
| cpd:C01801 |  | Deoxyribose; 2-Deoxy-D-erythro-pentose; Thyminose; 2-Deoxy-D-ribose | 1 | ec:2.7.1.15 |
| cpd:C01368 |  | 3'-UMP; Uridine 3'-monophosphate; Uridine 3'-phosphate | 1 | ec:3.1.4.16 |
| cpd:C01367 |  | 3'-AMP; 3'-Adenylic acid; 3'-Adenosine monophosphate; Adenosine-3'-monophosphate; Adenosine 3'-phosphate; AMP 3'-phosphate | 1 | ec:3.1.4.16 |
| cpd:C05635 |  | 5-Hydroxyindoleacetate | 1 | ec:1.2.1.3 |
| cpd:C05634 |  | 5-Hydroxyindoleacetaldehyde | 1 | ec:1.2.1.3 |
| cpd:C01001 |  | Formylmethanofuran | 1 | ec:1.2.99.5 |
| cpd:C10700 |  | 4'-Hydroxyacetophenone; (4-Hydroxyphenyl)ethan-1-one | 1 |  |
| cpd:C04554 |  | 3alpha,7alpha-Dihydroxy-5beta-cholestanate; 3alpha,7alpha-Dihydroxy-5beta-cholestanoate | 1 | ec:1.2.1.3 |
| cpd:C02835 |  | Imidazole-4-acetate; Imidazoleacetic acid; 4-Imidazoleacetate | 1 | ec:1.2.1.3 |
| cpd:C04153 |  | rRNA containing N2-methylguanine | 1 | ec:2.1.1.171 |
| cpd:C02430 |  | L-Methionyl-tRNA; L-Methionyl-tRNA(Met) | 1 | ec:6.1.1.10 |
| cpd:C05985 |  | 2-Propynal; 2-Propyn-1-al; Propiolaldehyde | 1 | ec:1.2.1.3 |
| cpd:C00315 |  | Spermidine; N-(3-Aminopropyl)-1,4-butane-diamine | 1 | ec:2.5.1.16 |
| cpd:C00671 |  | (S)-3-Methyl-2-oxopentanoic acid; (S)-3-Methyl-2-oxopentanoate; (3S)-3-Methyl-2-oxopentanoic acid; (3S)-3-Methyl-2-oxopentanoate | 1 | ec:4.2.1.9 |
| cpd:C03506 |  | Indoleglycerol phosphate; 1-C-(Indol-3-yl)glycerol 3-phosphate; (3-Indolyl)-glycerol phosphate; C1-(3-Indolyl)-glycerol 3-phosphate; (1S,2R)-1-C-(Indol-3-yl)glycerol 3-phosphate; Indole-3-glycerol phosphate | 1 | ec:4.2.1.20 |
| cpd:C19799 |  | Bis(1L-myo-inositol)-3,1'-phosphate 1-phosphate | 1 | ec:2.7.7.74 ec:2.7.8.34 |
| cpd:C19794 |  | CDP-1L-myo-inositol | 1 | ec:2.7.7.74 ec:2.7.8.34 |
| cpd:C00701 |  | Base; Nucleobase; Base1; Base2 | 1 | ec:2.4.2.6 |
| cpd:C16636 |  | tRNA(Sec) | 1 | ec:6.1.1.11 |
| cpd:C04895 |  | 7,8-Dihydroneopterin 3'-triphosphate; 2-Amino-4-hydroxy-6-(erythro-1,2,3-trihydroxypropyl)dihydropteridine triphosphate; 6-(L-erythro-1,2-Dihydroxypropyl 3-triphosphate)-7,8-dihydropterin; 6-[(1S,2R)-1,2-Dihydroxy-3-triphosphooxypropyl]-7,8-dihydropterin | 1 | ec:3.5.4.16 |
| cpd:C04133 |  | N-Acetyl-L-glutamate 5-phosphate; N-Acetyl-L-glutamyl 5-phosphate | 1 | ec:1.2.1.38 |
| cpd:C00258 |  | D-Glycerate; Glycerate; (R)-Glycerate; Glyceric acid | 1 | ec:1.2.1.3 |
| cpd:C05951 |  | Leukotriene D4; LTD4 | 1 | ec:2.3.2.2 |
| cpd:C00249 |  | Hexadecanoic acid; Hexadecanoate; Hexadecylic acid; Palmitic acid; Palmitate; Cetylic acid | 1 | ec:6.2.1.3 |
| cpd:C00240 |  | rRNA; Ribosomal RNA | 1 | ec:2.1.1.171 |
| cpd:C06194 |  | 2',3'-Cyclic GMP | 1 | ec:3.1.4.16 |
| cpd:C06193 |  | Guanosine 3'-phosphate; 3'-GMP; 3'-Guanylic acid; Guo-3'-P; Gp | 1 | ec:3.1.4.16 |
| cpd:C03794 |  | N6-(1,2-Dicarboxyethyl)-AMP; N6-(1,2-Dicarboxyethyl)AMP; Adenylosuccinate; Adenylosuccinic acid | 1 | ec:4.3.2.2 |
| cpd:C02355 |  | 2',3'-Cyclic UMP | 1 | ec:3.1.4.16 |
| cpd:C00637 |  | Indole-3-acetaldehyde; 2-(Indol-3-yl)acetaldehyde; Indoleacetaldehyde | 1 | ec:1.2.1.3 |
| cpd:C02354 |  | 2',3'-Cyclic CMP | 1 | ec:3.1.4.16 |
| cpd:C02353 |  | 2',3'-Cyclic AMP | 1 | ec:3.1.4.16 |
| cpd:C00993 |  | D-Alanyl-D-alanine; D-Ala-D-Ala | 1 | ec:6.3.2.4 |
| cpd:C01672 |  | Cadaverine; 1,5-Pentanediamine; 1,5-Diaminopentane; Pentamethylenediamine | 1 | ec:2.5.1.16 |
| cpd:C00233 |  | 4-Methyl-2-oxopentanoate; 2-Oxoisocaproate | 1 | ec:1.1.1.85 |
| cpd:C16565 |  | Aminopropylcadaverine | 1 | ec:2.5.1.16 |
| cpd:C03785 |  | D-Tagatose 1,6-bisphosphate | 1 | ec:2.7.1.11 |
| cpd:C05936 |  | N4-Acetylaminobutanal; 4-Acetamidobutanal | 1 | ec:1.2.1.3 |
| cpd:C06615 |  | cis-3-Chloroacrylic acid | 1 | ec:1.2.1.3 |
| cpd:C06614 |  | trans-3-Chloroacrylic acid | 1 | ec:1.2.1.3 |
| cpd:C06613 |  | trans-3-Chloroallyl aldehyde; trans-3-Chloro-2-propenal | 1 | ec:1.2.1.3 |
| cpd:C00224 |  | Adenylyl sulfate; Adenosine 5'-phosphosulfate; APS; 5'-Adenylyl sulfate | 1 | ec:3.1.3.7 |
| cpd:C00582 |  | Phenylacetyl-CoA; Phenylacetyl coenzyme A | 1 | ec:6.2.1.30 |
| cpd:C00189 |  | Ethanolamine; Aminoethanol; 2-Hydroxyethylamine | 1 | ec:4.3.1.7 |
| cpd:C00183 |  | L-Valine; 2-Amino-3-methylbutyric acid | 1 | ec:6.1.1.9 |
| cpd:C05130 |  | Imidazole-4-acetaldehyde; Imidazole acetaldehyde | 1 | ec:1.2.1.3 |
| cpd:C05923 |  | 2,5-Diaminopyrimidine nucleoside triphosphate | 1 | ec:3.5.4.16 |
| cpd:C01653 |  | tRNA(Val) | 1 | ec:6.1.1.9 |
| cpd:C05922 |  | Formamidopyrimidine nucleoside triphosphate | 1 | ec:3.5.4.16 |
| cpd:C01650 |  | tRNA(Ser) | 1 | ec:6.1.1.11 |
| cpd:C00577 |  | D-Glyceraldehyde | 1 | ec:1.2.1.3 |
| cpd:C00214 |  | Thymidine; Deoxythymidine | 1 | ec:2.4.2.6 |
| cpd:C01250 |  | N-Acetyl-L-glutamate 5-semialdehyde; 2-Acetamido-5-oxopentanoate | 1 | ec:1.2.1.38 |
| cpd:C00178 |  | Thymine; 5-Methyluracil | 1 | ec:2.4.2.6 |
| cpd:C05125 |  | 2-(alpha-Hydroxyethyl)thiamine diphosphate; 2-Hydroxyethyl-ThPP | 1 | ec:2.2.1.6 |
| cpd:C00170 |  | 5'-Methylthioadenosine; Methylthioadenosine; S-Methyl-5'-thioadenosine; 5-Methylthioadenosine; 5'-Deoxy-5'-(methylthio)adenosine; Thiomethyladenosine; MTA | 1 | ec:2.5.1.16 |
| cpd:C01647 |  | tRNA(Met) | 1 | ec:6.1.1.10 |
| cpd:C03363 |  | 5-L-Glutamyl amino acid; L-gamma-Glutamyl amino acid | 1 | ec:2.3.2.2 |
| cpd:C01640 |  | tRNA(Gln) | 1 | ec:6.1.1.24 |
| cpd:C01241 |  | Phosphatidyl-N-methylethanolamine | 1 | ec:2.1.1.17 ec:2.1.1.71 |
| cpd:C00169 |  | Carbamoyl phosphate | 1 | ec:2.1.3.2 |
| cpd:C14818 |  | Fe2+; Fe(II); Ferrous ion; Iron(2+) | 1 | ec:4.99.1.4 ec:1.3.1.76 ec:2.1.1.107 |
| cpd:C00162 |  | Fatty acid | 1 | ec:1.2.1.3 |
| cpd:C04039 |  | 2,3-Dihydroxy-3-methylbutanoate; 2,3-Dihydroxy-isovalerate; 2,3-Dihydroxy-isovaleric acid | 1 | ec:4.2.1.9 |
| cpd:C01638 |  | tRNA(Asp) | 1 | ec:6.1.1.12 |
| cpd:C00954 |  | Indole-3-acetate; Indole-3-acetic acid; (Indol-3-yl)acetate; Indoleacetate; Indoleacetic acid; IAA | 1 | ec:1.2.1.3 |
| cpd:C02670 |  | D-Glucuronolactone; Glucurone; D-Glucurono-3,6-lactone; D-Glucurone | 1 | ec:1.2.1.3 |
| cpd:C06548 |  | Ethylene oxide | 1 |  |
| cpd:C00556 |  | Benzyl alcohol; alpha-Hydroxytoluene; Benzenemethanol; Phenylmethanol; Phenylcarbinol; Hydroxymethylbenzene | 1 |  |
| cpd:C00555 |  | 4-Aminobutyraldehyde; 4-Aminobutanal | 1 | ec:1.2.1.3 |
| cpd:C04823 |  | 1-(5'-Phosphoribosyl)-5-amino-4-(N-succinocarboxamide)-imidazole; 1-(5'-Phosphoribosyl)-4-(N-succinocarboxamide)-5-aminoimidazole; 5'-Phosphoribosyl-4-(N-succinocarboxamide)-5-aminoimidazole; (S)-2-[5-Amino-1-(5-phospho-D-ribosyl)imidazole-4-carboxamido]succinate; SAICAR | 1 | ec:4.3.2.2 |
| cpd:C06148 |  | 2,5-Diamino-6-(5'-triphosphoryl-3',4'-trihydroxy-2'-oxopentyl)-amino-4-oxopyrimidine | 1 | ec:3.5.4.16 |
| cpd:C00158 |  | Citrate; Citric acid; 2-Hydroxy-1,2,3-propanetricarboxylic acid; 2-Hydroxytricarballylic acid | 1 | ec:2.3.3.1 |
| cpd:C00157 |  | Phosphatidylcholine; Lecithin; Phosphatidyl-N-trimethylethanolamine; 1,2-Diacyl-sn-glycero-3-phosphocholine; Choline phosphatide; 3-sn-Phosphatidylcholine | 1 | ec:2.1.1.17 ec:2.1.1.71 |
| cpd:C06142 |  | 1-Butanol; n-Butanol | 1 |  |
| cpd:C00154 |  | Palmitoyl-CoA; Hexadecanoyl-CoA | 1 | ec:6.2.1.3 |
| cpd:C00152 |  | L-Asparagine; 2-Aminosuccinamic acid | 1 | ec:3.5.1.1 |
| cpd:C00151 |  | L-Amino acid; L-2-Amino acid | 1 | ec:2.3.2.2 |
| cpd:C03740 |  | (5-L-Glutamyl)-L-amino acid; L-gamma-Glutamyl-L-amino acid | 1 | ec:2.3.2.2 |
| cpd:C14765 |  | 13-OxoODE; 13-KODE; (9Z,11E)-13-Oxooctadeca-9,11-dienoic acid | 1 |  |
| cpd:C14762 |  | 13(S)-HODE; (13S)-Hydroxyoctadecadienoic acid; (9Z,11E)-(13S)-13-Hydroxyoctadeca-9,11-dienoic acid | 1 |  |
| cpd:C00944 |  | 3-Dehydroquinate; 3-Dehydroquinic acid; 5-Dehydroquinate; 5-Dehydroquinic acid | 1 | ec:4.2.3.4 |
| cpd:C02269 |  | Deoxynucleoside; 2'-Deoxynucleoside; 2-Deoxy-D-ribosyl-base; 2-Deoxy-D-ribosyl-base1; 2-Deoxy-D-ribosyl-base2 | 1 | ec:2.4.2.6 |
| cpd:C01181 |  | 4-Trimethylammoniobutanoate; Butyro-betaine; gamma-Butyrobetaine | 1 | ec:1.2.1.3 |
| cpd:C00141 |  | 3-Methyl-2-oxobutanoic acid; 3-Methyl-2-oxobutyric acid; 3-Methyl-2-oxobutanoate; 2-Oxo-3-methylbutanoate; 2-Oxoisovalerate; 2-Oxoisopentanoate; alpha-Ketovaline; 2-Ketovaline; 2-Keto-3-methylbutyric acid | 1 | ec:4.2.1.9 |
| cpd:C00931 |  | Porphobilinogen | 1 | ec:4.2.1.24 |
| cpd:C05844 |  | 5-L-Glutamyl-taurine; 5-Glutamyl-taurine; Glutaurine | 1 | ec:2.3.2.2 |
| cpd:C00536 |  | Triphosphate; Triphosphoric acid; Tripolyphosphate; Inorganic triphosphate | 1 | ec:2.5.1.17 |
| cpd:C05840 |  | Iminoaspartate; Iminoaspartic acid; Iminosuccinate | 1 | ec:2.5.1.72 |
| cpd:C05445 |  | 3alpha,7alpha-Dihydroxy-5beta-cholestan-26-al | 1 | ec:1.2.1.3 |
| cpd:C06481 |  | L-Seryl-tRNA(Sec) | 1 | ec:6.1.1.11 |
| cpd:C00134 |  | Putrescine; 1,4-Butanediamine; 1,4-Diaminobutane; Tetramethylenediamine; Butane-1,4-diamine | 1 | ec:2.5.1.16 |
| cpd:C00493 |  | Shikimate; Shikimic acid; 3,4,5-Trihydroxy-1-cyclohexenecarboxylic acid | 1 | ec:2.7.1.71 |
| cpd:C00133 |  | D-Alanine; D-2-Aminopropionic acid; D-Ala | 1 | ec:6.3.2.4 |
| cpd:C03722 |  | Quinolinate; Pyridine-2,3-dicarboxylate; Quinolinic acid; 2,3-Pyridinedicarboxylic acid | 1 | ec:2.5.1.72 |
| cpd:C00099 |  | beta-Alanine; 3-Aminopropionic acid; 3-Aminopropanoate | 1 | ec:1.2.1.3 |
| cpd:C00092 |  | D-Glucose 6-phosphate; Glucose 6-phosphate; Robison ester | 1 | ec:5.5.1.4 |
| cpd:C06510 |  | Adenosine-GDP-cobinamide; Adenosylcobinamide-GDP | 1 | ec:2.7.7.62 ec:2.7.1.156 |
| cpd:C00882 |  | Dephospho-CoA; Dephosphocoenzyme A; 3'-Dephospho-CoA | 1 | ec:2.7.7.3 |
| cpd:C06114 |  | gamma-Glutamyl-beta-aminopropiononitrile; gamma-Glutamyl-3-aminopropiononitrile | 1 | ec:2.3.2.2 |
| cpd:C06112 |  | L-Glutamyl-tRNA(Gln); Glu-tRNA(Gln); Glutamyl-tRNA(Gln) | 1 | ec:6.1.1.24 |
| cpd:C15812 |  | [Enzyme]-S-sulfanylcysteine; Thiamine biosynthesis intermediate 3 | 1 | ec:2.8.1.7 |
| cpd:C15811 |  | [Enzyme]-cysteine; Thiamine biosynthesis intermediate 2 | 1 | ec:2.8.1.7 |
| cpd:C00122 |  | Fumarate; Fumaric acid; trans-Butenedioic acid | 1 | ec:4.3.2.2 |
| cpd:C00121 |  | D-Ribose | 1 | ec:2.7.1.15 |
| cpd:C11145 |  | Methanesulfonic acid; Methanesulfonate | 1 |  |
| cpd:C00081 |  | ITP; Inosine 5'-triphosphate; Inosine triphosphate; Inosine tripolyphosphate | 1 | ec:2.7.1.11 |
| cpd:C06506 |  | Adenosyl cobyrinate a,c diamide; Adenosyl cobyrinate diamide; Adenosylcob(III)yrinic acid a,c-diamide; Adenosylcobyrinic acid a,c-diamide | 1 | ec:2.5.1.17 |
| cpd:C06505 |  | Cob(I)yrinate a,c diamide; Cob(I)yrinate diamide; Cob(I)yrinic acid a,c-diamide | 1 | ec:2.5.1.17 |
| cpd:C05822 |  | 3'-CMP; Cytidine 3'-phosphate | 1 | ec:3.1.4.16 |
| cpd:C00117 |  | D-Ribose 5-phosphate; Ribose 5-phosphate | 1 | ec:2.7.1.15 |
| cpd:C00111 |  | Glycerone phosphate; Dihydroxyacetone phosphate | 1 | ec:2.5.1.72 |
| cpd:C00078 |  | L-Tryptophan; Tryptophan; (S)-alpha-Amino-beta-(3-indolyl)-propionic acid | 1 | ec:4.2.1.20 |
| cpd:C00075 |  | UTP; Uridine 5'-triphosphate; Uridine triphosphate | 1 | ec:2.7.1.11 |
| cpd:C02984 |  | L-Aspartyl-tRNA(Asp) | 1 | ec:6.1.1.12 |
| cpd:C00071 |  | Aldehyde; RCHO | 1 | ec:1.2.1.3 |
| cpd:C00900 |  | 2-Acetolactate | 1 | ec:2.2.1.6 |
| cpd:C11924 |  | Perillic acid | 1 | ec:1.2.1.3 |
| cpd:C00862 |  | Methanofuran; Carbon dioxide reduction factor | 1 | ec:1.2.99.5 |
| cpd:C05778 |  | Sirohydrochlorin | 1 | ec:4.99.1.4 ec:1.3.1.76 ec:2.1.1.107 |
| cpd:C01149 |  | 4-Trimethylammoniobutanal | 1 | ec:1.2.1.3 |
| cpd:C05774 |  | Cobinamide; Cob(I)inamide | 1 | ec:2.5.1.17 |
| cpd:C00104 |  | IDP; Inosine 5'-diphosphate; Inosine diphosphate | 1 | ec:2.7.1.11 |
| cpd:C00463 |  | Indole; 2,3-Benzopyrrole | 1 | ec:4.2.1.20 |
| cpd:C05378 |  | beta-D-Fructose 1,6-bisphosphate | 1 | ec:2.7.1.11 |
| cpd:C05375 |  | 2-Hydroxy-2-hydropyrone-4,6-dicarboxylate | 1 | ec:1.1.1.312 |
| cpd:C00067 |  | Formaldehyde; Methanal; Oxomethane; Oxomethylene; Methylene oxide; Formalin | 1 |  |
| cpd:C04691 |  | 2-Dehydro-3-deoxy-D-arabino-heptonate 7-phosphate; 3-Deoxy-D-arabino-hept-2-ulosonate 7-phosphate; 3-Deoxy-arabino-heptulonate 7-phosphate; 3-Deoxy-D-arabino-heptulosonic acid 7-phosphate; DAHP; 2-Dahp | 1 | ec:4.2.3.4 |
| cpd:C13638 |  | 1-(4'-Hydroxyphenyl)ethanol; 4-Hydroxy-alpha-methyl-benzenemethanol; 4-(1-Hydroxyethyl)phenol | 1 |  |
| cpd:C02576 |  | Perillyl aldehyde; Perillaldehyde | 1 | ec:1.2.1.3 |
| cpd:C02211 |  | tRNA precursor | 1 | ec:2.7.7.72 |
| cpd:C01134 |  | Pantetheine 4'-phosphate; 4'-Phosphopantetheine; Phosphopantetheine; D-Pantetheine 4'-phosphate | 1 | ec:2.7.7.3 |
| cpd:C07086 |  | Phenylacetic acid; Benzylformic acid; Phenylacetate; Benzeneacetic acid | 1 | ec:6.2.1.30 |
| cpd:C02170 |  | Methylmalonate; Methylmalonic acid | 1 | ec:1.2.1.3 |
| cpd:C01097 |  | D-Tagatose 6-phosphate | 1 | ec:2.7.1.11 |
| cpd:C01096 |  | Sorbitol 6-phosphate; D-Sorbitol 6-phosphate; D-Glucitol 6-phosphate | 1 |  |
| cpd:C00053 |  | 3'-Phosphoadenylyl sulfate; 3'-Phosphoadenosine 5'-phosphosulfate; 3'-Phospho-5'-adenylyl sulfate; PAPS | 1 | ec:3.1.3.7 |
| cpd:C02166 |  | Leukotriene C4; LTC4 | 1 | ec:2.3.2.2 |
| cpd:C04677 |  | 1-(5'-Phosphoribosyl)-5-amino-4-imidazolecarboxamide; 5'-Phosphoribosyl-5-amino-4-imidazolecarboxamide; 5'-Phospho-ribosyl-5-amino-4-imidazole carboxamide; AICAR; 5-Aminoimidazole-4-carboxamide ribotide; 5-Phosphoribosyl-4-carbamoyl-5-aminoimidazole; 5-Amino-1-(5-phospho-D-ribosyl)imidazole-4-carboxamide | 1 | ec:4.3.2.2 |
| cpd:C06033 |  | Parapyruvate; 4-Hydroxy-4-methyl-2-oxoglutarate | 1 | ec:4.1.3.17 |
| cpd:C00045 |  | Amino acid; Amino acids | 1 | ec:2.3.2.2 |
| cpd:C04272 |  | (R)-2,3-Dihydroxy-3-methylbutanoate; (R)-2,3-Dihydroxy-isovalerate; (R)-2,3-Dihydroxy-isovaleric acid; (2R)-2,3-Dihydroxy-3-methylbutanoate | 1 | ec:4.2.1.9 |
| cpd:C02554 |  | L-Valyl-tRNA(Val) | 1 | ec:6.1.1.9 |
| cpd:C02553 |  | L-Seryl-tRNA(Ser) | 1 | ec:6.1.1.11 |
| cpd:C00438 |  | N-Carbamoyl-L-aspartate | 1 | ec:2.1.3.2 |
| cpd:C03193 |  | (5-L-Glutamyl)-peptide | 1 | ec:2.3.2.2 |
| cpd:C00433 |  | 2,5-Dioxopentanoate; 2-Oxoglutarate semialdehyde | 1 | ec:1.2.1.3 |
| cpd:C04308 |  | Phosphatidyl-N-dimethylethanolamine | 1 | ec:2.1.1.17 ec:2.1.1.71 |
| cpd:C00430 |  | 5-Aminolevulinate; 5-Amino-4-oxopentanoate; 5-Amino-4-oxovaleric acid | 1 | ec:4.2.1.24 |
| cpd:C05345 |  | beta-D-Fructose 6-phosphate | 1 | ec:2.7.1.11 |
| cpd:C02946 |  | 4-Acetamidobutanoate; N4-Acetylaminobutanoate | 1 | ec:1.2.1.3 |
| cpd:C00030 |  | Reduced acceptor; AH2; Hydrogen-donor; Donor | 1 | ec:1.2.99.5 |
| cpd:C01468 |  | 4-Cresol; p-Cresol; 4-Hydroxytoluene; 4-Methylphenol | 1 |  |
| cpd:C01467 |  | 3-Cresol; m-Cresol; 3-Hydroxytoluene | 1 |  |
| cpd:C05336 |  | Selenomethionyl-tRNA(Met) | 1 | ec:6.1.1.10 |
| cpd:C05695 |  | gamma-Glutamyl-Se-methylselenocysteine; 5-L-Glutamyl-Se-methylselenocysteine | 1 | ec:2.3.2.2 |
| cpd:C05335 |  | L-Selenomethionine | 1 | ec:6.1.1.10 |
| cpd:C00028 |  | Acceptor; Hydrogen-acceptor; A; Oxidized donor | 1 | ec:1.2.99.5 |
| cpd:C00027 |  | Hydrogen peroxide; H2O2; Oxydol | 1 | ec:1.4.3.19 |
| cpd:C06010 |  | (S)-2-Acetolactate; (S)-2-Hydroxy-2-methyl-3-oxobutanoate | 1 | ec:2.2.1.6 |
| cpd:C18902 |  | Methylselenic acid; Methylseleninate | 1 | ec:1.8.1.9 |
| cpd:C00818 |  | D-Glucarate; D-Glucaric acid; L-Gularic acid; D-Saccharic acid; D-Glucosaccharic acid; Glucaric acid; Glucarate | 1 | ec:1.2.1.3 |
| cpd:C05729 |  | R-S-Cysteinylglycine | 1 | ec:2.3.2.2 |
| cpd:C03175 |  | Shikimate 3-phosphate; Shikimate 5-phosphate | 1 | ec:2.7.1.71 |
| cpd:C01455 |  | Toluene; Methylbenzene; Toluol | 1 |  |
| cpd:C05689 |  | Se-Methyl-L-selenocysteine | 1 | ec:2.3.2.2 |
| cpd:C06007 |  | (R)-2,3-Dihydroxy-3-methylpentanoate; (R)-2,3-Dihydroxy-3-methylvalerate; (2R,3R)-2,3-Dihydroxy-3-methylpentanoate | 1 | ec:4.2.1.9 |
| cpd:C06006 |  | (S)-2-Aceto-2-hydroxybutanoate; (S)-2-Hydroxy-2-ethyl-3-oxobutanoate | 1 | ec:2.2.1.6 |
| cpd:C05684 |  | Selenite | 1 | ec:1.8.1.9 |
| cpd:C06002 |  | (S)-Methylmalonate semialdehyde | 1 | ec:1.2.1.3 |
| cpd:C16348 |  | cis-3-Chloroallyl aldehyde; cis-3-Chloro-2-propenal | 1 | ec:1.2.1.3 |
| cpd:C04642 |  | 2-Hydroxy-5-carboxymethylmuconate semialdehyde; 5-Carboxymethyl-2-hydroxymuconate semialdehyde; 5-Carboxymethyl-2-hydroxymuconic semialdehyde | 1 | ec:1.1.1.312 |
| cpd:C01051 |  | Uroporphyrinogen III | 1 | ec:4.99.1.4 ec:1.3.1.76 ec:2.1.1.107 |
| cpd:C00012 |  | Peptide | 1 | ec:2.3.2.2 |
| cpd:C02888 |  | Sorbose 1-phosphate; L-Sorbose 1P; L-xylo-Hexulose 1-phosphate; L-Sorbose 1-phosphate | 1 |  |
| cpd:C00804 |  | Propynoate; Propiolic acid; Acetylenecarboxylic acid; Acetylenemonocarboxylate | 1 | ec:1.2.1.3 |
| cpd:C06755 |  | Chloroacetic acid; Chloroethanoic acid | 1 | ec:1.2.1.3 |
| cpd:C06754 |  | Chloroacetaldehyde; 2-Chloroethanal | 1 | ec:1.2.1.3 |
| cpd:C05711 |  | gamma-Glutamyl-beta-cyanoalanine | 1 | ec:2.3.2.2 |
| cpd:C05670 |  | 3-Aminopropiononitrile; beta-Aminopropionitrile | 1 | ec:2.3.2.2 |
| cpd:C04236 |  | (2S)-2-Isopropyl-3-oxosuccinate; 3-Carboxy-4-methyl-2-oxopentanoate; 2-Oxo-4-methyl-3-carboxypentanoate | 1 | ec:1.1.1.85 |
| cpd:C02512 |  | 3-Cyano-L-alanine; L-3-Cyanoalanine; L-beta-Cyanoalanine | 1 | ec:2.3.2.2 |
| cpd:C05703 |  | Methaneselenol; Methylselenol | 1 | ec:1.8.1.9 |
| cpd:C00750 |  | Spermine; N,N'-Bis(3-aminopropyl)-1,4-butanediamine | 1 | ec:2.5.1.16 |
| cpd:C05665 |  | 3-Aminopropanal; beta-Aminopropion aldehyde | 1 | ec:1.2.1.3 |
| cpd:C19085 |  | tRNA with a 3' CCA end | 1 | ec:2.7.7.72 |
| cpd:C00350 |  | Phosphatidylethanolamine; (3-Phosphatidyl)ethanolamine; (3-Phosphatidyl)-ethanolamine; Cephalin; O-(1-beta-Acyl-2-acyl-sn-glycero-3-phospho)ethanolamine; 1-Acyl-2-acyl-sn-glycero-3-phosphoethanolamine; L-1-Phosphatidylethanolamine | 1 | ec:2.1.1.17 ec:2.1.1.71 |
| cpd:C19080 |  | tRNA with a 3' CC end; tRNA with a 3' CC | 1 | ec:2.7.7.72 |
| cpd:C04186 |  | 5-Carboxymethyl-2-hydroxymuconate | 1 | ec:1.1.1.312 |
| cpd:C06735 |  | Aminoacetaldehyde; 2-Aminoacetaldehyde | 1 | ec:1.14.11.17 |
| cpd:C00748 |  | Siroheme | 1 | ec:4.99.1.4 ec:1.3.1.76 ec:2.1.1.107 |
| cpd:C02463 |  | Precorrin 2; Dihydrosirohydrochlorin | 1 | ec:4.99.1.4 ec:1.3.1.76 ec:2.1.1.107 |
| cpd:C19078 |  | tRNA with a 3' cytidine | 1 | ec:2.7.7.72 |

  
**Over-represented Pathway Summary**: Collection of the KEGG metabolic pathways containing the proteins identified in the "Over-represented Metabolite Summary" ranked by the highest number of hits per pathway  

| Pathway ID | EC | EC Frequency | Name |
| --- | --- | --- | --- |
| map00910 | ec:1.4.1.13 ec:1.18.6.1 ec:1.7.2.5 ec:1.13.12.16 ec:1.4.1.14 | 28 | path:map00910 Nitrogen metabolism |
| map00620 | ec:1.1.1.27 ec:2.7.2.1 ec:2.3.3.14 ec:1.2.1.3 ec:1.8.1.4 ec:2.3.1.8 ec:6.4.1.2 ec:2.3.3.9 | 25 | path:map00620 Pyruvate metabolism |
| map00730 | ec:2.5.1.3 ec:2.8.1.7 ec:2.7.4.16 ec:1.4.3.19 | 22 | path:map00730 Thiamine metabolism |
| map00362 | ec:5.3.2.8 ec:4.1.3.17 ec:3.1.1.24 ec:4.2.1.83 ec:5.3.2.6 ec:3.1.1.57 ec:1.13.11.8 ec:1.1.1.312 | 21 | path:map00362 Benzoate degradation |
| map00640 | ec:4.1.1.9 ec:1.1.1.27 ec:2.7.2.1 ec:5.4.99.2 ec:1.2.1.3 ec:2.3.1.8 ec:6.4.1.2 | 20 | path:map00640 Propanoate metabolism |
| map00630 | ec:5.4.99.2 ec:1.2.1.2 ec:2.3.3.1 ec:2.3.3.9 | 20 | path:map00630 Glyoxylate and dicarboxylate metabolism |
| map00780 | ec:2.3.1.47 ec:3.1.1.85 ec:1.3.1.10 ec:6.3.3.3 ec:2.1.1.197 ec:2.8.1.6 | 19 | path:map00780 Biotin metabolism |
| map00680 | ec:2.7.2.1 ec:1.2.99.5 ec:1.2.1.2 ec:2.3.1.8 ec:2.7.1.11 | 19 | path:map00680 Methane metabolism |
| map00720 | ec:2.7.2.1 ec:5.4.99.2 ec:1.2.7.3 ec:2.3.1.8 ec:6.4.1.2 | 18 | path:map00720 Carbon fixation pathways in prokaryotes |
| map00260 | ec:2.7.1.39 ec:4.2.1.20 ec:1.8.1.4 | 17 | path:map00260 Glycine, serine and threonine metabolism |
| map00625 | ec:1.18.6.1 ec:1.2.1.3 | 17 | path:map00625 Chloroalkane and chloroalkene degradation |
| map00240 | ec:2.4.2.6 ec:3.1.4.16 ec:1.8.1.9 ec:2.1.3.2 ec:1.17.4.1 ec:2.7.7.7 ec:2.4.2.10 | 16 | path:map00240 Pyrimidine metabolism |
| map00230 | ec:3.1.4.16 ec:2.7.6.5 ec:4.3.2.2 ec:1.17.4.1 ec:4.6.1.1 ec:2.7.7.7 | 15 | path:map00230 Purine metabolism |
| map00250 | ec:1.4.1.13 ec:2.1.3.2 ec:4.3.2.2 ec:3.5.1.2 ec:3.5.1.1 ec:1.4.1.14 | 13 | path:map00250 Alanine, aspartate and glutamate metabolism |
| map00270 | ec:4.1.1.50 ec:1.1.1.27 ec:2.1.1.10 ec:2.5.1.16 ec:2.3.1.30 ec:2.5.1.49 ec:2.5.1.48 ec:2.5.1.47 | 13 | path:map00270 Cysteine and methionine metabolism |
| map00500 | ec:3.2.1.1 ec:5.4.99.16 ec:2.4.1.18 ec:2.4.99.16 ec:2.4.1.1 | 12 | path:map00500 Starch and sucrose metabolism |
| map00622 | ec:5.3.2.6 | 11 | path:map00622 Xylene degradation |
| map00621 | ec:5.3.2.6 | 11 | path:map00621 Dioxin degradation |
| map00480 | ec:2.5.1.18 ec:2.5.1.16 ec:1.17.4.1 ec:1.11.1.15 ec:2.3.2.2 | 11 | path:map00480 Glutathione metabolism |
| map00061 | ec:6.3.4.14 ec:1.3.1.9 ec:6.4.1.2 | 10 | path:map00061 Fatty acid biosynthesis |
| map00430 | ec:2.7.2.1 ec:1.14.11.17 ec:2.3.1.8 ec:2.3.2.2 | 9 | path:map00430 Taurine and hypotaurine metabolism |
| map00770 | ec:2.7.7.3 ec:2.2.1.6 ec:2.7.8.7 ec:4.2.1.9 | 9 | path:map00770 Pantothenate and CoA biosynthesis |
| map00860 | ec:2.7.1.156 ec:2.5.1.17 ec:6.3.1.10 ec:6.1.1.17 ec:2.7.7.62 ec:2.1.1.107 ec:4.99.1.4 ec:4.2.1.24 ec:1.3.1.76 | 9 | path:map00860 Porphyrin and chlorophyll metabolism |
| map00920 | ec:1.14.11.17 ec:3.1.3.7 ec:2.3.1.30 ec:2.5.1.48 ec:2.5.1.47 | 8 | path:map00920 Sulfur metabolism |
| map00520 | ec:5.1.3.10 ec:3.2.1.52 | 8 | path:map00520 Amino sugar and nucleotide sugar metabolism |
| map00020 | ec:1.2.7.3 ec:2.3.3.1 ec:1.8.1.4 | 8 | path:map00020 Citrate cycle (TCA cycle) |
| map00290 | ec:4.2.1.35 ec:2.2.1.6 ec:1.1.1.85 ec:4.2.1.33 ec:4.2.1.9 | 7 | path:map00290 Valine, leucine and isoleucine biosynthesis |
| map00010 | ec:1.1.1.27 ec:1.2.1.3 ec:1.8.1.4 ec:2.7.1.11 | 6 | path:map00010 Glycolysis / Gluconeogenesis |
| map00564 | ec:4.3.1.7 ec:3.1.3.27 ec:2.1.1.71 ec:2.7.8.5 ec:2.1.1.17 | 6 | path:map00564 Glycerophospholipid metabolism |
| map00750 | ec:2.6.99.2 | 6 | path:map00750 Vitamin B6 metabolism |
| map00450 | ec:1.8.1.9 ec:6.1.1.10 ec:2.5.1.48 | 6 | path:map00450 Selenocompound metabolism |
| map00531 | ec:3.2.1.52 | 6 | path:map00531 Glycosaminoglycan degradation |
| map00982 | ec:2.5.1.18 | 6 | path:map00982 Drug metabolism - cytochrome P450 |
| map00330 | ec:4.1.1.50 ec:1.2.1.38 ec:1.2.1.3 ec:2.5.1.16 ec:3.5.1.2 | 6 | path:map00330 Arginine and proline metabolism |
| map00980 | ec:2.5.1.18 | 6 | path:map00980 Metabolism of xenobiotics by cytochrome P450 |
| map00970 | ec:6.1.1.12 ec:6.1.1.24 ec:6.1.1.11 ec:6.1.1.10 ec:6.1.1.9 ec:6.1.1.17 | 6 | path:map00970 Aminoacyl-tRNA biosynthesis |
| map00513 | ec:3.2.1.52 | 6 | path:map00513 Various types of N-glycan biosynthesis |
| map00511 | ec:3.2.1.52 | 6 | path:map00511 Other glycan degradation |
| map00410 | ec:4.1.1.9 ec:1.2.1.3 ec:2.5.1.16 | 6 | path:map00410 beta-Alanine metabolism |
| map00604 | ec:3.2.1.52 | 6 | path:map00604 Glycosphingolipid biosynthesis - ganglio series |
| map00603 | ec:3.2.1.52 | 6 | path:map00603 Glycosphingolipid biosynthesis - globo series |
| map00400 | ec:2.6.1.9 ec:4.2.3.4 ec:4.2.1.20 ec:2.7.1.71 | 5 | path:map00400 Phenylalanine, tyrosine and tryptophan biosynthesis |
| map00280 | ec:5.4.99.2 ec:1.2.1.3 ec:1.8.1.4 | 5 | path:map00280 Valine, leucine and isoleucine degradation |
| map00660 | ec:4.1.3.17 ec:4.2.1.35 ec:2.2.1.6 | 4 | path:map00660 C5-Branched dibasic acid metabolism |
| map04660 | ec:3.1.3.16 | 4 | path:map04660 T cell receptor signaling pathway |
| map00254 | ec:6.4.1.2 | 4 | path:map00254 Aflatoxin biosynthesis |
| map00253 | ec:6.4.1.2 | 4 | path:map00253 Tetracycline biosynthesis |
| map00627 | ec:3.1.1.57 ec:1.13.11.8 ec:1.1.1.312 | 4 | path:map00627 Aminobenzoate degradation |
| map00190 | ec:3.6.1.1 ec:1.9.3.1 | 4 | path:map00190 Oxidative phosphorylation |
| map00562 | ec:5.5.1.4 ec:2.7.7.74 ec:2.7.8.34 | 3 | path:map00562 Inositol phosphate metabolism |
| map00360 | ec:2.6.1.9 ec:6.2.1.30 | 3 | path:map00360 Phenylalanine metabolism |
| map00350 | ec:2.6.1.9 ec:1.1.1.312 | 3 | path:map00350 Tyrosine metabolism |
| map00340 | ec:2.6.1.9 ec:1.2.1.3 | 3 | path:map00340 Histidine metabolism |
| map00983 | ec:2.4.2.10 | 3 | path:map00983 Drug metabolism - other enzymes |
| map00030 | ec:4.1.2.4 ec:2.7.1.15 ec:2.7.1.11 | 3 | path:map00030 Pentose phosphate pathway |
| map00471 | ec:3.5.1.2 ec:5.1.1.3 | 3 | path:map00471 D-Glutamine and D-glutamate metabolism |
| map00071 | ec:1.2.1.3 ec:6.2.1.3 | 2 | path:map00071 Fatty acid degradation |
| map00401 | ec:2.6.1.9 | 2 | path:map00401 Novobiocin biosynthesis |
| map00460 | ec:3.5.1.1 ec:2.3.2.2 | 2 | path:map00460 Cyanoamino acid metabolism |
| map00300 | ec:2.3.3.14 | 2 | path:map00300 Lysine biosynthesis |
| map00550 | ec:3.4.16.4 ec:6.3.2.4 | 2 | path:map00550 Peptidoglycan biosynthesis |
| map00440 | ec:3.11.1.2 | 2 | path:map00440 Phosphonate and phosphinate metabolism |
| map00633 | ec:1.12.99.6 | 2 | path:map00633 Nitrotoluene degradation |
| map00790 | ec:3.5.4.16 ec:1.7.1.13 | 2 | path:map00790 Folate biosynthesis |
| map00624 | ec:1.13.11.8 | 2 | path:map00624 Polycyclic aromatic hydrocarbon degradation |
| map00523 | ec:5.1.3.25 | 2 | path:map00523 Polyketide sugar unit biosynthesis |
| map00960 | ec:2.6.1.9 | 2 | path:map00960 Tropane, piperidine and pyridine alkaloid biosynthesis |
| map00561 | ec:1.2.1.3 | 1 | path:map00561 Glycerolipid metabolism |
| map00650 | ec:2.2.1.6 | 1 | path:map00650 Butanoate metabolism |
| map00053 | ec:1.2.1.3 | 1 | path:map00053 Ascorbate and aldarate metabolism |
| map00052 | ec:2.7.1.11 | 1 | path:map00052 Galactose metabolism |
| map00051 | ec:2.7.1.11 | 1 | path:map00051 Fructose and mannose metabolism |
| map00040 | ec:1.2.1.3 | 1 | path:map00040 Pentose and glucuronate interconversions |
| map00590 | ec:2.3.2.2 | 1 | path:map00590 Arachidonic acid metabolism |
| map00521 | ec:5.5.1.4 | 1 | path:map00521 Streptomycin biosynthesis |
| map00380 | ec:1.2.1.3 | 1 | path:map00380 Tryptophan metabolism |
| map00903 | ec:1.2.1.3 | 1 | path:map00903 Limonene and pinene degradation |
| map00473 | ec:6.3.2.4 | 1 | path:map00473 D-Alanine metabolism |
| map00310 | ec:1.2.1.3 | 1 | path:map00310 Lysine degradation |

  
Analysis performed on 2014/02/15 00:38:59
