# Supplementary figures and images for: Automated genomic context analysis and experimental validation platform for discovery of prokaryote transcriptional regulator functions
Source: BMC Genomics. 2014 Dec 18;15(1):1142. doi: 10.1186/1471-2164-15-1142 (PMC4349456; doi:10.1186/1471-2164-15-1142)

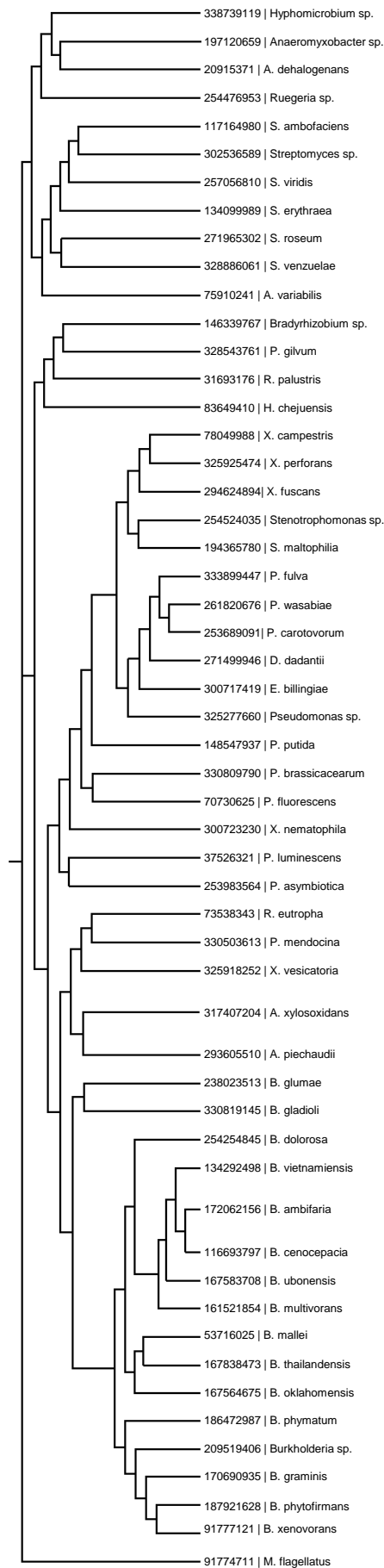

Supplement: Supplementary file 12 — Additional file 12: Figure Af1: Dendrogram of microbial Bxe_B3018 Homologues. BLAST analysis (utilizing a 40 aa-IDc) identified the existence of 81 homologs. These homologs were found only in Burkholderia or Pseudomonas genomes. (PDF 15 KB) [file 12864_2014_6995_MOESM12_ESM.pdf]

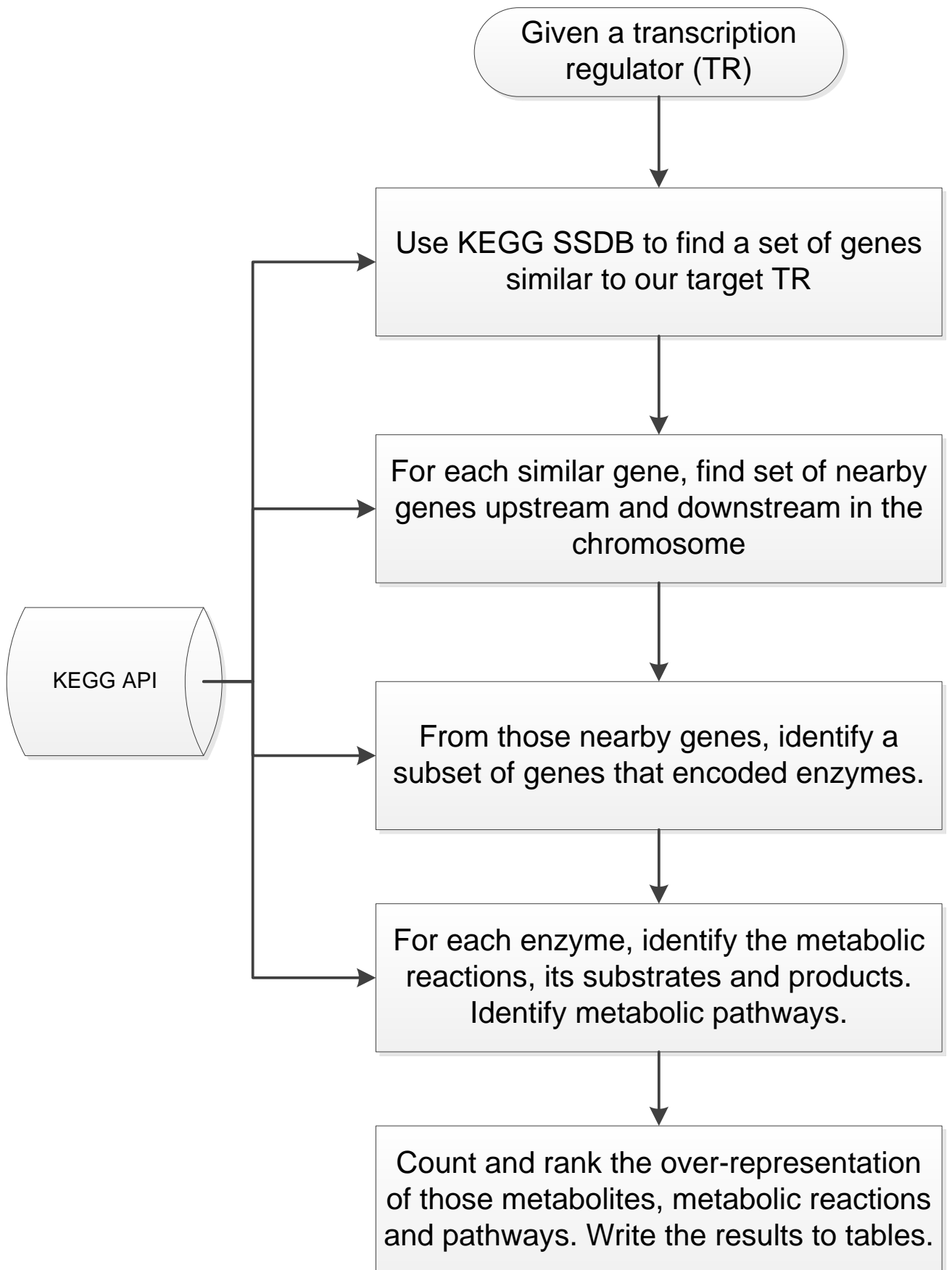

Supplement: Supplementary file 15 — Additional file 15: Figure Af3: Flowchart describing the steps used by the Function Discovery V1.0 software to predict the metabolic involvement of a given TR. (PDF 9 KB) [file 12864_2014_6995_MOESM15_ESM.pdf]
