# Supplementary material for: Automated genomic context analysis and experimental validation platform for discovery of prokaryote transcriptional regulator functions
Source: BMC Genomics. 2014 Dec 18;15(1):1142. doi: 10.1186/1471-2164-15-1142 (PMC4349456; doi:10.1186/1471-2164-15-1142)
Supplement: Supplementary file 13 — Additional file 13: Result Bxe_B3018. Function Discovery V1.0 output (.html format) for the previously unknown function transcriptional regulator (Bxe_ B3018, MgxR). For detailed instructions on how to analyze the results please refer to the Function Discovery V1.0, a gene neighborhood analysis tool section in the Results part of the main text. (HTML 551 KB) [file 12864_2014_6995_MOESM13_ESM.html]

```
ENTRY       Bxe_B3018         CDS       T00340
DEFINITION  TetR family transcriptional regulator
ORGANISM    bxe  Burkholderia xenovorans
POSITION    2:12472..13137
MOTIF       Pfam: TetR_N
DBLINKS     NCBI-GI: 91777121
            NCBI-GeneID: 4006480
            JGI: BxeB3018
            UniProt: Q13SG0
AASEQ       221
            MATETHIESVPPGSARERLLDAAEALIYAGGIHATGVDAIVKQSGTARKSFYTHFESKDA
            LVAAALDRRDERWMNWFIAGTQRHGKTARKRLLGMFEVLREWFASKDFHGCAFLNASGEI
            ASADDPIRIVARKHKERLLAFVRTECDGLAAESGMDARRAARLSRQWLILLDGAIAVALV
            SGEPDAALDAQAAAQVLLDAECACERGTPPSKRPPSRRTAT
NTSEQ       666
            atggctaccgaaactcacattgaatcagtgccgccgggcagcgcccgcgagcggctgctg
            gatgcggcggaggcgttgatctacgccggcggcattcacgcaaccggcgtggacgcgatc
            gtcaagcagtccggcacggcgcgcaaaagcttctacacgcatttcgaatcgaaagacgca
            ctcgtggcggctgcgctcgaccggcgcgacgaacgctggatgaactggttcatcgcaggc
            acgcaacggcacggtaagacggcgcgcaaacgtctgctcggcatgttcgaggtgctgcgc
            gaatggtttgcgtcgaaagattttcatggttgcgcgtttctgaatgcgtccggtgagatc
            gcttccgcggacgatccgatcaggatcgtcgcgcgcaaacacaaggaacgcctgctggcg
            ttcgtgcggaccgaatgcgacgggttggctgcggagtcgggcatggatgcccgccgcgcc
            gcgcgtctgtcgcgtcaatggttgatcctgctcgacggcgcgattgccgtcgcgctggta
            agcggcgagcccgatgcggcgctcgacgcgcaagctgcagcccaagtcctgctcgacgcg
            gagtgtgcgtgcgagcgaggcacaccgccaagcaaacgaccgccatcccggcgaaccgca
            acctga
///
```

  
**Homolog ID**: Table of closest homologs  

```
                 Homologs                                       len   identity overlap
---------------------------------------------------------------------------------
bpy:Bphyt_7001 TetR family transcriptional regulator          221     0.932    221 
bge:BC1002_5113 TetR family transcriptional regulator         223     0.785    214 
bgf:BC1003_4755 regulatory protein TetR                       233     0.742    233 
bph:Bphy_4162 TetR family transcriptional regulator           210     0.797    197 
bte:BTH_II0010 TetR family transcriptional regulator          205     0.706    197 
bma:BMAA0008 TetR family transcriptional regulator            205     0.701    197 
bml:BMA10229_1436 TetR family transcriptional regulator       205     0.701    197 
bmn:BMA10247_A0011 TetR family transcriptional regulato       205     0.701    197 
bmv:BMASAVP1_1156 TetR family transcriptional regulator       205     0.701    197 
bpd:BURPS668_A0010 TetR family transcriptional regulato       205     0.701    197 
bpl:BURPS1106A_A0010 TetR family transcriptional regula       205     0.701    197 
bpm:BURPS1710b_A1515 TetR family transcriptional regula       205     0.701    197 
bps:BPSS0008 TetR family transcriptional regulator            205     0.701    197 
bur:Bcep18194_B3175 TetR family transcriptional regulat       201     0.692    195 
bch:Bcen2424_5704 TetR family transcriptional regulator       201     0.707    188 
bcm:Bcenmc03_4534 TetR family transcriptional regulator       201     0.707    188 
bcn:Bcen_5155 TetR family transcriptional regulator           201     0.707    188 
bac:BamMC406_3117 TetR family transcriptional regulator       204     0.677    195 
bam:Bamb_4974 TetR family transcriptional regulator           204     0.677    195 
bcj:BCAM0014 TetR family regulatory protein                   201     0.688    189 
bvi:Bcep1808_3786 TetR family transcriptional regulator       204     0.663    199 
bmj:BMULJ_03202 TetR family transcriptional regulator         208     0.686    194 
bmu:Bmul_5319 TetR family transcriptional regulator           208     0.686    194 
bgl:bglu_2g00130 TetR family transcriptional regulator        225     0.681    188 
pay:PAU_02084 transcriptional regulator, tetr family          201     0.456    195 
smt:Smal_2003 TetR family transcriptional regulator           189     0.484    184 
reu:Reut_B4516 TetR family transcriptional regulator          197     0.513    187 
ebi:EbC_28440 TetR family transcripitonal regulator           199     0.471    189 
pfl:PFL_3261 TetR family transcriptional regulator            187     0.489    184 
ddc:Dd586_1390 TetR family transcriptional regulator          188     0.470    185 
pfo:Pfl01_2877 TetR family transcriptional regulator          188     0.484    186 
xcv:XCV4432 TetR family transcriptional regulator             189     0.457    188 
ddd:Dda3937_03586 TetR family transcriptional regulator       200     0.470    185 
pct:PC1_2715 TetR family transcriptional regulator            189     0.464    183 
pwa:Pecwa_1371 TetR family transcriptional regulator          189     0.471    187 
ppf:Pput_2721 TetR family transcriptional regulator           194     0.447    188 
plu:plu2425 hypothetical protein                              201     0.416    197 
ppw:PputW619_3097 TetR family transcriptional regulator       189     0.447    190 
pfs:PFLU2916 TetR family transcriptional regulator            187     0.481    183 
ppu:PP_2951 TetR family transcriptional regulator             188     0.454    183 
xne:XNC1_2294 TetR family transcriptional regulator           208     0.437    183 
acp:A2cp1_0250 TetR family transcriptional regulator          201     0.418    208 
ank:AnaeK_0239 TetR family transcriptional regulator          201     0.413    208 
ade:Adeh_0228 TetR family transcriptional regulator           201     0.413    208 
mfa:Mfla_0355 TetR family transcriptional regulator           192     0.402    194 
ava:Ava_4037 TetR family transcriptional regulator            200     0.400    185 
sen:SACE_3451 TetR family transcriptional regulator           197     0.460    189 
sro:Sros_3836 TetR family transcriptional regulator           179     0.439    187 
svi:Svir_28340 transcriptional regulator, TetR family         188     0.410    183 
fri:FraEuI1c_3599 TetR family transcriptional regulator       199     0.402    189 
sco:SCO0772 regulatory protein                                192     0.402    204 
cai:Caci_5341 TetR family transcriptional regulator           204     0.402    184 
xau:Xaut_2611 TetR family transcriptional regulator           228     0.404    193 
cwo:Cwoe_0417 TetR family transcriptional regulator           183     0.403    186
```

**Neighborhood Representations**: Table of genes in the defined genetic neighborhoods of the entry protein and its closest homologs  
  
**Neighborhood Representations for "bxe:Bxe\_B3018"**  

| ID | Annotation | EC number |
| --- | --- | --- |
| bxe:Bxe\_B3028 | putative partition protein ParA; K03496 chromosome partitioning protein |  |
| bxe:Bxe\_B3027 | ParB family protein; K03497 chromosome partitioning protein, ParB family |  |
| bxe:Bxe\_B3026 | putative replication protein |  |
| bxe:Bxe\_B3025 | hypothetical protein |  |
| bxe:Bxe\_B3024 | putative phage integrase |  |
| bxe:Bxe\_B3023 | hypothetical protein |  |
| bxe:Bxe\_B3022 | XRE family transcriptional regulator |  |
| bxe:Bxe\_B3021 | 2-amino-3-ketobutyrate coenzyme A ligase (EC:2.3.1.29); K00639 glycine C-acetyltransferase [EC:2.3.1.29] | ec:2.3.1.29 |
| bxe:Bxe\_B3020 | tdh; L-threonine 3-dehydrogenase (EC:1.1.1.103); K00060 threonine 3-dehydrogenase [EC:1.1.1.103] | ec:1.1.1.103 |
| bxe:Bxe\_B3019 | putative YceI like family protein |  |
| bxe:Bxe\_B3018 | TetR family transcriptional regulator |  |
| bxe:Bxe\_B3017 | hypothetical protein; K09958 hypothetical protein |  |
| bxe:Bxe\_B3016 | hypothetical protein |  |
| bxe:Bxe\_B3015 | MarR family transcriptional regulator |  |
| bxe:Bxe\_B3014 | hypothetical protein |  |
| bxe:Bxe\_B3013 | DNA-7-methylguanine glycosylase (EC:3.2.2.-) |  |
| bxe:Bxe\_B3012 | hypothetical protein |  |
| bxe:Bxe\_B3011 | CheA signal transduction histidine kinases (STHK) (EC:2.7.3.-); K00936 [EC:2.7.3.-] |  |
| bxe:Bxe\_B3010 | methyl-accepting chemotaxis sensory transducer |  |
| bxe:Bxe\_B3009 | hypothetical protein |  |
| bxe:Bxe\_B3008 | hypothetical protein |  |

  
**Neighborhood Representations for "bpy:Bphyt\_7001"**  

| ID | Annotation | EC number |
| --- | --- | --- |
| bpy:Bphyt\_6991 | hydrophobe/amphiphile efflux-1 (HAE1) family transporter; K03296 hydrophobic/amphiphilic exporter-1 (mainly G- bacteria), HAE1 family |  |
| bpy:Bphyt\_6992 | RND family efflux transporter MFP subunit; K03585 membrane fusion protein |  |
| bpy:Bphyt\_6993 | two component winged helix family transcriptional regulator |  |
| bpy:Bphyt\_6994 | integral membrane sensor signal transduction histidine kinase; K07638 two-component system, OmpR family, osmolarity sensor histidine kinase EnvZ [EC:2.7.13.3] | ec:2.7.13.3 |
| bpy:Bphyt\_6995 | hypothetical protein |  |
| bpy:Bphyt\_6996 | hypothetical protein |  |
| bpy:Bphyt\_6997 | hypothetical protein |  |
| bpy:Bphyt\_6998 | signal peptide transmembrane protein |  |
| bpy:Bphyt\_6999 | hypothetical protein |  |
| bpy:Bphyt\_7000 | hypothetical protein; K09958 hypothetical protein |  |
| bpy:Bphyt\_7001 | TetR family transcriptional regulator |  |
| bpy:Bphyt\_7002 | hypothetical protein |  |
| bpy:Bphyt\_7003 | tdh; L-threonine 3-dehydrogenase; K00060 threonine 3-dehydrogenase [EC:1.1.1.103] | ec:1.1.1.103 |
| bpy:Bphyt\_7004 | 2-amino-3-ketobutyrate coenzyme A ligase (EC:2.3.1.29); K00639 glycine C-acetyltransferase [EC:2.3.1.29] | ec:2.3.1.29 |
| bpy:Bphyt\_7005 | XRE family transcriptional regulator |  |
| bpy:Bphyt\_7006 | hypothetical protein |  |
| bpy:Bphyt\_7007 | integrase family protein |  |
| bpy:Bphyt\_7008 | hypothetical protein |  |
| bpy:Bphyt\_7009 | replication protein |  |
| bpy:Bphyt\_7010 | parB-like partition protein; K03497 chromosome partitioning protein, ParB family |  |
| bpy:Bphyt\_7011 | cobyrinic acid a,c-diamide synthase; K03496 chromosome partitioning protein |  |

  
**Neighborhood Representations for "bge:BC1002\_5113"**  

| ID | Annotation | EC number |
| --- | --- | --- |
| bge:BC1002\_5103 | cobyrinic acid ac-diamide synthase; K03496 chromosome partitioning protein |  |
| bge:BC1002\_5104 | parB-like partition protein; K03497 chromosome partitioning protein, ParB family |  |
| bge:BC1002\_5105 | initiator RepB protein |  |
| bge:BC1002\_5106 | hypothetical protein |  |
| bge:BC1002\_5107 | integrase family protein |  |
| bge:BC1002\_5108 | hypothetical protein |  |
| bge:BC1002\_5109 | XRE family transcriptional regulator |  |
| bge:BC1002\_5110 | 2-amino-3-ketobutyrate coenzyme A ligase (EC:2.3.1.29); K00639 glycine C-acetyltransferase [EC:2.3.1.29] | ec:2.3.1.29 |
| bge:BC1002\_5111 | L-threonine 3-dehydrogenase; K00060 threonine 3-dehydrogenase [EC:1.1.1.103] | ec:1.1.1.103 |
| bge:BC1002\_5112 | hypothetical protein |  |
| bge:BC1002\_5113 | TetR family transcriptional regulator |  |
| bge:BC1002\_5114 | hypothetical protein; K09958 hypothetical protein |  |
| bge:BC1002\_5115 | hypothetical protein |  |
| bge:BC1002\_5116 | general substrate transporter |  |
| bge:BC1002\_5117 | major facilitator superfamily protein |  |
| bge:BC1002\_5118 | ferredoxin; K03863 vanillate monooxygenase [EC:1.14.13.82] | ec:1.14.13.82 |
| bge:BC1002\_5119 | LysR family transcriptional regulator |  |
| bge:BC1002\_5120 | Rieske (2Fe-2S) iron-sulfur domain-containing protein |  |
| bge:BC1002\_5121 | aromatic-ring-hydroxylating dioxygenase subunit beta |  |
| bge:BC1002\_5122 | 4-oxalocrotonate tautomerase; K01821 4-oxalocrotonate tautomerase [EC:5.3.2.6] | ec:5.3.2.6 |
| bge:BC1002\_5123 | PAS/PAC sensor signal transduction histidine kinase |  |

  
**Neighborhood Representations for "bgf:BC1003\_4755"**  

| ID | Annotation | EC number |
| --- | --- | --- |
| bgf:BC1003\_4745 | ribose-phosphate pyrophosphokinase (EC:2.7.6.1); K00948 ribose-phosphate pyrophosphokinase [EC:2.7.6.1] | ec:2.7.6.1 |
| bgf:BC1003\_4746 | hypothetical protein |  |
| bgf:BC1003\_4747 | nicotinate phosphoribosyltransferase; K00763 nicotinate phosphoribosyltransferase [EC:6.3.4.21] | ec:6.3.4.21 |
| bgf:BC1003\_4748 | PHP domain-containing protein; K02347 DNA polymerase (family X) |  |
| bgf:BC1003\_4749 | AMP-dependent synthetase and ligase |  |
| bgf:BC1003\_4750 | AMP-dependent synthetase and ligase |  |
| bgf:BC1003\_4751 | pseudogene |  |
| bgf:BC1003\_4752 | hypothetical protein |  |
| bgf:BC1003\_4753 | hypothetical protein |  |
| bgf:BC1003\_4754 | hypothetical protein; K09958 hypothetical protein |  |
| bgf:BC1003\_4755 | regulatory protein TetR |  |
| bgf:BC1003\_4756 | YceI family protein |  |
| bgf:BC1003\_4757 | L-threonine 3-dehydrogenase; K00060 threonine 3-dehydrogenase [EC:1.1.1.103] | ec:1.1.1.103 |
| bgf:BC1003\_4758 | 2-amino-3-ketobutyrate coenzyme A ligase (EC:2.3.1.29); K00639 glycine C-acetyltransferase [EC:2.3.1.29] | ec:2.3.1.29 |
| bgf:BC1003\_4759 | helix-turn-helix domain-containing protein |  |
| bgf:BC1003\_4760 | hypothetical protein |  |
| bgf:BC1003\_4761 | integrase family protein |  |
| bgf:BC1003\_4762 | hypothetical protein |  |
| bgf:BC1003\_4763 | hypothetical protein |  |
| bgf:BC1003\_4764 | initiator RepB protein |  |
| bgf:BC1003\_4765 | parB-like partition protein; K03497 chromosome partitioning protein, ParB family |  |

  
**Neighborhood Representations for "bph:Bphy\_4162"**  

| ID | Annotation | EC number |
| --- | --- | --- |
| bph:Bphy\_4152 | parB-like partition protein; K03497 chromosome partitioning protein, ParB family |  |
| bph:Bphy\_4153 | putative replication protein |  |
| bph:Bphy\_4154 | hypothetical protein |  |
| bph:Bphy\_4155 | hypothetical protein |  |
| bph:Bphy\_4156 | integrase family protein |  |
| bph:Bphy\_4157 | hypothetical protein |  |
| bph:Bphy\_4158 | XRE family transcriptional regulator |  |
| bph:Bphy\_4159 | 2-amino-3-ketobutyrate coenzyme A ligase (EC:2.3.1.29); K00639 glycine C-acetyltransferase [EC:2.3.1.29] | ec:2.3.1.29 |
| bph:Bphy\_4160 | tdh; L-threonine 3-dehydrogenase; K00060 threonine 3-dehydrogenase [EC:1.1.1.103] | ec:1.1.1.103 |
| bph:Bphy\_4161 | hypothetical protein |  |
| bph:Bphy\_4162 | TetR family transcriptional regulator |  |
| bph:Bphy\_4163 | hypothetical protein; K09958 hypothetical protein |  |
| bph:Bphy\_4164 | hypothetical protein |  |
| bph:Bphy\_4165 | LysR family transcriptional regulator |  |
| bph:Bphy\_4166 | alcohol dehydrogenase |  |
| bph:Bphy\_4167 | GAF sensor hybrid histidine kinase (EC:2.7.13.3) |  |
| bph:Bphy\_4168 | glutathione-dependent formaldehyde-activating GFA |  |
| bph:Bphy\_4169 | hypothetical protein |  |
| bph:Bphy\_4170 | hypothetical protein |  |
| bph:Bphy\_4171 | hypothetical protein |  |
| bph:Bphy\_4172 | short-chain dehydrogenase/reductase SDR |  |

  
**Neighborhood Representations for "bte:BTH\_II0010"**  

| ID | Annotation | EC number |
| --- | --- | --- |
| bte:BTH\_II2373 | plasmid replication protein |  |
| bte:BTH\_II0001 | hypothetical protein |  |
| bte:BTH\_II0002 | phage integrase |  |
| bte:BTH\_II0003 | hypothetical protein |  |
| bte:BTH\_II0004 | DNA-binding protein |  |
| bte:BTH\_II0005 | kbl; 2-amino-3-ketobutyrate CoA ligase (EC:2.3.1.29); K00639 glycine C-acetyltransferase [EC:2.3.1.29] | ec:2.3.1.29 |
| bte:BTH\_II0006 | tdh; L-threonine 3-dehydrogenase (EC:1.1.1.103); K00060 threonine 3-dehydrogenase [EC:1.1.1.103] | ec:1.1.1.103 |
| bte:BTH\_II0007 | K+-transporting ATPase subunit A |  |
| bte:BTH\_II0008 | acetyltransferase |  |
| bte:BTH\_II0009 | hypothetical protein |  |
| bte:BTH\_II0010 | TetR family transcriptional regulator |  |
| bte:BTH\_II0011 | hypothetical protein; K09958 hypothetical protein |  |
| bte:BTH\_II0012 | succinylglutamate desuccinylase / aspartoacylase |  |
| bte:BTH\_II0013 | tartrate dehydrogenase (EC:1.1.1.93); K07246 tartrate dehydrogenase/decarboxylase / D-malate dehydrogenase [EC:1.1.1.93 4.1.1.73 1.1.1.83] | ec:1.1.1.93 ec:1.1.1.83 ec:4.1.1.73 |
| bte:BTH\_II0014 | LysR family transcriptional regulator; K16135 LysR family transcriptional regulator, transcriptional activator for dmlA |  |
| bte:BTH\_II0015 | hypothetical protein |  |
| bte:BTH\_II0016 | glutathione S-transferase; K00799 glutathione S-transferase [EC:2.5.1.18] | ec:2.5.1.18 |
| bte:BTH\_II0017 | hypothetical protein |  |
| bte:BTH\_II0018 | patatin-like phospholipase; K07001 NTE family protein |  |
| bte:BTH\_II0019 | 3-hydroxybutyrate dehydrogenase (EC:1.1.1.30); K00019 3-hydroxybutyrate dehydrogenase [EC:1.1.1.30] | ec:1.1.1.30 |
| bte:BTH\_II0020 | acetoacetate decarboxylase (EC:4.1.1.4); K01574 acetoacetate decarboxylase [EC:4.1.1.4] | ec:4.1.1.4 |

  
**Neighborhood Representations for "bma:BMAA0008"**  

| ID | Annotation | EC number |
| --- | --- | --- |
| bma:BMAA2116 | ISBma2, transposase |  |
| bma:BMAA2117 | plasmid replication protein |  |
| bma:BMAA2118 | hypothetical protein |  |
| bma:BMAA0001 | hypothetical protein |  |
| bma:BMAA0002 | phage integrase family protein |  |
| bma:BMAA0003 | hypothetical protein |  |
| bma:BMAA0004 | DNA-binding protein |  |
| bma:BMAA0005 | kbl; 2-amino-3-ketobutyrate CoA ligase (EC:2.3.1.29); K00639 glycine C-acetyltransferase [EC:2.3.1.29] | ec:2.3.1.29 |
| bma:BMAA0006 | tdh; L-threonine 3-dehydrogenase (EC:1.1.1.103); K00060 threonine 3-dehydrogenase [EC:1.1.1.103] | ec:1.1.1.103 |
| bma:BMAA0007 | hypothetical protein |  |
| bma:BMAA0008 | TetR family transcriptional regulator |  |
| bma:BMAA0009 | hypothetical protein; K09958 hypothetical protein |  |
| bma:BMAA0010 | succinylglutamate desuccinylase |  |
| bma:BMAA0011 | ttuC; tartrate dehydrogenase (EC:1.1.1.93); K07246 tartrate dehydrogenase/decarboxylase / D-malate dehydrogenase [EC:1.1.1.93 4.1.1.73 1.1.1.83] | ec:1.1.1.93 ec:1.1.1.83 ec:4.1.1.73 |
| bma:BMAA0012 | LysR family transcriptional regulator; K16135 LysR family transcriptional regulator, transcriptional activator for dmlA |  |
| bma:BMAA0013 | hypothetical protein |  |
| bma:BMAA0014 | glutathione S-transferase; K00799 glutathione S-transferase [EC:2.5.1.18] | ec:2.5.1.18 |
| bma:BMAA0015 | hypothetical protein |  |
| bma:BMAA0016 | patatin-like phospholipase; K07001 NTE family protein |  |
| bma:BMAA0017 | bdhA-1; 3-hydroxybutyrate dehydrogenase (EC:1.1.1.30); K00019 3-hydroxybutyrate dehydrogenase [EC:1.1.1.30] | ec:1.1.1.30 |
| bma:BMAA0018 | adc; acetoacetate decarboxylase (EC:4.1.1.4); K01574 acetoacetate decarboxylase [EC:4.1.1.4] | ec:4.1.1.4 |

  
**Neighborhood Representations for "bml:BMA10229\_1436"**  

| ID | Annotation | EC number |
| --- | --- | --- |
| bml:BMA10229\_1426 | hypothetical protein |  |
| bml:BMA10229\_1427 | hypothetical protein |  |
| bml:BMA10229\_1428 | phage integrase family protein |  |
| bml:BMA10229\_1429 | hypothetical protein |  |
| bml:BMA10229\_1430 | DNA-binding protein |  |
| bml:BMA10229\_1431 | kbl; 2-amino-3-ketobutyrate CoA ligase; K00639 glycine C-acetyltransferase [EC:2.3.1.29] | ec:2.3.1.29 |
| bml:BMA10229\_1432 | tdh; L-threonine 3-dehydrogenase; K00060 threonine 3-dehydrogenase [EC:1.1.1.103] | ec:1.1.1.103 |
| bml:BMA10229\_1433 | hypothetical protein |  |
| bml:BMA10229\_1434 | hypothetical protein |  |
| bml:BMA10229\_1435 | hypothetical protein |  |
| bml:BMA10229\_1436 | TetR family transcriptional regulator |  |
| bml:BMA10229\_1437 | hypothetical protein; K09958 hypothetical protein |  |
| bml:BMA10229\_1438 | succinylglutamate desuccinylase |  |
| bml:BMA10229\_1439 | hypothetical protein |  |
| bml:BMA10229\_1440 | ttuC; tartrate dehydrogenase; K07246 tartrate dehydrogenase/decarboxylase / D-malate dehydrogenase [EC:1.1.1.93 4.1.1.73 1.1.1.83] | ec:1.1.1.93 ec:1.1.1.83 ec:4.1.1.73 |
| bml:BMA10229\_1441 | LysR family transcriptional regulator; K16135 LysR family transcriptional regulator, transcriptional activator for dmlA |  |
| bml:BMA10229\_1442 | hypothetical protein |  |
| bml:BMA10229\_1443 | glutathione S-transferase; K00799 glutathione S-transferase [EC:2.5.1.18] | ec:2.5.1.18 |
| bml:BMA10229\_1444 | hypothetical protein |  |
| bml:BMA10229\_1445 | hypothetical protein |  |
| bml:BMA10229\_1446 | patatin-like phospholipase; K07001 NTE family protein |  |

  
**Neighborhood Representations for "bmn:BMA10247\_A0011"**  

| ID | Annotation | EC number |
| --- | --- | --- |
| bmn:BMA10247\_A0001 | hypothetical protein |  |
| bmn:BMA10247\_A0002 | hypothetical protein |  |
| bmn:BMA10247\_A0003 | phage integrase family site specific recombinase |  |
| bmn:BMA10247\_A0004 | hypothetical protein |  |
| bmn:BMA10247\_A0005 | DNA-binding protein |  |
| bmn:BMA10247\_A0006 | kbl; 2-amino-3-ketobutyrate coenzyme A ligase (EC:2.3.1.29); K00639 glycine C-acetyltransferase [EC:2.3.1.29] | ec:2.3.1.29 |
| bmn:BMA10247\_A0007 | tdh; L-threonine 3-dehydrogenase (EC:1.1.1.103); K00060 threonine 3-dehydrogenase [EC:1.1.1.103] | ec:1.1.1.103 |
| bmn:BMA10247\_A0008 | hypothetical protein |  |
| bmn:BMA10247\_A0009 | putative lipoprotein |  |
| bmn:BMA10247\_A0010 | hypothetical protein |  |
| bmn:BMA10247\_A0011 | TetR family transcriptional regulator |  |
| bmn:BMA10247\_A0012 | hypothetical protein; K09958 hypothetical protein |  |
| bmn:BMA10247\_A0013 | succinylglutamate desuccinylase / aspartoacylase family protein |  |
| bmn:BMA10247\_A0014 | ttuC; tartrate dehydrogenase (EC:1.1.1.93); K07246 tartrate dehydrogenase/decarboxylase / D-malate dehydrogenase [EC:1.1.1.93 4.1.1.73 1.1.1.83] | ec:1.1.1.93 ec:1.1.1.83 ec:4.1.1.73 |
| bmn:BMA10247\_A0015 | LysR family transcriptional regulator; K16135 LysR family transcriptional regulator, transcriptional activator for dmlA |  |
| bmn:BMA10247\_A0016 | hypothetical protein |  |
| bmn:BMA10247\_A0017 | hypothetical protein |  |
| bmn:BMA10247\_A0018 | glutathione S-transferase; K00799 glutathione S-transferase [EC:2.5.1.18] | ec:2.5.1.18 |
| bmn:BMA10247\_A0019 | hypothetical protein |  |
| bmn:BMA10247\_A0020 | patatin family phospholipase; K07001 NTE family protein |  |
| bmn:BMA10247\_A0021 | bdhA-1; 3-hydroxybutyrate dehydrogenase (EC:1.1.1.30); K00019 3-hydroxybutyrate dehydrogenase [EC:1.1.1.30] | ec:1.1.1.30 |

  
**Neighborhood Representations for "bmv:BMASAVP1\_1156"**  

| ID | Annotation | EC number |
| --- | --- | --- |
| bmv:BMASAVP1\_1146 | hypothetical protein |  |
| bmv:BMASAVP1\_1147 | hypothetical protein |  |
| bmv:BMASAVP1\_1148 | phage integrase |  |
| bmv:BMASAVP1\_1149 | hypothetical protein |  |
| bmv:BMASAVP1\_1150 | DNA-binding protein |  |
| bmv:BMASAVP1\_1151 | kbl; 2-amino-3-ketobutyrate CoA ligase; K00639 glycine C-acetyltransferase [EC:2.3.1.29] | ec:2.3.1.29 |
| bmv:BMASAVP1\_1152 | tdh; L-threonine 3-dehydrogenase; K00060 threonine 3-dehydrogenase [EC:1.1.1.103] | ec:1.1.1.103 |
| bmv:BMASAVP1\_1153 | hypothetical protein |  |
| bmv:BMASAVP1\_1154 | lipoprotein |  |
| bmv:BMASAVP1\_1155 | hypothetical protein |  |
| bmv:BMASAVP1\_1156 | TetR family transcriptional regulator |  |
| bmv:BMASAVP1\_1157 | hypothetical protein; K09958 hypothetical protein |  |
| bmv:BMASAVP1\_1158 | succinylglutamate desuccinylase / aspartoacylase |  |
| bmv:BMASAVP1\_1159 | ttuC; tartrate dehydrogenase; K07246 tartrate dehydrogenase/decarboxylase / D-malate dehydrogenase [EC:1.1.1.93 4.1.1.73 1.1.1.83] | ec:1.1.1.93 ec:1.1.1.83 ec:4.1.1.73 |
| bmv:BMASAVP1\_1160 | LysR family transcriptional regulator; K16135 LysR family transcriptional regulator, transcriptional activator for dmlA |  |
| bmv:BMASAVP1\_1161 | hypothetical protein |  |
| bmv:BMASAVP1\_1162 | hypothetical protein |  |
| bmv:BMASAVP1\_1163 | hypothetical protein |  |
| bmv:BMASAVP1\_1164 | glutathione S-transferase; K00799 glutathione S-transferase [EC:2.5.1.18] | ec:2.5.1.18 |
| bmv:BMASAVP1\_1165 | hypothetical protein |  |
| bmv:BMASAVP1\_1166 | patatin family phospholipase; K07001 NTE family protein |  |

  
**Neighborhood Representations for "bpd:BURPS668\_A0010"**  

| ID | Annotation | EC number |
| --- | --- | --- |
| bpd:BURPS668\_A3293 | hypothetical protein |  |
| bpd:BURPS668\_A3294 | hypothetical protein |  |
| bpd:BURPS668\_A0001 | chromosome segregation ATPase |  |
| bpd:BURPS668\_A0002 | integrase |  |
| bpd:BURPS668\_A0003 | Serine/threonine protein kinase |  |
| bpd:BURPS668\_A0004 | DNA-binding protein |  |
| bpd:BURPS668\_A0005 | kbl; 2-amino-3-ketobutyrate coenzyme A ligase (EC:2.3.1.29); K00639 glycine C-acetyltransferase [EC:2.3.1.29] | ec:2.3.1.29 |
| bpd:BURPS668\_A0006 | tdh; L-threonine 3-dehydrogenase (EC:1.1.1.103); K00060 threonine 3-dehydrogenase [EC:1.1.1.103] | ec:1.1.1.103 |
| bpd:BURPS668\_A0007 | hypothetical protein |  |
| bpd:BURPS668\_A0008 | hypothetical protein |  |
| bpd:BURPS668\_A0010 | TetR family transcriptional regulator |  |
| bpd:BURPS668\_A0012 | hypothetical protein; K09958 hypothetical protein |  |
| bpd:BURPS668\_A0011 | ttbk1 protein |  |
| bpd:BURPS668\_A0013 | succinylglutamate desuccinylase |  |
| bpd:BURPS668\_A0014 | hypothetical protein |  |
| bpd:BURPS668\_A0015 | tartrate dehydrogenase (EC:1.1.1.93); K07246 tartrate dehydrogenase/decarboxylase / D-malate dehydrogenase [EC:1.1.1.93 4.1.1.73 1.1.1.83] | ec:1.1.1.93 ec:1.1.1.83 ec:4.1.1.73 |
| bpd:BURPS668\_A0017 | hypothetical protein |  |
| bpd:BURPS668\_A0018 | hypothetical protein |  |
| bpd:BURPS668\_A0019 | glutathione S-transferase family protein (EC:2.5.1.18); K00799 glutathione S-transferase [EC:2.5.1.18] | ec:2.5.1.18 |
| bpd:BURPS668\_A0020 | hypothetical protein |  |
| bpd:BURPS668\_A0021 | hypothetical protein |  |

  
**Neighborhood Representations for "bpl:BURPS1106A\_A0010"**  

| ID | Annotation | EC number |
| --- | --- | --- |
| bpl:BURPS1106A\_A3178 | putative replication protein |  |
| bpl:BURPS1106A\_A0001 | hypothetical protein |  |
| bpl:BURPS1106A\_A0002 | phage integrase family site specific recombinase |  |
| bpl:BURPS1106A\_A0003 | hypothetical protein |  |
| bpl:BURPS1106A\_A0004 | DNA-binding cupin domain-containing protein |  |
| bpl:BURPS1106A\_A0005 | kbl; 2-amino-3-ketobutyrate coenzyme A ligase (EC:2.3.1.29); K00639 glycine C-acetyltransferase [EC:2.3.1.29] | ec:2.3.1.29 |
| bpl:BURPS1106A\_A0006 | tdh; L-threonine 3-dehydrogenase (EC:1.1.1.103); K00060 threonine 3-dehydrogenase [EC:1.1.1.103] | ec:1.1.1.103 |
| bpl:BURPS1106A\_A0007 | hypothetical protein |  |
| bpl:BURPS1106A\_A0008 | putative lipoprotein |  |
| bpl:BURPS1106A\_A0009 | hypothetical protein |  |
| bpl:BURPS1106A\_A0010 | TetR family transcriptional regulator |  |
| bpl:BURPS1106A\_A0012 | hypothetical protein; K09958 hypothetical protein |  |
| bpl:BURPS1106A\_A0011 | ttbk1 protein |  |
| bpl:BURPS1106A\_A0013 | succinylglutamate desuccinylase / aspartoacylase family protein |  |
| bpl:BURPS1106A\_A0014 | tartrate dehydrogenase (EC:1.1.1.93); K07246 tartrate dehydrogenase/decarboxylase / D-malate dehydrogenase [EC:1.1.1.93 4.1.1.73 1.1.1.83] | ec:1.1.1.93 ec:1.1.1.83 ec:4.1.1.73 |
| bpl:BURPS1106A\_A0015 | LysR family transcriptional regulator; K16135 LysR family transcriptional regulator, transcriptional activator for dmlA |  |
| bpl:BURPS1106A\_A0016 | isoprenylcysteine carboxyl methyltransferase family protein |  |
| bpl:BURPS1106A\_A0017 | glutathione S-transferase family protein (EC:2.5.1.18); K00799 glutathione S-transferase [EC:2.5.1.18] | ec:2.5.1.18 |
| bpl:BURPS1106A\_A0018 | hypothetical protein |  |
| bpl:BURPS1106A\_A0019 | hypothetical protein |  |
| bpl:BURPS1106A\_A0020 | patatin family phospholipase; K07001 NTE family protein |  |

  
**Neighborhood Representations for "bpm:BURPS1710b\_A1515"**  

| ID | Annotation | EC number |
| --- | --- | --- |
| bpm:BURPS1710b\_A1505 | plasmid replication protein |  |
| bpm:BURPS1710b\_A1506 | hypothetical protein |  |
| bpm:BURPS1710b\_A1507 | phage integrase family protein |  |
| bpm:BURPS1710b\_A1508 | hypothetical protein |  |
| bpm:BURPS1710b\_A1509 | putative DNA-binding protein |  |
| bpm:BURPS1710b\_A1510 | kbl; 2-amino-3-ketobutyrate CoA ligase; K00639 glycine C-acetyltransferase [EC:2.3.1.29] | ec:2.3.1.29 |
| bpm:BURPS1710b\_A1511 | hypothetical protein |  |
| bpm:BURPS1710b\_A1512 | tdh; L-threonine 3-dehydrogenase; K00060 threonine 3-dehydrogenase [EC:1.1.1.103] | ec:1.1.1.103 |
| bpm:BURPS1710b\_A1513 | hypothetical protein |  |
| bpm:BURPS1710b\_A1514 | hypothetical protein |  |
| bpm:BURPS1710b\_A1515 | TetR family transcriptional regulator |  |
| bpm:BURPS1710b\_A1516 | hypothetical protein; K09958 hypothetical protein |  |
| bpm:BURPS1710b\_A1517 | succinylglutamate desuccinylase |  |
| bpm:BURPS1710b\_A1518 | tartrate dehydrogenase; K07246 tartrate dehydrogenase/decarboxylase / D-malate dehydrogenase [EC:1.1.1.93 4.1.1.73 1.1.1.83] | ec:1.1.1.93 ec:1.1.1.83 ec:4.1.1.73 |
| bpm:BURPS1710b\_A1519 | LysR family transcriptional regulator; K16135 LysR family transcriptional regulator, transcriptional activator for dmlA |  |
| bpm:BURPS1710b\_A1520 | hypothetical protein |  |
| bpm:BURPS1710b\_A1521 | glutathione S-transferase; K00799 glutathione S-transferase [EC:2.5.1.18] | ec:2.5.1.18 |
| bpm:BURPS1710b\_A1522 | hypothetical protein |  |
| bpm:BURPS1710b\_A1523 | patatin-like phospholipase; K07001 NTE family protein |  |
| bpm:BURPS1710b\_A1524 | bdhA-1; 3-hydroxybutyrate dehydrogenase; K00019 3-hydroxybutyrate dehydrogenase [EC:1.1.1.30] | ec:1.1.1.30 |
| bpm:BURPS1710b\_A1525 | acetoacetate decarboxylase; K01574 acetoacetate decarboxylase [EC:4.1.1.4] | ec:4.1.1.4 |

  
**Neighborhood Representations for "bps:BPSS0008"**  

| ID | Annotation | EC number |
| --- | --- | --- |
| bps:BPSS2349 | partition protein ParA; K03496 chromosome partitioning protein |  |
| bps:BPSS2350 | partitioning protein ParB; K03497 chromosome partitioning protein, ParB family |  |
| bps:BPSS2351 | replication protein |  |
| bps:BPSS0001 | hypothetical protein |  |
| bps:BPSS0002 | integrase |  |
| bps:BPSS0003 | hypothetical protein |  |
| bps:BPSS0004 | DNA-binding protein |  |
| bps:BPSS0005 | kbl; 2-amino-3-ketobutyrate CoA ligase (EC:2.3.1.29); K00639 glycine C-acetyltransferase [EC:2.3.1.29] | ec:2.3.1.29 |
| bps:BPSS0006 | tdh; L-threonine 3-dehydrogenase (EC:1.1.1.103); K00060 threonine 3-dehydrogenase [EC:1.1.1.103] | ec:1.1.1.103 |
| bps:BPSS0007 | hypothetical protein |  |
| bps:BPSS0008 | TetR family transcriptional regulator |  |
| bps:BPSS0009 | hypothetical protein; K09958 hypothetical protein |  |
| bps:BPSS0010 | hypothetical protein |  |
| bps:BPSS0011 | tartrate dehydrogenase (EC:1.1.1.93); K07246 tartrate dehydrogenase/decarboxylase / D-malate dehydrogenase [EC:1.1.1.93 4.1.1.73 1.1.1.83] | ec:1.1.1.93 ec:1.1.1.83 ec:4.1.1.73 |
| bps:BPSS0012 | LysR family regulatory protein; K16135 LysR family transcriptional regulator, transcriptional activator for dmlA |  |
| bps:BPSS0013 | hypothetical protein |  |
| bps:BPSS0014 | glutathione S-transferase like protein; K00799 glutathione S-transferase [EC:2.5.1.18] | ec:2.5.1.18 |
| bps:BPSS0015 | hypothetical protein |  |
| bps:BPSS0016 | phospholipase; K07001 NTE family protein |  |
| bps:BPSS0017 | 3-hydroxybutyrate dehydrogenase (EC:1.1.1.30); K00019 3-hydroxybutyrate dehydrogenase [EC:1.1.1.30] | ec:1.1.1.30 |
| bps:BPSS0018 | adc; acetoacetate decarboxylase (EC:4.1.1.4); K01574 acetoacetate decarboxylase [EC:4.1.1.4] | ec:4.1.1.4 |

  
**Neighborhood Representations for "bur:Bcep18194\_B3175"**  

| ID | Annotation | EC number |
| --- | --- | --- |
| bur:Bcep18194\_B3165 | undecaprenyl-diphosphatase (EC:3.6.1.27) |  |
| bur:Bcep18194\_B3166 | acetoacetate decarboxylase (EC:4.1.1.4); K01574 acetoacetate decarboxylase [EC:4.1.1.4] | ec:4.1.1.4 |
| bur:Bcep18194\_B3167 | 3-hydroxybutyrate dehydrogenase (EC:1.1.1.30); K00019 3-hydroxybutyrate dehydrogenase [EC:1.1.1.30] | ec:1.1.1.30 |
| bur:Bcep18194\_B3168 | patatin; K07001 NTE family protein |  |
| bur:Bcep18194\_B3169 | hypothetical protein |  |
| bur:Bcep18194\_B3170 | LysR family transcriptional regulator |  |
| bur:Bcep18194\_B3171 | N-acetyl-gamma-glutamyl-phosphate reductase (EC:1.2.1.38); K00145 N-acetyl-gamma-glutamyl-phosphate reductase [EC:1.2.1.38] | ec:1.2.1.38 |
| bur:Bcep18194\_B3172 | LysR family transcriptional regulator; K16135 LysR family transcriptional regulator, transcriptional activator for dmlA |  |
| bur:Bcep18194\_B3173 | tartrate dehydrogenase (EC:1.1.1.93); K07246 tartrate dehydrogenase/decarboxylase / D-malate dehydrogenase [EC:1.1.1.93 4.1.1.73 1.1.1.83] | ec:1.1.1.93 ec:1.1.1.83 ec:4.1.1.73 |
| bur:Bcep18194\_B3174 | hypothetical protein; K09958 hypothetical protein |  |
| bur:Bcep18194\_B3175 | TetR family transcriptional regulator |  |
| bur:Bcep18194\_B3176 | GCN5-related N-acetyltransferase |  |
| bur:Bcep18194\_B3177 | hypothetical protein |  |
| bur:Bcep18194\_B3178 | tdh; L-threonine 3-dehydrogenase (EC:1.1.1.103); K00060 threonine 3-dehydrogenase [EC:1.1.1.103] | ec:1.1.1.103 |
| bur:Bcep18194\_B3179 | 2-amino-3-ketobutyrate CoA ligase (EC:2.3.1.29); K00639 glycine C-acetyltransferase [EC:2.3.1.29] | ec:2.3.1.29 |
| bur:Bcep18194\_B3180 | XRE family transcriptional regulator |  |
| bur:Bcep18194\_B3181 | hypothetical protein |  |
| bur:Bcep18194\_B0001 | Phage integrase |  |
| bur:Bcep18194\_B0002 | hypothetical protein |  |
| bur:Bcep18194\_B0003 | hypothetical protein |  |
| bur:Bcep18194\_B0004 | ParB family protein; K03497 chromosome partitioning protein, ParB family |  |

  
**Neighborhood Representations for "bch:Bcen2424\_5704"**  

| ID | Annotation | EC number |
| --- | --- | --- |
| bch:Bcen2424\_5694 | parB-like partition proteins; K03497 chromosome partitioning protein, ParB family |  |
| bch:Bcen2424\_5695 | hypothetical protein |  |
| bch:Bcen2424\_5696 | hypothetical protein |  |
| bch:Bcen2424\_5697 | hypothetical protein |  |
| bch:Bcen2424\_5698 | phage integrase family protein |  |
| bch:Bcen2424\_5699 | hypothetical protein |  |
| bch:Bcen2424\_5700 | XRE family transcriptional regulator |  |
| bch:Bcen2424\_5701 | 2-amino-3-ketobutyrate CoA ligase (EC:2.3.1.29); K00639 glycine C-acetyltransferase [EC:2.3.1.29] | ec:2.3.1.29 |
| bch:Bcen2424\_5702 | tdh; L-threonine 3-dehydrogenase (EC:1.1.1.103); K00060 threonine 3-dehydrogenase [EC:1.1.1.103] | ec:1.1.1.103 |
| bch:Bcen2424\_5703 | hypothetical protein |  |
| bch:Bcen2424\_5704 | TetR family transcriptional regulator |  |
| bch:Bcen2424\_5705 | hypothetical protein; K09958 hypothetical protein |  |
| bch:Bcen2424\_5706 | hypothetical protein |  |
| bch:Bcen2424\_5707 | tartrate dehydrogenase (EC:1.1.1.93); K07246 tartrate dehydrogenase/decarboxylase / D-malate dehydrogenase [EC:1.1.1.93 4.1.1.73 1.1.1.83] | ec:1.1.1.93 ec:1.1.1.83 ec:4.1.1.73 |
| bch:Bcen2424\_5708 | LysR family transcriptional regulator; K16135 LysR family transcriptional regulator, transcriptional activator for dmlA |  |
| bch:Bcen2424\_5709 | N-acetyl-gamma-glutamyl-phosphate reductase (EC:1.2.1.38); K00145 N-acetyl-gamma-glutamyl-phosphate reductase [EC:1.2.1.38] | ec:1.2.1.38 |
| bch:Bcen2424\_5710 | LysR family transcriptional regulator |  |
| bch:Bcen2424\_5711 | hypothetical protein |  |
| bch:Bcen2424\_5712 | patatin; K07001 NTE family protein |  |
| bch:Bcen2424\_5713 | 3-hydroxybutyrate dehydrogenase (EC:1.1.1.30); K00019 3-hydroxybutyrate dehydrogenase [EC:1.1.1.30] | ec:1.1.1.30 |
| bch:Bcen2424\_5714 | acetoacetate decarboxylase (EC:4.1.1.4); K01574 acetoacetate decarboxylase [EC:4.1.1.4] | ec:4.1.1.4 |

  
**Neighborhood Representations for "bcm:Bcenmc03\_4534"**  

| ID | Annotation | EC number |
| --- | --- | --- |
| bcm:Bcenmc03\_4524 | PA-phosphatase-like protein |  |
| bcm:Bcenmc03\_4525 | acetoacetate decarboxylase (EC:4.1.1.4); K01574 acetoacetate decarboxylase [EC:4.1.1.4] | ec:4.1.1.4 |
| bcm:Bcenmc03\_4526 | 3-hydroxybutyrate dehydrogenase; K00019 3-hydroxybutyrate dehydrogenase [EC:1.1.1.30] | ec:1.1.1.30 |
| bcm:Bcenmc03\_4527 | patatin; K07001 NTE family protein |  |
| bcm:Bcenmc03\_4528 | hypothetical protein |  |
| bcm:Bcenmc03\_4529 | LysR family transcriptional regulator |  |
| bcm:Bcenmc03\_4530 | N-acetyl-gamma-glutamyl-phosphate reductase (EC:1.2.1.38); K00145 N-acetyl-gamma-glutamyl-phosphate reductase [EC:1.2.1.38] | ec:1.2.1.38 |
| bcm:Bcenmc03\_4531 | LysR family transcriptional regulator; K16135 LysR family transcriptional regulator, transcriptional activator for dmlA |  |
| bcm:Bcenmc03\_4532 | tartrate dehydrogenase (EC:4.1.1.73); K07246 tartrate dehydrogenase/decarboxylase / D-malate dehydrogenase [EC:1.1.1.93 4.1.1.73 1.1.1.83] | ec:1.1.1.93 ec:1.1.1.83 ec:4.1.1.73 |
| bcm:Bcenmc03\_4533 | hypothetical protein; K09958 hypothetical protein |  |
| bcm:Bcenmc03\_4534 | TetR family transcriptional regulator |  |
| bcm:Bcenmc03\_4535 | tdh; L-threonine 3-dehydrogenase; K00060 threonine 3-dehydrogenase [EC:1.1.1.103] | ec:1.1.1.103 |
| bcm:Bcenmc03\_4536 | 2-amino-3-ketobutyrate coenzyme A ligase (EC:2.3.1.29); K00639 glycine C-acetyltransferase [EC:2.3.1.29] | ec:2.3.1.29 |
| bcm:Bcenmc03\_4537 | XRE family transcriptional regulator |  |
| bcm:Bcenmc03\_4538 | hypothetical protein |  |
| bcm:Bcenmc03\_4539 | integrase family protein |  |
| bcm:Bcenmc03\_4540 | hypothetical protein |  |
| bcm:Bcenmc03\_4541 | hypothetical protein |  |
| bcm:Bcenmc03\_4542 | parB-like partition protein; K03497 chromosome partitioning protein, ParB family |  |
| bcm:Bcenmc03\_4543 | cobyrinic acid ac-diamide synthase; K03496 chromosome partitioning protein |  |
| bcm:Bcenmc03\_4544 | arsenate reductase; K00537 arsenate reductase [EC:1.20.4.1] | ec:1.20.4.1 |

  
**Neighborhood Representations for "bcn:Bcen\_5155"**  

| ID | Annotation | EC number |
| --- | --- | --- |
| bcn:Bcen\_5145 | acetoacetate decarboxylase (EC:4.1.1.4); K01574 acetoacetate decarboxylase [EC:4.1.1.4] | ec:4.1.1.4 |
| bcn:Bcen\_5146 | 3-hydroxybutyrate dehydrogenase (EC:1.1.1.30); K00019 3-hydroxybutyrate dehydrogenase [EC:1.1.1.30] | ec:1.1.1.30 |
| bcn:Bcen\_5147 | patatin; K07001 NTE family protein |  |
| bcn:Bcen\_5148 | hypothetical protein |  |
| bcn:Bcen\_5149 | LysR family transcriptional regulator |  |
| bcn:Bcen\_5150 | N-acetyl-gamma-glutamyl-phosphate reductase (EC:1.2.1.38); K00145 N-acetyl-gamma-glutamyl-phosphate reductase [EC:1.2.1.38] | ec:1.2.1.38 |
| bcn:Bcen\_5151 | LysR family transcriptional regulator; K16135 LysR family transcriptional regulator, transcriptional activator for dmlA |  |
| bcn:Bcen\_5152 | tartrate dehydrogenase (EC:1.1.1.93); K07246 tartrate dehydrogenase/decarboxylase / D-malate dehydrogenase [EC:1.1.1.93 4.1.1.73 1.1.1.83] | ec:1.1.1.93 ec:1.1.1.83 ec:4.1.1.73 |
| bcn:Bcen\_5153 | hypothetical protein |  |
| bcn:Bcen\_5154 | hypothetical protein; K09958 hypothetical protein |  |
| bcn:Bcen\_5155 | TetR family transcriptional regulator |  |
| bcn:Bcen\_5156 | hypothetical protein |  |
| bcn:Bcen\_5157 | tdh; L-threonine 3-dehydrogenase (EC:1.1.1.103); K00060 threonine 3-dehydrogenase [EC:1.1.1.103] | ec:1.1.1.103 |
| bcn:Bcen\_5158 | 2-amino-3-ketobutyrate coenzyme A ligase (EC:2.3.1.29); K00639 glycine C-acetyltransferase [EC:2.3.1.29] | ec:2.3.1.29 |
| bcn:Bcen\_5159 | XRE family transcriptional regulator |  |
| bcn:Bcen\_5160 | hypothetical protein |  |
| bcn:Bcen\_5161 | phage integrase |  |
| bcn:Bcen\_5162 | hypothetical protein |  |
| bcn:Bcen\_5163 | hypothetical protein |  |
| bcn:Bcen\_5164 | hypothetical protein |  |
| bcn:Bcen\_5165 | parB-like partition proteins; K03497 chromosome partitioning protein, ParB family |  |

  
**Neighborhood Representations for "bac:BamMC406\_3117"**  

| ID | Annotation | EC number |
| --- | --- | --- |
| bac:BamMC406\_3107 | parB-like partition protein; K03497 chromosome partitioning protein, ParB family |  |
| bac:BamMC406\_3108 | hypothetical protein |  |
| bac:BamMC406\_3109 | hypothetical protein |  |
| bac:BamMC406\_3110 | pseudogene |  |
| bac:BamMC406\_3111 | hypothetical protein |  |
| bac:BamMC406\_3112 | integrase family protein |  |
| bac:BamMC406\_3113 | hypothetical protein |  |
| bac:BamMC406\_3114 | XRE family transcriptional regulator |  |
| bac:BamMC406\_3115 | 2-amino-3-ketobutyrate coenzyme A ligase (EC:2.3.1.29); K00639 glycine C-acetyltransferase [EC:2.3.1.29] | ec:2.3.1.29 |
| bac:BamMC406\_3116 | tdh; L-threonine 3-dehydrogenase; K00060 threonine 3-dehydrogenase [EC:1.1.1.103] | ec:1.1.1.103 |
| bac:BamMC406\_3117 | TetR family transcriptional regulator |  |
| bac:BamMC406\_3118 | hypothetical protein; K09958 hypothetical protein |  |
| bac:BamMC406\_3119 | tartrate dehydrogenase (EC:1.1.1.93); K07246 tartrate dehydrogenase/decarboxylase / D-malate dehydrogenase [EC:1.1.1.93 4.1.1.73 1.1.1.83] | ec:1.1.1.93 ec:1.1.1.83 ec:4.1.1.73 |
| bac:BamMC406\_3120 | LysR family transcriptional regulator; K16135 LysR family transcriptional regulator, transcriptional activator for dmlA |  |
| bac:BamMC406\_3121 | N-acetyl-gamma-glutamyl-phosphate reductase (EC:1.2.1.38); K00145 N-acetyl-gamma-glutamyl-phosphate reductase [EC:1.2.1.38] | ec:1.2.1.38 |
| bac:BamMC406\_3122 | LysR family transcriptional regulator |  |
| bac:BamMC406\_3123 | hypothetical protein |  |
| bac:BamMC406\_3124 | patatin; K07001 NTE family protein |  |
| bac:BamMC406\_3125 | 3-hydroxybutyrate dehydrogenase; K00019 3-hydroxybutyrate dehydrogenase [EC:1.1.1.30] | ec:1.1.1.30 |
| bac:BamMC406\_3126 | acetoacetate decarboxylase (EC:4.1.1.4); K01574 acetoacetate decarboxylase [EC:4.1.1.4] | ec:4.1.1.4 |
| bac:BamMC406\_3127 | PA-phosphatase-like phosphoesterase |  |

  
**Neighborhood Representations for "bam:Bamb\_4974"**  

| ID | Annotation | EC number |
| --- | --- | --- |
| bam:Bamb\_4964 | arsenate reductase; K00537 arsenate reductase [EC:1.20.4.1] | ec:1.20.4.1 |
| bam:Bamb\_4965 | cobyrinic acid a,c-diamide synthase; K03496 chromosome partitioning protein |  |
| bam:Bamb\_4966 | parB-like partition proteins; K03497 chromosome partitioning protein, ParB family |  |
| bam:Bamb\_4967 | hypothetical protein |  |
| bam:Bamb\_4968 | hypothetical protein |  |
| bam:Bamb\_4969 | phage integrase family protein |  |
| bam:Bamb\_4970 | hypothetical protein |  |
| bam:Bamb\_4971 | XRE family transcriptional regulator |  |
| bam:Bamb\_4972 | 2-amino-3-ketobutyrate CoA ligase (EC:2.3.1.29); K00639 glycine C-acetyltransferase [EC:2.3.1.29] | ec:2.3.1.29 |
| bam:Bamb\_4973 | tdh; L-threonine 3-dehydrogenase (EC:1.1.1.103); K00060 threonine 3-dehydrogenase [EC:1.1.1.103] | ec:1.1.1.103 |
| bam:Bamb\_4974 | TetR family transcriptional regulator |  |
| bam:Bamb\_4975 | hypothetical protein; K09958 hypothetical protein |  |
| bam:Bamb\_4976 | tartrate dehydrogenase (EC:1.1.1.93); K07246 tartrate dehydrogenase/decarboxylase / D-malate dehydrogenase [EC:1.1.1.93 4.1.1.73 1.1.1.83] | ec:1.1.1.93 ec:1.1.1.83 ec:4.1.1.73 |
| bam:Bamb\_4977 | LysR family transcriptional regulator; K16135 LysR family transcriptional regulator, transcriptional activator for dmlA |  |
| bam:Bamb\_4978 | N-acetyl-gamma-glutamyl-phosphate reductase (EC:1.2.1.38); K00145 N-acetyl-gamma-glutamyl-phosphate reductase [EC:1.2.1.38] | ec:1.2.1.38 |
| bam:Bamb\_4979 | LysR family transcriptional regulator |  |
| bam:Bamb\_4980 | hypothetical protein |  |
| bam:Bamb\_4981 | patatin; K07001 NTE family protein |  |
| bam:Bamb\_4982 | 3-hydroxybutyrate dehydrogenase (EC:1.1.1.30); K00019 3-hydroxybutyrate dehydrogenase [EC:1.1.1.30] | ec:1.1.1.30 |
| bam:Bamb\_4983 | acetoacetate decarboxylase (EC:4.1.1.4); K01574 acetoacetate decarboxylase [EC:4.1.1.4] | ec:4.1.1.4 |
| bam:Bamb\_4984 | PA-phosphatase-like phosphoesterase |  |

  
**Neighborhood Representations for "bcj:BCAM0014"**  

| ID | Annotation | EC number |
| --- | --- | --- |
| bcj:BCAM0004 | putative partitioning protein ParB; K03497 chromosome partitioning protein, ParB family |  |
| bcj:BCAM0005 | putative replication protein |  |
| bcj:BCAM0006 | hypothetical protein |  |
| bcj:BCAM0007 | putative phage integrase |  |
| bcj:BCAM0008 | hypothetical protein |  |
| bcj:BCAM0009 | putative DNA-binding protein |  |
| bcj:BCAM0010 | kbl; 2-amino-3-ketobutyrate coenzyme A ligase (EC:2.3.1.29); K00639 glycine C-acetyltransferase [EC:2.3.1.29] | ec:2.3.1.29 |
| bcj:BCAM0011 | tdh; L-threonine 3-dehydrogenase (EC:1.1.1.103); K00060 threonine 3-dehydrogenase [EC:1.1.1.103] | ec:1.1.1.103 |
| bcj:BCAM0012 | hypothetical protein |  |
| bcj:BCAM0013 | putative acetyltransferase |  |
| bcj:BCAM0014 | TetR family regulatory protein |  |
| bcj:BCAM0015 | hypothetical protein; K09958 hypothetical protein |  |
| bcj:BCAM0016 | tartrate dehydrogenase (EC:1.1.1.93); K07246 tartrate dehydrogenase/decarboxylase / D-malate dehydrogenase [EC:1.1.1.93 4.1.1.73 1.1.1.83] | ec:1.1.1.93 ec:1.1.1.83 ec:4.1.1.73 |
| bcj:BCAM0017 | LysR family regulatory protein; K16135 LysR family transcriptional regulator, transcriptional activator for dmlA |  |
| bcj:BCAM0018 | argC; N-acetyl-gamma-glutamyl-phosphate reductase (EC:1.2.1.38); K00145 N-acetyl-gamma-glutamyl-phosphate reductase [EC:1.2.1.38] | ec:1.2.1.38 |
| bcj:BCAM0019 | LysR family regulatory protein |  |
| bcj:BCAM0020 | hypothetical protein |  |
| bcj:BCAM0021 | putative patatin-like phospholipase; K07001 NTE family protein |  |
| bcj:BCAM0022 | 3-hydroxybutyrate dehydrogenase; K00019 3-hydroxybutyrate dehydrogenase [EC:1.1.1.30] | ec:1.1.1.30 |
| bcj:BCAM0023 | adc; acetoacetate decarboxylase (EC:4.1.1.4); K01574 acetoacetate decarboxylase [EC:4.1.1.4] | ec:4.1.1.4 |
| bcj:BCAM0024 | putative undecaprenyl-diphosphatase (EC:3.6.1.27) |  |

  
**Neighborhood Representations for "bvi:Bcep1808\_3786"**  

| ID | Annotation | EC number |
| --- | --- | --- |
| bvi:Bcep1808\_3776 | undecaprenyl-diphosphatase (EC:3.6.1.27) |  |
| bvi:Bcep1808\_3777 | acetoacetate decarboxylase (EC:4.1.1.4); K01574 acetoacetate decarboxylase [EC:4.1.1.4] | ec:4.1.1.4 |
| bvi:Bcep1808\_3778 | 3-hydroxybutyrate dehydrogenase (EC:1.1.1.30); K00019 3-hydroxybutyrate dehydrogenase [EC:1.1.1.30] | ec:1.1.1.30 |
| bvi:Bcep1808\_3779 | patatin; K07001 NTE family protein |  |
| bvi:Bcep1808\_3780 | hypothetical protein |  |
| bvi:Bcep1808\_3781 | LysR family transcriptional regulator |  |
| bvi:Bcep1808\_3782 | N-acetyl-gamma-glutamyl-phosphate reductase (EC:1.2.1.38); K00145 N-acetyl-gamma-glutamyl-phosphate reductase [EC:1.2.1.38] | ec:1.2.1.38 |
| bvi:Bcep1808\_3783 | LysR family transcriptional regulator; K16135 LysR family transcriptional regulator, transcriptional activator for dmlA |  |
| bvi:Bcep1808\_3784 | tartrate dehydrogenase (EC:1.1.1.93); K07246 tartrate dehydrogenase/decarboxylase / D-malate dehydrogenase [EC:1.1.1.93 4.1.1.73 1.1.1.83] | ec:1.1.1.93 ec:1.1.1.83 ec:4.1.1.73 |
| bvi:Bcep1808\_3785 | hypothetical protein; K09958 hypothetical protein |  |
| bvi:Bcep1808\_3786 | TetR family transcriptional regulator |  |
| bvi:Bcep1808\_3787 | tdh; L-threonine 3-dehydrogenase (EC:1.1.1.103); K00060 threonine 3-dehydrogenase [EC:1.1.1.103] | ec:1.1.1.103 |
| bvi:Bcep1808\_3788 | 2-amino-3-ketobutyrate CoA ligase (EC:2.3.1.29); K00639 glycine C-acetyltransferase [EC:2.3.1.29] | ec:2.3.1.29 |
| bvi:Bcep1808\_3789 | XRE family transcriptional regulator |  |
| bvi:Bcep1808\_3790 | hypothetical protein |  |
| bvi:Bcep1808\_3791 | phage integrase family protein |  |
| bvi:Bcep1808\_3792 | hypothetical protein |  |
| bvi:Bcep1808\_3793 | hypothetical protein |  |
| bvi:Bcep1808\_3794 | ParB family protein; K03497 chromosome partitioning protein, ParB family |  |
| bvi:Bcep1808\_3795 | cobyrinic acid a,c-diamide synthase; K03496 chromosome partitioning protein |  |
| bvi:Bcep1808\_3796 | response regulator receiver protein; K00537 arsenate reductase [EC:1.20.4.1] | ec:1.20.4.1 |

  
**Neighborhood Representations for "bmj:BMULJ\_03202"**  

| ID | Annotation | EC number |
| --- | --- | --- |
| bmj:BMULJ\_03192 | hypothetical protein; K07117 |  |
| bmj:BMULJ\_03193 | fadH; NADPH2-dependent 2,4-dienoyl-CoA reductase (EC:1.3.1.34) |  |
| bmj:BMULJ\_03194 | guaA; glutamine amidotransferase (EC:6.3.5.2); K01951 GMP synthase (glutamine-hydrolysing) [EC:6.3.5.2] | ec:6.3.5.2 |
| bmj:BMULJ\_03195 | putative MarR-family transcriptional regulator |  |
| bmj:BMULJ\_03196 | smc; SMC protein |  |
| bmj:BMULJ\_03197 | tyrosine recombinase |  |
| bmj:BMULJ\_03198 | serine/threonine protein kinase |  |
| bmj:BMULJ\_03199 | XRE family transcriptional regulator |  |
| bmj:BMULJ\_03200 | kbl; 2-amino-3-ketobutyrate CoA ligase (EC:2.3.1.29); K00639 glycine C-acetyltransferase [EC:2.3.1.29] | ec:2.3.1.29 |
| bmj:BMULJ\_03201 | tdh; L-threonine 3-dehydrogenase (EC:1.1.1.103); K00060 threonine 3-dehydrogenase [EC:1.1.1.103] | ec:1.1.1.103 |
| bmj:BMULJ\_03202 | TetR family transcriptional regulator |  |
| bmj:BMULJ\_03203 | hypothetical protein; K09958 hypothetical protein |  |
| bmj:BMULJ\_03204 | ttuC; tartrate dehydrogenase (EC:1.1.1.93); K07246 tartrate dehydrogenase/decarboxylase / D-malate dehydrogenase [EC:1.1.1.93 4.1.1.73 1.1.1.83] | ec:1.1.1.93 ec:1.1.1.83 ec:4.1.1.73 |
| bmj:BMULJ\_03205 | LysR family transcriptional regulator; K16135 LysR family transcriptional regulator, transcriptional activator for dmlA |  |
| bmj:BMULJ\_03206 | hypothetical protein |  |
| bmj:BMULJ\_03207 | hypothetical protein |  |
| bmj:BMULJ\_03208 | patatin-like phospholipase; K07001 NTE family protein |  |
| bmj:BMULJ\_03209 | bdh; 3-hydroxybutyrate dehydrogenase (EC:1.1.1.30); K00019 3-hydroxybutyrate dehydrogenase [EC:1.1.1.30] | ec:1.1.1.30 |
| bmj:BMULJ\_03210 | adc; acetoacetate decarboxylase (EC:4.1.1.4); K01574 acetoacetate decarboxylase [EC:4.1.1.4] | ec:4.1.1.4 |
| bmj:BMULJ\_03211 | membrane-associated phospholipid phosphatase |  |
| bmj:BMULJ\_03212 | hypothetical protein |  |

  
**Neighborhood Representations for "bmu:Bmul\_5319"**  

| ID | Annotation | EC number |
| --- | --- | --- |
| bmu:Bmul\_5309 | hypothetical protein |  |
| bmu:Bmul\_5310 | PA-phosphatase like phosphoesterase |  |
| bmu:Bmul\_5311 | acetoacetate decarboxylase (EC:4.1.1.4); K01574 acetoacetate decarboxylase [EC:4.1.1.4] | ec:4.1.1.4 |
| bmu:Bmul\_5312 | 3-hydroxybutyrate dehydrogenase; K00019 3-hydroxybutyrate dehydrogenase [EC:1.1.1.30] | ec:1.1.1.30 |
| bmu:Bmul\_5313 | patatin; K07001 NTE family protein |  |
| bmu:Bmul\_5314 | hypothetical protein |  |
| bmu:Bmul\_5315 | hypothetical protein |  |
| bmu:Bmul\_5316 | LysR family transcriptional regulator; K16135 LysR family transcriptional regulator, transcriptional activator for dmlA |  |
| bmu:Bmul\_5317 | tartrate dehydrogenase (EC:1.1.1.93); K07246 tartrate dehydrogenase/decarboxylase / D-malate dehydrogenase [EC:1.1.1.93 4.1.1.73 1.1.1.83] | ec:1.1.1.93 ec:1.1.1.83 ec:4.1.1.73 |
| bmu:Bmul\_5318 | hypothetical protein; K09958 hypothetical protein |  |
| bmu:Bmul\_5319 | TetR family transcriptional regulator |  |
| bmu:Bmul\_5320 | tdh; L-threonine 3-dehydrogenase; K00060 threonine 3-dehydrogenase [EC:1.1.1.103] | ec:1.1.1.103 |
| bmu:Bmul\_5321 | 2-amino-3-ketobutyrate CoA ligase (EC:2.3.1.29); K00639 glycine C-acetyltransferase [EC:2.3.1.29] | ec:2.3.1.29 |
| bmu:Bmul\_5322 | XRE family transcriptional regulator |  |
| bmu:Bmul\_5323 | hypothetical protein |  |
| bmu:Bmul\_5324 | hypothetical protein |  |
| bmu:Bmul\_5325 | LysR family transcriptional regulator; K03566 LysR family transcriptional regulator, glycine cleavage system transcriptional activator |  |
| bmu:Bmul\_5326 | adenylosuccinate synthetase (EC:6.3.4.4); K01939 adenylosuccinate synthase [EC:6.3.4.4] | ec:6.3.4.4 |
| bmu:Bmul\_5327 | LysR family transcriptional regulator |  |
| bmu:Bmul\_5328 | carboxymethylenebutenolidase (EC:3.1.1.45); K01061 carboxymethylenebutenolidase [EC:3.1.1.45] | ec:3.1.1.45 |
| bmu:Bmul\_5329 | hypothetical protein |  |

  
**Neighborhood Representations for "bgl:bglu\_2g00130"**  

| ID | Annotation | EC number |
| --- | --- | --- |
| bgl:bglu\_2g00030 | putative plasmid replication protein |  |
| bgl:bglu\_2g00040 | pseudogene |  |
| bgl:bglu\_2g00050 | chromosome segregation ATPase |  |
| bgl:bglu\_2g00060 | phage integrase family site-specific recombinase |  |
| bgl:bglu\_2g00070 | Serine/threonine protein kinase |  |
| bgl:bglu\_2g00080 | XRE family transcriptional regulator |  |
| bgl:bglu\_2g00090 | 2-amino-3-ketobutyrate coenzyme A ligase; K00639 glycine C-acetyltransferase [EC:2.3.1.29] | ec:2.3.1.29 |
| bgl:bglu\_2g00100 | L-threonine 3-dehydrogenase; K00060 threonine 3-dehydrogenase [EC:1.1.1.103] | ec:1.1.1.103 |
| bgl:bglu\_2g00110 | GNAT family acetyltransferase |  |
| bgl:bglu\_2g00120 | hypothetical protein |  |
| bgl:bglu\_2g00130 | TetR family transcriptional regulator |  |
| bgl:bglu\_2g00140 | hypothetical protein; K09958 hypothetical protein |  |
| bgl:bglu\_2g00150 | Tartrate dehydrogenase; K07246 tartrate dehydrogenase/decarboxylase / D-malate dehydrogenase [EC:1.1.1.93 4.1.1.73 1.1.1.83] | ec:1.1.1.93 ec:1.1.1.83 ec:4.1.1.73 |
| bgl:bglu\_2g00160 | LysR family transcriptional regulator; K16135 LysR family transcriptional regulator, transcriptional activator for dmlA |  |
| bgl:bglu\_2g00170 | GntR family transcriptional regulator |  |
| bgl:bglu\_2g00180 | mandelate racemase/muconate lactonizing enzyme family protein; K01706 glucarate dehydratase [EC:4.2.1.40] | ec:4.2.1.40 |
| bgl:bglu\_2g00190 | 5-dehydro-4-deoxyglucarate dehydratase; K01707 5-dehydro-4-deoxyglucarate dehydratase [EC:4.2.1.41] | ec:4.2.1.41 |
| bgl:bglu\_2g00200 | hypothetical protein |  |
| bgl:bglu\_2g00210 | patatin family phospholipase; K07001 NTE family protein |  |
| bgl:bglu\_2g00220 | 3-hydroxybutyrate dehydrogenase; K00019 3-hydroxybutyrate dehydrogenase [EC:1.1.1.30] | ec:1.1.1.30 |
| bgl:bglu\_2g00230 | acetoacetate decarboxylase; K01574 acetoacetate decarboxylase [EC:4.1.1.4] | ec:4.1.1.4 |

  
**Neighborhood Representations for "pay:PAU\_02084"**  

| ID | Annotation | EC number |
| --- | --- | --- |
| pay:PAU\_02074 | topA; DNA topoisomerase (EC:5.99.1.2); K03168 DNA topoisomerase I [EC:5.99.1.2] | ec:5.99.1.2 |
| pay:PAU\_02075 | cysB; hth-type transcriptional regulator cysb (cys regulon transcriptiona activator); K13634 LysR family transcriptional regulator, cys regulon transcriptional activator |  |
| pay:PAU\_02076 | hypothetical protein |  |
| pay:PAU\_02077 | acnA; aconitate hydrase 1; K01681 aconitate hydratase [EC:4.2.1.3] | ec:4.2.1.3 |
| pay:PAU\_02078 | ribA; gtp cyclohydrolase-2 (EC:3.5.4.25); K01497 GTP cyclohydrolase II [EC:3.5.4.25] | ec:3.5.4.25 |
| pay:PAU\_02079 | pgpB; phosphatidylglycerophosphatase B (EC:3.1.3.27) |  |
| pay:PAU\_02080 | yciS; putative inner membrane protein ycis; K08992 putative membrane protein |  |
| pay:PAU\_02081 | yciM; hypothetical protein |  |
| pay:PAU\_02082 | pyrF; orotidine 5'-phosphate decarboxylase (EC:4.1.1.23); K01591 orotidine-5'-phosphate decarboxylase [EC:4.1.1.23] | ec:4.1.1.23 |
| pay:PAU\_02083 | yciH; bacterial translation initiation factor SUI1; K03113 translation initiation factor 1 |  |
| pay:PAU\_02084 | transcriptional regulator, tetr family |  |
| pay:PAU\_02085 | hypothetical protein; K09958 hypothetical protein |  |
| pay:PAU\_02086 | hypothetical protein; K07154 serine/threonine-protein kinase HipA [EC:2.7.11.1] | ec:2.7.11.1 |
| pay:PAU\_02087 | hypothetical protein |  |
| pay:PAU\_02088 | Filamentation induced by cAMP protein Fic |  |
| pay:PAU\_02089 | dnt; cytotoxic necrotizing factor |  |
| pay:PAU\_02090 | nitrilase (EC:3.5.5.7); K01502 aliphatic nitrilase [EC:3.5.5.7] | ec:3.5.5.7 |
| pay:PAU\_02091 | hypothetical protein |  |
| pay:PAU\_02092 | hypothetical protein |  |
| pay:PAU\_02093 | hypothetical protein |  |
| pay:PAU\_02094 | hypothetical protein |  |

  
**Neighborhood Representations for "smt:Smal\_2003"**  

| ID | Annotation | EC number |
| --- | --- | --- |
| smt:Smal\_1993 | putative xanthine dehydrogenase iron-sulfur-binding subunit; K13483 xanthine dehydrogenase YagT iron-sulfur-binding subunit |  |
| smt:Smal\_1994 | OsmC family protein |  |
| smt:Smal\_1995 | NmrA family protein |  |
| smt:Smal\_1996 | LysR family transcriptional regulator (EC:4.2.1.1) |  |
| smt:Smal\_1997 | ECF subfamily RNA polymerase sigma-24 subunit |  |
| smt:Smal\_1998 | alkylhydroperoxidase-like protein |  |
| smt:Smal\_1999 | cupin |  |
| smt:Smal\_2000 | ECF subfamily RNA polymerase sigma-24 subunit; K03088 RNA polymerase sigma-70 factor, ECF subfamily |  |
| smt:Smal\_2001 | beta-lactamase domain-containing protein |  |
| smt:Smal\_2002 | hypothetical protein; K09958 hypothetical protein |  |
| smt:Smal\_2003 | TetR family transcriptional regulator |  |
| smt:Smal\_2004 | alpha/beta hydrolase fold domain-containing protein; K00433 Non-heme chloroperoxidase [EC:1.11.1.10] | ec:1.11.1.10 |
| smt:Smal\_2005 | TetR family transcriptional regulator; K16137 TetR/AcrR family transcriptional regulator, transcriptional repressor for nem operon |  |
| smt:Smal\_2006 | NADH:flavin oxidoreductase; K10680 N-ethylmaleimide reductase [EC:1.-.-.-] |  |
| smt:Smal\_2007 | short-chain dehydrogenase/reductase SDR; K07124 |  |
| smt:Smal\_2008 | alcohol dehydrogenase zinc-binding domain-containing protein |  |
| smt:Smal\_2009 | excinuclease ABC subunit A; K03701 excinuclease ABC subunit A |  |
| smt:Smal\_2010 | LysR family transcriptional regulator (EC:4.2.1.1) |  |
| smt:Smal\_2011 | major facilitator superfamily protein |  |
| smt:Smal\_2012 | aldo/keto reductase |  |
| smt:Smal\_2013 | cupin |  |

  
**Neighborhood Representations for "reu:Reut\_B4516"**  

| ID | Annotation | EC number |
| --- | --- | --- |
| reu:Reut\_B4506 | molybdopterin dehydrogenase :CO dehydrogenase flavoprotein, C-terminal; K11178 xanthine dehydrogenase YagS FAD-binding subunit [EC:1.17.1.4] | ec:1.17.1.4 |
| reu:Reut\_B4507 | xanthine dehydrogenase, molybdenum binding subunit apoprotein (EC:1.17.1.4); K11177 xanthine dehydrogenase YagR molybdenum-binding subunit [EC:1.17.1.4] | ec:1.17.1.4 |
| reu:Reut\_B4508 | MltA-interacting MipA |  |
| reu:Reut\_B4509 | hypothetical protein |  |
| reu:Reut\_B4510 | hypothetical protein |  |
| reu:Reut\_B4511 | hypothetical protein |  |
| reu:Reut\_B4512 | fructose-bisphosphate aldolase (EC:4.1.2.13); K01623 fructose-bisphosphate aldolase, class I [EC:4.1.2.13] | ec:4.1.2.13 |
| reu:Reut\_B4513 | cytochrome B561 |  |
| reu:Reut\_B4514 | signal peptide protein |  |
| reu:Reut\_B4515 | hypothetical protein; K09958 hypothetical protein |  |
| reu:Reut\_B4516 | TetR family transcriptional regulator |  |
| reu:Reut\_B4517 | regulatory protein IclR |  |
| reu:Reut\_B4518 | L-carnitine dehydratase/bile acid-inducible protein F |  |
| reu:Reut\_B4519 | acyl-CoA dehydrogenase; K00249 acyl-CoA dehydrogenase [EC:1.3.8.7] | ec:1.3.8.7 |
| reu:Reut\_B4520 | hypothetical protein |  |
| reu:Reut\_B4521 | hypothetical protein |  |
| reu:Reut\_B4522 | uracil-DNA glycosylase |  |
| reu:Reut\_B4523 | dnaE2; error-prone DNA polymerase (EC:2.7.7.7); K14162 error-prone DNA polymerase [EC:2.7.7.7] | ec:2.7.7.7 |
| reu:Reut\_B4524 | hypothetical protein; K14161 protein ImuB |  |
| reu:Reut\_B4525 | hypothetical protein; K14160 protein ImuA |  |
| reu:Reut\_B4526 | enoyl-CoA hydratase (EC:4.2.1.17) |  |

  
**Neighborhood Representations for "ebi:EbC\_28440"**  

| ID | Annotation | EC number |
| --- | --- | --- |
| ebi:EbC\_28340 | FAD dependent D-amino acid oxidase |  |
| ebi:EbC\_28350 | 2-haloacid halidohydrolase IVa; K01560 2-haloacid dehalogenase [EC:3.8.1.2] | ec:3.8.1.2 |
| ebi:EbC\_28360 | betaine aldehyde dehydrogenase; K00128 aldehyde dehydrogenase (NAD+) [EC:1.2.1.3] | ec:1.2.1.3 |
| ebi:EbC\_28370 | succinate-semialdehyde dehydrogenase; K00135 succinate-semialdehyde dehydrogenase / glutarate-semialdehyde dehydrogenase [EC:1.2.1.16 1.2.1.79 1.2.1.20] | ec:1.2.1.79 ec:1.2.1.16 ec:1.2.1.20 |
| ebi:EbC\_28380 | N-acetyltransferase GCN5 |  |
| ebi:EbC\_28390 | N-acetyltransferase GCN5 |  |
| ebi:EbC\_28400 | LysR family transcripitonal regulator |  |
| ebi:EbC\_28410 | esterase/lipase |  |
| ebi:EbC\_28420 | hypothetical protein; K09702 hypothetical protein |  |
| ebi:EbC\_28430 | hypothetical protein; K09958 hypothetical protein |  |
| ebi:EbC\_28440 | TetR family transcripitonal regulator |  |
| ebi:EbC\_28450 | short-chain dehydrogenase |  |
| ebi:EbC\_28460 | NADH:flavin oxidoreductase; K10680 N-ethylmaleimide reductase [EC:1.-.-.-] |  |
| ebi:EbC\_28470 | TetR family transcripitonal regulator; K16137 TetR/AcrR family transcriptional regulator, transcriptional repressor for nem operon |  |
| ebi:EbC\_28480 | TetR family transcripitonal regulator |  |
| ebi:EbC\_28490 | hypothetical protein |  |
| ebi:EbC\_28500 | hypothetical protein |  |
| ebi:EbC\_28510 | LysR family transcripitonal regulator |  |
| ebi:EbC\_28520 | NADH-dependent flavin oxidoreductase |  |
| ebi:EbC\_28530 | N-acetyltransferase GCN5 |  |
| ebi:EbC\_28540 | zinc-binding oxidoreductase |  |

  
**Neighborhood Representations for "pfl:PFL\_3261"**  

| ID | Annotation | EC number |
| --- | --- | --- |
| pfl:PFL\_3251 | CHASE3/GAF sensor histidine kinase/response regulator |  |
| pfl:PFL\_3252 | response regulator |  |
| pfl:PFL\_3253 | hypothetical protein |  |
| pfl:PFL\_3254 | mtlR; transcriptional activator MtlR |  |
| pfl:PFL\_3255 | efeU; ferrous iron permease EfeU; K07243 high-affinity iron transporter |  |
| pfl:PFL\_3256 | efeO; periplasmic iron transport protein EfeO |  |
| pfl:PFL\_3257 | efeB; heme-containing peroxidase/deferrochelatase EfeB; K16301 deferrochelatase/peroxidase EfeB [EC:1.11.1.-] |  |
| pfl:PFL\_3258 | hypothetical protein; K07224 iron uptake system component EfeO |  |
| pfl:PFL\_3259 | major facilitator family transporter |  |
| pfl:PFL\_3260 | pssA\_2; phosphatidylserine synthase (EC:2.7.8.8); K00998 CDP-diacylglycerol---serine O-phosphatidyltransferase [EC:2.7.8.8] | ec:2.7.8.8 |
| pfl:PFL\_3261 | TetR family transcriptional regulator |  |
| pfl:PFL\_3262 | hypothetical protein; K09958 hypothetical protein |  |
| pfl:PFL\_3263 | sfnG; dimethyl sulfone monooxygenase SfnG (EC:1.14.-.-); K17228 FMNH2-dependent dimethyl sulfone monooxygenase |  |
| pfl:PFL\_3264 | acyl-CoA dehydrogenase |  |
| pfl:PFL\_3265 | hypothetical protein; K07090 |  |
| pfl:PFL\_3266 | nudC; NADH pyrophosphatase (EC:3.6.1.22); K03426 NAD+ diphosphatase [EC:3.6.1.22] | ec:3.6.1.22 |
| pfl:PFL\_3267 | enoyl-CoA hydratase; K01692 enoyl-CoA hydratase [EC:4.2.1.17] | ec:4.2.1.17 |
| pfl:PFL\_3268 | rpeA; sensor histidine kinase RpeA; K02484 two-component system, OmpR family, sensor kinase [EC:2.7.13.3] | ec:2.7.13.3 |
| pfl:PFL\_3269 | DNA-binding response regulator; K02483 two-component system, OmpR family, response regulator |  |
| pfl:PFL\_3270 | acrA; acriflavine resistance protein A; K03585 membrane fusion protein |  |
| pfl:PFL\_3271 | acrB; acriflavine resistance protein B; K03296 hydrophobic/amphiphilic exporter-1 (mainly G- bacteria), HAE1 family |  |

  
**Neighborhood Representations for "ddc:Dd586\_1390"**  

| ID | Annotation | EC number |
| --- | --- | --- |
| ddc:Dd586\_1380 | Peptidoglycan-binding lysin domain-containing protein |  |
| ddc:Dd586\_1381 | hypothetical protein |  |
| ddc:Dd586\_1382 | pseudogene |  |
| ddc:Dd586\_1383 | hypothetical protein |  |
| ddc:Dd586\_1384 | LysR family transcriptional regulator |  |
| ddc:Dd586\_1385 | alcohol dehydrogenase zinc-binding domain-containing protein |  |
| ddc:Dd586\_1386 | antibiotic biosynthesis monooxygenase |  |
| ddc:Dd586\_1387 | glyoxalase/bleomycin resistance protein/dioxygenase |  |
| ddc:Dd586\_1388 | alpha/beta hydrolase fold protein |  |
| ddc:Dd586\_1389 | hypothetical protein |  |
| ddc:Dd586\_1390 | TetR family transcriptional regulator |  |
| ddc:Dd586\_1391 | hypothetical protein; K09958 hypothetical protein |  |
| ddc:Dd586\_1392 | glutamine amidotransferase class-I; K01951 GMP synthase (glutamine-hydrolysing) [EC:6.3.5.2] | ec:6.3.5.2 |
| ddc:Dd586\_1393 | AraC family transcriptional regulator |  |
| ddc:Dd586\_1394 | HNH endonuclease |  |
| ddc:Dd586\_1395 | 2-nitropropane dioxygenase NPD; K02371 enoyl-[acyl-carrier protein] reductase II [EC:1.3.1.-] |  |
| ddc:Dd586\_1396 | LysR family transcriptional regulator |  |
| ddc:Dd586\_1397 | DSBA oxidoreductase; K03673 thiol:disulfide interchange protein DsbA |  |
| ddc:Dd586\_1398 | MATE efflux family protein |  |
| ddc:Dd586\_1399 | RND efflux system outer membrane lipoprotein |  |
| ddc:Dd586\_1400 | TetR family transcriptional regulator |  |

  
**Neighborhood Representations for "pfo:Pfl01\_2877"**  

| ID | Annotation | EC number |
| --- | --- | --- |
| pfo:Pfl01\_2867 | acyl-CoA dehydrogenase; K00257 [EC:1.3.99.-] |  |
| pfo:Pfl01\_2868 | thiolase; K00626 acetyl-CoA C-acetyltransferase [EC:2.3.1.9] | ec:2.3.1.9 |
| pfo:Pfl01\_2869 | Short-chain dehydrogenase/reductase SDR |  |
| pfo:Pfl01\_2870 | AMP-dependent synthetase/ligase; K01895 acetyl-CoA synthetase [EC:6.2.1.1] | ec:6.2.1.1 |
| pfo:Pfl01\_2871 | AraC family transcriptional regulator |  |
| pfo:Pfl01\_2872 | iron permease; K07243 high-affinity iron transporter |  |
| pfo:Pfl01\_2873 | hypothetical protein |  |
| pfo:Pfl01\_2874 | Tat-translocated enzyme; K16301 deferrochelatase/peroxidase EfeB [EC:1.11.1.-] |  |
| pfo:Pfl01\_2875 | hypothetical protein; K07224 iron uptake system component EfeO |  |
| pfo:Pfl01\_2876 | pssA; phosphatidylserine synthase (EC:2.7.8.8); K00998 CDP-diacylglycerol---serine O-phosphatidyltransferase [EC:2.7.8.8] | ec:2.7.8.8 |
| pfo:Pfl01\_2877 | TetR family transcriptional regulator |  |
| pfo:Pfl01\_2878 | hypothetical protein; K09958 hypothetical protein |  |
| pfo:Pfl01\_2879 | luciferase-like protein; K17228 FMNH2-dependent dimethyl sulfone monooxygenase |  |
| pfo:Pfl01\_2880 | hypothetical protein; K07090 |  |
| pfo:Pfl01\_2881 | nudC; NADH pyrophosphatase; K03426 NAD+ diphosphatase [EC:3.6.1.22] | ec:3.6.1.22 |
| pfo:Pfl01\_2882 | enoyl-CoA hydratase (EC:4.2.1.17); K01692 enoyl-CoA hydratase [EC:4.2.1.17] | ec:4.2.1.17 |
| pfo:Pfl01\_2883 | hypothetical protein |  |
| pfo:Pfl01\_2884 | hypothetical protein |  |
| pfo:Pfl01\_2885 | sigma-24 (FecI); K03088 RNA polymerase sigma-70 factor, ECF subfamily |  |
| pfo:Pfl01\_2886 | transmembrane transcriptional regulator |  |
| pfo:Pfl01\_2887 | nicotinamidase (EC:3.5.1.19); K08281 nicotinamidase/pyrazinamidase [EC:3.5.1.19 3.5.1.-] | ec:3.5.1.19 |

  
**Neighborhood Representations for "xcv:XCV4432"**  

| ID | Annotation | EC number |
| --- | --- | --- |
| xcv:XCV4422 | pseudouridylate synthase; K06177 tRNA pseudouridine32 synthase / 23S rRNA pseudouridine746 synthase [EC:5.4.99.28 5.4.99.29] | ec:5.4.99.28 ec:5.4.99.29 |
| xcv:XCV4423 | hypothetical protein |  |
| xcv:XCV4424 | hypothetical protein |  |
| xcv:XCV4425 | hypothetical protein |  |
| xcv:XCV4426 | hypothetical protein |  |
| xcv:XCV4427 | hypothetical protein |  |
| xcv:XCV4428 | avrRxo1; avirulence protein AvrRxo1 |  |
| xcv:XCV4429 | hypothetical protein |  |
| xcv:XCV4430 | hypothetical protein |  |
| xcv:XCV4431 | hypothetical protein; K09958 hypothetical protein |  |
| xcv:XCV4432 | TetR family transcriptional regulator |  |
| xcv:XCV4433 | TetR family transcriptional regulator |  |
| xcv:XCV4434 | short chain dehydrogenase |  |
| xcv:XCV4435 | hypothetical protein |  |
| xcv:XCV4436 | hypothetical protein |  |
| xcv:XCV4437 | aminopeptidase precursor |  |
| xcv:XCV4438 | xopQ; outer protein Q |  |
| xcv:XCV4439 | DNA-binding protein |  |
| xcv:XCV4440 | hypothetical protein; K07334 proteic killer suppression protein |  |
| xcv:XCV4441 | recD; exodeoxyribonuclease V subunit alpha (EC:3.1.11.5); K03581 exodeoxyribonuclease V alpha subunit [EC:3.1.11.5] | ec:3.1.11.5 |
| xcv:XCV4442 | recB; exodeoxyribonuclease V subunit beta (EC:3.1.11.5); K03582 exodeoxyribonuclease V beta subunit [EC:3.1.11.5] | ec:3.1.11.5 |

  
**Neighborhood Representations for "ddd:Dda3937\_03586"**  

| ID | Annotation | EC number |
| --- | --- | --- |
| ddd:Dda3937\_00643 | amino acid ABC transporter ATP-binding protein; K02028 polar amino acid transport system ATP-binding protein [EC:3.6.3.21] | ec:3.6.3.21 |
| ddd:Dda3937\_04319 | 2-hydroxy-6-oxo-6-phenylhexa-2,4-dienoate hydrolase |  |
| ddd:Dda3937\_03579 | dienelactone hydrolase-like enzyme |  |
| ddd:Dda3937\_04533 | hypothetical protein |  |
| ddd:Dda3937\_03580 | Rhs family protein |  |
| ddd:Dda3937\_03581 | hypothetical protein |  |
| ddd:Dda3937\_03582 | pyridoxamine 5'-phosphate oxidase; K07006 |  |
| ddd:Dda3937\_03583 | glyoxalase family protein |  |
| ddd:Dda3937\_03584 | ycdJ; alpha/beta fold family hydrolase |  |
| ddd:Dda3937\_03585 | hypothetical protein |  |
| ddd:Dda3937\_03586 | TetR family transcriptional regulator |  |
| ddd:Dda3937\_03587 | hypothetical protein; K09958 hypothetical protein |  |
| ddd:Dda3937\_04709 | Lipase |  |
| ddd:Dda3937\_03588 | glutamine amidotransferase; K01951 GMP synthase (glutamine-hydrolysing) [EC:6.3.5.2] | ec:6.3.5.2 |
| ddd:Dda3937\_03589 | AraC family transcriptional regulator |  |
| ddd:Dda3937\_03590 | cynS; cyanate hydratase; K01725 cyanate lyase [EC:4.2.1.104] | ec:4.2.1.104 |
| ddd:Dda3937\_03591 | carbonic anhydrase; K01673 carbonic anhydrase [EC:4.2.1.1] | ec:4.2.1.1 |
| ddd:Dda3937\_03592 | Conjugative transfer protein TrbF |  |
| ddd:Dda3937\_03593 | Conjugative transfer protein TrbI |  |
| ddd:Dda3937\_03594 | transposase |  |
| ddd:Dda3937\_03595 | Nonribosomal peptide synthetase |  |

  
**Neighborhood Representations for "pct:PC1\_2715"**  

| ID | Annotation | EC number |
| --- | --- | --- |
| pct:PC1\_2705 | hypothetical protein |  |
| pct:PC1\_2706 | hypothetical protein |  |
| pct:PC1\_2707 | XRE family transcriptional regulator; K07727 putative transcriptional regulator |  |
| pct:PC1\_2708 | hypothetical protein |  |
| pct:PC1\_2709 | hypothetical protein |  |
| pct:PC1\_2710 | hypothetical protein |  |
| pct:PC1\_2711 | lysine exporter protein LysE/YggA |  |
| pct:PC1\_2712 | AraC family transcriptional regulator |  |
| pct:PC1\_2713 | class I glutamine amidotransferase; K01951 GMP synthase (glutamine-hydrolysing) [EC:6.3.5.2] | ec:6.3.5.2 |
| pct:PC1\_2714 | hypothetical protein; K09958 hypothetical protein |  |
| pct:PC1\_2715 | TetR family transcriptional regulator |  |
| pct:PC1\_2716 | NADH:flavin oxidoreductase/NADH oxidase |  |
| pct:PC1\_2717 | putative ArsR family transcriptional regulator |  |
| pct:PC1\_2718 | carbohydrate kinase FGGY; K00854 xylulokinase [EC:2.7.1.17] | ec:2.7.1.17 |
| pct:PC1\_2719 | NAD-binding D-isomer specific 2-hydroxyacid dehydrogenase; K00058 D-3-phosphoglycerate dehydrogenase [EC:1.1.1.95] | ec:1.1.1.95 |
| pct:PC1\_2720 | PfkB domain-containing protein |  |
| pct:PC1\_2721 | inner-membrane translocator; K10440 ribose transport system permease protein |  |
| pct:PC1\_2722 | inner-membrane translocator; K10440 ribose transport system permease protein |  |
| pct:PC1\_2723 | ABC transporter-like protein; K10441 ribose transport system ATP-binding protein [EC:3.6.3.17] | ec:3.6.3.17 |
| pct:PC1\_2724 | periplasmic binding protein/LacI transcriptional regulator; K10439 ribose transport system substrate-binding protein |  |
| pct:PC1\_2725 | Acireductone dioxygenase ARD; K08967 1,2-dihydroxy-3-keto-5-methylthiopentene dioxygenase [EC:1.13.11.53 1.13.11.54] | ec:1.13.11.54 ec:1.13.11.53 |

  
**Neighborhood Representations for "pwa:Pecwa\_1371"**  

| ID | Annotation | EC number |
| --- | --- | --- |
| pwa:Pecwa\_1361 | lipoprotein |  |
| pwa:Pecwa\_1362 | poly(glycerophosphate chain) D-alanine transfer protein; K03740 D-alanine transfer protein |  |
| pwa:Pecwa\_1363 | hypothetical protein |  |
| pwa:Pecwa\_1364 | membrane bound O-acyl transferase MBOAT family protein; K03739 membrane protein involved in D-alanine export |  |
| pwa:Pecwa\_1365 | AMP-dependent synthetase and ligase; K03367 D-alanine--poly(phosphoribitol) ligase subunit 1 [EC:6.1.1.13] | ec:6.1.1.13 |
| pwa:Pecwa\_1366 | acyl carrier protein; K14188 D-alanine--poly(phosphoribitol) ligase subunit 2 [EC:6.1.1.13] | ec:6.1.1.13 |
| pwa:Pecwa\_1367 | 5-methyltetrahydropteroyltriglutamate/homocysteine S-methyltransferase; K00549 5-methyltetrahydropteroyltriglutamate--homocysteine methyltransferase [EC:2.1.1.14] | ec:2.1.1.14 |
| pwa:Pecwa\_1368 | hypothetical protein |  |
| pwa:Pecwa\_1369 | pseudogene |  |
| pwa:Pecwa\_1370 | NUDIX hydrolase |  |
| pwa:Pecwa\_1371 | TetR family transcriptional regulator |  |
| pwa:Pecwa\_1372 | pseudogene |  |
| pwa:Pecwa\_1373 | pseudogene |  |
| pwa:Pecwa\_1374 | transposase IS3/IS911 family protein |  |
| pwa:Pecwa\_1375 | pseudogene |  |
| pwa:Pecwa\_1376 | hypothetical protein |  |
| pwa:Pecwa\_1377 | integrase |  |
| pwa:Pecwa\_1378 | pseudogene |  |
| pwa:Pecwa\_1379 | hypothetical protein |  |
| pwa:Pecwa\_1380 | hypothetical protein |  |
| pwa:Pecwa\_1381 | hypothetical protein |  |

  
**Neighborhood Representations for "ppf:Pput\_2721"**  

| ID | Annotation | EC number |
| --- | --- | --- |
| ppf:Pput\_2711 | LysR family transcriptional regulator |  |
| ppf:Pput\_2712 | helix-turn-helix domain-containing protein |  |
| ppf:Pput\_2713 | peroxidase-like protein |  |
| ppf:Pput\_2714 | glutaredoxin 3; K03676 glutaredoxin 3 |  |
| ppf:Pput\_2715 | N-acetyltransferase GCN5 |  |
| ppf:Pput\_2716 | RimK domain-containing protein ATP-grasp |  |
| ppf:Pput\_2717 | CorA family protein Mg2+ transporter protein; K03284 magnesium transporter |  |
| ppf:Pput\_2718 | antibiotic biosynthesis monooxygenase |  |
| ppf:Pput\_2719 | zinc-binding alcohol dehydrogenase |  |
| ppf:Pput\_2720 | LysR family transcriptional regulator |  |
| ppf:Pput\_2721 | TetR family transcriptional regulator |  |
| ppf:Pput\_2722 | hypothetical protein; K09958 hypothetical protein |  |
| ppf:Pput\_2723 | helix-turn-helix domain-containing protein |  |
| ppf:Pput\_2724 | phenylacetaldoxime dehydratase; K13028 aldoxime dehydratase [EC:4.99.1.5] | ec:4.99.1.5 |
| ppf:Pput\_2725 | meta-pathway phenol degradation-like protein |  |
| ppf:Pput\_2726 | globin; K06886 hemoglobin |  |
| ppf:Pput\_2727 | amidase; K01426 amidase [EC:3.5.1.4] | ec:3.5.1.4 |
| ppf:Pput\_2728 | nitrile hydratase subunit alpha; K01721 nitrile hydratase [EC:4.2.1.84] | ec:4.2.1.84 |
| ppf:Pput\_2729 | nitrile hydratase; K01721 nitrile hydratase [EC:4.2.1.84] | ec:4.2.1.84 |
| ppf:Pput\_2730 | cobalamin synthesis protein, P47K |  |
| ppf:Pput\_2731 | methyl-accepting chemotaxis sensory transducer |  |

  
**Neighborhood Representations for "plu:plu2425"**  

| ID | Annotation | EC number |
| --- | --- | --- |
| plu:plu2415 | pseudogene |  |
| plu:plu2416 | hypothetical protein |  |
| plu:plu2417 | pseudogene |  |
| plu:plu2418 | pseudogene |  |
| plu:plu2419 | ISPlu3S; IS630 family transposase |  |
| plu:plu2420 | hypothetical protein |  |
| plu:plu2421 | hypothetical protein |  |
| plu:plu2422 | hypothetical protein |  |
| plu:plu2423 | hypothetical protein; K07154 serine/threonine-protein kinase HipA [EC:2.7.11.1] | ec:2.7.11.1 |
| plu:plu2424 | hypothetical protein; K09958 hypothetical protein |  |
| plu:plu2425 | hypothetical protein |  |
| plu:plu2426 | translation initiation factor Sui1; K03113 translation initiation factor 1 |  |
| plu:plu2427 | pyrF; orotidine 5'-phosphate decarboxylase (EC:4.1.1.23); K01591 orotidine-5'-phosphate decarboxylase [EC:4.1.1.23] | ec:4.1.1.23 |
| plu:plu2428 | tetratricopeptide repeat protein |  |
| plu:plu2429 | hypothetical protein; K08992 putative membrane protein |  |
| plu:plu2430 | pseudogene |  |
| plu:plu2431 | ribA; GTP cyclohydrolase II (EC:3.5.4.25); K01497 GTP cyclohydrolase II [EC:3.5.4.25] | ec:3.5.4.25 |
| plu:plu2432 | acnA; aconitate hydratase (EC:4.2.1.3); K01681 aconitate hydratase [EC:4.2.1.3] | ec:4.2.1.3 |
| plu:plu2433 | hypothetical protein |  |
| plu:plu2434 | cysB; transcriptional regulator CysB; K13634 LysR family transcriptional regulator, cys regulon transcriptional activator |  |
| plu:plu2435 | topA; DNA topoisomerase I (EC:5.99.1.2); K03168 DNA topoisomerase I [EC:5.99.1.2] | ec:5.99.1.2 |

  
**Neighborhood Representations for "ppw:PputW619\_3097"**  

| ID | Annotation | EC number |
| --- | --- | --- |
| ppw:PputW619\_3087 | antibiotic biosynthesis monooxygenase |  |
| ppw:PputW619\_3088 | cupin |  |
| ppw:PputW619\_3089 | carboxymuconolactone decarboxylase; K01607 4-carboxymuconolactone decarboxylase [EC:4.1.1.44] | ec:4.1.1.44 |
| ppw:PputW619\_3090 | LysR family transcriptional regulator (EC:4.2.1.1) |  |
| ppw:PputW619\_3091 | hypothetical protein |  |
| ppw:PputW619\_3092 | cyanate hydratase (EC:4.2.1.104); K01725 cyanate lyase [EC:4.2.1.104] | ec:4.2.1.104 |
| ppw:PputW619\_3093 | carbonate dehydratase (EC:4.2.1.1); K01673 carbonic anhydrase [EC:4.2.1.1] | ec:4.2.1.1 |
| ppw:PputW619\_3094 | DNA-binding transcriptional regulator CynR; K11921 LysR family transcriptional regulator, cyn operon transcriptional activator |  |
| ppw:PputW619\_3095 | hypothetical protein; K09958 hypothetical protein |  |
| ppw:PputW619\_3096 | hypothetical protein; K09958 hypothetical protein |  |
| ppw:PputW619\_3097 | TetR family transcriptional regulator |  |
| ppw:PputW619\_3098 | alpha/beta hydrolase domain-containing protein |  |
| ppw:PputW619\_3099 | transposase, IS4 |  |
| ppw:PputW619\_3100 | hypothetical protein |  |
| ppw:PputW619\_3101 | hypothetical protein |  |
| ppw:PputW619\_3102 | hypothetical protein |  |
| ppw:PputW619\_3103 | hypothetical protein |  |
| ppw:PputW619\_3104 | alkylhydroperoxidase |  |
| ppw:PputW619\_3105 | NAD(P)H dehydrogenase; K01118 FMN-dependent NADH-azoreductase [EC:1.7.-.-] |  |
| ppw:PputW619\_3106 | N-acetyltransferase GCN5 |  |
| ppw:PputW619\_3107 | transcriptional regulator; K00375 GntR family transcriptional regulator / MocR family aminotransferase |  |

  
**Neighborhood Representations for "pfs:PFLU2916"**  

| ID | Annotation | EC number |
| --- | --- | --- |
| pfs:PFLU2906 | putative ABC transporter membrane protein |  |
| pfs:PFLU2907 | hypothetical protein |  |
| pfs:PFLU2908 | hypothetical protein |  |
| pfs:PFLU2909 | putative chain length determinant protein |  |
| pfs:PFLU2910 | putative beta-hydroxylase; K12979 beta-hydroxylase [EC:1.14.11.-] |  |
| pfs:PFLU2911 | ligD; ATP-dependent DNA ligase; K01971 DNA ligase (ATP) [EC:6.5.1.1] | ec:6.5.1.1 |
| pfs:PFLU2912 | hypothetical protein; K10979 DNA end-binding protein Ku |  |
| pfs:PFLU2913 | hypothetical protein |  |
| pfs:PFLU2914 | hypothetical protein |  |
| pfs:PFLU2915 | hypothetical protein; K09958 hypothetical protein |  |
| pfs:PFLU2916 | TetR family transcriptional regulator |  |
| pfs:PFLU2917 | pssA; phosphatidylserine synthase (EC:2.7.8.8); K00998 CDP-diacylglycerol---serine O-phosphatidyltransferase [EC:2.7.8.8] | ec:2.7.8.8 |
| pfs:PFLU2918 | putative lipoprotein; K07224 iron uptake system component EfeO |  |
| pfs:PFLU2919 | hypothetical protein; K16301 deferrochelatase/peroxidase EfeB [EC:1.11.1.-] |  |
| pfs:PFLU2920 | hypothetical protein |  |
| pfs:PFLU2921 | hypothetical protein; K07243 high-affinity iron transporter |  |
| pfs:PFLU2922 | hypothetical protein |  |
| pfs:PFLU2923 | AraC family transcriptional regulator |  |
| pfs:PFLU2924 | hypothetical protein |  |
| pfs:PFLU2925 | putative ThiF protein |  |
| pfs:PFLU2926 | putative transporter-like protein |  |

  
**Neighborhood Representations for "ppu:PP\_2951"**  

| ID | Annotation | EC number |
| --- | --- | --- |
| ppu:PP\_2941 | hypothetical protein |  |
| ppu:PP\_2942 | response regulator |  |
| ppu:PP\_2943 | cytochrome c551 peroxidase; K00428 cytochrome c peroxidase [EC:1.11.1.5] | ec:1.11.1.5 |
| ppu:PP\_2944 | sensor histidine kinase |  |
| ppu:PP\_2945 | sensor histidine kinase/response regulator; K02482 two-component system, NtrC family, sensor kinase [EC:2.7.13.3] | ec:2.7.13.3 |
| ppu:PP\_2946 | peptidyl-tRNA hydrolase domain-containing protein; K15034 ribosome-associated protein |  |
| ppu:PP\_2947 | transcriptional regulator MvaT, P16 subunit |  |
| ppu:PP\_2948 | GntR family transcriptional regulator |  |
| ppu:PP\_2949 | hypothetical protein |  |
| ppu:PP\_2950 | hypothetical protein; K09958 hypothetical protein |  |
| ppu:PP\_2951 | TetR family transcriptional regulator |  |
| ppu:PP\_2952 | LysR family transcriptional regulator |  |
| ppu:PP\_2953 | zinc-containing alcohol dehydrogenase |  |
| ppu:PP\_2954 | hypothetical protein |  |
| ppu:PP\_2955 | magnesium/cobalt transporter CorA family protein; K03284 magnesium transporter |  |
| ppu:PP\_2956 | ribosomal protein S6 modification protein-like protein |  |
| ppu:PP\_2957 | acetyltransferase |  |
| ppu:PP\_2958 | glutaredoxin; K03676 glutaredoxin 3 |  |
| ppu:PP\_2959 | alkylhydroperoxidase |  |
| ppu:PP\_2960 | AraC family transcriptional regulator |  |
| ppu:PP\_2961 | LysR family transcriptional regulator |  |

  
**Neighborhood Representations for "xne:XNC1\_2294"**  

| ID | Annotation | EC number |
| --- | --- | --- |
| xne:XNC1\_2284 | hypothetical protein |  |
| xne:XNC1\_2285 | hypothetical protein |  |
| xne:XNC1\_2286 | transposase (fragment) |  |
| xne:XNC1\_2287 | hypothetical protein; K03885 NADH dehydrogenase [EC:1.6.99.3] | ec:1.6.99.3 |
| xne:XNC1\_2288 | hypothetical protein |  |
| xne:XNC1\_2289 | hypothetical protein |  |
| xne:XNC1\_2290 | amidotransferase (EC:6.3.5.2); K01951 GMP synthase (glutamine-hydrolysing) [EC:6.3.5.2] | ec:6.3.5.2 |
| xne:XNC1\_2291 | transcriptional regulatory protein ptsJ |  |
| xne:XNC1\_2292 | Ribitol kinase (EC:2.7.1.16) |  |
| xne:XNC1\_2293 | transposase (fragment) |  |
| xne:XNC1\_2294 | TetR family transcriptional regulator |  |
| xne:XNC1\_2295 | hypothetical protein; K09958 hypothetical protein |  |
| xne:XNC1\_2296 | pseudogene |  |
| xne:XNC1\_2297 | pseudogene |  |
| xne:XNC1\_2298 | hypothetical protein |  |
| xne:XNC1\_2299 | peptide synthetase |  |
| xne:XNC1\_2300 | peptide synthetase |  |
| xne:XNC1\_2301 | hypothetical protein |  |
| xne:XNC1\_2302 | hypothetical protein |  |
| xne:XNC1\_2303 | hypothetical protein |  |
| xne:XNC1\_2304 | hypothetical protein |  |

  
**Neighborhood Representations for "acp:A2cp1\_0250"**  

| ID | Annotation | EC number |
| --- | --- | --- |
| acp:A2cp1\_0240 | serine/threonine protein kinase |  |
| acp:A2cp1\_0241 | pyruvate flavodoxin/ferredoxin oxidoreductase domain-containing protein; K03737 putative pyruvate-flavodoxin oxidoreductase [EC:1.2.7.-] |  |
| acp:A2cp1\_0242 | hypothetical protein |  |
| acp:A2cp1\_0243 | NAD-dependent epimerase/dehydratase; K08679 UDP-glucuronate 4-epimerase [EC:5.1.3.6] | ec:5.1.3.6 |
| acp:A2cp1\_0244 | WecB/TagA/CpsF family glycosyl transferase |  |
| acp:A2cp1\_0245 | hypothetical protein |  |
| acp:A2cp1\_0246 | endonuclease/exonuclease/phosphatase |  |
| acp:A2cp1\_0247 | RND family efflux transporter MFP subunit |  |
| acp:A2cp1\_0248 | acriflavin resistance protein; K03296 hydrophobic/amphiphilic exporter-1 (mainly G- bacteria), HAE1 family |  |
| acp:A2cp1\_0249 | hypothetical protein |  |
| acp:A2cp1\_0250 | TetR family transcriptional regulator |  |
| acp:A2cp1\_0251 | FAD dependent oxidoreductase |  |
| acp:A2cp1\_0252 | RNA polymerase sigma factor; K03088 RNA polymerase sigma-70 factor, ECF subfamily |  |
| acp:A2cp1\_0253 | Fe-S oxidoreductase |  |
| acp:A2cp1\_0254 | teichoic acid biosynthesis-like protein |  |
| acp:A2cp1\_0255 | hypothetical protein; K00783 23S rRNA (pseudouridine1915-N3)-methyltransferase [EC:2.1.1.177] | ec:2.1.1.177 |
| acp:A2cp1\_0256 | peptidase S8/S53 subtilisin kexin sedolisin; K14645 serine protease [EC:3.4.21.-] |  |
| acp:A2cp1\_0257 | phosphoglyceromutase (EC:5.4.2.1); K15633 2,3-bisphosphoglycerate-independent phosphoglycerate mutase [EC:5.4.2.12] | ec:5.4.2.12 |
| acp:A2cp1\_0258 | phosphoribosyltransferase |  |
| acp:A2cp1\_0259 | hypothetical protein |  |
| acp:A2cp1\_0260 | hypothetical protein |  |

  
**Neighborhood Representations for "ank:AnaeK\_0239"**  

| ID | Annotation | EC number |
| --- | --- | --- |
| ank:AnaeK\_0229 | serine/threonine protein kinase |  |
| ank:AnaeK\_0230 | pyruvate flavodoxin/ferredoxin oxidoreductase domain-containing protein; K03737 putative pyruvate-flavodoxin oxidoreductase [EC:1.2.7.-] |  |
| ank:AnaeK\_0231 | hypothetical protein |  |
| ank:AnaeK\_0232 | NAD-dependent epimerase/dehydratase; K08679 UDP-glucuronate 4-epimerase [EC:5.1.3.6] | ec:5.1.3.6 |
| ank:AnaeK\_0233 | WecB/TagA/CpsF family glycosyl transferase |  |
| ank:AnaeK\_0234 | hypothetical protein |  |
| ank:AnaeK\_0235 | endonuclease/exonuclease/phosphatase |  |
| ank:AnaeK\_0236 | RND family efflux transporter MFP subunit |  |
| ank:AnaeK\_0237 | acriflavin resistance protein; K03296 hydrophobic/amphiphilic exporter-1 (mainly G- bacteria), HAE1 family |  |
| ank:AnaeK\_0238 | hypothetical protein |  |
| ank:AnaeK\_0239 | TetR family transcriptional regulator |  |
| ank:AnaeK\_0240 | FAD dependent oxidoreductase |  |
| ank:AnaeK\_0241 | RNA polymerase sigma factor; K03088 RNA polymerase sigma-70 factor, ECF subfamily |  |
| ank:AnaeK\_0242 | Fe-S oxidoreductase |  |
| ank:AnaeK\_0243 | teichoic acid biosynthesis-like protein |  |
| ank:AnaeK\_0244 | hypothetical protein; K00783 23S rRNA (pseudouridine1915-N3)-methyltransferase [EC:2.1.1.177] | ec:2.1.1.177 |
| ank:AnaeK\_0245 | peptidase S8/S53 subtilisin kexin sedolisin; K14645 serine protease [EC:3.4.21.-] |  |
| ank:AnaeK\_0246 | phosphoglyceromutase (EC:5.4.2.1); K15633 2,3-bisphosphoglycerate-independent phosphoglycerate mutase [EC:5.4.2.12] | ec:5.4.2.12 |
| ank:AnaeK\_0247 | phosphoribosyltransferase |  |
| ank:AnaeK\_0248 | hypothetical protein |  |
| ank:AnaeK\_0249 | hypothetical protein |  |

  
**Neighborhood Representations for "ade:Adeh\_0228"**  

| ID | Annotation | EC number |
| --- | --- | --- |
| ade:Adeh\_0218 | pyruvate-ferredoxin (flavodoxin) oxidoreductase; K03737 putative pyruvate-flavodoxin oxidoreductase [EC:1.2.7.-] |  |
| ade:Adeh\_0219 | hemerythrin-like, metal-binding protein; K07216 hemerythrin |  |
| ade:Adeh\_0220 | hypothetical protein |  |
| ade:Adeh\_0221 | NAD-dependent epimerase/dehydratase; K08679 UDP-glucuronate 4-epimerase [EC:5.1.3.6] | ec:5.1.3.6 |
| ade:Adeh\_0222 | WecB/TagA/CpsF family glycosyl transferase |  |
| ade:Adeh\_0223 | hypothetical protein |  |
| ade:Adeh\_0224 | endonuclease/exonuclease/phosphatase |  |
| ade:Adeh\_0225 | secretion protein HlyD |  |
| ade:Adeh\_0226 | acriflavin resistance protein; K03296 hydrophobic/amphiphilic exporter-1 (mainly G- bacteria), HAE1 family |  |
| ade:Adeh\_0227 | hypothetical protein |  |
| ade:Adeh\_0228 | TetR family transcriptional regulator |  |
| ade:Adeh\_0229 | FAD dependent oxidoreductase |  |
| ade:Adeh\_0230 | RNA polymerase sigma factor; K03088 RNA polymerase sigma-70 factor, ECF subfamily |  |
| ade:Adeh\_0231 | Fe-S oxidoreductase |  |
| ade:Adeh\_0232 | teichoic acid biosynthesis-like protein |  |
| ade:Adeh\_0233 | hypothetical protein; K00783 23S rRNA (pseudouridine1915-N3)-methyltransferase [EC:2.1.1.177] | ec:2.1.1.177 |
| ade:Adeh\_0234 | peptidase S8/S53 subtilisin kexin sedolisin; K14645 serine protease [EC:3.4.21.-] |  |
| ade:Adeh\_0235 | phosphoglyceromutase (EC:5.4.2.1); K15633 2,3-bisphosphoglycerate-independent phosphoglycerate mutase [EC:5.4.2.12] | ec:5.4.2.12 |
| ade:Adeh\_0236 | phosphoribosyltransferase |  |
| ade:Adeh\_0237 | hypothetical protein |  |
| ade:Adeh\_0238 | hypothetical protein |  |

  
**Neighborhood Representations for "mfa:Mfla\_0355"**  

| ID | Annotation | EC number |
| --- | --- | --- |
| mfa:Mfla\_0345 | amino acid permease-associated region; K03294 basic amino acid/polyamine antiporter, APA family |  |
| mfa:Mfla\_0346 | coproporphyrinogen III oxidase (EC:1.3.3.3); K00228 coproporphyrinogen III oxidase [EC:1.3.3.3] | ec:1.3.3.3 |
| mfa:Mfla\_0347 | SUA5/yciO/yrdC-like protein; K07566 L-threonylcarbamoyladenylate synthase [EC:2.7.7.87] | ec:2.7.7.87 |
| mfa:Mfla\_0348 | phosphoribosylamine--glycine ligase (EC:6.3.4.13); K01945 phosphoribosylamine--glycine ligase [EC:6.3.4.13] | ec:6.3.4.13 |
| mfa:Mfla\_0349 | purH; bifunctional phosphoribosylaminoimidazolecarboxamide formyltransferase/IMP cyclohydrolase (EC:2.1.2.3 3.5.4.10); K00602 phosphoribosylaminoimidazolecarboxamide formyltransferase / IMP cyclohydrolase [EC:2.1.2.3 3.5.4.10] | ec:3.5.4.10 ec:2.1.2.3 |
| mfa:Mfla\_0350 | helix-turn-helix, Fis-type; K03557 Fis family transcriptional regulator, factor for inversion stimulation protein |  |
| mfa:Mfla\_0351 | dihydrouridine synthase TIM-barrel protein nifR3; K05540 tRNA-dihydrouridine synthase B [EC:1.-.-.-] |  |
| mfa:Mfla\_0352 | methyl-accepting chemotaxis sensory transducer; K03406 methyl-accepting chemotaxis protein |  |
| mfa:Mfla\_0353 | hypothetical protein |  |
| mfa:Mfla\_0354 | ThiJ/PfpI |  |
| mfa:Mfla\_0355 | TetR family transcriptional regulator |  |
| mfa:Mfla\_0356 | hypothetical protein; K06940 |  |
| mfa:Mfla\_0357 | molybdopterin binding domain-containing protein |  |
| mfa:Mfla\_0358 | DNA polymerase I (EC:2.7.7.7); K02335 DNA polymerase I [EC:2.7.7.7] | ec:2.7.7.7 |
| mfa:Mfla\_0359 | hypothetical protein; K06966 |  |
| mfa:Mfla\_0360 | hypothetical protein |  |
| mfa:Mfla\_0361 | homoserine kinase (EC:2.7.1.39); K02204 homoserine kinase type II [EC:2.7.1.39] | ec:2.7.1.39 |
| mfa:Mfla\_0362 | hypothetical protein |  |
| mfa:Mfla\_0363 | ATP-dependent DNA helicase UvrD (EC:3.6.1.-); K03657 DNA helicase II / ATP-dependent DNA helicase PcrA [EC:3.6.4.12] | ec:3.6.4.12 |
| mfa:Mfla\_0364 | chaperonin GroEL; K04077 chaperonin GroEL |  |
| mfa:Mfla\_0365 | groES; co-chaperonin GroES; K04078 chaperonin GroES |  |

  
**Neighborhood Representations for "ava:Ava\_4037"**  

| ID | Annotation | EC number |
| --- | --- | --- |
| ava:Ava\_4027 | oxidoreductase/nitrogenase, component 1 (EC:1.18.6.1); K02591 nitrogenase molybdenum-iron protein beta chain [EC:1.18.6.1] | ec:1.18.6.1 |
| ava:Ava\_4028 | oxidoreductase/nitrogenase, component 1 (EC:1.18.6.1); K02586 nitrogenase molybdenum-iron protein alpha chain [EC:1.18.6.1] | ec:1.18.6.1 |
| ava:Ava\_4029 | oxidoreductase/nitrogenase, component 1 (EC:1.18.6.1); K02591 nitrogenase molybdenum-iron protein beta chain [EC:1.18.6.1] | ec:1.18.6.1 |
| ava:Ava\_4030 | hypothetical protein; K07066 |  |
| ava:Ava\_4031 | hypothetical protein |  |
| ava:Ava\_4032 | hypothetical protein |  |
| ava:Ava\_4033 | hypothetical protein |  |
| ava:Ava\_4034 | PilT protein-like protein |  |
| ava:Ava\_4035 | nitroreductase (EC:1.6.99.3); K00356 NADH dehydrogenase [EC:1.6.99.3] | ec:1.6.99.3 |
| ava:Ava\_4036 | prolyl 4-hydroxylase subunit alpha |  |
| ava:Ava\_4037 | TetR family transcriptional regulator |  |
| ava:Ava\_4038 | ABC transporter-like protein (EC:3.6.3.25); K06857 tungstate transport system ATP-binding protein [EC:3.6.3.55] | ec:3.6.3.55 |
| ava:Ava\_4039 | binding-protein dependent transport system inner membrane protein; K05773 tungstate transport system permease protein |  |
| ava:Ava\_4040 | tungstate ABC transporter permease; K05772 tungstate transport system substrate-binding protein |  |
| ava:Ava\_4041 | aksA; trans-homoaconitate synthase (EC:2.3.3.14); K02594 homocitrate synthase NifV [EC:2.3.3.14] | ec:2.3.3.14 |
| ava:Ava\_4042 | molybdate metabolism transcriptional regulator |  |
| ava:Ava\_4043 | PilT protein-like protein |  |
| ava:Ava\_4044 | prevent-host-death protein |  |
| ava:Ava\_4045 | hypothetical protein |  |
| ava:Ava\_4046 | nitrogenase iron protein (EC:1.18.6.1); K02588 nitrogenase iron protein NifH [EC:1.18.6.1] | ec:1.18.6.1 |
| ava:Ava\_4047 | cytochrome bd ubiquinol oxidase subunit II; K00426 cytochrome d ubiquinol oxidase subunit II [EC:1.10.3.-] |  |

  
**Neighborhood Representations for "sen:SACE\_3451"**  

| ID | Annotation | EC number |
| --- | --- | --- |
| sen:SACE\_3441 | epoxide hydrolase (EC:3.3.2.9); K01253 microsomal epoxide hydrolase [EC:3.3.2.9] | ec:3.3.2.9 |
| sen:SACE\_3442 | DeoR family transcriptional regulator |  |
| sen:SACE\_3443 | TetR/AcrR family transcriptional regulator |  |
| sen:SACE\_3444 | AMP-dependent synthetase/ligase (EC:6.2.1.3); K01897 long-chain acyl-CoA synthetase [EC:6.2.1.3] | ec:6.2.1.3 |
| sen:SACE\_3445 | short-chain dehydrogenase/reductase SDR (EC:1.1.1.35) |  |
| sen:SACE\_3446 | TetR family transcriptional regulator |  |
| sen:SACE\_3447 | long-chain-fatty-acid--CoA ligase (EC:2.3.1.86); K00666 fatty-acyl-CoA synthase [EC:6.2.1.-] |  |
| sen:SACE\_3448 | phosphoglycerate mutase (EC:5.4.2.1); K15634 probable phosphoglycerate mutase [EC:5.4.2.12] | ec:5.4.2.12 |
| sen:SACE\_3449 | gluconolactonase (EC:3.1.1.17); K01053 gluconolactonase [EC:3.1.1.17] | ec:3.1.1.17 |
| sen:SACE\_3450 | enoyl-CoA hydratase/isomerase (EC:4.2.1.17) |  |
| sen:SACE\_3451 | TetR family transcriptional regulator |  |
| sen:SACE\_3452 | hypothetical protein |  |
| sen:SACE\_3453 | alpha/beta hydrolase |  |
| sen:SACE\_3454 | histidine kinase/response regulator hybrid protein |  |
| sen:SACE\_3455 | pyridine nucleotide-disulphide oxidoreductase (EC:1.16.1.1); K00520 mercuric reductase [EC:1.16.1.1] | ec:1.16.1.1 |
| sen:SACE\_3456 | hypothetical protein |  |
| sen:SACE\_3457 | rhtB; homoserine/threonine efflux protein |  |
| sen:SACE\_3458 | hypothetical protein |  |
| sen:SACE\_3459 | ygbK; hypothetical protein |  |
| sen:SACE\_3460 | pdxA; 4-hydroxythreonine-4-phosphate dehydrogenase (EC:1.1.1.262); K00097 4-hydroxythreonine-4-phosphate dehydrogenase [EC:1.1.1.262] | ec:1.1.1.262 |
| sen:SACE\_3461 | sugar transporter |  |

  
**Neighborhood Representations for "sro:Sros\_3836"**  

| ID | Annotation | EC number |
| --- | --- | --- |
| sro:Sros\_3826 | pseudogene |  |
| sro:Sros\_3827 | phytoene desaturase; K10027 phytoene desaturase [EC:1.3.99.26 1.3.99.28 1.3.99.29 1.3.99.31] | ec:1.3.99.31 ec:1.3.99.26 ec:1.3.99.28 ec:1.3.99.29 |
| sro:Sros\_3828 | phytoene synthase (EC:2.5.1.32); K02291 phytoene synthase [EC:2.5.1.32] | ec:2.5.1.32 |
| sro:Sros\_3829 | phosphate:acyl-(acyl carrier protein) acyltransferase |  |
| sro:Sros\_3830 | spheroidene monooxygenase |  |
| sro:Sros\_3831 | acyl-phosphate glycerol-3-phosphate acyltransferase; K08591 glycerol-3-phosphate acyltransferase PlsY [EC:2.3.1.15] | ec:2.3.1.15 |
| sro:Sros\_3832 | glycosyl transferase group 2 family protein; K00721 dolichol-phosphate mannosyltransferase [EC:2.4.1.83] | ec:2.4.1.83 |
| sro:Sros\_3833 | hydroxyneurosporene dehydrogenase |  |
| sro:Sros\_3834 | membrane protein; K08977 putative membrane protein |  |
| sro:Sros\_3835 | 3-dehydroquinate synthase; K00891 shikimate kinase [EC:2.7.1.71] | ec:2.7.1.71 |
| sro:Sros\_3836 | TetR family transcriptional regulator |  |
| sro:Sros\_3837 | alkylhydroperoxidase |  |
| sro:Sros\_3838 | hypothetical protein |  |
| sro:Sros\_3839 | hypothetical protein; K09955 hypothetical protein |  |
| sro:Sros\_3840 | permease of sugar ABC transporter; K02026 multiple sugar transport system permease protein |  |
| sro:Sros\_3841 | sugar ABC transporter; K02025 multiple sugar transport system permease protein |  |
| sro:Sros\_3842 | extracellular solute-binding protein; K02027 multiple sugar transport system substrate-binding protein |  |
| sro:Sros\_3843 | transcriptional regulator; K02529 LacI family transcriptional regulator |  |
| sro:Sros\_3844 | TenA family transcriptional activator; K03707 thiaminase (transcriptional activator TenA) [EC:3.5.99.2] | ec:3.5.99.2 |
| sro:Sros\_3845 | pseudogene |  |
| sro:Sros\_3846 | hypothetical protein |  |

  
**Neighborhood Representations for "svi:Svir\_28340"**  

| ID | Annotation | EC number |
| --- | --- | --- |
| svi:Svir\_28240 | enoyl-CoA hydratase/carnithine racemase |  |
| svi:Svir\_28250 | electron transfer flavoprotein, alpha subunit; K03522 electron transfer flavoprotein alpha subunit |  |
| svi:Svir\_28260 | electron transfer flavoprotein, beta subunit; K03521 electron transfer flavoprotein beta subunit |  |
| svi:Svir\_28270 | degV family protein |  |
| svi:Svir\_28280 | methyltransferase family protein |  |
| svi:Svir\_28290 | hypothetical protein; K16149 1,4-alpha-glucan branching enzyme [EC:2.4.1.18] | ec:2.4.1.18 |
| svi:Svir\_28300 | beta-galactosidase/beta-glucuronidase |  |
| svi:Svir\_28310 | glycosyltransferase; K16150 glycogen(starch) synthase [EC:2.4.1.11] | ec:2.4.1.11 |
| svi:Svir\_28320 | hypothetical protein |  |
| svi:Svir\_28330 | hypothetical protein; K09958 hypothetical protein |  |
| svi:Svir\_28340 | transcriptional regulator, TetR family |  |
| svi:Svir\_28350 | malate synthase A; K01638 malate synthase [EC:2.3.3.9] | ec:2.3.3.9 |
| svi:Svir\_28360 | isocitrate lyase; K01637 isocitrate lyase [EC:4.1.3.1] | ec:4.1.3.1 |
| svi:Svir\_28370 | putative transcriptional regulator; K07110 |  |
| svi:Svir\_28380 | Zinc carboxypeptidase |  |
| svi:Svir\_28390 | acetyltransferase (isoleucine patch superfamily) |  |
| svi:Svir\_28400 | Inosine-uridine nucleoside N-ribohydrolase; K01250 pyrimidine-specific ribonucleoside hydrolase [EC:3.2.-.-] |  |
| svi:Svir\_28410 | cell wall-associated hydrolase, invasion-associated protein |  |
| svi:Svir\_28420 | Excinuclease ATPase subunit |  |
| svi:Svir\_28430 | hypothetical protein |  |
| svi:Svir\_28440 | hypothetical protein |  |

  
**Neighborhood Representations for "fri:FraEuI1c\_3599"**  

| ID | Annotation | EC number |
| --- | --- | --- |
| fri:FraEuI1c\_3589 | Clp domain-containing protein |  |
| fri:FraEuI1c\_3590 | inner-membrane translocator |  |
| fri:FraEuI1c\_3591 | ABC transporter; K01996 branched-chain amino acid transport system ATP-binding protein |  |
| fri:FraEuI1c\_3592 | hypothetical protein; K01999 branched-chain amino acid transport system substrate-binding protein |  |
| fri:FraEuI1c\_3593 | NUDIX hydrolase |  |
| fri:FraEuI1c\_3594 | hypothetical protein |  |
| fri:FraEuI1c\_3595 | GPI-anchored protein |  |
| fri:FraEuI1c\_3596 | glycoside hydrolase family protein |  |
| fri:FraEuI1c\_3597 | hypothetical protein |  |
| fri:FraEuI1c\_3598 | wyosine base formation |  |
| fri:FraEuI1c\_3599 | TetR family transcriptional regulator |  |
| fri:FraEuI1c\_3600 | alkylhydroperoxidase |  |
| fri:FraEuI1c\_3601 | hypothetical protein |  |
| fri:FraEuI1c\_3602 | SARP family transcriptional regulator |  |
| fri:FraEuI1c\_3603 | hemerythrin HHE cation binding domain protein |  |
| fri:FraEuI1c\_3604 | hemerythrin HHE cation binding domain-containing protein |  |
| fri:FraEuI1c\_3605 | hypothetical protein |  |
| fri:FraEuI1c\_3606 | major facilitator superfamily protein |  |
| fri:FraEuI1c\_3607 | cupin |  |
| fri:FraEuI1c\_3608 | TetR family transcriptional regulator |  |
| fri:FraEuI1c\_3609 | hypothetical protein |  |

  
**Neighborhood Representations for "sco:SCO0772"**  

| ID | Annotation | EC number |
| --- | --- | --- |
| sco:SCO0762 | SCF81.21c, sti1; protease inhibitor protein |  |
| sco:SCO0763 | SCF81.22; oxidoreductase |  |
| sco:SCO0764 | SCF81.23c; hydrolase |  |
| sco:SCO0765 | cel1, SCF81.24c; endoglucanase |  |
| sco:SCO0766 | SCF81.25c; beta-galactosidase; K01190 beta-galactosidase [EC:3.2.1.23] | ec:3.2.1.23 |
| sco:SCO0767 | SCF81.26; hypothetical protein |  |
| sco:SCO0768 | SCF81.27; lipoprotein |  |
| sco:SCO0769 | 3SCF60.01c, SCF81.28c; aldo/keto reductase |  |
| sco:SCO0770 | 3SCF60.02c; hypothetical protein |  |
| sco:SCO0771 | 3SCF60.03c; hypothetical protein; K09958 hypothetical protein |  |
| sco:SCO0772 | 3SCF60.04; regulatory protein |  |
| sco:SCO0773 | 3SCF60.05c, soyB2; ferredoxin |  |
| sco:SCO0774 | 3SCF60.06c; cytochrome P450; K17876 pentalenic acid synthase [EC:1.14.15.11] | ec:1.14.15.11 |
| sco:SCO0775 | 3SCF60.07; hypothetical protein |  |
| sco:SCO0776 | 3SCF60.08c; hypothetical protein |  |
| sco:SCO0777 | 3SCF60.09; hypothetical protein; K07393 putative glutathione S-transferase |  |
| sco:SCO0778 | 3SCF60.10; hypothetical protein |  |
| sco:SCO0779 | 3SCF60.11c; hypothetical protein |  |
| sco:SCO0780 | 3SCF60.12; zinc-binding oxidoreductase |  |
| sco:SCO0781 | 3SCF60.13; anti sigma factor antagonist |  |
| sco:SCO0782 | 3SCF60.14c, prsA; ribose-phosphate pyrophosphokinase; K00948 ribose-phosphate pyrophosphokinase [EC:2.7.6.1] | ec:2.7.6.1 |

  
**Neighborhood Representations for "cai:Caci\_5341"**  

| ID | Annotation | EC number |
| --- | --- | --- |
| cai:Caci\_5331 | binding-protein-dependent transporters inner membrane component; K10241 cellobiose transport system permease protein |  |
| cai:Caci\_5332 | binding-protein-dependent transporters inner membrane component; K10242 cellobiose transport system permease protein |  |
| cai:Caci\_5333 | beta-galactosidase (EC:3.2.1.21); K05350 beta-glucosidase [EC:3.2.1.21] | ec:3.2.1.21 |
| cai:Caci\_5334 | LacI family transcriptional regulator (EC:5.1.1.1) |  |
| cai:Caci\_5335 | hypothetical protein |  |
| cai:Caci\_5336 | ROK family protein |  |
| cai:Caci\_5337 | oxidoreductase domain-containing protein |  |
| cai:Caci\_5338 | xylose isomerase |  |
| cai:Caci\_5339 | xylose isomerase |  |
| cai:Caci\_5340 | oxidoreductase domain-containing protein |  |
| cai:Caci\_5341 | TetR family transcriptional regulator |  |
| cai:Caci\_5342 | S-adenosylmethionine synthetase (EC:2.5.1.6); K00789 S-adenosylmethionine synthetase [EC:2.5.1.6] | ec:2.5.1.6 |
| cai:Caci\_5343 | bifunctional phosphopantothenoylcysteine decarboxylase/phosphopantothenate synthase; K13038 phosphopantothenoylcysteine decarboxylase / phosphopantothenate--cysteine ligase [EC:4.1.1.36 6.3.2.5] | ec:6.3.2.5 ec:4.1.1.36 |
| cai:Caci\_5344 | DNA-directed RNA polymerase subunit omega; K03060 DNA-directed RNA polymerase subunit omega [EC:2.7.7.6] | ec:2.7.7.6 |
| cai:Caci\_5345 | guanylate kinase (EC:2.7.4.8); K00942 guanylate kinase [EC:2.7.4.8] | ec:2.7.4.8 |
| cai:Caci\_5346 | hypothetical protein |  |
| cai:Caci\_5347 | hypothetical protein; K07054 |  |
| cai:Caci\_5348 | von Willebrand factor type A |  |
| cai:Caci\_5349 | hypothetical protein |  |
| cai:Caci\_5350 | hypothetical protein |  |
| cai:Caci\_5351 | hypothetical protein |  |

  
**Neighborhood Representations for "xau:Xaut\_2611"**  

| ID | Annotation | EC number |
| --- | --- | --- |
| xau:Xaut\_2601 | dehydratase |  |
| xau:Xaut\_2602 | hypothetical protein |  |
| xau:Xaut\_2603 | hypothetical protein |  |
| xau:Xaut\_2604 | dehydratase |  |
| xau:Xaut\_2605 | GTP-dependent nucleic acid-binding protein EngD; K06942 |  |
| xau:Xaut\_2606 | peptidyl-tRNA hydrolase; K01056 peptidyl-tRNA hydrolase, PTH1 family [EC:3.1.1.29] | ec:3.1.1.29 |
| xau:Xaut\_2607 | 50S ribosomal protein L25/general stress protein Ctc; K02897 large subunit ribosomal protein L25 |  |
| xau:Xaut\_2608 | cytochrome c class I |  |
| xau:Xaut\_2609 | hypothetical protein |  |
| xau:Xaut\_2610 | alcohol dehydrogenase; K00001 alcohol dehydrogenase [EC:1.1.1.1] | ec:1.1.1.1 |
| xau:Xaut\_2611 | TetR family transcriptional regulator |  |
| xau:Xaut\_2612 | OsmC family protein |  |
| xau:Xaut\_2613 | putative acyl-CoA dehydrogenase family protein; K00257 [EC:1.3.99.-] |  |
| xau:Xaut\_2614 | rhodanese domain-containing protein; K01011 thiosulfate/3-mercaptopyruvate sulfurtransferase [EC:2.8.1.1 2.8.1.2] | ec:2.8.1.1 ec:2.8.1.2 |
| xau:Xaut\_2615 | DSBA oxidoreductase |  |
| xau:Xaut\_2616 | FAD-dependent pyridine nucleotide-disulfide oxidoreductase; K00529 ferredoxin--NAD+ reductase [EC:1.18.1.3] | ec:1.18.1.3 |
| xau:Xaut\_2617 | ABC transporter nitrate-binding protein; K15576 nitrate/nitrite transport system substrate-binding protein |  |
| xau:Xaut\_2618 | nitrate ABC transporter inner membrane subunit; K15577 nitrate/nitrite transport system permease protein |  |
| xau:Xaut\_2619 | ABC transporter-like protein; K15578 nitrate/nitrite transport system ATP-binding protein [EC:3.6.3.-] |  |
| xau:Xaut\_2620 | cyanate hydratase; K01725 cyanate lyase [EC:4.2.1.104] | ec:4.2.1.104 |
| xau:Xaut\_2621 | hypothetical protein |  |

  
**Neighborhood Representations for "cwo:Cwoe\_0417"**  

| ID | Annotation | EC number |
| --- | --- | --- |
| cwo:Cwoe\_0407 | major facilitator superfamily protein |  |
| cwo:Cwoe\_0408 | TetR family transcriptional regulator |  |
| cwo:Cwoe\_0409 | short-chain dehydrogenase/reductase SDR; K00059 3-oxoacyl-[acyl-carrier protein] reductase [EC:1.1.1.100] | ec:1.1.1.100 |
| cwo:Cwoe\_0410 | acyl-CoA dehydrogenase |  |
| cwo:Cwoe\_0411 | AMP-dependent synthetase and ligase; K01897 long-chain acyl-CoA synthetase [EC:6.2.1.3] | ec:6.2.1.3 |
| cwo:Cwoe\_0412 | hypothetical protein |  |
| cwo:Cwoe\_0413 | ABC transporter |  |
| cwo:Cwoe\_0414 | TetR family transcriptional regulator |  |
| cwo:Cwoe\_0415 | hypothetical protein |  |
| cwo:Cwoe\_0416 | major facilitator superfamily protein; K08156 MFS transporter, DHA1 family, arabinose polymer transporter |  |
| cwo:Cwoe\_0417 | TetR family transcriptional regulator |  |
| cwo:Cwoe\_0418 | hypothetical protein |  |
| cwo:Cwoe\_0419 | dihydroxy-acid dehydratase (EC:4.2.1.9); K01687 dihydroxy-acid dehydratase [EC:4.2.1.9] | ec:4.2.1.9 |
| cwo:Cwoe\_0420 | TetR family transcriptional regulator |  |
| cwo:Cwoe\_0421 | adenosylmethionine--8-amino-7-oxononanoate aminotransferase; K00833 adenosylmethionine-8-amino-7-oxononanoate aminotransferase [EC:2.6.1.62] | ec:2.6.1.62 |
| cwo:Cwoe\_0422 | biotin synthase (EC:2.8.1.6); K01012 biotin synthase [EC:2.8.1.6] | ec:2.8.1.6 |
| cwo:Cwoe\_0423 | hypothetical protein |  |
| cwo:Cwoe\_0424 | 8-amino-7-oxononanoate synthase (EC:2.3.1.47); K00652 8-amino-7-oxononanoate synthase [EC:2.3.1.47] | ec:2.3.1.47 |
| cwo:Cwoe\_0425 | PKD domain-containing protein |  |
| cwo:Cwoe\_0426 | hypothetical protein |  |
| cwo:Cwoe\_0427 | hypothetical protein |  |

  
**Over-represented Enzyme Summary**: Table of E.C. identified protein in the "Neighborhood Representation" ranked by frequency of occurrence  

| EC number | Frequency | Annotation | Reactions |
| --- | --- | --- | --- |
| ec:1.1.1.103 | 25 | L-threonine 3-dehydrogenase; L-threonine dehydrogenase; threonine 3-dehydrogenase; threonine dehydrogenase; TDH | L-threonine + NAD+ = L-2-amino-3-oxobutanoate + NADH + H+ [RN:R01465] |
| ec:2.3.1.29 | 25 | glycine C-acetyltransferase; 2-amino-3-ketobutyrate CoA ligase; 2-amino-3-ketobutyrate coenzyme A ligase; 2-amino-3-ketobutyrate-CoA ligase; glycine acetyltransferase; aminoacetone synthase; aminoacetone synthetase; KBL; AKB ligase | acetyl-CoA + glycine = CoA + L-2-amino-3-oxobutanoate [RN:R00371] |
| ec:4.1.1.73 | 20 | tartrate decarboxylase; (R,R)-tartrate carboxy-lyase | (R,R)-tartrate = D-glycerate + CO2 [RN:R01751] |
| ec:1.1.1.93 | 20 | tartrate dehydrogenase; mesotartrate dehydrogenase | tartrate + NAD+ = oxaloglycolate + NADH + H+ [RN:R02545 R06180] |
| ec:1.1.1.83 | 20 | D-malate dehydrogenase (decarboxylating); D-malate dehydrogenase; D-malic enzyme; bifunctional L(+)-tartrate dehydrogenase-D(+)-malate (decarboxylating) | (R)-malate + NAD+ = pyruvate + CO2 + NADH [RN:R00215] |
| ec:1.1.1.30 | 16 | 3-hydroxybutyrate dehydrogenase; NAD-beta-hydroxybutyrate dehydrogenase; hydroxybutyrate oxidoreductase; beta-hydroxybutyrate dehydrogenase; D-beta-hydroxybutyrate dehydrogenase; D-3-hydroxybutyrate dehydrogenase; D-(-)-3-hydroxybutyrate dehydrogenase; beta-hydroxybutyric acid dehydrogenase; 3-D-hydroxybutyrate dehydrogenase; beta-hydroxybutyric dehydrogenase | (R)-3-hydroxybutanoate + NAD+ = acetoacetate + NADH + H+ [RN:R01361] |
| ec:4.1.1.4 | 15 | acetoacetate decarboxylase; acetoacetic acid decarboxylase; acetoacetate carboxy-lyase | acetoacetate + H+ = acetone + CO2 [RN:R01366] |
| ec:2.5.1.18 | 9 | glutathione transferase; glutathione S-transferase; glutathione S-alkyltransferase; glutathione S-aryltransferase; S-(hydroxyalkyl)glutathione lyase; glutathione S-aralkyltransferase; glutathione S-alkyl transferase; GST | RX + glutathione = HX + R-S-glutathione [RN:R03522 R08511 R08512] |
| ec:1.2.1.38 | 8 | N-acetyl-gamma-glutamyl-phosphate reductase; reductase, acetyl-gamma-glutamyl phosphate; N-acetylglutamate 5-semialdehyde dehydrogenase; N-acetylglutamic gamma-semialdehyde dehydrogenase; N-acetyl-L-glutamate gamma-semialdehyde:NADP+ oxidoreductase (phosphorylating) | N-acetyl-L-glutamate 5-semialdehyde + NADP+ + phosphate = N-acetyl-L-glutamyl 5-phosphate + NADPH + H+ [RN:R03443] |
| ec:6.3.5.2 | 5 | GMP synthase (glutamine-hydrolysing); GMP synthetase (glutamine-hydrolysing); guanylate synthetase (glutamine-hydrolyzing); guanosine monophosphate synthetase (glutamine-hydrolyzing); xanthosine 5'-phosphate amidotransferase; guanosine 5'-monophosphate synthetase | ATP + XMP + L-glutamine + H2O = AMP + diphosphate + GMP + L-glutamate (overall reaction) [RN:R01231]; (1a) L-glutamine + H2O = L-glutamate + NH3 [RN:R00256]; (1b) ATP + XMP + NH3 = AMP + diphosphate + GMP [RN:R01230] |
| ec:5.4.2.12 | 4 | phosphoglycerate mutase (2,3-diphosphoglycerate-independent); cofactor independent phosphoglycerate mutase; 2,3-diphosphoglycerate-independent phosphoglycerate mutase; phosphoglycerate phosphomutase (ambiguous); phosphoglyceromutase (ambiguous); monophosphoglycerate mutase (ambiguous); monophosphoglyceromutase (ambiguous); GriP mutase (ambiguous); PGA mutase (ambiguous); iPGM; iPGAM; PGAM-i | 2-phospho-D-glycerate = 3-phospho-D-glycerate [RN:R01518] |
| ec:1.18.6.1 | 4 | nitrogenase | 8 reduced ferredoxin + 8 H+ + N2 + 16 ATP + 16 H2O = 8 oxidized ferredoxin + H2 + 2 NH3 + 16 ADP + 16 phosphate [RN:R05185] |
| ec:5.1.3.6 | 3 | UDP-glucuronate 4-epimerase; uridine diphospho-D-galacturonic acid; UDP glucuronic epimerase; uridine diphosphoglucuronic epimerase; UDP-galacturonate 4-epimerase; uridine diphosphoglucuronate epimerase; UDP-D-galacturonic acid 4-epimerase | UDP-glucuronate = UDP-D-galacturonate [RN:R01385] |
| ec:4.2.1.104 | 3 | cyanase; cyanate lyase; cyanate hydrolase; cyanate aminohydrolase; cyanate C-N-lyase; cyanate hydratase | cyanate + HCO3- + 2 H+ = NH3 + 2 CO2 (overall reaction) [RN:R10079]; (1a) cyanate + HCO3- + H+ = carbamate + CO2 [RN:R03546]; (1b) carbamate + H+ = NH3 + CO2 (spontaneous) [RN:R07316] |
| ec:2.1.1.177 | 3 | 23S rRNA (pseudouridine1915-N3)-methyltransferase; YbeA; RlmH; pseudouridine methyltransferase; m3Psi methyltransferase; Psi1915-specific methyltransferase; rRNA large subunit methyltransferase H | S-adenosyl-L-methionine + pseudouridine1915 in 23S rRNA = S-adenosyl-L-homocysteine + N3-methylpseudouridine1915 in 23S rRNA |
| ec:2.7.13.3 | 3 | histidine kinase; EnvZ; histidine kinase (ambiguous); histidine protein kinase (ambiguous); protein histidine kinase (ambiguous); protein kinase (histidine) (ambiguous); HK1; HP165; Sln1p | ATP + protein L-histidine = ADP + protein N-phospho-L-histidine |
| ec:2.7.8.8 | 3 | CDP-diacylglycerol---serine O-phosphatidyltransferase; phosphatidylserine synthase; CDPdiglyceride-serine O-phosphatidyltransferase; PS synthase; cytidine 5'-diphospho-1,2-diacyl-sn-glycerol (CDPdiglyceride):L-serine O-phosphatidyltransferase; phosphatidylserine synthetase; CDP-diacylglycerol-L-serine O-phosphatidyltransferase; cytidine diphosphoglyceride-serine O-phosphatidyltransferase; CDP-diglyceride-L-serine phosphatidyltransferase; CDP-diglyceride:serine phosphatidyltransferase; cytidine 5'-diphospho-1,2-diacyl-sn-glycerol:L-serine O-phosphatidyltransferase; CDP-diacylglycerol:L-serine 3-O-phosphatidyltransferase | CDP-diacylglycerol + L-serine = CMP + (3-sn-phosphatidyl)-L-serine [RN:R01800] |
| ec:1.20.4.1 | 3 | arsenate reductase (glutaredoxin) | arsenate + glutaredoxin = arsenite + glutaredoxin disulfide + H2O [RN:R05747] |
| ec:3.1.11.5 | 2 | exodeoxyribonuclease V; Escherichia coli exonuclease V; E. coli exonuclease V; gene recBC endoenzyme; RecBC deoxyribonuclease; gene recBC DNase; exonuclease V; gene recBCD enzymes | Exonucleolytic cleavage (in the presence of ATP) in either 5'- to 3'- or 3'- to 5'-direction to yield 5'-phosphooligonucleotides |
| ec:5.99.1.2 | 2 | DNA topoisomerase; type I DNA topoisomerase; untwisting enzyme; relaxing enzyme; nicking-closing enzyme; swivelase; omega-protein; deoxyribonucleate topoisomerase; topoisomerase; type I DNA topoisomerase | ATP-independent breakage of single-stranded DNA, followed by passage and rejoining |
| ec:4.2.1.84 | 2 | nitrile hydratase; nitrilase (ambiguous); 3-cyanopyridine hydratase; NHase; L-NHase; H-NHase; acrylonitrile hydratase; aliphatic nitrile hydratase; nitrile hydro-lyase | an aliphatic amide = a nitrile + H2O [RN:R02826] |
| ec:3.5.4.25 | 2 | GTP cyclohydrolase II; guanosine triphosphate cyclohydrolase II; GTP-8-formylhydrolase | GTP + 3 H2O = formate + 2,5-diamino-6-hydroxy-4-(5-phospho-D-ribosylamino)pyrimidine + diphosphate [RN:R00425] |
| ec:4.2.1.17 | 2 | enoyl-CoA hydratase; enoyl hydrase; unsaturated acyl-CoA hydratase; beta-hydroxyacyl-CoA dehydrase; beta-hydroxyacid dehydrase; hydratase, enoyl coenzyme A; acyl coenzyme A hydrase; crotonase; crotonyl hydrase; 2-octenoyl coenzyme A hydrase; enoyl coenzyme A hydratase; 2-enoyl-CoA hydratase; short-chain enoyl-CoA hydratase; ECH; trans-2-enoyl-CoA hydratase; enoyl coenzyme A hydrase (D); enoyl coenzyme A hydrase (L); short chain enoyl coenzyme A hydratase; D-3-hydroxyacyl-CoA dehydratase; enol-CoA hydratase | (3S)-3-hydroxyacyl-CoA = trans-2(or 3)-enoyl-CoA + H2O [RN:R02685 R07314] |
| ec:1.17.1.4 | 2 | xanthine dehydrogenase; NAD+-xanthine dehydrogenase; xanthine-NAD+ oxidoreductase; xanthine/NAD+ oxidoreductase; xanthine oxidoreductase | xanthine + NAD+ + H2O = urate + NADH + H+ [RN:R02103] |
| ec:3.6.1.22 | 2 | NAD+ diphosphatase; nicotinamide adenine dinucleotide pyrophosphatase; NADP pyrophosphatase; NADH pyrophosphatase | NAD+ + H2O = AMP + NMN [RN:R00103] |
| ec:4.2.1.3 | 2 | aconitate hydratase; cis-aconitase; aconitase; AcnB; 2-methylaconitate hydratase; citrate(isocitrate) hydro-lyase | citrate = isocitrate (overall reaction) [RN:R01324]; (1a) citrate = cis-aconitate + H2O [RN:R01325]; (1b) cis-aconitate + H2O = isocitrate [RN:R01900] |
| ec:4.2.1.1 | 2 | carbonate dehydratase; carbonic anhydrase; anhydrase; carbonate anhydrase; carbonic acid anhydrase; carboxyanhydrase; carbonic anhydrase A; carbonate hydro-lyase | H2CO3 = CO2 + H2O [RN:R00132] |
| ec:2.7.11.1 | 2 | non-specific serine/threonine protein kinase; A-kinase; AP50 kinase; ATP-protein transphosphorylase; calcium-dependent protein kinase C; calcium/phospholipid-dependent protein kinase; cAMP-dependent protein kinase; cAMP-dependent protein kinase A; casein kinase; casein kinase (phosphorylating); casein kinase 2; casein kinase I; casein kinase II; cGMP-dependent protein kinase; CK-2; CKI; CKII; cyclic AMP-dependent protein kinase; cyclic AMP-dependent protein kinase A; cyclic monophosphate-dependent protein kinase; cyclic nucleotide-dependent protein kinase; cyclin-dependent kinase; cytidine 3',5'-cyclic monophosphate-responsive protein kinase; dsk1; glycogen synthase a kinase; glycogen synthase kinase; HIPK2; Hpr kinase; hydroxyalkyl-protein kinase; hydroxyalkyl-protein kinase; M phase-specific cdc2 kinase; mitogen-activated S6 kinase; p82 kinase; phosphorylase b kinase kinase; PKA; protein glutamyl kinase; protein kinase (phosphorylating); protein kinase A; protein kinase CK2; protein kinase p58; protein phosphokinase; protein serine kinase; protein serine-threonine kinase; protein-aspartyl kinase; protein-cysteine kinase; protein-serine kinase; Prp4 protein kinase; Raf kinase; Raf-1; ribosomal protein S6 kinase II; ribosomal S6 protein kinase; serine kinase; serine protein kinase; serine-specific protein kinase; serine(threonine) protein kinase; serine/threonine protein kinase; STK32; T-antigen kinase; threonine-specific protein kinase; twitchin kinase; type-2 casein kinase; betaIIPKC; epsilon PKC; Wee 1-like kinase; Wee-kinase; WEE1Hu | ATP + a protein = ADP + a phosphoprotein [RN:R00162] |
| ec:2.7.7.7 | 2 | DNA-directed DNA polymerase; DNA polymerase I; DNA polymerase II; DNA polymerase III; DNA polymerase alpha; DNA polymerase beta; DNA polymerase gamma; DNA nucleotidyltransferase (DNA-directed); DNA nucleotidyltransferase (DNA-directed); deoxyribonucleate nucleotidyltransferase; deoxynucleate polymerase; deoxyribonucleic acid duplicase; deoxyribonucleic acid polymerase; deoxyribonucleic duplicase; deoxyribonucleic polymerase; deoxyribonucleic polymerase I; DNA duplicase; DNA nucleotidyltransferase; DNA polymerase; DNA replicase; DNA-dependent DNA polymerase; duplicase; Klenow fragment; sequenase; Taq DNA polymerase; Taq Pol I; Tca DNA polymerase | deoxynucleoside triphosphate + DNAn = diphosphate + DNAn+1 [RN:R00379] |
| ec:2.7.6.1 | 2 | ribose-phosphate diphosphokinase; ribose-phosphate pyrophosphokinase; PRPP synthetase; phosphoribosylpyrophosphate synthetase; PPRibP synthetase; PP-ribose P synthetase; 5-phosphoribosyl-1-pyrophosphate synthetase; 5-phosphoribose pyrophosphorylase; 5-phosphoribosyl-alpha-1-pyrophosphate synthetase; phosphoribosyl-diphosphate synthetase; phosphoribosylpyrophosphate synthase; pyrophosphoribosylphosphate synthetase; ribophosphate pyrophosphokinase; ribose-5-phosphate pyrophosphokinase | ATP + D-ribose 5-phosphate = AMP + 5-phospho-alpha-D-ribose 1-diphosphate [RN:R01049] |
| ec:1.6.99.3 | 2 | NADH dehydrogenase; cytochrome c reductase; type 1 dehydrogenase; beta-NADH dehydrogenase dinucleotide; diaphorase; dihydrocodehydrogenase I dehydrogenase; dihydronicotinamide adenine dinucleotide dehydrogenase; diphosphopyridine diaphorase; DPNH diaphorase; NADH diaphorase; NADH hydrogenase; NADH oxidoreductase; NADH-menadione oxidoreductase; reduced diphosphopyridine nucleotide diaphorase; NADH:cytochrome c oxidoreductase; NADH2 dehydrogenase; NADH:(acceptor) oxidoreductase | NADH + H+ + acceptor = NAD+ + reduced acceptor [RN:R00281] |
| ec:4.1.1.23 | 2 | orotidine-5'-phosphate decarboxylase; orotidine-5'-monophosphate decarboxylase; orotodylate decarboxylase; orotidine phosphate decarboxylase; OMP decarboxylase; orotate monophosphate decarboxylase; orotidine monophosphate decarboxylase; orotidine phosphate decarboxylase; OMP-DC; orotate decarboxylase; orotidine 5'-phosphate decarboxylase; orotidylic decarboxylase; orotidylic acid decarboxylase; orotodylate decarboxylase; ODCase; orotic decarboxylase; orotidine-5'-phosphate carboxy-lyase | orotidine 5'-phosphate = UMP + CO2 [RN:R00965] |
| ec:6.2.1.3 | 2 | long-chain-fatty-acid---CoA ligase; acyl-CoA synthetase; fatty acid thiokinase (long chain); acyl-activating enzyme; palmitoyl-CoA synthase; lignoceroyl-CoA synthase; arachidonyl-CoA synthetase; acyl coenzyme A synthetase; acyl-CoA ligase; palmitoyl coenzyme A synthetase; thiokinase; palmitoyl-CoA ligase; acyl-coenzyme A ligase; fatty acid CoA ligase; long-chain fatty acyl coenzyme A synthetase; oleoyl-CoA synthetase; stearoyl-CoA synthetase; long chain fatty acyl-CoA synthetase; long-chain acyl CoA synthetase; fatty acid elongase; LCFA synthetase; pristanoyl-CoA synthetase; ACS3; long-chain acyl-CoA synthetase I; long-chain acyl-CoA synthetase II; fatty acyl-coenzyme A synthetase; long-chain acyl-coenzyme A synthetase; FAA1 | ATP + a long-chain fatty acid + CoA = AMP + diphosphate + an acyl-CoA [RN:R00390] |
| ec:6.1.1.13 | 2 | D-alanine---poly(phosphoribitol) ligase; D-alanyl-poly(phosphoribitol) synthetase; D-alanine: membrane acceptor ligase; D-alanine-D-alanyl carrier protein ligase; D-alanine-membrane acceptor ligase; D-alanine-activating enzyme | ATP + D-alanine + poly(ribitol phosphate) = AMP + diphosphate + O-D-alanyl-poly(ribitol phosphate) [RN:R02718] |
| ec:2.3.1.47 | 1 | 8-amino-7-oxononanoate synthase; 7-keto-8-aminopelargonic acid synthetase; 7-keto-8-aminopelargonic synthetase; 8-amino-7-oxopelargonate synthase; bioF (gene name) | pimeloyl-[acyl-carrier protein] + L-alanine = 8-amino-7-oxononanoate + CO2 + holo-[acyl-carrier protein] [RN:R03210] |
| ec:1.1.1.1 | 1 | alcohol dehydrogenase; aldehyde reductase; ADH; alcohol dehydrogenase (NAD); aliphatic alcohol dehydrogenase; ethanol dehydrogenase; NAD-dependent alcohol dehydrogenase; NAD-specific aromatic alcohol dehydrogenase; NADH-alcohol dehydrogenase; NADH-aldehyde dehydrogenase; primary alcohol dehydrogenase; yeast alcohol dehydrogenase | (1) a primary alcohol + NAD+ = an aldehyde + NADH + H+ [RN:R07326]; (2) a secondary alcohol + NAD+ = a ketone + NADH + H+ [RN:R07327] |
| ec:5.4.99.29 | 1 | 23S rRNA pseudouridine746 synthase; RluA (ambiguous); 23S RNA PSI746 synthase; 23S rRNA pseudouridine synthase; pseudouridine synthase RluA (ambiguous) | 23S rRNA uridine746 = 23S rRNA pseudouridine746 |
| ec:1.18.1.3 | 1 | ferredoxin---NAD+ reductase; ferredoxin-nicotinamide adenine dinucleotide reductase; ferredoxin reductase (ambiguous); NAD+-ferredoxin reductase; NADH-ferredoxin oxidoreductase; reductase, reduced nicotinamide adenine dinucleotide-ferredoxin; ferredoxin-NAD+ reductase; NADH-ferredoxin reductase; NADH2-ferredoxin oxidoreductase; NADH flavodoxin oxidoreductase; NADH-ferredoxin NAP reductase (component of naphthalene dioxygenase multicomponent enzyme system); ferredoxin-linked NAD+ reductase; NADH-ferredoxin TOL reductase (component of toluene dioxygenase); ferredoxin---NAD reductase | (1) 2 reduced [2Fe-2S] ferredoxin + NAD+ + H+ = 2 oxidized [2Fe-2S] ferredoxin + NADH [RN:R05875]; (2) reduced 2[4Fe-4S] ferredoxin + NAD+ + H+ = oxidized 2[4Fe-4S] ferredoxin + NADH |
| ec:5.4.99.28 | 1 | tRNA pseudouridine32 synthase; RluA (ambiguous); pseudouridine synthase RluA (ambiguous); Pus9p; Rib2/Pus8p | tRNA uridine32 = tRNA pseudouridine32 |
| ec:1.14.15.11 | 1 | pentalenic acid synthase; CYP105D7; sav7469 (gene name) | 1-deoxypentalenate + reduced ferredoxin + O2 = pentalenate + oxidized ferredoxin + H2O [RN:R10158] |
| ec:4.1.2.13 | 1 | fructose-bisphosphate aldolase; aldolase; fructose-1,6-bisphosphate triosephosphate-lyase; fructose diphosphate aldolase; diphosphofructose aldolase; fructose 1,6-diphosphate aldolase; ketose 1-phosphate aldolase; phosphofructoaldolase; zymohexase; fructoaldolase; fructose 1-phosphate aldolase; fructose 1-monophosphate aldolase; 1,6-Diphosphofructose aldolase; SMALDO; D-fructose-1,6-bisphosphate D-glyceraldehyde-3-phosphate-lyase | D-fructose 1,6-bisphosphate = glycerone phosphate + D-glyceraldehyde 3-phosphate [RN:R01068] |
| ec:2.5.1.6 | 1 | methionine adenosyltransferase; adenosylmethionine synthetase; ATP-methionine adenosyltransferase; methionine S-adenosyltransferase; methionine-activating enzyme; S-adenosyl-L-methionine synthetase; S-adenosylmethionine synthase; S-adenosylmethionine synthetase; AdoMet synthetase | ATP + L-methionine + H2O = phosphate + diphosphate + S-adenosyl-L-methionine [RN:R00177] |
| ec:2.7.1.17 | 1 | xylulokinase; xylulokinase (phosphorylating); D-xylulokinase | ATP + D-xylulose = ADP + D-xylulose 5-phosphate [RN:R01639] |
| ec:2.7.7.87 | 1 | L-threonylcarbamoyladenylate synthase; yrdC (gene name); Sua5; ywlC (gene name) | L-threonine + ATP + bicarbonate = L-threonylcarbamoyladenylate + diphosphate + H2O [RN:R10463] |
| ec:3.1.1.45 | 1 | carboxymethylenebutenolidase; maleylacetate enol-lactonase; dienelactone hydrolase; carboxymethylene butenolide hydrolase | 4-carboxymethylenebut-2-en-4-olide + H2O = 4-oxohex-2-enedioate [RN:R03893] |
| ec:2.6.1.62 | 1 | adenosylmethionine---8-amino-7-oxononanoate transaminase; 7,8-diaminonanoate transaminase; 7,8-diaminononanoate transaminase; DAPA transaminase (ambiguous); 7,8-diaminopelargonic acid aminotransferase; DAPA aminotransferase (ambiguous); 7-keto-8-aminopelargonic acid; diaminopelargonate synthase; 7-keto-8-aminopelargonic acid aminotransferase | S-adenosyl-L-methionine + 8-amino-7-oxononanoate = S-adenosyl-4-methylthio-2-oxobutanoate + 7,8-diaminononanoate [RN:R03231] |
| ec:2.7.1.71 | 1 | shikimate kinase; shikimate kinase (phosphorylating); shikimate kinase II | ATP + shikimate = ADP + 3-phosphoshikimate [RN:R02412] |
| ec:2.1.1.14 | 1 | 5-methyltetrahydropteroyltriglutamate---homocysteine S-methyltransferase; tetrahydropteroyltriglutamate methyltransferase; homocysteine methylase; methyltransferase, tetrahydropteroylglutamate-homocysteine transmethylase; methyltetrahydropteroylpolyglutamate:homocysteine methyltransferase; cobalamin-independent methionine synthase; methionine synthase (cobalamin-independent); MetE | 5-methyltetrahydropteroyltri-L-glutamate + L-homocysteine = tetrahydropteroyltri-L-glutamate + L-methionine [RN:R04405] |
| ec:3.3.2.9 | 1 | microsomal epoxide hydrolase; epoxide hydratase (ambiguous); microsomal epoxide hydratase (ambiguous); epoxide hydrase; microsomal epoxide hydrase; arene-oxide hydratase (ambiguous); benzo[a]pyrene-4,5-oxide hydratase; benzo(a)pyrene-4,5-epoxide hydratase; aryl epoxide hydrase (ambiguous); cis-epoxide hydrolase; mEH | cis-stilbene oxide + H2O = (+)-(1R,2R)-1,2-diphenylethane-1,2-diol [RN:R07627] |
| ec:1.1.1.262 | 1 | 4-hydroxythreonine-4-phosphate dehydrogenase; NAD+-dependent threonine 4-phosphate dehydrogenase; L-threonine 4-phosphate dehydrogenase; 4-(phosphohydroxy)-L-threonine dehydrogenase; PdxA; 4-(phosphonooxy)-L-threonine:NAD+ oxidoreductase | 4-phosphonooxy-L-threonine + NAD+ = 3-amino-2-oxopropyl phosphate + CO2 + NADH + H+ (overall reaction) [RN:R05837]; (1a) 4-phosphonooxy-L-threonine + NAD+ = (2S)-2-amino-3-oxo-4-phosphonooxybutanoate + NADH + H+ [RN:R05681]; (1b) (2S)-2-amino-3-oxo-4-phosphonooxybutanoate = 3-amino-2-oxopropyl phosphate + CO2 (spontaneous) [RN:R07406] |
| ec:6.3.4.21 | 1 | nicotinate phosphoribosyltransferase; niacin ribonucleotidase; nicotinic acid mononucleotide glycohydrolase; nicotinic acid mononucleotide pyrophosphorylase; nicotinic acid phosphoribosyltransferase; nicotinate-nucleotide:diphosphate phospho-alpha-D-ribosyltransferase | nicotinate + 5-phospho-alpha-D-ribose 1-diphosphate + ATP + H2O = beta-nicotinate D-ribonucleotide + diphosphate + ADP + phosphate [RN:R01724] |
| ec:1.1.1.100 | 1 | 3-oxoacyl-[acyl-carrier-protein] reductase; beta-ketoacyl-[acyl-carrier protein](ACP) reductase; beta-ketoacyl acyl carrier protein (ACP) reductase; beta-ketoacyl reductase; beta-ketoacyl thioester reductase; beta-ketoacyl-ACP reductase; beta-ketoacyl-acyl carrier protein reductase; 3-ketoacyl acyl carrier protein reductase; NADPH-specific 3-oxoacyl-[acylcarrier protein]reductase; 3-oxoacyl-[ACP]reductase; (3R)-3-hydroxyacyl-[acyl-carrier-protein]:NADP+ oxidoreductase | a (3R)-3-hydroxyacyl-[acyl-carrier protein] + NADP+ = a 3-oxoacyl-[acyl-carrier protein] + NADPH + H+ [RN:R02767] |
| ec:1.3.99.31 | 1 | phytoene desaturase (lycopene-forming); 4-step phytoene desaturase; four-step phytoene desaturase; phytoene desaturase (ambiguous); CrtI (ambiguous) | 15-cis-phytoene + 4 acceptor = all-trans-lycopene + 4 reduced acceptor (overall reaction) [RN:R09716]; (1a) 15-cis-phytoene + acceptor = all-trans-phytofluene + reduced acceptor [RN:R09692]; (1b) all-trans-phytofluene + acceptor = all-trans-zeta-carotene + reduced acceptor [RN:R04787]; (1c) all-trans-zeta-carotene + acceptor = all-trans-neurosporene + reduced acceptor [RN:R04798]; (1d) all-trans-neurosporene + acceptor = all-trans-lycopene + reduced acceptor [RN:R04800] |
| ec:3.5.1.19 | 1 | nicotinamidase; nicotinamide deaminase; nicotinamide amidase; YNDase | nicotinamide + H2O = nicotinate + NH3 [RN:R01268] |
| ec:1.11.1.10 | 1 | chloride peroxidase; chloroperoxidase; CPO; vanadium haloperoxidase | RH + chloride + H2O2 = RCl + 2 H2O [RN:R00052] |
| ec:4.2.1.9 | 1 | dihydroxy-acid dehydratase; acetohydroxyacid dehydratase; alpha,beta-dihydroxyacid dehydratase; 2,3-dihydroxyisovalerate dehydratase; alpha,beta-dihydroxyisovalerate dehydratase; dihydroxy acid dehydrase; DHAD; 2,3-dihydroxy-acid hydro-lyase | 2,3-dihydroxy-3-methylbutanoate = 3-methyl-2-oxobutanoate + H2O [RN:R01209] |
| ec:3.8.1.2 | 1 | (S)-2-haloacid dehalogenase; 2-haloacid dehalogenase[ambiguous]; 2-haloacid halidohydrolase [ambiguous][ambiguous]; 2-haloalkanoic acid dehalogenase; 2-haloalkanoid acid halidohydrolase; 2-halocarboxylic acid dehalogenase II; DL-2-haloacid dehalogenase[ambiguous]; L-2-haloacid dehalogenase; L-DEX | (S)-2-haloacid + H2O = (R)-2-hydroxyacid + halide [RN:R03830] |
| ec:1.1.1.95 | 1 | phosphoglycerate dehydrogenase; D-3-phosphoglycerate:NAD+ oxidoreductase; alpha-phosphoglycerate dehydrogenase; 3-phosphoglycerate dehydrogenase; 3-phosphoglyceric acid dehydrogenase; D-3-phosphoglycerate dehydrogenase; glycerate 3-phosphate dehydrogenase; glycerate-1,3-phosphate dehydrogenase; phosphoglycerate oxidoreductase; phosphoglyceric acid dehydrogenase; SerA; 3-phosphoglycerate:NAD+ 2-oxidoreductase; SerA 3PG dehydrogenase; 3PHP reductase; alphaKG reductase; D- and L-HGA | (1) 3-phospho-D-glycerate + NAD+ = 3-phosphonooxypyruvate + NADH + H+ [RN:R01513]; (2) 2-hydroxyglutarate + NAD+ = 2-oxoglutarate + NADH + H+ [RN:R08198] |
| ec:3.6.3.21 | 1 | polar-amino-acid-transporting ATPase; histidine permease | ATP + H2O + polar amino acidout = ADP + phosphate + polar amino acidin [RN:R00086] |
| ec:1.3.99.29 | 1 | phytoene desaturase (zeta-carotene-forming); CrtIa; 2-step phytoene desaturase (ambiguous); two-step phytoene desaturase (ambiguous) | 15-cis-phytoene + 2 acceptor = all-trans-zeta-carotene + 2 reduced acceptor (overall reaction) [RN:R09693]; (1a) 15-cis-phytoene + acceptor = all-trans-phytofluene + reduced acceptor [RN:R09692]; (1b) all-trans-phytofluene + acceptor = all-trans-zeta-carotene + reduced acceptor [RN:R04787] |
| ec:1.3.99.28 | 1 | phytoene desaturase (neurosporene-forming); 3-step phytoene desaturase; three-step phytoene desaturase; phytoene desaturase (ambiguous); CrtI (ambiguous) | 15-cis-phytoene + 3 acceptor = all-trans-neurosporene + 3 reduced acceptor (overall reaction) [RN:R09691]; (1a) 15-cis-phytoene + acceptor = all-trans-phytofluene + reduced acceptor [RN:R09692]; (1b) all-trans-phytofluene + acceptor = all-trans-zeta-carotene + reduced acceptor [RN:R04787]; (1c) all-trans-zeta-carotene + acceptor = all-trans-neurosporene + reduced acceptor [RN:R04798] |
| ec:3.5.4.10 | 1 | IMP cyclohydrolase; inosinicase; inosinate cyclohydrolase | IMP + H2O = 5-formamido-1-(5-phospho-D-ribosyl)imidazole-4-carboxamide [RN:R01127] |
| ec:6.3.4.13 | 1 | phosphoribosylamine---glycine ligase; phosphoribosylglycinamide synthetase; glycinamide ribonucleotide synthetase; phosphoribosylglycineamide synthetase; glycineamide ribonucleotide synthetase; 2-amino-N-ribosylacetamide 5'-phosphate kinosynthase; 5'-phosphoribosylglycinamide synthetase; GAR | ATP + 5-phospho-D-ribosylamine + glycine = ADP + phosphate + N1-(5-phospho-D-ribosyl)glycinamide [RN:R04144] |
| ec:1.3.99.26 | 1 | all-trans-zeta-carotene desaturase; Crtlb; phytoene desaturase (ambiguous); 2-step phytoene desaturase (ambiguous); two-step phytoene desaturase (ambiguous); CrtI (ambiguous) | all-trans-zeta-carotene + 2 acceptor = all-trans-lycopene + 2 reduced acceptor (overall reaction) [RN:R09659]; (1a) all-trans-zeta-carotene + acceptor = all-trans-neurosporene + reduced acceptor [RN:R04798]; (1b) all-trans-neurosporene + acceptor = all-trans-lycopene + reduced acceptor [RN:R04800] |
| ec:1.11.1.5 | 1 | cytochrome-c peroxidase; cytochrome peroxidase; cytochrome c-551 peroxidase; apocytochrome c peroxidase; mesocytochrome c peroxidase azide; mesocytochrome c peroxidase cyanide; mesocytochrome c peroxidase cyanate; cytochrome c-H2O oxidoreductase; cytochrome c peroxidase | 2 ferrocytochrome c + H2O2 = 2 ferricytochrome c + 2 H2O [RN:R00017] |
| ec:6.5.1.1 | 1 | DNA ligase (ATP); polydeoxyribonucleotide synthase (ATP); polynucleotide ligase; sealase; DNA repair enzyme; DNA joinase; DNA ligase; deoxyribonucleic ligase; deoxyribonucleate ligase; DNA-joining enzyme; deoxyribonucleic-joining enzyme; deoxyribonucleic acid-joining enzyme; deoxyribonucleic repair enzyme; deoxyribonucleic joinase; deoxyribonucleic acid ligase; deoxyribonucleic acid joinase; deoxyribonucleic acid repair enzyme | ATP + (deoxyribonucleotide)n + (deoxyribonucleotide)m = AMP + diphosphate + (deoxyribonucleotide)n+m [RN:R00381] |
| ec:1.3.8.7 | 1 | medium-chain acyl-CoA dehydrogenase; fatty acyl coenzyme A dehydrogenase (ambiguous); acyl coenzyme A dehydrogenase (ambiguous); acyl dehydrogenase (ambiguous); fatty-acyl-CoA dehydrogenase (ambiguous); acyl CoA dehydrogenase (ambiguous); general acyl CoA dehydrogenase (ambiguous); medium-chain acyl-coenzyme A dehydrogenase; acyl-CoA:(acceptor) 2,3-oxidoreductase (ambiguous); ACADM (gene name). | a medium-chain acyl-CoA + electron-transfer flavoprotein = a medium-chain trans-2,3-dehydroacyl-CoA + reduced electron-transfer flavoprotein [RN:R00392] |
| ec:2.3.3.9 | 1 | malate synthase; L-malate glyoxylate-lyase (CoA-acetylating); glyoxylate transacetylase; glyoxylate transacetase; glyoxylic transacetase; malate condensing enzyme; malate synthetase; malic synthetase; malic-condensing enzyme | acetyl-CoA + H2O + glyoxylate = (S)-malate + CoA [RN:R00472] |
| ec:3.1.1.29 | 1 | aminoacyl-tRNA hydrolase; aminoacyl-transfer ribonucleate hydrolase; N-substituted aminoacyl transfer RNA hydrolase; peptidyl-tRNA hydrolase | N-substituted aminoacyl-tRNA + H2O = N-substituted amino acid + tRNA [RN:R04238] |
| ec:1.16.1.1 | 1 | mercury(II) reductase; mercuric reductase; mercurate(II) reductase; mercuric ion reductase; mercury reductase; reduced NADP:mercuric ion oxidoreductase; mer A | Hg + NADP+ + H+ = Hg2+ + NADPH [RN:R02807] |
| ec:4.1.3.1 | 1 | isocitrate lyase; isocitrase; isocitritase; isocitratase; threo-Ds-isocitrate glyoxylate-lyase; isocitrate glyoxylate-lyase | isocitrate = succinate + glyoxylate [RN:R00479] |
| ec:3.6.3.17 | 1 | monosaccharide-transporting ATPase | ATP + H2O + monosaccharideout = ADP + phosphate + monosaccharidein [RN:R00086] |
| ec:2.3.1.9 | 1 | acetyl-CoA C-acetyltransferase; acetoacetyl-CoA thiolase; beta-acetoacetyl coenzyme A thiolase; 2-methylacetoacetyl-CoA thiolase [misleading]; 3-oxothiolase; acetyl coenzyme A thiolase; acetyl-CoA acetyltransferase; acetyl-CoA:N-acetyltransferase; thiolase II | 2 acetyl-CoA = CoA + acetoacetyl-CoA [RN:R00238] |
| ec:2.3.1.15 | 1 | glycerol-3-phosphate 1-O-acyltransferase; alpha-glycerophosphate acyltransferase; 3-glycerophosphate acyltransferase; ACP:sn-glycerol-3-phosphate acyltransferase; glycerol 3-phosphate acyltransferase; glycerol phosphate acyltransferase; glycerol phosphate transacylase; glycerophosphate acyltransferase; glycerophosphate transacylase; sn-glycerol 3-phosphate acyltransferase; sn-glycerol-3-phosphate acyltransferase; glycerol-3-phosphate O-acyltransferase (ambiguous) | acyl-CoA + sn-glycerol 3-phosphate = CoA + 1-acyl-sn-glycerol 3-phosphate [RN:R00851] |
| ec:2.5.1.32 | 1 | 15-cis-phytoene synthase; prephytoene-diphosphate synthase (ambiguous); phytoene synthetase (ambiguous); PSase (ambiguous); geranylgeranyl-diphosphate geranylgeranyltransferase (ambiguous) | 2 geranylgeranyl diphosphate = 15-cis-phytoene + 2 diphosphate (overall reaction) [RN:R10177]; (1a) 2 geranylgeranyl diphosphate = diphosphate + prephytoene diphosphate [RN:R02065]; (1b) prephytoene diphosphate = 15-cis-phytoene + diphosphate [RN:R04218] |
| ec:3.6.4.12 | 1 | DNA helicase; 3' to 5' DNA helicase; 3'-5' DNA helicase; 3'-5' PfDH; 5' to 3' DNA helicase; AvDH1; BACH1 helicase; BcMCM; BLM protein; BRCA1-associated C-terminal helicase; CeWRN-1; Dbp9p; DmRECQ5; DNA helicase 120; DNA helicase A; DNA helicase E; DNA helicase II; DNA helicase III; DNA helicase RECQL5beta; DNA helicase VI; dnaB; DnaB helicase E1; helicase HDH IV; Hel E; helicase DnaB; helicase domain of bacteriophage T7 gene 4 protein helicase; PcrA helicase; UvrD; hHcsA; Hmi1p; hPif1; MCM helicase; MCM protein; MER3 helicase; MER3 protein; MPH1; PcrA; PcrA helicase; PDH120; PfDH A; Pfh1p; PIF1 | ATP + H2O = ADP + phosphate [RN:R00086] |
| ec:2.4.1.83 | 1 | dolichyl-phosphate beta-D-mannosyltransferase; GDP-Man:DolP mannosyltransferase; dolichyl mannosyl phosphate synthase; dolichyl-phospho-mannose synthase; GDP-mannose:dolichyl-phosphate mannosyltransferase; guanosine diphosphomannose-dolichol phosphate mannosyltransferase; dolichol phosphate mannose synthase; dolichyl phosphate mannosyltransferase; dolichyl-phosphate mannose synthase; GDP-mannose-dolichol phosphate mannosyltransferase; GDP-mannose-dolichylmonophosphate mannosyltransferase; mannosylphosphodolichol synthase; mannosylphosphoryldolichol synthase | GDP-mannose + dolichyl phosphate = GDP + dolichyl D-mannosyl phosphate [RN:R01009] |
| ec:2.8.1.6 | 1 | biotin synthase; dethiobiotin:sulfur sulfurtransferase | dethiobiotin + sulfur-(sulfur carrier) + 2 S-adenosyl-L-methionine = biotin + (sulfur carrier) + 2 L-methionine + 2 5'-deoxyadenosine [RN:R01078] |
| ec:2.7.7.6 | 1 | DNA-directed RNA polymerase; RNA polymerase; RNA nucleotidyltransferase (DNA-directed); RNA polymerase I; RNA polymerase II; RNA polymerase III; C RNA formation factors; deoxyribonucleic acid-dependent ribonucleic acid polymerase; DNA-dependent ribonucleate nucleotidyltransferase; DNA-dependent RNA nucleotidyltransferase; DNA-dependent RNA polymerase; ribonucleate nucleotidyltransferase; ribonucleate polymerase; C ribonucleic acid formation factors; ribonucleic acid nucleotidyltransferase; ribonucleic acid polymerase; ribonucleic acid transcriptase; ribonucleic polymerase; ribonucleic transcriptase; RNA nucleotidyltransferase; RNA transcriptase; transcriptase; RNA nucleotidyltransferase I | nucleoside triphosphate + RNAn = diphosphate + RNAn+1 [RN:R00444] |
| ec:2.3.3.14 | 1 | homocitrate synthase; 2-hydroxybutane-1,2,4-tricarboxylate 2-oxoglutarate-lyase (CoA-acetylating); acetyl-coenzyme A:2-ketoglutarate C-acetyl transferase; homocitrate synthetase; HCS | acetyl-CoA + H2O + 2-oxoglutarate = (2R)-2-hydroxybutane-1,2,4-tricarboxylate + CoA [RN:R00271] |
| ec:1.13.11.54 | 1 | acireductone dioxygenase [iron(II)-requiring]; ARD'; 2-hydroxy-3-keto-5-thiomethylpent-1-ene dioxygenase (ambiguous); acireductone dioxygenase (ambiguous); E-2'; E-3 dioxygenase | 1,2-dihydroxy-5-(methylthio)pent-1-en-3-one + O2 = 4-(methylthio)-2-oxobutanoate + formate [RN:R07364] |
| ec:1.13.11.53 | 1 | acireductone dioxygenase (Ni2+-requiring); ARD; 2-hydroxy-3-keto-5-thiomethylpent-1-ene dioxygenase (ambiguous); acireductone dioxygenase (ambiguous); E-2 | 1,2-dihydroxy-5-(methylthio)pent-1-en-3-one + O2 = 3-(methylthio)propanoate + formate + CO [RN:R07363] |
| ec:2.8.1.2 | 1 | 3-mercaptopyruvate sulfurtransferase; beta-mercaptopyruvate sulfurtransferase | 3-mercaptopyruvate + cyanide = pyruvate + thiocyanate [RN:R03106] |
| ec:4.99.1.5 | 1 | aliphatic aldoxime dehydratase; OxdA; aliphatic aldoxime hydro-lyase | an aliphatic aldoxime = an aliphatic nitrile + H2O [RN:R02827] |
| ec:2.8.1.1 | 1 | thiosulfate sulfurtransferase; thiosulfate cyanide transsulfurase; thiosulfate thiotransferase; rhodanese; rhodanase | thiosulfate + cyanide = sulfite + thiocyanate [RN:R01931] |
| ec:1.3.3.3 | 1 | coproporphyrinogen oxidase; coproporphyrinogen III oxidase; coproporphyrinogenase | coproporphyrinogen III + O2 + 2 H+ = protoporphyrinogen-IX + 2 CO2 + 2 H2O [RN:R03220] |
| ec:4.1.1.44 | 1 | 4-carboxymuconolactone decarboxylase; gamma-4-carboxymuconolactone decarboxylase; 4-carboxymuconolactone carboxy-lyase; 2-carboxy-2,5-dihydro-5-oxofuran-2-acetate carboxy-lyase (4,5-dihydro-5-oxofuran-2-acetate-forming) | (R)-2-carboxy-2,5-dihydro-5-oxofuran-2-acetate = 4,5-dihydro-5-oxofuran-2-acetate + CO2 [RN:R03470] |
| ec:3.1.1.17 | 1 | gluconolactonase; lactonase; aldonolactonase; glucono-delta-lactonase; gulonolactonase | D-glucono-1,5-lactone + H2O = D-gluconate [RN:R01519] |
| ec:3.5.99.2 | 1 | aminopyrimidine aminohydrolase; thiaminase; thiaminase II; tenA (gene name) | (1) 4-amino-5-aminomethyl-2-methylpyrimidine + H2O = 4-amino-5-hydroxymethyl-2-methylpyrimidine + ammonia [RN:R09993]; (2) thiamine + H2O = 4-amino-5-hydroxymethyl-2-methylpyrimidine + 5-(2-hydroxyethyl)-4-methylthiazole [RN:R02133] |
| ec:2.7.4.8 | 1 | guanylate kinase; deoxyguanylate kinase; 5'-GMP kinase; GMP kinase; guanosine monophosphate kinase; ATP:GMP phosphotransferase | ATP + GMP = ADP + GDP [RN:R00332] |
| ec:3.2.1.23 | 1 | beta-galactosidase; lactase (ambiguous); beta-lactosidase; maxilact; hydrolact; beta-D-lactosidase; S 2107; lactozym; trilactase; beta-D-galactanase; oryzatym; sumiklat | Hydrolysis of terminal non-reducing beta-D-galactose residues in beta-D-galactosides |
| ec:2.4.1.18 | 1 | 1,4-alpha-glucan branching enzyme; branching enzyme; amylo-(1,4->1,6)-transglycosylase; Q-enzyme; alpha-glucan-branching glycosyltransferase; amylose isomerase; enzymatic branching factor; branching glycosyltransferase; enzyme Q; glucosan transglycosylase; glycogen branching enzyme; plant branching enzyme; alpha-1,4-glucan:alpha-1,4-glucan-6-glycosyltransferase; starch branching enzyme; 1,4-alpha-D-glucan:1,4-alpha-D-glucan 6-alpha-D-(1,4-alpha-D-glucano)-transferase | Transfers a segment of a (1->4)-alpha-D-glucan chain to a primary hydroxy group in a similar glucan chain [RN:R02110 R06186] |
| ec:3.2.1.21 | 1 | beta-glucosidase; gentiobiase; cellobiase; emulsin; elaterase; aryl-beta-glucosidase; beta-D-glucosidase; beta-glucoside glucohydrolase; arbutinase; amygdalinase; p-nitrophenyl beta-glucosidase; primeverosidase; amygdalase; linamarase; salicilinase; beta-1,6-glucosidase | Hydrolysis of terminal, non-reducing beta-D-glucosyl residues with release of beta-D-glucose |
| ec:1.2.1.20 | 1 | glutarate-semialdehyde dehydrogenase; glutarate semialdehyde dehydrogenase | 5-oxopentanoate + NAD+ + H2O = glutarate + NADH + 2 H+ [RN:R02401] |
| ec:6.3.4.4 | 1 | adenylosuccinate synthase; IMP---aspartate ligase; adenylosuccinate synthetase; succinoadenylic kinosynthetase; succino-AMP synthetase | GTP + IMP + L-aspartate = GDP + phosphate + N6-(1,2-dicarboxyethyl)-AMP [RN:R01135] |
| ec:2.4.1.11 | 1 | glycogen(starch) synthase; UDP-glucose---glycogen glucosyltransferase; glycogen (starch) synthetase; UDP-glucose-glycogen glucosyltransferase; UDP-glycogen synthase; UDPG-glycogen synthetase; UDPG-glycogen transglucosylase; uridine diphosphoglucose-glycogen glucosyltransferase | UDP-glucose + [(1->4)-alpha-D-glucosyl]n = UDP + [(1->4)-alpha-D-glucosyl]n+1 [RN:R00292 R06051] |
| ec:6.3.2.5 | 1 | phosphopantothenate---cysteine ligase; phosphopantothenoylcysteine synthetase | CTP + (R)-4'-phosphopantothenate + L-cysteine = CMP + diphosphate + N-[(R)-4'-phosphopantothenoyl]-L-cysteine [RN:R04231] |
| ec:3.5.5.7 | 1 | aliphatic nitrilase | R-CN + 2 H2O = R-COOH + NH3 [RN:R00540] |
| ec:4.1.1.36 | 1 | phosphopantothenoylcysteine decarboxylase; 4-phosphopantotheoylcysteine decarboxylase; 4-phosphopantothenoyl-L-cysteine decarboxylase; PPC-decarboxylase; N-[(R)-4'-phosphopantothenoyl]-L-cysteine carboxy-lyase | N-[(R)-4'-phosphopantothenoyl]-L-cysteine = pantotheine 4'-phosphate + CO2 [RN:R03269] |
| ec:4.2.1.41 | 1 | 5-dehydro-4-deoxyglucarate dehydratase; 5-keto-4-deoxy-glucarate dehydratase; 5-keto-4-deoxy-glucarate dehydratase; deoxyketoglucarate dehydratase; D-4-deoxy-5-ketoglucarate hydro-lyase; 5-dehydro-4-deoxy-D-glucarate hydro-lyase (decarboxylating) | 5-dehydro-4-deoxy-D-glucarate = 2,5-dioxopentanoate + H2O + CO2 [RN:R02279] |
| ec:4.2.1.40 | 1 | glucarate dehydratase; D-glucarate dehydratase; D-glucarate hydro-lyase | D-glucarate = 5-dehydro-4-deoxy-D-glucarate + H2O [RN:R02752] |
| ec:1.2.1.16 | 1 | succinate-semialdehyde dehydrogenase [NAD(P)+]; succinate semialdehyde dehydrogenase (nicotinamide adenine dinucleotide (phosphate)); succinate-semialdehyde dehydrogenase [NAD(P)] | succinate semialdehyde + NAD(P)+ + H2O = succinate + NAD(P)H + 2 H+ [RN:R00713 R00714] |
| ec:2.7.1.39 | 1 | homoserine kinase; homoserine kinase (phosphorylating); HSK | ATP + L-homoserine = ADP + O-phospho-L-homoserine [RN:R01771] |
| ec:1.2.1.79 | 1 | succinate-semialdehyde dehydrogenase (NADP+); succinic semialdehyde dehydrogenase (NADP+); succinyl semialdehyde dehydrogenase (NADP+); succinate semialdehyde:NADP+ oxidoreductase; NADP-dependent succinate-semialdehyde dehydrogenase; GabD | succinate semialdehyde + NADP+ + H2O = succinate + NADPH + 2 H+ [RN:R00714] |
| ec:3.6.3.55 | 1 | tungstate-importing ATPase; tungstate transporter; WtpABC; TupABC; tungstate-specific ABC transporter | ATP + H2O + tungstate[side 1] = ADP + phosphate + tungstate[side 2] [RN:R10531] |
| ec:1.2.1.3 | 1 | aldehyde dehydrogenase (NAD+); CoA-independent aldehyde dehydrogenase; m-methylbenzaldehyde dehydrogenase; NAD-aldehyde dehydrogenase; NAD-dependent 4-hydroxynonenal dehydrogenase; NAD-dependent aldehyde dehydrogenase; NAD-linked aldehyde dehydrogenase; propionaldehyde dehydrogenase; aldehyde dehydrogenase (NAD) | an aldehyde + NAD+ + H2O = a carboxylate + NADH + H+ [RN:R00538] |
| ec:5.3.2.6 | 1 | 2-hydroxymuconate tautomerase; 4-oxalocrotonate tautomerase (misleading); 4-oxalocrotonate isomerase (misleading); cnbG (gene name); praC (gene name); xylH (gene name) | (2Z,4E)-2-hydroxyhexa-2,4-dienedioate = (3E)-2-oxohex-3-enedioate |
| ec:2.1.2.3 | 1 | phosphoribosylaminoimidazolecarboxamide formyltransferase; 5-amino-4-imidazolecarboxamide ribonucleotide transformylase; AICAR transformylase; 10-formyltetrahydrofolate:5'-phosphoribosyl-5-amino-4-imidazolecarboxamide formyltransferase; 5'-phosphoribosyl-5-amino-4-imidazolecarboxamide formyltransferase; 5-amino-1-ribosyl-4-imidazolecarboxamide 5'-phosphate transformylase; 5-amino-4-imidazolecarboxamide ribotide transformylase; AICAR formyltransferase; aminoimidazolecarboxamide ribonucleotide transformylase | 10-formyltetrahydrofolate + 5-amino-1-(5-phospho-D-ribosyl)imidazole-4-carboxamide = tetrahydrofolate + 5-formamido-1-(5-phospho-D-ribosyl)imidazole-4-carboxamide [RN:R04560] |
| ec:3.5.1.4 | 1 | amidase; acylamidase; acylase (misleading); amidohydrolase (ambiguous); deaminase (ambiguous); fatty acylamidase; N-acetylaminohydrolase (ambiguous) | a monocarboxylic acid amide + H2O = a monocarboxylate + NH3 [RN:R03909] |
| ec:1.14.13.82 | 1 | vanillate monooxygenase; 4-hydroxy-3-methoxybenzoate demethylase; vanillate demethylase | vanillate + O2 + NADH + H+ = 3,4-dihydroxybenzoate + NAD+ + H2O + formaldehyde [RN:R05274] |
| ec:6.2.1.1 | 1 | acetate---CoA ligase; acetyl-CoA synthetase; acetyl activating enzyme; acetate thiokinase; acyl-activating enzyme; acetyl coenzyme A synthetase; acetic thiokinase; acetyl CoA ligase; acetyl CoA synthase; acetyl-coenzyme A synthase; short chain fatty acyl-CoA synthetase; short-chain acyl-coenzyme A synthetase; ACS | ATP + acetate + CoA = AMP + diphosphate + acetyl-CoA [RN:R00235] |

  
**Over-represented Metabolite Summary**: Collection of the metabolites identified as substrates or products of the proteins representaed the "Over-represented Enzyme Summary" ranked by frequency of occurrence  

| ID | Structure | Name | Frequency | EC |
| --- | --- | --- | --- | --- |
| cpd:C00080 |  | H+; Hydron | 106 | ec:1.1.1.103  ec:3.5.99.2 ec:1.2.1.79 ec:2.8.1.6 ec:4.2.1.1 ec:1.1.1.93 ec:1.2.1.38 ec:1.1.1.30 ec:1.1.1.95 ec:1.2.1.20 ec:1.1.1.83 ec:1.2.1.3 ec:6.3.4.21 ec:1.2.1.16 ec:2.5.1.18 ec:1.18.1.3 ec:1.1.1.262 ec:4.2.1.104 ec:1.1.1.100 ec:1.18.6.1 ec:1.1.1.1 ec:4.1.1.73 ec:1.17.1.4 |
| cpd:C00003 |  | NAD+; NAD; Nicotinamide adenine dinucleotide; DPN; Diphosphopyridine nucleotide; Nadide | 75 | ec:1.1.1.103  ec:1.2.1.79 ec:3.6.1.22 ec:1.1.1.93 ec:1.1.1.30 ec:1.1.1.95 ec:1.1.1.83 ec:1.2.1.3 ec:1.2.1.20 ec:1.2.1.16 ec:1.18.1.3 ec:1.1.1.262 ec:1.1.1.1 ec:4.1.1.73 ec:1.17.1.4 |
| cpd:C00004 |  | NADH; DPNH; Reduced nicotinamide adenine dinucleotide | 73 | ec:1.1.1.103 ec:1.2.1.79 ec:1.2.1.16 ec:1.18.1.3 ec:1.1.1.262 ec:1.1.1.93 ec:1.1.1.30 ec:1.1.1.95 ec:1.2.1.20 ec:1.2.1.3 ec:1.1.1.83 ec:1.1.1.1 ec:1.17.1.4 ec:4.1.1.73 |
| cpd:C00001 |  | H2O; Water | 63 | ec:3.3.2.9  ec:6.3.5.2 ec:3.5.99.2 ec:1.2.1.79 ec:3.5.1.4 ec:1.3.99.26 ec:2.3.3.14 ec:2.5.1.6 ec:3.5.1.19 ec:3.6.1.22 ec:4.2.1.17 ec:1.3.3.3 ec:1.2.1.3 ec:2.1.2.3 ec:3.1.1.45 ec:3.5.4.10 ec:2.5.1.18 ec:1.2.1.16 ec:3.8.1.2 ec:1.17.1.4 ec:4.2.1.3 ec:1.3.99.31 ec:4.2.1.1 ec:4.99.1.5 ec:1.2.1.20 ec:4.2.1.9 ec:2.3.3.9 ec:3.2.1.21 ec:3.2.1.23 ec:6.3.4.21 ec:4.2.1.40 ec:3.6.3.55 ec:3.5.4.25 ec:3.1.1.17 ec:4.2.1.41 ec:1.18.1.3 ec:4.2.1.84 ec:1.3.99.28 ec:1.18.6.1 ec:1.1.1.1 ec:1.3.99.29 ec:3.5.5.7 |
| cpd:C00011 |  | CO2; Carbon dioxide | 52 | ec:4.2.1.1 ec:4.1.1.4 ec:4.1.1.36 ec:1.1.1.93 ec:4.1.1.23 ec:1.3.3.3 ec:1.1.1.83 ec:4.1.1.44 ec:6.3.2.5 ec:4.2.1.41 ec:1.18.1.3 ec:2.3.1.47 ec:1.1.1.262 ec:4.2.1.104 ec:4.1.1.73 |
| cpd:C03508 |  | L-2-Amino-3-oxobutanoic acid; L-2-Amino-3-oxobutanoate; L-2-Amino-acetoacetate; (S)-2-Amino-3-oxobutanoic acid | 50 | ec:1.1.1.103 ec:2.3.1.29 |
| cpd:C00010 |  | CoA; Coenzyme A; CoA-SH | 36 | ec:2.3.1.15 ec:2.3.1.9 ec:2.3.3.14 ec:2.3.1.47 ec:2.3.1.29 ec:6.2.1.3 ec:6.2.1.1 ec:2.3.3.9 |
| cpd:C00024 |  | Acetyl-CoA; Acetyl coenzyme A | 32 | ec:2.3.1.9 ec:2.3.3.14 ec:2.3.1.29 ec:6.2.1.1 ec:2.3.3.9 |
| cpd:C00164 |  | Acetoacetate; 3-Oxobutanoic acid; beta-Ketobutyric acid; Acetoacetic acid | 31 | ec:4.1.1.4 ec:1.1.1.30 |
| cpd:C00037 |  | Glycine; Aminoacetic acid; Gly | 26 | ec:6.3.4.13 ec:2.3.1.29 |
| cpd:C00002 |  | ATP; Adenosine 5'-triphosphate | 26 | ec:2.7.4.8 ec:6.3.5.2 ec:2.5.1.6 ec:2.7.1.39 ec:4.1.1.36 ec:6.2.1.3 ec:6.2.1.1 ec:2.7.6.1 ec:6.3.4.21 ec:2.7.7.6 ec:6.3.4.13 ec:6.3.2.5 ec:3.6.3.55 ec:2.7.1.71 ec:2.7.1.17 ec:1.18.6.1 ec:6.1.1.13 |
| cpd:C00188 |  | L-Threonine; 2-Amino-3-hydroxybutyric acid | 25 | ec:1.1.1.103 |
| cpd:C00022 |  | Pyruvate; Pyruvic acid; 2-Oxopropanoate; 2-Oxopropanoic acid; Pyroracemic acid | 24 | ec:2.8.1.1 ec:2.8.1.2 ec:1.1.1.93 ec:1.1.1.83 ec:4.1.1.73 |
| cpd:C00258 |  | D-Glycerate; Glycerate; (R)-Glycerate; Glyceric acid | 21 | ec:1.1.1.93 ec:1.2.1.3 ec:1.1.1.83 ec:4.1.1.73 |
| cpd:C03459 |  | 2-Hydroxy-3-oxosuccinate; Oxaloglycolate | 20 | ec:1.1.1.93 ec:1.1.1.83 ec:4.1.1.73 |
| cpd:C00552 |  | meso-Tartaric acid; meso-Tartrate | 20 | ec:1.1.1.93 ec:1.1.1.83 ec:4.1.1.73 |
| cpd:C00898 |  | (R,R)-Tartaric acid; (R,R)-Tartrate; L-Tartaric acid; Tartaric acid; Tartrate; 2,3-Dihydroxybutanedioic acid; (2R,3R)-Tartaric acid; (+)-Tartaric acid | 20 | ec:1.1.1.93 ec:1.1.1.83 ec:4.1.1.73 |
| cpd:C00497 |  | (R)-Malate; D-Malate; D-Malic acid | 20 | ec:1.1.1.93 ec:1.1.1.83 ec:4.1.1.73 |
| cpd:C00013 |  | Diphosphate; Diphosphoric acid; Pyrophosphate; Pyrophosphoric acid; PPi | 18 | ec:2.7.7.7 ec:6.3.4.21 ec:2.7.7.6 ec:6.3.5.2 ec:6.3.2.5 ec:3.5.4.25 ec:2.5.1.6 ec:4.1.1.36 ec:6.2.1.3 ec:2.5.1.32 ec:6.2.1.1 ec:6.1.1.13 |
| cpd:C00014 |  | Ammonia; NH3 | 17 | ec:6.3.5.2 ec:3.5.99.2 ec:3.5.1.4 ec:3.5.1.19 ec:1.18.1.3 ec:4.2.1.104 ec:1.18.6.1 ec:3.5.5.7 |
| cpd:C00009 |  | Orthophosphate; Phosphate; Phosphoric acid; Orthophosphoric acid | 17 | ec:6.3.4.21 ec:6.3.4.13 ec:6.3.4.4 ec:3.6.3.55 ec:2.5.1.6 ec:1.2.1.38 ec:1.18.6.1 |
| cpd:C01089 |  | (R)-3-Hydroxybutanoate; (R)-3-Hydroxybutanoic acid; (R)-3-Hydroxybutyric acid; D-beta-Hydroxybutyric acid | 16 | ec:1.1.1.30 |
| cpd:C00207 |  | Acetone; Dimethyl ketone; 2-Propanone | 15 | ec:4.1.1.4 |
| cpd:C00020 |  | AMP; Adenosine 5'-monophosphate; Adenylic acid; Adenylate; 5'-AMP; 5'-Adenylic acid; 5'-Adenosine monophosphate; Adenosine 5'-phosphate | 15 | ec:6.3.5.2 ec:6.3.2.5 ec:3.6.1.22 ec:4.1.1.36 ec:6.2.1.3 ec:6.2.1.1 ec:6.1.1.13 ec:2.7.6.1 |
| cpd:C00006 |  | NADP+; NADP; Nicotinamide adenine dinucleotide phosphate; beta-Nicotinamide adenine dinucleotide phosphate; TPN; Triphosphopyridine nucleotide | 14 | ec:1.2.1.79 ec:1.2.1.16 ec:1.18.1.3 ec:1.2.1.38 ec:1.1.1.100 ec:1.2.1.20 ec:1.2.1.3 |
| cpd:C00005 |  | NADPH; TPNH; Reduced nicotinamide adenine dinucleotide phosphate | 14 | ec:1.2.1.79 ec:1.2.1.16 ec:1.18.1.3 ec:1.2.1.38 ec:1.1.1.100 ec:1.2.1.20 ec:1.2.1.3 |
| cpd:C01327 |  | Hydrochloric acid; HCl; Hydrogen chloride; Hydrochloride | 11 | ec:2.5.1.18 ec:1.18.1.3 ec:3.8.1.2 |
| cpd:C00008 |  | ADP; Adenosine 5'-diphosphate | 11 | ec:2.7.4.8 ec:6.3.4.21 ec:6.3.4.13 ec:2.7.1.71 ec:3.6.3.55 ec:2.7.1.17 ec:2.7.1.39 ec:1.18.6.1 |
| cpd:C14852 |  | Benzo[a]pyrene-7,8-diol; Benzo[a]pyrene-7,8-dihydrodiol | 10 | ec:3.3.2.9 ec:2.5.1.18 |
| cpd:C14840 |  | Bromobenzene-2,3-oxide; Bromobenzene-2,3-epoxide | 10 | ec:3.3.2.9 ec:2.5.1.18 |
| cpd:C14839 |  | Bromobenzene-3,4-oxide; Bromobenzene-3,4-epoxide | 10 | ec:3.3.2.9 ec:2.5.1.18 |
| cpd:C07645 |  | Aldophosphamide | 10 | ec:2.5.1.18 ec:1.1.1.1 |
| cpd:C14787 |  | (1S,2R)-Naphthalene 1,2-oxide; (1S,2R)-Naphthalene epoxide | 10 | ec:3.3.2.9 ec:2.5.1.18 |
| cpd:C14786 |  | (1R,2S)-Naphthalene 1,2-oxide; (1R,2S)-Naphthalene epoxide | 10 | ec:3.3.2.9 ec:2.5.1.18 |
| cpd:C14800 |  | 1-Nitronaphthalene-5,6-oxide | 10 | ec:3.3.2.9 ec:2.5.1.18 |
| cpd:C19586 |  | Aflatoxin B1-exo-8,9-epoxide; 2,3-Epoxyaflatoxin B1 | 10 | ec:3.3.2.9 ec:2.5.1.18 |
| cpd:C06790 |  | Trichloroethene; Trichloroethylene; TCE | 10 | ec:2.5.1.18 ec:1.18.1.3 |
| cpd:C14874 |  | Glutathione episulfonium ion | 9 | ec:2.5.1.18 |
| cpd:C14871 |  | S-(Formylmethyl)glutathione | 9 | ec:2.5.1.18 |
| cpd:C14870 |  | 2-Bromoacetaldehyde | 9 | ec:2.5.1.18 |
| cpd:C14868 |  | S-(1,2-Dichlorovinyl)glutathione; DCVG | 9 | ec:2.5.1.18 |
| cpd:C11278 |  | Aflatoxin B1exo-8,9-epoxide-GSH; 8,9-Dihydro-8-(S-glutathionyl)-9-hydroxyaflatoxin B1 | 9 | ec:2.5.1.18 |
| cpd:C14865 |  | 2-(S-Glutathionyl)acetyl chloride | 9 | ec:2.5.1.18 |
| cpd:C14864 |  | S-(2-Chloroacetyl)glutathione | 9 | ec:2.5.1.18 |
| cpd:C14863 |  | 2-(S-Glutathionyl)acetyl glutathione | 9 | ec:2.5.1.18 |
| cpd:C14861 |  | S-(2,2-Dichloro-1-hydroxy)ethyl glutathione | 9 | ec:2.5.1.18 |
| cpd:C01322 |  | RX; Organic halide | 9 | ec:2.5.1.18 |
| cpd:C14859 |  | Chloroacetyl chloride | 9 | ec:2.5.1.18 |
| cpd:C14858 |  | 2,2-Dichloroacetaldehyde | 9 | ec:2.5.1.18 |
| cpd:C14857 |  | 1,1-Dichloroethylene epoxide; 2,2-Dichlorooxirane | 9 | ec:2.5.1.18 |
| cpd:C14856 |  | 7,8-Dihydro-7-hydroxy-8-S-glutathionyl-benzo[a]pyrene | 9 | ec:2.5.1.18 |
| cpd:C14855 |  | 4,5-Dihydro-4-hydroxy-5-S-glutathionyl-benzo[a]pyrene | 9 | ec:2.5.1.18 |
| cpd:C14851 |  | Benzo[a]pyrene-4,5-oxide; Benzo[a]pyrene-4,5-epoxide | 9 | ec:2.5.1.18 |
| cpd:C14848 |  | 2,3-Dihydro-2-S-glutathionyl-3-hydroxy bromobenzene | 9 | ec:2.5.1.18 |
| cpd:C14847 |  | 3,4-Dihydro-3-hydroxy-4-S-glutathionyl bromobenzene | 9 | ec:2.5.1.18 |
| cpd:C14793 |  | (1R)-Glutathionyl-(2R)-hydroxy-1,2-dihydronaphthalene | 9 | ec:2.5.1.18 |
| cpd:C14792 |  | (1S)-Hydroxy-(2S)-glutathionyl-1,2-dihydronaphthalene | 9 | ec:2.5.1.18 |
| cpd:C14791 |  | (1R)-Hydroxy-(2R)-glutathionyl-1,2-dihydronaphthalene | 9 | ec:2.5.1.18 |
| cpd:C02320 |  | R-S-Glutathione | 9 | ec:2.5.1.18 |
| cpd:C11583 |  | 4-Glutathionyl cyclophosphamide | 9 | ec:2.5.1.18 |
| cpd:C14806 |  | 1-Nitro-5-glutathionyl-6-hydroxy-5,6-dihydronaphthalene | 9 | ec:2.5.1.18 |
| cpd:C14805 |  | 1-Nitro-5-hydroxy-6-glutathionyl-5,6-dihydronaphthalene | 9 | ec:2.5.1.18 |
| cpd:C14804 |  | 1-Nitro-7-glutathionyl-8-hydroxy-7,8-dihydronaphthalene | 9 | ec:2.5.1.18 |
| cpd:C14803 |  | 1-Nitro-7-hydroxy-8-glutathionyl-7,8-dihydronaphthalene | 9 | ec:2.5.1.18 |
| cpd:C14802 |  | 1-Nitronaphthalene-7,8-oxide | 9 | ec:2.5.1.18 |
| cpd:C13645 |  | Hydrobromic acid; HBr | 9 | ec:2.5.1.18 |
| cpd:C00462 |  | Halide; Hydrogen halide; HX; Halo acid | 9 | ec:2.5.1.18 |
| cpd:C11088 |  | 1,2-Dibromoethane; Ethylene dibromide | 9 | ec:2.5.1.18 |
| cpd:C00051 |  | Glutathione; 5-L-Glutamyl-L-cysteinylglycine; N-(N-gamma-L-Glutamyl-L-cysteinyl)glycine; gamma-L-Glutamyl-L-cysteinyl-glycine; GSH; Reduced glutathione | 9 | ec:2.5.1.18 |
| cpd:C04133 |  | N-Acetyl-L-glutamate 5-phosphate; N-Acetyl-L-glutamyl 5-phosphate | 8 | ec:1.2.1.38 |
| cpd:C01250 |  | N-Acetyl-L-glutamate 5-semialdehyde; 2-Acetamido-5-oxopentanoate | 8 | ec:1.2.1.38 |
| cpd:C00139 |  | Oxidized ferredoxin | 7 | ec:1.18.6.1 |
| cpd:C00138 |  | Reduced ferredoxin | 7 | ec:1.18.6.1 |
| cpd:C00144 |  | GMP; Guanosine 5'-phosphate; Guanosine monophosphate; Guanosine 5'-monophosphate; Guanylic acid | 6 | ec:2.7.4.8 ec:6.3.5.2 |
| cpd:C00007 |  | Oxygen; O2 | 6 | ec:1.3.99.26 ec:1.3.99.31 ec:1.18.1.3 ec:1.13.11.54 ec:1.3.3.3 ec:1.13.11.53 ec:1.3.99.28 ec:1.3.99.29 |
| cpd:C00288 |  | HCO3-; Bicarbonate; Hydrogencarbonate; Acid carbonate | 5 | ec:4.2.1.1 ec:4.2.1.104 |
| cpd:C00655 |  | Xanthosine 5'-phosphate; Xanthylic acid; XMP; (9-D-Ribosylxanthine)-5'-phosphate | 5 | ec:6.3.5.2 |
| cpd:C16619 |  | 6-Thioguanosine monophosphate | 5 | ec:6.3.5.2 |
| cpd:C16618 |  | 6-Thioxanthine 5'-monophosphate | 5 | ec:6.3.5.2 |
| cpd:C00197 |  | 3-Phospho-D-glycerate; D-Glycerate 3-phosphate; 3-Phospho-(R)-glycerate; 3-Phosphoglycerate | 5 | ec:5.4.2.12 ec:1.1.1.95 |
| cpd:C00064 |  | L-Glutamine; L-2-Aminoglutaramic acid | 5 | ec:6.3.5.2 |
| cpd:C00025 |  | L-Glutamate; L-Glutamic acid; L-Glutaminic acid; Glutamate | 5 | ec:6.3.5.2 |
| cpd:C00697 |  | Nitrogen; N2 | 4 | ec:1.18.6.1 |
| cpd:C00282 |  | Hydrogen; H2 | 4 | ec:1.18.6.1 |
| cpd:C00631 |  | 2-Phospho-D-glycerate; D-Glycerate 2-phosphate; 2-Phospho-(R)-glycerate | 4 | ec:5.4.2.12 |
| cpd:C06547 |  | Ethylene | 4 | ec:1.18.6.1 |
| cpd:C16468 |  | (2E)-5-Methylhexa-2,4-dienoyl-CoA | 4 | ec:4.2.1.17 |
| cpd:C01548 |  | Acetylene; Ethyne | 4 | ec:1.18.6.1 |
| cpd:C00058 |  | Formate; Methanoic acid; Formic acid | 4 | ec:3.5.4.25 ec:1.18.1.3 ec:1.13.11.54 ec:1.13.11.53 |
| cpd:C00055 |  | CMP; Cytidine-5'-monophosphate; Cytidylic acid | 4 | ec:6.3.2.5 ec:4.1.1.36 ec:2.7.8.8 |
| cpd:C00044 |  | GTP; Guanosine 5'-triphosphate | 4 | ec:2.7.7.6 ec:6.3.4.4 ec:3.5.4.25 |
| cpd:C01417 |  | Cyanate; Cyanic acid | 3 | ec:4.2.1.104 |
| cpd:C00726 |  | Nitrile; R-CN | 3 | ec:4.2.1.84 ec:4.99.1.5 |
| cpd:C01352 |  | FADH2 | 3 | ec:1.3.8.7 |
| cpd:C00311 |  | Isocitrate; Isocitric acid; 1-Hydroxytricarballylic acid; 1-Hydroxypropane-1,2,3-tricarboxylic acid | 3 | ec:4.2.1.3 ec:4.1.3.1 |
| cpd:C03069 |  | 3-Methylcrotonyl-CoA; 3-Methylbut-2-enoyl-CoA; 3-Methylcrotonoyl-CoA; Dimethylacryloyl-CoA | 3 | ec:1.3.8.7 ec:4.2.1.17 |
| cpd:C03460 |  | 2-Methylprop-2-enoyl-CoA; Methacrylyl-CoA; Methylacrylyl-CoA | 3 | ec:1.3.8.7 ec:4.2.1.17 |
| cpd:C00269 |  | CDP-diacylglycerol; CDP-1,2-diacylglycerol; 1,2-Diacyl-sn-glycero-3-cytidine-5'-diphosphate | 3 | ec:2.7.8.8 |
| cpd:C00262 |  | Hypoxanthine; Purine-6-ol | 3 | ec:1.17.1.4 |
| cpd:C09815 |  | Benzamide | 3 | ec:3.5.1.4 ec:4.2.1.84 |
| cpd:C02737 |  | Phosphatidylserine; Phosphatidyl-L-serine; 1,2-Diacyl-sn-glycerol 3-phospho-L-serine; 3-O-sn-Phosphatidyl-L-serine; O3-Phosphatidyl-L-serine; L-1-Phosphatidylserine | 3 | ec:2.7.8.8 |
| cpd:C01659 |  | Acrylamide; 2-Propenamide | 3 | ec:3.5.1.4 ec:4.2.1.84 |
| cpd:C00617 |  | UDP-D-galacturonate; UDPgalacturonate | 3 | ec:5.1.3.6 |
| cpd:C02693 |  | (Indol-3-yl)acetamide; Indole-3-acetamide | 3 | ec:3.5.1.4 ec:4.2.1.84 |
| cpd:C00167 |  | UDP-glucuronate; UDPglucuronate; UDP-D-glucuronate; UDP-alpha-D-glucuronate | 3 | ec:5.1.3.6 |
| cpd:C01998 |  | Acrylonitrile; Propenenitrile; Vinyl cyanide | 3 | ec:4.2.1.84 ec:3.5.5.7 |
| cpd:C00154 |  | Palmitoyl-CoA; Hexadecanoyl-CoA | 3 | ec:1.3.8.7 ec:6.2.1.3 |
| cpd:C03345 |  | 2-Methylbut-2-enoyl-CoA; trans-2-Methylbut-2-enoyl-CoA; Tiglyl-CoA; (E)-2-Methylcrotonoyl-CoA; Methylcrotonoyl-CoA; Methylcrotonyl-CoA; Tigloyl-CoA; 2-Methylcrotanoyl-CoA | 3 | ec:1.3.8.7 ec:4.2.1.17 |
| cpd:C01185 |  | Nicotinate D-ribonucleotide; beta-Nicotinate D-ribonucleotide; Nicotinate ribonucleotide; Nicotinic acid ribonucleotide | 3 | ec:6.3.4.21 ec:3.6.1.22 |
| cpd:C00894 |  | Propenoyl-CoA; Acryloyl-CoA; Acrylyl-CoA | 3 | ec:1.3.8.7 ec:4.2.1.17 |
| cpd:C01563 |  | Carbamate; Carbamic acid; Aminoformic acid | 3 | ec:4.2.1.104 |
| cpd:C00877 |  | Crotonoyl-CoA; Crotonyl-CoA; 2-Butenoyl-CoA; trans-But-2-enoyl-CoA; But-2-enoyl-CoA; (E)-But-2-enoyl-CoA | 3 | ec:1.3.8.7 ec:4.2.1.17 |
| cpd:C00119 |  | 5-Phospho-alpha-D-ribose 1-diphosphate; 5-Phosphoribosyl diphosphate; 5-Phosphoribosyl 1-pyrophosphate; PRPP | 3 | ec:6.3.4.21 ec:2.7.6.1 |
| cpd:C00073 |  | L-Methionine; Methionine; L-2-Amino-4methylthiobutyric acid | 3 | ec:2.8.1.6 ec:2.5.1.6 ec:2.1.1.14 |
| cpd:C00065 |  | L-Serine; L-2-Amino-3-hydroxypropionic acid; L-3-Hydroxy-alanine; Serine | 3 | ec:2.7.8.8 |
| cpd:C00048 |  | Glyoxylate; Glyoxalate; Glyoxylic acid | 3 | ec:4.1.3.1 ec:1.18.1.3 ec:2.3.3.9 |
| cpd:C00035 |  | GDP; Guanosine 5'-diphosphate; Guanosine diphosphate | 3 | ec:2.7.4.8 ec:2.4.1.83 ec:6.3.4.4 |
| cpd:C03620 |  | Monocarboxylic acid amide | 3 | ec:3.5.1.4 ec:4.2.1.84 |
| cpd:C03221 |  | 2-trans-Dodecenoyl-CoA; (2E)-Dodec-2-enoyl-CoA; (2E)-Dodecenoyl-CoA | 3 | ec:1.3.8.7 ec:4.2.1.17 |
| cpd:C00385 |  | Xanthine | 3 | ec:1.17.1.4 |
| cpd:C00019 |  | S-Adenosyl-L-methionine; S-Adenosylmethionine; AdoMet; SAM | 3 | ec:2.8.1.6 ec:2.6.1.62 ec:2.5.1.6 |
| cpd:C00016 |  | FAD; Flavin adenine dinucleotide | 3 | ec:1.3.8.7 |
| cpd:C00366 |  | Urate; Uric acid | 3 | ec:1.17.1.4 |
| cpd:C05276 |  | trans-Oct-2-enoyl-CoA; (2E)-Octenoyl-CoA | 3 | ec:1.3.8.7 ec:4.2.1.17 |
| cpd:C05275 |  | trans-Dec-2-enoyl-CoA; (2E)-Decenoyl-CoA | 3 | ec:1.3.8.7 ec:4.2.1.17 |
| cpd:C05273 |  | trans-Tetradec-2-enoyl-CoA; (2E)-Tetradecenoyl-CoA | 3 | ec:1.3.8.7 ec:4.2.1.17 |
| cpd:C05272 |  | trans-Hexadec-2-enoyl-CoA; trans-2-Hexadecenoyl-CoA; (2E)-Hexadecenoyl-CoA | 3 | ec:1.3.8.7 ec:4.2.1.17 |
| cpd:C05271 |  | trans-Hex-2-enoyl-CoA; (2E)-Hexenoyl-CoA | 3 | ec:1.3.8.7 ec:4.2.1.17 |
| cpd:C02505 |  | 2-Phenylacetamide; alpha-Phenylacetamide | 3 | ec:3.5.1.4 ec:4.2.1.84 |
| cpd:C05258 |  | (S)-3-Hydroxyhexadecanoyl-CoA | 2 | ec:4.2.1.17 |
| cpd:C04178 |  | Bromoxynil; 3,5-Dibromo-4-hydroxybenzonitrile | 2 | ec:4.2.1.84 |
| cpd:C05998 |  | 3-Hydroxyisovaleryl-CoA; 3-Hydroxyisovaleryl coenzyme A | 2 | ec:4.2.1.17 |
| cpd:C14145 |  | (3S)-3-Hydroxyadipyl-CoA | 2 | ec:4.2.1.17 |
| cpd:C14144 |  | 5-Carboxy-2-pentenoyl-CoA; 2,3-Dehydroadipyl-CoA | 2 | ec:4.2.1.17 |
| cpd:C00718 |  | Amylose; Amylose chain; (1,4-alpha-D-Glucosyl)n; (1,4-alpha-D-Glucosyl)n+1; (1,4-alpha-D-Glucosyl)n-1; 4-{(1,4)-alpha-D-Glucosyl}(n-1)-D-glucose; 1,4-alpha-D-Glucan | 2 | ec:2.4.1.18 ec:2.4.1.11 |
| cpd:C00679 |  | 5-Dehydro-4-deoxy-D-glucarate | 2 | ec:4.2.1.40 ec:4.2.1.41 |
| cpd:C01353 |  | Carbonic acid; Dihydrogen carbonate; H2CO3 | 2 | ec:4.2.1.1 |
| cpd:C16246 |  | 3,5-Dibromo-4-hydroxybenzamide | 2 | ec:4.2.1.84 |
| cpd:C00267 |  | alpha-D-Glucose | 2 | ec:3.2.1.23 ec:3.2.1.21 |
| cpd:C00653 |  | Poly(ribitol phosphate); Poly-1,5-ribitol phosphate; (Ribitol phosphate)n; (Ribitol phosphate)n+1 | 2 | ec:6.1.1.13 |
| cpd:C00253 |  | Nicotinate; Nicotinic acid; Niacin; 3-Pyridinecarboxylic acid | 2 | ec:6.3.4.21 ec:3.5.1.19 |
| cpd:C00249 |  | Hexadecanoic acid; Hexadecanoate; Hexadecylic acid; Palmitic acid; Palmitate; Cetylic acid | 2 | ec:6.2.1.3 |
| cpd:C09814 |  | Benzonitrile; Phenyl cyanide; Cyanobenzene | 2 | ec:4.2.1.84 |
| cpd:C06613 |  | trans-3-Chloroallyl aldehyde; trans-3-Chloro-2-propenal | 2 | ec:1.2.1.3 ec:1.1.1.1 |
| cpd:C01304 |  | 2,5-Diamino-6-(5-phospho-D-ribosylamino)pyrimidin-4(3H)-one; 2,5-Diamino-6-(1-D-ribosylamino)pyrimidin-4(3H)-one 5'-phosphate | 2 | ec:3.5.4.25 |
| cpd:C00229 |  | Acyl-carrier protein; ACP; [Acyl-carrier protein]; Holo-[acyl-carrier protein] | 2 | ec:2.3.1.15 ec:2.3.1.47 |
| cpd:C00221 |  | beta-D-Glucose | 2 | ec:3.2.1.23 ec:3.2.1.21 |
| cpd:C00577 |  | D-Glyceraldehyde | 2 | ec:1.2.1.3 ec:4.1.2.13 |
| cpd:C05116 |  | 3-Hydroxybutanoyl-CoA; 3-Hydroxybutyryl-CoA | 2 | ec:4.2.1.17 |
| cpd:C00954 |  | Indole-3-acetate; Indole-3-acetic acid; (Indol-3-yl)acetate; Indoleacetate; Indoleacetic acid; IAA | 2 | ec:3.5.1.4 ec:1.2.1.3 |
| cpd:C00158 |  | Citrate; Citric acid; 2-Hydroxy-1,2,3-propanetricarboxylic acid; 2-Hydroxytricarballylic acid | 2 | ec:4.2.1.3 |
| cpd:C05460 |  | 3alpha,7alpha,12alpha-Trihydroxy-5beta-cholest-24-enoyl-CoA | 2 |  |
| cpd:C16470 |  | 5-Methylhex-4-enoyl-CoA | 2 |  |
| cpd:C16074 |  | Phenylacetonitrile; Benzyl cyanide | 2 | ec:4.2.1.84 |
| cpd:C05447 |  | 3alpha,7alpha-Dihydroxy-5beta-cholest-24-enoyl-CoA | 2 |  |
| cpd:C05445 |  | 3alpha,7alpha-Dihydroxy-5beta-cholestan-26-al | 2 | ec:1.2.1.3 ec:1.1.1.1 |
| cpd:C04405 |  | (2S,3S)-3-Hydroxy-2-methylbutanoyl-CoA; (S)-3-Hydroxy-2-methylbutyryl-CoA | 2 | ec:4.2.1.17 |
| cpd:C00136 |  | Butanoyl-CoA; Butyryl-CoA | 2 | ec:1.3.8.7 ec:2.3.1.9 |
| cpd:C16469 |  | 3-Hydroxy-5-methylhex-4-enoyl-CoA | 2 | ec:4.2.1.17 |
| cpd:C00133 |  | D-Alanine; D-2-Aminopropionic acid; D-Ala | 2 | ec:6.1.1.13 |
| cpd:C04760 |  | 3alpha,7alpha,12alpha-Trihydroxy-5beta-cholestanoyl-CoA | 2 |  |
| cpd:C00130 |  | IMP; Inosinic acid; Inosine monophosphate; Inosine 5'-monophosphate; Inosine 5'-phosphate; 5'-Inosinate; 5'-Inosinic acid; 5'-Inosine monophosphate; 5'-IMP | 2 | ec:6.3.4.4 ec:3.5.4.10 ec:2.1.2.3 |
| cpd:C11947 |  | 3-Hydroxy-2,6-dimethyl-5-methylene-heptanoyl-CoA | 2 | ec:4.2.1.17 |
| cpd:C11946 |  | cis-2-Methyl-5-isopropylhexa-2,5-dienoyl-CoA | 2 | ec:4.2.1.17 |
| cpd:C11945 |  | trans-2-Methyl-5-isopropylhexa-2,5-dienoyl-CoA | 2 | ec:4.2.1.17 |
| cpd:C11145 |  | Methanesulfonic acid; Methanesulfonate | 2 |  |
| cpd:C00084 |  | Acetaldehyde; Ethanal | 2 | ec:1.2.1.3 ec:1.1.1.1 |
| cpd:C11142 |  | Dimethyl sulfone; Sulfonylbismethane | 2 |  |
| cpd:C00511 |  | Acrylic acid; Propenoate; Acrylate; 2-Propenoic acid; Vinylformic acid | 2 | ec:3.5.1.4 ec:3.5.5.7 |
| cpd:C00117 |  | D-Ribose 5-phosphate; Ribose 5-phosphate | 2 | ec:2.7.6.1 |
| cpd:C05421 |  | 15-cis-Phytoene; Phytoene | 2 | ec:1.3.99.26 ec:1.3.99.31 ec:2.5.1.32 ec:1.3.99.28 ec:1.3.99.29 |
| cpd:C00071 |  | Aldehyde; RCHO | 2 | ec:1.2.1.3 ec:1.1.1.1 |
| cpd:C01144 |  | (S)-3-Hydroxybutanoyl-CoA; (S)-3-Hydroxybutyryl-CoA | 2 | ec:4.2.1.17 |
| cpd:C00105 |  | UMP; Uridylic acid; Uridine monophosphate; Uridine 5'-monophosphate; 5'Uridylic acid | 2 | ec:4.1.1.23 |
| cpd:C00100 |  | Propanoyl-CoA; Propionyl-CoA; Propionyl coenzyme A | 2 | ec:1.3.8.7 ec:6.2.1.1 |
| cpd:C00067 |  | Formaldehyde; Methanal; Oxomethane; Oxomethylene; Methylene oxide; Formalin | 2 |  |
| cpd:C00063 |  | CTP; Cytidine 5'-triphosphate; Cytidine triphosphate | 2 | ec:2.7.7.6 ec:6.3.2.5 ec:4.1.1.36 |
| cpd:C00061 |  | FMN; Riboflavin-5-phosphate; Flavin mononucleotide | 2 |  |
| cpd:C16393 |  | 2-Hydroxylamino-4,6-dinitrotoluene | 2 |  |
| cpd:C16392 |  | 4-Hydroxylamino-2,6-dinitrotoluene | 2 |  |
| cpd:C16391 |  | Trinitrotoluene; 2,4,6-Trinitrotoluene | 2 |  |
| cpd:C00857 |  | Deamino-NAD+; Deamido-NAD+; Deamido-NAD | 2 | ec:3.6.1.22 |
| cpd:C00455 |  | Nicotinamide D-ribonucleotide; NMN; Nicotinamide mononucleotide; Nicotinamide ribonucleotide; Nicotinamide nucleotide; beta-Nicotinamide D-ribonucleotide; beta-Nicotinamide ribonucleotide; beta-Nicotinamide mononucleotide | 2 | ec:3.6.1.22 |
| cpd:C01092 |  | 8-Amino-7-oxononanoate; 8-Amino-7-oxononanoic acid | 2 | ec:2.6.1.62 ec:2.3.1.47 |
| cpd:C00042 |  | Succinate; Succinic acid; Butanedionic acid; Ethylenesuccinic acid | 2 | ec:1.2.1.79 ec:4.1.3.1 ec:1.2.1.16 ec:1.2.1.20 |
| cpd:C00433 |  | 2,5-Dioxopentanoate; 2-Oxoglutarate semialdehyde | 2 | ec:4.2.1.41 ec:1.2.1.3 |
| cpd:C00033 |  | Acetate; Acetic acid; Ethanoic acid | 2 | ec:6.2.1.1 ec:1.2.1.3 |
| cpd:C00031 |  | D-Glucose; Grape sugar; Dextrose; Glucose; D-Glucopyranose | 2 | ec:3.2.1.23 ec:3.2.1.21 |
| cpd:C00030 |  | Reduced acceptor; AH2; Hydrogen-donor; Donor | 2 | ec:1.3.8.7 ec:1.3.99.26 ec:1.3.99.31 ec:1.3.99.28 ec:1.3.99.29 |
| cpd:C04260 |  | O-D-Alanyl-poly(ribitol phosphate) | 2 | ec:6.1.1.13 |
| cpd:C01103 |  | Orotidine 5'-phosphate; Orotidylic acid | 2 | ec:4.1.1.23 |
| cpd:C00028 |  | Acceptor; Hydrogen-acceptor; A; Oxidized donor | 2 | ec:1.3.8.7 ec:1.3.99.26 ec:1.3.99.31 ec:1.3.99.28 ec:1.3.99.29 |
| cpd:C05335 |  | L-Selenomethionine | 2 | ec:2.5.1.6 ec:2.1.1.14 |
| cpd:C02938 |  | 3-Indoleacetonitrile; Indol-3-ylacetonitrile; Indole-3-acetonitrile; (Indol-3-yl)acetonitrile | 2 | ec:4.2.1.84 |
| cpd:C00026 |  | 2-Oxoglutarate; Oxoglutaric acid; 2-Ketoglutaric acid; alpha-Ketoglutaric acid | 2 | ec:2.3.3.14 ec:1.2.1.3 |
| cpd:C00818 |  | D-Glucarate; D-Glucaric acid; L-Gularic acid; D-Saccharic acid; D-Glucosaccharic acid; Glucaric acid; Glucarate | 2 | ec:4.2.1.40 ec:1.2.1.3 |
| cpd:C00417 |  | cis-Aconitate; cis-Aconitic acid | 2 | ec:4.2.1.3 |
| cpd:C04644 |  | 3alpha,7alpha-Dihydroxy-5beta-cholestanoyl-CoA | 2 |  |
| cpd:C16348 |  | cis-3-Chloroallyl aldehyde; cis-3-Chloro-2-propenal | 2 | ec:1.2.1.3 ec:1.1.1.1 |
| cpd:C06000 |  | (S)-3-Hydroxyisobutyryl-CoA | 2 | ec:4.2.1.17 |
| cpd:C01847 |  | Reduced FMN; FMNH2 | 2 |  |
| cpd:C06755 |  | Chloroacetic acid; Chloroethanoic acid | 2 | ec:3.8.1.2 ec:1.2.1.3 |
| cpd:C05668 |  | 3-Hydroxypropionyl-CoA; 3-Hydroxypropionyl coenzyme A; 3-Hydroxypropanoyl-CoA; 3-Hydroxypropanoyl coenzymeA; beta-Hydroxypropionyl-CoA | 2 | ec:4.2.1.17 |
| cpd:C05268 |  | (S)-Hydroxyhexanoyl-CoA; (S)-3-Hydroxyhexanoyl-CoA | 2 | ec:4.2.1.17 |
| cpd:C05266 |  | (S)-3-Hydroxyoctanoyl-CoA; (S)-3-Hydroxycapryloyl-CoA; (S)-Hydroxyoctanoyl-CoA | 2 | ec:4.2.1.17 |
| cpd:C05264 |  | (S)-Hydroxydecanoyl-CoA; (S)-3-Hydroxydecanoyl-CoA | 2 | ec:4.2.1.17 |
| cpd:C05262 |  | (S)-3-Hydroxydodecanoyl-CoA | 2 | ec:4.2.1.17 |
| cpd:C05260 |  | (S)-3-Hydroxytetradecanoyl-CoA | 2 | ec:4.2.1.17 |
| cpd:C00340 |  | Reduced rubredoxin | 1 | ec:1.18.1.3 |
| cpd:C04570 |  | Reduced electron-transferring flavoprotein; Reduced electron-transfer flavoprotein | 1 | ec:1.3.8.7 |
| cpd:C06727 |  | cis-1,2-Dihydro-3-ethylcatechol; cis-2,3-Dihydroxy-2,3-dihydroethylbenzene; cis-3-Ethyl-cyclohexa-3,5-diene-1,2-diol | 1 | ec:1.18.1.3 |
| cpd:C12838 |  | trans-4-Carboxymethylenebut-2-en-4-olide | 1 | ec:3.1.1.45 |
| cpd:C12835 |  | 2,5-Dichloro-4-oxohex-2-enedioate | 1 | ec:3.1.1.45 |
| cpd:C03492 |  | D-4'-Phosphopantothenate; (R)-4'-Phosphopantothenate | 1 | ec:6.3.2.5 ec:4.1.1.36 |
| cpd:C12834 |  | 2,5-Dichloro-carboxymethylenebut-2-en-4-olide | 1 | ec:3.1.1.45 |
| cpd:C06329 |  | 2-Chloromaleylacetate | 1 | ec:3.1.1.45 |
| cpd:C20267 |  | 4-Amino-5-aminomethyl-2-methylpyrimidine | 1 | ec:3.5.99.2 |
| cpd:C03090 |  | 5-Phosphoribosylamine; 5-Phospho-beta-D-ribosylamine; 5-Phospho-D-ribosylamine; 5-Phosphoribosyl-1-amine | 1 | ec:6.3.4.13 |
| cpd:C00334 |  | 4-Aminobutanoate; 4-Aminobutanoic acid; 4-Aminobutyrate; 4-Aminobutyric acid; gamma-Aminobutyric acid; GABA | 1 | ec:1.2.1.3 |
| cpd:C00332 |  | Acetoacetyl-CoA; Acetoacetyl coenzyme A; 3-Acetoacetyl-CoA | 1 | ec:2.3.1.9 |
| cpd:C03921 |  | 2-Dehydro-3-deoxy-D-glucarate | 1 | ec:4.2.1.40 |
| cpd:C15980 |  | (S)-2-Methylbutanoyl-CoA | 1 | ec:1.3.8.7 |
| cpd:C01407 |  | Benzene | 1 | ec:1.18.1.3 |
| cpd:C05635 |  | 5-Hydroxyindoleacetate | 1 | ec:1.2.1.3 |
| cpd:C05634 |  | 5-Hydroxyindoleacetaldehyde | 1 | ec:1.2.1.3 |
| cpd:C05993 |  | Acetyl adenylate; 5'-Acetylphosphoadenosine | 1 | ec:6.2.1.1 |
| cpd:C00681 |  | 1-Acyl-sn-glycerol 3-phosphate; 2-Lysophosphatidate; Lysophosphatidate; Lysophosphatidic acid | 1 | ec:2.3.1.15 |
| cpd:C00320 |  | Thiosulfate; Hyposulfite | 1 | ec:2.8.1.1 ec:2.8.1.2 |
| cpd:C00286 |  | dGTP; 2'-Deoxyguanosine 5'-triphosphate; Deoxyguanosine 5'-triphosphate; Deoxyguanosine triphosphate | 1 | ec:2.7.7.7 |
| cpd:C04554 |  | 3alpha,7alpha-Dihydroxy-5beta-cholestanate; 3alpha,7alpha-Dihydroxy-5beta-cholestanoate | 1 | ec:1.2.1.3 |
| cpd:C02835 |  | Imidazole-4-acetate; Imidazoleacetic acid; 4-Imidazoleacetate | 1 | ec:1.2.1.3 |
| cpd:C05198 |  | 5'-Deoxyadenosine | 1 | ec:2.8.1.6 |
| cpd:C19845 |  | Pimeloyl-[acyl-carrier protein]; Pimeloyl-[acp]; Pimelyl-[acyl-carrier protein]; Pimelyl-[acp]; 7-Hydroxy-7-oxoheptanoyl-[acyl-carrier protein] | 1 | ec:2.3.1.47 |
| cpd:C01755 |  | Thiocyanate; Thiocyanic acid | 1 | ec:2.8.1.1 ec:2.8.1.2 |
| cpd:C03078 |  | 4-Guanidinobutanamide | 1 | ec:3.5.1.4 |
| cpd:C05629 |  | Phenylpropanoate; 3-Phenyl-propionic acid; 3-Phenylpropanoic acid; 3-Phenylpropionic acid | 1 | ec:1.18.1.3 |
| cpd:C05985 |  | 2-Propynal; 2-Propyn-1-al; Propiolaldehyde | 1 | ec:1.2.1.3 |
| cpd:C14099 |  | 2-Naphthaldehyde; 2-Naphthalenecarboxaldehyde | 1 | ec:1.1.1.1 |
| cpd:C05983 |  | Propionyladenylate; Propionyl-adenosine monophosphate | 1 | ec:6.2.1.1 |
| cpd:C00671 |  | (S)-3-Methyl-2-oxopentanoic acid; (S)-3-Methyl-2-oxopentanoate; (3S)-3-Methyl-2-oxopentanoic acid; (3S)-3-Methyl-2-oxopentanoate | 1 | ec:4.2.1.9 |
| cpd:C00279 |  | D-Erythrose 4-phosphate | 1 | ec:4.1.2.13 |
| cpd:C15606 |  | 1,2-Dihydroxy-5-(methylthio)pent-1-en-3-one | 1 | ec:1.13.11.54 ec:1.13.11.53 |
| cpd:C00310 |  | D-Xylulose; D-threo-Pentulose; D-Lyxulose | 1 | ec:2.7.1.17 |
| cpd:C14090 |  | 1-Naphthaldehyde; 1-Formylnaphthalene | 1 | ec:1.1.1.1 |
| cpd:C05223 |  | Dodecanoyl-[acyl-carrier protein]; Dodecanoyl-[acp]; Lauroyl-[acyl-carrier protein] | 1 |  |
| cpd:C03862 |  | Dolichyl phosphate D-mannose; Dolichyl D-mannosyl phosphate | 1 | ec:2.4.1.83 |
| cpd:C04144 |  | Tetrahydropteroyltri-L-glutamate | 1 | ec:2.1.1.14 |
| cpd:C07335 |  | 2-Amino-3-oxo-4-phosphonooxybutyrate; L-2-Amino-3-oxo-4-phosphonooxybutyrate; (2S)-2-Amino-3-oxo-4-phosphonooxybutanoate | 1 | ec:1.1.1.262 |
| cpd:C14089 |  | 1-Hydroxymethylnaphthalene; 1-Naphthalenemethanol | 1 | ec:1.1.1.1 |
| cpd:C05577 |  | 3,4-Dihydroxymandelaldehyde; 3,4-Dihydroxyphenylglycolaldehyde | 1 | ec:1.1.1.1 |
| cpd:C05576 |  | 3,4-Dihydroxyphenylethyleneglycol | 1 | ec:1.1.1.1 |
| cpd:C00263 |  | L-Homoserine; 2-Amino-4-hydroxybutyric acid | 1 | ec:2.7.1.39 |
| cpd:C16596 |  | 5-Phenyl-1,3-oxazinane-2,4-dione | 1 | ec:1.1.1.1 |
| cpd:C16595 |  | 4-Hydroxy-5-phenyltetrahydro-1,3-oxazin-2-one | 1 | ec:1.1.1.1 |
| cpd:C03453 |  | gamma-Oxalocrotonate; (Z)-5-Oxohex-2-enedioate; 4-Oxalocrotonate | 1 | ec:5.3.2.6 |
| cpd:C04091 |  | cis-1,2-Dihydrobenzene-1,2-diol; cis-Benzeneglycol; cis-Cyclohexa-3,5-diene-1,2-diol | 1 | ec:1.18.1.3 |
| cpd:C01335 |  | ROH | 1 | ec:3.2.1.21 |
| cpd:C00257 |  | D-Gluconic acid; D-Gluconate; D-gluco-Hexonic acid | 1 | ec:3.1.1.17 |
| cpd:C16587 |  | 3-Carbamoyl-2-phenylpropionaldehyde | 1 | ec:1.1.1.1 |
| cpd:C16586 |  | 2-Phenyl-1,3-propanediol monocarbamate | 1 | ec:1.1.1.1 |
| cpd:C01290 |  | beta-D-Galactosyl-(1->4)-beta-D-glucosyl-(1<->1)-ceramide; beta-D-Galactosyl-1,4-beta-D-glucosylceramide; Lactosylceramide; Gal-beta1->4Glc-beta1->1'Cer; LacCer; Lactosyl-N-acylsphingosine; D-Galactosyl-1,4-beta-D-glucosylceramide | 1 | ec:3.2.1.23 |
| cpd:C04489 |  | 5-Methyltetrahydropteroyltri-L-glutamate | 1 | ec:2.1.1.14 |
| cpd:C16221 |  | (2E)-Octadecenoyl-[acp]; trans-Octadec-2-enoyl-[acp] | 1 |  |
| cpd:C16220 |  | 3-Hydroxyoctadecanoyl-[acp]; 3-Hydroxystearoyl-[acp] | 1 | ec:1.1.1.100 |
| cpd:C04088 |  | Octadecanoyl-[acyl-carrier protein]; Stearoyl-[acyl-carrier protein] | 1 |  |
| cpd:C01326 |  | Hydrogen cyanide; HCN | 1 | ec:2.8.1.1 ec:2.8.1.2 |
| cpd:C03838 |  | 5'-Phosphoribosylglycinamide; GAR; N1-(5-Phospho-D-ribosyl)glycinamide; Glycinamide ribonucleotide | 1 | ec:6.3.4.13 |
| cpd:C16219 |  | 3-Oxostearoyl-[acp]; 3-Oxooctadecanoyl-[acp]; beta-Ketostearoyl-[acp]; 3-Ketostearoyl-[acp] | 1 | ec:1.1.1.100 |
| cpd:C00243 |  | Lactose; 1-beta-D-Galactopyranosyl-4-D-glucopyranose; beta-D-Gal-(1->4)-D-Glc; Milk sugar | 1 | ec:3.2.1.23 |
| cpd:C16217 |  | 3-Hydroxyoctadecanoyl-CoA; 3-Hydroxystearoyl-CoA; beta-Hydroxystearoyl-CoA | 1 | ec:1.1.1.100 |
| cpd:C16216 |  | 3-Oxostearoyl-CoA; 3-Oxooctadecanoyl-CoA; beta-Ketostearoyl-CoA; 3-Ketostearoyl-CoA | 1 | ec:1.1.1.100 |
| cpd:C03794 |  | N6-(1,2-Dicarboxyethyl)-AMP; N6-(1,2-Dicarboxyethyl)AMP; Adenylosuccinate; Adenylosuccinic acid | 1 | ec:6.3.4.4 |
| cpd:C14850 |  | Benzo[a]pyrene-7,8-oxide; Benzo[a]pyrene-7,8-epoxide | 1 | ec:3.3.2.9 |
| cpd:C00637 |  | Indole-3-acetaldehyde; 2-(Indol-3-yl)acetaldehyde; Indoleacetaldehyde | 1 | ec:1.2.1.3 |
| cpd:C06589 |  | cis-2,3-Dihydro-2,3-dihydroxybiphenyl; cis-3-Phenylcyclohexa-3,5-diene-1,2-diol; (1S,2R)-3-Phenylcyclohexa-3,5-diene-1,2-diol | 1 | ec:1.18.1.3 |
| cpd:C06588 |  | Biphenyl; Phenylbenzene; 1,1'-Biphenyl; 1,1'-Diphenyl | 1 | ec:1.18.1.3 |
| cpd:C01279 |  | 4-Amino-5-hydroxymethyl-2-methylpyrimidine; Toxopyrimidine; 4-Amino-2-methyl-5-pyrimidinemethanol | 1 | ec:3.5.99.2 |
| cpd:C06585 |  | cis-2,3-Dihydro-2,3-dihydroxy-4'-chlorobiphenyl | 1 | ec:1.18.1.3 |
| cpd:C01278 |  | 2-Carboxy-2,5-dihydro-5-oxofuran-2-acetate; 5-Carboxy-2,5-dihydro-2-oxofuran-5-acetate; 4-Carboxymuconolactone; gamma-Carboxymuconolactone | 1 | ec:4.1.1.44 |
| cpd:C06584 |  | 4-Chlorobiphenyl; 1-Chloro-4-phenyl benzene; 4-Monochloro-biphenyl | 1 | ec:1.18.1.3 |
| cpd:C00630 |  | 2-Methylpropanoyl-CoA; 2-Methylpropionyl-CoA; Isobutyryl-CoA | 1 | ec:1.3.8.7 |
| cpd:C00237 |  | CO; Carbon monoxide | 1 | ec:1.13.11.54 ec:1.13.11.53 |
| cpd:C00234 |  | 10-Formyltetrahydrofolate; 10-Formyl-THF | 1 | ec:3.5.4.10 ec:2.1.2.3 |
| cpd:C00232 |  | Succinate semialdehyde; Succinic semialdehyde; 4-Oxobutanoate | 1 | ec:1.2.1.79 ec:1.2.1.16 ec:1.2.1.20 |
| cpd:C00231 |  | D-Xylulose 5-phosphate | 1 | ec:2.7.1.17 |
| cpd:C00198 |  | D-Glucono-1,5-lactone; Gluconic lactone; Gluconic acid lactone; 1,5-Gluconolactone; delta-Gluconolactone; D-Gluconolactone; Gluconolactone | 1 | ec:3.1.1.17 |
| cpd:C03427 |  | Prephytoene diphosphate | 1 | ec:2.5.1.32 |
| cpd:C05143 |  | Dhurrin; (S)-4-Hydroxymandelonitrile beta-D-glucoside | 1 | ec:3.2.1.21 |
| cpd:C14844 |  | Bromobenzene-3,4-dihydrodiol; 4-Bromo-3,5-cyclohexadiene-1,2-diol | 1 | ec:3.3.2.9 |
| cpd:C14842 |  | Bromobenzene-2,3-dihydrodiol; 3-Bromo-3,5-cyclohexadiene-1,2-diol | 1 | ec:3.3.2.9 |
| cpd:C08334 |  | Lotaustralin | 1 | ec:3.2.1.21 |
| cpd:C06615 |  | cis-3-Chloroacrylic acid | 1 | ec:1.2.1.3 |
| cpd:C05936 |  | N4-Acetylaminobutanal; 4-Acetamidobutanal | 1 | ec:1.2.1.3 |
| cpd:C06614 |  | trans-3-Chloroacrylic acid | 1 | ec:1.2.1.3 |
| cpd:C06612 |  | cis-3-Chloro-2-propene-1-ol; cis-3-Chloroallyl alcohol | 1 | ec:1.1.1.1 |
| cpd:C06579 |  | cis-2,3-Dihydroxy-2,3-dihydro-p-cumate; cis-5,6-Dihydroxy-4-isopropylcyclohexa-1,3-dienecarboxylate | 1 | ec:1.18.1.3 |
| cpd:C06611 |  | trans-3-Chloro-2-propene-1-ol; trans-3-Chloroallyl alcohol | 1 | ec:1.1.1.1 |
| cpd:C06578 |  | p-Cumate | 1 | ec:1.18.1.3 |
| cpd:C18310 |  | 3-Chloro-2-methylmaleylacetate | 1 | ec:3.1.1.45 |
| cpd:C00226 |  | Primary alcohol; 1-Alcohol | 1 | ec:1.1.1.1 |
| cpd:C16551 |  | Alcophosphamide | 1 | ec:1.1.1.1 |
| cpd:C00185 |  | Cellobiose; 1-beta-D-Glucopyranosyl-4-D-glucopyranose | 1 | ec:3.2.1.21 |
| cpd:C05130 |  | Imidazole-4-acetaldehyde; Imidazole acetaldehyde | 1 | ec:1.2.1.3 |
| cpd:C00180 |  | Benzoate; Benzoic acid; Benzenecarboxylic acid; Phenylformic acid; Dracylic acid | 1 | ec:3.5.1.4 |
| cpd:C08325 |  | Amygdalin; (R)-Amygdalin; (R)-Amygdaloside; (R)-Laenitrile | 1 | ec:3.2.1.21 |
| cpd:C18309 |  | 3-Chloro-2-methyldienelactone | 1 | ec:3.1.1.45 |
| cpd:C18305 |  | 2-Chloro-5-methylmaleylacetate | 1 | ec:3.1.1.45 |
| cpd:C18304 |  | 2-Chloro-5-methyl-cis-dienelactone | 1 | ec:3.1.1.45 |
| cpd:C11638 |  | 3-Amino-2-oxopropyl phosphate; 1-Amino-3-(phosphohydroxy)propan-2-one | 1 | ec:1.1.1.262 |
| cpd:C06205 |  | 1,2-Dihydronaphthalene-1,2-diol | 1 | ec:3.3.2.9 |
| cpd:C01251 |  | (R)-2-Hydroxybutane-1,2,4-tricarboxylate; Homocitrate; Homocitric acid; 3-Hydroxy-3-carboxyadipic acid; (R)-2-Hydroxy-1,2,4-butanetricarboxylic acid | 1 | ec:2.3.3.14 |
| cpd:C00177 |  | Cyanide; Prussiate; CN-; Cyano | 1 | ec:2.8.1.1 ec:2.8.1.2 |
| cpd:C00173 |  | Acyl-[acyl-carrier protein] | 1 | ec:2.3.1.15 |
| cpd:C08276 |  | 3-(Methylthio)propionic acid; 3-Methylthiopropionate | 1 | ec:1.13.11.54 ec:1.13.11.53 |
| cpd:C11588 |  | cis-3-(Carboxy-ethyl)-3,5-cyclo-hexadiene-1,2-diol; cis-3-(2-Carboxy-ethyl)-3,5-cyclo-hexadiene-1,2-diol; 3-(cis-5,6-Dihydroxycyclohexa-1,3-dien-1-yl)propanoate | 1 | ec:1.18.1.3 |
| cpd:C00561 |  | Mandelonitrile; Benzaldehyde cyanohydrin; (R)-Mandelonitrile | 1 | ec:3.2.1.21 |
| cpd:C00163 |  | Propanoate; Propionate; Propanoic acid; Propionic acid | 1 | ec:6.2.1.1 |
| cpd:C00162 |  | Fatty acid | 1 | ec:1.2.1.3 |
| cpd:C04431 |  | cis-4-Carboxymethylenebut-2-en-4-olide; 4-Carboxymethylenebut-2-en-4-olide | 1 | ec:3.1.1.45 |
| cpd:C04039 |  | 2,3-Dihydroxy-3-methylbutanoate; 2,3-Dihydroxy-isovalerate; 2,3-Dihydroxy-isovaleric acid | 1 | ec:4.2.1.9 |
| cpd:C00160 |  | Glycolate; Glycolic acid; Hydroxyacetic acid | 1 | ec:3.8.1.2 |
| cpd:C00957 |  | Mercaptopyruvate; 3-Mercaptopyruvic acid; 3-Mercaptopyruvate | 1 | ec:2.8.1.1 ec:2.8.1.2 |
| cpd:C02670 |  | D-Glucuronolactone; Glucurone; D-Glucurono-3,6-lactone; D-Glucurone | 1 | ec:1.2.1.3 |
| cpd:C01594 |  | Linamarin; Phaseolunatin | 1 | ec:3.2.1.21 |
| cpd:C00555 |  | 4-Aminobutyraldehyde; 4-Aminobutanal | 1 | ec:1.2.1.3 |
| cpd:C18243 |  | 2,3,5-Trichloromaleylacetate | 1 | ec:3.1.1.45 |
| cpd:C18242 |  | 2,3,5-Trichlorodienelactone | 1 | ec:3.1.1.45 |
| cpd:C04425 |  | S-Adenosyl-4-methylthio-2-oxobutanoate | 1 | ec:2.6.1.62 |
| cpd:C00155 |  | L-Homocysteine; L-2-Amino-4-mercaptobutyric acid | 1 | ec:2.1.1.14 |
| cpd:C00153 |  | Nicotinamide; Nicotinic acid amide; Niacinamide; Vitamin PP | 1 | ec:3.5.1.19 |
| cpd:C16487 |  | Hydrofluoric acid; Hydrogen fluoride; Fluoride | 1 | ec:3.1.1.45 |
| cpd:C03742 |  | (S)-4-Hydroxymandelonitrile | 1 | ec:3.2.1.21 |
| cpd:C01190 |  | beta-D-Glucosyl-(1<->1)-ceramide; Glucosylceramide; Glucocerebroside; D-Glucosyl-N-acylsphingosine | 1 | ec:3.2.1.23 |
| cpd:C14801 |  | 1-Nitro-5,6-dihydroxy-dihydronaphthalene; 1,2-Dihydro-5-nitro-1,2-naphthalenediol | 1 | ec:3.3.2.9 |
| cpd:C06899 |  | Chloral hydrate | 1 | ec:1.1.1.1 |
| cpd:C00149 |  | (S)-Malate; L-Malate; L-Apple acid; L-Malic acid; L-2-Hydroxybutanedioic acid; Malate; Malic acid | 1 | ec:2.3.3.9 |
| cpd:C01181 |  | 4-Trimethylammoniobutanoate; Butyro-betaine; gamma-Butyrobetaine | 1 | ec:1.2.1.3 |
| cpd:C16477 |  | 5-Fluoromuconolactone | 1 | ec:3.1.1.45 |
| cpd:C01180 |  | 4-Methylthio-2-oxobutanoic acid; 4-Methylthio-2-oxobutanoate; 2-Keto-4-methylthiobutyric acid; 2-Oxo-4-methylthiobutanoate; 2-Oxo-4-methylthiobutanoic acid | 1 | ec:1.13.11.54 ec:1.13.11.53 |
| cpd:C16476 |  | 4-Fluoromuconolactone | 1 | ec:3.1.1.45 |
| cpd:C00141 |  | 3-Methyl-2-oxobutanoic acid; 3-Methyl-2-oxobutyric acid; 3-Methyl-2-oxobutanoate; 2-Oxo-3-methylbutanoate; 2-Oxoisovalerate; 2-Oxoisopentanoate; alpha-Ketovaline; 2-Ketovaline; 2-Keto-3-methylbutyric acid | 1 | ec:4.2.1.9 |
| cpd:C02659 |  | Acetone cyanohydrin; alpha-Hydroxyisobutyronitrile; 2-Hydroxy-2-methylpropanenitrile; 2-Methyllactonitrile; Acetone cyanhydrin; 2-Hydroxyisobutyronitrile | 1 | ec:3.2.1.21 |
| cpd:C02658 |  | Aldoxime; Aliphatic aldoxime | 1 | ec:4.99.1.5 |
| cpd:C00536 |  | Triphosphate; Triphosphoric acid; Tripolyphosphate; Inorganic triphosphate | 1 | ec:2.5.1.6 |
| cpd:C05444 |  | 3alpha,7alpha,26-Trihydroxy-5beta-cholestane; 5beta-Cholestane-3alpha,7alpha,26-triol | 1 | ec:1.1.1.1 |
| cpd:C00493 |  | Shikimate; Shikimic acid; 3,4,5-Trihydroxy-1-cyclohexenecarboxylic acid | 1 | ec:2.7.1.71 |
| cpd:C00131 |  | dATP; 2'-Deoxyadenosine 5'-triphosphate; Deoxyadenosine 5'-triphosphate; Deoxyadenosine triphosphate | 1 | ec:2.7.7.7 |
| cpd:C00099 |  | beta-Alanine; 3-Aminopropionic acid; 3-Aminopropanoate | 1 | ec:1.2.1.3 |
| cpd:C00097 |  | L-Cysteine; L-2-Amino-3-mercaptopropionic acid | 1 | ec:6.3.2.5 ec:4.1.1.36 |
| cpd:C00096 |  | GDP-mannose; GDP-D-mannose; GDP-alpha-D-mannose | 1 | ec:2.4.1.83 |
| cpd:C00094 |  | Sulfite; Sulfurous acid | 1 | ec:2.8.1.1 ec:2.8.1.2 |
| cpd:C00093 |  | sn-Glycerol 3-phosphate; Glycerophosphoric acid; D-Glycerol 1-phosphate | 1 | ec:2.3.1.15 |
| cpd:C00090 |  | Catechol; 1,2-Benzenediol; o-Benzenediol; 1,2-Dihydroxybenzene; Brenzcatechin; Pyrocatechol | 1 | ec:1.18.1.3 |
| cpd:C05839 |  | cis-beta-D-Glucosyl-2-hydroxycinnamate; beta-D-Glucosyl-2-coumarinate | 1 | ec:3.2.1.21 |
| cpd:C05838 |  | cis-2-Hydroxycinnamate; 2-Coumarinate | 1 | ec:3.2.1.21 |
| cpd:C12622 |  | cis-3-(3-Carboxyethenyl)-3,5-cyclohexadiene-1,2-diol; (2E)-3-(cis-5,6-Dihydroxycyclohexa-1,3-dien-1-yl)prop-2-enoate | 1 | ec:1.18.1.3 |
| cpd:C05796 |  | Galactan | 1 | ec:3.2.1.23 |
| cpd:C00489 |  | Glutarate; Glutaric acid; Pentanedioic acid; 1,3-Propanedicarboxylic acid | 1 | ec:1.2.1.79 ec:1.2.1.16 ec:1.2.1.20 |
| cpd:C05432 |  | Lycopene; all-trans-Lycopene | 1 | ec:1.3.99.26 ec:1.3.99.31 ec:1.3.99.28 ec:1.3.99.29 |
| cpd:C00124 |  | D-Galactose; D-Galactopyranose | 1 | ec:3.2.1.23 |
| cpd:C05431 |  | all-trans-Neurosporene | 1 | ec:1.3.99.26 ec:1.3.99.31 ec:1.3.99.28 ec:1.3.99.29 |
| cpd:C05430 |  | all-trans-zeta-Carotene | 1 | ec:1.3.99.26 ec:1.3.99.31 ec:1.3.99.28 ec:1.3.99.29 |
| cpd:C00120 |  | Biotin; D-Biotin; Vitamin H; Coenzyme R | 1 | ec:2.8.1.6 |
| cpd:C05394 |  | 3-Keto-beta-D-galactose | 1 | ec:3.2.1.23 |
| cpd:C04352 |  | (R)-4'-Phosphopantothenoyl-L-cysteine; N-[(R)-4'-Phosphopantothenoyl]-L-cysteine | 1 | ec:6.3.2.5 ec:4.1.1.36 |
| cpd:C03273 |  | 5-Oxopentanoate; Glutarate semialdehyde | 1 | ec:1.2.1.79 ec:1.2.1.16 ec:1.2.1.20 |
| cpd:C02593 |  | Tetradecanoyl-CoA; Myristoyl-CoA | 1 | ec:1.3.8.7 |
| cpd:C00118 |  | D-Glyceraldehyde 3-phosphate; (2R)-2-Hydroxy-3-(phosphonooxy)-propanal; Glyceraldehyde 3-phosphate | 1 | ec:4.1.2.13 |
| cpd:C06103 |  | 6-Hydroxyhexanoic acid; 6-Hydroxyhexanoate | 1 | ec:3.1.1.17 |
| cpd:C00473 |  | Retinol; all-trans-Retinol; Vitamin A; Vitamin A1 | 1 | ec:1.1.1.1 |
| cpd:C00111 |  | Glycerone phosphate; Dihydroxyacetone phosphate | 1 | ec:4.1.2.13 |
| cpd:C00110 |  | Dolichyl phosphate; Dolichol phosphate | 1 | ec:2.4.1.83 |
| cpd:C00075 |  | UTP; Uridine 5'-triphosphate; Uridine triphosphate | 1 | ec:2.7.7.6 |
| cpd:C01944 |  | Octanoyl-CoA | 1 | ec:1.3.8.7 |
| cpd:C03263 |  | Coproporphyrinogen III | 1 | ec:1.3.3.3 |
| cpd:C11924 |  | Perillic acid | 1 | ec:1.2.1.3 |
| cpd:C02222 |  | 2-Maleylacetate; 4-Oxohex-2-enedioate; Maleylacetate | 1 | ec:3.1.1.45 |
| cpd:C01149 |  | 4-Trimethylammoniobutanal | 1 | ec:1.2.1.3 |
| cpd:C00469 |  | Ethanol; Ethyl alcohol; Methylcarbinol | 1 | ec:1.1.1.1 |
| cpd:C00108 |  | Anthranilate; Anthranilic acid; o-Aminobenzoic acid; Vitamin L1; 2-Aminobenzoate | 1 | ec:1.18.1.3 |
| cpd:C07490 |  | Trichloroethanol; 2,2,2-Trichloroethanol | 1 | ec:1.1.1.1 |
| cpd:C05414 |  | all-trans-Phytofluene | 1 | ec:1.3.99.26 ec:1.3.99.31 ec:1.3.99.28 ec:1.3.99.29 |
| cpd:C05413 |  | all-trans-Phytoene | 1 | ec:2.5.1.32 |
| cpd:C04734 |  | 1-(5'-Phosphoribosyl)-5-formamido-4-imidazolecarboxamide; 5'-Phosphoribosyl-5-formamido-4-imidazolecarboxamide; 5-Formamido-1-(5-phosphoribosyl)imidazole-4-carboxamide; 5-Formamido-1-(5-phospho-D-ribosyl)imidazole-4-carboxamide; FAICAR | 1 | ec:3.5.4.10 ec:2.1.2.3 |
| cpd:C05378 |  | beta-D-Fructose 1,6-bisphosphate | 1 | ec:4.1.2.13 |
| cpd:C06055 |  | O-Phospho-4-hydroxy-L-threonine; 4-(Phosphonooxy)-threonine; 4-(Phosphonooxy)-L-threonine | 1 | ec:1.1.1.262 |
| cpd:C00101 |  | Tetrahydrofolate; 5,6,7,8-Tetrahydrofolate; Tetrahydrofolic acid; THF; (6S)-Tetrahydrofolate; (6S)-Tetrahydrofolic acid; (6S)-THFA | 1 | ec:3.5.4.10 ec:2.1.2.3 |
| cpd:C07091 |  | cis-Acetylacrylate | 1 | ec:3.1.1.45 |
| cpd:C07090 |  | Protoanemonin; 4-Methylenebut-2-en-4-olide; cis-4-Methylenebut-2-en-4-olide | 1 | ec:3.1.1.45 |
| cpd:C00060 |  | Carboxylate; R-COOH; Monocarboxylate; Carboxylic acid | 1 | ec:3.5.1.4 |
| cpd:C04294 |  | 5-(2-Hydroxyethyl)-4-methylthiazole; 4-Methyl-5-(2'-hydroxyethyl)-thiazole; 4-Methyl-5-(2-hydroxyethyl)-thiazole | 1 | ec:3.5.99.2 |
| cpd:C02576 |  | Perillyl aldehyde; Perillaldehyde | 1 | ec:1.2.1.3 |
| cpd:C19588 |  | Aflatoxin B1 diol; Aflatoxin B1-2,3-dihydrodiol | 1 | ec:3.3.2.9 |
| cpd:C00459 |  | dTTP; Deoxythymidine triphosphate; Deoxythymidine 5'-triphosphate; TTP | 1 | ec:2.7.7.7 |
| cpd:C00458 |  | dCTP; Deoxycytidine 5'-triphosphate; Deoxycytidine triphosphate; 2'-Deoxycytidine 5'-triphosphate | 1 | ec:2.7.7.7 |
| cpd:C05764 |  | Hexadecanoyl-[acp]; Hexadecanoyl-[acyl-carrier protein] | 1 |  |
| cpd:C05763 |  | trans-Hexadec-2-enoyl-[acp]; trans-Hexadec-2-enoyl-[acyl-carrier protein]; (2E)-Hexadecenoyl-[acp] | 1 |  |
| cpd:C05762 |  | 3-Oxohexadecanoyl-[acp]; 3-Oxohexadecanoyl-[acyl-carrier protein] | 1 | ec:1.1.1.100 |
| cpd:C01134 |  | Pantetheine 4'-phosphate; 4'-Phosphopantetheine; Phosphopantetheine; D-Pantetheine 4'-phosphate | 1 | ec:6.3.2.5 ec:4.1.1.36 |
| cpd:C05403 |  | 3-Ketolactose | 1 | ec:3.2.1.23 |
| cpd:C05761 |  | Tetradecanoyl-[acp]; Tetradecanoyl-[acyl-carrier protein]; Myristoyl-[acyl-carrier protein] | 1 |  |
| cpd:C07086 |  | Phenylacetic acid; Benzylformic acid; Phenylacetate; Benzeneacetic acid | 1 | ec:3.5.1.4 |
| cpd:C05760 |  | trans-Tetradec-2-enoyl-[acp]; trans-Tetradec-2-enoyl-[acyl-carrier protein]; (2E)-Tetradecenoyl-[acp] | 1 |  |
| cpd:C02170 |  | Methylmalonate; Methylmalonic acid | 1 | ec:1.2.1.3 |
| cpd:C04688 |  | (3R)-3-Hydroxytetradecanoyl-[acyl-carrier protein]; (R)-3-Hydroxytetradecanoyl-[acyl-carrier protein]; beta-Hydroxymyristyl-[acyl-carrier protein]; HMA | 1 | ec:1.1.1.100 |
| cpd:C01094 |  | D-Fructose 1-phosphate | 1 | ec:4.1.2.13 |
| cpd:C07479 |  | 2-Oxo-5-methyl-cis-muconate | 1 | ec:5.3.2.6 |
| cpd:C07478 |  | 2-Hydroxy-5-methyl-cis,cis-muconate | 1 | ec:5.3.2.6 |
| cpd:C00844 |  | Prunasin; (R)-Prunasin | 1 | ec:3.2.1.21 |
| cpd:C05759 |  | 3-Oxotetradecanoyl-[acp]; 3-Oxotetradecanoyl-[acyl-carrier protein] | 1 | ec:1.1.1.100 |
| cpd:C20377 |  | 3-Hydroxypimeloyl-[acp] methyl ester; 3-Hydroxypimeloyl-[acyl-carrier protein] methyl ester | 1 | ec:1.1.1.100 |
| cpd:C20376 |  | 3-Ketopimeloyl-[acp] methyl ester; 3-Ketopimeloyl-[acyl-carrier protein] methyl ester | 1 | ec:1.1.1.100 |
| cpd:C01880 |  | epsilon-Caprolactone; 6-Hexanolide; 1-Oxa-2-oxocycloheptane; 2-Oxepanone; Hexano-6-lactone | 1 | ec:3.1.1.17 |
| cpd:C05758 |  | trans-Dodec-2-enoyl-[acp]; trans-Dodec-2-enoyl-[acyl-carrier protein]; (2E)-Dodecenoyl-[acp] | 1 |  |
| cpd:C05757 |  | (R)-3-Hydroxydodecanoyl-[acp]; (R)-3-Hydroxydodecanoyl-[acyl-carrier protein]; D-3-Hydroxydodecanoyl-[acp]; D-3-Hydroxydodecanoyl-[acyl-carrier protein] | 1 | ec:1.1.1.100 |
| cpd:C05756 |  | 3-Oxododecanoyl-[acp]; 3-Oxododecanoyl-[acyl-carrier protein] | 1 | ec:1.1.1.100 |
| cpd:C20373 |  | 3-Hydroxyglutaryl-[acp] methyl ester; 3-Hydroxyglutaryl-[acyl-carrier protein] methyl ester | 1 | ec:1.1.1.100 |
| cpd:C05755 |  | Decanoyl-[acp]; Decanoyl-[acyl-carrier protein] | 1 |  |
| cpd:C20372 |  | 3-Ketoglutaryl-[acp] methyl ester; 3-Ketoglutaryl-[acyl-carrier protein] methyl ester | 1 | ec:1.1.1.100 |
| cpd:C05754 |  | trans-Dec-2-enoyl-[acp]; trans-Dec-2-enoyl-[acyl-carrier protein]; trans-2-Decenoyl-[acyl-carrier protein]; (2E)-Decenoyl-[acp] | 1 |  |
| cpd:C00447 |  | Sedoheptulose 1,7-bisphosphate; D-Sedoheptulose 1,7-bisphosphate; D-altro-Heptulose 1,7-biphosphate | 1 | ec:4.1.2.13 |
| cpd:C05753 |  | 3-Oxodecanoyl-[acp]; 3-Oxodecanoyl-[acyl-carrier protein] | 1 | ec:1.1.1.100 |
| cpd:C07111 |  | Ethylbenzene; Phenylethane; Ethylbenzol; Ethylenzene | 1 | ec:1.18.1.3 |
| cpd:C05752 |  | Octanoyl-[acp]; Octanoyl-[acyl-carrier protein] | 1 |  |
| cpd:C05751 |  | trans-Oct-2-enoyl-[acp]; trans-Oct-2-enoyl-[acyl-carrier protein]; Oct-2-enoyl-[acyl-carrier protein]; 2-Octenoyl-[acyl-carrier protein]; (2E)-Octenoyl-[acp] | 1 |  |
| cpd:C05359 |  | e-; Electron | 1 | ec:2.8.1.6 |
| cpd:C05750 |  | 3-Oxooctanoyl-[acp]; 3-Oxooctanoyl-[acyl-carrier protein] | 1 | ec:1.1.1.100 |
| cpd:C04677 |  | 1-(5'-Phosphoribosyl)-5-amino-4-imidazolecarboxamide; 5'-Phosphoribosyl-5-amino-4-imidazolecarboxamide; 5'-Phospho-ribosyl-5-amino-4-imidazole carboxamide; AICAR; 5-Aminoimidazole-4-carboxamide ribotide; 5-Phosphoribosyl-4-carbamoyl-5-aminoimidazole; 5-Amino-1-(5-phospho-D-ribosyl)imidazole-4-carboxamide | 1 | ec:3.5.4.10 ec:2.1.2.3 |
| cpd:C00049 |  | L-Aspartate; L-Aspartic acid; 2-Aminosuccinic acid; L-Asp | 1 | ec:6.3.4.4 |
| cpd:C00046 |  | RNA; RNAn; RNAn+1; RNA(linear); (Ribonucleotide)n; (Ribonucleotide)m; (Ribonucleotide)n+m; Ribonucleic acid | 1 | ec:2.7.7.6 |
| cpd:C19607 |  | trans-5,6-Dihydro-5,6-dihydroxy-7,12-dimethylbenz[a]anthracene; trans-DMBA-5,6-dihydrodiol | 1 | ec:3.3.2.9 |
| cpd:C00041 |  | L-Alanine; L-2-Aminopropionic acid; L-alpha-Alanine | 1 | ec:2.3.1.47 |
| cpd:C00040 |  | Acyl-CoA; Acyl coenzyme A | 1 | ec:2.3.1.15 |
| cpd:C19604 |  | 7,12-Dimethylbenz[a]anthracene 5,6-oxide; DMBA-5,6-epoxide | 1 | ec:3.3.2.9 |
| cpd:C04272 |  | (R)-2,3-Dihydroxy-3-methylbutanoate; (R)-2,3-Dihydroxy-isovalerate; (R)-2,3-Dihydroxy-isovaleric acid; (2R)-2,3-Dihydroxy-3-methylbutanoate | 1 | ec:4.2.1.9 |
| cpd:C03232 |  | 3-Phosphonooxypyruvate; 3-Phosphonooxypyruvic acid; 3-Phosphohydroxypyruvate; 3-Phosphohydroxypyruvic acid | 1 | ec:1.1.1.95 |
| cpd:C05749 |  | Hexanoyl-[acp]; Hexanoyl-[acyl-carrier protein] | 1 |  |
| cpd:C05748 |  | trans-Hex-2-enoyl-[acp]; trans-Hex-2-enoyl-[acyl-carrier protein]; (2E)-Hexenoyl-[acp] | 1 |  |
| cpd:C05747 |  | (R)-3-Hydroxyhexanoyl-[acp]; (R)-3-Hydroxyhexanoyl-[acyl-carrier protein]; D-3-Hydroxyhexanoyl-[acp]; D-3-Hydroxyhexanoyl-[acyl-carrier protein] | 1 | ec:1.1.1.100 |
| cpd:C05746 |  | 3-Oxohexanoyl-[acp]; 3-Oxohexanoyl-[acyl-carrier protein] | 1 | ec:1.1.1.100 |
| cpd:C05745 |  | Butyryl-[acp]; Butyryl-[acyl-carrier protein]; Butanoyl-[acp] | 1 |  |
| cpd:C05744 |  | Acetoacetyl-[acp]; Acetoacetyl-[acyl-carrier protein] | 1 | ec:1.1.1.100 |
| cpd:C04706 |  | cis-2-Chloro-4-carboxymethylenebut-2-en-1,4-olide; cis-2-Chlorodienelactone; cis-4-Carboxymethylene-2-chlorobut-2-en-4-olide | 1 | ec:3.1.1.45 |
| cpd:C00435 |  | Oxidized rubredoxin | 1 | ec:1.18.1.3 |
| cpd:C01079 |  | Protoporphyrinogen IX | 1 | ec:1.3.3.3 |
| cpd:C00039 |  | DNA; DNAn; DNAn+1; (Deoxyribonucleotide)n; (Deoxyribonucleotide)m; (Deoxyribonucleotide)n+m; Deoxyribonucleic acid | 1 | ec:2.7.7.7 |
| cpd:C01909 |  | Dethiobiotin; Desthiobiotin | 1 | ec:2.8.1.6 |
| cpd:C02946 |  | 4-Acetamidobutanoate; N4-Acetylaminobutanoate | 1 | ec:1.2.1.3 |
| cpd:C03586 |  | 2-Oxo-2,3-dihydrofuran-5-acetate; 3-Oxoadipate enol-lactone; 4,5-Dihydro-5-oxofuran-2-acetate; 5-Oxo-4,5-dihydrofuran-2-acetate | 1 | ec:4.1.1.44 |
| cpd:C00423 |  | trans-Cinnamate; trans-Cinnamic acid; (E)-Cinnamate | 1 | ec:1.18.1.3 |
| cpd:C01102 |  | O-Phospho-L-homoserine | 1 | ec:2.7.1.39 |
| cpd:C05698 |  | Selenohomocysteine | 1 | ec:2.1.1.14 |
| cpd:C00029 |  | UDP-glucose; UDPglucose; UDP-D-glucose; Uridine diphosphate glucose; UDP-alpha-D-glucose | 1 | ec:2.4.1.11 |
| cpd:C02939 |  | 3-Methylbutanoyl-CoA; Isovaleryl-CoA | 1 | ec:1.3.8.7 |
| cpd:C01063 |  | 6-Carboxyhexanoyl-CoA; Pimeloyl-CoA | 1 | ec:2.3.1.47 |
| cpd:C05691 |  | Se-Adenosylselenomethionine | 1 | ec:2.5.1.6 |
| cpd:C04253 |  | Electron-transferring flavoprotein; Electron-transfer flavoprotein | 1 | ec:1.3.8.7 |
| cpd:C03175 |  | Shikimate 3-phosphate; Shikimate 5-phosphate | 1 | ec:2.7.1.71 |
| cpd:C01455 |  | Toluene; Methylbenzene; Toluol | 1 | ec:1.18.1.3 |
| cpd:C06007 |  | (R)-2,3-Dihydroxy-3-methylpentanoate; (R)-2,3-Dihydroxy-3-methylvalerate; (2R,3R)-2,3-Dihydroxy-3-methylpentanoate | 1 | ec:4.2.1.9 |
| cpd:C00378 |  | Thiamine; Thiamin; Vitamin B1; Aneurin; Antiberiberi factor | 1 | ec:3.5.99.2 |
| cpd:C00376 |  | Retinal; Vitamin A aldehyde; Retinene; all-trans-Retinal; all-trans-Vitamin A aldehyde; all-trans-Retinene | 1 | ec:1.1.1.1 |
| cpd:C06002 |  | (S)-Methylmalonate semialdehyde | 1 | ec:1.2.1.3 |
| cpd:C00015 |  | UDP; Uridine 5'-diphosphate | 1 | ec:2.4.1.11 |
| cpd:C17023 |  | Sulfur donor; S-donor | 1 | ec:2.8.1.6 |
| cpd:C04246 |  | But-2-enoyl-[acyl-carrier protein] | 1 |  |
| cpd:C00804 |  | Propynoate; Propiolic acid; Acetylenecarboxylic acid; Acetylenemonocarboxylate | 1 | ec:1.2.1.3 |
| cpd:C00800 |  | L-Gulonate; L-Gulonic acid; Gulonate; Gulonic acid | 1 | ec:3.1.1.17 |
| cpd:C06754 |  | Chloroacetaldehyde; 2-Chloroethanal | 1 | ec:1.2.1.3 |
| cpd:C05713 |  | Cyanoglycoside; Cyanoglucoside | 1 | ec:3.2.1.21 |
| cpd:C05712 |  | Cyanohydrin | 1 | ec:3.2.1.21 |
| cpd:C00760 |  | Cellulose; (1,4-beta-D-Glucosyl)n; (1,4-beta-D-Glucosyl)n+1; (1,4-beta-D-Glucosyl)n-1; 1,4-beta-D-Glucan; Microcrystalline cellulose | 1 | ec:3.2.1.21 |
| cpd:C00369 |  | Starch | 1 | ec:2.4.1.18 |
| cpd:C19490 |  | trans-3,4-Dihydro-3,4-dihydroxy-7,12-dimethylbenz[a]anthracene; trans-DMBA-3,4-dihydrodiol | 1 | ec:3.3.2.9 |
| cpd:C04633 |  | (3R)-3-Hydroxypalmitoyl-[acyl-carrier protein]; (R)-3-Hydroxypalmitoyl-[acyl-carrier protein]; (3R)-3-Hydroxyhexadecanoyl-[acyl-carrier protein]; (R)-3-Hydroxyhexadecanoyl-[acyl-carrier protein] | 1 | ec:1.1.1.100 |
| cpd:C00362 |  | dGMP; 2'-Deoxyguanosine 5'-monophosphate; 2'-Deoxyguanosine 5'-phosphate; Deoxyguanylic acid; Deoxyguanosine monophosphate | 1 | ec:2.7.4.8 |
| cpd:C00361 |  | dGDP; 2'-Deoxyguanosine 5'-diphosphate | 1 | ec:2.7.4.8 |
| cpd:C01040 |  | L-Gulono-1,4-lactone; L-Gulono-gamma-lactone; gamma-Gulonolactone; L-Gulonic acid gamma-lactone; L-Gulonolactone | 1 | ec:3.1.1.17 |
| cpd:C05274 |  | Decanoyl-CoA | 1 | ec:1.3.8.7 |
| cpd:C04592 |  | Toluene-cis-dihydrodiol; (1S,2R)-3-Methylcyclohexa-3,5-diene-1,2-diol | 1 | ec:1.18.1.3 |
| cpd:C05270 |  | Hexanoyl-CoA | 1 | ec:1.3.8.7 |
| cpd:C01832 |  | Lauroyl-CoA; Lauroyl coenzyme A; Dodecanoyl-CoA | 1 | ec:1.3.8.7 |
| cpd:C01798 |  | D-Glucoside | 1 | ec:3.2.1.21 |
| cpd:C19489 |  | 1a,11b-Dihydro-4,9-dimethylbenz[a]anthra[3,4-b]oxirene | 1 | ec:3.3.2.9 |
| cpd:C01037 |  | 7,8-Diaminononanoate | 1 | ec:2.6.1.62 |
| cpd:C05665 |  | 3-Aminopropanal; beta-Aminopropion aldehyde | 1 | ec:1.2.1.3 |
| cpd:C02909 |  | (2-Naphthyl)methanol; 2-Naphthalenemethanol; 2-Hydroxymethylnaphthalene | 1 | ec:1.1.1.1 |
| cpd:C01035 |  | 4-Guanidinobutanoate; 4-Guanidinobutyric acid | 1 | ec:3.5.1.4 |
| cpd:C00354 |  | D-Fructose 1,6-bisphosphate | 1 | ec:4.1.2.13 |
| cpd:C00353 |  | Geranylgeranyl diphosphate; Geranylgeranyl pyrophosphate; all-trans-Geranylgeranyl diphosphate; all-trans-Geranylgeranyl pyrophosphate; (2E,6E,10E)-3,7,11,15-Tetramethylhexadeca-2,6,10,14-tetraen-1-yl diphosphate | 1 | ec:2.5.1.32 |
| cpd:C05269 |  | 3-Oxohexanoyl-CoA; 3-Ketohexanoyl-CoA | 1 | ec:2.3.1.9 |
| cpd:C04620 |  | (3R)-3-Hydroxyoctanoyl-[acyl-carrier protein]; (R)-3-Hydroxyoctanoyl-[acyl-carrier protein] | 1 | ec:1.1.1.100 |
| cpd:C20679 |  | Tungstate; Tungstic acid | 1 | ec:3.6.3.55 |
| cpd:C02501 |  | 2-Hydroxymuconate | 1 | ec:5.3.2.6 |
| cpd:C18796 |  | (2R)-2-Hydroxy-2-methylbutanenitrile; 2-Hydroxy-2-methylbutanenitrile | 1 | ec:3.2.1.21 |
| cpd:C04619 |  | (3R)-3-Hydroxydecanoyl-[acyl-carrier protein]; (R)-3-Hydroxydecanoyl-[acyl-carrier protein] | 1 | ec:1.1.1.100 |
| cpd:C04618 |  | (3R)-3-Hydroxybutanoyl-[acyl-carrier protein]; (R)-3-Hydroxybutanoyl-[acyl-carrier protein] | 1 | ec:1.1.1.100 |

  
**Over-represented Pathway Summary**: Collection of the KEGG metabolic pathways containing the proteins identified in the "Over-represented Metabolite Summary" ranked by the highest number of hits per pathway  

| Pathway ID | EC | EC Frequency | Name |
| --- | --- | --- | --- |
| map00260 | ec:1.1.1.1 ec:1.1.1.103 ec:2.7.8.8 ec:2.7.1.39 ec:1.1.1.95 ec:2.3.1.29 ec:5.4.2.12 | 60 | path:map00260 Glycine, serine and threonine metabolism |
| map00650 | ec:2.3.1.9 ec:1.1.1.83 ec:4.2.1.17 ec:1.2.1.79 ec:1.2.1.16 ec:1.1.1.30 | 41 | path:map00650 Butanoate metabolism |
| map00072 | ec:4.1.1.4 ec:2.3.1.9 ec:1.1.1.30 | 32 | path:map00072 Synthesis and degradation of ketone bodies |
| map00630 | ec:4.1.3.1 ec:4.2.1.3 ec:2.3.1.9 ec:1.1.1.93 ec:2.3.3.9 | 25 | path:map00630 Glyoxylate and dicarboxylate metabolism |
| map00640 | ec:1.3.8.7 ec:1.2.1.3 ec:4.1.1.4 ec:2.3.1.9 ec:4.2.1.17 ec:6.2.1.1 | 21 | path:map00640 Propanoate metabolism |
| map00230 | ec:2.7.4.8 ec:6.3.5.2 ec:1.17.1.4 ec:2.1.2.3 ec:6.3.4.13 ec:3.5.4.10 ec:2.7.7.7 ec:2.7.7.6 ec:2.7.6.1 ec:6.3.4.4 | 17 | path:map00230 Purine metabolism |
| map00980 | ec:1.1.1.1 ec:2.5.1.18 ec:3.3.2.9 | 11 | path:map00980 Metabolism of xenobiotics by cytochrome P450 |
| map00982 | ec:1.1.1.1 ec:2.5.1.18 | 10 | path:map00982 Drug metabolism - cytochrome P450 |
| map00330 | ec:1.2.1.38 ec:1.2.1.3 ec:3.5.1.4 | 10 | path:map00330 Arginine and proline metabolism |
| map00071 | ec:1.3.8.7 ec:1.1.1.1 ec:1.2.1.3 ec:2.3.1.9 ec:6.2.1.3 ec:4.2.1.17 ec:1.18.1.3 | 9 | path:map00071 Fatty acid degradation |
| map00910 | ec:4.2.1.104 ec:4.2.1.1 ec:1.18.6.1 | 9 | path:map00910 Nitrogen metabolism |
| map00480 | ec:2.5.1.18 | 9 | path:map00480 Glutathione metabolism |
| map00010 | ec:1.1.1.1 ec:1.2.1.3 ec:5.4.2.12 ec:6.2.1.1 ec:4.1.2.13 | 8 | path:map00010 Glycolysis / Gluconeogenesis |
| map00625 | ec:1.1.1.1 ec:3.8.1.2 ec:1.18.6.1 ec:1.2.1.3 | 7 | path:map00625 Chloroalkane and chloroalkene degradation |
| map00680 | ec:1.1.1.95 ec:5.4.2.12 ec:6.2.1.1 ec:4.1.2.13 | 7 | path:map00680 Methane metabolism |
| map00380 | ec:4.2.1.84 ec:1.2.1.3 ec:3.5.1.4 ec:2.3.1.9 ec:4.2.1.17 | 7 | path:map00380 Tryptophan metabolism |
| map00500 | ec:2.4.1.11 ec:3.2.1.21 ec:2.4.1.18 ec:5.1.3.6 | 6 | path:map00500 Starch and sucrose metabolism |
| map00627 | ec:4.2.1.84 ec:1.14.13.82 ec:3.5.1.4 ec:4.2.1.17 | 6 | path:map00627 Aminobenzoate degradation |
| map00720 | ec:4.2.1.3 ec:2.3.1.9 ec:4.2.1.17 ec:6.2.1.1 | 6 | path:map00720 Carbon fixation pathways in prokaryotes |
| map00362 | ec:5.3.2.6 ec:4.1.1.44 ec:2.3.1.9 ec:4.2.1.17 | 5 | path:map00362 Benzoate degradation |
| map00240 | ec:4.1.1.23 ec:2.7.7.7 ec:2.7.7.6 | 5 | path:map00240 Pyrimidine metabolism |
| map00983 | ec:6.3.5.2 | 5 | path:map00983 Drug metabolism - other enzymes |
| map00620 | ec:2.3.3.14 ec:1.2.1.3 ec:2.3.1.9 ec:6.2.1.1 ec:2.3.3.9 | 5 | path:map00620 Pyruvate metabolism |
| map00280 | ec:1.3.8.7 ec:1.2.1.3 ec:2.3.1.9 ec:4.2.1.17 | 5 | path:map00280 Valine, leucine and isoleucine degradation |
| map00310 | ec:1.2.1.3 ec:2.3.1.9 ec:1.2.1.20 ec:4.2.1.17 | 5 | path:map00310 Lysine degradation |
| map00270 | ec:2.1.1.14 ec:1.13.11.54 ec:2.8.1.2 ec:1.13.11.53 ec:2.5.1.6 | 5 | path:map00270 Cysteine and methionine metabolism |
| map00564 | ec:2.7.8.8 ec:2.3.1.15 | 4 | path:map00564 Glycerophospholipid metabolism |
| map00643 | ec:4.2.1.84 ec:3.5.1.4 ec:3.5.5.7 | 4 | path:map00643 Styrene degradation |
| map00053 | ec:1.2.1.3 ec:4.2.1.41 ec:3.1.1.17 ec:4.2.1.40 | 4 | path:map00053 Ascorbate and aldarate metabolism |
| map00780 | ec:2.3.1.47 ec:1.1.1.100 ec:2.8.1.6 ec:2.6.1.62 | 4 | path:map00780 Biotin metabolism |
| map00030 | ec:2.7.6.1 ec:3.1.1.17 ec:4.1.2.13 | 4 | path:map00030 Pentose phosphate pathway |
| map00906 | ec:1.3.99.28 ec:2.5.1.32 ec:1.3.99.26 ec:1.3.99.31 | 4 | path:map00906 Carotenoid biosynthesis |
| map00410 | ec:1.3.8.7 ec:1.2.1.3 ec:4.2.1.17 | 4 | path:map00410 beta-Alanine metabolism |
| map00760 | ec:3.5.1.19 ec:6.3.4.21 ec:3.6.1.22 | 4 | path:map00760 Nicotinate and nicotinamide metabolism |
| map00364 | ec:4.2.1.84 ec:3.1.1.45 | 3 | path:map00364 Fluorobenzoate degradation |
| map00360 | ec:3.5.1.4 ec:4.2.1.17 | 3 | path:map00360 Phenylalanine metabolism |
| map00250 | ec:6.3.4.4 ec:1.2.1.79 ec:1.2.1.16 | 3 | path:map00250 Alanine, aspartate and glutamate metabolism |
| map00930 | ec:4.2.1.17 ec:3.1.1.17 | 3 | path:map00930 Caprolactam degradation |
| map00520 | ec:5.1.3.6 | 3 | path:map00520 Amino sugar and nucleotide sugar metabolism |
| map00770 | ec:4.1.1.36 ec:4.2.1.9 ec:6.3.2.5 | 3 | path:map00770 Pantothenate and CoA biosynthesis |
| map00903 | ec:1.2.1.3 ec:4.2.1.17 | 3 | path:map00903 Limonene and pinene degradation |
| map00561 | ec:1.2.1.3 ec:2.3.1.15 | 2 | path:map00561 Glycerolipid metabolism |
| map00361 | ec:3.8.1.2 ec:3.1.1.45 | 2 | path:map00361 Chlorocyclohexane and chlorobenzene degradation |
| map00062 | ec:4.2.1.17 | 2 | path:map00062 Fatty acid elongation |
| map00350 | ec:1.1.1.1 ec:1.2.1.16 | 2 | path:map00350 Tyrosine metabolism |
| map00740 | ec:3.5.4.25 | 2 | path:map00740 Riboflavin metabolism |
| map04151 | ec:2.7.11.1 | 2 | path:map04151 PI3K-Akt signaling pathway |
| map04150 | ec:2.7.11.1 | 2 | path:map04150 mTOR signaling pathway |
| map00040 | ec:2.7.1.17 ec:1.2.1.3 | 2 | path:map00040 Pentose and glucuronate interconversions |
| map00592 | ec:4.2.1.17 | 2 | path:map00592 alpha-Linolenic acid metabolism |
| map00622 | ec:5.3.2.6 ec:1.18.1.3 | 2 | path:map00622 Xylene degradation |
| map00190 | ec:1.6.99.3 | 2 | path:map00190 Oxidative phosphorylation |
| map00281 | ec:4.2.1.17 | 2 | path:map00281 Geraniol degradation |
| map00020 | ec:4.2.1.3 | 2 | path:map00020 Citrate cycle (TCA cycle) |
| map00473 | ec:6.1.1.13 | 2 | path:map00473 D-Alanine metabolism |
| map00400 | ec:2.7.1.71 | 1 | path:map00400 Phenylalanine, tyrosine and tryptophan biosynthesis |
| map00460 | ec:3.2.1.21 | 1 | path:map00460 Cyanoamino acid metabolism |
| map00300 | ec:2.3.3.14 | 1 | path:map00300 Lysine biosynthesis |
| map00750 | ec:1.1.1.262 | 1 | path:map00750 Vitamin B6 metabolism |
| map00061 | ec:1.1.1.100 | 1 | path:map00061 Fatty acid biosynthesis |
| map00450 | ec:2.1.1.14 | 1 | path:map00450 Selenocompound metabolism |
| map00940 | ec:3.2.1.21 | 1 | path:map00940 Phenylpropanoid biosynthesis |
| map00052 | ec:3.2.1.23 | 1 | path:map00052 Galactose metabolism |
| map00051 | ec:4.1.2.13 | 1 | path:map00051 Fructose and mannose metabolism |
| map00340 | ec:1.2.1.3 | 1 | path:map00340 Histidine metabolism |
| map00830 | ec:1.1.1.1 | 1 | path:map00830 Retinol metabolism |
| map00730 | ec:3.5.99.2 | 1 | path:map00730 Thiamine metabolism |
| map00531 | ec:3.2.1.23 | 1 | path:map00531 Glycosaminoglycan degradation |
| map00920 | ec:2.8.1.1 | 1 | path:map00920 Sulfur metabolism |
| map00626 | ec:1.1.1.1 | 1 | path:map00626 Naphthalene degradation |
| map00623 | ec:3.1.1.45 | 1 | path:map00623 Toluene degradation |
| map00290 | ec:4.2.1.9 | 1 | path:map00290 Valine, leucine and isoleucine biosynthesis |
| map00621 | ec:5.3.2.6 | 1 | path:map00621 Dioxin degradation |
| map00710 | ec:4.1.2.13 | 1 | path:map00710 Carbon fixation in photosynthetic organisms |
| map00670 | ec:2.1.2.3 | 1 | path:map00670 One carbon pool by folate |
| map00511 | ec:3.2.1.23 | 1 | path:map00511 Other glycan degradation |
| map00510 | ec:2.4.1.83 | 1 | path:map00510 N-Glycan biosynthesis |
| map00900 | ec:2.3.1.9 | 1 | path:map00900 Terpenoid backbone biosynthesis |
| map00860 | ec:1.3.3.3 | 1 | path:map00860 Porphyrin and chlorophyll metabolism |
| map00604 | ec:3.2.1.23 | 1 | path:map00604 Glycosphingolipid biosynthesis - ganglio series |
| map00600 | ec:3.2.1.23 | 1 | path:map00600 Sphingolipid metabolism |

  
Analysis performed on 2014/02/14 19:54:06
