# Supplementary material for: Automated genomic context analysis and experimental validation platform for discovery of prokaryote transcriptional regulator functions
Source: BMC Genomics. 2014 Dec 18;15(1):1142. doi: 10.1186/1471-2164-15-1142 (PMC4349456; doi:10.1186/1471-2164-15-1142)
Supplement: Supplementary file 14 — Additional file 14: Figure Af2: Structures for the library of candidate effector metabolites predicted from the analysis of the Function Discovery V1.0 output for Bxe_B3018. (PDF 16 KB) [file 12864_2014_6995_MOESM14_ESM.pdf]

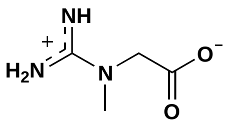

creatine

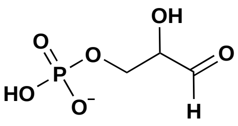

glyceraldehyde-3-phosphate

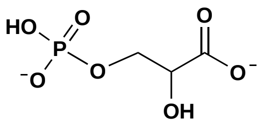

glycerate-3-phosphate

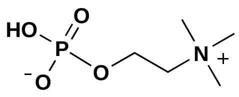

phosphcholine

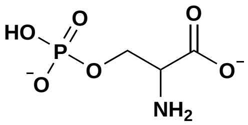

phosphateserine

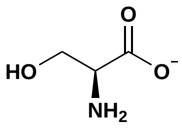

L-serine

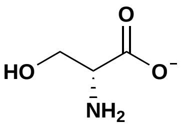

D-serine

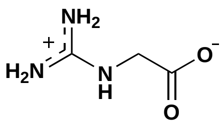

guanidinoactate

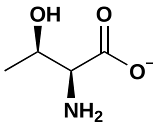

L-threonine

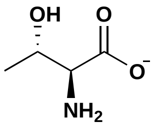

L-allothreonine

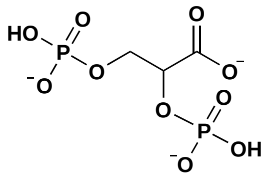

2,3-diphospho-D-glycerate

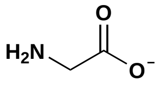

glycine

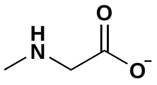

sacosine

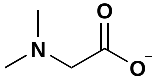

N-dimethylglycine

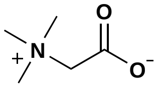

bethaine

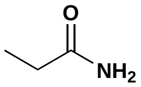

propionamine

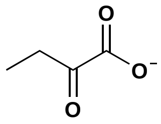

2-oxobutanoate

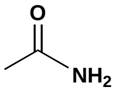

acetamide

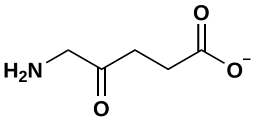

5-aminolevulinate

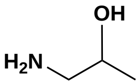

aminopropan-2-ol

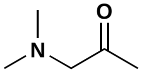

N,N-dimethylaminoacetone

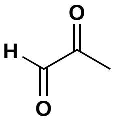

methylglyoxal

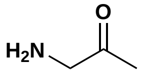

aminoacetone
